# Genome of Malassezia arunalokei and Its Distribution on Facial Skin

**DOI:** 10.1128/spectrum.00506-22

**Published:** 2022-06-01

**Authors:** Yong-Joon Cho, Taeyune Kim, Daniel Croll, Minji Park, Donghyeun Kim, Hye Lim Keum, Woo Jun Sul, Won Hee Jung

**Affiliations:** a School of Biological Sciences and Research Institute of Basic Sciences, Seoul National University, Seoul, South Korea; b Department of Systems Biotechnology, Chung-Ang University, Anseong, South Korea; c Laboratory of Evolutionary Genetics, Institute of Biology, University of Neuchâtel, Neuchâtel, Switzerland; University of Michigan

**Keywords:** genome, *Malassezia arunalokei*, *Malassezia restricta*, mycobiome, skin fungal community, skin mycobiome

## Abstract

*Malassezia* is a fungal genus found on the skin of humans and warm-blooded animals, with 18 species reported to date. In this study, we sequenced and annotated the genome of Malassezia arunalokei, which is the most recently identified *Malassezia* species, and compared it with Malassezia restricta, the predominant isolate from human skin. Additionally, we reanalyzed previously reported mycobiome data sets with a species-level resolution to investigate *M. arunalokei* distribution within the mycobiota of human facial skin. We discovered that the *M. arunalokei* genome is 7.24 Mbp in size and encodes 4,117 protein-coding genes, all of which were clustered with M. restricta. We also found that the average nucleotide identity value of the *M. arunalokei* genome was 93.5, compared with the genomes of three *M. restricta* strains, including *M. restricta* KCTC 27527. Our findings demonstrate that they indeed belong to different species and that *M. arunalokei* may have experienced specific gene loss events during speciation. Furthermore, our study showed that *M. arunalokei* was diverged from *M. restricta* approximately 7.1 million years ago and indicated that *M. arunalokei* is the most recently diverged species in the *Malassezia* lineage to date. Finally, our analysis of the facial mycobiome of previously recruited cohorts revealed that *M. arunalokei* abundance is not associated with seborrheic dermatitis/dandruff or acne, but was revealed to be more abundant on the forehead and cheek than on the scalp.

**IMPORTANCE** *Malassezia* is the fungus predominantly residing on the human skin and causes various skin diseases, including seborrheic dermatitis and dandruff. To date, 18 species have been reported, and among them, *M. restricta* is the most predominant on human skin, especially on the scalp. In this study, we sequenced and analyzed the genome of *M. arunalokei*, which is the most recently identified *Malassezia* species, and compared it with *M. restricta*. Moreover, we analyzed the fungal microbiome to investigate the *M. arunalokei* distribution on human facial skin. We found that *M. arunalokei* may have experienced specific gene loss events during speciation. Our study also showed that *M. arunalokei* was diverged from *M. restricta* approximately 7.1 million years ago and indicated that *M. arunalokei* is the most recently diverged species in the *Malassezia* lineage. Finally, our analysis of the facial mycobiome revealed that *M. arunalokei* has higher relative abundance on the forehead and cheek than the scalp.

## INTRODUCTION

*Malassezia* is a fungal genus predominantly residing on the skin of humans and warm-blooded animals. A major physiological characteristic of *Malassezia* is its lipid dependency, which reflects the sebum-rich environment it inhabits. The lipophilic nature of *Malassezia* is due to the absence of a gene encoding fatty acid synthase in the genome of most *Malassezia* species (1). To date, 18 species have been reported, of which 10 (*M. restricta*, *M. globosa*, *M. arunalokei*, *M. sympodialis*, *M. dermatis*, *M. slooffiae*, *M. furfur*, *M. obtusa*, *M. japonica*, and *M. yamatoensis*) are found on human skin. Among the *Malassezia* species, Malassezia restricta is the most predominant on human skin, especially on the scalp. It is associated with skin diseases such as seborrheic dermatitis. In addition to *M. restricta*, Malassezia arunalokei is of particular interest because of its high internal transcribed spacer (ITS) sequence similarity to *M. restricta* (2).

*M. arunalokei* was first identified and reported in 2016 by Honnavar et al., who originally isolated it from the scalp and nasolabial fold of patients with seborrheic dermatitis/dandruff (SD/D) and healthy individuals in northwestern India (2). Honnavar et al. showed that the appearance of *M. arunalokei* colonies was slightly different from those of *M. restricta* and that the average single-cell size of *M. arunalokei* was also larger than that of *M. restricta*, with a mean area of 5.5 versus 3.2 μm^2^, respectively. Additionally, they showed that *M. arunalokei* is catalase negative. This phenotype was previously found in *M. restricta*, whose genome lacks a catalase gene, but was not observed in other *Malassezia* species (2). A comparison of ITS sequences between *M. arunalokei* and *M. restricta* showed 6.4% variation, and a fluorescent amplified fragment length polymorphism (FAFLP) analysis showed only 25% similarity to *M. restricta*, underpinning that *M. arunalokei* is a novel species and differentiated from *M. restricta* (2).

In this study, we sequenced and annotated the genome of the *M. arunalokei* strain NCCPF 127130 (CBS 13387) and compared it with *M. restricta* KCTC 27527 (3, 4). Moreover, we reanalyzed previously reported amplicon sequencing data sets at a species-level resolution to investigate the distribution of *M. arunalokei* within facial skin mycobiota of East Asian individuals (5, 6). We also estimated the time at which *M. arunalokei* diverged within the *Malassezia* genus.

## RESULTS AND DISCUSSION

### Analysis of the *M. arunalokei* genome.

The genomic DNA of *M. arunalokei* NCCPF 127130 (equivalent to CBS 13387 and MTCC 12054) was extracted and sequenced using an Illumina NextSeq. We obtained 19 contigs, resulting in a genome size of 7.24 Mbp. The results of BUSCO analysis are presented in Table 1; the genome assembly is high quality in terms of completeness and contamination compared to the completely assembled genome of *M. restricta* KCTC 27527 (3, 4). However, there are differences in the numbers and lengths of exon and introns, which may be attributed to the use of *M. restricta*'s transcriptome sequencing (RNA-seq) data in gene prediction, which does not fully reflect all existing exon-intron structures. Genome annotation identified 4,117 protein-coding genes and 82 tRNA genes (see Table S3 in the supplemental material). To the best of our knowledge, this is the first study to present high-quality assembly and annotation of *M. arunalokei*, which represents an important contribution to the study of the *Malassezia* genus.

**TABLE 1:** Summary statistics of the *M. arunalokei* genome assembly in comparison with the *M. restricta* genome

Parameter	Result for:	
	*M. arunalokei*	*M. restricta*
Status	Draft	Complete
Total genome size (bp)	7,247,604	7,330,907
No. of contigs	19	9 (1)^*a*^
*N*_50_ (bp)	771,082	1,222,814
GC ratio (%)	55.6	55.8 (31.4)^*a*^
Total no. of tRNAs	82	74 (24)^*a*^
Total no. of CDSs^*b*^	4,117	4,390 (16)^*a*^
CDS length (avg/median)	1,525.4/1,287.0	1,473.8/1,236.0
Exon length (avg/median)	1,147.9/942.0	990.0/711.0
Intron length (avg/median)	68.1/40.0	37.2/30.0
No. of introns	1,354	2,150
No. of exons	5,471	6,556
No. (%) of spliced genes	858 (20.8)	1,194 (27.2)
Gene density (genes/Mb)	568.1	601.1
No. (%) of BUSCOs:		
Complete BUSCOs	712 (93.9)	715 (94.3)
Complete and single-copy BUSCOs	711 (93.8)	714 (94.2)
Complete and duplicated BUSCOs	1 (0.1)	1 (0.1)
Fragmented BUSCOs	6 (0.8)	8 (1.1)
Missing BUSCOs	40 (5.3)	35 (4.6)

a The value in parentheses shows the information for the mitochondrial genome.

b CDSs, coding DNA sequences.

A previous study by Honnavar et al., which was based on ITS sequencing analysis, showed that *M. arunalokei* is phylogenetically clustered with *M. restricta* (2). In their study, *M. restricta* type strain CBS 7877 was used, and the percentage of ITS2 sequence differences between *M. arunalokei* NCCPF 127130 and *M. restricta* CBS 7877 was 8.8%, with eight deletions and 16 substitutions. Our study showed that the identity of the entire 757-bp ITS region, amplified using the primers ITS4 and ITS5 in the *M. arunalokei* genome, was 97%, with eight deletions and nine substitutions and eight deletions and 11 substitutions compared with both *M. restricta* KCTC27527 and *M. restricta* CBS 7877, respectively.

**FIG 1:**
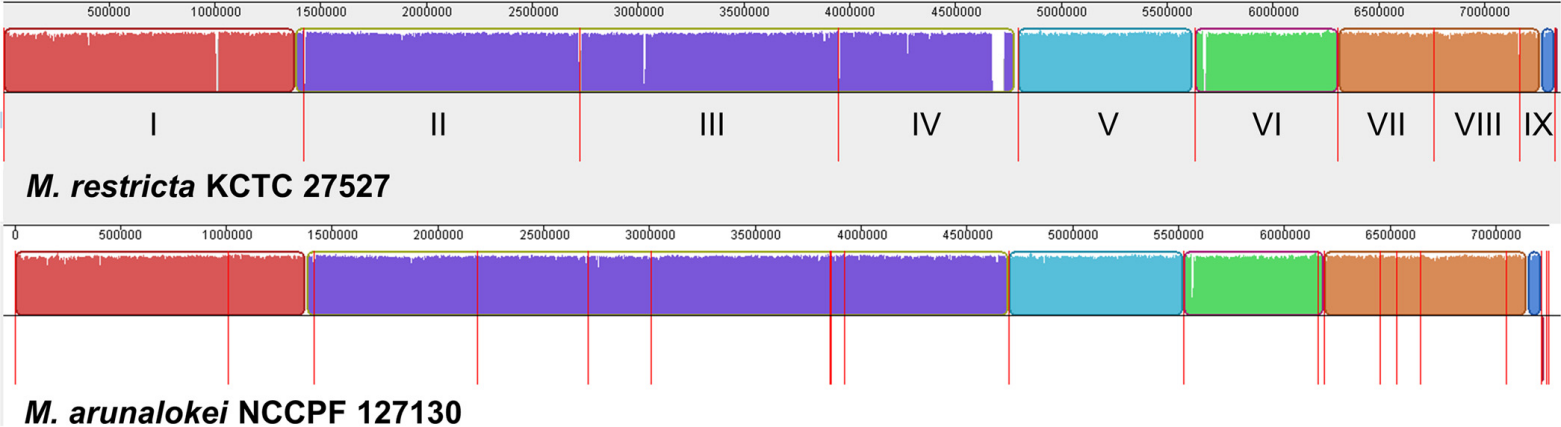
MAUVE genome alignment between *M. restricta* KCTC 27527 and *M. arunalokei* NCCPF 127130. Red lines denote chromosomal or contig partition.

**FIG 2:**
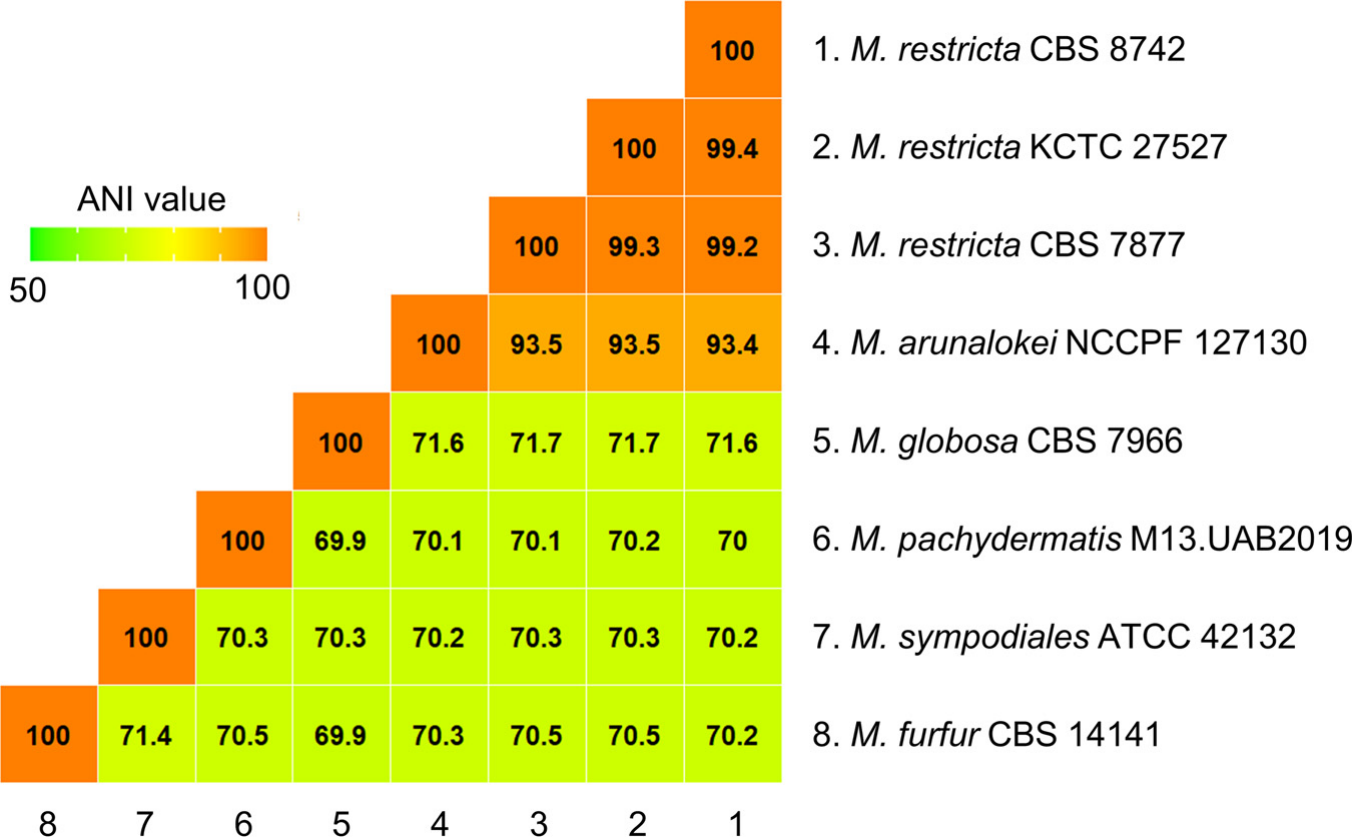
Heat map visualization based on ANI values to find the distance among several representative species of *Malassezia* genus. ANI values were calculated by the ANIb method based on BLAST+ from JSpecies-WS.

In our study, we extended the sequence comparisons between *M. arunalokei* and *M. restricta* to a genome-wide scale. The *M. arunalokei* NCCPF 127130 genome sequence obtained in the current study was compared with *M. restricta* KCTC 27527, which was previously analyzed by our group (3, 4). The results of the genome comparison showed that *M. arunalokei* and *M. restricta* possess highly conserved synteny sequence blocks (Fig. 1). Furthermore, we determined that the average nucleotide identity (ANI) value of the genome of *M. arunalokei* NCCPF 127130 compared with the genomes of the three *M. restricta* strains, including *M. restricta* KCTC 27527, was 93.5 indicating that they are different species (Fig. 2). The ANI value is widely used as a species boundary criterion in prokaryotes, whose genomes are generally comprised of about 10 to 20% intergenic regions (7). The intergenic and intronic sequences of eukaryotic genomes are likely to affect the calculation of the ANI value. Therefore, one may argue that evaluation of ANI values may not be applicable to the comparison of genomes of fungal organisms like the *Malassezia* species. However, we should note that *Malassezia* species have relatively small genomes compared to other fungi, and the proportions of intergenic regions in their genomes are similar to those observed in bacterial genomes. We note that intergenic regions in the *Malassezia* genome were included to calculate the ANI values in our study, and a similar methodology was successfully applied to analyze the evolutionary relatedness of *Malassezia* species in our previous study (4).

**FIG 3:**
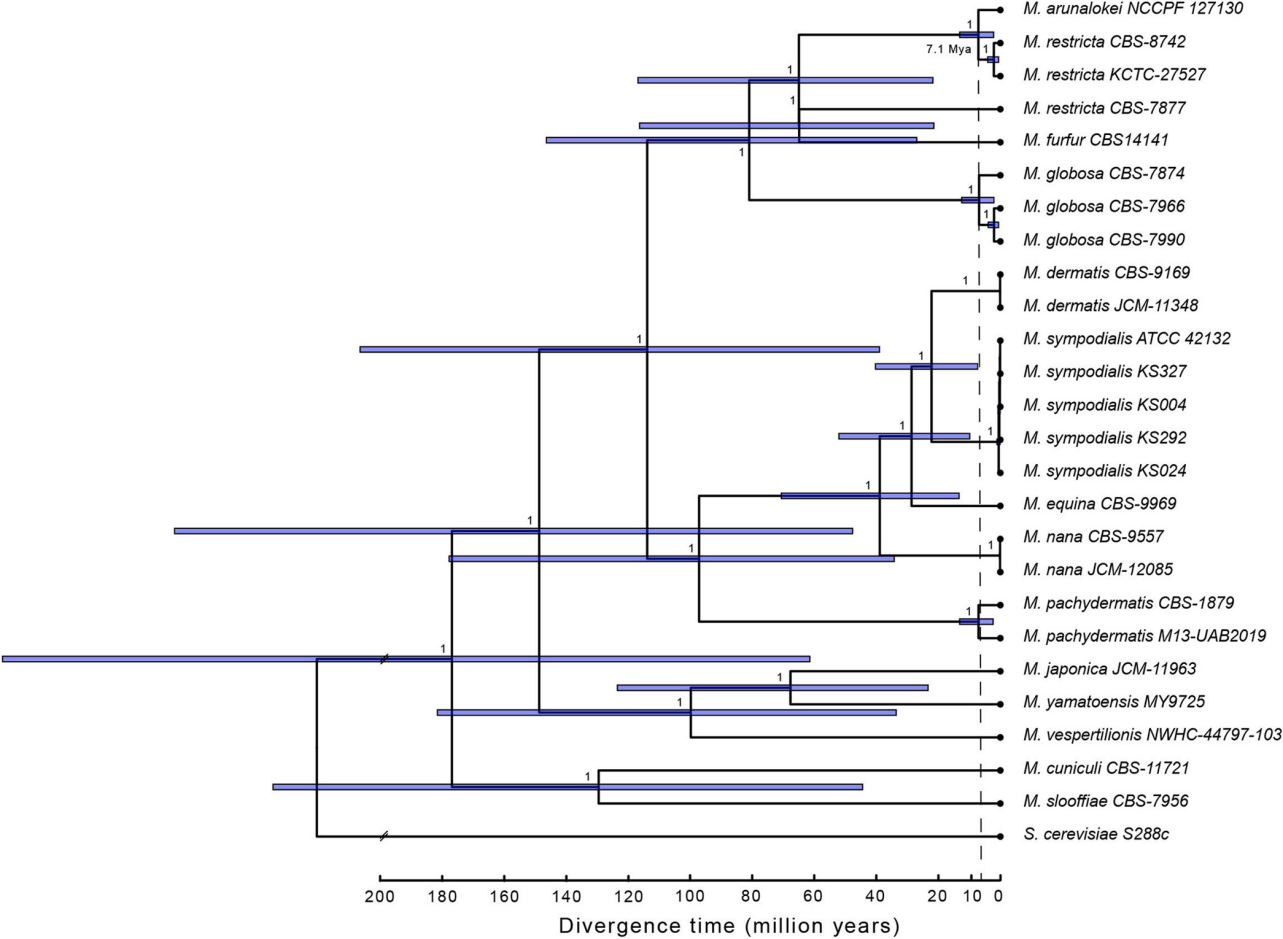
Divergence time estimation. Shown are Bayesian divergence time estimates of *Malassezia* species using a node calibration for the split between *M. furfur* and *M. restricta*. Node numbers identify the posterior probability; blue bars indicate the 95% credible posterior age range. Saccharomyces cerevisiae was used as an outgroup (branch not to scale).

High levels of similarity between the *M. arunalokei* and *M. restricta* genomes led us to estimate divergence times within the *Malassezia* genus. We used seven highly conserved fungal barcoding genes to determine times of divergence, and the tree was produced using Bayesian inference (8). We found the crown age of all included *Malassezia* species for estimating the divergence times to be ~180 million years ago (mya), with the split between *M. arunalokei* NCCPF 127130 and the most closely related *M. restricta* strains (KCTC 27527 and CBS 8742) dating back to 7.1 mya (2.2 to ~13.1 mya) (Fig. 3). Furthermore, the results of our phylogenetic analysis indicated that *M. arunalokei* is the most recently diverged species in the *Malassezia* lineage currently known.

Previous phylogenetic analysis conducted by Honnavar et al. as well as other previous analyses using D1/D2 domains of large subunit (LSU) ribosomal DNA (rDNA) sequences showed that *M. globosa*, *M. restricta*, and *M. arunalokei* are closely clustered within the same clade in the phylograms (1, 2, 9), and our result was in accordance with these findings. However, we also observed two distinct subspecies groups in the *M. restricta* species (*M. restricta* CBS 8741 and KCTC 27527 versus *M. restricta* CBS 7877) in the phylogram we generated. The existence of subspecies groups (amplicon strain variants) of *M. restricta* on the human skin was also described elsewhere (5). To confirm the presence of subspecies groups in genetic levels in detail, more genomes of *M. restricta* strains should be fully sequenced.

### Orthologous gene comparisons between *M. arunalokei* and *M. restricta*.

**FIG 4:**
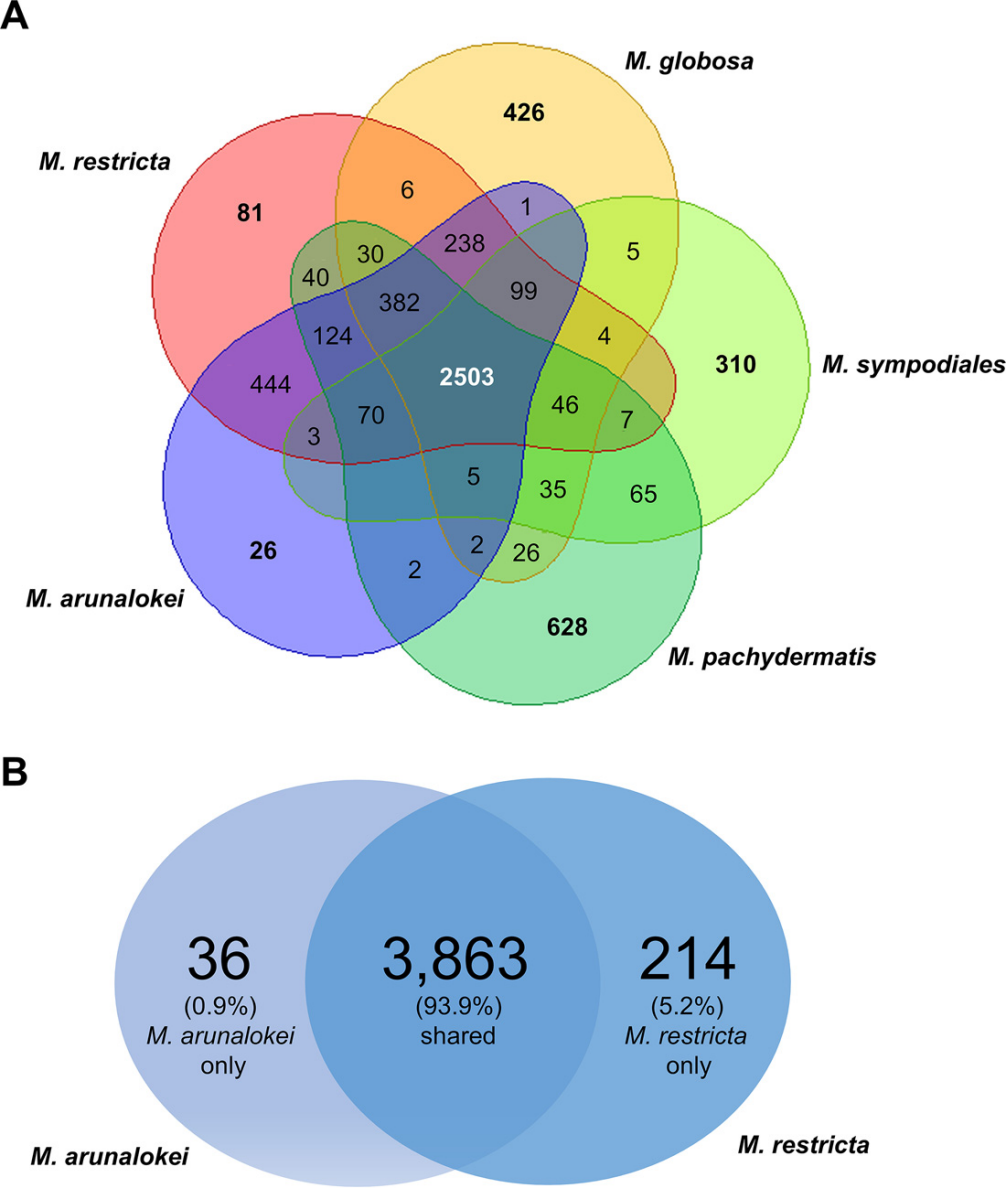
Venn diagram of orthologous gene clusters by mcl algorithm. (A) Number of orthologs between *M. restricta* KCTC 27527, *M. globosa* CBS 7966, *M. sympodiales* ATCC 42132, *M. pachydermatis* CBS 1879, and *M. arunalokei* NCCPF 127130. (B) Venn diagram only showing the number of orthologs between *M. restricta* KCTC 27527 and *M. arunalokei* NCCPF 127130.

As indicated by the whole-genome alignment presented in Fig. 1, most *M. arunalokei* genes are expected to belong to *M. restricta*. To confirm this, we compared orthologous genes between *M. arunalokei* NCCPF 127130 and *M. restricta* KCTC 27527. Type strains of other *Malassezia* species, such as *M. globosa* strain CBS 7966, *M. sympodiales* ATCC 42132, and *M. pachydermatis* CBS 1879, the genomes of which were sequenced and annotated, were also included for more comprehensive analysis. As shown in Table 1, the genome of *M. arunalokei* NCCPF 127130 contains 4,117 protein-coding genes. Overall, most of orthologs in *M. arunalokei* were shared with *M. restricta* (Fig. 4A). Notably, 444 (11.4%) orthologs of *M. arunalokei* were shared with *M. restricta* only, a significantly higher proportion than the number of orthologs shared with other strains (Fig. 4B). In comparison with *M. arunalokei* and *M. restricta* alone, 214 *M. restricta*-specific orthologs were identified against 36 *M. arunalokei*-specific ones, implying that exclusive gene loss occurred in *M. arunalokei* during speciation (Fig. 4A; Table S4). (3, 4). We then closely examined the absent genes in the *M. arunalokei* genome compared with *M. restricta*. Gene Ontology (GO) analysis found no significant enrichment of any GO term in the categories of biological process and molecular function. However, regarding GO terms related to the cellular component category, we discovered that mitochondrial localization was the most enriched term (67 out of 214) among genes absent in the *M. arunalokei* genome compared with *M. restricta*. Although most of the deleted genes encode mitochondrial proteins, we found no defects in the growth of *M. arunalokei* in aerobic environments and under oxidative stress conditions (data not shown).

Among the genes that are absent in *M. arunalokei*, we paid particular attention to the ortholog of *M. restricta* MRET_0913, which encodes imidazole glycerol-phosphate dehydratase (IGPD) required for histidine biosynthesis. IGPD is encoded by *HIS3* in the model yeast Saccharomyces cerevisiae, and deletion of the gene caused histidine auxotrophy of the fungus (10). On human skin, the proteogenic amino acid histidine plays an important role. Histidine is a natural moisturizing factor and is produced from the proteolysis of the histidine-rich protein filaggrin, which is mainly found in granular layer keratinocytes in the upper epidermis (11). Furthermore, studies have shown that histidine supplementation increases the barrier function of the skin and reduces atopic dermatitis symptoms (12, 13). Taken together, we hypothesize that the histidine-rich environment in the epidermal layer may have triggered the loss of the *HIS3* homolog in the *M. arunalokei* lineage.

Such gene loss is consistent with regressive evolution frequently observed in parasitic organisms, which are themselves associated with hosts that show metabolic redundancy when parasitized (14). Gene loss upon environmental change or condition has also been observed, most commonly in unicellular organisms, including pathogenic bacteria and fungi. Furthermore, in some cases, gene loss influenced the pathogenicity of the organisms (14). Examples include loss of the functional BNA genes in a pathogenic yeast, Candida glabrata. The BNA genes are required for nicotinic acid (NA). Therefore, loss of the gene caused NA auxotrophy, which in turn increased expression of the EPA genes encoding a family of adhesin proteins and the pathogenicity of C. glabrata (15). However, we do not have additional evidence to determine whether gene loss in *M. arunalokei*, such as the *HIS3* homolog, is associated with the pathogenicity of the fungus. This is mainly due to the lack of a tool for genetic manipulation.

### *M. arunalokei* has high relative abundance on the forehead and cheek compared to the scalp.

**FIG 5:**
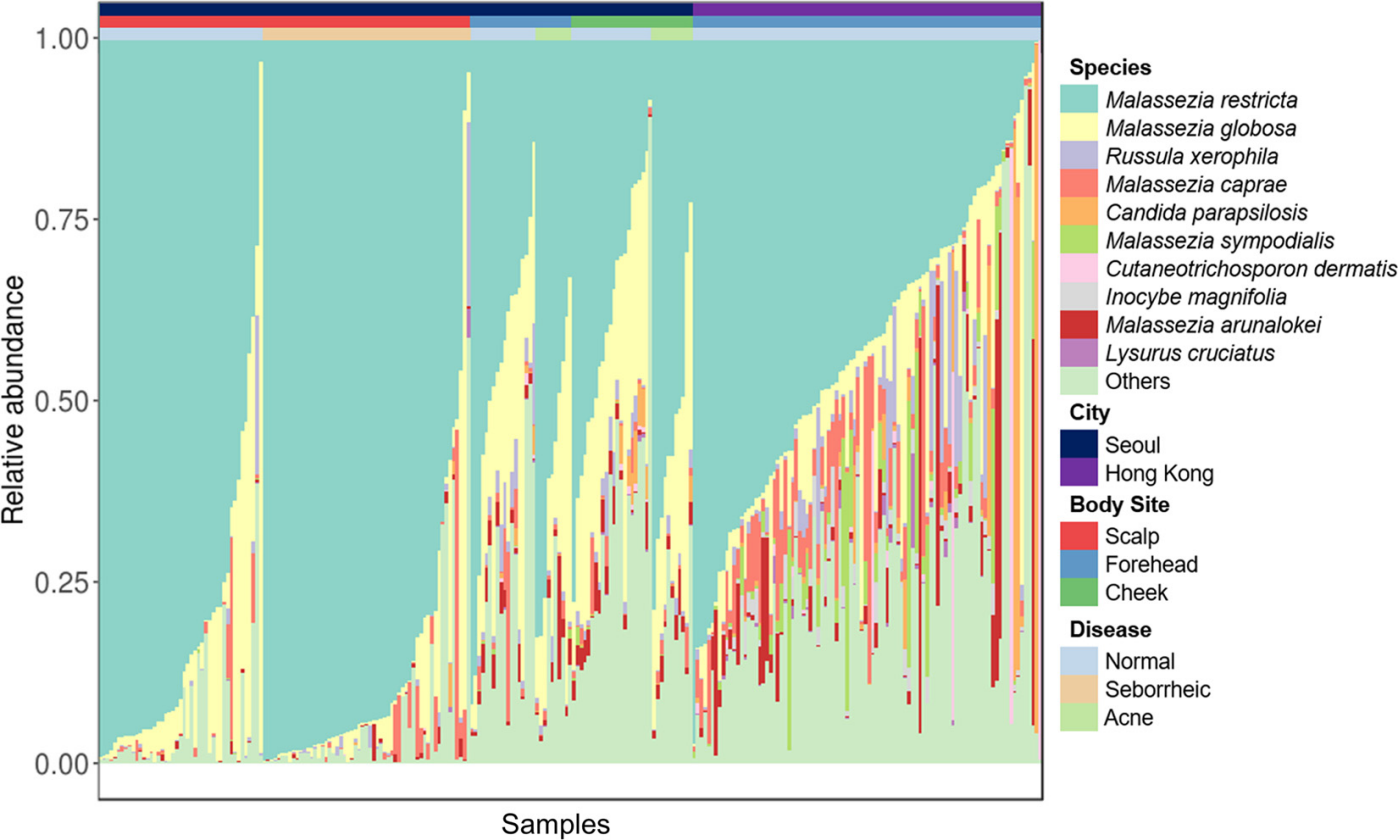
Relative abundance of various fungal species (*y* axis) in samples from different skin sites of the face (labeled on the *x* axis). Each column represents either healthy or diseased individuals. Fungal species, skin site, and affected status are indicated in the legend on the right.

**FIG 6:**
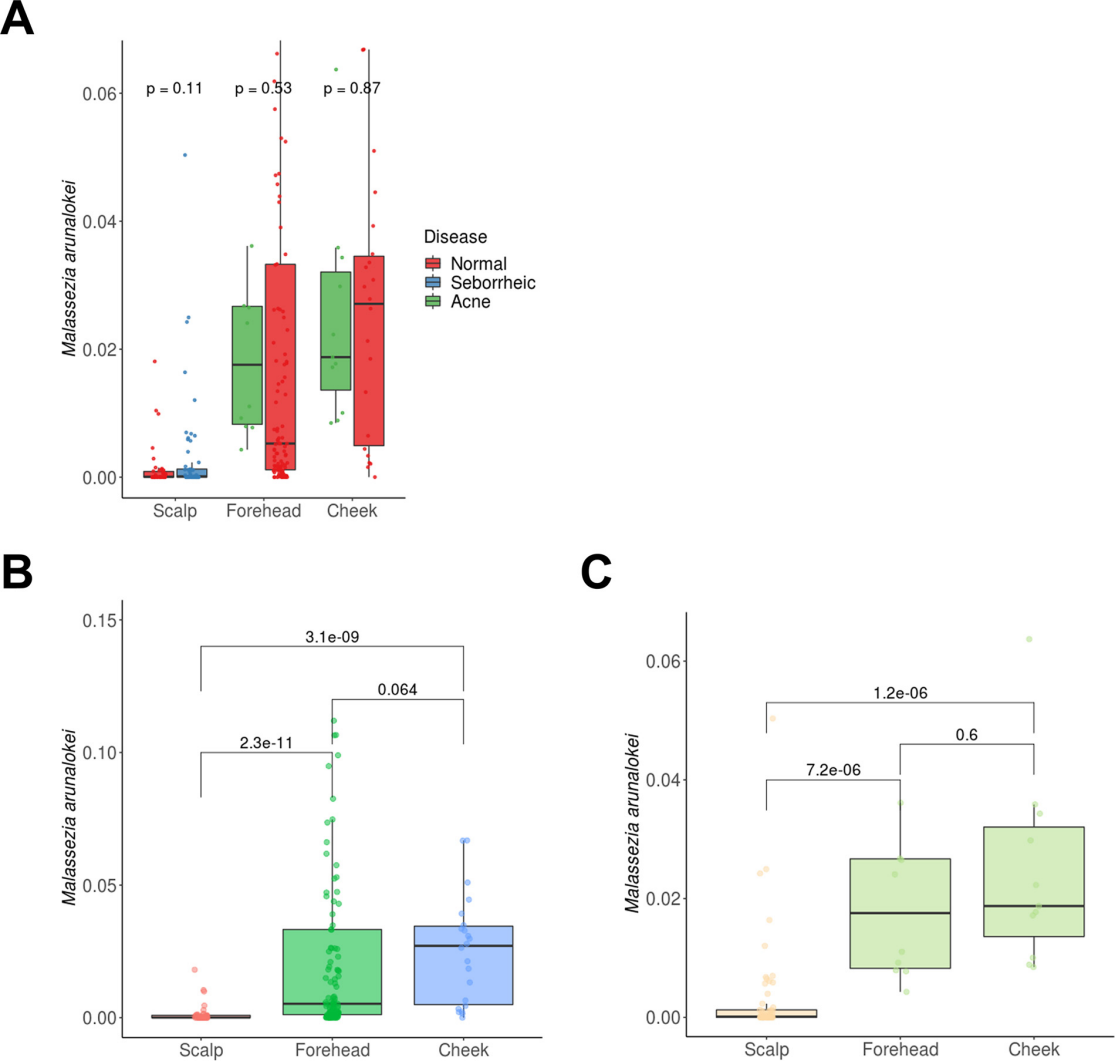
Relative abundance of *M. arunalokei* at different facial skin sites in study cohorts. (A) Relative abundance of *M. arunalokei* between patient (seborrheic or acne) and healthy (normal) groups. (B and C) Relative abundance of *M. arunalokei* at different skin sites (scalp versus forehead and cheeks) in the (B) healthy and (C) patient groups. Values above the bar graphs are *P* values. The numbers of the samples used in the analysis are listed in Table 2.

In our previous studies, we analyzed the mycobiomes on the scalp, forehead, and cheeks of healthy individuals and patients with SD/D or acne residing in Seoul, South Korea, using culture-independent amplicon sequencing (5, 6). These studies analyzed differences in the diversity of fungal communities between the patient groups and healthy groups, but the distribution of *M. arunalokei* among the fungal communities on the scalp, forehead, and cheeks could not be determined. Therefore, we reanalyzed the previously obtained mycobiome data sets at the species level in our current study in order to gain more precise information on the distribution of *M. arunalokei* on the scalp, forehead, and cheeks in our patient group and healthy group. Moreover, in addition to our own data sets, the amplicon sequencing data set generated by Tong et al. (16), who collected samples from multiple skin sites, including the forehead, on a healthy cohort residing Hong Kong, China, were included in the current study to expand the analysis (Table 2). A total of 356 species were detected in our mycobiome analysis. Among them, we assigned 346 species, while the remainder were unassigned (Table S5). The results of our mycobiome analysis showed that *M. arunalokei* was present in 80.7% of samples (209 out of 259), indicating that the fungus is universally present on the scalp, forehead, and cheeks, although its relative abundance is minimal compared with those of other *Malassezia* species (Fig. 5). We next performed a Wilcoxon rank sum test to determine the significance of the *M. arunalokei* abundance with respect to either disease status (SD/D or acne) or specific facial skin site (scalp, forehead, or cheeks). No significant differences in the abundance of *M. arunalokei* were observed between the patient group and the healthy group (Fig. 6A). However, we did find differences between *M. arunalokei* abundance and different skin sites (scalp versus forehead and cheeks). The abundance of *M. arunalokei* on the forehead and cheeks was significantly higher than that on the scalp in both the patient and healthy groups (Fig. 6B and C). These findings indicate that *M. arunalokei* abundance is not related to D/SD or acne but rather to the skin site. We also compared the number of nonchimeric reads with detection frequency of *M. arunalokei* and found no correlation between them (Fig. S1). This result further indicated that skin site has a greater influence on mycobiome composition than sequencing depth.

**TABLE 2:** Occurrence of *M. arunalokei* in swab samples

Parameter	Result for samples from^*a*^:						
	Seoul						Hong Kong, forehead, normal
	Scalp		Forehead		Cheek		
	Normal	SD/D	Normal	Acne	Normal	Acne	
% of occurrences	66.7	54.4	100	100	100	100	90.6
No. of samples	45	57	18	10	22	11	96
No. of occurrences	30	31	18	10	22	11	87

a Samples from Seoul represent facial skin mycobiome data sets generated in our previous study (5, 6). Samples from Hong Kong represent skin mycobiome data sets generated by Tong et al. (16). SD/D, seborrheic dermatitis/dandruff.

Overall, our data showed that *M. arunalokei* is a fungal species possessing a genome highly homologous to that of *M. restricta* and indicated that it is the most recent species in the lineage of *Malassezia* that has adapted to the skin environment. Furthermore, our analysis of the facial mycobiome in humans showed that *M. arunalokei* had distinct niche preferences.

## MATERIALS AND METHODS

### Genomic DNA library construction and sequencing.

*M. arunalokei* NCCPF 127130 was obtained from the Westerdijk Fungal Biodiversity Institute (https://www.knaw.nl/en/institutes/westerdijkinstitute). The strain was cultured, and its genomic DNA was extracted as described previously (4). Genomic DNA was sheared using a g-TUBE device (Covaris, Woburn, MA, USA) to fragments 20 kb in size, and DNA library construction was then performed using the PacBio platform (Pacific Biosciences, Menlo Park, CA, USA) according to the manufacturer’s instructions. Sequencing was performed using a SMRT Cell and PacBio Sequel system (Pacific Biosciences). Additionally, an Illumina sequencing library was constructed using a NEBNext Ultra II FS DNA library prep kit for Illumina (E7805S; New England Biolabs, Ipswich, MA, USA) according to the manufacturer’s instructions, followed by sequencing using a NextSeq 550 (Illumina, San Diego, CA, USA) to generate 2× 76-bp paired-end reads.

### Genome assembly and analysis.

Generated raw sequences were assembled using the hybrid assembler Unicycler v.0.4.8 (17), and the general statistics are shown as Table S1 in the supplemental material. Gene prediction and annotation were performed using FunGAP pipeline v.1.1.0 using previous RNA sequencing data of *M. restricta* KCTC 27527 (3, 18). Protein sequences were clustered for an ortholog search using DIAMOND v.2.0.6, with the parameter “-id 50” (sequence identity) and mcl 14–137 with the parameter “-I 2.0” (inflation value, where higher values indicate more fine-grained clustering) (19). To evaluate the quality of genome assembly, we conducted an analysis using BUSCO v.5.3.0 (20) based on fungi_odb10 (13 December 2019). Genome sequences were aligned using MAUVE v.2.4.0, and their average nucleotide identity (ANI) values were calculated by ANIb method based on BLAST+ from JSpecies-WS (21–23).

### Divergence time estimation.

We performed divergence time estimation based on Bayesian inference between species of the *Malassezia* genus. At first, we collected the genome sequences from the 25 strains belonging to 14 different species (Table S2). In addition, we used Saccharomyces cerevisiae S288c (BioProject accession no. PRJEB7245; assembly accession no. GCA_002057635.1) as an outgroup. Amino acid sequences of seven highly conserved fungal barcoding proteins were selected, namely, RPB2 (DNA-directed, RNA polymerase II subunit), phospholipase, lipase, actin, MCM7 (DNA replication licensing factor MCM7), TEF1 (elongation factor 1-alpha), and TUBB1 (beta-tubulin). We performed tBLASTn to target genomes using the barcoding protein sequences of *M. arunalokei* as queries and then extracted the resulting aligned sequences.

Amino acid sequences were aligned using MAFFT v.7.490 (24) with the “–maxiterate 1000” option used to refine the alignment. Aligned sequences were trimmed using CLIPKIT v.1.3.0 (25) using the smart-gap algorithm, which led to the removal of 110 sites, giving a final total alignment length of 4,753 sites.

BEAUti was used to define the parameter file for BEAST v.2.6.6 (26). We set the substitution model to Blosum62 with a gamma site model and estimated the substitution rate. The clock model was defined as strict. We used the Calibrated Yule model as the tree prior and estimated the birth rate. As an external calibration, we defined the split between *M. furfur* and *M. restricta* as ~50 million years ago (mya) (8) with a log-normal distribution. We centered the distribution at 4.1 (M) with a standard deviation of 0.4 (S) to reflect the uncertainty about the calibration. We set the birth rate and clock rate to a gamma distribution with the alpha and beta values set at 0.001 and 1,000, respectively. The Markov chain Monte Carlo (MCMC) was defined with a chain length of 10,000,000 and no pre-burn-in. We inspected BEAST output using Tracer v.1.7.4 (27) and checked whether the specified divergence prior matched the posterior distribution. We used TreeAnnotator v.2.6.6 (26) to identify the tree with the highest product of the posterior probability of all its nodes using the maximum clade credibility tree with mean heights as node heights. We visualized the final tree using FigTree v.1.4.4 (https://github.com/rambaut/figtree).

### Skin mycobiome data analysis.

Facial skin mycobiome data based on ITS1 amplicon sequencing were retrieved from NCBI (Seoul, scalp, PRJNA335788; Seoul, forehead and cheek, PRJNA673754; Hong Kong, forehead, PRJNA421247) and processed in this study (5, 6, 16). Adapter and primer sequences (18S-F and 5.8S-1R) (28) in data sets were trimmed from raw paired-end reads using cutadapt (v.3.2) with default parameters (29). Length trimming was performed using forward and reverse reads to 250 bp and 180 bp, respectively.

To analyze the amplicon sequence variants (ASVs), the trimmed, paired-end reads were denoised and merged using the R package DADA2 (v.1.18.0) with a default parameter (30). A total of 5,955 ASVs with 13,595,931 reads remained after chimeras were removed using the “consensus” method. For taxonomic classification of the resulting ASVs, we applied the top BLASTN hits (NCBI-BLAST-2.10.1+) in the NCBI ITS database (NCBI Targeted Loci project, downloaded 1 December 2021) (31, 32) to discover *M. arunalokei* instead of the Bayesian classifier (33). The Bayesian classifier uses the bootstrap value from the Bayesian algorithm as the taxonomy confidence level. However, depending on the database, the bootstrap value may be biased to one side that has more data. The more *M. restricta* sequences in the NCBI database than *M. arunalokei*, the more the Bayesian classifier assigns a query sequence to *M. restricta*, although the query sequence has higher bitscore with *M. arunalokei*. For statistical analysis, the Wilcoxon rank sum test was performed using R (v.4.0.4).

### Data availability.

The genome sequence of *M. arunalokei* NCCPF 127130 has been deposited in the National Center for Biotechnology Information (NCBI) under the accession no. PRJNA751706.
