# Supplementary material for: Genome of Malassezia arunalokei and Its Distribution on Facial Skin
Source: Microbiol Spectr. 2022 Jun 1;10(3):e00506-22. doi: 10.1128/spectrum.00506-22 (PMC9241646; doi:10.1128/spectrum.00506-22)
Supplement: SUPPLEMENTAL FILE 1 — Supplemental material. Download spectrum.00506-22-s0001.pdf, PDF file, 6.8 MB [file spectrum.00506-22-s0001.pdf]

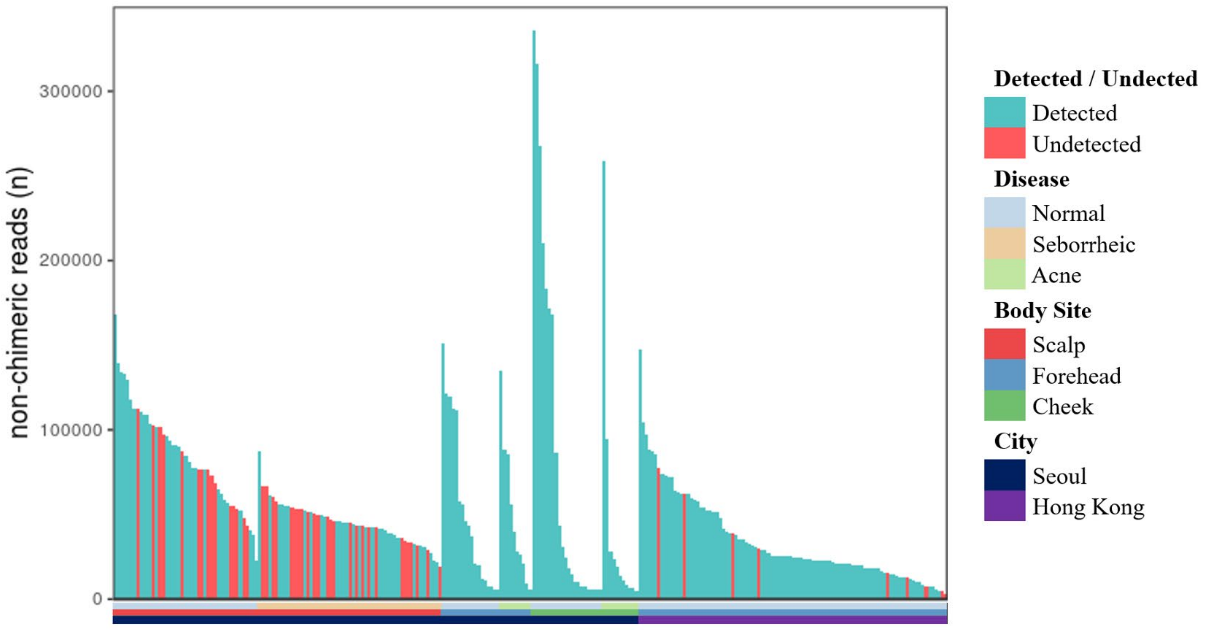

Figure S1. Analysis of *M. arunalokei* detection according to read depths (total non-chimeric reads)

Table S1. Genome assembly statistics

Attributes	Illumina	PacBio
Total bases (Mbp)	2,901	1,902
No. of raw reads	38,171,004	107,389
No. of filtered reads	37,873,126	-
N50 Length of raw reads (bp)	76	27,273
Mean Length of raw reads (bp)	76	17,696
Coverage (X)	368.8	117.3

Supplementary Table S2. **List of species and isolates included in the divergence time dating.**

Malassezia arunalokei NCCPF 127130
Malassezia cuniculi CBS-11721
Malassezia dermatis CBS-9169
Malassezia dermatis JCM-11348
Malassezia equina CBS-9969
Malassezia furfur CBS14141
Malassezia globosa CBS-7874
Malassezia globosa CBS-7966
Malassezia globosa CBS-7990
Malassezia japonica JCM-11963
Malassezia nana CBS-9557
Malassezia nana JCM-12085
Malassezia pachydermatis CBS-1879
Malassezia pachydermatis M13-UAB2019
Malassezia restricta CBS-7877
Malassezia restricta CBS-8742
Malassezia restricta KCTC-27527
Malassezia slooffiae CBS-7956
Malassezia sympodialis ATCC-42132
Malassezia sympodialis KS004
Malassezia sympodialis KS024
Malassezia sympodialis KS292
Malassezia sympodialis KS327
Malassezia vespertilionis NWHC-44797-103
Malassezia yamatoensis MY9725
Saccharomyces cerevisiae S288c

Supplementary Table S3. Annotation information.

##gff-version 3
##sequence-region contig001 1 1007197
##sequence-region contig002 1 408293
##sequence-region contig003 1 771082
##sequence-region contig004 1 523505
##sequence-region contig005 1 298276
##sequence-region contig006 1 843590
##sequence-region contig007 1 4573
##sequence-region contig008 1 65905
##sequence-region contig009 1 773937
##sequence-region contig010 1 826862
##sequence-region contig011 1 638652
##sequence-region contig012 1 27372
##sequence-region contig013 1 263122
##sequence-region contig014 1 79918
##sequence-region contig015 1 110976
##sequence-region contig016 1 404011
##sequence-region contig017 1 166469
##sequence-region contig018 1 26165
##sequence-region contig019 1 7699

contig001	annotation	remark	1	1E+06	.	+	.	gff-version=3
contig001	AUGUSTUS	gene	270	934	0.23	+	.	ID=MALK_00001;prediction_source=braker_MRET:g672.t1
contig001	AUGUSTUS	CDS	270	406	0.6	+	0	ID=MALK_00001.t1.c1;Parent=MALK_00001.t1
contig001	AUGUSTUS	CDS	490	658	0.6	+	0	ID=MALK_00001.t1.c2;Parent=MALK_00001.t1
contig001	AUGUSTUS	CDS	708	772	0.6	+	0	ID=MALK_00001.t1.c3;Parent=MALK_00001.t1
contig001	AUGUSTUS	CDS	802	934	0.6	+	0	ID=MALK_00001.t1.c4;Parent=MALK_00001.t1
contig001	AUGUSTUS	mRNA	270	934	0.23	+	.	ID=MALK_00001.t1;Parent=MALK_00001
contig001	AUGUSTUS	exon	270	406	.	+	.	ID=MALK_00001.t1.e1;Parent=MALK_00001.t1
contig001	AUGUSTUS	exon	490	658	.	+	.	ID=MALK_00001.t1.e2;Parent=MALK_00001.t1
contig001	AUGUSTUS	exon	708	772	.	+	.	ID=MALK_00001.t1.e3;Parent=MALK_00001.t1
contig001	AUGUSTUS	exon	802	934	.	+	.	ID=MALK_00001.t1.e4;Parent=MALK_00001.t1
contig001	maker	gene	1980	3236	.	+	.	ID=MALK_00002;prediction_source=maker_MRET:augustus_masked-contig001-processed-gene-0.29-mRNA-1
contig001	maker	CDS	1980	3236	.	+	0	ID=MALK_00002.t1.c1;Parent=MALK_00002.t1
contig001	maker	mRNA	1980	3236	.	+	.	ID=MALK_00002.t1;Parent=MALK_00002
contig001	maker	exon	1980	3236	.	+	.	ID=MALK_00002.t1.e1;Parent=MALK_00002.t1
contig001	AUGUSTUS	gene	4577	6256	1	-	.	ID=MALK_00003;prediction_source=augustus:contig001.g316.t1
contig001	AUGUSTUS	CDS	4577	6256	1	-	0	ID=MALK_00003.t1.c1;Parent=MALK_00003.t1
contig001	AUGUSTUS	mRNA	4577	6256	1	-	.	ID=MALK_00003.t1;Parent=MALK_00003
contig001	AUGUSTUS	exon	4577	6256	1	-	.	ID=MALK_00003.t1.e1;Parent=MALK_00003.t1
contig001	AUGUSTUS	gene	7173	8642	0.98	+	.	ID=MALK_00004;prediction_source=augustus:contig001.g318.t1
contig001	AUGUSTUS	CDS	7173	8642	0.98	+	0	ID=MALK_00004.t1.c1;Parent=MALK_00004.t1
contig001	AUGUSTUS	mRNA	7173	8642	0.98	+	.	ID=MALK_00004.t1;Parent=MALK_00004

contig001	AUGUSTUS	exon	7173	8642	0.98	+	.	ID=MALK_00004.t1.e1;Parent=MALK_00004.t1
contig001	maker	gene	8879	11272	.	+	.	ID=MALK_00005;prediction_source=maker_MRET:augustus_masked-contig001-processed-gene-0.31-mRNA-1
contig001	maker	CDS	8879	11272	.	+	0	ID=MALK_00005.t1.c1;Parent=MALK_00005.t1
contig001	maker	mRNA	8879	11272	.	+	.	ID=MALK_00005.t1;Parent=MALK_00005
contig001	maker	exon	8879	11272	.	+	.	ID=MALK_00005.t1.e1;Parent=MALK_00005.t1
contig001	AUGUSTUS	gene	11573	13111	0.95	+	.	ID=MALK_00006;prediction_source=augustus:contig001.g320.t1
contig001	AUGUSTUS	CDS	11573	13111	0.95	+	0	ID=MALK_00006.t1.c1;Parent=MALK_00006.t1
contig001	AUGUSTUS	mRNA	11573	13111	0.95	+	.	ID=MALK_00006.t1;Parent=MALK_00006
contig001	AUGUSTUS	exon	11573	13111	0.95	+	.	ID=MALK_00006.t1.e1;Parent=MALK_00006.t1
contig001	AUGUSTUS	gene	13114	15390	0.78	-	.	ID=MALK_00007;prediction_source=augustus:contig001.g321.t1
contig001	AUGUSTUS	CDS	13114	15390	0.78	-	0	ID=MALK_00007.t1.c1;Parent=MALK_00007.t1
contig001	AUGUSTUS	mRNA	13114	15390	0.78	-	.	ID=MALK_00007.t1;Parent=MALK_00007
contig001	AUGUSTUS	exon	13114	15390	0.78	-	.	ID=MALK_00007.t1.e1;Parent=MALK_00007.t1
contig001	maker	gene	15548	17551	.	+	.	ID=MALK_00008;prediction_source=maker_MRET:augustus_masked-contig001-processed-gene-0.33-mRNA-1
contig001	maker	CDS	15548	17551	.	+	0	ID=MALK_00008.t1.c1;Parent=MALK_00008.t1
contig001	maker	mRNA	15548	17551	.	+	.	ID=MALK_00008.t1;Parent=MALK_00008
contig001	maker	exon	15548	17551	.	+	.	ID=MALK_00008.t1.e1;Parent=MALK_00008.t1
contig001	maker	gene	17563	19011	.	-	.	ID=MALK_00009;prediction_source=maker_MRET:augustus_masked-contig001-processed-gene-0.54-mRNA-1
contig001	maker	CDS	17563	19011	.	-	0	ID=MALK_00009.t1.c1;Parent=MALK_00009.t1
contig001	maker	mRNA	17563	19011	.	-	.	ID=MALK_00009.t1;Parent=MALK_00009
contig001	maker	exon	17563	19011	.	-	.	ID=MALK_00009.t1.e1;Parent=MALK_00009.t1
contig001	maker	gene	19111	22206	.	+	.	ID=MALK_00010;prediction_source=maker_MRET:augustus_masked-contig001-processed-gene-0.34-mRNA-1
contig001	maker	CDS	19111	22206	.	+	0	ID=MALK_00010.t1.c1;Parent=MALK_00010.t1
contig001	maker	mRNA	19111	22206	.	+	.	ID=MALK_00010.t1;Parent=MALK_00010
contig001	maker	exon	19111	22206	.	+	.	ID=MALK_00010.t1.e1;Parent=MALK_00010.t1
contig001	maker	gene	22303	23685	.	+	.	ID=MALK_00011;prediction_source=maker_MRET:augustus_masked-contig001-processed-gene-0.35-mRNA-1
contig001	maker	CDS	22303	23685	.	+	0	ID=MALK_00011.t1.c1;Parent=MALK_00011.t1
contig001	maker	mRNA	22303	23685	.	+	.	ID=MALK_00011.t1;Parent=MALK_00011
contig001	maker	exon	22303	23685	.	+	.	ID=MALK_00011.t1.e1;Parent=MALK_00011.t1
contig001	AUGUSTUS	gene	23691	30806	1	-	.	ID=MALK_00012;prediction_source=augustus:contig001.g326.t1
contig001	AUGUSTUS	CDS	23691	30806	1	-	0	ID=MALK_00012.t1.c1;Parent=MALK_00012.t1
contig001	AUGUSTUS	mRNA	23691	30806	1	-	.	ID=MALK_00012.t1;Parent=MALK_00012
contig001	AUGUSTUS	exon	23691	30806	1	-	.	ID=MALK_00012.t1.e1;Parent=MALK_00012.t1
contig001	maker	gene	30975	31614	.	-	.	ID=MALK_00013;prediction_source=maker_MRET:augustus_masked-contig001-processed-gene-0.56-mRNA-1
contig001	maker	CDS	31270	31614	.	-	0	ID=MALK_00013.t1.c1;Parent=MALK_00013.t1
contig001	maker	CDS	30975	31187	.	-	0	ID=MALK_00013.t1.c2;Parent=MALK_00013.t1
contig001	maker	mRNA	30975	31614	.	-	.	ID=MALK_00013.t1;Parent=MALK_00013
contig001	maker	exon	31270	31614	.	-	.	ID=MALK_00013.t1.e1;Parent=MALK_00013.t1
contig001	maker	exon	30975	31187	.	-	.	ID=MALK_00013.t1.e2;Parent=MALK_00013.t1
contig001	maker	gene	31779	32249	.	-	.	ID=MALK_00014;prediction_source=maker_MRET:augustus_masked-contig001-processed-gene-0.57-mRNA-1
contig001	maker	CDS	31779	32249	.	-	0	ID=MALK_00014.t1.c1;Parent=MALK_00014.t1
contig001	maker	mRNA	31779	32249	.	-	.	ID=MALK_00014.t1;Parent=MALK_00014
contig001	maker	exon	31779	32249	.	-	.	ID=MALK_00014.t1.e1;Parent=MALK_00014.t1

contig001	AUGUSTUS	gene	33211	33509	0.66	-	.	ID=MALK_00015;prediction_source=braker_MRET:g684.t1
contig001	AUGUSTUS	CDS	33309	33509	0.87	-	0	ID=MALK_00015.t1.c2;Parent=MALK_00015.t1
contig001	AUGUSTUS	CDS	33211	33270	0.87	-	0	ID=MALK_00015.t1.c1;Parent=MALK_00015.t1
contig001	AUGUSTUS	mRNA	33211	33509	0.66	-	.	ID=MALK_00015.t1;Parent=MALK_00015
contig001	AUGUSTUS	exon	33309	33509	.	-	.	ID=MALK_00015.t1.e2;Parent=MALK_00015.t1
contig001	AUGUSTUS	exon	33211	33270	.	-	.	ID=MALK_00015.t1.e1;Parent=MALK_00015.t1
contig001	maker	gene	33997	35349	.	+	.	ID=MALK_00016;prediction_source=maker_MRET:augustus_masked-contig001-processed-gene-0.36-mRNA-1
contig001	maker	CDS	33997	34347	.	+	0	ID=MALK_00016.t1.c1;Parent=MALK_00016.t1
contig001	maker	CDS	34411	35349	.	+	0	ID=MALK_00016.t1.c2;Parent=MALK_00016.t1
contig001	maker	mRNA	33997	35349	.	+	.	ID=MALK_00016.t1;Parent=MALK_00016
contig001	maker	exon	33997	34347	.	+	.	ID=MALK_00016.t1.e1;Parent=MALK_00016.t1
contig001	maker	exon	34411	35349	.	+	.	ID=MALK_00016.t1.e2;Parent=MALK_00016.t1
contig001	AUGUSTUS	gene	35982	36428	0.79	+	.	ID=MALK_00017;prediction_source=augustus:contig001.g330.t1
contig001	AUGUSTUS	CDS	35982	36428	0.79	+	0	ID=MALK_00017.t1.c1;Parent=MALK_00017.t1
contig001	AUGUSTUS	mRNA	35982	36428	0.79	+	.	ID=MALK_00017.t1;Parent=MALK_00017
contig001	AUGUSTUS	exon	35982	36428	0.79	+	.	ID=MALK_00017.t1.e1;Parent=MALK_00017.t1
contig001	maker	gene	36845	37999	.	+	.	ID=MALK_00018;prediction_source=maker_MRET:augustus_masked-contig001-processed-gene-0.38-mRNA-1
contig001	maker	CDS	36845	37999	.	+	0	ID=MALK_00018.t1.c1;Parent=MALK_00018.t1
contig001	maker	mRNA	36845	37999	.	+	.	ID=MALK_00018.t1;Parent=MALK_00018
contig001	maker	exon	36845	37999	.	+	.	ID=MALK_00018.t1.e1;Parent=MALK_00018.t1
contig001	AUGUSTUS	gene	38016	40376	0.97	-	.	ID=MALK_00019;prediction_source=augustus:contig001.g332.t1
contig001	AUGUSTUS	CDS	38016	40376	0.97	-	0	ID=MALK_00019.t1.c1;Parent=MALK_00019.t1
contig001	AUGUSTUS	mRNA	38016	40376	0.97	-	.	ID=MALK_00019.t1;Parent=MALK_00019
contig001	AUGUSTUS	exon	38016	40376	0.97	-	.	ID=MALK_00019.t1.e1;Parent=MALK_00019.t1
contig001	AUGUSTUS	gene	42285	43199	1	+	.	ID=MALK_00020;prediction_source=augustus:contig001.g333.t1
contig001	AUGUSTUS	CDS	42285	43199	1	+	0	ID=MALK_00020.t1.c1;Parent=MALK_00020.t1
contig001	AUGUSTUS	mRNA	42285	43199	1	+	.	ID=MALK_00020.t1;Parent=MALK_00020
contig001	AUGUSTUS	exon	42285	43199	1	+	.	ID=MALK_00020.t1.e1;Parent=MALK_00020.t1
contig001	AUGUSTUS	gene	43718	45844	0.96	+	.	ID=MALK_00021;prediction_source=augustus:contig001.g335.t1
contig001	AUGUSTUS	CDS	43718	45844	0.96	+	0	ID=MALK_00021.t1.c1;Parent=MALK_00021.t1
contig001	AUGUSTUS	mRNA	43718	45844	0.96	+	.	ID=MALK_00021.t1;Parent=MALK_00021
contig001	AUGUSTUS	exon	43718	45844	0.96	+	.	ID=MALK_00021.t1.e1;Parent=MALK_00021.t1
contig001	maker	gene	46131	48188	.	+	.	ID=MALK_00022;prediction_source=maker_MRET:augustus_masked-contig001-processed-gene-0.41-mRNA-1
contig001	maker	CDS	46131	48188	.	+	0	ID=MALK_00022.t1.c1;Parent=MALK_00022.t1
contig001	maker	mRNA	46131	48188	.	+	.	ID=MALK_00022.t1;Parent=MALK_00022
contig001	maker	exon	46131	48188	.	+	.	ID=MALK_00022.t1.e1;Parent=MALK_00022.t1
contig001	AUGUSTUS	gene	48197	50044	1	-	.	ID=MALK_00023;prediction_source=augustus:contig001.g338.t1
contig001	AUGUSTUS	CDS	48197	50044	1	-	0	ID=MALK_00023.t1.c1;Parent=MALK_00023.t1
contig001	AUGUSTUS	mRNA	48197	50044	1	-	.	ID=MALK_00023.t1;Parent=MALK_00023
contig001	AUGUSTUS	exon	48197	50044	1	-	.	ID=MALK_00023.t1.e1;Parent=MALK_00023.t1
contig001	AUGUSTUS	gene	50289	50713	1	+	.	ID=MALK_00024;prediction_source=braker_MRET:g692.t1
contig001	AUGUSTUS	CDS	50289	50651	1	+	0	ID=MALK_00024.t1.c1;Parent=MALK_00024.t1
contig001	AUGUSTUS	CDS	50693	50713	1	+	0	ID=MALK_00024.t1.c2;Parent=MALK_00024.t1

contig001	AUGUSTUS	mRNA	50289	50713	1	+	.	ID=MALK_00024.t1;Parent=MALK_00024
contig001	AUGUSTUS	exon	50289	50651	.	+	.	ID=MALK_00024.t1.e1;Parent=MALK_00024.t1
contig001	AUGUSTUS	exon	50693	50713	.	+	.	ID=MALK_00024.t1.e2;Parent=MALK_00024.t1
contig001	AUGUSTUS	gene	50865	52004	1	-	.	ID=MALK_00025;prediction_source=augustus:contig001.g340.t1
contig001	AUGUSTUS	CDS	50865	52004	1	-	0	ID=MALK_00025.t1.c1;Parent=MALK_00025.t1
contig001	AUGUSTUS	mRNA	50865	52004	1	-	.	ID=MALK_00025.t1;Parent=MALK_00025
contig001	AUGUSTUS	exon	50865	52004	1	-	.	ID=MALK_00025.t1.e1;Parent=MALK_00025.t1
contig001	AUGUSTUS	gene	52176	56159	0.99	-	.	ID=MALK_00026;prediction_source=augustus:contig001.g342.t1
contig001	AUGUSTUS	CDS	52176	56159	0.99	-	0	ID=MALK_00026.t1.c1;Parent=MALK_00026.t1
contig001	AUGUSTUS	mRNA	52176	56159	0.99	-	.	ID=MALK_00026.t1;Parent=MALK_00026
contig001	AUGUSTUS	exon	52176	56159	0.99	-	.	ID=MALK_00026.t1.e1;Parent=MALK_00026.t1
contig001	AUGUSTUS	gene	56368	58653	0.76	+	.	ID=MALK_00027;prediction_source=augustus:contig001.g344.t1
contig001	AUGUSTUS	CDS	56368	58653	0.76	+	0	ID=MALK_00027.t1.c1;Parent=MALK_00027.t1
contig001	AUGUSTUS	mRNA	56368	58653	0.76	+	.	ID=MALK_00027.t1;Parent=MALK_00027
contig001	AUGUSTUS	exon	56368	58653	0.76	+	.	ID=MALK_00027.t1.e1;Parent=MALK_00027.t1
contig001	maker	gene	58665	61220	.	-	.	ID=MALK_00028;prediction_source=maker_MRET:augustus_masked-contig001-processed-gene-0.62-mRNA-1
contig001	maker	CDS	58665	61220	.	-	0	ID=MALK_00028.t1.c1;Parent=MALK_00028.t1
contig001	maker	mRNA	58665	61220	.	-	.	ID=MALK_00028.t1;Parent=MALK_00028
contig001	maker	exon	58665	61220	.	-	.	ID=MALK_00028.t1.e1;Parent=MALK_00028.t1
contig001	AUGUSTUS	gene	61740	65018	0.46	-	.	ID=MALK_00029;prediction_source=augustus:contig001.g346.t1
contig001	AUGUSTUS	CDS	61740	65018	0.46	-	0	ID=MALK_00029.t1.c1;Parent=MALK_00029.t1
contig001	AUGUSTUS	mRNA	61740	65018	0.46	-	.	ID=MALK_00029.t1;Parent=MALK_00029
contig001	AUGUSTUS	exon	61740	65018	0.46	-	.	ID=MALK_00029.t1.e1;Parent=MALK_00029.t1
contig001	AUGUSTUS	gene	65068	66939	0.88	-	.	ID=MALK_00030;prediction_source=braker_MRET:g697.t1
contig001	AUGUSTUS	CDS	65068	66939	0.88	-	0	ID=MALK_00030.t1.c1;Parent=MALK_00030.t1
contig001	AUGUSTUS	mRNA	65068	66939	0.88	-	.	ID=MALK_00030.t1;Parent=MALK_00030
contig001	AUGUSTUS	exon	65068	66939	.	-	.	ID=MALK_00030.t1.e1;Parent=MALK_00030.t1
contig001	AUGUSTUS	gene	68511	72203	1	+	.	ID=MALK_00031;prediction_source=augustus:contig001.g348.t1
contig001	AUGUSTUS	CDS	68511	72203	1	+	0	ID=MALK_00031.t1.c1;Parent=MALK_00031.t1
contig001	AUGUSTUS	mRNA	68511	72203	1	+	.	ID=MALK_00031.t1;Parent=MALK_00031
contig001	AUGUSTUS	exon	68511	72203	1	+	.	ID=MALK_00031.t1.e1;Parent=MALK_00031.t1
contig001	AUGUSTUS	gene	72733	73956	0.44	+	.	ID=MALK_00032;prediction_source=augustus:contig001.g349.t1
contig001	AUGUSTUS	CDS	72733	73956	0.44	+	0	ID=MALK_00032.t1.c1;Parent=MALK_00032.t1
contig001	AUGUSTUS	mRNA	72733	73956	0.44	+	.	ID=MALK_00032.t1;Parent=MALK_00032
contig001	AUGUSTUS	exon	72733	73956	0.44	+	.	ID=MALK_00032.t1.e1;Parent=MALK_00032.t1
contig001	AUGUSTUS	gene	74053	75285	0.61	+	.	ID=MALK_00033;prediction_source=augustus:contig001.g350.t1
contig001	AUGUSTUS	CDS	74053	75285	0.61	+	0	ID=MALK_00033.t1.c1;Parent=MALK_00033.t1
contig001	AUGUSTUS	mRNA	74053	75285	0.61	+	.	ID=MALK_00033.t1;Parent=MALK_00033
contig001	AUGUSTUS	exon	74053	75285	0.61	+	.	ID=MALK_00033.t1.e1;Parent=MALK_00033.t1
contig001	maker	gene	75462	76211	.	+	.	ID=MALK_00034;prediction_source=maker_MRET:augustus_masked-contig001-processed-gene-0.47-mRNA-1
contig001	maker	CDS	75462	76211	.	+	0	ID=MALK_00034.t1.c1;Parent=MALK_00034.t1
contig001	maker	mRNA	75462	76211	.	+	.	ID=MALK_00034.t1;Parent=MALK_00034
contig001	maker	exon	75462	76211	.	+	.	ID=MALK_00034.t1.e1;Parent=MALK_00034.t1

contig001	maker	gene	76499	78214	.	+	.	ID=MALK_00035;prediction_source=maker_MRET:augustus_masked-contig001-processed-gene-0.48-mRNA-1
contig001	maker	CDS	76499	77548	.	+	0	ID=MALK_00035.t1.c1;Parent=MALK_00035.t1
contig001	maker	CDS	77828	78214	.	+	0	ID=MALK_00035.t1.c2;Parent=MALK_00035.t1
contig001	maker	mRNA	76499	78214	.	+	.	ID=MALK_00035.t1;Parent=MALK_00035
contig001	maker	exon	76499	77548	.	+	.	ID=MALK_00035.t1.e1;Parent=MALK_00035.t1
contig001	maker	exon	77828	78214	.	+	.	ID=MALK_00035.t1.e2;Parent=MALK_00035.t1
contig001	AUGUSTUS	gene	78372	79136	0.85	+	.	ID=MALK_00036;prediction_source=braker_MRET:g701.t1
contig001	AUGUSTUS	CDS	78372	79136	0.85	+	0	ID=MALK_00036.t1.c1;Parent=MALK_00036.t1
contig001	AUGUSTUS	mRNA	78372	79136	0.85	+	.	ID=MALK_00036.t1;Parent=MALK_00036
contig001	AUGUSTUS	exon	78372	79136	.	+	.	ID=MALK_00036.t1.e1;Parent=MALK_00036.t1
contig001	maker	gene	79314	79949	.	-	.	ID=MALK_00037;prediction_source=maker_MRET:augustus_masked-contig001-processed-gene-0.65-mRNA-1
contig001	maker	CDS	79314	79949	.	-	0	ID=MALK_00037.t1.c1;Parent=MALK_00037.t1
contig001	maker	mRNA	79314	79949	.	-	.	ID=MALK_00037.t1;Parent=MALK_00037
contig001	maker	exon	79314	79949	.	-	.	ID=MALK_00037.t1.e1;Parent=MALK_00037.t1
contig001	AUGUSTUS	gene	80256	80746	0.61	-	.	ID=MALK_00038;prediction_source=braker_MRET:g703.t1
contig001	AUGUSTUS	CDS	80611	80746	0.9	-	0	ID=MALK_00038.t1.c3;Parent=MALK_00038.t1
contig001	AUGUSTUS	CDS	80395	80566	0.9	-	0	ID=MALK_00038.t1.c2;Parent=MALK_00038.t1
contig001	AUGUSTUS	CDS	80256	80301	0.9	-	0	ID=MALK_00038.t1.c1;Parent=MALK_00038.t1
contig001	AUGUSTUS	mRNA	80256	80746	0.61	-	.	ID=MALK_00038.t1;Parent=MALK_00038
contig001	AUGUSTUS	exon	80611	80746	.	-	.	ID=MALK_00038.t1.e3;Parent=MALK_00038.t1
contig001	AUGUSTUS	exon	80395	80566	.	-	.	ID=MALK_00038.t1.e2;Parent=MALK_00038.t1
contig001	AUGUSTUS	exon	80256	80301	.	-	.	ID=MALK_00038.t1.e1;Parent=MALK_00038.t1
contig001	AUGUSTUS	gene	80908	82374	0.89	+	.	ID=MALK_00039;prediction_source=augustus:contig001.g356.t1
contig001	AUGUSTUS	CDS	80908	82374	0.89	+	0	ID=MALK_00039.t1.c1;Parent=MALK_00039.t1
contig001	AUGUSTUS	mRNA	80908	82374	0.89	+	.	ID=MALK_00039.t1;Parent=MALK_00039
contig001	AUGUSTUS	exon	80908	82374	0.89	+	.	ID=MALK_00039.t1.e1;Parent=MALK_00039.t1
contig001	AUGUSTUS	gene	82421	83210	0.53	-	.	ID=MALK_00040;prediction_source=braker_MRET:g705.t1
contig001	AUGUSTUS	CDS	83199	83210	0.94	-	0	ID=MALK_00040.t1.c3;Parent=MALK_00040.t1
contig001	AUGUSTUS	CDS	82719	83150	0.94	-	0	ID=MALK_00040.t1.c2;Parent=MALK_00040.t1
contig001	AUGUSTUS	CDS	82421	82687	0.94	-	0	ID=MALK_00040.t1.c1;Parent=MALK_00040.t1
contig001	AUGUSTUS	mRNA	82421	83210	0.53	-	.	ID=MALK_00040.t1;Parent=MALK_00040
contig001	AUGUSTUS	exon	83199	83210	.	-	.	ID=MALK_00040.t1.e3;Parent=MALK_00040.t1
contig001	AUGUSTUS	exon	82719	83150	.	-	.	ID=MALK_00040.t1.e2;Parent=MALK_00040.t1
contig001	AUGUSTUS	exon	82421	82687	.	-	.	ID=MALK_00040.t1.e1;Parent=MALK_00040.t1
contig001	AUGUSTUS	gene	83335	83723	0.76	-	.	ID=MALK_00041;prediction_source=braker_MRET:g706.t1
contig001	AUGUSTUS	CDS	83460	83723	0.8	-	0	ID=MALK_00041.t1.c2;Parent=MALK_00041.t1
contig001	AUGUSTUS	CDS	83335	83400	0.8	-	0	ID=MALK_00041.t1.c1;Parent=MALK_00041.t1
contig001	AUGUSTUS	mRNA	83335	83723	0.76	-	.	ID=MALK_00041.t1;Parent=MALK_00041
contig001	AUGUSTUS	exon	83460	83723	.	-	.	ID=MALK_00041.t1.e2;Parent=MALK_00041.t1
contig001	AUGUSTUS	exon	83335	83400	.	-	.	ID=MALK_00041.t1.e1;Parent=MALK_00041.t1
contig001	AUGUSTUS	gene	83900	84554	1	+	.	ID=MALK_00042;prediction_source=braker_MRET:g707.t1
contig001	AUGUSTUS	CDS	83900	84326	1	+	0	ID=MALK_00042.t1.c1;Parent=MALK_00042.t1
contig001	AUGUSTUS	CDS	84365	84405	1	+	0	ID=MALK_00042.t1.c2;Parent=MALK_00042.t1

contig001	AUGUSTUS	CDS	84447	84478	1	+	0	ID=MALK_00042.t1.c3;Parent=MALK_00042.t1
contig001	AUGUSTUS	CDS	84518	84554	1	+	0	ID=MALK_00042.t1.c4;Parent=MALK_00042.t1
contig001	AUGUSTUS	mRNA	83900	84554	1	+	.	ID=MALK_00042.t1;Parent=MALK_00042
contig001	AUGUSTUS	exon	83900	84326	.	+	.	ID=MALK_00042.t1.e1;Parent=MALK_00042.t1
contig001	AUGUSTUS	exon	84365	84405	.	+	.	ID=MALK_00042.t1.e2;Parent=MALK_00042.t1
contig001	AUGUSTUS	exon	84447	84478	.	+	.	ID=MALK_00042.t1.e3;Parent=MALK_00042.t1
contig001	AUGUSTUS	exon	84518	84554	.	+	.	ID=MALK_00042.t1.e4;Parent=MALK_00042.t1
contig001	AUGUSTUS	gene	84795	88427	0.89	-	.	ID=MALK_00043;prediction_source=augustus:contig001.g360.t1
contig001	AUGUSTUS	CDS	84795	88427	0.89	-	0	ID=MALK_00043.t1.c1;Parent=MALK_00043.t1
contig001	AUGUSTUS	mRNA	84795	88427	0.89	-	.	ID=MALK_00043.t1;Parent=MALK_00043
contig001	AUGUSTUS	exon	84795	88427	0.89	-	.	ID=MALK_00043.t1.e1;Parent=MALK_00043.t1
contig001	maker	gene	88956	90248	.	-	.	ID=MALK_00044;prediction_source=maker_MRET:augustus_masked-contig001-processed-gene-0.70-mRNA-1
contig001	maker	CDS	88956	90248	.	-	0	ID=MALK_00044.t1.c1;Parent=MALK_00044.t1
contig001	maker	mRNA	88956	90248	.	-	.	ID=MALK_00044.t1;Parent=MALK_00044
contig001	maker	exon	88956	90248	.	-	.	ID=MALK_00044.t1.e1;Parent=MALK_00044.t1
contig001	AUGUSTUS	gene	90475	94389	0.81	+	.	ID=MALK_00045;prediction_source=augustus:contig001.g362.t1
contig001	AUGUSTUS	CDS	90475	94389	0.81	+	0	ID=MALK_00045.t1.c1;Parent=MALK_00045.t1
contig001	AUGUSTUS	mRNA	90475	94389	0.81	+	.	ID=MALK_00045.t1;Parent=MALK_00045
contig001	AUGUSTUS	exon	90475	94389	0.81	+	.	ID=MALK_00045.t1.e1;Parent=MALK_00045.t1
contig001	AUGUSTUS	gene	94493	97621	0.89	+	.	ID=MALK_00046;prediction_source=augustus:contig001.g364.t1
contig001	AUGUSTUS	CDS	94493	97621	0.89	+	0	ID=MALK_00046.t1.c1;Parent=MALK_00046.t1
contig001	AUGUSTUS	mRNA	94493	97621	0.89	+	.	ID=MALK_00046.t1;Parent=MALK_00046
contig001	AUGUSTUS	exon	94493	97621	0.89	+	.	ID=MALK_00046.t1.e1;Parent=MALK_00046.t1
contig001	AUGUSTUS	gene	97668	99593	0.23	-	.	ID=MALK_00047;prediction_source=augustus:contig001.g366.t1
contig001	AUGUSTUS	CDS	97668	99593	0.23	-	0	ID=MALK_00047.t1.c1;Parent=MALK_00047.t1
contig001	AUGUSTUS	mRNA	97668	99593	0.23	-	.	ID=MALK_00047.t1;Parent=MALK_00047
contig001	AUGUSTUS	exon	97668	99593	0.23	-	.	ID=MALK_00047.t1.e1;Parent=MALK_00047.t1
contig001	AUGUSTUS	gene	99972	100679	0.52	-	.	ID=MALK_00048;prediction_source=augustus:contig001.g368.t1
contig001	AUGUSTUS	CDS	99972	100679	0.52	-	0	ID=MALK_00048.t1.c1;Parent=MALK_00048.t1
contig001	AUGUSTUS	mRNA	99972	100679	0.52	-	.	ID=MALK_00048.t1;Parent=MALK_00048
contig001	AUGUSTUS	exon	99972	100679	0.52	-	.	ID=MALK_00048.t1.e1;Parent=MALK_00048.t1
contig001	AUGUSTUS	gene	100885	101811	0.96	-	.	ID=MALK_00049;prediction_source=augustus:contig001.g369.t1
contig001	AUGUSTUS	CDS	100885	101811	0.96	-	0	ID=MALK_00049.t1.c1;Parent=MALK_00049.t1
contig001	AUGUSTUS	mRNA	100885	101811	0.96	-	.	ID=MALK_00049.t1;Parent=MALK_00049
contig001	AUGUSTUS	exon	100885	101811	0.96	-	.	ID=MALK_00049.t1.e1;Parent=MALK_00049.t1
contig001	AUGUSTUS	gene	102284	103141	0.98	+	.	ID=MALK_00050;prediction_source=augustus:contig001.g370.t1
contig001	AUGUSTUS	CDS	102284	103141	0.98	+	0	ID=MALK_00050.t1.c1;Parent=MALK_00050.t1
contig001	AUGUSTUS	mRNA	102284	103141	0.98	+	.	ID=MALK_00050.t1;Parent=MALK_00050
contig001	AUGUSTUS	exon	102284	103141	0.98	+	.	ID=MALK_00050.t1.e1;Parent=MALK_00050.t1
contig001	AUGUSTUS	gene	104156	104930	0.77	+	.	ID=MALK_00051;prediction_source=braker_MRET:g714.t1
contig001	AUGUSTUS	CDS	104156	104655	0.99	+	0	ID=MALK_00051.t1.c1;Parent=MALK_00051.t1
contig001	AUGUSTUS	CDS	104684	104930	0.99	+	0	ID=MALK_00051.t1.c2;Parent=MALK_00051.t1
contig001	AUGUSTUS	mRNA	104156	104930	0.77	+	.	ID=MALK_00051.t1;Parent=MALK_00051

contig001	AUGUSTUS	exon	104156	104655	.	+	.	ID=MALK_00051.t1.e1;Parent=MALK_00051.t1
contig001	AUGUSTUS	exon	104684	104930	.	+	.	ID=MALK_00051.t1.e2;Parent=MALK_00051.t1
contig001	maker	gene	104927	105661	.	-	.	ID=MALK_00052;prediction_source=maker_MRET:augustus_masked-contig001-processed-gene-1.26-mRNA-1
contig001	maker	CDS	104927	105661	.	-	0	ID=MALK_00052.t1.c1;Parent=MALK_00052.t1
contig001	maker	mRNA	104927	105661	.	-	.	ID=MALK_00052.t1;Parent=MALK_00052
contig001	maker	exon	104927	105661	.	-	.	ID=MALK_00052.t1.e1;Parent=MALK_00052.t1
contig001	AUGUSTUS	gene	105861	107393	0.22	+	.	ID=MALK_00053;prediction_source=augustus:contig001.g373.t1
contig001	AUGUSTUS	CDS	105861	107393	0.22	+	0	ID=MALK_00053.t1.c1;Parent=MALK_00053.t1
contig001	AUGUSTUS	mRNA	105861	107393	0.22	+	.	ID=MALK_00053.t1;Parent=MALK_00053
contig001	AUGUSTUS	exon	105861	107393	0.22	+	.	ID=MALK_00053.t1.e1;Parent=MALK_00053.t1
contig001	AUGUSTUS	gene	107571	108383	0.31	+	.	ID=MALK_00054;prediction_source=augustus:contig001.g374.t1
contig001	AUGUSTUS	CDS	107571	108383	0.31	+	0	ID=MALK_00054.t1.c1;Parent=MALK_00054.t1
contig001	AUGUSTUS	mRNA	107571	108383	0.31	+	.	ID=MALK_00054.t1;Parent=MALK_00054
contig001	AUGUSTUS	exon	107571	108383	0.31	+	.	ID=MALK_00054.t1.e1;Parent=MALK_00054.t1
contig001	AUGUSTUS	gene	108387	110207	1	-	.	ID=MALK_00055;prediction_source=braker_MRET:g718.t1
contig001	AUGUSTUS	CDS	108387	110207	1	-	0	ID=MALK_00055.t1.c1;Parent=MALK_00055.t1
contig001	AUGUSTUS	mRNA	108387	110207	1	-	.	ID=MALK_00055.t1;Parent=MALK_00055
contig001	AUGUSTUS	exon	108387	110207	.	-	.	ID=MALK_00055.t1.e1;Parent=MALK_00055.t1
contig001	AUGUSTUS	gene	110258	111166	0.47	-	.	ID=MALK_00056;prediction_source=augustus:contig001.g375.t1
contig001	AUGUSTUS	CDS	110258	111166	0.47	-	0	ID=MALK_00056.t1.c1;Parent=MALK_00056.t1
contig001	AUGUSTUS	mRNA	110258	111166	0.47	-	.	ID=MALK_00056.t1;Parent=MALK_00056
contig001	AUGUSTUS	exon	110258	111166	0.47	-	.	ID=MALK_00056.t1.e1;Parent=MALK_00056.t1
contig001	AUGUSTUS	gene	111294	111686	0.53	+	.	ID=MALK_00057;prediction_source=braker_MRET:g720.t1
contig001	AUGUSTUS	CDS	111294	111686	0.53	+	0	ID=MALK_00057.t1.c1;Parent=MALK_00057.t1
contig001	AUGUSTUS	mRNA	111294	111686	0.53	+	.	ID=MALK_00057.t1;Parent=MALK_00057
contig001	AUGUSTUS	exon	111294	111686	.	+	.	ID=MALK_00057.t1.e1;Parent=MALK_00057.t1
contig001	AUGUSTUS	gene	111847	113202	0.64	+	.	ID=MALK_00058;prediction_source=augustus:contig001.g376.t1
contig001	AUGUSTUS	CDS	111847	113202	0.64	+	0	ID=MALK_00058.t1.c1;Parent=MALK_00058.t1
contig001	AUGUSTUS	mRNA	111847	113202	0.64	+	.	ID=MALK_00058.t1;Parent=MALK_00058
contig001	AUGUSTUS	exon	111847	113202	0.64	+	.	ID=MALK_00058.t1.e1;Parent=MALK_00058.t1
contig001	AUGUSTUS	gene	113254	114029	0.86	+	.	ID=MALK_00059;prediction_source=braker_MRET:g722.t1
contig001	AUGUSTUS	CDS	113254	113562	1	+	0	ID=MALK_00059.t1.c1;Parent=MALK_00059.t1
contig001	AUGUSTUS	CDS	113614	113668	1	+	0	ID=MALK_00059.t1.c2;Parent=MALK_00059.t1
contig001	AUGUSTUS	CDS	113698	114029	1	+	0	ID=MALK_00059.t1.c3;Parent=MALK_00059.t1
contig001	AUGUSTUS	mRNA	113254	114029	0.86	+	.	ID=MALK_00059.t1;Parent=MALK_00059
contig001	AUGUSTUS	exon	113254	113562	.	+	.	ID=MALK_00059.t1.e1;Parent=MALK_00059.t1
contig001	AUGUSTUS	exon	113614	113668	.	+	.	ID=MALK_00059.t1.e2;Parent=MALK_00059.t1
contig001	AUGUSTUS	exon	113698	114029	.	+	.	ID=MALK_00059.t1.e3;Parent=MALK_00059.t1
contig001	AUGUSTUS	gene	114044	115087	0.99	-	.	ID=MALK_00060;prediction_source=braker_MRET:g723.t1
contig001	AUGUSTUS	CDS	114044	115087	0.99	-	0	ID=MALK_00060.t1.c1;Parent=MALK_00060.t1
contig001	AUGUSTUS	mRNA	114044	115087	0.99	-	.	ID=MALK_00060.t1;Parent=MALK_00060
contig001	AUGUSTUS	exon	114044	115087	.	-	.	ID=MALK_00060.t1.e1;Parent=MALK_00060.t1
contig001	maker	gene	115262	117106	.	+	.	ID=MALK_00061;prediction_source=maker_MRET:augustus_masked-contig001-processed-gene-1.10-mRNA-1

contig001	maker	CDS	115262	117106	.	+	0	ID=MALK_00061.t1.c1;Parent=MALK_00061.t1
contig001	maker	mRNA	115262	117106	.	+	.	ID=MALK_00061.t1;Parent=MALK_00061
contig001	maker	exon	115262	117106	.	+	.	ID=MALK_00061.t1.e1;Parent=MALK_00061.t1
contig001	maker	gene	117533	118492	.	+	.	ID=MALK_00062;prediction_source=maker_MRET:augustus_masked-contig001-processed-gene-1.11-mRNA-1
contig001	maker	CDS	117533	118492	.	+	0	ID=MALK_00062.t1.c1;Parent=MALK_00062.t1
contig001	maker	mRNA	117533	118492	.	+	.	ID=MALK_00062.t1;Parent=MALK_00062
contig001	maker	exon	117533	118492	.	+	.	ID=MALK_00062.t1.e1;Parent=MALK_00062.t1
contig001	AUGUSTUS	gene	118590	120776	1	+	.	ID=MALK_00063;prediction_source=augustus:contig001.g382.t1
contig001	AUGUSTUS	CDS	118590	120776	1	+	0	ID=MALK_00063.t1.c1;Parent=MALK_00063.t1
contig001	AUGUSTUS	mRNA	118590	120776	1	+	.	ID=MALK_00063.t1;Parent=MALK_00063
contig001	AUGUSTUS	exon	118590	120776	1	+	.	ID=MALK_00063.t1.e1;Parent=MALK_00063.t1
contig001	AUGUSTUS	gene	120769	121371	0.77	-	.	ID=MALK_00064;prediction_source=braker_MRET:g727.t1
contig001	AUGUSTUS	CDS	120769	121371	0.77	-	0	ID=MALK_00064.t1.c1;Parent=MALK_00064.t1
contig001	AUGUSTUS	mRNA	120769	121371	0.77	-	.	ID=MALK_00064.t1;Parent=MALK_00064
contig001	AUGUSTUS	exon	120769	121371	.	-	.	ID=MALK_00064.t1.e1;Parent=MALK_00064.t1
contig001	AUGUSTUS	gene	121772	123745	0.69	-	.	ID=MALK_00065;prediction_source=braker_MRET:g728.t1
contig001	AUGUSTUS	CDS	121772	123745	0.69	-	0	ID=MALK_00065.t1.c1;Parent=MALK_00065.t1
contig001	AUGUSTUS	mRNA	121772	123745	0.69	-	.	ID=MALK_00065.t1;Parent=MALK_00065
contig001	AUGUSTUS	exon	121772	123745	.	-	.	ID=MALK_00065.t1.e1;Parent=MALK_00065.t1
contig001	AUGUSTUS	gene	123934	124713	0.53	+	.	ID=MALK_00066;prediction_source=augustus:contig001.g384.t1
contig001	AUGUSTUS	CDS	123934	124713	0.53	+	0	ID=MALK_00066.t1.c1;Parent=MALK_00066.t1
contig001	AUGUSTUS	mRNA	123934	124713	0.53	+	.	ID=MALK_00066.t1;Parent=MALK_00066
contig001	AUGUSTUS	exon	123934	124713	0.53	+	.	ID=MALK_00066.t1.e1;Parent=MALK_00066.t1
contig001	AUGUSTUS	gene	125390	127528	0.47	+	.	ID=MALK_00067;prediction_source=augustus:contig001.g385.t1
contig001	AUGUSTUS	CDS	125390	127528	0.47	+	0	ID=MALK_00067.t1.c1;Parent=MALK_00067.t1
contig001	AUGUSTUS	mRNA	125390	127528	0.47	+	.	ID=MALK_00067.t1;Parent=MALK_00067
contig001	AUGUSTUS	exon	125390	127528	0.47	+	.	ID=MALK_00067.t1.e1;Parent=MALK_00067.t1
contig001	maker	gene	127945	129813	.	+	.	ID=MALK_00068;prediction_source=maker_MRET:augustus_masked-contig001-processed-gene-1.15-mRNA-1
contig001	maker	CDS	127945	129813	.	+	0	ID=MALK_00068.t1.c1;Parent=MALK_00068.t1
contig001	maker	mRNA	127945	129813	.	+	.	ID=MALK_00068.t1;Parent=MALK_00068
contig001	maker	exon	127945	129813	.	+	.	ID=MALK_00068.t1.e1;Parent=MALK_00068.t1
contig001	AUGUSTUS	gene	130074	131033	0.83	+	.	ID=MALK_00069;prediction_source=braker_MRET:g731.t1
contig001	AUGUSTUS	CDS	130074	130113	0.95	+	0	ID=MALK_00069.t1.c1;Parent=MALK_00069.t1
contig001	AUGUSTUS	CDS	130145	130208	0.95	+	0	ID=MALK_00069.t1.c2;Parent=MALK_00069.t1
contig001	AUGUSTUS	CDS	130242	130426	0.95	+	0	ID=MALK_00069.t1.c3;Parent=MALK_00069.t1
contig001	AUGUSTUS	CDS	130501	131033	0.95	+	0	ID=MALK_00069.t1.c4;Parent=MALK_00069.t1
contig001	AUGUSTUS	mRNA	130074	131033	0.83	+	.	ID=MALK_00069.t1;Parent=MALK_00069
contig001	AUGUSTUS	exon	130074	130113	.	+	.	ID=MALK_00069.t1.e1;Parent=MALK_00069.t1
contig001	AUGUSTUS	exon	130145	130208	.	+	.	ID=MALK_00069.t1.e2;Parent=MALK_00069.t1
contig001	AUGUSTUS	exon	130242	130426	.	+	.	ID=MALK_00069.t1.e3;Parent=MALK_00069.t1
contig001	AUGUSTUS	exon	130501	131033	.	+	.	ID=MALK_00069.t1.e4;Parent=MALK_00069.t1
contig001	maker	gene	131107	132621	.	-	.	ID=MALK_00070;prediction_source=maker_MRET:augustus_masked-contig001-processed-gene-1.29-mRNA-1
contig001	maker	CDS	131107	132621	.	-	0	ID=MALK_00070.t1.c1;Parent=MALK_00070.t1

contig001	maker	mRNA	131107	132621	.	-	.	ID=MALK_00070.t1;Parent=MALK_00070
contig001	maker	exon	131107	132621	.	-	.	ID=MALK_00070.t1.e1;Parent=MALK_00070.t1
contig001	maker	gene	132801	133635	.	+	.	ID=MALK_00071;prediction_source=maker_MRET:augustus_masked-contig001-processed-gene-1.17-mRNA-1
contig001	maker	CDS	132801	133107	.	+	0	ID=MALK_00071.t1.c1;Parent=MALK_00071.t1
contig001	maker	CDS	133214	133635	.	+	0	ID=MALK_00071.t1.c2;Parent=MALK_00071.t1
contig001	maker	mRNA	132801	133635	.	+	.	ID=MALK_00071.t1;Parent=MALK_00071
contig001	maker	exon	132801	133107	.	+	.	ID=MALK_00071.t1.e1;Parent=MALK_00071.t1
contig001	maker	exon	133214	133635	.	+	.	ID=MALK_00071.t1.e2;Parent=MALK_00071.t1
contig001	AUGUSTUS	gene	133902	134729	0.65	-	.	ID=MALK_00072;prediction_source=braker_MRET:g734.t1
contig001	AUGUSTUS	CDS	133902	134729	0.65	-	0	ID=MALK_00072.t1.c1;Parent=MALK_00072.t1
contig001	AUGUSTUS	mRNA	133902	134729	0.65	-	.	ID=MALK_00072.t1;Parent=MALK_00072
contig001	AUGUSTUS	exon	133902	134729	.	-	.	ID=MALK_00072.t1.e1;Parent=MALK_00072.t1
contig001	AUGUSTUS	gene	135482	138709	0.72	-	.	ID=MALK_00073;prediction_source=augustus:contig001.g392.t1
contig001	AUGUSTUS	CDS	135482	138709	0.72	-	0	ID=MALK_00073.t1.c1;Parent=MALK_00073.t1
contig001	AUGUSTUS	mRNA	135482	138709	0.72	-	.	ID=MALK_00073.t1;Parent=MALK_00073
contig001	AUGUSTUS	exon	135482	138709	0.72	-	.	ID=MALK_00073.t1.e1;Parent=MALK_00073.t1
contig001	AUGUSTUS	gene	139087	140022	0.37	+	.	ID=MALK_00074;prediction_source=augustus:contig001.g394.t1
contig001	AUGUSTUS	CDS	139087	140022	0.37	+	0	ID=MALK_00074.t1.c1;Parent=MALK_00074.t1
contig001	AUGUSTUS	mRNA	139087	140022	0.37	+	.	ID=MALK_00074.t1;Parent=MALK_00074
contig001	AUGUSTUS	exon	139087	140022	0.37	+	.	ID=MALK_00074.t1.e1;Parent=MALK_00074.t1
contig001	maker	gene	140044	141789	.	-	.	ID=MALK_00075;prediction_source=maker_MRET:augustus_masked-contig001-processed-gene-1.32-mRNA-1
contig001	maker	CDS	140044	141789	.	-	0	ID=MALK_00075.t1.c1;Parent=MALK_00075.t1
contig001	maker	mRNA	140044	141789	.	-	.	ID=MALK_00075.t1;Parent=MALK_00075
contig001	maker	exon	140044	141789	.	-	.	ID=MALK_00075.t1.e1;Parent=MALK_00075.t1
contig001	AUGUSTUS	gene	142100	142501	0.3	+	.	ID=MALK_00076;prediction_source=braker_MRET:g737.t1
contig001	AUGUSTUS	CDS	142100	142501	0.3	+	0	ID=MALK_00076.t1.c1;Parent=MALK_00076.t1
contig001	AUGUSTUS	mRNA	142100	142501	0.3	+	.	ID=MALK_00076.t1;Parent=MALK_00076
contig001	AUGUSTUS	exon	142100	142501	.	+	.	ID=MALK_00076.t1.e1;Parent=MALK_00076.t1
contig001	maker	gene	143098	146502	.	-	.	ID=MALK_00077;prediction_source=maker_MRET:augustus_masked-contig001-processed-gene-1.33-mRNA-1
contig001	maker	CDS	143098	146502	.	-	0	ID=MALK_00077.t1.c1;Parent=MALK_00077.t1
contig001	maker	mRNA	143098	146502	.	-	.	ID=MALK_00077.t1;Parent=MALK_00077
contig001	maker	exon	143098	146502	.	-	.	ID=MALK_00077.t1.e1;Parent=MALK_00077.t1
contig001	maker	gene	146586	150197	.	-	.	ID=MALK_00078;prediction_source=maker_MRET:augustus_masked-contig001-processed-gene-1.34-mRNA-1
contig001	maker	CDS	146586	150197	.	-	0	ID=MALK_00078.t1.c1;Parent=MALK_00078.t1
contig001	maker	mRNA	146586	150197	.	-	.	ID=MALK_00078.t1;Parent=MALK_00078
contig001	maker	exon	146586	150197	.	-	.	ID=MALK_00078.t1.e1;Parent=MALK_00078.t1
contig001	maker	gene	150344	154075	.	-	.	ID=MALK_00079;prediction_source=maker_MRET:augustus_masked-contig001-processed-gene-1.35-mRNA-1
contig001	maker	CDS	150344	154075	.	-	0	ID=MALK_00079.t1.c1;Parent=MALK_00079.t1
contig001	maker	mRNA	150344	154075	.	-	.	ID=MALK_00079.t1;Parent=MALK_00079
contig001	maker	exon	150344	154075	.	-	.	ID=MALK_00079.t1.e1;Parent=MALK_00079.t1
contig001	maker	gene	156488	158065	.	+	.	ID=MALK_00080;prediction_source=maker_MRET:augustus_masked-contig001-processed-gene-1.19-mRNA-1
contig001	maker	CDS	156488	158065	.	+	0	ID=MALK_00080.t1.c1;Parent=MALK_00080.t1
contig001	maker	mRNA	156488	158065	.	+	.	ID=MALK_00080.t1;Parent=MALK_00080

contig001	maker	exon	156488	158065	.	+	.	ID=MALK_00080.t1.e1;Parent=MALK_00080.t1
contig001	AUGUSTUS	gene	158090	161106	0.24	-	.	ID=MALK_00081;prediction_source=braker_MRET:g742.t1
contig001	AUGUSTUS	CDS	161095	161106	0.98	-	0	ID=MALK_00081.t1.c5;Parent=MALK_00081.t1
contig001	AUGUSTUS	CDS	159779	161054	0.98	-	0	ID=MALK_00081.t1.c4;Parent=MALK_00081.t1
contig001	AUGUSTUS	CDS	159128	159497	0.98	-	0	ID=MALK_00081.t1.c3;Parent=MALK_00081.t1
contig001	AUGUSTUS	CDS	159030	159087	0.98	-	0	ID=MALK_00081.t1.c2;Parent=MALK_00081.t1
contig001	AUGUSTUS	CDS	158090	159001	0.98	-	0	ID=MALK_00081.t1.c1;Parent=MALK_00081.t1
contig001	AUGUSTUS	mRNA	158090	161106	0.24	-	.	ID=MALK_00081.t1;Parent=MALK_00081
contig001	AUGUSTUS	exon	161095	161106	.	-	.	ID=MALK_00081.t1.e5;Parent=MALK_00081.t1
contig001	AUGUSTUS	exon	159779	161054	.	-	.	ID=MALK_00081.t1.e4;Parent=MALK_00081.t1
contig001	AUGUSTUS	exon	159128	159497	.	-	.	ID=MALK_00081.t1.e3;Parent=MALK_00081.t1
contig001	AUGUSTUS	exon	159030	159087	.	-	.	ID=MALK_00081.t1.e2;Parent=MALK_00081.t1
contig001	AUGUSTUS	exon	158090	159001	.	-	.	ID=MALK_00081.t1.e1;Parent=MALK_00081.t1
contig001	AUGUSTUS	gene	161237	161959	0.98	+	.	ID=MALK_00082;prediction_source=augustus:contig001.g404.t1
contig001	AUGUSTUS	CDS	161237	161959	0.98	+	0	ID=MALK_00082.t1.c1;Parent=MALK_00082.t1
contig001	AUGUSTUS	mRNA	161237	161959	0.98	+	.	ID=MALK_00082.t1;Parent=MALK_00082
contig001	AUGUSTUS	exon	161237	161959	0.98	+	.	ID=MALK_00082.t1.e1;Parent=MALK_00082.t1
contig001	AUGUSTUS	gene	161960	163525	1	-	.	ID=MALK_00083;prediction_source=braker_MRET:g743.t1
contig001	AUGUSTUS	CDS	161960	163525	1	-	0	ID=MALK_00083.t1.c1;Parent=MALK_00083.t1
contig001	AUGUSTUS	mRNA	161960	163525	1	-	.	ID=MALK_00083.t1;Parent=MALK_00083
contig001	AUGUSTUS	exon	161960	163525	.	-	.	ID=MALK_00083.t1.e1;Parent=MALK_00083.t1
contig001	AUGUSTUS	gene	163710	164795	0.49	-	.	ID=MALK_00084;prediction_source=braker_MRET:g744.t1
contig001	AUGUSTUS	CDS	163710	164795	0.49	-	0	ID=MALK_00084.t1.c1;Parent=MALK_00084.t1
contig001	AUGUSTUS	mRNA	163710	164795	0.49	-	.	ID=MALK_00084.t1;Parent=MALK_00084
contig001	AUGUSTUS	exon	163710	164795	.	-	.	ID=MALK_00084.t1.e1;Parent=MALK_00084.t1
contig001	maker	gene	165477	166922	.	-	.	ID=MALK_00085;prediction_source=maker_MRET:augustus_masked-contig001-processed-gene-1.37-mRNA-1
contig001	maker	CDS	165477	166922	.	-	0	ID=MALK_00085.t1.c1;Parent=MALK_00085.t1
contig001	maker	mRNA	165477	166922	.	-	.	ID=MALK_00085.t1;Parent=MALK_00085
contig001	maker	exon	165477	166922	.	-	.	ID=MALK_00085.t1.e1;Parent=MALK_00085.t1
contig001	AUGUSTUS	gene	167268	168374	0.45	+	.	ID=MALK_00086;prediction_source=braker_MRET:g746.t1
contig001	AUGUSTUS	CDS	167268	168374	0.45	+	0	ID=MALK_00086.t1.c1;Parent=MALK_00086.t1
contig001	AUGUSTUS	mRNA	167268	168374	0.45	+	.	ID=MALK_00086.t1;Parent=MALK_00086
contig001	AUGUSTUS	exon	167268	168374	.	+	.	ID=MALK_00086.t1.e1;Parent=MALK_00086.t1
contig001	maker	gene	168382	170913	.	-	.	ID=MALK_00087;prediction_source=maker_MRET:augustus_masked-contig001-processed-gene-1.38-mRNA-1
contig001	maker	CDS	168382	170913	.	-	0	ID=MALK_00087.t1.c1;Parent=MALK_00087.t1
contig001	maker	mRNA	168382	170913	.	-	.	ID=MALK_00087.t1;Parent=MALK_00087
contig001	maker	exon	168382	170913	.	-	.	ID=MALK_00087.t1.e1;Parent=MALK_00087.t1
contig001	AUGUSTUS	gene	171068	172660	0.84	-	.	ID=MALK_00088;prediction_source=braker_MRET:g748.t1
contig001	AUGUSTUS	CDS	171068	172660	0.84	-	0	ID=MALK_00088.t1.c1;Parent=MALK_00088.t1
contig001	AUGUSTUS	mRNA	171068	172660	0.84	-	.	ID=MALK_00088.t1;Parent=MALK_00088
contig001	AUGUSTUS	exon	171068	172660	.	-	.	ID=MALK_00088.t1.e1;Parent=MALK_00088.t1
contig001	AUGUSTUS	gene	172821	173855	0.95	+	.	ID=MALK_00089;prediction_source=augustus:contig001.g409.t1
contig001	AUGUSTUS	CDS	172821	173855	0.95	+	0	ID=MALK_00089.t1.c1;Parent=MALK_00089.t1

contig001	AUGUSTUS	mRNA	172821	173855	0.95	+	.	ID=MALK_00089.t1;Parent=MALK_00089
contig001	AUGUSTUS	exon	172821	173855	0.95	+	.	ID=MALK_00089.t1.e1;Parent=MALK_00089.t1
contig001	maker	gene	174084	178862	.	-	.	ID=MALK_00090;prediction_source=maker_MRET:augustus_masked-contig001-processed-gene-1.40-mRNA-1
contig001	maker	CDS	174084	178862	.	-	0	ID=MALK_00090.t1.c1;Parent=MALK_00090.t1
contig001	maker	mRNA	174084	178862	.	-	.	ID=MALK_00090.t1;Parent=MALK_00090
contig001	maker	exon	174084	178862	.	-	.	ID=MALK_00090.t1.e1;Parent=MALK_00090.t1
contig001	maker	gene	179235	180332	.	-	.	ID=MALK_00091;prediction_source=maker_MRET:augustus_masked-contig001-processed-gene-1.41-mRNA-1
contig001	maker	CDS	179235	180332	.	-	0	ID=MALK_00091.t1.c1;Parent=MALK_00091.t1
contig001	maker	mRNA	179235	180332	.	-	.	ID=MALK_00091.t1;Parent=MALK_00091
contig001	maker	exon	179235	180332	.	-	.	ID=MALK_00091.t1.e1;Parent=MALK_00091.t1
contig001	AUGUSTUS	gene	180638	181454	0.35	+	.	ID=MALK_00092;prediction_source=braker_MRET:g752.t1
contig001	AUGUSTUS	CDS	180638	181217	0.43	+	0	ID=MALK_00092.t1.c1;Parent=MALK_00092.t1
contig001	AUGUSTUS	CDS	181246	181454	0.43	+	0	ID=MALK_00092.t1.c2;Parent=MALK_00092.t1
contig001	AUGUSTUS	mRNA	180638	181454	0.35	+	.	ID=MALK_00092.t1;Parent=MALK_00092
contig001	AUGUSTUS	exon	180638	181217	.	+	.	ID=MALK_00092.t1.e1;Parent=MALK_00092.t1
contig001	AUGUSTUS	exon	181246	181454	.	+	.	ID=MALK_00092.t1.e2;Parent=MALK_00092.t1
contig001	AUGUSTUS	gene	181455	183482	0.16	-	.	ID=MALK_00093;prediction_source=augustus:contig001.g417.t1
contig001	AUGUSTUS	CDS	181455	183482	0.16	-	0	ID=MALK_00093.t1.c1;Parent=MALK_00093.t1
contig001	AUGUSTUS	mRNA	181455	183482	0.16	-	.	ID=MALK_00093.t1;Parent=MALK_00093
contig001	AUGUSTUS	exon	181455	183482	0.16	-	.	ID=MALK_00093.t1.e1;Parent=MALK_00093.t1
contig001	AUGUSTUS	gene	183746	184204	0.83	+	.	ID=MALK_00094;prediction_source=augustus:contig001.g418.t1
contig001	AUGUSTUS	CDS	183746	184204	0.83	+	0	ID=MALK_00094.t1.c1;Parent=MALK_00094.t1
contig001	AUGUSTUS	mRNA	183746	184204	0.83	+	.	ID=MALK_00094.t1;Parent=MALK_00094
contig001	AUGUSTUS	exon	183746	184204	0.83	+	.	ID=MALK_00094.t1.e1;Parent=MALK_00094.t1
contig001	AUGUSTUS	gene	185541	186374	0.48	-	.	ID=MALK_00095;prediction_source=augustus:contig001.g419.t1
contig001	AUGUSTUS	CDS	185541	186374	0.48	-	0	ID=MALK_00095.t1.c1;Parent=MALK_00095.t1
contig001	AUGUSTUS	mRNA	185541	186374	0.48	-	.	ID=MALK_00095.t1;Parent=MALK_00095
contig001	AUGUSTUS	exon	185541	186374	0.48	-	.	ID=MALK_00095.t1.e1;Parent=MALK_00095.t1
contig001	AUGUSTUS	gene	186407	186751	0.81	+	.	ID=MALK_00096;prediction_source=braker_MRET:g756.t1
contig001	AUGUSTUS	CDS	186407	186453	1	+	0	ID=MALK_00096.t1.c1;Parent=MALK_00096.t1
contig001	AUGUSTUS	CDS	186484	186593	1	+	0	ID=MALK_00096.t1.c2;Parent=MALK_00096.t1
contig001	AUGUSTUS	CDS	186627	186751	1	+	0	ID=MALK_00096.t1.c3;Parent=MALK_00096.t1
contig001	AUGUSTUS	mRNA	186407	186751	0.81	+	.	ID=MALK_00096.t1;Parent=MALK_00096
contig001	AUGUSTUS	exon	186407	186453	.	+	.	ID=MALK_00096.t1.e1;Parent=MALK_00096.t1
contig001	AUGUSTUS	exon	186484	186593	.	+	.	ID=MALK_00096.t1.e2;Parent=MALK_00096.t1
contig001	AUGUSTUS	exon	186627	186751	.	+	.	ID=MALK_00096.t1.e3;Parent=MALK_00096.t1
contig001	maker	gene	186933	187319	.	-	.	ID=MALK_00097;prediction_source=maker_MRET:augustus_masked-contig001-processed-gene-1.44-mRNA-1
contig001	maker	CDS	186933	187319	.	-	0	ID=MALK_00097.t1.c1;Parent=MALK_00097.t1
contig001	maker	mRNA	186933	187319	.	-	.	ID=MALK_00097.t1;Parent=MALK_00097
contig001	maker	exon	186933	187319	.	-	.	ID=MALK_00097.t1.e1;Parent=MALK_00097.t1
contig001	maker	gene	187546	188559	.	-	.	ID=MALK_00098;prediction_source=maker_MRET:augustus_masked-contig001-processed-gene-1.45-mRNA-1
contig001	maker	CDS	187546	188559	.	-	0	ID=MALK_00098.t1.c1;Parent=MALK_00098.t1
contig001	maker	mRNA	187546	188559	.	-	.	ID=MALK_00098.t1;Parent=MALK_00098

contig001	maker	exon	187546	188559	.	-	.	ID=MALK_00098.t1.e1;Parent=MALK_00098.t1
contig001	maker	gene	188886	189662	.	-	.	ID=MALK_00099;prediction_source=maker_MRET:augustus_masked-contig001-processed-gene-1.46-mRNA-1
contig001	maker	CDS	188886	189662	.	-	0	ID=MALK_00099.t1.c1;Parent=MALK_00099.t1
contig001	maker	mRNA	188886	189662	.	-	.	ID=MALK_00099.t1;Parent=MALK_00099
contig001	maker	exon	188886	189662	.	-	.	ID=MALK_00099.t1.e1;Parent=MALK_00099.t1
contig001	AUGUSTUS	gene	189713	190633	0.94	+	.	ID=MALK_00100;prediction_source=augustus:contig001.g423.t1
contig001	AUGUSTUS	CDS	189713	190633	0.94	+	0	ID=MALK_00100.t1.c1;Parent=MALK_00100.t1
contig001	AUGUSTUS	mRNA	189713	190633	0.94	+	.	ID=MALK_00100.t1;Parent=MALK_00100
contig001	AUGUSTUS	exon	189713	190633	0.94	+	.	ID=MALK_00100.t1.e1;Parent=MALK_00100.t1
contig001	AUGUSTUS	gene	190634	192103	0.87	-	.	ID=MALK_00101;prediction_source=augustus:contig001.g424.t1
contig001	AUGUSTUS	CDS	190634	192103	0.87	-	0	ID=MALK_00101.t1.c1;Parent=MALK_00101.t1
contig001	AUGUSTUS	mRNA	190634	192103	0.87	-	.	ID=MALK_00101.t1;Parent=MALK_00101
contig001	AUGUSTUS	exon	190634	192103	0.87	-	.	ID=MALK_00101.t1.e1;Parent=MALK_00101.t1
contig001	AUGUSTUS	gene	192200	193188	0.73	+	.	ID=MALK_00102;prediction_source=braker_MRET:g762.t1
contig001	AUGUSTUS	CDS	192200	192998	1	+	0	ID=MALK_00102.t1.c1;Parent=MALK_00102.t1
contig001	AUGUSTUS	CDS	193026	193042	1	+	0	ID=MALK_00102.t1.c2;Parent=MALK_00102.t1
contig001	AUGUSTUS	CDS	193072	193188	1	+	0	ID=MALK_00102.t1.c3;Parent=MALK_00102.t1
contig001	AUGUSTUS	mRNA	192200	193188	0.73	+	.	ID=MALK_00102.t1;Parent=MALK_00102
contig001	AUGUSTUS	exon	192200	192998	.	+	.	ID=MALK_00102.t1.e1;Parent=MALK_00102.t1
contig001	AUGUSTUS	exon	193026	193042	.	+	.	ID=MALK_00102.t1.e2;Parent=MALK_00102.t1
contig001	AUGUSTUS	exon	193072	193188	.	+	.	ID=MALK_00102.t1.e3;Parent=MALK_00102.t1
contig001	AUGUSTUS	gene	193565	196859	0.87	+	.	ID=MALK_00103;prediction_source=braker_MRET:g763.t1
contig001	AUGUSTUS	CDS	193565	196785	0.97	+	0	ID=MALK_00103.t1.c1;Parent=MALK_00103.t1
contig001	AUGUSTUS	CDS	196814	196859	0.97	+	0	ID=MALK_00103.t1.c2;Parent=MALK_00103.t1
contig001	AUGUSTUS	mRNA	193565	196859	0.87	+	.	ID=MALK_00103.t1;Parent=MALK_00103
contig001	AUGUSTUS	exon	193565	196785	.	+	.	ID=MALK_00103.t1.e1;Parent=MALK_00103.t1
contig001	AUGUSTUS	exon	196814	196859	.	+	.	ID=MALK_00103.t1.e2;Parent=MALK_00103.t1
contig001	maker	gene	196862	198250	.	-	.	ID=MALK_00104;prediction_source=maker_MRET:augustus_masked-contig001-processed-gene-2.2-mRNA-1
contig001	maker	CDS	196862	198250	.	-	0	ID=MALK_00104.t1.c1;Parent=MALK_00104.t1
contig001	maker	mRNA	196862	198250	.	-	.	ID=MALK_00104.t1;Parent=MALK_00104
contig001	maker	exon	196862	198250	.	-	.	ID=MALK_00104.t1.e1;Parent=MALK_00104.t1
contig001	AUGUSTUS	gene	198249	200252	0.87	+	.	ID=MALK_00105;prediction_source=braker_MRET:g765.t1
contig001	AUGUSTUS	CDS	198249	200252	0.87	+	0	ID=MALK_00105.t1.c1;Parent=MALK_00105.t1
contig001	AUGUSTUS	mRNA	198249	200252	0.87	+	.	ID=MALK_00105.t1;Parent=MALK_00105
contig001	AUGUSTUS	exon	198249	200252	.	+	.	ID=MALK_00105.t1.e1;Parent=MALK_00105.t1
contig001	AUGUSTUS	gene	200267	203332	0.49	-	.	ID=MALK_00106;prediction_source=augustus:contig001.g429.t1
contig001	AUGUSTUS	CDS	200267	203332	0.49	-	0	ID=MALK_00106.t1.c1;Parent=MALK_00106.t1
contig001	AUGUSTUS	mRNA	200267	203332	0.49	-	.	ID=MALK_00106.t1;Parent=MALK_00106
contig001	AUGUSTUS	exon	200267	203332	0.49	-	.	ID=MALK_00106.t1.e1;Parent=MALK_00106.t1
contig001	AUGUSTUS	gene	203418	204398	0.39	-	.	ID=MALK_00107;prediction_source=augustus:contig001.g430.t1
contig001	AUGUSTUS	CDS	203418	204398	0.39	-	0	ID=MALK_00107.t1.c1;Parent=MALK_00107.t1
contig001	AUGUSTUS	mRNA	203418	204398	0.39	-	.	ID=MALK_00107.t1;Parent=MALK_00107
contig001	AUGUSTUS	exon	203418	204398	0.39	-	.	ID=MALK_00107.t1.e1;Parent=MALK_00107.t1

contig001	maker	gene	204577	206646	.	-	.	ID=MALK_00108;prediction_source=maker_MRET:augustus_masked-contig001-processed-gene-2.25-mRNA-1
contig001	maker	CDS	204577	206646	.	-	0	ID=MALK_00108.t1.c1;Parent=MALK_00108.t1
contig001	maker	mRNA	204577	206646	.	-	.	ID=MALK_00108.t1;Parent=MALK_00108
contig001	maker	exon	204577	206646	.	-	.	ID=MALK_00108.t1.e1;Parent=MALK_00108.t1
contig001	AUGUSTUS	gene	207039	208568	0.25	-	.	ID=MALK_00109;prediction_source=braker_MRET:g769.t1
contig001	AUGUSTUS	CDS	208354	208568	0.51	-	0	ID=MALK_00109.t1.c4;Parent=MALK_00109.t1
contig001	AUGUSTUS	CDS	207593	208177	0.51	-	0	ID=MALK_00109.t1.c3;Parent=MALK_00109.t1
contig001	AUGUSTUS	CDS	207340	207565	0.51	-	0	ID=MALK_00109.t1.c2;Parent=MALK_00109.t1
contig001	AUGUSTUS	CDS	207039	207311	0.51	-	0	ID=MALK_00109.t1.c1;Parent=MALK_00109.t1
contig001	AUGUSTUS	mRNA	207039	208568	0.25	-	.	ID=MALK_00109.t1;Parent=MALK_00109
contig001	AUGUSTUS	exon	208354	208568	.	-	.	ID=MALK_00109.t1.e4;Parent=MALK_00109.t1
contig001	AUGUSTUS	exon	207593	208177	.	-	.	ID=MALK_00109.t1.e3;Parent=MALK_00109.t1
contig001	AUGUSTUS	exon	207340	207565	.	-	.	ID=MALK_00109.t1.e2;Parent=MALK_00109.t1
contig001	AUGUSTUS	exon	207039	207311	.	-	.	ID=MALK_00109.t1.e1;Parent=MALK_00109.t1
contig001	AUGUSTUS	gene	208781	209495	1	+	.	ID=MALK_00110;prediction_source=braker_MRET:g770.t1
contig001	AUGUSTUS	CDS	208781	208792	1	+	0	ID=MALK_00110.t1.c1;Parent=MALK_00110.t1
contig001	AUGUSTUS	CDS	208823	209000	1	+	0	ID=MALK_00110.t1.c2;Parent=MALK_00110.t1
contig001	AUGUSTUS	CDS	209038	209495	1	+	0	ID=MALK_00110.t1.c3;Parent=MALK_00110.t1
contig001	AUGUSTUS	mRNA	208781	209495	1	+	.	ID=MALK_00110.t1;Parent=MALK_00110
contig001	AUGUSTUS	exon	208781	208792	.	+	.	ID=MALK_00110.t1.e1;Parent=MALK_00110.t1
contig001	AUGUSTUS	exon	208823	209000	.	+	.	ID=MALK_00110.t1.e2;Parent=MALK_00110.t1
contig001	AUGUSTUS	exon	209038	209495	.	+	.	ID=MALK_00110.t1.e3;Parent=MALK_00110.t1
contig001	AUGUSTUS	gene	209526	211787	0.18	-	.	ID=MALK_00111;prediction_source=augustus:contig001.g435.t1
contig001	AUGUSTUS	CDS	211634	211787	0.54	-	0	ID=MALK_00111.t1.c4;Parent=MALK_00111.t1
contig001	AUGUSTUS	CDS	210992	211506	0.54	-	0	ID=MALK_00111.t1.c3;Parent=MALK_00111.t1
contig001	AUGUSTUS	CDS	210640	210805	0.54	-	0	ID=MALK_00111.t1.c2;Parent=MALK_00111.t1
contig001	AUGUSTUS	CDS	209526	210061	0.54	-	0	ID=MALK_00111.t1.c1;Parent=MALK_00111.t1
contig001	AUGUSTUS	mRNA	209526	211787	0.18	-	.	ID=MALK_00111.t1;Parent=MALK_00111
contig001	AUGUSTUS	exon	211634	211787	0.54	-	.	ID=MALK_00111.t1.e4;Parent=MALK_00111.t1
contig001	AUGUSTUS	exon	210992	211506	0.54	-	.	ID=MALK_00111.t1.e3;Parent=MALK_00111.t1
contig001	AUGUSTUS	exon	210640	210805	0.54	-	.	ID=MALK_00111.t1.e2;Parent=MALK_00111.t1
contig001	AUGUSTUS	exon	209526	210061	0.54	-	.	ID=MALK_00111.t1.e1;Parent=MALK_00111.t1
contig001	AUGUSTUS	gene	211903	212592	0.47	-	.	ID=MALK_00112;prediction_source=augustus:contig001.g436.t1
contig001	AUGUSTUS	CDS	211903	212592	0.47	-	0	ID=MALK_00112.t1.c1;Parent=MALK_00112.t1
contig001	AUGUSTUS	mRNA	211903	212592	0.47	-	.	ID=MALK_00112.t1;Parent=MALK_00112
contig001	AUGUSTUS	exon	211903	212592	0.47	-	.	ID=MALK_00112.t1.e1;Parent=MALK_00112.t1
contig001	AUGUSTUS	gene	213352	214019	0.25	-	.	ID=MALK_00113;prediction_source=braker_MRET:g773.t1
contig001	AUGUSTUS	CDS	213940	214019	0.29	-	0	ID=MALK_00113.t1.c2;Parent=MALK_00113.t1
contig001	AUGUSTUS	CDS	213352	213907	0.29	-	0	ID=MALK_00113.t1.c1;Parent=MALK_00113.t1
contig001	AUGUSTUS	mRNA	213352	214019	0.25	-	.	ID=MALK_00113.t1;Parent=MALK_00113
contig001	AUGUSTUS	exon	213940	214019	.	-	.	ID=MALK_00113.t1.e2;Parent=MALK_00113.t1
contig001	AUGUSTUS	exon	213352	213907	.	-	.	ID=MALK_00113.t1.e1;Parent=MALK_00113.t1
contig001	AUGUSTUS	gene	214203	215504	0.7	-	.	ID=MALK_00114;prediction_source=braker_MRET:g774.t1

contig001	AUGUSTUS	CDS	214203	215504	0.7	-	0	ID=MALK_00114.t1.c1;Parent=MALK_00114.t1
contig001	AUGUSTUS	mRNA	214203	215504	0.7	-	.	ID=MALK_00114.t1;Parent=MALK_00114
contig001	AUGUSTUS	exon	214203	215504	.	-	.	ID=MALK_00114.t1.e1;Parent=MALK_00114.t1
contig001	AUGUSTUS	gene	215641	216147	0.87	+	.	ID=MALK_00115;prediction_source=braker_MRET:g775.t1
contig001	AUGUSTUS	CDS	215641	216147	0.87	+	0	ID=MALK_00115.t1.c1;Parent=MALK_00115.t1
contig001	AUGUSTUS	mRNA	215641	216147	0.87	+	.	ID=MALK_00115.t1;Parent=MALK_00115
contig001	AUGUSTUS	exon	215641	216147	.	+	.	ID=MALK_00115.t1.e1;Parent=MALK_00115.t1
contig001	AUGUSTUS	gene	216314	217433	0.67	+	.	ID=MALK_00116;prediction_source=braker_MRET:g776.t1
contig001	AUGUSTUS	CDS	216314	217360	0.68	+	0	ID=MALK_00116.t1.c1;Parent=MALK_00116.t1
contig001	AUGUSTUS	CDS	217404	217433	0.68	+	0	ID=MALK_00116.t1.c2;Parent=MALK_00116.t1
contig001	AUGUSTUS	mRNA	216314	217433	0.67	+	.	ID=MALK_00116.t1;Parent=MALK_00116
contig001	AUGUSTUS	exon	216314	217360	.	+	.	ID=MALK_00116.t1.e1;Parent=MALK_00116.t1
contig001	AUGUSTUS	exon	217404	217433	.	+	.	ID=MALK_00116.t1.e2;Parent=MALK_00116.t1
contig001	maker	gene	217640	218668	.	-	.	ID=MALK_00117;prediction_source=maker_MRET:augustus_masked-contig001-processed-gene-2.32-mRNA-1
contig001	maker	CDS	217640	218668	.	-	0	ID=MALK_00117.t1.c1;Parent=MALK_00117.t1
contig001	maker	mRNA	217640	218668	.	-	.	ID=MALK_00117.t1;Parent=MALK_00117
contig001	maker	exon	217640	218668	.	-	.	ID=MALK_00117.t1.e1;Parent=MALK_00117.t1
contig001	AUGUSTUS	gene	218712	220412	0.73	-	.	ID=MALK_00118;prediction_source=augustus:contig001.g441.t1
contig001	AUGUSTUS	CDS	218712	220412	0.73	-	0	ID=MALK_00118.t1.c1;Parent=MALK_00118.t1
contig001	AUGUSTUS	mRNA	218712	220412	0.73	-	.	ID=MALK_00118.t1;Parent=MALK_00118
contig001	AUGUSTUS	exon	218712	220412	0.73	-	.	ID=MALK_00118.t1.e1;Parent=MALK_00118.t1
contig001	AUGUSTUS	gene	220790	221668	0.55	+	.	ID=MALK_00119;prediction_source=augustus:contig001.g442.t1
contig001	AUGUSTUS	CDS	220790	221668	0.55	+	0	ID=MALK_00119.t1.c1;Parent=MALK_00119.t1
contig001	AUGUSTUS	mRNA	220790	221668	0.55	+	.	ID=MALK_00119.t1;Parent=MALK_00119
contig001	AUGUSTUS	exon	220790	221668	0.55	+	.	ID=MALK_00119.t1.e1;Parent=MALK_00119.t1
contig001	AUGUSTUS	gene	221617	223116	0.31	-	.	ID=MALK_00120;prediction_source=augustus:contig001.g443.t1
contig001	AUGUSTUS	CDS	221617	223116	0.31	-	0	ID=MALK_00120.t1.c1;Parent=MALK_00120.t1
contig001	AUGUSTUS	mRNA	221617	223116	0.31	-	.	ID=MALK_00120.t1;Parent=MALK_00120
contig001	AUGUSTUS	exon	221617	223116	0.31	-	.	ID=MALK_00120.t1.e1;Parent=MALK_00120.t1
contig001	AUGUSTUS	gene	223570	224832	0.84	+	.	ID=MALK_00121;prediction_source=augustus:contig001.g446.t1
contig001	AUGUSTUS	CDS	223570	224832	0.84	+	0	ID=MALK_00121.t1.c1;Parent=MALK_00121.t1
contig001	AUGUSTUS	mRNA	223570	224832	0.84	+	.	ID=MALK_00121.t1;Parent=MALK_00121
contig001	AUGUSTUS	exon	223570	224832	0.84	+	.	ID=MALK_00121.t1.e1;Parent=MALK_00121.t1
contig001	AUGUSTUS	gene	225734	227203	1	-	.	ID=MALK_00122;prediction_source=braker_MRET:g782.t1
contig001	AUGUSTUS	CDS	225734	227203	1	-	0	ID=MALK_00122.t1.c1;Parent=MALK_00122.t1
contig001	AUGUSTUS	mRNA	225734	227203	1	-	.	ID=MALK_00122.t1;Parent=MALK_00122
contig001	AUGUSTUS	exon	225734	227203	.	-	.	ID=MALK_00122.t1.e1;Parent=MALK_00122.t1
contig001	maker	gene	227270	229444	.	-	.	ID=MALK_00123;prediction_source=maker_MRET:augustus_masked-contig001-processed-gene-2.36-mRNA-1
contig001	maker	CDS	227270	229444	.	-	0	ID=MALK_00123.t1.c1;Parent=MALK_00123.t1
contig001	maker	mRNA	227270	229444	.	-	.	ID=MALK_00123.t1;Parent=MALK_00123
contig001	maker	exon	227270	229444	.	-	.	ID=MALK_00123.t1.e1;Parent=MALK_00123.t1
contig001	maker	gene	229551	231167	.	-	.	ID=MALK_00124;prediction_source=maker_MRET:augustus_masked-contig001-processed-gene-2.37-mRNA-1
contig001	maker	CDS	229551	231167	.	-	0	ID=MALK_00124.t1.c1;Parent=MALK_00124.t1

contig001	maker	mRNA	229551	231167	.	-	.	ID=MALK_00124.t1;Parent=MALK_00124
contig001	maker	exon	229551	231167	.	-	.	ID=MALK_00124.t1.e1;Parent=MALK_00124.t1
contig001	AUGUSTUS	gene	231530	232459	0.27	-	.	ID=MALK_00125;prediction_source=augustus:contig001.g451.t1
contig001	AUGUSTUS	CDS	231530	232459	0.27	-	0	ID=MALK_00125.t1.c1;Parent=MALK_00125.t1
contig001	AUGUSTUS	mRNA	231530	232459	0.27	-	.	ID=MALK_00125.t1;Parent=MALK_00125
contig001	AUGUSTUS	exon	231530	232459	0.27	-	.	ID=MALK_00125.t1.e1;Parent=MALK_00125.t1
contig001	maker	gene	232561	233625	.	-	.	ID=MALK_00126;prediction_source=maker_MRET:augustus_masked-contig001-processed-gene-2.39-mRNA-1
contig001	maker	CDS	232561	233625	.	-	0	ID=MALK_00126.t1.c1;Parent=MALK_00126.t1
contig001	maker	mRNA	232561	233625	.	-	.	ID=MALK_00126.t1;Parent=MALK_00126
contig001	maker	exon	232561	233625	.	-	.	ID=MALK_00126.t1.e1;Parent=MALK_00126.t1
contig001	AUGUSTUS	gene	233728	235719	0.28	+	.	ID=MALK_00127;prediction_source=braker_MRET:g786.t1
contig001	AUGUSTUS	CDS	233728	235719	0.28	+	0	ID=MALK_00127.t1.c1;Parent=MALK_00127.t1
contig001	AUGUSTUS	mRNA	233728	235719	0.28	+	.	ID=MALK_00127.t1;Parent=MALK_00127
contig001	AUGUSTUS	exon	233728	235719	.	+	.	ID=MALK_00127.t1.e1;Parent=MALK_00127.t1
contig001	AUGUSTUS	gene	235929	236612	0.12	+	.	ID=MALK_00128;prediction_source=braker_MRET:g787.t1
contig001	AUGUSTUS	CDS	235929	236612	0.12	+	0	ID=MALK_00128.t1.c1;Parent=MALK_00128.t1
contig001	AUGUSTUS	mRNA	235929	236612	0.12	+	.	ID=MALK_00128.t1;Parent=MALK_00128
contig001	AUGUSTUS	exon	235929	236612	.	+	.	ID=MALK_00128.t1.e1;Parent=MALK_00128.t1
contig001	AUGUSTUS	gene	236927	238108	0.58	+	.	ID=MALK_00129;prediction_source=braker_MRET:g788.t1
contig001	AUGUSTUS	CDS	236927	238108	0.58	+	0	ID=MALK_00129.t1.c1;Parent=MALK_00129.t1
contig001	AUGUSTUS	mRNA	236927	238108	0.58	+	.	ID=MALK_00129.t1;Parent=MALK_00129
contig001	AUGUSTUS	exon	236927	238108	.	+	.	ID=MALK_00129.t1.e1;Parent=MALK_00129.t1
contig001	maker	gene	238227	238940	.	-	.	ID=MALK_00130;prediction_source=maker_MRET:augustus_masked-contig001-processed-gene-2.40-mRNA-1
contig001	maker	CDS	238227	238940	.	-	0	ID=MALK_00130.t1.c1;Parent=MALK_00130.t1
contig001	maker	mRNA	238227	238940	.	-	.	ID=MALK_00130.t1;Parent=MALK_00130
contig001	maker	exon	238227	238940	.	-	.	ID=MALK_00130.t1.e1;Parent=MALK_00130.t1
contig001	AUGUSTUS	gene	239153	239665	0.7	-	.	ID=MALK_00131;prediction_source=augustus:contig001.g458.t1
contig001	AUGUSTUS	CDS	239153	239665	0.7	-	0	ID=MALK_00131.t1.c1;Parent=MALK_00131.t1
contig001	AUGUSTUS	mRNA	239153	239665	0.7	-	.	ID=MALK_00131.t1;Parent=MALK_00131
contig001	AUGUSTUS	exon	239153	239665	0.7	-	.	ID=MALK_00131.t1.e1;Parent=MALK_00131.t1
contig001	AUGUSTUS	gene	239848	241088	0.39	-	.	ID=MALK_00132;prediction_source=augustus:contig001.g459.t1
contig001	AUGUSTUS	CDS	240378	241088	0.6	-	0	ID=MALK_00132.t1.c2;Parent=MALK_00132.t1
contig001	AUGUSTUS	CDS	239848	240291	0.6	-	0	ID=MALK_00132.t1.c1;Parent=MALK_00132.t1
contig001	AUGUSTUS	mRNA	239848	241088	0.39	-	.	ID=MALK_00132.t1;Parent=MALK_00132
contig001	AUGUSTUS	exon	240378	241088	0.6	-	.	ID=MALK_00132.t1.e2;Parent=MALK_00132.t1
contig001	AUGUSTUS	exon	239848	240291	0.6	-	.	ID=MALK_00132.t1.e1;Parent=MALK_00132.t1
contig001	AUGUSTUS	gene	241139	241813	0.6	-	.	ID=MALK_00133;prediction_source=augustus:contig001.g460.t1
contig001	AUGUSTUS	CDS	241139	241813	0.6	-	0	ID=MALK_00133.t1.c1;Parent=MALK_00133.t1
contig001	AUGUSTUS	mRNA	241139	241813	0.6	-	.	ID=MALK_00133.t1;Parent=MALK_00133
contig001	AUGUSTUS	exon	241139	241813	0.6	-	.	ID=MALK_00133.t1.e1;Parent=MALK_00133.t1
contig001	maker	gene	242514	242939	.	+	.	ID=MALK_00134;prediction_source=maker_MRET:augustus_masked-contig001-processed-gene-2.8-mRNA-1
contig001	maker	CDS	242514	242939	.	+	0	ID=MALK_00134.t1.c1;Parent=MALK_00134.t1
contig001	maker	mRNA	242514	242939	.	+	.	ID=MALK_00134.t1;Parent=MALK_00134

contig001	maker	exon	242514	242939	.	+	.	ID=MALK_00134.t1.e1;Parent=MALK_00134.t1
contig001	AUGUSTUS	gene	242967	243704	0.64	-	.	ID=MALK_00135;prediction_source=braker_MRET:g792.t1
contig001	AUGUSTUS	CDS	243627	243704	1	-	0	ID=MALK_00135.t1.c4;Parent=MALK_00135.t1
contig001	AUGUSTUS	CDS	243485	243583	1	-	0	ID=MALK_00135.t1.c3;Parent=MALK_00135.t1
contig001	AUGUSTUS	CDS	243324	243453	1	-	0	ID=MALK_00135.t1.c2;Parent=MALK_00135.t1
contig001	AUGUSTUS	CDS	242967	243289	1	-	0	ID=MALK_00135.t1.c1;Parent=MALK_00135.t1
contig001	AUGUSTUS	mRNA	242967	243704	0.64	-	.	ID=MALK_00135.t1;Parent=MALK_00135
contig001	AUGUSTUS	exon	243627	243704	.	-	.	ID=MALK_00135.t1.e4;Parent=MALK_00135.t1
contig001	AUGUSTUS	exon	243485	243583	.	-	.	ID=MALK_00135.t1.e3;Parent=MALK_00135.t1
contig001	AUGUSTUS	exon	243324	243453	.	-	.	ID=MALK_00135.t1.e2;Parent=MALK_00135.t1
contig001	AUGUSTUS	exon	242967	243289	.	-	.	ID=MALK_00135.t1.e1;Parent=MALK_00135.t1
contig001	AUGUSTUS	gene	243882	245321	1	+	.	ID=MALK_00136;prediction_source=augustus:contig001.g462.t1
contig001	AUGUSTUS	CDS	243882	245321	1	+	0	ID=MALK_00136.t1.c1;Parent=MALK_00136.t1
contig001	AUGUSTUS	mRNA	243882	245321	1	+	.	ID=MALK_00136.t1;Parent=MALK_00136
contig001	AUGUSTUS	exon	243882	245321	1	+	.	ID=MALK_00136.t1.e1;Parent=MALK_00136.t1
contig001	AUGUSTUS	gene	245380	246701	0.49	-	.	ID=MALK_00137;prediction_source=braker_MRET:g794.t1
contig001	AUGUSTUS	CDS	245522	246701	0.96	-	0	ID=MALK_00137.t1.c2;Parent=MALK_00137.t1
contig001	AUGUSTUS	CDS	245380	245402	0.96	-	0	ID=MALK_00137.t1.c1;Parent=MALK_00137.t1
contig001	AUGUSTUS	mRNA	245380	246701	0.49	-	.	ID=MALK_00137.t1;Parent=MALK_00137
contig001	AUGUSTUS	exon	245522	246701	.	-	.	ID=MALK_00137.t1.e2;Parent=MALK_00137.t1
contig001	AUGUSTUS	exon	245380	245402	.	-	.	ID=MALK_00137.t1.e1;Parent=MALK_00137.t1
contig001	AUGUSTUS	gene	246752	247675	0.52	+	.	ID=MALK_00138;prediction_source=braker_MRET:g795.t2
contig001	AUGUSTUS	CDS	246752	247675	0.52	+	0	ID=MALK_00138.t1.c1;Parent=MALK_00138.t1
contig001	AUGUSTUS	mRNA	246752	247675	0.52	+	.	ID=MALK_00138.t1;Parent=MALK_00138
contig001	AUGUSTUS	exon	246752	247675	.	+	.	ID=MALK_00138.t1.e1;Parent=MALK_00138.t1
contig001	AUGUSTUS	gene	248405	248806	0.79	-	.	ID=MALK_00139;prediction_source=braker_MRET:g796.t1
contig001	AUGUSTUS	CDS	248405	248806	0.79	-	0	ID=MALK_00139.t1.c1;Parent=MALK_00139.t1
contig001	AUGUSTUS	mRNA	248405	248806	0.79	-	.	ID=MALK_00139.t1;Parent=MALK_00139
contig001	AUGUSTUS	exon	248405	248806	.	-	.	ID=MALK_00139.t1.e1;Parent=MALK_00139.t1
contig001	AUGUSTUS	gene	248922	249266	0.51	+	.	ID=MALK_00140;prediction_source=augustus:contig001.g465.t1
contig001	AUGUSTUS	CDS	248922	249266	0.51	+	0	ID=MALK_00140.t1.c1;Parent=MALK_00140.t1
contig001	AUGUSTUS	mRNA	248922	249266	0.51	+	.	ID=MALK_00140.t1;Parent=MALK_00140
contig001	AUGUSTUS	exon	248922	249266	0.51	+	.	ID=MALK_00140.t1.e1;Parent=MALK_00140.t1
contig001	AUGUSTUS	gene	249267	249839	0.84	-	.	ID=MALK_00141;prediction_source=augustus:contig001.g466.t1
contig001	AUGUSTUS	CDS	249267	249839	0.84	-	0	ID=MALK_00141.t1.c1;Parent=MALK_00141.t1
contig001	AUGUSTUS	mRNA	249267	249839	0.84	-	.	ID=MALK_00141.t1;Parent=MALK_00141
contig001	AUGUSTUS	exon	249267	249839	0.84	-	.	ID=MALK_00141.t1.e1;Parent=MALK_00141.t1
contig001	AUGUSTUS	gene	250012	250806	0.73	+	.	ID=MALK_00142;prediction_source=braker_MRET:g799.t1
contig001	AUGUSTUS	CDS	250012	250806	0.73	+	0	ID=MALK_00142.t1.c1;Parent=MALK_00142.t1
contig001	AUGUSTUS	mRNA	250012	250806	0.73	+	.	ID=MALK_00142.t1;Parent=MALK_00142
contig001	AUGUSTUS	exon	250012	250806	.	+	.	ID=MALK_00142.t1.e1;Parent=MALK_00142.t1
contig001	AUGUSTUS	gene	250914	252160	0.84	-	.	ID=MALK_00143;prediction_source=braker_MRET:g800.t1
contig001	AUGUSTUS	CDS	250982	252160	0.94	-	0	ID=MALK_00143.t1.c2;Parent=MALK_00143.t1

contig001	AUGUSTUS	CDS	250914	250931	0.94	-	0	ID=MALK_00143.t1.c1;Parent=MALK_00143.t1
contig001	AUGUSTUS	mRNA	250914	252160	0.84	-	.	ID=MALK_00143.t1;Parent=MALK_00143
contig001	AUGUSTUS	exon	250982	252160	.	-	.	ID=MALK_00143.t1.e2;Parent=MALK_00143.t1
contig001	AUGUSTUS	exon	250914	250931	.	-	.	ID=MALK_00143.t1.e1;Parent=MALK_00143.t1
contig001	AUGUSTUS	gene	252308	253159	0.65	+	.	ID=MALK_00144;prediction_source=augustus:contig001.g467.t1
contig001	AUGUSTUS	CDS	252308	253159	0.65	+	0	ID=MALK_00144.t1.c1;Parent=MALK_00144.t1
contig001	AUGUSTUS	mRNA	252308	253159	0.65	+	.	ID=MALK_00144.t1;Parent=MALK_00144
contig001	AUGUSTUS	exon	252308	253159	0.65	+	.	ID=MALK_00144.t1.e1;Parent=MALK_00144.t1
contig001	AUGUSTUS	gene	253113	255170	0.99	-	.	ID=MALK_00145;prediction_source=augustus:contig001.g468.t1
contig001	AUGUSTUS	CDS	253113	255170	0.99	-	0	ID=MALK_00145.t1.c1;Parent=MALK_00145.t1
contig001	AUGUSTUS	mRNA	253113	255170	0.99	-	.	ID=MALK_00145.t1;Parent=MALK_00145
contig001	AUGUSTUS	exon	253113	255170	0.99	-	.	ID=MALK_00145.t1.e1;Parent=MALK_00145.t1
contig001	AUGUSTUS	gene	255461	256291	0.72	+	.	ID=MALK_00146;prediction_source=augustus:contig001.g470.t1
contig001	AUGUSTUS	CDS	255461	256291	0.72	+	0	ID=MALK_00146.t1.c1;Parent=MALK_00146.t1
contig001	AUGUSTUS	mRNA	255461	256291	0.72	+	.	ID=MALK_00146.t1;Parent=MALK_00146
contig001	AUGUSTUS	exon	255461	256291	0.72	+	.	ID=MALK_00146.t1.e1;Parent=MALK_00146.t1
contig001	AUGUSTUS	gene	256293	259976	0.99	-	.	ID=MALK_00147;prediction_source=augustus:contig001.g471.t1
contig001	AUGUSTUS	CDS	256293	259976	0.99	-	0	ID=MALK_00147.t1.c1;Parent=MALK_00147.t1
contig001	AUGUSTUS	mRNA	256293	259976	0.99	-	.	ID=MALK_00147.t1;Parent=MALK_00147
contig001	AUGUSTUS	exon	256293	259976	0.99	-	.	ID=MALK_00147.t1.e1;Parent=MALK_00147.t1
contig001	AUGUSTUS	gene	260078	260960	0.66	+	.	ID=MALK_00148;prediction_source=braker_MRET:g805.t1
contig001	AUGUSTUS	CDS	260078	260908	0.66	+	0	ID=MALK_00148.t1.c1;Parent=MALK_00148.t1
contig001	AUGUSTUS	CDS	260943	260960	0.66	+	0	ID=MALK_00148.t1.c2;Parent=MALK_00148.t1
contig001	AUGUSTUS	mRNA	260078	260960	0.66	+	.	ID=MALK_00148.t1;Parent=MALK_00148
contig001	AUGUSTUS	exon	260078	260908	.	+	.	ID=MALK_00148.t1.e1;Parent=MALK_00148.t1
contig001	AUGUSTUS	exon	260943	260960	.	+	.	ID=MALK_00148.t1.e2;Parent=MALK_00148.t1
contig001	AUGUSTUS	gene	260987	262537	0.99	-	.	ID=MALK_00149;prediction_source=augustus:contig001.g473.t1
contig001	AUGUSTUS	CDS	260987	262537	0.99	-	0	ID=MALK_00149.t1.c1;Parent=MALK_00149.t1
contig001	AUGUSTUS	mRNA	260987	262537	0.99	-	.	ID=MALK_00149.t1;Parent=MALK_00149
contig001	AUGUSTUS	exon	260987	262537	0.99	-	.	ID=MALK_00149.t1.e1;Parent=MALK_00149.t1
contig001	AUGUSTUS	gene	263373	264335	0.08	+	.	ID=MALK_00150;prediction_source=braker_MRET:g807.t1
contig001	AUGUSTUS	CDS	263373	263481	1	+	0	ID=MALK_00150.t1.c1;Parent=MALK_00150.t1
contig001	AUGUSTUS	CDS	263509	263603	1	+	0	ID=MALK_00150.t1.c2;Parent=MALK_00150.t1
contig001	AUGUSTUS	CDS	263632	263759	1	+	0	ID=MALK_00150.t1.c3;Parent=MALK_00150.t1
contig001	AUGUSTUS	CDS	263789	264335	1	+	0	ID=MALK_00150.t1.c4;Parent=MALK_00150.t1
contig001	AUGUSTUS	mRNA	263373	264335	0.08	+	.	ID=MALK_00150.t1;Parent=MALK_00150
contig001	AUGUSTUS	exon	263373	263481	.	+	.	ID=MALK_00150.t1.e1;Parent=MALK_00150.t1
contig001	AUGUSTUS	exon	263509	263603	.	+	.	ID=MALK_00150.t1.e2;Parent=MALK_00150.t1
contig001	AUGUSTUS	exon	263632	263759	.	+	.	ID=MALK_00150.t1.e3;Parent=MALK_00150.t1
contig001	AUGUSTUS	exon	263789	264335	.	+	.	ID=MALK_00150.t1.e4;Parent=MALK_00150.t1
contig001	maker	gene	264489	265523	.	+	.	ID=MALK_00151;prediction_source=maker_MRET:augustus_masked-contig001-processed-gene-2.14-mRNA-1
contig001	maker	CDS	264489	265523	.	+	0	ID=MALK_00151.t1.c1;Parent=MALK_00151.t1
contig001	maker	mRNA	264489	265523	.	+	.	ID=MALK_00151.t1;Parent=MALK_00151

contig001	maker	exon	264489	265523	.	+	.	ID=MALK_00151.t1.e1;Parent=MALK_00151.t1
contig001	AUGUSTUS	gene	265624	266676	0.72	-	.	ID=MALK_00152;prediction_source=augustus:contig001.g477.t1
contig001	AUGUSTUS	CDS	265624	266676	0.72	-	0	ID=MALK_00152.t1.c1;Parent=MALK_00152.t1
contig001	AUGUSTUS	mRNA	265624	266676	0.72	-	.	ID=MALK_00152.t1;Parent=MALK_00152
contig001	AUGUSTUS	exon	265624	266676	0.72	-	.	ID=MALK_00152.t1.e1;Parent=MALK_00152.t1
contig001	AUGUSTUS	gene	266773	267609	0.98	+	.	ID=MALK_00153;prediction_source=augustus:contig001.g478.t1
contig001	AUGUSTUS	CDS	266773	267609	0.98	+	0	ID=MALK_00153.t1.c1;Parent=MALK_00153.t1
contig001	AUGUSTUS	mRNA	266773	267609	0.98	+	.	ID=MALK_00153.t1;Parent=MALK_00153
contig001	AUGUSTUS	exon	266773	267609	0.98	+	.	ID=MALK_00153.t1.e1;Parent=MALK_00153.t1
contig001	AUGUSTUS	gene	267637	268797	0.99	-	.	ID=MALK_00154;prediction_source=augustus:contig001.g479.t1
contig001	AUGUSTUS	CDS	267637	268797	0.99	-	0	ID=MALK_00154.t1.c1;Parent=MALK_00154.t1
contig001	AUGUSTUS	mRNA	267637	268797	0.99	-	.	ID=MALK_00154.t1;Parent=MALK_00154
contig001	AUGUSTUS	exon	267637	268797	0.99	-	.	ID=MALK_00154.t1.e1;Parent=MALK_00154.t1
contig001	maker	gene	269829	270470	.	+	.	ID=MALK_00155;prediction_source=maker_MRET:augustus_masked-contig001-processed-gene-2.16-mRNA-1
contig001	maker	CDS	269829	270470	.	+	0	ID=MALK_00155.t1.c1;Parent=MALK_00155.t1
contig001	maker	mRNA	269829	270470	.	+	.	ID=MALK_00155.t1;Parent=MALK_00155
contig001	maker	exon	269829	270470	.	+	.	ID=MALK_00155.t1.e1;Parent=MALK_00155.t1
contig001	maker	gene	271046	272833	.	+	.	ID=MALK_00156;prediction_source=maker_MRET:augustus_masked-contig001-processed-gene-2.17-mRNA-1
contig001	maker	CDS	271046	272833	.	+	0	ID=MALK_00156.t1.c1;Parent=MALK_00156.t1
contig001	maker	mRNA	271046	272833	.	+	.	ID=MALK_00156.t1;Parent=MALK_00156
contig001	maker	exon	271046	272833	.	+	.	ID=MALK_00156.t1.e1;Parent=MALK_00156.t1
contig001	AUGUSTUS	gene	272900	274618	0.74	-	.	ID=MALK_00157;prediction_source=augustus:contig001.g482.t1
contig001	AUGUSTUS	CDS	272900	274618	0.74	-	0	ID=MALK_00157.t1.c1;Parent=MALK_00157.t1
contig001	AUGUSTUS	mRNA	272900	274618	0.74	-	.	ID=MALK_00157.t1;Parent=MALK_00157
contig001	AUGUSTUS	exon	272900	274618	0.74	-	.	ID=MALK_00157.t1.e1;Parent=MALK_00157.t1
contig001	AUGUSTUS	gene	275062	276144	0.83	-	.	ID=MALK_00158;prediction_source=augustus:contig001.g483.t1
contig001	AUGUSTUS	CDS	275062	276144	0.83	-	0	ID=MALK_00158.t1.c1;Parent=MALK_00158.t1
contig001	AUGUSTUS	mRNA	275062	276144	0.83	-	.	ID=MALK_00158.t1;Parent=MALK_00158
contig001	AUGUSTUS	exon	275062	276144	0.83	-	.	ID=MALK_00158.t1.e1;Parent=MALK_00158.t1
contig001	AUGUSTUS	gene	276280	278850	0.99	+	.	ID=MALK_00159;prediction_source=augustus:contig001.g484.t1
contig001	AUGUSTUS	CDS	276280	278850	0.99	+	0	ID=MALK_00159.t1.c1;Parent=MALK_00159.t1
contig001	AUGUSTUS	mRNA	276280	278850	0.99	+	.	ID=MALK_00159.t1;Parent=MALK_00159
contig001	AUGUSTUS	exon	276280	278850	0.99	+	.	ID=MALK_00159.t1.e1;Parent=MALK_00159.t1
contig001	AUGUSTUS	gene	278874	279683	0.78	-	.	ID=MALK_00160;prediction_source=augustus:contig001.g485.t1
contig001	AUGUSTUS	CDS	278874	279683	0.78	-	0	ID=MALK_00160.t1.c1;Parent=MALK_00160.t1
contig001	AUGUSTUS	mRNA	278874	279683	0.78	-	.	ID=MALK_00160.t1;Parent=MALK_00160
contig001	AUGUSTUS	exon	278874	279683	0.78	-	.	ID=MALK_00160.t1.e1;Parent=MALK_00160.t1
contig001	AUGUSTUS	gene	279809	283531	1	+	.	ID=MALK_00161;prediction_source=augustus:contig001.g486.t1
contig001	AUGUSTUS	CDS	279809	283531	1	+	0	ID=MALK_00161.t1.c1;Parent=MALK_00161.t1
contig001	AUGUSTUS	mRNA	279809	283531	1	+	.	ID=MALK_00161.t1;Parent=MALK_00161
contig001	AUGUSTUS	exon	279809	283531	1	+	.	ID=MALK_00161.t1.e1;Parent=MALK_00161.t1
contig001	maker	gene	283482	285272	.	-	.	ID=MALK_00162;prediction_source=maker_MRET:augustus_masked-contig001-processed-gene-2.52-mRNA-1
contig001	maker	CDS	283482	285272	.	-	0	ID=MALK_00162.t1.c1;Parent=MALK_00162.t1

contig001	maker	mRNA	283482	285272	.	-	.	ID=MALK_00162.t1;Parent=MALK_00162
contig001	maker	exon	283482	285272	.	-	.	ID=MALK_00162.t1.e1;Parent=MALK_00162.t1
contig001	AUGUSTUS	gene	285398	285756	0.53	+	.	ID=MALK_00163;prediction_source=braker_MRET:g820.t1
contig001	AUGUSTUS	CDS	285398	285491	0.98	+	0	ID=MALK_00163.t1.c1;Parent=MALK_00163.t1
contig001	AUGUSTUS	CDS	285629	285756	0.98	+	0	ID=MALK_00163.t1.c2;Parent=MALK_00163.t1
contig001	AUGUSTUS	mRNA	285398	285756	0.53	+	.	ID=MALK_00163.t1;Parent=MALK_00163
contig001	AUGUSTUS	exon	285398	285491	.	+	.	ID=MALK_00163.t1.e1;Parent=MALK_00163.t1
contig001	AUGUSTUS	exon	285629	285756	.	+	.	ID=MALK_00163.t1.e2;Parent=MALK_00163.t1
contig001	AUGUSTUS	gene	285846	286423	0.54	-	.	ID=MALK_00164;prediction_source=braker_MRET:g821.t1
contig001	AUGUSTUS	CDS	286078	286423	0.62	-	0	ID=MALK_00164.t1.c2;Parent=MALK_00164.t1
contig001	AUGUSTUS	CDS	285846	286048	0.62	-	0	ID=MALK_00164.t1.c1;Parent=MALK_00164.t1
contig001	AUGUSTUS	mRNA	285846	286423	0.54	-	.	ID=MALK_00164.t1;Parent=MALK_00164
contig001	AUGUSTUS	exon	286078	286423	.	-	.	ID=MALK_00164.t1.e2;Parent=MALK_00164.t1
contig001	AUGUSTUS	exon	285846	286048	.	-	.	ID=MALK_00164.t1.e1;Parent=MALK_00164.t1
contig001	AUGUSTUS	gene	286453	287487	0.64	+	.	ID=MALK_00165;prediction_source=augustus:contig001.g488.t1
contig001	AUGUSTUS	CDS	286453	287487	0.64	+	0	ID=MALK_00165.t1.c1;Parent=MALK_00165.t1
contig001	AUGUSTUS	mRNA	286453	287487	0.64	+	.	ID=MALK_00165.t1;Parent=MALK_00165
contig001	AUGUSTUS	exon	286453	287487	0.64	+	.	ID=MALK_00165.t1.e1;Parent=MALK_00165.t1
contig001	AUGUSTUS	gene	287593	288708	0.81	+	.	ID=MALK_00166;prediction_source=braker_MRET:g823.t1
contig001	AUGUSTUS	CDS	287593	288708	0.81	+	0	ID=MALK_00166.t1.c1;Parent=MALK_00166.t1
contig001	AUGUSTUS	mRNA	287593	288708	0.81	+	.	ID=MALK_00166.t1;Parent=MALK_00166
contig001	AUGUSTUS	exon	287593	288708	.	+	.	ID=MALK_00166.t1.e1;Parent=MALK_00166.t1
contig001	AUGUSTUS	gene	288710	289582	0.36	-	.	ID=MALK_00167;prediction_source=braker_MRET:g824.t1
contig001	AUGUSTUS	CDS	288710	289582	0.36	-	0	ID=MALK_00167.t1.c1;Parent=MALK_00167.t1
contig001	AUGUSTUS	mRNA	288710	289582	0.36	-	.	ID=MALK_00167.t1;Parent=MALK_00167
contig001	AUGUSTUS	exon	288710	289582	.	-	.	ID=MALK_00167.t1.e1;Parent=MALK_00167.t1
contig001	AUGUSTUS	gene	291306	291686	1	+	.	ID=MALK_00168;prediction_source=augustus:contig001.g490.t1
contig001	AUGUSTUS	CDS	291306	291686	1	+	0	ID=MALK_00168.t1.c1;Parent=MALK_00168.t1
contig001	AUGUSTUS	mRNA	291306	291686	1	+	.	ID=MALK_00168.t1;Parent=MALK_00168
contig001	AUGUSTUS	exon	291306	291686	1	+	.	ID=MALK_00168.t1.e1;Parent=MALK_00168.t1
contig001	AUGUSTUS	gene	291721	293712	0.34	-	.	ID=MALK_00169;prediction_source=braker_MRET:g826.t1
contig001	AUGUSTUS	CDS	291721	293712	0.34	-	0	ID=MALK_00169.t1.c1;Parent=MALK_00169.t1
contig001	AUGUSTUS	mRNA	291721	293712	0.34	-	.	ID=MALK_00169.t1;Parent=MALK_00169
contig001	AUGUSTUS	exon	291721	293712	.	-	.	ID=MALK_00169.t1.e1;Parent=MALK_00169.t1
contig001	AUGUSTUS	gene	294176	295468	1	-	.	ID=MALK_00170;prediction_source=augustus:contig001.g492.t1
contig001	AUGUSTUS	CDS	294176	295468	1	-	0	ID=MALK_00170.t1.c1;Parent=MALK_00170.t1
contig001	AUGUSTUS	mRNA	294176	295468	1	-	.	ID=MALK_00170.t1;Parent=MALK_00170
contig001	AUGUSTUS	exon	294176	295468	1	-	.	ID=MALK_00170.t1.e1;Parent=MALK_00170.t1
contig001	maker	gene	295613	298651	.	-	.	ID=MALK_00171;prediction_source=maker_MRET:augustus_masked-contig001-processed-gene-3.51-mRNA-1
contig001	maker	CDS	295613	298651	.	-	0	ID=MALK_00171.t1.c1;Parent=MALK_00171.t1
contig001	maker	mRNA	295613	298651	.	-	.	ID=MALK_00171.t1;Parent=MALK_00171
contig001	maker	exon	295613	298651	.	-	.	ID=MALK_00171.t1.e1;Parent=MALK_00171.t1
contig001	maker	gene	298936	301844	.	+	.	ID=MALK_00172;prediction_source=maker_MRET:augustus_masked-contig001-processed-gene-3.49-mRNA-1

contig001	maker	CDS	298936	299139	.	+	0	ID=MALK_00172.t1.c1;Parent=MALK_00172.t1
contig001	maker	CDS	299284	299439	.	+	0	ID=MALK_00172.t1.c2;Parent=MALK_00172.t1
contig001	maker	CDS	300385	300609	.	+	0	ID=MALK_00172.t1.c3;Parent=MALK_00172.t1
contig001	maker	CDS	300771	301844	.	+	0	ID=MALK_00172.t1.c4;Parent=MALK_00172.t1
contig001	maker	mRNA	298936	301844	.	+	.	ID=MALK_00172.t1;Parent=MALK_00172
contig001	maker	exon	298936	299139	.	+	.	ID=MALK_00172.t1.e1;Parent=MALK_00172.t1
contig001	maker	exon	299284	299439	.	+	.	ID=MALK_00172.t1.e2;Parent=MALK_00172.t1
contig001	maker	exon	300385	300609	.	+	.	ID=MALK_00172.t1.e3;Parent=MALK_00172.t1
contig001	maker	exon	300771	301844	.	+	.	ID=MALK_00172.t1.e4;Parent=MALK_00172.t1
contig001	maker	gene	301849	303414	.	-	.	ID=MALK_00173;prediction_source=maker_MRET:augustus_masked-contig001-processed-gene-3.73-mRNA-1
contig001	maker	CDS	301849	303414	.	-	0	ID=MALK_00173.t1.c1;Parent=MALK_00173.t1
contig001	maker	mRNA	301849	303414	.	-	.	ID=MALK_00173.t1;Parent=MALK_00173
contig001	maker	exon	301849	303414	.	-	.	ID=MALK_00173.t1.e1;Parent=MALK_00173.t1
contig001	AUGUSTUS	gene	303508	304056	0.65	+	.	ID=MALK_00174;prediction_source=augustus:contig001.g496.t1
contig001	AUGUSTUS	CDS	303508	304056	0.65	+	0	ID=MALK_00174.t1.c1;Parent=MALK_00174.t1
contig001	AUGUSTUS	mRNA	303508	304056	0.65	+	.	ID=MALK_00174.t1;Parent=MALK_00174
contig001	AUGUSTUS	exon	303508	304056	0.65	+	.	ID=MALK_00174.t1.e1;Parent=MALK_00174.t1
contig001	AUGUSTUS	gene	304062	305492	0.86	-	.	ID=MALK_00175;prediction_source=augustus:contig001.g497.t1
contig001	AUGUSTUS	CDS	304062	305492	0.86	-	0	ID=MALK_00175.t1.c1;Parent=MALK_00175.t1
contig001	AUGUSTUS	mRNA	304062	305492	0.86	-	.	ID=MALK_00175.t1;Parent=MALK_00175
contig001	AUGUSTUS	exon	304062	305492	0.86	-	.	ID=MALK_00175.t1.e1;Parent=MALK_00175.t1
contig001	AUGUSTUS	gene	305742	307961	0.9	+	.	ID=MALK_00176;prediction_source=augustus:contig001.g498.t1
contig001	AUGUSTUS	CDS	305742	307961	0.9	+	0	ID=MALK_00176.t1.c1;Parent=MALK_00176.t1
contig001	AUGUSTUS	mRNA	305742	307961	0.9	+	.	ID=MALK_00176.t1;Parent=MALK_00176
contig001	AUGUSTUS	exon	305742	307961	0.9	+	.	ID=MALK_00176.t1.e1;Parent=MALK_00176.t1
contig001	AUGUSTUS	gene	308071	309327	0.68	+	.	ID=MALK_00177;prediction_source=braker_MRET:g834.t1
contig001	AUGUSTUS	CDS	308071	309327	0.68	+	0	ID=MALK_00177.t1.c1;Parent=MALK_00177.t1
contig001	AUGUSTUS	mRNA	308071	309327	0.68	+	.	ID=MALK_00177.t1;Parent=MALK_00177
contig001	AUGUSTUS	exon	308071	309327	.	+	.	ID=MALK_00177.t1.e1;Parent=MALK_00177.t1
contig001	maker	gene	309639	313496	.	+	.	ID=MALK_00178;prediction_source=maker_MRET:augustus_masked-contig001-processed-gene-3.55-mRNA-1
contig001	maker	CDS	309639	313496	.	+	0	ID=MALK_00178.t1.c1;Parent=MALK_00178.t1
contig001	maker	mRNA	309639	313496	.	+	.	ID=MALK_00178.t1;Parent=MALK_00178
contig001	maker	exon	309639	313496	.	+	.	ID=MALK_00178.t1.e1;Parent=MALK_00178.t1
contig001	AUGUSTUS	gene	313849	315394	0.3	+	.	ID=MALK_00179;prediction_source=augustus:contig001.g501.t1
contig001	AUGUSTUS	CDS	313849	314224	0.91	+	0	ID=MALK_00179.t1.c1;Parent=MALK_00179.t1
contig001	AUGUSTUS	CDS	314328	315394	0.91	+	0	ID=MALK_00179.t1.c2;Parent=MALK_00179.t1
contig001	AUGUSTUS	mRNA	313849	315394	0.3	+	.	ID=MALK_00179.t1;Parent=MALK_00179
contig001	AUGUSTUS	exon	313849	314224	0.91	+	.	ID=MALK_00179.t1.e1;Parent=MALK_00179.t1
contig001	AUGUSTUS	exon	314328	315394	0.91	+	.	ID=MALK_00179.t1.e2;Parent=MALK_00179.t1
contig001	AUGUSTUS	gene	315561	317027	0.39	-	.	ID=MALK_00180;prediction_source=augustus:contig001.g502.t1
contig001	AUGUSTUS	CDS	315561	317027	0.39	-	0	ID=MALK_00180.t1.c1;Parent=MALK_00180.t1
contig001	AUGUSTUS	mRNA	315561	317027	0.39	-	.	ID=MALK_00180.t1;Parent=MALK_00180
contig001	AUGUSTUS	exon	315561	317027	0.39	-	.	ID=MALK_00180.t1.e1;Parent=MALK_00180.t1

contig001	AUGUSTUS	gene	317170	319018	0.99	-	.	ID=MALK_00181;prediction_source=braker_MRET:g838.t1
contig001	AUGUSTUS	CDS	318544	319018	1	-	0	ID=MALK_00181.t1.c2;Parent=MALK_00181.t1
contig001	AUGUSTUS	CDS	317170	318356	1	-	0	ID=MALK_00181.t1.c1;Parent=MALK_00181.t1
contig001	AUGUSTUS	mRNA	317170	319018	0.99	-	.	ID=MALK_00181.t1;Parent=MALK_00181
contig001	AUGUSTUS	exon	318544	319018	.	-	.	ID=MALK_00181.t1.e2;Parent=MALK_00181.t1
contig001	AUGUSTUS	exon	317170	318356	.	-	.	ID=MALK_00181.t1.e1;Parent=MALK_00181.t1
contig001	AUGUSTUS	gene	319195	320661	1	+	.	ID=MALK_00182;prediction_source=augustus:contig001.g505.t1
contig001	AUGUSTUS	CDS	319195	320661	1	+	0	ID=MALK_00182.t1.c1;Parent=MALK_00182.t1
contig001	AUGUSTUS	mRNA	319195	320661	1	+	.	ID=MALK_00182.t1;Parent=MALK_00182
contig001	AUGUSTUS	exon	319195	320661	1	+	.	ID=MALK_00182.t1.e1;Parent=MALK_00182.t1
contig001	maker	gene	321142	322443	.	+	.	ID=MALK_00183;prediction_source=maker_MRET:augustus_masked-contig001-processed-gene-3.58-mRNA-1
contig001	maker	CDS	321142	322443	.	+	0	ID=MALK_00183.t1.c1;Parent=MALK_00183.t1
contig001	maker	mRNA	321142	322443	.	+	.	ID=MALK_00183.t1;Parent=MALK_00183
contig001	maker	exon	321142	322443	.	+	.	ID=MALK_00183.t1.e1;Parent=MALK_00183.t1
contig001	AUGUSTUS	gene	322478	323485	0.95	-	.	ID=MALK_00184;prediction_source=braker_MRET:g841.t1
contig001	AUGUSTUS	CDS	322478	323485	0.95	-	0	ID=MALK_00184.t1.c1;Parent=MALK_00184.t1
contig001	AUGUSTUS	mRNA	322478	323485	0.95	-	.	ID=MALK_00184.t1;Parent=MALK_00184
contig001	AUGUSTUS	exon	322478	323485	.	-	.	ID=MALK_00184.t1.e1;Parent=MALK_00184.t1
contig001	AUGUSTUS	gene	323657	326087	0.08	-	.	ID=MALK_00185;prediction_source=braker_MRET:g842.t1
contig001	AUGUSTUS	CDS	325981	326087	0.75	-	0	ID=MALK_00185.t1.c4;Parent=MALK_00185.t1
contig001	AUGUSTUS	CDS	325209	325896	0.75	-	0	ID=MALK_00185.t1.c3;Parent=MALK_00185.t1
contig001	AUGUSTUS	CDS	325051	325174	0.75	-	0	ID=MALK_00185.t1.c2;Parent=MALK_00185.t1
contig001	AUGUSTUS	CDS	323657	324876	0.75	-	0	ID=MALK_00185.t1.c1;Parent=MALK_00185.t1
contig001	AUGUSTUS	mRNA	323657	326087	0.08	-	.	ID=MALK_00185.t1;Parent=MALK_00185
contig001	AUGUSTUS	exon	325981	326087	.	-	.	ID=MALK_00185.t1.e4;Parent=MALK_00185.t1
contig001	AUGUSTUS	exon	325209	325896	.	-	.	ID=MALK_00185.t1.e3;Parent=MALK_00185.t1
contig001	AUGUSTUS	exon	325051	325174	.	-	.	ID=MALK_00185.t1.e2;Parent=MALK_00185.t1
contig001	AUGUSTUS	exon	323657	324876	.	-	.	ID=MALK_00185.t1.e1;Parent=MALK_00185.t1
contig001	AUGUSTUS	gene	326489	327601	0.84	+	.	ID=MALK_00186;prediction_source=augustus:contig001.g508.t1
contig001	AUGUSTUS	CDS	326489	327601	0.84	+	0	ID=MALK_00186.t1.c1;Parent=MALK_00186.t1
contig001	AUGUSTUS	mRNA	326489	327601	0.84	+	.	ID=MALK_00186.t1;Parent=MALK_00186
contig001	AUGUSTUS	exon	326489	327601	0.84	+	.	ID=MALK_00186.t1.e1;Parent=MALK_00186.t1
contig001	AUGUSTUS	gene	327879	329090	0.57	+	.	ID=MALK_00187;prediction_source=augustus:contig001.g509.t1
contig001	AUGUSTUS	CDS	327879	329090	0.57	+	0	ID=MALK_00187.t1.c1;Parent=MALK_00187.t1
contig001	AUGUSTUS	mRNA	327879	329090	0.57	+	.	ID=MALK_00187.t1;Parent=MALK_00187
contig001	AUGUSTUS	exon	327879	329090	0.57	+	.	ID=MALK_00187.t1.e1;Parent=MALK_00187.t1
contig001	maker	gene	329210	330154	.	+	.	ID=MALK_00188;prediction_source=maker_MRET:augustus_masked-contig001-processed-gene-3.61-mRNA-1
contig001	maker	CDS	329210	330154	.	+	0	ID=MALK_00188.t1.c1;Parent=MALK_00188.t1
contig001	maker	mRNA	329210	330154	.	+	.	ID=MALK_00188.t1;Parent=MALK_00188
contig001	maker	exon	329210	330154	.	+	.	ID=MALK_00188.t1.e1;Parent=MALK_00188.t1
contig001	maker	gene	330209	333016	.	-	.	ID=MALK_00189;prediction_source=maker_MRET:augustus_masked-contig001-processed-gene-3.79-mRNA-1
contig001	maker	CDS	330209	333016	.	-	0	ID=MALK_00189.t1.c1;Parent=MALK_00189.t1
contig001	maker	mRNA	330209	333016	.	-	.	ID=MALK_00189.t1;Parent=MALK_00189

contig001	maker	exon	330209	333016	.	-	.	ID=MALK_00189.t1.e1;Parent=MALK_00189.t1
contig001	maker	gene	333619	335367	.	-	.	ID=MALK_00190;prediction_source=maker_MRET:augustus_masked-contig001-processed-gene-3.80-mRNA-1
contig001	maker	CDS	333619	335367	.	-	0	ID=MALK_00190.t1.c1;Parent=MALK_00190.t1
contig001	maker	mRNA	333619	335367	.	-	.	ID=MALK_00190.t1;Parent=MALK_00190
contig001	maker	exon	333619	335367	.	-	.	ID=MALK_00190.t1.e1;Parent=MALK_00190.t1
contig001	AUGUSTUS	gene	335678	337753	0.99	+	.	ID=MALK_00191;prediction_source=braker_MRET:g848.t1
contig001	AUGUSTUS	CDS	335678	335795	1	+	0	ID=MALK_00191.t1.c1;Parent=MALK_00191.t1
contig001	AUGUSTUS	CDS	335826	337753	1	+	0	ID=MALK_00191.t1.c2;Parent=MALK_00191.t1
contig001	AUGUSTUS	mRNA	335678	337753	0.99	+	.	ID=MALK_00191.t1;Parent=MALK_00191
contig001	AUGUSTUS	exon	335678	335795	.	+	.	ID=MALK_00191.t1.e1;Parent=MALK_00191.t1
contig001	AUGUSTUS	exon	335826	337753	.	+	.	ID=MALK_00191.t1.e2;Parent=MALK_00191.t1
contig001	maker	gene	337766	339364	.	-	.	ID=MALK_00192;prediction_source=maker_MRET:augustus_masked-contig001-processed-gene-3.81-mRNA-1
contig001	maker	CDS	337766	339364	.	-	0	ID=MALK_00192.t1.c1;Parent=MALK_00192.t1
contig001	maker	mRNA	337766	339364	.	-	.	ID=MALK_00192.t1;Parent=MALK_00192
contig001	maker	exon	337766	339364	.	-	.	ID=MALK_00192.t1.e1;Parent=MALK_00192.t1
contig001	maker	gene	339428	342691	.	-	.	ID=MALK_00193;prediction_source=maker_MRET:augustus_masked-contig001-processed-gene-3.82-mRNA-1
contig001	maker	CDS	339428	342691	.	-	0	ID=MALK_00193.t1.c1;Parent=MALK_00193.t1
contig001	maker	mRNA	339428	342691	.	-	.	ID=MALK_00193.t1;Parent=MALK_00193
contig001	maker	exon	339428	342691	.	-	.	ID=MALK_00193.t1.e1;Parent=MALK_00193.t1
contig001	AUGUSTUS	gene	342795	344249	0.96	-	.	ID=MALK_00194;prediction_source=braker_MRET:g851.t1
contig001	AUGUSTUS	CDS	342795	344249	0.96	-	0	ID=MALK_00194.t1.c1;Parent=MALK_00194.t1
contig001	AUGUSTUS	mRNA	342795	344249	0.96	-	.	ID=MALK_00194.t1;Parent=MALK_00194
contig001	AUGUSTUS	exon	342795	344249	.	-	.	ID=MALK_00194.t1.e1;Parent=MALK_00194.t1
contig001	AUGUSTUS	gene	344478	346017	0.59	+	.	ID=MALK_00195;prediction_source=braker_MRET:g852.t1
contig001	AUGUSTUS	CDS	344478	344572	0.98	+	0	ID=MALK_00195.t1.c1;Parent=MALK_00195.t1
contig001	AUGUSTUS	CDS	344602	344615	0.98	+	0	ID=MALK_00195.t1.c2;Parent=MALK_00195.t1
contig001	AUGUSTUS	CDS	344645	346017	0.98	+	0	ID=MALK_00195.t1.c3;Parent=MALK_00195.t1
contig001	AUGUSTUS	mRNA	344478	346017	0.59	+	.	ID=MALK_00195.t1;Parent=MALK_00195
contig001	AUGUSTUS	exon	344478	344572	.	+	.	ID=MALK_00195.t1.e1;Parent=MALK_00195.t1
contig001	AUGUSTUS	exon	344602	344615	.	+	.	ID=MALK_00195.t1.e2;Parent=MALK_00195.t1
contig001	AUGUSTUS	exon	344645	346017	.	+	.	ID=MALK_00195.t1.e3;Parent=MALK_00195.t1
contig001	AUGUSTUS	gene	346014	346646	0.92	-	.	ID=MALK_00196;prediction_source=augustus:contig001.g519.t1
contig001	AUGUSTUS	CDS	346014	346646	0.92	-	0	ID=MALK_00196.t1.c1;Parent=MALK_00196.t1
contig001	AUGUSTUS	mRNA	346014	346646	0.92	-	.	ID=MALK_00196.t1;Parent=MALK_00196
contig001	AUGUSTUS	exon	346014	346646	0.92	-	.	ID=MALK_00196.t1.e1;Parent=MALK_00196.t1
contig001	AUGUSTUS	gene	346778	349459	0.83	+	.	ID=MALK_00197;prediction_source=augustus:contig001.g520.t1
contig001	AUGUSTUS	CDS	346778	349459	0.83	+	0	ID=MALK_00197.t1.c1;Parent=MALK_00197.t1
contig001	AUGUSTUS	mRNA	346778	349459	0.83	+	.	ID=MALK_00197.t1;Parent=MALK_00197
contig001	AUGUSTUS	exon	346778	349459	0.83	+	.	ID=MALK_00197.t1.e1;Parent=MALK_00197.t1
contig001	AUGUSTUS	gene	349494	350669	1	-	.	ID=MALK_00198;prediction_source=braker_MRET:g855.t1
contig001	AUGUSTUS	CDS	349494	350669	1	-	0	ID=MALK_00198.t1.c1;Parent=MALK_00198.t1
contig001	AUGUSTUS	mRNA	349494	350669	1	-	.	ID=MALK_00198.t1;Parent=MALK_00198
contig001	AUGUSTUS	exon	349494	350669	.	-	.	ID=MALK_00198.t1.e1;Parent=MALK_00198.t1

contig001	AUGUSTUS	gene	350701	352683	1	-	.	ID=MALK_00199;prediction_source=braker_MRET:g856.t1
contig001	AUGUSTUS	CDS	350701	352683	1	-	0	ID=MALK_00199.t1.c1;Parent=MALK_00199.t1
contig001	AUGUSTUS	mRNA	350701	352683	1	-	.	ID=MALK_00199.t1;Parent=MALK_00199
contig001	AUGUSTUS	exon	350701	352683	.	-	.	ID=MALK_00199.t1.e1;Parent=MALK_00199.t1
contig001	AUGUSTUS	gene	352788	354821	1	-	.	ID=MALK_00200;prediction_source=augustus:contig001.g522.t1
contig001	AUGUSTUS	CDS	352788	354821	1	-	0	ID=MALK_00200.t1.c1;Parent=MALK_00200.t1
contig001	AUGUSTUS	mRNA	352788	354821	1	-	.	ID=MALK_00200.t1;Parent=MALK_00200
contig001	AUGUSTUS	exon	352788	354821	1	-	.	ID=MALK_00200.t1.e1;Parent=MALK_00200.t1
contig001	AUGUSTUS	gene	355043	356137	0.87	+	.	ID=MALK_00201;prediction_source=augustus:contig001.g523.t1
contig001	AUGUSTUS	CDS	355043	356137	0.87	+	0	ID=MALK_00201.t1.c1;Parent=MALK_00201.t1
contig001	AUGUSTUS	mRNA	355043	356137	0.87	+	.	ID=MALK_00201.t1;Parent=MALK_00201
contig001	AUGUSTUS	exon	355043	356137	0.87	+	.	ID=MALK_00201.t1.e1;Parent=MALK_00201.t1
contig001	maker	gene	356163	358016	.	-	.	ID=MALK_00202;prediction_source=maker_MRET:augustus_masked-contig001-processed-gene-3.87-mRNA-1
contig001	maker	CDS	356163	358016	.	-	0	ID=MALK_00202.t1.c1;Parent=MALK_00202.t1
contig001	maker	mRNA	356163	358016	.	-	.	ID=MALK_00202.t1;Parent=MALK_00202
contig001	maker	exon	356163	358016	.	-	.	ID=MALK_00202.t1.e1;Parent=MALK_00202.t1
contig001	AUGUSTUS	gene	358210	358779	0.67	+	.	ID=MALK_00203;prediction_source=braker_MRET:g860.t1
contig001	AUGUSTUS	CDS	358210	358779	0.67	+	0	ID=MALK_00203.t1.c1;Parent=MALK_00203.t1
contig001	AUGUSTUS	mRNA	358210	358779	0.67	+	.	ID=MALK_00203.t1;Parent=MALK_00203
contig001	AUGUSTUS	exon	358210	358779	.	+	.	ID=MALK_00203.t1.e1;Parent=MALK_00203.t1
contig001	AUGUSTUS	gene	359129	362251	0.76	+	.	ID=MALK_00204;prediction_source=braker_MRET:g861.t1
contig001	AUGUSTUS	CDS	359129	362251	0.76	+	0	ID=MALK_00204.t1.c1;Parent=MALK_00204.t1
contig001	AUGUSTUS	mRNA	359129	362251	0.76	+	.	ID=MALK_00204.t1;Parent=MALK_00204
contig001	AUGUSTUS	exon	359129	362251	.	+	.	ID=MALK_00204.t1.e1;Parent=MALK_00204.t1
contig001	maker	gene	362266	364539	.	-	.	ID=MALK_00205;prediction_source=maker_MRET:augustus_masked-contig001-processed-gene-3.88-mRNA-1
contig001	maker	CDS	362266	364539	.	-	0	ID=MALK_00205.t1.c1;Parent=MALK_00205.t1
contig001	maker	mRNA	362266	364539	.	-	.	ID=MALK_00205.t1;Parent=MALK_00205
contig001	maker	exon	362266	364539	.	-	.	ID=MALK_00205.t1.e1;Parent=MALK_00205.t1
contig001	maker	gene	364648	366123	.	-	.	ID=MALK_00206;prediction_source=maker_MRET:augustus_masked-contig001-processed-gene-3.89-mRNA-1
contig001	maker	CDS	364648	366123	.	-	0	ID=MALK_00206.t1.c1;Parent=MALK_00206.t1
contig001	maker	mRNA	364648	366123	.	-	.	ID=MALK_00206.t1;Parent=MALK_00206
contig001	maker	exon	364648	366123	.	-	.	ID=MALK_00206.t1.e1;Parent=MALK_00206.t1
contig001	AUGUSTUS	gene	366325	367058	0.3	-	.	ID=MALK_00207;prediction_source=braker_MRET:g864.t1
contig001	AUGUSTUS	CDS	366753	367058	0.74	-	0	ID=MALK_00207.t1.c3;Parent=MALK_00207.t1
contig001	AUGUSTUS	CDS	366508	366722	0.74	-	0	ID=MALK_00207.t1.c2;Parent=MALK_00207.t1
contig001	AUGUSTUS	CDS	366325	366472	0.74	-	0	ID=MALK_00207.t1.c1;Parent=MALK_00207.t1
contig001	AUGUSTUS	mRNA	366325	367058	0.3	-	.	ID=MALK_00207.t1;Parent=MALK_00207
contig001	AUGUSTUS	exon	366753	367058	.	-	.	ID=MALK_00207.t1.e3;Parent=MALK_00207.t1
contig001	AUGUSTUS	exon	366508	366722	.	-	.	ID=MALK_00207.t1.e2;Parent=MALK_00207.t1
contig001	AUGUSTUS	exon	366325	366472	.	-	.	ID=MALK_00207.t1.e1;Parent=MALK_00207.t1
contig001	maker	gene	367128	368705	.	-	.	ID=MALK_00208;prediction_source=maker_MRET:augustus_masked-contig001-processed-gene-3.91-mRNA-1
contig001	maker	CDS	367128	368705	.	-	0	ID=MALK_00208.t1.c1;Parent=MALK_00208.t1
contig001	maker	mRNA	367128	368705	.	-	.	ID=MALK_00208.t1;Parent=MALK_00208

contig001	maker	exon	367128	368705	.	-	.	ID=MALK_00208.t1.e1;Parent=MALK_00208.t1
contig001	maker	gene	369225	371801	.	+	.	ID=MALK_00209;prediction_source=maker_MRET:augustus_masked-contig001-processed-gene-3.67-mRNA-1
contig001	maker	CDS	369225	371801	.	+	0	ID=MALK_00209.t1.c1;Parent=MALK_00209.t1
contig001	maker	mRNA	369225	371801	.	+	.	ID=MALK_00209.t1;Parent=MALK_00209
contig001	maker	exon	369225	371801	.	+	.	ID=MALK_00209.t1.e1;Parent=MALK_00209.t1
contig001	AUGUSTUS	gene	371940	373232	0.38	+	.	ID=MALK_00210;prediction_source=augustus:contig001.g532.t1
contig001	AUGUSTUS	CDS	371940	373232	0.38	+	0	ID=MALK_00210.t1.c1;Parent=MALK_00210.t1
contig001	AUGUSTUS	mRNA	371940	373232	0.38	+	.	ID=MALK_00210.t1;Parent=MALK_00210
contig001	AUGUSTUS	exon	371940	373232	0.38	+	.	ID=MALK_00210.t1.e1;Parent=MALK_00210.t1
contig001	AUGUSTUS	gene	373241	375589	0.99	-	.	ID=MALK_00211;prediction_source=augustus:contig001.g533.t1
contig001	AUGUSTUS	CDS	373241	375589	0.99	-	0	ID=MALK_00211.t1.c1;Parent=MALK_00211.t1
contig001	AUGUSTUS	mRNA	373241	375589	0.99	-	.	ID=MALK_00211.t1;Parent=MALK_00211
contig001	AUGUSTUS	exon	373241	375589	0.99	-	.	ID=MALK_00211.t1.e1;Parent=MALK_00211.t1
contig001	AUGUSTUS	gene	375468	378023	0.99	+	.	ID=MALK_00212;prediction_source=augustus:contig001.g534.t1
contig001	AUGUSTUS	CDS	375468	378023	0.99	+	0	ID=MALK_00212.t1.c1;Parent=MALK_00212.t1
contig001	AUGUSTUS	mRNA	375468	378023	0.99	+	.	ID=MALK_00212.t1;Parent=MALK_00212
contig001	AUGUSTUS	exon	375468	378023	0.99	+	.	ID=MALK_00212.t1.e1;Parent=MALK_00212.t1
contig001	AUGUSTUS	gene	378160	379322	0.64	-	.	ID=MALK_00213;prediction_source=braker_MRET:g870.t1
contig001	AUGUSTUS	CDS	378264	379322	0.64	-	0	ID=MALK_00213.t1.c2;Parent=MALK_00213.t1
contig001	AUGUSTUS	CDS	378160	378222	0.64	-	0	ID=MALK_00213.t1.c1;Parent=MALK_00213.t1
contig001	AUGUSTUS	mRNA	378160	379322	0.64	-	.	ID=MALK_00213.t1;Parent=MALK_00213
contig001	AUGUSTUS	exon	378264	379322	.	-	.	ID=MALK_00213.t1.e2;Parent=MALK_00213.t1
contig001	AUGUSTUS	exon	378160	378222	.	-	.	ID=MALK_00213.t1.e1;Parent=MALK_00213.t1
contig001	maker	gene	379516	380481	.	-	.	ID=MALK_00214;prediction_source=maker_MRET:augustus_masked-contig001-processed-gene-3.93-mRNA-1
contig001	maker	CDS	380370	380481	.	-	0	ID=MALK_00214.t1.c1;Parent=MALK_00214.t1
contig001	maker	CDS	379516	380306	.	-	0	ID=MALK_00214.t1.c2;Parent=MALK_00214.t1
contig001	maker	mRNA	379516	380481	.	-	.	ID=MALK_00214.t1;Parent=MALK_00214
contig001	maker	exon	380370	380481	.	-	.	ID=MALK_00214.t1.e1;Parent=MALK_00214.t1
contig001	maker	exon	379516	380306	.	-	.	ID=MALK_00214.t1.e2;Parent=MALK_00214.t1
contig001	AUGUSTUS	gene	380739	382007	0.98	-	.	ID=MALK_00215;prediction_source=braker_MRET:g872.t1
contig001	AUGUSTUS	CDS	380739	382007	0.98	-	0	ID=MALK_00215.t1.c1;Parent=MALK_00215.t1
contig001	AUGUSTUS	mRNA	380739	382007	0.98	-	.	ID=MALK_00215.t1;Parent=MALK_00215
contig001	AUGUSTUS	exon	380739	382007	.	-	.	ID=MALK_00215.t1.e1;Parent=MALK_00215.t1
contig001	maker	gene	382045	382779	.	-	.	ID=MALK_00216;prediction_source=maker_MRET:augustus_masked-contig001-processed-gene-3.95-mRNA-1
contig001	maker	CDS	382045	382779	.	-	0	ID=MALK_00216.t1.c1;Parent=MALK_00216.t1
contig001	maker	mRNA	382045	382779	.	-	.	ID=MALK_00216.t1;Parent=MALK_00216
contig001	maker	exon	382045	382779	.	-	.	ID=MALK_00216.t1.e1;Parent=MALK_00216.t1
contig001	maker	gene	383327	387856	.	+	.	ID=MALK_00217;prediction_source=maker_MRET:augustus_masked-contig001-processed-gene-3.70-mRNA-1
contig001	maker	CDS	383327	387856	.	+	0	ID=MALK_00217.t1.c1;Parent=MALK_00217.t1
contig001	maker	mRNA	383327	387856	.	+	.	ID=MALK_00217.t1;Parent=MALK_00217
contig001	maker	exon	383327	387856	.	+	.	ID=MALK_00217.t1.e1;Parent=MALK_00217.t1
contig001	maker	gene	387874	388500	.	-	.	ID=MALK_00218;prediction_source=maker_MRET:augustus_masked-contig001-processed-gene-3.96-mRNA-1
contig001	maker	CDS	387874	388500	.	-	0	ID=MALK_00218.t1.c1;Parent=MALK_00218.t1

contig001	maker	mRNA	387874	388500	.	-	.	ID=MALK_00218.t1;Parent=MALK_00218
contig001	maker	exon	387874	388500	.	-	.	ID=MALK_00218.t1.e1;Parent=MALK_00218.t1
contig001	maker	gene	388685	389644	.	+	.	ID=MALK_00219;prediction_source=maker_MRET:augustus_masked-contig001-processed-gene-3.71-mRNA-1
contig001	maker	CDS	388685	389644	.	+	0	ID=MALK_00219.t1.c1;Parent=MALK_00219.t1
contig001	maker	mRNA	388685	389644	.	+	.	ID=MALK_00219.t1;Parent=MALK_00219
contig001	maker	exon	388685	389644	.	+	.	ID=MALK_00219.t1.e1;Parent=MALK_00219.t1
contig001	maker	gene	389664	390626	.	-	.	ID=MALK_00220;prediction_source=maker_MRET:augustus_masked-contig001-processed-gene-3.97-mRNA-1
contig001	maker	CDS	389664	390626	.	-	0	ID=MALK_00220.t1.c1;Parent=MALK_00220.t1
contig001	maker	mRNA	389664	390626	.	-	.	ID=MALK_00220.t1;Parent=MALK_00220
contig001	maker	exon	389664	390626	.	-	.	ID=MALK_00220.t1.e1;Parent=MALK_00220.t1
contig001	AUGUSTUS	gene	391529	392176	0.96	+	.	ID=MALK_00221;prediction_source=braker_MRET:g878.t1
contig001	AUGUSTUS	CDS	391529	392176	0.96	+	0	ID=MALK_00221.t1.c1;Parent=MALK_00221.t1
contig001	AUGUSTUS	mRNA	391529	392176	0.96	+	.	ID=MALK_00221.t1;Parent=MALK_00221
contig001	AUGUSTUS	exon	391529	392176	.	+	.	ID=MALK_00221.t1.e1;Parent=MALK_00221.t1
contig001	maker	gene	393495	394502	.	+	.	ID=MALK_00222;prediction_source=maker_MRET:augustus_masked-contig001-processed-gene-3.72-mRNA-1
contig001	maker	CDS	393495	394502	.	+	0	ID=MALK_00222.t1.c1;Parent=MALK_00222.t1
contig001	maker	mRNA	393495	394502	.	+	.	ID=MALK_00222.t1;Parent=MALK_00222
contig001	maker	exon	393495	394502	.	+	.	ID=MALK_00222.t1.e1;Parent=MALK_00222.t1
contig001	maker	gene	394566	396689	.	-	.	ID=MALK_00223;prediction_source=maker_MRET:augustus_masked-contig001-processed-gene-4.1-mRNA-1
contig001	maker	CDS	394566	396689	.	-	0	ID=MALK_00223.t1.c1;Parent=MALK_00223.t1
contig001	maker	mRNA	394566	396689	.	-	.	ID=MALK_00223.t1;Parent=MALK_00223
contig001	maker	exon	394566	396689	.	-	.	ID=MALK_00223.t1.e1;Parent=MALK_00223.t1
contig001	maker	gene	396807	397397	.	+	.	ID=MALK_00224;prediction_source=maker_MRET:augustus_masked-contig001-processed-gene-4.0-mRNA-1
contig001	maker	CDS	396807	397397	.	+	0	ID=MALK_00224.t1.c1;Parent=MALK_00224.t1
contig001	maker	mRNA	396807	397397	.	+	.	ID=MALK_00224.t1;Parent=MALK_00224
contig001	maker	exon	396807	397397	.	+	.	ID=MALK_00224.t1.e1;Parent=MALK_00224.t1
contig001	AUGUSTUS	gene	397443	400490	0.68	+	.	ID=MALK_00225;prediction_source=braker_MRET:g882.t1
contig001	AUGUSTUS	CDS	397443	399374	0.75	+	0	ID=MALK_00225.t1.c1;Parent=MALK_00225.t1
contig001	AUGUSTUS	CDS	399417	400490	0.75	+	0	ID=MALK_00225.t1.c2;Parent=MALK_00225.t1
contig001	AUGUSTUS	mRNA	397443	400490	0.68	+	.	ID=MALK_00225.t1;Parent=MALK_00225
contig001	AUGUSTUS	exon	397443	399374	.	+	.	ID=MALK_00225.t1.e1;Parent=MALK_00225.t1
contig001	AUGUSTUS	exon	399417	400490	.	+	.	ID=MALK_00225.t1.e2;Parent=MALK_00225.t1
contig001	AUGUSTUS	gene	400612	401104	0.44	-	.	ID=MALK_00226;prediction_source=braker_MRET:g883.t1
contig001	AUGUSTUS	CDS	400661	401104	0.44	-	0	ID=MALK_00226.t1.c2;Parent=MALK_00226.t1
contig001	AUGUSTUS	CDS	400612	400632	0.44	-	0	ID=MALK_00226.t1.c1;Parent=MALK_00226.t1
contig001	AUGUSTUS	mRNA	400612	401104	0.44	-	.	ID=MALK_00226.t1;Parent=MALK_00226
contig001	AUGUSTUS	exon	400661	401104	.	-	.	ID=MALK_00226.t1.e2;Parent=MALK_00226.t1
contig001	AUGUSTUS	exon	400612	400632	.	-	.	ID=MALK_00226.t1.e1;Parent=MALK_00226.t1
contig001	AUGUSTUS	gene	401175	402718	0.45	+	.	ID=MALK_00227;prediction_source=braker_MRET:g884.t1
contig001	AUGUSTUS	CDS	401175	402208	0.66	+	0	ID=MALK_00227.t1.c1;Parent=MALK_00227.t1
contig001	AUGUSTUS	CDS	402241	402284	0.66	+	0	ID=MALK_00227.t1.c2;Parent=MALK_00227.t1
contig001	AUGUSTUS	CDS	402459	402718	0.66	+	0	ID=MALK_00227.t1.c3;Parent=MALK_00227.t1
contig001	AUGUSTUS	mRNA	401175	402718	0.45	+	.	ID=MALK_00227.t1;Parent=MALK_00227

contig001	AUGUSTUS	exon	401175	402208	.	+	.	ID=MALK_00227.t1.e1;Parent=MALK_00227.t1
contig001	AUGUSTUS	exon	402241	402284	.	+	.	ID=MALK_00227.t1.e2;Parent=MALK_00227.t1
contig001	AUGUSTUS	exon	402459	402718	.	+	.	ID=MALK_00227.t1.e3;Parent=MALK_00227.t1
contig001	AUGUSTUS	gene	402958	404969	0.58	+	.	ID=MALK_00228;prediction_source=braker_MRET:g885.t1
contig001	AUGUSTUS	CDS	402958	404088	0.61	+	0	ID=MALK_00228.t1.c1;Parent=MALK_00228.t1
contig001	AUGUSTUS	CDS	404235	404969	0.61	+	0	ID=MALK_00228.t1.c2;Parent=MALK_00228.t1
contig001	AUGUSTUS	mRNA	402958	404969	0.58	+	.	ID=MALK_00228.t1;Parent=MALK_00228
contig001	AUGUSTUS	exon	402958	404088	.	+	.	ID=MALK_00228.t1.e1;Parent=MALK_00228.t1
contig001	AUGUSTUS	exon	404235	404969	.	+	.	ID=MALK_00228.t1.e2;Parent=MALK_00228.t1
contig001	AUGUSTUS	gene	405228	407981	0.75	+	.	ID=MALK_00229;prediction_source=augustus:contig001.g551.t1
contig001	AUGUSTUS	CDS	405228	407981	0.75	+	0	ID=MALK_00229.t1.c1;Parent=MALK_00229.t1
contig001	AUGUSTUS	mRNA	405228	407981	0.75	+	.	ID=MALK_00229.t1;Parent=MALK_00229
contig001	AUGUSTUS	exon	405228	407981	0.75	+	.	ID=MALK_00229.t1.e1;Parent=MALK_00229.t1
contig001	maker	gene	407983	409617	.	-	.	ID=MALK_00230;prediction_source=maker_MRET:augustus_masked-contig001-processed-gene-4.28-mRNA-1
contig001	maker	CDS	407983	409617	.	-	0	ID=MALK_00230.t1.c1;Parent=MALK_00230.t1
contig001	maker	mRNA	407983	409617	.	-	.	ID=MALK_00230.t1;Parent=MALK_00230
contig001	maker	exon	407983	409617	.	-	.	ID=MALK_00230.t1.e1;Parent=MALK_00230.t1
contig001	AUGUSTUS	gene	409881	410710	0.57	+	.	ID=MALK_00231;prediction_source=braker_MRET:g888.t1
contig001	AUGUSTUS	CDS	409881	409907	0.57	+	0	ID=MALK_00231.t1.c1;Parent=MALK_00231.t1
contig001	AUGUSTUS	CDS	409937	410710	0.57	+	0	ID=MALK_00231.t1.c2;Parent=MALK_00231.t1
contig001	AUGUSTUS	mRNA	409881	410710	0.57	+	.	ID=MALK_00231.t1;Parent=MALK_00231
contig001	AUGUSTUS	exon	409881	409907	.	+	.	ID=MALK_00231.t1.e1;Parent=MALK_00231.t1
contig001	AUGUSTUS	exon	409937	410710	.	+	.	ID=MALK_00231.t1.e2;Parent=MALK_00231.t1
contig001	AUGUSTUS	gene	410811	412139	0.54	+	.	ID=MALK_00232;prediction_source=braker_MRET:g889.t1
contig001	AUGUSTUS	CDS	410811	410867	0.61	+	0	ID=MALK_00232.t1.c1;Parent=MALK_00232.t1
contig001	AUGUSTUS	CDS	410901	412139	0.61	+	0	ID=MALK_00232.t1.c2;Parent=MALK_00232.t1
contig001	AUGUSTUS	mRNA	410811	412139	0.54	+	.	ID=MALK_00232.t1;Parent=MALK_00232
contig001	AUGUSTUS	exon	410811	410867	.	+	.	ID=MALK_00232.t1.e1;Parent=MALK_00232.t1
contig001	AUGUSTUS	exon	410901	412139	.	+	.	ID=MALK_00232.t1.e2;Parent=MALK_00232.t1
contig001	maker	gene	412204	415053	.	+	.	ID=MALK_00233;prediction_source=maker_MRET:augustus_masked-contig001-processed-gene-4.6-mRNA-1
contig001	maker	CDS	412204	415053	.	+	0	ID=MALK_00233.t1.c1;Parent=MALK_00233.t1
contig001	maker	mRNA	412204	415053	.	+	.	ID=MALK_00233.t1;Parent=MALK_00233
contig001	maker	exon	412204	415053	.	+	.	ID=MALK_00233.t1.e1;Parent=MALK_00233.t1
contig001	AUGUSTUS	gene	415102	416534	0.74	-	.	ID=MALK_00234;prediction_source=braker_MRET:g891.t1
contig001	AUGUSTUS	CDS	415170	416534	0.74	-	0	ID=MALK_00234.t1.c2;Parent=MALK_00234.t1
contig001	AUGUSTUS	CDS	415102	415140	0.74	-	0	ID=MALK_00234.t1.c1;Parent=MALK_00234.t1
contig001	AUGUSTUS	mRNA	415102	416534	0.74	-	.	ID=MALK_00234.t1;Parent=MALK_00234
contig001	AUGUSTUS	exon	415170	416534	.	-	.	ID=MALK_00234.t1.e2;Parent=MALK_00234.t1
contig001	AUGUSTUS	exon	415102	415140	.	-	.	ID=MALK_00234.t1.e1;Parent=MALK_00234.t1
contig001	maker	gene	416637	417506	.	+	.	ID=MALK_00235;prediction_source=maker_MRET:augustus_masked-contig001-processed-gene-4.7-mRNA-1
contig001	maker	CDS	416637	417506	.	+	0	ID=MALK_00235.t1.c1;Parent=MALK_00235.t1
contig001	maker	mRNA	416637	417506	.	+	.	ID=MALK_00235.t1;Parent=MALK_00235
contig001	maker	exon	416637	417506	.	+	.	ID=MALK_00235.t1.e1;Parent=MALK_00235.t1

contig001	AUGUSTUS	gene	417539	418093	0.98	-	.	ID=MALK_00236;prediction_source=braker_MRET:g893.t1
contig001	AUGUSTUS	CDS	417539	418093	0.98	-	0	ID=MALK_00236.t1.c1;Parent=MALK_00236.t1
contig001	AUGUSTUS	mRNA	417539	418093	0.98	-	.	ID=MALK_00236.t1;Parent=MALK_00236
contig001	AUGUSTUS	exon	417539	418093	.	-	.	ID=MALK_00236.t1.e1;Parent=MALK_00236.t1
contig001	AUGUSTUS	gene	418478	423535	1	+	.	ID=MALK_00237;prediction_source=augustus:contig001.g558.t1
contig001	AUGUSTUS	CDS	418478	423535	1	+	0	ID=MALK_00237.t1.c1;Parent=MALK_00237.t1
contig001	AUGUSTUS	mRNA	418478	423535	1	+	.	ID=MALK_00237.t1;Parent=MALK_00237
contig001	AUGUSTUS	exon	418478	423535	1	+	.	ID=MALK_00237.t1.e1;Parent=MALK_00237.t1
contig001	AUGUSTUS	gene	423507	424613	0.93	-	.	ID=MALK_00238;prediction_source=augustus:contig001.g559.t1
contig001	AUGUSTUS	CDS	423507	424613	0.93	-	0	ID=MALK_00238.t1.c1;Parent=MALK_00238.t1
contig001	AUGUSTUS	mRNA	423507	424613	0.93	-	.	ID=MALK_00238.t1;Parent=MALK_00238
contig001	AUGUSTUS	exon	423507	424613	0.93	-	.	ID=MALK_00238.t1.e1;Parent=MALK_00238.t1
contig001	maker	gene	424747	426837	.	+	.	ID=MALK_00239;prediction_source=maker_MRET:augustus_masked-contig001-processed-gene-4.9-mRNA-1
contig001	maker	CDS	424747	426837	.	+	0	ID=MALK_00239.t1.c1;Parent=MALK_00239.t1
contig001	maker	mRNA	424747	426837	.	+	.	ID=MALK_00239.t1;Parent=MALK_00239
contig001	maker	exon	424747	426837	.	+	.	ID=MALK_00239.t1.e1;Parent=MALK_00239.t1
contig001	AUGUSTUS	gene	427010	428120	0.9	+	.	ID=MALK_00240;prediction_source=braker_MRET:g897.t1
contig001	AUGUSTUS	CDS	427010	427138	0.93	+	0	ID=MALK_00240.t1.c1;Parent=MALK_00240.t1
contig001	AUGUSTUS	CDS	427170	428120	0.93	+	0	ID=MALK_00240.t1.c2;Parent=MALK_00240.t1
contig001	AUGUSTUS	mRNA	427010	428120	0.9	+	.	ID=MALK_00240.t1;Parent=MALK_00240
contig001	AUGUSTUS	exon	427010	427138	.	+	.	ID=MALK_00240.t1.e1;Parent=MALK_00240.t1
contig001	AUGUSTUS	exon	427170	428120	.	+	.	ID=MALK_00240.t1.e2;Parent=MALK_00240.t1
contig001	AUGUSTUS	gene	428176	429012	0.58	-	.	ID=MALK_00241;prediction_source=braker_MRET:g898.t1
contig001	AUGUSTUS	CDS	428940	429012	0.61	-	0	ID=MALK_00241.t1.c3;Parent=MALK_00241.t1
contig001	AUGUSTUS	CDS	428728	428902	0.61	-	0	ID=MALK_00241.t1.c2;Parent=MALK_00241.t1
contig001	AUGUSTUS	CDS	428176	428695	0.61	-	0	ID=MALK_00241.t1.c1;Parent=MALK_00241.t1
contig001	AUGUSTUS	mRNA	428176	429012	0.58	-	.	ID=MALK_00241.t1;Parent=MALK_00241
contig001	AUGUSTUS	exon	428940	429012	.	-	.	ID=MALK_00241.t1.e3;Parent=MALK_00241.t1
contig001	AUGUSTUS	exon	428728	428902	.	-	.	ID=MALK_00241.t1.e2;Parent=MALK_00241.t1
contig001	AUGUSTUS	exon	428176	428695	.	-	.	ID=MALK_00241.t1.e1;Parent=MALK_00241.t1
contig001	AUGUSTUS	gene	429196	431373	0.96	+	.	ID=MALK_00242;prediction_source=augustus:contig001.g563.t1
contig001	AUGUSTUS	CDS	429196	431373	0.96	+	0	ID=MALK_00242.t1.c1;Parent=MALK_00242.t1
contig001	AUGUSTUS	mRNA	429196	431373	0.96	+	.	ID=MALK_00242.t1;Parent=MALK_00242
contig001	AUGUSTUS	exon	429196	431373	0.96	+	.	ID=MALK_00242.t1.e1;Parent=MALK_00242.t1
contig001	AUGUSTUS	gene	431370	433070	0.53	-	.	ID=MALK_00243;prediction_source=braker_MRET:g900.t1
contig001	AUGUSTUS	CDS	431370	433070	0.53	-	0	ID=MALK_00243.t1.c1;Parent=MALK_00243.t1
contig001	AUGUSTUS	mRNA	431370	433070	0.53	-	.	ID=MALK_00243.t1;Parent=MALK_00243
contig001	AUGUSTUS	exon	431370	433070	.	-	.	ID=MALK_00243.t1.e1;Parent=MALK_00243.t1
contig001	AUGUSTUS	gene	433313	435749	0.7	+	.	ID=MALK_00244;prediction_source=braker_MRET:g901.t1
contig001	AUGUSTUS	CDS	433313	435582	0.76	+	0	ID=MALK_00244.t1.c1;Parent=MALK_00244.t1
contig001	AUGUSTUS	CDS	435614	435749	0.76	+	0	ID=MALK_00244.t1.c2;Parent=MALK_00244.t1
contig001	AUGUSTUS	mRNA	433313	435749	0.7	+	.	ID=MALK_00244.t1;Parent=MALK_00244
contig001	AUGUSTUS	exon	433313	435582	.	+	.	ID=MALK_00244.t1.e1;Parent=MALK_00244.t1

contig001	AUGUSTUS	exon	435614	435749	.	+	.	ID=MALK_00244.t1.e2;Parent=MALK_00244.t1
contig001	maker	gene	436146	438026	.	+	.	ID=MALK_00245;prediction_source=maker_MRET:augustus_masked-contig001-processed-gene-4.13-mRNA-1
contig001	maker	CDS	436146	438026	.	+	0	ID=MALK_00245.t1.c1;Parent=MALK_00245.t1
contig001	maker	mRNA	436146	438026	.	+	.	ID=MALK_00245.t1;Parent=MALK_00245
contig001	maker	exon	436146	438026	.	+	.	ID=MALK_00245.t1.e1;Parent=MALK_00245.t1
contig001	AUGUSTUS	gene	438180	439283	0.82	+	.	ID=MALK_00246;prediction_source=augustus:contig001.g567.t1
contig001	AUGUSTUS	CDS	438180	438355	0.95	+	0	ID=MALK_00246.t1.c1;Parent=MALK_00246.t1
contig001	AUGUSTUS	CDS	438440	439283	0.95	+	0	ID=MALK_00246.t1.c2;Parent=MALK_00246.t1
contig001	AUGUSTUS	mRNA	438180	439283	0.82	+	.	ID=MALK_00246.t1;Parent=MALK_00246
contig001	AUGUSTUS	exon	438180	438355	0.95	+	.	ID=MALK_00246.t1.e1;Parent=MALK_00246.t1
contig001	AUGUSTUS	exon	438440	439283	0.95	+	.	ID=MALK_00246.t1.e2;Parent=MALK_00246.t1
contig001	maker	gene	439307	440659	.	-	.	ID=MALK_00247;prediction_source=maker_MRET:augustus_masked-contig001-processed-gene-4.33-mRNA-1
contig001	maker	CDS	439307	440659	.	-	0	ID=MALK_00247.t1.c1;Parent=MALK_00247.t1
contig001	maker	mRNA	439307	440659	.	-	.	ID=MALK_00247.t1;Parent=MALK_00247
contig001	maker	exon	439307	440659	.	-	.	ID=MALK_00247.t1.e1;Parent=MALK_00247.t1
contig001	maker	gene	440901	442991	.	+	.	ID=MALK_00248;prediction_source=maker_MRET:augustus_masked-contig001-processed-gene-4.15-mRNA-1
contig001	maker	CDS	440901	442991	.	+	0	ID=MALK_00248.t1.c1;Parent=MALK_00248.t1
contig001	maker	mRNA	440901	442991	.	+	.	ID=MALK_00248.t1;Parent=MALK_00248
contig001	maker	exon	440901	442991	.	+	.	ID=MALK_00248.t1.e1;Parent=MALK_00248.t1
contig001	maker	gene	442993	445353	.	-	.	ID=MALK_00249;prediction_source=maker_MRET:augustus_masked-contig001-processed-gene-4.34-mRNA-1
contig001	maker	CDS	442993	445353	.	-	0	ID=MALK_00249.t1.c1;Parent=MALK_00249.t1
contig001	maker	mRNA	442993	445353	.	-	.	ID=MALK_00249.t1;Parent=MALK_00249
contig001	maker	exon	442993	445353	.	-	.	ID=MALK_00249.t1.e1;Parent=MALK_00249.t1
contig001	AUGUSTUS	gene	445423	446676	0.34	+	.	ID=MALK_00250;prediction_source=augustus:contig001.g571.t1
contig001	AUGUSTUS	CDS	445423	446676	0.34	+	0	ID=MALK_00250.t1.c1;Parent=MALK_00250.t1
contig001	AUGUSTUS	mRNA	445423	446676	0.34	+	.	ID=MALK_00250.t1;Parent=MALK_00250
contig001	AUGUSTUS	exon	445423	446676	0.34	+	.	ID=MALK_00250.t1.e1;Parent=MALK_00250.t1
contig001	maker	gene	446681	447136	.	-	.	ID=MALK_00251;prediction_source=maker_MRET:augustus_masked-contig001-processed-gene-4.35-mRNA-1
contig001	maker	CDS	446681	447136	.	-	0	ID=MALK_00251.t1.c1;Parent=MALK_00251.t1
contig001	maker	mRNA	446681	447136	.	-	.	ID=MALK_00251.t1;Parent=MALK_00251
contig001	maker	exon	446681	447136	.	-	.	ID=MALK_00251.t1.e1;Parent=MALK_00251.t1
contig001	AUGUSTUS	gene	447165	448126	0.66	-	.	ID=MALK_00252;prediction_source=braker_MRET:g909.t1
contig001	AUGUSTUS	CDS	448056	448126	0.85	-	0	ID=MALK_00252.t1.c2;Parent=MALK_00252.t1
contig001	AUGUSTUS	CDS	447165	448026	0.85	-	0	ID=MALK_00252.t1.c1;Parent=MALK_00252.t1
contig001	AUGUSTUS	mRNA	447165	448126	0.66	-	.	ID=MALK_00252.t1;Parent=MALK_00252
contig001	AUGUSTUS	exon	448056	448126	.	-	.	ID=MALK_00252.t1.e2;Parent=MALK_00252.t1
contig001	AUGUSTUS	exon	447165	448026	.	-	.	ID=MALK_00252.t1.e1;Parent=MALK_00252.t1
contig001	AUGUSTUS	gene	448424	450544	1	+	.	ID=MALK_00253;prediction_source=braker_MRET:g910.t1
contig001	AUGUSTUS	CDS	448424	450544	1	+	0	ID=MALK_00253.t1.c1;Parent=MALK_00253.t1
contig001	AUGUSTUS	mRNA	448424	450544	1	+	.	ID=MALK_00253.t1;Parent=MALK_00253
contig001	AUGUSTUS	exon	448424	450544	.	+	.	ID=MALK_00253.t1.e1;Parent=MALK_00253.t1
contig001	AUGUSTUS	gene	450864	452471	0.13	+	.	ID=MALK_00254;prediction_source=braker_MRET:g911.t1
contig001	AUGUSTUS	CDS	450864	450910	0.79	+	0	ID=MALK_00254.t1.c1;Parent=MALK_00254.t1

contig001	AUGUSTUS	CDS	450947	451083	0.79	+	0	ID=MALK_00254.t1.c2;Parent=MALK_00254.t1
contig001	AUGUSTUS	CDS	451116	451362	0.79	+	0	ID=MALK_00254.t1.c3;Parent=MALK_00254.t1
contig001	AUGUSTUS	CDS	451394	451544	0.79	+	0	ID=MALK_00254.t1.c4;Parent=MALK_00254.t1
contig001	AUGUSTUS	CDS	451686	451794	0.79	+	0	ID=MALK_00254.t1.c5;Parent=MALK_00254.t1
contig001	AUGUSTUS	CDS	451835	452428	0.79	+	0	ID=MALK_00254.t1.c6;Parent=MALK_00254.t1
contig001	AUGUSTUS	CDS	452461	452471	0.79	+	0	ID=MALK_00254.t1.c7;Parent=MALK_00254.t1
contig001	AUGUSTUS	mRNA	450864	452471	0.13	+	.	ID=MALK_00254.t1;Parent=MALK_00254
contig001	AUGUSTUS	exon	450864	450910	.	+	.	ID=MALK_00254.t1.e1;Parent=MALK_00254.t1
contig001	AUGUSTUS	exon	450947	451083	.	+	.	ID=MALK_00254.t1.e2;Parent=MALK_00254.t1
contig001	AUGUSTUS	exon	451116	451362	.	+	.	ID=MALK_00254.t1.e3;Parent=MALK_00254.t1
contig001	AUGUSTUS	exon	451394	451544	.	+	.	ID=MALK_00254.t1.e4;Parent=MALK_00254.t1
contig001	AUGUSTUS	exon	451686	451794	.	+	.	ID=MALK_00254.t1.e5;Parent=MALK_00254.t1
contig001	AUGUSTUS	exon	451835	452428	.	+	.	ID=MALK_00254.t1.e6;Parent=MALK_00254.t1
contig001	AUGUSTUS	exon	452461	452471	.	+	.	ID=MALK_00254.t1.e7;Parent=MALK_00254.t1
contig001	AUGUSTUS	gene	452476	453975	0.92	-	.	ID=MALK_00255;prediction_source=augustus:contig001.g577.t1
contig001	AUGUSTUS	CDS	452476	453975	0.92	-	0	ID=MALK_00255.t1.c1;Parent=MALK_00255.t1
contig001	AUGUSTUS	mRNA	452476	453975	0.92	-	.	ID=MALK_00255.t1;Parent=MALK_00255
contig001	AUGUSTUS	exon	452476	453975	0.92	-	.	ID=MALK_00255.t1.e1;Parent=MALK_00255.t1
contig001	AUGUSTUS	gene	454116	454957	0.15	-	.	ID=MALK_00256;prediction_source=braker_MRET:g913.t1
contig001	AUGUSTUS	CDS	454491	454957	0.15	-	0	ID=MALK_00256.t1.c2;Parent=MALK_00256.t1
contig001	AUGUSTUS	CDS	454116	454446	0.15	-	0	ID=MALK_00256.t1.c1;Parent=MALK_00256.t1
contig001	AUGUSTUS	mRNA	454116	454957	0.15	-	.	ID=MALK_00256.t1;Parent=MALK_00256
contig001	AUGUSTUS	exon	454491	454957	.	-	.	ID=MALK_00256.t1.e2;Parent=MALK_00256.t1
contig001	AUGUSTUS	exon	454116	454446	.	-	.	ID=MALK_00256.t1.e1;Parent=MALK_00256.t1
contig001	maker	gene	455264	457204	.	-	.	ID=MALK_00257;prediction_source=maker_MRET:augustus_masked-contig001-processed-gene-4.38-mRNA-1
contig001	maker	CDS	455264	457204	.	-	0	ID=MALK_00257.t1.c1;Parent=MALK_00257.t1
contig001	maker	mRNA	455264	457204	.	-	.	ID=MALK_00257.t1;Parent=MALK_00257
contig001	maker	exon	455264	457204	.	-	.	ID=MALK_00257.t1.e1;Parent=MALK_00257.t1
contig001	AUGUSTUS	gene	457516	458370	0.99	+	.	ID=MALK_00258;prediction_source=braker_MRET:g915.t1
contig001	AUGUSTUS	CDS	457516	458370	0.99	+	0	ID=MALK_00258.t1.c1;Parent=MALK_00258.t1
contig001	AUGUSTUS	mRNA	457516	458370	0.99	+	.	ID=MALK_00258.t1;Parent=MALK_00258
contig001	AUGUSTUS	exon	457516	458370	.	+	.	ID=MALK_00258.t1.e1;Parent=MALK_00258.t1
contig001	AUGUSTUS	gene	458453	461197	0.97	-	.	ID=MALK_00259;prediction_source=augustus:contig001.g582.t1
contig001	AUGUSTUS	CDS	458453	461197	0.97	-	0	ID=MALK_00259.t1.c1;Parent=MALK_00259.t1
contig001	AUGUSTUS	mRNA	458453	461197	0.97	-	.	ID=MALK_00259.t1;Parent=MALK_00259
contig001	AUGUSTUS	exon	458453	461197	0.97	-	.	ID=MALK_00259.t1.e1;Parent=MALK_00259.t1
contig001	AUGUSTUS	gene	461300	461655	0.91	-	.	ID=MALK_00260;prediction_source=braker_MRET:g917.t1
contig001	AUGUSTUS	CDS	461556	461655	0.97	-	0	ID=MALK_00260.t1.c2;Parent=MALK_00260.t1
contig001	AUGUSTUS	CDS	461300	461508	0.97	-	0	ID=MALK_00260.t1.c1;Parent=MALK_00260.t1
contig001	AUGUSTUS	mRNA	461300	461655	0.91	-	.	ID=MALK_00260.t1;Parent=MALK_00260
contig001	AUGUSTUS	exon	461556	461655	.	-	.	ID=MALK_00260.t1.e2;Parent=MALK_00260.t1
contig001	AUGUSTUS	exon	461300	461508	.	-	.	ID=MALK_00260.t1.e1;Parent=MALK_00260.t1
contig001	maker	gene	461791	463551	.	+	.	ID=MALK_00261;prediction_source=maker_MRET:augustus_masked-contig001-processed-gene-4.21-mRNA-1

contig001	maker	CDS	461791	463551	.	+	0	ID=MALK_00261.t1.c1;Parent=MALK_00261.t1
contig001	maker	mRNA	461791	463551	.	+	.	ID=MALK_00261.t1;Parent=MALK_00261
contig001	maker	exon	461791	463551	.	+	.	ID=MALK_00261.t1.e1;Parent=MALK_00261.t1
contig001	AUGUSTUS	gene	463653	465943	0.35	-	.	ID=MALK_00262;prediction_source=braker_MRET:g919.t1
contig001	AUGUSTUS	CDS	465284	465943	0.35	-	0	ID=MALK_00262.t1.c2;Parent=MALK_00262.t1
contig001	AUGUSTUS	CDS	463653	465215	0.35	-	0	ID=MALK_00262.t1.c1;Parent=MALK_00262.t1
contig001	AUGUSTUS	mRNA	463653	465943	0.35	-	.	ID=MALK_00262.t1;Parent=MALK_00262
contig001	AUGUSTUS	exon	465284	465943	.	-	.	ID=MALK_00262.t1.e2;Parent=MALK_00262.t1
contig001	AUGUSTUS	exon	463653	465215	.	-	.	ID=MALK_00262.t1.e1;Parent=MALK_00262.t1
contig001	maker	gene	466347	469796	.	+	.	ID=MALK_00263;prediction_source=maker_MRET:augustus_masked-contig001-processed-gene-4.22-mRNA-1
contig001	maker	CDS	466347	469796	.	+	0	ID=MALK_00263.t1.c1;Parent=MALK_00263.t1
contig001	maker	mRNA	466347	469796	.	+	.	ID=MALK_00263.t1;Parent=MALK_00263
contig001	maker	exon	466347	469796	.	+	.	ID=MALK_00263.t1.e1;Parent=MALK_00263.t1
contig001	AUGUSTUS	gene	469899	470219	0.99	+	.	ID=MALK_00264;prediction_source=braker_MRET:g921.t1
contig001	AUGUSTUS	CDS	469899	470089	0.99	+	0	ID=MALK_00264.t1.c1;Parent=MALK_00264.t1
contig001	AUGUSTUS	CDS	470122	470146	0.99	+	0	ID=MALK_00264.t1.c2;Parent=MALK_00264.t1
contig001	AUGUSTUS	CDS	470181	470219	0.99	+	0	ID=MALK_00264.t1.c3;Parent=MALK_00264.t1
contig001	AUGUSTUS	mRNA	469899	470219	0.99	+	.	ID=MALK_00264.t1;Parent=MALK_00264
contig001	AUGUSTUS	exon	469899	470089	.	+	.	ID=MALK_00264.t1.e1;Parent=MALK_00264.t1
contig001	AUGUSTUS	exon	470122	470146	.	+	.	ID=MALK_00264.t1.e2;Parent=MALK_00264.t1
contig001	AUGUSTUS	exon	470181	470219	.	+	.	ID=MALK_00264.t1.e3;Parent=MALK_00264.t1
contig001	AUGUSTUS	gene	470623	472132	0.96	+	.	ID=MALK_00265;prediction_source=braker_MRET:g922.t1
contig001	AUGUSTUS	CDS	470623	470976	0.96	+	0	ID=MALK_00265.t1.c1;Parent=MALK_00265.t1
contig001	AUGUSTUS	CDS	471005	472132	0.96	+	0	ID=MALK_00265.t1.c2;Parent=MALK_00265.t1
contig001	AUGUSTUS	mRNA	470623	472132	0.96	+	.	ID=MALK_00265.t1;Parent=MALK_00265
contig001	AUGUSTUS	exon	470623	470976	.	+	.	ID=MALK_00265.t1.e1;Parent=MALK_00265.t1
contig001	AUGUSTUS	exon	471005	472132	.	+	.	ID=MALK_00265.t1.e2;Parent=MALK_00265.t1
contig001	AUGUSTUS	gene	472744	473732	0.59	+	.	ID=MALK_00266;prediction_source=braker_MRET:g923.t1
contig001	AUGUSTUS	CDS	472744	473668	0.92	+	0	ID=MALK_00266.t1.c1;Parent=MALK_00266.t1
contig001	AUGUSTUS	CDS	473725	473732	0.92	+	0	ID=MALK_00266.t1.c2;Parent=MALK_00266.t1
contig001	AUGUSTUS	mRNA	472744	473732	0.59	+	.	ID=MALK_00266.t1;Parent=MALK_00266
contig001	AUGUSTUS	exon	472744	473668	.	+	.	ID=MALK_00266.t1.e1;Parent=MALK_00266.t1
contig001	AUGUSTUS	exon	473725	473732	.	+	.	ID=MALK_00266.t1.e2;Parent=MALK_00266.t1
contig001	maker	gene	473808	474782	.	-	.	ID=MALK_00267;prediction_source=maker_MRET:augustus_masked-contig001-processed-gene-4.41-mRNA-1
contig001	maker	CDS	473808	474782	.	-	0	ID=MALK_00267.t1.c1;Parent=MALK_00267.t1
contig001	maker	mRNA	473808	474782	.	-	.	ID=MALK_00267.t1;Parent=MALK_00267
contig001	maker	exon	473808	474782	.	-	.	ID=MALK_00267.t1.e1;Parent=MALK_00267.t1
contig001	AUGUSTUS	gene	474883	475989	0.99	+	.	ID=MALK_00268;prediction_source=augustus:contig001.g591.t1
contig001	AUGUSTUS	CDS	474883	475989	0.99	+	0	ID=MALK_00268.t1.c1;Parent=MALK_00268.t1
contig001	AUGUSTUS	mRNA	474883	475989	0.99	+	.	ID=MALK_00268.t1;Parent=MALK_00268
contig001	AUGUSTUS	exon	474883	475989	0.99	+	.	ID=MALK_00268.t1.e1;Parent=MALK_00268.t1
contig001	AUGUSTUS	gene	476907	478085	0.99	-	.	ID=MALK_00269;prediction_source=augustus:contig001.g592.t1
contig001	AUGUSTUS	CDS	476907	478085	0.99	-	0	ID=MALK_00269.t1.c1;Parent=MALK_00269.t1

contig001	AUGUSTUS	mRNA	476907	478085	0.99	-	.	ID=MALK_00269.t1;Parent=MALK_00269
contig001	AUGUSTUS	exon	476907	478085	0.99	-	.	ID=MALK_00269.t1.e1;Parent=MALK_00269.t1
contig001	maker	gene	478154	479944	.	-	.	ID=MALK_00270;prediction_source=maker_MRET:augustus_masked-contig001-processed-gene-4.43-mRNA-1
contig001	maker	CDS	478154	479944	.	-	0	ID=MALK_00270.t1.c1;Parent=MALK_00270.t1
contig001	maker	mRNA	478154	479944	.	-	.	ID=MALK_00270.t1;Parent=MALK_00270
contig001	maker	exon	478154	479944	.	-	.	ID=MALK_00270.t1.e1;Parent=MALK_00270.t1
contig001	AUGUSTUS	gene	480570	482216	1	-	.	ID=MALK_00271;prediction_source=augustus:contig001.g595.t1
contig001	AUGUSTUS	CDS	480570	482216	1	-	0	ID=MALK_00271.t1.c1;Parent=MALK_00271.t1
contig001	AUGUSTUS	mRNA	480570	482216	1	-	.	ID=MALK_00271.t1;Parent=MALK_00271
contig001	AUGUSTUS	exon	480570	482216	1	-	.	ID=MALK_00271.t1.e1;Parent=MALK_00271.t1
contig001	maker	gene	482368	485091	.	+	.	ID=MALK_00272;prediction_source=maker_MRET:augustus_masked-contig001-processed-gene-4.25-mRNA-1
contig001	maker	CDS	482368	485091	.	+	0	ID=MALK_00272.t1.c1;Parent=MALK_00272.t1
contig001	maker	mRNA	482368	485091	.	+	.	ID=MALK_00272.t1;Parent=MALK_00272
contig001	maker	exon	482368	485091	.	+	.	ID=MALK_00272.t1.e1;Parent=MALK_00272.t1
contig001	AUGUSTUS	gene	486695	487465	0.89	+	.	ID=MALK_00273;prediction_source=braker_MRET:g930.t1
contig001	AUGUSTUS	CDS	486695	486724	1	+	0	ID=MALK_00273.t1.c1;Parent=MALK_00273.t1
contig001	AUGUSTUS	CDS	486756	486899	1	+	0	ID=MALK_00273.t1.c2;Parent=MALK_00273.t1
contig001	AUGUSTUS	CDS	486929	487056	1	+	0	ID=MALK_00273.t1.c3;Parent=MALK_00273.t1
contig001	AUGUSTUS	CDS	487086	487221	1	+	0	ID=MALK_00273.t1.c4;Parent=MALK_00273.t1
contig001	AUGUSTUS	CDS	487259	487395	1	+	0	ID=MALK_00273.t1.c5;Parent=MALK_00273.t1
contig001	AUGUSTUS	CDS	487438	487465	1	+	0	ID=MALK_00273.t1.c6;Parent=MALK_00273.t1
contig001	AUGUSTUS	mRNA	486695	487465	0.89	+	.	ID=MALK_00273.t1;Parent=MALK_00273
contig001	AUGUSTUS	exon	486695	486724	.	+	.	ID=MALK_00273.t1.e1;Parent=MALK_00273.t1
contig001	AUGUSTUS	exon	486756	486899	.	+	.	ID=MALK_00273.t1.e2;Parent=MALK_00273.t1
contig001	AUGUSTUS	exon	486929	487056	.	+	.	ID=MALK_00273.t1.e3;Parent=MALK_00273.t1
contig001	AUGUSTUS	exon	487086	487221	.	+	.	ID=MALK_00273.t1.e4;Parent=MALK_00273.t1
contig001	AUGUSTUS	exon	487259	487395	.	+	.	ID=MALK_00273.t1.e5;Parent=MALK_00273.t1
contig001	AUGUSTUS	exon	487438	487465	.	+	.	ID=MALK_00273.t1.e6;Parent=MALK_00273.t1
contig001	AUGUSTUS	gene	487665	488741	1	+	.	ID=MALK_00274;prediction_source=augustus:contig001.g597.t1
contig001	AUGUSTUS	CDS	487665	488741	1	+	0	ID=MALK_00274.t1.c1;Parent=MALK_00274.t1
contig001	AUGUSTUS	mRNA	487665	488741	1	+	.	ID=MALK_00274.t1;Parent=MALK_00274
contig001	AUGUSTUS	exon	487665	488741	1	+	.	ID=MALK_00274.t1.e1;Parent=MALK_00274.t1
contig001	AUGUSTUS	gene	488881	489232	0.72	-	.	ID=MALK_00275;prediction_source=braker_MRET:g932.t1
contig001	AUGUSTUS	CDS	489177	489232	0.72	-	0	ID=MALK_00275.t1.c2;Parent=MALK_00275.t1
contig001	AUGUSTUS	CDS	488881	489148	0.72	-	0	ID=MALK_00275.t1.c1;Parent=MALK_00275.t1
contig001	AUGUSTUS	mRNA	488881	489232	0.72	-	.	ID=MALK_00275.t1;Parent=MALK_00275
contig001	AUGUSTUS	exon	489177	489232	.	-	.	ID=MALK_00275.t1.e2;Parent=MALK_00275.t1
contig001	AUGUSTUS	exon	488881	489148	.	-	.	ID=MALK_00275.t1.e1;Parent=MALK_00275.t1
contig001	AUGUSTUS	gene	489509	492409	0.71	+	.	ID=MALK_00276;prediction_source=augustus:contig001.g598.t1
contig001	AUGUSTUS	CDS	489509	492409	0.71	+	0	ID=MALK_00276.t1.c1;Parent=MALK_00276.t1
contig001	AUGUSTUS	mRNA	489509	492409	0.71	+	.	ID=MALK_00276.t1;Parent=MALK_00276
contig001	AUGUSTUS	exon	489509	492409	0.71	+	.	ID=MALK_00276.t1.e1;Parent=MALK_00276.t1
contig001	AUGUSTUS	gene	492510	495482	0.88	-	.	ID=MALK_00277;prediction_source=augustus:contig001.g599.t1

contig001	AUGUSTUS	CDS	492510	495482	0.88	-	0	ID=MALK_00277.t1.c1;Parent=MALK_00277.t1
contig001	AUGUSTUS	mRNA	492510	495482	0.88	-	.	ID=MALK_00277.t1;Parent=MALK_00277
contig001	AUGUSTUS	exon	492510	495482	0.88	-	.	ID=MALK_00277.t1.e1;Parent=MALK_00277.t1
contig001	AUGUSTUS	gene	495925	498309	0.83	-	.	ID=MALK_00278;prediction_source=augustus:contig001.g602.t1
contig001	AUGUSTUS	CDS	495925	498309	0.83	-	0	ID=MALK_00278.t1.c1;Parent=MALK_00278.t1
contig001	AUGUSTUS	mRNA	495925	498309	0.83	-	.	ID=MALK_00278.t1;Parent=MALK_00278
contig001	AUGUSTUS	exon	495925	498309	0.83	-	.	ID=MALK_00278.t1.e1;Parent=MALK_00278.t1
contig001	AUGUSTUS	gene	498585	499496	0.72	-	.	ID=MALK_00279;prediction_source=augustus:contig001.g603.t1
contig001	AUGUSTUS	CDS	498585	499496	0.72	-	0	ID=MALK_00279.t1.c1;Parent=MALK_00279.t1
contig001	AUGUSTUS	mRNA	498585	499496	0.72	-	.	ID=MALK_00279.t1;Parent=MALK_00279
contig001	AUGUSTUS	exon	498585	499496	0.72	-	.	ID=MALK_00279.t1.e1;Parent=MALK_00279.t1
contig001	maker	gene	499752	500714	.	+	.	ID=MALK_00280;prediction_source=maker_MRET:augustus_masked-contig001-processed-gene-5.46-mRNA-1
contig001	maker	CDS	499752	500714	.	+	0	ID=MALK_00280.t1.c1;Parent=MALK_00280.t1
contig001	maker	mRNA	499752	500714	.	+	.	ID=MALK_00280.t1;Parent=MALK_00280
contig001	maker	exon	499752	500714	.	+	.	ID=MALK_00280.t1.e1;Parent=MALK_00280.t1
contig001	AUGUSTUS	gene	500812	502050	0.91	-	.	ID=MALK_00281;prediction_source=augustus:contig001.g606.t1
contig001	AUGUSTUS	CDS	500812	502050	0.91	-	0	ID=MALK_00281.t1.c1;Parent=MALK_00281.t1
contig001	AUGUSTUS	mRNA	500812	502050	0.91	-	.	ID=MALK_00281.t1;Parent=MALK_00281
contig001	AUGUSTUS	exon	500812	502050	0.91	-	.	ID=MALK_00281.t1.e1;Parent=MALK_00281.t1
contig001	AUGUSTUS	gene	502120	503001	1	+	.	ID=MALK_00282;prediction_source=augustus:contig001.g608.t1
contig001	AUGUSTUS	CDS	502120	503001	1	+	0	ID=MALK_00282.t1.c1;Parent=MALK_00282.t1
contig001	AUGUSTUS	mRNA	502120	503001	1	+	.	ID=MALK_00282.t1;Parent=MALK_00282
contig001	AUGUSTUS	exon	502120	503001	1	+	.	ID=MALK_00282.t1.e1;Parent=MALK_00282.t1
contig001	AUGUSTUS	gene	503100	503717	0.92	-	.	ID=MALK_00283;prediction_source=braker_MRET:g940.t1
contig001	AUGUSTUS	CDS	503100	503717	0.92	-	0	ID=MALK_00283.t1.c1;Parent=MALK_00283.t1
contig001	AUGUSTUS	mRNA	503100	503717	0.92	-	.	ID=MALK_00283.t1;Parent=MALK_00283
contig001	AUGUSTUS	exon	503100	503717	.	-	.	ID=MALK_00283.t1.e1;Parent=MALK_00283.t1
contig001	AUGUSTUS	gene	504003	506285	0.95	+	.	ID=MALK_00284;prediction_source=augustus:contig001.g610.t1
contig001	AUGUSTUS	CDS	504003	506285	0.95	+	0	ID=MALK_00284.t1.c1;Parent=MALK_00284.t1
contig001	AUGUSTUS	mRNA	504003	506285	0.95	+	.	ID=MALK_00284.t1;Parent=MALK_00284
contig001	AUGUSTUS	exon	504003	506285	0.95	+	.	ID=MALK_00284.t1.e1;Parent=MALK_00284.t1
contig001	AUGUSTUS	gene	506992	507819	0.97	+	.	ID=MALK_00285;prediction_source=augustus:contig001.g611.t1
contig001	AUGUSTUS	CDS	506992	507819	0.97	+	0	ID=MALK_00285.t1.c1;Parent=MALK_00285.t1
contig001	AUGUSTUS	mRNA	506992	507819	0.97	+	.	ID=MALK_00285.t1;Parent=MALK_00285
contig001	AUGUSTUS	exon	506992	507819	0.97	+	.	ID=MALK_00285.t1.e1;Parent=MALK_00285.t1
contig001	AUGUSTUS	gene	507821	509467	0.83	-	.	ID=MALK_00286;prediction_source=augustus:contig001.g612.t1
contig001	AUGUSTUS	CDS	507821	509467	0.83	-	0	ID=MALK_00286.t1.c1;Parent=MALK_00286.t1
contig001	AUGUSTUS	mRNA	507821	509467	0.83	-	.	ID=MALK_00286.t1;Parent=MALK_00286
contig001	AUGUSTUS	exon	507821	509467	0.83	-	.	ID=MALK_00286.t1.e1;Parent=MALK_00286.t1
contig001	AUGUSTUS	gene	509526	510380	0.49	+	.	ID=MALK_00287;prediction_source=braker_MRET:g944.t1
contig001	AUGUSTUS	CDS	509526	509616	0.87	+	0	ID=MALK_00287.t1.c1;Parent=MALK_00287.t1
contig001	AUGUSTUS	CDS	509654	509739	0.87	+	0	ID=MALK_00287.t1.c2;Parent=MALK_00287.t1
contig001	AUGUSTUS	CDS	509772	510380	0.87	+	0	ID=MALK_00287.t1.c3;Parent=MALK_00287.t1

contig001	AUGUSTUS	mRNA	509526	510380	0.49	+	.	ID=MALK_00287.t1;Parent=MALK_00287
contig001	AUGUSTUS	exon	509526	509616	.	+	.	ID=MALK_00287.t1.e1;Parent=MALK_00287.t1
contig001	AUGUSTUS	exon	509654	509739	.	+	.	ID=MALK_00287.t1.e2;Parent=MALK_00287.t1
contig001	AUGUSTUS	exon	509772	510380	.	+	.	ID=MALK_00287.t1.e3;Parent=MALK_00287.t1
contig001	AUGUSTUS	gene	510424	511689	0.9	+	.	ID=MALK_00288;prediction_source=braker_MRET:g945.t1
contig001	AUGUSTUS	CDS	510424	511689	0.9	+	0	ID=MALK_00288.t1.c1;Parent=MALK_00288.t1
contig001	AUGUSTUS	mRNA	510424	511689	0.9	+	.	ID=MALK_00288.t1;Parent=MALK_00288
contig001	AUGUSTUS	exon	510424	511689	.	+	.	ID=MALK_00288.t1.e1;Parent=MALK_00288.t1
contig001	AUGUSTUS	gene	511700	514765	0.95	-	.	ID=MALK_00289;prediction_source=augustus:contig001.g614.t1
contig001	AUGUSTUS	CDS	511700	514765	0.95	-	0	ID=MALK_00289.t1.c1;Parent=MALK_00289.t1
contig001	AUGUSTUS	mRNA	511700	514765	0.95	-	.	ID=MALK_00289.t1;Parent=MALK_00289
contig001	AUGUSTUS	exon	511700	514765	0.95	-	.	ID=MALK_00289.t1.e1;Parent=MALK_00289.t1
contig001	AUGUSTUS	gene	514723	515733	0.9	+	.	ID=MALK_00290;prediction_source=augustus:contig001.g615.t1
contig001	AUGUSTUS	CDS	514723	515733	0.9	+	0	ID=MALK_00290.t1.c1;Parent=MALK_00290.t1
contig001	AUGUSTUS	mRNA	514723	515733	0.9	+	.	ID=MALK_00290.t1;Parent=MALK_00290
contig001	AUGUSTUS	exon	514723	515733	0.9	+	.	ID=MALK_00290.t1.e1;Parent=MALK_00290.t1
contig001	maker	gene	515730	518117	.	-	.	ID=MALK_00291;prediction_source=maker_MRET:augustus_masked-contig001-processed-gene-5.75-mRNA-1
contig001	maker	CDS	515730	518117	.	-	0	ID=MALK_00291.t1.c1;Parent=MALK_00291.t1
contig001	maker	mRNA	515730	518117	.	-	.	ID=MALK_00291.t1;Parent=MALK_00291
contig001	maker	exon	515730	518117	.	-	.	ID=MALK_00291.t1.e1;Parent=MALK_00291.t1
contig001	AUGUSTUS	gene	518285	520189	0.86	+	.	ID=MALK_00292;prediction_source=augustus:contig001.g617.t1
contig001	AUGUSTUS	CDS	518285	520189	0.86	+	0	ID=MALK_00292.t1.c1;Parent=MALK_00292.t1
contig001	AUGUSTUS	mRNA	518285	520189	0.86	+	.	ID=MALK_00292.t1;Parent=MALK_00292
contig001	AUGUSTUS	exon	518285	520189	0.86	+	.	ID=MALK_00292.t1.e1;Parent=MALK_00292.t1
contig001	maker	gene	520199	520837	.	-	.	ID=MALK_00293;prediction_source=maker_MRET:augustus_masked-contig001-processed-gene-5.76-mRNA-1
contig001	maker	CDS	520199	520837	.	-	0	ID=MALK_00293.t1.c1;Parent=MALK_00293.t1
contig001	maker	mRNA	520199	520837	.	-	.	ID=MALK_00293.t1;Parent=MALK_00293
contig001	maker	exon	520199	520837	.	-	.	ID=MALK_00293.t1.e1;Parent=MALK_00293.t1
contig001	AUGUSTUS	gene	520984	524325	0.81	-	.	ID=MALK_00294;prediction_source=augustus:contig001.g619.t1
contig001	AUGUSTUS	CDS	520984	524325	0.81	-	0	ID=MALK_00294.t1.c1;Parent=MALK_00294.t1
contig001	AUGUSTUS	mRNA	520984	524325	0.81	-	.	ID=MALK_00294.t1;Parent=MALK_00294
contig001	AUGUSTUS	exon	520984	524325	0.81	-	.	ID=MALK_00294.t1.e1;Parent=MALK_00294.t1
contig001	AUGUSTUS	gene	524915	526336	0.85	-	.	ID=MALK_00295;prediction_source=braker_MRET:g952.t1
contig001	AUGUSTUS	CDS	524915	526336	0.85	-	0	ID=MALK_00295.t1.c1;Parent=MALK_00295.t1
contig001	AUGUSTUS	mRNA	524915	526336	0.85	-	.	ID=MALK_00295.t1;Parent=MALK_00295
contig001	AUGUSTUS	exon	524915	526336	.	-	.	ID=MALK_00295.t1.e1;Parent=MALK_00295.t1
contig001	maker	gene	526476	528152	.	+	.	ID=MALK_00296;prediction_source=maker_MRET:augustus_masked-contig001-processed-gene-5.56-mRNA-1
contig001	maker	CDS	526476	528152	.	+	0	ID=MALK_00296.t1.c1;Parent=MALK_00296.t1
contig001	maker	mRNA	526476	528152	.	+	.	ID=MALK_00296.t1;Parent=MALK_00296
contig001	maker	exon	526476	528152	.	+	.	ID=MALK_00296.t1.e1;Parent=MALK_00296.t1
contig001	AUGUSTUS	gene	528226	529035	1	-	.	ID=MALK_00297;prediction_source=braker_MRET:g954.t1
contig001	AUGUSTUS	CDS	528226	529035	1	-	0	ID=MALK_00297.t1.c1;Parent=MALK_00297.t1
contig001	AUGUSTUS	mRNA	528226	529035	1	-	.	ID=MALK_00297.t1;Parent=MALK_00297

contig001	AUGUSTUS	exon	528226	529035	.	-	.	ID=MALK_00297.t1.e1;Parent=MALK_00297.t1
contig001	AUGUSTUS	gene	529062	531572	0.95	+	.	ID=MALK_00298;prediction_source=augustus:contig001.g624.t1
contig001	AUGUSTUS	CDS	529062	531572	0.95	+	0	ID=MALK_00298.t1.c1;Parent=MALK_00298.t1
contig001	AUGUSTUS	mRNA	529062	531572	0.95	+	.	ID=MALK_00298.t1;Parent=MALK_00298
contig001	AUGUSTUS	exon	529062	531572	0.95	+	.	ID=MALK_00298.t1.e1;Parent=MALK_00298.t1
contig001	AUGUSTUS	gene	531591	532898	1	-	.	ID=MALK_00299;prediction_source=augustus:contig001.g625.t1
contig001	AUGUSTUS	CDS	531591	532898	1	-	0	ID=MALK_00299.t1.c1;Parent=MALK_00299.t1
contig001	AUGUSTUS	mRNA	531591	532898	1	-	.	ID=MALK_00299.t1;Parent=MALK_00299
contig001	AUGUSTUS	exon	531591	532898	1	-	.	ID=MALK_00299.t1.e1;Parent=MALK_00299.t1
contig001	AUGUSTUS	gene	533253	533777	1	+	.	ID=MALK_00300;prediction_source=braker_MRET:g957.t1
contig001	AUGUSTUS	CDS	533253	533255	1	+	0	ID=MALK_00300.t1.c1;Parent=MALK_00300.t1
contig001	AUGUSTUS	CDS	533298	533777	1	+	0	ID=MALK_00300.t1.c2;Parent=MALK_00300.t1
contig001	AUGUSTUS	mRNA	533253	533777	1	+	.	ID=MALK_00300.t1;Parent=MALK_00300
contig001	AUGUSTUS	exon	533253	533255	.	+	.	ID=MALK_00300.t1.e1;Parent=MALK_00300.t1
contig001	AUGUSTUS	exon	533298	533777	.	+	.	ID=MALK_00300.t1.e2;Parent=MALK_00300.t1
contig001	maker	gene	533900	535852	.	-	.	ID=MALK_00301;prediction_source=maker_MRET:augustus_masked-contig001-processed-gene-5.80-mRNA-1
contig001	maker	CDS	533900	535852	.	-	0	ID=MALK_00301.t1.c1;Parent=MALK_00301.t1
contig001	maker	mRNA	533900	535852	.	-	.	ID=MALK_00301.t1;Parent=MALK_00301
contig001	maker	exon	533900	535852	.	-	.	ID=MALK_00301.t1.e1;Parent=MALK_00301.t1
contig001	maker	gene	536249	537610	.	+	.	ID=MALK_00302;prediction_source=maker_MRET:augustus_masked-contig001-processed-gene-5.59-mRNA-1
contig001	maker	CDS	536249	537610	.	+	0	ID=MALK_00302.t1.c1;Parent=MALK_00302.t1
contig001	maker	mRNA	536249	537610	.	+	.	ID=MALK_00302.t1;Parent=MALK_00302
contig001	maker	exon	536249	537610	.	+	.	ID=MALK_00302.t1.e1;Parent=MALK_00302.t1
contig001	maker	gene	537685	543830	.	-	.	ID=MALK_00303;prediction_source=maker_MRET:augustus_masked-contig001-processed-gene-5.81-mRNA-1
contig001	maker	CDS	543358	543830	.	-	0	ID=MALK_00303.t1.c1;Parent=MALK_00303.t1
contig001	maker	CDS	537685	543199	.	-	0	ID=MALK_00303.t1.c2;Parent=MALK_00303.t1
contig001	maker	mRNA	537685	543830	.	-	.	ID=MALK_00303.t1;Parent=MALK_00303
contig001	maker	exon	543358	543830	.	-	.	ID=MALK_00303.t1.e1;Parent=MALK_00303.t1
contig001	maker	exon	537685	543199	.	-	.	ID=MALK_00303.t1.e2;Parent=MALK_00303.t1
contig001	maker	gene	544351	545358	.	-	.	ID=MALK_00304;prediction_source=maker_MRET:augustus_masked-contig001-processed-gene-5.82-mRNA-1
contig001	maker	CDS	544351	545358	.	-	0	ID=MALK_00304.t1.c1;Parent=MALK_00304.t1
contig001	maker	mRNA	544351	545358	.	-	.	ID=MALK_00304.t1;Parent=MALK_00304
contig001	maker	exon	544351	545358	.	-	.	ID=MALK_00304.t1.e1;Parent=MALK_00304.t1
contig001	AUGUSTUS	gene	545719	546450	0.77	+	.	ID=MALK_00305;prediction_source=braker_MRET:g961.t1
contig001	AUGUSTUS	CDS	545719	546450	0.77	+	0	ID=MALK_00305.t1.c1;Parent=MALK_00305.t1
contig001	AUGUSTUS	mRNA	545719	546450	0.77	+	.	ID=MALK_00305.t1;Parent=MALK_00305
contig001	AUGUSTUS	exon	545719	546450	.	+	.	ID=MALK_00305.t1.e1;Parent=MALK_00305.t1
contig001	AUGUSTUS	gene	546451	548440	0.92	-	.	ID=MALK_00306;prediction_source=augustus:contig001.g634.t1
contig001	AUGUSTUS	CDS	548351	548440	0.98	-	0	ID=MALK_00306.t1.c3;Parent=MALK_00306.t1
contig001	AUGUSTUS	CDS	547477	548287	0.98	-	0	ID=MALK_00306.t1.c2;Parent=MALK_00306.t1
contig001	AUGUSTUS	CDS	546451	547355	0.98	-	0	ID=MALK_00306.t1.c1;Parent=MALK_00306.t1
contig001	AUGUSTUS	mRNA	546451	548440	0.92	-	.	ID=MALK_00306.t1;Parent=MALK_00306
contig001	AUGUSTUS	exon	548351	548440	0.98	-	.	ID=MALK_00306.t1.e3;Parent=MALK_00306.t1

contig001	AUGUSTUS	exon	547477	548287	0.98	-	.	ID=MALK_00306.t1.e2;Parent=MALK_00306.t1
contig001	AUGUSTUS	exon	546451	547355	0.98	-	.	ID=MALK_00306.t1.e1;Parent=MALK_00306.t1
contig001	AUGUSTUS	gene	548838	549890	0.53	+	.	ID=MALK_00307;prediction_source=augustus:contig001.g636.t1
contig001	AUGUSTUS	CDS	548838	549890	0.53	+	0	ID=MALK_00307.t1.c1;Parent=MALK_00307.t1
contig001	AUGUSTUS	mRNA	548838	549890	0.53	+	.	ID=MALK_00307.t1;Parent=MALK_00307
contig001	AUGUSTUS	exon	548838	549890	0.53	+	.	ID=MALK_00307.t1.e1;Parent=MALK_00307.t1
contig001	AUGUSTUS	gene	549897	550949	0.61	-	.	ID=MALK_00308;prediction_source=braker_MRET:g964.t1
contig001	AUGUSTUS	CDS	550043	550949	0.61	-	0	ID=MALK_00308.t1.c2;Parent=MALK_00308.t1
contig001	AUGUSTUS	CDS	549897	550009	0.61	-	0	ID=MALK_00308.t1.c1;Parent=MALK_00308.t1
contig001	AUGUSTUS	mRNA	549897	550949	0.61	-	.	ID=MALK_00308.t1;Parent=MALK_00308
contig001	AUGUSTUS	exon	550043	550949	.	-	.	ID=MALK_00308.t1.e2;Parent=MALK_00308.t1
contig001	AUGUSTUS	exon	549897	550009	.	-	.	ID=MALK_00308.t1.e1;Parent=MALK_00308.t1
contig001	maker	gene	552184	553125	.	+	.	ID=MALK_00309;prediction_source=maker_MRET:augustus_masked-contig001-processed-gene-5.61-mRNA-1
contig001	maker	CDS	552184	553125	.	+	0	ID=MALK_00309.t1.c1;Parent=MALK_00309.t1
contig001	maker	mRNA	552184	553125	.	+	.	ID=MALK_00309.t1;Parent=MALK_00309
contig001	maker	exon	552184	553125	.	+	.	ID=MALK_00309.t1.e1;Parent=MALK_00309.t1
contig001	AUGUSTUS	gene	553700	554689	1	+	.	ID=MALK_00310;prediction_source=augustus:contig001.g639.t1
contig001	AUGUSTUS	CDS	553700	554689	1	+	0	ID=MALK_00310.t1.c1;Parent=MALK_00310.t1
contig001	AUGUSTUS	mRNA	553700	554689	1	+	.	ID=MALK_00310.t1;Parent=MALK_00310
contig001	AUGUSTUS	exon	553700	554689	1	+	.	ID=MALK_00310.t1.e1;Parent=MALK_00310.t1
contig001	AUGUSTUS	gene	555524	556836	0.52	-	.	ID=MALK_00311;prediction_source=braker_MRET:g967.t1
contig001	AUGUSTUS	CDS	556770	556836	0.94	-	0	ID=MALK_00311.t1.c6;Parent=MALK_00311.t1
contig001	AUGUSTUS	CDS	556607	556736	0.94	-	0	ID=MALK_00311.t1.c5;Parent=MALK_00311.t1
contig001	AUGUSTUS	CDS	556492	556577	0.94	-	0	ID=MALK_00311.t1.c4;Parent=MALK_00311.t1
contig001	AUGUSTUS	CDS	556374	556452	0.94	-	0	ID=MALK_00311.t1.c3;Parent=MALK_00311.t1
contig001	AUGUSTUS	CDS	556048	556193	0.94	-	0	ID=MALK_00311.t1.c2;Parent=MALK_00311.t1
contig001	AUGUSTUS	CDS	555524	556020	0.94	-	0	ID=MALK_00311.t1.c1;Parent=MALK_00311.t1
contig001	AUGUSTUS	mRNA	555524	556836	0.52	-	.	ID=MALK_00311.t1;Parent=MALK_00311
contig001	AUGUSTUS	exon	556770	556836	.	-	.	ID=MALK_00311.t1.e6;Parent=MALK_00311.t1
contig001	AUGUSTUS	exon	556607	556736	.	-	.	ID=MALK_00311.t1.e5;Parent=MALK_00311.t1
contig001	AUGUSTUS	exon	556492	556577	.	-	.	ID=MALK_00311.t1.e4;Parent=MALK_00311.t1
contig001	AUGUSTUS	exon	556374	556452	.	-	.	ID=MALK_00311.t1.e3;Parent=MALK_00311.t1
contig001	AUGUSTUS	exon	556048	556193	.	-	.	ID=MALK_00311.t1.e2;Parent=MALK_00311.t1
contig001	AUGUSTUS	exon	555524	556020	.	-	.	ID=MALK_00311.t1.e1;Parent=MALK_00311.t1
contig001	AUGUSTUS	gene	557000	558309	0.97	+	.	ID=MALK_00312;prediction_source=braker_MRET:g968.t1
contig001	AUGUSTUS	CDS	557000	558254	0.99	+	0	ID=MALK_00312.t1.c1;Parent=MALK_00312.t1
contig001	AUGUSTUS	CDS	558296	558309	0.99	+	0	ID=MALK_00312.t1.c2;Parent=MALK_00312.t1
contig001	AUGUSTUS	mRNA	557000	558309	0.97	+	.	ID=MALK_00312.t1;Parent=MALK_00312
contig001	AUGUSTUS	exon	557000	558254	.	+	.	ID=MALK_00312.t1.e1;Parent=MALK_00312.t1
contig001	AUGUSTUS	exon	558296	558309	.	+	.	ID=MALK_00312.t1.e2;Parent=MALK_00312.t1
contig001	AUGUSTUS	gene	558382	560094	0.82	-	.	ID=MALK_00313;prediction_source=augustus:contig001.g644.t1
contig001	AUGUSTUS	CDS	558382	560094	0.82	-	0	ID=MALK_00313.t1.c1;Parent=MALK_00313.t1
contig001	AUGUSTUS	mRNA	558382	560094	0.82	-	.	ID=MALK_00313.t1;Parent=MALK_00313

contig001	AUGUSTUS	exon	558382	560094	0.82	-	.	ID=MALK_00313.t1.e1;Parent=MALK_00313.t1
contig001	AUGUSTUS	gene	560289	561260	0.96	+	.	ID=MALK_00314;prediction_source=augustus:contig001.g645.t1
contig001	AUGUSTUS	CDS	560289	561260	0.96	+	0	ID=MALK_00314.t1.c1;Parent=MALK_00314.t1
contig001	AUGUSTUS	mRNA	560289	561260	0.96	+	.	ID=MALK_00314.t1;Parent=MALK_00314
contig001	AUGUSTUS	exon	560289	561260	0.96	+	.	ID=MALK_00314.t1.e1;Parent=MALK_00314.t1
contig001	AUGUSTUS	gene	561257	562798	1	-	.	ID=MALK_00315;prediction_source=augustus:contig001.g646.t1
contig001	AUGUSTUS	CDS	561257	562798	1	-	0	ID=MALK_00315.t1.c1;Parent=MALK_00315.t1
contig001	AUGUSTUS	mRNA	561257	562798	1	-	.	ID=MALK_00315.t1;Parent=MALK_00315
contig001	AUGUSTUS	exon	561257	562798	1	-	.	ID=MALK_00315.t1.e1;Parent=MALK_00315.t1
contig001	AUGUSTUS	gene	562967	563986	1	+	.	ID=MALK_00316;prediction_source=augustus:contig001.g647.t1
contig001	AUGUSTUS	CDS	562967	563986	1	+	0	ID=MALK_00316.t1.c1;Parent=MALK_00316.t1
contig001	AUGUSTUS	mRNA	562967	563986	1	+	.	ID=MALK_00316.t1;Parent=MALK_00316
contig001	AUGUSTUS	exon	562967	563986	1	+	.	ID=MALK_00316.t1.e1;Parent=MALK_00316.t1
contig001	AUGUSTUS	gene	564893	566368	0.9	+	.	ID=MALK_00317;prediction_source=augustus:contig001.g648.t1
contig001	AUGUSTUS	CDS	564893	566368	0.9	+	0	ID=MALK_00317.t1.c1;Parent=MALK_00317.t1
contig001	AUGUSTUS	mRNA	564893	566368	0.9	+	.	ID=MALK_00317.t1;Parent=MALK_00317
contig001	AUGUSTUS	exon	564893	566368	0.9	+	.	ID=MALK_00317.t1.e1;Parent=MALK_00317.t1
contig001	AUGUSTUS	gene	566637	567579	0.9	-	.	ID=MALK_00318;prediction_source=braker_MRET:g974.t1
contig001	AUGUSTUS	CDS	567542	567579	0.9	-	0	ID=MALK_00318.t1.c2;Parent=MALK_00318.t1
contig001	AUGUSTUS	CDS	566637	567513	0.9	-	0	ID=MALK_00318.t1.c1;Parent=MALK_00318.t1
contig001	AUGUSTUS	mRNA	566637	567579	0.9	-	.	ID=MALK_00318.t1;Parent=MALK_00318
contig001	AUGUSTUS	exon	567542	567579	.	-	.	ID=MALK_00318.t1.e2;Parent=MALK_00318.t1
contig001	AUGUSTUS	exon	566637	567513	.	-	.	ID=MALK_00318.t1.e1;Parent=MALK_00318.t1
contig001	maker	gene	567695	568828	.	-	.	ID=MALK_00319;prediction_source=maker_MRET:augustus_masked-contig001-processed-gene-5.89-mRNA-1
contig001	maker	CDS	567695	568828	.	-	0	ID=MALK_00319.t1.c1;Parent=MALK_00319.t1
contig001	maker	mRNA	567695	568828	.	-	.	ID=MALK_00319.t1;Parent=MALK_00319
contig001	maker	exon	567695	568828	.	-	.	ID=MALK_00319.t1.e1;Parent=MALK_00319.t1
contig001	maker	gene	569127	572927	.	+	.	ID=MALK_00320;prediction_source=maker_MRET:augustus_masked-contig001-processed-gene-5.67-mRNA-1
contig001	maker	CDS	569127	572927	.	+	0	ID=MALK_00320.t1.c1;Parent=MALK_00320.t1
contig001	maker	mRNA	569127	572927	.	+	.	ID=MALK_00320.t1;Parent=MALK_00320
contig001	maker	exon	569127	572927	.	+	.	ID=MALK_00320.t1.e1;Parent=MALK_00320.t1
contig001	AUGUSTUS	gene	573031	575280	0.7	-	.	ID=MALK_00321;prediction_source=augustus:contig001.g655.t1
contig001	AUGUSTUS	CDS	573031	575280	0.7	-	0	ID=MALK_00321.t1.c1;Parent=MALK_00321.t1
contig001	AUGUSTUS	mRNA	573031	575280	0.7	-	.	ID=MALK_00321.t1;Parent=MALK_00321
contig001	AUGUSTUS	exon	573031	575280	0.7	-	.	ID=MALK_00321.t1.e1;Parent=MALK_00321.t1
contig001	maker	gene	575329	581862	.	-	.	ID=MALK_00322;prediction_source=maker_MRET:augustus_masked-contig001-processed-gene-5.91-mRNA-1
contig001	maker	CDS	575329	581862	.	-	0	ID=MALK_00322.t1.c1;Parent=MALK_00322.t1
contig001	maker	mRNA	575329	581862	.	-	.	ID=MALK_00322.t1;Parent=MALK_00322
contig001	maker	exon	575329	581862	.	-	.	ID=MALK_00322.t1.e1;Parent=MALK_00322.t1
contig001	maker	gene	582136	583713	.	+	.	ID=MALK_00323;prediction_source=maker_MRET:augustus_masked-contig001-processed-gene-5.68-mRNA-1
contig001	maker	CDS	582136	583713	.	+	0	ID=MALK_00323.t1.c1;Parent=MALK_00323.t1
contig001	maker	mRNA	582136	583713	.	+	.	ID=MALK_00323.t1;Parent=MALK_00323
contig001	maker	exon	582136	583713	.	+	.	ID=MALK_00323.t1.e1;Parent=MALK_00323.t1

contig001	maker	gene	585043	588261	.	+	.	ID=MALK_00324;prediction_source=maker_MRET:augustus_masked-contig001-processed-gene-5.69-mRNA-1
contig001	maker	CDS	585043	588261	.	+	0	ID=MALK_00324.t1.c1;Parent=MALK_00324.t1
contig001	maker	mRNA	585043	588261	.	+	.	ID=MALK_00324.t1;Parent=MALK_00324
contig001	maker	exon	585043	588261	.	+	.	ID=MALK_00324.t1.e1;Parent=MALK_00324.t1
contig001	AUGUSTUS	gene	588728	589555	0.76	+	.	ID=MALK_00325;prediction_source=augustus:contig001.g663.t1
contig001	AUGUSTUS	CDS	588728	589555	0.76	+	0	ID=MALK_00325.t1.c1;Parent=MALK_00325.t1
contig001	AUGUSTUS	mRNA	588728	589555	0.76	+	.	ID=MALK_00325.t1;Parent=MALK_00325
contig001	AUGUSTUS	exon	588728	589555	0.76	+	.	ID=MALK_00325.t1.e1;Parent=MALK_00325.t1
contig001	AUGUSTUS	gene	589595	590953	0.98	-	.	ID=MALK_00326;prediction_source=augustus:contig001.g664.t1
contig001	AUGUSTUS	CDS	589595	590953	0.98	-	0	ID=MALK_00326.t1.c1;Parent=MALK_00326.t1
contig001	AUGUSTUS	mRNA	589595	590953	0.98	-	.	ID=MALK_00326.t1;Parent=MALK_00326
contig001	AUGUSTUS	exon	589595	590953	0.98	-	.	ID=MALK_00326.t1.e1;Parent=MALK_00326.t1
contig001	AUGUSTUS	gene	591217	596298	0.8	+	.	ID=MALK_00327;prediction_source=augustus:contig001.g665.t1
contig001	AUGUSTUS	CDS	591217	596298	0.8	+	0	ID=MALK_00327.t1.c1;Parent=MALK_00327.t1
contig001	AUGUSTUS	mRNA	591217	596298	0.8	+	.	ID=MALK_00327.t1;Parent=MALK_00327
contig001	AUGUSTUS	exon	591217	596298	0.8	+	.	ID=MALK_00327.t1.e1;Parent=MALK_00327.t1
contig001	AUGUSTUS	gene	597111	598035	0.68	+	.	ID=MALK_00328;prediction_source=braker_MRET:g984.t1
contig001	AUGUSTUS	CDS	597111	597117	1	+	0	ID=MALK_00328.t1.c1;Parent=MALK_00328.t1
contig001	AUGUSTUS	CDS	597152	597255	1	+	0	ID=MALK_00328.t1.c2;Parent=MALK_00328.t1
contig001	AUGUSTUS	CDS	597287	597921	1	+	0	ID=MALK_00328.t1.c3;Parent=MALK_00328.t1
contig001	AUGUSTUS	CDS	597954	598035	1	+	0	ID=MALK_00328.t1.c4;Parent=MALK_00328.t1
contig001	AUGUSTUS	mRNA	597111	598035	0.68	+	.	ID=MALK_00328.t1;Parent=MALK_00328
contig001	AUGUSTUS	exon	597111	597117	.	+	.	ID=MALK_00328.t1.e1;Parent=MALK_00328.t1
contig001	AUGUSTUS	exon	597152	597255	.	+	.	ID=MALK_00328.t1.e2;Parent=MALK_00328.t1
contig001	AUGUSTUS	exon	597287	597921	.	+	.	ID=MALK_00328.t1.e3;Parent=MALK_00328.t1
contig001	AUGUSTUS	exon	597954	598035	.	+	.	ID=MALK_00328.t1.e4;Parent=MALK_00328.t1
contig001	maker	gene	598559	600007	.	+	.	ID=MALK_00329;prediction_source=maker_MRET:augustus_masked-contig001-processed-gene-6.36-mRNA-1
contig001	maker	CDS	598559	600007	.	+	0	ID=MALK_00329.t1.c1;Parent=MALK_00329.t1
contig001	maker	mRNA	598559	600007	.	+	.	ID=MALK_00329.t1;Parent=MALK_00329
contig001	maker	exon	598559	600007	.	+	.	ID=MALK_00329.t1.e1;Parent=MALK_00329.t1
contig001	AUGUSTUS	gene	600046	602560	1	-	.	ID=MALK_00330;prediction_source=braker_MRET:g986.t1
contig001	AUGUSTUS	CDS	602482	602560	1	-	0	ID=MALK_00330.t1.c2;Parent=MALK_00330.t1
contig001	AUGUSTUS	CDS	600046	602279	1	-	0	ID=MALK_00330.t1.c1;Parent=MALK_00330.t1
contig001	AUGUSTUS	mRNA	600046	602560	1	-	.	ID=MALK_00330.t1;Parent=MALK_00330
contig001	AUGUSTUS	exon	602482	602560	.	-	.	ID=MALK_00330.t1.e2;Parent=MALK_00330.t1
contig001	AUGUSTUS	exon	600046	602279	.	-	.	ID=MALK_00330.t1.e1;Parent=MALK_00330.t1
contig001	maker	gene	603181	603685	.	+	.	ID=MALK_00331;prediction_source=maker_MRET:augustus_masked-contig001-processed-gene-6.37-mRNA-1
contig001	maker	CDS	603181	603315	.	+	0	ID=MALK_00331.t1.c1;Parent=MALK_00331.t1
contig001	maker	CDS	603416	603685	.	+	0	ID=MALK_00331.t1.c2;Parent=MALK_00331.t1
contig001	maker	mRNA	603181	603685	.	+	.	ID=MALK_00331.t1;Parent=MALK_00331
contig001	maker	exon	603181	603315	.	+	.	ID=MALK_00331.t1.e1;Parent=MALK_00331.t1
contig001	maker	exon	603416	603685	.	+	.	ID=MALK_00331.t1.e2;Parent=MALK_00331.t1
contig001	AUGUSTUS	gene	603918	605294	0.25	-	.	ID=MALK_00332;prediction_source=augustus:contig001.g673.t1

contig001	AUGUSTUS	CDS	603918	605294	0.25	-	0	ID=MALK_00332.t1.c1;Parent=MALK_00332.t1
contig001	AUGUSTUS	mRNA	603918	605294	0.25	-	.	ID=MALK_00332.t1;Parent=MALK_00332
contig001	AUGUSTUS	exon	603918	605294	0.25	-	.	ID=MALK_00332.t1.e1;Parent=MALK_00332.t1
contig001	AUGUSTUS	gene	605670	607295	0.46	-	.	ID=MALK_00333;prediction_source=augustus:contig001.g674.t1
contig001	AUGUSTUS	CDS	605670	607295	0.46	-	0	ID=MALK_00333.t1.c1;Parent=MALK_00333.t1
contig001	AUGUSTUS	mRNA	605670	607295	0.46	-	.	ID=MALK_00333.t1;Parent=MALK_00333
contig001	AUGUSTUS	exon	605670	607295	0.46	-	.	ID=MALK_00333.t1.e1;Parent=MALK_00333.t1
contig001	AUGUSTUS	gene	607534	611163	0.9	+	.	ID=MALK_00334;prediction_source=augustus:contig001.g675.t1
contig001	AUGUSTUS	CDS	607534	611163	0.9	+	0	ID=MALK_00334.t1.c1;Parent=MALK_00334.t1
contig001	AUGUSTUS	mRNA	607534	611163	0.9	+	.	ID=MALK_00334.t1;Parent=MALK_00334
contig001	AUGUSTUS	exon	607534	611163	0.9	+	.	ID=MALK_00334.t1.e1;Parent=MALK_00334.t1
contig001	AUGUSTUS	gene	611312	612409	0.14	+	.	ID=MALK_00335;prediction_source=braker_MRET:g990.t1
contig001	AUGUSTUS	CDS	611312	611457	0.6	+	0	ID=MALK_00335.t1.c1;Parent=MALK_00335.t1
contig001	AUGUSTUS	CDS	611495	611503	0.6	+	0	ID=MALK_00335.t1.c2;Parent=MALK_00335.t1
contig001	AUGUSTUS	CDS	611539	612409	0.6	+	0	ID=MALK_00335.t1.c3;Parent=MALK_00335.t1
contig001	AUGUSTUS	mRNA	611312	612409	0.14	+	.	ID=MALK_00335.t1;Parent=MALK_00335
contig001	AUGUSTUS	exon	611312	611457	.	+	.	ID=MALK_00335.t1.e1;Parent=MALK_00335.t1
contig001	AUGUSTUS	exon	611495	611503	.	+	.	ID=MALK_00335.t1.e2;Parent=MALK_00335.t1
contig001	AUGUSTUS	exon	611539	612409	.	+	.	ID=MALK_00335.t1.e3;Parent=MALK_00335.t1
contig001	AUGUSTUS	gene	612568	615222	0.94	-	.	ID=MALK_00336;prediction_source=augustus:contig001.g677.t1
contig001	AUGUSTUS	CDS	612568	615222	0.94	-	0	ID=MALK_00336.t1.c1;Parent=MALK_00336.t1
contig001	AUGUSTUS	mRNA	612568	615222	0.94	-	.	ID=MALK_00336.t1;Parent=MALK_00336
contig001	AUGUSTUS	exon	612568	615222	0.94	-	.	ID=MALK_00336.t1.e1;Parent=MALK_00336.t1
contig001	maker	gene	615943	616536	.	+	.	ID=MALK_00337;prediction_source=maker_MRET:augustus_masked-contig001-processed-gene-6.40-mRNA-1
contig001	maker	CDS	615943	616536	.	+	0	ID=MALK_00337.t1.c1;Parent=MALK_00337.t1
contig001	maker	mRNA	615943	616536	.	+	.	ID=MALK_00337.t1;Parent=MALK_00337
contig001	maker	exon	615943	616536	.	+	.	ID=MALK_00337.t1.e1;Parent=MALK_00337.t1
contig001	AUGUSTUS	gene	617030	618431	0.72	-	.	ID=MALK_00338;prediction_source=braker_MRET:g993.t1
contig001	AUGUSTUS	CDS	618363	618431	0.72	-	0	ID=MALK_00338.t1.c4;Parent=MALK_00338.t1
contig001	AUGUSTUS	CDS	618313	618335	0.72	-	0	ID=MALK_00338.t1.c3;Parent=MALK_00338.t1
contig001	AUGUSTUS	CDS	617734	618256	0.72	-	0	ID=MALK_00338.t1.c2;Parent=MALK_00338.t1
contig001	AUGUSTUS	CDS	617030	617446	0.72	-	0	ID=MALK_00338.t1.c1;Parent=MALK_00338.t1
contig001	AUGUSTUS	mRNA	617030	618431	0.72	-	.	ID=MALK_00338.t1;Parent=MALK_00338
contig001	AUGUSTUS	exon	618363	618431	.	-	.	ID=MALK_00338.t1.e4;Parent=MALK_00338.t1
contig001	AUGUSTUS	exon	618313	618335	.	-	.	ID=MALK_00338.t1.e3;Parent=MALK_00338.t1
contig001	AUGUSTUS	exon	617734	618256	.	-	.	ID=MALK_00338.t1.e2;Parent=MALK_00338.t1
contig001	AUGUSTUS	exon	617030	617446	.	-	.	ID=MALK_00338.t1.e1;Parent=MALK_00338.t1
contig001	AUGUSTUS	gene	618576	619151	0.87	+	.	ID=MALK_00339;prediction_source=augustus:contig001.g681.t1
contig001	AUGUSTUS	CDS	618576	618686	0.96	+	0	ID=MALK_00339.t1.c1;Parent=MALK_00339.t1
contig001	AUGUSTUS	CDS	618765	619151	0.96	+	0	ID=MALK_00339.t1.c2;Parent=MALK_00339.t1
contig001	AUGUSTUS	mRNA	618576	619151	0.87	+	.	ID=MALK_00339.t1;Parent=MALK_00339
contig001	AUGUSTUS	exon	618576	618686	0.96	+	.	ID=MALK_00339.t1.e1;Parent=MALK_00339.t1
contig001	AUGUSTUS	exon	618765	619151	0.96	+	.	ID=MALK_00339.t1.e2;Parent=MALK_00339.t1

contig001	maker	gene	619430	619966	.	-	.	ID=MALK_00340;prediction_source=maker_MRET:augustus_masked-contig001-processed-gene-6.62-mRNA-1
contig001	maker	CDS	619430	619966	.	-	0	ID=MALK_00340.t1.c1;Parent=MALK_00340.t1
contig001	maker	mRNA	619430	619966	.	-	.	ID=MALK_00340.t1;Parent=MALK_00340
contig001	maker	exon	619430	619966	.	-	.	ID=MALK_00340.t1.e1;Parent=MALK_00340.t1
contig001	AUGUSTUS	gene	621434	623191	0.59	+	.	ID=MALK_00341;prediction_source=augustus:contig001.g683.t1
contig001	AUGUSTUS	CDS	621434	623191	0.59	+	0	ID=MALK_00341.t1.c1;Parent=MALK_00341.t1
contig001	AUGUSTUS	mRNA	621434	623191	0.59	+	.	ID=MALK_00341.t1;Parent=MALK_00341
contig001	AUGUSTUS	exon	621434	623191	0.59	+	.	ID=MALK_00341.t1.e1;Parent=MALK_00341.t1
contig001	AUGUSTUS	gene	623188	624303	0.98	-	.	ID=MALK_00342;prediction_source=augustus:contig001.g684.t1
contig001	AUGUSTUS	CDS	623188	624303	0.98	-	0	ID=MALK_00342.t1.c1;Parent=MALK_00342.t1
contig001	AUGUSTUS	mRNA	623188	624303	0.98	-	.	ID=MALK_00342.t1;Parent=MALK_00342
contig001	AUGUSTUS	exon	623188	624303	0.98	-	.	ID=MALK_00342.t1.e1;Parent=MALK_00342.t1
contig001	AUGUSTUS	gene	624443	625801	0.73	+	.	ID=MALK_00343;prediction_source=augustus:contig001.g685.t1
contig001	AUGUSTUS	CDS	624443	625801	0.73	+	0	ID=MALK_00343.t1.c1;Parent=MALK_00343.t1
contig001	AUGUSTUS	mRNA	624443	625801	0.73	+	.	ID=MALK_00343.t1;Parent=MALK_00343
contig001	AUGUSTUS	exon	624443	625801	0.73	+	.	ID=MALK_00343.t1.e1;Parent=MALK_00343.t1
contig001	maker	gene	625803	626354	.	-	.	ID=MALK_00344;prediction_source=maker_MRET:augustus_masked-contig001-processed-gene-6.64-mRNA-1
contig001	maker	CDS	625803	626354	.	-	0	ID=MALK_00344.t1.c1;Parent=MALK_00344.t1
contig001	maker	mRNA	625803	626354	.	-	.	ID=MALK_00344.t1;Parent=MALK_00344
contig001	maker	exon	625803	626354	.	-	.	ID=MALK_00344.t1.e1;Parent=MALK_00344.t1
contig001	AUGUSTUS	gene	626464	629589	0.98	-	.	ID=MALK_00345;prediction_source=augustus:contig001.g688.t1
contig001	AUGUSTUS	CDS	626464	629589	0.98	-	0	ID=MALK_00345.t1.c1;Parent=MALK_00345.t1
contig001	AUGUSTUS	mRNA	626464	629589	0.98	-	.	ID=MALK_00345.t1;Parent=MALK_00345
contig001	AUGUSTUS	exon	626464	629589	0.98	-	.	ID=MALK_00345.t1.e1;Parent=MALK_00345.t1
contig001	AUGUSTUS	gene	629797	631383	1	+	.	ID=MALK_00346;prediction_source=augustus:contig001.g689.t1
contig001	AUGUSTUS	CDS	629797	631383	1	+	0	ID=MALK_00346.t1.c1;Parent=MALK_00346.t1
contig001	AUGUSTUS	mRNA	629797	631383	1	+	.	ID=MALK_00346.t1;Parent=MALK_00346
contig001	AUGUSTUS	exon	629797	631383	1	+	.	ID=MALK_00346.t1.e1;Parent=MALK_00346.t1
contig001	maker	gene	631596	633665	.	+	.	ID=MALK_00347;prediction_source=maker_MRET:augustus_masked-contig001-processed-gene-6.45-mRNA-1
contig001	maker	CDS	631596	633665	.	+	0	ID=MALK_00347.t1.c1;Parent=MALK_00347.t1
contig001	maker	mRNA	631596	633665	.	+	.	ID=MALK_00347.t1;Parent=MALK_00347
contig001	maker	exon	631596	633665	.	+	.	ID=MALK_00347.t1.e1;Parent=MALK_00347.t1
contig001	AUGUSTUS	gene	633781	636808	0.23	+	.	ID=MALK_00348;prediction_source=braker_MRET:g1003.t1
contig001	AUGUSTUS	CDS	633781	634382	0.33	+	0	ID=MALK_00348.t1.c1;Parent=MALK_00348.t1
contig001	AUGUSTUS	CDS	634558	636808	0.33	+	0	ID=MALK_00348.t1.c2;Parent=MALK_00348.t1
contig001	AUGUSTUS	mRNA	633781	636808	0.23	+	.	ID=MALK_00348.t1;Parent=MALK_00348
contig001	AUGUSTUS	exon	633781	634382	.	+	.	ID=MALK_00348.t1.e1;Parent=MALK_00348.t1
contig001	AUGUSTUS	exon	634558	636808	.	+	.	ID=MALK_00348.t1.e2;Parent=MALK_00348.t1
contig001	AUGUSTUS	gene	636818	637788	0.67	-	.	ID=MALK_00349;prediction_source=braker_MRET:g1004.t1
contig001	AUGUSTUS	CDS	637653	637788	0.67	-	0	ID=MALK_00349.t1.c2;Parent=MALK_00349.t1
contig001	AUGUSTUS	CDS	636818	637623	0.67	-	0	ID=MALK_00349.t1.c1;Parent=MALK_00349.t1
contig001	AUGUSTUS	mRNA	636818	637788	0.67	-	.	ID=MALK_00349.t1;Parent=MALK_00349
contig001	AUGUSTUS	exon	637653	637788	.	-	.	ID=MALK_00349.t1.e2;Parent=MALK_00349.t1

contig001	AUGUSTUS	exon	636818	637623	.	-	.	ID=MALK_00349.t1.e1;Parent=MALK_00349.t1
contig001	maker	gene	638342	639160	.	+	.	ID=MALK_00350;prediction_source=maker_MRET:augustus_masked-contig001-processed-gene-6.48-mRNA-1
contig001	maker	CDS	638342	639160	.	+	0	ID=MALK_00350.t1.c1;Parent=MALK_00350.t1
contig001	maker	mRNA	638342	639160	.	+	.	ID=MALK_00350.t1;Parent=MALK_00350
contig001	maker	exon	638342	639160	.	+	.	ID=MALK_00350.t1.e1;Parent=MALK_00350.t1
contig001	AUGUSTUS	gene	639178	641928	0.68	-	.	ID=MALK_00351;prediction_source=braker_MRET:g1006.t1
contig001	AUGUSTUS	CDS	639178	641928	0.68	-	0	ID=MALK_00351.t1.c1;Parent=MALK_00351.t1
contig001	AUGUSTUS	mRNA	639178	641928	0.68	-	.	ID=MALK_00351.t1;Parent=MALK_00351
contig001	AUGUSTUS	exon	639178	641928	.	-	.	ID=MALK_00351.t1.e1;Parent=MALK_00351.t1
contig001	maker	gene	642129	643526	.	+	.	ID=MALK_00352;prediction_source=maker_MRET:augustus_masked-contig001-processed-gene-6.49-mRNA-1
contig001	maker	CDS	642129	643526	.	+	0	ID=MALK_00352.t1.c1;Parent=MALK_00352.t1
contig001	maker	mRNA	642129	643526	.	+	.	ID=MALK_00352.t1;Parent=MALK_00352
contig001	maker	exon	642129	643526	.	+	.	ID=MALK_00352.t1.e1;Parent=MALK_00352.t1
contig001	AUGUSTUS	gene	643659	646868	0.74	+	.	ID=MALK_00353;prediction_source=augustus:contig001.g698.t1
contig001	AUGUSTUS	CDS	643659	646868	0.74	+	0	ID=MALK_00353.t1.c1;Parent=MALK_00353.t1
contig001	AUGUSTUS	mRNA	643659	646868	0.74	+	.	ID=MALK_00353.t1;Parent=MALK_00353
contig001	AUGUSTUS	exon	643659	646868	0.74	+	.	ID=MALK_00353.t1.e1;Parent=MALK_00353.t1
contig001	AUGUSTUS	gene	646893	650456	0.99	-	.	ID=MALK_00354;prediction_source=augustus:contig001.g699.t1
contig001	AUGUSTUS	CDS	646893	650456	0.99	-	0	ID=MALK_00354.t1.c1;Parent=MALK_00354.t1
contig001	AUGUSTUS	mRNA	646893	650456	0.99	-	.	ID=MALK_00354.t1;Parent=MALK_00354
contig001	AUGUSTUS	exon	646893	650456	0.99	-	.	ID=MALK_00354.t1.e1;Parent=MALK_00354.t1
contig001	maker	gene	650810	657292	.	+	.	ID=MALK_00355;prediction_source=maker_MRET:augustus_masked-contig001-processed-gene-6.51-mRNA-1
contig001	maker	CDS	650810	657292	.	+	0	ID=MALK_00355.t1.c1;Parent=MALK_00355.t1
contig001	maker	mRNA	650810	657292	.	+	.	ID=MALK_00355.t1;Parent=MALK_00355
contig001	maker	exon	650810	657292	.	+	.	ID=MALK_00355.t1.e1;Parent=MALK_00355.t1
contig001	AUGUSTUS	gene	657309	657896	0.91	-	.	ID=MALK_00356;prediction_source=augustus:contig001.g702.t1
contig001	AUGUSTUS	CDS	657309	657896	0.91	-	0	ID=MALK_00356.t1.c1;Parent=MALK_00356.t1
contig001	AUGUSTUS	mRNA	657309	657896	0.91	-	.	ID=MALK_00356.t1;Parent=MALK_00356
contig001	AUGUSTUS	exon	657309	657896	0.91	-	.	ID=MALK_00356.t1.e1;Parent=MALK_00356.t1
contig001	AUGUSTUS	gene	658114	660144	0.96	+	.	ID=MALK_00357;prediction_source=augustus:contig001.g703.t1
contig001	AUGUSTUS	CDS	658114	660144	0.96	+	0	ID=MALK_00357.t1.c1;Parent=MALK_00357.t1
contig001	AUGUSTUS	mRNA	658114	660144	0.96	+	.	ID=MALK_00357.t1;Parent=MALK_00357
contig001	AUGUSTUS	exon	658114	660144	0.96	+	.	ID=MALK_00357.t1.e1;Parent=MALK_00357.t1
contig001	maker	gene	660232	662421	.	-	.	ID=MALK_00358;prediction_source=maker_MRET:augustus_masked-contig001-processed-gene-6.70-mRNA-1
contig001	maker	CDS	660232	662421	.	-	0	ID=MALK_00358.t1.c1;Parent=MALK_00358.t1
contig001	maker	mRNA	660232	662421	.	-	.	ID=MALK_00358.t1;Parent=MALK_00358
contig001	maker	exon	660232	662421	.	-	.	ID=MALK_00358.t1.e1;Parent=MALK_00358.t1
contig001	AUGUSTUS	gene	662609	666343	1	-	.	ID=MALK_00359;prediction_source=augustus:contig001.g705.t1
contig001	AUGUSTUS	CDS	662609	666343	1	-	0	ID=MALK_00359.t1.c1;Parent=MALK_00359.t1
contig001	AUGUSTUS	mRNA	662609	666343	1	-	.	ID=MALK_00359.t1;Parent=MALK_00359
contig001	AUGUSTUS	exon	662609	666343	1	-	.	ID=MALK_00359.t1.e1;Parent=MALK_00359.t1
contig001	maker	gene	666408	670196	.	-	.	ID=MALK_00360;prediction_source=maker_MRET:augustus_masked-contig001-processed-gene-6.72-mRNA-1
contig001	maker	CDS	666408	670196	.	-	0	ID=MALK_00360.t1.c1;Parent=MALK_00360.t1

contig001	maker	mRNA	666408	670196	.	-	.	ID=MALK_00360.t1;Parent=MALK_00360
contig001	maker	exon	666408	670196	.	-	.	ID=MALK_00360.t1.e1;Parent=MALK_00360.t1
contig001	AUGUSTUS	gene	670338	671690	0.8	+	.	ID=MALK_00361;prediction_source=augustus:contig001.g708.t1
contig001	AUGUSTUS	CDS	670338	671690	0.8	+	0	ID=MALK_00361.t1.c1;Parent=MALK_00361.t1
contig001	AUGUSTUS	mRNA	670338	671690	0.8	+	.	ID=MALK_00361.t1;Parent=MALK_00361
contig001	AUGUSTUS	exon	670338	671690	0.8	+	.	ID=MALK_00361.t1.e1;Parent=MALK_00361.t1
contig001	AUGUSTUS	gene	671644	672657	0.66	-	.	ID=MALK_00362;prediction_source=braker_MRET:g1017.t1
contig001	AUGUSTUS	CDS	671644	672657	0.66	-	0	ID=MALK_00362.t1.c1;Parent=MALK_00362.t1
contig001	AUGUSTUS	mRNA	671644	672657	0.66	-	.	ID=MALK_00362.t1;Parent=MALK_00362
contig001	AUGUSTUS	exon	671644	672657	.	-	.	ID=MALK_00362.t1.e1;Parent=MALK_00362.t1
contig001	AUGUSTUS	gene	672688	676487	0.22	-	.	ID=MALK_00363;prediction_source=braker_MRET:g1018.t1
contig001	AUGUSTUS	CDS	675110	676487	0.56	-	0	ID=MALK_00363.t1.c4;Parent=MALK_00363.t1
contig001	AUGUSTUS	CDS	675008	675072	0.56	-	0	ID=MALK_00363.t1.c3;Parent=MALK_00363.t1
contig001	AUGUSTUS	CDS	674688	674860	0.56	-	0	ID=MALK_00363.t1.c2;Parent=MALK_00363.t1
contig001	AUGUSTUS	CDS	672688	674656	0.56	-	0	ID=MALK_00363.t1.c1;Parent=MALK_00363.t1
contig001	AUGUSTUS	mRNA	672688	676487	0.22	-	.	ID=MALK_00363.t1;Parent=MALK_00363
contig001	AUGUSTUS	exon	675110	676487	.	-	.	ID=MALK_00363.t1.e4;Parent=MALK_00363.t1
contig001	AUGUSTUS	exon	675008	675072	.	-	.	ID=MALK_00363.t1.e3;Parent=MALK_00363.t1
contig001	AUGUSTUS	exon	674688	674860	.	-	.	ID=MALK_00363.t1.e2;Parent=MALK_00363.t1
contig001	AUGUSTUS	exon	672688	674656	.	-	.	ID=MALK_00363.t1.e1;Parent=MALK_00363.t1
contig001	AUGUSTUS	gene	676701	678383	1	-	.	ID=MALK_00364;prediction_source=braker_MRET:g1019.t1
contig001	AUGUSTUS	CDS	678239	678383	1	-	0	ID=MALK_00364.t1.c2;Parent=MALK_00364.t1
contig001	AUGUSTUS	CDS	676701	678190	1	-	0	ID=MALK_00364.t1.c1;Parent=MALK_00364.t1
contig001	AUGUSTUS	mRNA	676701	678383	1	-	.	ID=MALK_00364.t1;Parent=MALK_00364
contig001	AUGUSTUS	exon	678239	678383	.	-	.	ID=MALK_00364.t1.e2;Parent=MALK_00364.t1
contig001	AUGUSTUS	exon	676701	678190	.	-	.	ID=MALK_00364.t1.e1;Parent=MALK_00364.t1
contig001	AUGUSTUS	gene	679273	682104	0.86	+	.	ID=MALK_00365;prediction_source=augustus:contig001.g712.t1
contig001	AUGUSTUS	CDS	679273	682104	0.86	+	0	ID=MALK_00365.t1.c1;Parent=MALK_00365.t1
contig001	AUGUSTUS	mRNA	679273	682104	0.86	+	.	ID=MALK_00365.t1;Parent=MALK_00365
contig001	AUGUSTUS	exon	679273	682104	0.86	+	.	ID=MALK_00365.t1.e1;Parent=MALK_00365.t1
contig001	AUGUSTUS	gene	682086	683183	1	-	.	ID=MALK_00366;prediction_source=augustus:contig001.g713.t1
contig001	AUGUSTUS	CDS	682086	683183	1	-	0	ID=MALK_00366.t1.c1;Parent=MALK_00366.t1
contig001	AUGUSTUS	mRNA	682086	683183	1	-	.	ID=MALK_00366.t1;Parent=MALK_00366
contig001	AUGUSTUS	exon	682086	683183	1	-	.	ID=MALK_00366.t1.e1;Parent=MALK_00366.t1
contig001	maker	gene	683809	685614	.	+	.	ID=MALK_00367;prediction_source=maker_MRET:augustus_masked-contig001-processed-gene-6.54-mRNA-1
contig001	maker	CDS	683809	685614	.	+	0	ID=MALK_00367.t1.c1;Parent=MALK_00367.t1
contig001	maker	mRNA	683809	685614	.	+	.	ID=MALK_00367.t1;Parent=MALK_00367
contig001	maker	exon	683809	685614	.	+	.	ID=MALK_00367.t1.e1;Parent=MALK_00367.t1
contig001	AUGUSTUS	gene	685623	686415	0.78	-	.	ID=MALK_00368;prediction_source=braker_MRET:g1023.t1
contig001	AUGUSTUS	CDS	685834	686415	0.85	-	0	ID=MALK_00368.t1.c2;Parent=MALK_00368.t1
contig001	AUGUSTUS	CDS	685623	685793	0.85	-	0	ID=MALK_00368.t1.c1;Parent=MALK_00368.t1
contig001	AUGUSTUS	mRNA	685623	686415	0.78	-	.	ID=MALK_00368.t1;Parent=MALK_00368
contig001	AUGUSTUS	exon	685834	686415	.	-	.	ID=MALK_00368.t1.e2;Parent=MALK_00368.t1

contig001	AUGUSTUS	exon	685623	685793	.	-	.	ID=MALK_00368.t1.e1;Parent=MALK_00368.t1
contig001	maker	gene	686550	687779	.	+	.	ID=MALK_00369;prediction_source=maker_MRET:augustus_masked-contig001-processed-gene-6.55-mRNA-1
contig001	maker	CDS	686550	687779	.	+	0	ID=MALK_00369.t1.c1;Parent=MALK_00369.t1
contig001	maker	mRNA	686550	687779	.	+	.	ID=MALK_00369.t1;Parent=MALK_00369
contig001	maker	exon	686550	687779	.	+	.	ID=MALK_00369.t1.e1;Parent=MALK_00369.t1
contig001	maker	gene	688123	690126	.	-	.	ID=MALK_00370;prediction_source=maker_MRET:augustus_masked-contig001-processed-gene-6.77-mRNA-1
contig001	maker	CDS	688123	690126	.	-	0	ID=MALK_00370.t1.c1;Parent=MALK_00370.t1
contig001	maker	mRNA	688123	690126	.	-	.	ID=MALK_00370.t1;Parent=MALK_00370
contig001	maker	exon	688123	690126	.	-	.	ID=MALK_00370.t1.e1;Parent=MALK_00370.t1
contig001	maker	gene	690438	690926	.	+	.	ID=MALK_00371;prediction_source=maker_MRET:augustus_masked-contig001-processed-gene-6.56-mRNA-1
contig001	maker	CDS	690438	690926	.	+	0	ID=MALK_00371.t1.c1;Parent=MALK_00371.t1
contig001	maker	mRNA	690438	690926	.	+	.	ID=MALK_00371.t1;Parent=MALK_00371
contig001	maker	exon	690438	690926	.	+	.	ID=MALK_00371.t1.e1;Parent=MALK_00371.t1
contig001	AUGUSTUS	gene	690937	692385	0.96	-	.	ID=MALK_00372;prediction_source=braker_MRET:g1027.t1
contig001	AUGUSTUS	CDS	690937	692385	0.96	-	0	ID=MALK_00372.t1.c1;Parent=MALK_00372.t1
contig001	AUGUSTUS	mRNA	690937	692385	0.96	-	.	ID=MALK_00372.t1;Parent=MALK_00372
contig001	AUGUSTUS	exon	690937	692385	.	-	.	ID=MALK_00372.t1.e1;Parent=MALK_00372.t1
contig001	AUGUSTUS	gene	692738	694117	0.92	-	.	ID=MALK_00373;prediction_source=braker_MRET:g1028.t1
contig001	AUGUSTUS	CDS	692738	694117	0.92	-	0	ID=MALK_00373.t1.c1;Parent=MALK_00373.t1
contig001	AUGUSTUS	mRNA	692738	694117	0.92	-	.	ID=MALK_00373.t1;Parent=MALK_00373
contig001	AUGUSTUS	exon	692738	694117	.	-	.	ID=MALK_00373.t1.e1;Parent=MALK_00373.t1
contig001	AUGUSTUS	gene	694302	694667	0.29	+	.	ID=MALK_00374;prediction_source=braker_MRET:g1029.t1
contig001	AUGUSTUS	CDS	694302	694368	0.37	+	0	ID=MALK_00374.t1.c1;Parent=MALK_00374.t1
contig001	AUGUSTUS	CDS	694407	694461	0.37	+	0	ID=MALK_00374.t1.c2;Parent=MALK_00374.t1
contig001	AUGUSTUS	CDS	694505	694667	0.37	+	0	ID=MALK_00374.t1.c3;Parent=MALK_00374.t1
contig001	AUGUSTUS	mRNA	694302	694667	0.29	+	.	ID=MALK_00374.t1;Parent=MALK_00374
contig001	AUGUSTUS	exon	694302	694368	.	+	.	ID=MALK_00374.t1.e1;Parent=MALK_00374.t1
contig001	AUGUSTUS	exon	694407	694461	.	+	.	ID=MALK_00374.t1.e2;Parent=MALK_00374.t1
contig001	AUGUSTUS	exon	694505	694667	.	+	.	ID=MALK_00374.t1.e3;Parent=MALK_00374.t1
contig001	AUGUSTUS	gene	694674	695144	0.72	-	.	ID=MALK_00375;prediction_source=braker_MRET:g1030.t1
contig001	AUGUSTUS	CDS	694674	695144	0.72	-	0	ID=MALK_00375.t1.c1;Parent=MALK_00375.t1
contig001	AUGUSTUS	mRNA	694674	695144	0.72	-	.	ID=MALK_00375.t1;Parent=MALK_00375
contig001	AUGUSTUS	exon	694674	695144	.	-	.	ID=MALK_00375.t1.e1;Parent=MALK_00375.t1
contig001	maker	gene	695556	700124	.	+	.	ID=MALK_00376;prediction_source=maker_MRET:augustus_masked-contig001-processed-gene-7.0-mRNA-1
contig001	maker	CDS	695556	700124	.	+	0	ID=MALK_00376.t1.c1;Parent=MALK_00376.t1
contig001	maker	mRNA	695556	700124	.	+	.	ID=MALK_00376.t1;Parent=MALK_00376
contig001	maker	exon	695556	700124	.	+	.	ID=MALK_00376.t1.e1;Parent=MALK_00376.t1
contig001	maker	gene	700149	703595	.	-	.	ID=MALK_00377;prediction_source=maker_MRET:augustus_masked-contig001-processed-gene-7.28-mRNA-1
contig001	maker	CDS	700149	703595	.	-	0	ID=MALK_00377.t1.c1;Parent=MALK_00377.t1
contig001	maker	mRNA	700149	703595	.	-	.	ID=MALK_00377.t1;Parent=MALK_00377
contig001	maker	exon	700149	703595	.	-	.	ID=MALK_00377.t1.e1;Parent=MALK_00377.t1
contig001	AUGUSTUS	gene	703750	704571	1	+	.	ID=MALK_00378;prediction_source=augustus:contig001.g722.t1
contig001	AUGUSTUS	CDS	703750	704571	1	+	0	ID=MALK_00378.t1.c1;Parent=MALK_00378.t1

contig001	AUGUSTUS	mRNA	703750	704571	1	+	.	ID=MALK_00378.t1;Parent=MALK_00378
contig001	AUGUSTUS	exon	703750	704571	1	+	.	ID=MALK_00378.t1.e1;Parent=MALK_00378.t1
contig001	maker	gene	704584	707199	.	-	.	ID=MALK_00379;prediction_source=maker_MRET:augustus_masked-contig001-processed-gene-7.29-mRNA-1
contig001	maker	CDS	704584	707199	.	-	0	ID=MALK_00379.t1.c1;Parent=MALK_00379.t1
contig001	maker	mRNA	704584	707199	.	-	.	ID=MALK_00379.t1;Parent=MALK_00379
contig001	maker	exon	704584	707199	.	-	.	ID=MALK_00379.t1.e1;Parent=MALK_00379.t1
contig001	AUGUSTUS	gene	707351	708634	0.37	+	.	ID=MALK_00380;prediction_source=augustus:contig001.g724.t1
contig001	AUGUSTUS	CDS	707351	708634	0.37	+	0	ID=MALK_00380.t1.c1;Parent=MALK_00380.t1
contig001	AUGUSTUS	mRNA	707351	708634	0.37	+	.	ID=MALK_00380.t1;Parent=MALK_00380
contig001	AUGUSTUS	exon	707351	708634	0.37	+	.	ID=MALK_00380.t1.e1;Parent=MALK_00380.t1
contig001	AUGUSTUS	gene	708653	709354	0.33	-	.	ID=MALK_00381;prediction_source=augustus:contig001.g725.t1
contig001	AUGUSTUS	CDS	708653	709354	0.33	-	0	ID=MALK_00381.t1.c1;Parent=MALK_00381.t1
contig001	AUGUSTUS	mRNA	708653	709354	0.33	-	.	ID=MALK_00381.t1;Parent=MALK_00381
contig001	AUGUSTUS	exon	708653	709354	0.33	-	.	ID=MALK_00381.t1.e1;Parent=MALK_00381.t1
contig001	maker	gene	710373	710966	.	-	.	ID=MALK_00382;prediction_source=maker_MRET:augustus_masked-contig001-processed-gene-7.31-mRNA-1
contig001	maker	CDS	710373	710966	.	-	0	ID=MALK_00382.t1.c1;Parent=MALK_00382.t1
contig001	maker	mRNA	710373	710966	.	-	.	ID=MALK_00382.t1;Parent=MALK_00382
contig001	maker	exon	710373	710966	.	-	.	ID=MALK_00382.t1.e1;Parent=MALK_00382.t1
contig001	maker	gene	711046	711834	.	+	.	ID=MALK_00383;prediction_source=maker_MRET:augustus_masked-contig001-processed-gene-7.4-mRNA-1
contig001	maker	CDS	711046	711834	.	+	0	ID=MALK_00383.t1.c1;Parent=MALK_00383.t1
contig001	maker	mRNA	711046	711834	.	+	.	ID=MALK_00383.t1;Parent=MALK_00383
contig001	maker	exon	711046	711834	.	+	.	ID=MALK_00383.t1.e1;Parent=MALK_00383.t1
contig001	maker	gene	712084	715281	.	+	.	ID=MALK_00384;prediction_source=maker_MRET:augustus_masked-contig001-processed-gene-7.5-mRNA-1
contig001	maker	CDS	712084	715281	.	+	0	ID=MALK_00384.t1.c1;Parent=MALK_00384.t1
contig001	maker	mRNA	712084	715281	.	+	.	ID=MALK_00384.t1;Parent=MALK_00384
contig001	maker	exon	712084	715281	.	+	.	ID=MALK_00384.t1.e1;Parent=MALK_00384.t1
contig001	AUGUSTUS	gene	715342	718575	0.92	-	.	ID=MALK_00385;prediction_source=braker_MRET:g1039.t1
contig001	AUGUSTUS	CDS	718570	718575	0.92	-	0	ID=MALK_00385.t1.c2;Parent=MALK_00385.t1
contig001	AUGUSTUS	CDS	715342	718530	0.92	-	0	ID=MALK_00385.t1.c1;Parent=MALK_00385.t1
contig001	AUGUSTUS	mRNA	715342	718575	0.92	-	.	ID=MALK_00385.t1;Parent=MALK_00385
contig001	AUGUSTUS	exon	718570	718575	.	-	.	ID=MALK_00385.t1.e2;Parent=MALK_00385.t1
contig001	AUGUSTUS	exon	715342	718530	.	-	.	ID=MALK_00385.t1.e1;Parent=MALK_00385.t1
contig001	AUGUSTUS	gene	718848	719934	0.45	-	.	ID=MALK_00386;prediction_source=braker_MRET:g1040.t1
contig001	AUGUSTUS	CDS	719714	719934	0.46	-	0	ID=MALK_00386.t1.c2;Parent=MALK_00386.t1
contig001	AUGUSTUS	CDS	718848	719673	0.46	-	0	ID=MALK_00386.t1.c1;Parent=MALK_00386.t1
contig001	AUGUSTUS	mRNA	718848	719934	0.45	-	.	ID=MALK_00386.t1;Parent=MALK_00386
contig001	AUGUSTUS	exon	719714	719934	.	-	.	ID=MALK_00386.t1.e2;Parent=MALK_00386.t1
contig001	AUGUSTUS	exon	718848	719673	.	-	.	ID=MALK_00386.t1.e1;Parent=MALK_00386.t1
contig001	AUGUSTUS	gene	720043	720726	0.62	+	.	ID=MALK_00387;prediction_source=augustus:contig001.g731.t1
contig001	AUGUSTUS	CDS	720043	720726	0.62	+	0	ID=MALK_00387.t1.c1;Parent=MALK_00387.t1
contig001	AUGUSTUS	mRNA	720043	720726	0.62	+	.	ID=MALK_00387.t1;Parent=MALK_00387
contig001	AUGUSTUS	exon	720043	720726	0.62	+	.	ID=MALK_00387.t1.e1;Parent=MALK_00387.t1
contig001	maker	gene	720720	721928	.	-	.	ID=MALK_00388;prediction_source=maker_MRET:augustus_masked-contig001-processed-gene-7.33-mRNA-1

contig001	maker	CDS	720720	721928	.	-	0	ID=MALK_00388.t1.c1;Parent=MALK_00388.t1
contig001	maker	mRNA	720720	721928	.	-	.	ID=MALK_00388.t1;Parent=MALK_00388
contig001	maker	exon	720720	721928	.	-	.	ID=MALK_00388.t1.e1;Parent=MALK_00388.t1
contig001	AUGUSTUS	gene	722013	722591	0.98	+	.	ID=MALK_00389;prediction_source=augustus:contig001.g733.t1
contig001	AUGUSTUS	CDS	722013	722591	0.98	+	0	ID=MALK_00389.t1.c1;Parent=MALK_00389.t1
contig001	AUGUSTUS	mRNA	722013	722591	0.98	+	.	ID=MALK_00389.t1;Parent=MALK_00389
contig001	AUGUSTUS	exon	722013	722591	0.98	+	.	ID=MALK_00389.t1.e1;Parent=MALK_00389.t1
contig001	AUGUSTUS	gene	722899	724080	0.67	+	.	ID=MALK_00390;prediction_source=augustus:contig001.g734.t1
contig001	AUGUSTUS	CDS	722899	724080	0.67	+	0	ID=MALK_00390.t1.c1;Parent=MALK_00390.t1
contig001	AUGUSTUS	mRNA	722899	724080	0.67	+	.	ID=MALK_00390.t1;Parent=MALK_00390
contig001	AUGUSTUS	exon	722899	724080	0.67	+	.	ID=MALK_00390.t1.e1;Parent=MALK_00390.t1
contig001	AUGUSTUS	gene	724081	725433	0.9	-	.	ID=MALK_00391;prediction_source=braker_MRET:g1045.t1
contig001	AUGUSTUS	CDS	724081	725433	0.9	-	0	ID=MALK_00391.t1.c1;Parent=MALK_00391.t1
contig001	AUGUSTUS	mRNA	724081	725433	0.9	-	.	ID=MALK_00391.t1;Parent=MALK_00391
contig001	AUGUSTUS	exon	724081	725433	.	-	.	ID=MALK_00391.t1.e1;Parent=MALK_00391.t1
contig001	AUGUSTUS	gene	725860	726226	0.84	+	.	ID=MALK_00392;prediction_source=braker_MRET:g1046.t1
contig001	AUGUSTUS	CDS	725860	726109	0.85	+	0	ID=MALK_00392.t1.c1;Parent=MALK_00392.t1
contig001	AUGUSTUS	CDS	726189	726226	0.85	+	0	ID=MALK_00392.t1.c2;Parent=MALK_00392.t1
contig001	AUGUSTUS	mRNA	725860	726226	0.84	+	.	ID=MALK_00392.t1;Parent=MALK_00392
contig001	AUGUSTUS	exon	725860	726109	.	+	.	ID=MALK_00392.t1.e1;Parent=MALK_00392.t1
contig001	AUGUSTUS	exon	726189	726226	.	+	.	ID=MALK_00392.t1.e2;Parent=MALK_00392.t1
contig001	AUGUSTUS	gene	726333	727649	0.68	-	.	ID=MALK_00393;prediction_source=braker_MRET:g1047.t1
contig001	AUGUSTUS	CDS	726333	727649	0.68	-	0	ID=MALK_00393.t1.c1;Parent=MALK_00393.t1
contig001	AUGUSTUS	mRNA	726333	727649	0.68	-	.	ID=MALK_00393.t1;Parent=MALK_00393
contig001	AUGUSTUS	exon	726333	727649	.	-	.	ID=MALK_00393.t1.e1;Parent=MALK_00393.t1
contig001	AUGUSTUS	gene	727692	730133	0.98	-	.	ID=MALK_00394;prediction_source=braker_MRET:g1048.t1
contig001	AUGUSTUS	CDS	727692	730133	0.98	-	0	ID=MALK_00394.t1.c1;Parent=MALK_00394.t1
contig001	AUGUSTUS	mRNA	727692	730133	0.98	-	.	ID=MALK_00394.t1;Parent=MALK_00394
contig001	AUGUSTUS	exon	727692	730133	.	-	.	ID=MALK_00394.t1.e1;Parent=MALK_00394.t1
contig001	AUGUSTUS	gene	730248	731042	0.58	+	.	ID=MALK_00395;prediction_source=augustus:contig001.g737.t1
contig001	AUGUSTUS	CDS	730248	731042	0.58	+	0	ID=MALK_00395.t1.c1;Parent=MALK_00395.t1
contig001	AUGUSTUS	mRNA	730248	731042	0.58	+	.	ID=MALK_00395.t1;Parent=MALK_00395
contig001	AUGUSTUS	exon	730248	731042	0.58	+	.	ID=MALK_00395.t1.e1;Parent=MALK_00395.t1
contig001	AUGUSTUS	gene	731136	732740	0.99	+	.	ID=MALK_00396;prediction_source=braker_MRET:g1050.t1
contig001	AUGUSTUS	CDS	731136	732740	0.99	+	0	ID=MALK_00396.t1.c1;Parent=MALK_00396.t1
contig001	AUGUSTUS	mRNA	731136	732740	0.99	+	.	ID=MALK_00396.t1;Parent=MALK_00396
contig001	AUGUSTUS	exon	731136	732740	.	+	.	ID=MALK_00396.t1.e1;Parent=MALK_00396.t1
contig001	AUGUSTUS	gene	732748	733163	0.81	-	.	ID=MALK_00397;prediction_source=braker_MRET:g1051.t1
contig001	AUGUSTUS	CDS	733161	733163	0.88	-	0	ID=MALK_00397.t1.c3;Parent=MALK_00397.t1
contig001	AUGUSTUS	CDS	733114	733132	0.88	-	0	ID=MALK_00397.t1.c2;Parent=MALK_00397.t1
contig001	AUGUSTUS	CDS	732748	733025	0.88	-	0	ID=MALK_00397.t1.c1;Parent=MALK_00397.t1
contig001	AUGUSTUS	mRNA	732748	733163	0.81	-	.	ID=MALK_00397.t1;Parent=MALK_00397
contig001	AUGUSTUS	exon	733161	733163	.	-	.	ID=MALK_00397.t1.e3;Parent=MALK_00397.t1

contig001	AUGUSTUS	exon	733114	733132	.	-	.	ID=MALK_00397.t1.e2;Parent=MALK_00397.t1
contig001	AUGUSTUS	exon	732748	733025	.	-	.	ID=MALK_00397.t1.e1;Parent=MALK_00397.t1
contig001	AUGUSTUS	gene	733304	736339	0.88	+	.	ID=MALK_00398;prediction_source=augustus:contig001.g740.t1
contig001	AUGUSTUS	CDS	733304	736339	0.88	+	0	ID=MALK_00398.t1.c1;Parent=MALK_00398.t1
contig001	AUGUSTUS	mRNA	733304	736339	0.88	+	.	ID=MALK_00398.t1;Parent=MALK_00398
contig001	AUGUSTUS	exon	733304	736339	0.88	+	.	ID=MALK_00398.t1.e1;Parent=MALK_00398.t1
contig001	AUGUSTUS	gene	736326	738422	0.99	-	.	ID=MALK_00399;prediction_source=augustus:contig001.g741.t1
contig001	AUGUSTUS	CDS	736326	738422	0.99	-	0	ID=MALK_00399.t1.c1;Parent=MALK_00399.t1
contig001	AUGUSTUS	mRNA	736326	738422	0.99	-	.	ID=MALK_00399.t1;Parent=MALK_00399
contig001	AUGUSTUS	exon	736326	738422	0.99	-	.	ID=MALK_00399.t1.e1;Parent=MALK_00399.t1
contig001	AUGUSTUS	gene	738562	739314	0.97	+	.	ID=MALK_00400;prediction_source=augustus:contig001.g742.t1
contig001	AUGUSTUS	CDS	738562	739314	0.97	+	0	ID=MALK_00400.t1.c1;Parent=MALK_00400.t1
contig001	AUGUSTUS	mRNA	738562	739314	0.97	+	.	ID=MALK_00400.t1;Parent=MALK_00400
contig001	AUGUSTUS	exon	738562	739314	0.97	+	.	ID=MALK_00400.t1.e1;Parent=MALK_00400.t1
contig001	maker	gene	739316	739693	.	-	.	ID=MALK_00401;prediction_source=maker_MRET:augustus_masked-contig001-processed-gene-7.37-mRNA-1
contig001	maker	CDS	739316	739693	.	-	0	ID=MALK_00401.t1.c1;Parent=MALK_00401.t1
contig001	maker	mRNA	739316	739693	.	-	.	ID=MALK_00401.t1;Parent=MALK_00401
contig001	maker	exon	739316	739693	.	-	.	ID=MALK_00401.t1.e1;Parent=MALK_00401.t1
contig001	maker	gene	739889	742225	.	+	.	ID=MALK_00402;prediction_source=maker_MRET:augustus_masked-contig001-processed-gene-7.12-mRNA-1
contig001	maker	CDS	739889	742225	.	+	0	ID=MALK_00402.t1.c1;Parent=MALK_00402.t1
contig001	maker	mRNA	739889	742225	.	+	.	ID=MALK_00402.t1;Parent=MALK_00402
contig001	maker	exon	739889	742225	.	+	.	ID=MALK_00402.t1.e1;Parent=MALK_00402.t1
contig001	maker	gene	742691	745162	.	+	.	ID=MALK_00403;prediction_source=maker_MRET:augustus_masked-contig001-processed-gene-7.13-mRNA-1
contig001	maker	CDS	742691	745162	.	+	0	ID=MALK_00403.t1.c1;Parent=MALK_00403.t1
contig001	maker	mRNA	742691	745162	.	+	.	ID=MALK_00403.t1;Parent=MALK_00403
contig001	maker	exon	742691	745162	.	+	.	ID=MALK_00403.t1.e1;Parent=MALK_00403.t1
contig001	AUGUSTUS	gene	745404	746735	0.92	-	.	ID=MALK_00404;prediction_source=augustus:contig001.g749.t1
contig001	AUGUSTUS	CDS	745404	746735	0.92	-	0	ID=MALK_00404.t1.c1;Parent=MALK_00404.t1
contig001	AUGUSTUS	mRNA	745404	746735	0.92	-	.	ID=MALK_00404.t1;Parent=MALK_00404
contig001	AUGUSTUS	exon	745404	746735	0.92	-	.	ID=MALK_00404.t1.e1;Parent=MALK_00404.t1
contig001	AUGUSTUS	gene	746887	748468	0.92	+	.	ID=MALK_00405;prediction_source=braker_MRET:g1059.t1
contig001	AUGUSTUS	CDS	746887	746914	0.93	+	0	ID=MALK_00405.t1.c1;Parent=MALK_00405.t1
contig001	AUGUSTUS	CDS	746949	747003	0.93	+	0	ID=MALK_00405.t1.c2;Parent=MALK_00405.t1
contig001	AUGUSTUS	CDS	747031	748468	0.93	+	0	ID=MALK_00405.t1.c3;Parent=MALK_00405.t1
contig001	AUGUSTUS	mRNA	746887	748468	0.92	+	.	ID=MALK_00405.t1;Parent=MALK_00405
contig001	AUGUSTUS	exon	746887	746914	.	+	.	ID=MALK_00405.t1.e1;Parent=MALK_00405.t1
contig001	AUGUSTUS	exon	746949	747003	.	+	.	ID=MALK_00405.t1.e2;Parent=MALK_00405.t1
contig001	AUGUSTUS	exon	747031	748468	.	+	.	ID=MALK_00405.t1.e3;Parent=MALK_00405.t1
contig001	AUGUSTUS	gene	748563	750749	0.9	+	.	ID=MALK_00406;prediction_source=augustus:contig001.g751.t1
contig001	AUGUSTUS	CDS	748563	750749	0.9	+	0	ID=MALK_00406.t1.c1;Parent=MALK_00406.t1
contig001	AUGUSTUS	mRNA	748563	750749	0.9	+	.	ID=MALK_00406.t1;Parent=MALK_00406
contig001	AUGUSTUS	exon	748563	750749	0.9	+	.	ID=MALK_00406.t1.e1;Parent=MALK_00406.t1
contig001	AUGUSTUS	gene	750769	751713	1	-	.	ID=MALK_00407;prediction_source=braker_MRET:g1061.t1

contig001	AUGUSTUS	CDS	750769	751713	1	-	0	ID=MALK_00407.t1.c1;Parent=MALK_00407.t1
contig001	AUGUSTUS	mRNA	750769	751713	1	-	.	ID=MALK_00407.t1;Parent=MALK_00407
contig001	AUGUSTUS	exon	750769	751713	.	-	.	ID=MALK_00407.t1.e1;Parent=MALK_00407.t1
contig001	AUGUSTUS	gene	751886	752572	0.78	+	.	ID=MALK_00408;prediction_source=braker_MRET:g1062.t1
contig001	AUGUSTUS	CDS	751886	752572	0.78	+	0	ID=MALK_00408.t1.c1;Parent=MALK_00408.t1
contig001	AUGUSTUS	mRNA	751886	752572	0.78	+	.	ID=MALK_00408.t1;Parent=MALK_00408
contig001	AUGUSTUS	exon	751886	752572	.	+	.	ID=MALK_00408.t1.e1;Parent=MALK_00408.t1
contig001	maker	gene	752577	756125	.	-	.	ID=MALK_00409;prediction_source=maker_MRET:augustus_masked-contig001-processed-gene-7.40-mRNA-1
contig001	maker	CDS	752577	756125	.	-	0	ID=MALK_00409.t1.c1;Parent=MALK_00409.t1
contig001	maker	mRNA	752577	756125	.	-	.	ID=MALK_00409.t1;Parent=MALK_00409
contig001	maker	exon	752577	756125	.	-	.	ID=MALK_00409.t1.e1;Parent=MALK_00409.t1
contig001	AUGUSTUS	gene	756818	757439	1	+	.	ID=MALK_00410;prediction_source=braker_MRET:g1064.t1
contig001	AUGUSTUS	CDS	756818	756867	1	+	0	ID=MALK_00410.t1.c1;Parent=MALK_00410.t1
contig001	AUGUSTUS	CDS	756921	757043	1	+	0	ID=MALK_00410.t1.c2;Parent=MALK_00410.t1
contig001	AUGUSTUS	CDS	757148	757439	1	+	0	ID=MALK_00410.t1.c3;Parent=MALK_00410.t1
contig001	AUGUSTUS	mRNA	756818	757439	1	+	.	ID=MALK_00410.t1;Parent=MALK_00410
contig001	AUGUSTUS	exon	756818	756867	.	+	.	ID=MALK_00410.t1.e1;Parent=MALK_00410.t1
contig001	AUGUSTUS	exon	756921	757043	.	+	.	ID=MALK_00410.t1.e2;Parent=MALK_00410.t1
contig001	AUGUSTUS	exon	757148	757439	.	+	.	ID=MALK_00410.t1.e3;Parent=MALK_00410.t1
contig001	maker	gene	757947	758906	.	+	.	ID=MALK_00411;prediction_source=maker_MRET:augustus_masked-contig001-processed-gene-7.18-mRNA-1
contig001	maker	CDS	757947	758906	.	+	0	ID=MALK_00411.t1.c1;Parent=MALK_00411.t1
contig001	maker	mRNA	757947	758906	.	+	.	ID=MALK_00411.t1;Parent=MALK_00411
contig001	maker	exon	757947	758906	.	+	.	ID=MALK_00411.t1.e1;Parent=MALK_00411.t1
contig001	AUGUSTUS	gene	759169	760983	0.99	-	.	ID=MALK_00412;prediction_source=augustus:contig001.g760.t1
contig001	AUGUSTUS	CDS	759169	760983	0.99	-	0	ID=MALK_00412.t1.c1;Parent=MALK_00412.t1
contig001	AUGUSTUS	mRNA	759169	760983	0.99	-	.	ID=MALK_00412.t1;Parent=MALK_00412
contig001	AUGUSTUS	exon	759169	760983	0.99	-	.	ID=MALK_00412.t1.e1;Parent=MALK_00412.t1
contig001	maker	gene	761548	762426	.	-	.	ID=MALK_00413;prediction_source=maker_MRET:augustus_masked-contig001-processed-gene-7.42-mRNA-1
contig001	maker	CDS	761548	762426	.	-	0	ID=MALK_00413.t1.c1;Parent=MALK_00413.t1
contig001	maker	mRNA	761548	762426	.	-	.	ID=MALK_00413.t1;Parent=MALK_00413
contig001	maker	exon	761548	762426	.	-	.	ID=MALK_00413.t1.e1;Parent=MALK_00413.t1
contig001	maker	gene	762616	763554	.	+	.	ID=MALK_00414;prediction_source=maker_MRET:augustus_masked-contig001-processed-gene-7.19-mRNA-1
contig001	maker	CDS	762616	763554	.	+	0	ID=MALK_00414.t1.c1;Parent=MALK_00414.t1
contig001	maker	mRNA	762616	763554	.	+	.	ID=MALK_00414.t1;Parent=MALK_00414
contig001	maker	exon	762616	763554	.	+	.	ID=MALK_00414.t1.e1;Parent=MALK_00414.t1
contig001	maker	gene	763716	764357	.	-	.	ID=MALK_00415;prediction_source=maker_MRET:augustus_masked-contig001-processed-gene-7.43-mRNA-1
contig001	maker	CDS	763716	764357	.	-	0	ID=MALK_00415.t1.c1;Parent=MALK_00415.t1
contig001	maker	mRNA	763716	764357	.	-	.	ID=MALK_00415.t1;Parent=MALK_00415
contig001	maker	exon	763716	764357	.	-	.	ID=MALK_00415.t1.e1;Parent=MALK_00415.t1
contig001	AUGUSTUS	gene	764576	765243	0.28	-	.	ID=MALK_00416;prediction_source=braker_MRET:g1070.t1
contig001	AUGUSTUS	CDS	765219	765243	0.31	-	0	ID=MALK_00416.t1.c3;Parent=MALK_00416.t1
contig001	AUGUSTUS	CDS	765081	765179	0.31	-	0	ID=MALK_00416.t1.c2;Parent=MALK_00416.t1
contig001	AUGUSTUS	CDS	764576	765039	0.31	-	0	ID=MALK_00416.t1.c1;Parent=MALK_00416.t1

contig001	AUGUSTUS	mRNA	764576	765243	0.28	-	.	ID=MALK_00416.t1;Parent=MALK_00416
contig001	AUGUSTUS	exon	765219	765243	.	-	.	ID=MALK_00416.t1.e3;Parent=MALK_00416.t1
contig001	AUGUSTUS	exon	765081	765179	.	-	.	ID=MALK_00416.t1.e2;Parent=MALK_00416.t1
contig001	AUGUSTUS	exon	764576	765039	.	-	.	ID=MALK_00416.t1.e1;Parent=MALK_00416.t1
contig001	AUGUSTUS	gene	765331	767301	0.57	-	.	ID=MALK_00417;prediction_source=augustus:contig001.g764.t1
contig001	AUGUSTUS	CDS	765331	767301	0.57	-	0	ID=MALK_00417.t1.c1;Parent=MALK_00417.t1
contig001	AUGUSTUS	mRNA	765331	767301	0.57	-	.	ID=MALK_00417.t1;Parent=MALK_00417
contig001	AUGUSTUS	exon	765331	767301	0.57	-	.	ID=MALK_00417.t1.e1;Parent=MALK_00417.t1
contig001	maker	gene	767712	769694	.	+	.	ID=MALK_00418;prediction_source=maker_MRET:augustus_masked-contig001-processed-gene-7.20-mRNA-1
contig001	maker	CDS	767712	769694	.	+	0	ID=MALK_00418.t1.c1;Parent=MALK_00418.t1
contig001	maker	mRNA	767712	769694	.	+	.	ID=MALK_00418.t1;Parent=MALK_00418
contig001	maker	exon	767712	769694	.	+	.	ID=MALK_00418.t1.e1;Parent=MALK_00418.t1
contig001	AUGUSTUS	gene	769697	772390	0.89	-	.	ID=MALK_00419;prediction_source=augustus:contig001.g766.t1
contig001	AUGUSTUS	CDS	769697	772390	0.89	-	0	ID=MALK_00419.t1.c1;Parent=MALK_00419.t1
contig001	AUGUSTUS	mRNA	769697	772390	0.89	-	.	ID=MALK_00419.t1;Parent=MALK_00419
contig001	AUGUSTUS	exon	769697	772390	0.89	-	.	ID=MALK_00419.t1.e1;Parent=MALK_00419.t1
contig001	AUGUSTUS	gene	772800	773471	0.97	+	.	ID=MALK_00420;prediction_source=braker_MRET:g1074.t1
contig001	AUGUSTUS	CDS	772800	773471	0.97	+	0	ID=MALK_00420.t1.c1;Parent=MALK_00420.t1
contig001	AUGUSTUS	mRNA	772800	773471	0.97	+	.	ID=MALK_00420.t1;Parent=MALK_00420
contig001	AUGUSTUS	exon	772800	773471	.	+	.	ID=MALK_00420.t1.e1;Parent=MALK_00420.t1
contig001	maker	gene	773490	774353	.	-	.	ID=MALK_00421;prediction_source=maker_MRET:augustus_masked-contig001-processed-gene-7.47-mRNA-1
contig001	maker	CDS	773490	774353	.	-	0	ID=MALK_00421.t1.c1;Parent=MALK_00421.t1
contig001	maker	mRNA	773490	774353	.	-	.	ID=MALK_00421.t1;Parent=MALK_00421
contig001	maker	exon	773490	774353	.	-	.	ID=MALK_00421.t1.e1;Parent=MALK_00421.t1
contig001	AUGUSTUS	gene	774592	775082	0.52	+	.	ID=MALK_00422;prediction_source=braker_MRET:g1076.t1
contig001	AUGUSTUS	CDS	774592	774993	1	+	0	ID=MALK_00422.t1.c1;Parent=MALK_00422.t1
contig001	AUGUSTUS	CDS	775023	775082	1	+	0	ID=MALK_00422.t1.c2;Parent=MALK_00422.t1
contig001	AUGUSTUS	mRNA	774592	775082	0.52	+	.	ID=MALK_00422.t1;Parent=MALK_00422
contig001	AUGUSTUS	exon	774592	774993	.	+	.	ID=MALK_00422.t1.e1;Parent=MALK_00422.t1
contig001	AUGUSTUS	exon	775023	775082	.	+	.	ID=MALK_00422.t1.e2;Parent=MALK_00422.t1
contig001	AUGUSTUS	gene	775792	776250	0.39	-	.	ID=MALK_00423;prediction_source=augustus:contig001.g770.t1
contig001	AUGUSTUS	CDS	775792	776250	0.39	-	0	ID=MALK_00423.t1.c1;Parent=MALK_00423.t1
contig001	AUGUSTUS	mRNA	775792	776250	0.39	-	.	ID=MALK_00423.t1;Parent=MALK_00423
contig001	AUGUSTUS	exon	775792	776250	0.39	-	.	ID=MALK_00423.t1.e1;Parent=MALK_00423.t1
contig001	AUGUSTUS	gene	776225	776759	0.23	+	.	ID=MALK_00424;prediction_source=augustus:contig001.g771.t1
contig001	AUGUSTUS	CDS	776225	776260	0.63	+	0	ID=MALK_00424.t1.c1;Parent=MALK_00424.t1
contig001	AUGUSTUS	CDS	776379	776759	0.63	+	0	ID=MALK_00424.t1.c2;Parent=MALK_00424.t1
contig001	AUGUSTUS	mRNA	776225	776759	0.23	+	.	ID=MALK_00424.t1;Parent=MALK_00424
contig001	AUGUSTUS	exon	776225	776260	0.63	+	.	ID=MALK_00424.t1.e1;Parent=MALK_00424.t1
contig001	AUGUSTUS	exon	776379	776759	0.63	+	.	ID=MALK_00424.t1.e2;Parent=MALK_00424.t1
contig001	maker	gene	776765	778426	.	-	.	ID=MALK_00425;prediction_source=maker_MRET:augustus_masked-contig001-processed-gene-7.48-mRNA-1
contig001	maker	CDS	776765	778426	.	-	0	ID=MALK_00425.t1.c1;Parent=MALK_00425.t1
contig001	maker	mRNA	776765	778426	.	-	.	ID=MALK_00425.t1;Parent=MALK_00425

contig001	maker	exon	776765	778426	.	-	.	ID=MALK_00425.t1.e1;Parent=MALK_00425.t1
contig001	AUGUSTUS	gene	778396	780984	0.99	+	.	ID=MALK_00426;prediction_source=augustus:contig001.g773.t1
contig001	AUGUSTUS	CDS	778396	780984	0.99	+	0	ID=MALK_00426.t1.c1;Parent=MALK_00426.t1
contig001	AUGUSTUS	mRNA	778396	780984	0.99	+	.	ID=MALK_00426.t1;Parent=MALK_00426
contig001	AUGUSTUS	exon	778396	780984	0.99	+	.	ID=MALK_00426.t1.e1;Parent=MALK_00426.t1
contig001	AUGUSTUS	gene	781000	782898	0.71	-	.	ID=MALK_00427;prediction_source=augustus:contig001.g774.t1
contig001	AUGUSTUS	CDS	781000	782898	0.71	-	0	ID=MALK_00427.t1.c1;Parent=MALK_00427.t1
contig001	AUGUSTUS	mRNA	781000	782898	0.71	-	.	ID=MALK_00427.t1;Parent=MALK_00427
contig001	AUGUSTUS	exon	781000	782898	0.71	-	.	ID=MALK_00427.t1.e1;Parent=MALK_00427.t1
contig001	maker	gene	783053	784690	.	+	.	ID=MALK_00428;prediction_source=maker_MRET:augustus_masked-contig001-processed-gene-7.25-mRNA-1
contig001	maker	CDS	783053	784690	.	+	0	ID=MALK_00428.t1.c1;Parent=MALK_00428.t1
contig001	maker	mRNA	783053	784690	.	+	.	ID=MALK_00428.t1;Parent=MALK_00428
contig001	maker	exon	783053	784690	.	+	.	ID=MALK_00428.t1.e1;Parent=MALK_00428.t1
contig001	AUGUSTUS	gene	784665	785120	0.77	-	.	ID=MALK_00429;prediction_source=braker_MRET:g1083.t1
contig001	AUGUSTUS	CDS	784665	785120	0.77	-	0	ID=MALK_00429.t1.c1;Parent=MALK_00429.t1
contig001	AUGUSTUS	mRNA	784665	785120	0.77	-	.	ID=MALK_00429.t1;Parent=MALK_00429
contig001	AUGUSTUS	exon	784665	785120	.	-	.	ID=MALK_00429.t1.e1;Parent=MALK_00429.t1
contig001	maker	gene	785142	788341	.	+	.	ID=MALK_00430;prediction_source=maker_MRET:augustus_masked-contig001-processed-gene-7.26-mRNA-1
contig001	maker	CDS	785142	785159	.	+	0	ID=MALK_00430.t1.c1;Parent=MALK_00430.t1
contig001	maker	CDS	785251	788173	.	+	0	ID=MALK_00430.t1.c2;Parent=MALK_00430.t1
contig001	maker	CDS	788265	788341	.	+	0	ID=MALK_00430.t1.c3;Parent=MALK_00430.t1
contig001	maker	mRNA	785142	788341	.	+	.	ID=MALK_00430.t1;Parent=MALK_00430
contig001	maker	exon	785142	785159	.	+	.	ID=MALK_00430.t1.e1;Parent=MALK_00430.t1
contig001	maker	exon	785251	788173	.	+	.	ID=MALK_00430.t1.e2;Parent=MALK_00430.t1
contig001	maker	exon	788265	788341	.	+	.	ID=MALK_00430.t1.e3;Parent=MALK_00430.t1
contig001	AUGUSTUS	gene	788383	790050	0.55	-	.	ID=MALK_00431;prediction_source=augustus:contig001.g779.t1
contig001	AUGUSTUS	CDS	788383	790050	0.55	-	0	ID=MALK_00431.t1.c1;Parent=MALK_00431.t1
contig001	AUGUSTUS	mRNA	788383	790050	0.55	-	.	ID=MALK_00431.t1;Parent=MALK_00431
contig001	AUGUSTUS	exon	788383	790050	0.55	-	.	ID=MALK_00431.t1.e1;Parent=MALK_00431.t1
contig001	AUGUSTUS	gene	790254	791471	0.75	+	.	ID=MALK_00432;prediction_source=augustus:contig001.g780.t1
contig001	AUGUSTUS	CDS	790254	791471	0.75	+	0	ID=MALK_00432.t1.c1;Parent=MALK_00432.t1
contig001	AUGUSTUS	mRNA	790254	791471	0.75	+	.	ID=MALK_00432.t1;Parent=MALK_00432
contig001	AUGUSTUS	exon	790254	791471	0.75	+	.	ID=MALK_00432.t1.e1;Parent=MALK_00432.t1
contig001	maker	gene	791468	792685	.	-	.	ID=MALK_00433;prediction_source=maker_MRET:augustus_masked-contig001-processed-gene-7.51-mRNA-1
contig001	maker	CDS	791468	792685	.	-	0	ID=MALK_00433.t1.c1;Parent=MALK_00433.t1
contig001	maker	mRNA	791468	792685	.	-	.	ID=MALK_00433.t1;Parent=MALK_00433
contig001	maker	exon	791468	792685	.	-	.	ID=MALK_00433.t1.e1;Parent=MALK_00433.t1
contig001	AUGUSTUS	gene	792852	795155	0.65	+	.	ID=MALK_00434;prediction_source=augustus:contig001.g782.t1
contig001	AUGUSTUS	CDS	792852	795155	0.65	+	0	ID=MALK_00434.t1.c1;Parent=MALK_00434.t1
contig001	AUGUSTUS	mRNA	792852	795155	0.65	+	.	ID=MALK_00434.t1;Parent=MALK_00434
contig001	AUGUSTUS	exon	792852	795155	0.65	+	.	ID=MALK_00434.t1.e1;Parent=MALK_00434.t1
contig001	maker	gene	795152	796991	.	-	.	ID=MALK_00435;prediction_source=maker_MRET:augustus_masked-contig001-processed-gene-8.55-mRNA-1
contig001	maker	CDS	796919	796991	.	-	0	ID=MALK_00435.t1.c1;Parent=MALK_00435.t1

contig001	maker	CDS	795152	796776	.	-	0	ID=MALK_00435.t1.c2;Parent=MALK_00435.t1
contig001	maker	mRNA	795152	796991	.	-	.	ID=MALK_00435.t1;Parent=MALK_00435
contig001	maker	exon	796919	796991	.	-	.	ID=MALK_00435.t1.e1;Parent=MALK_00435.t1
contig001	maker	exon	795152	796776	.	-	.	ID=MALK_00435.t1.e2;Parent=MALK_00435.t1
contig001	maker	gene	797877	798779	.	-	.	ID=MALK_00436;prediction_source=maker_MRET:augustus_masked-contig001-processed-gene-8.56-mRNA-1
contig001	maker	CDS	797877	798779	.	-	0	ID=MALK_00436.t1.c1;Parent=MALK_00436.t1
contig001	maker	mRNA	797877	798779	.	-	.	ID=MALK_00436.t1;Parent=MALK_00436
contig001	maker	exon	797877	798779	.	-	.	ID=MALK_00436.t1.e1;Parent=MALK_00436.t1
contig001	AUGUSTUS	gene	798860	802225	0.96	-	.	ID=MALK_00437;prediction_source=augustus:contig001.g786.t1
contig001	AUGUSTUS	CDS	798860	802225	0.96	-	0	ID=MALK_00437.t1.c1;Parent=MALK_00437.t1
contig001	AUGUSTUS	mRNA	798860	802225	0.96	-	.	ID=MALK_00437.t1;Parent=MALK_00437
contig001	AUGUSTUS	exon	798860	802225	0.96	-	.	ID=MALK_00437.t1.e1;Parent=MALK_00437.t1
contig001	AUGUSTUS	gene	802512	805121	0.64	+	.	ID=MALK_00438;prediction_source=braker_MRET:g1092.t1
contig001	AUGUSTUS	CDS	802512	802557	0.68	+	0	ID=MALK_00438.t1.c1;Parent=MALK_00438.t1
contig001	AUGUSTUS	CDS	802597	805121	0.68	+	0	ID=MALK_00438.t1.c2;Parent=MALK_00438.t1
contig001	AUGUSTUS	mRNA	802512	805121	0.64	+	.	ID=MALK_00438.t1;Parent=MALK_00438
contig001	AUGUSTUS	exon	802512	802557	.	+	.	ID=MALK_00438.t1.e1;Parent=MALK_00438.t1
contig001	AUGUSTUS	exon	802597	805121	.	+	.	ID=MALK_00438.t1.e2;Parent=MALK_00438.t1
contig001	maker	gene	805132	806484	.	-	.	ID=MALK_00439;prediction_source=maker_MRET:augustus_masked-contig001-processed-gene-8.82-mRNA-1
contig001	maker	CDS	805132	806484	.	-	0	ID=MALK_00439.t1.c1;Parent=MALK_00439.t1
contig001	maker	mRNA	805132	806484	.	-	.	ID=MALK_00439.t1;Parent=MALK_00439
contig001	maker	exon	805132	806484	.	-	.	ID=MALK_00439.t1.e1;Parent=MALK_00439.t1
contig001	maker	gene	807017	807244	.	-	.	ID=MALK_00440;prediction_source=maker_MRET:augustus_masked-contig001-processed-gene-8.83-mRNA-1
contig001	maker	CDS	807017	807244	.	-	0	ID=MALK_00440.t1.c1;Parent=MALK_00440.t1
contig001	maker	mRNA	807017	807244	.	-	.	ID=MALK_00440.t1;Parent=MALK_00440
contig001	maker	exon	807017	807244	.	-	.	ID=MALK_00440.t1.e1;Parent=MALK_00440.t1
contig001	AUGUSTUS	gene	807728	810883	0.87	-	.	ID=MALK_00441;prediction_source=augustus:contig001.g792.t1
contig001	AUGUSTUS	CDS	807728	810883	0.87	-	0	ID=MALK_00441.t1.c1;Parent=MALK_00441.t1
contig001	AUGUSTUS	mRNA	807728	810883	0.87	-	.	ID=MALK_00441.t1;Parent=MALK_00441
contig001	AUGUSTUS	exon	807728	810883	0.87	-	.	ID=MALK_00441.t1.e1;Parent=MALK_00441.t1
contig001	AUGUSTUS	gene	811052	812487	0.53	-	.	ID=MALK_00442;prediction_source=braker_MRET:g1095.t1
contig001	AUGUSTUS	CDS	812135	812487	1	-	0	ID=MALK_00442.t1.c2;Parent=MALK_00442.t1
contig001	AUGUSTUS	CDS	811052	812105	1	-	0	ID=MALK_00442.t1.c1;Parent=MALK_00442.t1
contig001	AUGUSTUS	mRNA	811052	812487	0.53	-	.	ID=MALK_00442.t1;Parent=MALK_00442
contig001	AUGUSTUS	exon	812135	812487	.	-	.	ID=MALK_00442.t1.e2;Parent=MALK_00442.t1
contig001	AUGUSTUS	exon	811052	812105	.	-	.	ID=MALK_00442.t1.e1;Parent=MALK_00442.t1
contig001	AUGUSTUS	gene	812761	814218	0.99	+	.	ID=MALK_00443;prediction_source=augustus:contig001.g796.t1
contig001	AUGUSTUS	CDS	812761	814218	0.99	+	0	ID=MALK_00443.t1.c1;Parent=MALK_00443.t1
contig001	AUGUSTUS	mRNA	812761	814218	0.99	+	.	ID=MALK_00443.t1;Parent=MALK_00443
contig001	AUGUSTUS	exon	812761	814218	0.99	+	.	ID=MALK_00443.t1.e1;Parent=MALK_00443.t1
contig001	AUGUSTUS	gene	814231	815829	1	-	.	ID=MALK_00444;prediction_source=augustus:contig001.g798.t1
contig001	AUGUSTUS	CDS	814231	815829	1	-	0	ID=MALK_00444.t1.c1;Parent=MALK_00444.t1
contig001	AUGUSTUS	mRNA	814231	815829	1	-	.	ID=MALK_00444.t1;Parent=MALK_00444

contig001	AUGUSTUS	exon	814231	815829	1	-	.	ID=MALK_00444.t1.e1;Parent=MALK_00444.t1
contig001	AUGUSTUS	gene	816017	818608	1	+	.	ID=MALK_00445;prediction_source=augustus:contig001.g799.t1
contig001	AUGUSTUS	CDS	816017	818608	1	+	0	ID=MALK_00445.t1.c1;Parent=MALK_00445.t1
contig001	AUGUSTUS	mRNA	816017	818608	1	+	.	ID=MALK_00445.t1;Parent=MALK_00445
contig001	AUGUSTUS	exon	816017	818608	1	+	.	ID=MALK_00445.t1.e1;Parent=MALK_00445.t1
contig001	AUGUSTUS	gene	818639	819394	0.99	+	.	ID=MALK_00446;prediction_source=braker_MRET:g1099.t1
contig001	AUGUSTUS	CDS	818639	819394	0.99	+	0	ID=MALK_00446.t1.c1;Parent=MALK_00446.t1
contig001	AUGUSTUS	mRNA	818639	819394	0.99	+	.	ID=MALK_00446.t1;Parent=MALK_00446
contig001	AUGUSTUS	exon	818639	819394	.	+	.	ID=MALK_00446.t1.e1;Parent=MALK_00446.t1
contig001	maker	gene	819402	821411	.	-	.	ID=MALK_00447;prediction_source=maker_MRET:augustus_masked-contig001-processed-gene-8.87-mRNA-1
contig001	maker	CDS	819402	821411	.	-	0	ID=MALK_00447.t1.c1;Parent=MALK_00447.t1
contig001	maker	mRNA	819402	821411	.	-	.	ID=MALK_00447.t1;Parent=MALK_00447
contig001	maker	exon	819402	821411	.	-	.	ID=MALK_00447.t1.e1;Parent=MALK_00447.t1
contig001	AUGUSTUS	gene	821570	822643	0.63	+	.	ID=MALK_00448;prediction_source=augustus:contig001.g802.t1
contig001	AUGUSTUS	CDS	821570	822643	0.63	+	0	ID=MALK_00448.t1.c1;Parent=MALK_00448.t1
contig001	AUGUSTUS	mRNA	821570	822643	0.63	+	.	ID=MALK_00448.t1;Parent=MALK_00448
contig001	AUGUSTUS	exon	821570	822643	0.63	+	.	ID=MALK_00448.t1.e1;Parent=MALK_00448.t1
contig001	AUGUSTUS	gene	822668	824941	0.8	-	.	ID=MALK_00449;prediction_source=augustus:contig001.g803.t1
contig001	AUGUSTUS	CDS	822668	824941	0.8	-	0	ID=MALK_00449.t1.c1;Parent=MALK_00449.t1
contig001	AUGUSTUS	mRNA	822668	824941	0.8	-	.	ID=MALK_00449.t1;Parent=MALK_00449
contig001	AUGUSTUS	exon	822668	824941	0.8	-	.	ID=MALK_00449.t1.e1;Parent=MALK_00449.t1
contig001	AUGUSTUS	gene	825203	825905	0.88	-	.	ID=MALK_00450;prediction_source=braker_MRET:g1103.t1
contig001	AUGUSTUS	CDS	825883	825905	0.96	-	0	ID=MALK_00450.t1.c3;Parent=MALK_00450.t1
contig001	AUGUSTUS	CDS	825797	825809	0.96	-	0	ID=MALK_00450.t1.c2;Parent=MALK_00450.t1
contig001	AUGUSTUS	CDS	825203	825694	0.96	-	0	ID=MALK_00450.t1.c1;Parent=MALK_00450.t1
contig001	AUGUSTUS	mRNA	825203	825905	0.88	-	.	ID=MALK_00450.t1;Parent=MALK_00450
contig001	AUGUSTUS	exon	825883	825905	.	-	.	ID=MALK_00450.t1.e3;Parent=MALK_00450.t1
contig001	AUGUSTUS	exon	825797	825809	.	-	.	ID=MALK_00450.t1.e2;Parent=MALK_00450.t1
contig001	AUGUSTUS	exon	825203	825694	.	-	.	ID=MALK_00450.t1.e1;Parent=MALK_00450.t1
contig001	maker	gene	826418	828151	.	+	.	ID=MALK_00451;prediction_source=maker_MRET:augustus_masked-contig001-processed-gene-8.63-mRNA-1
contig001	maker	CDS	826418	828151	.	+	0	ID=MALK_00451.t1.c1;Parent=MALK_00451.t1
contig001	maker	mRNA	826418	828151	.	+	.	ID=MALK_00451.t1;Parent=MALK_00451
contig001	maker	exon	826418	828151	.	+	.	ID=MALK_00451.t1.e1;Parent=MALK_00451.t1
contig001	AUGUSTUS	gene	828418	830394	0.79	+	.	ID=MALK_00452;prediction_source=augustus:contig001.g808.t1
contig001	AUGUSTUS	CDS	828418	830394	0.79	+	0	ID=MALK_00452.t1.c1;Parent=MALK_00452.t1
contig001	AUGUSTUS	mRNA	828418	830394	0.79	+	.	ID=MALK_00452.t1;Parent=MALK_00452
contig001	AUGUSTUS	exon	828418	830394	0.79	+	.	ID=MALK_00452.t1.e1;Parent=MALK_00452.t1
contig001	maker	gene	830631	831476	.	+	.	ID=MALK_00453;prediction_source=maker_MRET:augustus_masked-contig001-processed-gene-8.65-mRNA-1
contig001	maker	CDS	830631	831476	.	+	0	ID=MALK_00453.t1.c1;Parent=MALK_00453.t1
contig001	maker	mRNA	830631	831476	.	+	.	ID=MALK_00453.t1;Parent=MALK_00453
contig001	maker	exon	830631	831476	.	+	.	ID=MALK_00453.t1.e1;Parent=MALK_00453.t1
contig001	AUGUSTUS	gene	831514	832749	0.78	+	.	ID=MALK_00454;prediction_source=augustus:contig001.g811.t1
contig001	AUGUSTUS	CDS	831514	832749	0.78	+	0	ID=MALK_00454.t1.c1;Parent=MALK_00454.t1

contig001	AUGUSTUS	mRNA	831514	832749	0.78	+	.	ID=MALK_00454.t1;Parent=MALK_00454
contig001	AUGUSTUS	exon	831514	832749	0.78	+	.	ID=MALK_00454.t1.e1;Parent=MALK_00454.t1
contig001	AUGUSTUS	gene	832797	833442	0.46	-	.	ID=MALK_00455;prediction_source=braker_MRET:g1107.t1
contig001	AUGUSTUS	CDS	833440	833442	1	-	0	ID=MALK_00455.t1.c3;Parent=MALK_00455.t1
contig001	AUGUSTUS	CDS	833272	833407	1	-	0	ID=MALK_00455.t1.c2;Parent=MALK_00455.t1
contig001	AUGUSTUS	CDS	832797	833239	1	-	0	ID=MALK_00455.t1.c1;Parent=MALK_00455.t1
contig001	AUGUSTUS	mRNA	832797	833442	0.46	-	.	ID=MALK_00455.t1;Parent=MALK_00455
contig001	AUGUSTUS	exon	833440	833442	.	-	.	ID=MALK_00455.t1.e3;Parent=MALK_00455.t1
contig001	AUGUSTUS	exon	833272	833407	.	-	.	ID=MALK_00455.t1.e2;Parent=MALK_00455.t1
contig001	AUGUSTUS	exon	832797	833239	.	-	.	ID=MALK_00455.t1.e1;Parent=MALK_00455.t1
contig001	AUGUSTUS	gene	833567	835018	0.58	+	.	ID=MALK_00456;prediction_source=augustus:contig001.g814.t1
contig001	AUGUSTUS	CDS	833567	835018	0.58	+	0	ID=MALK_00456.t1.c1;Parent=MALK_00456.t1
contig001	AUGUSTUS	mRNA	833567	835018	0.58	+	.	ID=MALK_00456.t1;Parent=MALK_00456
contig001	AUGUSTUS	exon	833567	835018	0.58	+	.	ID=MALK_00456.t1.e1;Parent=MALK_00456.t1
contig001	maker	gene	835022	835261	.	-	.	ID=MALK_00457;prediction_source=maker_MRET:augustus_masked-contig001-processed-gene-8.91-mRNA-1
contig001	maker	CDS	835022	835261	.	-	0	ID=MALK_00457.t1.c1;Parent=MALK_00457.t1
contig001	maker	mRNA	835022	835261	.	-	.	ID=MALK_00457.t1;Parent=MALK_00457
contig001	maker	exon	835022	835261	.	-	.	ID=MALK_00457.t1.e1;Parent=MALK_00457.t1
contig001	AUGUSTUS	gene	835338	837971	0.75	-	.	ID=MALK_00458;prediction_source=augustus:contig001.g817.t1
contig001	AUGUSTUS	CDS	835338	837971	0.75	-	0	ID=MALK_00458.t1.c1;Parent=MALK_00458.t1
contig001	AUGUSTUS	mRNA	835338	837971	0.75	-	.	ID=MALK_00458.t1;Parent=MALK_00458
contig001	AUGUSTUS	exon	835338	837971	0.75	-	.	ID=MALK_00458.t1.e1;Parent=MALK_00458.t1
contig001	maker	gene	838468	841074	.	+	.	ID=MALK_00459;prediction_source=maker_MRET:augustus_masked-contig001-processed-gene-8.68-mRNA-1
contig001	maker	CDS	838468	841074	.	+	0	ID=MALK_00459.t1.c1;Parent=MALK_00459.t1
contig001	maker	mRNA	838468	841074	.	+	.	ID=MALK_00459.t1;Parent=MALK_00459
contig001	maker	exon	838468	841074	.	+	.	ID=MALK_00459.t1.e1;Parent=MALK_00459.t1
contig001	AUGUSTUS	gene	841077	843426	0.72	-	.	ID=MALK_00460;prediction_source=braker_MRET:g1112.t1
contig001	AUGUSTUS	CDS	843370	843426	0.72	-	0	ID=MALK_00460.t1.c2;Parent=MALK_00460.t1
contig001	AUGUSTUS	CDS	841077	843341	0.72	-	0	ID=MALK_00460.t1.c1;Parent=MALK_00460.t1
contig001	AUGUSTUS	mRNA	841077	843426	0.72	-	.	ID=MALK_00460.t1;Parent=MALK_00460
contig001	AUGUSTUS	exon	843370	843426	.	-	.	ID=MALK_00460.t1.e2;Parent=MALK_00460.t1
contig001	AUGUSTUS	exon	841077	843341	.	-	.	ID=MALK_00460.t1.e1;Parent=MALK_00460.t1
contig001	AUGUSTUS	gene	843718	846219	0.81	+	.	ID=MALK_00461;prediction_source=augustus:contig001.g821.t1
contig001	AUGUSTUS	CDS	843718	846219	0.81	+	0	ID=MALK_00461.t1.c1;Parent=MALK_00461.t1
contig001	AUGUSTUS	mRNA	843718	846219	0.81	+	.	ID=MALK_00461.t1;Parent=MALK_00461
contig001	AUGUSTUS	exon	843718	846219	0.81	+	.	ID=MALK_00461.t1.e1;Parent=MALK_00461.t1
contig001	AUGUSTUS	gene	846351	849605	0.74	+	.	ID=MALK_00462;prediction_source=augustus:contig001.g823.t1
contig001	AUGUSTUS	CDS	846351	849605	0.74	+	0	ID=MALK_00462.t1.c1;Parent=MALK_00462.t1
contig001	AUGUSTUS	mRNA	846351	849605	0.74	+	.	ID=MALK_00462.t1;Parent=MALK_00462
contig001	AUGUSTUS	exon	846351	849605	0.74	+	.	ID=MALK_00462.t1.e1;Parent=MALK_00462.t1
contig001	AUGUSTUS	gene	849630	850937	0.23	-	.	ID=MALK_00463;prediction_source=augustus:contig001.g824.t1
contig001	AUGUSTUS	CDS	849630	850937	0.23	-	0	ID=MALK_00463.t1.c1;Parent=MALK_00463.t1
contig001	AUGUSTUS	mRNA	849630	850937	0.23	-	.	ID=MALK_00463.t1;Parent=MALK_00463

contig001	AUGUSTUS	exon	849630	850937	0.23	-	.	ID=MALK_00463.t1.e1;Parent=MALK_00463.t1
contig001	AUGUSTUS	gene	851023	853077	0.49	-	.	ID=MALK_00464;prediction_source=augustus:contig001.g825.t1
contig001	AUGUSTUS	CDS	851023	853077	0.49	-	0	ID=MALK_00464.t1.c1;Parent=MALK_00464.t1
contig001	AUGUSTUS	mRNA	851023	853077	0.49	-	.	ID=MALK_00464.t1;Parent=MALK_00464
contig001	AUGUSTUS	exon	851023	853077	0.49	-	.	ID=MALK_00464.t1.e1;Parent=MALK_00464.t1
contig001	AUGUSTUS	gene	853273	854403	0.71	+	.	ID=MALK_00465;prediction_source=braker_MRET:g1117.t1
contig001	AUGUSTUS	CDS	853273	854403	0.71	+	0	ID=MALK_00465.t1.c1;Parent=MALK_00465.t1
contig001	AUGUSTUS	mRNA	853273	854403	0.71	+	.	ID=MALK_00465.t1;Parent=MALK_00465
contig001	AUGUSTUS	exon	853273	854403	.	+	.	ID=MALK_00465.t1.e1;Parent=MALK_00465.t1
contig001	AUGUSTUS	gene	854469	855380	0.42	+	.	ID=MALK_00466;prediction_source=braker_MRET:g1118.t1
contig001	AUGUSTUS	CDS	854469	855380	0.42	+	0	ID=MALK_00466.t1.c1;Parent=MALK_00466.t1
contig001	AUGUSTUS	mRNA	854469	855380	0.42	+	.	ID=MALK_00466.t1;Parent=MALK_00466
contig001	AUGUSTUS	exon	854469	855380	.	+	.	ID=MALK_00466.t1.e1;Parent=MALK_00466.t1
contig001	maker	gene	855450	857588	.	+	.	ID=MALK_00467;prediction_source=maker_MRET:augustus_masked-contig001-processed-gene-8.72-mRNA-1
contig001	maker	CDS	855450	857588	.	+	0	ID=MALK_00467.t1.c1;Parent=MALK_00467.t1
contig001	maker	mRNA	855450	857588	.	+	.	ID=MALK_00467.t1;Parent=MALK_00467
contig001	maker	exon	855450	857588	.	+	.	ID=MALK_00467.t1.e1;Parent=MALK_00467.t1
contig001	AUGUSTUS	gene	857581	859527	0.63	-	.	ID=MALK_00468;prediction_source=braker_MRET:g1120.t1
contig001	AUGUSTUS	CDS	857581	859527	0.63	-	0	ID=MALK_00468.t1.c1;Parent=MALK_00468.t1
contig001	AUGUSTUS	mRNA	857581	859527	0.63	-	.	ID=MALK_00468.t1;Parent=MALK_00468
contig001	AUGUSTUS	exon	857581	859527	.	-	.	ID=MALK_00468.t1.e1;Parent=MALK_00468.t1
contig001	AUGUSTUS	gene	859589	860470	0.99	-	.	ID=MALK_00469;prediction_source=braker_MRET:g1121.t1
contig001	AUGUSTUS	CDS	859589	860470	0.99	-	0	ID=MALK_00469.t1.c1;Parent=MALK_00469.t1
contig001	AUGUSTUS	mRNA	859589	860470	0.99	-	.	ID=MALK_00469.t1;Parent=MALK_00469
contig001	AUGUSTUS	exon	859589	860470	.	-	.	ID=MALK_00469.t1.e1;Parent=MALK_00469.t1
contig001	AUGUSTUS	gene	860617	861811	0.68	+	.	ID=MALK_00470;prediction_source=braker_MRET:g1122.t1
contig001	AUGUSTUS	CDS	860617	860633	0.99	+	0	ID=MALK_00470.t1.c1;Parent=MALK_00470.t1
contig001	AUGUSTUS	CDS	860677	860880	0.99	+	0	ID=MALK_00470.t1.c2;Parent=MALK_00470.t1
contig001	AUGUSTUS	CDS	861043	861058	0.99	+	0	ID=MALK_00470.t1.c3;Parent=MALK_00470.t1
contig001	AUGUSTUS	CDS	861155	861198	0.99	+	0	ID=MALK_00470.t1.c4;Parent=MALK_00470.t1
contig001	AUGUSTUS	CDS	861274	861310	0.99	+	0	ID=MALK_00470.t1.c5;Parent=MALK_00470.t1
contig001	AUGUSTUS	CDS	861431	861811	0.99	+	0	ID=MALK_00470.t1.c6;Parent=MALK_00470.t1
contig001	AUGUSTUS	mRNA	860617	861811	0.68	+	.	ID=MALK_00470.t1;Parent=MALK_00470
contig001	AUGUSTUS	exon	860617	860633	.	+	.	ID=MALK_00470.t1.e1;Parent=MALK_00470.t1
contig001	AUGUSTUS	exon	860677	860880	.	+	.	ID=MALK_00470.t1.e2;Parent=MALK_00470.t1
contig001	AUGUSTUS	exon	861043	861058	.	+	.	ID=MALK_00470.t1.e3;Parent=MALK_00470.t1
contig001	AUGUSTUS	exon	861155	861198	.	+	.	ID=MALK_00470.t1.e4;Parent=MALK_00470.t1
contig001	AUGUSTUS	exon	861274	861310	.	+	.	ID=MALK_00470.t1.e5;Parent=MALK_00470.t1
contig001	AUGUSTUS	exon	861431	861811	.	+	.	ID=MALK_00470.t1.e6;Parent=MALK_00470.t1
contig001	AUGUSTUS	gene	862064	863905	0.74	+	.	ID=MALK_00471;prediction_source=augustus:contig001.g830.t1
contig001	AUGUSTUS	CDS	862064	863905	0.74	+	0	ID=MALK_00471.t1.c1;Parent=MALK_00471.t1
contig001	AUGUSTUS	mRNA	862064	863905	0.74	+	.	ID=MALK_00471.t1;Parent=MALK_00471
contig001	AUGUSTUS	exon	862064	863905	0.74	+	.	ID=MALK_00471.t1.e1;Parent=MALK_00471.t1

contig001	AUGUSTUS	gene	863939	865699	1	-	.	ID=MALK_00472;prediction_source=braker_MRET:g1124.t1
contig001	AUGUSTUS	CDS	863939	865699	1	-	0	ID=MALK_00472.t1.c1;Parent=MALK_00472.t1
contig001	AUGUSTUS	mRNA	863939	865699	1	-	.	ID=MALK_00472.t1;Parent=MALK_00472
contig001	AUGUSTUS	exon	863939	865699	.	-	.	ID=MALK_00472.t1.e1;Parent=MALK_00472.t1
contig001	maker	gene	865711	866724	.	+	.	ID=MALK_00473;prediction_source=maker_MRET:augustus_masked-contig001-processed-gene-8.75-mRNA-1
contig001	maker	CDS	865711	866724	.	+	0	ID=MALK_00473.t1.c1;Parent=MALK_00473.t1
contig001	maker	mRNA	865711	866724	.	+	.	ID=MALK_00473.t1;Parent=MALK_00473
contig001	maker	exon	865711	866724	.	+	.	ID=MALK_00473.t1.e1;Parent=MALK_00473.t1
contig001	AUGUSTUS	gene	866823	868331	0.99	-	.	ID=MALK_00474;prediction_source=augustus:contig001.g833.t1
contig001	AUGUSTUS	CDS	866823	868331	0.99	-	0	ID=MALK_00474.t1.c1;Parent=MALK_00474.t1
contig001	AUGUSTUS	mRNA	866823	868331	0.99	-	.	ID=MALK_00474.t1;Parent=MALK_00474
contig001	AUGUSTUS	exon	866823	868331	0.99	-	.	ID=MALK_00474.t1.e1;Parent=MALK_00474.t1
contig001	AUGUSTUS	gene	869342	870412	0.81	+	.	ID=MALK_00475;prediction_source=augustus:contig001.g834.t1
contig001	AUGUSTUS	CDS	869342	870412	0.81	+	0	ID=MALK_00475.t1.c1;Parent=MALK_00475.t1
contig001	AUGUSTUS	mRNA	869342	870412	0.81	+	.	ID=MALK_00475.t1;Parent=MALK_00475
contig001	AUGUSTUS	exon	869342	870412	0.81	+	.	ID=MALK_00475.t1.e1;Parent=MALK_00475.t1
contig001	AUGUSTUS	gene	870419	871297	0.96	-	.	ID=MALK_00476;prediction_source=augustus:contig001.g835.t1
contig001	AUGUSTUS	CDS	870419	871297	0.96	-	0	ID=MALK_00476.t1.c1;Parent=MALK_00476.t1
contig001	AUGUSTUS	mRNA	870419	871297	0.96	-	.	ID=MALK_00476.t1;Parent=MALK_00476
contig001	AUGUSTUS	exon	870419	871297	0.96	-	.	ID=MALK_00476.t1.e1;Parent=MALK_00476.t1
contig001	AUGUSTUS	gene	871440	874175	0.98	+	.	ID=MALK_00477;prediction_source=augustus:contig001.g836.t1
contig001	AUGUSTUS	CDS	871440	874175	0.98	+	0	ID=MALK_00477.t1.c1;Parent=MALK_00477.t1
contig001	AUGUSTUS	mRNA	871440	874175	0.98	+	.	ID=MALK_00477.t1;Parent=MALK_00477
contig001	AUGUSTUS	exon	871440	874175	0.98	+	.	ID=MALK_00477.t1.e1;Parent=MALK_00477.t1
contig001	AUGUSTUS	gene	874172	874825	0.35	-	.	ID=MALK_00478;prediction_source=augustus:contig001.g837.t1
contig001	AUGUSTUS	CDS	874172	874825	0.35	-	0	ID=MALK_00478.t1.c1;Parent=MALK_00478.t1
contig001	AUGUSTUS	mRNA	874172	874825	0.35	-	.	ID=MALK_00478.t1;Parent=MALK_00478
contig001	AUGUSTUS	exon	874172	874825	0.35	-	.	ID=MALK_00478.t1.e1;Parent=MALK_00478.t1
contig001	maker	gene	874913	876295	.	+	.	ID=MALK_00479;prediction_source=maker_MRET:augustus_masked-contig001-processed-gene-8.78-mRNA-1
contig001	maker	CDS	874913	876295	.	+	0	ID=MALK_00479.t1.c1;Parent=MALK_00479.t1
contig001	maker	mRNA	874913	876295	.	+	.	ID=MALK_00479.t1;Parent=MALK_00479
contig001	maker	exon	874913	876295	.	+	.	ID=MALK_00479.t1.e1;Parent=MALK_00479.t1
contig001	AUGUSTUS	gene	876362	878245	0.89	+	.	ID=MALK_00480;prediction_source=braker_MRET:g1132.t1
contig001	AUGUSTUS	CDS	876362	878245	0.89	+	0	ID=MALK_00480.t1.c1;Parent=MALK_00480.t1
contig001	AUGUSTUS	mRNA	876362	878245	0.89	+	.	ID=MALK_00480.t1;Parent=MALK_00480
contig001	AUGUSTUS	exon	876362	878245	.	+	.	ID=MALK_00480.t1.e1;Parent=MALK_00480.t1
contig001	AUGUSTUS	gene	878283	880563	0.37	+	.	ID=MALK_00481;prediction_source=braker_MRET:g1133.t1
contig001	AUGUSTUS	CDS	878283	878535	0.51	+	0	ID=MALK_00481.t1.c1;Parent=MALK_00481.t1
contig001	AUGUSTUS	CDS	878576	880563	0.51	+	0	ID=MALK_00481.t1.c2;Parent=MALK_00481.t1
contig001	AUGUSTUS	mRNA	878283	880563	0.37	+	.	ID=MALK_00481.t1;Parent=MALK_00481
contig001	AUGUSTUS	exon	878283	878535	.	+	.	ID=MALK_00481.t1.e1;Parent=MALK_00481.t1
contig001	AUGUSTUS	exon	878576	880563	.	+	.	ID=MALK_00481.t1.e2;Parent=MALK_00481.t1
contig001	AUGUSTUS	gene	880567	880794	0.94	-	.	ID=MALK_00482;prediction_source=augustus:contig001.g840.t1

contig001	AUGUSTUS	CDS	880567	880794	0.94	-	0	ID=MALK_00482.t1.c1;Parent=MALK_00482.t1
contig001	AUGUSTUS	mRNA	880567	880794	0.94	-	.	ID=MALK_00482.t1;Parent=MALK_00482
contig001	AUGUSTUS	exon	880567	880794	0.94	-	.	ID=MALK_00482.t1.e1;Parent=MALK_00482.t1
contig001	AUGUSTUS	gene	880990	882111	0.71	+	.	ID=MALK_00483;prediction_source=braker_MRET:g1135.t1
contig001	AUGUSTUS	CDS	880990	882111	0.71	+	0	ID=MALK_00483.t1.c1;Parent=MALK_00483.t1
contig001	AUGUSTUS	mRNA	880990	882111	0.71	+	.	ID=MALK_00483.t1;Parent=MALK_00483
contig001	AUGUSTUS	exon	880990	882111	.	+	.	ID=MALK_00483.t1.e1;Parent=MALK_00483.t1
contig001	AUGUSTUS	gene	882230	882663	1	-	.	ID=MALK_00484;prediction_source=braker_MRET:g1136.t1
contig001	AUGUSTUS	CDS	882335	882663	1	-	0	ID=MALK_00484.t1.c2;Parent=MALK_00484.t1
contig001	AUGUSTUS	CDS	882230	882293	1	-	0	ID=MALK_00484.t1.c1;Parent=MALK_00484.t1
contig001	AUGUSTUS	mRNA	882230	882663	1	-	.	ID=MALK_00484.t1;Parent=MALK_00484
contig001	AUGUSTUS	exon	882335	882663	.	-	.	ID=MALK_00484.t1.e2;Parent=MALK_00484.t1
contig001	AUGUSTUS	exon	882230	882293	.	-	.	ID=MALK_00484.t1.e1;Parent=MALK_00484.t1
contig001	AUGUSTUS	gene	882912	883561	0.99	+	.	ID=MALK_00485;prediction_source=braker_MRET:g1137.t1
contig001	AUGUSTUS	CDS	882912	883070	1	+	0	ID=MALK_00485.t1.c1;Parent=MALK_00485.t1
contig001	AUGUSTUS	CDS	883106	883561	1	+	0	ID=MALK_00485.t1.c2;Parent=MALK_00485.t1
contig001	AUGUSTUS	mRNA	882912	883561	0.99	+	.	ID=MALK_00485.t1;Parent=MALK_00485
contig001	AUGUSTUS	exon	882912	883070	.	+	.	ID=MALK_00485.t1.e1;Parent=MALK_00485.t1
contig001	AUGUSTUS	exon	883106	883561	.	+	.	ID=MALK_00485.t1.e2;Parent=MALK_00485.t1
contig001	maker	gene	883616	883954	.	-	.	ID=MALK_00486;prediction_source=maker_MRET:augustus_masked-contig001-processed-gene-8.99-mRNA-1
contig001	maker	CDS	883616	883954	.	-	0	ID=MALK_00486.t1.c1;Parent=MALK_00486.t1
contig001	maker	mRNA	883616	883954	.	-	.	ID=MALK_00486.t1;Parent=MALK_00486
contig001	maker	exon	883616	883954	.	-	.	ID=MALK_00486.t1.e1;Parent=MALK_00486.t1
contig001	AUGUSTUS	gene	884301	885782	1	-	.	ID=MALK_00487;prediction_source=braker_MRET:g1138.t1
contig001	AUGUSTUS	CDS	884301	885782	1	-	0	ID=MALK_00487.t1.c1;Parent=MALK_00487.t1
contig001	AUGUSTUS	mRNA	884301	885782	1	-	.	ID=MALK_00487.t1;Parent=MALK_00487
contig001	AUGUSTUS	exon	884301	885782	.	-	.	ID=MALK_00487.t1.e1;Parent=MALK_00487.t1
contig001	AUGUSTUS	gene	885964	888561	0.34	+	.	ID=MALK_00488;prediction_source=augustus:contig001.g845.t1
contig001	AUGUSTUS	CDS	885964	888561	0.34	+	0	ID=MALK_00488.t1.c1;Parent=MALK_00488.t1
contig001	AUGUSTUS	mRNA	885964	888561	0.34	+	.	ID=MALK_00488.t1;Parent=MALK_00488
contig001	AUGUSTUS	exon	885964	888561	0.34	+	.	ID=MALK_00488.t1.e1;Parent=MALK_00488.t1
contig001	maker	gene	888629	889525	.	-	.	ID=MALK_00489;prediction_source=maker_MRET:augustus_masked-contig001-processed-gene-8.101-mRNA-1
contig001	maker	CDS	888629	889525	.	-	0	ID=MALK_00489.t1.c1;Parent=MALK_00489.t1
contig001	maker	mRNA	888629	889525	.	-	.	ID=MALK_00489.t1;Parent=MALK_00489
contig001	maker	exon	888629	889525	.	-	.	ID=MALK_00489.t1.e1;Parent=MALK_00489.t1
contig001	AUGUSTUS	gene	890002	890787	1	-	.	ID=MALK_00490;prediction_source=augustus:contig001.g847.t1
contig001	AUGUSTUS	CDS	890002	890787	1	-	0	ID=MALK_00490.t1.c1;Parent=MALK_00490.t1
contig001	AUGUSTUS	mRNA	890002	890787	1	-	.	ID=MALK_00490.t1;Parent=MALK_00490
contig001	AUGUSTUS	exon	890002	890787	1	-	.	ID=MALK_00490.t1.e1;Parent=MALK_00490.t1
contig001	AUGUSTUS	gene	891482	894109	0.93	-	.	ID=MALK_00491;prediction_source=augustus:contig001.g848.t1
contig001	AUGUSTUS	CDS	891482	894109	0.93	-	0	ID=MALK_00491.t1.c1;Parent=MALK_00491.t1
contig001	AUGUSTUS	mRNA	891482	894109	0.93	-	.	ID=MALK_00491.t1;Parent=MALK_00491
contig001	AUGUSTUS	exon	891482	894109	0.93	-	.	ID=MALK_00491.t1.e1;Parent=MALK_00491.t1

contig001	maker	gene	894375	895700	.	-	.	ID=MALK_00492;prediction_source=maker_MRET:augustus_masked-contig001-processed-gene-9.3-mRNA-1
contig001	maker	CDS	894375	895700	.	-	0	ID=MALK_00492.t1.c1;Parent=MALK_00492.t1
contig001	maker	mRNA	894375	895700	.	-	.	ID=MALK_00492.t1;Parent=MALK_00492
contig001	maker	exon	894375	895700	.	-	.	ID=MALK_00492.t1.e1;Parent=MALK_00492.t1
contig001	maker	gene	895903	897276	.	+	.	ID=MALK_00493;prediction_source=maker_MRET:augustus_masked-contig001-processed-gene-9.0-mRNA-1
contig001	maker	CDS	895903	897276	.	+	0	ID=MALK_00493.t1.c1;Parent=MALK_00493.t1
contig001	maker	mRNA	895903	897276	.	+	.	ID=MALK_00493.t1;Parent=MALK_00493
contig001	maker	exon	895903	897276	.	+	.	ID=MALK_00493.t1.e1;Parent=MALK_00493.t1
contig001	AUGUSTUS	gene	897279	897854	0.99	-	.	ID=MALK_00494;prediction_source=augustus:contig001.g852.t1
contig001	AUGUSTUS	CDS	897279	897854	0.99	-	0	ID=MALK_00494.t1.c1;Parent=MALK_00494.t1
contig001	AUGUSTUS	mRNA	897279	897854	0.99	-	.	ID=MALK_00494.t1;Parent=MALK_00494
contig001	AUGUSTUS	exon	897279	897854	0.99	-	.	ID=MALK_00494.t1.e1;Parent=MALK_00494.t1
contig001	AUGUSTUS	gene	897935	898870	0.96	+	.	ID=MALK_00495;prediction_source=augustus:contig001.g853.t1
contig001	AUGUSTUS	CDS	897935	898870	0.96	+	0	ID=MALK_00495.t1.c1;Parent=MALK_00495.t1
contig001	AUGUSTUS	mRNA	897935	898870	0.96	+	.	ID=MALK_00495.t1;Parent=MALK_00495
contig001	AUGUSTUS	exon	897935	898870	0.96	+	.	ID=MALK_00495.t1.e1;Parent=MALK_00495.t1
contig001	AUGUSTUS	gene	898874	899638	0.83	-	.	ID=MALK_00496;prediction_source=augustus:contig001.g854.t1
contig001	AUGUSTUS	CDS	898874	899638	0.83	-	0	ID=MALK_00496.t1.c1;Parent=MALK_00496.t1
contig001	AUGUSTUS	mRNA	898874	899638	0.83	-	.	ID=MALK_00496.t1;Parent=MALK_00496
contig001	AUGUSTUS	exon	898874	899638	0.83	-	.	ID=MALK_00496.t1.e1;Parent=MALK_00496.t1
contig001	AUGUSTUS	gene	900101	901273	0.72	+	.	ID=MALK_00497;prediction_source=augustus:contig001.g855.t1
contig001	AUGUSTUS	CDS	900101	901273	0.72	+	0	ID=MALK_00497.t1.c1;Parent=MALK_00497.t1
contig001	AUGUSTUS	mRNA	900101	901273	0.72	+	.	ID=MALK_00497.t1;Parent=MALK_00497
contig001	AUGUSTUS	exon	900101	901273	0.72	+	.	ID=MALK_00497.t1.e1;Parent=MALK_00497.t1
contig001	AUGUSTUS	gene	901301	902137	1	-	.	ID=MALK_00498;prediction_source=augustus:contig001.g856.t1
contig001	AUGUSTUS	CDS	901301	902137	1	-	0	ID=MALK_00498.t1.c1;Parent=MALK_00498.t1
contig001	AUGUSTUS	mRNA	901301	902137	1	-	.	ID=MALK_00498.t1;Parent=MALK_00498
contig001	AUGUSTUS	exon	901301	902137	1	-	.	ID=MALK_00498.t1.e1;Parent=MALK_00498.t1
contig001	AUGUSTUS	gene	902512	903127	0.27	+	.	ID=MALK_00499;prediction_source=braker_MRET:g1150.t1
contig001	AUGUSTUS	CDS	902512	902653	0.74	+	0	ID=MALK_00499.t1.c1;Parent=MALK_00499.t1
contig001	AUGUSTUS	CDS	902682	903127	0.74	+	0	ID=MALK_00499.t1.c2;Parent=MALK_00499.t1
contig001	AUGUSTUS	mRNA	902512	903127	0.27	+	.	ID=MALK_00499.t1;Parent=MALK_00499
contig001	AUGUSTUS	exon	902512	902653	.	+	.	ID=MALK_00499.t1.e1;Parent=MALK_00499.t1
contig001	AUGUSTUS	exon	902682	903127	.	+	.	ID=MALK_00499.t1.e2;Parent=MALK_00499.t1
contig001	AUGUSTUS	gene	903172	903613	0.9	-	.	ID=MALK_00500;prediction_source=braker_MRET:g1151.t1
contig001	AUGUSTUS	CDS	903535	903613	0.92	-	0	ID=MALK_00500.t1.c3;Parent=MALK_00500.t1
contig001	AUGUSTUS	CDS	903473	903501	0.92	-	0	ID=MALK_00500.t1.c2;Parent=MALK_00500.t1
contig001	AUGUSTUS	CDS	903172	903435	0.92	-	0	ID=MALK_00500.t1.c1;Parent=MALK_00500.t1
contig001	AUGUSTUS	mRNA	903172	903613	0.9	-	.	ID=MALK_00500.t1;Parent=MALK_00500
contig001	AUGUSTUS	exon	903535	903613	.	-	.	ID=MALK_00500.t1.e3;Parent=MALK_00500.t1
contig001	AUGUSTUS	exon	903473	903501	.	-	.	ID=MALK_00500.t1.e2;Parent=MALK_00500.t1
contig001	AUGUSTUS	exon	903172	903435	.	-	.	ID=MALK_00500.t1.e1;Parent=MALK_00500.t1
contig001	AUGUSTUS	gene	903820	905925	0.33	-	.	ID=MALK_00501;prediction_source=augustus:contig001.g859.t1

contig001	AUGUSTUS	CDS	903820	905925	0.33	-	0	ID=MALK_00501.t1.c1;Parent=MALK_00501.t1
contig001	AUGUSTUS	mRNA	903820	905925	0.33	-	.	ID=MALK_00501.t1;Parent=MALK_00501
contig001	AUGUSTUS	exon	903820	905925	0.33	-	.	ID=MALK_00501.t1.e1;Parent=MALK_00501.t1
contig001	AUGUSTUS	gene	906077	908509	1	+	.	ID=MALK_00502;prediction_source=augustus:contig001.g860.t1
contig001	AUGUSTUS	CDS	906077	908509	1	+	0	ID=MALK_00502.t1.c1;Parent=MALK_00502.t1
contig001	AUGUSTUS	mRNA	906077	908509	1	+	.	ID=MALK_00502.t1;Parent=MALK_00502
contig001	AUGUSTUS	exon	906077	908509	1	+	.	ID=MALK_00502.t1.e1;Parent=MALK_00502.t1
contig001	AUGUSTUS	gene	908514	910583	0.46	-	.	ID=MALK_00503;prediction_source=augustus:contig001.g862.t1
contig001	AUGUSTUS	CDS	908514	910583	0.46	-	0	ID=MALK_00503.t1.c1;Parent=MALK_00503.t1
contig001	AUGUSTUS	mRNA	908514	910583	0.46	-	.	ID=MALK_00503.t1;Parent=MALK_00503
contig001	AUGUSTUS	exon	908514	910583	0.46	-	.	ID=MALK_00503.t1.e1;Parent=MALK_00503.t1
contig001	maker	gene	910907	912691	.	+	.	ID=MALK_00504;prediction_source=maker_MRET:augustus_masked-contig001-processed-gene-9.9-mRNA-1
contig001	maker	CDS	910907	912691	.	+	0	ID=MALK_00504.t1.c1;Parent=MALK_00504.t1
contig001	maker	mRNA	910907	912691	.	+	.	ID=MALK_00504.t1;Parent=MALK_00504
contig001	maker	exon	910907	912691	.	+	.	ID=MALK_00504.t1.e1;Parent=MALK_00504.t1
contig001	AUGUSTUS	gene	912704	915194	0.25	-	.	ID=MALK_00505;prediction_source=braker_MRET:g1156.t1
contig001	AUGUSTUS	CDS	913377	915194	0.29	-	0	ID=MALK_00505.t1.c2;Parent=MALK_00505.t1
contig001	AUGUSTUS	CDS	912704	913327	0.29	-	0	ID=MALK_00505.t1.c1;Parent=MALK_00505.t1
contig001	AUGUSTUS	mRNA	912704	915194	0.25	-	.	ID=MALK_00505.t1;Parent=MALK_00505
contig001	AUGUSTUS	exon	913377	915194	.	-	.	ID=MALK_00505.t1.e2;Parent=MALK_00505.t1
contig001	AUGUSTUS	exon	912704	913327	.	-	.	ID=MALK_00505.t1.e1;Parent=MALK_00505.t1
contig001	AUGUSTUS	gene	915328	916137	0.85	-	.	ID=MALK_00506;prediction_source=braker_MRET:g1157.t1
contig001	AUGUSTUS	CDS	915328	916137	0.85	-	0	ID=MALK_00506.t1.c1;Parent=MALK_00506.t1
contig001	AUGUSTUS	mRNA	915328	916137	0.85	-	.	ID=MALK_00506.t1;Parent=MALK_00506
contig001	AUGUSTUS	exon	915328	916137	.	-	.	ID=MALK_00506.t1.e1;Parent=MALK_00506.t1
contig001	maker	gene	916286	917095	.	-	.	ID=MALK_00507;prediction_source=maker_MRET:augustus_masked-contig001-processed-gene-9.36-mRNA-1
contig001	maker	CDS	916286	917095	.	-	0	ID=MALK_00507.t1.c1;Parent=MALK_00507.t1
contig001	maker	mRNA	916286	917095	.	-	.	ID=MALK_00507.t1;Parent=MALK_00507
contig001	maker	exon	916286	917095	.	-	.	ID=MALK_00507.t1.e1;Parent=MALK_00507.t1
contig001	AUGUSTUS	gene	917591	918661	0.97	-	.	ID=MALK_00508;prediction_source=augustus:contig001.g867.t1
contig001	AUGUSTUS	CDS	917591	918661	0.97	-	0	ID=MALK_00508.t1.c1;Parent=MALK_00508.t1
contig001	AUGUSTUS	mRNA	917591	918661	0.97	-	.	ID=MALK_00508.t1;Parent=MALK_00508
contig001	AUGUSTUS	exon	917591	918661	0.97	-	.	ID=MALK_00508.t1.e1;Parent=MALK_00508.t1
contig001	AUGUSTUS	gene	918804	919172	0.7	+	.	ID=MALK_00509;prediction_source=braker_MRET:g1160.t1
contig001	AUGUSTUS	CDS	918804	919172	0.7	+	0	ID=MALK_00509.t1.c1;Parent=MALK_00509.t1
contig001	AUGUSTUS	mRNA	918804	919172	0.7	+	.	ID=MALK_00509.t1;Parent=MALK_00509
contig001	AUGUSTUS	exon	918804	919172	.	+	.	ID=MALK_00509.t1.e1;Parent=MALK_00509.t1
contig001	maker	gene	919311	921569	.	+	.	ID=MALK_00510;prediction_source=maker_MRET:augustus_masked-contig001-processed-gene-9.11-mRNA-1
contig001	maker	CDS	919311	921569	.	+	0	ID=MALK_00510.t1.c1;Parent=MALK_00510.t1
contig001	maker	mRNA	919311	921569	.	+	.	ID=MALK_00510.t1;Parent=MALK_00510
contig001	maker	exon	919311	921569	.	+	.	ID=MALK_00510.t1.e1;Parent=MALK_00510.t1
contig001	AUGUSTUS	gene	921570	923660	0.68	-	.	ID=MALK_00511;prediction_source=augustus:contig001.g871.t1
contig001	AUGUSTUS	CDS	921570	923660	0.68	-	0	ID=MALK_00511.t1.c1;Parent=MALK_00511.t1

contig001	AUGUSTUS	mRNA	921570	923660	0.68	-	.	ID=MALK_00511.t1;Parent=MALK_00511
contig001	AUGUSTUS	exon	921570	923660	0.68	-	.	ID=MALK_00511.t1.e1;Parent=MALK_00511.t1
contig001	AUGUSTUS	gene	924019	927387	1	+	.	ID=MALK_00512;prediction_source=braker_MRET:g1163.t1
contig001	AUGUSTUS	CDS	924019	927387	1	+	0	ID=MALK_00512.t1.c1;Parent=MALK_00512.t1
contig001	AUGUSTUS	mRNA	924019	927387	1	+	.	ID=MALK_00512.t1;Parent=MALK_00512
contig001	AUGUSTUS	exon	924019	927387	.	+	.	ID=MALK_00512.t1.e1;Parent=MALK_00512.t1
contig001	AUGUSTUS	gene	927401	929371	1	-	.	ID=MALK_00513;prediction_source=augustus:contig001.g874.t1
contig001	AUGUSTUS	CDS	927401	929371	1	-	0	ID=MALK_00513.t1.c1;Parent=MALK_00513.t1
contig001	AUGUSTUS	mRNA	927401	929371	1	-	.	ID=MALK_00513.t1;Parent=MALK_00513
contig001	AUGUSTUS	exon	927401	929371	1	-	.	ID=MALK_00513.t1.e1;Parent=MALK_00513.t1
contig001	maker	gene	929609	931288	.	+	.	ID=MALK_00514;prediction_source=maker_MRET:augustus_masked-contig001-processed-gene-9.13-mRNA-1
contig001	maker	CDS	929609	931288	.	+	0	ID=MALK_00514.t1.c1;Parent=MALK_00514.t1
contig001	maker	mRNA	929609	931288	.	+	.	ID=MALK_00514.t1;Parent=MALK_00514
contig001	maker	exon	929609	931288	.	+	.	ID=MALK_00514.t1.e1;Parent=MALK_00514.t1
contig001	AUGUSTUS	gene	931574	931923	1	-	.	ID=MALK_00515;prediction_source=braker_MRET:g1166.t1
contig001	AUGUSTUS	CDS	931921	931923	1	-	0	ID=MALK_00515.t1.c3;Parent=MALK_00515.t1
contig001	AUGUSTUS	CDS	931657	931855	1	-	0	ID=MALK_00515.t1.c2;Parent=MALK_00515.t1
contig001	AUGUSTUS	CDS	931574	931614	1	-	0	ID=MALK_00515.t1.c1;Parent=MALK_00515.t1
contig001	AUGUSTUS	mRNA	931574	931923	1	-	.	ID=MALK_00515.t1;Parent=MALK_00515
contig001	AUGUSTUS	exon	931921	931923	.	-	.	ID=MALK_00515.t1.e3;Parent=MALK_00515.t1
contig001	AUGUSTUS	exon	931657	931855	.	-	.	ID=MALK_00515.t1.e2;Parent=MALK_00515.t1
contig001	AUGUSTUS	exon	931574	931614	.	-	.	ID=MALK_00515.t1.e1;Parent=MALK_00515.t1
contig001	maker	gene	932187	932713	.	+	.	ID=MALK_00516;prediction_source=maker_MRET:augustus_masked-contig001-processed-gene-9.14-mRNA-1
contig001	maker	CDS	932187	932281	.	+	0	ID=MALK_00516.t1.c1;Parent=MALK_00516.t1
contig001	maker	CDS	932365	932713	.	+	0	ID=MALK_00516.t1.c2;Parent=MALK_00516.t1
contig001	maker	mRNA	932187	932713	.	+	.	ID=MALK_00516.t1;Parent=MALK_00516
contig001	maker	exon	932187	932281	.	+	.	ID=MALK_00516.t1.e1;Parent=MALK_00516.t1
contig001	maker	exon	932365	932713	.	+	.	ID=MALK_00516.t1.e2;Parent=MALK_00516.t1
contig001	AUGUSTUS	gene	932978	933620	0.16	-	.	ID=MALK_00517;prediction_source=braker_MRET:g1168.t1
contig001	AUGUSTUS	CDS	933393	933620	0.46	-	0	ID=MALK_00517.t1.c5;Parent=MALK_00517.t1
contig001	AUGUSTUS	CDS	933307	933355	0.46	-	0	ID=MALK_00517.t1.c4;Parent=MALK_00517.t1
contig001	AUGUSTUS	CDS	933124	933265	0.46	-	0	ID=MALK_00517.t1.c3;Parent=MALK_00517.t1
contig001	AUGUSTUS	CDS	933074	933095	0.46	-	0	ID=MALK_00517.t1.c2;Parent=MALK_00517.t1
contig001	AUGUSTUS	CDS	932978	933043	0.46	-	0	ID=MALK_00517.t1.c1;Parent=MALK_00517.t1
contig001	AUGUSTUS	mRNA	932978	933620	0.16	-	.	ID=MALK_00517.t1;Parent=MALK_00517
contig001	AUGUSTUS	exon	933393	933620	.	-	.	ID=MALK_00517.t1.e5;Parent=MALK_00517.t1
contig001	AUGUSTUS	exon	933307	933355	.	-	.	ID=MALK_00517.t1.e4;Parent=MALK_00517.t1
contig001	AUGUSTUS	exon	933124	933265	.	-	.	ID=MALK_00517.t1.e3;Parent=MALK_00517.t1
contig001	AUGUSTUS	exon	933074	933095	.	-	.	ID=MALK_00517.t1.e2;Parent=MALK_00517.t1
contig001	AUGUSTUS	exon	932978	933043	.	-	.	ID=MALK_00517.t1.e1;Parent=MALK_00517.t1
contig001	maker	gene	933747	936557	.	-	.	ID=MALK_00518;prediction_source=maker_MRET:augustus_masked-contig001-processed-gene-9.41-mRNA-1
contig001	maker	CDS	933747	936557	.	-	0	ID=MALK_00518.t1.c1;Parent=MALK_00518.t1
contig001	maker	mRNA	933747	936557	.	-	.	ID=MALK_00518.t1;Parent=MALK_00518

contig001	maker	exon	933747	936557	.	-	.	ID=MALK_00518.t1.e1;Parent=MALK_00518.t1
contig001	AUGUSTUS	gene	936664	938211	0.94	-	.	ID=MALK_00519;prediction_source=augustus:contig001.g880.t1
contig001	AUGUSTUS	CDS	936664	938211	0.94	-	0	ID=MALK_00519.t1.c1;Parent=MALK_00519.t1
contig001	AUGUSTUS	mRNA	936664	938211	0.94	-	.	ID=MALK_00519.t1;Parent=MALK_00519
contig001	AUGUSTUS	exon	936664	938211	0.94	-	.	ID=MALK_00519.t1.e1;Parent=MALK_00519.t1
contig001	AUGUSTUS	gene	938412	939177	0.28	+	.	ID=MALK_00520;prediction_source=braker_MRET:g1171.t1
contig001	AUGUSTUS	CDS	938412	938458	0.4	+	0	ID=MALK_00520.t1.c1;Parent=MALK_00520.t1
contig001	AUGUSTUS	CDS	938506	938551	0.4	+	0	ID=MALK_00520.t1.c2;Parent=MALK_00520.t1
contig001	AUGUSTUS	CDS	938590	939177	0.4	+	0	ID=MALK_00520.t1.c3;Parent=MALK_00520.t1
contig001	AUGUSTUS	mRNA	938412	939177	0.28	+	.	ID=MALK_00520.t1;Parent=MALK_00520
contig001	AUGUSTUS	exon	938412	938458	.	+	.	ID=MALK_00520.t1.e1;Parent=MALK_00520.t1
contig001	AUGUSTUS	exon	938506	938551	.	+	.	ID=MALK_00520.t1.e2;Parent=MALK_00520.t1
contig001	AUGUSTUS	exon	938590	939177	.	+	.	ID=MALK_00520.t1.e3;Parent=MALK_00520.t1
contig001	AUGUSTUS	gene	939249	941171	0.45	-	.	ID=MALK_00521;prediction_source=braker_MRET:g1172.t1
contig001	AUGUSTUS	CDS	940821	941171	0.88	-	0	ID=MALK_00521.t1.c3;Parent=MALK_00521.t1
contig001	AUGUSTUS	CDS	940738	940788	0.88	-	0	ID=MALK_00521.t1.c2;Parent=MALK_00521.t1
contig001	AUGUSTUS	CDS	939249	940706	0.88	-	0	ID=MALK_00521.t1.c1;Parent=MALK_00521.t1
contig001	AUGUSTUS	mRNA	939249	941171	0.45	-	.	ID=MALK_00521.t1;Parent=MALK_00521
contig001	AUGUSTUS	exon	940821	941171	.	-	.	ID=MALK_00521.t1.e3;Parent=MALK_00521.t1
contig001	AUGUSTUS	exon	940738	940788	.	-	.	ID=MALK_00521.t1.e2;Parent=MALK_00521.t1
contig001	AUGUSTUS	exon	939249	940706	.	-	.	ID=MALK_00521.t1.e1;Parent=MALK_00521.t1
contig001	maker	gene	941567	945238	.	-	.	ID=MALK_00522;prediction_source=maker_MRET:augustus_masked-contig001-processed-gene-9.45-mRNA-1
contig001	maker	CDS	941567	945238	.	-	0	ID=MALK_00522.t1.c1;Parent=MALK_00522.t1
contig001	maker	mRNA	941567	945238	.	-	.	ID=MALK_00522.t1;Parent=MALK_00522
contig001	maker	exon	941567	945238	.	-	.	ID=MALK_00522.t1.e1;Parent=MALK_00522.t1
contig001	maker	gene	945377	947857	.	-	.	ID=MALK_00523;prediction_source=maker_MRET:augustus_masked-contig001-processed-gene-9.46-mRNA-1
contig001	maker	CDS	945377	947857	.	-	0	ID=MALK_00523.t1.c1;Parent=MALK_00523.t1
contig001	maker	mRNA	945377	947857	.	-	.	ID=MALK_00523.t1;Parent=MALK_00523
contig001	maker	exon	945377	947857	.	-	.	ID=MALK_00523.t1.e1;Parent=MALK_00523.t1
contig001	maker	gene	948339	951644	.	+	.	ID=MALK_00524;prediction_source=maker_MRET:augustus_masked-contig001-processed-gene-9.16-mRNA-1
contig001	maker	CDS	948339	951644	.	+	0	ID=MALK_00524.t1.c1;Parent=MALK_00524.t1
contig001	maker	mRNA	948339	951644	.	+	.	ID=MALK_00524.t1;Parent=MALK_00524
contig001	maker	exon	948339	951644	.	+	.	ID=MALK_00524.t1.e1;Parent=MALK_00524.t1
contig001	maker	gene	951668	954427	.	-	.	ID=MALK_00525;prediction_source=maker_MRET:augustus_masked-contig001-processed-gene-9.47-mRNA-1
contig001	maker	CDS	951668	954427	.	-	0	ID=MALK_00525.t1.c1;Parent=MALK_00525.t1
contig001	maker	mRNA	951668	954427	.	-	.	ID=MALK_00525.t1;Parent=MALK_00525
contig001	maker	exon	951668	954427	.	-	.	ID=MALK_00525.t1.e1;Parent=MALK_00525.t1
contig001	AUGUSTUS	gene	954531	955517	0.93	-	.	ID=MALK_00526;prediction_source=braker_MRET:g1177.t1
contig001	AUGUSTUS	CDS	954531	955517	0.93	-	0	ID=MALK_00526.t1.c1;Parent=MALK_00526.t1
contig001	AUGUSTUS	mRNA	954531	955517	0.93	-	.	ID=MALK_00526.t1;Parent=MALK_00526
contig001	AUGUSTUS	exon	954531	955517	.	-	.	ID=MALK_00526.t1.e1;Parent=MALK_00526.t1
contig001	AUGUSTUS	gene	955647	957689	0.99	-	.	ID=MALK_00527;prediction_source=braker_MRET:g1178.t1
contig001	AUGUSTUS	CDS	955647	957689	0.99	-	0	ID=MALK_00527.t1.c1;Parent=MALK_00527.t1

contig001	AUGUSTUS	mRNA	955647	957689	0.99	-	.	ID=MALK_00527.t1;Parent=MALK_00527
contig001	AUGUSTUS	exon	955647	957689	.	-	.	ID=MALK_00527.t1.e1;Parent=MALK_00527.t1
contig001	AUGUSTUS	gene	957978	959939	0.95	-	.	ID=MALK_00528;prediction_source=braker_MRET:g1179.t1
contig001	AUGUSTUS	CDS	957978	959939	0.95	-	0	ID=MALK_00528.t1.c1;Parent=MALK_00528.t1
contig001	AUGUSTUS	mRNA	957978	959939	0.95	-	.	ID=MALK_00528.t1;Parent=MALK_00528
contig001	AUGUSTUS	exon	957978	959939	.	-	.	ID=MALK_00528.t1.e1;Parent=MALK_00528.t1
contig001	maker	gene	960086	962065	.	+	.	ID=MALK_00529;prediction_source=maker_MRET:augustus_masked-contig001-processed-gene-9.17-mRNA-1
contig001	maker	CDS	960086	962065	.	+	0	ID=MALK_00529.t1.c1;Parent=MALK_00529.t1
contig001	maker	mRNA	960086	962065	.	+	.	ID=MALK_00529.t1;Parent=MALK_00529
contig001	maker	exon	960086	962065	.	+	.	ID=MALK_00529.t1.e1;Parent=MALK_00529.t1
contig001	AUGUSTUS	gene	962135	962935	0.98	+	.	ID=MALK_00530;prediction_source=augustus:contig001.g891.t1
contig001	AUGUSTUS	CDS	962135	962935	0.98	+	0	ID=MALK_00530.t1.c1;Parent=MALK_00530.t1
contig001	AUGUSTUS	mRNA	962135	962935	0.98	+	.	ID=MALK_00530.t1;Parent=MALK_00530
contig001	AUGUSTUS	exon	962135	962935	0.98	+	.	ID=MALK_00530.t1.e1;Parent=MALK_00530.t1
contig001	AUGUSTUS	gene	963086	965167	0.94	+	.	ID=MALK_00531;prediction_source=augustus:contig001.g893.t1
contig001	AUGUSTUS	CDS	963086	965167	0.94	+	0	ID=MALK_00531.t1.c1;Parent=MALK_00531.t1
contig001	AUGUSTUS	mRNA	963086	965167	0.94	+	.	ID=MALK_00531.t1;Parent=MALK_00531
contig001	AUGUSTUS	exon	963086	965167	0.94	+	.	ID=MALK_00531.t1.e1;Parent=MALK_00531.t1
contig001	AUGUSTUS	gene	965225	966309	0.77	+	.	ID=MALK_00532;prediction_source=braker_MRET:g1183.t1
contig001	AUGUSTUS	CDS	965225	966179	0.94	+	0	ID=MALK_00532.t1.c1;Parent=MALK_00532.t1
contig001	AUGUSTUS	CDS	966221	966309	0.94	+	0	ID=MALK_00532.t1.c2;Parent=MALK_00532.t1
contig001	AUGUSTUS	mRNA	965225	966309	0.77	+	.	ID=MALK_00532.t1;Parent=MALK_00532
contig001	AUGUSTUS	exon	965225	966179	.	+	.	ID=MALK_00532.t1.e1;Parent=MALK_00532.t1
contig001	AUGUSTUS	exon	966221	966309	.	+	.	ID=MALK_00532.t1.e2;Parent=MALK_00532.t1
contig001	AUGUSTUS	gene	966411	967976	0.99	+	.	ID=MALK_00533;prediction_source=braker_MRET:g1184.t1
contig001	AUGUSTUS	CDS	966411	967976	0.99	+	0	ID=MALK_00533.t1.c1;Parent=MALK_00533.t1
contig001	AUGUSTUS	mRNA	966411	967976	0.99	+	.	ID=MALK_00533.t1;Parent=MALK_00533
contig001	AUGUSTUS	exon	966411	967976	.	+	.	ID=MALK_00533.t1.e1;Parent=MALK_00533.t1
contig001	maker	gene	968205	969221	.	+	.	ID=MALK_00534;prediction_source=maker_MRET:augustus_masked-contig001-processed-gene-9.21-mRNA-1
contig001	maker	CDS	968205	969221	.	+	0	ID=MALK_00534.t1.c1;Parent=MALK_00534.t1
contig001	maker	mRNA	968205	969221	.	+	.	ID=MALK_00534.t1;Parent=MALK_00534
contig001	maker	exon	968205	969221	.	+	.	ID=MALK_00534.t1.e1;Parent=MALK_00534.t1
contig001	AUGUSTUS	gene	969362	969949	0.71	+	.	ID=MALK_00535;prediction_source=braker_MRET:g1186.t1
contig001	AUGUSTUS	CDS	969362	969949	0.71	+	0	ID=MALK_00535.t1.c1;Parent=MALK_00535.t1
contig001	AUGUSTUS	mRNA	969362	969949	0.71	+	.	ID=MALK_00535.t1;Parent=MALK_00535
contig001	AUGUSTUS	exon	969362	969949	.	+	.	ID=MALK_00535.t1.e1;Parent=MALK_00535.t1
contig001	maker	gene	969959	970828	.	-	.	ID=MALK_00536;prediction_source=maker_MRET:augustus_masked-contig001-processed-gene-9.49-mRNA-1
contig001	maker	CDS	969959	970828	.	-	0	ID=MALK_00536.t1.c1;Parent=MALK_00536.t1
contig001	maker	mRNA	969959	970828	.	-	.	ID=MALK_00536.t1;Parent=MALK_00536
contig001	maker	exon	969959	970828	.	-	.	ID=MALK_00536.t1.e1;Parent=MALK_00536.t1
contig001	AUGUSTUS	gene	971124	977253	0.4	+	.	ID=MALK_00537;prediction_source=braker_MRET:g1188.t1
contig001	AUGUSTUS	CDS	971124	971394	0.51	+	0	ID=MALK_00537.t1.c1;Parent=MALK_00537.t1
contig001	AUGUSTUS	CDS	971423	971553	0.51	+	0	ID=MALK_00537.t1.c2;Parent=MALK_00537.t1

contig001	AUGUSTUS	CDS	971722	977253	0.51	+	0	ID=MALK_00537.t1.c3;Parent=MALK_00537.t1
contig001	AUGUSTUS	mRNA	971124	977253	0.4	+	.	ID=MALK_00537.t1;Parent=MALK_00537
contig001	AUGUSTUS	exon	971124	971394	.	+	.	ID=MALK_00537.t1.e1;Parent=MALK_00537.t1
contig001	AUGUSTUS	exon	971423	971553	.	+	.	ID=MALK_00537.t1.e2;Parent=MALK_00537.t1
contig001	AUGUSTUS	exon	971722	977253	.	+	.	ID=MALK_00537.t1.e3;Parent=MALK_00537.t1
contig001	AUGUSTUS	gene	977820	979433	0.47	+	.	ID=MALK_00538;prediction_source=braker_MRET:g1189.t1
contig001	AUGUSTUS	CDS	977820	979433	0.47	+	0	ID=MALK_00538.t1.c1;Parent=MALK_00538.t1
contig001	AUGUSTUS	mRNA	977820	979433	0.47	+	.	ID=MALK_00538.t1;Parent=MALK_00538
contig001	AUGUSTUS	exon	977820	979433	.	+	.	ID=MALK_00538.t1.e1;Parent=MALK_00538.t1
contig001	AUGUSTUS	gene	979514	980311	0.43	+	.	ID=MALK_00539;prediction_source=braker_MRET:g1190.t1
contig001	AUGUSTUS	CDS	979514	980311	0.43	+	0	ID=MALK_00539.t1.c1;Parent=MALK_00539.t1
contig001	AUGUSTUS	mRNA	979514	980311	0.43	+	.	ID=MALK_00539.t1;Parent=MALK_00539
contig001	AUGUSTUS	exon	979514	980311	.	+	.	ID=MALK_00539.t1.e1;Parent=MALK_00539.t1
contig001	AUGUSTUS	gene	980382	980973	0.56	-	.	ID=MALK_00540;prediction_source=braker_MRET:g1191.t1
contig001	AUGUSTUS	CDS	980523	980973	0.61	-	0	ID=MALK_00540.t1.c2;Parent=MALK_00540.t1
contig001	AUGUSTUS	CDS	980382	980479	0.61	-	0	ID=MALK_00540.t1.c1;Parent=MALK_00540.t1
contig001	AUGUSTUS	mRNA	980382	980973	0.56	-	.	ID=MALK_00540.t1;Parent=MALK_00540
contig001	AUGUSTUS	exon	980523	980973	.	-	.	ID=MALK_00540.t1.e2;Parent=MALK_00540.t1
contig001	AUGUSTUS	exon	980382	980479	.	-	.	ID=MALK_00540.t1.e1;Parent=MALK_00540.t1
contig001	AUGUSTUS	gene	981047	982459	0.41	+	.	ID=MALK_00541;prediction_source=augustus:contig001.g899.t1
contig001	AUGUSTUS	CDS	981047	982459	0.41	+	0	ID=MALK_00541.t1.c1;Parent=MALK_00541.t1
contig001	AUGUSTUS	mRNA	981047	982459	0.41	+	.	ID=MALK_00541.t1;Parent=MALK_00541
contig001	AUGUSTUS	exon	981047	982459	0.41	+	.	ID=MALK_00541.t1.e1;Parent=MALK_00541.t1
contig001	AUGUSTUS	gene	982445	985312	0.55	-	.	ID=MALK_00542;prediction_source=augustus:contig001.g900.t1
contig001	AUGUSTUS	CDS	982445	985312	0.55	-	0	ID=MALK_00542.t1.c1;Parent=MALK_00542.t1
contig001	AUGUSTUS	mRNA	982445	985312	0.55	-	.	ID=MALK_00542.t1;Parent=MALK_00542
contig001	AUGUSTUS	exon	982445	985312	0.55	-	.	ID=MALK_00542.t1.e1;Parent=MALK_00542.t1
contig001	AUGUSTUS	gene	985673	987151	0.51	+	.	ID=MALK_00543;prediction_source=braker_MRET:g1194.t1
contig001	AUGUSTUS	CDS	985673	985691	0.89	+	0	ID=MALK_00543.t1.c1;Parent=MALK_00543.t1
contig001	AUGUSTUS	CDS	985723	985756	0.89	+	0	ID=MALK_00543.t1.c2;Parent=MALK_00543.t1
contig001	AUGUSTUS	CDS	985789	987151	0.89	+	0	ID=MALK_00543.t1.c3;Parent=MALK_00543.t1
contig001	AUGUSTUS	mRNA	985673	987151	0.51	+	.	ID=MALK_00543.t1;Parent=MALK_00543
contig001	AUGUSTUS	exon	985673	985691	.	+	.	ID=MALK_00543.t1.e1;Parent=MALK_00543.t1
contig001	AUGUSTUS	exon	985723	985756	.	+	.	ID=MALK_00543.t1.e2;Parent=MALK_00543.t1
contig001	AUGUSTUS	exon	985789	987151	.	+	.	ID=MALK_00543.t1.e3;Parent=MALK_00543.t1
contig001	maker	gene	987262	988938	.	+	.	ID=MALK_00544;prediction_source=maker_MRET:augustus_masked-contig001-processed-gene-9.24-mRNA-1
contig001	maker	CDS	987262	988938	.	+	0	ID=MALK_00544.t1.c1;Parent=MALK_00544.t1
contig001	maker	mRNA	987262	988938	.	+	.	ID=MALK_00544.t1;Parent=MALK_00544
contig001	maker	exon	987262	988938	.	+	.	ID=MALK_00544.t1.e1;Parent=MALK_00544.t1
contig001	maker	gene	988944	990098	.	-	.	ID=MALK_00545;prediction_source=maker_MRET:augustus_masked-contig001-processed-gene-9.51-mRNA-1
contig001	maker	CDS	988944	990098	.	-	0	ID=MALK_00545.t1.c1;Parent=MALK_00545.t1
contig001	maker	mRNA	988944	990098	.	-	.	ID=MALK_00545.t1;Parent=MALK_00545
contig001	maker	exon	988944	990098	.	-	.	ID=MALK_00545.t1.e1;Parent=MALK_00545.t1

contig001	maker	gene	990465	990812	.	+	.	ID=MALK_00546;prediction_source=maker_MRET:augustus_masked-contig001-processed-gene-9.25-mRNA-1
contig001	maker	CDS	990465	990812	.	+	0	ID=MALK_00546.t1.c1;Parent=MALK_00546.t1
contig001	maker	mRNA	990465	990812	.	+	.	ID=MALK_00546.t1;Parent=MALK_00546
contig001	maker	exon	990465	990812	.	+	.	ID=MALK_00546.t1.e1;Parent=MALK_00546.t1
contig001	maker	gene	990888	994561	.	-	.	ID=MALK_00547;prediction_source=maker_MRET:augustus_masked-contig001-processed-gene-9.52-mRNA-1
contig001	maker	CDS	994529	994561	.	-	0	ID=MALK_00547.t1.c1;Parent=MALK_00547.t1
contig001	maker	CDS	990888	994130	.	-	0	ID=MALK_00547.t1.c2;Parent=MALK_00547.t1
contig001	maker	mRNA	990888	994561	.	-	.	ID=MALK_00547.t1;Parent=MALK_00547
contig001	maker	exon	994529	994561	.	-	.	ID=MALK_00547.t1.e1;Parent=MALK_00547.t1
contig001	maker	exon	990888	994130	.	-	.	ID=MALK_00547.t1.e2;Parent=MALK_00547.t1
contig001	maker	gene	994617	997778	.	+	.	ID=MALK_00548;prediction_source=maker_MRET:augustus_masked-contig001-processed-gene-9.26-mRNA-1
contig001	maker	CDS	994617	997778	.	+	0	ID=MALK_00548.t1.c1;Parent=MALK_00548.t1
contig001	maker	mRNA	994617	997778	.	+	.	ID=MALK_00548.t1;Parent=MALK_00548
contig001	maker	exon	994617	997778	.	+	.	ID=MALK_00548.t1.e1;Parent=MALK_00548.t1
contig001	AUGUSTUS	gene	997813	998523	0.98	-	.	ID=MALK_00549;prediction_source=braker_MRET:g1200.t1
contig001	AUGUSTUS	CDS	997813	998523	0.98	-	0	ID=MALK_00549.t1.c1;Parent=MALK_00549.t1
contig001	AUGUSTUS	mRNA	997813	998523	0.98	-	.	ID=MALK_00549.t1;Parent=MALK_00549
contig001	AUGUSTUS	exon	997813	998523	.	-	.	ID=MALK_00549.t1.e1;Parent=MALK_00549.t1
contig001	maker	gene	999001	1E+06	.	+	.	ID=MALK_00550;prediction_source=maker_MRET:augustus_masked-contig001-processed-gene-9.27-mRNA-1
contig001	maker	CDS	999001	1E+06	.	+	0	ID=MALK_00550.t1.c1;Parent=MALK_00550.t1
contig001	maker	mRNA	999001	1E+06	.	+	.	ID=MALK_00550.t1;Parent=MALK_00550
contig001	maker	exon	999001	1E+06	.	+	.	ID=MALK_00550.t1.e1;Parent=MALK_00550.t1
contig001	maker	gene	1E+06	1E+06	.	+	.	ID=MALK_00551;prediction_source=maker_MRET:augustus_masked-contig001-processed-gene-9.28-mRNA-1
contig001	maker	CDS	1E+06	1E+06	.	+	0	ID=MALK_00551.t1.c1;Parent=MALK_00551.t1
contig001	maker	mRNA	1E+06	1E+06	.	+	.	ID=MALK_00551.t1;Parent=MALK_00551
contig001	maker	exon	1E+06	1E+06	.	+	.	ID=MALK_00551.t1.e1;Parent=MALK_00551.t1
contig001	maker	gene	1E+06	1E+06	.	-	.	ID=MALK_00552;prediction_source=maker_MRET:augustus_masked-contig001-processed-gene-9.53-mRNA-1
contig001	maker	CDS	1E+06	1E+06	.	-	0	ID=MALK_00552.t1.c1;Parent=MALK_00552.t1
contig001	maker	mRNA	1E+06	1E+06	.	-	.	ID=MALK_00552.t1;Parent=MALK_00552
contig001	maker	exon	1E+06	1E+06	.	-	.	ID=MALK_00552.t1.e1;Parent=MALK_00552.t1
contig001	AUGUSTUS	gene	1E+06	1E+06	0.69	+	.	ID=MALK_00553;prediction_source=braker_MRET:g1204.t1
contig001	AUGUSTUS	CDS	1E+06	1E+06	0.83	+	0	ID=MALK_00553.t1.c1;Parent=MALK_00553.t1
contig001	AUGUSTUS	CDS	1E+06	1E+06	0.83	+	0	ID=MALK_00553.t1.c2;Parent=MALK_00553.t1
contig001	AUGUSTUS	mRNA	1E+06	1E+06	0.69	+	.	ID=MALK_00553.t1;Parent=MALK_00553
contig001	AUGUSTUS	exon	1E+06	1E+06	.	+	.	ID=MALK_00553.t1.e1;Parent=MALK_00553.t1
contig001	AUGUSTUS	exon	1E+06	1E+06	.	+	.	ID=MALK_00553.t1.e2;Parent=MALK_00553.t1
contig001	AUGUSTUS	gene	1E+06	1E+06	0.73	+	.	ID=MALK_00554;prediction_source=augustus:contig001.g912.t1
contig001	AUGUSTUS	CDS	1E+06	1E+06	0.73	+	0	ID=MALK_00554.t1.c1;Parent=MALK_00554.t1
contig001	AUGUSTUS	mRNA	1E+06	1E+06	0.73	+	.	ID=MALK_00554.t1;Parent=MALK_00554
contig001	AUGUSTUS	exon	1E+06	1E+06	0.73	+	.	ID=MALK_00554.t1.e1;Parent=MALK_00554.t1
contig002	annotation	remark	1	408293	.	+	.	gff-version=3
contig002	maker	gene	440	3808	.	-	.	ID=MALK_00555;prediction_source=maker_MRET:augustus_masked-contig002-processed-gene-0.69-mRNA-1
contig002	maker	CDS	440	3808	.	-	0	ID=MALK_00555.t1.c1;Parent=MALK_00555.t1

contig002	maker	mRNA	440	3808	.	-	.	ID=MALK_00555.t1;Parent=MALK_00555
contig002	maker	exon	440	3808	.	-	.	ID=MALK_00555.t1.e1;Parent=MALK_00555.t1
contig002	maker	gene	4586	4957	.	+	.	ID=MALK_00556;prediction_source=maker_MRET:augustus_masked-contig002-processed-gene-0.52-mRNA-1
contig002	maker	CDS	4586	4957	.	+	0	ID=MALK_00556.t1.c1;Parent=MALK_00556.t1
contig002	maker	mRNA	4586	4957	.	+	.	ID=MALK_00556.t1;Parent=MALK_00556
contig002	maker	exon	4586	4957	.	+	.	ID=MALK_00556.t1.e1;Parent=MALK_00556.t1
contig002	maker	gene	5251	7722	.	+	.	ID=MALK_00557;prediction_source=maker_MRET:augustus_masked-contig002-processed-gene-0.53-mRNA-1
contig002	maker	CDS	5251	7722	.	+	0	ID=MALK_00557.t1.c1;Parent=MALK_00557.t1
contig002	maker	mRNA	5251	7722	.	+	.	ID=MALK_00557.t1;Parent=MALK_00557
contig002	maker	exon	5251	7722	.	+	.	ID=MALK_00557.t1.e1;Parent=MALK_00557.t1
contig002	AUGUSTUS	gene	7768	8877	0.94	-	.	ID=MALK_00558;prediction_source=augustus:contig002.g3971.t1
contig002	AUGUSTUS	CDS	7768	8877	0.94	-	0	ID=MALK_00558.t1.c1;Parent=MALK_00558.t1
contig002	AUGUSTUS	mRNA	7768	8877	0.94	-	.	ID=MALK_00558.t1;Parent=MALK_00558
contig002	AUGUSTUS	exon	7768	8877	0.94	-	.	ID=MALK_00558.t1.e1;Parent=MALK_00558.t1
contig002	maker	gene	9004	10842	.	-	.	ID=MALK_00559;prediction_source=maker_MRET:augustus_masked-contig002-processed-gene-0.71-mRNA-1
contig002	maker	CDS	9004	10842	.	-	0	ID=MALK_00559.t1.c1;Parent=MALK_00559.t1
contig002	maker	mRNA	9004	10842	.	-	.	ID=MALK_00559.t1;Parent=MALK_00559
contig002	maker	exon	9004	10842	.	-	.	ID=MALK_00559.t1.e1;Parent=MALK_00559.t1
contig002	AUGUSTUS	gene	11259	12200	0.79	+	.	ID=MALK_00560;prediction_source=braker_MRET:g119.t1
contig002	AUGUSTUS	CDS	11259	12200	0.79	+	0	ID=MALK_00560.t1.c1;Parent=MALK_00560.t1
contig002	AUGUSTUS	mRNA	11259	12200	0.79	+	.	ID=MALK_00560.t1;Parent=MALK_00560
contig002	AUGUSTUS	exon	11259	12200	.	+	.	ID=MALK_00560.t1.e1;Parent=MALK_00560.t1
contig002	AUGUSTUS	gene	12211	13857	0.98	-	.	ID=MALK_00561;prediction_source=augustus:contig002.g3974.t1
contig002	AUGUSTUS	CDS	12211	13857	0.98	-	0	ID=MALK_00561.t1.c1;Parent=MALK_00561.t1
contig002	AUGUSTUS	mRNA	12211	13857	0.98	-	.	ID=MALK_00561.t1;Parent=MALK_00561
contig002	AUGUSTUS	exon	12211	13857	0.98	-	.	ID=MALK_00561.t1.e1;Parent=MALK_00561.t1
contig002	AUGUSTUS	gene	14974	17034	0.96	-	.	ID=MALK_00562;prediction_source=braker_MRET:g121.t1
contig002	AUGUSTUS	CDS	14974	17034	0.96	-	0	ID=MALK_00562.t1.c1;Parent=MALK_00562.t1
contig002	AUGUSTUS	mRNA	14974	17034	0.96	-	.	ID=MALK_00562.t1;Parent=MALK_00562
contig002	AUGUSTUS	exon	14974	17034	.	-	.	ID=MALK_00562.t1.e1;Parent=MALK_00562.t1
contig002	AUGUSTUS	gene	17107	17889	0.97	+	.	ID=MALK_00563;prediction_source=braker_MRET:g122.t1
contig002	AUGUSTUS	CDS	17107	17889	0.97	+	0	ID=MALK_00563.t1.c1;Parent=MALK_00563.t1
contig002	AUGUSTUS	mRNA	17107	17889	0.97	+	.	ID=MALK_00563.t1;Parent=MALK_00563
contig002	AUGUSTUS	exon	17107	17889	.	+	.	ID=MALK_00563.t1.e1;Parent=MALK_00563.t1
contig002	AUGUSTUS	gene	17988	19430	0.43	+	.	ID=MALK_00564;prediction_source=augustus:contig002.g3976.t1
contig002	AUGUSTUS	CDS	17988	19430	0.43	+	0	ID=MALK_00564.t1.c1;Parent=MALK_00564.t1
contig002	AUGUSTUS	mRNA	17988	19430	0.43	+	.	ID=MALK_00564.t1;Parent=MALK_00564
contig002	AUGUSTUS	exon	17988	19430	0.43	+	.	ID=MALK_00564.t1.e1;Parent=MALK_00564.t1
contig002	AUGUSTUS	gene	19405	20853	1	-	.	ID=MALK_00565;prediction_source=augustus:contig002.g3977.t1
contig002	AUGUSTUS	CDS	19405	20853	1	-	0	ID=MALK_00565.t1.c1;Parent=MALK_00565.t1
contig002	AUGUSTUS	mRNA	19405	20853	1	-	.	ID=MALK_00565.t1;Parent=MALK_00565
contig002	AUGUSTUS	exon	19405	20853	1	-	.	ID=MALK_00565.t1.e1;Parent=MALK_00565.t1
contig002	AUGUSTUS	gene	20939	22691	0.66	+	.	ID=MALK_00566;prediction_source=braker_MRET:g125.t1

contig002	AUGUSTUS	CDS	20939	22531	0.7	+	0	ID=MALK_00566.t1.c1;Parent=MALK_00566.t1
contig002	AUGUSTUS	CDS	22617	22691	0.7	+	0	ID=MALK_00566.t1.c2;Parent=MALK_00566.t1
contig002	AUGUSTUS	mRNA	20939	22691	0.66	+	.	ID=MALK_00566.t1;Parent=MALK_00566
contig002	AUGUSTUS	exon	20939	22531	.	+	.	ID=MALK_00566.t1.e1;Parent=MALK_00566.t1
contig002	AUGUSTUS	exon	22617	22691	.	+	.	ID=MALK_00566.t1.e2;Parent=MALK_00566.t1
contig002	AUGUSTUS	gene	22734	24803	0.76	-	.	ID=MALK_00567;prediction_source=braker_MRET:g126.t1
contig002	AUGUSTUS	CDS	22734	24803	0.76	-	0	ID=MALK_00567.t1.c1;Parent=MALK_00567.t1
contig002	AUGUSTUS	mRNA	22734	24803	0.76	-	.	ID=MALK_00567.t1;Parent=MALK_00567
contig002	AUGUSTUS	exon	22734	24803	.	-	.	ID=MALK_00567.t1.e1;Parent=MALK_00567.t1
contig002	AUGUSTUS	gene	25838	27997	1	-	.	ID=MALK_00568;prediction_source=braker_MRET:g127.t1
contig002	AUGUSTUS	CDS	25838	27997	1	-	0	ID=MALK_00568.t1.c1;Parent=MALK_00568.t1
contig002	AUGUSTUS	mRNA	25838	27997	1	-	.	ID=MALK_00568.t1;Parent=MALK_00568
contig002	AUGUSTUS	exon	25838	27997	.	-	.	ID=MALK_00568.t1.e1;Parent=MALK_00568.t1
contig002	AUGUSTUS	gene	28111	31443	1	+	.	ID=MALK_00569;prediction_source=braker_MRET:g128.t1
contig002	AUGUSTUS	CDS	28111	31443	1	+	0	ID=MALK_00569.t1.c1;Parent=MALK_00569.t1
contig002	AUGUSTUS	mRNA	28111	31443	1	+	.	ID=MALK_00569.t1;Parent=MALK_00569
contig002	AUGUSTUS	exon	28111	31443	.	+	.	ID=MALK_00569.t1.e1;Parent=MALK_00569.t1
contig002	AUGUSTUS	gene	31525	32517	1	+	.	ID=MALK_00570;prediction_source=augustus:contig002.g3981.t1
contig002	AUGUSTUS	CDS	31525	32517	1	+	0	ID=MALK_00570.t1.c1;Parent=MALK_00570.t1
contig002	AUGUSTUS	mRNA	31525	32517	1	+	.	ID=MALK_00570.t1;Parent=MALK_00570
contig002	AUGUSTUS	exon	31525	32517	1	+	.	ID=MALK_00570.t1.e1;Parent=MALK_00570.t1
contig002	AUGUSTUS	gene	32545	34695	0.55	+	.	ID=MALK_00571;prediction_source=braker_MRET:g130.t1
contig002	AUGUSTUS	CDS	32545	34695	0.55	+	0	ID=MALK_00571.t1.c1;Parent=MALK_00571.t1
contig002	AUGUSTUS	mRNA	32545	34695	0.55	+	.	ID=MALK_00571.t1;Parent=MALK_00571
contig002	AUGUSTUS	exon	32545	34695	.	+	.	ID=MALK_00571.t1.e1;Parent=MALK_00571.t1
contig002	maker	gene	34749	35840	.	-	.	ID=MALK_00572;prediction_source=maker_MRET:augustus_masked-contig002-processed-gene-0.77-mRNA-1
contig002	maker	CDS	34749	35840	.	-	0	ID=MALK_00572.t1.c1;Parent=MALK_00572.t1
contig002	maker	mRNA	34749	35840	.	-	.	ID=MALK_00572.t1;Parent=MALK_00572
contig002	maker	exon	34749	35840	.	-	.	ID=MALK_00572.t1.e1;Parent=MALK_00572.t1
contig002	AUGUSTUS	gene	35924	38545	0.49	-	.	ID=MALK_00573;prediction_source=augustus:contig002.g3985.t1
contig002	AUGUSTUS	CDS	35924	38545	0.49	-	0	ID=MALK_00573.t1.c1;Parent=MALK_00573.t1
contig002	AUGUSTUS	mRNA	35924	38545	0.49	-	.	ID=MALK_00573.t1;Parent=MALK_00573
contig002	AUGUSTUS	exon	35924	38545	0.49	-	.	ID=MALK_00573.t1.e1;Parent=MALK_00573.t1
contig002	AUGUSTUS	gene	38638	41562	0.33	-	.	ID=MALK_00574;prediction_source=augustus:contig002.g3987.t1
contig002	AUGUSTUS	CDS	38638	41562	0.33	-	0	ID=MALK_00574.t1.c1;Parent=MALK_00574.t1
contig002	AUGUSTUS	mRNA	38638	41562	0.33	-	.	ID=MALK_00574.t1;Parent=MALK_00574
contig002	AUGUSTUS	exon	38638	41562	0.33	-	.	ID=MALK_00574.t1.e1;Parent=MALK_00574.t1
contig002	AUGUSTUS	gene	41739	42203	0.61	-	.	ID=MALK_00575;prediction_source=braker_MRET:g133.t1
contig002	AUGUSTUS	CDS	41739	42203	0.61	-	0	ID=MALK_00575.t1.c1;Parent=MALK_00575.t1
contig002	AUGUSTUS	mRNA	41739	42203	0.61	-	.	ID=MALK_00575.t1;Parent=MALK_00575
contig002	AUGUSTUS	exon	41739	42203	.	-	.	ID=MALK_00575.t1.e1;Parent=MALK_00575.t1
contig002	AUGUSTUS	gene	42344	43525	0.71	+	.	ID=MALK_00576;prediction_source=augustus:contig002.g3989.t1
contig002	AUGUSTUS	CDS	42344	43525	0.71	+	0	ID=MALK_00576.t1.c1;Parent=MALK_00576.t1

contig002	AUGUSTUS	mRNA	42344	43525	0.71	+	.	ID=MALK_00576.t1;Parent=MALK_00576
contig002	AUGUSTUS	exon	42344	43525	0.71	+	.	ID=MALK_00576.t1.e1;Parent=MALK_00576.t1
contig002	AUGUSTUS	gene	43526	46084	0.51	-	.	ID=MALK_00577;prediction_source=augustus:contig002.g3990.t1
contig002	AUGUSTUS	CDS	43526	46084	0.51	-	0	ID=MALK_00577.t1.c1;Parent=MALK_00577.t1
contig002	AUGUSTUS	mRNA	43526	46084	0.51	-	.	ID=MALK_00577.t1;Parent=MALK_00577
contig002	AUGUSTUS	exon	43526	46084	0.51	-	.	ID=MALK_00577.t1.e1;Parent=MALK_00577.t1
contig002	maker	gene	46183	47874	.	-	.	ID=MALK_00578;prediction_source=maker_MRET:augustus_masked-contig002-processed-gene-0.80-mRNA-1
contig002	maker	CDS	46183	47874	.	-	0	ID=MALK_00578.t1.c1;Parent=MALK_00578.t1
contig002	maker	mRNA	46183	47874	.	-	.	ID=MALK_00578.t1;Parent=MALK_00578
contig002	maker	exon	46183	47874	.	-	.	ID=MALK_00578.t1.e1;Parent=MALK_00578.t1
contig002	maker	gene	47987	49381	.	-	.	ID=MALK_00579;prediction_source=maker_MRET:augustus_masked-contig002-processed-gene-0.81-mRNA-1
contig002	maker	CDS	47987	49381	.	-	0	ID=MALK_00579.t1.c1;Parent=MALK_00579.t1
contig002	maker	mRNA	47987	49381	.	-	.	ID=MALK_00579.t1;Parent=MALK_00579
contig002	maker	exon	47987	49381	.	-	.	ID=MALK_00579.t1.e1;Parent=MALK_00579.t1
contig002	AUGUSTUS	gene	49493	50729	0.96	-	.	ID=MALK_00580;prediction_source=braker_MRET:g138.t1
contig002	AUGUSTUS	CDS	50459	50729	0.99	-	0	ID=MALK_00580.t1.c2;Parent=MALK_00580.t1
contig002	AUGUSTUS	CDS	49493	50427	0.99	-	0	ID=MALK_00580.t1.c1;Parent=MALK_00580.t1
contig002	AUGUSTUS	mRNA	49493	50729	0.96	-	.	ID=MALK_00580.t1;Parent=MALK_00580
contig002	AUGUSTUS	exon	50459	50729	.	-	.	ID=MALK_00580.t1.e2;Parent=MALK_00580.t1
contig002	AUGUSTUS	exon	49493	50427	.	-	.	ID=MALK_00580.t1.e1;Parent=MALK_00580.t1
contig002	AUGUSTUS	gene	50801	52321	0.79	+	.	ID=MALK_00581;prediction_source=braker_MRET:g139.t1
contig002	AUGUSTUS	CDS	50801	52321	0.79	+	0	ID=MALK_00581.t1.c1;Parent=MALK_00581.t1
contig002	AUGUSTUS	mRNA	50801	52321	0.79	+	.	ID=MALK_00581.t1;Parent=MALK_00581
contig002	AUGUSTUS	exon	50801	52321	.	+	.	ID=MALK_00581.t1.e1;Parent=MALK_00581.t1
contig002	AUGUSTUS	gene	52318	54210	0.87	-	.	ID=MALK_00582;prediction_source=augustus:contig002.g3998.t1
contig002	AUGUSTUS	CDS	52318	54210	0.87	-	0	ID=MALK_00582.t1.c1;Parent=MALK_00582.t1
contig002	AUGUSTUS	mRNA	52318	54210	0.87	-	.	ID=MALK_00582.t1;Parent=MALK_00582
contig002	AUGUSTUS	exon	52318	54210	0.87	-	.	ID=MALK_00582.t1.e1;Parent=MALK_00582.t1
contig002	maker	gene	54209	55174	.	+	.	ID=MALK_00583;prediction_source=maker_MRET:augustus_masked-contig002-processed-gene-0.59-mRNA-1
contig002	maker	CDS	54209	55174	.	+	0	ID=MALK_00583.t1.c1;Parent=MALK_00583.t1
contig002	maker	mRNA	54209	55174	.	+	.	ID=MALK_00583.t1;Parent=MALK_00583
contig002	maker	exon	54209	55174	.	+	.	ID=MALK_00583.t1.e1;Parent=MALK_00583.t1
contig002	AUGUSTUS	gene	55215	56285	0.95	+	.	ID=MALK_00584;prediction_source=augustus:contig002.g4000.t1
contig002	AUGUSTUS	CDS	55215	56285	0.95	+	0	ID=MALK_00584.t1.c1;Parent=MALK_00584.t1
contig002	AUGUSTUS	mRNA	55215	56285	0.95	+	.	ID=MALK_00584.t1;Parent=MALK_00584
contig002	AUGUSTUS	exon	55215	56285	0.95	+	.	ID=MALK_00584.t1.e1;Parent=MALK_00584.t1
contig002	AUGUSTUS	gene	56454	56732	0.54	-	.	ID=MALK_00585;prediction_source=augustus:contig002.g4002.t1
contig002	AUGUSTUS	CDS	56454	56732	0.54	-	0	ID=MALK_00585.t1.c1;Parent=MALK_00585.t1
contig002	AUGUSTUS	mRNA	56454	56732	0.54	-	.	ID=MALK_00585.t1;Parent=MALK_00585
contig002	AUGUSTUS	exon	56454	56732	0.54	-	.	ID=MALK_00585.t1.e1;Parent=MALK_00585.t1
contig002	maker	gene	56893	58074	.	-	.	ID=MALK_00586;prediction_source=maker_MRET:augustus_masked-contig002-processed-gene-0.85-mRNA-1
contig002	maker	CDS	56893	58074	.	-	0	ID=MALK_00586.t1.c1;Parent=MALK_00586.t1
contig002	maker	mRNA	56893	58074	.	-	.	ID=MALK_00586.t1;Parent=MALK_00586

contig002	maker	exon	56893	58074	.	-	.	ID=MALK_00586.t1.e1;Parent=MALK_00586.t1
contig002	AUGUSTUS	gene	58207	59470	0.65	-	.	ID=MALK_00587;prediction_source=braker_MRET:g145.t1
contig002	AUGUSTUS	CDS	59467	59470	0.67	-	0	ID=MALK_00587.t1.c3;Parent=MALK_00587.t1
contig002	AUGUSTUS	CDS	59339	59386	0.67	-	0	ID=MALK_00587.t1.c2;Parent=MALK_00587.t1
contig002	AUGUSTUS	CDS	58207	59306	0.67	-	0	ID=MALK_00587.t1.c1;Parent=MALK_00587.t1
contig002	AUGUSTUS	mRNA	58207	59470	0.65	-	.	ID=MALK_00587.t1;Parent=MALK_00587
contig002	AUGUSTUS	exon	59467	59470	.	-	.	ID=MALK_00587.t1.e3;Parent=MALK_00587.t1
contig002	AUGUSTUS	exon	59339	59386	.	-	.	ID=MALK_00587.t1.e2;Parent=MALK_00587.t1
contig002	AUGUSTUS	exon	58207	59306	.	-	.	ID=MALK_00587.t1.e1;Parent=MALK_00587.t1
contig002	AUGUSTUS	gene	60059	61543	0.67	+	.	ID=MALK_00588;prediction_source=braker_MRET:g146.t1
contig002	AUGUSTUS	CDS	60059	60253	0.99	+	0	ID=MALK_00588.t1.c1;Parent=MALK_00588.t1
contig002	AUGUSTUS	CDS	60310	60462	0.99	+	0	ID=MALK_00588.t1.c2;Parent=MALK_00588.t1
contig002	AUGUSTUS	CDS	60491	61543	0.99	+	0	ID=MALK_00588.t1.c3;Parent=MALK_00588.t1
contig002	AUGUSTUS	mRNA	60059	61543	0.67	+	.	ID=MALK_00588.t1;Parent=MALK_00588
contig002	AUGUSTUS	exon	60059	60253	.	+	.	ID=MALK_00588.t1.e1;Parent=MALK_00588.t1
contig002	AUGUSTUS	exon	60310	60462	.	+	.	ID=MALK_00588.t1.e2;Parent=MALK_00588.t1
contig002	AUGUSTUS	exon	60491	61543	.	+	.	ID=MALK_00588.t1.e3;Parent=MALK_00588.t1
contig002	AUGUSTUS	gene	61554	64676	0.6	-	.	ID=MALK_00589;prediction_source=braker_MRET:g147.t1
contig002	AUGUSTUS	CDS	61554	64676	0.6	-	0	ID=MALK_00589.t1.c1;Parent=MALK_00589.t1
contig002	AUGUSTUS	mRNA	61554	64676	0.6	-	.	ID=MALK_00589.t1;Parent=MALK_00589
contig002	AUGUSTUS	exon	61554	64676	.	-	.	ID=MALK_00589.t1.e1;Parent=MALK_00589.t1
contig002	AUGUSTUS	gene	65058	67802	0.97	+	.	ID=MALK_00590;prediction_source=augustus:contig002.g4010.t1
contig002	AUGUSTUS	CDS	65058	67802	0.97	+	0	ID=MALK_00590.t1.c1;Parent=MALK_00590.t1
contig002	AUGUSTUS	mRNA	65058	67802	0.97	+	.	ID=MALK_00590.t1;Parent=MALK_00590
contig002	AUGUSTUS	exon	65058	67802	0.97	+	.	ID=MALK_00590.t1.e1;Parent=MALK_00590.t1
contig002	maker	gene	67907	68422	.	-	.	ID=MALK_00591;prediction_source=maker_MRET:augustus_masked-contig002-processed-gene-0.88-mRNA-1
contig002	maker	CDS	67907	68422	.	-	0	ID=MALK_00591.t1.c1;Parent=MALK_00591.t1
contig002	maker	mRNA	67907	68422	.	-	.	ID=MALK_00591.t1;Parent=MALK_00591
contig002	maker	exon	67907	68422	.	-	.	ID=MALK_00591.t1.e1;Parent=MALK_00591.t1
contig002	maker	gene	68694	70382	.	+	.	ID=MALK_00592;prediction_source=maker_MRET:augustus_masked-contig002-processed-gene-0.64-mRNA-1
contig002	maker	CDS	68694	70382	.	+	0	ID=MALK_00592.t1.c1;Parent=MALK_00592.t1
contig002	maker	mRNA	68694	70382	.	+	.	ID=MALK_00592.t1;Parent=MALK_00592
contig002	maker	exon	68694	70382	.	+	.	ID=MALK_00592.t1.e1;Parent=MALK_00592.t1
contig002	AUGUSTUS	gene	70547	70965	0.99	-	.	ID=MALK_00593;prediction_source=braker_MRET:g151.t1
contig002	AUGUSTUS	CDS	70751	70965	1	-	0	ID=MALK_00593.t1.c2;Parent=MALK_00593.t1
contig002	AUGUSTUS	CDS	70547	70718	1	-	0	ID=MALK_00593.t1.c1;Parent=MALK_00593.t1
contig002	AUGUSTUS	mRNA	70547	70965	0.99	-	.	ID=MALK_00593.t1;Parent=MALK_00593
contig002	AUGUSTUS	exon	70751	70965	.	-	.	ID=MALK_00593.t1.e2;Parent=MALK_00593.t1
contig002	AUGUSTUS	exon	70547	70718	.	-	.	ID=MALK_00593.t1.e1;Parent=MALK_00593.t1
contig002	AUGUSTUS	gene	71158	72402	0.99	+	.	ID=MALK_00594;prediction_source=braker_MRET:g152.t1
contig002	AUGUSTUS	CDS	71158	72402	0.99	+	0	ID=MALK_00594.t1.c1;Parent=MALK_00594.t1
contig002	AUGUSTUS	mRNA	71158	72402	0.99	+	.	ID=MALK_00594.t1;Parent=MALK_00594
contig002	AUGUSTUS	exon	71158	72402	.	+	.	ID=MALK_00594.t1.e1;Parent=MALK_00594.t1

contig002	AUGUSTUS	gene	72425	73861	0.69	-	.	ID=MALK_00595;prediction_source=augustus:contig002.g4014.t1
contig002	AUGUSTUS	CDS	72425	73861	0.69	-	0	ID=MALK_00595.t1.c1;Parent=MALK_00595.t1
contig002	AUGUSTUS	mRNA	72425	73861	0.69	-	.	ID=MALK_00595.t1;Parent=MALK_00595
contig002	AUGUSTUS	exon	72425	73861	0.69	-	.	ID=MALK_00595.t1.e1;Parent=MALK_00595.t1
contig002	AUGUSTUS	gene	74080	76899	0.82	+	.	ID=MALK_00596;prediction_source=augustus:contig002.g4015.t1
contig002	AUGUSTUS	CDS	74080	76899	0.82	+	0	ID=MALK_00596.t1.c1;Parent=MALK_00596.t1
contig002	AUGUSTUS	mRNA	74080	76899	0.82	+	.	ID=MALK_00596.t1;Parent=MALK_00596
contig002	AUGUSTUS	exon	74080	76899	0.82	+	.	ID=MALK_00596.t1.e1;Parent=MALK_00596.t1
contig002	AUGUSTUS	gene	76955	77896	0.76	-	.	ID=MALK_00597;prediction_source=braker_MRET:g155.t1
contig002	AUGUSTUS	CDS	76955	77896	0.76	-	0	ID=MALK_00597.t1.c1;Parent=MALK_00597.t1
contig002	AUGUSTUS	mRNA	76955	77896	0.76	-	.	ID=MALK_00597.t1;Parent=MALK_00597
contig002	AUGUSTUS	exon	76955	77896	.	-	.	ID=MALK_00597.t1.e1;Parent=MALK_00597.t1
contig002	AUGUSTUS	gene	77953	80268	0.63	-	.	ID=MALK_00598;prediction_source=braker_MRET:g156.t1
contig002	AUGUSTUS	CDS	77953	80268	0.63	-	0	ID=MALK_00598.t1.c1;Parent=MALK_00598.t1
contig002	AUGUSTUS	mRNA	77953	80268	0.63	-	.	ID=MALK_00598.t1;Parent=MALK_00598
contig002	AUGUSTUS	exon	77953	80268	.	-	.	ID=MALK_00598.t1.e1;Parent=MALK_00598.t1
contig002	AUGUSTUS	gene	80375	81712	0.73	+	.	ID=MALK_00599;prediction_source=augustus:contig002.g4017.t1
contig002	AUGUSTUS	CDS	80375	81712	0.73	+	0	ID=MALK_00599.t1.c1;Parent=MALK_00599.t1
contig002	AUGUSTUS	mRNA	80375	81712	0.73	+	.	ID=MALK_00599.t1;Parent=MALK_00599
contig002	AUGUSTUS	exon	80375	81712	0.73	+	.	ID=MALK_00599.t1.e1;Parent=MALK_00599.t1
contig002	AUGUSTUS	gene	81758	84066	0.41	-	.	ID=MALK_00600;prediction_source=braker_MRET:g158.t1
contig002	AUGUSTUS	CDS	81850	84066	0.41	-	0	ID=MALK_00600.t1.c2;Parent=MALK_00600.t1
contig002	AUGUSTUS	CDS	81758	81769	0.41	-	0	ID=MALK_00600.t1.c1;Parent=MALK_00600.t1
contig002	AUGUSTUS	mRNA	81758	84066	0.41	-	.	ID=MALK_00600.t1;Parent=MALK_00600
contig002	AUGUSTUS	exon	81850	84066	.	-	.	ID=MALK_00600.t1.e2;Parent=MALK_00600.t1
contig002	AUGUSTUS	exon	81758	81769	.	-	.	ID=MALK_00600.t1.e1;Parent=MALK_00600.t1
contig002	AUGUSTUS	gene	84172	85549	0.33	+	.	ID=MALK_00601;prediction_source=augustus:contig002.g4018.t1
contig002	AUGUSTUS	CDS	84172	84192	0.35	+	0	ID=MALK_00601.t1.c1;Parent=MALK_00601.t1
contig002	AUGUSTUS	CDS	84311	85549	0.35	+	0	ID=MALK_00601.t1.c2;Parent=MALK_00601.t1
contig002	AUGUSTUS	mRNA	84172	85549	0.33	+	.	ID=MALK_00601.t1;Parent=MALK_00601
contig002	AUGUSTUS	exon	84172	84192	0.35	+	.	ID=MALK_00601.t1.e1;Parent=MALK_00601.t1
contig002	AUGUSTUS	exon	84311	85549	0.35	+	.	ID=MALK_00601.t1.e2;Parent=MALK_00601.t1
contig002	AUGUSTUS	gene	85700	86203	0.3	+	.	ID=MALK_00602;prediction_source=braker_MRET:g160.t1
contig002	AUGUSTUS	CDS	85700	85911	0.74	+	0	ID=MALK_00602.t1.c1;Parent=MALK_00602.t1
contig002	AUGUSTUS	CDS	86110	86203	0.74	+	0	ID=MALK_00602.t1.c2;Parent=MALK_00602.t1
contig002	AUGUSTUS	mRNA	85700	86203	0.3	+	.	ID=MALK_00602.t1;Parent=MALK_00602
contig002	AUGUSTUS	exon	85700	85911	.	+	.	ID=MALK_00602.t1.e1;Parent=MALK_00602.t1
contig002	AUGUSTUS	exon	86110	86203	.	+	.	ID=MALK_00602.t1.e2;Parent=MALK_00602.t1
contig002	maker	gene	86464	86826	.	-	.	ID=MALK_00603;prediction_source=maker_MRET:augustus_masked-contig002-processed-gene-0.91-mRNA-1
contig002	maker	CDS	86464	86826	.	-	0	ID=MALK_00603.t1.c1;Parent=MALK_00603.t1
contig002	maker	mRNA	86464	86826	.	-	.	ID=MALK_00603.t1;Parent=MALK_00603
contig002	maker	exon	86464	86826	.	-	.	ID=MALK_00603.t1.e1;Parent=MALK_00603.t1
contig002	AUGUSTUS	gene	87095	87371	0.72	+	.	ID=MALK_00604;prediction_source=braker_MRET:g162.t1

contig002	AUGUSTUS	CDS	87095	87322	0.75	+	0	ID=MALK_00604.t1.c1;Parent=MALK_00604.t1
contig002	AUGUSTUS	CDS	87360	87371	0.75	+	0	ID=MALK_00604.t1.c2;Parent=MALK_00604.t1
contig002	AUGUSTUS	mRNA	87095	87371	0.72	+	.	ID=MALK_00604.t1;Parent=MALK_00604
contig002	AUGUSTUS	exon	87095	87322	.	+	.	ID=MALK_00604.t1.e1;Parent=MALK_00604.t1
contig002	AUGUSTUS	exon	87360	87371	.	+	.	ID=MALK_00604.t1.e2;Parent=MALK_00604.t1
contig002	maker	gene	87377	90151	.	-	.	ID=MALK_00605;prediction_source=maker_MRET:augustus_masked-contig002-processed-gene-0.92-mRNA-1
contig002	maker	CDS	87377	90151	.	-	0	ID=MALK_00605.t1.c1;Parent=MALK_00605.t1
contig002	maker	mRNA	87377	90151	.	-	.	ID=MALK_00605.t1;Parent=MALK_00605
contig002	maker	exon	87377	90151	.	-	.	ID=MALK_00605.t1.e1;Parent=MALK_00605.t1
contig002	maker	gene	90422	91465	.	-	.	ID=MALK_00606;prediction_source=maker_MRET:augustus_masked-contig002-processed-gene-0.93-mRNA-1
contig002	maker	CDS	90422	91465	.	-	0	ID=MALK_00606.t1.c1;Parent=MALK_00606.t1
contig002	maker	mRNA	90422	91465	.	-	.	ID=MALK_00606.t1;Parent=MALK_00606
contig002	maker	exon	90422	91465	.	-	.	ID=MALK_00606.t1.e1;Parent=MALK_00606.t1
contig002	AUGUSTUS	gene	91583	95884	0.68	+	.	ID=MALK_00607;prediction_source=braker_MRET:g165.t1
contig002	AUGUSTUS	CDS	91583	95884	0.68	+	0	ID=MALK_00607.t1.c1;Parent=MALK_00607.t1
contig002	AUGUSTUS	mRNA	91583	95884	0.68	+	.	ID=MALK_00607.t1;Parent=MALK_00607
contig002	AUGUSTUS	exon	91583	95884	.	+	.	ID=MALK_00607.t1.e1;Parent=MALK_00607.t1
contig002	AUGUSTUS	gene	95886	96839	0.44	-	.	ID=MALK_00608;prediction_source=augustus:contig002.g4024.t1
contig002	AUGUSTUS	CDS	95886	96839	0.44	-	0	ID=MALK_00608.t1.c1;Parent=MALK_00608.t1
contig002	AUGUSTUS	mRNA	95886	96839	0.44	-	.	ID=MALK_00608.t1;Parent=MALK_00608
contig002	AUGUSTUS	exon	95886	96839	0.44	-	.	ID=MALK_00608.t1.e1;Parent=MALK_00608.t1
contig002	maker	gene	97097	99214	.	+	.	ID=MALK_00609;prediction_source=maker_MRET:augustus_masked-contig002-processed-gene-1.47-mRNA-1
contig002	maker	CDS	97097	99214	.	+	0	ID=MALK_00609.t1.c1;Parent=MALK_00609.t1
contig002	maker	mRNA	97097	99214	.	+	.	ID=MALK_00609.t1;Parent=MALK_00609
contig002	maker	exon	97097	99214	.	+	.	ID=MALK_00609.t1.e1;Parent=MALK_00609.t1
contig002	AUGUSTUS	gene	99223	100050	1	-	.	ID=MALK_00610;prediction_source=braker_MRET:g168.t1
contig002	AUGUSTUS	CDS	99223	100050	1	-	0	ID=MALK_00610.t1.c1;Parent=MALK_00610.t1
contig002	AUGUSTUS	mRNA	99223	100050	1	-	.	ID=MALK_00610.t1;Parent=MALK_00610
contig002	AUGUSTUS	exon	99223	100050	.	-	.	ID=MALK_00610.t1.e1;Parent=MALK_00610.t1
contig002	AUGUSTUS	gene	100311	103217	0.99	+	.	ID=MALK_00611;prediction_source=augustus:contig002.g4026.t1
contig002	AUGUSTUS	CDS	100311	103217	0.99	+	0	ID=MALK_00611.t1.c1;Parent=MALK_00611.t1
contig002	AUGUSTUS	mRNA	100311	103217	0.99	+	.	ID=MALK_00611.t1;Parent=MALK_00611
contig002	AUGUSTUS	exon	100311	103217	0.99	+	.	ID=MALK_00611.t1.e1;Parent=MALK_00611.t1
contig002	AUGUSTUS	gene	103240	104328	1	-	.	ID=MALK_00612;prediction_source=braker_MRET:g170.t1
contig002	AUGUSTUS	CDS	103240	104328	1	-	0	ID=MALK_00612.t1.c1;Parent=MALK_00612.t1
contig002	AUGUSTUS	mRNA	103240	104328	1	-	.	ID=MALK_00612.t1;Parent=MALK_00612
contig002	AUGUSTUS	exon	103240	104328	.	-	.	ID=MALK_00612.t1.e1;Parent=MALK_00612.t1
contig002	maker	gene	104413	106773	.	+	.	ID=MALK_00613;prediction_source=maker_MRET:augustus_masked-contig002-processed-gene-1.50-mRNA-1
contig002	maker	CDS	104413	106773	.	+	0	ID=MALK_00613.t1.c1;Parent=MALK_00613.t1
contig002	maker	mRNA	104413	106773	.	+	.	ID=MALK_00613.t1;Parent=MALK_00613
contig002	maker	exon	104413	106773	.	+	.	ID=MALK_00613.t1.e1;Parent=MALK_00613.t1
contig002	AUGUSTUS	gene	106770	108206	0.27	-	.	ID=MALK_00614;prediction_source=augustus:contig002.g4029.t1
contig002	AUGUSTUS	CDS	106770	108206	0.27	-	0	ID=MALK_00614.t1.c1;Parent=MALK_00614.t1

contig002	AUGUSTUS	mRNA	106770	108206	0.27	-	.	ID=MALK_00614.t1;Parent=MALK_00614
contig002	AUGUSTUS	exon	106770	108206	0.27	-	.	ID=MALK_00614.t1.e1;Parent=MALK_00614.t1
contig002	maker	gene	108632	110878	.	-	.	ID=MALK_00615;prediction_source=maker_MRET:augustus_masked-contig002-processed-gene-1.69-mRNA-1
contig002	maker	CDS	108632	110878	.	-	0	ID=MALK_00615.t1.c1;Parent=MALK_00615.t1
contig002	maker	mRNA	108632	110878	.	-	.	ID=MALK_00615.t1;Parent=MALK_00615
contig002	maker	exon	108632	110878	.	-	.	ID=MALK_00615.t1.e1;Parent=MALK_00615.t1
contig002	AUGUSTUS	gene	110918	111808	0.44	-	.	ID=MALK_00616;prediction_source=augustus:contig002.g4031.t1
contig002	AUGUSTUS	CDS	110918	111808	0.44	-	0	ID=MALK_00616.t1.c1;Parent=MALK_00616.t1
contig002	AUGUSTUS	mRNA	110918	111808	0.44	-	.	ID=MALK_00616.t1;Parent=MALK_00616
contig002	AUGUSTUS	exon	110918	111808	0.44	-	.	ID=MALK_00616.t1.e1;Parent=MALK_00616.t1
contig002	AUGUSTUS	gene	112189	115929	0.72	+	.	ID=MALK_00617;prediction_source=braker_MRET:g175.t1
contig002	AUGUSTUS	CDS	112189	115929	0.72	+	0	ID=MALK_00617.t1.c1;Parent=MALK_00617.t1
contig002	AUGUSTUS	mRNA	112189	115929	0.72	+	.	ID=MALK_00617.t1;Parent=MALK_00617
contig002	AUGUSTUS	exon	112189	115929	.	+	.	ID=MALK_00617.t1.e1;Parent=MALK_00617.t1
contig002	AUGUSTUS	gene	115964	116269	0.83	+	.	ID=MALK_00618;prediction_source=braker_MRET:g176.t1
contig002	AUGUSTUS	CDS	115964	116269	0.83	+	0	ID=MALK_00618.t1.c1;Parent=MALK_00618.t1
contig002	AUGUSTUS	mRNA	115964	116269	0.83	+	.	ID=MALK_00618.t1;Parent=MALK_00618
contig002	AUGUSTUS	exon	115964	116269	.	+	.	ID=MALK_00618.t1.e1;Parent=MALK_00618.t1
contig002	AUGUSTUS	gene	116348	116779	0.98	+	.	ID=MALK_00619;prediction_source=braker_MRET:g177.t1
contig002	AUGUSTUS	CDS	116348	116779	0.98	+	0	ID=MALK_00619.t1.c1;Parent=MALK_00619.t1
contig002	AUGUSTUS	mRNA	116348	116779	0.98	+	.	ID=MALK_00619.t1;Parent=MALK_00619
contig002	AUGUSTUS	exon	116348	116779	.	+	.	ID=MALK_00619.t1.e1;Parent=MALK_00619.t1
contig002	AUGUSTUS	gene	116793	117449	0.7	-	.	ID=MALK_00620;prediction_source=augustus:contig002.g4033.t1
contig002	AUGUSTUS	CDS	116793	117449	0.7	-	0	ID=MALK_00620.t1.c1;Parent=MALK_00620.t1
contig002	AUGUSTUS	mRNA	116793	117449	0.7	-	.	ID=MALK_00620.t1;Parent=MALK_00620
contig002	AUGUSTUS	exon	116793	117449	0.7	-	.	ID=MALK_00620.t1.e1;Parent=MALK_00620.t1
contig002	AUGUSTUS	gene	117613	120918	0.96	+	.	ID=MALK_00621;prediction_source=braker_MRET:g179.t1
contig002	AUGUSTUS	CDS	117613	120918	0.96	+	0	ID=MALK_00621.t1.c1;Parent=MALK_00621.t1
contig002	AUGUSTUS	mRNA	117613	120918	0.96	+	.	ID=MALK_00621.t1;Parent=MALK_00621
contig002	AUGUSTUS	exon	117613	120918	.	+	.	ID=MALK_00621.t1.e1;Parent=MALK_00621.t1
contig002	maker	gene	120915	122843	.	-	.	ID=MALK_00622;prediction_source=maker_MRET:augustus_masked-contig002-processed-gene-1.71-mRNA-1
contig002	maker	CDS	120915	122843	.	-	0	ID=MALK_00622.t1.c1;Parent=MALK_00622.t1
contig002	maker	mRNA	120915	122843	.	-	.	ID=MALK_00622.t1;Parent=MALK_00622
contig002	maker	exon	120915	122843	.	-	.	ID=MALK_00622.t1.e1;Parent=MALK_00622.t1
contig002	AUGUSTUS	gene	122945	123796	0.62	-	.	ID=MALK_00623;prediction_source=braker_MRET:g181.t1
contig002	AUGUSTUS	CDS	122945	123796	0.62	-	0	ID=MALK_00623.t1.c1;Parent=MALK_00623.t1
contig002	AUGUSTUS	mRNA	122945	123796	0.62	-	.	ID=MALK_00623.t1;Parent=MALK_00623
contig002	AUGUSTUS	exon	122945	123796	.	-	.	ID=MALK_00623.t1.e1;Parent=MALK_00623.t1
contig002	AUGUSTUS	gene	123844	125133	0.97	+	.	ID=MALK_00624;prediction_source=braker_MRET:g182.t1
contig002	AUGUSTUS	CDS	123844	125133	0.97	+	0	ID=MALK_00624.t1.c1;Parent=MALK_00624.t1
contig002	AUGUSTUS	mRNA	123844	125133	0.97	+	.	ID=MALK_00624.t1;Parent=MALK_00624
contig002	AUGUSTUS	exon	123844	125133	.	+	.	ID=MALK_00624.t1.e1;Parent=MALK_00624.t1
contig002	AUGUSTUS	gene	125093	126355	0.99	-	.	ID=MALK_00625;prediction_source=augustus:contig002.g4039.t1

contig002	AUGUSTUS	CDS	125093	126355	0.99	-	0	ID=MALK_00625.t1.c1;Parent=MALK_00625.t1
contig002	AUGUSTUS	mRNA	125093	126355	0.99	-	.	ID=MALK_00625.t1;Parent=MALK_00625
contig002	AUGUSTUS	exon	125093	126355	0.99	-	.	ID=MALK_00625.t1.e1;Parent=MALK_00625.t1
contig002	AUGUSTUS	gene	126420	128096	0.44	+	.	ID=MALK_00626;prediction_source=augustus:contig002.g4040.t1
contig002	AUGUSTUS	CDS	126420	128096	0.44	+	0	ID=MALK_00626.t1.c1;Parent=MALK_00626.t1
contig002	AUGUSTUS	mRNA	126420	128096	0.44	+	.	ID=MALK_00626.t1;Parent=MALK_00626
contig002	AUGUSTUS	exon	126420	128096	0.44	+	.	ID=MALK_00626.t1.e1;Parent=MALK_00626.t1
contig002	AUGUSTUS	gene	128729	130954	0.92	+	.	ID=MALK_00627;prediction_source=augustus:contig002.g4041.t1
contig002	AUGUSTUS	CDS	128729	130954	0.92	+	0	ID=MALK_00627.t1.c1;Parent=MALK_00627.t1
contig002	AUGUSTUS	mRNA	128729	130954	0.92	+	.	ID=MALK_00627.t1;Parent=MALK_00627
contig002	AUGUSTUS	exon	128729	130954	0.92	+	.	ID=MALK_00627.t1.e1;Parent=MALK_00627.t1
contig002	AUGUSTUS	gene	131238	133580	0.89	+	.	ID=MALK_00628;prediction_source=braker_MRET:g186.t1
contig002	AUGUSTUS	CDS	131238	133580	0.89	+	0	ID=MALK_00628.t1.c1;Parent=MALK_00628.t1
contig002	AUGUSTUS	mRNA	131238	133580	0.89	+	.	ID=MALK_00628.t1;Parent=MALK_00628
contig002	AUGUSTUS	exon	131238	133580	.	+	.	ID=MALK_00628.t1.e1;Parent=MALK_00628.t1
contig002	AUGUSTUS	gene	133609	133977	0.62	-	.	ID=MALK_00629;prediction_source=braker_MRET:g187.t1
contig002	AUGUSTUS	CDS	133609	133977	0.62	-	0	ID=MALK_00629.t1.c1;Parent=MALK_00629.t1
contig002	AUGUSTUS	mRNA	133609	133977	0.62	-	.	ID=MALK_00629.t1;Parent=MALK_00629
contig002	AUGUSTUS	exon	133609	133977	.	-	.	ID=MALK_00629.t1.e1;Parent=MALK_00629.t1
contig002	AUGUSTUS	gene	134116	135480	0.75	-	.	ID=MALK_00630;prediction_source=braker_MRET:g188.t1
contig002	AUGUSTUS	CDS	134116	135480	0.75	-	0	ID=MALK_00630.t1.c1;Parent=MALK_00630.t1
contig002	AUGUSTUS	mRNA	134116	135480	0.75	-	.	ID=MALK_00630.t1;Parent=MALK_00630
contig002	AUGUSTUS	exon	134116	135480	.	-	.	ID=MALK_00630.t1.e1;Parent=MALK_00630.t1
contig002	maker	gene	135667	138996	.	-	.	ID=MALK_00631;prediction_source=maker_MRET:augustus_masked-contig002-processed-gene-1.74-mRNA-1
contig002	maker	CDS	135667	138996	.	-	0	ID=MALK_00631.t1.c1;Parent=MALK_00631.t1
contig002	maker	mRNA	135667	138996	.	-	.	ID=MALK_00631.t1;Parent=MALK_00631
contig002	maker	exon	135667	138996	.	-	.	ID=MALK_00631.t1.e1;Parent=MALK_00631.t1
contig002	AUGUSTUS	gene	139173	142951	0.62	+	.	ID=MALK_00632;prediction_source=braker_MRET:g190.t1
contig002	AUGUSTUS	CDS	139173	142809	0.62	+	0	ID=MALK_00632.t1.c1;Parent=MALK_00632.t1
contig002	AUGUSTUS	CDS	142908	142951	0.62	+	0	ID=MALK_00632.t1.c2;Parent=MALK_00632.t1
contig002	AUGUSTUS	mRNA	139173	142951	0.62	+	.	ID=MALK_00632.t1;Parent=MALK_00632
contig002	AUGUSTUS	exon	139173	142809	.	+	.	ID=MALK_00632.t1.e1;Parent=MALK_00632.t1
contig002	AUGUSTUS	exon	142908	142951	.	+	.	ID=MALK_00632.t1.e2;Parent=MALK_00632.t1
contig002	AUGUSTUS	gene	142957	143869	0.31	-	.	ID=MALK_00633;prediction_source=braker_MRET:g191.t1
contig002	AUGUSTUS	CDS	143781	143869	0.45	-	0	ID=MALK_00633.t1.c4;Parent=MALK_00633.t1
contig002	AUGUSTUS	CDS	143667	143742	0.45	-	0	ID=MALK_00633.t1.c3;Parent=MALK_00633.t1
contig002	AUGUSTUS	CDS	143142	143639	0.45	-	0	ID=MALK_00633.t1.c2;Parent=MALK_00633.t1
contig002	AUGUSTUS	CDS	142957	143109	0.45	-	0	ID=MALK_00633.t1.c1;Parent=MALK_00633.t1
contig002	AUGUSTUS	mRNA	142957	143869	0.31	-	.	ID=MALK_00633.t1;Parent=MALK_00633
contig002	AUGUSTUS	exon	143781	143869	.	-	.	ID=MALK_00633.t1.e4;Parent=MALK_00633.t1
contig002	AUGUSTUS	exon	143667	143742	.	-	.	ID=MALK_00633.t1.e3;Parent=MALK_00633.t1
contig002	AUGUSTUS	exon	143142	143639	.	-	.	ID=MALK_00633.t1.e2;Parent=MALK_00633.t1
contig002	AUGUSTUS	exon	142957	143109	.	-	.	ID=MALK_00633.t1.e1;Parent=MALK_00633.t1

contig002	maker	gene	144009	148031	.	+	.	ID=MALK_00634;prediction_source=maker_MRET:augustus_masked-contig002-processed-gene-1.58-mRNA-1
contig002	maker	CDS	144009	148031	.	+	0	ID=MALK_00634.t1.c1;Parent=MALK_00634.t1
contig002	maker	mRNA	144009	148031	.	+	.	ID=MALK_00634.t1;Parent=MALK_00634
contig002	maker	exon	144009	148031	.	+	.	ID=MALK_00634.t1.e1;Parent=MALK_00634.t1
contig002	maker	gene	148034	149761	.	-	.	ID=MALK_00635;prediction_source=maker_MRET:augustus_masked-contig002-processed-gene-1.75-mRNA-1
contig002	maker	CDS	148034	149761	.	-	0	ID=MALK_00635.t1.c1;Parent=MALK_00635.t1
contig002	maker	mRNA	148034	149761	.	-	.	ID=MALK_00635.t1;Parent=MALK_00635
contig002	maker	exon	148034	149761	.	-	.	ID=MALK_00635.t1.e1;Parent=MALK_00635.t1
contig002	maker	gene	149954	151603	.	+	.	ID=MALK_00636;prediction_source=maker_MRET:augustus_masked-contig002-processed-gene-1.59-mRNA-1
contig002	maker	CDS	149954	151603	.	+	0	ID=MALK_00636.t1.c1;Parent=MALK_00636.t1
contig002	maker	mRNA	149954	151603	.	+	.	ID=MALK_00636.t1;Parent=MALK_00636
contig002	maker	exon	149954	151603	.	+	.	ID=MALK_00636.t1.e1;Parent=MALK_00636.t1
contig002	maker	gene	151615	155634	.	-	.	ID=MALK_00637;prediction_source=maker_MRET:augustus_masked-contig002-processed-gene-1.76-mRNA-1
contig002	maker	CDS	151615	155634	.	-	0	ID=MALK_00637.t1.c1;Parent=MALK_00637.t1
contig002	maker	mRNA	151615	155634	.	-	.	ID=MALK_00637.t1;Parent=MALK_00637
contig002	maker	exon	151615	155634	.	-	.	ID=MALK_00637.t1.e1;Parent=MALK_00637.t1
contig002	AUGUSTUS	gene	156314	167617	0.64	+	.	ID=MALK_00638;prediction_source=augustus:contig002.g4054.t1
contig002	AUGUSTUS	CDS	156314	167617	0.64	+	0	ID=MALK_00638.t1.c1;Parent=MALK_00638.t1
contig002	AUGUSTUS	mRNA	156314	167617	0.64	+	.	ID=MALK_00638.t1;Parent=MALK_00638
contig002	AUGUSTUS	exon	156314	167617	0.64	+	.	ID=MALK_00638.t1.e1;Parent=MALK_00638.t1
contig002	maker	gene	167618	170404	.	-	.	ID=MALK_00639;prediction_source=maker_MRET:augustus_masked-contig002-processed-gene-1.77-mRNA-1
contig002	maker	CDS	167618	170404	.	-	0	ID=MALK_00639.t1.c1;Parent=MALK_00639.t1
contig002	maker	mRNA	167618	170404	.	-	.	ID=MALK_00639.t1;Parent=MALK_00639
contig002	maker	exon	167618	170404	.	-	.	ID=MALK_00639.t1.e1;Parent=MALK_00639.t1
contig002	maker	gene	170491	172323	.	+	.	ID=MALK_00640;prediction_source=maker_MRET:augustus_masked-contig002-processed-gene-1.61-mRNA-1
contig002	maker	CDS	170491	172323	.	+	0	ID=MALK_00640.t1.c1;Parent=MALK_00640.t1
contig002	maker	mRNA	170491	172323	.	+	.	ID=MALK_00640.t1;Parent=MALK_00640
contig002	maker	exon	170491	172323	.	+	.	ID=MALK_00640.t1.e1;Parent=MALK_00640.t1
contig002	AUGUSTUS	gene	172331	173266	0.43	-	.	ID=MALK_00641;prediction_source=augustus:contig002.g4058.t1
contig002	AUGUSTUS	CDS	172331	173266	0.43	-	0	ID=MALK_00641.t1.c1;Parent=MALK_00641.t1
contig002	AUGUSTUS	mRNA	172331	173266	0.43	-	.	ID=MALK_00641.t1;Parent=MALK_00641
contig002	AUGUSTUS	exon	172331	173266	0.43	-	.	ID=MALK_00641.t1.e1;Parent=MALK_00641.t1
contig002	AUGUSTUS	gene	173431	177349	0.57	+	.	ID=MALK_00642;prediction_source=braker_MRET:g200.t1
contig002	AUGUSTUS	CDS	173431	174229	0.85	+	0	ID=MALK_00642.t1.c1;Parent=MALK_00642.t1
contig002	AUGUSTUS	CDS	174414	177349	0.85	+	0	ID=MALK_00642.t1.c2;Parent=MALK_00642.t1
contig002	AUGUSTUS	mRNA	173431	177349	0.57	+	.	ID=MALK_00642.t1;Parent=MALK_00642
contig002	AUGUSTUS	exon	173431	174229	.	+	.	ID=MALK_00642.t1.e1;Parent=MALK_00642.t1
contig002	AUGUSTUS	exon	174414	177349	.	+	.	ID=MALK_00642.t1.e2;Parent=MALK_00642.t1
contig002	AUGUSTUS	gene	177496	178704	1	+	.	ID=MALK_00643;prediction_source=braker_MRET:g201.t1
contig002	AUGUSTUS	CDS	177496	178704	1	+	0	ID=MALK_00643.t1.c1;Parent=MALK_00643.t1
contig002	AUGUSTUS	mRNA	177496	178704	1	+	.	ID=MALK_00643.t1;Parent=MALK_00643
contig002	AUGUSTUS	exon	177496	178704	.	+	.	ID=MALK_00643.t1.e1;Parent=MALK_00643.t1
contig002	AUGUSTUS	gene	178711	179032	1	-	.	ID=MALK_00644;prediction_source=braker_MRET:g202.t1

contig002	AUGUSTUS	CDS	179030	179032	1	-	0	ID=MALK_00644.t1.c3;Parent=MALK_00644.t1
contig002	AUGUSTUS	CDS	178790	178997	1	-	0	ID=MALK_00644.t1.c2;Parent=MALK_00644.t1
contig002	AUGUSTUS	CDS	178711	178760	1	-	0	ID=MALK_00644.t1.c1;Parent=MALK_00644.t1
contig002	AUGUSTUS	mRNA	178711	179032	1	-	.	ID=MALK_00644.t1;Parent=MALK_00644
contig002	AUGUSTUS	exon	179030	179032	.	-	.	ID=MALK_00644.t1.e3;Parent=MALK_00644.t1
contig002	AUGUSTUS	exon	178790	178997	.	-	.	ID=MALK_00644.t1.e2;Parent=MALK_00644.t1
contig002	AUGUSTUS	exon	178711	178760	.	-	.	ID=MALK_00644.t1.e1;Parent=MALK_00644.t1
contig002	AUGUSTUS	gene	179041	179619	0.69	+	.	ID=MALK_00645;prediction_source=augustus:contig002.g4060.t1
contig002	AUGUSTUS	CDS	179041	179619	0.69	+	0	ID=MALK_00645.t1.c1;Parent=MALK_00645.t1
contig002	AUGUSTUS	mRNA	179041	179619	0.69	+	.	ID=MALK_00645.t1;Parent=MALK_00645
contig002	AUGUSTUS	exon	179041	179619	0.69	+	.	ID=MALK_00645.t1.e1;Parent=MALK_00645.t1
contig002	AUGUSTUS	gene	179807	181276	0.69	+	.	ID=MALK_00646;prediction_source=braker_MRET:g204.t1
contig002	AUGUSTUS	CDS	179807	181276	0.69	+	0	ID=MALK_00646.t1.c1;Parent=MALK_00646.t1
contig002	AUGUSTUS	mRNA	179807	181276	0.69	+	.	ID=MALK_00646.t1;Parent=MALK_00646
contig002	AUGUSTUS	exon	179807	181276	.	+	.	ID=MALK_00646.t1.e1;Parent=MALK_00646.t1
contig002	AUGUSTUS	gene	181398	182510	0.89	+	.	ID=MALK_00647;prediction_source=braker_MRET:g205.t1
contig002	AUGUSTUS	CDS	181398	182510	0.89	+	0	ID=MALK_00647.t1.c1;Parent=MALK_00647.t1
contig002	AUGUSTUS	mRNA	181398	182510	0.89	+	.	ID=MALK_00647.t1;Parent=MALK_00647
contig002	AUGUSTUS	exon	181398	182510	.	+	.	ID=MALK_00647.t1.e1;Parent=MALK_00647.t1
contig002	AUGUSTUS	gene	182657	185101	0.62	+	.	ID=MALK_00648;prediction_source=augustus:contig002.g4062.t1
contig002	AUGUSTUS	CDS	182657	185101	0.62	+	0	ID=MALK_00648.t1.c1;Parent=MALK_00648.t1
contig002	AUGUSTUS	mRNA	182657	185101	0.62	+	.	ID=MALK_00648.t1;Parent=MALK_00648
contig002	AUGUSTUS	exon	182657	185101	0.62	+	.	ID=MALK_00648.t1.e1;Parent=MALK_00648.t1
contig002	maker	gene	185102	186304	.	-	.	ID=MALK_00649;prediction_source=maker_MRET:augustus_masked-contig002-processed-gene-1.79-mRNA-1
contig002	maker	CDS	185102	186304	.	-	0	ID=MALK_00649.t1.c1;Parent=MALK_00649.t1
contig002	maker	mRNA	185102	186304	.	-	.	ID=MALK_00649.t1;Parent=MALK_00649
contig002	maker	exon	185102	186304	.	-	.	ID=MALK_00649.t1.e1;Parent=MALK_00649.t1
contig002	AUGUSTUS	gene	186422	188281	1	+	.	ID=MALK_00650;prediction_source=braker_MRET:g208.t1
contig002	AUGUSTUS	CDS	186422	188281	1	+	0	ID=MALK_00650.t1.c1;Parent=MALK_00650.t1
contig002	AUGUSTUS	mRNA	186422	188281	1	+	.	ID=MALK_00650.t1;Parent=MALK_00650
contig002	AUGUSTUS	exon	186422	188281	.	+	.	ID=MALK_00650.t1.e1;Parent=MALK_00650.t1
contig002	AUGUSTUS	gene	188397	188945	0.98	-	.	ID=MALK_00651;prediction_source=braker_MRET:g209.t1
contig002	AUGUSTUS	CDS	188397	188945	0.98	-	0	ID=MALK_00651.t1.c1;Parent=MALK_00651.t1
contig002	AUGUSTUS	mRNA	188397	188945	0.98	-	.	ID=MALK_00651.t1;Parent=MALK_00651
contig002	AUGUSTUS	exon	188397	188945	.	-	.	ID=MALK_00651.t1.e1;Parent=MALK_00651.t1
contig002	maker	gene	189027	190931	.	+	.	ID=MALK_00652;prediction_source=maker_MRET:augustus_masked-contig002-processed-gene-1.66-mRNA-1
contig002	maker	CDS	189027	190931	.	+	0	ID=MALK_00652.t1.c1;Parent=MALK_00652.t1
contig002	maker	mRNA	189027	190931	.	+	.	ID=MALK_00652.t1;Parent=MALK_00652
contig002	maker	exon	189027	190931	.	+	.	ID=MALK_00652.t1.e1;Parent=MALK_00652.t1
contig002	maker	gene	190948	191664	.	-	.	ID=MALK_00653;prediction_source=maker_MRET:augustus_masked-contig002-processed-gene-1.80-mRNA-1
contig002	maker	CDS	190948	191664	.	-	0	ID=MALK_00653.t1.c1;Parent=MALK_00653.t1
contig002	maker	mRNA	190948	191664	.	-	.	ID=MALK_00653.t1;Parent=MALK_00653
contig002	maker	exon	190948	191664	.	-	.	ID=MALK_00653.t1.e1;Parent=MALK_00653.t1

contig002	AUGUSTUS	gene	191786	192382	0.98	+	.	ID=MALK_00654;prediction_source=augustus:contig002.g4068.t1
contig002	AUGUSTUS	CDS	191786	192382	0.98	+	0	ID=MALK_00654.t1.c1;Parent=MALK_00654.t1
contig002	AUGUSTUS	mRNA	191786	192382	0.98	+	.	ID=MALK_00654.t1;Parent=MALK_00654
contig002	AUGUSTUS	exon	191786	192382	0.98	+	.	ID=MALK_00654.t1.e1;Parent=MALK_00654.t1
contig002	maker	gene	192437	193032	.	-	.	ID=MALK_00655;prediction_source=maker_MRET:augustus_masked-contig002-processed-gene-2.56-mRNA-1
contig002	maker	CDS	192984	193032	.	-	0	ID=MALK_00655.t1.c1;Parent=MALK_00655.t1
contig002	maker	CDS	192437	192909	.	-	0	ID=MALK_00655.t1.c2;Parent=MALK_00655.t1
contig002	maker	mRNA	192437	193032	.	-	.	ID=MALK_00655.t1;Parent=MALK_00655
contig002	maker	exon	192984	193032	.	-	.	ID=MALK_00655.t1.e1;Parent=MALK_00655.t1
contig002	maker	exon	192437	192909	.	-	.	ID=MALK_00655.t1.e2;Parent=MALK_00655.t1
contig002	AUGUSTUS	gene	193160	194941	0.98	-	.	ID=MALK_00656;prediction_source=augustus:contig002.g4069.t1
contig002	AUGUSTUS	CDS	193160	194941	0.98	-	0	ID=MALK_00656.t1.c1;Parent=MALK_00656.t1
contig002	AUGUSTUS	mRNA	193160	194941	0.98	-	.	ID=MALK_00656.t1;Parent=MALK_00656
contig002	AUGUSTUS	exon	193160	194941	0.98	-	.	ID=MALK_00656.t1.e1;Parent=MALK_00656.t1
contig002	maker	gene	195115	197292	.	+	.	ID=MALK_00657;prediction_source=maker_MRET:augustus_masked-contig002-processed-gene-2.53-mRNA-1
contig002	maker	CDS	195115	197292	.	+	0	ID=MALK_00657.t1.c1;Parent=MALK_00657.t1
contig002	maker	mRNA	195115	197292	.	+	.	ID=MALK_00657.t1;Parent=MALK_00657
contig002	maker	exon	195115	197292	.	+	.	ID=MALK_00657.t1.e1;Parent=MALK_00657.t1
contig002	maker	gene	197377	197934	.	+	.	ID=MALK_00658;prediction_source=maker_MRET:augustus_masked-contig002-processed-gene-2.54-mRNA-1
contig002	maker	CDS	197377	197934	.	+	0	ID=MALK_00658.t1.c1;Parent=MALK_00658.t1
contig002	maker	mRNA	197377	197934	.	+	.	ID=MALK_00658.t1;Parent=MALK_00658
contig002	maker	exon	197377	197934	.	+	.	ID=MALK_00658.t1.e1;Parent=MALK_00658.t1
contig002	AUGUSTUS	gene	198168	200201	0.94	+	.	ID=MALK_00659;prediction_source=braker_MRET:g217.t1
contig002	AUGUSTUS	CDS	198168	200201	0.94	+	0	ID=MALK_00659.t1.c1;Parent=MALK_00659.t1
contig002	AUGUSTUS	mRNA	198168	200201	0.94	+	.	ID=MALK_00659.t1;Parent=MALK_00659
contig002	AUGUSTUS	exon	198168	200201	.	+	.	ID=MALK_00659.t1.e1;Parent=MALK_00659.t1
contig002	AUGUSTUS	gene	200268	201585	0.56	+	.	ID=MALK_00660;prediction_source=braker_MRET:g218.t1
contig002	AUGUSTUS	CDS	200268	201163	0.71	+	0	ID=MALK_00660.t1.c1;Parent=MALK_00660.t1
contig002	AUGUSTUS	CDS	201222	201397	0.71	+	0	ID=MALK_00660.t1.c2;Parent=MALK_00660.t1
contig002	AUGUSTUS	CDS	201425	201585	0.71	+	0	ID=MALK_00660.t1.c3;Parent=MALK_00660.t1
contig002	AUGUSTUS	mRNA	200268	201585	0.56	+	.	ID=MALK_00660.t1;Parent=MALK_00660
contig002	AUGUSTUS	exon	200268	201163	.	+	.	ID=MALK_00660.t1.e1;Parent=MALK_00660.t1
contig002	AUGUSTUS	exon	201222	201397	.	+	.	ID=MALK_00660.t1.e2;Parent=MALK_00660.t1
contig002	AUGUSTUS	exon	201425	201585	.	+	.	ID=MALK_00660.t1.e3;Parent=MALK_00660.t1
contig002	AUGUSTUS	gene	201926	204529	0.38	-	.	ID=MALK_00661;prediction_source=augustus:contig002.g4073.t1
contig002	AUGUSTUS	CDS	201926	204529	0.38	-	0	ID=MALK_00661.t1.c1;Parent=MALK_00661.t1
contig002	AUGUSTUS	mRNA	201926	204529	0.38	-	.	ID=MALK_00661.t1;Parent=MALK_00661
contig002	AUGUSTUS	exon	201926	204529	0.38	-	.	ID=MALK_00661.t1.e1;Parent=MALK_00661.t1
contig002	maker	gene	204545	205882	.	+	.	ID=MALK_00662;prediction_source=maker_MRET:augustus_masked-contig002-processed-gene-2.58-mRNA-1
contig002	maker	CDS	204545	205882	.	+	0	ID=MALK_00662.t1.c1;Parent=MALK_00662.t1
contig002	maker	mRNA	204545	205882	.	+	.	ID=MALK_00662.t1;Parent=MALK_00662
contig002	maker	exon	204545	205882	.	+	.	ID=MALK_00662.t1.e1;Parent=MALK_00662.t1
contig002	maker	gene	206170	208686	.	-	.	ID=MALK_00663;prediction_source=maker_MRET:augustus_masked-contig002-processed-gene-2.78-mRNA-1

contig002	maker	CDS	206170	208686	.	-	0	ID=MALK_00663.t1.c1;Parent=MALK_00663.t1
contig002	maker	mRNA	206170	208686	.	-	.	ID=MALK_00663.t1;Parent=MALK_00663
contig002	maker	exon	206170	208686	.	-	.	ID=MALK_00663.t1.e1;Parent=MALK_00663.t1
contig002	AUGUSTUS	gene	209524	211107	0.83	+	.	ID=MALK_00664;prediction_source=augustus:contig002.g4076.t1
contig002	AUGUSTUS	CDS	209524	211107	0.83	+	0	ID=MALK_00664.t1.c1;Parent=MALK_00664.t1
contig002	AUGUSTUS	mRNA	209524	211107	0.83	+	.	ID=MALK_00664.t1;Parent=MALK_00664
contig002	AUGUSTUS	exon	209524	211107	0.83	+	.	ID=MALK_00664.t1.e1;Parent=MALK_00664.t1
contig002	AUGUSTUS	gene	211217	211660	0.79	-	.	ID=MALK_00665;prediction_source=augustus:contig002.g4077.t1
contig002	AUGUSTUS	CDS	211217	211660	0.79	-	0	ID=MALK_00665.t1.c1;Parent=MALK_00665.t1
contig002	AUGUSTUS	mRNA	211217	211660	0.79	-	.	ID=MALK_00665.t1;Parent=MALK_00665
contig002	AUGUSTUS	exon	211217	211660	0.79	-	.	ID=MALK_00665.t1.e1;Parent=MALK_00665.t1
contig002	maker	gene	212084	213751	.	+	.	ID=MALK_00666;prediction_source=maker_MRET:augustus_masked-contig002-processed-gene-2.60-mRNA-1
contig002	maker	CDS	212084	213751	.	+	0	ID=MALK_00666.t1.c1;Parent=MALK_00666.t1
contig002	maker	mRNA	212084	213751	.	+	.	ID=MALK_00666.t1;Parent=MALK_00666
contig002	maker	exon	212084	213751	.	+	.	ID=MALK_00666.t1.e1;Parent=MALK_00666.t1
contig002	AUGUSTUS	gene	214337	216196	0.99	-	.	ID=MALK_00667;prediction_source=augustus:contig002.g4079.t1
contig002	AUGUSTUS	CDS	214337	216196	0.99	-	0	ID=MALK_00667.t1.c1;Parent=MALK_00667.t1
contig002	AUGUSTUS	mRNA	214337	216196	0.99	-	.	ID=MALK_00667.t1;Parent=MALK_00667
contig002	AUGUSTUS	exon	214337	216196	0.99	-	.	ID=MALK_00667.t1.e1;Parent=MALK_00667.t1
contig002	AUGUSTUS	gene	216333	221753	0.87	+	.	ID=MALK_00668;prediction_source=augustus:contig002.g4081.t1
contig002	AUGUSTUS	CDS	216333	221753	0.87	+	0	ID=MALK_00668.t1.c1;Parent=MALK_00668.t1
contig002	AUGUSTUS	mRNA	216333	221753	0.87	+	.	ID=MALK_00668.t1;Parent=MALK_00668
contig002	AUGUSTUS	exon	216333	221753	0.87	+	.	ID=MALK_00668.t1.e1;Parent=MALK_00668.t1
contig002	maker	gene	221970	222959	.	+	.	ID=MALK_00669;prediction_source=maker_MRET:augustus_masked-contig002-processed-gene-2.62-mRNA-1
contig002	maker	CDS	221970	222959	.	+	0	ID=MALK_00669.t1.c1;Parent=MALK_00669.t1
contig002	maker	mRNA	221970	222959	.	+	.	ID=MALK_00669.t1;Parent=MALK_00669
contig002	maker	exon	221970	222959	.	+	.	ID=MALK_00669.t1.e1;Parent=MALK_00669.t1
contig002	AUGUSTUS	gene	223161	224885	1	+	.	ID=MALK_00670;prediction_source=augustus:contig002.g4086.t1
contig002	AUGUSTUS	CDS	223161	224885	1	+	0	ID=MALK_00670.t1.c1;Parent=MALK_00670.t1
contig002	AUGUSTUS	mRNA	223161	224885	1	+	.	ID=MALK_00670.t1;Parent=MALK_00670
contig002	AUGUSTUS	exon	223161	224885	1	+	.	ID=MALK_00670.t1.e1;Parent=MALK_00670.t1
contig002	AUGUSTUS	gene	225074	225755	0.67	-	.	ID=MALK_00671;prediction_source=braker_MRET:g229.t1
contig002	AUGUSTUS	CDS	225486	225755	1	-	0	ID=MALK_00671.t1.c3;Parent=MALK_00671.t1
contig002	AUGUSTUS	CDS	225374	225458	1	-	0	ID=MALK_00671.t1.c2;Parent=MALK_00671.t1
contig002	AUGUSTUS	CDS	225074	225342	1	-	0	ID=MALK_00671.t1.c1;Parent=MALK_00671.t1
contig002	AUGUSTUS	mRNA	225074	225755	0.67	-	.	ID=MALK_00671.t1;Parent=MALK_00671
contig002	AUGUSTUS	exon	225486	225755	.	-	.	ID=MALK_00671.t1.e3;Parent=MALK_00671.t1
contig002	AUGUSTUS	exon	225374	225458	.	-	.	ID=MALK_00671.t1.e2;Parent=MALK_00671.t1
contig002	AUGUSTUS	exon	225074	225342	.	-	.	ID=MALK_00671.t1.e1;Parent=MALK_00671.t1
contig002	AUGUSTUS	gene	225838	227718	0.97	+	.	ID=MALK_00672;prediction_source=braker_MRET:g230.t1
contig002	AUGUSTUS	CDS	225838	227718	0.97	+	0	ID=MALK_00672.t1.c1;Parent=MALK_00672.t1
contig002	AUGUSTUS	mRNA	225838	227718	0.97	+	.	ID=MALK_00672.t1;Parent=MALK_00672
contig002	AUGUSTUS	exon	225838	227718	.	+	.	ID=MALK_00672.t1.e1;Parent=MALK_00672.t1

contig002	maker	gene	227715	228716	.	-	.	ID=MALK_00673;prediction_source=maker_MRET:augustus_masked-contig002-processed-gene-2.82-mRNA-1
contig002	maker	CDS	227715	228716	.	-	0	ID=MALK_00673.t1.c1;Parent=MALK_00673.t1
contig002	maker	mRNA	227715	228716	.	-	.	ID=MALK_00673.t1;Parent=MALK_00673
contig002	maker	exon	227715	228716	.	-	.	ID=MALK_00673.t1.e1;Parent=MALK_00673.t1
contig002	AUGUSTUS	gene	229209	230177	1	-	.	ID=MALK_00674;prediction_source=augustus:contig002.g4090.t1
contig002	AUGUSTUS	CDS	229209	230177	1	-	0	ID=MALK_00674.t1.c1;Parent=MALK_00674.t1
contig002	AUGUSTUS	mRNA	229209	230177	1	-	.	ID=MALK_00674.t1;Parent=MALK_00674
contig002	AUGUSTUS	exon	229209	230177	1	-	.	ID=MALK_00674.t1.e1;Parent=MALK_00674.t1
contig002	AUGUSTUS	gene	230249	232009	0.97	+	.	ID=MALK_00675;prediction_source=braker_MRET:g233.t1
contig002	AUGUSTUS	CDS	230249	232009	0.97	+	0	ID=MALK_00675.t1.c1;Parent=MALK_00675.t1
contig002	AUGUSTUS	mRNA	230249	232009	0.97	+	.	ID=MALK_00675.t1;Parent=MALK_00675
contig002	AUGUSTUS	exon	230249	232009	.	+	.	ID=MALK_00675.t1.e1;Parent=MALK_00675.t1
contig002	AUGUSTUS	gene	232078	234285	0.48	+	.	ID=MALK_00676;prediction_source=augustus:contig002.g4092.t1
contig002	AUGUSTUS	CDS	232078	234285	0.48	+	0	ID=MALK_00676.t1.c1;Parent=MALK_00676.t1
contig002	AUGUSTUS	mRNA	232078	234285	0.48	+	.	ID=MALK_00676.t1;Parent=MALK_00676
contig002	AUGUSTUS	exon	232078	234285	0.48	+	.	ID=MALK_00676.t1.e1;Parent=MALK_00676.t1
contig002	AUGUSTUS	gene	234336	239414	0.61	+	.	ID=MALK_00677;prediction_source=augustus:contig002.g4093.t1
contig002	AUGUSTUS	CDS	234336	239414	0.61	+	0	ID=MALK_00677.t1.c1;Parent=MALK_00677.t1
contig002	AUGUSTUS	mRNA	234336	239414	0.61	+	.	ID=MALK_00677.t1;Parent=MALK_00677
contig002	AUGUSTUS	exon	234336	239414	0.61	+	.	ID=MALK_00677.t1.e1;Parent=MALK_00677.t1
contig002	maker	gene	239453	241189	.	+	.	ID=MALK_00678;prediction_source=maker_MRET:augustus_masked-contig002-processed-gene-2.66-mRNA-1
contig002	maker	CDS	239453	241189	.	+	0	ID=MALK_00678.t1.c1;Parent=MALK_00678.t1
contig002	maker	mRNA	239453	241189	.	+	.	ID=MALK_00678.t1;Parent=MALK_00678
contig002	maker	exon	239453	241189	.	+	.	ID=MALK_00678.t1.e1;Parent=MALK_00678.t1
contig002	AUGUSTUS	gene	241301	244327	0.7	+	.	ID=MALK_00679;prediction_source=augustus:contig002.g4095.t1
contig002	AUGUSTUS	CDS	241301	244327	0.7	+	0	ID=MALK_00679.t1.c1;Parent=MALK_00679.t1
contig002	AUGUSTUS	mRNA	241301	244327	0.7	+	.	ID=MALK_00679.t1;Parent=MALK_00679
contig002	AUGUSTUS	exon	241301	244327	0.7	+	.	ID=MALK_00679.t1.e1;Parent=MALK_00679.t1
contig002	AUGUSTUS	gene	244410	245882	0.96	+	.	ID=MALK_00680;prediction_source=braker_MRET:g237.t1
contig002	AUGUSTUS	CDS	244410	245882	0.96	+	0	ID=MALK_00680.t1.c1;Parent=MALK_00680.t1
contig002	AUGUSTUS	mRNA	244410	245882	0.96	+	.	ID=MALK_00680.t1;Parent=MALK_00680
contig002	AUGUSTUS	exon	244410	245882	.	+	.	ID=MALK_00680.t1.e1;Parent=MALK_00680.t1
contig002	AUGUSTUS	gene	247312	248424	0.91	-	.	ID=MALK_00681;prediction_source=augustus:contig002.g4097.t1
contig002	AUGUSTUS	CDS	247312	248424	0.91	-	0	ID=MALK_00681.t1.c1;Parent=MALK_00681.t1
contig002	AUGUSTUS	mRNA	247312	248424	0.91	-	.	ID=MALK_00681.t1;Parent=MALK_00681
contig002	AUGUSTUS	exon	247312	248424	0.91	-	.	ID=MALK_00681.t1.e1;Parent=MALK_00681.t1
contig002	AUGUSTUS	gene	248972	249952	0.41	+	.	ID=MALK_00682;prediction_source=braker_MRET:g239.t1
contig002	AUGUSTUS	CDS	248972	249952	0.41	+	0	ID=MALK_00682.t1.c1;Parent=MALK_00682.t1
contig002	AUGUSTUS	mRNA	248972	249952	0.41	+	.	ID=MALK_00682.t1;Parent=MALK_00682
contig002	AUGUSTUS	exon	248972	249952	.	+	.	ID=MALK_00682.t1.e1;Parent=MALK_00682.t1
contig002	AUGUSTUS	gene	249994	250509	0.99	-	.	ID=MALK_00683;prediction_source=augustus:contig002.g4099.t1
contig002	AUGUSTUS	CDS	249994	250509	0.99	-	0	ID=MALK_00683.t1.c1;Parent=MALK_00683.t1
contig002	AUGUSTUS	mRNA	249994	250509	0.99	-	.	ID=MALK_00683.t1;Parent=MALK_00683

contig002	AUGUSTUS	exon	249994	250509	0.99	-	.	ID=MALK_00683.t1.e1;Parent=MALK_00683.t1
contig002	AUGUSTUS	gene	250762	252048	0.58	-	.	ID=MALK_00684;prediction_source=augustus:contig002.g4100.t1
contig002	AUGUSTUS	CDS	250762	252048	0.58	-	0	ID=MALK_00684.t1.c1;Parent=MALK_00684.t1
contig002	AUGUSTUS	mRNA	250762	252048	0.58	-	.	ID=MALK_00684.t1;Parent=MALK_00684
contig002	AUGUSTUS	exon	250762	252048	0.58	-	.	ID=MALK_00684.t1.e1;Parent=MALK_00684.t1
contig002	maker	gene	252177	252524	.	+	.	ID=MALK_00685;prediction_source=maker_MRET:augustus_masked-contig002-processed-gene-2.70-mRNA-1
contig002	maker	CDS	252177	252524	.	+	0	ID=MALK_00685.t1.c1;Parent=MALK_00685.t1
contig002	maker	mRNA	252177	252524	.	+	.	ID=MALK_00685.t1;Parent=MALK_00685
contig002	maker	exon	252177	252524	.	+	.	ID=MALK_00685.t1.e1;Parent=MALK_00685.t1
contig002	AUGUSTUS	gene	252838	255237	0.68	-	.	ID=MALK_00686;prediction_source=augustus:contig002.g4102.t1
contig002	AUGUSTUS	CDS	252838	255237	0.68	-	0	ID=MALK_00686.t1.c1;Parent=MALK_00686.t1
contig002	AUGUSTUS	mRNA	252838	255237	0.68	-	.	ID=MALK_00686.t1;Parent=MALK_00686
contig002	AUGUSTUS	exon	252838	255237	0.68	-	.	ID=MALK_00686.t1.e1;Parent=MALK_00686.t1
contig002	AUGUSTUS	gene	255502	256365	0.99	-	.	ID=MALK_00687;prediction_source=augustus:contig002.g4104.t1
contig002	AUGUSTUS	CDS	255502	256365	0.99	-	0	ID=MALK_00687.t1.c1;Parent=MALK_00687.t1
contig002	AUGUSTUS	mRNA	255502	256365	0.99	-	.	ID=MALK_00687.t1;Parent=MALK_00687
contig002	AUGUSTUS	exon	255502	256365	0.99	-	.	ID=MALK_00687.t1.e1;Parent=MALK_00687.t1
contig002	AUGUSTUS	gene	256677	258014	0.75	-	.	ID=MALK_00688;prediction_source=augustus:contig002.g4105.t1
contig002	AUGUSTUS	CDS	256677	258014	0.75	-	0	ID=MALK_00688.t1.c1;Parent=MALK_00688.t1
contig002	AUGUSTUS	mRNA	256677	258014	0.75	-	.	ID=MALK_00688.t1;Parent=MALK_00688
contig002	AUGUSTUS	exon	256677	258014	0.75	-	.	ID=MALK_00688.t1.e1;Parent=MALK_00688.t1
contig002	maker	gene	258593	260674	.	-	.	ID=MALK_00689;prediction_source=maker_MRET:augustus_masked-contig002-processed-gene-2.90-mRNA-1
contig002	maker	CDS	258593	260674	.	-	0	ID=MALK_00689.t1.c1;Parent=MALK_00689.t1
contig002	maker	mRNA	258593	260674	.	-	.	ID=MALK_00689.t1;Parent=MALK_00689
contig002	maker	exon	258593	260674	.	-	.	ID=MALK_00689.t1.e1;Parent=MALK_00689.t1
contig002	AUGUSTUS	gene	260826	262860	0.9	+	.	ID=MALK_00690;prediction_source=braker_MRET:g247.t1
contig002	AUGUSTUS	CDS	260826	262753	0.92	+	0	ID=MALK_00690.t1.c1;Parent=MALK_00690.t1
contig002	AUGUSTUS	CDS	262800	262860	0.92	+	0	ID=MALK_00690.t1.c2;Parent=MALK_00690.t1
contig002	AUGUSTUS	mRNA	260826	262860	0.9	+	.	ID=MALK_00690.t1;Parent=MALK_00690
contig002	AUGUSTUS	exon	260826	262753	.	+	.	ID=MALK_00690.t1.e1;Parent=MALK_00690.t1
contig002	AUGUSTUS	exon	262800	262860	.	+	.	ID=MALK_00690.t1.e2;Parent=MALK_00690.t1
contig002	maker	gene	262950	264365	.	-	.	ID=MALK_00691;prediction_source=maker_MRET:augustus_masked-contig002-processed-gene-2.91-mRNA-1
contig002	maker	CDS	262950	264365	.	-	0	ID=MALK_00691.t1.c1;Parent=MALK_00691.t1
contig002	maker	mRNA	262950	264365	.	-	.	ID=MALK_00691.t1;Parent=MALK_00691
contig002	maker	exon	262950	264365	.	-	.	ID=MALK_00691.t1.e1;Parent=MALK_00691.t1
contig002	AUGUSTUS	gene	264685	265260	0.95	-	.	ID=MALK_00692;prediction_source=augustus:contig002.g4111.t1
contig002	AUGUSTUS	CDS	264685	265260	0.95	-	0	ID=MALK_00692.t1.c1;Parent=MALK_00692.t1
contig002	AUGUSTUS	mRNA	264685	265260	0.95	-	.	ID=MALK_00692.t1;Parent=MALK_00692
contig002	AUGUSTUS	exon	264685	265260	0.95	-	.	ID=MALK_00692.t1.e1;Parent=MALK_00692.t1
contig002	maker	gene	265531	266415	.	+	.	ID=MALK_00693;prediction_source=maker_MRET:augustus_masked-contig002-processed-gene-2.72-mRNA-1
contig002	maker	CDS	265531	266415	.	+	0	ID=MALK_00693.t1.c1;Parent=MALK_00693.t1
contig002	maker	mRNA	265531	266415	.	+	.	ID=MALK_00693.t1;Parent=MALK_00693
contig002	maker	exon	265531	266415	.	+	.	ID=MALK_00693.t1.e1;Parent=MALK_00693.t1

contig002	AUGUSTUS	gene	266429	268723	0.98	-	.	ID=MALK_00694;prediction_source=braker_MRET:g250.t1
contig002	AUGUSTUS	CDS	266429	268723	0.98	-	0	ID=MALK_00694.t1.c1;Parent=MALK_00694.t1
contig002	AUGUSTUS	mRNA	266429	268723	0.98	-	.	ID=MALK_00694.t1;Parent=MALK_00694
contig002	AUGUSTUS	exon	266429	268723	.	-	.	ID=MALK_00694.t1.e1;Parent=MALK_00694.t1
contig002	AUGUSTUS	gene	268783	271299	0.99	-	.	ID=MALK_00695;prediction_source=braker_MRET:g251.t1
contig002	AUGUSTUS	CDS	268783	271299	0.99	-	0	ID=MALK_00695.t1.c1;Parent=MALK_00695.t1
contig002	AUGUSTUS	mRNA	268783	271299	0.99	-	.	ID=MALK_00695.t1;Parent=MALK_00695
contig002	AUGUSTUS	exon	268783	271299	.	-	.	ID=MALK_00695.t1.e1;Parent=MALK_00695.t1
contig002	AUGUSTUS	gene	271360	273240	0.82	-	.	ID=MALK_00696;prediction_source=braker_MRET:g252.t1
contig002	AUGUSTUS	CDS	271360	273240	0.82	-	0	ID=MALK_00696.t1.c1;Parent=MALK_00696.t1
contig002	AUGUSTUS	mRNA	271360	273240	0.82	-	.	ID=MALK_00696.t1;Parent=MALK_00696
contig002	AUGUSTUS	exon	271360	273240	.	-	.	ID=MALK_00696.t1.e1;Parent=MALK_00696.t1
contig002	AUGUSTUS	gene	273190	274073	0.52	+	.	ID=MALK_00697;prediction_source=augustus:contig002.g4114.t1
contig002	AUGUSTUS	CDS	273190	273198	0.92	+	0	ID=MALK_00697.t1.c1;Parent=MALK_00697.t1
contig002	AUGUSTUS	CDS	273345	274073	0.92	+	0	ID=MALK_00697.t1.c2;Parent=MALK_00697.t1
contig002	AUGUSTUS	mRNA	273190	274073	0.52	+	.	ID=MALK_00697.t1;Parent=MALK_00697
contig002	AUGUSTUS	exon	273190	273198	0.92	+	.	ID=MALK_00697.t1.e1;Parent=MALK_00697.t1
contig002	AUGUSTUS	exon	273345	274073	0.92	+	.	ID=MALK_00697.t1.e2;Parent=MALK_00697.t1
contig002	AUGUSTUS	gene	274205	275307	0.43	-	.	ID=MALK_00698;prediction_source=braker_MRET:g254.t1
contig002	AUGUSTUS	CDS	274334	275307	0.43	-	0	ID=MALK_00698.t1.c2;Parent=MALK_00698.t1
contig002	AUGUSTUS	CDS	274205	274304	0.43	-	0	ID=MALK_00698.t1.c1;Parent=MALK_00698.t1
contig002	AUGUSTUS	mRNA	274205	275307	0.43	-	.	ID=MALK_00698.t1;Parent=MALK_00698
contig002	AUGUSTUS	exon	274334	275307	.	-	.	ID=MALK_00698.t1.e2;Parent=MALK_00698.t1
contig002	AUGUSTUS	exon	274205	274304	.	-	.	ID=MALK_00698.t1.e1;Parent=MALK_00698.t1
contig002	AUGUSTUS	gene	275485	276105	0.99	-	.	ID=MALK_00699;prediction_source=braker_MRET:g255.t1
contig002	AUGUSTUS	CDS	276074	276105	0.99	-	0	ID=MALK_00699.t1.c4;Parent=MALK_00699.t1
contig002	AUGUSTUS	CDS	275967	276000	0.99	-	0	ID=MALK_00699.t1.c3;Parent=MALK_00699.t1
contig002	AUGUSTUS	CDS	275716	275868	0.99	-	0	ID=MALK_00699.t1.c2;Parent=MALK_00699.t1
contig002	AUGUSTUS	CDS	275485	275676	0.99	-	0	ID=MALK_00699.t1.c1;Parent=MALK_00699.t1
contig002	AUGUSTUS	mRNA	275485	276105	0.99	-	.	ID=MALK_00699.t1;Parent=MALK_00699
contig002	AUGUSTUS	exon	276074	276105	.	-	.	ID=MALK_00699.t1.e4;Parent=MALK_00699.t1
contig002	AUGUSTUS	exon	275967	276000	.	-	.	ID=MALK_00699.t1.e3;Parent=MALK_00699.t1
contig002	AUGUSTUS	exon	275716	275868	.	-	.	ID=MALK_00699.t1.e2;Parent=MALK_00699.t1
contig002	AUGUSTUS	exon	275485	275676	.	-	.	ID=MALK_00699.t1.e1;Parent=MALK_00699.t1
contig002	maker	gene	276302	276733	.	+	.	ID=MALK_00700;prediction_source=maker_MRET:augustus_masked-contig002-processed-gene-2.73-mRNA-1
contig002	maker	CDS	276302	276733	.	+	0	ID=MALK_00700.t1.c1;Parent=MALK_00700.t1
contig002	maker	mRNA	276302	276733	.	+	.	ID=MALK_00700.t1;Parent=MALK_00700
contig002	maker	exon	276302	276733	.	+	.	ID=MALK_00700.t1.e1;Parent=MALK_00700.t1
contig002	AUGUSTUS	gene	276946	277280	0.76	-	.	ID=MALK_00701;prediction_source=braker_MRET:g257.t1
contig002	AUGUSTUS	CDS	277017	277280	0.99	-	0	ID=MALK_00701.t1.c2;Parent=MALK_00701.t1
contig002	AUGUSTUS	CDS	276946	276978	0.99	-	0	ID=MALK_00701.t1.c1;Parent=MALK_00701.t1
contig002	AUGUSTUS	mRNA	276946	277280	0.76	-	.	ID=MALK_00701.t1;Parent=MALK_00701
contig002	AUGUSTUS	exon	277017	277280	.	-	.	ID=MALK_00701.t1.e2;Parent=MALK_00701.t1

contig002	AUGUSTUS	exon	276946	276978	.	-	.	ID=MALK_00701.t1.e1;Parent=MALK_00701.t1
contig002	maker	gene	277561	278469	.	+	.	ID=MALK_00702;prediction_source=maker_MRET:augustus_masked-contig002-processed-gene-2.74-mRNA-1
contig002	maker	CDS	277561	278469	.	+	0	ID=MALK_00702.t1.c1;Parent=MALK_00702.t1
contig002	maker	mRNA	277561	278469	.	+	.	ID=MALK_00702.t1;Parent=MALK_00702
contig002	maker	exon	277561	278469	.	+	.	ID=MALK_00702.t1.e1;Parent=MALK_00702.t1
contig002	AUGUSTUS	gene	278471	279004	1	-	.	ID=MALK_00703;prediction_source=braker_MRET:g259.t1
contig002	AUGUSTUS	CDS	278471	279004	1	-	0	ID=MALK_00703.t1.c1;Parent=MALK_00703.t1
contig002	AUGUSTUS	mRNA	278471	279004	1	-	.	ID=MALK_00703.t1;Parent=MALK_00703
contig002	AUGUSTUS	exon	278471	279004	.	-	.	ID=MALK_00703.t1.e1;Parent=MALK_00703.t1
contig002	AUGUSTUS	gene	279445	281562	1	-	.	ID=MALK_00704;prediction_source=braker_MRET:g260.t1
contig002	AUGUSTUS	CDS	279445	281562	1	-	0	ID=MALK_00704.t1.c1;Parent=MALK_00704.t1
contig002	AUGUSTUS	mRNA	279445	281562	1	-	.	ID=MALK_00704.t1;Parent=MALK_00704
contig002	AUGUSTUS	exon	279445	281562	.	-	.	ID=MALK_00704.t1.e1;Parent=MALK_00704.t1
contig002	AUGUSTUS	gene	281588	282813	0.73	+	.	ID=MALK_00705;prediction_source=braker_MRET:g261.t1
contig002	AUGUSTUS	CDS	281588	282741	0.75	+	0	ID=MALK_00705.t1.c1;Parent=MALK_00705.t1
contig002	AUGUSTUS	CDS	282783	282813	0.75	+	0	ID=MALK_00705.t1.c2;Parent=MALK_00705.t1
contig002	AUGUSTUS	mRNA	281588	282813	0.73	+	.	ID=MALK_00705.t1;Parent=MALK_00705
contig002	AUGUSTUS	exon	281588	282741	.	+	.	ID=MALK_00705.t1.e1;Parent=MALK_00705.t1
contig002	AUGUSTUS	exon	282783	282813	.	+	.	ID=MALK_00705.t1.e2;Parent=MALK_00705.t1
contig002	AUGUSTUS	gene	282882	284612	0.78	-	.	ID=MALK_00706;prediction_source=augustus:contig002.g4118.t1
contig002	AUGUSTUS	CDS	282882	284612	0.78	-	0	ID=MALK_00706.t1.c1;Parent=MALK_00706.t1
contig002	AUGUSTUS	mRNA	282882	284612	0.78	-	.	ID=MALK_00706.t1;Parent=MALK_00706
contig002	AUGUSTUS	exon	282882	284612	0.78	-	.	ID=MALK_00706.t1.e1;Parent=MALK_00706.t1
contig002	AUGUSTUS	gene	284788	286956	1	+	.	ID=MALK_00707;prediction_source=augustus:contig002.g4119.t1
contig002	AUGUSTUS	CDS	284788	286956	1	+	0	ID=MALK_00707.t1.c1;Parent=MALK_00707.t1
contig002	AUGUSTUS	mRNA	284788	286956	1	+	.	ID=MALK_00707.t1;Parent=MALK_00707
contig002	AUGUSTUS	exon	284788	286956	1	+	.	ID=MALK_00707.t1.e1;Parent=MALK_00707.t1
contig002	AUGUSTUS	gene	286925	287854	1	-	.	ID=MALK_00708;prediction_source=augustus:contig002.g4120.t1
contig002	AUGUSTUS	CDS	286925	287854	1	-	0	ID=MALK_00708.t1.c1;Parent=MALK_00708.t1
contig002	AUGUSTUS	mRNA	286925	287854	1	-	.	ID=MALK_00708.t1;Parent=MALK_00708
contig002	AUGUSTUS	exon	286925	287854	1	-	.	ID=MALK_00708.t1.e1;Parent=MALK_00708.t1
contig002	maker	gene	288015	292157	.	+	.	ID=MALK_00709;prediction_source=maker_MRET:augustus_masked-contig002-processed-gene-2.76-mRNA-1
contig002	maker	CDS	288015	292157	.	+	0	ID=MALK_00709.t1.c1;Parent=MALK_00709.t1
contig002	maker	mRNA	288015	292157	.	+	.	ID=MALK_00709.t1;Parent=MALK_00709
contig002	maker	exon	288015	292157	.	+	.	ID=MALK_00709.t1.e1;Parent=MALK_00709.t1
contig002	AUGUSTUS	gene	292192	294061	0.96	-	.	ID=MALK_00710;prediction_source=braker_MRET:g266.t1
contig002	AUGUSTUS	CDS	294007	294061	1	-	0	ID=MALK_00710.t1.c2;Parent=MALK_00710.t1
contig002	AUGUSTUS	CDS	292192	293978	1	-	0	ID=MALK_00710.t1.c1;Parent=MALK_00710.t1
contig002	AUGUSTUS	mRNA	292192	294061	0.96	-	.	ID=MALK_00710.t1;Parent=MALK_00710
contig002	AUGUSTUS	exon	294007	294061	.	-	.	ID=MALK_00710.t1.e2;Parent=MALK_00710.t1
contig002	AUGUSTUS	exon	292192	293978	.	-	.	ID=MALK_00710.t1.e1;Parent=MALK_00710.t1
contig002	AUGUSTUS	gene	294090	295007	0.98	+	.	ID=MALK_00711;prediction_source=augustus:contig002.g4125.t1
contig002	AUGUSTUS	CDS	294090	295007	0.98	+	0	ID=MALK_00711.t1.c1;Parent=MALK_00711.t1

contig002	AUGUSTUS	mRNA	294090	295007	0.98	+	.	ID=MALK_00711.t1;Parent=MALK_00711
contig002	AUGUSTUS	exon	294090	295007	0.98	+	.	ID=MALK_00711.t1.e1;Parent=MALK_00711.t1
contig002	maker	gene	295066	295773	.	-	.	ID=MALK_00712;prediction_source=maker_MRET:augustus_masked-contig002-processed-gene-3.3-mRNA-1
contig002	maker	CDS	295549	295773	.	-	0	ID=MALK_00712.t1.c1;Parent=MALK_00712.t1
contig002	maker	CDS	295066	295464	.	-	0	ID=MALK_00712.t1.c2;Parent=MALK_00712.t1
contig002	maker	mRNA	295066	295773	.	-	.	ID=MALK_00712.t1;Parent=MALK_00712
contig002	maker	exon	295549	295773	.	-	.	ID=MALK_00712.t1.e1;Parent=MALK_00712.t1
contig002	maker	exon	295066	295464	.	-	.	ID=MALK_00712.t1.e2;Parent=MALK_00712.t1
contig002	maker	gene	295862	297121	.	-	.	ID=MALK_00713;prediction_source=maker_MRET:augustus_masked-contig002-processed-gene-3.4-mRNA-1
contig002	maker	CDS	295862	297121	.	-	0	ID=MALK_00713.t1.c1;Parent=MALK_00713.t1
contig002	maker	mRNA	295862	297121	.	-	.	ID=MALK_00713.t1;Parent=MALK_00713
contig002	maker	exon	295862	297121	.	-	.	ID=MALK_00713.t1.e1;Parent=MALK_00713.t1
contig002	AUGUSTUS	gene	297491	297811	0.48	+	.	ID=MALK_00714;prediction_source=braker_MRET:g269.t1
contig002	AUGUSTUS	CDS	297491	297517	0.54	+	0	ID=MALK_00714.t1.c1;Parent=MALK_00714.t1
contig002	AUGUSTUS	CDS	297552	297664	0.54	+	0	ID=MALK_00714.t1.c2;Parent=MALK_00714.t1
contig002	AUGUSTUS	CDS	297700	297811	0.54	+	0	ID=MALK_00714.t1.c3;Parent=MALK_00714.t1
contig002	AUGUSTUS	mRNA	297491	297811	0.48	+	.	ID=MALK_00714.t1;Parent=MALK_00714
contig002	AUGUSTUS	exon	297491	297517	.	+	.	ID=MALK_00714.t1.e1;Parent=MALK_00714.t1
contig002	AUGUSTUS	exon	297552	297664	.	+	.	ID=MALK_00714.t1.e2;Parent=MALK_00714.t1
contig002	AUGUSTUS	exon	297700	297811	.	+	.	ID=MALK_00714.t1.e3;Parent=MALK_00714.t1
contig002	maker	gene	297989	299320	.	+	.	ID=MALK_00715;prediction_source=maker_MRET:augustus_masked-contig002-processed-gene-3.1-mRNA-1
contig002	maker	CDS	297989	299320	.	+	0	ID=MALK_00715.t1.c1;Parent=MALK_00715.t1
contig002	maker	mRNA	297989	299320	.	+	.	ID=MALK_00715.t1;Parent=MALK_00715
contig002	maker	exon	297989	299320	.	+	.	ID=MALK_00715.t1.e1;Parent=MALK_00715.t1
contig002	AUGUSTUS	gene	299344	300726	0.86	+	.	ID=MALK_00716;prediction_source=braker_MRET:g271.t1
contig002	AUGUSTUS	CDS	299344	300726	0.86	+	0	ID=MALK_00716.t1.c1;Parent=MALK_00716.t1
contig002	AUGUSTUS	mRNA	299344	300726	0.86	+	.	ID=MALK_00716.t1;Parent=MALK_00716
contig002	AUGUSTUS	exon	299344	300726	.	+	.	ID=MALK_00716.t1.e1;Parent=MALK_00716.t1
contig002	AUGUSTUS	gene	300719	302452	0.59	-	.	ID=MALK_00717;prediction_source=augustus:contig002.g4130.t1
contig002	AUGUSTUS	CDS	300719	302452	0.59	-	0	ID=MALK_00717.t1.c1;Parent=MALK_00717.t1
contig002	AUGUSTUS	mRNA	300719	302452	0.59	-	.	ID=MALK_00717.t1;Parent=MALK_00717
contig002	AUGUSTUS	exon	300719	302452	0.59	-	.	ID=MALK_00717.t1.e1;Parent=MALK_00717.t1
contig002	AUGUSTUS	gene	302831	304045	0.76	+	.	ID=MALK_00718;prediction_source=braker_MRET:g273.t1
contig002	AUGUSTUS	CDS	302831	304045	0.76	+	0	ID=MALK_00718.t1.c1;Parent=MALK_00718.t1
contig002	AUGUSTUS	mRNA	302831	304045	0.76	+	.	ID=MALK_00718.t1;Parent=MALK_00718
contig002	AUGUSTUS	exon	302831	304045	.	+	.	ID=MALK_00718.t1.e1;Parent=MALK_00718.t1
contig002	AUGUSTUS	gene	304153	305107	0.89	+	.	ID=MALK_00719;prediction_source=braker_MRET:g274.t1
contig002	AUGUSTUS	CDS	304153	304795	1	+	0	ID=MALK_00719.t1.c1;Parent=MALK_00719.t1
contig002	AUGUSTUS	CDS	304824	305107	1	+	0	ID=MALK_00719.t1.c2;Parent=MALK_00719.t1
contig002	AUGUSTUS	mRNA	304153	305107	0.89	+	.	ID=MALK_00719.t1;Parent=MALK_00719
contig002	AUGUSTUS	exon	304153	304795	.	+	.	ID=MALK_00719.t1.e1;Parent=MALK_00719.t1
contig002	AUGUSTUS	exon	304824	305107	.	+	.	ID=MALK_00719.t1.e2;Parent=MALK_00719.t1
contig002	AUGUSTUS	gene	305381	307069	1	+	.	ID=MALK_00720;prediction_source=augustus:contig002.g4133.t1

contig002	AUGUSTUS	CDS	305381	307069	1	+	0	ID=MALK_00720.t1.c1;Parent=MALK_00720.t1
contig002	AUGUSTUS	mRNA	305381	307069	1	+	.	ID=MALK_00720.t1;Parent=MALK_00720
contig002	AUGUSTUS	exon	305381	307069	1	+	.	ID=MALK_00720.t1.e1;Parent=MALK_00720.t1
contig002	AUGUSTUS	gene	307066	308034	0.35	-	.	ID=MALK_00721;prediction_source=augustus:contig002.g4134.t1
contig002	AUGUSTUS	CDS	307066	308034	0.35	-	0	ID=MALK_00721.t1.c1;Parent=MALK_00721.t1
contig002	AUGUSTUS	mRNA	307066	308034	0.35	-	.	ID=MALK_00721.t1;Parent=MALK_00721
contig002	AUGUSTUS	exon	307066	308034	0.35	-	.	ID=MALK_00721.t1.e1;Parent=MALK_00721.t1
contig002	AUGUSTUS	gene	308152	309459	0.98	-	.	ID=MALK_00722;prediction_source=braker_MRET:g277.t1
contig002	AUGUSTUS	CDS	308152	309459	0.98	-	0	ID=MALK_00722.t1.c1;Parent=MALK_00722.t1
contig002	AUGUSTUS	mRNA	308152	309459	0.98	-	.	ID=MALK_00722.t1;Parent=MALK_00722
contig002	AUGUSTUS	exon	308152	309459	.	-	.	ID=MALK_00722.t1.e1;Parent=MALK_00722.t1
contig002	AUGUSTUS	gene	309523	310264	1	-	.	ID=MALK_00723;prediction_source=braker_MRET:g278.t1
contig002	AUGUSTUS	CDS	310229	310264	1	-	0	ID=MALK_00723.t1.c3;Parent=MALK_00723.t1
contig002	AUGUSTUS	CDS	310129	310189	1	-	0	ID=MALK_00723.t1.c2;Parent=MALK_00723.t1
contig002	AUGUSTUS	CDS	309523	310088	1	-	0	ID=MALK_00723.t1.c1;Parent=MALK_00723.t1
contig002	AUGUSTUS	mRNA	309523	310264	1	-	.	ID=MALK_00723.t1;Parent=MALK_00723
contig002	AUGUSTUS	exon	310229	310264	.	-	.	ID=MALK_00723.t1.e3;Parent=MALK_00723.t1
contig002	AUGUSTUS	exon	310129	310189	.	-	.	ID=MALK_00723.t1.e2;Parent=MALK_00723.t1
contig002	AUGUSTUS	exon	309523	310088	.	-	.	ID=MALK_00723.t1.e1;Parent=MALK_00723.t1
contig002	AUGUSTUS	gene	310467	313130	0.99	+	.	ID=MALK_00724;prediction_source=augustus:contig002.g4137.t1
contig002	AUGUSTUS	CDS	310467	313130	0.99	+	0	ID=MALK_00724.t1.c1;Parent=MALK_00724.t1
contig002	AUGUSTUS	mRNA	310467	313130	0.99	+	.	ID=MALK_00724.t1;Parent=MALK_00724
contig002	AUGUSTUS	exon	310467	313130	0.99	+	.	ID=MALK_00724.t1.e1;Parent=MALK_00724.t1
contig002	AUGUSTUS	gene	313145	314839	0.74	-	.	ID=MALK_00725;prediction_source=augustus:contig002.g4139.t1
contig002	AUGUSTUS	CDS	313145	314839	0.74	-	0	ID=MALK_00725.t1.c1;Parent=MALK_00725.t1
contig002	AUGUSTUS	mRNA	313145	314839	0.74	-	.	ID=MALK_00725.t1;Parent=MALK_00725
contig002	AUGUSTUS	exon	313145	314839	0.74	-	.	ID=MALK_00725.t1.e1;Parent=MALK_00725.t1
contig002	maker	gene	314932	315726	.	+	.	ID=MALK_00726;prediction_source=maker_MRET:augustus_masked-contig002-processed-gene-3.8-mRNA-1
contig002	maker	CDS	314932	315726	.	+	0	ID=MALK_00726.t1.c1;Parent=MALK_00726.t1
contig002	maker	mRNA	314932	315726	.	+	.	ID=MALK_00726.t1;Parent=MALK_00726
contig002	maker	exon	314932	315726	.	+	.	ID=MALK_00726.t1.e1;Parent=MALK_00726.t1
contig002	maker	gene	315738	317879	.	-	.	ID=MALK_00727;prediction_source=maker_MRET:augustus_masked-contig002-processed-gene-3.33-mRNA-1
contig002	maker	CDS	315738	317879	.	-	0	ID=MALK_00727.t1.c1;Parent=MALK_00727.t1
contig002	maker	mRNA	315738	317879	.	-	.	ID=MALK_00727.t1;Parent=MALK_00727
contig002	maker	exon	315738	317879	.	-	.	ID=MALK_00727.t1.e1;Parent=MALK_00727.t1
contig002	maker	gene	318147	318827	.	+	.	ID=MALK_00728;prediction_source=maker_MRET:augustus_masked-contig002-processed-gene-3.9-mRNA-1
contig002	maker	CDS	318147	318827	.	+	0	ID=MALK_00728.t1.c1;Parent=MALK_00728.t1
contig002	maker	mRNA	318147	318827	.	+	.	ID=MALK_00728.t1;Parent=MALK_00728
contig002	maker	exon	318147	318827	.	+	.	ID=MALK_00728.t1.e1;Parent=MALK_00728.t1
contig002	AUGUSTUS	gene	318866	319654	1	-	.	ID=MALK_00729;prediction_source=braker_MRET:g284.t1
contig002	AUGUSTUS	CDS	318866	319654	1	-	0	ID=MALK_00729.t1.c1;Parent=MALK_00729.t1
contig002	AUGUSTUS	mRNA	318866	319654	1	-	.	ID=MALK_00729.t1;Parent=MALK_00729
contig002	AUGUSTUS	exon	318866	319654	.	-	.	ID=MALK_00729.t1.e1;Parent=MALK_00729.t1

contig002	AUGUSTUS	gene	319776	320369	0.99	+	.	ID=MALK_00730;prediction_source=braker_MRET:g285.t1
contig002	AUGUSTUS	CDS	319776	320369	0.99	+	0	ID=MALK_00730.t1.c1;Parent=MALK_00730.t1
contig002	AUGUSTUS	mRNA	319776	320369	0.99	+	.	ID=MALK_00730.t1;Parent=MALK_00730
contig002	AUGUSTUS	exon	319776	320369	.	+	.	ID=MALK_00730.t1.e1;Parent=MALK_00730.t1
contig002	AUGUSTUS	gene	320403	322118	0.9	-	.	ID=MALK_00731;prediction_source=braker_MRET:g286.t1
contig002	AUGUSTUS	CDS	320403	322118	0.9	-	0	ID=MALK_00731.t1.c1;Parent=MALK_00731.t1
contig002	AUGUSTUS	mRNA	320403	322118	0.9	-	.	ID=MALK_00731.t1;Parent=MALK_00731
contig002	AUGUSTUS	exon	320403	322118	.	-	.	ID=MALK_00731.t1.e1;Parent=MALK_00731.t1
contig002	AUGUSTUS	gene	322777	323199	1	+	.	ID=MALK_00732;prediction_source=augustus:contig002.g4145.t1
contig002	AUGUSTUS	CDS	322777	323199	1	+	0	ID=MALK_00732.t1.c1;Parent=MALK_00732.t1
contig002	AUGUSTUS	mRNA	322777	323199	1	+	.	ID=MALK_00732.t1;Parent=MALK_00732
contig002	AUGUSTUS	exon	322777	323199	1	+	.	ID=MALK_00732.t1.e1;Parent=MALK_00732.t1
contig002	AUGUSTUS	gene	323375	324667	0.64	-	.	ID=MALK_00733;prediction_source=augustus:contig002.g4146.t1
contig002	AUGUSTUS	CDS	323375	324667	0.64	-	0	ID=MALK_00733.t1.c1;Parent=MALK_00733.t1
contig002	AUGUSTUS	mRNA	323375	324667	0.64	-	.	ID=MALK_00733.t1;Parent=MALK_00733
contig002	AUGUSTUS	exon	323375	324667	0.64	-	.	ID=MALK_00733.t1.e1;Parent=MALK_00733.t1
contig002	AUGUSTUS	gene	324728	325714	0.89	-	.	ID=MALK_00734;prediction_source=augustus:contig002.g4147.t1
contig002	AUGUSTUS	CDS	324728	325714	0.89	-	0	ID=MALK_00734.t1.c1;Parent=MALK_00734.t1
contig002	AUGUSTUS	mRNA	324728	325714	0.89	-	.	ID=MALK_00734.t1;Parent=MALK_00734
contig002	AUGUSTUS	exon	324728	325714	0.89	-	.	ID=MALK_00734.t1.e1;Parent=MALK_00734.t1
contig002	maker	gene	326022	326654	.	+	.	ID=MALK_00735;prediction_source=maker_MRET:augustus_masked-contig002-processed-gene-3.11-mRNA-1
contig002	maker	CDS	326022	326654	.	+	0	ID=MALK_00735.t1.c1;Parent=MALK_00735.t1
contig002	maker	mRNA	326022	326654	.	+	.	ID=MALK_00735.t1;Parent=MALK_00735
contig002	maker	exon	326022	326654	.	+	.	ID=MALK_00735.t1.e1;Parent=MALK_00735.t1
contig002	maker	gene	326793	328073	.	-	.	ID=MALK_00736;prediction_source=maker_MRET:augustus_masked-contig002-processed-gene-3.37-mRNA-1
contig002	maker	CDS	326793	328073	.	-	0	ID=MALK_00736.t1.c1;Parent=MALK_00736.t1
contig002	maker	mRNA	326793	328073	.	-	.	ID=MALK_00736.t1;Parent=MALK_00736
contig002	maker	exon	326793	328073	.	-	.	ID=MALK_00736.t1.e1;Parent=MALK_00736.t1
contig002	maker	gene	328139	329488	.	+	.	ID=MALK_00737;prediction_source=maker_MRET:augustus_masked-contig002-processed-gene-3.12-mRNA-1
contig002	maker	CDS	328139	329488	.	+	0	ID=MALK_00737.t1.c1;Parent=MALK_00737.t1
contig002	maker	mRNA	328139	329488	.	+	.	ID=MALK_00737.t1;Parent=MALK_00737
contig002	maker	exon	328139	329488	.	+	.	ID=MALK_00737.t1.e1;Parent=MALK_00737.t1
contig002	maker	gene	329492	331605	.	-	.	ID=MALK_00738;prediction_source=maker_MRET:augustus_masked-contig002-processed-gene-3.38-mRNA-1
contig002	maker	CDS	329637	331605	.	-	0	ID=MALK_00738.t1.c1;Parent=MALK_00738.t1
contig002	maker	CDS	329492	329565	.	-	0	ID=MALK_00738.t1.c2;Parent=MALK_00738.t1
contig002	maker	mRNA	329492	331605	.	-	.	ID=MALK_00738.t1;Parent=MALK_00738
contig002	maker	exon	329637	331605	.	-	.	ID=MALK_00738.t1.e1;Parent=MALK_00738.t1
contig002	maker	exon	329492	329565	.	-	.	ID=MALK_00738.t1.e2;Parent=MALK_00738.t1
contig002	AUGUSTUS	gene	331805	333817	0.95	+	.	ID=MALK_00739;prediction_source=braker_MRET:g294.t1
contig002	AUGUSTUS	CDS	331805	333817	0.95	+	0	ID=MALK_00739.t1.c1;Parent=MALK_00739.t1
contig002	AUGUSTUS	mRNA	331805	333817	0.95	+	.	ID=MALK_00739.t1;Parent=MALK_00739
contig002	AUGUSTUS	exon	331805	333817	.	+	.	ID=MALK_00739.t1.e1;Parent=MALK_00739.t1
contig002	AUGUSTUS	gene	333913	334292	0.41	+	.	ID=MALK_00740;prediction_source=braker_MRET:g295.t1

contig002	AUGUSTUS	CDS	333913	334054	0.97	+	0	ID=MALK_00740.t1.c1;Parent=MALK_00740.t1
contig002	AUGUSTUS	CDS	334102	334292	0.97	+	0	ID=MALK_00740.t1.c2;Parent=MALK_00740.t1
contig002	AUGUSTUS	mRNA	333913	334292	0.41	+	.	ID=MALK_00740.t1;Parent=MALK_00740
contig002	AUGUSTUS	exon	333913	334054	.	+	.	ID=MALK_00740.t1.e1;Parent=MALK_00740.t1
contig002	AUGUSTUS	exon	334102	334292	.	+	.	ID=MALK_00740.t1.e2;Parent=MALK_00740.t1
contig002	AUGUSTUS	gene	334378	336402	0.66	+	.	ID=MALK_00741;prediction_source=braker_MRET:g296.t1
contig002	AUGUSTUS	CDS	334378	334672	0.78	+	0	ID=MALK_00741.t1.c1;Parent=MALK_00741.t1
contig002	AUGUSTUS	CDS	334737	335760	0.78	+	0	ID=MALK_00741.t1.c2;Parent=MALK_00741.t1
contig002	AUGUSTUS	CDS	335814	336402	0.78	+	0	ID=MALK_00741.t1.c3;Parent=MALK_00741.t1
contig002	AUGUSTUS	mRNA	334378	336402	0.66	+	.	ID=MALK_00741.t1;Parent=MALK_00741
contig002	AUGUSTUS	exon	334378	334672	.	+	.	ID=MALK_00741.t1.e1;Parent=MALK_00741.t1
contig002	AUGUSTUS	exon	334737	335760	.	+	.	ID=MALK_00741.t1.e2;Parent=MALK_00741.t1
contig002	AUGUSTUS	exon	335814	336402	.	+	.	ID=MALK_00741.t1.e3;Parent=MALK_00741.t1
contig002	AUGUSTUS	gene	336449	337068	0.93	-	.	ID=MALK_00742;prediction_source=braker_MRET:g297.t1
contig002	AUGUSTUS	CDS	337061	337068	0.93	-	0	ID=MALK_00742.t1.c3;Parent=MALK_00742.t1
contig002	AUGUSTUS	CDS	336869	337026	0.93	-	0	ID=MALK_00742.t1.c2;Parent=MALK_00742.t1
contig002	AUGUSTUS	CDS	336449	336840	0.93	-	0	ID=MALK_00742.t1.c1;Parent=MALK_00742.t1
contig002	AUGUSTUS	mRNA	336449	337068	0.93	-	.	ID=MALK_00742.t1;Parent=MALK_00742
contig002	AUGUSTUS	exon	337061	337068	.	-	.	ID=MALK_00742.t1.e3;Parent=MALK_00742.t1
contig002	AUGUSTUS	exon	336869	337026	.	-	.	ID=MALK_00742.t1.e2;Parent=MALK_00742.t1
contig002	AUGUSTUS	exon	336449	336840	.	-	.	ID=MALK_00742.t1.e1;Parent=MALK_00742.t1
contig002	AUGUSTUS	gene	337160	337744	0.54	-	.	ID=MALK_00743;prediction_source=augustus:contig002.g4154.t1
contig002	AUGUSTUS	CDS	337160	337744	0.54	-	0	ID=MALK_00743.t1.c1;Parent=MALK_00743.t1
contig002	AUGUSTUS	mRNA	337160	337744	0.54	-	.	ID=MALK_00743.t1;Parent=MALK_00743
contig002	AUGUSTUS	exon	337160	337744	0.54	-	.	ID=MALK_00743.t1.e1;Parent=MALK_00743.t1
contig002	maker	gene	337855	341277	.	-	.	ID=MALK_00744;prediction_source=maker_MRET:augustus_masked-contig002-processed-gene-3.40-mRNA-1
contig002	maker	CDS	337855	341277	.	-	0	ID=MALK_00744.t1.c1;Parent=MALK_00744.t1
contig002	maker	mRNA	337855	341277	.	-	.	ID=MALK_00744.t1;Parent=MALK_00744
contig002	maker	exon	337855	341277	.	-	.	ID=MALK_00744.t1.e1;Parent=MALK_00744.t1
contig002	maker	gene	341747	342214	.	-	.	ID=MALK_00745;prediction_source=maker_MRET:augustus_masked-contig002-processed-gene-3.41-mRNA-1
contig002	maker	CDS	341747	342214	.	-	0	ID=MALK_00745.t1.c1;Parent=MALK_00745.t1
contig002	maker	mRNA	341747	342214	.	-	.	ID=MALK_00745.t1;Parent=MALK_00745
contig002	maker	exon	341747	342214	.	-	.	ID=MALK_00745.t1.e1;Parent=MALK_00745.t1
contig002	AUGUSTUS	gene	342439	343776	0.54	+	.	ID=MALK_00746;prediction_source=braker_MRET:g301.t1
contig002	AUGUSTUS	CDS	342439	343776	0.54	+	0	ID=MALK_00746.t1.c1;Parent=MALK_00746.t1
contig002	AUGUSTUS	mRNA	342439	343776	0.54	+	.	ID=MALK_00746.t1;Parent=MALK_00746
contig002	AUGUSTUS	exon	342439	343776	.	+	.	ID=MALK_00746.t1.e1;Parent=MALK_00746.t1
contig002	AUGUSTUS	gene	343969	346404	0.95	+	.	ID=MALK_00747;prediction_source=augustus:contig002.g4158.t1
contig002	AUGUSTUS	CDS	343969	346404	0.95	+	0	ID=MALK_00747.t1.c1;Parent=MALK_00747.t1
contig002	AUGUSTUS	mRNA	343969	346404	0.95	+	.	ID=MALK_00747.t1;Parent=MALK_00747
contig002	AUGUSTUS	exon	343969	346404	0.95	+	.	ID=MALK_00747.t1.e1;Parent=MALK_00747.t1
contig002	AUGUSTUS	gene	346574	347320	0.57	+	.	ID=MALK_00748;prediction_source=augustus:contig002.g4159.t1
contig002	AUGUSTUS	CDS	346574	347320	0.57	+	0	ID=MALK_00748.t1.c1;Parent=MALK_00748.t1

contig002	AUGUSTUS	mRNA	346574	347320	0.57	+	.	ID=MALK_00748.t1;Parent=MALK_00748
contig002	AUGUSTUS	exon	346574	347320	0.57	+	.	ID=MALK_00748.t1.e1;Parent=MALK_00748.t1
contig002	maker	gene	347345	350424	.	-	.	ID=MALK_00749;prediction_source=maker_MRET:augustus_masked-contig002-processed-gene-3.42-mRNA-1
contig002	maker	CDS	348595	350424	.	-	0	ID=MALK_00749.t1.c1;Parent=MALK_00749.t1
contig002	maker	CDS	347345	348469	.	-	0	ID=MALK_00749.t1.c2;Parent=MALK_00749.t1
contig002	maker	mRNA	347345	350424	.	-	.	ID=MALK_00749.t1;Parent=MALK_00749
contig002	maker	exon	348595	350424	.	-	.	ID=MALK_00749.t1.e1;Parent=MALK_00749.t1
contig002	maker	exon	347345	348469	.	-	.	ID=MALK_00749.t1.e2;Parent=MALK_00749.t1
contig002	maker	gene	350681	351970	.	+	.	ID=MALK_00750;prediction_source=maker_MRET:augustus_masked-contig002-processed-gene-3.16-mRNA-1
contig002	maker	CDS	350681	351970	.	+	0	ID=MALK_00750.t1.c1;Parent=MALK_00750.t1
contig002	maker	mRNA	350681	351970	.	+	.	ID=MALK_00750.t1;Parent=MALK_00750
contig002	maker	exon	350681	351970	.	+	.	ID=MALK_00750.t1.e1;Parent=MALK_00750.t1
contig002	AUGUSTUS	gene	351994	352552	0.66	-	.	ID=MALK_00751;prediction_source=braker_MRET:g306.t1
contig002	AUGUSTUS	CDS	352539	352552	0.89	-	0	ID=MALK_00751.t1.c2;Parent=MALK_00751.t1
contig002	AUGUSTUS	CDS	351994	352507	0.89	-	0	ID=MALK_00751.t1.c1;Parent=MALK_00751.t1
contig002	AUGUSTUS	mRNA	351994	352552	0.66	-	.	ID=MALK_00751.t1;Parent=MALK_00751
contig002	AUGUSTUS	exon	352539	352552	.	-	.	ID=MALK_00751.t1.e2;Parent=MALK_00751.t1
contig002	AUGUSTUS	exon	351994	352507	.	-	.	ID=MALK_00751.t1.e1;Parent=MALK_00751.t1
contig002	AUGUSTUS	gene	352626	353201	0.81	+	.	ID=MALK_00752;prediction_source=braker_MRET:g307.t1
contig002	AUGUSTUS	CDS	352626	353201	0.81	+	0	ID=MALK_00752.t1.c1;Parent=MALK_00752.t1
contig002	AUGUSTUS	mRNA	352626	353201	0.81	+	.	ID=MALK_00752.t1;Parent=MALK_00752
contig002	AUGUSTUS	exon	352626	353201	.	+	.	ID=MALK_00752.t1.e1;Parent=MALK_00752.t1
contig002	AUGUSTUS	gene	353220	356471	1	-	.	ID=MALK_00753;prediction_source=augustus:contig002.g4167.t1
contig002	AUGUSTUS	CDS	353220	356471	1	-	0	ID=MALK_00753.t1.c1;Parent=MALK_00753.t1
contig002	AUGUSTUS	mRNA	353220	356471	1	-	.	ID=MALK_00753.t1;Parent=MALK_00753
contig002	AUGUSTUS	exon	353220	356471	1	-	.	ID=MALK_00753.t1.e1;Parent=MALK_00753.t1
contig002	maker	gene	356757	357680	.	-	.	ID=MALK_00754;prediction_source=maker_MRET:augustus_masked-contig002-processed-gene-3.45-mRNA-1
contig002	maker	CDS	356757	357680	.	-	0	ID=MALK_00754.t1.c1;Parent=MALK_00754.t1
contig002	maker	mRNA	356757	357680	.	-	.	ID=MALK_00754.t1;Parent=MALK_00754
contig002	maker	exon	356757	357680	.	-	.	ID=MALK_00754.t1.e1;Parent=MALK_00754.t1
contig002	AUGUSTUS	gene	357969	358892	0.95	+	.	ID=MALK_00755;prediction_source=augustus:contig002.g4169.t1
contig002	AUGUSTUS	CDS	357969	358892	0.95	+	0	ID=MALK_00755.t1.c1;Parent=MALK_00755.t1
contig002	AUGUSTUS	mRNA	357969	358892	0.95	+	.	ID=MALK_00755.t1;Parent=MALK_00755
contig002	AUGUSTUS	exon	357969	358892	0.95	+	.	ID=MALK_00755.t1.e1;Parent=MALK_00755.t1
contig002	AUGUSTUS	gene	358929	360119	0.97	-	.	ID=MALK_00756;prediction_source=augustus:contig002.g4170.t1
contig002	AUGUSTUS	CDS	358929	360119	0.97	-	0	ID=MALK_00756.t1.c1;Parent=MALK_00756.t1
contig002	AUGUSTUS	mRNA	358929	360119	0.97	-	.	ID=MALK_00756.t1;Parent=MALK_00756
contig002	AUGUSTUS	exon	358929	360119	0.97	-	.	ID=MALK_00756.t1.e1;Parent=MALK_00756.t1
contig002	maker	gene	360249	360779	.	-	.	ID=MALK_00757;prediction_source=maker_MRET:augustus_masked-contig002-processed-gene-3.47-mRNA-1
contig002	maker	CDS	360249	360779	.	-	0	ID=MALK_00757.t1.c1;Parent=MALK_00757.t1
contig002	maker	mRNA	360249	360779	.	-	.	ID=MALK_00757.t1;Parent=MALK_00757
contig002	maker	exon	360249	360779	.	-	.	ID=MALK_00757.t1.e1;Parent=MALK_00757.t1
contig002	AUGUSTUS	gene	361793	362264	0.48	-	.	ID=MALK_00758;prediction_source=augustus:contig002.g4173.t1

contig002	AUGUSTUS	CDS	362228	362264	0.5	-	0	ID=MALK_00758.t1.c2;Parent=MALK_00758.t1
contig002	AUGUSTUS	CDS	361793	362133	0.5	-	0	ID=MALK_00758.t1.c1;Parent=MALK_00758.t1
contig002	AUGUSTUS	mRNA	361793	362264	0.48	-	.	ID=MALK_00758.t1;Parent=MALK_00758
contig002	AUGUSTUS	exon	362228	362264	0.5	-	.	ID=MALK_00758.t1.e2;Parent=MALK_00758.t1
contig002	AUGUSTUS	exon	361793	362133	0.5	-	.	ID=MALK_00758.t1.e1;Parent=MALK_00758.t1
contig002	maker	gene	362413	363441	.	-	.	ID=MALK_00759;prediction_source=maker_MRET:augustus_masked-contig002-processed-gene-3.48-mRNA-1
contig002	maker	CDS	362413	363441	.	-	0	ID=MALK_00759.t1.c1;Parent=MALK_00759.t1
contig002	maker	mRNA	362413	363441	.	-	.	ID=MALK_00759.t1;Parent=MALK_00759
contig002	maker	exon	362413	363441	.	-	.	ID=MALK_00759.t1.e1;Parent=MALK_00759.t1
contig002	AUGUSTUS	gene	363893	366475	1	+	.	ID=MALK_00760;prediction_source=augustus:contig002.g4175.t1
contig002	AUGUSTUS	CDS	363893	366475	1	+	0	ID=MALK_00760.t1.c1;Parent=MALK_00760.t1
contig002	AUGUSTUS	mRNA	363893	366475	1	+	.	ID=MALK_00760.t1;Parent=MALK_00760
contig002	AUGUSTUS	exon	363893	366475	1	+	.	ID=MALK_00760.t1.e1;Parent=MALK_00760.t1
contig002	AUGUSTUS	gene	366761	367962	0.39	-	.	ID=MALK_00761;prediction_source=braker_MRET:g315.t1
contig002	AUGUSTUS	CDS	367832	367962	0.8	-	0	ID=MALK_00761.t1.c5;Parent=MALK_00761.t1
contig002	AUGUSTUS	CDS	367633	367790	0.8	-	0	ID=MALK_00761.t1.c4;Parent=MALK_00761.t1
contig002	AUGUSTUS	CDS	367170	367454	0.8	-	0	ID=MALK_00761.t1.c3;Parent=MALK_00761.t1
contig002	AUGUSTUS	CDS	366957	367141	0.8	-	0	ID=MALK_00761.t1.c2;Parent=MALK_00761.t1
contig002	AUGUSTUS	CDS	366761	366841	0.8	-	0	ID=MALK_00761.t1.c1;Parent=MALK_00761.t1
contig002	AUGUSTUS	mRNA	366761	367962	0.39	-	.	ID=MALK_00761.t1;Parent=MALK_00761
contig002	AUGUSTUS	exon	367832	367962	.	-	.	ID=MALK_00761.t1.e5;Parent=MALK_00761.t1
contig002	AUGUSTUS	exon	367633	367790	.	-	.	ID=MALK_00761.t1.e4;Parent=MALK_00761.t1
contig002	AUGUSTUS	exon	367170	367454	.	-	.	ID=MALK_00761.t1.e3;Parent=MALK_00761.t1
contig002	AUGUSTUS	exon	366957	367141	.	-	.	ID=MALK_00761.t1.e2;Parent=MALK_00761.t1
contig002	AUGUSTUS	exon	366761	366841	.	-	.	ID=MALK_00761.t1.e1;Parent=MALK_00761.t1
contig002	AUGUSTUS	gene	368028	368562	0.22	+	.	ID=MALK_00762;prediction_source=braker_MRET:g316.t1
contig002	AUGUSTUS	CDS	368028	368132	0.33	+	0	ID=MALK_00762.t1.c1;Parent=MALK_00762.t1
contig002	AUGUSTUS	CDS	368173	368562	0.33	+	0	ID=MALK_00762.t1.c2;Parent=MALK_00762.t1
contig002	AUGUSTUS	mRNA	368028	368562	0.22	+	.	ID=MALK_00762.t1;Parent=MALK_00762
contig002	AUGUSTUS	exon	368028	368132	.	+	.	ID=MALK_00762.t1.e1;Parent=MALK_00762.t1
contig002	AUGUSTUS	exon	368173	368562	.	+	.	ID=MALK_00762.t1.e2;Parent=MALK_00762.t1
contig002	AUGUSTUS	gene	368628	369287	0.29	+	.	ID=MALK_00763;prediction_source=braker_MRET:g317.t1
contig002	AUGUSTUS	CDS	368628	369287	0.29	+	0	ID=MALK_00763.t1.c1;Parent=MALK_00763.t1
contig002	AUGUSTUS	mRNA	368628	369287	0.29	+	.	ID=MALK_00763.t1;Parent=MALK_00763
contig002	AUGUSTUS	exon	368628	369287	.	+	.	ID=MALK_00763.t1.e1;Parent=MALK_00763.t1
contig002	AUGUSTUS	gene	369352	369744	0.8	-	.	ID=MALK_00764;prediction_source=augustus:contig002.g4178.t1
contig002	AUGUSTUS	CDS	369352	369744	0.8	-	0	ID=MALK_00764.t1.c1;Parent=MALK_00764.t1
contig002	AUGUSTUS	mRNA	369352	369744	0.8	-	.	ID=MALK_00764.t1;Parent=MALK_00764
contig002	AUGUSTUS	exon	369352	369744	0.8	-	.	ID=MALK_00764.t1.e1;Parent=MALK_00764.t1
contig002	AUGUSTUS	gene	370523	371668	0.11	-	.	ID=MALK_00765;prediction_source=braker_MRET:g319.t1
contig002	AUGUSTUS	CDS	371503	371668	0.41	-	0	ID=MALK_00765.t1.c5;Parent=MALK_00765.t1
contig002	AUGUSTUS	CDS	370980	371445	0.41	-	0	ID=MALK_00765.t1.c4;Parent=MALK_00765.t1
contig002	AUGUSTUS	CDS	370886	370937	0.41	-	0	ID=MALK_00765.t1.c3;Parent=MALK_00765.t1

contig002	AUGUSTUS	CDS	370664	370851	0.41	-	0	ID=MALK_00765.t1.c2;Parent=MALK_00765.t1
contig002	AUGUSTUS	CDS	370523	370613	0.41	-	0	ID=MALK_00765.t1.c1;Parent=MALK_00765.t1
contig002	AUGUSTUS	mRNA	370523	371668	0.11	-	.	ID=MALK_00765.t1;Parent=MALK_00765
contig002	AUGUSTUS	exon	371503	371668	.	-	.	ID=MALK_00765.t1.e5;Parent=MALK_00765.t1
contig002	AUGUSTUS	exon	370980	371445	.	-	.	ID=MALK_00765.t1.e4;Parent=MALK_00765.t1
contig002	AUGUSTUS	exon	370886	370937	.	-	.	ID=MALK_00765.t1.e3;Parent=MALK_00765.t1
contig002	AUGUSTUS	exon	370664	370851	.	-	.	ID=MALK_00765.t1.e2;Parent=MALK_00765.t1
contig002	AUGUSTUS	exon	370523	370613	.	-	.	ID=MALK_00765.t1.e1;Parent=MALK_00765.t1
contig002	AUGUSTUS	gene	372250	373476	0.93	-	.	ID=MALK_00766;prediction_source=braker_MRET:g320.t1
contig002	AUGUSTUS	CDS	372250	373476	0.93	-	0	ID=MALK_00766.t1.c1;Parent=MALK_00766.t1
contig002	AUGUSTUS	mRNA	372250	373476	0.93	-	.	ID=MALK_00766.t1;Parent=MALK_00766
contig002	AUGUSTUS	exon	372250	373476	.	-	.	ID=MALK_00766.t1.e1;Parent=MALK_00766.t1
contig002	AUGUSTUS	gene	373799	376378	0.86	-	.	ID=MALK_00767;prediction_source=augustus:contig002.g4180.t1
contig002	AUGUSTUS	CDS	373799	376378	0.86	-	0	ID=MALK_00767.t1.c1;Parent=MALK_00767.t1
contig002	AUGUSTUS	mRNA	373799	376378	0.86	-	.	ID=MALK_00767.t1;Parent=MALK_00767
contig002	AUGUSTUS	exon	373799	376378	0.86	-	.	ID=MALK_00767.t1.e1;Parent=MALK_00767.t1
contig002	AUGUSTUS	gene	376727	377755	0.42	+	.	ID=MALK_00768;prediction_source=augustus:contig002.g4182.t1
contig002	AUGUSTUS	CDS	376727	377755	0.42	+	0	ID=MALK_00768.t1.c1;Parent=MALK_00768.t1
contig002	AUGUSTUS	mRNA	376727	377755	0.42	+	.	ID=MALK_00768.t1;Parent=MALK_00768
contig002	AUGUSTUS	exon	376727	377755	0.42	+	.	ID=MALK_00768.t1.e1;Parent=MALK_00768.t1
contig002	AUGUSTUS	gene	377808	378896	0.69	+	.	ID=MALK_00769;prediction_source=augustus:contig002.g4183.t1
contig002	AUGUSTUS	CDS	377808	378896	0.69	+	0	ID=MALK_00769.t1.c1;Parent=MALK_00769.t1
contig002	AUGUSTUS	mRNA	377808	378896	0.69	+	.	ID=MALK_00769.t1;Parent=MALK_00769
contig002	AUGUSTUS	exon	377808	378896	0.69	+	.	ID=MALK_00769.t1.e1;Parent=MALK_00769.t1
contig002	AUGUSTUS	gene	378893	380458	0.85	-	.	ID=MALK_00770;prediction_source=augustus:contig002.g4184.t1
contig002	AUGUSTUS	CDS	378893	380458	0.85	-	0	ID=MALK_00770.t1.c1;Parent=MALK_00770.t1
contig002	AUGUSTUS	mRNA	378893	380458	0.85	-	.	ID=MALK_00770.t1;Parent=MALK_00770
contig002	AUGUSTUS	exon	378893	380458	0.85	-	.	ID=MALK_00770.t1.e1;Parent=MALK_00770.t1
contig002	AUGUSTUS	gene	380637	381398	1	+	.	ID=MALK_00771;prediction_source=augustus:contig002.g4185.t1
contig002	AUGUSTUS	CDS	380637	381398	1	+	0	ID=MALK_00771.t1.c1;Parent=MALK_00771.t1
contig002	AUGUSTUS	mRNA	380637	381398	1	+	.	ID=MALK_00771.t1;Parent=MALK_00771
contig002	AUGUSTUS	exon	380637	381398	1	+	.	ID=MALK_00771.t1.e1;Parent=MALK_00771.t1
contig002	maker	gene	381514	383842	.	-	.	ID=MALK_00772;prediction_source=maker_MRET:augustus_masked-contig002-processed-gene-3.54-mRNA-1
contig002	maker	CDS	383767	383842	.	-	0	ID=MALK_00772.t1.c1;Parent=MALK_00772.t1
contig002	maker	CDS	382170	383600	.	-	0	ID=MALK_00772.t1.c2;Parent=MALK_00772.t1
contig002	maker	CDS	381514	382073	.	-	0	ID=MALK_00772.t1.c3;Parent=MALK_00772.t1
contig002	maker	mRNA	381514	383842	.	-	.	ID=MALK_00772.t1;Parent=MALK_00772
contig002	maker	exon	383767	383842	.	-	.	ID=MALK_00772.t1.e1;Parent=MALK_00772.t1
contig002	maker	exon	382170	383600	.	-	.	ID=MALK_00772.t1.e2;Parent=MALK_00772.t1
contig002	maker	exon	381514	382073	.	-	.	ID=MALK_00772.t1.e3;Parent=MALK_00772.t1
contig002	AUGUSTUS	gene	383748	385208	0.53	+	.	ID=MALK_00773;prediction_source=augustus:contig002.g4187.t1
contig002	AUGUSTUS	CDS	383748	385208	0.53	+	0	ID=MALK_00773.t1.c1;Parent=MALK_00773.t1
contig002	AUGUSTUS	mRNA	383748	385208	0.53	+	.	ID=MALK_00773.t1;Parent=MALK_00773

contig002	AUGUSTUS	exon	383748	385208	0.53	+	.	ID=MALK_00773.t1.e1;Parent=MALK_00773.t1
contig002	AUGUSTUS	gene	385201	387396	0.62	-	.	ID=MALK_00774;prediction_source=augustus:contig002.g4188.t1
contig002	AUGUSTUS	CDS	385201	387396	0.62	-	0	ID=MALK_00774.t1.c1;Parent=MALK_00774.t1
contig002	AUGUSTUS	mRNA	385201	387396	0.62	-	.	ID=MALK_00774.t1;Parent=MALK_00774
contig002	AUGUSTUS	exon	385201	387396	0.62	-	.	ID=MALK_00774.t1.e1;Parent=MALK_00774.t1
contig002	AUGUSTUS	gene	387690	389552	0.58	+	.	ID=MALK_00775;prediction_source=augustus:contig002.g4189.t1
contig002	AUGUSTUS	CDS	387690	389552	0.58	+	0	ID=MALK_00775.t1.c1;Parent=MALK_00775.t1
contig002	AUGUSTUS	mRNA	387690	389552	0.58	+	.	ID=MALK_00775.t1;Parent=MALK_00775
contig002	AUGUSTUS	exon	387690	389552	0.58	+	.	ID=MALK_00775.t1.e1;Parent=MALK_00775.t1
contig002	AUGUSTUS	gene	389487	391274	0.93	-	.	ID=MALK_00776;prediction_source=augustus:contig002.g4190.t1
contig002	AUGUSTUS	CDS	389487	391274	0.93	-	0	ID=MALK_00776.t1.c1;Parent=MALK_00776.t1
contig002	AUGUSTUS	mRNA	389487	391274	0.93	-	.	ID=MALK_00776.t1;Parent=MALK_00776
contig002	AUGUSTUS	exon	389487	391274	0.93	-	.	ID=MALK_00776.t1.e1;Parent=MALK_00776.t1
contig002	AUGUSTUS	gene	391355	395461	0.97	+	.	ID=MALK_00777;prediction_source=braker_MRET:g331.t1
contig002	AUGUSTUS	CDS	391355	395461	0.97	+	0	ID=MALK_00777.t1.c1;Parent=MALK_00777.t1
contig002	AUGUSTUS	mRNA	391355	395461	0.97	+	.	ID=MALK_00777.t1;Parent=MALK_00777
contig002	AUGUSTUS	exon	391355	395461	.	+	.	ID=MALK_00777.t1.e1;Parent=MALK_00777.t1
contig002	AUGUSTUS	gene	395802	396770	0.94	+	.	ID=MALK_00778;prediction_source=augustus:contig002.g4193.t1
contig002	AUGUSTUS	CDS	395802	396770	0.94	+	0	ID=MALK_00778.t1.c1;Parent=MALK_00778.t1
contig002	AUGUSTUS	mRNA	395802	396770	0.94	+	.	ID=MALK_00778.t1;Parent=MALK_00778
contig002	AUGUSTUS	exon	395802	396770	0.94	+	.	ID=MALK_00778.t1.e1;Parent=MALK_00778.t1
contig002	AUGUSTUS	gene	396771	399671	0.93	-	.	ID=MALK_00779;prediction_source=augustus:contig002.g4194.t1
contig002	AUGUSTUS	CDS	396771	399671	0.93	-	0	ID=MALK_00779.t1.c1;Parent=MALK_00779.t1
contig002	AUGUSTUS	mRNA	396771	399671	0.93	-	.	ID=MALK_00779.t1;Parent=MALK_00779
contig002	AUGUSTUS	exon	396771	399671	0.93	-	.	ID=MALK_00779.t1.e1;Parent=MALK_00779.t1
contig002	maker	gene	401724	403946	.	+	.	ID=MALK_00780;prediction_source=maker_MRET:augustus_masked-contig002-processed-gene-3.26-mRNA-1
contig002	maker	CDS	401724	403946	.	+	0	ID=MALK_00780.t1.c1;Parent=MALK_00780.t1
contig002	maker	mRNA	401724	403946	.	+	.	ID=MALK_00780.t1;Parent=MALK_00780
contig002	maker	exon	401724	403946	.	+	.	ID=MALK_00780.t1.e1;Parent=MALK_00780.t1
contig002	AUGUSTUS	gene	403989	404936	0.33	-	.	ID=MALK_00781;prediction_source=augustus:contig002.g4196.t1
contig002	AUGUSTUS	CDS	403989	404936	0.33	-	0	ID=MALK_00781.t1.c1;Parent=MALK_00781.t1
contig002	AUGUSTUS	mRNA	403989	404936	0.33	-	.	ID=MALK_00781.t1;Parent=MALK_00781
contig002	AUGUSTUS	exon	403989	404936	0.33	-	.	ID=MALK_00781.t1.e1;Parent=MALK_00781.t1
contig002	AUGUSTUS	gene	404977	405468	0.53	+	.	ID=MALK_00782;prediction_source=augustus:contig002.g4197.t1
contig002	AUGUSTUS	CDS	404977	405468	0.53	+	0	ID=MALK_00782.t1.c1;Parent=MALK_00782.t1
contig002	AUGUSTUS	mRNA	404977	405468	0.53	+	.	ID=MALK_00782.t1;Parent=MALK_00782
contig002	AUGUSTUS	exon	404977	405468	0.53	+	.	ID=MALK_00782.t1.e1;Parent=MALK_00782.t1
contig002	AUGUSTUS	gene	406455	407054	0.96	+	.	ID=MALK_00783;prediction_source=augustus:contig002.g4198.t1
contig002	AUGUSTUS	CDS	406455	407054	0.96	+	0	ID=MALK_00783.t1.c1;Parent=MALK_00783.t1
contig002	AUGUSTUS	mRNA	406455	407054	0.96	+	.	ID=MALK_00783.t1;Parent=MALK_00783
contig002	AUGUSTUS	exon	406455	407054	0.96	+	.	ID=MALK_00783.t1.e1;Parent=MALK_00783.t1
contig003	annotation	remark	1	771082	.	+	.	gff-version=3
contig003	AUGUSTUS	gene	2	778	0.4	-	.	ID=MALK_00784;prediction_source=braker_MRET:g2833.t1

contig003	AUGUSTUS	CDS	2	778	0.4	-	0	ID=MALK_00784.t1.c1;Parent=MALK_00784.t1
contig003	AUGUSTUS	mRNA	2	778	0.4	-	.	ID=MALK_00784.t1;Parent=MALK_00784
contig003	AUGUSTUS	exon	2	778	.	-	.	ID=MALK_00784.t1.e1;Parent=MALK_00784.t1
contig003	AUGUSTUS	gene	1192	1606	0.81	+	.	ID=MALK_00785;prediction_source=braker_MRET:g2834.t1
contig003	AUGUSTUS	CDS	1192	1402	0.84	+	0	ID=MALK_00785.t1.c1;Parent=MALK_00785.t1
contig003	AUGUSTUS	CDS	1431	1606	0.84	+	0	ID=MALK_00785.t1.c2;Parent=MALK_00785.t1
contig003	AUGUSTUS	mRNA	1192	1606	0.81	+	.	ID=MALK_00785.t1;Parent=MALK_00785
contig003	AUGUSTUS	exon	1192	1402	.	+	.	ID=MALK_00785.t1.e1;Parent=MALK_00785.t1
contig003	AUGUSTUS	exon	1431	1606	.	+	.	ID=MALK_00785.t1.e2;Parent=MALK_00785.t1
contig003	AUGUSTUS	gene	1788	2570	0.66	+	.	ID=MALK_00786;prediction_source=braker_MRET:g2835.t1
contig003	AUGUSTUS	CDS	1788	2570	0.66	+	0	ID=MALK_00786.t1.c1;Parent=MALK_00786.t1
contig003	AUGUSTUS	mRNA	1788	2570	0.66	+	.	ID=MALK_00786.t1;Parent=MALK_00786
contig003	AUGUSTUS	exon	1788	2570	.	+	.	ID=MALK_00786.t1.e1;Parent=MALK_00786.t1
contig003	AUGUSTUS	gene	2951	5365	0.65	-	.	ID=MALK_00787;prediction_source=braker_MRET:g2836.t1
contig003	AUGUSTUS	CDS	2951	5365	0.65	-	0	ID=MALK_00787.t1.c1;Parent=MALK_00787.t1
contig003	AUGUSTUS	mRNA	2951	5365	0.65	-	.	ID=MALK_00787.t1;Parent=MALK_00787
contig003	AUGUSTUS	exon	2951	5365	.	-	.	ID=MALK_00787.t1.e1;Parent=MALK_00787.t1
contig003	AUGUSTUS	gene	5471	7777	0.7	+	.	ID=MALK_00788;prediction_source=braker_MRET:g2837.t1
contig003	AUGUSTUS	CDS	5471	7777	0.7	+	0	ID=MALK_00788.t1.c1;Parent=MALK_00788.t1
contig003	AUGUSTUS	mRNA	5471	7777	0.7	+	.	ID=MALK_00788.t1;Parent=MALK_00788
contig003	AUGUSTUS	exon	5471	7777	.	+	.	ID=MALK_00788.t1.e1;Parent=MALK_00788.t1
contig003	maker	gene	8464	9777	.	-	.	ID=MALK_00789;prediction_source=maker_MRET:augustus_masked-contig003-processed-gene-0.22-mRNA-1
contig003	maker	CDS	8464	9777	.	-	0	ID=MALK_00789.t1.c1;Parent=MALK_00789.t1
contig003	maker	mRNA	8464	9777	.	-	.	ID=MALK_00789.t1;Parent=MALK_00789
contig003	maker	exon	8464	9777	.	-	.	ID=MALK_00789.t1.e1;Parent=MALK_00789.t1
contig003	maker	gene	9912	11162	.	+	.	ID=MALK_00790;prediction_source=maker_MRET:augustus_masked-contig003-processed-gene-0.2-mRNA-1
contig003	maker	CDS	9912	11162	.	+	0	ID=MALK_00790.t1.c1;Parent=MALK_00790.t1
contig003	maker	mRNA	9912	11162	.	+	.	ID=MALK_00790.t1;Parent=MALK_00790
contig003	maker	exon	9912	11162	.	+	.	ID=MALK_00790.t1.e1;Parent=MALK_00790.t1
contig003	AUGUSTUS	gene	11149	13563	0.56	-	.	ID=MALK_00791;prediction_source=augustus:contig003.g3112.t1
contig003	AUGUSTUS	CDS	11149	13563	0.56	-	0	ID=MALK_00791.t1.c1;Parent=MALK_00791.t1
contig003	AUGUSTUS	mRNA	11149	13563	0.56	-	.	ID=MALK_00791.t1;Parent=MALK_00791
contig003	AUGUSTUS	exon	11149	13563	0.56	-	.	ID=MALK_00791.t1.e1;Parent=MALK_00791.t1
contig003	maker	gene	13505	14851	.	+	.	ID=MALK_00792;prediction_source=maker_MRET:augustus_masked-contig003-processed-gene-0.3-mRNA-1
contig003	maker	CDS	13505	14851	.	+	0	ID=MALK_00792.t1.c1;Parent=MALK_00792.t1
contig003	maker	mRNA	13505	14851	.	+	.	ID=MALK_00792.t1;Parent=MALK_00792
contig003	maker	exon	13505	14851	.	+	.	ID=MALK_00792.t1.e1;Parent=MALK_00792.t1
contig003	AUGUSTUS	gene	14917	15964	0.32	-	.	ID=MALK_00793;prediction_source=braker_MRET:g2842.t1
contig003	AUGUSTUS	CDS	15077	15964	0.37	-	0	ID=MALK_00793.t1.c2;Parent=MALK_00793.t1
contig003	AUGUSTUS	CDS	14917	15015	0.37	-	0	ID=MALK_00793.t1.c1;Parent=MALK_00793.t1
contig003	AUGUSTUS	mRNA	14917	15964	0.32	-	.	ID=MALK_00793.t1;Parent=MALK_00793
contig003	AUGUSTUS	exon	15077	15964	.	-	.	ID=MALK_00793.t1.e2;Parent=MALK_00793.t1
contig003	AUGUSTUS	exon	14917	15015	.	-	.	ID=MALK_00793.t1.e1;Parent=MALK_00793.t1

contig003	maker	gene	15994	17070	.	-	.	ID=MALK_00794;prediction_source=maker_MRET:augustus_masked-contig003-processed-gene-0.24-mRNA-1
contig003	maker	CDS	15994	17070	.	-	0	ID=MALK_00794.t1.c1;Parent=MALK_00794.t1
contig003	maker	mRNA	15994	17070	.	-	.	ID=MALK_00794.t1;Parent=MALK_00794
contig003	maker	exon	15994	17070	.	-	.	ID=MALK_00794.t1.e1;Parent=MALK_00794.t1
contig003	AUGUSTUS	gene	17141	17695	0.87	+	.	ID=MALK_00795;prediction_source=braker_MRET:g2844.t1
contig003	AUGUSTUS	CDS	17141	17695	0.87	+	0	ID=MALK_00795.t1.c1;Parent=MALK_00795.t1
contig003	AUGUSTUS	mRNA	17141	17695	0.87	+	.	ID=MALK_00795.t1;Parent=MALK_00795
contig003	AUGUSTUS	exon	17141	17695	.	+	.	ID=MALK_00795.t1.e1;Parent=MALK_00795.t1
contig003	AUGUSTUS	gene	17882	21967	0.96	+	.	ID=MALK_00796;prediction_source=augustus:contig003.g3117.t1
contig003	AUGUSTUS	CDS	17882	21967	0.96	+	0	ID=MALK_00796.t1.c1;Parent=MALK_00796.t1
contig003	AUGUSTUS	mRNA	17882	21967	0.96	+	.	ID=MALK_00796.t1;Parent=MALK_00796
contig003	AUGUSTUS	exon	17882	21967	0.96	+	.	ID=MALK_00796.t1.e1;Parent=MALK_00796.t1
contig003	AUGUSTUS	gene	23461	23859	0.42	-	.	ID=MALK_00797;prediction_source=braker_MRET:g2846.t1
contig003	AUGUSTUS	CDS	23857	23859	0.53	-	0	ID=MALK_00797.t1.c3;Parent=MALK_00797.t1
contig003	AUGUSTUS	CDS	23695	23823	0.53	-	0	ID=MALK_00797.t1.c2;Parent=MALK_00797.t1
contig003	AUGUSTUS	CDS	23461	23661	0.53	-	0	ID=MALK_00797.t1.c1;Parent=MALK_00797.t1
contig003	AUGUSTUS	mRNA	23461	23859	0.42	-	.	ID=MALK_00797.t1;Parent=MALK_00797
contig003	AUGUSTUS	exon	23857	23859	.	-	.	ID=MALK_00797.t1.e3;Parent=MALK_00797.t1
contig003	AUGUSTUS	exon	23695	23823	.	-	.	ID=MALK_00797.t1.e2;Parent=MALK_00797.t1
contig003	AUGUSTUS	exon	23461	23661	.	-	.	ID=MALK_00797.t1.e1;Parent=MALK_00797.t1
contig003	AUGUSTUS	gene	24101	24473	0.66	+	.	ID=MALK_00798;prediction_source=braker_MRET:g2847.t1
contig003	AUGUSTUS	CDS	24101	24140	0.79	+	0	ID=MALK_00798.t1.c1;Parent=MALK_00798.t1
contig003	AUGUSTUS	CDS	24178	24473	0.79	+	0	ID=MALK_00798.t1.c2;Parent=MALK_00798.t1
contig003	AUGUSTUS	mRNA	24101	24473	0.66	+	.	ID=MALK_00798.t1;Parent=MALK_00798
contig003	AUGUSTUS	exon	24101	24140	.	+	.	ID=MALK_00798.t1.e1;Parent=MALK_00798.t1
contig003	AUGUSTUS	exon	24178	24473	.	+	.	ID=MALK_00798.t1.e2;Parent=MALK_00798.t1
contig003	AUGUSTUS	gene	24571	25689	0.98	+	.	ID=MALK_00799;prediction_source=augustus:contig003.g3118.t1
contig003	AUGUSTUS	CDS	24571	25689	0.98	+	0	ID=MALK_00799.t1.c1;Parent=MALK_00799.t1
contig003	AUGUSTUS	mRNA	24571	25689	0.98	+	.	ID=MALK_00799.t1;Parent=MALK_00799
contig003	AUGUSTUS	exon	24571	25689	0.98	+	.	ID=MALK_00799.t1.e1;Parent=MALK_00799.t1
contig003	maker	gene	26198	26903	.	-	.	ID=MALK_00800;prediction_source=maker_MRET:augustus_masked-contig003-processed-gene-0.25-mRNA-1
contig003	maker	CDS	26891	26903	.	-	0	ID=MALK_00800.t1.c1;Parent=MALK_00800.t1
contig003	maker	CDS	26198	26820	.	-	0	ID=MALK_00800.t1.c2;Parent=MALK_00800.t1
contig003	maker	mRNA	26198	26903	.	-	.	ID=MALK_00800.t1;Parent=MALK_00800
contig003	maker	exon	26891	26903	.	-	.	ID=MALK_00800.t1.e1;Parent=MALK_00800.t1
contig003	maker	exon	26198	26820	.	-	.	ID=MALK_00800.t1.e2;Parent=MALK_00800.t1
contig003	maker	gene	26999	28750	.	+	.	ID=MALK_00801;prediction_source=maker_MRET:augustus_masked-contig003-processed-gene-0.7-mRNA-1
contig003	maker	CDS	26999	28750	.	+	0	ID=MALK_00801.t1.c1;Parent=MALK_00801.t1
contig003	maker	mRNA	26999	28750	.	+	.	ID=MALK_00801.t1;Parent=MALK_00801
contig003	maker	exon	26999	28750	.	+	.	ID=MALK_00801.t1.e1;Parent=MALK_00801.t1
contig003	maker	gene	28767	29354	.	-	.	ID=MALK_00802;prediction_source=maker_MRET:augustus_masked-contig003-processed-gene-0.26-mRNA-1
contig003	maker	CDS	28767	29354	.	-	0	ID=MALK_00802.t1.c1;Parent=MALK_00802.t1
contig003	maker	mRNA	28767	29354	.	-	.	ID=MALK_00802.t1;Parent=MALK_00802

contig003	maker	exon	28767	29354	.	-	.	ID=MALK_00802.t1.e1;Parent=MALK_00802.t1
contig003	maker	gene	29551	30234	.	+	.	ID=MALK_00803;prediction_source=maker_MRET:augustus_masked-contig003-processed-gene-0.8-mRNA-1
contig003	maker	CDS	29551	30234	.	+	0	ID=MALK_00803.t1.c1;Parent=MALK_00803.t1
contig003	maker	mRNA	29551	30234	.	+	.	ID=MALK_00803.t1;Parent=MALK_00803
contig003	maker	exon	29551	30234	.	+	.	ID=MALK_00803.t1.e1;Parent=MALK_00803.t1
contig003	AUGUSTUS	gene	30236	31690	1	-	.	ID=MALK_00804;prediction_source=braker_MRET:g2853.t1
contig003	AUGUSTUS	CDS	30236	31690	1	-	0	ID=MALK_00804.t1.c1;Parent=MALK_00804.t1
contig003	AUGUSTUS	mRNA	30236	31690	1	-	.	ID=MALK_00804.t1;Parent=MALK_00804
contig003	AUGUSTUS	exon	30236	31690	.	-	.	ID=MALK_00804.t1.e1;Parent=MALK_00804.t1
contig003	AUGUSTUS	gene	32660	34459	0.62	+	.	ID=MALK_00805;prediction_source=augustus:contig003.g3125.t1
contig003	AUGUSTUS	CDS	32660	34459	0.62	+	0	ID=MALK_00805.t1.c1;Parent=MALK_00805.t1
contig003	AUGUSTUS	mRNA	32660	34459	0.62	+	.	ID=MALK_00805.t1;Parent=MALK_00805
contig003	AUGUSTUS	exon	32660	34459	0.62	+	.	ID=MALK_00805.t1.e1;Parent=MALK_00805.t1
contig003	AUGUSTUS	gene	34456	35022	0.97	-	.	ID=MALK_00806;prediction_source=augustus:contig003.g3126.t1
contig003	AUGUSTUS	CDS	34456	35022	0.97	-	0	ID=MALK_00806.t1.c1;Parent=MALK_00806.t1
contig003	AUGUSTUS	mRNA	34456	35022	0.97	-	.	ID=MALK_00806.t1;Parent=MALK_00806
contig003	AUGUSTUS	exon	34456	35022	0.97	-	.	ID=MALK_00806.t1.e1;Parent=MALK_00806.t1
contig003	AUGUSTUS	gene	35060	36424	0.63	+	.	ID=MALK_00807;prediction_source=braker_MRET:g2856.t1
contig003	AUGUSTUS	CDS	35060	36424	0.63	+	0	ID=MALK_00807.t1.c1;Parent=MALK_00807.t1
contig003	AUGUSTUS	mRNA	35060	36424	0.63	+	.	ID=MALK_00807.t1;Parent=MALK_00807
contig003	AUGUSTUS	exon	35060	36424	.	+	.	ID=MALK_00807.t1.e1;Parent=MALK_00807.t1
contig003	AUGUSTUS	gene	36923	37624	0.37	+	.	ID=MALK_00808;prediction_source=braker_MRET:g2857.t1
contig003	AUGUSTUS	CDS	36923	37624	0.37	+	0	ID=MALK_00808.t1.c1;Parent=MALK_00808.t1
contig003	AUGUSTUS	mRNA	36923	37624	0.37	+	.	ID=MALK_00808.t1;Parent=MALK_00808
contig003	AUGUSTUS	exon	36923	37624	.	+	.	ID=MALK_00808.t1.e1;Parent=MALK_00808.t1
contig003	maker	gene	37953	39311	.	-	.	ID=MALK_00809;prediction_source=maker_MRET:augustus_masked-contig003-processed-gene-0.28-mRNA-1
contig003	maker	CDS	37953	39311	.	-	0	ID=MALK_00809.t1.c1;Parent=MALK_00809.t1
contig003	maker	mRNA	37953	39311	.	-	.	ID=MALK_00809.t1;Parent=MALK_00809
contig003	maker	exon	37953	39311	.	-	.	ID=MALK_00809.t1.e1;Parent=MALK_00809.t1
contig003	AUGUSTUS	gene	39448	40197	1	-	.	ID=MALK_00810;prediction_source=braker_MRET:g2859.t1
contig003	AUGUSTUS	CDS	39448	40197	1	-	0	ID=MALK_00810.t1.c1;Parent=MALK_00810.t1
contig003	AUGUSTUS	mRNA	39448	40197	1	-	.	ID=MALK_00810.t1;Parent=MALK_00810
contig003	AUGUSTUS	exon	39448	40197	.	-	.	ID=MALK_00810.t1.e1;Parent=MALK_00810.t1
contig003	AUGUSTUS	gene	40260	42002	0.99	+	.	ID=MALK_00811;prediction_source=augustus:contig003.g3129.t1
contig003	AUGUSTUS	CDS	40260	42002	0.99	+	0	ID=MALK_00811.t1.c1;Parent=MALK_00811.t1
contig003	AUGUSTUS	mRNA	40260	42002	0.99	+	.	ID=MALK_00811.t1;Parent=MALK_00811
contig003	AUGUSTUS	exon	40260	42002	0.99	+	.	ID=MALK_00811.t1.e1;Parent=MALK_00811.t1
contig003	AUGUSTUS	gene	42009	48461	1	-	.	ID=MALK_00812;prediction_source=augustus:contig003.g3130.t1
contig003	AUGUSTUS	CDS	42009	48461	1	-	0	ID=MALK_00812.t1.c1;Parent=MALK_00812.t1
contig003	AUGUSTUS	mRNA	42009	48461	1	-	.	ID=MALK_00812.t1;Parent=MALK_00812
contig003	AUGUSTUS	exon	42009	48461	1	-	.	ID=MALK_00812.t1.e1;Parent=MALK_00812.t1
contig003	AUGUSTUS	gene	48676	49691	0.77	-	.	ID=MALK_00813;prediction_source=braker_MRET:g2862.t1
contig003	AUGUSTUS	CDS	49617	49691	0.78	-	0	ID=MALK_00813.t1.c2;Parent=MALK_00813.t1

contig003	AUGUSTUS	CDS	48676	49569	0.78	-	0	ID=MALK_00813.t1.c1;Parent=MALK_00813.t1
contig003	AUGUSTUS	mRNA	48676	49691	0.77	-	.	ID=MALK_00813.t1;Parent=MALK_00813
contig003	AUGUSTUS	exon	49617	49691	.	-	.	ID=MALK_00813.t1.e2;Parent=MALK_00813.t1
contig003	AUGUSTUS	exon	48676	49569	.	-	.	ID=MALK_00813.t1.e1;Parent=MALK_00813.t1
contig003	AUGUSTUS	gene	49976	50773	0.71	+	.	ID=MALK_00814;prediction_source=braker_MRET:g2863.t1
contig003	AUGUSTUS	CDS	49976	50773	0.71	+	0	ID=MALK_00814.t1.c1;Parent=MALK_00814.t1
contig003	AUGUSTUS	mRNA	49976	50773	0.71	+	.	ID=MALK_00814.t1;Parent=MALK_00814
contig003	AUGUSTUS	exon	49976	50773	.	+	.	ID=MALK_00814.t1.e1;Parent=MALK_00814.t1
contig003	AUGUSTUS	gene	50811	52596	0.43	-	.	ID=MALK_00815;prediction_source=braker_MRET:g2864.t1
contig003	AUGUSTUS	CDS	50912	52596	0.7	-	0	ID=MALK_00815.t1.c2;Parent=MALK_00815.t1
contig003	AUGUSTUS	CDS	50811	50817	0.7	-	0	ID=MALK_00815.t1.c1;Parent=MALK_00815.t1
contig003	AUGUSTUS	mRNA	50811	52596	0.43	-	.	ID=MALK_00815.t1;Parent=MALK_00815
contig003	AUGUSTUS	exon	50912	52596	.	-	.	ID=MALK_00815.t1.e2;Parent=MALK_00815.t1
contig003	AUGUSTUS	exon	50811	50817	.	-	.	ID=MALK_00815.t1.e1;Parent=MALK_00815.t1
contig003	AUGUSTUS	gene	52640	53391	0.72	-	.	ID=MALK_00816;prediction_source=braker_MRET:g2865.t1
contig003	AUGUSTUS	CDS	53286	53391	0.92	-	0	ID=MALK_00816.t1.c3;Parent=MALK_00816.t1
contig003	AUGUSTUS	CDS	52952	53238	0.92	-	0	ID=MALK_00816.t1.c2;Parent=MALK_00816.t1
contig003	AUGUSTUS	CDS	52640	52912	0.92	-	0	ID=MALK_00816.t1.c1;Parent=MALK_00816.t1
contig003	AUGUSTUS	mRNA	52640	53391	0.72	-	.	ID=MALK_00816.t1;Parent=MALK_00816
contig003	AUGUSTUS	exon	53286	53391	.	-	.	ID=MALK_00816.t1.e3;Parent=MALK_00816.t1
contig003	AUGUSTUS	exon	52952	53238	.	-	.	ID=MALK_00816.t1.e2;Parent=MALK_00816.t1
contig003	AUGUSTUS	exon	52640	52912	.	-	.	ID=MALK_00816.t1.e1;Parent=MALK_00816.t1
contig003	maker	gene	53531	53866	.	+	.	ID=MALK_00817;prediction_source=maker_MRET:augustus_masked-contig003-processed-gene-0.12-mRNA-1
contig003	maker	CDS	53531	53866	.	+	0	ID=MALK_00817.t1.c1;Parent=MALK_00817.t1
contig003	maker	mRNA	53531	53866	.	+	.	ID=MALK_00817.t1;Parent=MALK_00817
contig003	maker	exon	53531	53866	.	+	.	ID=MALK_00817.t1.e1;Parent=MALK_00817.t1
contig003	maker	gene	53945	55441	.	-	.	ID=MALK_00818;prediction_source=maker_MRET:augustus_masked-contig003-processed-gene-0.32-mRNA-1
contig003	maker	CDS	53945	55441	.	-	0	ID=MALK_00818.t1.c1;Parent=MALK_00818.t1
contig003	maker	mRNA	53945	55441	.	-	.	ID=MALK_00818.t1;Parent=MALK_00818
contig003	maker	exon	53945	55441	.	-	.	ID=MALK_00818.t1.e1;Parent=MALK_00818.t1
contig003	AUGUSTUS	gene	55761	57164	0.93	-	.	ID=MALK_00819;prediction_source=braker_MRET:g2868.t1
contig003	AUGUSTUS	CDS	55761	57164	0.93	-	0	ID=MALK_00819.t1.c1;Parent=MALK_00819.t1
contig003	AUGUSTUS	mRNA	55761	57164	0.93	-	.	ID=MALK_00819.t1;Parent=MALK_00819
contig003	AUGUSTUS	exon	55761	57164	.	-	.	ID=MALK_00819.t1.e1;Parent=MALK_00819.t1
contig003	AUGUSTUS	gene	57192	58047	0.84	-	.	ID=MALK_00820;prediction_source=braker_MRET:g2869.t1
contig003	AUGUSTUS	CDS	57742	58047	0.84	-	0	ID=MALK_00820.t1.c2;Parent=MALK_00820.t1
contig003	AUGUSTUS	CDS	57192	57713	0.84	-	0	ID=MALK_00820.t1.c1;Parent=MALK_00820.t1
contig003	AUGUSTUS	mRNA	57192	58047	0.84	-	.	ID=MALK_00820.t1;Parent=MALK_00820
contig003	AUGUSTUS	exon	57742	58047	.	-	.	ID=MALK_00820.t1.e2;Parent=MALK_00820.t1
contig003	AUGUSTUS	exon	57192	57713	.	-	.	ID=MALK_00820.t1.e1;Parent=MALK_00820.t1
contig003	AUGUSTUS	gene	58099	59130	0.84	-	.	ID=MALK_00821;prediction_source=braker_MRET:g2870.t1
contig003	AUGUSTUS	CDS	58099	59130	0.84	-	0	ID=MALK_00821.t1.c1;Parent=MALK_00821.t1
contig003	AUGUSTUS	mRNA	58099	59130	0.84	-	.	ID=MALK_00821.t1;Parent=MALK_00821

contig003	AUGUSTUS	exon	58099	59130	.	-	.	ID=MALK_00821.t1.e1;Parent=MALK_00821.t1
contig003	AUGUSTUS	gene	59215	59895	0.93	+	.	ID=MALK_00822;prediction_source=braker_MRET:g2871.t1
contig003	AUGUSTUS	CDS	59215	59895	0.93	+	0	ID=MALK_00822.t1.c1;Parent=MALK_00822.t1
contig003	AUGUSTUS	mRNA	59215	59895	0.93	+	.	ID=MALK_00822.t1;Parent=MALK_00822
contig003	AUGUSTUS	exon	59215	59895	.	+	.	ID=MALK_00822.t1.e1;Parent=MALK_00822.t1
contig003	maker	gene	59890	61497	.	-	.	ID=MALK_00823;prediction_source=maker_MRET:augustus_masked-contig003-processed-gene-0.33-mRNA-1
contig003	maker	CDS	59890	61497	.	-	0	ID=MALK_00823.t1.c1;Parent=MALK_00823.t1
contig003	maker	mRNA	59890	61497	.	-	.	ID=MALK_00823.t1;Parent=MALK_00823
contig003	maker	exon	59890	61497	.	-	.	ID=MALK_00823.t1.e1;Parent=MALK_00823.t1
contig003	maker	gene	62170	63801	.	+	.	ID=MALK_00824;prediction_source=maker_MRET:augustus_masked-contig003-processed-gene-0.13-mRNA-1
contig003	maker	CDS	62170	63801	.	+	0	ID=MALK_00824.t1.c1;Parent=MALK_00824.t1
contig003	maker	mRNA	62170	63801	.	+	.	ID=MALK_00824.t1;Parent=MALK_00824
contig003	maker	exon	62170	63801	.	+	.	ID=MALK_00824.t1.e1;Parent=MALK_00824.t1
contig003	AUGUSTUS	gene	63881	64836	0.93	-	.	ID=MALK_00825;prediction_source=braker_MRET:g2874.t1
contig003	AUGUSTUS	CDS	63986	64836	0.97	-	0	ID=MALK_00825.t1.c2;Parent=MALK_00825.t1
contig003	AUGUSTUS	CDS	63881	63896	0.97	-	0	ID=MALK_00825.t1.c1;Parent=MALK_00825.t1
contig003	AUGUSTUS	mRNA	63881	64836	0.93	-	.	ID=MALK_00825.t1;Parent=MALK_00825
contig003	AUGUSTUS	exon	63986	64836	.	-	.	ID=MALK_00825.t1.e2;Parent=MALK_00825.t1
contig003	AUGUSTUS	exon	63881	63896	.	-	.	ID=MALK_00825.t1.e1;Parent=MALK_00825.t1
contig003	AUGUSTUS	gene	64934	65773	1	+	.	ID=MALK_00826;prediction_source=braker_MRET:g2875.t1
contig003	AUGUSTUS	CDS	64934	65773	1	+	0	ID=MALK_00826.t1.c1;Parent=MALK_00826.t1
contig003	AUGUSTUS	mRNA	64934	65773	1	+	.	ID=MALK_00826.t1;Parent=MALK_00826
contig003	AUGUSTUS	exon	64934	65773	.	+	.	ID=MALK_00826.t1.e1;Parent=MALK_00826.t1
contig003	AUGUSTUS	gene	65831	66466	0.18	-	.	ID=MALK_00827;prediction_source=braker_MRET:g2876.t1
contig003	AUGUSTUS	CDS	66389	66466	0.48	-	0	ID=MALK_00827.t1.c5;Parent=MALK_00827.t1
contig003	AUGUSTUS	CDS	66326	66349	0.48	-	0	ID=MALK_00827.t1.c4;Parent=MALK_00827.t1
contig003	AUGUSTUS	CDS	66115	66298	0.48	-	0	ID=MALK_00827.t1.c3;Parent=MALK_00827.t1
contig003	AUGUSTUS	CDS	65903	66025	0.48	-	0	ID=MALK_00827.t1.c2;Parent=MALK_00827.t1
contig003	AUGUSTUS	CDS	65831	65859	0.48	-	0	ID=MALK_00827.t1.c1;Parent=MALK_00827.t1
contig003	AUGUSTUS	mRNA	65831	66466	0.18	-	.	ID=MALK_00827.t1;Parent=MALK_00827
contig003	AUGUSTUS	exon	66389	66466	.	-	.	ID=MALK_00827.t1.e5;Parent=MALK_00827.t1
contig003	AUGUSTUS	exon	66326	66349	.	-	.	ID=MALK_00827.t1.e4;Parent=MALK_00827.t1
contig003	AUGUSTUS	exon	66115	66298	.	-	.	ID=MALK_00827.t1.e3;Parent=MALK_00827.t1
contig003	AUGUSTUS	exon	65903	66025	.	-	.	ID=MALK_00827.t1.e2;Parent=MALK_00827.t1
contig003	AUGUSTUS	exon	65831	65859	.	-	.	ID=MALK_00827.t1.e1;Parent=MALK_00827.t1
contig003	AUGUSTUS	gene	66690	69413	0.97	+	.	ID=MALK_00828;prediction_source=braker_MRET:g2877.t1
contig003	AUGUSTUS	CDS	66690	69413	0.97	+	0	ID=MALK_00828.t1.c1;Parent=MALK_00828.t1
contig003	AUGUSTUS	mRNA	66690	69413	0.97	+	.	ID=MALK_00828.t1;Parent=MALK_00828
contig003	AUGUSTUS	exon	66690	69413	.	+	.	ID=MALK_00828.t1.e1;Parent=MALK_00828.t1
contig003	AUGUSTUS	gene	69451	70788	0.98	+	.	ID=MALK_00829;prediction_source=braker_MRET:g2878.t1
contig003	AUGUSTUS	CDS	69451	70788	0.98	+	0	ID=MALK_00829.t1.c1;Parent=MALK_00829.t1
contig003	AUGUSTUS	mRNA	69451	70788	0.98	+	.	ID=MALK_00829.t1;Parent=MALK_00829
contig003	AUGUSTUS	exon	69451	70788	.	+	.	ID=MALK_00829.t1.e1;Parent=MALK_00829.t1

contig003	AUGUSTUS	gene	70820	72250	0.84	+	.	ID=MALK_00830;prediction_source=augustus:contig003.g3141.t1
contig003	AUGUSTUS	CDS	70820	72250	0.84	+	0	ID=MALK_00830.t1.c1;Parent=MALK_00830.t1
contig003	AUGUSTUS	mRNA	70820	72250	0.84	+	.	ID=MALK_00830.t1;Parent=MALK_00830
contig003	AUGUSTUS	exon	70820	72250	0.84	+	.	ID=MALK_00830.t1.e1;Parent=MALK_00830.t1
contig003	AUGUSTUS	gene	72230	72841	0.73	-	.	ID=MALK_00831;prediction_source=augustus:contig003.g3142.t1
contig003	AUGUSTUS	CDS	72230	72841	0.73	-	0	ID=MALK_00831.t1.c1;Parent=MALK_00831.t1
contig003	AUGUSTUS	mRNA	72230	72841	0.73	-	.	ID=MALK_00831.t1;Parent=MALK_00831
contig003	AUGUSTUS	exon	72230	72841	0.73	-	.	ID=MALK_00831.t1.e1;Parent=MALK_00831.t1
contig003	maker	gene	72962	73921	.	+	.	ID=MALK_00832;prediction_source=maker_MRET:augustus_masked-contig003-processed-gene-0.16-mRNA-1
contig003	maker	CDS	72962	73921	.	+	0	ID=MALK_00832.t1.c1;Parent=MALK_00832.t1
contig003	maker	mRNA	72962	73921	.	+	.	ID=MALK_00832.t1;Parent=MALK_00832
contig003	maker	exon	72962	73921	.	+	.	ID=MALK_00832.t1.e1;Parent=MALK_00832.t1
contig003	maker	gene	74183	75040	.	-	.	ID=MALK_00833;prediction_source=maker_MRET:augustus_masked-contig003-processed-gene-0.36-mRNA-1
contig003	maker	CDS	74183	75040	.	-	0	ID=MALK_00833.t1.c1;Parent=MALK_00833.t1
contig003	maker	mRNA	74183	75040	.	-	.	ID=MALK_00833.t1;Parent=MALK_00833
contig003	maker	exon	74183	75040	.	-	.	ID=MALK_00833.t1.e1;Parent=MALK_00833.t1
contig003	AUGUSTUS	gene	75133	76481	0.51	-	.	ID=MALK_00834;prediction_source=braker_MRET:g2883.t1
contig003	AUGUSTUS	CDS	76365	76481	0.51	-	0	ID=MALK_00834.t1.c2;Parent=MALK_00834.t1
contig003	AUGUSTUS	CDS	75133	76326	0.51	-	0	ID=MALK_00834.t1.c1;Parent=MALK_00834.t1
contig003	AUGUSTUS	mRNA	75133	76481	0.51	-	.	ID=MALK_00834.t1;Parent=MALK_00834
contig003	AUGUSTUS	exon	76365	76481	.	-	.	ID=MALK_00834.t1.e2;Parent=MALK_00834.t1
contig003	AUGUSTUS	exon	75133	76326	.	-	.	ID=MALK_00834.t1.e1;Parent=MALK_00834.t1
contig003	AUGUSTUS	gene	76730	77822	0.21	-	.	ID=MALK_00835;prediction_source=braker_MRET:g2884.t1
contig003	AUGUSTUS	CDS	77776	77822	0.41	-	0	ID=MALK_00835.t1.c3;Parent=MALK_00835.t1
contig003	AUGUSTUS	CDS	77412	77671	0.41	-	0	ID=MALK_00835.t1.c2;Parent=MALK_00835.t1
contig003	AUGUSTUS	CDS	76730	77379	0.41	-	0	ID=MALK_00835.t1.c1;Parent=MALK_00835.t1
contig003	AUGUSTUS	mRNA	76730	77822	0.21	-	.	ID=MALK_00835.t1;Parent=MALK_00835
contig003	AUGUSTUS	exon	77776	77822	.	-	.	ID=MALK_00835.t1.e3;Parent=MALK_00835.t1
contig003	AUGUSTUS	exon	77412	77671	.	-	.	ID=MALK_00835.t1.e2;Parent=MALK_00835.t1
contig003	AUGUSTUS	exon	76730	77379	.	-	.	ID=MALK_00835.t1.e1;Parent=MALK_00835.t1
contig003	AUGUSTUS	gene	77977	79438	0.62	+	.	ID=MALK_00836;prediction_source=braker_MRET:g2885.t1
contig003	AUGUSTUS	CDS	77977	78012	0.88	+	0	ID=MALK_00836.t1.c1;Parent=MALK_00836.t1
contig003	AUGUSTUS	CDS	78041	79438	0.88	+	0	ID=MALK_00836.t1.c2;Parent=MALK_00836.t1
contig003	AUGUSTUS	mRNA	77977	79438	0.62	+	.	ID=MALK_00836.t1;Parent=MALK_00836
contig003	AUGUSTUS	exon	77977	78012	.	+	.	ID=MALK_00836.t1.e1;Parent=MALK_00836.t1
contig003	AUGUSTUS	exon	78041	79438	.	+	.	ID=MALK_00836.t1.e2;Parent=MALK_00836.t1
contig003	AUGUSTUS	gene	79577	82669	0.87	+	.	ID=MALK_00837;prediction_source=augustus:contig003.g3148.t1
contig003	AUGUSTUS	CDS	79577	82669	0.87	+	0	ID=MALK_00837.t1.c1;Parent=MALK_00837.t1
contig003	AUGUSTUS	mRNA	79577	82669	0.87	+	.	ID=MALK_00837.t1;Parent=MALK_00837
contig003	AUGUSTUS	exon	79577	82669	0.87	+	.	ID=MALK_00837.t1.e1;Parent=MALK_00837.t1
contig003	maker	gene	82802	84007	.	-	.	ID=MALK_00838;prediction_source=maker_MRET:augustus_masked-contig003-processed-gene-0.39-mRNA-1
contig003	maker	CDS	82802	84007	.	-	0	ID=MALK_00838.t1.c1;Parent=MALK_00838.t1
contig003	maker	mRNA	82802	84007	.	-	.	ID=MALK_00838.t1;Parent=MALK_00838

contig003	maker	exon	82802	84007	.	-	.	ID=MALK_00838.t1.e1;Parent=MALK_00838.t1
contig003	AUGUSTUS	gene	84076	86922	0.94	+	.	ID=MALK_00839;prediction_source=braker_MRET:g2888.t1
contig003	AUGUSTUS	CDS	84076	86922	0.94	+	0	ID=MALK_00839.t1.c1;Parent=MALK_00839.t1
contig003	AUGUSTUS	mRNA	84076	86922	0.94	+	.	ID=MALK_00839.t1;Parent=MALK_00839
contig003	AUGUSTUS	exon	84076	86922	.	+	.	ID=MALK_00839.t1.e1;Parent=MALK_00839.t1
contig003	AUGUSTUS	gene	86953	88063	0.85	+	.	ID=MALK_00840;prediction_source=braker_MRET:g2889.t1
contig003	AUGUSTUS	CDS	86953	87931	0.98	+	0	ID=MALK_00840.t1.c1;Parent=MALK_00840.t1
contig003	AUGUSTUS	CDS	87963	88063	0.98	+	0	ID=MALK_00840.t1.c2;Parent=MALK_00840.t1
contig003	AUGUSTUS	mRNA	86953	88063	0.85	+	.	ID=MALK_00840.t1;Parent=MALK_00840
contig003	AUGUSTUS	exon	86953	87931	.	+	.	ID=MALK_00840.t1.e1;Parent=MALK_00840.t1
contig003	AUGUSTUS	exon	87963	88063	.	+	.	ID=MALK_00840.t1.e2;Parent=MALK_00840.t1
contig003	AUGUSTUS	gene	88069	91863	0.41	-	.	ID=MALK_00841;prediction_source=braker_MRET:g2890.t1
contig003	AUGUSTUS	CDS	88069	91863	0.41	-	0	ID=MALK_00841.t1.c1;Parent=MALK_00841.t1
contig003	AUGUSTUS	mRNA	88069	91863	0.41	-	.	ID=MALK_00841.t1;Parent=MALK_00841
contig003	AUGUSTUS	exon	88069	91863	.	-	.	ID=MALK_00841.t1.e1;Parent=MALK_00841.t1
contig003	AUGUSTUS	gene	91997	94525	0.75	-	.	ID=MALK_00842;prediction_source=braker_MRET:g2891.t1
contig003	AUGUSTUS	CDS	91997	94525	0.75	-	0	ID=MALK_00842.t1.c1;Parent=MALK_00842.t1
contig003	AUGUSTUS	mRNA	91997	94525	0.75	-	.	ID=MALK_00842.t1;Parent=MALK_00842
contig003	AUGUSTUS	exon	91997	94525	.	-	.	ID=MALK_00842.t1.e1;Parent=MALK_00842.t1
contig003	AUGUSTUS	gene	94627	95661	1	+	.	ID=MALK_00843;prediction_source=braker_MRET:g2892.t1
contig003	AUGUSTUS	CDS	94627	95661	1	+	0	ID=MALK_00843.t1.c1;Parent=MALK_00843.t1
contig003	AUGUSTUS	mRNA	94627	95661	1	+	.	ID=MALK_00843.t1;Parent=MALK_00843
contig003	AUGUSTUS	exon	94627	95661	.	+	.	ID=MALK_00843.t1.e1;Parent=MALK_00843.t1
contig003	AUGUSTUS	gene	96148	97742	0.48	+	.	ID=MALK_00844;prediction_source=braker_MRET:g2893.t1
contig003	AUGUSTUS	CDS	96148	97449	0.5	+	0	ID=MALK_00844.t1.c1;Parent=MALK_00844.t1
contig003	AUGUSTUS	CDS	97485	97742	0.5	+	0	ID=MALK_00844.t1.c2;Parent=MALK_00844.t1
contig003	AUGUSTUS	mRNA	96148	97742	0.48	+	.	ID=MALK_00844.t1;Parent=MALK_00844
contig003	AUGUSTUS	exon	96148	97449	.	+	.	ID=MALK_00844.t1.e1;Parent=MALK_00844.t1
contig003	AUGUSTUS	exon	97485	97742	.	+	.	ID=MALK_00844.t1.e2;Parent=MALK_00844.t1
contig003	AUGUSTUS	gene	97872	98330	0.46	+	.	ID=MALK_00845;prediction_source=braker_MRET:g2894.t1
contig003	AUGUSTUS	CDS	97872	98330	0.46	+	0	ID=MALK_00845.t1.c1;Parent=MALK_00845.t1
contig003	AUGUSTUS	mRNA	97872	98330	0.46	+	.	ID=MALK_00845.t1;Parent=MALK_00845
contig003	AUGUSTUS	exon	97872	98330	.	+	.	ID=MALK_00845.t1.e1;Parent=MALK_00845.t1
contig003	AUGUSTUS	gene	98496	99743	0.89	+	.	ID=MALK_00846;prediction_source=braker_MRET:g2895.t1
contig003	AUGUSTUS	CDS	98496	99743	0.89	+	0	ID=MALK_00846.t1.c1;Parent=MALK_00846.t1
contig003	AUGUSTUS	mRNA	98496	99743	0.89	+	.	ID=MALK_00846.t1;Parent=MALK_00846
contig003	AUGUSTUS	exon	98496	99743	.	+	.	ID=MALK_00846.t1.e1;Parent=MALK_00846.t1
contig003	AUGUSTUS	gene	99761	100465	0.98	-	.	ID=MALK_00847;prediction_source=braker_MRET:g2896.t1
contig003	AUGUSTUS	CDS	99761	100465	0.98	-	0	ID=MALK_00847.t1.c1;Parent=MALK_00847.t1
contig003	AUGUSTUS	mRNA	99761	100465	0.98	-	.	ID=MALK_00847.t1;Parent=MALK_00847
contig003	AUGUSTUS	exon	99761	100465	.	-	.	ID=MALK_00847.t1.e1;Parent=MALK_00847.t1
contig003	AUGUSTUS	gene	100686	102356	0.8	+	.	ID=MALK_00848;prediction_source=augustus:contig003.g3155.t1
contig003	AUGUSTUS	CDS	100686	102356	0.8	+	0	ID=MALK_00848.t1.c1;Parent=MALK_00848.t1

contig003	AUGUSTUS	mRNA	100686	102356	0.8	+	.	ID=MALK_00848.t1;Parent=MALK_00848
contig003	AUGUSTUS	exon	100686	102356	0.8	+	.	ID=MALK_00848.t1.e1;Parent=MALK_00848.t1
contig003	AUGUSTUS	gene	102407	104503	1	-	.	ID=MALK_00849;prediction_source=braker_MRET:g2898.t1
contig003	AUGUSTUS	CDS	102407	104503	1	-	0	ID=MALK_00849.t1.c1;Parent=MALK_00849.t1
contig003	AUGUSTUS	mRNA	102407	104503	1	-	.	ID=MALK_00849.t1;Parent=MALK_00849
contig003	AUGUSTUS	exon	102407	104503	.	-	.	ID=MALK_00849.t1.e1;Parent=MALK_00849.t1
contig003	AUGUSTUS	gene	104575	108275	0.25	+	.	ID=MALK_00850;prediction_source=braker_MRET:g2899.t1
contig003	AUGUSTUS	CDS	104575	105723	0.64	+	0	ID=MALK_00850.t1.c1;Parent=MALK_00850.t1
contig003	AUGUSTUS	CDS	105761	105817	0.64	+	0	ID=MALK_00850.t1.c2;Parent=MALK_00850.t1
contig003	AUGUSTUS	CDS	105847	106381	0.64	+	0	ID=MALK_00850.t1.c3;Parent=MALK_00850.t1
contig003	AUGUSTUS	CDS	106411	108275	0.64	+	0	ID=MALK_00850.t1.c4;Parent=MALK_00850.t1
contig003	AUGUSTUS	mRNA	104575	108275	0.25	+	.	ID=MALK_00850.t1;Parent=MALK_00850
contig003	AUGUSTUS	exon	104575	105723	.	+	.	ID=MALK_00850.t1.e1;Parent=MALK_00850.t1
contig003	AUGUSTUS	exon	105761	105817	.	+	.	ID=MALK_00850.t1.e2;Parent=MALK_00850.t1
contig003	AUGUSTUS	exon	105847	106381	.	+	.	ID=MALK_00850.t1.e3;Parent=MALK_00850.t1
contig003	AUGUSTUS	exon	106411	108275	.	+	.	ID=MALK_00850.t1.e4;Parent=MALK_00850.t1
contig003	AUGUSTUS	gene	108444	108845	0.57	-	.	ID=MALK_00851;prediction_source=braker_MRET:g2900.t1
contig003	AUGUSTUS	CDS	108444	108845	0.57	-	0	ID=MALK_00851.t1.c1;Parent=MALK_00851.t1
contig003	AUGUSTUS	mRNA	108444	108845	0.57	-	.	ID=MALK_00851.t1;Parent=MALK_00851
contig003	AUGUSTUS	exon	108444	108845	.	-	.	ID=MALK_00851.t1.e1;Parent=MALK_00851.t1
contig003	AUGUSTUS	gene	108877	110055	0.57	-	.	ID=MALK_00852;prediction_source=braker_MRET:g2901.t1
contig003	AUGUSTUS	CDS	108877	110055	0.57	-	0	ID=MALK_00852.t1.c1;Parent=MALK_00852.t1
contig003	AUGUSTUS	mRNA	108877	110055	0.57	-	.	ID=MALK_00852.t1;Parent=MALK_00852
contig003	AUGUSTUS	exon	108877	110055	.	-	.	ID=MALK_00852.t1.e1;Parent=MALK_00852.t1
contig003	AUGUSTUS	gene	110140	111013	0.76	+	.	ID=MALK_00853;prediction_source=braker_MRET:g2902.t1
contig003	AUGUSTUS	CDS	110140	110910	0.99	+	0	ID=MALK_00853.t1.c1;Parent=MALK_00853.t1
contig003	AUGUSTUS	CDS	110939	111013	0.99	+	0	ID=MALK_00853.t1.c2;Parent=MALK_00853.t1
contig003	AUGUSTUS	mRNA	110140	111013	0.76	+	.	ID=MALK_00853.t1;Parent=MALK_00853
contig003	AUGUSTUS	exon	110140	110910	.	+	.	ID=MALK_00853.t1.e1;Parent=MALK_00853.t1
contig003	AUGUSTUS	exon	110939	111013	.	+	.	ID=MALK_00853.t1.e2;Parent=MALK_00853.t1
contig003	AUGUSTUS	gene	111186	112517	0.99	-	.	ID=MALK_00854;prediction_source=braker_MRET:g2903.t1
contig003	AUGUSTUS	CDS	111186	112517	0.99	-	0	ID=MALK_00854.t1.c1;Parent=MALK_00854.t1
contig003	AUGUSTUS	mRNA	111186	112517	0.99	-	.	ID=MALK_00854.t1;Parent=MALK_00854
contig003	AUGUSTUS	exon	111186	112517	.	-	.	ID=MALK_00854.t1.e1;Parent=MALK_00854.t1
contig003	maker	gene	112589	113359	.	-	.	ID=MALK_00855;prediction_source=maker_MRET:augustus_masked-contig003-processed-gene-1.86-mRNA-1
contig003	maker	CDS	112589	113359	.	-	0	ID=MALK_00855.t1.c1;Parent=MALK_00855.t1
contig003	maker	mRNA	112589	113359	.	-	.	ID=MALK_00855.t1;Parent=MALK_00855
contig003	maker	exon	112589	113359	.	-	.	ID=MALK_00855.t1.e1;Parent=MALK_00855.t1
contig003	AUGUSTUS	gene	113826	116780	1	+	.	ID=MALK_00856;prediction_source=augustus:contig003.g3162.t1
contig003	AUGUSTUS	CDS	113826	116780	1	+	0	ID=MALK_00856.t1.c1;Parent=MALK_00856.t1
contig003	AUGUSTUS	mRNA	113826	116780	1	+	.	ID=MALK_00856.t1;Parent=MALK_00856
contig003	AUGUSTUS	exon	113826	116780	1	+	.	ID=MALK_00856.t1.e1;Parent=MALK_00856.t1
contig003	maker	gene	116846	118243	.	+	.	ID=MALK_00857;prediction_source=maker_MRET:augustus_masked-contig003-processed-gene-1.64-mRNA-1

contig003	maker	CDS	116846	118243	.	+	0	ID=MALK_00857.t1.c1;Parent=MALK_00857.t1
contig003	maker	mRNA	116846	118243	.	+	.	ID=MALK_00857.t1;Parent=MALK_00857
contig003	maker	exon	116846	118243	.	+	.	ID=MALK_00857.t1.e1;Parent=MALK_00857.t1
contig003	AUGUSTUS	gene	118312	119148	0.51	-	.	ID=MALK_00858;prediction_source=braker_MRET:g2907.t1
contig003	AUGUSTUS	CDS	118312	119148	0.51	-	0	ID=MALK_00858.t1.c1;Parent=MALK_00858.t1
contig003	AUGUSTUS	mRNA	118312	119148	0.51	-	.	ID=MALK_00858.t1;Parent=MALK_00858
contig003	AUGUSTUS	exon	118312	119148	.	-	.	ID=MALK_00858.t1.e1;Parent=MALK_00858.t1
contig003	AUGUSTUS	gene	119278	120315	0.81	-	.	ID=MALK_00859;prediction_source=augustus:contig003.g3165.t1
contig003	AUGUSTUS	CDS	119278	120315	0.81	-	0	ID=MALK_00859.t1.c1;Parent=MALK_00859.t1
contig003	AUGUSTUS	mRNA	119278	120315	0.81	-	.	ID=MALK_00859.t1;Parent=MALK_00859
contig003	AUGUSTUS	exon	119278	120315	0.81	-	.	ID=MALK_00859.t1.e1;Parent=MALK_00859.t1
contig003	maker	gene	120466	120876	.	-	.	ID=MALK_00860;prediction_source=maker_MRET:augustus_masked-contig003-processed-gene-1.88-mRNA-1
contig003	maker	CDS	120466	120876	.	-	0	ID=MALK_00860.t1.c1;Parent=MALK_00860.t1
contig003	maker	mRNA	120466	120876	.	-	.	ID=MALK_00860.t1;Parent=MALK_00860
contig003	maker	exon	120466	120876	.	-	.	ID=MALK_00860.t1.e1;Parent=MALK_00860.t1
contig003	AUGUSTUS	gene	121303	121614	0.99	+	.	ID=MALK_00861;prediction_source=augustus:contig003.g3168.t1
contig003	AUGUSTUS	CDS	121303	121614	0.99	+	0	ID=MALK_00861.t1.c1;Parent=MALK_00861.t1
contig003	AUGUSTUS	mRNA	121303	121614	0.99	+	.	ID=MALK_00861.t1;Parent=MALK_00861
contig003	AUGUSTUS	exon	121303	121614	0.99	+	.	ID=MALK_00861.t1.e1;Parent=MALK_00861.t1
contig003	AUGUSTUS	gene	121922	123130	1	+	.	ID=MALK_00862;prediction_source=augustus:contig003.g3169.t1
contig003	AUGUSTUS	CDS	121922	123130	1	+	0	ID=MALK_00862.t1.c1;Parent=MALK_00862.t1
contig003	AUGUSTUS	mRNA	121922	123130	1	+	.	ID=MALK_00862.t1;Parent=MALK_00862
contig003	AUGUSTUS	exon	121922	123130	1	+	.	ID=MALK_00862.t1.e1;Parent=MALK_00862.t1
contig003	maker	gene	123593	124600	.	+	.	ID=MALK_00863;prediction_source=maker_MRET:augustus_masked-contig003-processed-gene-1.67-mRNA-1
contig003	maker	CDS	123593	124600	.	+	0	ID=MALK_00863.t1.c1;Parent=MALK_00863.t1
contig003	maker	mRNA	123593	124600	.	+	.	ID=MALK_00863.t1;Parent=MALK_00863
contig003	maker	exon	123593	124600	.	+	.	ID=MALK_00863.t1.e1;Parent=MALK_00863.t1
contig003	maker	gene	125237	126733	.	+	.	ID=MALK_00864;prediction_source=maker_MRET:augustus_masked-contig003-processed-gene-1.68-mRNA-1
contig003	maker	CDS	125237	126733	.	+	0	ID=MALK_00864.t1.c1;Parent=MALK_00864.t1
contig003	maker	mRNA	125237	126733	.	+	.	ID=MALK_00864.t1;Parent=MALK_00864
contig003	maker	exon	125237	126733	.	+	.	ID=MALK_00864.t1.e1;Parent=MALK_00864.t1
contig003	maker	gene	127280	128920	.	+	.	ID=MALK_00865;prediction_source=maker_MRET:augustus_masked-contig003-processed-gene-1.69-mRNA-1
contig003	maker	CDS	127280	128920	.	+	0	ID=MALK_00865.t1.c1;Parent=MALK_00865.t1
contig003	maker	mRNA	127280	128920	.	+	.	ID=MALK_00865.t1;Parent=MALK_00865
contig003	maker	exon	127280	128920	.	+	.	ID=MALK_00865.t1.e1;Parent=MALK_00865.t1
contig003	maker	gene	129053	129418	.	-	.	ID=MALK_00866;prediction_source=maker_MRET:augustus_masked-contig003-processed-gene-1.89-mRNA-1
contig003	maker	CDS	129053	129418	.	-	0	ID=MALK_00866.t1.c1;Parent=MALK_00866.t1
contig003	maker	mRNA	129053	129418	.	-	.	ID=MALK_00866.t1;Parent=MALK_00866
contig003	maker	exon	129053	129418	.	-	.	ID=MALK_00866.t1.e1;Parent=MALK_00866.t1
contig003	maker	gene	129555	130421	.	+	.	ID=MALK_00867;prediction_source=maker_MRET:augustus_masked-contig003-processed-gene-1.70-mRNA-1
contig003	maker	CDS	129555	130421	.	+	0	ID=MALK_00867.t1.c1;Parent=MALK_00867.t1
contig003	maker	mRNA	129555	130421	.	+	.	ID=MALK_00867.t1;Parent=MALK_00867
contig003	maker	exon	129555	130421	.	+	.	ID=MALK_00867.t1.e1;Parent=MALK_00867.t1

contig003	AUGUSTUS	gene	130424	131905	0.83	-	.	ID=MALK_00868;prediction_source=augustus:contig003.g3177.t1
contig003	AUGUSTUS	CDS	130424	131905	0.83	-	0	ID=MALK_00868.t1.c1;Parent=MALK_00868.t1
contig003	AUGUSTUS	mRNA	130424	131905	0.83	-	.	ID=MALK_00868.t1;Parent=MALK_00868
contig003	AUGUSTUS	exon	130424	131905	0.83	-	.	ID=MALK_00868.t1.e1;Parent=MALK_00868.t1
contig003	AUGUSTUS	gene	131969	133018	0.88	-	.	ID=MALK_00869;prediction_source=augustus:contig003.g3179.t1
contig003	AUGUSTUS	CDS	131969	133018	0.88	-	0	ID=MALK_00869.t1.c1;Parent=MALK_00869.t1
contig003	AUGUSTUS	mRNA	131969	133018	0.88	-	.	ID=MALK_00869.t1;Parent=MALK_00869
contig003	AUGUSTUS	exon	131969	133018	0.88	-	.	ID=MALK_00869.t1.e1;Parent=MALK_00869.t1
contig003	AUGUSTUS	gene	133441	134526	0.91	-	.	ID=MALK_00870;prediction_source=augustus:contig003.g3180.t1
contig003	AUGUSTUS	CDS	133441	134526	0.91	-	0	ID=MALK_00870.t1.c1;Parent=MALK_00870.t1
contig003	AUGUSTUS	mRNA	133441	134526	0.91	-	.	ID=MALK_00870.t1;Parent=MALK_00870
contig003	AUGUSTUS	exon	133441	134526	0.91	-	.	ID=MALK_00870.t1.e1;Parent=MALK_00870.t1
contig003	AUGUSTUS	gene	134846	136585	0.88	+	.	ID=MALK_00871;prediction_source=braker_MRET:g2919.t1
contig003	AUGUSTUS	CDS	134846	136585	0.88	+	0	ID=MALK_00871.t1.c1;Parent=MALK_00871.t1
contig003	AUGUSTUS	mRNA	134846	136585	0.88	+	.	ID=MALK_00871.t1;Parent=MALK_00871
contig003	AUGUSTUS	exon	134846	136585	.	+	.	ID=MALK_00871.t1.e1;Parent=MALK_00871.t1
contig003	AUGUSTUS	gene	136764	137796	0.53	+	.	ID=MALK_00872;prediction_source=braker_MRET:g2920.t1
contig003	AUGUSTUS	CDS	136764	136829	1	+	0	ID=MALK_00872.t1.c1;Parent=MALK_00872.t1
contig003	AUGUSTUS	CDS	136869	136937	1	+	0	ID=MALK_00872.t1.c2;Parent=MALK_00872.t1
contig003	AUGUSTUS	CDS	137143	137796	1	+	0	ID=MALK_00872.t1.c3;Parent=MALK_00872.t1
contig003	AUGUSTUS	mRNA	136764	137796	0.53	+	.	ID=MALK_00872.t1;Parent=MALK_00872
contig003	AUGUSTUS	exon	136764	136829	.	+	.	ID=MALK_00872.t1.e1;Parent=MALK_00872.t1
contig003	AUGUSTUS	exon	136869	136937	.	+	.	ID=MALK_00872.t1.e2;Parent=MALK_00872.t1
contig003	AUGUSTUS	exon	137143	137796	.	+	.	ID=MALK_00872.t1.e3;Parent=MALK_00872.t1
contig003	AUGUSTUS	gene	138049	139350	0.82	+	.	ID=MALK_00873;prediction_source=augustus:contig003.g3183.t1
contig003	AUGUSTUS	CDS	138049	139350	0.82	+	0	ID=MALK_00873.t1.c1;Parent=MALK_00873.t1
contig003	AUGUSTUS	mRNA	138049	139350	0.82	+	.	ID=MALK_00873.t1;Parent=MALK_00873
contig003	AUGUSTUS	exon	138049	139350	0.82	+	.	ID=MALK_00873.t1.e1;Parent=MALK_00873.t1
contig003	AUGUSTUS	gene	139519	141038	0.71	-	.	ID=MALK_00874;prediction_source=braker_MRET:g2922.t1
contig003	AUGUSTUS	CDS	139705	141038	0.9	-	0	ID=MALK_00874.t1.c2;Parent=MALK_00874.t1
contig003	AUGUSTUS	CDS	139519	139588	0.9	-	0	ID=MALK_00874.t1.c1;Parent=MALK_00874.t1
contig003	AUGUSTUS	mRNA	139519	141038	0.71	-	.	ID=MALK_00874.t1;Parent=MALK_00874
contig003	AUGUSTUS	exon	139705	141038	.	-	.	ID=MALK_00874.t1.e2;Parent=MALK_00874.t1
contig003	AUGUSTUS	exon	139519	139588	.	-	.	ID=MALK_00874.t1.e1;Parent=MALK_00874.t1
contig003	AUGUSTUS	gene	141103	141849	1	-	.	ID=MALK_00875;prediction_source=braker_MRET:g2923.t1
contig003	AUGUSTUS	CDS	141103	141849	1	-	0	ID=MALK_00875.t1.c1;Parent=MALK_00875.t1
contig003	AUGUSTUS	mRNA	141103	141849	1	-	.	ID=MALK_00875.t1;Parent=MALK_00875
contig003	AUGUSTUS	exon	141103	141849	.	-	.	ID=MALK_00875.t1.e1;Parent=MALK_00875.t1
contig003	AUGUSTUS	gene	141902	143056	0.99	-	.	ID=MALK_00876;prediction_source=braker_MRET:g2924.t1
contig003	AUGUSTUS	CDS	141902	143056	0.99	-	0	ID=MALK_00876.t1.c1;Parent=MALK_00876.t1
contig003	AUGUSTUS	mRNA	141902	143056	0.99	-	.	ID=MALK_00876.t1;Parent=MALK_00876
contig003	AUGUSTUS	exon	141902	143056	.	-	.	ID=MALK_00876.t1.e1;Parent=MALK_00876.t1
contig003	AUGUSTUS	gene	143117	144781	0.64	-	.	ID=MALK_00877;prediction_source=braker_MRET:g2925.t1

contig003	AUGUSTUS	CDS	143117	144781	0.64	-	0	ID=MALK_00877.t1.c1;Parent=MALK_00877.t1
contig003	AUGUSTUS	mRNA	143117	144781	0.64	-	.	ID=MALK_00877.t1;Parent=MALK_00877
contig003	AUGUSTUS	exon	143117	144781	.	-	.	ID=MALK_00877.t1.e1;Parent=MALK_00877.t1
contig003	AUGUSTUS	gene	144929	147124	0.94	+	.	ID=MALK_00878;prediction_source=braker_MRET:g2926.t1
contig003	AUGUSTUS	CDS	144929	147124	0.94	+	0	ID=MALK_00878.t1.c1;Parent=MALK_00878.t1
contig003	AUGUSTUS	mRNA	144929	147124	0.94	+	.	ID=MALK_00878.t1;Parent=MALK_00878
contig003	AUGUSTUS	exon	144929	147124	.	+	.	ID=MALK_00878.t1.e1;Parent=MALK_00878.t1
contig003	maker	gene	147293	148210	.	-	.	ID=MALK_00879;prediction_source=maker_MRET:augustus_masked-contig003-processed-gene-1.94-mRNA-1
contig003	maker	CDS	147293	148210	.	-	0	ID=MALK_00879.t1.c1;Parent=MALK_00879.t1
contig003	maker	mRNA	147293	148210	.	-	.	ID=MALK_00879.t1;Parent=MALK_00879
contig003	maker	exon	147293	148210	.	-	.	ID=MALK_00879.t1.e1;Parent=MALK_00879.t1
contig003	maker	gene	148320	149873	.	+	.	ID=MALK_00880;prediction_source=maker_MRET:augustus_masked-contig003-processed-gene-1.75-mRNA-1
contig003	maker	CDS	148320	149873	.	+	0	ID=MALK_00880.t1.c1;Parent=MALK_00880.t1
contig003	maker	mRNA	148320	149873	.	+	.	ID=MALK_00880.t1;Parent=MALK_00880
contig003	maker	exon	148320	149873	.	+	.	ID=MALK_00880.t1.e1;Parent=MALK_00880.t1
contig003	AUGUSTUS	gene	149882	150931	1	-	.	ID=MALK_00881;prediction_source=braker_MRET:g2929.t1
contig003	AUGUSTUS	CDS	149882	150931	1	-	0	ID=MALK_00881.t1.c1;Parent=MALK_00881.t1
contig003	AUGUSTUS	mRNA	149882	150931	1	-	.	ID=MALK_00881.t1;Parent=MALK_00881
contig003	AUGUSTUS	exon	149882	150931	.	-	.	ID=MALK_00881.t1.e1;Parent=MALK_00881.t1
contig003	maker	gene	150994	151863	.	+	.	ID=MALK_00882;prediction_source=maker_MRET:augustus_masked-contig003-processed-gene-1.76-mRNA-1
contig003	maker	CDS	150994	151863	.	+	0	ID=MALK_00882.t1.c1;Parent=MALK_00882.t1
contig003	maker	mRNA	150994	151863	.	+	.	ID=MALK_00882.t1;Parent=MALK_00882
contig003	maker	exon	150994	151863	.	+	.	ID=MALK_00882.t1.e1;Parent=MALK_00882.t1
contig003	AUGUSTUS	gene	151974	152435	0.48	+	.	ID=MALK_00883;prediction_source=braker_MRET:g2931.t1
contig003	AUGUSTUS	CDS	151974	152435	0.48	+	0	ID=MALK_00883.t1.c1;Parent=MALK_00883.t1
contig003	AUGUSTUS	mRNA	151974	152435	0.48	+	.	ID=MALK_00883.t1;Parent=MALK_00883
contig003	AUGUSTUS	exon	151974	152435	.	+	.	ID=MALK_00883.t1.e1;Parent=MALK_00883.t1
contig003	AUGUSTUS	gene	152599	156169	0.97	+	.	ID=MALK_00884;prediction_source=braker_MRET:g2932.t1
contig003	AUGUSTUS	CDS	152599	152667	1	+	0	ID=MALK_00884.t1.c1;Parent=MALK_00884.t1
contig003	AUGUSTUS	CDS	152704	152739	1	+	0	ID=MALK_00884.t1.c2;Parent=MALK_00884.t1
contig003	AUGUSTUS	CDS	152780	153766	1	+	0	ID=MALK_00884.t1.c3;Parent=MALK_00884.t1
contig003	AUGUSTUS	CDS	153866	156169	1	+	0	ID=MALK_00884.t1.c4;Parent=MALK_00884.t1
contig003	AUGUSTUS	mRNA	152599	156169	0.97	+	.	ID=MALK_00884.t1;Parent=MALK_00884
contig003	AUGUSTUS	exon	152599	152667	.	+	.	ID=MALK_00884.t1.e1;Parent=MALK_00884.t1
contig003	AUGUSTUS	exon	152704	152739	.	+	.	ID=MALK_00884.t1.e2;Parent=MALK_00884.t1
contig003	AUGUSTUS	exon	152780	153766	.	+	.	ID=MALK_00884.t1.e3;Parent=MALK_00884.t1
contig003	AUGUSTUS	exon	153866	156169	.	+	.	ID=MALK_00884.t1.e4;Parent=MALK_00884.t1
contig003	AUGUSTUS	gene	156220	157203	0.81	-	.	ID=MALK_00885;prediction_source=augustus:contig003.g3192.t1
contig003	AUGUSTUS	CDS	156220	157203	0.81	-	0	ID=MALK_00885.t1.c1;Parent=MALK_00885.t1
contig003	AUGUSTUS	mRNA	156220	157203	0.81	-	.	ID=MALK_00885.t1;Parent=MALK_00885
contig003	AUGUSTUS	exon	156220	157203	0.81	-	.	ID=MALK_00885.t1.e1;Parent=MALK_00885.t1
contig003	maker	gene	157534	158670	.	-	.	ID=MALK_00886;prediction_source=maker_MRET:augustus_masked-contig003-processed-gene-1.96-mRNA-1
contig003	maker	CDS	157534	158670	.	-	0	ID=MALK_00886.t1.c1;Parent=MALK_00886.t1

contig003	maker	mRNA	157534	158670	.	-	.	ID=MALK_00886.t1;Parent=MALK_00886
contig003	maker	exon	157534	158670	.	-	.	ID=MALK_00886.t1.e1;Parent=MALK_00886.t1
contig003	AUGUSTUS	gene	158800	159969	0.99	+	.	ID=MALK_00887;prediction_source=augustus:contig003.g3194.t1
contig003	AUGUSTUS	CDS	158800	159969	0.99	+	0	ID=MALK_00887.t1.c1;Parent=MALK_00887.t1
contig003	AUGUSTUS	mRNA	158800	159969	0.99	+	.	ID=MALK_00887.t1;Parent=MALK_00887
contig003	AUGUSTUS	exon	158800	159969	0.99	+	.	ID=MALK_00887.t1.e1;Parent=MALK_00887.t1
contig003	maker	gene	159973	162018	.	-	.	ID=MALK_00888;prediction_source=maker_MRET:augustus_masked-contig003-processed-gene-1.97-mRNA-1
contig003	maker	CDS	159973	162018	.	-	0	ID=MALK_00888.t1.c1;Parent=MALK_00888.t1
contig003	maker	mRNA	159973	162018	.	-	.	ID=MALK_00888.t1;Parent=MALK_00888
contig003	maker	exon	159973	162018	.	-	.	ID=MALK_00888.t1.e1;Parent=MALK_00888.t1
contig003	AUGUSTUS	gene	162130	163479	0.9	+	.	ID=MALK_00889;prediction_source=augustus:contig003.g3196.t1
contig003	AUGUSTUS	CDS	162130	163479	0.9	+	0	ID=MALK_00889.t1.c1;Parent=MALK_00889.t1
contig003	AUGUSTUS	mRNA	162130	163479	0.9	+	.	ID=MALK_00889.t1;Parent=MALK_00889
contig003	AUGUSTUS	exon	162130	163479	0.9	+	.	ID=MALK_00889.t1.e1;Parent=MALK_00889.t1
contig003	AUGUSTUS	gene	163476	164657	0.54	-	.	ID=MALK_00890;prediction_source=augustus:contig003.g3197.t1
contig003	AUGUSTUS	CDS	163476	164657	0.54	-	0	ID=MALK_00890.t1.c1;Parent=MALK_00890.t1
contig003	AUGUSTUS	mRNA	163476	164657	0.54	-	.	ID=MALK_00890.t1;Parent=MALK_00890
contig003	AUGUSTUS	exon	163476	164657	0.54	-	.	ID=MALK_00890.t1.e1;Parent=MALK_00890.t1
contig003	AUGUSTUS	gene	164611	168957	0.97	+	.	ID=MALK_00891;prediction_source=augustus:contig003.g3198.t1
contig003	AUGUSTUS	CDS	164611	168957	0.97	+	0	ID=MALK_00891.t1.c1;Parent=MALK_00891.t1
contig003	AUGUSTUS	mRNA	164611	168957	0.97	+	.	ID=MALK_00891.t1;Parent=MALK_00891
contig003	AUGUSTUS	exon	164611	168957	0.97	+	.	ID=MALK_00891.t1.e1;Parent=MALK_00891.t1
contig003	AUGUSTUS	gene	169114	172021	0.48	-	.	ID=MALK_00892;prediction_source=braker_MRET:g2940.t1
contig003	AUGUSTUS	CDS	169163	172021	0.48	-	0	ID=MALK_00892.t1.c2;Parent=MALK_00892.t1
contig003	AUGUSTUS	CDS	169114	169134	0.48	-	0	ID=MALK_00892.t1.c1;Parent=MALK_00892.t1
contig003	AUGUSTUS	mRNA	169114	172021	0.48	-	.	ID=MALK_00892.t1;Parent=MALK_00892
contig003	AUGUSTUS	exon	169163	172021	.	-	.	ID=MALK_00892.t1.e2;Parent=MALK_00892.t1
contig003	AUGUSTUS	exon	169114	169134	.	-	.	ID=MALK_00892.t1.e1;Parent=MALK_00892.t1
contig003	AUGUSTUS	gene	172135	186660	0.39	+	.	ID=MALK_00893;prediction_source=braker_MRET:g2941.t1
contig003	AUGUSTUS	CDS	172135	172179	0.67	+	0	ID=MALK_00893.t1.c1;Parent=MALK_00893.t1
contig003	AUGUSTUS	CDS	172219	186660	0.67	+	0	ID=MALK_00893.t1.c2;Parent=MALK_00893.t1
contig003	AUGUSTUS	mRNA	172135	186660	0.39	+	.	ID=MALK_00893.t1;Parent=MALK_00893
contig003	AUGUSTUS	exon	172135	172179	.	+	.	ID=MALK_00893.t1.e1;Parent=MALK_00893.t1
contig003	AUGUSTUS	exon	172219	186660	.	+	.	ID=MALK_00893.t1.e2;Parent=MALK_00893.t1
contig003	maker	gene	186676	189564	.	-	.	ID=MALK_00894;prediction_source=maker_MRET:augustus_masked-contig003-processed-gene-1.98-mRNA-1
contig003	maker	CDS	186676	189564	.	-	0	ID=MALK_00894.t1.c1;Parent=MALK_00894.t1
contig003	maker	mRNA	186676	189564	.	-	.	ID=MALK_00894.t1;Parent=MALK_00894
contig003	maker	exon	186676	189564	.	-	.	ID=MALK_00894.t1.e1;Parent=MALK_00894.t1
contig003	maker	gene	189713	191332	.	+	.	ID=MALK_00895;prediction_source=maker_MRET:augustus_masked-contig003-processed-gene-1.83-mRNA-1
contig003	maker	CDS	189713	191332	.	+	0	ID=MALK_00895.t1.c1;Parent=MALK_00895.t1
contig003	maker	mRNA	189713	191332	.	+	.	ID=MALK_00895.t1;Parent=MALK_00895
contig003	maker	exon	189713	191332	.	+	.	ID=MALK_00895.t1.e1;Parent=MALK_00895.t1
contig003	AUGUSTUS	gene	191360	194059	0.55	-	.	ID=MALK_00896;prediction_source=braker_MRET:g2944.t1

contig003	AUGUSTUS	CDS	191360	194059	0.55	-	0	ID=MALK_00896.t1.c1;Parent=MALK_00896.t1
contig003	AUGUSTUS	mRNA	191360	194059	0.55	-	.	ID=MALK_00896.t1;Parent=MALK_00896
contig003	AUGUSTUS	exon	191360	194059	.	-	.	ID=MALK_00896.t1.e1;Parent=MALK_00896.t1
contig003	maker	gene	194190	196289	.	+	.	ID=MALK_00897;prediction_source=maker_MRET:augustus_masked-contig003-processed-gene-2.51-mRNA-1
contig003	maker	CDS	194190	196289	.	+	0	ID=MALK_00897.t1.c1;Parent=MALK_00897.t1
contig003	maker	mRNA	194190	196289	.	+	.	ID=MALK_00897.t1;Parent=MALK_00897
contig003	maker	exon	194190	196289	.	+	.	ID=MALK_00897.t1.e1;Parent=MALK_00897.t1
contig003	maker	gene	196901	198214	.	+	.	ID=MALK_00898;prediction_source=maker_MRET:augustus_masked-contig003-processed-gene-2.52-mRNA-1
contig003	maker	CDS	196901	198214	.	+	0	ID=MALK_00898.t1.c1;Parent=MALK_00898.t1
contig003	maker	mRNA	196901	198214	.	+	.	ID=MALK_00898.t1;Parent=MALK_00898
contig003	maker	exon	196901	198214	.	+	.	ID=MALK_00898.t1.e1;Parent=MALK_00898.t1
contig003	AUGUSTUS	gene	198958	199314	0.63	+	.	ID=MALK_00899;prediction_source=augustus:contig003.g3208.t1
contig003	AUGUSTUS	CDS	198958	199314	0.63	+	0	ID=MALK_00899.t1.c1;Parent=MALK_00899.t1
contig003	AUGUSTUS	mRNA	198958	199314	0.63	+	.	ID=MALK_00899.t1;Parent=MALK_00899
contig003	AUGUSTUS	exon	198958	199314	0.63	+	.	ID=MALK_00899.t1.e1;Parent=MALK_00899.t1
contig003	AUGUSTUS	gene	199653	200468	0.63	+	.	ID=MALK_00900;prediction_source=augustus:contig003.g3210.t1
contig003	AUGUSTUS	CDS	199653	200468	0.63	+	0	ID=MALK_00900.t1.c1;Parent=MALK_00900.t1
contig003	AUGUSTUS	mRNA	199653	200468	0.63	+	.	ID=MALK_00900.t1;Parent=MALK_00900
contig003	AUGUSTUS	exon	199653	200468	0.63	+	.	ID=MALK_00900.t1.e1;Parent=MALK_00900.t1
contig003	maker	gene	200684	201706	.	+	.	ID=MALK_00901;prediction_source=maker_MRET:augustus_masked-contig003-processed-gene-2.55-mRNA-1
contig003	maker	CDS	200684	201706	.	+	0	ID=MALK_00901.t1.c1;Parent=MALK_00901.t1
contig003	maker	mRNA	200684	201706	.	+	.	ID=MALK_00901.t1;Parent=MALK_00901
contig003	maker	exon	200684	201706	.	+	.	ID=MALK_00901.t1.e1;Parent=MALK_00901.t1
contig003	AUGUSTUS	gene	201721	203298	0.99	-	.	ID=MALK_00902;prediction_source=augustus:contig003.g3212.t1
contig003	AUGUSTUS	CDS	201721	203298	0.99	-	0	ID=MALK_00902.t1.c1;Parent=MALK_00902.t1
contig003	AUGUSTUS	mRNA	201721	203298	0.99	-	.	ID=MALK_00902.t1;Parent=MALK_00902
contig003	AUGUSTUS	exon	201721	203298	0.99	-	.	ID=MALK_00902.t1.e1;Parent=MALK_00902.t1
contig003	AUGUSTUS	gene	203474	204026	0.65	+	.	ID=MALK_00903;prediction_source=braker_MRET:g2951.t1
contig003	AUGUSTUS	CDS	203474	203482	0.65	+	0	ID=MALK_00903.t1.c1;Parent=MALK_00903.t1
contig003	AUGUSTUS	CDS	203536	203691	0.65	+	0	ID=MALK_00903.t1.c2;Parent=MALK_00903.t1
contig003	AUGUSTUS	CDS	203769	203888	0.65	+	0	ID=MALK_00903.t1.c3;Parent=MALK_00903.t1
contig003	AUGUSTUS	CDS	203925	204026	0.65	+	0	ID=MALK_00903.t1.c4;Parent=MALK_00903.t1
contig003	AUGUSTUS	mRNA	203474	204026	0.65	+	.	ID=MALK_00903.t1;Parent=MALK_00903
contig003	AUGUSTUS	exon	203474	203482	.	+	.	ID=MALK_00903.t1.e1;Parent=MALK_00903.t1
contig003	AUGUSTUS	exon	203536	203691	.	+	.	ID=MALK_00903.t1.e2;Parent=MALK_00903.t1
contig003	AUGUSTUS	exon	203769	203888	.	+	.	ID=MALK_00903.t1.e3;Parent=MALK_00903.t1
contig003	AUGUSTUS	exon	203925	204026	.	+	.	ID=MALK_00903.t1.e4;Parent=MALK_00903.t1
contig003	maker	gene	204628	205593	.	+	.	ID=MALK_00904;prediction_source=maker_MRET:augustus_masked-contig003-processed-gene-2.56-mRNA-1
contig003	maker	CDS	204628	205593	.	+	0	ID=MALK_00904.t1.c1;Parent=MALK_00904.t1
contig003	maker	mRNA	204628	205593	.	+	.	ID=MALK_00904.t1;Parent=MALK_00904
contig003	maker	exon	204628	205593	.	+	.	ID=MALK_00904.t1.e1;Parent=MALK_00904.t1
contig003	AUGUSTUS	gene	206228	208921	0.98	-	.	ID=MALK_00905;prediction_source=braker_MRET:g2953.t1
contig003	AUGUSTUS	CDS	206228	208921	0.98	-	0	ID=MALK_00905.t1.c1;Parent=MALK_00905.t1

contig003	AUGUSTUS	mRNA	206228	208921	0.98	-	.	ID=MALK_00905.t1;Parent=MALK_00905
contig003	AUGUSTUS	exon	206228	208921	.	-	.	ID=MALK_00905.t1.e1;Parent=MALK_00905.t1
contig003	AUGUSTUS	gene	208978	209292	0.45	+	.	ID=MALK_00906;prediction_source=braker_MRET:g2954.t1
contig003	AUGUSTUS	CDS	208978	209292	0.45	+	0	ID=MALK_00906.t1.c1;Parent=MALK_00906.t1
contig003	AUGUSTUS	mRNA	208978	209292	0.45	+	.	ID=MALK_00906.t1;Parent=MALK_00906
contig003	AUGUSTUS	exon	208978	209292	.	+	.	ID=MALK_00906.t1.e1;Parent=MALK_00906.t1
contig003	maker	gene	209301	210140	.	-	.	ID=MALK_00907;prediction_source=maker_MRET:augustus_masked-contig003-processed-gene-2.81-mRNA-1
contig003	maker	CDS	209301	210140	.	-	0	ID=MALK_00907.t1.c1;Parent=MALK_00907.t1
contig003	maker	mRNA	209301	210140	.	-	.	ID=MALK_00907.t1;Parent=MALK_00907
contig003	maker	exon	209301	210140	.	-	.	ID=MALK_00907.t1.e1;Parent=MALK_00907.t1
contig003	AUGUSTUS	gene	210197	211060	0.62	+	.	ID=MALK_00908;prediction_source=augustus:contig003.g3216.t1
contig003	AUGUSTUS	CDS	210197	211060	0.62	+	0	ID=MALK_00908.t1.c1;Parent=MALK_00908.t1
contig003	AUGUSTUS	mRNA	210197	211060	0.62	+	.	ID=MALK_00908.t1;Parent=MALK_00908
contig003	AUGUSTUS	exon	210197	211060	0.62	+	.	ID=MALK_00908.t1.e1;Parent=MALK_00908.t1
contig003	AUGUSTUS	gene	211061	212926	0.77	-	.	ID=MALK_00909;prediction_source=augustus:contig003.g3217.t1
contig003	AUGUSTUS	CDS	211061	212926	0.77	-	0	ID=MALK_00909.t1.c1;Parent=MALK_00909.t1
contig003	AUGUSTUS	mRNA	211061	212926	0.77	-	.	ID=MALK_00909.t1;Parent=MALK_00909
contig003	AUGUSTUS	exon	211061	212926	0.77	-	.	ID=MALK_00909.t1.e1;Parent=MALK_00909.t1
contig003	maker	gene	213997	216471	.	+	.	ID=MALK_00910;prediction_source=maker_MRET:augustus_masked-contig003-processed-gene-2.57-mRNA-1
contig003	maker	CDS	213997	216471	.	+	0	ID=MALK_00910.t1.c1;Parent=MALK_00910.t1
contig003	maker	mRNA	213997	216471	.	+	.	ID=MALK_00910.t1;Parent=MALK_00910
contig003	maker	exon	213997	216471	.	+	.	ID=MALK_00910.t1.e1;Parent=MALK_00910.t1
contig003	maker	gene	216606	217247	.	+	.	ID=MALK_00911;prediction_source=maker_MRET:augustus_masked-contig003-processed-gene-2.58-mRNA-1
contig003	maker	CDS	216606	217247	.	+	0	ID=MALK_00911.t1.c1;Parent=MALK_00911.t1
contig003	maker	mRNA	216606	217247	.	+	.	ID=MALK_00911.t1;Parent=MALK_00911
contig003	maker	exon	216606	217247	.	+	.	ID=MALK_00911.t1.e1;Parent=MALK_00911.t1
contig003	AUGUSTUS	gene	217289	217765	0.98	-	.	ID=MALK_00912;prediction_source=braker_MRET:g2960.t1
contig003	AUGUSTUS	CDS	217289	217765	0.98	-	0	ID=MALK_00912.t1.c1;Parent=MALK_00912.t1
contig003	AUGUSTUS	mRNA	217289	217765	0.98	-	.	ID=MALK_00912.t1;Parent=MALK_00912
contig003	AUGUSTUS	exon	217289	217765	.	-	.	ID=MALK_00912.t1.e1;Parent=MALK_00912.t1
contig003	AUGUSTUS	gene	217760	220966	0.44	+	.	ID=MALK_00913;prediction_source=augustus:contig003.g3221.t1
contig003	AUGUSTUS	CDS	217760	220966	0.44	+	0	ID=MALK_00913.t1.c1;Parent=MALK_00913.t1
contig003	AUGUSTUS	mRNA	217760	220966	0.44	+	.	ID=MALK_00913.t1;Parent=MALK_00913
contig003	AUGUSTUS	exon	217760	220966	0.44	+	.	ID=MALK_00913.t1.e1;Parent=MALK_00913.t1
contig003	maker	gene	221109	223916	.	+	.	ID=MALK_00914;prediction_source=maker_MRET:augustus_masked-contig003-processed-gene-2.60-mRNA-1
contig003	maker	CDS	221109	223916	.	+	0	ID=MALK_00914.t1.c1;Parent=MALK_00914.t1
contig003	maker	mRNA	221109	223916	.	+	.	ID=MALK_00914.t1;Parent=MALK_00914
contig003	maker	exon	221109	223916	.	+	.	ID=MALK_00914.t1.e1;Parent=MALK_00914.t1
contig003	maker	gene	223933	225105	.	-	.	ID=MALK_00915;prediction_source=maker_MRET:augustus_masked-contig003-processed-gene-2.83-mRNA-1
contig003	maker	CDS	223933	225105	.	-	0	ID=MALK_00915.t1.c1;Parent=MALK_00915.t1
contig003	maker	mRNA	223933	225105	.	-	.	ID=MALK_00915.t1;Parent=MALK_00915
contig003	maker	exon	223933	225105	.	-	.	ID=MALK_00915.t1.e1;Parent=MALK_00915.t1
contig003	maker	gene	225367	228537	.	+	.	ID=MALK_00916;prediction_source=maker_MRET:augustus_masked-contig003-processed-gene-2.61-mRNA-1

contig003	maker	CDS	225367	228537	.	+	0	ID=MALK_00916.t1.c1;Parent=MALK_00916.t1
contig003	maker	mRNA	225367	228537	.	+	.	ID=MALK_00916.t1;Parent=MALK_00916
contig003	maker	exon	225367	228537	.	+	.	ID=MALK_00916.t1.e1;Parent=MALK_00916.t1
contig003	AUGUSTUS	gene	228595	230013	0.96	-	.	ID=MALK_00917;prediction_source=augustus:contig003.g3225.t1
contig003	AUGUSTUS	CDS	228595	230013	0.96	-	0	ID=MALK_00917.t1.c1;Parent=MALK_00917.t1
contig003	AUGUSTUS	mRNA	228595	230013	0.96	-	.	ID=MALK_00917.t1;Parent=MALK_00917
contig003	AUGUSTUS	exon	228595	230013	0.96	-	.	ID=MALK_00917.t1.e1;Parent=MALK_00917.t1
contig003	maker	gene	230356	231567	.	+	.	ID=MALK_00918;prediction_source=maker_MRET:augustus_masked-contig003-processed-gene-2.62-mRNA-1
contig003	maker	CDS	230356	231567	.	+	0	ID=MALK_00918.t1.c1;Parent=MALK_00918.t1
contig003	maker	mRNA	230356	231567	.	+	.	ID=MALK_00918.t1;Parent=MALK_00918
contig003	maker	exon	230356	231567	.	+	.	ID=MALK_00918.t1.e1;Parent=MALK_00918.t1
contig003	AUGUSTUS	gene	231597	232844	0.98	-	.	ID=MALK_00919;prediction_source=braker_MRET:g2966.t1
contig003	AUGUSTUS	CDS	231597	232844	0.98	-	0	ID=MALK_00919.t1.c1;Parent=MALK_00919.t1
contig003	AUGUSTUS	mRNA	231597	232844	0.98	-	.	ID=MALK_00919.t1;Parent=MALK_00919
contig003	AUGUSTUS	exon	231597	232844	.	-	.	ID=MALK_00919.t1.e1;Parent=MALK_00919.t1
contig003	AUGUSTUS	gene	232988	233512	0.93	+	.	ID=MALK_00920;prediction_source=augustus:contig003.g3228.t1
contig003	AUGUSTUS	CDS	232988	233512	0.93	+	0	ID=MALK_00920.t1.c1;Parent=MALK_00920.t1
contig003	AUGUSTUS	mRNA	232988	233512	0.93	+	.	ID=MALK_00920.t1;Parent=MALK_00920
contig003	AUGUSTUS	exon	232988	233512	0.93	+	.	ID=MALK_00920.t1.e1;Parent=MALK_00920.t1
contig003	AUGUSTUS	gene	233557	235098	0.98	-	.	ID=MALK_00921;prediction_source=augustus:contig003.g3229.t1
contig003	AUGUSTUS	CDS	233557	235098	0.98	-	0	ID=MALK_00921.t1.c1;Parent=MALK_00921.t1
contig003	AUGUSTUS	mRNA	233557	235098	0.98	-	.	ID=MALK_00921.t1;Parent=MALK_00921
contig003	AUGUSTUS	exon	233557	235098	0.98	-	.	ID=MALK_00921.t1.e1;Parent=MALK_00921.t1
contig003	maker	gene	235246	236154	.	+	.	ID=MALK_00922;prediction_source=maker_MRET:augustus_masked-contig003-processed-gene-2.64-mRNA-1
contig003	maker	CDS	235246	236154	.	+	0	ID=MALK_00922.t1.c1;Parent=MALK_00922.t1
contig003	maker	mRNA	235246	236154	.	+	.	ID=MALK_00922.t1;Parent=MALK_00922
contig003	maker	exon	235246	236154	.	+	.	ID=MALK_00922.t1.e1;Parent=MALK_00922.t1
contig003	AUGUSTUS	gene	236817	238232	0.98	-	.	ID=MALK_00923;prediction_source=augustus:contig003.g3231.t1
contig003	AUGUSTUS	CDS	236817	238232	0.98	-	0	ID=MALK_00923.t1.c1;Parent=MALK_00923.t1
contig003	AUGUSTUS	mRNA	236817	238232	0.98	-	.	ID=MALK_00923.t1;Parent=MALK_00923
contig003	AUGUSTUS	exon	236817	238232	0.98	-	.	ID=MALK_00923.t1.e1;Parent=MALK_00923.t1
contig003	AUGUSTUS	gene	238569	240611	1	-	.	ID=MALK_00924;prediction_source=augustus:contig003.g3232.t1
contig003	AUGUSTUS	CDS	238569	240611	1	-	0	ID=MALK_00924.t1.c1;Parent=MALK_00924.t1
contig003	AUGUSTUS	mRNA	238569	240611	1	-	.	ID=MALK_00924.t1;Parent=MALK_00924
contig003	AUGUSTUS	exon	238569	240611	1	-	.	ID=MALK_00924.t1.e1;Parent=MALK_00924.t1
contig003	maker	gene	241093	242658	.	+	.	ID=MALK_00925;prediction_source=maker_MRET:augustus_masked-contig003-processed-gene-2.65-mRNA-1
contig003	maker	CDS	241093	242658	.	+	0	ID=MALK_00925.t1.c1;Parent=MALK_00925.t1
contig003	maker	mRNA	241093	242658	.	+	.	ID=MALK_00925.t1;Parent=MALK_00925
contig003	maker	exon	241093	242658	.	+	.	ID=MALK_00925.t1.e1;Parent=MALK_00925.t1
contig003	maker	gene	243326	244030	.	+	.	ID=MALK_00926;prediction_source=maker_MRET:augustus_masked-contig003-processed-gene-2.66-mRNA-1
contig003	maker	CDS	243326	244030	.	+	0	ID=MALK_00926.t1.c1;Parent=MALK_00926.t1
contig003	maker	mRNA	243326	244030	.	+	.	ID=MALK_00926.t1;Parent=MALK_00926
contig003	maker	exon	243326	244030	.	+	.	ID=MALK_00926.t1.e1;Parent=MALK_00926.t1

contig003	maker	gene	244181	249427	.	-	.	ID=MALK_00927;prediction_source=maker_MRET:augustus_masked-contig003-processed-gene-2.89-mRNA-1
contig003	maker	CDS	244181	249427	.	-	0	ID=MALK_00927.t1.c1;Parent=MALK_00927.t1
contig003	maker	mRNA	244181	249427	.	-	.	ID=MALK_00927.t1;Parent=MALK_00927
contig003	maker	exon	244181	249427	.	-	.	ID=MALK_00927.t1.e1;Parent=MALK_00927.t1
contig003	maker	gene	249921	251552	.	-	.	ID=MALK_00928;prediction_source=maker_MRET:augustus_masked-contig003-processed-gene-2.90-mRNA-1
contig003	maker	CDS	249921	251552	.	-	0	ID=MALK_00928.t1.c1;Parent=MALK_00928.t1
contig003	maker	mRNA	249921	251552	.	-	.	ID=MALK_00928.t1;Parent=MALK_00928
contig003	maker	exon	249921	251552	.	-	.	ID=MALK_00928.t1.e1;Parent=MALK_00928.t1
contig003	maker	gene	251733	253718	.	-	.	ID=MALK_00929;prediction_source=maker_MRET:augustus_masked-contig003-processed-gene-2.91-mRNA-1
contig003	maker	CDS	251733	253718	.	-	0	ID=MALK_00929.t1.c1;Parent=MALK_00929.t1
contig003	maker	mRNA	251733	253718	.	-	.	ID=MALK_00929.t1;Parent=MALK_00929
contig003	maker	exon	251733	253718	.	-	.	ID=MALK_00929.t1.e1;Parent=MALK_00929.t1
contig003	maker	gene	254035	255111	.	+	.	ID=MALK_00930;prediction_source=maker_MRET:augustus_masked-contig003-processed-gene-2.67-mRNA-1
contig003	maker	CDS	254035	255111	.	+	0	ID=MALK_00930.t1.c1;Parent=MALK_00930.t1
contig003	maker	mRNA	254035	255111	.	+	.	ID=MALK_00930.t1;Parent=MALK_00930
contig003	maker	exon	254035	255111	.	+	.	ID=MALK_00930.t1.e1;Parent=MALK_00930.t1
contig003	AUGUSTUS	gene	255815	256549	0.49	+	.	ID=MALK_00931;prediction_source=braker_MRET:g2978.t1
contig003	AUGUSTUS	CDS	255815	256549	0.49	+	0	ID=MALK_00931.t1.c1;Parent=MALK_00931.t1
contig003	AUGUSTUS	mRNA	255815	256549	0.49	+	.	ID=MALK_00931.t1;Parent=MALK_00931
contig003	AUGUSTUS	exon	255815	256549	.	+	.	ID=MALK_00931.t1.e1;Parent=MALK_00931.t1
contig003	maker	gene	256624	257049	.	-	.	ID=MALK_00932;prediction_source=maker_MRET:augustus_masked-contig003-processed-gene-2.92-mRNA-1
contig003	maker	CDS	256624	257049	.	-	0	ID=MALK_00932.t1.c1;Parent=MALK_00932.t1
contig003	maker	mRNA	256624	257049	.	-	.	ID=MALK_00932.t1;Parent=MALK_00932
contig003	maker	exon	256624	257049	.	-	.	ID=MALK_00932.t1.e1;Parent=MALK_00932.t1
contig003	maker	gene	257576	257986	.	+	.	ID=MALK_00933;prediction_source=maker_MRET:augustus_masked-contig003-processed-gene-2.69-mRNA-1
contig003	maker	CDS	257576	257986	.	+	0	ID=MALK_00933.t1.c1;Parent=MALK_00933.t1
contig003	maker	mRNA	257576	257986	.	+	.	ID=MALK_00933.t1;Parent=MALK_00933
contig003	maker	exon	257576	257986	.	+	.	ID=MALK_00933.t1.e1;Parent=MALK_00933.t1
contig003	AUGUSTUS	gene	258098	259303	0.99	+	.	ID=MALK_00934;prediction_source=braker_MRET:g2981.t1
contig003	AUGUSTUS	CDS	258098	259303	0.99	+	0	ID=MALK_00934.t1.c1;Parent=MALK_00934.t1
contig003	AUGUSTUS	mRNA	258098	259303	0.99	+	.	ID=MALK_00934.t1;Parent=MALK_00934
contig003	AUGUSTUS	exon	258098	259303	.	+	.	ID=MALK_00934.t1.e1;Parent=MALK_00934.t1
contig003	AUGUSTUS	gene	259310	260459	0.69	-	.	ID=MALK_00935;prediction_source=braker_MRET:g2982.t1
contig003	AUGUSTUS	CDS	260361	260459	0.72	-	0	ID=MALK_00935.t1.c2;Parent=MALK_00935.t1
contig003	AUGUSTUS	CDS	259310	260332	0.72	-	0	ID=MALK_00935.t1.c1;Parent=MALK_00935.t1
contig003	AUGUSTUS	mRNA	259310	260459	0.69	-	.	ID=MALK_00935.t1;Parent=MALK_00935
contig003	AUGUSTUS	exon	260361	260459	.	-	.	ID=MALK_00935.t1.e2;Parent=MALK_00935.t1
contig003	AUGUSTUS	exon	259310	260332	.	-	.	ID=MALK_00935.t1.e1;Parent=MALK_00935.t1
contig003	maker	gene	260742	261815	.	+	.	ID=MALK_00936;prediction_source=maker_MRET:augustus_masked-contig003-processed-gene-2.71-mRNA-1
contig003	maker	CDS	260742	261815	.	+	0	ID=MALK_00936.t1.c1;Parent=MALK_00936.t1
contig003	maker	mRNA	260742	261815	.	+	.	ID=MALK_00936.t1;Parent=MALK_00936
contig003	maker	exon	260742	261815	.	+	.	ID=MALK_00936.t1.e1;Parent=MALK_00936.t1
contig003	maker	gene	261856	262722	.	-	.	ID=MALK_00937;prediction_source=maker_MRET:augustus_masked-contig003-processed-gene-2.94-mRNA-1

contig003	maker	CDS	261856	262722	.	-	0	ID=MALK_00937.t1.c1;Parent=MALK_00937.t1
contig003	maker	mRNA	261856	262722	.	-	.	ID=MALK_00937.t1;Parent=MALK_00937
contig003	maker	exon	261856	262722	.	-	.	ID=MALK_00937.t1.e1;Parent=MALK_00937.t1
contig003	AUGUSTUS	gene	263108	263686	0.52	+	.	ID=MALK_00938;prediction_source=augustus:contig003.g3244.t1
contig003	AUGUSTUS	CDS	263108	263686	0.52	+	0	ID=MALK_00938.t1.c1;Parent=MALK_00938.t1
contig003	AUGUSTUS	mRNA	263108	263686	0.52	+	.	ID=MALK_00938.t1;Parent=MALK_00938
contig003	AUGUSTUS	exon	263108	263686	0.52	+	.	ID=MALK_00938.t1.e1;Parent=MALK_00938.t1
contig003	AUGUSTUS	gene	263815	266337	0.99	-	.	ID=MALK_00939;prediction_source=augustus:contig003.g3245.t1
contig003	AUGUSTUS	CDS	263815	266337	0.99	-	0	ID=MALK_00939.t1.c1;Parent=MALK_00939.t1
contig003	AUGUSTUS	mRNA	263815	266337	0.99	-	.	ID=MALK_00939.t1;Parent=MALK_00939
contig003	AUGUSTUS	exon	263815	266337	0.99	-	.	ID=MALK_00939.t1.e1;Parent=MALK_00939.t1
contig003	AUGUSTUS	gene	266949	268268	0.93	+	.	ID=MALK_00940;prediction_source=braker_MRET:g2987.t1
contig003	AUGUSTUS	CDS	266949	268268	0.93	+	0	ID=MALK_00940.t1.c1;Parent=MALK_00940.t1
contig003	AUGUSTUS	mRNA	266949	268268	0.93	+	.	ID=MALK_00940.t1;Parent=MALK_00940
contig003	AUGUSTUS	exon	266949	268268	.	+	.	ID=MALK_00940.t1.e1;Parent=MALK_00940.t1
contig003	AUGUSTUS	gene	268673	270934	0.85	+	.	ID=MALK_00941;prediction_source=braker_MRET:g2988.t1
contig003	AUGUSTUS	CDS	268673	270934	0.85	+	0	ID=MALK_00941.t1.c1;Parent=MALK_00941.t1
contig003	AUGUSTUS	mRNA	268673	270934	0.85	+	.	ID=MALK_00941.t1;Parent=MALK_00941
contig003	AUGUSTUS	exon	268673	270934	.	+	.	ID=MALK_00941.t1.e1;Parent=MALK_00941.t1
contig003	AUGUSTUS	gene	270964	272916	0.34	-	.	ID=MALK_00942;prediction_source=braker_MRET:g2989.t1
contig003	AUGUSTUS	CDS	271812	272916	0.58	-	0	ID=MALK_00942.t1.c3;Parent=MALK_00942.t1
contig003	AUGUSTUS	CDS	271027	271777	0.58	-	0	ID=MALK_00942.t1.c2;Parent=MALK_00942.t1
contig003	AUGUSTUS	CDS	270964	270973	0.58	-	0	ID=MALK_00942.t1.c1;Parent=MALK_00942.t1
contig003	AUGUSTUS	mRNA	270964	272916	0.34	-	.	ID=MALK_00942.t1;Parent=MALK_00942
contig003	AUGUSTUS	exon	271812	272916	.	-	.	ID=MALK_00942.t1.e3;Parent=MALK_00942.t1
contig003	AUGUSTUS	exon	271027	271777	.	-	.	ID=MALK_00942.t1.e2;Parent=MALK_00942.t1
contig003	AUGUSTUS	exon	270964	270973	.	-	.	ID=MALK_00942.t1.e1;Parent=MALK_00942.t1
contig003	AUGUSTUS	gene	272953	274161	0.88	+	.	ID=MALK_00943;prediction_source=augustus:contig003.g3248.t1
contig003	AUGUSTUS	CDS	272953	274161	0.88	+	0	ID=MALK_00943.t1.c1;Parent=MALK_00943.t1
contig003	AUGUSTUS	mRNA	272953	274161	0.88	+	.	ID=MALK_00943.t1;Parent=MALK_00943
contig003	AUGUSTUS	exon	272953	274161	0.88	+	.	ID=MALK_00943.t1.e1;Parent=MALK_00943.t1
contig003	AUGUSTUS	gene	274524	275099	0.47	-	.	ID=MALK_00944;prediction_source=braker_MRET:g2991.t1
contig003	AUGUSTUS	CDS	274524	275099	0.47	-	0	ID=MALK_00944.t1.c1;Parent=MALK_00944.t1
contig003	AUGUSTUS	mRNA	274524	275099	0.47	-	.	ID=MALK_00944.t1;Parent=MALK_00944
contig003	AUGUSTUS	exon	274524	275099	.	-	.	ID=MALK_00944.t1.e1;Parent=MALK_00944.t1
contig003	AUGUSTUS	gene	275147	277174	0.53	+	.	ID=MALK_00945;prediction_source=augustus:contig003.g3249.t1
contig003	AUGUSTUS	CDS	275147	277174	0.53	+	0	ID=MALK_00945.t1.c1;Parent=MALK_00945.t1
contig003	AUGUSTUS	mRNA	275147	277174	0.53	+	.	ID=MALK_00945.t1;Parent=MALK_00945
contig003	AUGUSTUS	exon	275147	277174	0.53	+	.	ID=MALK_00945.t1.e1;Parent=MALK_00945.t1
contig003	AUGUSTUS	gene	277590	278268	0.35	-	.	ID=MALK_00946;prediction_source=braker_MRET:g2993.t1
contig003	AUGUSTUS	CDS	277957	278268	0.46	-	0	ID=MALK_00946.t1.c2;Parent=MALK_00946.t1
contig003	AUGUSTUS	CDS	277590	277925	0.46	-	0	ID=MALK_00946.t1.c1;Parent=MALK_00946.t1
contig003	AUGUSTUS	mRNA	277590	278268	0.35	-	.	ID=MALK_00946.t1;Parent=MALK_00946

contig003	AUGUSTUS	exon	277957	278268	.	-	.	ID=MALK_00946.t1.e2;Parent=MALK_00946.t1
contig003	AUGUSTUS	exon	277590	277925	.	-	.	ID=MALK_00946.t1.e1;Parent=MALK_00946.t1
contig003	maker	gene	278697	279515	.	-	.	ID=MALK_00947;prediction_source=maker_MRET:augustus_masked-contig003-processed-gene-2.97-mRNA-1
contig003	maker	CDS	278697	279515	.	-	0	ID=MALK_00947.t1.c1;Parent=MALK_00947.t1
contig003	maker	mRNA	278697	279515	.	-	.	ID=MALK_00947.t1;Parent=MALK_00947
contig003	maker	exon	278697	279515	.	-	.	ID=MALK_00947.t1.e1;Parent=MALK_00947.t1
contig003	AUGUSTUS	gene	279938	281167	0.62	+	.	ID=MALK_00948;prediction_source=augustus:contig003.g3252.t1
contig003	AUGUSTUS	CDS	279938	281167	0.62	+	0	ID=MALK_00948.t1.c1;Parent=MALK_00948.t1
contig003	AUGUSTUS	mRNA	279938	281167	0.62	+	.	ID=MALK_00948.t1;Parent=MALK_00948
contig003	AUGUSTUS	exon	279938	281167	0.62	+	.	ID=MALK_00948.t1.e1;Parent=MALK_00948.t1
contig003	maker	gene	281346	282332	.	+	.	ID=MALK_00949;prediction_source=maker_MRET:augustus_masked-contig003-processed-gene-2.77-mRNA-1
contig003	maker	CDS	281346	282332	.	+	0	ID=MALK_00949.t1.c1;Parent=MALK_00949.t1
contig003	maker	mRNA	281346	282332	.	+	.	ID=MALK_00949.t1;Parent=MALK_00949
contig003	maker	exon	281346	282332	.	+	.	ID=MALK_00949.t1.e1;Parent=MALK_00949.t1
contig003	AUGUSTUS	gene	282498	283886	0.95	-	.	ID=MALK_00950;prediction_source=braker_MRET:g2997.t1
contig003	AUGUSTUS	CDS	282498	283886	0.95	-	0	ID=MALK_00950.t1.c1;Parent=MALK_00950.t1
contig003	AUGUSTUS	mRNA	282498	283886	0.95	-	.	ID=MALK_00950.t1;Parent=MALK_00950
contig003	AUGUSTUS	exon	282498	283886	.	-	.	ID=MALK_00950.t1.e1;Parent=MALK_00950.t1
contig003	AUGUSTUS	gene	283858	285165	0.92	+	.	ID=MALK_00951;prediction_source=augustus:contig003.g3255.t1
contig003	AUGUSTUS	CDS	283858	285165	0.92	+	0	ID=MALK_00951.t1.c1;Parent=MALK_00951.t1
contig003	AUGUSTUS	mRNA	283858	285165	0.92	+	.	ID=MALK_00951.t1;Parent=MALK_00951
contig003	AUGUSTUS	exon	283858	285165	0.92	+	.	ID=MALK_00951.t1.e1;Parent=MALK_00951.t1
contig003	AUGUSTUS	gene	285162	288311	0.94	-	.	ID=MALK_00952;prediction_source=augustus:contig003.g3256.t1
contig003	AUGUSTUS	CDS	285162	288311	0.94	-	0	ID=MALK_00952.t1.c1;Parent=MALK_00952.t1
contig003	AUGUSTUS	mRNA	285162	288311	0.94	-	.	ID=MALK_00952.t1;Parent=MALK_00952
contig003	AUGUSTUS	exon	285162	288311	0.94	-	.	ID=MALK_00952.t1.e1;Parent=MALK_00952.t1
contig003	maker	gene	288349	289803	.	-	.	ID=MALK_00953;prediction_source=maker_MRET:augustus_masked-contig003-processed-gene-2.100-mRNA-1
contig003	maker	CDS	288349	289803	.	-	0	ID=MALK_00953.t1.c1;Parent=MALK_00953.t1
contig003	maker	mRNA	288349	289803	.	-	.	ID=MALK_00953.t1;Parent=MALK_00953
contig003	maker	exon	288349	289803	.	-	.	ID=MALK_00953.t1.e1;Parent=MALK_00953.t1
contig003	maker	gene	289946	293173	.	+	.	ID=MALK_00954;prediction_source=maker_MRET:augustus_masked-contig003-processed-gene-2.78-mRNA-1
contig003	maker	CDS	289946	293173	.	+	0	ID=MALK_00954.t1.c1;Parent=MALK_00954.t1
contig003	maker	mRNA	289946	293173	.	+	.	ID=MALK_00954.t1;Parent=MALK_00954
contig003	maker	exon	289946	293173	.	+	.	ID=MALK_00954.t1.e1;Parent=MALK_00954.t1
contig003	AUGUSTUS	gene	293334	293851	0.51	-	.	ID=MALK_00955;prediction_source=braker_MRET:g3002.t1
contig003	AUGUSTUS	CDS	293454	293851	0.52	-	0	ID=MALK_00955.t1.c2;Parent=MALK_00955.t1
contig003	AUGUSTUS	CDS	293334	293412	0.52	-	0	ID=MALK_00955.t1.c1;Parent=MALK_00955.t1
contig003	AUGUSTUS	mRNA	293334	293851	0.51	-	.	ID=MALK_00955.t1;Parent=MALK_00955
contig003	AUGUSTUS	exon	293454	293851	.	-	.	ID=MALK_00955.t1.e2;Parent=MALK_00955.t1
contig003	AUGUSTUS	exon	293334	293412	.	-	.	ID=MALK_00955.t1.e1;Parent=MALK_00955.t1
contig003	AUGUSTUS	gene	293871	296543	0.77	-	.	ID=MALK_00956;prediction_source=braker_MRET:g3003.t1
contig003	AUGUSTUS	CDS	293871	296543	0.77	-	0	ID=MALK_00956.t1.c1;Parent=MALK_00956.t1
contig003	AUGUSTUS	mRNA	293871	296543	0.77	-	.	ID=MALK_00956.t1;Parent=MALK_00956

contig003	AUGUSTUS	exon	293871	296543	.	-	.	ID=MALK_00956.t1.e1;Parent=MALK_00956.t1
contig003	AUGUSTUS	gene	296852	298615	0.94	+	.	ID=MALK_00957;prediction_source=braker_MRET:g3004.t1
contig003	AUGUSTUS	CDS	296852	298615	0.94	+	0	ID=MALK_00957.t1.c1;Parent=MALK_00957.t1
contig003	AUGUSTUS	mRNA	296852	298615	0.94	+	.	ID=MALK_00957.t1;Parent=MALK_00957
contig003	AUGUSTUS	exon	296852	298615	.	+	.	ID=MALK_00957.t1.e1;Parent=MALK_00957.t1
contig003	AUGUSTUS	gene	298612	299292	0.96	-	.	ID=MALK_00958;prediction_source=augustus:contig003.g3261.t1
contig003	AUGUSTUS	CDS	298612	299292	0.96	-	0	ID=MALK_00958.t1.c1;Parent=MALK_00958.t1
contig003	AUGUSTUS	mRNA	298612	299292	0.96	-	.	ID=MALK_00958.t1;Parent=MALK_00958
contig003	AUGUSTUS	exon	298612	299292	0.96	-	.	ID=MALK_00958.t1.e1;Parent=MALK_00958.t1
contig003	AUGUSTUS	gene	299952	300214	0.91	+	.	ID=MALK_00959;prediction_source=braker_MRET:g3006.t1
contig003	AUGUSTUS	CDS	299952	300048	0.99	+	0	ID=MALK_00959.t1.c1;Parent=MALK_00959.t1
contig003	AUGUSTUS	CDS	300084	300214	0.99	+	0	ID=MALK_00959.t1.c2;Parent=MALK_00959.t1
contig003	AUGUSTUS	mRNA	299952	300214	0.91	+	.	ID=MALK_00959.t1;Parent=MALK_00959
contig003	AUGUSTUS	exon	299952	300048	.	+	.	ID=MALK_00959.t1.e1;Parent=MALK_00959.t1
contig003	AUGUSTUS	exon	300084	300214	.	+	.	ID=MALK_00959.t1.e2;Parent=MALK_00959.t1
contig003	maker	gene	300573	301274	.	-	.	ID=MALK_00960;prediction_source=maker_MRET:augustus_masked-contig003-processed-gene-3.27-mRNA-1
contig003	maker	CDS	300573	301274	.	-	0	ID=MALK_00960.t1.c1;Parent=MALK_00960.t1
contig003	maker	mRNA	300573	301274	.	-	.	ID=MALK_00960.t1;Parent=MALK_00960
contig003	maker	exon	300573	301274	.	-	.	ID=MALK_00960.t1.e1;Parent=MALK_00960.t1
contig003	AUGUSTUS	gene	301457	302180	0.72	+	.	ID=MALK_00961;prediction_source=braker_MRET:g3008.t1
contig003	AUGUSTUS	CDS	301457	301490	0.99	+	0	ID=MALK_00961.t1.c1;Parent=MALK_00961.t1
contig003	AUGUSTUS	CDS	301548	301562	0.99	+	0	ID=MALK_00961.t1.c2;Parent=MALK_00961.t1
contig003	AUGUSTUS	CDS	301681	301961	0.99	+	0	ID=MALK_00961.t1.c3;Parent=MALK_00961.t1
contig003	AUGUSTUS	CDS	302001	302180	0.99	+	0	ID=MALK_00961.t1.c4;Parent=MALK_00961.t1
contig003	AUGUSTUS	mRNA	301457	302180	0.72	+	.	ID=MALK_00961.t1;Parent=MALK_00961
contig003	AUGUSTUS	exon	301457	301490	.	+	.	ID=MALK_00961.t1.e1;Parent=MALK_00961.t1
contig003	AUGUSTUS	exon	301548	301562	.	+	.	ID=MALK_00961.t1.e2;Parent=MALK_00961.t1
contig003	AUGUSTUS	exon	301681	301961	.	+	.	ID=MALK_00961.t1.e3;Parent=MALK_00961.t1
contig003	AUGUSTUS	exon	302001	302180	.	+	.	ID=MALK_00961.t1.e4;Parent=MALK_00961.t1
contig003	AUGUSTUS	gene	302431	305682	0.86	+	.	ID=MALK_00962;prediction_source=augustus:contig003.g3264.t1
contig003	AUGUSTUS	CDS	302431	305682	0.86	+	0	ID=MALK_00962.t1.c1;Parent=MALK_00962.t1
contig003	AUGUSTUS	mRNA	302431	305682	0.86	+	.	ID=MALK_00962.t1;Parent=MALK_00962
contig003	AUGUSTUS	exon	302431	305682	0.86	+	.	ID=MALK_00962.t1.e1;Parent=MALK_00962.t1
contig003	maker	gene	305864	307537	.	+	.	ID=MALK_00963;prediction_source=maker_MRET:augustus_masked-contig003-processed-gene-3.5-mRNA-1
contig003	maker	CDS	305864	307537	.	+	0	ID=MALK_00963.t1.c1;Parent=MALK_00963.t1
contig003	maker	mRNA	305864	307537	.	+	.	ID=MALK_00963.t1;Parent=MALK_00963
contig003	maker	exon	305864	307537	.	+	.	ID=MALK_00963.t1.e1;Parent=MALK_00963.t1
contig003	AUGUSTUS	gene	307671	308070	0.21	+	.	ID=MALK_00964;prediction_source=braker_MRET:g3011.t1
contig003	AUGUSTUS	CDS	307671	307972	0.46	+	0	ID=MALK_00964.t1.c1;Parent=MALK_00964.t1
contig003	AUGUSTUS	CDS	308001	308070	0.46	+	0	ID=MALK_00964.t1.c2;Parent=MALK_00964.t1
contig003	AUGUSTUS	mRNA	307671	308070	0.21	+	.	ID=MALK_00964.t1;Parent=MALK_00964
contig003	AUGUSTUS	exon	307671	307972	.	+	.	ID=MALK_00964.t1.e1;Parent=MALK_00964.t1
contig003	AUGUSTUS	exon	308001	308070	.	+	.	ID=MALK_00964.t1.e2;Parent=MALK_00964.t1

contig003	AUGUSTUS	gene	308337	310175	0.52	-	.	ID=MALK_00965;prediction_source=augustus:contig003.g3266.t1
contig003	AUGUSTUS	CDS	308337	310175	0.52	-	0	ID=MALK_00965.t1.c1;Parent=MALK_00965.t1
contig003	AUGUSTUS	mRNA	308337	310175	0.52	-	.	ID=MALK_00965.t1;Parent=MALK_00965
contig003	AUGUSTUS	exon	308337	310175	0.52	-	.	ID=MALK_00965.t1.e1;Parent=MALK_00965.t1
contig003	AUGUSTUS	gene	310257	312257	0.93	-	.	ID=MALK_00966;prediction_source=braker_MRET:g3013.t1
contig003	AUGUSTUS	CDS	310257	312257	0.93	-	0	ID=MALK_00966.t1.c1;Parent=MALK_00966.t1
contig003	AUGUSTUS	mRNA	310257	312257	0.93	-	.	ID=MALK_00966.t1;Parent=MALK_00966
contig003	AUGUSTUS	exon	310257	312257	.	-	.	ID=MALK_00966.t1.e1;Parent=MALK_00966.t1
contig003	AUGUSTUS	gene	312424	314841	0.74	+	.	ID=MALK_00967;prediction_source=braker_MRET:g3014.t1
contig003	AUGUSTUS	CDS	312424	314841	0.74	+	0	ID=MALK_00967.t1.c1;Parent=MALK_00967.t1
contig003	AUGUSTUS	mRNA	312424	314841	0.74	+	.	ID=MALK_00967.t1;Parent=MALK_00967
contig003	AUGUSTUS	exon	312424	314841	.	+	.	ID=MALK_00967.t1.e1;Parent=MALK_00967.t1
contig003	maker	gene	314844	315659	.	-	.	ID=MALK_00968;prediction_source=maker_MRET:augustus_masked-contig003-processed-gene-3.30-mRNA-1
contig003	maker	CDS	314844	315659	.	-	0	ID=MALK_00968.t1.c1;Parent=MALK_00968.t1
contig003	maker	mRNA	314844	315659	.	-	.	ID=MALK_00968.t1;Parent=MALK_00968
contig003	maker	exon	314844	315659	.	-	.	ID=MALK_00968.t1.e1;Parent=MALK_00968.t1
contig003	AUGUSTUS	gene	316579	318279	0.41	+	.	ID=MALK_00969;prediction_source=augustus:contig003.g3270.t1
contig003	AUGUSTUS	CDS	316579	318279	0.41	+	0	ID=MALK_00969.t1.c1;Parent=MALK_00969.t1
contig003	AUGUSTUS	mRNA	316579	318279	0.41	+	.	ID=MALK_00969.t1;Parent=MALK_00969
contig003	AUGUSTUS	exon	316579	318279	0.41	+	.	ID=MALK_00969.t1.e1;Parent=MALK_00969.t1
contig003	maker	gene	318346	320274	.	+	.	ID=MALK_00970;prediction_source=maker_MRET:augustus_masked-contig003-processed-gene-3.8-mRNA-1
contig003	maker	CDS	318346	320274	.	+	0	ID=MALK_00970.t1.c1;Parent=MALK_00970.t1
contig003	maker	mRNA	318346	320274	.	+	.	ID=MALK_00970.t1;Parent=MALK_00970
contig003	maker	exon	318346	320274	.	+	.	ID=MALK_00970.t1.e1;Parent=MALK_00970.t1
contig003	AUGUSTUS	gene	320269	321195	0.52	-	.	ID=MALK_00971;prediction_source=braker_MRET:g3018.t1
contig003	AUGUSTUS	CDS	320269	321195	0.52	-	0	ID=MALK_00971.t1.c1;Parent=MALK_00971.t1
contig003	AUGUSTUS	mRNA	320269	321195	0.52	-	.	ID=MALK_00971.t1;Parent=MALK_00971
contig003	AUGUSTUS	exon	320269	321195	.	-	.	ID=MALK_00971.t1.e1;Parent=MALK_00971.t1
contig003	AUGUSTUS	gene	321220	322140	0.51	+	.	ID=MALK_00972;prediction_source=braker_MRET:g3019.t1
contig003	AUGUSTUS	CDS	321220	322140	0.51	+	0	ID=MALK_00972.t1.c1;Parent=MALK_00972.t1
contig003	AUGUSTUS	mRNA	321220	322140	0.51	+	.	ID=MALK_00972.t1;Parent=MALK_00972
contig003	AUGUSTUS	exon	321220	322140	.	+	.	ID=MALK_00972.t1.e1;Parent=MALK_00972.t1
contig003	maker	gene	322321	323694	.	+	.	ID=MALK_00973;prediction_source=maker_MRET:augustus_masked-contig003-processed-gene-3.10-mRNA-1
contig003	maker	CDS	322321	323694	.	+	0	ID=MALK_00973.t1.c1;Parent=MALK_00973.t1
contig003	maker	mRNA	322321	323694	.	+	.	ID=MALK_00973.t1;Parent=MALK_00973
contig003	maker	exon	322321	323694	.	+	.	ID=MALK_00973.t1.e1;Parent=MALK_00973.t1
contig003	AUGUSTUS	gene	323729	324655	0.76	-	.	ID=MALK_00974;prediction_source=braker_MRET:g3021.t1
contig003	AUGUSTUS	CDS	323729	324655	0.76	-	0	ID=MALK_00974.t1.c1;Parent=MALK_00974.t1
contig003	AUGUSTUS	mRNA	323729	324655	0.76	-	.	ID=MALK_00974.t1;Parent=MALK_00974
contig003	AUGUSTUS	exon	323729	324655	.	-	.	ID=MALK_00974.t1.e1;Parent=MALK_00974.t1
contig003	AUGUSTUS	gene	324740	328630	0.89	-	.	ID=MALK_00975;prediction_source=braker_MRET:g3022.t1
contig003	AUGUSTUS	CDS	324740	328630	0.89	-	0	ID=MALK_00975.t1.c1;Parent=MALK_00975.t1
contig003	AUGUSTUS	mRNA	324740	328630	0.89	-	.	ID=MALK_00975.t1;Parent=MALK_00975

contig003	AUGUSTUS	exon	324740	328630	.	-	.	ID=MALK_00975.t1.e1;Parent=MALK_00975.t1
contig003	AUGUSTUS	gene	328725	331115	1	-	.	ID=MALK_00976;prediction_source=augustus:contig003.g3277.t1
contig003	AUGUSTUS	CDS	328725	331115	1	-	0	ID=MALK_00976.t1.c1;Parent=MALK_00976.t1
contig003	AUGUSTUS	mRNA	328725	331115	1	-	.	ID=MALK_00976.t1;Parent=MALK_00976
contig003	AUGUSTUS	exon	328725	331115	1	-	.	ID=MALK_00976.t1.e1;Parent=MALK_00976.t1
contig003	AUGUSTUS	gene	332020	333132	1	-	.	ID=MALK_00977;prediction_source=braker_MRET:g3024.t1
contig003	AUGUSTUS	CDS	332020	333132	1	-	0	ID=MALK_00977.t1.c1;Parent=MALK_00977.t1
contig003	AUGUSTUS	mRNA	332020	333132	1	-	.	ID=MALK_00977.t1;Parent=MALK_00977
contig003	AUGUSTUS	exon	332020	333132	.	-	.	ID=MALK_00977.t1.e1;Parent=MALK_00977.t1
contig003	AUGUSTUS	gene	333131	334123	0.99	+	.	ID=MALK_00978;prediction_source=augustus:contig003.g3279.t1
contig003	AUGUSTUS	CDS	333131	334123	0.99	+	0	ID=MALK_00978.t1.c1;Parent=MALK_00978.t1
contig003	AUGUSTUS	mRNA	333131	334123	0.99	+	.	ID=MALK_00978.t1;Parent=MALK_00978
contig003	AUGUSTUS	exon	333131	334123	0.99	+	.	ID=MALK_00978.t1.e1;Parent=MALK_00978.t1
contig003	AUGUSTUS	gene	334120	335730	0.92	-	.	ID=MALK_00979;prediction_source=augustus:contig003.g3280.t1
contig003	AUGUSTUS	CDS	334120	335730	0.92	-	0	ID=MALK_00979.t1.c1;Parent=MALK_00979.t1
contig003	AUGUSTUS	mRNA	334120	335730	0.92	-	.	ID=MALK_00979.t1;Parent=MALK_00979
contig003	AUGUSTUS	exon	334120	335730	0.92	-	.	ID=MALK_00979.t1.e1;Parent=MALK_00979.t1
contig003	AUGUSTUS	gene	335848	337131	0.12	+	.	ID=MALK_00980;prediction_source=braker_MRET:g3027.t1
contig003	AUGUSTUS	CDS	335848	336339	1	+	0	ID=MALK_00980.t1.c1;Parent=MALK_00980.t1
contig003	AUGUSTUS	CDS	336443	336595	1	+	0	ID=MALK_00980.t1.c2;Parent=MALK_00980.t1
contig003	AUGUSTUS	CDS	336632	336669	1	+	0	ID=MALK_00980.t1.c3;Parent=MALK_00980.t1
contig003	AUGUSTUS	CDS	336750	336872	1	+	0	ID=MALK_00980.t1.c4;Parent=MALK_00980.t1
contig003	AUGUSTUS	CDS	336915	337021	1	+	0	ID=MALK_00980.t1.c5;Parent=MALK_00980.t1
contig003	AUGUSTUS	CDS	337049	337131	1	+	0	ID=MALK_00980.t1.c6;Parent=MALK_00980.t1
contig003	AUGUSTUS	mRNA	335848	337131	0.12	+	.	ID=MALK_00980.t1;Parent=MALK_00980
contig003	AUGUSTUS	exon	335848	336339	.	+	.	ID=MALK_00980.t1.e1;Parent=MALK_00980.t1
contig003	AUGUSTUS	exon	336443	336595	.	+	.	ID=MALK_00980.t1.e2;Parent=MALK_00980.t1
contig003	AUGUSTUS	exon	336632	336669	.	+	.	ID=MALK_00980.t1.e3;Parent=MALK_00980.t1
contig003	AUGUSTUS	exon	336750	336872	.	+	.	ID=MALK_00980.t1.e4;Parent=MALK_00980.t1
contig003	AUGUSTUS	exon	336915	337021	.	+	.	ID=MALK_00980.t1.e5;Parent=MALK_00980.t1
contig003	AUGUSTUS	exon	337049	337131	.	+	.	ID=MALK_00980.t1.e6;Parent=MALK_00980.t1
contig003	maker	gene	337289	338626	.	+	.	ID=MALK_00981;prediction_source=maker_MRET:augustus_masked-contig003-processed-gene-3.13-mRNA-1
contig003	maker	CDS	337289	338626	.	+	0	ID=MALK_00981.t1.c1;Parent=MALK_00981.t1
contig003	maker	mRNA	337289	338626	.	+	.	ID=MALK_00981.t1;Parent=MALK_00981
contig003	maker	exon	337289	338626	.	+	.	ID=MALK_00981.t1.e1;Parent=MALK_00981.t1
contig003	AUGUSTUS	gene	338808	339394	0.82	-	.	ID=MALK_00982;prediction_source=braker_MRET:g3029.t1
contig003	AUGUSTUS	CDS	339128	339394	0.98	-	0	ID=MALK_00982.t1.c2;Parent=MALK_00982.t1
contig003	AUGUSTUS	CDS	338808	339092	0.98	-	0	ID=MALK_00982.t1.c1;Parent=MALK_00982.t1
contig003	AUGUSTUS	mRNA	338808	339394	0.82	-	.	ID=MALK_00982.t1;Parent=MALK_00982
contig003	AUGUSTUS	exon	339128	339394	.	-	.	ID=MALK_00982.t1.e2;Parent=MALK_00982.t1
contig003	AUGUSTUS	exon	338808	339092	.	-	.	ID=MALK_00982.t1.e1;Parent=MALK_00982.t1
contig003	AUGUSTUS	gene	339436	340206	0.96	+	.	ID=MALK_00983;prediction_source=braker_MRET:g3030.t1
contig003	AUGUSTUS	CDS	339436	339586	0.98	+	0	ID=MALK_00983.t1.c1;Parent=MALK_00983.t1

contig003	AUGUSTUS	CDS	339620	340206	0.98	+	0	ID=MALK_00983.t1.c2;Parent=MALK_00983.t1
contig003	AUGUSTUS	mRNA	339436	340206	0.96	+	.	ID=MALK_00983.t1;Parent=MALK_00983
contig003	AUGUSTUS	exon	339436	339586	.	+	.	ID=MALK_00983.t1.e1;Parent=MALK_00983.t1
contig003	AUGUSTUS	exon	339620	340206	.	+	.	ID=MALK_00983.t1.e2;Parent=MALK_00983.t1
contig003	maker	gene	340507	343968	.	+	.	ID=MALK_00984;prediction_source=maker_MRET:augustus_masked-contig003-processed-gene-3.14-mRNA-1
contig003	maker	CDS	340507	343968	.	+	0	ID=MALK_00984.t1.c1;Parent=MALK_00984.t1
contig003	maker	mRNA	340507	343968	.	+	.	ID=MALK_00984.t1;Parent=MALK_00984
contig003	maker	exon	340507	343968	.	+	.	ID=MALK_00984.t1.e1;Parent=MALK_00984.t1
contig003	maker	gene	344000	346021	.	+	.	ID=MALK_00985;prediction_source=maker_MRET:augustus_masked-contig003-processed-gene-3.15-mRNA-1
contig003	maker	CDS	344000	346021	.	+	0	ID=MALK_00985.t1.c1;Parent=MALK_00985.t1
contig003	maker	mRNA	344000	346021	.	+	.	ID=MALK_00985.t1;Parent=MALK_00985
contig003	maker	exon	344000	346021	.	+	.	ID=MALK_00985.t1.e1;Parent=MALK_00985.t1
contig003	AUGUSTUS	gene	346030	347757	0.71	-	.	ID=MALK_00986;prediction_source=augustus:contig003.g3287.t1
contig003	AUGUSTUS	CDS	346030	347757	0.71	-	0	ID=MALK_00986.t1.c1;Parent=MALK_00986.t1
contig003	AUGUSTUS	mRNA	346030	347757	0.71	-	.	ID=MALK_00986.t1;Parent=MALK_00986
contig003	AUGUSTUS	exon	346030	347757	0.71	-	.	ID=MALK_00986.t1.e1;Parent=MALK_00986.t1
contig003	maker	gene	347943	348593	.	+	.	ID=MALK_00987;prediction_source=maker_MRET:augustus_masked-contig003-processed-gene-3.16-mRNA-1
contig003	maker	CDS	347943	348593	.	+	0	ID=MALK_00987.t1.c1;Parent=MALK_00987.t1
contig003	maker	mRNA	347943	348593	.	+	.	ID=MALK_00987.t1;Parent=MALK_00987
contig003	maker	exon	347943	348593	.	+	.	ID=MALK_00987.t1.e1;Parent=MALK_00987.t1
contig003	AUGUSTUS	gene	348628	349225	0.79	-	.	ID=MALK_00988;prediction_source=augustus:contig003.g3289.t1
contig003	AUGUSTUS	CDS	349003	349225	0.79	-	0	ID=MALK_00988.t1.c2;Parent=MALK_00988.t1
contig003	AUGUSTUS	CDS	348628	348917	0.79	-	0	ID=MALK_00988.t1.c1;Parent=MALK_00988.t1
contig003	AUGUSTUS	mRNA	348628	349225	0.79	-	.	ID=MALK_00988.t1;Parent=MALK_00988
contig003	AUGUSTUS	exon	349003	349225	0.79	-	.	ID=MALK_00988.t1.e2;Parent=MALK_00988.t1
contig003	AUGUSTUS	exon	348628	348917	0.79	-	.	ID=MALK_00988.t1.e1;Parent=MALK_00988.t1
contig003	maker	gene	349284	351083	.	+	.	ID=MALK_00989;prediction_source=maker_MRET:augustus_masked-contig003-processed-gene-3.17-mRNA-1
contig003	maker	CDS	349284	351083	.	+	0	ID=MALK_00989.t1.c1;Parent=MALK_00989.t1
contig003	maker	mRNA	349284	351083	.	+	.	ID=MALK_00989.t1;Parent=MALK_00989
contig003	maker	exon	349284	351083	.	+	.	ID=MALK_00989.t1.e1;Parent=MALK_00989.t1
contig003	AUGUSTUS	gene	351824	354880	0.41	-	.	ID=MALK_00990;prediction_source=augustus:contig003.g3292.t1
contig003	AUGUSTUS	CDS	351824	354880	0.41	-	0	ID=MALK_00990.t1.c1;Parent=MALK_00990.t1
contig003	AUGUSTUS	mRNA	351824	354880	0.41	-	.	ID=MALK_00990.t1;Parent=MALK_00990
contig003	AUGUSTUS	exon	351824	354880	0.41	-	.	ID=MALK_00990.t1.e1;Parent=MALK_00990.t1
contig003	maker	gene	355064	357376	.	+	.	ID=MALK_00991;prediction_source=maker_MRET:augustus_masked-contig003-processed-gene-3.18-mRNA-1
contig003	maker	CDS	355064	357376	.	+	0	ID=MALK_00991.t1.c1;Parent=MALK_00991.t1
contig003	maker	mRNA	355064	357376	.	+	.	ID=MALK_00991.t1;Parent=MALK_00991
contig003	maker	exon	355064	357376	.	+	.	ID=MALK_00991.t1.e1;Parent=MALK_00991.t1
contig003	AUGUSTUS	gene	357382	358821	1	-	.	ID=MALK_00992;prediction_source=braker_MRET:g3039.t1
contig003	AUGUSTUS	CDS	357382	358821	1	-	0	ID=MALK_00992.t1.c1;Parent=MALK_00992.t1
contig003	AUGUSTUS	mRNA	357382	358821	1	-	.	ID=MALK_00992.t1;Parent=MALK_00992
contig003	AUGUSTUS	exon	357382	358821	.	-	.	ID=MALK_00992.t1.e1;Parent=MALK_00992.t1
contig003	AUGUSTUS	gene	358896	359352	0.65	+	.	ID=MALK_00993;prediction_source=braker_MRET:g3040.t1

contig003	AUGUSTUS	CDS	358896	359020	0.73	+	0	ID=MALK_00993.t1.c1;Parent=MALK_00993.t1
contig003	AUGUSTUS	CDS	359048	359087	0.73	+	0	ID=MALK_00993.t1.c2;Parent=MALK_00993.t1
contig003	AUGUSTUS	CDS	359134	359352	0.73	+	0	ID=MALK_00993.t1.c3;Parent=MALK_00993.t1
contig003	AUGUSTUS	mRNA	358896	359352	0.65	+	.	ID=MALK_00993.t1;Parent=MALK_00993
contig003	AUGUSTUS	exon	358896	359020	.	+	.	ID=MALK_00993.t1.e1;Parent=MALK_00993.t1
contig003	AUGUSTUS	exon	359048	359087	.	+	.	ID=MALK_00993.t1.e2;Parent=MALK_00993.t1
contig003	AUGUSTUS	exon	359134	359352	.	+	.	ID=MALK_00993.t1.e3;Parent=MALK_00993.t1
contig003	AUGUSTUS	gene	359547	361061	0.89	-	.	ID=MALK_00994;prediction_source=augustus:contig003.g3295.t1
contig003	AUGUSTUS	CDS	359547	361061	0.89	-	0	ID=MALK_00994.t1.c1;Parent=MALK_00994.t1
contig003	AUGUSTUS	mRNA	359547	361061	0.89	-	.	ID=MALK_00994.t1;Parent=MALK_00994
contig003	AUGUSTUS	exon	359547	361061	0.89	-	.	ID=MALK_00994.t1.e1;Parent=MALK_00994.t1
contig003	AUGUSTUS	gene	361164	364301	0.93	+	.	ID=MALK_00995;prediction_source=braker_MRET:g3042.t1
contig003	AUGUSTUS	CDS	361164	364301	0.93	+	0	ID=MALK_00995.t1.c1;Parent=MALK_00995.t1
contig003	AUGUSTUS	mRNA	361164	364301	0.93	+	.	ID=MALK_00995.t1;Parent=MALK_00995
contig003	AUGUSTUS	exon	361164	364301	.	+	.	ID=MALK_00995.t1.e1;Parent=MALK_00995.t1
contig003	AUGUSTUS	gene	364448	365227	0.47	+	.	ID=MALK_00996;prediction_source=braker_MRET:g3043.t1
contig003	AUGUSTUS	CDS	364448	365227	0.47	+	0	ID=MALK_00996.t1.c1;Parent=MALK_00996.t1
contig003	AUGUSTUS	mRNA	364448	365227	0.47	+	.	ID=MALK_00996.t1;Parent=MALK_00996
contig003	AUGUSTUS	exon	364448	365227	.	+	.	ID=MALK_00996.t1.e1;Parent=MALK_00996.t1
contig003	AUGUSTUS	gene	365366	366205	0.92	+	.	ID=MALK_00997;prediction_source=augustus:contig003.g3297.t1
contig003	AUGUSTUS	CDS	365366	366205	0.92	+	0	ID=MALK_00997.t1.c1;Parent=MALK_00997.t1
contig003	AUGUSTUS	mRNA	365366	366205	0.92	+	.	ID=MALK_00997.t1;Parent=MALK_00997
contig003	AUGUSTUS	exon	365366	366205	0.92	+	.	ID=MALK_00997.t1.e1;Parent=MALK_00997.t1
contig003	AUGUSTUS	gene	366570	367508	0.88	+	.	ID=MALK_00998;prediction_source=braker_MRET:g3045.t1
contig003	AUGUSTUS	CDS	366570	366752	0.99	+	0	ID=MALK_00998.t1.c1;Parent=MALK_00998.t1
contig003	AUGUSTUS	CDS	366816	367508	0.99	+	0	ID=MALK_00998.t1.c2;Parent=MALK_00998.t1
contig003	AUGUSTUS	mRNA	366570	367508	0.88	+	.	ID=MALK_00998.t1;Parent=MALK_00998
contig003	AUGUSTUS	exon	366570	366752	.	+	.	ID=MALK_00998.t1.e1;Parent=MALK_00998.t1
contig003	AUGUSTUS	exon	366816	367508	.	+	.	ID=MALK_00998.t1.e2;Parent=MALK_00998.t1
contig003	AUGUSTUS	gene	367525	368943	0.21	-	.	ID=MALK_00999;prediction_source=augustus:contig003.g3299.t1
contig003	AUGUSTUS	CDS	367525	368943	0.21	-	0	ID=MALK_00999.t1.c1;Parent=MALK_00999.t1
contig003	AUGUSTUS	mRNA	367525	368943	0.21	-	.	ID=MALK_00999.t1;Parent=MALK_00999
contig003	AUGUSTUS	exon	367525	368943	0.21	-	.	ID=MALK_00999.t1.e1;Parent=MALK_00999.t1
contig003	AUGUSTUS	gene	369077	370537	0.36	-	.	ID=MALK_01000;prediction_source=augustus:contig003.g3300.t1
contig003	AUGUSTUS	CDS	369077	370537	0.36	-	0	ID=MALK_01000.t1.c1;Parent=MALK_01000.t1
contig003	AUGUSTUS	mRNA	369077	370537	0.36	-	.	ID=MALK_01000.t1;Parent=MALK_01000
contig003	AUGUSTUS	exon	369077	370537	0.36	-	.	ID=MALK_01000.t1.e1;Parent=MALK_01000.t1
contig003	AUGUSTUS	gene	370794	372518	0.98	+	.	ID=MALK_01001;prediction_source=augustus:contig003.g3301.t1
contig003	AUGUSTUS	CDS	370794	372518	0.98	+	0	ID=MALK_01001.t1.c1;Parent=MALK_01001.t1
contig003	AUGUSTUS	mRNA	370794	372518	0.98	+	.	ID=MALK_01001.t1;Parent=MALK_01001
contig003	AUGUSTUS	exon	370794	372518	0.98	+	.	ID=MALK_01001.t1.e1;Parent=MALK_01001.t1
contig003	maker	gene	372546	373919	.	-	.	ID=MALK_01002;prediction_source=maker_MRET:augustus_masked-contig003-processed-gene-3.40-mRNA-1
contig003	maker	CDS	372546	373919	.	-	0	ID=MALK_01002.t1.c1;Parent=MALK_01002.t1

contig003	maker	mRNA	372546	373919	.	-	.	ID=MALK_01002.t1;Parent=MALK_01002
contig003	maker	exon	372546	373919	.	-	.	ID=MALK_01002.t1.e1;Parent=MALK_01002.t1
contig003	AUGUSTUS	gene	374094	374469	0.36	+	.	ID=MALK_01003;prediction_source=braker_MRET:g3050.t1
contig003	AUGUSTUS	CDS	374094	374357	0.55	+	0	ID=MALK_01003.t1.c1;Parent=MALK_01003.t1
contig003	AUGUSTUS	CDS	374389	374469	0.55	+	0	ID=MALK_01003.t1.c2;Parent=MALK_01003.t1
contig003	AUGUSTUS	mRNA	374094	374469	0.36	+	.	ID=MALK_01003.t1;Parent=MALK_01003
contig003	AUGUSTUS	exon	374094	374357	.	+	.	ID=MALK_01003.t1.e1;Parent=MALK_01003.t1
contig003	AUGUSTUS	exon	374389	374469	.	+	.	ID=MALK_01003.t1.e2;Parent=MALK_01003.t1
contig003	AUGUSTUS	gene	374472	375042	0.52	-	.	ID=MALK_01004;prediction_source=braker_MRET:g3051.t1
contig003	AUGUSTUS	CDS	374646	375042	0.53	-	0	ID=MALK_01004.t1.c2;Parent=MALK_01004.t1
contig003	AUGUSTUS	CDS	374472	374611	0.53	-	0	ID=MALK_01004.t1.c1;Parent=MALK_01004.t1
contig003	AUGUSTUS	mRNA	374472	375042	0.52	-	.	ID=MALK_01004.t1;Parent=MALK_01004
contig003	AUGUSTUS	exon	374646	375042	.	-	.	ID=MALK_01004.t1.e2;Parent=MALK_01004.t1
contig003	AUGUSTUS	exon	374472	374611	.	-	.	ID=MALK_01004.t1.e1;Parent=MALK_01004.t1
contig003	maker	gene	375273	379682	.	+	.	ID=MALK_01005;prediction_source=maker_MRET:augustus_masked-contig003-processed-gene-3.23-mRNA-1
contig003	maker	CDS	375273	379682	.	+	0	ID=MALK_01005.t1.c1;Parent=MALK_01005.t1
contig003	maker	mRNA	375273	379682	.	+	.	ID=MALK_01005.t1;Parent=MALK_01005
contig003	maker	exon	375273	379682	.	+	.	ID=MALK_01005.t1.e1;Parent=MALK_01005.t1
contig003	AUGUSTUS	gene	379649	386377	0.7	-	.	ID=MALK_01006;prediction_source=augustus:contig003.g3305.t1
contig003	AUGUSTUS	CDS	379649	386377	0.7	-	0	ID=MALK_01006.t1.c1;Parent=MALK_01006.t1
contig003	AUGUSTUS	mRNA	379649	386377	0.7	-	.	ID=MALK_01006.t1;Parent=MALK_01006
contig003	AUGUSTUS	exon	379649	386377	0.7	-	.	ID=MALK_01006.t1.e1;Parent=MALK_01006.t1
contig003	maker	gene	386551	388206	.	+	.	ID=MALK_01007;prediction_source=maker_MRET:augustus_masked-contig003-processed-gene-3.24-mRNA-1
contig003	maker	CDS	386551	388206	.	+	0	ID=MALK_01007.t1.c1;Parent=MALK_01007.t1
contig003	maker	mRNA	386551	388206	.	+	.	ID=MALK_01007.t1;Parent=MALK_01007
contig003	maker	exon	386551	388206	.	+	.	ID=MALK_01007.t1.e1;Parent=MALK_01007.t1
contig003	AUGUSTUS	gene	388299	388957	0.6	-	.	ID=MALK_01008;prediction_source=braker_MRET:g3055.t1
contig003	AUGUSTUS	CDS	388345	388957	0.6	-	0	ID=MALK_01008.t1.c2;Parent=MALK_01008.t1
contig003	AUGUSTUS	CDS	388299	388306	0.6	-	0	ID=MALK_01008.t1.c1;Parent=MALK_01008.t1
contig003	AUGUSTUS	mRNA	388299	388957	0.6	-	.	ID=MALK_01008.t1;Parent=MALK_01008
contig003	AUGUSTUS	exon	388345	388957	.	-	.	ID=MALK_01008.t1.e2;Parent=MALK_01008.t1
contig003	AUGUSTUS	exon	388299	388306	.	-	.	ID=MALK_01008.t1.e1;Parent=MALK_01008.t1
contig003	AUGUSTUS	gene	389286	391244	0.73	+	.	ID=MALK_01009;prediction_source=braker_MRET:g3056.t1
contig003	AUGUSTUS	CDS	389286	391244	0.73	+	0	ID=MALK_01009.t1.c1;Parent=MALK_01009.t1
contig003	AUGUSTUS	mRNA	389286	391244	0.73	+	.	ID=MALK_01009.t1;Parent=MALK_01009
contig003	AUGUSTUS	exon	389286	391244	.	+	.	ID=MALK_01009.t1.e1;Parent=MALK_01009.t1
contig003	AUGUSTUS	gene	391259	392167	0.97	-	.	ID=MALK_01010;prediction_source=braker_MRET:g3057.t1
contig003	AUGUSTUS	CDS	391259	392167	0.97	-	0	ID=MALK_01010.t1.c1;Parent=MALK_01010.t1
contig003	AUGUSTUS	mRNA	391259	392167	0.97	-	.	ID=MALK_01010.t1;Parent=MALK_01010
contig003	AUGUSTUS	exon	391259	392167	.	-	.	ID=MALK_01010.t1.e1;Parent=MALK_01010.t1
contig003	AUGUSTUS	gene	392206	392883	0.34	-	.	ID=MALK_01011;prediction_source=braker_MRET:g3058.t1
contig003	AUGUSTUS	CDS	392773	392883	0.55	-	0	ID=MALK_01011.t1.c3;Parent=MALK_01011.t1
contig003	AUGUSTUS	CDS	392327	392605	0.55	-	0	ID=MALK_01011.t1.c2;Parent=MALK_01011.t1

contig003	AUGUSTUS	CDS	392206	392298	0.55	-	0	ID=MALK_01011.t1.c1;Parent=MALK_01011.t1
contig003	AUGUSTUS	mRNA	392206	392883	0.34	-	.	ID=MALK_01011.t1;Parent=MALK_01011
contig003	AUGUSTUS	exon	392773	392883	.	-	.	ID=MALK_01011.t1.e3;Parent=MALK_01011.t1
contig003	AUGUSTUS	exon	392327	392605	.	-	.	ID=MALK_01011.t1.e2;Parent=MALK_01011.t1
contig003	AUGUSTUS	exon	392206	392298	.	-	.	ID=MALK_01011.t1.e1;Parent=MALK_01011.t1
contig003	AUGUSTUS	gene	392932	393421	0.55	+	.	ID=MALK_01012;prediction_source=braker_MRET:g3059.t1
contig003	AUGUSTUS	CDS	392932	393059	1	+	0	ID=MALK_01012.t1.c1;Parent=MALK_01012.t1
contig003	AUGUSTUS	CDS	393092	393191	1	+	0	ID=MALK_01012.t1.c2;Parent=MALK_01012.t1
contig003	AUGUSTUS	CDS	393326	393421	1	+	0	ID=MALK_01012.t1.c3;Parent=MALK_01012.t1
contig003	AUGUSTUS	mRNA	392932	393421	0.55	+	.	ID=MALK_01012.t1;Parent=MALK_01012
contig003	AUGUSTUS	exon	392932	393059	.	+	.	ID=MALK_01012.t1.e1;Parent=MALK_01012.t1
contig003	AUGUSTUS	exon	393092	393191	.	+	.	ID=MALK_01012.t1.e2;Parent=MALK_01012.t1
contig003	AUGUSTUS	exon	393326	393421	.	+	.	ID=MALK_01012.t1.e3;Parent=MALK_01012.t1
contig003	maker	gene	393681	394580	.	+	.	ID=MALK_01013;prediction_source=maker_MRET:augustus_masked-contig003-processed-gene-3.26-mRNA-1
contig003	maker	CDS	393681	394580	.	+	0	ID=MALK_01013.t1.c1;Parent=MALK_01013.t1
contig003	maker	mRNA	393681	394580	.	+	.	ID=MALK_01013.t1;Parent=MALK_01013
contig003	maker	exon	393681	394580	.	+	.	ID=MALK_01013.t1.e1;Parent=MALK_01013.t1
contig003	AUGUSTUS	gene	394583	396808	0.94	-	.	ID=MALK_01014;prediction_source=augustus:contig003.g3311.t1
contig003	AUGUSTUS	CDS	394583	396808	0.94	-	0	ID=MALK_01014.t1.c1;Parent=MALK_01014.t1
contig003	AUGUSTUS	mRNA	394583	396808	0.94	-	.	ID=MALK_01014.t1;Parent=MALK_01014
contig003	AUGUSTUS	exon	394583	396808	0.94	-	.	ID=MALK_01014.t1.e1;Parent=MALK_01014.t1
contig003	maker	gene	397570	399327	.	-	.	ID=MALK_01015;prediction_source=maker_MRET:augustus_masked-contig003-processed-gene-4.2-mRNA-1
contig003	maker	CDS	397570	399327	.	-	0	ID=MALK_01015.t1.c1;Parent=MALK_01015.t1
contig003	maker	mRNA	397570	399327	.	-	.	ID=MALK_01015.t1;Parent=MALK_01015
contig003	maker	exon	397570	399327	.	-	.	ID=MALK_01015.t1.e1;Parent=MALK_01015.t1
contig003	AUGUSTUS	gene	399471	400415	0.65	+	.	ID=MALK_01016;prediction_source=braker_MRET:g3063.t1
contig003	AUGUSTUS	CDS	399471	400415	0.65	+	0	ID=MALK_01016.t1.c1;Parent=MALK_01016.t1
contig003	AUGUSTUS	mRNA	399471	400415	0.65	+	.	ID=MALK_01016.t1;Parent=MALK_01016
contig003	AUGUSTUS	exon	399471	400415	.	+	.	ID=MALK_01016.t1.e1;Parent=MALK_01016.t1
contig003	AUGUSTUS	gene	400446	400961	0.56	-	.	ID=MALK_01017;prediction_source=braker_MRET:g3064.t1
contig003	AUGUSTUS	CDS	400446	400961	0.56	-	0	ID=MALK_01017.t1.c1;Parent=MALK_01017.t1
contig003	AUGUSTUS	mRNA	400446	400961	0.56	-	.	ID=MALK_01017.t1;Parent=MALK_01017
contig003	AUGUSTUS	exon	400446	400961	.	-	.	ID=MALK_01017.t1.e1;Parent=MALK_01017.t1
contig003	AUGUSTUS	gene	401220	402806	0.73	+	.	ID=MALK_01018;prediction_source=augustus:contig003.g3315.t1
contig003	AUGUSTUS	CDS	401220	402806	0.73	+	0	ID=MALK_01018.t1.c1;Parent=MALK_01018.t1
contig003	AUGUSTUS	mRNA	401220	402806	0.73	+	.	ID=MALK_01018.t1;Parent=MALK_01018
contig003	AUGUSTUS	exon	401220	402806	0.73	+	.	ID=MALK_01018.t1.e1;Parent=MALK_01018.t1
contig003	maker	gene	402842	404467	.	-	.	ID=MALK_01019;prediction_source=maker_MRET:augustus_masked-contig003-processed-gene-4.30-mRNA-1
contig003	maker	CDS	402842	404467	.	-	0	ID=MALK_01019.t1.c1;Parent=MALK_01019.t1
contig003	maker	mRNA	402842	404467	.	-	.	ID=MALK_01019.t1;Parent=MALK_01019
contig003	maker	exon	402842	404467	.	-	.	ID=MALK_01019.t1.e1;Parent=MALK_01019.t1
contig003	AUGUSTUS	gene	404553	406112	0.99	+	.	ID=MALK_01020;prediction_source=augustus:contig003.g3317.t1
contig003	AUGUSTUS	CDS	404553	406112	0.99	+	0	ID=MALK_01020.t1.c1;Parent=MALK_01020.t1

contig003	AUGUSTUS	mRNA	404553	406112	0.99	+	.	ID=MALK_01020.t1;Parent=MALK_01020
contig003	AUGUSTUS	exon	404553	406112	0.99	+	.	ID=MALK_01020.t1.e1;Parent=MALK_01020.t1
contig003	AUGUSTUS	gene	406202	406840	1	-	.	ID=MALK_01021;prediction_source=braker_MRET:g3068.t1
contig003	AUGUSTUS	CDS	406805	406840	1	-	0	ID=MALK_01021.t1.c3;Parent=MALK_01021.t1
contig003	AUGUSTUS	CDS	406509	406764	1	-	0	ID=MALK_01021.t1.c2;Parent=MALK_01021.t1
contig003	AUGUSTUS	CDS	406202	406479	1	-	0	ID=MALK_01021.t1.c1;Parent=MALK_01021.t1
contig003	AUGUSTUS	mRNA	406202	406840	1	-	.	ID=MALK_01021.t1;Parent=MALK_01021
contig003	AUGUSTUS	exon	406805	406840	.	-	.	ID=MALK_01021.t1.e3;Parent=MALK_01021.t1
contig003	AUGUSTUS	exon	406509	406764	.	-	.	ID=MALK_01021.t1.e2;Parent=MALK_01021.t1
contig003	AUGUSTUS	exon	406202	406479	.	-	.	ID=MALK_01021.t1.e1;Parent=MALK_01021.t1
contig003	maker	gene	406983	407546	.	+	.	ID=MALK_01022;prediction_source=maker_MRET:augustus_masked-contig003-processed-gene-4.5-mRNA-1
contig003	maker	CDS	406983	407546	.	+	0	ID=MALK_01022.t1.c1;Parent=MALK_01022.t1
contig003	maker	mRNA	406983	407546	.	+	.	ID=MALK_01022.t1;Parent=MALK_01022
contig003	maker	exon	406983	407546	.	+	.	ID=MALK_01022.t1.e1;Parent=MALK_01022.t1
contig003	AUGUSTUS	gene	407630	408625	1	-	.	ID=MALK_01023;prediction_source=braker_MRET:g3070.t1
contig003	AUGUSTUS	CDS	407630	408625	1	-	0	ID=MALK_01023.t1.c1;Parent=MALK_01023.t1
contig003	AUGUSTUS	mRNA	407630	408625	1	-	.	ID=MALK_01023.t1;Parent=MALK_01023
contig003	AUGUSTUS	exon	407630	408625	.	-	.	ID=MALK_01023.t1.e1;Parent=MALK_01023.t1
contig003	AUGUSTUS	gene	408891	409868	0.53	+	.	ID=MALK_01024;prediction_source=braker_MRET:g3071.t1
contig003	AUGUSTUS	CDS	408891	409868	0.53	+	0	ID=MALK_01024.t1.c1;Parent=MALK_01024.t1
contig003	AUGUSTUS	mRNA	408891	409868	0.53	+	.	ID=MALK_01024.t1;Parent=MALK_01024
contig003	AUGUSTUS	exon	408891	409868	.	+	.	ID=MALK_01024.t1.e1;Parent=MALK_01024.t1
contig003	AUGUSTUS	gene	410061	417155	0.54	+	.	ID=MALK_01025;prediction_source=braker_MRET:g3072.t1
contig003	AUGUSTUS	CDS	410061	411671	0.66	+	0	ID=MALK_01025.t1.c1;Parent=MALK_01025.t1
contig003	AUGUSTUS	CDS	411876	417155	0.66	+	0	ID=MALK_01025.t1.c2;Parent=MALK_01025.t1
contig003	AUGUSTUS	mRNA	410061	417155	0.54	+	.	ID=MALK_01025.t1;Parent=MALK_01025
contig003	AUGUSTUS	exon	410061	411671	.	+	.	ID=MALK_01025.t1.e1;Parent=MALK_01025.t1
contig003	AUGUSTUS	exon	411876	417155	.	+	.	ID=MALK_01025.t1.e2;Parent=MALK_01025.t1
contig003	AUGUSTUS	gene	417236	419080	0.74	+	.	ID=MALK_01026;prediction_source=braker_MRET:g3073.t1
contig003	AUGUSTUS	CDS	417236	419080	0.74	+	0	ID=MALK_01026.t1.c1;Parent=MALK_01026.t1
contig003	AUGUSTUS	mRNA	417236	419080	0.74	+	.	ID=MALK_01026.t1;Parent=MALK_01026
contig003	AUGUSTUS	exon	417236	419080	.	+	.	ID=MALK_01026.t1.e1;Parent=MALK_01026.t1
contig003	maker	gene	419179	420738	.	+	.	ID=MALK_01027;prediction_source=maker_MRET:augustus_masked-contig003-processed-gene-4.8-mRNA-1
contig003	maker	CDS	419179	420738	.	+	0	ID=MALK_01027.t1.c1;Parent=MALK_01027.t1
contig003	maker	mRNA	419179	420738	.	+	.	ID=MALK_01027.t1;Parent=MALK_01027
contig003	maker	exon	419179	420738	.	+	.	ID=MALK_01027.t1.e1;Parent=MALK_01027.t1
contig003	maker	gene	420826	424095	.	+	.	ID=MALK_01028;prediction_source=maker_MRET:augustus_masked-contig003-processed-gene-4.9-mRNA-1
contig003	maker	CDS	420826	424095	.	+	0	ID=MALK_01028.t1.c1;Parent=MALK_01028.t1
contig003	maker	mRNA	420826	424095	.	+	.	ID=MALK_01028.t1;Parent=MALK_01028
contig003	maker	exon	420826	424095	.	+	.	ID=MALK_01028.t1.e1;Parent=MALK_01028.t1
contig003	AUGUSTUS	gene	424287	424898	0.85	+	.	ID=MALK_01029;prediction_source=augustus:contig003.g3329.t1
contig003	AUGUSTUS	CDS	424287	424898	0.85	+	0	ID=MALK_01029.t1.c1;Parent=MALK_01029.t1
contig003	AUGUSTUS	mRNA	424287	424898	0.85	+	.	ID=MALK_01029.t1;Parent=MALK_01029

contig003	AUGUSTUS	exon	424287	424898	0.85	+	.	ID=MALK_01029.t1.e1;Parent=MALK_01029.t1
contig003	AUGUSTUS	gene	425006	426052	0.7	-	.	ID=MALK_01030;prediction_source=augustus:contig003.g3330.t1
contig003	AUGUSTUS	CDS	425006	426052	0.7	-	0	ID=MALK_01030.t1.c1;Parent=MALK_01030.t1
contig003	AUGUSTUS	mRNA	425006	426052	0.7	-	.	ID=MALK_01030.t1;Parent=MALK_01030
contig003	AUGUSTUS	exon	425006	426052	0.7	-	.	ID=MALK_01030.t1.e1;Parent=MALK_01030.t1
contig003	AUGUSTUS	gene	426197	427054	0.4	+	.	ID=MALK_01031;prediction_source=augustus:contig003.g3331.t1
contig003	AUGUSTUS	CDS	426197	427054	0.4	+	0	ID=MALK_01031.t1.c1;Parent=MALK_01031.t1
contig003	AUGUSTUS	mRNA	426197	427054	0.4	+	.	ID=MALK_01031.t1;Parent=MALK_01031
contig003	AUGUSTUS	exon	426197	427054	0.4	+	.	ID=MALK_01031.t1.e1;Parent=MALK_01031.t1
contig003	AUGUSTUS	gene	427081	427966	0.25	-	.	ID=MALK_01032;prediction_source=braker_MRET:g3078.t1
contig003	AUGUSTUS	CDS	427666	427966	0.26	-	0	ID=MALK_01032.t1.c2;Parent=MALK_01032.t1
contig003	AUGUSTUS	CDS	427081	427622	0.26	-	0	ID=MALK_01032.t1.c1;Parent=MALK_01032.t1
contig003	AUGUSTUS	mRNA	427081	427966	0.25	-	.	ID=MALK_01032.t1;Parent=MALK_01032
contig003	AUGUSTUS	exon	427666	427966	.	-	.	ID=MALK_01032.t1.e2;Parent=MALK_01032.t1
contig003	AUGUSTUS	exon	427081	427622	.	-	.	ID=MALK_01032.t1.e1;Parent=MALK_01032.t1
contig003	AUGUSTUS	gene	428010	429362	0.81	-	.	ID=MALK_01033;prediction_source=augustus:contig003.g3333.t1
contig003	AUGUSTUS	CDS	428010	429362	0.81	-	0	ID=MALK_01033.t1.c1;Parent=MALK_01033.t1
contig003	AUGUSTUS	mRNA	428010	429362	0.81	-	.	ID=MALK_01033.t1;Parent=MALK_01033
contig003	AUGUSTUS	exon	428010	429362	0.81	-	.	ID=MALK_01033.t1.e1;Parent=MALK_01033.t1
contig003	AUGUSTUS	gene	429398	430404	0.7	-	.	ID=MALK_01034;prediction_source=augustus:contig003.g3334.t1
contig003	AUGUSTUS	CDS	429923	430404	0.7	-	0	ID=MALK_01034.t1.c2;Parent=MALK_01034.t1
contig003	AUGUSTUS	CDS	429398	429845	0.7	-	0	ID=MALK_01034.t1.c1;Parent=MALK_01034.t1
contig003	AUGUSTUS	mRNA	429398	430404	0.7	-	.	ID=MALK_01034.t1;Parent=MALK_01034
contig003	AUGUSTUS	exon	429923	430404	0.7	-	.	ID=MALK_01034.t1.e2;Parent=MALK_01034.t1
contig003	AUGUSTUS	exon	429398	429845	0.7	-	.	ID=MALK_01034.t1.e1;Parent=MALK_01034.t1
contig003	AUGUSTUS	gene	430661	431947	0.79	+	.	ID=MALK_01035;prediction_source=augustus:contig003.g3335.t1
contig003	AUGUSTUS	CDS	430661	431947	0.79	+	0	ID=MALK_01035.t1.c1;Parent=MALK_01035.t1
contig003	AUGUSTUS	mRNA	430661	431947	0.79	+	.	ID=MALK_01035.t1;Parent=MALK_01035
contig003	AUGUSTUS	exon	430661	431947	0.79	+	.	ID=MALK_01035.t1.e1;Parent=MALK_01035.t1
contig003	AUGUSTUS	gene	432063	433016	0.95	+	.	ID=MALK_01036;prediction_source=augustus:contig003.g3337.t1
contig003	AUGUSTUS	CDS	432063	433016	0.95	+	0	ID=MALK_01036.t1.c1;Parent=MALK_01036.t1
contig003	AUGUSTUS	mRNA	432063	433016	0.95	+	.	ID=MALK_01036.t1;Parent=MALK_01036
contig003	AUGUSTUS	exon	432063	433016	0.95	+	.	ID=MALK_01036.t1.e1;Parent=MALK_01036.t1
contig003	AUGUSTUS	gene	433123	434050	0.91	-	.	ID=MALK_01037;prediction_source=braker_MRET:g3082.t1
contig003	AUGUSTUS	CDS	433214	434050	0.91	-	0	ID=MALK_01037.t1.c2;Parent=MALK_01037.t1
contig003	AUGUSTUS	CDS	433123	433179	0.91	-	0	ID=MALK_01037.t1.c1;Parent=MALK_01037.t1
contig003	AUGUSTUS	mRNA	433123	434050	0.91	-	.	ID=MALK_01037.t1;Parent=MALK_01037
contig003	AUGUSTUS	exon	433214	434050	.	-	.	ID=MALK_01037.t1.e2;Parent=MALK_01037.t1
contig003	AUGUSTUS	exon	433123	433179	.	-	.	ID=MALK_01037.t1.e1;Parent=MALK_01037.t1
contig003	maker	gene	434836	435826	.	+	.	ID=MALK_01038;prediction_source=maker_MRET:augustus_masked-contig003-processed-gene-4.13-mRNA-1
contig003	maker	CDS	434836	434958	.	+	0	ID=MALK_01038.t1.c1;Parent=MALK_01038.t1
contig003	maker	CDS	435212	435826	.	+	0	ID=MALK_01038.t1.c2;Parent=MALK_01038.t1
contig003	maker	mRNA	434836	435826	.	+	.	ID=MALK_01038.t1;Parent=MALK_01038

contig003	maker	exon	434836	434958	.	+	.	ID=MALK_01038.t1.e1;Parent=MALK_01038.t1
contig003	maker	exon	435212	435826	.	+	.	ID=MALK_01038.t1.e2;Parent=MALK_01038.t1
contig003	maker	gene	436305	438641	.	+	.	ID=MALK_01039;prediction_source=maker_MRET:augustus_masked-contig003-processed-gene-4.14-mRNA-1
contig003	maker	CDS	436305	438641	.	+	0	ID=MALK_01039.t1.c1;Parent=MALK_01039.t1
contig003	maker	mRNA	436305	438641	.	+	.	ID=MALK_01039.t1;Parent=MALK_01039
contig003	maker	exon	436305	438641	.	+	.	ID=MALK_01039.t1.e1;Parent=MALK_01039.t1
contig003	AUGUSTUS	gene	438749	439819	0.95	-	.	ID=MALK_01040;prediction_source=braker_MRET:g3085.t1
contig003	AUGUSTUS	CDS	438749	439819	0.95	-	0	ID=MALK_01040.t1.c1;Parent=MALK_01040.t1
contig003	AUGUSTUS	mRNA	438749	439819	0.95	-	.	ID=MALK_01040.t1;Parent=MALK_01040
contig003	AUGUSTUS	exon	438749	439819	.	-	.	ID=MALK_01040.t1.e1;Parent=MALK_01040.t1
contig003	AUGUSTUS	gene	439926	442981	0.82	-	.	ID=MALK_01041;prediction_source=braker_MRET:g3086.t1
contig003	AUGUSTUS	CDS	442684	442981	0.94	-	0	ID=MALK_01041.t1.c2;Parent=MALK_01041.t1
contig003	AUGUSTUS	CDS	439926	442618	0.94	-	0	ID=MALK_01041.t1.c1;Parent=MALK_01041.t1
contig003	AUGUSTUS	mRNA	439926	442981	0.82	-	.	ID=MALK_01041.t1;Parent=MALK_01041
contig003	AUGUSTUS	exon	442684	442981	.	-	.	ID=MALK_01041.t1.e2;Parent=MALK_01041.t1
contig003	AUGUSTUS	exon	439926	442618	.	-	.	ID=MALK_01041.t1.e1;Parent=MALK_01041.t1
contig003	maker	gene	443387	445732	.	+	.	ID=MALK_01042;prediction_source=maker_MRET:augustus_masked-contig003-processed-gene-4.15-mRNA-1
contig003	maker	CDS	443387	445732	.	+	0	ID=MALK_01042.t1.c1;Parent=MALK_01042.t1
contig003	maker	mRNA	443387	445732	.	+	.	ID=MALK_01042.t1;Parent=MALK_01042
contig003	maker	exon	443387	445732	.	+	.	ID=MALK_01042.t1.e1;Parent=MALK_01042.t1
contig003	maker	gene	445778	447562	.	-	.	ID=MALK_01043;prediction_source=maker_MRET:augustus_masked-contig003-processed-gene-4.40-mRNA-1
contig003	maker	CDS	445778	447562	.	-	0	ID=MALK_01043.t1.c1;Parent=MALK_01043.t1
contig003	maker	mRNA	445778	447562	.	-	.	ID=MALK_01043.t1;Parent=MALK_01043
contig003	maker	exon	445778	447562	.	-	.	ID=MALK_01043.t1.e1;Parent=MALK_01043.t1
contig003	AUGUSTUS	gene	447715	449655	0.98	+	.	ID=MALK_01044;prediction_source=braker_MRET:g3089.t1
contig003	AUGUSTUS	CDS	447715	449655	0.98	+	0	ID=MALK_01044.t1.c1;Parent=MALK_01044.t1
contig003	AUGUSTUS	mRNA	447715	449655	0.98	+	.	ID=MALK_01044.t1;Parent=MALK_01044
contig003	AUGUSTUS	exon	447715	449655	.	+	.	ID=MALK_01044.t1.e1;Parent=MALK_01044.t1
contig003	maker	gene	450085	450894	.	+	.	ID=MALK_01045;prediction_source=maker_MRET:augustus_masked-contig003-processed-gene-4.17-mRNA-1
contig003	maker	CDS	450085	450894	.	+	0	ID=MALK_01045.t1.c1;Parent=MALK_01045.t1
contig003	maker	mRNA	450085	450894	.	+	.	ID=MALK_01045.t1;Parent=MALK_01045
contig003	maker	exon	450085	450894	.	+	.	ID=MALK_01045.t1.e1;Parent=MALK_01045.t1
contig003	AUGUSTUS	gene	451257	452963	0.86	+	.	ID=MALK_01046;prediction_source=augustus:contig003.g3348.t1
contig003	AUGUSTUS	CDS	451257	452963	0.86	+	0	ID=MALK_01046.t1.c1;Parent=MALK_01046.t1
contig003	AUGUSTUS	mRNA	451257	452963	0.86	+	.	ID=MALK_01046.t1;Parent=MALK_01046
contig003	AUGUSTUS	exon	451257	452963	0.86	+	.	ID=MALK_01046.t1.e1;Parent=MALK_01046.t1
contig003	AUGUSTUS	gene	452971	454205	0.86	-	.	ID=MALK_01047;prediction_source=braker_MRET:g3091.t1
contig003	AUGUSTUS	CDS	454181	454205	0.94	-	0	ID=MALK_01047.t1.c2;Parent=MALK_01047.t1
contig003	AUGUSTUS	CDS	452971	454142	0.94	-	0	ID=MALK_01047.t1.c1;Parent=MALK_01047.t1
contig003	AUGUSTUS	mRNA	452971	454205	0.86	-	.	ID=MALK_01047.t1;Parent=MALK_01047
contig003	AUGUSTUS	exon	454181	454205	.	-	.	ID=MALK_01047.t1.e2;Parent=MALK_01047.t1
contig003	AUGUSTUS	exon	452971	454142	.	-	.	ID=MALK_01047.t1.e1;Parent=MALK_01047.t1
contig003	AUGUSTUS	gene	454484	454708	0.99	+	.	ID=MALK_01048;prediction_source=augustus:contig003.g3350.t1

contig003	AUGUSTUS	CDS	454484	454708	0.99	+	0	ID=MALK_01048.t1.c1;Parent=MALK_01048.t1
contig003	AUGUSTUS	mRNA	454484	454708	0.99	+	.	ID=MALK_01048.t1;Parent=MALK_01048
contig003	AUGUSTUS	exon	454484	454708	0.99	+	.	ID=MALK_01048.t1.e1;Parent=MALK_01048.t1
contig003	maker	gene	454942	456020	.	+	.	ID=MALK_01049;prediction_source=maker_MRET:augustus_masked-contig003-processed-gene-4.20-mRNA-1
contig003	maker	CDS	454942	455082	.	+	0	ID=MALK_01049.t1.c1;Parent=MALK_01049.t1
contig003	maker	CDS	455179	455593	.	+	0	ID=MALK_01049.t1.c2;Parent=MALK_01049.t1
contig003	maker	CDS	455680	456020	.	+	0	ID=MALK_01049.t1.c3;Parent=MALK_01049.t1
contig003	maker	mRNA	454942	456020	.	+	.	ID=MALK_01049.t1;Parent=MALK_01049
contig003	maker	exon	454942	455082	.	+	.	ID=MALK_01049.t1.e1;Parent=MALK_01049.t1
contig003	maker	exon	455179	455593	.	+	.	ID=MALK_01049.t1.e2;Parent=MALK_01049.t1
contig003	maker	exon	455680	456020	.	+	.	ID=MALK_01049.t1.e3;Parent=MALK_01049.t1
contig003	maker	gene	456323	456814	.	+	.	ID=MALK_01050;prediction_source=maker_MRET:augustus_masked-contig003-processed-gene-4.21-mRNA-1
contig003	maker	CDS	456323	456814	.	+	0	ID=MALK_01050.t1.c1;Parent=MALK_01050.t1
contig003	maker	mRNA	456323	456814	.	+	.	ID=MALK_01050.t1;Parent=MALK_01050
contig003	maker	exon	456323	456814	.	+	.	ID=MALK_01050.t1.e1;Parent=MALK_01050.t1
contig003	AUGUSTUS	gene	457237	459114	0.59	-	.	ID=MALK_01051;prediction_source=braker_MRET:g3093.t1
contig003	AUGUSTUS	CDS	459042	459114	0.59	-	0	ID=MALK_01051.t1.c2;Parent=MALK_01051.t1
contig003	AUGUSTUS	CDS	457237	458996	0.59	-	0	ID=MALK_01051.t1.c1;Parent=MALK_01051.t1
contig003	AUGUSTUS	mRNA	457237	459114	0.59	-	.	ID=MALK_01051.t1;Parent=MALK_01051
contig003	AUGUSTUS	exon	459042	459114	.	-	.	ID=MALK_01051.t1.e2;Parent=MALK_01051.t1
contig003	AUGUSTUS	exon	457237	458996	.	-	.	ID=MALK_01051.t1.e1;Parent=MALK_01051.t1
contig003	maker	gene	459645	461207	.	-	.	ID=MALK_01052;prediction_source=maker_MRET:augustus_masked-contig003-processed-gene-4.43-mRNA-1
contig003	maker	CDS	459645	461207	.	-	0	ID=MALK_01052.t1.c1;Parent=MALK_01052.t1
contig003	maker	mRNA	459645	461207	.	-	.	ID=MALK_01052.t1;Parent=MALK_01052
contig003	maker	exon	459645	461207	.	-	.	ID=MALK_01052.t1.e1;Parent=MALK_01052.t1
contig003	AUGUSTUS	gene	461299	462360	0.35	+	.	ID=MALK_01053;prediction_source=augustus:contig003.g3355.t1
contig003	AUGUSTUS	CDS	461299	462360	0.35	+	0	ID=MALK_01053.t1.c1;Parent=MALK_01053.t1
contig003	AUGUSTUS	mRNA	461299	462360	0.35	+	.	ID=MALK_01053.t1;Parent=MALK_01053
contig003	AUGUSTUS	exon	461299	462360	0.35	+	.	ID=MALK_01053.t1.e1;Parent=MALK_01053.t1
contig003	maker	gene	462350	464473	.	-	.	ID=MALK_01054;prediction_source=maker_MRET:augustus_masked-contig003-processed-gene-4.44-mRNA-1
contig003	maker	CDS	462350	464473	.	-	0	ID=MALK_01054.t1.c1;Parent=MALK_01054.t1
contig003	maker	mRNA	462350	464473	.	-	.	ID=MALK_01054.t1;Parent=MALK_01054
contig003	maker	exon	462350	464473	.	-	.	ID=MALK_01054.t1.e1;Parent=MALK_01054.t1
contig003	AUGUSTUS	gene	464618	465463	0.92	+	.	ID=MALK_01055;prediction_source=augustus:contig003.g3357.t1
contig003	AUGUSTUS	CDS	464618	465463	0.92	+	0	ID=MALK_01055.t1.c1;Parent=MALK_01055.t1
contig003	AUGUSTUS	mRNA	464618	465463	0.92	+	.	ID=MALK_01055.t1;Parent=MALK_01055
contig003	AUGUSTUS	exon	464618	465463	0.92	+	.	ID=MALK_01055.t1.e1;Parent=MALK_01055.t1
contig003	AUGUSTUS	gene	465393	467930	0.51	-	.	ID=MALK_01056;prediction_source=braker_MRET:g3098.t1
contig003	AUGUSTUS	CDS	465393	467930	0.51	-	0	ID=MALK_01056.t1.c1;Parent=MALK_01056.t1
contig003	AUGUSTUS	mRNA	465393	467930	0.51	-	.	ID=MALK_01056.t1;Parent=MALK_01056
contig003	AUGUSTUS	exon	465393	467930	.	-	.	ID=MALK_01056.t1.e1;Parent=MALK_01056.t1
contig003	AUGUSTUS	gene	468003	468602	0.76	-	.	ID=MALK_01057;prediction_source=augustus:contig003.g3358.t1
contig003	AUGUSTUS	CDS	468003	468602	0.76	-	0	ID=MALK_01057.t1.c1;Parent=MALK_01057.t1

contig003	AUGUSTUS	mRNA	468003	468602	0.76	-	.	ID=MALK_01057.t1;Parent=MALK_01057
contig003	AUGUSTUS	exon	468003	468602	0.76	-	.	ID=MALK_01057.t1.e1;Parent=MALK_01057.t1
contig003	maker	gene	468698	470692	.	+	.	ID=MALK_01058;prediction_source=maker_MRET:augustus_masked-contig003-processed-gene-4.23-mRNA-1
contig003	maker	CDS	468698	470692	.	+	0	ID=MALK_01058.t1.c1;Parent=MALK_01058.t1
contig003	maker	mRNA	468698	470692	.	+	.	ID=MALK_01058.t1;Parent=MALK_01058
contig003	maker	exon	468698	470692	.	+	.	ID=MALK_01058.t1.e1;Parent=MALK_01058.t1
contig003	AUGUSTUS	gene	470777	473211	0.53	-	.	ID=MALK_01059;prediction_source=braker_MRET:g3101.t1
contig003	AUGUSTUS	CDS	470837	473211	0.53	-	0	ID=MALK_01059.t1.c2;Parent=MALK_01059.t1
contig003	AUGUSTUS	CDS	470777	470795	0.53	-	0	ID=MALK_01059.t1.c1;Parent=MALK_01059.t1
contig003	AUGUSTUS	mRNA	470777	473211	0.53	-	.	ID=MALK_01059.t1;Parent=MALK_01059
contig003	AUGUSTUS	exon	470837	473211	.	-	.	ID=MALK_01059.t1.e2;Parent=MALK_01059.t1
contig003	AUGUSTUS	exon	470777	470795	.	-	.	ID=MALK_01059.t1.e1;Parent=MALK_01059.t1
contig003	AUGUSTUS	gene	473411	473980	0.99	+	.	ID=MALK_01060;prediction_source=braker_MRET:g3102.t1
contig003	AUGUSTUS	CDS	473411	473980	0.99	+	0	ID=MALK_01060.t1.c1;Parent=MALK_01060.t1
contig003	AUGUSTUS	mRNA	473411	473980	0.99	+	.	ID=MALK_01060.t1;Parent=MALK_01060
contig003	AUGUSTUS	exon	473411	473980	.	+	.	ID=MALK_01060.t1.e1;Parent=MALK_01060.t1
contig003	maker	gene	473994	475769	.	-	.	ID=MALK_01061;prediction_source=maker_MRET:augustus_masked-contig003-processed-gene-4.46-mRNA-1
contig003	maker	CDS	473994	475769	.	-	0	ID=MALK_01061.t1.c1;Parent=MALK_01061.t1
contig003	maker	mRNA	473994	475769	.	-	.	ID=MALK_01061.t1;Parent=MALK_01061
contig003	maker	exon	473994	475769	.	-	.	ID=MALK_01061.t1.e1;Parent=MALK_01061.t1
contig003	maker	gene	475882	477129	.	+	.	ID=MALK_01062;prediction_source=maker_MRET:augustus_masked-contig003-processed-gene-4.24-mRNA-1
contig003	maker	CDS	475882	477129	.	+	0	ID=MALK_01062.t1.c1;Parent=MALK_01062.t1
contig003	maker	mRNA	475882	477129	.	+	.	ID=MALK_01062.t1;Parent=MALK_01062
contig003	maker	exon	475882	477129	.	+	.	ID=MALK_01062.t1.e1;Parent=MALK_01062.t1
contig003	AUGUSTUS	gene	477266	477631	0.21	+	.	ID=MALK_01063;prediction_source=augustus:contig003.g3363.t1
contig003	AUGUSTUS	CDS	477266	477274	0.21	+	0	ID=MALK_01063.t1.c1;Parent=MALK_01063.t1
contig003	AUGUSTUS	CDS	477380	477631	0.21	+	0	ID=MALK_01063.t1.c2;Parent=MALK_01063.t1
contig003	AUGUSTUS	mRNA	477266	477631	0.21	+	.	ID=MALK_01063.t1;Parent=MALK_01063
contig003	AUGUSTUS	exon	477266	477274	0.21	+	.	ID=MALK_01063.t1.e1;Parent=MALK_01063.t1
contig003	AUGUSTUS	exon	477380	477631	0.21	+	.	ID=MALK_01063.t1.e2;Parent=MALK_01063.t1
contig003	AUGUSTUS	gene	477884	479317	0.57	+	.	ID=MALK_01064;prediction_source=augustus:contig003.g3364.t1
contig003	AUGUSTUS	CDS	477884	479317	0.57	+	0	ID=MALK_01064.t1.c1;Parent=MALK_01064.t1
contig003	AUGUSTUS	mRNA	477884	479317	0.57	+	.	ID=MALK_01064.t1;Parent=MALK_01064
contig003	AUGUSTUS	exon	477884	479317	0.57	+	.	ID=MALK_01064.t1.e1;Parent=MALK_01064.t1
contig003	maker	gene	479314	480705	.	-	.	ID=MALK_01065;prediction_source=maker_MRET:augustus_masked-contig003-processed-gene-4.47-mRNA-1
contig003	maker	CDS	479314	480705	.	-	0	ID=MALK_01065.t1.c1;Parent=MALK_01065.t1
contig003	maker	mRNA	479314	480705	.	-	.	ID=MALK_01065.t1;Parent=MALK_01065
contig003	maker	exon	479314	480705	.	-	.	ID=MALK_01065.t1.e1;Parent=MALK_01065.t1
contig003	AUGUSTUS	gene	480748	482879	0.84	+	.	ID=MALK_01066;prediction_source=augustus:contig003.g3366.t1
contig003	AUGUSTUS	CDS	480748	482067	0.89	+	0	ID=MALK_01066.t1.c1;Parent=MALK_01066.t1
contig003	AUGUSTUS	CDS	482160	482879	0.89	+	0	ID=MALK_01066.t1.c2;Parent=MALK_01066.t1
contig003	AUGUSTUS	mRNA	480748	482879	0.84	+	.	ID=MALK_01066.t1;Parent=MALK_01066
contig003	AUGUSTUS	exon	480748	482067	0.89	+	.	ID=MALK_01066.t1.e1;Parent=MALK_01066.t1

contig003	AUGUSTUS	exon	482160	482879	0.89	+	.	ID=MALK_01066.t1.e2;Parent=MALK_01066.t1
contig003	maker	gene	483222	488273	.	+	.	ID=MALK_01067;prediction_source=maker_MRET:augustus_masked-contig003-processed-gene-4.27-mRNA-1
contig003	maker	CDS	483222	488273	.	+	0	ID=MALK_01067.t1.c1;Parent=MALK_01067.t1
contig003	maker	mRNA	483222	488273	.	+	.	ID=MALK_01067.t1;Parent=MALK_01067
contig003	maker	exon	483222	488273	.	+	.	ID=MALK_01067.t1.e1;Parent=MALK_01067.t1
contig003	AUGUSTUS	gene	488349	489721	0.39	+	.	ID=MALK_01068;prediction_source=augustus:contig003.g3371.t1
contig003	AUGUSTUS	CDS	488349	488481	0.52	+	0	ID=MALK_01068.t1.c1;Parent=MALK_01068.t1
contig003	AUGUSTUS	CDS	488556	489721	0.52	+	0	ID=MALK_01068.t1.c2;Parent=MALK_01068.t1
contig003	AUGUSTUS	mRNA	488349	489721	0.39	+	.	ID=MALK_01068.t1;Parent=MALK_01068
contig003	AUGUSTUS	exon	488349	488481	0.52	+	.	ID=MALK_01068.t1.e1;Parent=MALK_01068.t1
contig003	AUGUSTUS	exon	488556	489721	0.52	+	.	ID=MALK_01068.t1.e2;Parent=MALK_01068.t1
contig003	AUGUSTUS	gene	489785	490936	0.45	+	.	ID=MALK_01069;prediction_source=augustus:contig003.g3373.t1
contig003	AUGUSTUS	CDS	489785	490936	0.45	+	0	ID=MALK_01069.t1.c1;Parent=MALK_01069.t1
contig003	AUGUSTUS	mRNA	489785	490936	0.45	+	.	ID=MALK_01069.t1;Parent=MALK_01069
contig003	AUGUSTUS	exon	489785	490936	0.45	+	.	ID=MALK_01069.t1.e1;Parent=MALK_01069.t1
contig003	AUGUSTUS	gene	491011	492048	0.56	+	.	ID=MALK_01070;prediction_source=augustus:contig003.g3374.t1
contig003	AUGUSTUS	CDS	491011	492048	0.56	+	0	ID=MALK_01070.t1.c1;Parent=MALK_01070.t1
contig003	AUGUSTUS	mRNA	491011	492048	0.56	+	.	ID=MALK_01070.t1;Parent=MALK_01070
contig003	AUGUSTUS	exon	491011	492048	0.56	+	.	ID=MALK_01070.t1.e1;Parent=MALK_01070.t1
contig003	AUGUSTUS	gene	492182	495025	0.34	-	.	ID=MALK_01071;prediction_source=augustus:contig003.g3375.t1
contig003	AUGUSTUS	CDS	493108	495025	0.34	-	0	ID=MALK_01071.t1.c2;Parent=MALK_01071.t1
contig003	AUGUSTUS	CDS	492182	492717	0.34	-	0	ID=MALK_01071.t1.c1;Parent=MALK_01071.t1
contig003	AUGUSTUS	mRNA	492182	495025	0.34	-	.	ID=MALK_01071.t1;Parent=MALK_01071
contig003	AUGUSTUS	exon	493108	495025	0.34	-	.	ID=MALK_01071.t1.e2;Parent=MALK_01071.t1
contig003	AUGUSTUS	exon	492182	492717	0.34	-	.	ID=MALK_01071.t1.e1;Parent=MALK_01071.t1
contig003	AUGUSTUS	gene	496227	497521	0.34	-	.	ID=MALK_01072;prediction_source=braker_MRET:g3112.t1
contig003	AUGUSTUS	CDS	496891	497521	0.49	-	0	ID=MALK_01072.t1.c2;Parent=MALK_01072.t1
contig003	AUGUSTUS	CDS	496227	496801	0.49	-	0	ID=MALK_01072.t1.c1;Parent=MALK_01072.t1
contig003	AUGUSTUS	mRNA	496227	497521	0.34	-	.	ID=MALK_01072.t1;Parent=MALK_01072
contig003	AUGUSTUS	exon	496891	497521	.	-	.	ID=MALK_01072.t1.e2;Parent=MALK_01072.t1
contig003	AUGUSTUS	exon	496227	496801	.	-	.	ID=MALK_01072.t1.e1;Parent=MALK_01072.t1
contig003	maker	gene	497607	498611	.	-	.	ID=MALK_01073;prediction_source=maker_MRET:augustus_masked-contig003-processed-gene-5.3-mRNA-1
contig003	maker	CDS	497607	498611	.	-	0	ID=MALK_01073.t1.c1;Parent=MALK_01073.t1
contig003	maker	mRNA	497607	498611	.	-	.	ID=MALK_01073.t1;Parent=MALK_01073
contig003	maker	exon	497607	498611	.	-	.	ID=MALK_01073.t1.e1;Parent=MALK_01073.t1
contig003	maker	gene	498728	500452	.	+	.	ID=MALK_01074;prediction_source=maker_MRET:augustus_masked-contig003-processed-gene-5.0-mRNA-1
contig003	maker	CDS	498728	500452	.	+	0	ID=MALK_01074.t1.c1;Parent=MALK_01074.t1
contig003	maker	mRNA	498728	500452	.	+	.	ID=MALK_01074.t1;Parent=MALK_01074
contig003	maker	exon	498728	500452	.	+	.	ID=MALK_01074.t1.e1;Parent=MALK_01074.t1
contig003	maker	gene	500506	502338	.	-	.	ID=MALK_01075;prediction_source=maker_MRET:augustus_masked-contig003-processed-gene-5.30-mRNA-1
contig003	maker	CDS	500506	502338	.	-	0	ID=MALK_01075.t1.c1;Parent=MALK_01075.t1
contig003	maker	mRNA	500506	502338	.	-	.	ID=MALK_01075.t1;Parent=MALK_01075
contig003	maker	exon	500506	502338	.	-	.	ID=MALK_01075.t1.e1;Parent=MALK_01075.t1

contig003	maker	gene	502685	504754	.	-	.	ID=MALK_01076;prediction_source=maker_MRET:augustus_masked-contig003-processed-gene-5.31-mRNA-1
contig003	maker	CDS	502685	504754	.	-	0	ID=MALK_01076.t1.c1;Parent=MALK_01076.t1
contig003	maker	mRNA	502685	504754	.	-	.	ID=MALK_01076.t1;Parent=MALK_01076
contig003	maker	exon	502685	504754	.	-	.	ID=MALK_01076.t1.e1;Parent=MALK_01076.t1
contig003	maker	gene	505050	507545	.	-	.	ID=MALK_01077;prediction_source=maker_MRET:augustus_masked-contig003-processed-gene-5.32-mRNA-1
contig003	maker	CDS	505050	507545	.	-	0	ID=MALK_01077.t1.c1;Parent=MALK_01077.t1
contig003	maker	mRNA	505050	507545	.	-	.	ID=MALK_01077.t1;Parent=MALK_01077
contig003	maker	exon	505050	507545	.	-	.	ID=MALK_01077.t1.e1;Parent=MALK_01077.t1
contig003	AUGUSTUS	gene	507794	509160	0.21	-	.	ID=MALK_01078;prediction_source=braker_MRET:g3118.t1
contig003	AUGUSTUS	CDS	509148	509160	1	-	0	ID=MALK_01078.t1.c6;Parent=MALK_01078.t1
contig003	AUGUSTUS	CDS	508448	509112	1	-	0	ID=MALK_01078.t1.c5;Parent=MALK_01078.t1
contig003	AUGUSTUS	CDS	508132	508344	1	-	0	ID=MALK_01078.t1.c4;Parent=MALK_01078.t1
contig003	AUGUSTUS	CDS	508045	508094	1	-	0	ID=MALK_01078.t1.c3;Parent=MALK_01078.t1
contig003	AUGUSTUS	CDS	507930	508013	1	-	0	ID=MALK_01078.t1.c2;Parent=MALK_01078.t1
contig003	AUGUSTUS	CDS	507794	507890	1	-	0	ID=MALK_01078.t1.c1;Parent=MALK_01078.t1
contig003	AUGUSTUS	mRNA	507794	509160	0.21	-	.	ID=MALK_01078.t1;Parent=MALK_01078
contig003	AUGUSTUS	exon	509148	509160	.	-	.	ID=MALK_01078.t1.e6;Parent=MALK_01078.t1
contig003	AUGUSTUS	exon	508448	509112	.	-	.	ID=MALK_01078.t1.e5;Parent=MALK_01078.t1
contig003	AUGUSTUS	exon	508132	508344	.	-	.	ID=MALK_01078.t1.e4;Parent=MALK_01078.t1
contig003	AUGUSTUS	exon	508045	508094	.	-	.	ID=MALK_01078.t1.e3;Parent=MALK_01078.t1
contig003	AUGUSTUS	exon	507930	508013	.	-	.	ID=MALK_01078.t1.e2;Parent=MALK_01078.t1
contig003	AUGUSTUS	exon	507794	507890	.	-	.	ID=MALK_01078.t1.e1;Parent=MALK_01078.t1
contig003	AUGUSTUS	gene	509261	511339	0.86	+	.	ID=MALK_01079;prediction_source=braker_MRET:g3119.t1
contig003	AUGUSTUS	CDS	509261	511339	0.86	+	0	ID=MALK_01079.t1.c1;Parent=MALK_01079.t1
contig003	AUGUSTUS	mRNA	509261	511339	0.86	+	.	ID=MALK_01079.t1;Parent=MALK_01079
contig003	AUGUSTUS	exon	509261	511339	.	+	.	ID=MALK_01079.t1.e1;Parent=MALK_01079.t1
contig003	AUGUSTUS	gene	511340	512887	0.94	-	.	ID=MALK_01080;prediction_source=augustus:contig003.g3388.t1
contig003	AUGUSTUS	CDS	511340	512887	0.94	-	0	ID=MALK_01080.t1.c1;Parent=MALK_01080.t1
contig003	AUGUSTUS	mRNA	511340	512887	0.94	-	.	ID=MALK_01080.t1;Parent=MALK_01080
contig003	AUGUSTUS	exon	511340	512887	0.94	-	.	ID=MALK_01080.t1.e1;Parent=MALK_01080.t1
contig003	AUGUSTUS	gene	513134	514204	0.61	+	.	ID=MALK_01081;prediction_source=augustus:contig003.g3389.t1
contig003	AUGUSTUS	CDS	513134	514204	0.61	+	0	ID=MALK_01081.t1.c1;Parent=MALK_01081.t1
contig003	AUGUSTUS	mRNA	513134	514204	0.61	+	.	ID=MALK_01081.t1;Parent=MALK_01081
contig003	AUGUSTUS	exon	513134	514204	0.61	+	.	ID=MALK_01081.t1.e1;Parent=MALK_01081.t1
contig003	AUGUSTUS	gene	514416	515021	0.93	-	.	ID=MALK_01082;prediction_source=augustus:contig003.g3390.t1
contig003	AUGUSTUS	CDS	514416	515021	0.93	-	0	ID=MALK_01082.t1.c1;Parent=MALK_01082.t1
contig003	AUGUSTUS	mRNA	514416	515021	0.93	-	.	ID=MALK_01082.t1;Parent=MALK_01082
contig003	AUGUSTUS	exon	514416	515021	0.93	-	.	ID=MALK_01082.t1.e1;Parent=MALK_01082.t1
contig003	maker	gene	516065	516268	.	+	.	ID=MALK_01083;prediction_source=maker_MRET:augustus_masked-contig003-processed-gene-5.6-mRNA-1
contig003	maker	CDS	516065	516268	.	+	0	ID=MALK_01083.t1.c1;Parent=MALK_01083.t1
contig003	maker	mRNA	516065	516268	.	+	.	ID=MALK_01083.t1;Parent=MALK_01083
contig003	maker	exon	516065	516268	.	+	.	ID=MALK_01083.t1.e1;Parent=MALK_01083.t1
contig003	maker	gene	516856	518478	.	-	.	ID=MALK_01084;prediction_source=maker_MRET:augustus_masked-contig003-processed-gene-5.36-mRNA-1

contig003	maker	CDS	516856	518478	.	-	0	ID=MALK_01084.t1.c1;Parent=MALK_01084.t1
contig003	maker	mRNA	516856	518478	.	-	.	ID=MALK_01084.t1;Parent=MALK_01084
contig003	maker	exon	516856	518478	.	-	.	ID=MALK_01084.t1.e1;Parent=MALK_01084.t1
contig003	maker	gene	518704	522471	.	+	.	ID=MALK_01085;prediction_source=maker_MRET:augustus_masked-contig003-processed-gene-5.7-mRNA-1
contig003	maker	CDS	518704	522471	.	+	0	ID=MALK_01085.t1.c1;Parent=MALK_01085.t1
contig003	maker	mRNA	518704	522471	.	+	.	ID=MALK_01085.t1;Parent=MALK_01085
contig003	maker	exon	518704	522471	.	+	.	ID=MALK_01085.t1.e1;Parent=MALK_01085.t1
contig003	AUGUSTUS	gene	522468	524957	0.38	-	.	ID=MALK_01086;prediction_source=augustus:contig003.g3394.t1
contig003	AUGUSTUS	CDS	522468	524957	0.38	-	0	ID=MALK_01086.t1.c1;Parent=MALK_01086.t1
contig003	AUGUSTUS	mRNA	522468	524957	0.38	-	.	ID=MALK_01086.t1;Parent=MALK_01086
contig003	AUGUSTUS	exon	522468	524957	0.38	-	.	ID=MALK_01086.t1.e1;Parent=MALK_01086.t1
contig003	AUGUSTUS	gene	525609	526914	0.47	+	.	ID=MALK_01087;prediction_source=braker_MRET:g3126.t1
contig003	AUGUSTUS	CDS	525609	526029	0.85	+	0	ID=MALK_01087.t1.c1;Parent=MALK_01087.t1
contig003	AUGUSTUS	CDS	526073	526914	0.85	+	0	ID=MALK_01087.t1.c2;Parent=MALK_01087.t1
contig003	AUGUSTUS	mRNA	525609	526914	0.47	+	.	ID=MALK_01087.t1;Parent=MALK_01087
contig003	AUGUSTUS	exon	525609	526029	.	+	.	ID=MALK_01087.t1.e1;Parent=MALK_01087.t1
contig003	AUGUSTUS	exon	526073	526914	.	+	.	ID=MALK_01087.t1.e2;Parent=MALK_01087.t1
contig003	AUGUSTUS	gene	527181	528143	0.47	+	.	ID=MALK_01088;prediction_source=augustus:contig003.g3396.t1
contig003	AUGUSTUS	CDS	527181	528143	0.47	+	0	ID=MALK_01088.t1.c1;Parent=MALK_01088.t1
contig003	AUGUSTUS	mRNA	527181	528143	0.47	+	.	ID=MALK_01088.t1;Parent=MALK_01088
contig003	AUGUSTUS	exon	527181	528143	0.47	+	.	ID=MALK_01088.t1.e1;Parent=MALK_01088.t1
contig003	AUGUSTUS	gene	528306	529178	0.66	-	.	ID=MALK_01089;prediction_source=braker_MRET:g3128.t1
contig003	AUGUSTUS	CDS	529119	529178	0.66	-	0	ID=MALK_01089.t1.c2;Parent=MALK_01089.t1
contig003	AUGUSTUS	CDS	528306	529085	0.66	-	0	ID=MALK_01089.t1.c1;Parent=MALK_01089.t1
contig003	AUGUSTUS	mRNA	528306	529178	0.66	-	.	ID=MALK_01089.t1;Parent=MALK_01089
contig003	AUGUSTUS	exon	529119	529178	.	-	.	ID=MALK_01089.t1.e2;Parent=MALK_01089.t1
contig003	AUGUSTUS	exon	528306	529085	.	-	.	ID=MALK_01089.t1.e1;Parent=MALK_01089.t1
contig003	AUGUSTUS	gene	529444	532221	0.95	-	.	ID=MALK_01090;prediction_source=braker_MRET:g3129.t1
contig003	AUGUSTUS	CDS	529444	532221	0.95	-	0	ID=MALK_01090.t1.c1;Parent=MALK_01090.t1
contig003	AUGUSTUS	mRNA	529444	532221	0.95	-	.	ID=MALK_01090.t1;Parent=MALK_01090
contig003	AUGUSTUS	exon	529444	532221	.	-	.	ID=MALK_01090.t1.e1;Parent=MALK_01090.t1
contig003	AUGUSTUS	gene	532816	534741	1	+	.	ID=MALK_01091;prediction_source=braker_MRET:g3130.t1
contig003	AUGUSTUS	CDS	532816	534741	1	+	0	ID=MALK_01091.t1.c1;Parent=MALK_01091.t1
contig003	AUGUSTUS	mRNA	532816	534741	1	+	.	ID=MALK_01091.t1;Parent=MALK_01091
contig003	AUGUSTUS	exon	532816	534741	.	+	.	ID=MALK_01091.t1.e1;Parent=MALK_01091.t1
contig003	AUGUSTUS	gene	534831	535187	1	-	.	ID=MALK_01092;prediction_source=braker_MRET:g3131.t1
contig003	AUGUSTUS	CDS	535086	535187	1	-	0	ID=MALK_01092.t1.c4;Parent=MALK_01092.t1
contig003	AUGUSTUS	CDS	535035	535038	1	-	0	ID=MALK_01092.t1.c3;Parent=MALK_01092.t1
contig003	AUGUSTUS	CDS	534882	534987	1	-	0	ID=MALK_01092.t1.c2;Parent=MALK_01092.t1
contig003	AUGUSTUS	CDS	534831	534843	1	-	0	ID=MALK_01092.t1.c1;Parent=MALK_01092.t1
contig003	AUGUSTUS	mRNA	534831	535187	1	-	.	ID=MALK_01092.t1;Parent=MALK_01092
contig003	AUGUSTUS	exon	535086	535187	.	-	.	ID=MALK_01092.t1.e4;Parent=MALK_01092.t1
contig003	AUGUSTUS	exon	535035	535038	.	-	.	ID=MALK_01092.t1.e3;Parent=MALK_01092.t1

contig003	AUGUSTUS	exon	534882	534987	.	-	.	ID=MALK_01092.t1.e2;Parent=MALK_01092.t1
contig003	AUGUSTUS	exon	534831	534843	.	-	.	ID=MALK_01092.t1.e1;Parent=MALK_01092.t1
contig003	AUGUSTUS	gene	535317	536615	0.89	+	.	ID=MALK_01093;prediction_source=augustus:contig003.g3400.t1
contig003	AUGUSTUS	CDS	535317	536615	0.89	+	0	ID=MALK_01093.t1.c1;Parent=MALK_01093.t1
contig003	AUGUSTUS	mRNA	535317	536615	0.89	+	.	ID=MALK_01093.t1;Parent=MALK_01093
contig003	AUGUSTUS	exon	535317	536615	0.89	+	.	ID=MALK_01093.t1.e1;Parent=MALK_01093.t1
contig003	AUGUSTUS	gene	536627	538952	0.83	-	.	ID=MALK_01094;prediction_source=braker_MRET:g3133.t1
contig003	AUGUSTUS	CDS	538693	538952	0.84	-	0	ID=MALK_01094.t1.c2;Parent=MALK_01094.t1
contig003	AUGUSTUS	CDS	536627	538664	0.84	-	0	ID=MALK_01094.t1.c1;Parent=MALK_01094.t1
contig003	AUGUSTUS	mRNA	536627	538952	0.83	-	.	ID=MALK_01094.t1;Parent=MALK_01094
contig003	AUGUSTUS	exon	538693	538952	.	-	.	ID=MALK_01094.t1.e2;Parent=MALK_01094.t1
contig003	AUGUSTUS	exon	536627	538664	.	-	.	ID=MALK_01094.t1.e1;Parent=MALK_01094.t1
contig003	maker	gene	539068	540246	.	+	.	ID=MALK_01095;prediction_source=maker_MRET:augustus_masked-contig003-processed-gene-5.12-mRNA-1
contig003	maker	CDS	539068	540246	.	+	0	ID=MALK_01095.t1.c1;Parent=MALK_01095.t1
contig003	maker	mRNA	539068	540246	.	+	.	ID=MALK_01095.t1;Parent=MALK_01095
contig003	maker	exon	539068	540246	.	+	.	ID=MALK_01095.t1.e1;Parent=MALK_01095.t1
contig003	AUGUSTUS	gene	540299	541333	0.99	-	.	ID=MALK_01096;prediction_source=braker_MRET:g3135.t1
contig003	AUGUSTUS	CDS	541268	541333	1	-	0	ID=MALK_01096.t1.c2;Parent=MALK_01096.t1
contig003	AUGUSTUS	CDS	540299	541231	1	-	0	ID=MALK_01096.t1.c1;Parent=MALK_01096.t1
contig003	AUGUSTUS	mRNA	540299	541333	0.99	-	.	ID=MALK_01096.t1;Parent=MALK_01096
contig003	AUGUSTUS	exon	541268	541333	.	-	.	ID=MALK_01096.t1.e2;Parent=MALK_01096.t1
contig003	AUGUSTUS	exon	540299	541231	.	-	.	ID=MALK_01096.t1.e1;Parent=MALK_01096.t1
contig003	AUGUSTUS	gene	541558	544761	0.38	-	.	ID=MALK_01097;prediction_source=braker_MRET:g3136.t1
contig003	AUGUSTUS	CDS	541558	544761	0.38	-	0	ID=MALK_01097.t1.c1;Parent=MALK_01097.t1
contig003	AUGUSTUS	mRNA	541558	544761	0.38	-	.	ID=MALK_01097.t1;Parent=MALK_01097
contig003	AUGUSTUS	exon	541558	544761	.	-	.	ID=MALK_01097.t1.e1;Parent=MALK_01097.t1
contig003	AUGUSTUS	gene	544959	545263	0.41	-	.	ID=MALK_01098;prediction_source=braker_MRET:g3137.t1
contig003	AUGUSTUS	CDS	545255	545263	0.43	-	0	ID=MALK_01098.t1.c2;Parent=MALK_01098.t1
contig003	AUGUSTUS	CDS	544959	545219	0.43	-	0	ID=MALK_01098.t1.c1;Parent=MALK_01098.t1
contig003	AUGUSTUS	mRNA	544959	545263	0.41	-	.	ID=MALK_01098.t1;Parent=MALK_01098
contig003	AUGUSTUS	exon	545255	545263	.	-	.	ID=MALK_01098.t1.e2;Parent=MALK_01098.t1
contig003	AUGUSTUS	exon	544959	545219	.	-	.	ID=MALK_01098.t1.e1;Parent=MALK_01098.t1
contig003	AUGUSTUS	gene	545601	546236	0.83	+	.	ID=MALK_01099;prediction_source=augustus:contig003.g3404.t1
contig003	AUGUSTUS	CDS	545601	546236	0.83	+	0	ID=MALK_01099.t1.c1;Parent=MALK_01099.t1
contig003	AUGUSTUS	mRNA	545601	546236	0.83	+	.	ID=MALK_01099.t1;Parent=MALK_01099
contig003	AUGUSTUS	exon	545601	546236	0.83	+	.	ID=MALK_01099.t1.e1;Parent=MALK_01099.t1
contig003	AUGUSTUS	gene	546499	548457	0.72	-	.	ID=MALK_01100;prediction_source=braker_MRET:g3139.t1
contig003	AUGUSTUS	CDS	546499	548457	0.72	-	0	ID=MALK_01100.t1.c1;Parent=MALK_01100.t1
contig003	AUGUSTUS	mRNA	546499	548457	0.72	-	.	ID=MALK_01100.t1;Parent=MALK_01100
contig003	AUGUSTUS	exon	546499	548457	.	-	.	ID=MALK_01100.t1.e1;Parent=MALK_01100.t1
contig003	maker	gene	548657	550447	.	+	.	ID=MALK_01101;prediction_source=maker_MRET:augustus_masked-contig003-processed-gene-5.14-mRNA-1
contig003	maker	CDS	548657	550447	.	+	0	ID=MALK_01101.t1.c1;Parent=MALK_01101.t1
contig003	maker	mRNA	548657	550447	.	+	.	ID=MALK_01101.t1;Parent=MALK_01101

contig003	maker	exon	548657	550447	.	+	.	ID=MALK_01101.t1.e1;Parent=MALK_01101.t1
contig003	AUGUSTUS	gene	550815	552224	0.77	+	.	ID=MALK_01102;prediction_source=augustus:contig003.g3407.t1
contig003	AUGUSTUS	CDS	550815	552224	0.77	+	0	ID=MALK_01102.t1.c1;Parent=MALK_01102.t1
contig003	AUGUSTUS	mRNA	550815	552224	0.77	+	.	ID=MALK_01102.t1;Parent=MALK_01102
contig003	AUGUSTUS	exon	550815	552224	0.77	+	.	ID=MALK_01102.t1.e1;Parent=MALK_01102.t1
contig003	AUGUSTUS	gene	552265	553296	1	-	.	ID=MALK_01103;prediction_source=braker_MRET:g3142.t1
contig003	AUGUSTUS	CDS	552265	553296	1	-	0	ID=MALK_01103.t1.c1;Parent=MALK_01103.t1
contig003	AUGUSTUS	mRNA	552265	553296	1	-	.	ID=MALK_01103.t1;Parent=MALK_01103
contig003	AUGUSTUS	exon	552265	553296	.	-	.	ID=MALK_01103.t1.e1;Parent=MALK_01103.t1
contig003	AUGUSTUS	gene	553391	555223	0.68	+	.	ID=MALK_01104;prediction_source=augustus:contig003.g3409.t1
contig003	AUGUSTUS	CDS	553391	555223	0.68	+	0	ID=MALK_01104.t1.c1;Parent=MALK_01104.t1
contig003	AUGUSTUS	mRNA	553391	555223	0.68	+	.	ID=MALK_01104.t1;Parent=MALK_01104
contig003	AUGUSTUS	exon	553391	555223	0.68	+	.	ID=MALK_01104.t1.e1;Parent=MALK_01104.t1
contig003	maker	gene	555241	556632	.	-	.	ID=MALK_01105;prediction_source=maker_MRET:augustus_masked-contig003-processed-gene-5.43-mRNA-1
contig003	maker	CDS	555241	556632	.	-	0	ID=MALK_01105.t1.c1;Parent=MALK_01105.t1
contig003	maker	mRNA	555241	556632	.	-	.	ID=MALK_01105.t1;Parent=MALK_01105
contig003	maker	exon	555241	556632	.	-	.	ID=MALK_01105.t1.e1;Parent=MALK_01105.t1
contig003	AUGUSTUS	gene	556807	559155	0.99	-	.	ID=MALK_01106;prediction_source=augustus:contig003.g3411.t1
contig003	AUGUSTUS	CDS	556807	559155	0.99	-	0	ID=MALK_01106.t1.c1;Parent=MALK_01106.t1
contig003	AUGUSTUS	mRNA	556807	559155	0.99	-	.	ID=MALK_01106.t1;Parent=MALK_01106
contig003	AUGUSTUS	exon	556807	559155	0.99	-	.	ID=MALK_01106.t1.e1;Parent=MALK_01106.t1
contig003	AUGUSTUS	gene	559215	560199	0.98	-	.	ID=MALK_01107;prediction_source=braker_MRET:g3146.t1
contig003	AUGUSTUS	CDS	559874	560199	0.98	-	0	ID=MALK_01107.t1.c3;Parent=MALK_01107.t1
contig003	AUGUSTUS	CDS	559742	559841	0.98	-	0	ID=MALK_01107.t1.c2;Parent=MALK_01107.t1
contig003	AUGUSTUS	CDS	559215	559709	0.98	-	0	ID=MALK_01107.t1.c1;Parent=MALK_01107.t1
contig003	AUGUSTUS	mRNA	559215	560199	0.98	-	.	ID=MALK_01107.t1;Parent=MALK_01107
contig003	AUGUSTUS	exon	559874	560199	.	-	.	ID=MALK_01107.t1.e3;Parent=MALK_01107.t1
contig003	AUGUSTUS	exon	559742	559841	.	-	.	ID=MALK_01107.t1.e2;Parent=MALK_01107.t1
contig003	AUGUSTUS	exon	559215	559709	.	-	.	ID=MALK_01107.t1.e1;Parent=MALK_01107.t1
contig003	AUGUSTUS	gene	560491	561917	0.21	+	.	ID=MALK_01108;prediction_source=braker_MRET:g3147.t1
contig003	AUGUSTUS	CDS	560491	560545	0.75	+	0	ID=MALK_01108.t1.c1;Parent=MALK_01108.t1
contig003	AUGUSTUS	CDS	560577	560707	0.75	+	0	ID=MALK_01108.t1.c2;Parent=MALK_01108.t1
contig003	AUGUSTUS	CDS	560751	560885	0.75	+	0	ID=MALK_01108.t1.c3;Parent=MALK_01108.t1
contig003	AUGUSTUS	CDS	561013	561239	0.75	+	0	ID=MALK_01108.t1.c4;Parent=MALK_01108.t1
contig003	AUGUSTUS	CDS	561343	561461	0.75	+	0	ID=MALK_01108.t1.c5;Parent=MALK_01108.t1
contig003	AUGUSTUS	CDS	561502	561917	0.75	+	0	ID=MALK_01108.t1.c6;Parent=MALK_01108.t1
contig003	AUGUSTUS	mRNA	560491	561917	0.21	+	.	ID=MALK_01108.t1;Parent=MALK_01108
contig003	AUGUSTUS	exon	560491	560545	.	+	.	ID=MALK_01108.t1.e1;Parent=MALK_01108.t1
contig003	AUGUSTUS	exon	560577	560707	.	+	.	ID=MALK_01108.t1.e2;Parent=MALK_01108.t1
contig003	AUGUSTUS	exon	560751	560885	.	+	.	ID=MALK_01108.t1.e3;Parent=MALK_01108.t1
contig003	AUGUSTUS	exon	561013	561239	.	+	.	ID=MALK_01108.t1.e4;Parent=MALK_01108.t1
contig003	AUGUSTUS	exon	561343	561461	.	+	.	ID=MALK_01108.t1.e5;Parent=MALK_01108.t1
contig003	AUGUSTUS	exon	561502	561917	.	+	.	ID=MALK_01108.t1.e6;Parent=MALK_01108.t1

contig003	AUGUSTUS	gene	562536	563198	0.83	+	.	ID=MALK_01109;prediction_source=braker_MRET:g3148.t1
contig003	AUGUSTUS	CDS	562536	563198	0.83	+	0	ID=MALK_01109.t1.c1;Parent=MALK_01109.t1
contig003	AUGUSTUS	mRNA	562536	563198	0.83	+	.	ID=MALK_01109.t1;Parent=MALK_01109
contig003	AUGUSTUS	exon	562536	563198	.	+	.	ID=MALK_01109.t1.e1;Parent=MALK_01109.t1
contig003	AUGUSTUS	gene	563364	566171	1	+	.	ID=MALK_01110;prediction_source=augustus:contig003.g3417.t1
contig003	AUGUSTUS	CDS	563364	566171	1	+	0	ID=MALK_01110.t1.c1;Parent=MALK_01110.t1
contig003	AUGUSTUS	mRNA	563364	566171	1	+	.	ID=MALK_01110.t1;Parent=MALK_01110
contig003	AUGUSTUS	exon	563364	566171	1	+	.	ID=MALK_01110.t1.e1;Parent=MALK_01110.t1
contig003	AUGUSTUS	gene	566186	568384	0.73	-	.	ID=MALK_01111;prediction_source=augustus:contig003.g3418.t1
contig003	AUGUSTUS	CDS	566186	568384	0.73	-	0	ID=MALK_01111.t1.c1;Parent=MALK_01111.t1
contig003	AUGUSTUS	mRNA	566186	568384	0.73	-	.	ID=MALK_01111.t1;Parent=MALK_01111
contig003	AUGUSTUS	exon	566186	568384	0.73	-	.	ID=MALK_01111.t1.e1;Parent=MALK_01111.t1
contig003	AUGUSTUS	gene	569184	570839	0.99	-	.	ID=MALK_01112;prediction_source=augustus:contig003.g3419.t1
contig003	AUGUSTUS	CDS	569184	570839	0.99	-	0	ID=MALK_01112.t1.c1;Parent=MALK_01112.t1
contig003	AUGUSTUS	mRNA	569184	570839	0.99	-	.	ID=MALK_01112.t1;Parent=MALK_01112
contig003	AUGUSTUS	exon	569184	570839	0.99	-	.	ID=MALK_01112.t1.e1;Parent=MALK_01112.t1
contig003	AUGUSTUS	gene	570908	571434	0.19	+	.	ID=MALK_01113;prediction_source=braker_MRET:g3152.t1
contig003	AUGUSTUS	CDS	570908	571372	0.25	+	0	ID=MALK_01113.t1.c1;Parent=MALK_01113.t1
contig003	AUGUSTUS	CDS	571408	571434	0.25	+	0	ID=MALK_01113.t1.c2;Parent=MALK_01113.t1
contig003	AUGUSTUS	mRNA	570908	571434	0.19	+	.	ID=MALK_01113.t1;Parent=MALK_01113
contig003	AUGUSTUS	exon	570908	571372	.	+	.	ID=MALK_01113.t1.e1;Parent=MALK_01113.t1
contig003	AUGUSTUS	exon	571408	571434	.	+	.	ID=MALK_01113.t1.e2;Parent=MALK_01113.t1
contig003	AUGUSTUS	gene	571666	572670	1	-	.	ID=MALK_01114;prediction_source=braker_MRET:g3153.t1
contig003	AUGUSTUS	CDS	571666	572670	1	-	0	ID=MALK_01114.t1.c1;Parent=MALK_01114.t1
contig003	AUGUSTUS	mRNA	571666	572670	1	-	.	ID=MALK_01114.t1;Parent=MALK_01114
contig003	AUGUSTUS	exon	571666	572670	.	-	.	ID=MALK_01114.t1.e1;Parent=MALK_01114.t1
contig003	AUGUSTUS	gene	572697	573648	0.94	+	.	ID=MALK_01115;prediction_source=braker_MRET:g3154.t1
contig003	AUGUSTUS	CDS	572697	573580	0.97	+	0	ID=MALK_01115.t1.c1;Parent=MALK_01115.t1
contig003	AUGUSTUS	CDS	573633	573648	0.97	+	0	ID=MALK_01115.t1.c2;Parent=MALK_01115.t1
contig003	AUGUSTUS	mRNA	572697	573648	0.94	+	.	ID=MALK_01115.t1;Parent=MALK_01115
contig003	AUGUSTUS	exon	572697	573580	.	+	.	ID=MALK_01115.t1.e1;Parent=MALK_01115.t1
contig003	AUGUSTUS	exon	573633	573648	.	+	.	ID=MALK_01115.t1.e2;Parent=MALK_01115.t1
contig003	AUGUSTUS	gene	573653	574201	0.42	-	.	ID=MALK_01116;prediction_source=augustus:contig003.g3421.t1
contig003	AUGUSTUS	CDS	573653	574201	0.42	-	0	ID=MALK_01116.t1.c1;Parent=MALK_01116.t1
contig003	AUGUSTUS	mRNA	573653	574201	0.42	-	.	ID=MALK_01116.t1;Parent=MALK_01116
contig003	AUGUSTUS	exon	573653	574201	0.42	-	.	ID=MALK_01116.t1.e1;Parent=MALK_01116.t1
contig003	maker	gene	574228	577953	.	-	.	ID=MALK_01117;prediction_source=maker_MRET:augustus_masked-contig003-processed-gene-5.49-mRNA-1
contig003	maker	CDS	574228	577953	.	-	0	ID=MALK_01117.t1.c1;Parent=MALK_01117.t1
contig003	maker	mRNA	574228	577953	.	-	.	ID=MALK_01117.t1;Parent=MALK_01117
contig003	maker	exon	574228	577953	.	-	.	ID=MALK_01117.t1.e1;Parent=MALK_01117.t1
contig003	AUGUSTUS	gene	578052	579137	1	+	.	ID=MALK_01118;prediction_source=braker_MRET:g3157.t1
contig003	AUGUSTUS	CDS	578052	579137	1	+	0	ID=MALK_01118.t1.c1;Parent=MALK_01118.t1
contig003	AUGUSTUS	mRNA	578052	579137	1	+	.	ID=MALK_01118.t1;Parent=MALK_01118

contig003	AUGUSTUS	exon	578052	579137	.	+	.	ID=MALK_01118.t1.e1;Parent=MALK_01118.t1
contig003	AUGUSTUS	gene	579134	579830	0.33	-	.	ID=MALK_01119;prediction_source=augustus:contig003.g3424.t1
contig003	AUGUSTUS	CDS	579797	579830	0.6	-	0	ID=MALK_01119.t1.c2;Parent=MALK_01119.t1
contig003	AUGUSTUS	CDS	579134	579672	0.6	-	0	ID=MALK_01119.t1.c1;Parent=MALK_01119.t1
contig003	AUGUSTUS	mRNA	579134	579830	0.33	-	.	ID=MALK_01119.t1;Parent=MALK_01119
contig003	AUGUSTUS	exon	579797	579830	0.6	-	.	ID=MALK_01119.t1.e2;Parent=MALK_01119.t1
contig003	AUGUSTUS	exon	579134	579672	0.6	-	.	ID=MALK_01119.t1.e1;Parent=MALK_01119.t1
contig003	AUGUSTUS	gene	579951	581069	0.84	+	.	ID=MALK_01120;prediction_source=braker_MRET:g3159.t1
contig003	AUGUSTUS	CDS	579951	581069	0.84	+	0	ID=MALK_01120.t1.c1;Parent=MALK_01120.t1
contig003	AUGUSTUS	mRNA	579951	581069	0.84	+	.	ID=MALK_01120.t1;Parent=MALK_01120
contig003	AUGUSTUS	exon	579951	581069	.	+	.	ID=MALK_01120.t1.e1;Parent=MALK_01120.t1
contig003	AUGUSTUS	gene	581133	581708	0.51	+	.	ID=MALK_01121;prediction_source=augustus:contig003.g3426.t1
contig003	AUGUSTUS	CDS	581133	581708	0.51	+	0	ID=MALK_01121.t1.c1;Parent=MALK_01121.t1
contig003	AUGUSTUS	mRNA	581133	581708	0.51	+	.	ID=MALK_01121.t1;Parent=MALK_01121
contig003	AUGUSTUS	exon	581133	581708	0.51	+	.	ID=MALK_01121.t1.e1;Parent=MALK_01121.t1
contig003	AUGUSTUS	gene	581709	583106	0.93	-	.	ID=MALK_01122;prediction_source=augustus:contig003.g3427.t1
contig003	AUGUSTUS	CDS	581709	583106	0.93	-	0	ID=MALK_01122.t1.c1;Parent=MALK_01122.t1
contig003	AUGUSTUS	mRNA	581709	583106	0.93	-	.	ID=MALK_01122.t1;Parent=MALK_01122
contig003	AUGUSTUS	exon	581709	583106	0.93	-	.	ID=MALK_01122.t1.e1;Parent=MALK_01122.t1
contig003	AUGUSTUS	gene	583204	583971	0.8	+	.	ID=MALK_01123;prediction_source=augustus:contig003.g3428.t1
contig003	AUGUSTUS	CDS	583204	583971	0.8	+	0	ID=MALK_01123.t1.c1;Parent=MALK_01123.t1
contig003	AUGUSTUS	mRNA	583204	583971	0.8	+	.	ID=MALK_01123.t1;Parent=MALK_01123
contig003	AUGUSTUS	exon	583204	583971	0.8	+	.	ID=MALK_01123.t1.e1;Parent=MALK_01123.t1
contig003	AUGUSTUS	gene	584047	584515	0.6	-	.	ID=MALK_01124;prediction_source=braker_MRET:g3163.t1
contig003	AUGUSTUS	CDS	584147	584515	0.95	-	0	ID=MALK_01124.t1.c2;Parent=MALK_01124.t1
contig003	AUGUSTUS	CDS	584047	584106	0.95	-	0	ID=MALK_01124.t1.c1;Parent=MALK_01124.t1
contig003	AUGUSTUS	mRNA	584047	584515	0.6	-	.	ID=MALK_01124.t1;Parent=MALK_01124
contig003	AUGUSTUS	exon	584147	584515	.	-	.	ID=MALK_01124.t1.e2;Parent=MALK_01124.t1
contig003	AUGUSTUS	exon	584047	584106	.	-	.	ID=MALK_01124.t1.e1;Parent=MALK_01124.t1
contig003	maker	gene	584806	585486	.	+	.	ID=MALK_01125;prediction_source=maker_MRET:augustus_masked-contig003-processed-gene-5.25-mRNA-1
contig003	maker	CDS	584806	585486	.	+	0	ID=MALK_01125.t1.c1;Parent=MALK_01125.t1
contig003	maker	mRNA	584806	585486	.	+	.	ID=MALK_01125.t1;Parent=MALK_01125
contig003	maker	exon	584806	585486	.	+	.	ID=MALK_01125.t1.e1;Parent=MALK_01125.t1
contig003	AUGUSTUS	gene	585925	586833	0.87	+	.	ID=MALK_01126;prediction_source=augustus:contig003.g3430.t1
contig003	AUGUSTUS	CDS	585925	586833	0.87	+	0	ID=MALK_01126.t1.c1;Parent=MALK_01126.t1
contig003	AUGUSTUS	mRNA	585925	586833	0.87	+	.	ID=MALK_01126.t1;Parent=MALK_01126
contig003	AUGUSTUS	exon	585925	586833	0.87	+	.	ID=MALK_01126.t1.e1;Parent=MALK_01126.t1
contig003	AUGUSTUS	gene	586850	587815	1	-	.	ID=MALK_01127;prediction_source=braker_MRET:g3166.t1
contig003	AUGUSTUS	CDS	586850	587815	1	-	0	ID=MALK_01127.t1.c1;Parent=MALK_01127.t1
contig003	AUGUSTUS	mRNA	586850	587815	1	-	.	ID=MALK_01127.t1;Parent=MALK_01127
contig003	AUGUSTUS	exon	586850	587815	.	-	.	ID=MALK_01127.t1.e1;Parent=MALK_01127.t1
contig003	AUGUSTUS	gene	588065	589453	0.54	+	.	ID=MALK_01128;prediction_source=augustus:contig003.g3432.t1
contig003	AUGUSTUS	CDS	588065	589453	0.54	+	0	ID=MALK_01128.t1.c1;Parent=MALK_01128.t1

contig003	AUGUSTUS	mRNA	588065	589453	0.54	+	.	ID=MALK_01128.t1;Parent=MALK_01128
contig003	AUGUSTUS	exon	588065	589453	0.54	+	.	ID=MALK_01128.t1.e1;Parent=MALK_01128.t1
contig003	AUGUSTUS	gene	589766	591388	1	-	.	ID=MALK_01129;prediction_source=braker_MRET:g3168.t1
contig003	AUGUSTUS	CDS	591222	591388	1	-	0	ID=MALK_01129.t1.c2;Parent=MALK_01129.t1
contig003	AUGUSTUS	CDS	589766	591194	1	-	0	ID=MALK_01129.t1.c1;Parent=MALK_01129.t1
contig003	AUGUSTUS	mRNA	589766	591388	1	-	.	ID=MALK_01129.t1;Parent=MALK_01129
contig003	AUGUSTUS	exon	591222	591388	.	-	.	ID=MALK_01129.t1.e2;Parent=MALK_01129.t1
contig003	AUGUSTUS	exon	589766	591194	.	-	.	ID=MALK_01129.t1.e1;Parent=MALK_01129.t1
contig003	maker	gene	591500	592225	.	+	.	ID=MALK_01130;prediction_source=maker_MRET:augustus_masked-contig003-processed-gene-5.28-mRNA-1
contig003	maker	CDS	591500	592225	.	+	0	ID=MALK_01130.t1.c1;Parent=MALK_01130.t1
contig003	maker	mRNA	591500	592225	.	+	.	ID=MALK_01130.t1;Parent=MALK_01130
contig003	maker	exon	591500	592225	.	+	.	ID=MALK_01130.t1.e1;Parent=MALK_01130.t1
contig003	AUGUSTUS	gene	592238	592840	0.85	-	.	ID=MALK_01131;prediction_source=braker_MRET:g3170.t1
contig003	AUGUSTUS	CDS	592238	592840	0.85	-	0	ID=MALK_01131.t1.c1;Parent=MALK_01131.t1
contig003	AUGUSTUS	mRNA	592238	592840	0.85	-	.	ID=MALK_01131.t1;Parent=MALK_01131
contig003	AUGUSTUS	exon	592238	592840	.	-	.	ID=MALK_01131.t1.e1;Parent=MALK_01131.t1
contig003	maker	gene	592889	594202	.	+	.	ID=MALK_01132;prediction_source=maker_MRET:augustus_masked-contig003-processed-gene-5.29-mRNA-1
contig003	maker	CDS	592889	594202	.	+	0	ID=MALK_01132.t1.c1;Parent=MALK_01132.t1
contig003	maker	mRNA	592889	594202	.	+	.	ID=MALK_01132.t1;Parent=MALK_01132
contig003	maker	exon	592889	594202	.	+	.	ID=MALK_01132.t1.e1;Parent=MALK_01132.t1
contig003	AUGUSTUS	gene	594647	595787	0.38	-	.	ID=MALK_01133;prediction_source=augustus:contig003.g3436.t1
contig003	AUGUSTUS	CDS	595388	595787	0.38	-	0	ID=MALK_01133.t1.c2;Parent=MALK_01133.t1
contig003	AUGUSTUS	CDS	594647	594975	0.38	-	0	ID=MALK_01133.t1.c1;Parent=MALK_01133.t1
contig003	AUGUSTUS	mRNA	594647	595787	0.38	-	.	ID=MALK_01133.t1;Parent=MALK_01133
contig003	AUGUSTUS	exon	595388	595787	0.38	-	.	ID=MALK_01133.t1.e2;Parent=MALK_01133.t1
contig003	AUGUSTUS	exon	594647	594975	0.38	-	.	ID=MALK_01133.t1.e1;Parent=MALK_01133.t1
contig003	AUGUSTUS	gene	595895	598087	0.99	+	.	ID=MALK_01134;prediction_source=braker_MRET:g3173.t1
contig003	AUGUSTUS	CDS	595895	598087	0.99	+	0	ID=MALK_01134.t1.c1;Parent=MALK_01134.t1
contig003	AUGUSTUS	mRNA	595895	598087	0.99	+	.	ID=MALK_01134.t1;Parent=MALK_01134
contig003	AUGUSTUS	exon	595895	598087	.	+	.	ID=MALK_01134.t1.e1;Parent=MALK_01134.t1
contig003	maker	gene	598717	600018	.	+	.	ID=MALK_01135;prediction_source=maker_MRET:augustus_masked-contig003-processed-gene-6.1-mRNA-1
contig003	maker	CDS	598717	600018	.	+	0	ID=MALK_01135.t1.c1;Parent=MALK_01135.t1
contig003	maker	mRNA	598717	600018	.	+	.	ID=MALK_01135.t1;Parent=MALK_01135
contig003	maker	exon	598717	600018	.	+	.	ID=MALK_01135.t1.e1;Parent=MALK_01135.t1
contig003	AUGUSTUS	gene	600288	602114	0.98	+	.	ID=MALK_01136;prediction_source=augustus:contig003.g3440.t1
contig003	AUGUSTUS	CDS	600288	602114	0.98	+	0	ID=MALK_01136.t1.c1;Parent=MALK_01136.t1
contig003	AUGUSTUS	mRNA	600288	602114	0.98	+	.	ID=MALK_01136.t1;Parent=MALK_01136
contig003	AUGUSTUS	exon	600288	602114	0.98	+	.	ID=MALK_01136.t1.e1;Parent=MALK_01136.t1
contig003	AUGUSTUS	gene	602111	603064	0.57	-	.	ID=MALK_01137;prediction_source=augustus:contig003.g3441.t1
contig003	AUGUSTUS	CDS	602111	603064	0.57	-	0	ID=MALK_01137.t1.c1;Parent=MALK_01137.t1
contig003	AUGUSTUS	mRNA	602111	603064	0.57	-	.	ID=MALK_01137.t1;Parent=MALK_01137
contig003	AUGUSTUS	exon	602111	603064	0.57	-	.	ID=MALK_01137.t1.e1;Parent=MALK_01137.t1
contig003	AUGUSTUS	gene	603117	604157	0.95	-	.	ID=MALK_01138;prediction_source=braker_MRET:g3177.t1

contig003	AUGUSTUS	CDS	603117	604157	0.95	-	0	ID=MALK_01138.t1.c1;Parent=MALK_01138.t1
contig003	AUGUSTUS	mRNA	603117	604157	0.95	-	.	ID=MALK_01138.t1;Parent=MALK_01138
contig003	AUGUSTUS	exon	603117	604157	.	-	.	ID=MALK_01138.t1.e1;Parent=MALK_01138.t1
contig003	AUGUSTUS	gene	604427	605086	0.55	+	.	ID=MALK_01139;prediction_source=augustus:contig003.g3443.t1
contig003	AUGUSTUS	CDS	604427	605086	0.55	+	0	ID=MALK_01139.t1.c1;Parent=MALK_01139.t1
contig003	AUGUSTUS	mRNA	604427	605086	0.55	+	.	ID=MALK_01139.t1;Parent=MALK_01139
contig003	AUGUSTUS	exon	604427	605086	0.55	+	.	ID=MALK_01139.t1.e1;Parent=MALK_01139.t1
contig003	AUGUSTUS	gene	605083	606813	0.59	-	.	ID=MALK_01140;prediction_source=braker_MRET:g3179.t1
contig003	AUGUSTUS	CDS	605083	606813	0.59	-	0	ID=MALK_01140.t1.c1;Parent=MALK_01140.t1
contig003	AUGUSTUS	mRNA	605083	606813	0.59	-	.	ID=MALK_01140.t1;Parent=MALK_01140
contig003	AUGUSTUS	exon	605083	606813	.	-	.	ID=MALK_01140.t1.e1;Parent=MALK_01140.t1
contig003	AUGUSTUS	gene	607057	609727	0.51	+	.	ID=MALK_01141;prediction_source=braker_MRET:g3180.t1
contig003	AUGUSTUS	CDS	607057	609019	0.9	+	0	ID=MALK_01141.t1.c1;Parent=MALK_01141.t1
contig003	AUGUSTUS	CDS	609057	609727	0.9	+	0	ID=MALK_01141.t1.c2;Parent=MALK_01141.t1
contig003	AUGUSTUS	mRNA	607057	609727	0.51	+	.	ID=MALK_01141.t1;Parent=MALK_01141
contig003	AUGUSTUS	exon	607057	609019	.	+	.	ID=MALK_01141.t1.e1;Parent=MALK_01141.t1
contig003	AUGUSTUS	exon	609057	609727	.	+	.	ID=MALK_01141.t1.e2;Parent=MALK_01141.t1
contig003	AUGUSTUS	gene	609841	610752	0.72	+	.	ID=MALK_01142;prediction_source=braker_MRET:g3181.t1
contig003	AUGUSTUS	CDS	609841	610752	0.72	+	0	ID=MALK_01142.t1.c1;Parent=MALK_01142.t1
contig003	AUGUSTUS	mRNA	609841	610752	0.72	+	.	ID=MALK_01142.t1;Parent=MALK_01142
contig003	AUGUSTUS	exon	609841	610752	.	+	.	ID=MALK_01142.t1.e1;Parent=MALK_01142.t1
contig003	maker	gene	610868	611782	.	-	.	ID=MALK_01143;prediction_source=maker_MRET:augustus_masked-contig003-processed-gene-6.53-mRNA-1
contig003	maker	CDS	610868	611782	.	-	0	ID=MALK_01143.t1.c1;Parent=MALK_01143.t1
contig003	maker	mRNA	610868	611782	.	-	.	ID=MALK_01143.t1;Parent=MALK_01143
contig003	maker	exon	610868	611782	.	-	.	ID=MALK_01143.t1.e1;Parent=MALK_01143.t1
contig003	maker	gene	611930	613696	.	-	.	ID=MALK_01144;prediction_source=maker_MRET:augustus_masked-contig003-processed-gene-6.54-mRNA-1
contig003	maker	CDS	611930	613696	.	-	0	ID=MALK_01144.t1.c1;Parent=MALK_01144.t1
contig003	maker	mRNA	611930	613696	.	-	.	ID=MALK_01144.t1;Parent=MALK_01144
contig003	maker	exon	611930	613696	.	-	.	ID=MALK_01144.t1.e1;Parent=MALK_01144.t1
contig003	AUGUSTUS	gene	614410	617706	0.39	+	.	ID=MALK_01145;prediction_source=augustus:contig003.g3451.t1
contig003	AUGUSTUS	CDS	614410	617706	0.39	+	0	ID=MALK_01145.t1.c1;Parent=MALK_01145.t1
contig003	AUGUSTUS	mRNA	614410	617706	0.39	+	.	ID=MALK_01145.t1;Parent=MALK_01145
contig003	AUGUSTUS	exon	614410	617706	0.39	+	.	ID=MALK_01145.t1.e1;Parent=MALK_01145.t1
contig003	AUGUSTUS	gene	617707	620469	0.23	-	.	ID=MALK_01146;prediction_source=augustus:contig003.g3452.t1
contig003	AUGUSTUS	CDS	617707	620469	0.23	-	0	ID=MALK_01146.t1.c1;Parent=MALK_01146.t1
contig003	AUGUSTUS	mRNA	617707	620469	0.23	-	.	ID=MALK_01146.t1;Parent=MALK_01146
contig003	AUGUSTUS	exon	617707	620469	0.23	-	.	ID=MALK_01146.t1.e1;Parent=MALK_01146.t1
contig003	AUGUSTUS	gene	620663	621453	0.81	-	.	ID=MALK_01147;prediction_source=braker_MRET:g3186.t1
contig003	AUGUSTUS	CDS	621446	621453	0.81	-	0	ID=MALK_01147.t1.c5;Parent=MALK_01147.t1
contig003	AUGUSTUS	CDS	621355	621406	0.81	-	0	ID=MALK_01147.t1.c4;Parent=MALK_01147.t1
contig003	AUGUSTUS	CDS	621240	621326	0.81	-	0	ID=MALK_01147.t1.c3;Parent=MALK_01147.t1
contig003	AUGUSTUS	CDS	621164	621174	0.81	-	0	ID=MALK_01147.t1.c2;Parent=MALK_01147.t1
contig003	AUGUSTUS	CDS	620663	621125	0.81	-	0	ID=MALK_01147.t1.c1;Parent=MALK_01147.t1

contig003	AUGUSTUS	mRNA	620663	621453	0.81	-	.	ID=MALK_01147.t1;Parent=MALK_01147
contig003	AUGUSTUS	exon	621446	621453	.	-	.	ID=MALK_01147.t1.e5;Parent=MALK_01147.t1
contig003	AUGUSTUS	exon	621355	621406	.	-	.	ID=MALK_01147.t1.e4;Parent=MALK_01147.t1
contig003	AUGUSTUS	exon	621240	621326	.	-	.	ID=MALK_01147.t1.e3;Parent=MALK_01147.t1
contig003	AUGUSTUS	exon	621164	621174	.	-	.	ID=MALK_01147.t1.e2;Parent=MALK_01147.t1
contig003	AUGUSTUS	exon	620663	621125	.	-	.	ID=MALK_01147.t1.e1;Parent=MALK_01147.t1
contig003	AUGUSTUS	gene	621771	623415	0.27	+	.	ID=MALK_01148;prediction_source=braker_MRET:g3187.t1
contig003	AUGUSTUS	CDS	621771	621797	0.68	+	0	ID=MALK_01148.t1.c1;Parent=MALK_01148.t1
contig003	AUGUSTUS	CDS	621843	621995	0.68	+	0	ID=MALK_01148.t1.c2;Parent=MALK_01148.t1
contig003	AUGUSTUS	CDS	622072	622207	0.68	+	0	ID=MALK_01148.t1.c3;Parent=MALK_01148.t1
contig003	AUGUSTUS	CDS	622250	622362	0.68	+	0	ID=MALK_01148.t1.c4;Parent=MALK_01148.t1
contig003	AUGUSTUS	CDS	622402	623415	0.68	+	0	ID=MALK_01148.t1.c5;Parent=MALK_01148.t1
contig003	AUGUSTUS	mRNA	621771	623415	0.27	+	.	ID=MALK_01148.t1;Parent=MALK_01148
contig003	AUGUSTUS	exon	621771	621797	.	+	.	ID=MALK_01148.t1.e1;Parent=MALK_01148.t1
contig003	AUGUSTUS	exon	621843	621995	.	+	.	ID=MALK_01148.t1.e2;Parent=MALK_01148.t1
contig003	AUGUSTUS	exon	622072	622207	.	+	.	ID=MALK_01148.t1.e3;Parent=MALK_01148.t1
contig003	AUGUSTUS	exon	622250	622362	.	+	.	ID=MALK_01148.t1.e4;Parent=MALK_01148.t1
contig003	AUGUSTUS	exon	622402	623415	.	+	.	ID=MALK_01148.t1.e5;Parent=MALK_01148.t1
contig003	AUGUSTUS	gene	624049	626181	0.99	+	.	ID=MALK_01149;prediction_source=augustus:contig003.g3455.t1
contig003	AUGUSTUS	CDS	624049	626181	0.99	+	0	ID=MALK_01149.t1.c1;Parent=MALK_01149.t1
contig003	AUGUSTUS	mRNA	624049	626181	0.99	+	.	ID=MALK_01149.t1;Parent=MALK_01149
contig003	AUGUSTUS	exon	624049	626181	0.99	+	.	ID=MALK_01149.t1.e1;Parent=MALK_01149.t1
contig003	AUGUSTUS	gene	626397	626905	0.43	-	.	ID=MALK_01150;prediction_source=braker_MRET:g3189.t1
contig003	AUGUSTUS	CDS	626879	626905	0.43	-	0	ID=MALK_01150.t1.c3;Parent=MALK_01150.t1
contig003	AUGUSTUS	CDS	626734	626848	0.43	-	0	ID=MALK_01150.t1.c2;Parent=MALK_01150.t1
contig003	AUGUSTUS	CDS	626397	626704	0.43	-	0	ID=MALK_01150.t1.c1;Parent=MALK_01150.t1
contig003	AUGUSTUS	mRNA	626397	626905	0.43	-	.	ID=MALK_01150.t1;Parent=MALK_01150
contig003	AUGUSTUS	exon	626879	626905	.	-	.	ID=MALK_01150.t1.e3;Parent=MALK_01150.t1
contig003	AUGUSTUS	exon	626734	626848	.	-	.	ID=MALK_01150.t1.e2;Parent=MALK_01150.t1
contig003	AUGUSTUS	exon	626397	626704	.	-	.	ID=MALK_01150.t1.e1;Parent=MALK_01150.t1
contig003	maker	gene	627091	628446	.	+	.	ID=MALK_01151;prediction_source=maker_MRET:augustus_masked-contig003-processed-gene-6.10-mRNA-1
contig003	maker	CDS	627091	628446	.	+	0	ID=MALK_01151.t1.c1;Parent=MALK_01151.t1
contig003	maker	mRNA	627091	628446	.	+	.	ID=MALK_01151.t1;Parent=MALK_01151
contig003	maker	exon	627091	628446	.	+	.	ID=MALK_01151.t1.e1;Parent=MALK_01151.t1
contig003	AUGUSTUS	gene	628678	630060	0.87	-	.	ID=MALK_01152;prediction_source=augustus:contig003.g3461.t1
contig003	AUGUSTUS	CDS	628678	630060	0.87	-	0	ID=MALK_01152.t1.c1;Parent=MALK_01152.t1
contig003	AUGUSTUS	mRNA	628678	630060	0.87	-	.	ID=MALK_01152.t1;Parent=MALK_01152
contig003	AUGUSTUS	exon	628678	630060	0.87	-	.	ID=MALK_01152.t1.e1;Parent=MALK_01152.t1
contig003	AUGUSTUS	gene	630539	631963	0.77	+	.	ID=MALK_01153;prediction_source=augustus:contig003.g3462.t1
contig003	AUGUSTUS	CDS	630539	631963	0.77	+	0	ID=MALK_01153.t1.c1;Parent=MALK_01153.t1
contig003	AUGUSTUS	mRNA	630539	631963	0.77	+	.	ID=MALK_01153.t1;Parent=MALK_01153
contig003	AUGUSTUS	exon	630539	631963	0.77	+	.	ID=MALK_01153.t1.e1;Parent=MALK_01153.t1
contig003	maker	gene	632018	633547	.	-	.	ID=MALK_01154;prediction_source=maker_MRET:augustus_masked-contig003-processed-gene-6.59-mRNA-1

contig003	maker	CDS	632018	633547	.	-	0	ID=MALK_01154.t1.c1;Parent=MALK_01154.t1
contig003	maker	mRNA	632018	633547	.	-	.	ID=MALK_01154.t1;Parent=MALK_01154
contig003	maker	exon	632018	633547	.	-	.	ID=MALK_01154.t1.e1;Parent=MALK_01154.t1
contig003	maker	gene	634029	634733	.	+	.	ID=MALK_01155;prediction_source=maker_MRET:augustus_masked-contig003-processed-gene-6.12-mRNA-1
contig003	maker	CDS	634029	634733	.	+	0	ID=MALK_01155.t1.c1;Parent=MALK_01155.t1
contig003	maker	mRNA	634029	634733	.	+	.	ID=MALK_01155.t1;Parent=MALK_01155
contig003	maker	exon	634029	634733	.	+	.	ID=MALK_01155.t1.e1;Parent=MALK_01155.t1
contig003	AUGUSTUS	gene	634730	636679	0.68	-	.	ID=MALK_01156;prediction_source=braker_MRET:g3195.t1
contig003	AUGUSTUS	CDS	634730	636679	0.68	-	0	ID=MALK_01156.t1.c1;Parent=MALK_01156.t1
contig003	AUGUSTUS	mRNA	634730	636679	0.68	-	.	ID=MALK_01156.t1;Parent=MALK_01156
contig003	AUGUSTUS	exon	634730	636679	.	-	.	ID=MALK_01156.t1.e1;Parent=MALK_01156.t1
contig003	AUGUSTUS	gene	636721	638064	0.97	+	.	ID=MALK_01157;prediction_source=braker_MRET:g3196.t1
contig003	AUGUSTUS	CDS	636721	638064	0.97	+	0	ID=MALK_01157.t1.c1;Parent=MALK_01157.t1
contig003	AUGUSTUS	mRNA	636721	638064	0.97	+	.	ID=MALK_01157.t1;Parent=MALK_01157
contig003	AUGUSTUS	exon	636721	638064	.	+	.	ID=MALK_01157.t1.e1;Parent=MALK_01157.t1
contig003	AUGUSTUS	gene	638090	638944	0.88	-	.	ID=MALK_01158;prediction_source=braker_MRET:g3197.t1
contig003	AUGUSTUS	CDS	638090	638944	0.88	-	0	ID=MALK_01158.t1.c1;Parent=MALK_01158.t1
contig003	AUGUSTUS	mRNA	638090	638944	0.88	-	.	ID=MALK_01158.t1;Parent=MALK_01158
contig003	AUGUSTUS	exon	638090	638944	.	-	.	ID=MALK_01158.t1.e1;Parent=MALK_01158.t1
contig003	maker	gene	638976	639671	.	+	.	ID=MALK_01159;prediction_source=maker_MRET:augustus_masked-contig003-processed-gene-6.14-mRNA-1
contig003	maker	CDS	638976	639671	.	+	0	ID=MALK_01159.t1.c1;Parent=MALK_01159.t1
contig003	maker	mRNA	638976	639671	.	+	.	ID=MALK_01159.t1;Parent=MALK_01159
contig003	maker	exon	638976	639671	.	+	.	ID=MALK_01159.t1.e1;Parent=MALK_01159.t1
contig003	maker	gene	639677	642640	.	-	.	ID=MALK_01160;prediction_source=maker_MRET:augustus_masked-contig003-processed-gene-6.62-mRNA-1
contig003	maker	CDS	639677	642640	.	-	0	ID=MALK_01160.t1.c1;Parent=MALK_01160.t1
contig003	maker	mRNA	639677	642640	.	-	.	ID=MALK_01160.t1;Parent=MALK_01160
contig003	maker	exon	639677	642640	.	-	.	ID=MALK_01160.t1.e1;Parent=MALK_01160.t1
contig003	maker	gene	643035	647222	.	+	.	ID=MALK_01161;prediction_source=maker_MRET:augustus_masked-contig003-processed-gene-6.15-mRNA-1
contig003	maker	CDS	643035	647222	.	+	0	ID=MALK_01161.t1.c1;Parent=MALK_01161.t1
contig003	maker	mRNA	643035	647222	.	+	.	ID=MALK_01161.t1;Parent=MALK_01161
contig003	maker	exon	643035	647222	.	+	.	ID=MALK_01161.t1.e1;Parent=MALK_01161.t1
contig003	AUGUSTUS	gene	647445	648830	1	+	.	ID=MALK_01162;prediction_source=augustus:contig003.g3472.t1
contig003	AUGUSTUS	CDS	647445	648830	1	+	0	ID=MALK_01162.t1.c1;Parent=MALK_01162.t1
contig003	AUGUSTUS	mRNA	647445	648830	1	+	.	ID=MALK_01162.t1;Parent=MALK_01162
contig003	AUGUSTUS	exon	647445	648830	1	+	.	ID=MALK_01162.t1.e1;Parent=MALK_01162.t1
contig003	maker	gene	649320	653156	.	+	.	ID=MALK_01163;prediction_source=maker_MRET:augustus_masked-contig003-processed-gene-6.17-mRNA-1
contig003	maker	CDS	649320	653156	.	+	0	ID=MALK_01163.t1.c1;Parent=MALK_01163.t1
contig003	maker	mRNA	649320	653156	.	+	.	ID=MALK_01163.t1;Parent=MALK_01163
contig003	maker	exon	649320	653156	.	+	.	ID=MALK_01163.t1.e1;Parent=MALK_01163.t1
contig003	maker	gene	653915	655645	.	-	.	ID=MALK_01164;prediction_source=maker_MRET:augustus_masked-contig003-processed-gene-6.63-mRNA-1
contig003	maker	CDS	653915	655645	.	-	0	ID=MALK_01164.t1.c1;Parent=MALK_01164.t1
contig003	maker	mRNA	653915	655645	.	-	.	ID=MALK_01164.t1;Parent=MALK_01164
contig003	maker	exon	653915	655645	.	-	.	ID=MALK_01164.t1.e1;Parent=MALK_01164.t1

contig003	maker	gene	655914	657653	.	-	.	ID=MALK_01165;prediction_source=maker_MRET:augustus_masked-contig003-processed-gene-6.64-mRNA-1
contig003	maker	CDS	655914	657653	.	-	0	ID=MALK_01165.t1.c1;Parent=MALK_01165.t1
contig003	maker	mRNA	655914	657653	.	-	.	ID=MALK_01165.t1;Parent=MALK_01165
contig003	maker	exon	655914	657653	.	-	.	ID=MALK_01165.t1.e1;Parent=MALK_01165.t1
contig003	maker	gene	658014	659750	.	+	.	ID=MALK_01166;prediction_source=maker_MRET:augustus_masked-contig003-processed-gene-6.18-mRNA-1
contig003	maker	CDS	658014	659750	.	+	0	ID=MALK_01166.t1.c1;Parent=MALK_01166.t1
contig003	maker	mRNA	658014	659750	.	+	.	ID=MALK_01166.t1;Parent=MALK_01166
contig003	maker	exon	658014	659750	.	+	.	ID=MALK_01166.t1.e1;Parent=MALK_01166.t1
contig003	maker	gene	659999	661906	.	+	.	ID=MALK_01167;prediction_source=maker_MRET:augustus_masked-contig003-processed-gene-6.19-mRNA-1
contig003	maker	CDS	659999	661906	.	+	0	ID=MALK_01167.t1.c1;Parent=MALK_01167.t1
contig003	maker	mRNA	659999	661906	.	+	.	ID=MALK_01167.t1;Parent=MALK_01167
contig003	maker	exon	659999	661906	.	+	.	ID=MALK_01167.t1.e1;Parent=MALK_01167.t1
contig003	AUGUSTUS	gene	662013	662408	0.73	+	.	ID=MALK_01168;prediction_source=braker_MRET:g3207.t1
contig003	AUGUSTUS	CDS	662013	662408	0.73	+	0	ID=MALK_01168.t1.c1;Parent=MALK_01168.t1
contig003	AUGUSTUS	mRNA	662013	662408	0.73	+	.	ID=MALK_01168.t1;Parent=MALK_01168
contig003	AUGUSTUS	exon	662013	662408	.	+	.	ID=MALK_01168.t1.e1;Parent=MALK_01168.t1
contig003	AUGUSTUS	gene	662609	663413	0.3	+	.	ID=MALK_01169;prediction_source=braker_MRET:g3208.t1
contig003	AUGUSTUS	CDS	662609	663364	0.51	+	0	ID=MALK_01169.t1.c1;Parent=MALK_01169.t1
contig003	AUGUSTUS	CDS	663399	663413	0.51	+	0	ID=MALK_01169.t1.c2;Parent=MALK_01169.t1
contig003	AUGUSTUS	mRNA	662609	663413	0.3	+	.	ID=MALK_01169.t1;Parent=MALK_01169
contig003	AUGUSTUS	exon	662609	663364	.	+	.	ID=MALK_01169.t1.e1;Parent=MALK_01169.t1
contig003	AUGUSTUS	exon	663399	663413	.	+	.	ID=MALK_01169.t1.e2;Parent=MALK_01169.t1
contig003	maker	gene	663494	664705	.	-	.	ID=MALK_01170;prediction_source=maker_MRET:augustus_masked-contig003-processed-gene-6.65-mRNA-1
contig003	maker	CDS	663494	664705	.	-	0	ID=MALK_01170.t1.c1;Parent=MALK_01170.t1
contig003	maker	mRNA	663494	664705	.	-	.	ID=MALK_01170.t1;Parent=MALK_01170
contig003	maker	exon	663494	664705	.	-	.	ID=MALK_01170.t1.e1;Parent=MALK_01170.t1
contig003	AUGUSTUS	gene	664734	665652	0.65	+	.	ID=MALK_01171;prediction_source=braker_MRET:g3210.t1
contig003	AUGUSTUS	CDS	664734	665572	0.7	+	0	ID=MALK_01171.t1.c1;Parent=MALK_01171.t1
contig003	AUGUSTUS	CDS	665640	665652	0.7	+	0	ID=MALK_01171.t1.c2;Parent=MALK_01171.t1
contig003	AUGUSTUS	mRNA	664734	665652	0.65	+	.	ID=MALK_01171.t1;Parent=MALK_01171
contig003	AUGUSTUS	exon	664734	665572	.	+	.	ID=MALK_01171.t1.e1;Parent=MALK_01171.t1
contig003	AUGUSTUS	exon	665640	665652	.	+	.	ID=MALK_01171.t1.e2;Parent=MALK_01171.t1
contig003	AUGUSTUS	gene	665705	666509	0.69	-	.	ID=MALK_01172;prediction_source=braker_MRET:g3211.t1
contig003	AUGUSTUS	CDS	665979	666509	0.75	-	0	ID=MALK_01172.t1.c2;Parent=MALK_01172.t1
contig003	AUGUSTUS	CDS	665705	665914	0.75	-	0	ID=MALK_01172.t1.c1;Parent=MALK_01172.t1
contig003	AUGUSTUS	mRNA	665705	666509	0.69	-	.	ID=MALK_01172.t1;Parent=MALK_01172
contig003	AUGUSTUS	exon	665979	666509	.	-	.	ID=MALK_01172.t1.e2;Parent=MALK_01172.t1
contig003	AUGUSTUS	exon	665705	665914	.	-	.	ID=MALK_01172.t1.e1;Parent=MALK_01172.t1
contig003	maker	gene	666827	668227	.	+	.	ID=MALK_01173;prediction_source=maker_MRET:augustus_masked-contig003-processed-gene-6.21-mRNA-1
contig003	maker	CDS	666827	668227	.	+	0	ID=MALK_01173.t1.c1;Parent=MALK_01173.t1
contig003	maker	mRNA	666827	668227	.	+	.	ID=MALK_01173.t1;Parent=MALK_01173
contig003	maker	exon	666827	668227	.	+	.	ID=MALK_01173.t1.e1;Parent=MALK_01173.t1
contig003	AUGUSTUS	gene	668428	669846	0.63	+	.	ID=MALK_01174;prediction_source=augustus:contig003.g3482.t1

contig003	AUGUSTUS	CDS	668428	669846	0.63	+	0	ID=MALK_01174.t1.c1;Parent=MALK_01174.t1
contig003	AUGUSTUS	mRNA	668428	669846	0.63	+	.	ID=MALK_01174.t1;Parent=MALK_01174
contig003	AUGUSTUS	exon	668428	669846	0.63	+	.	ID=MALK_01174.t1.e1;Parent=MALK_01174.t1
contig003	AUGUSTUS	gene	669913	671355	0.39	+	.	ID=MALK_01175;prediction_source=braker_MRET:g3214.t1
contig003	AUGUSTUS	CDS	669913	670038	0.63	+	0	ID=MALK_01175.t1.c1;Parent=MALK_01175.t1
contig003	AUGUSTUS	CDS	670066	671355	0.63	+	0	ID=MALK_01175.t1.c2;Parent=MALK_01175.t1
contig003	AUGUSTUS	mRNA	669913	671355	0.39	+	.	ID=MALK_01175.t1;Parent=MALK_01175
contig003	AUGUSTUS	exon	669913	670038	.	+	.	ID=MALK_01175.t1.e1;Parent=MALK_01175.t1
contig003	AUGUSTUS	exon	670066	671355	.	+	.	ID=MALK_01175.t1.e2;Parent=MALK_01175.t1
contig003	maker	gene	671862	672770	.	+	.	ID=MALK_01176;prediction_source=maker_MRET:augustus_masked-contig003-processed-gene-6.24-mRNA-1
contig003	maker	CDS	671862	672770	.	+	0	ID=MALK_01176.t1.c1;Parent=MALK_01176.t1
contig003	maker	mRNA	671862	672770	.	+	.	ID=MALK_01176.t1;Parent=MALK_01176
contig003	maker	exon	671862	672770	.	+	.	ID=MALK_01176.t1.e1;Parent=MALK_01176.t1
contig003	maker	gene	673262	676348	.	+	.	ID=MALK_01177;prediction_source=maker_MRET:augustus_masked-contig003-processed-gene-6.25-mRNA-1
contig003	maker	CDS	673262	676348	.	+	0	ID=MALK_01177.t1.c1;Parent=MALK_01177.t1
contig003	maker	mRNA	673262	676348	.	+	.	ID=MALK_01177.t1;Parent=MALK_01177
contig003	maker	exon	673262	676348	.	+	.	ID=MALK_01177.t1.e1;Parent=MALK_01177.t1
contig003	AUGUSTUS	gene	676351	677571	0.99	-	.	ID=MALK_01178;prediction_source=augustus:contig003.g3488.t1
contig003	AUGUSTUS	CDS	676351	677571	0.99	-	0	ID=MALK_01178.t1.c1;Parent=MALK_01178.t1
contig003	AUGUSTUS	mRNA	676351	677571	0.99	-	.	ID=MALK_01178.t1;Parent=MALK_01178
contig003	AUGUSTUS	exon	676351	677571	0.99	-	.	ID=MALK_01178.t1.e1;Parent=MALK_01178.t1
contig003	AUGUSTUS	gene	677690	678856	0.38	+	.	ID=MALK_01179;prediction_source=augustus:contig003.g3489.t1
contig003	AUGUSTUS	CDS	677690	678856	0.38	+	0	ID=MALK_01179.t1.c1;Parent=MALK_01179.t1
contig003	AUGUSTUS	mRNA	677690	678856	0.38	+	.	ID=MALK_01179.t1;Parent=MALK_01179
contig003	AUGUSTUS	exon	677690	678856	0.38	+	.	ID=MALK_01179.t1.e1;Parent=MALK_01179.t1
contig003	AUGUSTUS	gene	678879	679602	0.22	-	.	ID=MALK_01180;prediction_source=braker_MRET:g3219.t1
contig003	AUGUSTUS	CDS	679518	679602	0.22	-	0	ID=MALK_01180.t1.c2;Parent=MALK_01180.t1
contig003	AUGUSTUS	CDS	678879	679474	0.22	-	0	ID=MALK_01180.t1.c1;Parent=MALK_01180.t1
contig003	AUGUSTUS	mRNA	678879	679602	0.22	-	.	ID=MALK_01180.t1;Parent=MALK_01180
contig003	AUGUSTUS	exon	679518	679602	.	-	.	ID=MALK_01180.t1.e2;Parent=MALK_01180.t1
contig003	AUGUSTUS	exon	678879	679474	.	-	.	ID=MALK_01180.t1.e1;Parent=MALK_01180.t1
contig003	AUGUSTUS	gene	679844	681760	1	-	.	ID=MALK_01181;prediction_source=augustus:contig003.g3490.t1
contig003	AUGUSTUS	CDS	679844	681760	1	-	0	ID=MALK_01181.t1.c1;Parent=MALK_01181.t1
contig003	AUGUSTUS	mRNA	679844	681760	1	-	.	ID=MALK_01181.t1;Parent=MALK_01181
contig003	AUGUSTUS	exon	679844	681760	1	-	.	ID=MALK_01181.t1.e1;Parent=MALK_01181.t1
contig003	AUGUSTUS	gene	682271	683800	0.66	-	.	ID=MALK_01182;prediction_source=augustus:contig003.g3492.t1
contig003	AUGUSTUS	CDS	682271	683800	0.66	-	0	ID=MALK_01182.t1.c1;Parent=MALK_01182.t1
contig003	AUGUSTUS	mRNA	682271	683800	0.66	-	.	ID=MALK_01182.t1;Parent=MALK_01182
contig003	AUGUSTUS	exon	682271	683800	0.66	-	.	ID=MALK_01182.t1.e1;Parent=MALK_01182.t1
contig003	AUGUSTUS	gene	683900	684685	0.89	+	.	ID=MALK_01183;prediction_source=augustus:contig003.g3493.t1
contig003	AUGUSTUS	CDS	683900	684685	0.89	+	0	ID=MALK_01183.t1.c1;Parent=MALK_01183.t1
contig003	AUGUSTUS	mRNA	683900	684685	0.89	+	.	ID=MALK_01183.t1;Parent=MALK_01183
contig003	AUGUSTUS	exon	683900	684685	0.89	+	.	ID=MALK_01183.t1.e1;Parent=MALK_01183.t1

contig003	maker	gene	684702	693083	.	-	.	ID=MALK_01184;prediction_source=maker_MRET:augustus_masked-contig003-processed-gene-6.70-mRNA-1
contig003	maker	CDS	684702	693083	.	-	0	ID=MALK_01184.t1.c1;Parent=MALK_01184.t1
contig003	maker	mRNA	684702	693083	.	-	.	ID=MALK_01184.t1;Parent=MALK_01184
contig003	maker	exon	684702	693083	.	-	.	ID=MALK_01184.t1.e1;Parent=MALK_01184.t1
contig003	maker	gene	693237	695366	.	+	.	ID=MALK_01185;prediction_source=maker_MRET:augustus_masked-contig003-processed-gene-6.28-mRNA-1
contig003	maker	CDS	693237	695366	.	+	0	ID=MALK_01185.t1.c1;Parent=MALK_01185.t1
contig003	maker	mRNA	693237	695366	.	+	.	ID=MALK_01185.t1;Parent=MALK_01185
contig003	maker	exon	693237	695366	.	+	.	ID=MALK_01185.t1.e1;Parent=MALK_01185.t1
contig003	AUGUSTUS	gene	695363	697030	0.93	-	.	ID=MALK_01186;prediction_source=augustus:contig003.g3497.t1
contig003	AUGUSTUS	CDS	695363	697030	0.93	-	0	ID=MALK_01186.t1.c1;Parent=MALK_01186.t1
contig003	AUGUSTUS	mRNA	695363	697030	0.93	-	.	ID=MALK_01186.t1;Parent=MALK_01186
contig003	AUGUSTUS	exon	695363	697030	0.93	-	.	ID=MALK_01186.t1.e1;Parent=MALK_01186.t1
contig003	AUGUSTUS	gene	697064	698035	0.99	-	.	ID=MALK_01187;prediction_source=braker_MRET:g3226.t1
contig003	AUGUSTUS	CDS	697064	698035	0.99	-	0	ID=MALK_01187.t1.c1;Parent=MALK_01187.t1
contig003	AUGUSTUS	mRNA	697064	698035	0.99	-	.	ID=MALK_01187.t1;Parent=MALK_01187
contig003	AUGUSTUS	exon	697064	698035	.	-	.	ID=MALK_01187.t1.e1;Parent=MALK_01187.t1
contig003	AUGUSTUS	gene	698088	698924	0.61	-	.	ID=MALK_01188;prediction_source=augustus:contig003.g3499.t1
contig003	AUGUSTUS	CDS	698088	698924	0.61	-	0	ID=MALK_01188.t1.c1;Parent=MALK_01188.t1
contig003	AUGUSTUS	mRNA	698088	698924	0.61	-	.	ID=MALK_01188.t1;Parent=MALK_01188
contig003	AUGUSTUS	exon	698088	698924	0.61	-	.	ID=MALK_01188.t1.e1;Parent=MALK_01188.t1
contig003	AUGUSTUS	gene	699610	700803	0.41	+	.	ID=MALK_01189;prediction_source=augustus:contig003.g3500.t1
contig003	AUGUSTUS	CDS	699610	700803	0.41	+	0	ID=MALK_01189.t1.c1;Parent=MALK_01189.t1
contig003	AUGUSTUS	mRNA	699610	700803	0.41	+	.	ID=MALK_01189.t1;Parent=MALK_01189
contig003	AUGUSTUS	exon	699610	700803	0.41	+	.	ID=MALK_01189.t1.e1;Parent=MALK_01189.t1
contig003	AUGUSTUS	gene	700947	702602	0.9	-	.	ID=MALK_01190;prediction_source=augustus:contig003.g3501.t1
contig003	AUGUSTUS	CDS	700947	702602	0.9	-	0	ID=MALK_01190.t1.c1;Parent=MALK_01190.t1
contig003	AUGUSTUS	mRNA	700947	702602	0.9	-	.	ID=MALK_01190.t1;Parent=MALK_01190
contig003	AUGUSTUS	exon	700947	702602	0.9	-	.	ID=MALK_01190.t1.e1;Parent=MALK_01190.t1
contig003	maker	gene	702935	703825	.	+	.	ID=MALK_01191;prediction_source=maker_MRET:augustus_masked-contig003-processed-gene-6.30-mRNA-1
contig003	maker	CDS	702935	703825	.	+	0	ID=MALK_01191.t1.c1;Parent=MALK_01191.t1
contig003	maker	mRNA	702935	703825	.	+	.	ID=MALK_01191.t1;Parent=MALK_01191
contig003	maker	exon	702935	703825	.	+	.	ID=MALK_01191.t1.e1;Parent=MALK_01191.t1
contig003	AUGUSTUS	gene	703834	704687	0.1	-	.	ID=MALK_01192;prediction_source=braker_MRET:g3231.t1
contig003	AUGUSTUS	CDS	703995	704687	0.19	-	0	ID=MALK_01192.t1.c3;Parent=MALK_01192.t1
contig003	AUGUSTUS	CDS	703907	703943	0.19	-	0	ID=MALK_01192.t1.c2;Parent=MALK_01192.t1
contig003	AUGUSTUS	CDS	703834	703874	0.19	-	0	ID=MALK_01192.t1.c1;Parent=MALK_01192.t1
contig003	AUGUSTUS	mRNA	703834	704687	0.1	-	.	ID=MALK_01192.t1;Parent=MALK_01192
contig003	AUGUSTUS	exon	703995	704687	.	-	.	ID=MALK_01192.t1.e3;Parent=MALK_01192.t1
contig003	AUGUSTUS	exon	703907	703943	.	-	.	ID=MALK_01192.t1.e2;Parent=MALK_01192.t1
contig003	AUGUSTUS	exon	703834	703874	.	-	.	ID=MALK_01192.t1.e1;Parent=MALK_01192.t1
contig003	maker	gene	704744	705352	.	-	.	ID=MALK_01193;prediction_source=maker_MRET:augustus_masked-contig003-processed-gene-6.73-mRNA-1
contig003	maker	CDS	704744	705352	.	-	0	ID=MALK_01193.t1.c1;Parent=MALK_01193.t1
contig003	maker	mRNA	704744	705352	.	-	.	ID=MALK_01193.t1;Parent=MALK_01193

contig003	maker	exon	704744	705352	.	-	.	ID=MALK_01193.t1.e1;Parent=MALK_01193.t1
contig003	AUGUSTUS	gene	705598	707547	0.9	+	.	ID=MALK_01194;prediction_source=augustus:contig003.g3504.t1
contig003	AUGUSTUS	CDS	705598	707547	0.9	+	0	ID=MALK_01194.t1.c1;Parent=MALK_01194.t1
contig003	AUGUSTUS	mRNA	705598	707547	0.9	+	.	ID=MALK_01194.t1;Parent=MALK_01194
contig003	AUGUSTUS	exon	705598	707547	0.9	+	.	ID=MALK_01194.t1.e1;Parent=MALK_01194.t1
contig003	maker	gene	707585	708739	.	-	.	ID=MALK_01195;prediction_source=maker_MRET:augustus_masked-contig003-processed-gene-6.74-mRNA-1
contig003	maker	CDS	707585	708739	.	-	0	ID=MALK_01195.t1.c1;Parent=MALK_01195.t1
contig003	maker	mRNA	707585	708739	.	-	.	ID=MALK_01195.t1;Parent=MALK_01195
contig003	maker	exon	707585	708739	.	-	.	ID=MALK_01195.t1.e1;Parent=MALK_01195.t1
contig003	maker	gene	708855	710666	.	+	.	ID=MALK_01196;prediction_source=maker_MRET:augustus_masked-contig003-processed-gene-6.32-mRNA-1
contig003	maker	CDS	708855	710666	.	+	0	ID=MALK_01196.t1.c1;Parent=MALK_01196.t1
contig003	maker	mRNA	708855	710666	.	+	.	ID=MALK_01196.t1;Parent=MALK_01196
contig003	maker	exon	708855	710666	.	+	.	ID=MALK_01196.t1.e1;Parent=MALK_01196.t1
contig003	AUGUSTUS	gene	710663	712156	1	-	.	ID=MALK_01197;prediction_source=augustus:contig003.g3509.t1
contig003	AUGUSTUS	CDS	710663	712156	1	-	0	ID=MALK_01197.t1.c1;Parent=MALK_01197.t1
contig003	AUGUSTUS	mRNA	710663	712156	1	-	.	ID=MALK_01197.t1;Parent=MALK_01197
contig003	AUGUSTUS	exon	710663	712156	1	-	.	ID=MALK_01197.t1.e1;Parent=MALK_01197.t1
contig003	AUGUSTUS	gene	712399	713029	0.45	+	.	ID=MALK_01198;prediction_source=braker_MRET:g3237.t1
contig003	AUGUSTUS	CDS	712399	712414	0.64	+	0	ID=MALK_01198.t1.c1;Parent=MALK_01198.t1
contig003	AUGUSTUS	CDS	712447	712605	0.64	+	0	ID=MALK_01198.t1.c2;Parent=MALK_01198.t1
contig003	AUGUSTUS	CDS	712640	712699	0.64	+	0	ID=MALK_01198.t1.c3;Parent=MALK_01198.t1
contig003	AUGUSTUS	CDS	712730	712940	0.64	+	0	ID=MALK_01198.t1.c4;Parent=MALK_01198.t1
contig003	AUGUSTUS	CDS	712975	713029	0.64	+	0	ID=MALK_01198.t1.c5;Parent=MALK_01198.t1
contig003	AUGUSTUS	mRNA	712399	713029	0.45	+	.	ID=MALK_01198.t1;Parent=MALK_01198
contig003	AUGUSTUS	exon	712399	712414	.	+	.	ID=MALK_01198.t1.e1;Parent=MALK_01198.t1
contig003	AUGUSTUS	exon	712447	712605	.	+	.	ID=MALK_01198.t1.e2;Parent=MALK_01198.t1
contig003	AUGUSTUS	exon	712640	712699	.	+	.	ID=MALK_01198.t1.e3;Parent=MALK_01198.t1
contig003	AUGUSTUS	exon	712730	712940	.	+	.	ID=MALK_01198.t1.e4;Parent=MALK_01198.t1
contig003	AUGUSTUS	exon	712975	713029	.	+	.	ID=MALK_01198.t1.e5;Parent=MALK_01198.t1
contig003	AUGUSTUS	gene	713130	713646	0.92	-	.	ID=MALK_01199;prediction_source=braker_MRET:g3238.t1
contig003	AUGUSTUS	CDS	713644	713646	0.92	-	0	ID=MALK_01199.t1.c2;Parent=MALK_01199.t1
contig003	AUGUSTUS	CDS	713130	713612	0.92	-	0	ID=MALK_01199.t1.c1;Parent=MALK_01199.t1
contig003	AUGUSTUS	mRNA	713130	713646	0.92	-	.	ID=MALK_01199.t1;Parent=MALK_01199
contig003	AUGUSTUS	exon	713644	713646	.	-	.	ID=MALK_01199.t1.e2;Parent=MALK_01199.t1
contig003	AUGUSTUS	exon	713130	713612	.	-	.	ID=MALK_01199.t1.e1;Parent=MALK_01199.t1
contig003	maker	gene	714414	716351	.	+	.	ID=MALK_01200;prediction_source=maker_MRET:augustus_masked-contig003-processed-gene-6.33-mRNA-1
contig003	maker	CDS	714414	716351	.	+	0	ID=MALK_01200.t1.c1;Parent=MALK_01200.t1
contig003	maker	mRNA	714414	716351	.	+	.	ID=MALK_01200.t1;Parent=MALK_01200
contig003	maker	exon	714414	716351	.	+	.	ID=MALK_01200.t1.e1;Parent=MALK_01200.t1
contig003	AUGUSTUS	gene	717029	719566	0.55	+	.	ID=MALK_01201;prediction_source=braker_MRET:g3240.t1
contig003	AUGUSTUS	CDS	717029	717205	0.85	+	0	ID=MALK_01201.t1.c1;Parent=MALK_01201.t1
contig003	AUGUSTUS	CDS	717242	719566	0.85	+	0	ID=MALK_01201.t1.c2;Parent=MALK_01201.t1
contig003	AUGUSTUS	mRNA	717029	719566	0.55	+	.	ID=MALK_01201.t1;Parent=MALK_01201

contig003	AUGUSTUS	exon	717029	717205	.	+	.	ID=MALK_01201.t1.e1;Parent=MALK_01201.t1
contig003	AUGUSTUS	exon	717242	719566	.	+	.	ID=MALK_01201.t1.e2;Parent=MALK_01201.t1
contig003	maker	gene	719585	720358	.	-	.	ID=MALK_01202;prediction_source=maker_MRET:augustus_masked-contig003-processed-gene-6.77-mRNA-1
contig003	maker	CDS	719585	720358	.	-	0	ID=MALK_01202.t1.c1;Parent=MALK_01202.t1
contig003	maker	mRNA	719585	720358	.	-	.	ID=MALK_01202.t1;Parent=MALK_01202
contig003	maker	exon	719585	720358	.	-	.	ID=MALK_01202.t1.e1;Parent=MALK_01202.t1
contig003	AUGUSTUS	gene	720454	721086	0.87	+	.	ID=MALK_01203;prediction_source=braker_MRET:g3242.t1
contig003	AUGUSTUS	CDS	720454	721086	0.87	+	0	ID=MALK_01203.t1.c1;Parent=MALK_01203.t1
contig003	AUGUSTUS	mRNA	720454	721086	0.87	+	.	ID=MALK_01203.t1;Parent=MALK_01203
contig003	AUGUSTUS	exon	720454	721086	.	+	.	ID=MALK_01203.t1.e1;Parent=MALK_01203.t1
contig003	AUGUSTUS	gene	721623	722786	0.97	-	.	ID=MALK_01204;prediction_source=augustus:contig003.g3515.t1
contig003	AUGUSTUS	CDS	721623	722786	0.97	-	0	ID=MALK_01204.t1.c1;Parent=MALK_01204.t1
contig003	AUGUSTUS	mRNA	721623	722786	0.97	-	.	ID=MALK_01204.t1;Parent=MALK_01204
contig003	AUGUSTUS	exon	721623	722786	0.97	-	.	ID=MALK_01204.t1.e1;Parent=MALK_01204.t1
contig003	maker	gene	722891	724078	.	+	.	ID=MALK_01205;prediction_source=maker_MRET:augustus_masked-contig003-processed-gene-6.36-mRNA-1
contig003	maker	CDS	722891	724078	.	+	0	ID=MALK_01205.t1.c1;Parent=MALK_01205.t1
contig003	maker	mRNA	722891	724078	.	+	.	ID=MALK_01205.t1;Parent=MALK_01205
contig003	maker	exon	722891	724078	.	+	.	ID=MALK_01205.t1.e1;Parent=MALK_01205.t1
contig003	maker	gene	724102	725734	.	-	.	ID=MALK_01206;prediction_source=maker_MRET:augustus_masked-contig003-processed-gene-6.79-mRNA-1
contig003	maker	CDS	725679	725734	.	-	0	ID=MALK_01206.t1.c1;Parent=MALK_01206.t1
contig003	maker	CDS	724167	725336	.	-	0	ID=MALK_01206.t1.c2;Parent=MALK_01206.t1
contig003	maker	CDS	724102	724105	.	-	0	ID=MALK_01206.t1.c3;Parent=MALK_01206.t1
contig003	maker	mRNA	724102	725734	.	-	.	ID=MALK_01206.t1;Parent=MALK_01206
contig003	maker	exon	725679	725734	.	-	.	ID=MALK_01206.t1.e1;Parent=MALK_01206.t1
contig003	maker	exon	724167	725336	.	-	.	ID=MALK_01206.t1.e2;Parent=MALK_01206.t1
contig003	maker	exon	724102	724105	.	-	.	ID=MALK_01206.t1.e3;Parent=MALK_01206.t1
contig003	maker	gene	725790	726872	.	+	.	ID=MALK_01207;prediction_source=maker_MRET:augustus_masked-contig003-processed-gene-6.37-mRNA-1
contig003	maker	CDS	725790	726872	.	+	0	ID=MALK_01207.t1.c1;Parent=MALK_01207.t1
contig003	maker	mRNA	725790	726872	.	+	.	ID=MALK_01207.t1;Parent=MALK_01207
contig003	maker	exon	725790	726872	.	+	.	ID=MALK_01207.t1.e1;Parent=MALK_01207.t1
contig003	AUGUSTUS	gene	726972	729119	1	+	.	ID=MALK_01208;prediction_source=augustus:contig003.g3520.t1
contig003	AUGUSTUS	CDS	726972	729119	1	+	0	ID=MALK_01208.t1.c1;Parent=MALK_01208.t1
contig003	AUGUSTUS	mRNA	726972	729119	1	+	.	ID=MALK_01208.t1;Parent=MALK_01208
contig003	AUGUSTUS	exon	726972	729119	1	+	.	ID=MALK_01208.t1.e1;Parent=MALK_01208.t1
contig003	maker	gene	729239	730690	.	+	.	ID=MALK_01209;prediction_source=maker_MRET:augustus_masked-contig003-processed-gene-6.39-mRNA-1
contig003	maker	CDS	729239	730690	.	+	0	ID=MALK_01209.t1.c1;Parent=MALK_01209.t1
contig003	maker	mRNA	729239	730690	.	+	.	ID=MALK_01209.t1;Parent=MALK_01209
contig003	maker	exon	729239	730690	.	+	.	ID=MALK_01209.t1.e1;Parent=MALK_01209.t1
contig003	maker	gene	730749	733037	.	+	.	ID=MALK_01210;prediction_source=maker_MRET:augustus_masked-contig003-processed-gene-6.40-mRNA-1
contig003	maker	CDS	730749	733037	.	+	0	ID=MALK_01210.t1.c1;Parent=MALK_01210.t1
contig003	maker	mRNA	730749	733037	.	+	.	ID=MALK_01210.t1;Parent=MALK_01210
contig003	maker	exon	730749	733037	.	+	.	ID=MALK_01210.t1.e1;Parent=MALK_01210.t1
contig003	maker	gene	733041	734537	.	-	.	ID=MALK_01211;prediction_source=maker_MRET:augustus_masked-contig003-processed-gene-6.80-mRNA-1

contig003	maker	CDS	733041	734537	.	-	0	ID=MALK_01211.t1.c1;Parent=MALK_01211.t1
contig003	maker	mRNA	733041	734537	.	-	.	ID=MALK_01211.t1;Parent=MALK_01211
contig003	maker	exon	733041	734537	.	-	.	ID=MALK_01211.t1.e1;Parent=MALK_01211.t1
contig003	maker	gene	734595	736373	.	+	.	ID=MALK_01212;prediction_source=maker_MRET:augustus_masked-contig003-processed-gene-6.41-mRNA-1
contig003	maker	CDS	734595	736373	.	+	0	ID=MALK_01212.t1.c1;Parent=MALK_01212.t1
contig003	maker	mRNA	734595	736373	.	+	.	ID=MALK_01212.t1;Parent=MALK_01212
contig003	maker	exon	734595	736373	.	+	.	ID=MALK_01212.t1.e1;Parent=MALK_01212.t1
contig003	AUGUSTUS	gene	736609	738630	1	+	.	ID=MALK_01213;prediction_source=augustus:contig003.g3527.t1
contig003	AUGUSTUS	CDS	736609	738630	1	+	0	ID=MALK_01213.t1.c1;Parent=MALK_01213.t1
contig003	AUGUSTUS	mRNA	736609	738630	1	+	.	ID=MALK_01213.t1;Parent=MALK_01213
contig003	AUGUSTUS	exon	736609	738630	1	+	.	ID=MALK_01213.t1.e1;Parent=MALK_01213.t1
contig003	AUGUSTUS	gene	738737	739996	0.81	-	.	ID=MALK_01214;prediction_source=augustus:contig003.g3529.t1
contig003	AUGUSTUS	CDS	738737	739996	0.81	-	0	ID=MALK_01214.t1.c1;Parent=MALK_01214.t1
contig003	AUGUSTUS	mRNA	738737	739996	0.81	-	.	ID=MALK_01214.t1;Parent=MALK_01214
contig003	AUGUSTUS	exon	738737	739996	0.81	-	.	ID=MALK_01214.t1.e1;Parent=MALK_01214.t1
contig003	maker	gene	740533	741315	.	+	.	ID=MALK_01215;prediction_source=maker_MRET:augustus_masked-contig003-processed-gene-6.43-mRNA-1
contig003	maker	CDS	740533	741315	.	+	0	ID=MALK_01215.t1.c1;Parent=MALK_01215.t1
contig003	maker	mRNA	740533	741315	.	+	.	ID=MALK_01215.t1;Parent=MALK_01215
contig003	maker	exon	740533	741315	.	+	.	ID=MALK_01215.t1.e1;Parent=MALK_01215.t1
contig003	maker	gene	741693	742766	.	+	.	ID=MALK_01216;prediction_source=maker_MRET:augustus_masked-contig003-processed-gene-6.44-mRNA-1
contig003	maker	CDS	741693	742766	.	+	0	ID=MALK_01216.t1.c1;Parent=MALK_01216.t1
contig003	maker	mRNA	741693	742766	.	+	.	ID=MALK_01216.t1;Parent=MALK_01216
contig003	maker	exon	741693	742766	.	+	.	ID=MALK_01216.t1.e1;Parent=MALK_01216.t1
contig003	maker	gene	742793	745564	.	-	.	ID=MALK_01217;prediction_source=maker_MRET:augustus_masked-contig003-processed-gene-6.82-mRNA-1
contig003	maker	CDS	742793	745564	.	-	0	ID=MALK_01217.t1.c1;Parent=MALK_01217.t1
contig003	maker	mRNA	742793	745564	.	-	.	ID=MALK_01217.t1;Parent=MALK_01217
contig003	maker	exon	742793	745564	.	-	.	ID=MALK_01217.t1.e1;Parent=MALK_01217.t1
contig003	AUGUSTUS	gene	745891	747255	0.63	+	.	ID=MALK_01218;prediction_source=augustus:contig003.g3535.t1
contig003	AUGUSTUS	CDS	745891	747255	0.63	+	0	ID=MALK_01218.t1.c1;Parent=MALK_01218.t1
contig003	AUGUSTUS	mRNA	745891	747255	0.63	+	.	ID=MALK_01218.t1;Parent=MALK_01218
contig003	AUGUSTUS	exon	745891	747255	0.63	+	.	ID=MALK_01218.t1.e1;Parent=MALK_01218.t1
contig003	AUGUSTUS	gene	747259	748842	0.91	-	.	ID=MALK_01219;prediction_source=augustus:contig003.g3537.t1
contig003	AUGUSTUS	CDS	747259	748842	0.91	-	0	ID=MALK_01219.t1.c1;Parent=MALK_01219.t1
contig003	AUGUSTUS	mRNA	747259	748842	0.91	-	.	ID=MALK_01219.t1;Parent=MALK_01219
contig003	AUGUSTUS	exon	747259	748842	0.91	-	.	ID=MALK_01219.t1.e1;Parent=MALK_01219.t1
contig003	AUGUSTUS	gene	748970	749914	0.93	+	.	ID=MALK_01220;prediction_source=augustus:contig003.g3538.t1
contig003	AUGUSTUS	CDS	748970	749914	0.93	+	0	ID=MALK_01220.t1.c1;Parent=MALK_01220.t1
contig003	AUGUSTUS	mRNA	748970	749914	0.93	+	.	ID=MALK_01220.t1;Parent=MALK_01220
contig003	AUGUSTUS	exon	748970	749914	0.93	+	.	ID=MALK_01220.t1.e1;Parent=MALK_01220.t1
contig003	maker	gene	749925	750722	.	-	.	ID=MALK_01221;prediction_source=maker_MRET:augustus_masked-contig003-processed-gene-6.84-mRNA-1
contig003	maker	CDS	749925	750722	.	-	0	ID=MALK_01221.t1.c1;Parent=MALK_01221.t1
contig003	maker	mRNA	749925	750722	.	-	.	ID=MALK_01221.t1;Parent=MALK_01221
contig003	maker	exon	749925	750722	.	-	.	ID=MALK_01221.t1.e1;Parent=MALK_01221.t1

contig003	AUGUSTUS	gene	751055	753547	0.6	+	.	ID=MALK_01222;prediction_source=augustus:contig003.g3540.t1
contig003	AUGUSTUS	CDS	751055	753547	0.6	+	0	ID=MALK_01222.t1.c1;Parent=MALK_01222.t1
contig003	AUGUSTUS	mRNA	751055	753547	0.6	+	.	ID=MALK_01222.t1;Parent=MALK_01222
contig003	AUGUSTUS	exon	751055	753547	0.6	+	.	ID=MALK_01222.t1.e1;Parent=MALK_01222.t1
contig003	AUGUSTUS	gene	753619	754842	0.75	-	.	ID=MALK_01223;prediction_source=braker_MRET:g3261.t1
contig003	AUGUSTUS	CDS	753619	754842	0.75	-	0	ID=MALK_01223.t1.c1;Parent=MALK_01223.t1
contig003	AUGUSTUS	mRNA	753619	754842	0.75	-	.	ID=MALK_01223.t1;Parent=MALK_01223
contig003	AUGUSTUS	exon	753619	754842	.	-	.	ID=MALK_01223.t1.e1;Parent=MALK_01223.t1
contig003	maker	gene	755044	756396	.	+	.	ID=MALK_01224;prediction_source=maker_MRET:augustus_masked-contig003-processed-gene-6.48-mRNA-1
contig003	maker	CDS	755044	756396	.	+	0	ID=MALK_01224.t1.c1;Parent=MALK_01224.t1
contig003	maker	mRNA	755044	756396	.	+	.	ID=MALK_01224.t1;Parent=MALK_01224
contig003	maker	exon	755044	756396	.	+	.	ID=MALK_01224.t1.e1;Parent=MALK_01224.t1
contig003	AUGUSTUS	gene	756808	758223	0.93	+	.	ID=MALK_01225;prediction_source=augustus:contig003.g3545.t1
contig003	AUGUSTUS	CDS	756808	758223	0.93	+	0	ID=MALK_01225.t1.c1;Parent=MALK_01225.t1
contig003	AUGUSTUS	mRNA	756808	758223	0.93	+	.	ID=MALK_01225.t1;Parent=MALK_01225
contig003	AUGUSTUS	exon	756808	758223	0.93	+	.	ID=MALK_01225.t1.e1;Parent=MALK_01225.t1
contig003	maker	gene	758386	760275	.	+	.	ID=MALK_01226;prediction_source=maker_MRET:augustus_masked-contig003-processed-gene-6.50-mRNA-1
contig003	maker	CDS	758386	760275	.	+	0	ID=MALK_01226.t1.c1;Parent=MALK_01226.t1
contig003	maker	mRNA	758386	760275	.	+	.	ID=MALK_01226.t1;Parent=MALK_01226
contig003	maker	exon	758386	760275	.	+	.	ID=MALK_01226.t1.e1;Parent=MALK_01226.t1
contig003	maker	gene	760302	764282	.	-	.	ID=MALK_01227;prediction_source=maker_MRET:augustus_masked-contig003-processed-gene-6.86-mRNA-1
contig003	maker	CDS	760302	764282	.	-	0	ID=MALK_01227.t1.c1;Parent=MALK_01227.t1
contig003	maker	mRNA	760302	764282	.	-	.	ID=MALK_01227.t1;Parent=MALK_01227
contig003	maker	exon	760302	764282	.	-	.	ID=MALK_01227.t1.e1;Parent=MALK_01227.t1
contig003	maker	gene	764461	767142	.	+	.	ID=MALK_01228;prediction_source=maker_MRET:augustus_masked-contig003-processed-gene-6.51-mRNA-1
contig003	maker	CDS	764461	767142	.	+	0	ID=MALK_01228.t1.c1;Parent=MALK_01228.t1
contig003	maker	mRNA	764461	767142	.	+	.	ID=MALK_01228.t1;Parent=MALK_01228
contig003	maker	exon	764461	767142	.	+	.	ID=MALK_01228.t1.e1;Parent=MALK_01228.t1
contig003	AUGUSTUS	gene	767120	768772	0.98	-	.	ID=MALK_01229;prediction_source=braker_MRET:g3267.t1
contig003	AUGUSTUS	CDS	767120	768772	0.98	-	0	ID=MALK_01229.t1.c1;Parent=MALK_01229.t1
contig003	AUGUSTUS	mRNA	767120	768772	0.98	-	.	ID=MALK_01229.t1;Parent=MALK_01229
contig003	AUGUSTUS	exon	767120	768772	.	-	.	ID=MALK_01229.t1.e1;Parent=MALK_01229.t1
contig003	AUGUSTUS	gene	768873	770613	0.14	-	.	ID=MALK_01230;prediction_source=augustus:contig003.g3551.t1
contig003	AUGUSTUS	CDS	769813	770613	0.23	-	0	ID=MALK_01230.t1.c2;Parent=MALK_01230.t1
contig003	AUGUSTUS	CDS	768873	769502	0.23	-	0	ID=MALK_01230.t1.c1;Parent=MALK_01230.t1
contig003	AUGUSTUS	mRNA	768873	770613	0.14	-	.	ID=MALK_01230.t1;Parent=MALK_01230
contig003	AUGUSTUS	exon	769813	770613	0.23	-	.	ID=MALK_01230.t1.e2;Parent=MALK_01230.t1
contig003	AUGUSTUS	exon	768873	769502	0.23	-	.	ID=MALK_01230.t1.e1;Parent=MALK_01230.t1
contig004	annotation	remark	1	523505	.	+	.	gff-version=3
contig004	AUGUSTUS	gene	2	3509	0.61	+	.	ID=MALK_01231;prediction_source=braker_MRET:g397.t1
contig004	AUGUSTUS	CDS	2	3509	0.61	+	1	ID=MALK_01231.t1.c1;Parent=MALK_01231.t1
contig004	AUGUSTUS	mRNA	2	3509	0.61	+	.	ID=MALK_01231.t1;Parent=MALK_01231
contig004	AUGUSTUS	exon	2	3509	.	+	.	ID=MALK_01231.t1.e1;Parent=MALK_01231.t1

contig004	AUGUSTUS	gene	4033	5532	0.92	+	.	ID=MALK_01232;prediction_source=braker_MRET:g398.t1
contig004	AUGUSTUS	CDS	4033	5532	0.92	+	0	ID=MALK_01232.t1.c1;Parent=MALK_01232.t1
contig004	AUGUSTUS	mRNA	4033	5532	0.92	+	.	ID=MALK_01232.t1;Parent=MALK_01232
contig004	AUGUSTUS	exon	4033	5532	.	+	.	ID=MALK_01232.t1.e1;Parent=MALK_01232.t1
contig004	AUGUSTUS	gene	5595	6126	0.93	-	.	ID=MALK_01233;prediction_source=braker_MRET:g399.t1
contig004	AUGUSTUS	CDS	5660	6126	0.93	-	0	ID=MALK_01233.t1.c2;Parent=MALK_01233.t1
contig004	AUGUSTUS	CDS	5595	5631	0.93	-	0	ID=MALK_01233.t1.c1;Parent=MALK_01233.t1
contig004	AUGUSTUS	mRNA	5595	6126	0.93	-	.	ID=MALK_01233.t1;Parent=MALK_01233
contig004	AUGUSTUS	exon	5660	6126	.	-	.	ID=MALK_01233.t1.e2;Parent=MALK_01233.t1
contig004	AUGUSTUS	exon	5595	5631	.	-	.	ID=MALK_01233.t1.e1;Parent=MALK_01233.t1
contig004	maker	gene	6358	8082	.	+	.	ID=MALK_01234;prediction_source=maker_MRET:augustus_masked-contig004-processed-gene-0.46-mRNA-1
contig004	maker	CDS	6358	8082	.	+	0	ID=MALK_01234.t1.c1;Parent=MALK_01234.t1
contig004	maker	mRNA	6358	8082	.	+	.	ID=MALK_01234.t1;Parent=MALK_01234
contig004	maker	exon	6358	8082	.	+	.	ID=MALK_01234.t1.e1;Parent=MALK_01234.t1
contig004	AUGUSTUS	gene	8170	9720	0.77	+	.	ID=MALK_01235;prediction_source=augustus:contig004.g5.t1
contig004	AUGUSTUS	CDS	8170	9720	0.77	+	0	ID=MALK_01235.t1.c1;Parent=MALK_01235.t1
contig004	AUGUSTUS	mRNA	8170	9720	0.77	+	.	ID=MALK_01235.t1;Parent=MALK_01235
contig004	AUGUSTUS	exon	8170	9720	0.77	+	.	ID=MALK_01235.t1.e1;Parent=MALK_01235.t1
contig004	AUGUSTUS	gene	9732	10775	0.98	-	.	ID=MALK_01236;prediction_source=augustus:contig004.g6.t1
contig004	AUGUSTUS	CDS	9732	10775	0.98	-	0	ID=MALK_01236.t1.c1;Parent=MALK_01236.t1
contig004	AUGUSTUS	mRNA	9732	10775	0.98	-	.	ID=MALK_01236.t1;Parent=MALK_01236
contig004	AUGUSTUS	exon	9732	10775	0.98	-	.	ID=MALK_01236.t1.e1;Parent=MALK_01236.t1
contig004	AUGUSTUS	gene	10861	11669	0.92	+	.	ID=MALK_01237;prediction_source=braker_MRET:g403.t1
contig004	AUGUSTUS	CDS	10861	10900	1	+	0	ID=MALK_01237.t1.c1;Parent=MALK_01237.t1
contig004	AUGUSTUS	CDS	10929	11148	1	+	0	ID=MALK_01237.t1.c2;Parent=MALK_01237.t1
contig004	AUGUSTUS	CDS	11177	11669	1	+	0	ID=MALK_01237.t1.c3;Parent=MALK_01237.t1
contig004	AUGUSTUS	mRNA	10861	11669	0.92	+	.	ID=MALK_01237.t1;Parent=MALK_01237
contig004	AUGUSTUS	exon	10861	10900	.	+	.	ID=MALK_01237.t1.e1;Parent=MALK_01237.t1
contig004	AUGUSTUS	exon	10929	11148	.	+	.	ID=MALK_01237.t1.e2;Parent=MALK_01237.t1
contig004	AUGUSTUS	exon	11177	11669	.	+	.	ID=MALK_01237.t1.e3;Parent=MALK_01237.t1
contig004	AUGUSTUS	gene	11756	12302	1	-	.	ID=MALK_01238;prediction_source=braker_MRET:g404.t1
contig004	AUGUSTUS	CDS	12071	12302	1	-	0	ID=MALK_01238.t1.c2;Parent=MALK_01238.t1
contig004	AUGUSTUS	CDS	11756	12033	1	-	0	ID=MALK_01238.t1.c1;Parent=MALK_01238.t1
contig004	AUGUSTUS	mRNA	11756	12302	1	-	.	ID=MALK_01238.t1;Parent=MALK_01238
contig004	AUGUSTUS	exon	12071	12302	.	-	.	ID=MALK_01238.t1.e2;Parent=MALK_01238.t1
contig004	AUGUSTUS	exon	11756	12033	.	-	.	ID=MALK_01238.t1.e1;Parent=MALK_01238.t1
contig004	AUGUSTUS	gene	12540	12875	0.51	+	.	ID=MALK_01239;prediction_source=braker_MRET:g405.t1
contig004	AUGUSTUS	CDS	12540	12750	1	+	0	ID=MALK_01239.t1.c1;Parent=MALK_01239.t1
contig004	AUGUSTUS	CDS	12802	12875	1	+	0	ID=MALK_01239.t1.c2;Parent=MALK_01239.t1
contig004	AUGUSTUS	mRNA	12540	12875	0.51	+	.	ID=MALK_01239.t1;Parent=MALK_01239
contig004	AUGUSTUS	exon	12540	12750	.	+	.	ID=MALK_01239.t1.e1;Parent=MALK_01239.t1
contig004	AUGUSTUS	exon	12802	12875	.	+	.	ID=MALK_01239.t1.e2;Parent=MALK_01239.t1
contig004	AUGUSTUS	gene	13036	15081	0.86	-	.	ID=MALK_01240;prediction_source=augustus:contig004.g10.t1

contig004	AUGUSTUS	CDS	13036	15081	0.86	-	0	ID=MALK_01240.t1.c1;Parent=MALK_01240.t1
contig004	AUGUSTUS	mRNA	13036	15081	0.86	-	.	ID=MALK_01240.t1;Parent=MALK_01240
contig004	AUGUSTUS	exon	13036	15081	0.86	-	.	ID=MALK_01240.t1.e1;Parent=MALK_01240.t1
contig004	AUGUSTUS	gene	15654	16008	0.42	-	.	ID=MALK_01241;prediction_source=braker_MRET:g407.t1
contig004	AUGUSTUS	CDS	15931	16008	0.47	-	0	ID=MALK_01241.t1.c3;Parent=MALK_01241.t1
contig004	AUGUSTUS	CDS	15830	15895	0.47	-	0	ID=MALK_01241.t1.c2;Parent=MALK_01241.t1
contig004	AUGUSTUS	CDS	15654	15791	0.47	-	0	ID=MALK_01241.t1.c1;Parent=MALK_01241.t1
contig004	AUGUSTUS	mRNA	15654	16008	0.42	-	.	ID=MALK_01241.t1;Parent=MALK_01241
contig004	AUGUSTUS	exon	15931	16008	.	-	.	ID=MALK_01241.t1.e3;Parent=MALK_01241.t1
contig004	AUGUSTUS	exon	15830	15895	.	-	.	ID=MALK_01241.t1.e2;Parent=MALK_01241.t1
contig004	AUGUSTUS	exon	15654	15791	.	-	.	ID=MALK_01241.t1.e1;Parent=MALK_01241.t1
contig004	maker	gene	16169	17209	.	+	.	ID=MALK_01242;prediction_source=maker_MRET:augustus_masked-contig004-processed-gene-0.50-mRNA-1
contig004	maker	CDS	16169	17209	.	+	0	ID=MALK_01242.t1.c1;Parent=MALK_01242.t1
contig004	maker	mRNA	16169	17209	.	+	.	ID=MALK_01242.t1;Parent=MALK_01242
contig004	maker	exon	16169	17209	.	+	.	ID=MALK_01242.t1.e1;Parent=MALK_01242.t1
contig004	AUGUSTUS	gene	17350	20547	1	-	.	ID=MALK_01243;prediction_source=augustus:contig004.g13.t1
contig004	AUGUSTUS	CDS	17350	20547	1	-	0	ID=MALK_01243.t1.c1;Parent=MALK_01243.t1
contig004	AUGUSTUS	mRNA	17350	20547	1	-	.	ID=MALK_01243.t1;Parent=MALK_01243
contig004	AUGUSTUS	exon	17350	20547	1	-	.	ID=MALK_01243.t1.e1;Parent=MALK_01243.t1
contig004	maker	gene	20879	23371	.	-	.	ID=MALK_01244;prediction_source=maker_MRET:augustus_masked-contig004-processed-gene-0.72-mRNA-1
contig004	maker	CDS	20879	23371	.	-	0	ID=MALK_01244.t1.c1;Parent=MALK_01244.t1
contig004	maker	mRNA	20879	23371	.	-	.	ID=MALK_01244.t1;Parent=MALK_01244
contig004	maker	exon	20879	23371	.	-	.	ID=MALK_01244.t1.e1;Parent=MALK_01244.t1
contig004	AUGUSTUS	gene	23517	24446	0.63	+	.	ID=MALK_01245;prediction_source=braker_MRET:g411.t1
contig004	AUGUSTUS	CDS	23517	24446	0.63	+	0	ID=MALK_01245.t1.c1;Parent=MALK_01245.t1
contig004	AUGUSTUS	mRNA	23517	24446	0.63	+	.	ID=MALK_01245.t1;Parent=MALK_01245
contig004	AUGUSTUS	exon	23517	24446	.	+	.	ID=MALK_01245.t1.e1;Parent=MALK_01245.t1
contig004	AUGUSTUS	gene	24575	26806	0.68	+	.	ID=MALK_01246;prediction_source=augustus:contig004.g16.t1
contig004	AUGUSTUS	CDS	24575	26806	0.68	+	0	ID=MALK_01246.t1.c1;Parent=MALK_01246.t1
contig004	AUGUSTUS	mRNA	24575	26806	0.68	+	.	ID=MALK_01246.t1;Parent=MALK_01246
contig004	AUGUSTUS	exon	24575	26806	0.68	+	.	ID=MALK_01246.t1.e1;Parent=MALK_01246.t1
contig004	AUGUSTUS	gene	26861	27289	0.9	+	.	ID=MALK_01247;prediction_source=braker_MRET:g413.t1
contig004	AUGUSTUS	CDS	26861	27289	0.9	+	0	ID=MALK_01247.t1.c1;Parent=MALK_01247.t1
contig004	AUGUSTUS	mRNA	26861	27289	0.9	+	.	ID=MALK_01247.t1;Parent=MALK_01247
contig004	AUGUSTUS	exon	26861	27289	.	+	.	ID=MALK_01247.t1.e1;Parent=MALK_01247.t1
contig004	AUGUSTUS	gene	27291	29057	1	-	.	ID=MALK_01248;prediction_source=augustus:contig004.g19.t1
contig004	AUGUSTUS	CDS	27291	29057	1	-	0	ID=MALK_01248.t1.c1;Parent=MALK_01248.t1
contig004	AUGUSTUS	mRNA	27291	29057	1	-	.	ID=MALK_01248.t1;Parent=MALK_01248
contig004	AUGUSTUS	exon	27291	29057	1	-	.	ID=MALK_01248.t1.e1;Parent=MALK_01248.t1
contig004	AUGUSTUS	gene	29358	31985	0.95	+	.	ID=MALK_01249;prediction_source=augustus:contig004.g20.t1
contig004	AUGUSTUS	CDS	29358	31985	0.95	+	0	ID=MALK_01249.t1.c1;Parent=MALK_01249.t1
contig004	AUGUSTUS	mRNA	29358	31985	0.95	+	.	ID=MALK_01249.t1;Parent=MALK_01249
contig004	AUGUSTUS	exon	29358	31985	0.95	+	.	ID=MALK_01249.t1.e1;Parent=MALK_01249.t1

contig004	AUGUSTUS	gene	32230	36181	0.36	-	.	ID=MALK_01250;prediction_source=braker_MRET:g416.t1
contig004	AUGUSTUS	CDS	36080	36181	0.39	-	0	ID=MALK_01250.t1.c3;Parent=MALK_01250.t1
contig004	AUGUSTUS	CDS	35919	35992	0.39	-	0	ID=MALK_01250.t1.c2;Parent=MALK_01250.t1
contig004	AUGUSTUS	CDS	32230	35890	0.39	-	0	ID=MALK_01250.t1.c1;Parent=MALK_01250.t1
contig004	AUGUSTUS	mRNA	32230	36181	0.36	-	.	ID=MALK_01250.t1;Parent=MALK_01250
contig004	AUGUSTUS	exon	36080	36181	.	-	.	ID=MALK_01250.t1.e3;Parent=MALK_01250.t1
contig004	AUGUSTUS	exon	35919	35992	.	-	.	ID=MALK_01250.t1.e2;Parent=MALK_01250.t1
contig004	AUGUSTUS	exon	32230	35890	.	-	.	ID=MALK_01250.t1.e1;Parent=MALK_01250.t1
contig004	maker	gene	36209	37123	.	+	.	ID=MALK_01251;prediction_source=maker_MRET:augustus_masked-contig004-processed-gene-0.55-mRNA-1
contig004	maker	CDS	36209	37123	.	+	0	ID=MALK_01251.t1.c1;Parent=MALK_01251.t1
contig004	maker	mRNA	36209	37123	.	+	.	ID=MALK_01251.t1;Parent=MALK_01251
contig004	maker	exon	36209	37123	.	+	.	ID=MALK_01251.t1.e1;Parent=MALK_01251.t1
contig004	AUGUSTUS	gene	37195	37611	0.87	-	.	ID=MALK_01252;prediction_source=augustus:contig004.g25.t1
contig004	AUGUSTUS	CDS	37195	37611	0.87	-	0	ID=MALK_01252.t1.c1;Parent=MALK_01252.t1
contig004	AUGUSTUS	mRNA	37195	37611	0.87	-	.	ID=MALK_01252.t1;Parent=MALK_01252
contig004	AUGUSTUS	exon	37195	37611	0.87	-	.	ID=MALK_01252.t1.e1;Parent=MALK_01252.t1
contig004	maker	gene	37683	40511	.	-	.	ID=MALK_01253;prediction_source=maker_MRET:augustus_masked-contig004-processed-gene-0.76-mRNA-1
contig004	maker	CDS	37683	40511	.	-	0	ID=MALK_01253.t1.c1;Parent=MALK_01253.t1
contig004	maker	mRNA	37683	40511	.	-	.	ID=MALK_01253.t1;Parent=MALK_01253
contig004	maker	exon	37683	40511	.	-	.	ID=MALK_01253.t1.e1;Parent=MALK_01253.t1
contig004	AUGUSTUS	gene	40934	44992	0.48	+	.	ID=MALK_01254;prediction_source=augustus:contig004.g27.t1
contig004	AUGUSTUS	CDS	40934	44992	0.48	+	0	ID=MALK_01254.t1.c1;Parent=MALK_01254.t1
contig004	AUGUSTUS	mRNA	40934	44992	0.48	+	.	ID=MALK_01254.t1;Parent=MALK_01254
contig004	AUGUSTUS	exon	40934	44992	0.48	+	.	ID=MALK_01254.t1.e1;Parent=MALK_01254.t1
contig004	maker	gene	44994	46457	.	-	.	ID=MALK_01255;prediction_source=maker_MRET:augustus_masked-contig004-processed-gene-0.77-mRNA-1
contig004	maker	CDS	44994	46457	.	-	0	ID=MALK_01255.t1.c1;Parent=MALK_01255.t1
contig004	maker	mRNA	44994	46457	.	-	.	ID=MALK_01255.t1;Parent=MALK_01255
contig004	maker	exon	44994	46457	.	-	.	ID=MALK_01255.t1.e1;Parent=MALK_01255.t1
contig004	AUGUSTUS	gene	46566	47275	0.64	+	.	ID=MALK_01256;prediction_source=braker_MRET:g421.t1
contig004	AUGUSTUS	CDS	46566	46571	0.99	+	0	ID=MALK_01256.t1.c1;Parent=MALK_01256.t1
contig004	AUGUSTUS	CDS	46600	46603	0.99	+	0	ID=MALK_01256.t1.c2;Parent=MALK_01256.t1
contig004	AUGUSTUS	CDS	46632	47275	0.99	+	0	ID=MALK_01256.t1.c3;Parent=MALK_01256.t1
contig004	AUGUSTUS	mRNA	46566	47275	0.64	+	.	ID=MALK_01256.t1;Parent=MALK_01256
contig004	AUGUSTUS	exon	46566	46571	.	+	.	ID=MALK_01256.t1.e1;Parent=MALK_01256.t1
contig004	AUGUSTUS	exon	46600	46603	.	+	.	ID=MALK_01256.t1.e2;Parent=MALK_01256.t1
contig004	AUGUSTUS	exon	46632	47275	.	+	.	ID=MALK_01256.t1.e3;Parent=MALK_01256.t1
contig004	AUGUSTUS	gene	47348	49258	0.99	+	.	ID=MALK_01257;prediction_source=braker_MRET:g422.t1
contig004	AUGUSTUS	CDS	47348	49258	0.99	+	0	ID=MALK_01257.t1.c1;Parent=MALK_01257.t1
contig004	AUGUSTUS	mRNA	47348	49258	0.99	+	.	ID=MALK_01257.t1;Parent=MALK_01257
contig004	AUGUSTUS	exon	47348	49258	.	+	.	ID=MALK_01257.t1.e1;Parent=MALK_01257.t1
contig004	AUGUSTUS	gene	49354	51000	1	+	.	ID=MALK_01258;prediction_source=braker_MRET:g423.t1
contig004	AUGUSTUS	CDS	49354	51000	1	+	0	ID=MALK_01258.t1.c1;Parent=MALK_01258.t1
contig004	AUGUSTUS	mRNA	49354	51000	1	+	.	ID=MALK_01258.t1;Parent=MALK_01258

contig004	AUGUSTUS	exon	49354	51000	.	+	.	ID=MALK_01258.t1.e1;Parent=MALK_01258.t1
contig004	AUGUSTUS	gene	51013	51630	0.79	-	.	ID=MALK_01259;prediction_source=braker_MRET:g424.t1
contig004	AUGUSTUS	CDS	51013	51630	0.79	-	0	ID=MALK_01259.t1.c1;Parent=MALK_01259.t1
contig004	AUGUSTUS	mRNA	51013	51630	0.79	-	.	ID=MALK_01259.t1;Parent=MALK_01259
contig004	AUGUSTUS	exon	51013	51630	.	-	.	ID=MALK_01259.t1.e1;Parent=MALK_01259.t1
contig004	AUGUSTUS	gene	51747	52973	0.55	+	.	ID=MALK_01260;prediction_source=braker_MRET:g425.t1
contig004	AUGUSTUS	CDS	51747	51863	0.68	+	0	ID=MALK_01260.t1.c1;Parent=MALK_01260.t1
contig004	AUGUSTUS	CDS	51945	52973	0.68	+	0	ID=MALK_01260.t1.c2;Parent=MALK_01260.t1
contig004	AUGUSTUS	mRNA	51747	52973	0.55	+	.	ID=MALK_01260.t1;Parent=MALK_01260
contig004	AUGUSTUS	exon	51747	51863	.	+	.	ID=MALK_01260.t1.e1;Parent=MALK_01260.t1
contig004	AUGUSTUS	exon	51945	52973	.	+	.	ID=MALK_01260.t1.e2;Parent=MALK_01260.t1
contig004	maker	gene	52994	54106	.	-	.	ID=MALK_01261;prediction_source=maker_MRET:augustus_masked-contig004-processed-gene-0.78-mRNA-1
contig004	maker	CDS	52994	54106	.	-	0	ID=MALK_01261.t1.c1;Parent=MALK_01261.t1
contig004	maker	mRNA	52994	54106	.	-	.	ID=MALK_01261.t1;Parent=MALK_01261
contig004	maker	exon	52994	54106	.	-	.	ID=MALK_01261.t1.e1;Parent=MALK_01261.t1
contig004	AUGUSTUS	gene	54168	55361	1	+	.	ID=MALK_01262;prediction_source=augustus:contig004.g33.t1
contig004	AUGUSTUS	CDS	54168	55361	1	+	0	ID=MALK_01262.t1.c1;Parent=MALK_01262.t1
contig004	AUGUSTUS	mRNA	54168	55361	1	+	.	ID=MALK_01262.t1;Parent=MALK_01262
contig004	AUGUSTUS	exon	54168	55361	1	+	.	ID=MALK_01262.t1.e1;Parent=MALK_01262.t1
contig004	AUGUSTUS	gene	55415	57058	0.85	-	.	ID=MALK_01263;prediction_source=augustus:contig004.g34.t1
contig004	AUGUSTUS	CDS	55415	57058	0.85	-	0	ID=MALK_01263.t1.c1;Parent=MALK_01263.t1
contig004	AUGUSTUS	mRNA	55415	57058	0.85	-	.	ID=MALK_01263.t1;Parent=MALK_01263
contig004	AUGUSTUS	exon	55415	57058	0.85	-	.	ID=MALK_01263.t1.e1;Parent=MALK_01263.t1
contig004	maker	gene	57642	58865	.	-	.	ID=MALK_01264;prediction_source=maker_MRET:augustus_masked-contig004-processed-gene-0.80-mRNA-1
contig004	maker	CDS	57642	58865	.	-	0	ID=MALK_01264.t1.c1;Parent=MALK_01264.t1
contig004	maker	mRNA	57642	58865	.	-	.	ID=MALK_01264.t1;Parent=MALK_01264
contig004	maker	exon	57642	58865	.	-	.	ID=MALK_01264.t1.e1;Parent=MALK_01264.t1
contig004	maker	gene	59030	59887	.	-	.	ID=MALK_01265;prediction_source=maker_MRET:augustus_masked-contig004-processed-gene-0.81-mRNA-1
contig004	maker	CDS	59030	59887	.	-	0	ID=MALK_01265.t1.c1;Parent=MALK_01265.t1
contig004	maker	mRNA	59030	59887	.	-	.	ID=MALK_01265.t1;Parent=MALK_01265
contig004	maker	exon	59030	59887	.	-	.	ID=MALK_01265.t1.e1;Parent=MALK_01265.t1
contig004	maker	gene	60181	61692	.	+	.	ID=MALK_01266;prediction_source=maker_MRET:augustus_masked-contig004-processed-gene-0.59-mRNA-1
contig004	maker	CDS	60181	61692	.	+	0	ID=MALK_01266.t1.c1;Parent=MALK_01266.t1
contig004	maker	mRNA	60181	61692	.	+	.	ID=MALK_01266.t1;Parent=MALK_01266
contig004	maker	exon	60181	61692	.	+	.	ID=MALK_01266.t1.e1;Parent=MALK_01266.t1
contig004	maker	gene	61983	62864	.	-	.	ID=MALK_01267;prediction_source=maker_MRET:augustus_masked-contig004-processed-gene-0.82-mRNA-1
contig004	maker	CDS	61983	62864	.	-	0	ID=MALK_01267.t1.c1;Parent=MALK_01267.t1
contig004	maker	mRNA	61983	62864	.	-	.	ID=MALK_01267.t1;Parent=MALK_01267
contig004	maker	exon	61983	62864	.	-	.	ID=MALK_01267.t1.e1;Parent=MALK_01267.t1
contig004	maker	gene	63151	64305	.	+	.	ID=MALK_01268;prediction_source=maker_MRET:augustus_masked-contig004-processed-gene-0.60-mRNA-1
contig004	maker	CDS	63151	64305	.	+	0	ID=MALK_01268.t1.c1;Parent=MALK_01268.t1
contig004	maker	mRNA	63151	64305	.	+	.	ID=MALK_01268.t1;Parent=MALK_01268
contig004	maker	exon	63151	64305	.	+	.	ID=MALK_01268.t1.e1;Parent=MALK_01268.t1

contig004	AUGUSTUS	gene	64340	65926	1	-	.	ID=MALK_01269;prediction_source=braker_MRET:g433.t1
contig004	AUGUSTUS	CDS	65871	65926	1	-	0	ID=MALK_01269.t1.c3;Parent=MALK_01269.t1
contig004	AUGUSTUS	CDS	65797	65841	1	-	0	ID=MALK_01269.t1.c2;Parent=MALK_01269.t1
contig004	AUGUSTUS	CDS	64340	65756	1	-	0	ID=MALK_01269.t1.c1;Parent=MALK_01269.t1
contig004	AUGUSTUS	mRNA	64340	65926	1	-	.	ID=MALK_01269.t1;Parent=MALK_01269
contig004	AUGUSTUS	exon	65871	65926	.	-	.	ID=MALK_01269.t1.e3;Parent=MALK_01269.t1
contig004	AUGUSTUS	exon	65797	65841	.	-	.	ID=MALK_01269.t1.e2;Parent=MALK_01269.t1
contig004	AUGUSTUS	exon	64340	65756	.	-	.	ID=MALK_01269.t1.e1;Parent=MALK_01269.t1
contig004	AUGUSTUS	gene	66728	67180	0.91	-	.	ID=MALK_01270;prediction_source=braker_MRET:g434.t1
contig004	AUGUSTUS	CDS	66728	67180	0.91	-	0	ID=MALK_01270.t1.c1;Parent=MALK_01270.t1
contig004	AUGUSTUS	mRNA	66728	67180	0.91	-	.	ID=MALK_01270.t1;Parent=MALK_01270
contig004	AUGUSTUS	exon	66728	67180	.	-	.	ID=MALK_01270.t1.e1;Parent=MALK_01270.t1
contig004	AUGUSTUS	gene	67297	68169	0.5	+	.	ID=MALK_01271;prediction_source=augustus:contig004.g43.t1
contig004	AUGUSTUS	CDS	67297	68169	0.5	+	0	ID=MALK_01271.t1.c1;Parent=MALK_01271.t1
contig004	AUGUSTUS	mRNA	67297	68169	0.5	+	.	ID=MALK_01271.t1;Parent=MALK_01271
contig004	AUGUSTUS	exon	67297	68169	0.5	+	.	ID=MALK_01271.t1.e1;Parent=MALK_01271.t1
contig004	maker	gene	68219	70342	.	-	.	ID=MALK_01272;prediction_source=maker_MRET:augustus_masked-contig004-processed-gene-0.84-mRNA-1
contig004	maker	CDS	68219	70342	.	-	0	ID=MALK_01272.t1.c1;Parent=MALK_01272.t1
contig004	maker	mRNA	68219	70342	.	-	.	ID=MALK_01272.t1;Parent=MALK_01272
contig004	maker	exon	68219	70342	.	-	.	ID=MALK_01272.t1.e1;Parent=MALK_01272.t1
contig004	maker	gene	70471	72132	.	-	.	ID=MALK_01273;prediction_source=maker_MRET:augustus_masked-contig004-processed-gene-0.85-mRNA-1
contig004	maker	CDS	70471	72132	.	-	0	ID=MALK_01273.t1.c1;Parent=MALK_01273.t1
contig004	maker	mRNA	70471	72132	.	-	.	ID=MALK_01273.t1;Parent=MALK_01273
contig004	maker	exon	70471	72132	.	-	.	ID=MALK_01273.t1.e1;Parent=MALK_01273.t1
contig004	AUGUSTUS	gene	72229	75354	1	+	.	ID=MALK_01274;prediction_source=augustus:contig004.g47.t1
contig004	AUGUSTUS	CDS	72229	75354	1	+	0	ID=MALK_01274.t1.c1;Parent=MALK_01274.t1
contig004	AUGUSTUS	mRNA	72229	75354	1	+	.	ID=MALK_01274.t1;Parent=MALK_01274
contig004	AUGUSTUS	exon	72229	75354	1	+	.	ID=MALK_01274.t1.e1;Parent=MALK_01274.t1
contig004	AUGUSTUS	gene	76090	76516	0.99	+	.	ID=MALK_01275;prediction_source=braker_MRET:g439.t1
contig004	AUGUSTUS	CDS	76090	76092	1	+	0	ID=MALK_01275.t1.c1;Parent=MALK_01275.t1
contig004	AUGUSTUS	CDS	76206	76444	1	+	0	ID=MALK_01275.t1.c2;Parent=MALK_01275.t1
contig004	AUGUSTUS	CDS	76480	76516	1	+	0	ID=MALK_01275.t1.c3;Parent=MALK_01275.t1
contig004	AUGUSTUS	mRNA	76090	76516	0.99	+	.	ID=MALK_01275.t1;Parent=MALK_01275
contig004	AUGUSTUS	exon	76090	76092	.	+	.	ID=MALK_01275.t1.e1;Parent=MALK_01275.t1
contig004	AUGUSTUS	exon	76206	76444	.	+	.	ID=MALK_01275.t1.e2;Parent=MALK_01275.t1
contig004	AUGUSTUS	exon	76480	76516	.	+	.	ID=MALK_01275.t1.e3;Parent=MALK_01275.t1
contig004	AUGUSTUS	gene	76708	77230	0.46	-	.	ID=MALK_01276;prediction_source=braker_MRET:g440.t1
contig004	AUGUSTUS	CDS	77049	77230	0.48	-	0	ID=MALK_01276.t1.c3;Parent=MALK_01276.t1
contig004	AUGUSTUS	CDS	76907	77020	0.48	-	0	ID=MALK_01276.t1.c2;Parent=MALK_01276.t1
contig004	AUGUSTUS	CDS	76708	76879	0.48	-	0	ID=MALK_01276.t1.c1;Parent=MALK_01276.t1
contig004	AUGUSTUS	mRNA	76708	77230	0.46	-	.	ID=MALK_01276.t1;Parent=MALK_01276
contig004	AUGUSTUS	exon	77049	77230	.	-	.	ID=MALK_01276.t1.e3;Parent=MALK_01276.t1
contig004	AUGUSTUS	exon	76907	77020	.	-	.	ID=MALK_01276.t1.e2;Parent=MALK_01276.t1

contig004	AUGUSTUS	exon	76708	76879	.	-	.	ID=MALK_01276.t1.e1;Parent=MALK_01276.t1
contig004	AUGUSTUS	gene	77346	77792	0.57	+	.	ID=MALK_01277;prediction_source=braker_MRET:g441.t1
contig004	AUGUSTUS	CDS	77346	77792	0.57	+	0	ID=MALK_01277.t1.c1;Parent=MALK_01277.t1
contig004	AUGUSTUS	mRNA	77346	77792	0.57	+	.	ID=MALK_01277.t1;Parent=MALK_01277
contig004	AUGUSTUS	exon	77346	77792	.	+	.	ID=MALK_01277.t1.e1;Parent=MALK_01277.t1
contig004	AUGUSTUS	gene	77820	79205	0.94	+	.	ID=MALK_01278;prediction_source=augustus:contig004.g49.t1
contig004	AUGUSTUS	CDS	77820	79205	0.94	+	0	ID=MALK_01278.t1.c1;Parent=MALK_01278.t1
contig004	AUGUSTUS	mRNA	77820	79205	0.94	+	.	ID=MALK_01278.t1;Parent=MALK_01278
contig004	AUGUSTUS	exon	77820	79205	0.94	+	.	ID=MALK_01278.t1.e1;Parent=MALK_01278.t1
contig004	AUGUSTUS	gene	79202	81520	1	-	.	ID=MALK_01279;prediction_source=augustus:contig004.g50.t1
contig004	AUGUSTUS	CDS	79202	81520	1	-	0	ID=MALK_01279.t1.c1;Parent=MALK_01279.t1
contig004	AUGUSTUS	mRNA	79202	81520	1	-	.	ID=MALK_01279.t1;Parent=MALK_01279
contig004	AUGUSTUS	exon	79202	81520	1	-	.	ID=MALK_01279.t1.e1;Parent=MALK_01279.t1
contig004	maker	gene	81692	86038	.	+	.	ID=MALK_01280;prediction_source=maker_MRET:augustus_masked-contig004-processed-gene-0.65-mRNA-1
contig004	maker	CDS	81692	86038	.	+	0	ID=MALK_01280.t1.c1;Parent=MALK_01280.t1
contig004	maker	mRNA	81692	86038	.	+	.	ID=MALK_01280.t1;Parent=MALK_01280
contig004	maker	exon	81692	86038	.	+	.	ID=MALK_01280.t1.e1;Parent=MALK_01280.t1
contig004	AUGUSTUS	gene	86344	87609	0.97	+	.	ID=MALK_01281;prediction_source=braker_MRET:g445.t1
contig004	AUGUSTUS	CDS	86344	87609	0.97	+	0	ID=MALK_01281.t1.c1;Parent=MALK_01281.t1
contig004	AUGUSTUS	mRNA	86344	87609	0.97	+	.	ID=MALK_01281.t1;Parent=MALK_01281
contig004	AUGUSTUS	exon	86344	87609	.	+	.	ID=MALK_01281.t1.e1;Parent=MALK_01281.t1
contig004	AUGUSTUS	gene	87644	88672	0.3	+	.	ID=MALK_01282;prediction_source=augustus:contig004.g55.t1
contig004	AUGUSTUS	CDS	87644	88672	0.3	+	0	ID=MALK_01282.t1.c1;Parent=MALK_01282.t1
contig004	AUGUSTUS	mRNA	87644	88672	0.3	+	.	ID=MALK_01282.t1;Parent=MALK_01282
contig004	AUGUSTUS	exon	87644	88672	0.3	+	.	ID=MALK_01282.t1.e1;Parent=MALK_01282.t1
contig004	AUGUSTUS	gene	89620	91012	0.37	+	.	ID=MALK_01283;prediction_source=augustus:contig004.g57.t1
contig004	AUGUSTUS	CDS	89620	90524	0.4	+	0	ID=MALK_01283.t1.c1;Parent=MALK_01283.t1
contig004	AUGUSTUS	CDS	90823	91012	0.4	+	0	ID=MALK_01283.t1.c2;Parent=MALK_01283.t1
contig004	AUGUSTUS	mRNA	89620	91012	0.37	+	.	ID=MALK_01283.t1;Parent=MALK_01283
contig004	AUGUSTUS	exon	89620	90524	0.4	+	.	ID=MALK_01283.t1.e1;Parent=MALK_01283.t1
contig004	AUGUSTUS	exon	90823	91012	0.4	+	.	ID=MALK_01283.t1.e2;Parent=MALK_01283.t1
contig004	AUGUSTUS	gene	91330	91970	0.28	+	.	ID=MALK_01284;prediction_source=braker_MRET:g449.t1
contig004	AUGUSTUS	CDS	91330	91356	0.48	+	0	ID=MALK_01284.t1.c1;Parent=MALK_01284.t1
contig004	AUGUSTUS	CDS	91387	91575	0.48	+	0	ID=MALK_01284.t1.c2;Parent=MALK_01284.t1
contig004	AUGUSTUS	CDS	91614	91653	0.48	+	0	ID=MALK_01284.t1.c3;Parent=MALK_01284.t1
contig004	AUGUSTUS	CDS	91699	91970	0.48	+	0	ID=MALK_01284.t1.c4;Parent=MALK_01284.t1
contig004	AUGUSTUS	mRNA	91330	91970	0.28	+	.	ID=MALK_01284.t1;Parent=MALK_01284
contig004	AUGUSTUS	exon	91330	91356	.	+	.	ID=MALK_01284.t1.e1;Parent=MALK_01284.t1
contig004	AUGUSTUS	exon	91387	91575	.	+	.	ID=MALK_01284.t1.e2;Parent=MALK_01284.t1
contig004	AUGUSTUS	exon	91614	91653	.	+	.	ID=MALK_01284.t1.e3;Parent=MALK_01284.t1
contig004	AUGUSTUS	exon	91699	91970	.	+	.	ID=MALK_01284.t1.e4;Parent=MALK_01284.t1
contig004	AUGUSTUS	gene	92179	93384	0.91	-	.	ID=MALK_01285;prediction_source=augustus:contig004.g58.t1
contig004	AUGUSTUS	CDS	92179	93384	0.91	-	0	ID=MALK_01285.t1.c1;Parent=MALK_01285.t1

contig004	AUGUSTUS	mRNA	92179	93384	0.91	-	.	ID=MALK_01285.t1;Parent=MALK_01285
contig004	AUGUSTUS	exon	92179	93384	0.91	-	.	ID=MALK_01285.t1.e1;Parent=MALK_01285.t1
contig004	maker	gene	93568	95451	.	-	.	ID=MALK_01286;prediction_source=maker_MRET:augustus_masked-contig004-processed-gene-1.4-mRNA-1
contig004	maker	CDS	93568	95451	.	-	0	ID=MALK_01286.t1.c1;Parent=MALK_01286.t1
contig004	maker	mRNA	93568	95451	.	-	.	ID=MALK_01286.t1;Parent=MALK_01286
contig004	maker	exon	93568	95451	.	-	.	ID=MALK_01286.t1.e1;Parent=MALK_01286.t1
contig004	maker	gene	95577	96698	.	+	.	ID=MALK_01287;prediction_source=maker_MRET:augustus_masked-contig004-processed-gene-1.0-mRNA-1
contig004	maker	CDS	95577	96698	.	+	0	ID=MALK_01287.t1.c1;Parent=MALK_01287.t1
contig004	maker	mRNA	95577	96698	.	+	.	ID=MALK_01287.t1;Parent=MALK_01287
contig004	maker	exon	95577	96698	.	+	.	ID=MALK_01287.t1.e1;Parent=MALK_01287.t1
contig004	AUGUSTUS	gene	96908	97813	0.66	+	.	ID=MALK_01288;prediction_source=braker_MRET:g453.t1
contig004	AUGUSTUS	CDS	96908	97063	1	+	0	ID=MALK_01288.t1.c1;Parent=MALK_01288.t1
contig004	AUGUSTUS	CDS	97103	97813	1	+	0	ID=MALK_01288.t1.c2;Parent=MALK_01288.t1
contig004	AUGUSTUS	mRNA	96908	97813	0.66	+	.	ID=MALK_01288.t1;Parent=MALK_01288
contig004	AUGUSTUS	exon	96908	97063	.	+	.	ID=MALK_01288.t1.e1;Parent=MALK_01288.t1
contig004	AUGUSTUS	exon	97103	97813	.	+	.	ID=MALK_01288.t1.e2;Parent=MALK_01288.t1
contig004	maker	gene	97861	99126	.	-	.	ID=MALK_01289;prediction_source=maker_MRET:augustus_masked-contig004-processed-gene-1.5-mRNA-1
contig004	maker	CDS	97861	99126	.	-	0	ID=MALK_01289.t1.c1;Parent=MALK_01289.t1
contig004	maker	mRNA	97861	99126	.	-	.	ID=MALK_01289.t1;Parent=MALK_01289
contig004	maker	exon	97861	99126	.	-	.	ID=MALK_01289.t1.e1;Parent=MALK_01289.t1
contig004	maker	gene	99361	100248	.	+	.	ID=MALK_01290;prediction_source=maker_MRET:augustus_masked-contig004-processed-gene-1.2-mRNA-1
contig004	maker	CDS	99361	100248	.	+	0	ID=MALK_01290.t1.c1;Parent=MALK_01290.t1
contig004	maker	mRNA	99361	100248	.	+	.	ID=MALK_01290.t1;Parent=MALK_01290
contig004	maker	exon	99361	100248	.	+	.	ID=MALK_01290.t1.e1;Parent=MALK_01290.t1
contig004	maker	gene	100267	101400	.	-	.	ID=MALK_01291;prediction_source=maker_MRET:augustus_masked-contig004-processed-gene-1.26-mRNA-1
contig004	maker	CDS	100267	101400	.	-	0	ID=MALK_01291.t1.c1;Parent=MALK_01291.t1
contig004	maker	mRNA	100267	101400	.	-	.	ID=MALK_01291.t1;Parent=MALK_01291
contig004	maker	exon	100267	101400	.	-	.	ID=MALK_01291.t1.e1;Parent=MALK_01291.t1
contig004	AUGUSTUS	gene	102576	105311	0.96	+	.	ID=MALK_01292;prediction_source=augustus:contig004.g64.t1
contig004	AUGUSTUS	CDS	102576	105311	0.96	+	0	ID=MALK_01292.t1.c1;Parent=MALK_01292.t1
contig004	AUGUSTUS	mRNA	102576	105311	0.96	+	.	ID=MALK_01292.t1;Parent=MALK_01292
contig004	AUGUSTUS	exon	102576	105311	0.96	+	.	ID=MALK_01292.t1.e1;Parent=MALK_01292.t1
contig004	maker	gene	105340	109335	.	-	.	ID=MALK_01293;prediction_source=maker_MRET:augustus_masked-contig004-processed-gene-1.27-mRNA-1
contig004	maker	CDS	105340	109335	.	-	0	ID=MALK_01293.t1.c1;Parent=MALK_01293.t1
contig004	maker	mRNA	105340	109335	.	-	.	ID=MALK_01293.t1;Parent=MALK_01293
contig004	maker	exon	105340	109335	.	-	.	ID=MALK_01293.t1.e1;Parent=MALK_01293.t1
contig004	maker	gene	109478	110818	.	-	.	ID=MALK_01294;prediction_source=maker_MRET:augustus_masked-contig004-processed-gene-1.28-mRNA-1
contig004	maker	CDS	109478	110818	.	-	0	ID=MALK_01294.t1.c1;Parent=MALK_01294.t1
contig004	maker	mRNA	109478	110818	.	-	.	ID=MALK_01294.t1;Parent=MALK_01294
contig004	maker	exon	109478	110818	.	-	.	ID=MALK_01294.t1.e1;Parent=MALK_01294.t1
contig004	maker	gene	110996	111751	.	+	.	ID=MALK_01295;prediction_source=maker_MRET:augustus_masked-contig004-processed-gene-1.7-mRNA-1
contig004	maker	CDS	110996	111751	.	+	0	ID=MALK_01295.t1.c1;Parent=MALK_01295.t1
contig004	maker	mRNA	110996	111751	.	+	.	ID=MALK_01295.t1;Parent=MALK_01295

contig004	maker	exon	110996	111751	.	+	.	ID=MALK_01295.t1.e1;Parent=MALK_01295.t1
contig004	maker	gene	111804	112646	.	+	.	ID=MALK_01296;prediction_source=maker_MRET:augustus_masked-contig004-processed-gene-1.8-mRNA-1
contig004	maker	CDS	111804	112646	.	+	0	ID=MALK_01296.t1.c1;Parent=MALK_01296.t1
contig004	maker	mRNA	111804	112646	.	+	.	ID=MALK_01296.t1;Parent=MALK_01296
contig004	maker	exon	111804	112646	.	+	.	ID=MALK_01296.t1.e1;Parent=MALK_01296.t1
contig004	AUGUSTUS	gene	113080	114204	0.68	-	.	ID=MALK_01297;prediction_source=braker_MRET:g462.t1
contig004	AUGUSTUS	CDS	113080	114204	0.68	-	0	ID=MALK_01297.t1.c1;Parent=MALK_01297.t1
contig004	AUGUSTUS	mRNA	113080	114204	0.68	-	.	ID=MALK_01297.t1;Parent=MALK_01297
contig004	AUGUSTUS	exon	113080	114204	.	-	.	ID=MALK_01297.t1.e1;Parent=MALK_01297.t1
contig004	maker	gene	114266	115978	.	-	.	ID=MALK_01298;prediction_source=maker_MRET:augustus_masked-contig004-processed-gene-1.29-mRNA-1
contig004	maker	CDS	114266	115978	.	-	0	ID=MALK_01298.t1.c1;Parent=MALK_01298.t1
contig004	maker	mRNA	114266	115978	.	-	.	ID=MALK_01298.t1;Parent=MALK_01298
contig004	maker	exon	114266	115978	.	-	.	ID=MALK_01298.t1.e1;Parent=MALK_01298.t1
contig004	maker	gene	116091	118196	.	+	.	ID=MALK_01299;prediction_source=maker_MRET:augustus_masked-contig004-processed-gene-1.9-mRNA-1
contig004	maker	CDS	116091	118196	.	+	0	ID=MALK_01299.t1.c1;Parent=MALK_01299.t1
contig004	maker	mRNA	116091	118196	.	+	.	ID=MALK_01299.t1;Parent=MALK_01299
contig004	maker	exon	116091	118196	.	+	.	ID=MALK_01299.t1.e1;Parent=MALK_01299.t1
contig004	AUGUSTUS	gene	118233	119391	0.89	-	.	ID=MALK_01300;prediction_source=braker_MRET:g465.t1
contig004	AUGUSTUS	CDS	118315	119391	0.98	-	0	ID=MALK_01300.t1.c2;Parent=MALK_01300.t1
contig004	AUGUSTUS	CDS	118233	118271	0.98	-	0	ID=MALK_01300.t1.c1;Parent=MALK_01300.t1
contig004	AUGUSTUS	mRNA	118233	119391	0.89	-	.	ID=MALK_01300.t1;Parent=MALK_01300
contig004	AUGUSTUS	exon	118315	119391	.	-	.	ID=MALK_01300.t1.e2;Parent=MALK_01300.t1
contig004	AUGUSTUS	exon	118233	118271	.	-	.	ID=MALK_01300.t1.e1;Parent=MALK_01300.t1
contig004	AUGUSTUS	gene	119484	120635	1	+	.	ID=MALK_01301;prediction_source=braker_MRET:g466.t1
contig004	AUGUSTUS	CDS	119484	120635	1	+	0	ID=MALK_01301.t1.c1;Parent=MALK_01301.t1
contig004	AUGUSTUS	mRNA	119484	120635	1	+	.	ID=MALK_01301.t1;Parent=MALK_01301
contig004	AUGUSTUS	exon	119484	120635	.	+	.	ID=MALK_01301.t1.e1;Parent=MALK_01301.t1
contig004	AUGUSTUS	gene	120642	121508	0.81	-	.	ID=MALK_01302;prediction_source=augustus:contig004.g74.t1
contig004	AUGUSTUS	CDS	121337	121508	0.81	-	0	ID=MALK_01302.t1.c2;Parent=MALK_01302.t1
contig004	AUGUSTUS	CDS	120642	121252	0.81	-	0	ID=MALK_01302.t1.c1;Parent=MALK_01302.t1
contig004	AUGUSTUS	mRNA	120642	121508	0.81	-	.	ID=MALK_01302.t1;Parent=MALK_01302
contig004	AUGUSTUS	exon	121337	121508	0.81	-	.	ID=MALK_01302.t1.e2;Parent=MALK_01302.t1
contig004	AUGUSTUS	exon	120642	121252	0.81	-	.	ID=MALK_01302.t1.e1;Parent=MALK_01302.t1
contig004	AUGUSTUS	gene	122227	124206	0.71	+	.	ID=MALK_01303;prediction_source=augustus:contig004.g75.t1
contig004	AUGUSTUS	CDS	122227	124206	0.71	+	0	ID=MALK_01303.t1.c1;Parent=MALK_01303.t1
contig004	AUGUSTUS	mRNA	122227	124206	0.71	+	.	ID=MALK_01303.t1;Parent=MALK_01303
contig004	AUGUSTUS	exon	122227	124206	0.71	+	.	ID=MALK_01303.t1.e1;Parent=MALK_01303.t1
contig004	maker	gene	124295	126940	.	-	.	ID=MALK_01304;prediction_source=maker_MRET:augustus_masked-contig004-processed-gene-1.31-mRNA-1
contig004	maker	CDS	124295	126940	.	-	0	ID=MALK_01304.t1.c1;Parent=MALK_01304.t1
contig004	maker	mRNA	124295	126940	.	-	.	ID=MALK_01304.t1;Parent=MALK_01304
contig004	maker	exon	124295	126940	.	-	.	ID=MALK_01304.t1.e1;Parent=MALK_01304.t1
contig004	maker	gene	127096	127773	.	+	.	ID=MALK_01305;prediction_source=maker_MRET:augustus_masked-contig004-processed-gene-1.12-mRNA-1
contig004	maker	CDS	127096	127773	.	+	0	ID=MALK_01305.t1.c1;Parent=MALK_01305.t1

contig004	maker	mRNA	127096	127773	.	+	.	ID=MALK_01305.t1;Parent=MALK_01305
contig004	maker	exon	127096	127773	.	+	.	ID=MALK_01305.t1.e1;Parent=MALK_01305.t1
contig004	maker	gene	127824	130067	.	-	.	ID=MALK_01306;prediction_source=maker_MRET:augustus_masked-contig004-processed-gene-1.32-mRNA-1
contig004	maker	CDS	127824	130067	.	-	0	ID=MALK_01306.t1.c1;Parent=MALK_01306.t1
contig004	maker	mRNA	127824	130067	.	-	.	ID=MALK_01306.t1;Parent=MALK_01306
contig004	maker	exon	127824	130067	.	-	.	ID=MALK_01306.t1.e1;Parent=MALK_01306.t1
contig004	AUGUSTUS	gene	130178	132709	0.97	-	.	ID=MALK_01307;prediction_source=augustus:contig004.g81.t1
contig004	AUGUSTUS	CDS	130178	132709	0.97	-	0	ID=MALK_01307.t1.c1;Parent=MALK_01307.t1
contig004	AUGUSTUS	mRNA	130178	132709	0.97	-	.	ID=MALK_01307.t1;Parent=MALK_01307
contig004	AUGUSTUS	exon	130178	132709	0.97	-	.	ID=MALK_01307.t1.e1;Parent=MALK_01307.t1
contig004	AUGUSTUS	gene	132835	134310	0.48	-	.	ID=MALK_01308;prediction_source=braker_MRET:g473.t1
contig004	AUGUSTUS	CDS	133992	134310	0.63	-	0	ID=MALK_01308.t1.c6;Parent=MALK_01308.t1
contig004	AUGUSTUS	CDS	133797	133959	0.63	-	0	ID=MALK_01308.t1.c5;Parent=MALK_01308.t1
contig004	AUGUSTUS	CDS	133733	133767	0.63	-	0	ID=MALK_01308.t1.c4;Parent=MALK_01308.t1
contig004	AUGUSTUS	CDS	133547	133698	0.63	-	0	ID=MALK_01308.t1.c3;Parent=MALK_01308.t1
contig004	AUGUSTUS	CDS	133368	133393	0.63	-	0	ID=MALK_01308.t1.c2;Parent=MALK_01308.t1
contig004	AUGUSTUS	CDS	132835	133336	0.63	-	0	ID=MALK_01308.t1.c1;Parent=MALK_01308.t1
contig004	AUGUSTUS	mRNA	132835	134310	0.48	-	.	ID=MALK_01308.t1;Parent=MALK_01308
contig004	AUGUSTUS	exon	133992	134310	.	-	.	ID=MALK_01308.t1.e6;Parent=MALK_01308.t1
contig004	AUGUSTUS	exon	133797	133959	.	-	.	ID=MALK_01308.t1.e5;Parent=MALK_01308.t1
contig004	AUGUSTUS	exon	133733	133767	.	-	.	ID=MALK_01308.t1.e4;Parent=MALK_01308.t1
contig004	AUGUSTUS	exon	133547	133698	.	-	.	ID=MALK_01308.t1.e3;Parent=MALK_01308.t1
contig004	AUGUSTUS	exon	133368	133393	.	-	.	ID=MALK_01308.t1.e2;Parent=MALK_01308.t1
contig004	AUGUSTUS	exon	132835	133336	.	-	.	ID=MALK_01308.t1.e1;Parent=MALK_01308.t1
contig004	maker	gene	134346	137030	.	+	.	ID=MALK_01309;prediction_source=maker_MRET:augustus_masked-contig004-processed-gene-1.13-mRNA-1
contig004	maker	CDS	134346	137030	.	+	0	ID=MALK_01309.t1.c1;Parent=MALK_01309.t1
contig004	maker	mRNA	134346	137030	.	+	.	ID=MALK_01309.t1;Parent=MALK_01309
contig004	maker	exon	134346	137030	.	+	.	ID=MALK_01309.t1.e1;Parent=MALK_01309.t1
contig004	AUGUSTUS	gene	137436	139286	0.5	+	.	ID=MALK_01310;prediction_source=augustus:contig004.g87.t1
contig004	AUGUSTUS	CDS	137436	139286	0.5	+	0	ID=MALK_01310.t1.c1;Parent=MALK_01310.t1
contig004	AUGUSTUS	mRNA	137436	139286	0.5	+	.	ID=MALK_01310.t1;Parent=MALK_01310
contig004	AUGUSTUS	exon	137436	139286	0.5	+	.	ID=MALK_01310.t1.e1;Parent=MALK_01310.t1
contig004	AUGUSTUS	gene	139415	141508	0.96	+	.	ID=MALK_01311;prediction_source=augustus:contig004.g88.t1
contig004	AUGUSTUS	CDS	139415	141508	0.96	+	0	ID=MALK_01311.t1.c1;Parent=MALK_01311.t1
contig004	AUGUSTUS	mRNA	139415	141508	0.96	+	.	ID=MALK_01311.t1;Parent=MALK_01311
contig004	AUGUSTUS	exon	139415	141508	0.96	+	.	ID=MALK_01311.t1.e1;Parent=MALK_01311.t1
contig004	AUGUSTUS	gene	141638	143584	0.97	+	.	ID=MALK_01312;prediction_source=augustus:contig004.g90.t1
contig004	AUGUSTUS	CDS	141638	143584	0.97	+	0	ID=MALK_01312.t1.c1;Parent=MALK_01312.t1
contig004	AUGUSTUS	mRNA	141638	143584	0.97	+	.	ID=MALK_01312.t1;Parent=MALK_01312
contig004	AUGUSTUS	exon	141638	143584	0.97	+	.	ID=MALK_01312.t1.e1;Parent=MALK_01312.t1
contig004	AUGUSTUS	gene	143803	145749	1	+	.	ID=MALK_01313;prediction_source=augustus:contig004.g91.t1
contig004	AUGUSTUS	CDS	143803	145749	1	+	0	ID=MALK_01313.t1.c1;Parent=MALK_01313.t1
contig004	AUGUSTUS	mRNA	143803	145749	1	+	.	ID=MALK_01313.t1;Parent=MALK_01313

contig004	AUGUSTUS	exon	143803	145749	1	+	.	ID=MALK_01313.t1.e1;Parent=MALK_01313.t1
contig004	AUGUSTUS	gene	145944	147911	0.89	+	.	ID=MALK_01314;prediction_source=augustus:contig004.g93.t1
contig004	AUGUSTUS	CDS	145944	147911	0.89	+	0	ID=MALK_01314.t1.c1;Parent=MALK_01314.t1
contig004	AUGUSTUS	mRNA	145944	147911	0.89	+	.	ID=MALK_01314.t1;Parent=MALK_01314
contig004	AUGUSTUS	exon	145944	147911	0.89	+	.	ID=MALK_01314.t1.e1;Parent=MALK_01314.t1
contig004	maker	gene	148261	150216	.	+	.	ID=MALK_01315;prediction_source=maker_MRET:augustus_masked-contig004-processed-gene-1.19-mRNA-1
contig004	maker	CDS	148261	150216	.	+	0	ID=MALK_01315.t1.c1;Parent=MALK_01315.t1
contig004	maker	mRNA	148261	150216	.	+	.	ID=MALK_01315.t1;Parent=MALK_01315
contig004	maker	exon	148261	150216	.	+	.	ID=MALK_01315.t1.e1;Parent=MALK_01315.t1
contig004	AUGUSTUS	gene	150405	151322	1	-	.	ID=MALK_01316;prediction_source=augustus:contig004.g95.t1
contig004	AUGUSTUS	CDS	150405	151322	1	-	0	ID=MALK_01316.t1.c1;Parent=MALK_01316.t1
contig004	AUGUSTUS	mRNA	150405	151322	1	-	.	ID=MALK_01316.t1;Parent=MALK_01316
contig004	AUGUSTUS	exon	150405	151322	1	-	.	ID=MALK_01316.t1.e1;Parent=MALK_01316.t1
contig004	AUGUSTUS	gene	151747	153591	0.69	-	.	ID=MALK_01317;prediction_source=augustus:contig004.g96.t1
contig004	AUGUSTUS	CDS	151747	153591	0.69	-	0	ID=MALK_01317.t1.c1;Parent=MALK_01317.t1
contig004	AUGUSTUS	mRNA	151747	153591	0.69	-	.	ID=MALK_01317.t1;Parent=MALK_01317
contig004	AUGUSTUS	exon	151747	153591	0.69	-	.	ID=MALK_01317.t1.e1;Parent=MALK_01317.t1
contig004	maker	gene	153894	157247	.	-	.	ID=MALK_01318;prediction_source=maker_MRET:augustus_masked-contig004-processed-gene-1.38-mRNA-1
contig004	maker	CDS	156333	157247	.	-	0	ID=MALK_01318.t1.c1;Parent=MALK_01318.t1
contig004	maker	CDS	154603	156219	.	-	0	ID=MALK_01318.t1.c2;Parent=MALK_01318.t1
contig004	maker	CDS	153894	154469	.	-	0	ID=MALK_01318.t1.c3;Parent=MALK_01318.t1
contig004	maker	mRNA	153894	157247	.	-	.	ID=MALK_01318.t1;Parent=MALK_01318
contig004	maker	exon	156333	157247	.	-	.	ID=MALK_01318.t1.e1;Parent=MALK_01318.t1
contig004	maker	exon	154603	156219	.	-	.	ID=MALK_01318.t1.e2;Parent=MALK_01318.t1
contig004	maker	exon	153894	154469	.	-	.	ID=MALK_01318.t1.e3;Parent=MALK_01318.t1
contig004	AUGUSTUS	gene	157340	158374	0.99	+	.	ID=MALK_01319;prediction_source=augustus:contig004.g99.t1
contig004	AUGUSTUS	CDS	157340	158374	0.99	+	0	ID=MALK_01319.t1.c1;Parent=MALK_01319.t1
contig004	AUGUSTUS	mRNA	157340	158374	0.99	+	.	ID=MALK_01319.t1;Parent=MALK_01319
contig004	AUGUSTUS	exon	157340	158374	0.99	+	.	ID=MALK_01319.t1.e1;Parent=MALK_01319.t1
contig004	AUGUSTUS	gene	158417	160369	0.67	-	.	ID=MALK_01320;prediction_source=augustus:contig004.g100.t1
contig004	AUGUSTUS	CDS	158417	160369	0.67	-	0	ID=MALK_01320.t1.c1;Parent=MALK_01320.t1
contig004	AUGUSTUS	mRNA	158417	160369	0.67	-	.	ID=MALK_01320.t1;Parent=MALK_01320
contig004	AUGUSTUS	exon	158417	160369	0.67	-	.	ID=MALK_01320.t1.e1;Parent=MALK_01320.t1
contig004	AUGUSTUS	gene	160554	163538	0.58	-	.	ID=MALK_01321;prediction_source=augustus:contig004.g101.t1
contig004	AUGUSTUS	CDS	160554	163538	0.58	-	0	ID=MALK_01321.t1.c1;Parent=MALK_01321.t1
contig004	AUGUSTUS	mRNA	160554	163538	0.58	-	.	ID=MALK_01321.t1;Parent=MALK_01321
contig004	AUGUSTUS	exon	160554	163538	0.58	-	.	ID=MALK_01321.t1.e1;Parent=MALK_01321.t1
contig004	AUGUSTUS	gene	163759	164209	1	+	.	ID=MALK_01322;prediction_source=braker_MRET:g487.t1
contig004	AUGUSTUS	CDS	163759	163784	1	+	0	ID=MALK_01322.t1.c1;Parent=MALK_01322.t1
contig004	AUGUSTUS	CDS	163831	163985	1	+	0	ID=MALK_01322.t1.c2;Parent=MALK_01322.t1
contig004	AUGUSTUS	CDS	164025	164209	1	+	0	ID=MALK_01322.t1.c3;Parent=MALK_01322.t1
contig004	AUGUSTUS	mRNA	163759	164209	1	+	.	ID=MALK_01322.t1;Parent=MALK_01322
contig004	AUGUSTUS	exon	163759	163784	.	+	.	ID=MALK_01322.t1.e1;Parent=MALK_01322.t1

contig004	AUGUSTUS	exon	163831	163985	.	+	.	ID=MALK_01322.t1.e2;Parent=MALK_01322.t1
contig004	AUGUSTUS	exon	164025	164209	.	+	.	ID=MALK_01322.t1.e3;Parent=MALK_01322.t1
contig004	AUGUSTUS	gene	164283	167882	0.81	-	.	ID=MALK_01323;prediction_source=braker_MRET:g488.t1
contig004	AUGUSTUS	CDS	164283	167882	0.81	-	0	ID=MALK_01323.t1.c1;Parent=MALK_01323.t1
contig004	AUGUSTUS	mRNA	164283	167882	0.81	-	.	ID=MALK_01323.t1;Parent=MALK_01323
contig004	AUGUSTUS	exon	164283	167882	.	-	.	ID=MALK_01323.t1.e1;Parent=MALK_01323.t1
contig004	AUGUSTUS	gene	167934	168668	0.95	-	.	ID=MALK_01324;prediction_source=augustus:contig004.g106.t1
contig004	AUGUSTUS	CDS	167934	168668	0.95	-	0	ID=MALK_01324.t1.c1;Parent=MALK_01324.t1
contig004	AUGUSTUS	mRNA	167934	168668	0.95	-	.	ID=MALK_01324.t1;Parent=MALK_01324
contig004	AUGUSTUS	exon	167934	168668	0.95	-	.	ID=MALK_01324.t1.e1;Parent=MALK_01324.t1
contig004	maker	gene	168735	170156	.	-	.	ID=MALK_01325;prediction_source=maker_MRET:augustus_masked-contig004-processed-gene-1.43-mRNA-1
contig004	maker	CDS	168735	170156	.	-	0	ID=MALK_01325.t1.c1;Parent=MALK_01325.t1
contig004	maker	mRNA	168735	170156	.	-	.	ID=MALK_01325.t1;Parent=MALK_01325
contig004	maker	exon	168735	170156	.	-	.	ID=MALK_01325.t1.e1;Parent=MALK_01325.t1
contig004	AUGUSTUS	gene	170377	171732	0.99	-	.	ID=MALK_01326;prediction_source=augustus:contig004.g109.t1
contig004	AUGUSTUS	CDS	170377	171732	0.99	-	0	ID=MALK_01326.t1.c1;Parent=MALK_01326.t1
contig004	AUGUSTUS	mRNA	170377	171732	0.99	-	.	ID=MALK_01326.t1;Parent=MALK_01326
contig004	AUGUSTUS	exon	170377	171732	0.99	-	.	ID=MALK_01326.t1.e1;Parent=MALK_01326.t1
contig004	AUGUSTUS	gene	172425	174738	0.77	+	.	ID=MALK_01327;prediction_source=braker_MRET:g492.t1
contig004	AUGUSTUS	CDS	172425	174496	1	+	0	ID=MALK_01327.t1.c1;Parent=MALK_01327.t1
contig004	AUGUSTUS	CDS	174534	174666	1	+	0	ID=MALK_01327.t1.c2;Parent=MALK_01327.t1
contig004	AUGUSTUS	CDS	174712	174738	1	+	0	ID=MALK_01327.t1.c3;Parent=MALK_01327.t1
contig004	AUGUSTUS	mRNA	172425	174738	0.77	+	.	ID=MALK_01327.t1;Parent=MALK_01327
contig004	AUGUSTUS	exon	172425	174496	.	+	.	ID=MALK_01327.t1.e1;Parent=MALK_01327.t1
contig004	AUGUSTUS	exon	174534	174666	.	+	.	ID=MALK_01327.t1.e2;Parent=MALK_01327.t1
contig004	AUGUSTUS	exon	174712	174738	.	+	.	ID=MALK_01327.t1.e3;Parent=MALK_01327.t1
contig004	maker	gene	175032	177158	.	+	.	ID=MALK_01328;prediction_source=maker_MRET:augustus_masked-contig004-processed-gene-1.22-mRNA-1
contig004	maker	CDS	175032	177158	.	+	0	ID=MALK_01328.t1.c1;Parent=MALK_01328.t1
contig004	maker	mRNA	175032	177158	.	+	.	ID=MALK_01328.t1;Parent=MALK_01328
contig004	maker	exon	175032	177158	.	+	.	ID=MALK_01328.t1.e1;Parent=MALK_01328.t1
contig004	AUGUSTUS	gene	177441	178093	0.95	+	.	ID=MALK_01329;prediction_source=braker_MRET:g494.t1
contig004	AUGUSTUS	CDS	177441	177574	0.99	+	0	ID=MALK_01329.t1.c1;Parent=MALK_01329.t1
contig004	AUGUSTUS	CDS	177613	178093	0.99	+	0	ID=MALK_01329.t1.c2;Parent=MALK_01329.t1
contig004	AUGUSTUS	mRNA	177441	178093	0.95	+	.	ID=MALK_01329.t1;Parent=MALK_01329
contig004	AUGUSTUS	exon	177441	177574	.	+	.	ID=MALK_01329.t1.e1;Parent=MALK_01329.t1
contig004	AUGUSTUS	exon	177613	178093	.	+	.	ID=MALK_01329.t1.e2;Parent=MALK_01329.t1
contig004	AUGUSTUS	gene	178213	179712	0.65	-	.	ID=MALK_01330;prediction_source=augustus:contig004.g114.t1
contig004	AUGUSTUS	CDS	178213	179712	0.65	-	0	ID=MALK_01330.t1.c1;Parent=MALK_01330.t1
contig004	AUGUSTUS	mRNA	178213	179712	0.65	-	.	ID=MALK_01330.t1;Parent=MALK_01330
contig004	AUGUSTUS	exon	178213	179712	0.65	-	.	ID=MALK_01330.t1.e1;Parent=MALK_01330.t1
contig004	AUGUSTUS	gene	180125	182944	0.87	+	.	ID=MALK_01331;prediction_source=augustus:contig004.g115.t1
contig004	AUGUSTUS	CDS	180125	182944	0.87	+	0	ID=MALK_01331.t1.c1;Parent=MALK_01331.t1
contig004	AUGUSTUS	mRNA	180125	182944	0.87	+	.	ID=MALK_01331.t1;Parent=MALK_01331

contig004	AUGUSTUS	exon	180125	182944	0.87	+	.	ID=MALK_01331.t1.e1;Parent=MALK_01331.t1
contig004	AUGUSTUS	gene	183111	183395	1	-	.	ID=MALK_01332;prediction_source=braker_MRET:g497.t1
contig004	AUGUSTUS	CDS	183354	183395	1	-	0	ID=MALK_01332.t1.c3;Parent=MALK_01332.t1
contig004	AUGUSTUS	CDS	183153	183310	1	-	0	ID=MALK_01332.t1.c2;Parent=MALK_01332.t1
contig004	AUGUSTUS	CDS	183111	183123	1	-	0	ID=MALK_01332.t1.c1;Parent=MALK_01332.t1
contig004	AUGUSTUS	mRNA	183111	183395	1	-	.	ID=MALK_01332.t1;Parent=MALK_01332
contig004	AUGUSTUS	exon	183354	183395	.	-	.	ID=MALK_01332.t1.e3;Parent=MALK_01332.t1
contig004	AUGUSTUS	exon	183153	183310	.	-	.	ID=MALK_01332.t1.e2;Parent=MALK_01332.t1
contig004	AUGUSTUS	exon	183111	183123	.	-	.	ID=MALK_01332.t1.e1;Parent=MALK_01332.t1
contig004	AUGUSTUS	gene	183667	184995	0.93	+	.	ID=MALK_01333;prediction_source=braker_MRET:g498.t1
contig004	AUGUSTUS	CDS	183667	184995	0.93	+	0	ID=MALK_01333.t1.c1;Parent=MALK_01333.t1
contig004	AUGUSTUS	mRNA	183667	184995	0.93	+	.	ID=MALK_01333.t1;Parent=MALK_01333
contig004	AUGUSTUS	exon	183667	184995	.	+	.	ID=MALK_01333.t1.e1;Parent=MALK_01333.t1
contig004	AUGUSTUS	gene	185297	189568	0.41	+	.	ID=MALK_01334;prediction_source=braker_MRET:g499.t1
contig004	AUGUSTUS	CDS	185297	185422	0.95	+	0	ID=MALK_01334.t1.c1;Parent=MALK_01334.t1
contig004	AUGUSTUS	CDS	185453	189568	0.95	+	0	ID=MALK_01334.t1.c2;Parent=MALK_01334.t1
contig004	AUGUSTUS	mRNA	185297	189568	0.41	+	.	ID=MALK_01334.t1;Parent=MALK_01334
contig004	AUGUSTUS	exon	185297	185422	.	+	.	ID=MALK_01334.t1.e1;Parent=MALK_01334.t1
contig004	AUGUSTUS	exon	185453	189568	.	+	.	ID=MALK_01334.t1.e2;Parent=MALK_01334.t1
contig004	AUGUSTUS	gene	189582	190747	0.18	-	.	ID=MALK_01335;prediction_source=braker_MRET:g500.t1
contig004	AUGUSTUS	CDS	189953	190747	0.66	-	0	ID=MALK_01335.t1.c3;Parent=MALK_01335.t1
contig004	AUGUSTUS	CDS	189829	189893	0.66	-	0	ID=MALK_01335.t1.c2;Parent=MALK_01335.t1
contig004	AUGUSTUS	CDS	189582	189780	0.66	-	0	ID=MALK_01335.t1.c1;Parent=MALK_01335.t1
contig004	AUGUSTUS	mRNA	189582	190747	0.18	-	.	ID=MALK_01335.t1;Parent=MALK_01335
contig004	AUGUSTUS	exon	189953	190747	.	-	.	ID=MALK_01335.t1.e3;Parent=MALK_01335.t1
contig004	AUGUSTUS	exon	189829	189893	.	-	.	ID=MALK_01335.t1.e2;Parent=MALK_01335.t1
contig004	AUGUSTUS	exon	189582	189780	.	-	.	ID=MALK_01335.t1.e1;Parent=MALK_01335.t1
contig004	AUGUSTUS	gene	190782	192590	0.64	-	.	ID=MALK_01336;prediction_source=augustus:contig004.g118.t1
contig004	AUGUSTUS	CDS	190782	192590	0.64	-	0	ID=MALK_01336.t1.c1;Parent=MALK_01336.t1
contig004	AUGUSTUS	mRNA	190782	192590	0.64	-	.	ID=MALK_01336.t1;Parent=MALK_01336
contig004	AUGUSTUS	exon	190782	192590	0.64	-	.	ID=MALK_01336.t1.e1;Parent=MALK_01336.t1
contig004	AUGUSTUS	gene	192705	193817	1	-	.	ID=MALK_01337;prediction_source=augustus:contig004.g119.t1
contig004	AUGUSTUS	CDS	192705	193817	1	-	0	ID=MALK_01337.t1.c1;Parent=MALK_01337.t1
contig004	AUGUSTUS	mRNA	192705	193817	1	-	.	ID=MALK_01337.t1;Parent=MALK_01337
contig004	AUGUSTUS	exon	192705	193817	1	-	.	ID=MALK_01337.t1.e1;Parent=MALK_01337.t1
contig004	AUGUSTUS	gene	194004	194807	0.44	+	.	ID=MALK_01338;prediction_source=braker_MRET:g503.t1
contig004	AUGUSTUS	CDS	194004	194807	0.44	+	0	ID=MALK_01338.t1.c1;Parent=MALK_01338.t1
contig004	AUGUSTUS	mRNA	194004	194807	0.44	+	.	ID=MALK_01338.t1;Parent=MALK_01338
contig004	AUGUSTUS	exon	194004	194807	.	+	.	ID=MALK_01338.t1.e1;Parent=MALK_01338.t1
contig004	AUGUSTUS	gene	194824	195435	0.86	-	.	ID=MALK_01339;prediction_source=braker_MRET:g504.t1
contig004	AUGUSTUS	CDS	194824	195435	0.86	-	0	ID=MALK_01339.t1.c1;Parent=MALK_01339.t1
contig004	AUGUSTUS	mRNA	194824	195435	0.86	-	.	ID=MALK_01339.t1;Parent=MALK_01339
contig004	AUGUSTUS	exon	194824	195435	.	-	.	ID=MALK_01339.t1.e1;Parent=MALK_01339.t1

contig004	AUGUSTUS	gene	195493	196350	0.98	-	.	ID=MALK_01340;prediction_source=augustus:contig004.g122.t1
contig004	AUGUSTUS	CDS	195493	196350	0.98	-	0	ID=MALK_01340.t1.c1;Parent=MALK_01340.t1
contig004	AUGUSTUS	mRNA	195493	196350	0.98	-	.	ID=MALK_01340.t1;Parent=MALK_01340
contig004	AUGUSTUS	exon	195493	196350	0.98	-	.	ID=MALK_01340.t1.e1;Parent=MALK_01340.t1
contig004	maker	gene	196475	197914	.	+	.	ID=MALK_01341;prediction_source=maker_MRET:augustus_masked-contig004-processed-gene-2.48-mRNA-1
contig004	maker	CDS	196475	197914	.	+	0	ID=MALK_01341.t1.c1;Parent=MALK_01341.t1
contig004	maker	mRNA	196475	197914	.	+	.	ID=MALK_01341.t1;Parent=MALK_01341
contig004	maker	exon	196475	197914	.	+	.	ID=MALK_01341.t1.e1;Parent=MALK_01341.t1
contig004	maker	gene	197930	198691	.	-	.	ID=MALK_01342;prediction_source=maker_MRET:augustus_masked-contig004-processed-gene-2.50-mRNA-1
contig004	maker	CDS	197930	198691	.	-	0	ID=MALK_01342.t1.c1;Parent=MALK_01342.t1
contig004	maker	mRNA	197930	198691	.	-	.	ID=MALK_01342.t1;Parent=MALK_01342
contig004	maker	exon	197930	198691	.	-	.	ID=MALK_01342.t1.e1;Parent=MALK_01342.t1
contig004	AUGUSTUS	gene	199848	201101	0.34	-	.	ID=MALK_01343;prediction_source=braker_MRET:g508.t1
contig004	AUGUSTUS	CDS	200668	201101	0.63	-	0	ID=MALK_01343.t1.c3;Parent=MALK_01343.t1
contig004	AUGUSTUS	CDS	200593	200629	0.63	-	0	ID=MALK_01343.t1.c2;Parent=MALK_01343.t1
contig004	AUGUSTUS	CDS	199848	200498	0.63	-	0	ID=MALK_01343.t1.c1;Parent=MALK_01343.t1
contig004	AUGUSTUS	mRNA	199848	201101	0.34	-	.	ID=MALK_01343.t1;Parent=MALK_01343
contig004	AUGUSTUS	exon	200668	201101	.	-	.	ID=MALK_01343.t1.e3;Parent=MALK_01343.t1
contig004	AUGUSTUS	exon	200593	200629	.	-	.	ID=MALK_01343.t1.e2;Parent=MALK_01343.t1
contig004	AUGUSTUS	exon	199848	200498	.	-	.	ID=MALK_01343.t1.e1;Parent=MALK_01343.t1
contig004	maker	gene	201100	202428	.	+	.	ID=MALK_01344;prediction_source=maker_MRET:augustus_masked-contig004-processed-gene-2.51-mRNA-1
contig004	maker	CDS	201100	202428	.	+	0	ID=MALK_01344.t1.c1;Parent=MALK_01344.t1
contig004	maker	mRNA	201100	202428	.	+	.	ID=MALK_01344.t1;Parent=MALK_01344
contig004	maker	exon	201100	202428	.	+	.	ID=MALK_01344.t1.e1;Parent=MALK_01344.t1
contig004	AUGUSTUS	gene	202629	204233	0.71	+	.	ID=MALK_01345;prediction_source=braker_MRET:g510.t1
contig004	AUGUSTUS	CDS	202629	204233	0.71	+	0	ID=MALK_01345.t1.c1;Parent=MALK_01345.t1
contig004	AUGUSTUS	mRNA	202629	204233	0.71	+	.	ID=MALK_01345.t1;Parent=MALK_01345
contig004	AUGUSTUS	exon	202629	204233	.	+	.	ID=MALK_01345.t1.e1;Parent=MALK_01345.t1
contig004	maker	gene	204256	205785	.	-	.	ID=MALK_01346;prediction_source=maker_MRET:augustus_masked-contig004-processed-gene-2.78-mRNA-1
contig004	maker	CDS	204256	205785	.	-	0	ID=MALK_01346.t1.c1;Parent=MALK_01346.t1
contig004	maker	mRNA	204256	205785	.	-	.	ID=MALK_01346.t1;Parent=MALK_01346
contig004	maker	exon	204256	205785	.	-	.	ID=MALK_01346.t1.e1;Parent=MALK_01346.t1
contig004	AUGUSTUS	gene	206027	209443	0.67	+	.	ID=MALK_01347;prediction_source=augustus:contig004.g131.t1
contig004	AUGUSTUS	CDS	206027	209443	0.67	+	0	ID=MALK_01347.t1.c1;Parent=MALK_01347.t1
contig004	AUGUSTUS	mRNA	206027	209443	0.67	+	.	ID=MALK_01347.t1;Parent=MALK_01347
contig004	AUGUSTUS	exon	206027	209443	0.67	+	.	ID=MALK_01347.t1.e1;Parent=MALK_01347.t1
contig004	AUGUSTUS	gene	210271	213459	0.93	+	.	ID=MALK_01348;prediction_source=augustus:contig004.g132.t1
contig004	AUGUSTUS	CDS	210271	213459	0.93	+	0	ID=MALK_01348.t1.c1;Parent=MALK_01348.t1
contig004	AUGUSTUS	mRNA	210271	213459	0.93	+	.	ID=MALK_01348.t1;Parent=MALK_01348
contig004	AUGUSTUS	exon	210271	213459	0.93	+	.	ID=MALK_01348.t1.e1;Parent=MALK_01348.t1
contig004	AUGUSTUS	gene	213579	214895	0.94	+	.	ID=MALK_01349;prediction_source=augustus:contig004.g133.t1
contig004	AUGUSTUS	CDS	213579	214895	0.94	+	0	ID=MALK_01349.t1.c1;Parent=MALK_01349.t1
contig004	AUGUSTUS	mRNA	213579	214895	0.94	+	.	ID=MALK_01349.t1;Parent=MALK_01349

contig004	AUGUSTUS	exon	213579	214895	0.94	+	.	ID=MALK_01349.t1.e1;Parent=MALK_01349.t1
contig004	maker	gene	215379	217013	.	-	.	ID=MALK_01350;prediction_source=maker_MRET:augustus_masked-contig004-processed-gene-2.79-mRNA-1
contig004	maker	CDS	215379	217013	.	-	0	ID=MALK_01350.t1.c1;Parent=MALK_01350.t1
contig004	maker	mRNA	215379	217013	.	-	.	ID=MALK_01350.t1;Parent=MALK_01350
contig004	maker	exon	215379	217013	.	-	.	ID=MALK_01350.t1.e1;Parent=MALK_01350.t1
contig004	AUGUSTUS	gene	217161	218574	0.69	+	.	ID=MALK_01351;prediction_source=augustus:contig004.g135.t1
contig004	AUGUSTUS	CDS	217161	217262	0.69	+	0	ID=MALK_01351.t1.c1;Parent=MALK_01351.t1
contig004	AUGUSTUS	CDS	217342	218574	0.69	+	0	ID=MALK_01351.t1.c2;Parent=MALK_01351.t1
contig004	AUGUSTUS	mRNA	217161	218574	0.69	+	.	ID=MALK_01351.t1;Parent=MALK_01351
contig004	AUGUSTUS	exon	217161	217262	0.69	+	.	ID=MALK_01351.t1.e1;Parent=MALK_01351.t1
contig004	AUGUSTUS	exon	217342	218574	0.69	+	.	ID=MALK_01351.t1.e2;Parent=MALK_01351.t1
contig004	maker	gene	219402	221117	.	+	.	ID=MALK_01352;prediction_source=maker_MRET:augustus_masked-contig004-processed-gene-2.57-mRNA-1
contig004	maker	CDS	219402	221117	.	+	0	ID=MALK_01352.t1.c1;Parent=MALK_01352.t1
contig004	maker	mRNA	219402	221117	.	+	.	ID=MALK_01352.t1;Parent=MALK_01352
contig004	maker	exon	219402	221117	.	+	.	ID=MALK_01352.t1.e1;Parent=MALK_01352.t1
contig004	AUGUSTUS	gene	221241	222381	0.64	-	.	ID=MALK_01353;prediction_source=braker_MRET:g518.t1
contig004	AUGUSTUS	CDS	222036	222381	0.64	-	0	ID=MALK_01353.t1.c2;Parent=MALK_01353.t1
contig004	AUGUSTUS	CDS	221241	222007	0.64	-	0	ID=MALK_01353.t1.c1;Parent=MALK_01353.t1
contig004	AUGUSTUS	mRNA	221241	222381	0.64	-	.	ID=MALK_01353.t1;Parent=MALK_01353
contig004	AUGUSTUS	exon	222036	222381	.	-	.	ID=MALK_01353.t1.e2;Parent=MALK_01353.t1
contig004	AUGUSTUS	exon	221241	222007	.	-	.	ID=MALK_01353.t1.e1;Parent=MALK_01353.t1
contig004	maker	gene	222490	224313	.	-	.	ID=MALK_01354;prediction_source=maker_MRET:augustus_masked-contig004-processed-gene-2.81-mRNA-1
contig004	maker	CDS	222490	224313	.	-	0	ID=MALK_01354.t1.c1;Parent=MALK_01354.t1
contig004	maker	mRNA	222490	224313	.	-	.	ID=MALK_01354.t1;Parent=MALK_01354
contig004	maker	exon	222490	224313	.	-	.	ID=MALK_01354.t1.e1;Parent=MALK_01354.t1
contig004	AUGUSTUS	gene	224737	225291	0.99	+	.	ID=MALK_01355;prediction_source=augustus:contig004.g139.t1
contig004	AUGUSTUS	CDS	224737	225291	0.99	+	0	ID=MALK_01355.t1.c1;Parent=MALK_01355.t1
contig004	AUGUSTUS	mRNA	224737	225291	0.99	+	.	ID=MALK_01355.t1;Parent=MALK_01355
contig004	AUGUSTUS	exon	224737	225291	0.99	+	.	ID=MALK_01355.t1.e1;Parent=MALK_01355.t1
contig004	AUGUSTUS	gene	225617	227581	0.94	+	.	ID=MALK_01356;prediction_source=augustus:contig004.g140.t1
contig004	AUGUSTUS	CDS	225617	227581	0.94	+	0	ID=MALK_01356.t1.c1;Parent=MALK_01356.t1
contig004	AUGUSTUS	mRNA	225617	227581	0.94	+	.	ID=MALK_01356.t1;Parent=MALK_01356
contig004	AUGUSTUS	exon	225617	227581	0.94	+	.	ID=MALK_01356.t1.e1;Parent=MALK_01356.t1
contig004	AUGUSTUS	gene	227863	230607	0.6	+	.	ID=MALK_01357;prediction_source=augustus:contig004.g141.t1
contig004	AUGUSTUS	CDS	227863	230607	0.6	+	0	ID=MALK_01357.t1.c1;Parent=MALK_01357.t1
contig004	AUGUSTUS	mRNA	227863	230607	0.6	+	.	ID=MALK_01357.t1;Parent=MALK_01357
contig004	AUGUSTUS	exon	227863	230607	0.6	+	.	ID=MALK_01357.t1.e1;Parent=MALK_01357.t1
contig004	AUGUSTUS	gene	231108	235784	0.93	+	.	ID=MALK_01358;prediction_source=augustus:contig004.g142.t1
contig004	AUGUSTUS	CDS	231108	235784	0.93	+	0	ID=MALK_01358.t1.c1;Parent=MALK_01358.t1
contig004	AUGUSTUS	mRNA	231108	235784	0.93	+	.	ID=MALK_01358.t1;Parent=MALK_01358
contig004	AUGUSTUS	exon	231108	235784	0.93	+	.	ID=MALK_01358.t1.e1;Parent=MALK_01358.t1
contig004	AUGUSTUS	gene	236080	236595	0.95	+	.	ID=MALK_01359;prediction_source=braker_MRET:g523.t1
contig004	AUGUSTUS	CDS	236080	236114	1	+	0	ID=MALK_01359.t1.c1;Parent=MALK_01359.t1

contig004	AUGUSTUS	CDS	236150	236392	1	+	0	ID=MALK_01359.t1.c2;Parent=MALK_01359.t1
contig004	AUGUSTUS	CDS	236433	236595	1	+	0	ID=MALK_01359.t1.c3;Parent=MALK_01359.t1
contig004	AUGUSTUS	mRNA	236080	236595	0.95	+	.	ID=MALK_01359.t1;Parent=MALK_01359
contig004	AUGUSTUS	exon	236080	236114	.	+	.	ID=MALK_01359.t1.e1;Parent=MALK_01359.t1
contig004	AUGUSTUS	exon	236150	236392	.	+	.	ID=MALK_01359.t1.e2;Parent=MALK_01359.t1
contig004	AUGUSTUS	exon	236433	236595	.	+	.	ID=MALK_01359.t1.e3;Parent=MALK_01359.t1
contig004	maker	gene	237345	238784	.	+	.	ID=MALK_01360;prediction_source=maker_MRET:augustus_masked-contig004-processed-gene-2.63-mRNA-1
contig004	maker	CDS	237345	238784	.	+	0	ID=MALK_01360.t1.c1;Parent=MALK_01360.t1
contig004	maker	mRNA	237345	238784	.	+	.	ID=MALK_01360.t1;Parent=MALK_01360
contig004	maker	exon	237345	238784	.	+	.	ID=MALK_01360.t1.e1;Parent=MALK_01360.t1
contig004	AUGUSTUS	gene	238801	241446	0.99	-	.	ID=MALK_01361;prediction_source=augustus:contig004.g145.t1
contig004	AUGUSTUS	CDS	238801	241446	0.99	-	0	ID=MALK_01361.t1.c1;Parent=MALK_01361.t1
contig004	AUGUSTUS	mRNA	238801	241446	0.99	-	.	ID=MALK_01361.t1;Parent=MALK_01361
contig004	AUGUSTUS	exon	238801	241446	0.99	-	.	ID=MALK_01361.t1.e1;Parent=MALK_01361.t1
contig004	AUGUSTUS	gene	241515	242531	0.91	+	.	ID=MALK_01362;prediction_source=augustus:contig004.g146.t1
contig004	AUGUSTUS	CDS	241515	242531	0.91	+	0	ID=MALK_01362.t1.c1;Parent=MALK_01362.t1
contig004	AUGUSTUS	mRNA	241515	242531	0.91	+	.	ID=MALK_01362.t1;Parent=MALK_01362
contig004	AUGUSTUS	exon	241515	242531	0.91	+	.	ID=MALK_01362.t1.e1;Parent=MALK_01362.t1
contig004	maker	gene	242580	245729	.	-	.	ID=MALK_01363;prediction_source=maker_MRET:augustus_masked-contig004-processed-gene-2.83-mRNA-1
contig004	maker	CDS	242580	245729	.	-	0	ID=MALK_01363.t1.c1;Parent=MALK_01363.t1
contig004	maker	mRNA	242580	245729	.	-	.	ID=MALK_01363.t1;Parent=MALK_01363
contig004	maker	exon	242580	245729	.	-	.	ID=MALK_01363.t1.e1;Parent=MALK_01363.t1
contig004	maker	gene	246248	247087	.	-	.	ID=MALK_01364;prediction_source=maker_MRET:augustus_masked-contig004-processed-gene-2.84-mRNA-1
contig004	maker	CDS	246248	247087	.	-	0	ID=MALK_01364.t1.c1;Parent=MALK_01364.t1
contig004	maker	mRNA	246248	247087	.	-	.	ID=MALK_01364.t1;Parent=MALK_01364
contig004	maker	exon	246248	247087	.	-	.	ID=MALK_01364.t1.e1;Parent=MALK_01364.t1
contig004	AUGUSTUS	gene	247342	248967	0.96	+	.	ID=MALK_01365;prediction_source=augustus:contig004.g150.t1
contig004	AUGUSTUS	CDS	247342	248967	0.96	+	0	ID=MALK_01365.t1.c1;Parent=MALK_01365.t1
contig004	AUGUSTUS	mRNA	247342	248967	0.96	+	.	ID=MALK_01365.t1;Parent=MALK_01365
contig004	AUGUSTUS	exon	247342	248967	0.96	+	.	ID=MALK_01365.t1.e1;Parent=MALK_01365.t1
contig004	AUGUSTUS	gene	250164	251399	0.81	+	.	ID=MALK_01366;prediction_source=augustus:contig004.g151.t1
contig004	AUGUSTUS	CDS	250164	251399	0.81	+	0	ID=MALK_01366.t1.c1;Parent=MALK_01366.t1
contig004	AUGUSTUS	mRNA	250164	251399	0.81	+	.	ID=MALK_01366.t1;Parent=MALK_01366
contig004	AUGUSTUS	exon	250164	251399	0.81	+	.	ID=MALK_01366.t1.e1;Parent=MALK_01366.t1
contig004	AUGUSTUS	gene	251592	252329	0.99	+	.	ID=MALK_01367;prediction_source=augustus:contig004.g152.t1
contig004	AUGUSTUS	CDS	251592	252329	0.99	+	0	ID=MALK_01367.t1.c1;Parent=MALK_01367.t1
contig004	AUGUSTUS	mRNA	251592	252329	0.99	+	.	ID=MALK_01367.t1;Parent=MALK_01367
contig004	AUGUSTUS	exon	251592	252329	0.99	+	.	ID=MALK_01367.t1.e1;Parent=MALK_01367.t1
contig004	maker	gene	252404	254719	.	-	.	ID=MALK_01368;prediction_source=maker_MRET:augustus_masked-contig004-processed-gene-2.85-mRNA-1
contig004	maker	CDS	252404	254719	.	-	0	ID=MALK_01368.t1.c1;Parent=MALK_01368.t1
contig004	maker	mRNA	252404	254719	.	-	.	ID=MALK_01368.t1;Parent=MALK_01368
contig004	maker	exon	252404	254719	.	-	.	ID=MALK_01368.t1.e1;Parent=MALK_01368.t1
contig004	maker	gene	254818	257436	.	+	.	ID=MALK_01369;prediction_source=maker_MRET:augustus_masked-contig004-processed-gene-2.68-mRNA-1

contig004	maker	CDS	254818	257436	.	+	0	ID=MALK_01369.t1.c1;Parent=MALK_01369.t1
contig004	maker	mRNA	254818	257436	.	+	.	ID=MALK_01369.t1;Parent=MALK_01369
contig004	maker	exon	254818	257436	.	+	.	ID=MALK_01369.t1.e1;Parent=MALK_01369.t1
contig004	maker	gene	257551	257961	.	-	.	ID=MALK_01370;prediction_source=maker_MRET:augustus_masked-contig004-processed-gene-2.86-mRNA-1
contig004	maker	CDS	257551	257961	.	-	0	ID=MALK_01370.t1.c1;Parent=MALK_01370.t1
contig004	maker	mRNA	257551	257961	.	-	.	ID=MALK_01370.t1;Parent=MALK_01370
contig004	maker	exon	257551	257961	.	-	.	ID=MALK_01370.t1.e1;Parent=MALK_01370.t1
contig004	AUGUSTUS	gene	258073	259623	0.96	+	.	ID=MALK_01371;prediction_source=augustus:contig004.g156.t1
contig004	AUGUSTUS	CDS	258073	259623	0.96	+	0	ID=MALK_01371.t1.c1;Parent=MALK_01371.t1
contig004	AUGUSTUS	mRNA	258073	259623	0.96	+	.	ID=MALK_01371.t1;Parent=MALK_01371
contig004	AUGUSTUS	exon	258073	259623	0.96	+	.	ID=MALK_01371.t1.e1;Parent=MALK_01371.t1
contig004	maker	gene	259673	260479	.	-	.	ID=MALK_01372;prediction_source=maker_MRET:augustus_masked-contig004-processed-gene-2.87-mRNA-1
contig004	maker	CDS	259673	260479	.	-	0	ID=MALK_01372.t1.c1;Parent=MALK_01372.t1
contig004	maker	mRNA	259673	260479	.	-	.	ID=MALK_01372.t1;Parent=MALK_01372
contig004	maker	exon	259673	260479	.	-	.	ID=MALK_01372.t1.e1;Parent=MALK_01372.t1
contig004	AUGUSTUS	gene	260939	261648	1	-	.	ID=MALK_01373;prediction_source=braker_MRET:g537.t1
contig004	AUGUSTUS	CDS	261627	261648	1	-	0	ID=MALK_01373.t1.c3;Parent=MALK_01373.t1
contig004	AUGUSTUS	CDS	261518	261578	1	-	0	ID=MALK_01373.t1.c2;Parent=MALK_01373.t1
contig004	AUGUSTUS	CDS	260939	261476	1	-	0	ID=MALK_01373.t1.c1;Parent=MALK_01373.t1
contig004	AUGUSTUS	mRNA	260939	261648	1	-	.	ID=MALK_01373.t1;Parent=MALK_01373
contig004	AUGUSTUS	exon	261627	261648	.	-	.	ID=MALK_01373.t1.e3;Parent=MALK_01373.t1
contig004	AUGUSTUS	exon	261518	261578	.	-	.	ID=MALK_01373.t1.e2;Parent=MALK_01373.t1
contig004	AUGUSTUS	exon	260939	261476	.	-	.	ID=MALK_01373.t1.e1;Parent=MALK_01373.t1
contig004	AUGUSTUS	gene	261899	263653	0.76	-	.	ID=MALK_01374;prediction_source=augustus:contig004.g160.t1
contig004	AUGUSTUS	CDS	261899	263653	0.76	-	0	ID=MALK_01374.t1.c1;Parent=MALK_01374.t1
contig004	AUGUSTUS	mRNA	261899	263653	0.76	-	.	ID=MALK_01374.t1;Parent=MALK_01374
contig004	AUGUSTUS	exon	261899	263653	0.76	-	.	ID=MALK_01374.t1.e1;Parent=MALK_01374.t1
contig004	AUGUSTUS	gene	263847	264800	0.75	+	.	ID=MALK_01375;prediction_source=braker_MRET:g539.t1
contig004	AUGUSTUS	CDS	263847	264800	0.75	+	0	ID=MALK_01375.t1.c1;Parent=MALK_01375.t1
contig004	AUGUSTUS	mRNA	263847	264800	0.75	+	.	ID=MALK_01375.t1;Parent=MALK_01375
contig004	AUGUSTUS	exon	263847	264800	.	+	.	ID=MALK_01375.t1.e1;Parent=MALK_01375.t1
contig004	AUGUSTUS	gene	264821	267331	0.47	-	.	ID=MALK_01376;prediction_source=braker_MRET:g540.t1
contig004	AUGUSTUS	CDS	264821	267331	0.47	-	0	ID=MALK_01376.t1.c1;Parent=MALK_01376.t1
contig004	AUGUSTUS	mRNA	264821	267331	0.47	-	.	ID=MALK_01376.t1;Parent=MALK_01376
contig004	AUGUSTUS	exon	264821	267331	.	-	.	ID=MALK_01376.t1.e1;Parent=MALK_01376.t1
contig004	maker	gene	267561	268481	.	-	.	ID=MALK_01377;prediction_source=maker_MRET:augustus_masked-contig004-processed-gene-2.91-mRNA-1
contig004	maker	CDS	267561	268481	.	-	0	ID=MALK_01377.t1.c1;Parent=MALK_01377.t1
contig004	maker	mRNA	267561	268481	.	-	.	ID=MALK_01377.t1;Parent=MALK_01377
contig004	maker	exon	267561	268481	.	-	.	ID=MALK_01377.t1.e1;Parent=MALK_01377.t1
contig004	maker	gene	268652	269053	.	+	.	ID=MALK_01378;prediction_source=maker_MRET:augustus_masked-contig004-processed-gene-2.71-mRNA-1
contig004	maker	CDS	268652	269053	.	+	0	ID=MALK_01378.t1.c1;Parent=MALK_01378.t1
contig004	maker	mRNA	268652	269053	.	+	.	ID=MALK_01378.t1;Parent=MALK_01378
contig004	maker	exon	268652	269053	.	+	.	ID=MALK_01378.t1.e1;Parent=MALK_01378.t1

contig004	AUGUSTUS	gene	269139	271028	0.78	-	.	ID=MALK_01379;prediction_source=augustus:contig004.g166.t1
contig004	AUGUSTUS	CDS	269139	271028	0.78	-	0	ID=MALK_01379.t1.c1;Parent=MALK_01379.t1
contig004	AUGUSTUS	mRNA	269139	271028	0.78	-	.	ID=MALK_01379.t1;Parent=MALK_01379
contig004	AUGUSTUS	exon	269139	271028	0.78	-	.	ID=MALK_01379.t1.e1;Parent=MALK_01379.t1
contig004	maker	gene	271435	273909	.	-	.	ID=MALK_01380;prediction_source=maker_MRET:augustus_masked-contig004-processed-gene-2.93-mRNA-1
contig004	maker	CDS	271435	273909	.	-	0	ID=MALK_01380.t1.c1;Parent=MALK_01380.t1
contig004	maker	mRNA	271435	273909	.	-	.	ID=MALK_01380.t1;Parent=MALK_01380
contig004	maker	exon	271435	273909	.	-	.	ID=MALK_01380.t1.e1;Parent=MALK_01380.t1
contig004	maker	gene	274397	276925	.	+	.	ID=MALK_01381;prediction_source=maker_MRET:augustus_masked-contig004-processed-gene-2.72-mRNA-1
contig004	maker	CDS	274397	276925	.	+	0	ID=MALK_01381.t1.c1;Parent=MALK_01381.t1
contig004	maker	mRNA	274397	276925	.	+	.	ID=MALK_01381.t1;Parent=MALK_01381
contig004	maker	exon	274397	276925	.	+	.	ID=MALK_01381.t1.e1;Parent=MALK_01381.t1
contig004	maker	gene	277039	277422	.	-	.	ID=MALK_01382;prediction_source=maker_MRET:augustus_masked-contig004-processed-gene-2.94-mRNA-1
contig004	maker	CDS	277039	277422	.	-	0	ID=MALK_01382.t1.c1;Parent=MALK_01382.t1
contig004	maker	mRNA	277039	277422	.	-	.	ID=MALK_01382.t1;Parent=MALK_01382
contig004	maker	exon	277039	277422	.	-	.	ID=MALK_01382.t1.e1;Parent=MALK_01382.t1
contig004	AUGUSTUS	gene	277542	281999	0.57	+	.	ID=MALK_01383;prediction_source=augustus:contig004.g169.t1
contig004	AUGUSTUS	CDS	277542	281999	0.57	+	0	ID=MALK_01383.t1.c1;Parent=MALK_01383.t1
contig004	AUGUSTUS	mRNA	277542	281999	0.57	+	.	ID=MALK_01383.t1;Parent=MALK_01383
contig004	AUGUSTUS	exon	277542	281999	0.57	+	.	ID=MALK_01383.t1.e1;Parent=MALK_01383.t1
contig004	AUGUSTUS	gene	282031	283563	0.5	+	.	ID=MALK_01384;prediction_source=augustus:contig004.g171.t1
contig004	AUGUSTUS	CDS	282031	283563	0.5	+	0	ID=MALK_01384.t1.c1;Parent=MALK_01384.t1
contig004	AUGUSTUS	mRNA	282031	283563	0.5	+	.	ID=MALK_01384.t1;Parent=MALK_01384
contig004	AUGUSTUS	exon	282031	283563	0.5	+	.	ID=MALK_01384.t1.e1;Parent=MALK_01384.t1
contig004	maker	gene	283556	284151	.	-	.	ID=MALK_01385;prediction_source=maker_MRET:augustus_masked-contig004-processed-gene-2.95-mRNA-1
contig004	maker	CDS	284037	284151	.	-	0	ID=MALK_01385.t1.c1;Parent=MALK_01385.t1
contig004	maker	CDS	283556	283977	.	-	0	ID=MALK_01385.t1.c2;Parent=MALK_01385.t1
contig004	maker	mRNA	283556	284151	.	-	.	ID=MALK_01385.t1;Parent=MALK_01385
contig004	maker	exon	284037	284151	.	-	.	ID=MALK_01385.t1.e1;Parent=MALK_01385.t1
contig004	maker	exon	283556	283977	.	-	.	ID=MALK_01385.t1.e2;Parent=MALK_01385.t1
contig004	AUGUSTUS	gene	284197	285114	0.62	+	.	ID=MALK_01386;prediction_source=augustus:contig004.g173.t1
contig004	AUGUSTUS	CDS	284197	285114	0.62	+	0	ID=MALK_01386.t1.c1;Parent=MALK_01386.t1
contig004	AUGUSTUS	mRNA	284197	285114	0.62	+	.	ID=MALK_01386.t1;Parent=MALK_01386
contig004	AUGUSTUS	exon	284197	285114	0.62	+	.	ID=MALK_01386.t1.e1;Parent=MALK_01386.t1
contig004	AUGUSTUS	gene	285244	287336	0.67	+	.	ID=MALK_01387;prediction_source=braker_MRET:g551.t1
contig004	AUGUSTUS	CDS	285244	285269	0.85	+	0	ID=MALK_01387.t1.c1;Parent=MALK_01387.t1
contig004	AUGUSTUS	CDS	285306	285319	0.85	+	0	ID=MALK_01387.t1.c2;Parent=MALK_01387.t1
contig004	AUGUSTUS	CDS	285351	285443	0.85	+	0	ID=MALK_01387.t1.c3;Parent=MALK_01387.t1
contig004	AUGUSTUS	CDS	285473	287206	0.85	+	0	ID=MALK_01387.t1.c4;Parent=MALK_01387.t1
contig004	AUGUSTUS	CDS	287236	287336	0.85	+	0	ID=MALK_01387.t1.c5;Parent=MALK_01387.t1
contig004	AUGUSTUS	mRNA	285244	287336	0.67	+	.	ID=MALK_01387.t1;Parent=MALK_01387
contig004	AUGUSTUS	exon	285244	285269	.	+	.	ID=MALK_01387.t1.e1;Parent=MALK_01387.t1
contig004	AUGUSTUS	exon	285306	285319	.	+	.	ID=MALK_01387.t1.e2;Parent=MALK_01387.t1

contig004	AUGUSTUS	exon	285351	285443	.	+	.	ID=MALK_01387.t1.e3;Parent=MALK_01387.t1
contig004	AUGUSTUS	exon	285473	287206	.	+	.	ID=MALK_01387.t1.e4;Parent=MALK_01387.t1
contig004	AUGUSTUS	exon	287236	287336	.	+	.	ID=MALK_01387.t1.e5;Parent=MALK_01387.t1
contig004	AUGUSTUS	gene	287449	289074	0.72	+	.	ID=MALK_01388;prediction_source=braker_MRET:g552.t1
contig004	AUGUSTUS	CDS	287449	287586	0.98	+	0	ID=MALK_01388.t1.c1;Parent=MALK_01388.t1
contig004	AUGUSTUS	CDS	287623	289074	0.98	+	0	ID=MALK_01388.t1.c2;Parent=MALK_01388.t1
contig004	AUGUSTUS	mRNA	287449	289074	0.72	+	.	ID=MALK_01388.t1;Parent=MALK_01388
contig004	AUGUSTUS	exon	287449	287586	.	+	.	ID=MALK_01388.t1.e1;Parent=MALK_01388.t1
contig004	AUGUSTUS	exon	287623	289074	.	+	.	ID=MALK_01388.t1.e2;Parent=MALK_01388.t1
contig004	maker	gene	289077	291263	.	-	.	ID=MALK_01389;prediction_source=maker_MRET:augustus_masked-contig004-processed-gene-2.96-mRNA-1
contig004	maker	CDS	289077	291263	.	-	0	ID=MALK_01389.t1.c1;Parent=MALK_01389.t1
contig004	maker	mRNA	289077	291263	.	-	.	ID=MALK_01389.t1;Parent=MALK_01389
contig004	maker	exon	289077	291263	.	-	.	ID=MALK_01389.t1.e1;Parent=MALK_01389.t1
contig004	maker	gene	291481	292902	.	+	.	ID=MALK_01390;prediction_source=maker_MRET:augustus_masked-contig004-processed-gene-2.77-mRNA-1
contig004	maker	CDS	291481	292902	.	+	0	ID=MALK_01390.t1.c1;Parent=MALK_01390.t1
contig004	maker	mRNA	291481	292902	.	+	.	ID=MALK_01390.t1;Parent=MALK_01390
contig004	maker	exon	291481	292902	.	+	.	ID=MALK_01390.t1.e1;Parent=MALK_01390.t1
contig004	maker	gene	292948	294594	.	-	.	ID=MALK_01391;prediction_source=maker_MRET:augustus_masked-contig004-processed-gene-2.97-mRNA-1
contig004	maker	CDS	292948	294594	.	-	0	ID=MALK_01391.t1.c1;Parent=MALK_01391.t1
contig004	maker	mRNA	292948	294594	.	-	.	ID=MALK_01391.t1;Parent=MALK_01391
contig004	maker	exon	292948	294594	.	-	.	ID=MALK_01391.t1.e1;Parent=MALK_01391.t1
contig004	AUGUSTUS	gene	294753	295664	0.77	+	.	ID=MALK_01392;prediction_source=augustus:contig004.g180.t1
contig004	AUGUSTUS	CDS	294753	295664	0.77	+	0	ID=MALK_01392.t1.c1;Parent=MALK_01392.t1
contig004	AUGUSTUS	mRNA	294753	295664	0.77	+	.	ID=MALK_01392.t1;Parent=MALK_01392
contig004	AUGUSTUS	exon	294753	295664	0.77	+	.	ID=MALK_01392.t1.e1;Parent=MALK_01392.t1
contig004	AUGUSTUS	gene	295911	297089	1	+	.	ID=MALK_01393;prediction_source=augustus:contig004.g181.t1
contig004	AUGUSTUS	CDS	295911	297089	1	+	0	ID=MALK_01393.t1.c1;Parent=MALK_01393.t1
contig004	AUGUSTUS	mRNA	295911	297089	1	+	.	ID=MALK_01393.t1;Parent=MALK_01393
contig004	AUGUSTUS	exon	295911	297089	1	+	.	ID=MALK_01393.t1.e1;Parent=MALK_01393.t1
contig004	AUGUSTUS	gene	297108	300036	0.54	-	.	ID=MALK_01394;prediction_source=augustus:contig004.g182.t1
contig004	AUGUSTUS	CDS	299722	300036	0.83	-	0	ID=MALK_01394.t1.c2;Parent=MALK_01394.t1
contig004	AUGUSTUS	CDS	297108	299330	0.83	-	0	ID=MALK_01394.t1.c1;Parent=MALK_01394.t1
contig004	AUGUSTUS	mRNA	297108	300036	0.54	-	.	ID=MALK_01394.t1;Parent=MALK_01394
contig004	AUGUSTUS	exon	299722	300036	0.83	-	.	ID=MALK_01394.t1.e2;Parent=MALK_01394.t1
contig004	AUGUSTUS	exon	297108	299330	0.83	-	.	ID=MALK_01394.t1.e1;Parent=MALK_01394.t1
contig004	AUGUSTUS	gene	300301	302481	0.96	+	.	ID=MALK_01395;prediction_source=braker_MRET:g559.t1
contig004	AUGUSTUS	CDS	300301	302481	0.96	+	0	ID=MALK_01395.t1.c1;Parent=MALK_01395.t1
contig004	AUGUSTUS	mRNA	300301	302481	0.96	+	.	ID=MALK_01395.t1;Parent=MALK_01395
contig004	AUGUSTUS	exon	300301	302481	.	+	.	ID=MALK_01395.t1.e1;Parent=MALK_01395.t1
contig004	AUGUSTUS	gene	302467	303888	0.66	-	.	ID=MALK_01396;prediction_source=augustus:contig004.g185.t1
contig004	AUGUSTUS	CDS	302467	303888	0.66	-	0	ID=MALK_01396.t1.c1;Parent=MALK_01396.t1
contig004	AUGUSTUS	mRNA	302467	303888	0.66	-	.	ID=MALK_01396.t1;Parent=MALK_01396
contig004	AUGUSTUS	exon	302467	303888	0.66	-	.	ID=MALK_01396.t1.e1;Parent=MALK_01396.t1

contig004	AUGUSTUS	gene	303992	304699	0.46	+	.	ID=MALK_01397;prediction_source=augustus:contig004.g186.t1
contig004	AUGUSTUS	CDS	303992	304699	0.46	+	0	ID=MALK_01397.t1.c1;Parent=MALK_01397.t1
contig004	AUGUSTUS	mRNA	303992	304699	0.46	+	.	ID=MALK_01397.t1;Parent=MALK_01397
contig004	AUGUSTUS	exon	303992	304699	0.46	+	.	ID=MALK_01397.t1.e1;Parent=MALK_01397.t1
contig004	maker	gene	304737	305681	.	+	.	ID=MALK_01398;prediction_source=maker_MRET:augustus_masked-contig004-processed-gene-3.46-mRNA-1
contig004	maker	CDS	304737	305681	.	+	0	ID=MALK_01398.t1.c1;Parent=MALK_01398.t1
contig004	maker	mRNA	304737	305681	.	+	.	ID=MALK_01398.t1;Parent=MALK_01398
contig004	maker	exon	304737	305681	.	+	.	ID=MALK_01398.t1.e1;Parent=MALK_01398.t1
contig004	AUGUSTUS	gene	305689	307932	0.39	-	.	ID=MALK_01399;prediction_source=augustus:contig004.g188.t1
contig004	AUGUSTUS	CDS	305689	307932	0.39	-	0	ID=MALK_01399.t1.c1;Parent=MALK_01399.t1
contig004	AUGUSTUS	mRNA	305689	307932	0.39	-	.	ID=MALK_01399.t1;Parent=MALK_01399
contig004	AUGUSTUS	exon	305689	307932	0.39	-	.	ID=MALK_01399.t1.e1;Parent=MALK_01399.t1
contig004	AUGUSTUS	gene	308140	309093	0.88	-	.	ID=MALK_01400;prediction_source=augustus:contig004.g189.t1
contig004	AUGUSTUS	CDS	308140	309093	0.88	-	0	ID=MALK_01400.t1.c1;Parent=MALK_01400.t1
contig004	AUGUSTUS	mRNA	308140	309093	0.88	-	.	ID=MALK_01400.t1;Parent=MALK_01400
contig004	AUGUSTUS	exon	308140	309093	0.88	-	.	ID=MALK_01400.t1.e1;Parent=MALK_01400.t1
contig004	AUGUSTUS	gene	309159	309665	1	+	.	ID=MALK_01401;prediction_source=braker_MRET:g564.t1
contig004	AUGUSTUS	CDS	309159	309240	1	+	0	ID=MALK_01401.t1.c1;Parent=MALK_01401.t1
contig004	AUGUSTUS	CDS	309268	309665	1	+	0	ID=MALK_01401.t1.c2;Parent=MALK_01401.t1
contig004	AUGUSTUS	mRNA	309159	309665	1	+	.	ID=MALK_01401.t1;Parent=MALK_01401
contig004	AUGUSTUS	exon	309159	309240	.	+	.	ID=MALK_01401.t1.e1;Parent=MALK_01401.t1
contig004	AUGUSTUS	exon	309268	309665	.	+	.	ID=MALK_01401.t1.e2;Parent=MALK_01401.t1
contig004	AUGUSTUS	gene	309690	310849	0.65	-	.	ID=MALK_01402;prediction_source=braker_MRET:g565.t1
contig004	AUGUSTUS	CDS	309749	310849	0.65	-	0	ID=MALK_01402.t1.c2;Parent=MALK_01402.t1
contig004	AUGUSTUS	CDS	309690	309707	0.65	-	0	ID=MALK_01402.t1.c1;Parent=MALK_01402.t1
contig004	AUGUSTUS	mRNA	309690	310849	0.65	-	.	ID=MALK_01402.t1;Parent=MALK_01402
contig004	AUGUSTUS	exon	309749	310849	.	-	.	ID=MALK_01402.t1.e2;Parent=MALK_01402.t1
contig004	AUGUSTUS	exon	309690	309707	.	-	.	ID=MALK_01402.t1.e1;Parent=MALK_01402.t1
contig004	maker	gene	311020	315949	.	+	.	ID=MALK_01403;prediction_source=maker_MRET:augustus_masked-contig004-processed-gene-3.47-mRNA-1
contig004	maker	CDS	311020	312189	.	+	0	ID=MALK_01403.t1.c1;Parent=MALK_01403.t1
contig004	maker	CDS	312692	315949	.	+	0	ID=MALK_01403.t1.c2;Parent=MALK_01403.t1
contig004	maker	mRNA	311020	315949	.	+	.	ID=MALK_01403.t1;Parent=MALK_01403
contig004	maker	exon	311020	312189	.	+	.	ID=MALK_01403.t1.e1;Parent=MALK_01403.t1
contig004	maker	exon	312692	315949	.	+	.	ID=MALK_01403.t1.e2;Parent=MALK_01403.t1
contig004	AUGUSTUS	gene	315992	317827	0.88	-	.	ID=MALK_01404;prediction_source=augustus:contig004.g192.t1
contig004	AUGUSTUS	CDS	315992	317827	0.88	-	0	ID=MALK_01404.t1.c1;Parent=MALK_01404.t1
contig004	AUGUSTUS	mRNA	315992	317827	0.88	-	.	ID=MALK_01404.t1;Parent=MALK_01404
contig004	AUGUSTUS	exon	315992	317827	0.88	-	.	ID=MALK_01404.t1.e1;Parent=MALK_01404.t1
contig004	maker	gene	317998	321087	.	+	.	ID=MALK_01405;prediction_source=maker_MRET:augustus_masked-contig004-processed-gene-3.48-mRNA-1
contig004	maker	CDS	317998	321087	.	+	0	ID=MALK_01405.t1.c1;Parent=MALK_01405.t1
contig004	maker	mRNA	317998	321087	.	+	.	ID=MALK_01405.t1;Parent=MALK_01405
contig004	maker	exon	317998	321087	.	+	.	ID=MALK_01405.t1.e1;Parent=MALK_01405.t1
contig004	AUGUSTUS	gene	321248	324721	0.64	+	.	ID=MALK_01406;prediction_source=augustus:contig004.g195.t1

contig004	AUGUSTUS	CDS	321248	321316	0.79	+	0	ID=MALK_01406.t1.c1;Parent=MALK_01406.t1
contig004	AUGUSTUS	CDS	321401	324721	0.79	+	0	ID=MALK_01406.t1.c2;Parent=MALK_01406.t1
contig004	AUGUSTUS	mRNA	321248	324721	0.64	+	.	ID=MALK_01406.t1;Parent=MALK_01406
contig004	AUGUSTUS	exon	321248	321316	0.79	+	.	ID=MALK_01406.t1.e1;Parent=MALK_01406.t1
contig004	AUGUSTUS	exon	321401	324721	0.79	+	.	ID=MALK_01406.t1.e2;Parent=MALK_01406.t1
contig004	AUGUSTUS	gene	325059	326513	0.9	-	.	ID=MALK_01407;prediction_source=augustus:contig004.g196.t1
contig004	AUGUSTUS	CDS	325059	326513	0.9	-	0	ID=MALK_01407.t1.c1;Parent=MALK_01407.t1
contig004	AUGUSTUS	mRNA	325059	326513	0.9	-	.	ID=MALK_01407.t1;Parent=MALK_01407
contig004	AUGUSTUS	exon	325059	326513	0.9	-	.	ID=MALK_01407.t1.e1;Parent=MALK_01407.t1
contig004	AUGUSTUS	gene	326665	327528	0.28	-	.	ID=MALK_01408;prediction_source=braker_MRET:g571.t1
contig004	AUGUSTUS	CDS	327042	327528	0.65	-	0	ID=MALK_01408.t1.c3;Parent=MALK_01408.t1
contig004	AUGUSTUS	CDS	326902	327002	0.65	-	0	ID=MALK_01408.t1.c2;Parent=MALK_01408.t1
contig004	AUGUSTUS	CDS	326665	326817	0.65	-	0	ID=MALK_01408.t1.c1;Parent=MALK_01408.t1
contig004	AUGUSTUS	mRNA	326665	327528	0.28	-	.	ID=MALK_01408.t1;Parent=MALK_01408
contig004	AUGUSTUS	exon	327042	327528	.	-	.	ID=MALK_01408.t1.e3;Parent=MALK_01408.t1
contig004	AUGUSTUS	exon	326902	327002	.	-	.	ID=MALK_01408.t1.e2;Parent=MALK_01408.t1
contig004	AUGUSTUS	exon	326665	326817	.	-	.	ID=MALK_01408.t1.e1;Parent=MALK_01408.t1
contig004	maker	gene	327698	328507	.	+	.	ID=MALK_01409;prediction_source=maker_MRET:augustus_masked-contig004-processed-gene-3.50-mRNA-1
contig004	maker	CDS	327698	328507	.	+	0	ID=MALK_01409.t1.c1;Parent=MALK_01409.t1
contig004	maker	mRNA	327698	328507	.	+	.	ID=MALK_01409.t1;Parent=MALK_01409
contig004	maker	exon	327698	328507	.	+	.	ID=MALK_01409.t1.e1;Parent=MALK_01409.t1
contig004	AUGUSTUS	gene	328548	330278	0.61	-	.	ID=MALK_01410;prediction_source=augustus:contig004.g199.t1
contig004	AUGUSTUS	CDS	328548	330278	0.61	-	0	ID=MALK_01410.t1.c1;Parent=MALK_01410.t1
contig004	AUGUSTUS	mRNA	328548	330278	0.61	-	.	ID=MALK_01410.t1;Parent=MALK_01410
contig004	AUGUSTUS	exon	328548	330278	0.61	-	.	ID=MALK_01410.t1.e1;Parent=MALK_01410.t1
contig004	maker	gene	330483	334055	.	+	.	ID=MALK_01411;prediction_source=maker_MRET:augustus_masked-contig004-processed-gene-3.51-mRNA-1
contig004	maker	CDS	330483	334055	.	+	0	ID=MALK_01411.t1.c1;Parent=MALK_01411.t1
contig004	maker	mRNA	330483	334055	.	+	.	ID=MALK_01411.t1;Parent=MALK_01411
contig004	maker	exon	330483	334055	.	+	.	ID=MALK_01411.t1.e1;Parent=MALK_01411.t1
contig004	AUGUSTUS	gene	334061	334549	0.92	-	.	ID=MALK_01412;prediction_source=augustus:contig004.g202.t1
contig004	AUGUSTUS	CDS	334061	334549	0.92	-	0	ID=MALK_01412.t1.c1;Parent=MALK_01412.t1
contig004	AUGUSTUS	mRNA	334061	334549	0.92	-	.	ID=MALK_01412.t1;Parent=MALK_01412
contig004	AUGUSTUS	exon	334061	334549	0.92	-	.	ID=MALK_01412.t1.e1;Parent=MALK_01412.t1
contig004	AUGUSTUS	gene	334911	336104	0.59	-	.	ID=MALK_01413;prediction_source=braker_MRET:g576.t1
contig004	AUGUSTUS	CDS	334911	336104	0.59	-	0	ID=MALK_01413.t1.c1;Parent=MALK_01413.t1
contig004	AUGUSTUS	mRNA	334911	336104	0.59	-	.	ID=MALK_01413.t1;Parent=MALK_01413
contig004	AUGUSTUS	exon	334911	336104	.	-	.	ID=MALK_01413.t1.e1;Parent=MALK_01413.t1
contig004	maker	gene	336384	336782	.	+	.	ID=MALK_01414;prediction_source=maker_MRET:augustus_masked-contig004-processed-gene-3.52-mRNA-1
contig004	maker	CDS	336384	336782	.	+	0	ID=MALK_01414.t1.c1;Parent=MALK_01414.t1
contig004	maker	mRNA	336384	336782	.	+	.	ID=MALK_01414.t1;Parent=MALK_01414
contig004	maker	exon	336384	336782	.	+	.	ID=MALK_01414.t1.e1;Parent=MALK_01414.t1
contig004	AUGUSTUS	gene	336972	338863	0.98	-	.	ID=MALK_01415;prediction_source=braker_MRET:g578.t1
contig004	AUGUSTUS	CDS	338847	338863	0.98	-	0	ID=MALK_01415.t1.c2;Parent=MALK_01415.t1

contig004	AUGUSTUS	CDS	336972	338730	0.98	-	0	ID=MALK_01415.t1.c1;Parent=MALK_01415.t1
contig004	AUGUSTUS	mRNA	336972	338863	0.98	-	.	ID=MALK_01415.t1;Parent=MALK_01415
contig004	AUGUSTUS	exon	338847	338863	.	-	.	ID=MALK_01415.t1.e2;Parent=MALK_01415.t1
contig004	AUGUSTUS	exon	336972	338730	.	-	.	ID=MALK_01415.t1.e1;Parent=MALK_01415.t1
contig004	AUGUSTUS	gene	339376	341214	0.39	+	.	ID=MALK_01416;prediction_source=braker_MRET:g579.t1
contig004	AUGUSTUS	CDS	339376	341214	0.39	+	0	ID=MALK_01416.t1.c1;Parent=MALK_01416.t1
contig004	AUGUSTUS	mRNA	339376	341214	0.39	+	.	ID=MALK_01416.t1;Parent=MALK_01416
contig004	AUGUSTUS	exon	339376	341214	.	+	.	ID=MALK_01416.t1.e1;Parent=MALK_01416.t1
contig004	AUGUSTUS	gene	341309	341805	0.57	+	.	ID=MALK_01417;prediction_source=braker_MRET:g580.t1
contig004	AUGUSTUS	CDS	341309	341438	0.92	+	0	ID=MALK_01417.t1.c1;Parent=MALK_01417.t1
contig004	AUGUSTUS	CDS	341480	341550	0.92	+	0	ID=MALK_01417.t1.c2;Parent=MALK_01417.t1
contig004	AUGUSTUS	CDS	341583	341686	0.92	+	0	ID=MALK_01417.t1.c3;Parent=MALK_01417.t1
contig004	AUGUSTUS	CDS	341718	341805	0.92	+	0	ID=MALK_01417.t1.c4;Parent=MALK_01417.t1
contig004	AUGUSTUS	mRNA	341309	341805	0.57	+	.	ID=MALK_01417.t1;Parent=MALK_01417
contig004	AUGUSTUS	exon	341309	341438	.	+	.	ID=MALK_01417.t1.e1;Parent=MALK_01417.t1
contig004	AUGUSTUS	exon	341480	341550	.	+	.	ID=MALK_01417.t1.e2;Parent=MALK_01417.t1
contig004	AUGUSTUS	exon	341583	341686	.	+	.	ID=MALK_01417.t1.e3;Parent=MALK_01417.t1
contig004	AUGUSTUS	exon	341718	341805	.	+	.	ID=MALK_01417.t1.e4;Parent=MALK_01417.t1
contig004	AUGUSTUS	gene	341963	343299	1	-	.	ID=MALK_01418;prediction_source=braker_MRET:g581.t1
contig004	AUGUSTUS	CDS	343293	343299	1	-	0	ID=MALK_01418.t1.c5;Parent=MALK_01418.t1
contig004	AUGUSTUS	CDS	343196	343209	1	-	0	ID=MALK_01418.t1.c4;Parent=MALK_01418.t1
contig004	AUGUSTUS	CDS	343082	343155	1	-	0	ID=MALK_01418.t1.c3;Parent=MALK_01418.t1
contig004	AUGUSTUS	CDS	342984	343038	1	-	0	ID=MALK_01418.t1.c2;Parent=MALK_01418.t1
contig004	AUGUSTUS	CDS	341963	342940	1	-	0	ID=MALK_01418.t1.c1;Parent=MALK_01418.t1
contig004	AUGUSTUS	mRNA	341963	343299	1	-	.	ID=MALK_01418.t1;Parent=MALK_01418
contig004	AUGUSTUS	exon	343293	343299	.	-	.	ID=MALK_01418.t1.e5;Parent=MALK_01418.t1
contig004	AUGUSTUS	exon	343196	343209	.	-	.	ID=MALK_01418.t1.e4;Parent=MALK_01418.t1
contig004	AUGUSTUS	exon	343082	343155	.	-	.	ID=MALK_01418.t1.e3;Parent=MALK_01418.t1
contig004	AUGUSTUS	exon	342984	343038	.	-	.	ID=MALK_01418.t1.e2;Parent=MALK_01418.t1
contig004	AUGUSTUS	exon	341963	342940	.	-	.	ID=MALK_01418.t1.e1;Parent=MALK_01418.t1
contig004	maker	gene	343713	345257	.	-	.	ID=MALK_01419;prediction_source=maker_MRET:augustus_masked-contig004-processed-gene-3.71-mRNA-1
contig004	maker	CDS	343713	345257	.	-	0	ID=MALK_01419.t1.c1;Parent=MALK_01419.t1
contig004	maker	mRNA	343713	345257	.	-	.	ID=MALK_01419.t1;Parent=MALK_01419
contig004	maker	exon	343713	345257	.	-	.	ID=MALK_01419.t1.e1;Parent=MALK_01419.t1
contig004	AUGUSTUS	gene	345501	346331	1	+	.	ID=MALK_01420;prediction_source=augustus:contig004.g211.t1
contig004	AUGUSTUS	CDS	345501	346331	1	+	0	ID=MALK_01420.t1.c1;Parent=MALK_01420.t1
contig004	AUGUSTUS	mRNA	345501	346331	1	+	.	ID=MALK_01420.t1;Parent=MALK_01420
contig004	AUGUSTUS	exon	345501	346331	1	+	.	ID=MALK_01420.t1.e1;Parent=MALK_01420.t1
contig004	AUGUSTUS	gene	346342	350085	0.98	-	.	ID=MALK_01421;prediction_source=augustus:contig004.g212.t1
contig004	AUGUSTUS	CDS	346342	350085	0.98	-	0	ID=MALK_01421.t1.c1;Parent=MALK_01421.t1
contig004	AUGUSTUS	mRNA	346342	350085	0.98	-	.	ID=MALK_01421.t1;Parent=MALK_01421
contig004	AUGUSTUS	exon	346342	350085	0.98	-	.	ID=MALK_01421.t1.e1;Parent=MALK_01421.t1
contig004	maker	gene	350273	351037	.	+	.	ID=MALK_01422;prediction_source=maker_MRET:augustus_masked-contig004-processed-gene-3.55-mRNA-1

contig004	maker	CDS	350273	351037	.	+	0	ID=MALK_01422.t1.c1;Parent=MALK_01422.t1
contig004	maker	mRNA	350273	351037	.	+	.	ID=MALK_01422.t1;Parent=MALK_01422
contig004	maker	exon	350273	351037	.	+	.	ID=MALK_01422.t1.e1;Parent=MALK_01422.t1
contig004	AUGUSTUS	gene	350973	352085	0.87	-	.	ID=MALK_01423;prediction_source=augustus:contig004.g214.t1
contig004	AUGUSTUS	CDS	350973	352085	0.87	-	0	ID=MALK_01423.t1.c1;Parent=MALK_01423.t1
contig004	AUGUSTUS	mRNA	350973	352085	0.87	-	.	ID=MALK_01423.t1;Parent=MALK_01423
contig004	AUGUSTUS	exon	350973	352085	0.87	-	.	ID=MALK_01423.t1.e1;Parent=MALK_01423.t1
contig004	maker	gene	352365	353816	.	+	.	ID=MALK_01424;prediction_source=maker_MRET:augustus_masked-contig004-processed-gene-3.56-mRNA-1
contig004	maker	CDS	352365	353816	.	+	0	ID=MALK_01424.t1.c1;Parent=MALK_01424.t1
contig004	maker	mRNA	352365	353816	.	+	.	ID=MALK_01424.t1;Parent=MALK_01424
contig004	maker	exon	352365	353816	.	+	.	ID=MALK_01424.t1.e1;Parent=MALK_01424.t1
contig004	AUGUSTUS	gene	353915	354498	0.96	-	.	ID=MALK_01425;prediction_source=braker_MRET:g587.t1
contig004	AUGUSTUS	CDS	354078	354498	0.98	-	0	ID=MALK_01425.t1.c2;Parent=MALK_01425.t1
contig004	AUGUSTUS	CDS	353915	354048	0.98	-	0	ID=MALK_01425.t1.c1;Parent=MALK_01425.t1
contig004	AUGUSTUS	mRNA	353915	354498	0.96	-	.	ID=MALK_01425.t1;Parent=MALK_01425
contig004	AUGUSTUS	exon	354078	354498	.	-	.	ID=MALK_01425.t1.e2;Parent=MALK_01425.t1
contig004	AUGUSTUS	exon	353915	354048	.	-	.	ID=MALK_01425.t1.e1;Parent=MALK_01425.t1
contig004	AUGUSTUS	gene	354805	356106	1	-	.	ID=MALK_01426;prediction_source=augustus:contig004.g217.t1
contig004	AUGUSTUS	CDS	354805	356106	1	-	0	ID=MALK_01426.t1.c1;Parent=MALK_01426.t1
contig004	AUGUSTUS	mRNA	354805	356106	1	-	.	ID=MALK_01426.t1;Parent=MALK_01426
contig004	AUGUSTUS	exon	354805	356106	1	-	.	ID=MALK_01426.t1.e1;Parent=MALK_01426.t1
contig004	maker	gene	356272	358779	.	-	.	ID=MALK_01427;prediction_source=maker_MRET:augustus_masked-contig004-processed-gene-3.75-mRNA-1
contig004	maker	CDS	356272	358779	.	-	0	ID=MALK_01427.t1.c1;Parent=MALK_01427.t1
contig004	maker	mRNA	356272	358779	.	-	.	ID=MALK_01427.t1;Parent=MALK_01427
contig004	maker	exon	356272	358779	.	-	.	ID=MALK_01427.t1.e1;Parent=MALK_01427.t1
contig004	maker	gene	358850	359953	.	-	.	ID=MALK_01428;prediction_source=maker_MRET:augustus_masked-contig004-processed-gene-3.76-mRNA-1
contig004	maker	CDS	358850	359953	.	-	0	ID=MALK_01428.t1.c1;Parent=MALK_01428.t1
contig004	maker	mRNA	358850	359953	.	-	.	ID=MALK_01428.t1;Parent=MALK_01428
contig004	maker	exon	358850	359953	.	-	.	ID=MALK_01428.t1.e1;Parent=MALK_01428.t1
contig004	maker	gene	360078	362976	.	+	.	ID=MALK_01429;prediction_source=maker_MRET:augustus_masked-contig004-processed-gene-3.57-mRNA-1
contig004	maker	CDS	360078	361614	.	+	0	ID=MALK_01429.t1.c1;Parent=MALK_01429.t1
contig004	maker	CDS	361997	362976	.	+	0	ID=MALK_01429.t1.c2;Parent=MALK_01429.t1
contig004	maker	mRNA	360078	362976	.	+	.	ID=MALK_01429.t1;Parent=MALK_01429
contig004	maker	exon	360078	361614	.	+	.	ID=MALK_01429.t1.e1;Parent=MALK_01429.t1
contig004	maker	exon	361997	362976	.	+	.	ID=MALK_01429.t1.e2;Parent=MALK_01429.t1
contig004	AUGUSTUS	gene	362986	363534	0.93	-	.	ID=MALK_01430;prediction_source=augustus:contig004.g222.t1
contig004	AUGUSTUS	CDS	362986	363534	0.93	-	0	ID=MALK_01430.t1.c1;Parent=MALK_01430.t1
contig004	AUGUSTUS	mRNA	362986	363534	0.93	-	.	ID=MALK_01430.t1;Parent=MALK_01430
contig004	AUGUSTUS	exon	362986	363534	0.93	-	.	ID=MALK_01430.t1.e1;Parent=MALK_01430.t1
contig004	maker	gene	363681	368021	.	+	.	ID=MALK_01431;prediction_source=maker_MRET:augustus_masked-contig004-processed-gene-3.58-mRNA-1
contig004	maker	CDS	363681	368021	.	+	0	ID=MALK_01431.t1.c1;Parent=MALK_01431.t1
contig004	maker	mRNA	363681	368021	.	+	.	ID=MALK_01431.t1;Parent=MALK_01431
contig004	maker	exon	363681	368021	.	+	.	ID=MALK_01431.t1.e1;Parent=MALK_01431.t1

contig004	AUGUSTUS	gene	368534	368809	0.46	-	.	ID=MALK_01432;prediction_source=augustus:contig004.g225.t1
contig004	AUGUSTUS	CDS	368534	368809	0.46	-	0	ID=MALK_01432.t1.c1;Parent=MALK_01432.t1
contig004	AUGUSTUS	mRNA	368534	368809	0.46	-	.	ID=MALK_01432.t1;Parent=MALK_01432
contig004	AUGUSTUS	exon	368534	368809	0.46	-	.	ID=MALK_01432.t1.e1;Parent=MALK_01432.t1
contig004	AUGUSTUS	gene	369121	373008	0.89	+	.	ID=MALK_01433;prediction_source=augustus:contig004.g226.t1
contig004	AUGUSTUS	CDS	369121	373008	0.89	+	0	ID=MALK_01433.t1.c1;Parent=MALK_01433.t1
contig004	AUGUSTUS	mRNA	369121	373008	0.89	+	.	ID=MALK_01433.t1;Parent=MALK_01433
contig004	AUGUSTUS	exon	369121	373008	0.89	+	.	ID=MALK_01433.t1.e1;Parent=MALK_01433.t1
contig004	maker	gene	373038	375365	.	-	.	ID=MALK_01434;prediction_source=maker_MRET:augustus_masked-contig004-processed-gene-3.79-mRNA-1
contig004	maker	CDS	373038	375365	.	-	0	ID=MALK_01434.t1.c1;Parent=MALK_01434.t1
contig004	maker	mRNA	373038	375365	.	-	.	ID=MALK_01434.t1;Parent=MALK_01434
contig004	maker	exon	373038	375365	.	-	.	ID=MALK_01434.t1.e1;Parent=MALK_01434.t1
contig004	maker	gene	375538	376794	.	+	.	ID=MALK_01435;prediction_source=maker_MRET:augustus_masked-contig004-processed-gene-3.60-mRNA-1
contig004	maker	CDS	375538	376794	.	+	0	ID=MALK_01435.t1.c1;Parent=MALK_01435.t1
contig004	maker	mRNA	375538	376794	.	+	.	ID=MALK_01435.t1;Parent=MALK_01435
contig004	maker	exon	375538	376794	.	+	.	ID=MALK_01435.t1.e1;Parent=MALK_01435.t1
contig004	maker	gene	376857	378650	.	-	.	ID=MALK_01436;prediction_source=maker_MRET:augustus_masked-contig004-processed-gene-3.80-mRNA-1
contig004	maker	CDS	376857	378650	.	-	0	ID=MALK_01436.t1.c1;Parent=MALK_01436.t1
contig004	maker	mRNA	376857	378650	.	-	.	ID=MALK_01436.t1;Parent=MALK_01436
contig004	maker	exon	376857	378650	.	-	.	ID=MALK_01436.t1.e1;Parent=MALK_01436.t1
contig004	AUGUSTUS	gene	379647	380846	1	-	.	ID=MALK_01437;prediction_source=augustus:contig004.g232.t1
contig004	AUGUSTUS	CDS	379647	380846	1	-	0	ID=MALK_01437.t1.c1;Parent=MALK_01437.t1
contig004	AUGUSTUS	mRNA	379647	380846	1	-	.	ID=MALK_01437.t1;Parent=MALK_01437
contig004	AUGUSTUS	exon	379647	380846	1	-	.	ID=MALK_01437.t1.e1;Parent=MALK_01437.t1
contig004	AUGUSTUS	gene	381063	383495	0.97	-	.	ID=MALK_01438;prediction_source=augustus:contig004.g233.t1
contig004	AUGUSTUS	CDS	381063	383495	0.97	-	0	ID=MALK_01438.t1.c1;Parent=MALK_01438.t1
contig004	AUGUSTUS	mRNA	381063	383495	0.97	-	.	ID=MALK_01438.t1;Parent=MALK_01438
contig004	AUGUSTUS	exon	381063	383495	0.97	-	.	ID=MALK_01438.t1.e1;Parent=MALK_01438.t1
contig004	maker	gene	383764	384834	.	-	.	ID=MALK_01439;prediction_source=maker_MRET:augustus_masked-contig004-processed-gene-3.83-mRNA-1
contig004	maker	CDS	383764	384834	.	-	0	ID=MALK_01439.t1.c1;Parent=MALK_01439.t1
contig004	maker	mRNA	383764	384834	.	-	.	ID=MALK_01439.t1;Parent=MALK_01439
contig004	maker	exon	383764	384834	.	-	.	ID=MALK_01439.t1.e1;Parent=MALK_01439.t1
contig004	AUGUSTUS	gene	385011	386005	0.78	+	.	ID=MALK_01440;prediction_source=braker_MRET:g602.t1
contig004	AUGUSTUS	CDS	385011	385063	1	+	0	ID=MALK_01440.t1.c1;Parent=MALK_01440.t1
contig004	AUGUSTUS	CDS	385113	385309	1	+	0	ID=MALK_01440.t1.c2;Parent=MALK_01440.t1
contig004	AUGUSTUS	CDS	385362	385945	1	+	0	ID=MALK_01440.t1.c3;Parent=MALK_01440.t1
contig004	AUGUSTUS	CDS	385991	386005	1	+	0	ID=MALK_01440.t1.c4;Parent=MALK_01440.t1
contig004	AUGUSTUS	mRNA	385011	386005	0.78	+	.	ID=MALK_01440.t1;Parent=MALK_01440
contig004	AUGUSTUS	exon	385011	385063	.	+	.	ID=MALK_01440.t1.e1;Parent=MALK_01440.t1
contig004	AUGUSTUS	exon	385113	385309	.	+	.	ID=MALK_01440.t1.e2;Parent=MALK_01440.t1
contig004	AUGUSTUS	exon	385362	385945	.	+	.	ID=MALK_01440.t1.e3;Parent=MALK_01440.t1
contig004	AUGUSTUS	exon	385991	386005	.	+	.	ID=MALK_01440.t1.e4;Parent=MALK_01440.t1
contig004	AUGUSTUS	gene	386244	388068	0.44	-	.	ID=MALK_01441;prediction_source=braker_MRET:g603.t1

contig004	AUGUSTUS	CDS	388048	388068	0.53	-	0	ID=MALK_01441.t1.c3;Parent=MALK_01441.t1
contig004	AUGUSTUS	CDS	387768	388016	0.53	-	0	ID=MALK_01441.t1.c2;Parent=MALK_01441.t1
contig004	AUGUSTUS	CDS	386244	387728	0.53	-	0	ID=MALK_01441.t1.c1;Parent=MALK_01441.t1
contig004	AUGUSTUS	mRNA	386244	388068	0.44	-	.	ID=MALK_01441.t1;Parent=MALK_01441
contig004	AUGUSTUS	exon	388048	388068	.	-	.	ID=MALK_01441.t1.e3;Parent=MALK_01441.t1
contig004	AUGUSTUS	exon	387768	388016	.	-	.	ID=MALK_01441.t1.e2;Parent=MALK_01441.t1
contig004	AUGUSTUS	exon	386244	387728	.	-	.	ID=MALK_01441.t1.e1;Parent=MALK_01441.t1
contig004	AUGUSTUS	gene	389184	392716	0.93	+	.	ID=MALK_01442;prediction_source=braker_MRET:g604.t1
contig004	AUGUSTUS	CDS	389184	389199	0.93	+	0	ID=MALK_01442.t1.c1;Parent=MALK_01442.t1
contig004	AUGUSTUS	CDS	389244	392716	0.93	+	0	ID=MALK_01442.t1.c2;Parent=MALK_01442.t1
contig004	AUGUSTUS	mRNA	389184	392716	0.93	+	.	ID=MALK_01442.t1;Parent=MALK_01442
contig004	AUGUSTUS	exon	389184	389199	.	+	.	ID=MALK_01442.t1.e1;Parent=MALK_01442.t1
contig004	AUGUSTUS	exon	389244	392716	.	+	.	ID=MALK_01442.t1.e2;Parent=MALK_01442.t1
contig004	AUGUSTUS	gene	392870	395753	0.72	+	.	ID=MALK_01443;prediction_source=augustus:contig004.g241.t1
contig004	AUGUSTUS	CDS	392870	393089	0.78	+	0	ID=MALK_01443.t1.c1;Parent=MALK_01443.t1
contig004	AUGUSTUS	CDS	393175	395420	0.78	+	0	ID=MALK_01443.t1.c2;Parent=MALK_01443.t1
contig004	AUGUSTUS	CDS	395517	395753	0.78	+	0	ID=MALK_01443.t1.c3;Parent=MALK_01443.t1
contig004	AUGUSTUS	mRNA	392870	395753	0.72	+	.	ID=MALK_01443.t1;Parent=MALK_01443
contig004	AUGUSTUS	exon	392870	393089	0.78	+	.	ID=MALK_01443.t1.e1;Parent=MALK_01443.t1
contig004	AUGUSTUS	exon	393175	395420	0.78	+	.	ID=MALK_01443.t1.e2;Parent=MALK_01443.t1
contig004	AUGUSTUS	exon	395517	395753	0.78	+	.	ID=MALK_01443.t1.e3;Parent=MALK_01443.t1
contig004	maker	gene	395907	397193	.	-	.	ID=MALK_01444;prediction_source=maker_MRET:augustus_masked-contig004-processed-gene-4.2-mRNA-1
contig004	maker	CDS	395907	397193	.	-	0	ID=MALK_01444.t1.c1;Parent=MALK_01444.t1
contig004	maker	mRNA	395907	397193	.	-	.	ID=MALK_01444.t1;Parent=MALK_01444
contig004	maker	exon	395907	397193	.	-	.	ID=MALK_01444.t1.e1;Parent=MALK_01444.t1
contig004	AUGUSTUS	gene	397469	403936	0.99	-	.	ID=MALK_01445;prediction_source=augustus:contig004.g243.t1
contig004	AUGUSTUS	CDS	397469	403936	0.99	-	0	ID=MALK_01445.t1.c1;Parent=MALK_01445.t1
contig004	AUGUSTUS	mRNA	397469	403936	0.99	-	.	ID=MALK_01445.t1;Parent=MALK_01445
contig004	AUGUSTUS	exon	397469	403936	0.99	-	.	ID=MALK_01445.t1.e1;Parent=MALK_01445.t1
contig004	maker	gene	404474	406199	.	+	.	ID=MALK_01446;prediction_source=maker_MRET:augustus_masked-contig004-processed-gene-4.4-mRNA-1
contig004	maker	CDS	404474	404911	.	+	0	ID=MALK_01446.t1.c1;Parent=MALK_01446.t1
contig004	maker	CDS	404973	406199	.	+	0	ID=MALK_01446.t1.c2;Parent=MALK_01446.t1
contig004	maker	mRNA	404474	406199	.	+	.	ID=MALK_01446.t1;Parent=MALK_01446
contig004	maker	exon	404474	404911	.	+	.	ID=MALK_01446.t1.e1;Parent=MALK_01446.t1
contig004	maker	exon	404973	406199	.	+	.	ID=MALK_01446.t1.e2;Parent=MALK_01446.t1
contig004	maker	gene	406235	407566	.	-	.	ID=MALK_01447;prediction_source=maker_MRET:augustus_masked-contig004-processed-gene-4.37-mRNA-1
contig004	maker	CDS	406235	407566	.	-	0	ID=MALK_01447.t1.c1;Parent=MALK_01447.t1
contig004	maker	mRNA	406235	407566	.	-	.	ID=MALK_01447.t1;Parent=MALK_01447
contig004	maker	exon	406235	407566	.	-	.	ID=MALK_01447.t1.e1;Parent=MALK_01447.t1
contig004	AUGUSTUS	gene	407996	408722	0.46	-	.	ID=MALK_01448;prediction_source=braker_MRET:g610.t1
contig004	AUGUSTUS	CDS	408638	408722	0.57	-	0	ID=MALK_01448.t1.c5;Parent=MALK_01448.t1
contig004	AUGUSTUS	CDS	408531	408599	0.57	-	0	ID=MALK_01448.t1.c4;Parent=MALK_01448.t1
contig004	AUGUSTUS	CDS	408179	408490	0.57	-	0	ID=MALK_01448.t1.c3;Parent=MALK_01448.t1

contig004	AUGUSTUS	CDS	408125	408136	0.57	-	0	ID=MALK_01448.t1.c2;Parent=MALK_01448.t1
contig004	AUGUSTUS	CDS	407996	408042	0.57	-	0	ID=MALK_01448.t1.c1;Parent=MALK_01448.t1
contig004	AUGUSTUS	mRNA	407996	408722	0.46	-	.	ID=MALK_01448.t1;Parent=MALK_01448
contig004	AUGUSTUS	exon	408638	408722	.	-	.	ID=MALK_01448.t1.e5;Parent=MALK_01448.t1
contig004	AUGUSTUS	exon	408531	408599	.	-	.	ID=MALK_01448.t1.e4;Parent=MALK_01448.t1
contig004	AUGUSTUS	exon	408179	408490	.	-	.	ID=MALK_01448.t1.e3;Parent=MALK_01448.t1
contig004	AUGUSTUS	exon	408125	408136	.	-	.	ID=MALK_01448.t1.e2;Parent=MALK_01448.t1
contig004	AUGUSTUS	exon	407996	408042	.	-	.	ID=MALK_01448.t1.e1;Parent=MALK_01448.t1
contig004	AUGUSTUS	gene	409276	409668	0.49	-	.	ID=MALK_01449;prediction_source=braker_MRET:g611.t1
contig004	AUGUSTUS	CDS	409640	409668	0.98	-	0	ID=MALK_01449.t1.c4;Parent=MALK_01449.t1
contig004	AUGUSTUS	CDS	409580	409601	0.98	-	0	ID=MALK_01449.t1.c3;Parent=MALK_01449.t1
contig004	AUGUSTUS	CDS	409486	409511	0.98	-	0	ID=MALK_01449.t1.c2;Parent=MALK_01449.t1
contig004	AUGUSTUS	CDS	409276	409453	0.98	-	0	ID=MALK_01449.t1.c1;Parent=MALK_01449.t1
contig004	AUGUSTUS	mRNA	409276	409668	0.49	-	.	ID=MALK_01449.t1;Parent=MALK_01449
contig004	AUGUSTUS	exon	409640	409668	.	-	.	ID=MALK_01449.t1.e4;Parent=MALK_01449.t1
contig004	AUGUSTUS	exon	409580	409601	.	-	.	ID=MALK_01449.t1.e3;Parent=MALK_01449.t1
contig004	AUGUSTUS	exon	409486	409511	.	-	.	ID=MALK_01449.t1.e2;Parent=MALK_01449.t1
contig004	AUGUSTUS	exon	409276	409453	.	-	.	ID=MALK_01449.t1.e1;Parent=MALK_01449.t1
contig004	maker	gene	409833	411234	.	-	.	ID=MALK_01450;prediction_source=maker_MRET:augustus_masked-contig004-processed-gene-4.39-mRNA-1
contig004	maker	CDS	411223	411234	.	-	0	ID=MALK_01450.t1.c1;Parent=MALK_01450.t1
contig004	maker	CDS	409833	410921	.	-	0	ID=MALK_01450.t1.c2;Parent=MALK_01450.t1
contig004	maker	mRNA	409833	411234	.	-	.	ID=MALK_01450.t1;Parent=MALK_01450
contig004	maker	exon	411223	411234	.	-	.	ID=MALK_01450.t1.e1;Parent=MALK_01450.t1
contig004	maker	exon	409833	410921	.	-	.	ID=MALK_01450.t1.e2;Parent=MALK_01450.t1
contig004	maker	gene	411506	412264	.	+	.	ID=MALK_01451;prediction_source=maker_MRET:augustus_masked-contig004-processed-gene-4.5-mRNA-1
contig004	maker	CDS	411506	412264	.	+	0	ID=MALK_01451.t1.c1;Parent=MALK_01451.t1
contig004	maker	mRNA	411506	412264	.	+	.	ID=MALK_01451.t1;Parent=MALK_01451
contig004	maker	exon	411506	412264	.	+	.	ID=MALK_01451.t1.e1;Parent=MALK_01451.t1
contig004	AUGUSTUS	gene	412296	413042	0.26	+	.	ID=MALK_01452;prediction_source=braker_MRET:g614.t1
contig004	AUGUSTUS	CDS	412296	413042	0.26	+	0	ID=MALK_01452.t1.c1;Parent=MALK_01452.t1
contig004	AUGUSTUS	mRNA	412296	413042	0.26	+	.	ID=MALK_01452.t1;Parent=MALK_01452
contig004	AUGUSTUS	exon	412296	413042	.	+	.	ID=MALK_01452.t1.e1;Parent=MALK_01452.t1
contig004	AUGUSTUS	gene	413046	414392	1	-	.	ID=MALK_01453;prediction_source=braker_MRET:g615.t1
contig004	AUGUSTUS	CDS	413046	414392	1	-	0	ID=MALK_01453.t1.c1;Parent=MALK_01453.t1
contig004	AUGUSTUS	mRNA	413046	414392	1	-	.	ID=MALK_01453.t1;Parent=MALK_01453
contig004	AUGUSTUS	exon	413046	414392	.	-	.	ID=MALK_01453.t1.e1;Parent=MALK_01453.t1
contig004	AUGUSTUS	gene	414391	415818	0.31	+	.	ID=MALK_01454;prediction_source=augustus:contig004.g253.t1
contig004	AUGUSTUS	CDS	414391	415818	0.31	+	0	ID=MALK_01454.t1.c1;Parent=MALK_01454.t1
contig004	AUGUSTUS	mRNA	414391	415818	0.31	+	.	ID=MALK_01454.t1;Parent=MALK_01454
contig004	AUGUSTUS	exon	414391	415818	0.31	+	.	ID=MALK_01454.t1.e1;Parent=MALK_01454.t1
contig004	AUGUSTUS	gene	415845	417698	0.74	+	.	ID=MALK_01455;prediction_source=braker_MRET:g617.t1
contig004	AUGUSTUS	CDS	415845	417698	0.74	+	0	ID=MALK_01455.t1.c1;Parent=MALK_01455.t1
contig004	AUGUSTUS	mRNA	415845	417698	0.74	+	.	ID=MALK_01455.t1;Parent=MALK_01455

contig004	AUGUSTUS	exon	415845	417698	.	+	.	ID=MALK_01455.t1.e1;Parent=MALK_01455.t1
contig004	AUGUSTUS	gene	417713	417967	0.82	-	.	ID=MALK_01456;prediction_source=braker_MRET:g618.t1
contig004	AUGUSTUS	CDS	417713	417967	0.82	-	0	ID=MALK_01456.t1.c1;Parent=MALK_01456.t1
contig004	AUGUSTUS	mRNA	417713	417967	0.82	-	.	ID=MALK_01456.t1;Parent=MALK_01456
contig004	AUGUSTUS	exon	417713	417967	.	-	.	ID=MALK_01456.t1.e1;Parent=MALK_01456.t1
contig004	AUGUSTUS	gene	418187	419515	0.46	-	.	ID=MALK_01457;prediction_source=braker_MRET:g619.t1
contig004	AUGUSTUS	CDS	418187	419515	0.46	-	0	ID=MALK_01457.t1.c1;Parent=MALK_01457.t1
contig004	AUGUSTUS	mRNA	418187	419515	0.46	-	.	ID=MALK_01457.t1;Parent=MALK_01457
contig004	AUGUSTUS	exon	418187	419515	.	-	.	ID=MALK_01457.t1.e1;Parent=MALK_01457.t1
contig004	AUGUSTUS	gene	419586	420992	1	+	.	ID=MALK_01458;prediction_source=braker_MRET:g620.t1
contig004	AUGUSTUS	CDS	419586	420992	1	+	0	ID=MALK_01458.t1.c1;Parent=MALK_01458.t1
contig004	AUGUSTUS	mRNA	419586	420992	1	+	.	ID=MALK_01458.t1;Parent=MALK_01458
contig004	AUGUSTUS	exon	419586	420992	.	+	.	ID=MALK_01458.t1.e1;Parent=MALK_01458.t1
contig004	AUGUSTUS	gene	420999	422542	0.85	-	.	ID=MALK_01459;prediction_source=braker_MRET:g621.t1
contig004	AUGUSTUS	CDS	421138	422542	0.95	-	0	ID=MALK_01459.t1.c2;Parent=MALK_01459.t1
contig004	AUGUSTUS	CDS	420999	421102	0.95	-	0	ID=MALK_01459.t1.c1;Parent=MALK_01459.t1
contig004	AUGUSTUS	mRNA	420999	422542	0.85	-	.	ID=MALK_01459.t1;Parent=MALK_01459
contig004	AUGUSTUS	exon	421138	422542	.	-	.	ID=MALK_01459.t1.e2;Parent=MALK_01459.t1
contig004	AUGUSTUS	exon	420999	421102	.	-	.	ID=MALK_01459.t1.e1;Parent=MALK_01459.t1
contig004	maker	gene	422590	424158	.	-	.	ID=MALK_01460;prediction_source=maker_MRET:augustus_masked-contig004-processed-gene-4.42-mRNA-1
contig004	maker	CDS	422590	424158	.	-	0	ID=MALK_01460.t1.c1;Parent=MALK_01460.t1
contig004	maker	mRNA	422590	424158	.	-	.	ID=MALK_01460.t1;Parent=MALK_01460
contig004	maker	exon	422590	424158	.	-	.	ID=MALK_01460.t1.e1;Parent=MALK_01460.t1
contig004	AUGUSTUS	gene	424181	424957	0.78	+	.	ID=MALK_01461;prediction_source=braker_MRET:g623.t1
contig004	AUGUSTUS	CDS	424181	424957	0.78	+	0	ID=MALK_01461.t1.c1;Parent=MALK_01461.t1
contig004	AUGUSTUS	mRNA	424181	424957	0.78	+	.	ID=MALK_01461.t1;Parent=MALK_01461
contig004	AUGUSTUS	exon	424181	424957	.	+	.	ID=MALK_01461.t1.e1;Parent=MALK_01461.t1
contig004	maker	gene	425055	426491	.	+	.	ID=MALK_01462;prediction_source=maker_MRET:augustus_masked-contig004-processed-gene-4.10-mRNA-1
contig004	maker	CDS	425055	426491	.	+	0	ID=MALK_01462.t1.c1;Parent=MALK_01462.t1
contig004	maker	mRNA	425055	426491	.	+	.	ID=MALK_01462.t1;Parent=MALK_01462
contig004	maker	exon	425055	426491	.	+	.	ID=MALK_01462.t1.e1;Parent=MALK_01462.t1
contig004	AUGUSTUS	gene	426488	427495	0.9	-	.	ID=MALK_01463;prediction_source=braker_MRET:g625.t1
contig004	AUGUSTUS	CDS	426488	427495	0.9	-	0	ID=MALK_01463.t1.c1;Parent=MALK_01463.t1
contig004	AUGUSTUS	mRNA	426488	427495	0.9	-	.	ID=MALK_01463.t1;Parent=MALK_01463
contig004	AUGUSTUS	exon	426488	427495	.	-	.	ID=MALK_01463.t1.e1;Parent=MALK_01463.t1
contig004	maker	gene	427607	428188	.	+	.	ID=MALK_01464;prediction_source=maker_MRET:augustus_masked-contig004-processed-gene-4.11-mRNA-1
contig004	maker	CDS	427607	428188	.	+	0	ID=MALK_01464.t1.c1;Parent=MALK_01464.t1
contig004	maker	mRNA	427607	428188	.	+	.	ID=MALK_01464.t1;Parent=MALK_01464
contig004	maker	exon	427607	428188	.	+	.	ID=MALK_01464.t1.e1;Parent=MALK_01464.t1
contig004	maker	gene	428603	431251	.	+	.	ID=MALK_01465;prediction_source=maker_MRET:augustus_masked-contig004-processed-gene-4.12-mRNA-1
contig004	maker	CDS	428603	431251	.	+	0	ID=MALK_01465.t1.c1;Parent=MALK_01465.t1
contig004	maker	mRNA	428603	431251	.	+	.	ID=MALK_01465.t1;Parent=MALK_01465
contig004	maker	exon	428603	431251	.	+	.	ID=MALK_01465.t1.e1;Parent=MALK_01465.t1

contig004	AUGUSTUS	gene	431317	433356	1	-	.	ID=MALK_01466;prediction_source=augustus:contig004.g264.t1
contig004	AUGUSTUS	CDS	431317	433356	1	-	0	ID=MALK_01466.t1.c1;Parent=MALK_01466.t1
contig004	AUGUSTUS	mRNA	431317	433356	1	-	.	ID=MALK_01466.t1;Parent=MALK_01466
contig004	AUGUSTUS	exon	431317	433356	1	-	.	ID=MALK_01466.t1.e1;Parent=MALK_01466.t1
contig004	maker	gene	434697	435338	.	+	.	ID=MALK_01467;prediction_source=maker_MRET:augustus_masked-contig004-processed-gene-4.13-mRNA-1
contig004	maker	CDS	434697	435338	.	+	0	ID=MALK_01467.t1.c1;Parent=MALK_01467.t1
contig004	maker	mRNA	434697	435338	.	+	.	ID=MALK_01467.t1;Parent=MALK_01467
contig004	maker	exon	434697	435338	.	+	.	ID=MALK_01467.t1.e1;Parent=MALK_01467.t1
contig004	AUGUSTUS	gene	435361	436164	0.75	-	.	ID=MALK_01468;prediction_source=augustus:contig004.g266.t1
contig004	AUGUSTUS	CDS	435361	436164	0.75	-	0	ID=MALK_01468.t1.c1;Parent=MALK_01468.t1
contig004	AUGUSTUS	mRNA	435361	436164	0.75	-	.	ID=MALK_01468.t1;Parent=MALK_01468
contig004	AUGUSTUS	exon	435361	436164	0.75	-	.	ID=MALK_01468.t1.e1;Parent=MALK_01468.t1
contig004	AUGUSTUS	gene	436252	438744	0.95	+	.	ID=MALK_01469;prediction_source=augustus:contig004.g267.t1
contig004	AUGUSTUS	CDS	436252	438744	0.95	+	0	ID=MALK_01469.t1.c1;Parent=MALK_01469.t1
contig004	AUGUSTUS	mRNA	436252	438744	0.95	+	.	ID=MALK_01469.t1;Parent=MALK_01469
contig004	AUGUSTUS	exon	436252	438744	0.95	+	.	ID=MALK_01469.t1.e1;Parent=MALK_01469.t1
contig004	AUGUSTUS	gene	438755	439761	0.46	-	.	ID=MALK_01470;prediction_source=braker_MRET:g632.t1
contig004	AUGUSTUS	CDS	439539	439761	0.65	-	0	ID=MALK_01470.t1.c2;Parent=MALK_01470.t1
contig004	AUGUSTUS	CDS	438755	439497	0.65	-	0	ID=MALK_01470.t1.c1;Parent=MALK_01470.t1
contig004	AUGUSTUS	mRNA	438755	439761	0.46	-	.	ID=MALK_01470.t1;Parent=MALK_01470
contig004	AUGUSTUS	exon	439539	439761	.	-	.	ID=MALK_01470.t1.e2;Parent=MALK_01470.t1
contig004	AUGUSTUS	exon	438755	439497	.	-	.	ID=MALK_01470.t1.e1;Parent=MALK_01470.t1
contig004	AUGUSTUS	gene	439918	442266	0.81	-	.	ID=MALK_01471;prediction_source=braker_MRET:g633.t1
contig004	AUGUSTUS	CDS	439918	442266	0.81	-	0	ID=MALK_01471.t1.c1;Parent=MALK_01471.t1
contig004	AUGUSTUS	mRNA	439918	442266	0.81	-	.	ID=MALK_01471.t1;Parent=MALK_01471
contig004	AUGUSTUS	exon	439918	442266	.	-	.	ID=MALK_01471.t1.e1;Parent=MALK_01471.t1
contig004	maker	gene	442372	444141	.	+	.	ID=MALK_01472;prediction_source=maker_MRET:augustus_masked-contig004-processed-gene-4.15-mRNA-1
contig004	maker	CDS	442372	444141	.	+	0	ID=MALK_01472.t1.c1;Parent=MALK_01472.t1
contig004	maker	mRNA	442372	444141	.	+	.	ID=MALK_01472.t1;Parent=MALK_01472
contig004	maker	exon	442372	444141	.	+	.	ID=MALK_01472.t1.e1;Parent=MALK_01472.t1
contig004	maker	gene	444177	444725	.	-	.	ID=MALK_01473;prediction_source=maker_MRET:augustus_masked-contig004-processed-gene-4.46-mRNA-1
contig004	maker	CDS	444177	444725	.	-	0	ID=MALK_01473.t1.c1;Parent=MALK_01473.t1
contig004	maker	mRNA	444177	444725	.	-	.	ID=MALK_01473.t1;Parent=MALK_01473
contig004	maker	exon	444177	444725	.	-	.	ID=MALK_01473.t1.e1;Parent=MALK_01473.t1
contig004	AUGUSTUS	gene	444957	448046	0.47	+	.	ID=MALK_01474;prediction_source=augustus:contig004.g271.t1
contig004	AUGUSTUS	CDS	444957	448046	0.47	+	0	ID=MALK_01474.t1.c1;Parent=MALK_01474.t1
contig004	AUGUSTUS	mRNA	444957	448046	0.47	+	.	ID=MALK_01474.t1;Parent=MALK_01474
contig004	AUGUSTUS	exon	444957	448046	0.47	+	.	ID=MALK_01474.t1.e1;Parent=MALK_01474.t1
contig004	AUGUSTUS	gene	448060	451238	0.47	-	.	ID=MALK_01475;prediction_source=augustus:contig004.g273.t1
contig004	AUGUSTUS	CDS	451167	451238	0.62	-	0	ID=MALK_01475.t1.c2;Parent=MALK_01475.t1
contig004	AUGUSTUS	CDS	448060	450987	0.62	-	0	ID=MALK_01475.t1.c1;Parent=MALK_01475.t1
contig004	AUGUSTUS	mRNA	448060	451238	0.47	-	.	ID=MALK_01475.t1;Parent=MALK_01475
contig004	AUGUSTUS	exon	451167	451238	0.62	-	.	ID=MALK_01475.t1.e2;Parent=MALK_01475.t1

contig004	AUGUSTUS	exon	448060	450987	0.62	-	.	ID=MALK_01475.t1.e1;Parent=MALK_01475.t1
contig004	maker	gene	451206	452564	.	+	.	ID=MALK_01476;prediction_source=maker_MRET:augustus_masked-contig004-processed-gene-4.17-mRNA-1
contig004	maker	CDS	451206	452564	.	+	0	ID=MALK_01476.t1.c1;Parent=MALK_01476.t1
contig004	maker	mRNA	451206	452564	.	+	.	ID=MALK_01476.t1;Parent=MALK_01476
contig004	maker	exon	451206	452564	.	+	.	ID=MALK_01476.t1.e1;Parent=MALK_01476.t1
contig004	AUGUSTUS	gene	452561	453217	0.98	-	.	ID=MALK_01477;prediction_source=augustus:contig004.g275.t1
contig004	AUGUSTUS	CDS	452561	453217	0.98	-	0	ID=MALK_01477.t1.c1;Parent=MALK_01477.t1
contig004	AUGUSTUS	mRNA	452561	453217	0.98	-	.	ID=MALK_01477.t1;Parent=MALK_01477
contig004	AUGUSTUS	exon	452561	453217	0.98	-	.	ID=MALK_01477.t1.e1;Parent=MALK_01477.t1
contig004	maker	gene	453499	454773	.	+	.	ID=MALK_01478;prediction_source=maker_MRET:augustus_masked-contig004-processed-gene-4.18-mRNA-1
contig004	maker	CDS	453499	454773	.	+	0	ID=MALK_01478.t1.c1;Parent=MALK_01478.t1
contig004	maker	mRNA	453499	454773	.	+	.	ID=MALK_01478.t1;Parent=MALK_01478
contig004	maker	exon	453499	454773	.	+	.	ID=MALK_01478.t1.e1;Parent=MALK_01478.t1
contig004	AUGUSTUS	gene	454845	455561	0.53	+	.	ID=MALK_01479;prediction_source=braker_MRET:g641.t1
contig004	AUGUSTUS	CDS	454845	455561	0.53	+	0	ID=MALK_01479.t1.c1;Parent=MALK_01479.t1
contig004	AUGUSTUS	mRNA	454845	455561	0.53	+	.	ID=MALK_01479.t1;Parent=MALK_01479
contig004	AUGUSTUS	exon	454845	455561	.	+	.	ID=MALK_01479.t1.e1;Parent=MALK_01479.t1
contig004	AUGUSTUS	gene	455566	458583	0.57	-	.	ID=MALK_01480;prediction_source=augustus:contig004.g279.t1
contig004	AUGUSTUS	CDS	455566	458583	0.57	-	0	ID=MALK_01480.t1.c1;Parent=MALK_01480.t1
contig004	AUGUSTUS	mRNA	455566	458583	0.57	-	.	ID=MALK_01480.t1;Parent=MALK_01480
contig004	AUGUSTUS	exon	455566	458583	0.57	-	.	ID=MALK_01480.t1.e1;Parent=MALK_01480.t1
contig004	AUGUSTUS	gene	459036	460028	0.94	+	.	ID=MALK_01481;prediction_source=augustus:contig004.g280.t1
contig004	AUGUSTUS	CDS	459036	460028	0.94	+	0	ID=MALK_01481.t1.c1;Parent=MALK_01481.t1
contig004	AUGUSTUS	mRNA	459036	460028	0.94	+	.	ID=MALK_01481.t1;Parent=MALK_01481
contig004	AUGUSTUS	exon	459036	460028	0.94	+	.	ID=MALK_01481.t1.e1;Parent=MALK_01481.t1
contig004	maker	gene	460077	460682	.	-	.	ID=MALK_01482;prediction_source=maker_MRET:augustus_masked-contig004-processed-gene-4.49-mRNA-1
contig004	maker	CDS	460077	460682	.	-	0	ID=MALK_01482.t1.c1;Parent=MALK_01482.t1
contig004	maker	mRNA	460077	460682	.	-	.	ID=MALK_01482.t1;Parent=MALK_01482
contig004	maker	exon	460077	460682	.	-	.	ID=MALK_01482.t1.e1;Parent=MALK_01482.t1
contig004	AUGUSTUS	gene	460764	462094	0.53	+	.	ID=MALK_01483;prediction_source=braker_MRET:g645.t1
contig004	AUGUSTUS	CDS	460764	461894	0.97	+	0	ID=MALK_01483.t1.c1;Parent=MALK_01483.t1
contig004	AUGUSTUS	CDS	461936	462094	0.97	+	0	ID=MALK_01483.t1.c2;Parent=MALK_01483.t1
contig004	AUGUSTUS	mRNA	460764	462094	0.53	+	.	ID=MALK_01483.t1;Parent=MALK_01483
contig004	AUGUSTUS	exon	460764	461894	.	+	.	ID=MALK_01483.t1.e1;Parent=MALK_01483.t1
contig004	AUGUSTUS	exon	461936	462094	.	+	.	ID=MALK_01483.t1.e2;Parent=MALK_01483.t1
contig004	AUGUSTUS	gene	462163	464349	0.95	+	.	ID=MALK_01484;prediction_source=augustus:contig004.g283.t1
contig004	AUGUSTUS	CDS	462163	464349	0.95	+	0	ID=MALK_01484.t1.c1;Parent=MALK_01484.t1
contig004	AUGUSTUS	mRNA	462163	464349	0.95	+	.	ID=MALK_01484.t1;Parent=MALK_01484
contig004	AUGUSTUS	exon	462163	464349	0.95	+	.	ID=MALK_01484.t1.e1;Parent=MALK_01484.t1
contig004	maker	gene	464785	469074	.	+	.	ID=MALK_01485;prediction_source=maker_MRET:augustus_masked-contig004-processed-gene-4.23-mRNA-1
contig004	maker	CDS	464785	469074	.	+	0	ID=MALK_01485.t1.c1;Parent=MALK_01485.t1
contig004	maker	mRNA	464785	469074	.	+	.	ID=MALK_01485.t1;Parent=MALK_01485
contig004	maker	exon	464785	469074	.	+	.	ID=MALK_01485.t1.e1;Parent=MALK_01485.t1

contig004	maker	gene	469441	470094	.	+	.	ID=MALK_01486;prediction_source=maker_MRET:augustus_masked-contig004-processed-gene-4.24-mRNA-1
contig004	maker	CDS	469441	470094	.	+	0	ID=MALK_01486.t1.c1;Parent=MALK_01486.t1
contig004	maker	mRNA	469441	470094	.	+	.	ID=MALK_01486.t1;Parent=MALK_01486
contig004	maker	exon	469441	470094	.	+	.	ID=MALK_01486.t1.e1;Parent=MALK_01486.t1
contig004	maker	gene	470394	471569	.	-	.	ID=MALK_01487;prediction_source=maker_MRET:augustus_masked-contig004-processed-gene-4.50-mRNA-1
contig004	maker	CDS	470394	471569	.	-	0	ID=MALK_01487.t1.c1;Parent=MALK_01487.t1
contig004	maker	mRNA	470394	471569	.	-	.	ID=MALK_01487.t1;Parent=MALK_01487
contig004	maker	exon	470394	471569	.	-	.	ID=MALK_01487.t1.e1;Parent=MALK_01487.t1
contig004	AUGUSTUS	gene	471796	474729	0.38	-	.	ID=MALK_01488;prediction_source=augustus:contig004.g287.t1
contig004	AUGUSTUS	CDS	471796	474729	0.38	-	0	ID=MALK_01488.t1.c1;Parent=MALK_01488.t1
contig004	AUGUSTUS	mRNA	471796	474729	0.38	-	.	ID=MALK_01488.t1;Parent=MALK_01488
contig004	AUGUSTUS	exon	471796	474729	0.38	-	.	ID=MALK_01488.t1.e1;Parent=MALK_01488.t1
contig004	AUGUSTUS	gene	475038	476246	1	-	.	ID=MALK_01489;prediction_source=augustus:contig004.g288.t1
contig004	AUGUSTUS	CDS	475038	476246	1	-	0	ID=MALK_01489.t1.c1;Parent=MALK_01489.t1
contig004	AUGUSTUS	mRNA	475038	476246	1	-	.	ID=MALK_01489.t1;Parent=MALK_01489
contig004	AUGUSTUS	exon	475038	476246	1	-	.	ID=MALK_01489.t1.e1;Parent=MALK_01489.t1
contig004	AUGUSTUS	gene	476605	478512	0.94	+	.	ID=MALK_01490;prediction_source=augustus:contig004.g290.t1
contig004	AUGUSTUS	CDS	476605	478512	0.94	+	0	ID=MALK_01490.t1.c1;Parent=MALK_01490.t1
contig004	AUGUSTUS	mRNA	476605	478512	0.94	+	.	ID=MALK_01490.t1;Parent=MALK_01490
contig004	AUGUSTUS	exon	476605	478512	0.94	+	.	ID=MALK_01490.t1.e1;Parent=MALK_01490.t1
contig004	AUGUSTUS	gene	478838	480502	0.87	-	.	ID=MALK_01491;prediction_source=augustus:contig004.g291.t1
contig004	AUGUSTUS	CDS	478838	480502	0.87	-	0	ID=MALK_01491.t1.c1;Parent=MALK_01491.t1
contig004	AUGUSTUS	mRNA	478838	480502	0.87	-	.	ID=MALK_01491.t1;Parent=MALK_01491
contig004	AUGUSTUS	exon	478838	480502	0.87	-	.	ID=MALK_01491.t1.e1;Parent=MALK_01491.t1
contig004	AUGUSTUS	gene	481392	482810	0.86	+	.	ID=MALK_01492;prediction_source=augustus:contig004.g292.t1
contig004	AUGUSTUS	CDS	481392	482810	0.86	+	0	ID=MALK_01492.t1.c1;Parent=MALK_01492.t1
contig004	AUGUSTUS	mRNA	481392	482810	0.86	+	.	ID=MALK_01492.t1;Parent=MALK_01492
contig004	AUGUSTUS	exon	481392	482810	0.86	+	.	ID=MALK_01492.t1.e1;Parent=MALK_01492.t1
contig004	maker	gene	483146	484753	.	-	.	ID=MALK_01493;prediction_source=maker_MRET:augustus_masked-contig004-processed-gene-4.54-mRNA-1
contig004	maker	CDS	483146	484753	.	-	0	ID=MALK_01493.t1.c1;Parent=MALK_01493.t1
contig004	maker	mRNA	483146	484753	.	-	.	ID=MALK_01493.t1;Parent=MALK_01493
contig004	maker	exon	483146	484753	.	-	.	ID=MALK_01493.t1.e1;Parent=MALK_01493.t1
contig004	maker	gene	485705	486397	.	+	.	ID=MALK_01494;prediction_source=maker_MRET:augustus_masked-contig004-processed-gene-4.27-mRNA-1
contig004	maker	CDS	485705	486397	.	+	0	ID=MALK_01494.t1.c1;Parent=MALK_01494.t1
contig004	maker	mRNA	485705	486397	.	+	.	ID=MALK_01494.t1;Parent=MALK_01494
contig004	maker	exon	485705	486397	.	+	.	ID=MALK_01494.t1.e1;Parent=MALK_01494.t1
contig004	AUGUSTUS	gene	486857	488245	0.85	+	.	ID=MALK_01495;prediction_source=braker_MRET:g656.t1
contig004	AUGUSTUS	CDS	486857	488245	0.85	+	0	ID=MALK_01495.t1.c1;Parent=MALK_01495.t1
contig004	AUGUSTUS	mRNA	486857	488245	0.85	+	.	ID=MALK_01495.t1;Parent=MALK_01495
contig004	AUGUSTUS	exon	486857	488245	.	+	.	ID=MALK_01495.t1.e1;Parent=MALK_01495.t1
contig004	AUGUSTUS	gene	488293	489082	0.93	-	.	ID=MALK_01496;prediction_source=braker_MRET:g657.t1
contig004	AUGUSTUS	CDS	489066	489082	0.94	-	0	ID=MALK_01496.t1.c2;Parent=MALK_01496.t1
contig004	AUGUSTUS	CDS	488293	489019	0.94	-	0	ID=MALK_01496.t1.c1;Parent=MALK_01496.t1

contig004	AUGUSTUS	mRNA	488293	489082	0.93	-	.	ID=MALK_01496.t1;Parent=MALK_01496
contig004	AUGUSTUS	exon	489066	489082	.	-	.	ID=MALK_01496.t1.e2;Parent=MALK_01496.t1
contig004	AUGUSTUS	exon	488293	489019	.	-	.	ID=MALK_01496.t1.e1;Parent=MALK_01496.t1
contig004	maker	gene	489467	492206	.	+	.	ID=MALK_01497;prediction_source=maker_MRET:augustus_masked-contig004-processed-gene-4.29-mRNA-1
contig004	maker	CDS	489467	492024	.	+	0	ID=MALK_01497.t1.c1;Parent=MALK_01497.t1
contig004	maker	CDS	492122	492206	.	+	0	ID=MALK_01497.t1.c2;Parent=MALK_01497.t1
contig004	maker	mRNA	489467	492206	.	+	.	ID=MALK_01497.t1;Parent=MALK_01497
contig004	maker	exon	489467	492024	.	+	.	ID=MALK_01497.t1.e1;Parent=MALK_01497.t1
contig004	maker	exon	492122	492206	.	+	.	ID=MALK_01497.t1.e2;Parent=MALK_01497.t1
contig004	maker	gene	492211	493389	.	-	.	ID=MALK_01498;prediction_source=maker_MRET:augustus_masked-contig004-processed-gene-4.56-mRNA-1
contig004	maker	CDS	492211	493389	.	-	0	ID=MALK_01498.t1.c1;Parent=MALK_01498.t1
contig004	maker	mRNA	492211	493389	.	-	.	ID=MALK_01498.t1;Parent=MALK_01498
contig004	maker	exon	492211	493389	.	-	.	ID=MALK_01498.t1.e1;Parent=MALK_01498.t1
contig004	maker	gene	493541	495607	.	+	.	ID=MALK_01499;prediction_source=maker_MRET:augustus_masked-contig004-processed-gene-4.30-mRNA-1
contig004	maker	CDS	493541	495607	.	+	0	ID=MALK_01499.t1.c1;Parent=MALK_01499.t1
contig004	maker	mRNA	493541	495607	.	+	.	ID=MALK_01499.t1;Parent=MALK_01499
contig004	maker	exon	493541	495607	.	+	.	ID=MALK_01499.t1.e1;Parent=MALK_01499.t1
contig004	AUGUSTUS	gene	495572	496882	0.99	-	.	ID=MALK_01500;prediction_source=augustus:contig004.g300.t1
contig004	AUGUSTUS	CDS	495572	496882	0.99	-	0	ID=MALK_01500.t1.c1;Parent=MALK_01500.t1
contig004	AUGUSTUS	mRNA	495572	496882	0.99	-	.	ID=MALK_01500.t1;Parent=MALK_01500
contig004	AUGUSTUS	exon	495572	496882	0.99	-	.	ID=MALK_01500.t1.e1;Parent=MALK_01500.t1
contig004	AUGUSTUS	gene	497009	498055	0.6	+	.	ID=MALK_01501;prediction_source=augustus:contig004.g301.t1
contig004	AUGUSTUS	CDS	497009	498055	0.6	+	0	ID=MALK_01501.t1.c1;Parent=MALK_01501.t1
contig004	AUGUSTUS	mRNA	497009	498055	0.6	+	.	ID=MALK_01501.t1;Parent=MALK_01501
contig004	AUGUSTUS	exon	497009	498055	0.6	+	.	ID=MALK_01501.t1.e1;Parent=MALK_01501.t1
contig004	AUGUSTUS	gene	498079	500593	0.76	-	.	ID=MALK_01502;prediction_source=braker_MRET:g663.t1
contig004	AUGUSTUS	CDS	500199	500593	0.76	-	0	ID=MALK_01502.t1.c2;Parent=MALK_01502.t1
contig004	AUGUSTUS	CDS	498079	500167	0.76	-	0	ID=MALK_01502.t1.c1;Parent=MALK_01502.t1
contig004	AUGUSTUS	mRNA	498079	500593	0.76	-	.	ID=MALK_01502.t1;Parent=MALK_01502
contig004	AUGUSTUS	exon	500199	500593	.	-	.	ID=MALK_01502.t1.e2;Parent=MALK_01502.t1
contig004	AUGUSTUS	exon	498079	500167	.	-	.	ID=MALK_01502.t1.e1;Parent=MALK_01502.t1
contig004	AUGUSTUS	gene	500727	502649	0.98	-	.	ID=MALK_01503;prediction_source=augustus:contig004.g303.t1
contig004	AUGUSTUS	CDS	500727	502649	0.98	-	0	ID=MALK_01503.t1.c1;Parent=MALK_01503.t1
contig004	AUGUSTUS	mRNA	500727	502649	0.98	-	.	ID=MALK_01503.t1;Parent=MALK_01503
contig004	AUGUSTUS	exon	500727	502649	0.98	-	.	ID=MALK_01503.t1.e1;Parent=MALK_01503.t1
contig004	maker	gene	502785	505658	.	+	.	ID=MALK_01504;prediction_source=maker_MRET:augustus_masked-contig004-processed-gene-4.32-mRNA-1
contig004	maker	CDS	502785	505658	.	+	0	ID=MALK_01504.t1.c1;Parent=MALK_01504.t1
contig004	maker	mRNA	502785	505658	.	+	.	ID=MALK_01504.t1;Parent=MALK_01504
contig004	maker	exon	502785	505658	.	+	.	ID=MALK_01504.t1.e1;Parent=MALK_01504.t1
contig004	maker	gene	505913	506795	.	+	.	ID=MALK_01505;prediction_source=maker_MRET:augustus_masked-contig004-processed-gene-4.33-mRNA-1
contig004	maker	CDS	505913	506165	.	+	0	ID=MALK_01505.t1.c1;Parent=MALK_01505.t1
contig004	maker	CDS	506233	506795	.	+	0	ID=MALK_01505.t1.c2;Parent=MALK_01505.t1
contig004	maker	mRNA	505913	506795	.	+	.	ID=MALK_01505.t1;Parent=MALK_01505

contig004	maker	exon	505913	506165	.	+	.	ID=MALK_01505.t1.e1;Parent=MALK_01505.t1
contig004	maker	exon	506233	506795	.	+	.	ID=MALK_01505.t1.e2;Parent=MALK_01505.t1
contig004	AUGUSTUS	gene	506831	508051	0.66	-	.	ID=MALK_01506;prediction_source=augustus:contig004.g308.t1
contig004	AUGUSTUS	CDS	506831	508051	0.66	-	0	ID=MALK_01506.t1.c1;Parent=MALK_01506.t1
contig004	AUGUSTUS	mRNA	506831	508051	0.66	-	.	ID=MALK_01506.t1;Parent=MALK_01506
contig004	AUGUSTUS	exon	506831	508051	0.66	-	.	ID=MALK_01506.t1.e1;Parent=MALK_01506.t1
contig004	AUGUSTUS	gene	508453	513408	0.47	+	.	ID=MALK_01507;prediction_source=augustus:contig004.g310.t1
contig004	AUGUSTUS	CDS	508453	513408	0.47	+	0	ID=MALK_01507.t1.c1;Parent=MALK_01507.t1
contig004	AUGUSTUS	mRNA	508453	513408	0.47	+	.	ID=MALK_01507.t1;Parent=MALK_01507
contig004	AUGUSTUS	exon	508453	513408	0.47	+	.	ID=MALK_01507.t1.e1;Parent=MALK_01507.t1
contig004	AUGUSTUS	gene	514369	515629	0.51	-	.	ID=MALK_01508;prediction_source=braker_MRET:g669.t1
contig004	AUGUSTUS	CDS	514603	515629	0.51	-	0	ID=MALK_01508.t1.c2;Parent=MALK_01508.t1
contig004	AUGUSTUS	CDS	514369	514574	0.51	-	0	ID=MALK_01508.t1.c1;Parent=MALK_01508.t1
contig004	AUGUSTUS	mRNA	514369	515629	0.51	-	.	ID=MALK_01508.t1;Parent=MALK_01508
contig004	AUGUSTUS	exon	514603	515629	.	-	.	ID=MALK_01508.t1.e2;Parent=MALK_01508.t1
contig004	AUGUSTUS	exon	514369	514574	.	-	.	ID=MALK_01508.t1.e1;Parent=MALK_01508.t1
contig004	AUGUSTUS	gene	515737	517269	0.8	+	.	ID=MALK_01509;prediction_source=braker_MRET:g670.t1
contig004	AUGUSTUS	CDS	515737	516569	0.95	+	0	ID=MALK_01509.t1.c1;Parent=MALK_01509.t1
contig004	AUGUSTUS	CDS	516597	517269	0.95	+	0	ID=MALK_01509.t1.c2;Parent=MALK_01509.t1
contig004	AUGUSTUS	mRNA	515737	517269	0.8	+	.	ID=MALK_01509.t1;Parent=MALK_01509
contig004	AUGUSTUS	exon	515737	516569	.	+	.	ID=MALK_01509.t1.e1;Parent=MALK_01509.t1
contig004	AUGUSTUS	exon	516597	517269	.	+	.	ID=MALK_01509.t1.e2;Parent=MALK_01509.t1
contig004	AUGUSTUS	gene	517278	521351	0.99	-	.	ID=MALK_01510;prediction_source=augustus:contig004.g314.t1
contig004	AUGUSTUS	CDS	517278	521351	0.99	-	0	ID=MALK_01510.t1.c1;Parent=MALK_01510.t1
contig004	AUGUSTUS	mRNA	517278	521351	0.99	-	.	ID=MALK_01510.t1;Parent=MALK_01510
contig004	AUGUSTUS	exon	517278	521351	0.99	-	.	ID=MALK_01510.t1.e1;Parent=MALK_01510.t1
contig005	annotation	remark	1	298276	.	+	.	gff-version=3
contig005	AUGUSTUS	gene	682	3147	0.55	+	.	ID=MALK_01511;prediction_source=augustus:contig005.g3552.t1
contig005	AUGUSTUS	CDS	682	3147	0.55	+	0	ID=MALK_01511.t1.c1;Parent=MALK_01511.t1
contig005	AUGUSTUS	mRNA	682	3147	0.55	+	.	ID=MALK_01511.t1;Parent=MALK_01511
contig005	AUGUSTUS	exon	682	3147	0.55	+	.	ID=MALK_01511.t1.e1;Parent=MALK_01511.t1
contig005	AUGUSTUS	gene	3154	4182	0.93	-	.	ID=MALK_01512;prediction_source=braker_MRET:g2196.t1
contig005	AUGUSTUS	CDS	3154	4182	0.93	-	0	ID=MALK_01512.t1.c1;Parent=MALK_01512.t1
contig005	AUGUSTUS	mRNA	3154	4182	0.93	-	.	ID=MALK_01512.t1;Parent=MALK_01512
contig005	AUGUSTUS	exon	3154	4182	.	-	.	ID=MALK_01512.t1.e1;Parent=MALK_01512.t1
contig005	AUGUSTUS	gene	4289	5998	0.6	-	.	ID=MALK_01513;prediction_source=braker_MRET:g2197.t1
contig005	AUGUSTUS	CDS	4289	5998	0.6	-	0	ID=MALK_01513.t1.c1;Parent=MALK_01513.t1
contig005	AUGUSTUS	mRNA	4289	5998	0.6	-	.	ID=MALK_01513.t1;Parent=MALK_01513
contig005	AUGUSTUS	exon	4289	5998	.	-	.	ID=MALK_01513.t1.e1;Parent=MALK_01513.t1
contig005	maker	gene	6168	8771	.	+	.	ID=MALK_01514;prediction_source=maker_MRET:augustus_masked-contig005-processed-gene-0.1-mRNA-1
contig005	maker	CDS	6168	8771	.	+	0	ID=MALK_01514.t1.c1;Parent=MALK_01514.t1
contig005	maker	mRNA	6168	8771	.	+	.	ID=MALK_01514.t1;Parent=MALK_01514
contig005	maker	exon	6168	8771	.	+	.	ID=MALK_01514.t1.e1;Parent=MALK_01514.t1

contig005	AUGUSTUS	gene	8798	9673	0.46	-	.	ID=MALK_01515;prediction_source=augustus:contig005.g3556.t1
contig005	AUGUSTUS	CDS	8798	9673	0.46	-	0	ID=MALK_01515.t1.c1;Parent=MALK_01515.t1
contig005	AUGUSTUS	mRNA	8798	9673	0.46	-	.	ID=MALK_01515.t1;Parent=MALK_01515
contig005	AUGUSTUS	exon	8798	9673	0.46	-	.	ID=MALK_01515.t1.e1;Parent=MALK_01515.t1
contig005	AUGUSTUS	gene	9803	10711	0.36	-	.	ID=MALK_01516;prediction_source=augustus:contig005.g3558.t1
contig005	AUGUSTUS	CDS	9803	10711	0.36	-	0	ID=MALK_01516.t1.c1;Parent=MALK_01516.t1
contig005	AUGUSTUS	mRNA	9803	10711	0.36	-	.	ID=MALK_01516.t1;Parent=MALK_01516
contig005	AUGUSTUS	exon	9803	10711	0.36	-	.	ID=MALK_01516.t1.e1;Parent=MALK_01516.t1
contig005	AUGUSTUS	gene	11206	11485	0.69	+	.	ID=MALK_01517;prediction_source=braker_MRET:g2200.t1
contig005	AUGUSTUS	CDS	11206	11273	0.76	+	0	ID=MALK_01517.t1.c1;Parent=MALK_01517.t1
contig005	AUGUSTUS	CDS	11302	11485	0.76	+	0	ID=MALK_01517.t1.c2;Parent=MALK_01517.t1
contig005	AUGUSTUS	mRNA	11206	11485	0.69	+	.	ID=MALK_01517.t1;Parent=MALK_01517
contig005	AUGUSTUS	exon	11206	11273	.	+	.	ID=MALK_01517.t1.e1;Parent=MALK_01517.t1
contig005	AUGUSTUS	exon	11302	11485	.	+	.	ID=MALK_01517.t1.e2;Parent=MALK_01517.t1
contig005	maker	gene	11558	13330	.	+	.	ID=MALK_01518;prediction_source=maker_MRET:augustus_masked-contig005-processed-gene-0.2-mRNA-1
contig005	maker	CDS	11558	13330	.	+	0	ID=MALK_01518.t1.c1;Parent=MALK_01518.t1
contig005	maker	mRNA	11558	13330	.	+	.	ID=MALK_01518.t1;Parent=MALK_01518
contig005	maker	exon	11558	13330	.	+	.	ID=MALK_01518.t1.e1;Parent=MALK_01518.t1
contig005	AUGUSTUS	gene	13456	14802	1	-	.	ID=MALK_01519;prediction_source=braker_MRET:g2202.t1
contig005	AUGUSTUS	CDS	14767	14802	1	-	0	ID=MALK_01519.t1.c3;Parent=MALK_01519.t1
contig005	AUGUSTUS	CDS	14497	14665	1	-	0	ID=MALK_01519.t1.c2;Parent=MALK_01519.t1
contig005	AUGUSTUS	CDS	13456	14468	1	-	0	ID=MALK_01519.t1.c1;Parent=MALK_01519.t1
contig005	AUGUSTUS	mRNA	13456	14802	1	-	.	ID=MALK_01519.t1;Parent=MALK_01519
contig005	AUGUSTUS	exon	14767	14802	.	-	.	ID=MALK_01519.t1.e3;Parent=MALK_01519.t1
contig005	AUGUSTUS	exon	14497	14665	.	-	.	ID=MALK_01519.t1.e2;Parent=MALK_01519.t1
contig005	AUGUSTUS	exon	13456	14468	.	-	.	ID=MALK_01519.t1.e1;Parent=MALK_01519.t1
contig005	AUGUSTUS	gene	15019	16102	0.98	+	.	ID=MALK_01520;prediction_source=braker_MRET:g2203.t1
contig005	AUGUSTUS	CDS	15019	15021	0.98	+	0	ID=MALK_01520.t1.c1;Parent=MALK_01520.t1
contig005	AUGUSTUS	CDS	15062	16102	0.98	+	0	ID=MALK_01520.t1.c2;Parent=MALK_01520.t1
contig005	AUGUSTUS	mRNA	15019	16102	0.98	+	.	ID=MALK_01520.t1;Parent=MALK_01520
contig005	AUGUSTUS	exon	15019	15021	.	+	.	ID=MALK_01520.t1.e1;Parent=MALK_01520.t1
contig005	AUGUSTUS	exon	15062	16102	.	+	.	ID=MALK_01520.t1.e2;Parent=MALK_01520.t1
contig005	AUGUSTUS	gene	16252	17493	1	+	.	ID=MALK_01521;prediction_source=augustus:contig005.g3562.t1
contig005	AUGUSTUS	CDS	16252	17493	1	+	0	ID=MALK_01521.t1.c1;Parent=MALK_01521.t1
contig005	AUGUSTUS	mRNA	16252	17493	1	+	.	ID=MALK_01521.t1;Parent=MALK_01521
contig005	AUGUSTUS	exon	16252	17493	1	+	.	ID=MALK_01521.t1.e1;Parent=MALK_01521.t1
contig005	AUGUSTUS	gene	17534	19270	0.89	-	.	ID=MALK_01522;prediction_source=augustus:contig005.g3563.t1
contig005	AUGUSTUS	CDS	17534	19270	0.89	-	0	ID=MALK_01522.t1.c1;Parent=MALK_01522.t1
contig005	AUGUSTUS	mRNA	17534	19270	0.89	-	.	ID=MALK_01522.t1;Parent=MALK_01522
contig005	AUGUSTUS	exon	17534	19270	0.89	-	.	ID=MALK_01522.t1.e1;Parent=MALK_01522.t1
contig005	maker	gene	19368	21977	.	+	.	ID=MALK_01523;prediction_source=maker_MRET:augustus_masked-contig005-processed-gene-0.5-mRNA-1
contig005	maker	CDS	19368	21977	.	+	0	ID=MALK_01523.t1.c1;Parent=MALK_01523.t1
contig005	maker	mRNA	19368	21977	.	+	.	ID=MALK_01523.t1;Parent=MALK_01523

contig005	maker	exon	19368	21977	.	+	.	ID=MALK_01523.t1.e1;Parent=MALK_01523.t1
contig005	AUGUSTUS	gene	21974	23122	0.63	-	.	ID=MALK_01524;prediction_source=augustus:contig005.g3565.t1
contig005	AUGUSTUS	CDS	21974	23122	0.63	-	0	ID=MALK_01524.t1.c1;Parent=MALK_01524.t1
contig005	AUGUSTUS	mRNA	21974	23122	0.63	-	.	ID=MALK_01524.t1;Parent=MALK_01524
contig005	AUGUSTUS	exon	21974	23122	0.63	-	.	ID=MALK_01524.t1.e1;Parent=MALK_01524.t1
contig005	maker	gene	24242	25609	.	-	.	ID=MALK_01525;prediction_source=maker_MRET:augustus_masked-contig005-processed-gene-0.24-mRNA-1
contig005	maker	CDS	24242	25609	.	-	0	ID=MALK_01525.t1.c1;Parent=MALK_01525.t1
contig005	maker	mRNA	24242	25609	.	-	.	ID=MALK_01525.t1;Parent=MALK_01525
contig005	maker	exon	24242	25609	.	-	.	ID=MALK_01525.t1.e1;Parent=MALK_01525.t1
contig005	maker	gene	26059	27474	.	+	.	ID=MALK_01526;prediction_source=maker_MRET:augustus_masked-contig005-processed-gene-0.6-mRNA-1
contig005	maker	CDS	26059	27474	.	+	0	ID=MALK_01526.t1.c1;Parent=MALK_01526.t1
contig005	maker	mRNA	26059	27474	.	+	.	ID=MALK_01526.t1;Parent=MALK_01526
contig005	maker	exon	26059	27474	.	+	.	ID=MALK_01526.t1.e1;Parent=MALK_01526.t1
contig005	maker	gene	27544	29103	.	+	.	ID=MALK_01527;prediction_source=maker_MRET:augustus_masked-contig005-processed-gene-0.7-mRNA-1
contig005	maker	CDS	27544	29103	.	+	0	ID=MALK_01527.t1.c1;Parent=MALK_01527.t1
contig005	maker	mRNA	27544	29103	.	+	.	ID=MALK_01527.t1;Parent=MALK_01527
contig005	maker	exon	27544	29103	.	+	.	ID=MALK_01527.t1.e1;Parent=MALK_01527.t1
contig005	maker	gene	29145	30608	.	-	.	ID=MALK_01528;prediction_source=maker_MRET:augustus_masked-contig005-processed-gene-0.25-mRNA-1
contig005	maker	CDS	29145	30608	.	-	0	ID=MALK_01528.t1.c1;Parent=MALK_01528.t1
contig005	maker	mRNA	29145	30608	.	-	.	ID=MALK_01528.t1;Parent=MALK_01528
contig005	maker	exon	29145	30608	.	-	.	ID=MALK_01528.t1.e1;Parent=MALK_01528.t1
contig005	AUGUSTUS	gene	30720	33755	0.92	-	.	ID=MALK_01529;prediction_source=augustus:contig005.g3570.t1
contig005	AUGUSTUS	CDS	30720	33755	0.92	-	0	ID=MALK_01529.t1.c1;Parent=MALK_01529.t1
contig005	AUGUSTUS	mRNA	30720	33755	0.92	-	.	ID=MALK_01529.t1;Parent=MALK_01529
contig005	AUGUSTUS	exon	30720	33755	0.92	-	.	ID=MALK_01529.t1.e1;Parent=MALK_01529.t1
contig005	AUGUSTUS	gene	34079	35626	0.94	-	.	ID=MALK_01530;prediction_source=augustus:contig005.g3573.t1
contig005	AUGUSTUS	CDS	34079	35626	0.94	-	0	ID=MALK_01530.t1.c1;Parent=MALK_01530.t1
contig005	AUGUSTUS	mRNA	34079	35626	0.94	-	.	ID=MALK_01530.t1;Parent=MALK_01530
contig005	AUGUSTUS	exon	34079	35626	0.94	-	.	ID=MALK_01530.t1.e1;Parent=MALK_01530.t1
contig005	AUGUSTUS	gene	35776	36700	0.89	-	.	ID=MALK_01531;prediction_source=braker_MRET:g2214.t1
contig005	AUGUSTUS	CDS	36143	36700	0.96	-	0	ID=MALK_01531.t1.c3;Parent=MALK_01531.t1
contig005	AUGUSTUS	CDS	35877	36105	0.96	-	0	ID=MALK_01531.t1.c2;Parent=MALK_01531.t1
contig005	AUGUSTUS	CDS	35776	35849	0.96	-	0	ID=MALK_01531.t1.c1;Parent=MALK_01531.t1
contig005	AUGUSTUS	mRNA	35776	36700	0.89	-	.	ID=MALK_01531.t1;Parent=MALK_01531
contig005	AUGUSTUS	exon	36143	36700	.	-	.	ID=MALK_01531.t1.e3;Parent=MALK_01531.t1
contig005	AUGUSTUS	exon	35877	36105	.	-	.	ID=MALK_01531.t1.e2;Parent=MALK_01531.t1
contig005	AUGUSTUS	exon	35776	35849	.	-	.	ID=MALK_01531.t1.e1;Parent=MALK_01531.t1
contig005	AUGUSTUS	gene	36820	41347	1	+	.	ID=MALK_01532;prediction_source=augustus:contig005.g3576.t1
contig005	AUGUSTUS	CDS	36820	36918	1	+	0	ID=MALK_01532.t1.c1;Parent=MALK_01532.t1
contig005	AUGUSTUS	CDS	37037	41347	1	+	0	ID=MALK_01532.t1.c2;Parent=MALK_01532.t1
contig005	AUGUSTUS	mRNA	36820	41347	1	+	.	ID=MALK_01532.t1;Parent=MALK_01532
contig005	AUGUSTUS	exon	36820	36918	1	+	.	ID=MALK_01532.t1.e1;Parent=MALK_01532.t1
contig005	AUGUSTUS	exon	37037	41347	1	+	.	ID=MALK_01532.t1.e2;Parent=MALK_01532.t1

contig005	AUGUSTUS	gene	41353	43059	0.97	-	.	ID=MALK_01533;prediction_source=augustus:contig005.g3577.t1
contig005	AUGUSTUS	CDS	41353	43059	0.97	-	0	ID=MALK_01533.t1.c1;Parent=MALK_01533.t1
contig005	AUGUSTUS	mRNA	41353	43059	0.97	-	.	ID=MALK_01533.t1;Parent=MALK_01533
contig005	AUGUSTUS	exon	41353	43059	0.97	-	.	ID=MALK_01533.t1.e1;Parent=MALK_01533.t1
contig005	AUGUSTUS	gene	43104	46145	1	+	.	ID=MALK_01534;prediction_source=augustus:contig005.g3578.t1
contig005	AUGUSTUS	CDS	43104	46145	1	+	0	ID=MALK_01534.t1.c1;Parent=MALK_01534.t1
contig005	AUGUSTUS	mRNA	43104	46145	1	+	.	ID=MALK_01534.t1;Parent=MALK_01534
contig005	AUGUSTUS	exon	43104	46145	1	+	.	ID=MALK_01534.t1.e1;Parent=MALK_01534.t1
contig005	AUGUSTUS	gene	47397	48818	0.56	+	.	ID=MALK_01535;prediction_source=augustus:contig005.g3580.t1
contig005	AUGUSTUS	CDS	47397	48818	0.56	+	0	ID=MALK_01535.t1.c1;Parent=MALK_01535.t1
contig005	AUGUSTUS	mRNA	47397	48818	0.56	+	.	ID=MALK_01535.t1;Parent=MALK_01535
contig005	AUGUSTUS	exon	47397	48818	0.56	+	.	ID=MALK_01535.t1.e1;Parent=MALK_01535.t1
contig005	AUGUSTUS	gene	48805	50529	0.98	-	.	ID=MALK_01536;prediction_source=augustus:contig005.g3581.t1
contig005	AUGUSTUS	CDS	48805	50529	0.98	-	0	ID=MALK_01536.t1.c1;Parent=MALK_01536.t1
contig005	AUGUSTUS	mRNA	48805	50529	0.98	-	.	ID=MALK_01536.t1;Parent=MALK_01536
contig005	AUGUSTUS	exon	48805	50529	0.98	-	.	ID=MALK_01536.t1.e1;Parent=MALK_01536.t1
contig005	AUGUSTUS	gene	50595	57134	0.99	-	.	ID=MALK_01537;prediction_source=augustus:contig005.g3582.t1
contig005	AUGUSTUS	CDS	50595	57134	0.99	-	0	ID=MALK_01537.t1.c1;Parent=MALK_01537.t1
contig005	AUGUSTUS	mRNA	50595	57134	0.99	-	.	ID=MALK_01537.t1;Parent=MALK_01537
contig005	AUGUSTUS	exon	50595	57134	0.99	-	.	ID=MALK_01537.t1.e1;Parent=MALK_01537.t1
contig005	AUGUSTUS	gene	57299	58726	0.24	-	.	ID=MALK_01538;prediction_source=braker_MRET:g2221.t1
contig005	AUGUSTUS	CDS	58505	58726	0.7	-	0	ID=MALK_01538.t1.c3;Parent=MALK_01538.t1
contig005	AUGUSTUS	CDS	58284	58466	0.7	-	0	ID=MALK_01538.t1.c2;Parent=MALK_01538.t1
contig005	AUGUSTUS	CDS	57299	58255	0.7	-	0	ID=MALK_01538.t1.c1;Parent=MALK_01538.t1
contig005	AUGUSTUS	mRNA	57299	58726	0.24	-	.	ID=MALK_01538.t1;Parent=MALK_01538
contig005	AUGUSTUS	exon	58505	58726	.	-	.	ID=MALK_01538.t1.e3;Parent=MALK_01538.t1
contig005	AUGUSTUS	exon	58284	58466	.	-	.	ID=MALK_01538.t1.e2;Parent=MALK_01538.t1
contig005	AUGUSTUS	exon	57299	58255	.	-	.	ID=MALK_01538.t1.e1;Parent=MALK_01538.t1
contig005	AUGUSTUS	gene	58962	59900	0.99	+	.	ID=MALK_01539;prediction_source=augustus:contig005.g3585.t1
contig005	AUGUSTUS	CDS	58962	59900	0.99	+	0	ID=MALK_01539.t1.c1;Parent=MALK_01539.t1
contig005	AUGUSTUS	mRNA	58962	59900	0.99	+	.	ID=MALK_01539.t1;Parent=MALK_01539
contig005	AUGUSTUS	exon	58962	59900	0.99	+	.	ID=MALK_01539.t1.e1;Parent=MALK_01539.t1
contig005	maker	gene	60355	61371	.	-	.	ID=MALK_01540;prediction_source=maker_MRET:augustus_masked-contig005-processed-gene-0.34-mRNA-1
contig005	maker	CDS	60355	61371	.	-	0	ID=MALK_01540.t1.c1;Parent=MALK_01540.t1
contig005	maker	mRNA	60355	61371	.	-	.	ID=MALK_01540.t1;Parent=MALK_01540
contig005	maker	exon	60355	61371	.	-	.	ID=MALK_01540.t1.e1;Parent=MALK_01540.t1
contig005	maker	gene	61517	63352	.	+	.	ID=MALK_01541;prediction_source=maker_MRET:augustus_masked-contig005-processed-gene-0.12-mRNA-1
contig005	maker	CDS	61517	63352	.	+	0	ID=MALK_01541.t1.c1;Parent=MALK_01541.t1
contig005	maker	mRNA	61517	63352	.	+	.	ID=MALK_01541.t1;Parent=MALK_01541
contig005	maker	exon	61517	63352	.	+	.	ID=MALK_01541.t1.e1;Parent=MALK_01541.t1
contig005	AUGUSTUS	gene	63397	66879	0.95	-	.	ID=MALK_01542;prediction_source=augustus:contig005.g3588.t1
contig005	AUGUSTUS	CDS	63397	66879	0.95	-	0	ID=MALK_01542.t1.c1;Parent=MALK_01542.t1
contig005	AUGUSTUS	mRNA	63397	66879	0.95	-	.	ID=MALK_01542.t1;Parent=MALK_01542

contig005	AUGUSTUS	exon	63397	66879	0.95	-	.	ID=MALK_01542.t1.e1;Parent=MALK_01542.t1
contig005	AUGUSTUS	gene	67259	68718	0.7	+	.	ID=MALK_01543;prediction_source=braker_MRET:g2226.t1
contig005	AUGUSTUS	CDS	67259	67280	0.95	+	0	ID=MALK_01543.t1.c1;Parent=MALK_01543.t1
contig005	AUGUSTUS	CDS	67469	68718	0.95	+	0	ID=MALK_01543.t1.c2;Parent=MALK_01543.t1
contig005	AUGUSTUS	mRNA	67259	68718	0.7	+	.	ID=MALK_01543.t1;Parent=MALK_01543
contig005	AUGUSTUS	exon	67259	67280	.	+	.	ID=MALK_01543.t1.e1;Parent=MALK_01543.t1
contig005	AUGUSTUS	exon	67469	68718	.	+	.	ID=MALK_01543.t1.e2;Parent=MALK_01543.t1
contig005	AUGUSTUS	gene	68755	70002	0.53	-	.	ID=MALK_01544;prediction_source=braker_MRET:g2227.t1
contig005	AUGUSTUS	CDS	69965	70002	0.63	-	0	ID=MALK_01544.t1.c3;Parent=MALK_01544.t1
contig005	AUGUSTUS	CDS	68837	69935	0.63	-	0	ID=MALK_01544.t1.c2;Parent=MALK_01544.t1
contig005	AUGUSTUS	CDS	68755	68808	0.63	-	0	ID=MALK_01544.t1.c1;Parent=MALK_01544.t1
contig005	AUGUSTUS	mRNA	68755	70002	0.53	-	.	ID=MALK_01544.t1;Parent=MALK_01544
contig005	AUGUSTUS	exon	69965	70002	.	-	.	ID=MALK_01544.t1.e3;Parent=MALK_01544.t1
contig005	AUGUSTUS	exon	68837	69935	.	-	.	ID=MALK_01544.t1.e2;Parent=MALK_01544.t1
contig005	AUGUSTUS	exon	68755	68808	.	-	.	ID=MALK_01544.t1.e1;Parent=MALK_01544.t1
contig005	maker	gene	70212	72041	.	+	.	ID=MALK_01545;prediction_source=maker_MRET:augustus_masked-contig005-processed-gene-0.14-mRNA-1
contig005	maker	CDS	70212	72041	.	+	0	ID=MALK_01545.t1.c1;Parent=MALK_01545.t1
contig005	maker	mRNA	70212	72041	.	+	.	ID=MALK_01545.t1;Parent=MALK_01545
contig005	maker	exon	70212	72041	.	+	.	ID=MALK_01545.t1.e1;Parent=MALK_01545.t1
contig005	maker	gene	72085	73101	.	-	.	ID=MALK_01546;prediction_source=maker_MRET:augustus_masked-contig005-processed-gene-0.36-mRNA-1
contig005	maker	CDS	72085	73101	.	-	0	ID=MALK_01546.t1.c1;Parent=MALK_01546.t1
contig005	maker	mRNA	72085	73101	.	-	.	ID=MALK_01546.t1;Parent=MALK_01546
contig005	maker	exon	72085	73101	.	-	.	ID=MALK_01546.t1.e1;Parent=MALK_01546.t1
contig005	AUGUSTUS	gene	73313	74611	0.49	-	.	ID=MALK_01547;prediction_source=augustus:contig005.g3594.t1
contig005	AUGUSTUS	CDS	73313	74611	0.49	-	0	ID=MALK_01547.t1.c1;Parent=MALK_01547.t1
contig005	AUGUSTUS	mRNA	73313	74611	0.49	-	.	ID=MALK_01547.t1;Parent=MALK_01547
contig005	AUGUSTUS	exon	73313	74611	0.49	-	.	ID=MALK_01547.t1.e1;Parent=MALK_01547.t1
contig005	AUGUSTUS	gene	74760	75079	0.84	+	.	ID=MALK_01548;prediction_source=braker_MRET:g2231.t1
contig005	AUGUSTUS	CDS	74760	75024	0.9	+	0	ID=MALK_01548.t1.c1;Parent=MALK_01548.t1
contig005	AUGUSTUS	CDS	75054	75079	0.9	+	0	ID=MALK_01548.t1.c2;Parent=MALK_01548.t1
contig005	AUGUSTUS	mRNA	74760	75079	0.84	+	.	ID=MALK_01548.t1;Parent=MALK_01548
contig005	AUGUSTUS	exon	74760	75024	.	+	.	ID=MALK_01548.t1.e1;Parent=MALK_01548.t1
contig005	AUGUSTUS	exon	75054	75079	.	+	.	ID=MALK_01548.t1.e2;Parent=MALK_01548.t1
contig005	AUGUSTUS	gene	75123	77240	0.23	-	.	ID=MALK_01549;prediction_source=augustus:contig005.g3596.t1
contig005	AUGUSTUS	CDS	77089	77240	0.74	-	0	ID=MALK_01549.t1.c2;Parent=MALK_01549.t1
contig005	AUGUSTUS	CDS	75123	76632	0.74	-	0	ID=MALK_01549.t1.c1;Parent=MALK_01549.t1
contig005	AUGUSTUS	mRNA	75123	77240	0.23	-	.	ID=MALK_01549.t1;Parent=MALK_01549
contig005	AUGUSTUS	exon	77089	77240	0.74	-	.	ID=MALK_01549.t1.e2;Parent=MALK_01549.t1
contig005	AUGUSTUS	exon	75123	76632	0.74	-	.	ID=MALK_01549.t1.e1;Parent=MALK_01549.t1
contig005	AUGUSTUS	gene	78046	78844	0.86	-	.	ID=MALK_01550;prediction_source=braker_MRET:g2233.t1
contig005	AUGUSTUS	CDS	78773	78844	1	-	0	ID=MALK_01550.t1.c4;Parent=MALK_01550.t1
contig005	AUGUSTUS	CDS	78280	78722	1	-	0	ID=MALK_01550.t1.c3;Parent=MALK_01550.t1
contig005	AUGUSTUS	CDS	78136	78233	1	-	0	ID=MALK_01550.t1.c2;Parent=MALK_01550.t1

contig005	AUGUSTUS	CDS	78046	78092	1	-	0	ID=MALK_01550.t1.c1;Parent=MALK_01550.t1
contig005	AUGUSTUS	mRNA	78046	78844	0.86	-	.	ID=MALK_01550.t1;Parent=MALK_01550
contig005	AUGUSTUS	exon	78773	78844	.	-	.	ID=MALK_01550.t1.e4;Parent=MALK_01550.t1
contig005	AUGUSTUS	exon	78280	78722	.	-	.	ID=MALK_01550.t1.e3;Parent=MALK_01550.t1
contig005	AUGUSTUS	exon	78136	78233	.	-	.	ID=MALK_01550.t1.e2;Parent=MALK_01550.t1
contig005	AUGUSTUS	exon	78046	78092	.	-	.	ID=MALK_01550.t1.e1;Parent=MALK_01550.t1
contig005	maker	gene	79114	82353	.	-	.	ID=MALK_01551;prediction_source=maker_MRET:augustus_masked-contig005-processed-gene-0.39-mRNA-1
contig005	maker	CDS	79114	82353	.	-	0	ID=MALK_01551.t1.c1;Parent=MALK_01551.t1
contig005	maker	mRNA	79114	82353	.	-	.	ID=MALK_01551.t1;Parent=MALK_01551
contig005	maker	exon	79114	82353	.	-	.	ID=MALK_01551.t1.e1;Parent=MALK_01551.t1
contig005	AUGUSTUS	gene	82973	85975	0.91	+	.	ID=MALK_01552;prediction_source=augustus:contig005.g3602.t1
contig005	AUGUSTUS	CDS	82973	85975	0.91	+	0	ID=MALK_01552.t1.c1;Parent=MALK_01552.t1
contig005	AUGUSTUS	mRNA	82973	85975	0.91	+	.	ID=MALK_01552.t1;Parent=MALK_01552
contig005	AUGUSTUS	exon	82973	85975	0.91	+	.	ID=MALK_01552.t1.e1;Parent=MALK_01552.t1
contig005	maker	gene	85989	86720	.	-	.	ID=MALK_01553;prediction_source=maker_MRET:augustus_masked-contig005-processed-gene-0.40-mRNA-1
contig005	maker	CDS	85989	86720	.	-	0	ID=MALK_01553.t1.c1;Parent=MALK_01553.t1
contig005	maker	mRNA	85989	86720	.	-	.	ID=MALK_01553.t1;Parent=MALK_01553
contig005	maker	exon	85989	86720	.	-	.	ID=MALK_01553.t1.e1;Parent=MALK_01553.t1
contig005	AUGUSTUS	gene	86869	87987	0.95	+	.	ID=MALK_01554;prediction_source=braker_MRET:g2237.t1
contig005	AUGUSTUS	CDS	86869	87987	0.95	+	0	ID=MALK_01554.t1.c1;Parent=MALK_01554.t1
contig005	AUGUSTUS	mRNA	86869	87987	0.95	+	.	ID=MALK_01554.t1;Parent=MALK_01554
contig005	AUGUSTUS	exon	86869	87987	.	+	.	ID=MALK_01554.t1.e1;Parent=MALK_01554.t1
contig005	AUGUSTUS	gene	88036	89556	0.41	-	.	ID=MALK_01555;prediction_source=braker_MRET:g2238.t1
contig005	AUGUSTUS	CDS	88183	89556	0.45	-	0	ID=MALK_01555.t1.c2;Parent=MALK_01555.t1
contig005	AUGUSTUS	CDS	88036	88149	0.45	-	0	ID=MALK_01555.t1.c1;Parent=MALK_01555.t1
contig005	AUGUSTUS	mRNA	88036	89556	0.41	-	.	ID=MALK_01555.t1;Parent=MALK_01555
contig005	AUGUSTUS	exon	88183	89556	.	-	.	ID=MALK_01555.t1.e2;Parent=MALK_01555.t1
contig005	AUGUSTUS	exon	88036	88149	.	-	.	ID=MALK_01555.t1.e1;Parent=MALK_01555.t1
contig005	AUGUSTUS	gene	89874	90839	0.88	+	.	ID=MALK_01556;prediction_source=augustus:contig005.g3607.t1
contig005	AUGUSTUS	CDS	89874	90839	0.88	+	0	ID=MALK_01556.t1.c1;Parent=MALK_01556.t1
contig005	AUGUSTUS	mRNA	89874	90839	0.88	+	.	ID=MALK_01556.t1;Parent=MALK_01556
contig005	AUGUSTUS	exon	89874	90839	0.88	+	.	ID=MALK_01556.t1.e1;Parent=MALK_01556.t1
contig005	AUGUSTUS	gene	91943	92526	0.74	+	.	ID=MALK_01557;prediction_source=braker_MRET:g2240.t1
contig005	AUGUSTUS	CDS	91943	92114	0.97	+	0	ID=MALK_01557.t1.c1;Parent=MALK_01557.t1
contig005	AUGUSTUS	CDS	92150	92526	0.97	+	0	ID=MALK_01557.t1.c2;Parent=MALK_01557.t1
contig005	AUGUSTUS	mRNA	91943	92526	0.74	+	.	ID=MALK_01557.t1;Parent=MALK_01557
contig005	AUGUSTUS	exon	91943	92114	.	+	.	ID=MALK_01557.t1.e1;Parent=MALK_01557.t1
contig005	AUGUSTUS	exon	92150	92526	.	+	.	ID=MALK_01557.t1.e2;Parent=MALK_01557.t1
contig005	AUGUSTUS	gene	93086	94206	0.26	+	.	ID=MALK_01558;prediction_source=braker_MRET:g2241.t1
contig005	AUGUSTUS	CDS	93086	93096	0.26	+	0	ID=MALK_01558.t1.c1;Parent=MALK_01558.t1
contig005	AUGUSTUS	CDS	93135	94206	0.26	+	0	ID=MALK_01558.t1.c2;Parent=MALK_01558.t1
contig005	AUGUSTUS	mRNA	93086	94206	0.26	+	.	ID=MALK_01558.t1;Parent=MALK_01558
contig005	AUGUSTUS	exon	93086	93096	.	+	.	ID=MALK_01558.t1.e1;Parent=MALK_01558.t1

contig005	AUGUSTUS	exon	93135	94206	.	+	.	ID=MALK_01558.t1.e2;Parent=MALK_01558.t1
contig005	maker	gene	94210	95061	.	-	.	ID=MALK_01559;prediction_source=maker_MRET:augustus_masked-contig005-processed-gene-1.2-mRNA-1
contig005	maker	CDS	94210	95061	.	-	0	ID=MALK_01559.t1.c1;Parent=MALK_01559.t1
contig005	maker	mRNA	94210	95061	.	-	.	ID=MALK_01559.t1;Parent=MALK_01559
contig005	maker	exon	94210	95061	.	-	.	ID=MALK_01559.t1.e1;Parent=MALK_01559.t1
contig005	AUGUSTUS	gene	95116	96998	0.91	+	.	ID=MALK_01560;prediction_source=braker_MRET:g2243.t1
contig005	AUGUSTUS	CDS	95116	95193	1	+	0	ID=MALK_01560.t1.c1;Parent=MALK_01560.t1
contig005	AUGUSTUS	CDS	95232	96998	1	+	0	ID=MALK_01560.t1.c2;Parent=MALK_01560.t1
contig005	AUGUSTUS	mRNA	95116	96998	0.91	+	.	ID=MALK_01560.t1;Parent=MALK_01560
contig005	AUGUSTUS	exon	95116	95193	.	+	.	ID=MALK_01560.t1.e1;Parent=MALK_01560.t1
contig005	AUGUSTUS	exon	95232	96998	.	+	.	ID=MALK_01560.t1.e2;Parent=MALK_01560.t1
contig005	AUGUSTUS	gene	97151	98659	0.92	+	.	ID=MALK_01561;prediction_source=augustus:contig005.g3611.t1
contig005	AUGUSTUS	CDS	97151	98659	0.92	+	0	ID=MALK_01561.t1.c1;Parent=MALK_01561.t1
contig005	AUGUSTUS	mRNA	97151	98659	0.92	+	.	ID=MALK_01561.t1;Parent=MALK_01561
contig005	AUGUSTUS	exon	97151	98659	0.92	+	.	ID=MALK_01561.t1.e1;Parent=MALK_01561.t1
contig005	AUGUSTUS	gene	98767	100635	0.84	-	.	ID=MALK_01562;prediction_source=augustus:contig005.g3612.t1
contig005	AUGUSTUS	CDS	98767	100635	0.84	-	0	ID=MALK_01562.t1.c1;Parent=MALK_01562.t1
contig005	AUGUSTUS	mRNA	98767	100635	0.84	-	.	ID=MALK_01562.t1;Parent=MALK_01562
contig005	AUGUSTUS	exon	98767	100635	0.84	-	.	ID=MALK_01562.t1.e1;Parent=MALK_01562.t1
contig005	AUGUSTUS	gene	100956	101789	0.37	+	.	ID=MALK_01563;prediction_source=braker_MRET:g2246.t1
contig005	AUGUSTUS	CDS	100956	101188	0.39	+	0	ID=MALK_01563.t1.c1;Parent=MALK_01563.t1
contig005	AUGUSTUS	CDS	101232	101319	0.39	+	0	ID=MALK_01563.t1.c2;Parent=MALK_01563.t1
contig005	AUGUSTUS	CDS	101358	101789	0.39	+	0	ID=MALK_01563.t1.c3;Parent=MALK_01563.t1
contig005	AUGUSTUS	mRNA	100956	101789	0.37	+	.	ID=MALK_01563.t1;Parent=MALK_01563
contig005	AUGUSTUS	exon	100956	101188	.	+	.	ID=MALK_01563.t1.e1;Parent=MALK_01563.t1
contig005	AUGUSTUS	exon	101232	101319	.	+	.	ID=MALK_01563.t1.e2;Parent=MALK_01563.t1
contig005	AUGUSTUS	exon	101358	101789	.	+	.	ID=MALK_01563.t1.e3;Parent=MALK_01563.t1
contig005	AUGUSTUS	gene	102025	103440	0.18	+	.	ID=MALK_01564;prediction_source=augustus:contig005.g3616.t1
contig005	AUGUSTUS	CDS	102025	103440	0.18	+	0	ID=MALK_01564.t1.c1;Parent=MALK_01564.t1
contig005	AUGUSTUS	mRNA	102025	103440	0.18	+	.	ID=MALK_01564.t1;Parent=MALK_01564
contig005	AUGUSTUS	exon	102025	103440	0.18	+	.	ID=MALK_01564.t1.e1;Parent=MALK_01564.t1
contig005	AUGUSTUS	gene	103579	105900	0.32	+	.	ID=MALK_01565;prediction_source=braker_MRET:g2248.t1
contig005	AUGUSTUS	CDS	103579	103596	0.95	+	0	ID=MALK_01565.t1.c1;Parent=MALK_01565.t1
contig005	AUGUSTUS	CDS	103628	103685	0.95	+	0	ID=MALK_01565.t1.c2;Parent=MALK_01565.t1
contig005	AUGUSTUS	CDS	103718	105900	0.95	+	0	ID=MALK_01565.t1.c3;Parent=MALK_01565.t1
contig005	AUGUSTUS	mRNA	103579	105900	0.32	+	.	ID=MALK_01565.t1;Parent=MALK_01565
contig005	AUGUSTUS	exon	103579	103596	.	+	.	ID=MALK_01565.t1.e1;Parent=MALK_01565.t1
contig005	AUGUSTUS	exon	103628	103685	.	+	.	ID=MALK_01565.t1.e2;Parent=MALK_01565.t1
contig005	AUGUSTUS	exon	103718	105900	.	+	.	ID=MALK_01565.t1.e3;Parent=MALK_01565.t1
contig005	maker	gene	106041	108323	.	+	.	ID=MALK_01566;prediction_source=maker_MRET:augustus_masked-contig005-processed-gene-1.7-mRNA-1
contig005	maker	CDS	106041	108323	.	+	0	ID=MALK_01566.t1.c1;Parent=MALK_01566.t1
contig005	maker	mRNA	106041	108323	.	+	.	ID=MALK_01566.t1;Parent=MALK_01566
contig005	maker	exon	106041	108323	.	+	.	ID=MALK_01566.t1.e1;Parent=MALK_01566.t1

contig005	maker	gene	108427	110811	.	-	.	ID=MALK_01567;prediction_source=maker_MRET:augustus_masked-contig005-processed-gene-1.59-mRNA-1
contig005	maker	CDS	108427	110811	.	-	0	ID=MALK_01567.t1.c1;Parent=MALK_01567.t1
contig005	maker	mRNA	108427	110811	.	-	.	ID=MALK_01567.t1;Parent=MALK_01567
contig005	maker	exon	108427	110811	.	-	.	ID=MALK_01567.t1.e1;Parent=MALK_01567.t1
contig005	AUGUSTUS	gene	110939	111610	0.91	-	.	ID=MALK_01568;prediction_source=augustus:contig005.g3620.t1
contig005	AUGUSTUS	CDS	110939	111610	0.91	-	0	ID=MALK_01568.t1.c1;Parent=MALK_01568.t1
contig005	AUGUSTUS	mRNA	110939	111610	0.91	-	.	ID=MALK_01568.t1;Parent=MALK_01568
contig005	AUGUSTUS	exon	110939	111610	0.91	-	.	ID=MALK_01568.t1.e1;Parent=MALK_01568.t1
contig005	AUGUSTUS	gene	111838	113895	0.95	+	.	ID=MALK_01569;prediction_source=augustus:contig005.g3621.t1
contig005	AUGUSTUS	CDS	111838	113895	0.95	+	0	ID=MALK_01569.t1.c1;Parent=MALK_01569.t1
contig005	AUGUSTUS	mRNA	111838	113895	0.95	+	.	ID=MALK_01569.t1;Parent=MALK_01569
contig005	AUGUSTUS	exon	111838	113895	0.95	+	.	ID=MALK_01569.t1.e1;Parent=MALK_01569.t1
contig005	AUGUSTUS	gene	113863	117084	0.49	-	.	ID=MALK_01570;prediction_source=augustus:contig005.g3622.t1
contig005	AUGUSTUS	CDS	113863	117084	0.49	-	0	ID=MALK_01570.t1.c1;Parent=MALK_01570.t1
contig005	AUGUSTUS	mRNA	113863	117084	0.49	-	.	ID=MALK_01570.t1;Parent=MALK_01570
contig005	AUGUSTUS	exon	113863	117084	0.49	-	.	ID=MALK_01570.t1.e1;Parent=MALK_01570.t1
contig005	maker	gene	117246	118940	.	+	.	ID=MALK_01571;prediction_source=maker_MRET:augustus_masked-contig005-processed-gene-1.9-mRNA-1
contig005	maker	CDS	117246	118940	.	+	0	ID=MALK_01571.t1.c1;Parent=MALK_01571.t1
contig005	maker	mRNA	117246	118940	.	+	.	ID=MALK_01571.t1;Parent=MALK_01571
contig005	maker	exon	117246	118940	.	+	.	ID=MALK_01571.t1.e1;Parent=MALK_01571.t1
contig005	maker	gene	119000	120433	.	+	.	ID=MALK_01572;prediction_source=maker_MRET:augustus_masked-contig005-processed-gene-1.10-mRNA-1
contig005	maker	CDS	119000	120433	.	+	0	ID=MALK_01572.t1.c1;Parent=MALK_01572.t1
contig005	maker	mRNA	119000	120433	.	+	.	ID=MALK_01572.t1;Parent=MALK_01572
contig005	maker	exon	119000	120433	.	+	.	ID=MALK_01572.t1.e1;Parent=MALK_01572.t1
contig005	maker	gene	120477	122492	.	-	.	ID=MALK_01573;prediction_source=maker_MRET:augustus_masked-contig005-processed-gene-1.62-mRNA-1
contig005	maker	CDS	120477	122492	.	-	0	ID=MALK_01573.t1.c1;Parent=MALK_01573.t1
contig005	maker	mRNA	120477	122492	.	-	.	ID=MALK_01573.t1;Parent=MALK_01573
contig005	maker	exon	120477	122492	.	-	.	ID=MALK_01573.t1.e1;Parent=MALK_01573.t1
contig005	maker	gene	122905	123483	.	+	.	ID=MALK_01574;prediction_source=maker_MRET:augustus_masked-contig005-processed-gene-1.11-mRNA-1
contig005	maker	CDS	122905	123483	.	+	0	ID=MALK_01574.t1.c1;Parent=MALK_01574.t1
contig005	maker	mRNA	122905	123483	.	+	.	ID=MALK_01574.t1;Parent=MALK_01574
contig005	maker	exon	122905	123483	.	+	.	ID=MALK_01574.t1.e1;Parent=MALK_01574.t1
contig005	maker	gene	123486	124621	.	-	.	ID=MALK_01575;prediction_source=maker_MRET:augustus_masked-contig005-processed-gene-1.63-mRNA-1
contig005	maker	CDS	124614	124621	.	-	0	ID=MALK_01575.t1.c1;Parent=MALK_01575.t1
contig005	maker	CDS	123486	124332	.	-	0	ID=MALK_01575.t1.c2;Parent=MALK_01575.t1
contig005	maker	mRNA	123486	124621	.	-	.	ID=MALK_01575.t1;Parent=MALK_01575
contig005	maker	exon	124614	124621	.	-	.	ID=MALK_01575.t1.e1;Parent=MALK_01575.t1
contig005	maker	exon	123486	124332	.	-	.	ID=MALK_01575.t1.e2;Parent=MALK_01575.t1
contig005	AUGUSTUS	gene	125296	127425	0.99	+	.	ID=MALK_01576;prediction_source=augustus:contig005.g3629.t1
contig005	AUGUSTUS	CDS	125296	127425	0.99	+	0	ID=MALK_01576.t1.c1;Parent=MALK_01576.t1
contig005	AUGUSTUS	mRNA	125296	127425	0.99	+	.	ID=MALK_01576.t1;Parent=MALK_01576
contig005	AUGUSTUS	exon	125296	127425	0.99	+	.	ID=MALK_01576.t1.e1;Parent=MALK_01576.t1
contig005	AUGUSTUS	gene	127719	132374	0.86	+	.	ID=MALK_01577;prediction_source=augustus:contig005.g3630.t1

contig005	AUGUSTUS	CDS	127719	132374	0.86	+	0	ID=MALK_01577.t1.c1;Parent=MALK_01577.t1
contig005	AUGUSTUS	mRNA	127719	132374	0.86	+	.	ID=MALK_01577.t1;Parent=MALK_01577
contig005	AUGUSTUS	exon	127719	132374	0.86	+	.	ID=MALK_01577.t1.e1;Parent=MALK_01577.t1
contig005	maker	gene	132396	133352	.	-	.	ID=MALK_01578;prediction_source=maker_MRET:augustus_masked-contig005-processed-gene-1.64-mRNA-1
contig005	maker	CDS	132396	133352	.	-	0	ID=MALK_01578.t1.c1;Parent=MALK_01578.t1
contig005	maker	mRNA	132396	133352	.	-	.	ID=MALK_01578.t1;Parent=MALK_01578
contig005	maker	exon	132396	133352	.	-	.	ID=MALK_01578.t1.e1;Parent=MALK_01578.t1
contig005	AUGUSTUS	gene	134129	136396	0.7	+	.	ID=MALK_01579;prediction_source=braker_MRET:g2262.t1
contig005	AUGUSTUS	CDS	134129	136396	0.7	+	0	ID=MALK_01579.t1.c1;Parent=MALK_01579.t1
contig005	AUGUSTUS	mRNA	134129	136396	0.7	+	.	ID=MALK_01579.t1;Parent=MALK_01579
contig005	AUGUSTUS	exon	134129	136396	.	+	.	ID=MALK_01579.t1.e1;Parent=MALK_01579.t1
contig005	maker	gene	136393	137982	.	-	.	ID=MALK_01580;prediction_source=maker_MRET:augustus_masked-contig005-processed-gene-1.65-mRNA-1
contig005	maker	CDS	136393	137982	.	-	0	ID=MALK_01580.t1.c1;Parent=MALK_01580.t1
contig005	maker	mRNA	136393	137982	.	-	.	ID=MALK_01580.t1;Parent=MALK_01580
contig005	maker	exon	136393	137982	.	-	.	ID=MALK_01580.t1.e1;Parent=MALK_01580.t1
contig005	AUGUSTUS	gene	138072	138539	0.39	-	.	ID=MALK_01581;prediction_source=augustus:contig005.g3636.t1
contig005	AUGUSTUS	CDS	138072	138539	0.39	-	0	ID=MALK_01581.t1.c1;Parent=MALK_01581.t1
contig005	AUGUSTUS	mRNA	138072	138539	0.39	-	.	ID=MALK_01581.t1;Parent=MALK_01581
contig005	AUGUSTUS	exon	138072	138539	0.39	-	.	ID=MALK_01581.t1.e1;Parent=MALK_01581.t1
contig005	AUGUSTUS	gene	138863	140878	0.46	-	.	ID=MALK_01582;prediction_source=braker_MRET:g2264.t1
contig005	AUGUSTUS	CDS	138863	140878	0.46	-	0	ID=MALK_01582.t1.c1;Parent=MALK_01582.t1
contig005	AUGUSTUS	mRNA	138863	140878	0.46	-	.	ID=MALK_01582.t1;Parent=MALK_01582
contig005	AUGUSTUS	exon	138863	140878	.	-	.	ID=MALK_01582.t1.e1;Parent=MALK_01582.t1
contig005	AUGUSTUS	gene	140911	142209	0.74	-	.	ID=MALK_01583;prediction_source=braker_MRET:g2265.t1
contig005	AUGUSTUS	CDS	140911	142209	0.74	-	0	ID=MALK_01583.t1.c1;Parent=MALK_01583.t1
contig005	AUGUSTUS	mRNA	140911	142209	0.74	-	.	ID=MALK_01583.t1;Parent=MALK_01583
contig005	AUGUSTUS	exon	140911	142209	.	-	.	ID=MALK_01583.t1.e1;Parent=MALK_01583.t1
contig005	maker	gene	142331	145420	.	+	.	ID=MALK_01584;prediction_source=maker_MRET:augustus_masked-contig005-processed-gene-1.15-mRNA-1
contig005	maker	CDS	142331	145420	.	+	0	ID=MALK_01584.t1.c1;Parent=MALK_01584.t1
contig005	maker	mRNA	142331	145420	.	+	.	ID=MALK_01584.t1;Parent=MALK_01584
contig005	maker	exon	142331	145420	.	+	.	ID=MALK_01584.t1.e1;Parent=MALK_01584.t1
contig005	AUGUSTUS	gene	145499	146395	0.95	+	.	ID=MALK_01585;prediction_source=augustus:contig005.g3641.t1
contig005	AUGUSTUS	CDS	145499	146395	0.95	+	0	ID=MALK_01585.t1.c1;Parent=MALK_01585.t1
contig005	AUGUSTUS	mRNA	145499	146395	0.95	+	.	ID=MALK_01585.t1;Parent=MALK_01585
contig005	AUGUSTUS	exon	145499	146395	0.95	+	.	ID=MALK_01585.t1.e1;Parent=MALK_01585.t1
contig005	AUGUSTUS	gene	146571	148205	0.92	+	.	ID=MALK_01586;prediction_source=augustus:contig005.g3642.t1
contig005	AUGUSTUS	CDS	146571	148205	0.92	+	0	ID=MALK_01586.t1.c1;Parent=MALK_01586.t1
contig005	AUGUSTUS	mRNA	146571	148205	0.92	+	.	ID=MALK_01586.t1;Parent=MALK_01586
contig005	AUGUSTUS	exon	146571	148205	0.92	+	.	ID=MALK_01586.t1.e1;Parent=MALK_01586.t1
contig005	maker	gene	148278	150188	.	+	.	ID=MALK_01587;prediction_source=maker_MRET:augustus_masked-contig005-processed-gene-1.18-mRNA-1
contig005	maker	CDS	148278	150188	.	+	0	ID=MALK_01587.t1.c1;Parent=MALK_01587.t1
contig005	maker	mRNA	148278	150188	.	+	.	ID=MALK_01587.t1;Parent=MALK_01587
contig005	maker	exon	148278	150188	.	+	.	ID=MALK_01587.t1.e1;Parent=MALK_01587.t1

contig005	maker	gene	150342	152075	.	+	.	ID=MALK_01588;prediction_source=maker_MRET:augustus_masked-contig005-processed-gene-1.19-mRNA-1
contig005	maker	CDS	150342	152075	.	+	0	ID=MALK_01588.t1.c1;Parent=MALK_01588.t1
contig005	maker	mRNA	150342	152075	.	+	.	ID=MALK_01588.t1;Parent=MALK_01588
contig005	maker	exon	150342	152075	.	+	.	ID=MALK_01588.t1.e1;Parent=MALK_01588.t1
contig005	AUGUSTUS	gene	152320	152862	1	-	.	ID=MALK_01589;prediction_source=augustus:contig005.g3647.t1
contig005	AUGUSTUS	CDS	152320	152862	1	-	0	ID=MALK_01589.t1.c1;Parent=MALK_01589.t1
contig005	AUGUSTUS	mRNA	152320	152862	1	-	.	ID=MALK_01589.t1;Parent=MALK_01589
contig005	AUGUSTUS	exon	152320	152862	1	-	.	ID=MALK_01589.t1.e1;Parent=MALK_01589.t1
contig005	maker	gene	153053	154684	.	+	.	ID=MALK_01590;prediction_source=maker_MRET:augustus_masked-contig005-processed-gene-1.20-mRNA-1
contig005	maker	CDS	153053	154684	.	+	0	ID=MALK_01590.t1.c1;Parent=MALK_01590.t1
contig005	maker	mRNA	153053	154684	.	+	.	ID=MALK_01590.t1;Parent=MALK_01590
contig005	maker	exon	153053	154684	.	+	.	ID=MALK_01590.t1.e1;Parent=MALK_01590.t1
contig005	maker	gene	154758	158075	.	+	.	ID=MALK_01591;prediction_source=maker_MRET:augustus_masked-contig005-processed-gene-1.21-mRNA-1
contig005	maker	CDS	154758	158075	.	+	0	ID=MALK_01591.t1.c1;Parent=MALK_01591.t1
contig005	maker	mRNA	154758	158075	.	+	.	ID=MALK_01591.t1;Parent=MALK_01591
contig005	maker	exon	154758	158075	.	+	.	ID=MALK_01591.t1.e1;Parent=MALK_01591.t1
contig005	maker	gene	158558	159652	.	+	.	ID=MALK_01592;prediction_source=maker_MRET:augustus_masked-contig005-processed-gene-1.22-mRNA-1
contig005	maker	CDS	158558	159652	.	+	0	ID=MALK_01592.t1.c1;Parent=MALK_01592.t1
contig005	maker	mRNA	158558	159652	.	+	.	ID=MALK_01592.t1;Parent=MALK_01592
contig005	maker	exon	158558	159652	.	+	.	ID=MALK_01592.t1.e1;Parent=MALK_01592.t1
contig005	AUGUSTUS	gene	159670	161460	0.5	-	.	ID=MALK_01593;prediction_source=augustus:contig005.g3649.t1
contig005	AUGUSTUS	CDS	159670	161460	0.5	-	0	ID=MALK_01593.t1.c1;Parent=MALK_01593.t1
contig005	AUGUSTUS	mRNA	159670	161460	0.5	-	.	ID=MALK_01593.t1;Parent=MALK_01593
contig005	AUGUSTUS	exon	159670	161460	0.5	-	.	ID=MALK_01593.t1.e1;Parent=MALK_01593.t1
contig005	maker	gene	161543	164488	.	-	.	ID=MALK_01594;prediction_source=maker_MRET:augustus_masked-contig005-processed-gene-1.71-mRNA-1
contig005	maker	CDS	161543	164488	.	-	0	ID=MALK_01594.t1.c1;Parent=MALK_01594.t1
contig005	maker	mRNA	161543	164488	.	-	.	ID=MALK_01594.t1;Parent=MALK_01594
contig005	maker	exon	161543	164488	.	-	.	ID=MALK_01594.t1.e1;Parent=MALK_01594.t1
contig005	maker	gene	164974	165294	.	-	.	ID=MALK_01595;prediction_source=maker_MRET:augustus_masked-contig005-processed-gene-1.72-mRNA-1
contig005	maker	CDS	164974	165294	.	-	0	ID=MALK_01595.t1.c1;Parent=MALK_01595.t1
contig005	maker	mRNA	164974	165294	.	-	.	ID=MALK_01595.t1;Parent=MALK_01595
contig005	maker	exon	164974	165294	.	-	.	ID=MALK_01595.t1.e1;Parent=MALK_01595.t1
contig005	AUGUSTUS	gene	165913	166466	0.96	+	.	ID=MALK_01596;prediction_source=braker_MRET:g2276.t1
contig005	AUGUSTUS	CDS	165913	166009	1	+	0	ID=MALK_01596.t1.c1;Parent=MALK_01596.t1
contig005	AUGUSTUS	CDS	166058	166194	1	+	0	ID=MALK_01596.t1.c2;Parent=MALK_01596.t1
contig005	AUGUSTUS	CDS	166235	166245	1	+	0	ID=MALK_01596.t1.c3;Parent=MALK_01596.t1
contig005	AUGUSTUS	CDS	166344	166354	1	+	0	ID=MALK_01596.t1.c4;Parent=MALK_01596.t1
contig005	AUGUSTUS	CDS	166399	166466	1	+	0	ID=MALK_01596.t1.c5;Parent=MALK_01596.t1
contig005	AUGUSTUS	mRNA	165913	166466	0.96	+	.	ID=MALK_01596.t1;Parent=MALK_01596
contig005	AUGUSTUS	exon	165913	166009	.	+	.	ID=MALK_01596.t1.e1;Parent=MALK_01596.t1
contig005	AUGUSTUS	exon	166058	166194	.	+	.	ID=MALK_01596.t1.e2;Parent=MALK_01596.t1
contig005	AUGUSTUS	exon	166235	166245	.	+	.	ID=MALK_01596.t1.e3;Parent=MALK_01596.t1
contig005	AUGUSTUS	exon	166344	166354	.	+	.	ID=MALK_01596.t1.e4;Parent=MALK_01596.t1

contig005	AUGUSTUS	exon	166399	166466	.	+	.	ID=MALK_01596.t1.e5;Parent=MALK_01596.t1
contig005	maker	gene	166985	168445	.	+	.	ID=MALK_01597;prediction_source=maker_MRET:augustus_masked-contig005-processed-gene-1.23-mRNA-1
contig005	maker	CDS	166985	168445	.	+	0	ID=MALK_01597.t1.c1;Parent=MALK_01597.t1
contig005	maker	mRNA	166985	168445	.	+	.	ID=MALK_01597.t1;Parent=MALK_01597
contig005	maker	exon	166985	168445	.	+	.	ID=MALK_01597.t1.e1;Parent=MALK_01597.t1
contig005	maker	gene	168645	169259	.	-	.	ID=MALK_01598;prediction_source=maker_MRET:augustus_masked-contig005-processed-gene-1.73-mRNA-1
contig005	maker	CDS	168645	169259	.	-	0	ID=MALK_01598.t1.c1;Parent=MALK_01598.t1
contig005	maker	mRNA	168645	169259	.	-	.	ID=MALK_01598.t1;Parent=MALK_01598
contig005	maker	exon	168645	169259	.	-	.	ID=MALK_01598.t1.e1;Parent=MALK_01598.t1
contig005	AUGUSTUS	gene	169594	170538	1	+	.	ID=MALK_01599;prediction_source=braker_MRET:g2279.t1
contig005	AUGUSTUS	CDS	169594	169596	1	+	0	ID=MALK_01599.t1.c1;Parent=MALK_01599.t1
contig005	AUGUSTUS	CDS	169639	170538	1	+	0	ID=MALK_01599.t1.c2;Parent=MALK_01599.t1
contig005	AUGUSTUS	mRNA	169594	170538	1	+	.	ID=MALK_01599.t1;Parent=MALK_01599
contig005	AUGUSTUS	exon	169594	169596	.	+	.	ID=MALK_01599.t1.e1;Parent=MALK_01599.t1
contig005	AUGUSTUS	exon	169639	170538	.	+	.	ID=MALK_01599.t1.e2;Parent=MALK_01599.t1
contig005	AUGUSTUS	gene	170764	172248	0.5	+	.	ID=MALK_01600;prediction_source=braker_MRET:g2280.t1
contig005	AUGUSTUS	CDS	170764	172248	0.5	+	0	ID=MALK_01600.t1.c1;Parent=MALK_01600.t1
contig005	AUGUSTUS	mRNA	170764	172248	0.5	+	.	ID=MALK_01600.t1;Parent=MALK_01600
contig005	AUGUSTUS	exon	170764	172248	.	+	.	ID=MALK_01600.t1.e1;Parent=MALK_01600.t1
contig005	AUGUSTUS	gene	172335	174026	0.42	+	.	ID=MALK_01601;prediction_source=braker_MRET:g2281.t1
contig005	AUGUSTUS	CDS	172335	174026	0.42	+	0	ID=MALK_01601.t1.c1;Parent=MALK_01601.t1
contig005	AUGUSTUS	mRNA	172335	174026	0.42	+	.	ID=MALK_01601.t1;Parent=MALK_01601
contig005	AUGUSTUS	exon	172335	174026	.	+	.	ID=MALK_01601.t1.e1;Parent=MALK_01601.t1
contig005	AUGUSTUS	gene	174095	175642	1	-	.	ID=MALK_01602;prediction_source=augustus:contig005.g3657.t1
contig005	AUGUSTUS	CDS	174095	175642	1	-	0	ID=MALK_01602.t1.c1;Parent=MALK_01602.t1
contig005	AUGUSTUS	mRNA	174095	175642	1	-	.	ID=MALK_01602.t1;Parent=MALK_01602
contig005	AUGUSTUS	exon	174095	175642	1	-	.	ID=MALK_01602.t1.e1;Parent=MALK_01602.t1
contig005	AUGUSTUS	gene	176167	176508	0.4	+	.	ID=MALK_01603;prediction_source=braker_MRET:g2283.t1
contig005	AUGUSTUS	CDS	176167	176315	0.78	+	0	ID=MALK_01603.t1.c1;Parent=MALK_01603.t1
contig005	AUGUSTUS	CDS	176343	176508	0.78	+	0	ID=MALK_01603.t1.c2;Parent=MALK_01603.t1
contig005	AUGUSTUS	mRNA	176167	176508	0.4	+	.	ID=MALK_01603.t1;Parent=MALK_01603
contig005	AUGUSTUS	exon	176167	176315	.	+	.	ID=MALK_01603.t1.e1;Parent=MALK_01603.t1
contig005	AUGUSTUS	exon	176343	176508	.	+	.	ID=MALK_01603.t1.e2;Parent=MALK_01603.t1
contig005	AUGUSTUS	gene	177069	178112	0.65	-	.	ID=MALK_01604;prediction_source=augustus:contig005.g3658.t1
contig005	AUGUSTUS	CDS	177069	178112	0.65	-	0	ID=MALK_01604.t1.c1;Parent=MALK_01604.t1
contig005	AUGUSTUS	mRNA	177069	178112	0.65	-	.	ID=MALK_01604.t1;Parent=MALK_01604
contig005	AUGUSTUS	exon	177069	178112	0.65	-	.	ID=MALK_01604.t1.e1;Parent=MALK_01604.t1
contig005	maker	gene	178174	179835	.	+	.	ID=MALK_01605;prediction_source=maker_MRET:augustus_masked-contig005-processed-gene-1.26-mRNA-1
contig005	maker	CDS	178174	179835	.	+	0	ID=MALK_01605.t1.c1;Parent=MALK_01605.t1
contig005	maker	mRNA	178174	179835	.	+	.	ID=MALK_01605.t1;Parent=MALK_01605
contig005	maker	exon	178174	179835	.	+	.	ID=MALK_01605.t1.e1;Parent=MALK_01605.t1
contig005	AUGUSTUS	gene	179874	180338	0.99	-	.	ID=MALK_01606;prediction_source=braker_MRET:g2286.t1
contig005	AUGUSTUS	CDS	180336	180338	1	-	0	ID=MALK_01606.t1.c2;Parent=MALK_01606.t1

contig005	AUGUSTUS	CDS	179874	180290	1	-	0	ID=MALK_01606.t1.c1;Parent=MALK_01606.t1
contig005	AUGUSTUS	mRNA	179874	180338	0.99	-	.	ID=MALK_01606.t1;Parent=MALK_01606
contig005	AUGUSTUS	exon	180336	180338	.	-	.	ID=MALK_01606.t1.e2;Parent=MALK_01606.t1
contig005	AUGUSTUS	exon	179874	180290	.	-	.	ID=MALK_01606.t1.e1;Parent=MALK_01606.t1
contig005	AUGUSTUS	gene	180977	181768	0.82	+	.	ID=MALK_01607;prediction_source=braker_MRET:g2287.t1
contig005	AUGUSTUS	CDS	180977	181057	0.99	+	0	ID=MALK_01607.t1.c1;Parent=MALK_01607.t1
contig005	AUGUSTUS	CDS	181209	181223	0.99	+	0	ID=MALK_01607.t1.c2;Parent=MALK_01607.t1
contig005	AUGUSTUS	CDS	181274	181768	0.99	+	0	ID=MALK_01607.t1.c3;Parent=MALK_01607.t1
contig005	AUGUSTUS	mRNA	180977	181768	0.82	+	.	ID=MALK_01607.t1;Parent=MALK_01607
contig005	AUGUSTUS	exon	180977	181057	.	+	.	ID=MALK_01607.t1.e1;Parent=MALK_01607.t1
contig005	AUGUSTUS	exon	181209	181223	.	+	.	ID=MALK_01607.t1.e2;Parent=MALK_01607.t1
contig005	AUGUSTUS	exon	181274	181768	.	+	.	ID=MALK_01607.t1.e3;Parent=MALK_01607.t1
contig005	maker	gene	182088	183746	.	+	.	ID=MALK_01608;prediction_source=maker_MRET:augustus_masked-contig005-processed-gene-1.28-mRNA-1
contig005	maker	CDS	182088	183746	.	+	0	ID=MALK_01608.t1.c1;Parent=MALK_01608.t1
contig005	maker	mRNA	182088	183746	.	+	.	ID=MALK_01608.t1;Parent=MALK_01608
contig005	maker	exon	182088	183746	.	+	.	ID=MALK_01608.t1.e1;Parent=MALK_01608.t1
contig005	AUGUSTUS	gene	183759	184589	0.87	-	.	ID=MALK_01609;prediction_source=braker_MRET:g2289.t1
contig005	AUGUSTUS	CDS	183759	184589	0.87	-	0	ID=MALK_01609.t1.c1;Parent=MALK_01609.t1
contig005	AUGUSTUS	mRNA	183759	184589	0.87	-	.	ID=MALK_01609.t1;Parent=MALK_01609
contig005	AUGUSTUS	exon	183759	184589	.	-	.	ID=MALK_01609.t1.e1;Parent=MALK_01609.t1
contig005	AUGUSTUS	gene	184670	185572	0.76	+	.	ID=MALK_01610;prediction_source=augustus:contig005.g3663.t1
contig005	AUGUSTUS	CDS	184670	185572	0.76	+	0	ID=MALK_01610.t1.c1;Parent=MALK_01610.t1
contig005	AUGUSTUS	mRNA	184670	185572	0.76	+	.	ID=MALK_01610.t1;Parent=MALK_01610
contig005	AUGUSTUS	exon	184670	185572	0.76	+	.	ID=MALK_01610.t1.e1;Parent=MALK_01610.t1
contig005	AUGUSTUS	gene	185818	186345	0.71	-	.	ID=MALK_01611;prediction_source=augustus:contig005.g3664.t1
contig005	AUGUSTUS	CDS	185818	186345	0.71	-	0	ID=MALK_01611.t1.c1;Parent=MALK_01611.t1
contig005	AUGUSTUS	mRNA	185818	186345	0.71	-	.	ID=MALK_01611.t1;Parent=MALK_01611
contig005	AUGUSTUS	exon	185818	186345	0.71	-	.	ID=MALK_01611.t1.e1;Parent=MALK_01611.t1
contig005	maker	gene	186400	189465	.	+	.	ID=MALK_01612;prediction_source=maker_MRET:augustus_masked-contig005-processed-gene-1.30-mRNA-1
contig005	maker	CDS	186400	189465	.	+	0	ID=MALK_01612.t1.c1;Parent=MALK_01612.t1
contig005	maker	mRNA	186400	189465	.	+	.	ID=MALK_01612.t1;Parent=MALK_01612
contig005	maker	exon	186400	189465	.	+	.	ID=MALK_01612.t1.e1;Parent=MALK_01612.t1
contig005	maker	gene	189467	189886	.	-	.	ID=MALK_01613;prediction_source=maker_MRET:augustus_masked-contig005-processed-gene-1.78-mRNA-1
contig005	maker	CDS	189467	189886	.	-	0	ID=MALK_01613.t1.c1;Parent=MALK_01613.t1
contig005	maker	mRNA	189467	189886	.	-	.	ID=MALK_01613.t1;Parent=MALK_01613
contig005	maker	exon	189467	189886	.	-	.	ID=MALK_01613.t1.e1;Parent=MALK_01613.t1
contig005	AUGUSTUS	gene	190063	191646	0.62	+	.	ID=MALK_01614;prediction_source=augustus:contig005.g3667.t1
contig005	AUGUSTUS	CDS	190063	191646	0.62	+	0	ID=MALK_01614.t1.c1;Parent=MALK_01614.t1
contig005	AUGUSTUS	mRNA	190063	191646	0.62	+	.	ID=MALK_01614.t1;Parent=MALK_01614
contig005	AUGUSTUS	exon	190063	191646	0.62	+	.	ID=MALK_01614.t1.e1;Parent=MALK_01614.t1
contig005	AUGUSTUS	gene	191757	192893	0.63	+	.	ID=MALK_01615;prediction_source=augustus:contig005.g3668.t1
contig005	AUGUSTUS	CDS	191757	192893	0.63	+	0	ID=MALK_01615.t1.c1;Parent=MALK_01615.t1
contig005	AUGUSTUS	mRNA	191757	192893	0.63	+	.	ID=MALK_01615.t1;Parent=MALK_01615

contig005	AUGUSTUS	exon	191757	192893	0.63	+	.	ID=MALK_01615.t1.e1;Parent=MALK_01615.t1
contig005	AUGUSTUS	gene	192964	197082	0.63	-	.	ID=MALK_01616;prediction_source=augustus:contig005.g3669.t1
contig005	AUGUSTUS	CDS	192964	197082	0.63	-	0	ID=MALK_01616.t1.c1;Parent=MALK_01616.t1
contig005	AUGUSTUS	mRNA	192964	197082	0.63	-	.	ID=MALK_01616.t1;Parent=MALK_01616
contig005	AUGUSTUS	exon	192964	197082	0.63	-	.	ID=MALK_01616.t1.e1;Parent=MALK_01616.t1
contig005	AUGUSTUS	gene	197917	200313	0.4	+	.	ID=MALK_01617;prediction_source=augustus:contig005.g3670.t1
contig005	AUGUSTUS	CDS	197917	200313	0.4	+	0	ID=MALK_01617.t1.c1;Parent=MALK_01617.t1
contig005	AUGUSTUS	mRNA	197917	200313	0.4	+	.	ID=MALK_01617.t1;Parent=MALK_01617
contig005	AUGUSTUS	exon	197917	200313	0.4	+	.	ID=MALK_01617.t1.e1;Parent=MALK_01617.t1
contig005	maker	gene	200319	201263	.	-	.	ID=MALK_01618;prediction_source=maker_MRET:augustus_masked-contig005-processed-gene-1.80-mRNA-1
contig005	maker	CDS	200319	201263	.	-	0	ID=MALK_01618.t1.c1;Parent=MALK_01618.t1
contig005	maker	mRNA	200319	201263	.	-	.	ID=MALK_01618.t1;Parent=MALK_01618
contig005	maker	exon	200319	201263	.	-	.	ID=MALK_01618.t1.e1;Parent=MALK_01618.t1
contig005	AUGUSTUS	gene	201623	203281	0.89	+	.	ID=MALK_01619;prediction_source=braker_MRET:g2299.t1
contig005	AUGUSTUS	CDS	201623	203281	0.89	+	0	ID=MALK_01619.t1.c1;Parent=MALK_01619.t1
contig005	AUGUSTUS	mRNA	201623	203281	0.89	+	.	ID=MALK_01619.t1;Parent=MALK_01619
contig005	AUGUSTUS	exon	201623	203281	.	+	.	ID=MALK_01619.t1.e1;Parent=MALK_01619.t1
contig005	AUGUSTUS	gene	203422	204105	0.71	+	.	ID=MALK_01620;prediction_source=braker_MRET:g2300.t1
contig005	AUGUSTUS	CDS	203422	204105	0.71	+	0	ID=MALK_01620.t1.c1;Parent=MALK_01620.t1
contig005	AUGUSTUS	mRNA	203422	204105	0.71	+	.	ID=MALK_01620.t1;Parent=MALK_01620
contig005	AUGUSTUS	exon	203422	204105	.	+	.	ID=MALK_01620.t1.e1;Parent=MALK_01620.t1
contig005	AUGUSTUS	gene	204108	204680	0.56	-	.	ID=MALK_01621;prediction_source=braker_MRET:g2301.t1
contig005	AUGUSTUS	CDS	204474	204680	0.56	-	0	ID=MALK_01621.t1.c3;Parent=MALK_01621.t1
contig005	AUGUSTUS	CDS	204403	204438	0.56	-	0	ID=MALK_01621.t1.c2;Parent=MALK_01621.t1
contig005	AUGUSTUS	CDS	204108	204371	0.56	-	0	ID=MALK_01621.t1.c1;Parent=MALK_01621.t1
contig005	AUGUSTUS	mRNA	204108	204680	0.56	-	.	ID=MALK_01621.t1;Parent=MALK_01621
contig005	AUGUSTUS	exon	204474	204680	.	-	.	ID=MALK_01621.t1.e3;Parent=MALK_01621.t1
contig005	AUGUSTUS	exon	204403	204438	.	-	.	ID=MALK_01621.t1.e2;Parent=MALK_01621.t1
contig005	AUGUSTUS	exon	204108	204371	.	-	.	ID=MALK_01621.t1.e1;Parent=MALK_01621.t1
contig005	AUGUSTUS	gene	204840	208034	1	+	.	ID=MALK_01622;prediction_source=augustus:contig005.g3674.t1
contig005	AUGUSTUS	CDS	204840	208034	1	+	0	ID=MALK_01622.t1.c1;Parent=MALK_01622.t1
contig005	AUGUSTUS	mRNA	204840	208034	1	+	.	ID=MALK_01622.t1;Parent=MALK_01622
contig005	AUGUSTUS	exon	204840	208034	1	+	.	ID=MALK_01622.t1.e1;Parent=MALK_01622.t1
contig005	AUGUSTUS	gene	208048	212541	1	-	.	ID=MALK_01623;prediction_source=augustus:contig005.g3675.t1
contig005	AUGUSTUS	CDS	208048	212541	1	-	0	ID=MALK_01623.t1.c1;Parent=MALK_01623.t1
contig005	AUGUSTUS	mRNA	208048	212541	1	-	.	ID=MALK_01623.t1;Parent=MALK_01623
contig005	AUGUSTUS	exon	208048	212541	1	-	.	ID=MALK_01623.t1.e1;Parent=MALK_01623.t1
contig005	maker	gene	212649	214784	.	+	.	ID=MALK_01624;prediction_source=maker_MRET:augustus_masked-contig005-processed-gene-1.36-mRNA-1
contig005	maker	CDS	212649	214784	.	+	0	ID=MALK_01624.t1.c1;Parent=MALK_01624.t1
contig005	maker	mRNA	212649	214784	.	+	.	ID=MALK_01624.t1;Parent=MALK_01624
contig005	maker	exon	212649	214784	.	+	.	ID=MALK_01624.t1.e1;Parent=MALK_01624.t1
contig005	maker	gene	214852	215862	.	-	.	ID=MALK_01625;prediction_source=maker_MRET:augustus_masked-contig005-processed-gene-1.83-mRNA-1
contig005	maker	CDS	214852	215862	.	-	0	ID=MALK_01625.t1.c1;Parent=MALK_01625.t1

contig005	maker	mRNA	214852	215862	.	-	.	ID=MALK_01625.t1;Parent=MALK_01625
contig005	maker	exon	214852	215862	.	-	.	ID=MALK_01625.t1.e1;Parent=MALK_01625.t1
contig005	AUGUSTUS	gene	216072	218321	0.83	+	.	ID=MALK_01626;prediction_source=augustus:contig005.g3678.t1
contig005	AUGUSTUS	CDS	216072	218321	0.83	+	0	ID=MALK_01626.t1.c1;Parent=MALK_01626.t1
contig005	AUGUSTUS	mRNA	216072	218321	0.83	+	.	ID=MALK_01626.t1;Parent=MALK_01626
contig005	AUGUSTUS	exon	216072	218321	0.83	+	.	ID=MALK_01626.t1.e1;Parent=MALK_01626.t1
contig005	AUGUSTUS	gene	218433	219425	0.82	+	.	ID=MALK_01627;prediction_source=augustus:contig005.g3679.t1
contig005	AUGUSTUS	CDS	218433	219425	0.82	+	0	ID=MALK_01627.t1.c1;Parent=MALK_01627.t1
contig005	AUGUSTUS	mRNA	218433	219425	0.82	+	.	ID=MALK_01627.t1;Parent=MALK_01627
contig005	AUGUSTUS	exon	218433	219425	0.82	+	.	ID=MALK_01627.t1.e1;Parent=MALK_01627.t1
contig005	AUGUSTUS	gene	219660	220673	0.87	-	.	ID=MALK_01628;prediction_source=braker_MRET:g2308.t1
contig005	AUGUSTUS	CDS	219894	220673	0.93	-	0	ID=MALK_01628.t1.c2;Parent=MALK_01628.t1
contig005	AUGUSTUS	CDS	219660	219860	0.93	-	0	ID=MALK_01628.t1.c1;Parent=MALK_01628.t1
contig005	AUGUSTUS	mRNA	219660	220673	0.87	-	.	ID=MALK_01628.t1;Parent=MALK_01628
contig005	AUGUSTUS	exon	219894	220673	.	-	.	ID=MALK_01628.t1.e2;Parent=MALK_01628.t1
contig005	AUGUSTUS	exon	219660	219860	.	-	.	ID=MALK_01628.t1.e1;Parent=MALK_01628.t1
contig005	maker	gene	221377	224337	.	+	.	ID=MALK_01629;prediction_source=maker_MRET:augustus_masked-contig005-processed-gene-1.39-mRNA-1
contig005	maker	CDS	221377	224337	.	+	0	ID=MALK_01629.t1.c1;Parent=MALK_01629.t1
contig005	maker	mRNA	221377	224337	.	+	.	ID=MALK_01629.t1;Parent=MALK_01629
contig005	maker	exon	221377	224337	.	+	.	ID=MALK_01629.t1.e1;Parent=MALK_01629.t1
contig005	AUGUSTUS	gene	224900	225271	0.86	+	.	ID=MALK_01630;prediction_source=augustus:contig005.g3682.t1
contig005	AUGUSTUS	CDS	224900	225271	0.86	+	0	ID=MALK_01630.t1.c1;Parent=MALK_01630.t1
contig005	AUGUSTUS	mRNA	224900	225271	0.86	+	.	ID=MALK_01630.t1;Parent=MALK_01630
contig005	AUGUSTUS	exon	224900	225271	0.86	+	.	ID=MALK_01630.t1.e1;Parent=MALK_01630.t1
contig005	AUGUSTUS	gene	225347	229548	0.97	-	.	ID=MALK_01631;prediction_source=braker_MRET:g2311.t1
contig005	AUGUSTUS	CDS	227820	229548	1	-	0	ID=MALK_01631.t1.c2;Parent=MALK_01631.t1
contig005	AUGUSTUS	CDS	225347	227787	1	-	0	ID=MALK_01631.t1.c1;Parent=MALK_01631.t1
contig005	AUGUSTUS	mRNA	225347	229548	0.97	-	.	ID=MALK_01631.t1;Parent=MALK_01631
contig005	AUGUSTUS	exon	227820	229548	.	-	.	ID=MALK_01631.t1.e2;Parent=MALK_01631.t1
contig005	AUGUSTUS	exon	225347	227787	.	-	.	ID=MALK_01631.t1.e1;Parent=MALK_01631.t1
contig005	AUGUSTUS	gene	229673	230804	0.56	-	.	ID=MALK_01632;prediction_source=braker_MRET:g2312.t1
contig005	AUGUSTUS	CDS	229895	230804	0.62	-	0	ID=MALK_01632.t1.c3;Parent=MALK_01632.t1
contig005	AUGUSTUS	CDS	229734	229856	0.62	-	0	ID=MALK_01632.t1.c2;Parent=MALK_01632.t1
contig005	AUGUSTUS	CDS	229673	229704	0.62	-	0	ID=MALK_01632.t1.c1;Parent=MALK_01632.t1
contig005	AUGUSTUS	mRNA	229673	230804	0.56	-	.	ID=MALK_01632.t1;Parent=MALK_01632
contig005	AUGUSTUS	exon	229895	230804	.	-	.	ID=MALK_01632.t1.e3;Parent=MALK_01632.t1
contig005	AUGUSTUS	exon	229734	229856	.	-	.	ID=MALK_01632.t1.e2;Parent=MALK_01632.t1
contig005	AUGUSTUS	exon	229673	229704	.	-	.	ID=MALK_01632.t1.e1;Parent=MALK_01632.t1
contig005	AUGUSTUS	gene	230976	233577	0.83	+	.	ID=MALK_01633;prediction_source=braker_MRET:g2313.t1
contig005	AUGUSTUS	CDS	230976	232455	0.99	+	0	ID=MALK_01633.t1.c1;Parent=MALK_01633.t1
contig005	AUGUSTUS	CDS	232607	233577	0.99	+	0	ID=MALK_01633.t1.c2;Parent=MALK_01633.t1
contig005	AUGUSTUS	mRNA	230976	233577	0.83	+	.	ID=MALK_01633.t1;Parent=MALK_01633
contig005	AUGUSTUS	exon	230976	232455	.	+	.	ID=MALK_01633.t1.e1;Parent=MALK_01633.t1

contig005	AUGUSTUS	exon	232607	233577	.	+	.	ID=MALK_01633.t1.e2;Parent=MALK_01633.t1
contig005	maker	gene	233598	235454	.	-	.	ID=MALK_01634;prediction_source=maker_MRET:augustus_masked-contig005-processed-gene-1.88-mRNA-1
contig005	maker	CDS	233598	235454	.	-	0	ID=MALK_01634.t1.c1;Parent=MALK_01634.t1
contig005	maker	mRNA	233598	235454	.	-	.	ID=MALK_01634.t1;Parent=MALK_01634
contig005	maker	exon	233598	235454	.	-	.	ID=MALK_01634.t1.e1;Parent=MALK_01634.t1
contig005	maker	gene	235568	238489	.	-	.	ID=MALK_01635;prediction_source=maker_MRET:augustus_masked-contig005-processed-gene-1.89-mRNA-1
contig005	maker	CDS	235568	238489	.	-	0	ID=MALK_01635.t1.c1;Parent=MALK_01635.t1
contig005	maker	mRNA	235568	238489	.	-	.	ID=MALK_01635.t1;Parent=MALK_01635
contig005	maker	exon	235568	238489	.	-	.	ID=MALK_01635.t1.e1;Parent=MALK_01635.t1
contig005	AUGUSTUS	gene	238531	239331	0.23	+	.	ID=MALK_01636;prediction_source=braker_MRET:g2315.t1
contig005	AUGUSTUS	CDS	238531	238911	0.99	+	0	ID=MALK_01636.t1.c1;Parent=MALK_01636.t1
contig005	AUGUSTUS	CDS	238968	239268	0.99	+	0	ID=MALK_01636.t1.c2;Parent=MALK_01636.t1
contig005	AUGUSTUS	CDS	239297	239331	0.99	+	0	ID=MALK_01636.t1.c3;Parent=MALK_01636.t1
contig005	AUGUSTUS	mRNA	238531	239331	0.23	+	.	ID=MALK_01636.t1;Parent=MALK_01636
contig005	AUGUSTUS	exon	238531	238911	.	+	.	ID=MALK_01636.t1.e1;Parent=MALK_01636.t1
contig005	AUGUSTUS	exon	238968	239268	.	+	.	ID=MALK_01636.t1.e2;Parent=MALK_01636.t1
contig005	AUGUSTUS	exon	239297	239331	.	+	.	ID=MALK_01636.t1.e3;Parent=MALK_01636.t1
contig005	maker	gene	239589	239993	.	-	.	ID=MALK_01637;prediction_source=maker_MRET:augustus_masked-contig005-processed-gene-1.90-mRNA-1
contig005	maker	CDS	239589	239993	.	-	0	ID=MALK_01637.t1.c1;Parent=MALK_01637.t1
contig005	maker	mRNA	239589	239993	.	-	.	ID=MALK_01637.t1;Parent=MALK_01637
contig005	maker	exon	239589	239993	.	-	.	ID=MALK_01637.t1.e1;Parent=MALK_01637.t1
contig005	maker	gene	240526	243795	.	-	.	ID=MALK_01638;prediction_source=maker_MRET:augustus_masked-contig005-processed-gene-1.91-mRNA-1
contig005	maker	CDS	240526	243795	.	-	0	ID=MALK_01638.t1.c1;Parent=MALK_01638.t1
contig005	maker	mRNA	240526	243795	.	-	.	ID=MALK_01638.t1;Parent=MALK_01638
contig005	maker	exon	240526	243795	.	-	.	ID=MALK_01638.t1.e1;Parent=MALK_01638.t1
contig005	AUGUSTUS	gene	244446	244943	0.86	+	.	ID=MALK_01639;prediction_source=augustus:contig005.g3695.t1
contig005	AUGUSTUS	CDS	244446	244943	0.86	+	0	ID=MALK_01639.t1.c1;Parent=MALK_01639.t1
contig005	AUGUSTUS	mRNA	244446	244943	0.86	+	.	ID=MALK_01639.t1;Parent=MALK_01639
contig005	AUGUSTUS	exon	244446	244943	0.86	+	.	ID=MALK_01639.t1.e1;Parent=MALK_01639.t1
contig005	AUGUSTUS	gene	244948	250080	1	-	.	ID=MALK_01640;prediction_source=augustus:contig005.g3696.t1
contig005	AUGUSTUS	CDS	244948	250080	1	-	0	ID=MALK_01640.t1.c1;Parent=MALK_01640.t1
contig005	AUGUSTUS	mRNA	244948	250080	1	-	.	ID=MALK_01640.t1;Parent=MALK_01640
contig005	AUGUSTUS	exon	244948	250080	1	-	.	ID=MALK_01640.t1.e1;Parent=MALK_01640.t1
contig005	AUGUSTUS	gene	250766	253960	0.3	+	.	ID=MALK_01641;prediction_source=augustus:contig005.g3699.t1
contig005	AUGUSTUS	CDS	250766	253960	0.3	+	0	ID=MALK_01641.t1.c1;Parent=MALK_01641.t1
contig005	AUGUSTUS	mRNA	250766	253960	0.3	+	.	ID=MALK_01641.t1;Parent=MALK_01641
contig005	AUGUSTUS	exon	250766	253960	0.3	+	.	ID=MALK_01641.t1.e1;Parent=MALK_01641.t1
contig005	AUGUSTUS	gene	253942	256233	0.97	-	.	ID=MALK_01642;prediction_source=augustus:contig005.g3701.t1
contig005	AUGUSTUS	CDS	253942	256233	0.97	-	0	ID=MALK_01642.t1.c1;Parent=MALK_01642.t1
contig005	AUGUSTUS	mRNA	253942	256233	0.97	-	.	ID=MALK_01642.t1;Parent=MALK_01642
contig005	AUGUSTUS	exon	253942	256233	0.97	-	.	ID=MALK_01642.t1.e1;Parent=MALK_01642.t1
contig005	AUGUSTUS	gene	256438	257313	1	-	.	ID=MALK_01643;prediction_source=augustus:contig005.g3702.t1
contig005	AUGUSTUS	CDS	256438	257313	1	-	0	ID=MALK_01643.t1.c1;Parent=MALK_01643.t1

contig005	AUGUSTUS	mRNA	256438	257313	1	-	.	ID=MALK_01643.t1;Parent=MALK_01643
contig005	AUGUSTUS	exon	256438	257313	1	-	.	ID=MALK_01643.t1.e1;Parent=MALK_01643.t1
contig005	AUGUSTUS	gene	257693	258220	0.95	+	.	ID=MALK_01644;prediction_source=braker_MRET:g2321.t1
contig005	AUGUSTUS	CDS	257693	257728	1	+	0	ID=MALK_01644.t1.c1;Parent=MALK_01644.t1
contig005	AUGUSTUS	CDS	257780	258220	1	+	0	ID=MALK_01644.t1.c2;Parent=MALK_01644.t1
contig005	AUGUSTUS	mRNA	257693	258220	0.95	+	.	ID=MALK_01644.t1;Parent=MALK_01644
contig005	AUGUSTUS	exon	257693	257728	.	+	.	ID=MALK_01644.t1.e1;Parent=MALK_01644.t1
contig005	AUGUSTUS	exon	257780	258220	.	+	.	ID=MALK_01644.t1.e2;Parent=MALK_01644.t1
contig005	AUGUSTUS	gene	258465	258951	0.6	+	.	ID=MALK_01645;prediction_source=braker_MRET:g2322.t1
contig005	AUGUSTUS	CDS	258465	258834	0.62	+	0	ID=MALK_01645.t1.c1;Parent=MALK_01645.t1
contig005	AUGUSTUS	CDS	258863	258951	0.62	+	0	ID=MALK_01645.t1.c2;Parent=MALK_01645.t1
contig005	AUGUSTUS	mRNA	258465	258951	0.6	+	.	ID=MALK_01645.t1;Parent=MALK_01645
contig005	AUGUSTUS	exon	258465	258834	.	+	.	ID=MALK_01645.t1.e1;Parent=MALK_01645.t1
contig005	AUGUSTUS	exon	258863	258951	.	+	.	ID=MALK_01645.t1.e2;Parent=MALK_01645.t1
contig005	AUGUSTUS	gene	259035	261890	0.97	+	.	ID=MALK_01646;prediction_source=augustus:contig005.g3705.t1
contig005	AUGUSTUS	CDS	259035	261890	0.97	+	0	ID=MALK_01646.t1.c1;Parent=MALK_01646.t1
contig005	AUGUSTUS	mRNA	259035	261890	0.97	+	.	ID=MALK_01646.t1;Parent=MALK_01646
contig005	AUGUSTUS	exon	259035	261890	0.97	+	.	ID=MALK_01646.t1.e1;Parent=MALK_01646.t1
contig005	AUGUSTUS	gene	261900	263744	0.9	-	.	ID=MALK_01647;prediction_source=augustus:contig005.g3707.t1
contig005	AUGUSTUS	CDS	261900	263744	0.9	-	0	ID=MALK_01647.t1.c1;Parent=MALK_01647.t1
contig005	AUGUSTUS	mRNA	261900	263744	0.9	-	.	ID=MALK_01647.t1;Parent=MALK_01647
contig005	AUGUSTUS	exon	261900	263744	0.9	-	.	ID=MALK_01647.t1.e1;Parent=MALK_01647.t1
contig005	AUGUSTUS	gene	263779	264870	0.86	+	.	ID=MALK_01648;prediction_source=braker_MRET:g2325.t1
contig005	AUGUSTUS	CDS	263779	264870	0.86	+	0	ID=MALK_01648.t1.c1;Parent=MALK_01648.t1
contig005	AUGUSTUS	mRNA	263779	264870	0.86	+	.	ID=MALK_01648.t1;Parent=MALK_01648
contig005	AUGUSTUS	exon	263779	264870	.	+	.	ID=MALK_01648.t1.e1;Parent=MALK_01648.t1
contig005	AUGUSTUS	gene	264915	266150	0.6	+	.	ID=MALK_01649;prediction_source=augustus:contig005.g3709.t1
contig005	AUGUSTUS	CDS	264915	266150	0.6	+	0	ID=MALK_01649.t1.c1;Parent=MALK_01649.t1
contig005	AUGUSTUS	mRNA	264915	266150	0.6	+	.	ID=MALK_01649.t1;Parent=MALK_01649
contig005	AUGUSTUS	exon	264915	266150	0.6	+	.	ID=MALK_01649.t1.e1;Parent=MALK_01649.t1
contig005	AUGUSTUS	gene	266154	267560	0.98	-	.	ID=MALK_01650;prediction_source=braker_MRET:g2327.t1
contig005	AUGUSTUS	CDS	266154	267560	0.98	-	0	ID=MALK_01650.t1.c1;Parent=MALK_01650.t1
contig005	AUGUSTUS	mRNA	266154	267560	0.98	-	.	ID=MALK_01650.t1;Parent=MALK_01650
contig005	AUGUSTUS	exon	266154	267560	.	-	.	ID=MALK_01650.t1.e1;Parent=MALK_01650.t1
contig005	AUGUSTUS	gene	267828	269023	0.48	+	.	ID=MALK_01651;prediction_source=braker_MRET:g2328.t1
contig005	AUGUSTUS	CDS	267828	268713	0.94	+	0	ID=MALK_01651.t1.c1;Parent=MALK_01651.t1
contig005	AUGUSTUS	CDS	268839	269023	0.94	+	0	ID=MALK_01651.t1.c2;Parent=MALK_01651.t1
contig005	AUGUSTUS	mRNA	267828	269023	0.48	+	.	ID=MALK_01651.t1;Parent=MALK_01651
contig005	AUGUSTUS	exon	267828	268713	.	+	.	ID=MALK_01651.t1.e1;Parent=MALK_01651.t1
contig005	AUGUSTUS	exon	268839	269023	.	+	.	ID=MALK_01651.t1.e2;Parent=MALK_01651.t1
contig005	AUGUSTUS	gene	269030	269722	0.7	-	.	ID=MALK_01652;prediction_source=augustus:contig005.g3712.t1
contig005	AUGUSTUS	CDS	269030	269722	0.7	-	0	ID=MALK_01652.t1.c1;Parent=MALK_01652.t1
contig005	AUGUSTUS	mRNA	269030	269722	0.7	-	.	ID=MALK_01652.t1;Parent=MALK_01652

contig005	AUGUSTUS	exon	269030	269722	0.7	-	.	ID=MALK_01652.t1.e1;Parent=MALK_01652.t1
contig005	AUGUSTUS	gene	269992	271146	0.92	-	.	ID=MALK_01653;prediction_source=braker_MRET:g2330.t1
contig005	AUGUSTUS	CDS	269992	271146	0.92	-	0	ID=MALK_01653.t1.c1;Parent=MALK_01653.t1
contig005	AUGUSTUS	mRNA	269992	271146	0.92	-	.	ID=MALK_01653.t1;Parent=MALK_01653
contig005	AUGUSTUS	exon	269992	271146	.	-	.	ID=MALK_01653.t1.e1;Parent=MALK_01653.t1
contig005	AUGUSTUS	gene	271249	273093	1	-	.	ID=MALK_01654;prediction_source=braker_MRET:g2331.t1
contig005	AUGUSTUS	CDS	271249	273093	1	-	0	ID=MALK_01654.t1.c1;Parent=MALK_01654.t1
contig005	AUGUSTUS	mRNA	271249	273093	1	-	.	ID=MALK_01654.t1;Parent=MALK_01654
contig005	AUGUSTUS	exon	271249	273093	.	-	.	ID=MALK_01654.t1.e1;Parent=MALK_01654.t1
contig005	AUGUSTUS	gene	273209	273854	0.41	+	.	ID=MALK_01655;prediction_source=braker_MRET:g2332.t1
contig005	AUGUSTUS	CDS	273209	273365	0.89	+	0	ID=MALK_01655.t1.c1;Parent=MALK_01655.t1
contig005	AUGUSTUS	CDS	273412	273548	0.89	+	0	ID=MALK_01655.t1.c2;Parent=MALK_01655.t1
contig005	AUGUSTUS	CDS	273576	273854	0.89	+	0	ID=MALK_01655.t1.c3;Parent=MALK_01655.t1
contig005	AUGUSTUS	mRNA	273209	273854	0.41	+	.	ID=MALK_01655.t1;Parent=MALK_01655
contig005	AUGUSTUS	exon	273209	273365	.	+	.	ID=MALK_01655.t1.e1;Parent=MALK_01655.t1
contig005	AUGUSTUS	exon	273412	273548	.	+	.	ID=MALK_01655.t1.e2;Parent=MALK_01655.t1
contig005	AUGUSTUS	exon	273576	273854	.	+	.	ID=MALK_01655.t1.e3;Parent=MALK_01655.t1
contig005	AUGUSTUS	gene	273872	274153	0.75	-	.	ID=MALK_01656;prediction_source=braker_MRET:g2333.t1
contig005	AUGUSTUS	CDS	274019	274153	0.77	-	0	ID=MALK_01656.t1.c2;Parent=MALK_01656.t1
contig005	AUGUSTUS	CDS	273872	273958	0.77	-	0	ID=MALK_01656.t1.c1;Parent=MALK_01656.t1
contig005	AUGUSTUS	mRNA	273872	274153	0.75	-	.	ID=MALK_01656.t1;Parent=MALK_01656
contig005	AUGUSTUS	exon	274019	274153	.	-	.	ID=MALK_01656.t1.e2;Parent=MALK_01656.t1
contig005	AUGUSTUS	exon	273872	273958	.	-	.	ID=MALK_01656.t1.e1;Parent=MALK_01656.t1
contig005	AUGUSTUS	gene	274222	275499	0.89	+	.	ID=MALK_01657;prediction_source=braker_MRET:g2334.t1
contig005	AUGUSTUS	CDS	274222	275499	0.89	+	0	ID=MALK_01657.t1.c1;Parent=MALK_01657.t1
contig005	AUGUSTUS	mRNA	274222	275499	0.89	+	.	ID=MALK_01657.t1;Parent=MALK_01657
contig005	AUGUSTUS	exon	274222	275499	.	+	.	ID=MALK_01657.t1.e1;Parent=MALK_01657.t1
contig005	AUGUSTUS	gene	275521	277663	0.5	-	.	ID=MALK_01658;prediction_source=braker_MRET:g2335.t1
contig005	AUGUSTUS	CDS	275561	277663	0.5	-	0	ID=MALK_01658.t1.c2;Parent=MALK_01658.t1
contig005	AUGUSTUS	CDS	275521	275526	0.5	-	0	ID=MALK_01658.t1.c1;Parent=MALK_01658.t1
contig005	AUGUSTUS	mRNA	275521	277663	0.5	-	.	ID=MALK_01658.t1;Parent=MALK_01658
contig005	AUGUSTUS	exon	275561	277663	.	-	.	ID=MALK_01658.t1.e2;Parent=MALK_01658.t1
contig005	AUGUSTUS	exon	275521	275526	.	-	.	ID=MALK_01658.t1.e1;Parent=MALK_01658.t1
contig005	maker	gene	277843	278922	.	+	.	ID=MALK_01659;prediction_source=maker_MRET:augustus_masked-contig005-processed-gene-1.52-mRNA-1
contig005	maker	CDS	277843	278922	.	+	0	ID=MALK_01659.t1.c1;Parent=MALK_01659.t1
contig005	maker	mRNA	277843	278922	.	+	.	ID=MALK_01659.t1;Parent=MALK_01659
contig005	maker	exon	277843	278922	.	+	.	ID=MALK_01659.t1.e1;Parent=MALK_01659.t1
contig005	AUGUSTUS	gene	278941	280464	0.82	-	.	ID=MALK_01660;prediction_source=augustus:contig005.g3717.t1
contig005	AUGUSTUS	CDS	278941	280464	0.82	-	0	ID=MALK_01660.t1.c1;Parent=MALK_01660.t1
contig005	AUGUSTUS	mRNA	278941	280464	0.82	-	.	ID=MALK_01660.t1;Parent=MALK_01660
contig005	AUGUSTUS	exon	278941	280464	0.82	-	.	ID=MALK_01660.t1.e1;Parent=MALK_01660.t1
contig005	AUGUSTUS	gene	280907	283879	0.98	+	.	ID=MALK_01661;prediction_source=braker_MRET:g2338.t1
contig005	AUGUSTUS	CDS	280907	283879	0.98	+	0	ID=MALK_01661.t1.c1;Parent=MALK_01661.t1

contig005	AUGUSTUS	mRNA	280907	283879	0.98	+	.	ID=MALK_01661.t1;Parent=MALK_01661
contig005	AUGUSTUS	exon	280907	283879	.	+	.	ID=MALK_01661.t1.e1;Parent=MALK_01661.t1
contig005	AUGUSTUS	gene	284462	287215	0.37	+	.	ID=MALK_01662;prediction_source=braker_MRET:g2339.t1
contig005	AUGUSTUS	CDS	284462	287215	0.37	+	0	ID=MALK_01662.t1.c1;Parent=MALK_01662.t1
contig005	AUGUSTUS	mRNA	284462	287215	0.37	+	.	ID=MALK_01662.t1;Parent=MALK_01662
contig005	AUGUSTUS	exon	284462	287215	.	+	.	ID=MALK_01662.t1.e1;Parent=MALK_01662.t1
contig005	AUGUSTUS	gene	288013	289611	0.11	+	.	ID=MALK_01663;prediction_source=braker_MRET:g2340.t1
contig005	AUGUSTUS	CDS	288013	288285	0.26	+	0	ID=MALK_01663.t1.c1;Parent=MALK_01663.t1
contig005	AUGUSTUS	CDS	288315	288788	0.26	+	0	ID=MALK_01663.t1.c2;Parent=MALK_01663.t1
contig005	AUGUSTUS	CDS	288817	289611	0.26	+	0	ID=MALK_01663.t1.c3;Parent=MALK_01663.t1
contig005	AUGUSTUS	mRNA	288013	289611	0.11	+	.	ID=MALK_01663.t1;Parent=MALK_01663
contig005	AUGUSTUS	exon	288013	288285	.	+	.	ID=MALK_01663.t1.e1;Parent=MALK_01663.t1
contig005	AUGUSTUS	exon	288315	288788	.	+	.	ID=MALK_01663.t1.e2;Parent=MALK_01663.t1
contig005	AUGUSTUS	exon	288817	289611	.	+	.	ID=MALK_01663.t1.e3;Parent=MALK_01663.t1
contig005	AUGUSTUS	gene	289693	290525	0.14	+	.	ID=MALK_01664;prediction_source=braker_MRET:g2341.t1
contig005	AUGUSTUS	CDS	289693	289700	0.4	+	0	ID=MALK_01664.t1.c1;Parent=MALK_01664.t1
contig005	AUGUSTUS	CDS	289728	289871	0.4	+	0	ID=MALK_01664.t1.c2;Parent=MALK_01664.t1
contig005	AUGUSTUS	CDS	289931	290525	0.4	+	0	ID=MALK_01664.t1.c3;Parent=MALK_01664.t1
contig005	AUGUSTUS	mRNA	289693	290525	0.14	+	.	ID=MALK_01664.t1;Parent=MALK_01664
contig005	AUGUSTUS	exon	289693	289700	.	+	.	ID=MALK_01664.t1.e1;Parent=MALK_01664.t1
contig005	AUGUSTUS	exon	289728	289871	.	+	.	ID=MALK_01664.t1.e2;Parent=MALK_01664.t1
contig005	AUGUSTUS	exon	289931	290525	.	+	.	ID=MALK_01664.t1.e3;Parent=MALK_01664.t1
contig005	AUGUSTUS	gene	290522	292015	0.58	-	.	ID=MALK_01665;prediction_source=augustus:contig005.g3723.t1
contig005	AUGUSTUS	CDS	290522	292015	0.58	-	0	ID=MALK_01665.t1.c1;Parent=MALK_01665.t1
contig005	AUGUSTUS	mRNA	290522	292015	0.58	-	.	ID=MALK_01665.t1;Parent=MALK_01665
contig005	AUGUSTUS	exon	290522	292015	0.58	-	.	ID=MALK_01665.t1.e1;Parent=MALK_01665.t1
contig005	AUGUSTUS	gene	292549	294344	0.69	+	.	ID=MALK_01666;prediction_source=braker_MRET:g2343.t1
contig005	AUGUSTUS	CDS	292549	292671	0.76	+	0	ID=MALK_01666.t1.c1;Parent=MALK_01666.t1
contig005	AUGUSTUS	CDS	292710	292804	0.76	+	0	ID=MALK_01666.t1.c2;Parent=MALK_01666.t1
contig005	AUGUSTUS	CDS	292838	294344	0.76	+	0	ID=MALK_01666.t1.c3;Parent=MALK_01666.t1
contig005	AUGUSTUS	mRNA	292549	294344	0.69	+	.	ID=MALK_01666.t1;Parent=MALK_01666
contig005	AUGUSTUS	exon	292549	292671	.	+	.	ID=MALK_01666.t1.e1;Parent=MALK_01666.t1
contig005	AUGUSTUS	exon	292710	292804	.	+	.	ID=MALK_01666.t1.e2;Parent=MALK_01666.t1
contig005	AUGUSTUS	exon	292838	294344	.	+	.	ID=MALK_01666.t1.e3;Parent=MALK_01666.t1
contig005	AUGUSTUS	gene	294609	295526	0.82	+	.	ID=MALK_01667;prediction_source=augustus:contig005.g3725.t1
contig005	AUGUSTUS	CDS	294609	295526	0.82	+	0	ID=MALK_01667.t1.c1;Parent=MALK_01667.t1
contig005	AUGUSTUS	mRNA	294609	295526	0.82	+	.	ID=MALK_01667.t1;Parent=MALK_01667
contig005	AUGUSTUS	exon	294609	295526	0.82	+	.	ID=MALK_01667.t1.e1;Parent=MALK_01667.t1
contig005	AUGUSTUS	gene	295697	298276	0.84	+	.	ID=MALK_01668;prediction_source=braker_MRET:g2345.t1
contig005	AUGUSTUS	CDS	295697	298276	0.84	+	0	ID=MALK_01668.t1.c1;Parent=MALK_01668.t1
contig005	AUGUSTUS	mRNA	295697	298276	0.84	+	.	ID=MALK_01668.t1;Parent=MALK_01668
contig005	AUGUSTUS	exon	295697	298276	.	+	.	ID=MALK_01668.t1.e1;Parent=MALK_01668.t1
contig006	annotation	remark	1	843590	.	+	.	gff-version=3

contig006	AUGUSTUS	gene	247	1578	1	-	.	ID=MALK_01669;prediction_source=augustus:contig006.g1933.t1
contig006	AUGUSTUS	CDS	247	1578	1	-	0	ID=MALK_01669.t1.c1;Parent=MALK_01669.t1
contig006	AUGUSTUS	mRNA	247	1578	1	-	.	ID=MALK_01669.t1;Parent=MALK_01669
contig006	AUGUSTUS	exon	247	1578	1	-	.	ID=MALK_01669.t1.e1;Parent=MALK_01669.t1
contig006	AUGUSTUS	gene	1760	2293	1	+	.	ID=MALK_01670;prediction_source=braker_MRET:g1681.t1
contig006	AUGUSTUS	CDS	1760	1873	1	+	0	ID=MALK_01670.t1.c1;Parent=MALK_01670.t1
contig006	AUGUSTUS	CDS	1916	2293	1	+	0	ID=MALK_01670.t1.c2;Parent=MALK_01670.t1
contig006	AUGUSTUS	mRNA	1760	2293	1	+	.	ID=MALK_01670.t1;Parent=MALK_01670
contig006	AUGUSTUS	exon	1760	1873	.	+	.	ID=MALK_01670.t1.e1;Parent=MALK_01670.t1
contig006	AUGUSTUS	exon	1916	2293	.	+	.	ID=MALK_01670.t1.e2;Parent=MALK_01670.t1
contig006	AUGUSTUS	gene	2652	5509	0.5	-	.	ID=MALK_01671;prediction_source=braker_MRET:g1682.t1
contig006	AUGUSTUS	CDS	5371	5509	0.5	-	0	ID=MALK_01671.t1.c2;Parent=MALK_01671.t1
contig006	AUGUSTUS	CDS	2652	5242	0.5	-	0	ID=MALK_01671.t1.c1;Parent=MALK_01671.t1
contig006	AUGUSTUS	mRNA	2652	5509	0.5	-	.	ID=MALK_01671.t1;Parent=MALK_01671
contig006	AUGUSTUS	exon	5371	5509	.	-	.	ID=MALK_01671.t1.e2;Parent=MALK_01671.t1
contig006	AUGUSTUS	exon	2652	5242	.	-	.	ID=MALK_01671.t1.e1;Parent=MALK_01671.t1
contig006	AUGUSTUS	gene	5889	6718	0.44	-	.	ID=MALK_01672;prediction_source=braker_MRET:g1683.t1
contig006	AUGUSTUS	CDS	5999	6718	0.9	-	0	ID=MALK_01672.t1.c2;Parent=MALK_01672.t1
contig006	AUGUSTUS	CDS	5889	5963	0.9	-	0	ID=MALK_01672.t1.c1;Parent=MALK_01672.t1
contig006	AUGUSTUS	mRNA	5889	6718	0.44	-	.	ID=MALK_01672.t1;Parent=MALK_01672
contig006	AUGUSTUS	exon	5999	6718	.	-	.	ID=MALK_01672.t1.e2;Parent=MALK_01672.t1
contig006	AUGUSTUS	exon	5889	5963	.	-	.	ID=MALK_01672.t1.e1;Parent=MALK_01672.t1
contig006	maker	gene	6863	8854	.	+	.	ID=MALK_01673;prediction_source=maker_MRET:augustus_masked-contig006-processed-gene-0.1-mRNA-1
contig006	maker	CDS	6863	8854	.	+	0	ID=MALK_01673.t1.c1;Parent=MALK_01673.t1
contig006	maker	mRNA	6863	8854	.	+	.	ID=MALK_01673.t1;Parent=MALK_01673
contig006	maker	exon	6863	8854	.	+	.	ID=MALK_01673.t1.e1;Parent=MALK_01673.t1
contig006	maker	gene	8863	10152	.	-	.	ID=MALK_01674;prediction_source=maker_MRET:augustus_masked-contig006-processed-gene-0.26-mRNA-1
contig006	maker	CDS	8863	10152	.	-	0	ID=MALK_01674.t1.c1;Parent=MALK_01674.t1
contig006	maker	mRNA	8863	10152	.	-	.	ID=MALK_01674.t1;Parent=MALK_01674
contig006	maker	exon	8863	10152	.	-	.	ID=MALK_01674.t1.e1;Parent=MALK_01674.t1
contig006	AUGUSTUS	gene	10347	12101	0.23	+	.	ID=MALK_01675;prediction_source=braker_MRET:g1686.t1
contig006	AUGUSTUS	CDS	10347	10574	0.66	+	0	ID=MALK_01675.t1.c1;Parent=MALK_01675.t1
contig006	AUGUSTUS	CDS	10607	10737	0.66	+	0	ID=MALK_01675.t1.c2;Parent=MALK_01675.t1
contig006	AUGUSTUS	CDS	10769	10934	0.66	+	0	ID=MALK_01675.t1.c3;Parent=MALK_01675.t1
contig006	AUGUSTUS	CDS	11103	12101	0.66	+	0	ID=MALK_01675.t1.c4;Parent=MALK_01675.t1
contig006	AUGUSTUS	mRNA	10347	12101	0.23	+	.	ID=MALK_01675.t1;Parent=MALK_01675
contig006	AUGUSTUS	exon	10347	10574	.	+	.	ID=MALK_01675.t1.e1;Parent=MALK_01675.t1
contig006	AUGUSTUS	exon	10607	10737	.	+	.	ID=MALK_01675.t1.e2;Parent=MALK_01675.t1
contig006	AUGUSTUS	exon	10769	10934	.	+	.	ID=MALK_01675.t1.e3;Parent=MALK_01675.t1
contig006	AUGUSTUS	exon	11103	12101	.	+	.	ID=MALK_01675.t1.e4;Parent=MALK_01675.t1
contig006	maker	gene	12106	14370	.	-	.	ID=MALK_01676;prediction_source=maker_MRET:augustus_masked-contig006-processed-gene-0.27-mRNA-1
contig006	maker	CDS	12106	14370	.	-	0	ID=MALK_01676.t1.c1;Parent=MALK_01676.t1
contig006	maker	mRNA	12106	14370	.	-	.	ID=MALK_01676.t1;Parent=MALK_01676

contig006	maker	exon	12106	14370	.	-	.	ID=MALK_01676.t1.e1;Parent=MALK_01676.t1
contig006	maker	gene	15114	16247	.	+	.	ID=MALK_01677;prediction_source=maker_MRET:augustus_masked-contig006-processed-gene-0.2-mRNA-1
contig006	maker	CDS	15114	16247	.	+	0	ID=MALK_01677.t1.c1;Parent=MALK_01677.t1
contig006	maker	mRNA	15114	16247	.	+	.	ID=MALK_01677.t1;Parent=MALK_01677
contig006	maker	exon	15114	16247	.	+	.	ID=MALK_01677.t1.e1;Parent=MALK_01677.t1
contig006	AUGUSTUS	gene	16288	19608	0.85	+	.	ID=MALK_01678;prediction_source=augustus:contig006.g1942.t1
contig006	AUGUSTUS	CDS	16288	19608	0.85	+	0	ID=MALK_01678.t1.c1;Parent=MALK_01678.t1
contig006	AUGUSTUS	mRNA	16288	19608	0.85	+	.	ID=MALK_01678.t1;Parent=MALK_01678
contig006	AUGUSTUS	exon	16288	19608	0.85	+	.	ID=MALK_01678.t1.e1;Parent=MALK_01678.t1
contig006	AUGUSTUS	gene	19610	20428	0.99	-	.	ID=MALK_01679;prediction_source=braker_MRET:g1690.t1
contig006	AUGUSTUS	CDS	19610	20428	0.99	-	0	ID=MALK_01679.t1.c1;Parent=MALK_01679.t1
contig006	AUGUSTUS	mRNA	19610	20428	0.99	-	.	ID=MALK_01679.t1;Parent=MALK_01679
contig006	AUGUSTUS	exon	19610	20428	.	-	.	ID=MALK_01679.t1.e1;Parent=MALK_01679.t1
contig006	AUGUSTUS	gene	20462	22780	0.33	-	.	ID=MALK_01680;prediction_source=braker_MRET:g1691.t1
contig006	AUGUSTUS	CDS	20462	22780	0.33	-	0	ID=MALK_01680.t1.c1;Parent=MALK_01680.t1
contig006	AUGUSTUS	mRNA	20462	22780	0.33	-	.	ID=MALK_01680.t1;Parent=MALK_01680
contig006	AUGUSTUS	exon	20462	22780	.	-	.	ID=MALK_01680.t1.e1;Parent=MALK_01680.t1
contig006	maker	gene	22861	25971	.	+	.	ID=MALK_01681;prediction_source=maker_MRET:augustus_masked-contig006-processed-gene-0.4-mRNA-1
contig006	maker	CDS	22861	25971	.	+	0	ID=MALK_01681.t1.c1;Parent=MALK_01681.t1
contig006	maker	mRNA	22861	25971	.	+	.	ID=MALK_01681.t1;Parent=MALK_01681
contig006	maker	exon	22861	25971	.	+	.	ID=MALK_01681.t1.e1;Parent=MALK_01681.t1
contig006	AUGUSTUS	gene	26065	28520	0.8	+	.	ID=MALK_01682;prediction_source=braker_MRET:g1693.t1
contig006	AUGUSTUS	CDS	26065	26365	1	+	0	ID=MALK_01682.t1.c1;Parent=MALK_01682.t1
contig006	AUGUSTUS	CDS	26398	28520	1	+	0	ID=MALK_01682.t1.c2;Parent=MALK_01682.t1
contig006	AUGUSTUS	mRNA	26065	28520	0.8	+	.	ID=MALK_01682.t1;Parent=MALK_01682
contig006	AUGUSTUS	exon	26065	26365	.	+	.	ID=MALK_01682.t1.e1;Parent=MALK_01682.t1
contig006	AUGUSTUS	exon	26398	28520	.	+	.	ID=MALK_01682.t1.e2;Parent=MALK_01682.t1
contig006	AUGUSTUS	gene	28568	30742	0.74	-	.	ID=MALK_01683;prediction_source=augustus:contig006.g1947.t1
contig006	AUGUSTUS	CDS	28568	30742	0.74	-	0	ID=MALK_01683.t1.c1;Parent=MALK_01683.t1
contig006	AUGUSTUS	mRNA	28568	30742	0.74	-	.	ID=MALK_01683.t1;Parent=MALK_01683
contig006	AUGUSTUS	exon	28568	30742	0.74	-	.	ID=MALK_01683.t1.e1;Parent=MALK_01683.t1
contig006	AUGUSTUS	gene	32301	34787	0.47	+	.	ID=MALK_01684;prediction_source=braker_MRET:g1695.t1
contig006	AUGUSTUS	CDS	32301	32411	0.96	+	0	ID=MALK_01684.t1.c1;Parent=MALK_01684.t1
contig006	AUGUSTUS	CDS	32442	34787	0.96	+	0	ID=MALK_01684.t1.c2;Parent=MALK_01684.t1
contig006	AUGUSTUS	mRNA	32301	34787	0.47	+	.	ID=MALK_01684.t1;Parent=MALK_01684
contig006	AUGUSTUS	exon	32301	32411	.	+	.	ID=MALK_01684.t1.e1;Parent=MALK_01684.t1
contig006	AUGUSTUS	exon	32442	34787	.	+	.	ID=MALK_01684.t1.e2;Parent=MALK_01684.t1
contig006	maker	gene	34884	36764	.	+	.	ID=MALK_01685;prediction_source=maker_MRET:augustus_masked-contig006-processed-gene-0.8-mRNA-1
contig006	maker	CDS	34884	36764	.	+	0	ID=MALK_01685.t1.c1;Parent=MALK_01685.t1
contig006	maker	mRNA	34884	36764	.	+	.	ID=MALK_01685.t1;Parent=MALK_01685
contig006	maker	exon	34884	36764	.	+	.	ID=MALK_01685.t1.e1;Parent=MALK_01685.t1
contig006	AUGUSTUS	gene	37655	38736	1	-	.	ID=MALK_01686;prediction_source=braker_MRET:g1697.t1
contig006	AUGUSTUS	CDS	38602	38736	1	-	0	ID=MALK_01686.t1.c2;Parent=MALK_01686.t1

contig006	AUGUSTUS	CDS	37655	38560	1	-	0	ID=MALK_01686.t1.c1;Parent=MALK_01686.t1
contig006	AUGUSTUS	mRNA	37655	38736	1	-	.	ID=MALK_01686.t1;Parent=MALK_01686
contig006	AUGUSTUS	exon	38602	38736	.	-	.	ID=MALK_01686.t1.e2;Parent=MALK_01686.t1
contig006	AUGUSTUS	exon	37655	38560	.	-	.	ID=MALK_01686.t1.e1;Parent=MALK_01686.t1
contig006	maker	gene	38887	40716	.	+	.	ID=MALK_01687;prediction_source=maker_MRET:augustus_masked-contig006-processed-gene-0.9-mRNA-1
contig006	maker	CDS	38887	40716	.	+	0	ID=MALK_01687.t1.c1;Parent=MALK_01687.t1
contig006	maker	mRNA	38887	40716	.	+	.	ID=MALK_01687.t1;Parent=MALK_01687
contig006	maker	exon	38887	40716	.	+	.	ID=MALK_01687.t1.e1;Parent=MALK_01687.t1
contig006	AUGUSTUS	gene	40821	41504	0.56	+	.	ID=MALK_01688;prediction_source=augustus:contig006.g1953.t1
contig006	AUGUSTUS	CDS	40821	41504	0.56	+	0	ID=MALK_01688.t1.c1;Parent=MALK_01688.t1
contig006	AUGUSTUS	mRNA	40821	41504	0.56	+	.	ID=MALK_01688.t1;Parent=MALK_01688
contig006	AUGUSTUS	exon	40821	41504	0.56	+	.	ID=MALK_01688.t1.e1;Parent=MALK_01688.t1
contig006	maker	gene	41505	44720	.	-	.	ID=MALK_01689;prediction_source=maker_MRET:augustus_masked-contig006-processed-gene-0.31-mRNA-1
contig006	maker	CDS	41505	44720	.	-	0	ID=MALK_01689.t1.c1;Parent=MALK_01689.t1
contig006	maker	mRNA	41505	44720	.	-	.	ID=MALK_01689.t1;Parent=MALK_01689
contig006	maker	exon	41505	44720	.	-	.	ID=MALK_01689.t1.e1;Parent=MALK_01689.t1
contig006	AUGUSTUS	gene	44756	47518	0.96	+	.	ID=MALK_01690;prediction_source=augustus:contig006.g1955.t1
contig006	AUGUSTUS	CDS	44756	47518	0.96	+	0	ID=MALK_01690.t1.c1;Parent=MALK_01690.t1
contig006	AUGUSTUS	mRNA	44756	47518	0.96	+	.	ID=MALK_01690.t1;Parent=MALK_01690
contig006	AUGUSTUS	exon	44756	47518	0.96	+	.	ID=MALK_01690.t1.e1;Parent=MALK_01690.t1
contig006	AUGUSTUS	gene	47520	49127	0.95	-	.	ID=MALK_01691;prediction_source=augustus:contig006.g1956.t1
contig006	AUGUSTUS	CDS	47520	49127	0.95	-	0	ID=MALK_01691.t1.c1;Parent=MALK_01691.t1
contig006	AUGUSTUS	mRNA	47520	49127	0.95	-	.	ID=MALK_01691.t1;Parent=MALK_01691
contig006	AUGUSTUS	exon	47520	49127	0.95	-	.	ID=MALK_01691.t1.e1;Parent=MALK_01691.t1
contig006	AUGUSTUS	gene	49705	50600	0.55	-	.	ID=MALK_01692;prediction_source=augustus:contig006.g1958.t1
contig006	AUGUSTUS	CDS	50516	50600	0.55	-	0	ID=MALK_01692.t1.c2;Parent=MALK_01692.t1
contig006	AUGUSTUS	CDS	49705	50441	0.55	-	0	ID=MALK_01692.t1.c1;Parent=MALK_01692.t1
contig006	AUGUSTUS	mRNA	49705	50600	0.55	-	.	ID=MALK_01692.t1;Parent=MALK_01692
contig006	AUGUSTUS	exon	50516	50600	0.55	-	.	ID=MALK_01692.t1.e2;Parent=MALK_01692.t1
contig006	AUGUSTUS	exon	49705	50441	0.55	-	.	ID=MALK_01692.t1.e1;Parent=MALK_01692.t1
contig006	AUGUSTUS	gene	50780	52846	1	+	.	ID=MALK_01693;prediction_source=augustus:contig006.g1959.t1
contig006	AUGUSTUS	CDS	50780	52846	1	+	0	ID=MALK_01693.t1.c1;Parent=MALK_01693.t1
contig006	AUGUSTUS	mRNA	50780	52846	1	+	.	ID=MALK_01693.t1;Parent=MALK_01693
contig006	AUGUSTUS	exon	50780	52846	1	+	.	ID=MALK_01693.t1.e1;Parent=MALK_01693.t1
contig006	maker	gene	52856	53929	.	-	.	ID=MALK_01694;prediction_source=maker_MRET:augustus_masked-contig006-processed-gene-0.34-mRNA-1
contig006	maker	CDS	52856	53929	.	-	0	ID=MALK_01694.t1.c1;Parent=MALK_01694.t1
contig006	maker	mRNA	52856	53929	.	-	.	ID=MALK_01694.t1;Parent=MALK_01694
contig006	maker	exon	52856	53929	.	-	.	ID=MALK_01694.t1.e1;Parent=MALK_01694.t1
contig006	AUGUSTUS	gene	54268	57513	1	+	.	ID=MALK_01695;prediction_source=augustus:contig006.g1961.t1
contig006	AUGUSTUS	CDS	54268	57513	1	+	0	ID=MALK_01695.t1.c1;Parent=MALK_01695.t1
contig006	AUGUSTUS	mRNA	54268	57513	1	+	.	ID=MALK_01695.t1;Parent=MALK_01695
contig006	AUGUSTUS	exon	54268	57513	1	+	.	ID=MALK_01695.t1.e1;Parent=MALK_01695.t1
contig006	AUGUSTUS	gene	58409	59527	0.83	-	.	ID=MALK_01696;prediction_source=augustus:contig006.g1962.t1

contig006	AUGUSTUS	CDS	58409	59527	0.83	-	0	ID=MALK_01696.t1.c1;Parent=MALK_01696.t1
contig006	AUGUSTUS	mRNA	58409	59527	0.83	-	.	ID=MALK_01696.t1;Parent=MALK_01696
contig006	AUGUSTUS	exon	58409	59527	0.83	-	.	ID=MALK_01696.t1.e1;Parent=MALK_01696.t1
contig006	AUGUSTUS	gene	59660	60328	0.19	+	.	ID=MALK_01697;prediction_source=braker_MRET:g1707.t1
contig006	AUGUSTUS	CDS	59660	60264	0.56	+	0	ID=MALK_01697.t1.c1;Parent=MALK_01697.t1
contig006	AUGUSTUS	CDS	60295	60328	0.56	+	0	ID=MALK_01697.t1.c2;Parent=MALK_01697.t1
contig006	AUGUSTUS	mRNA	59660	60328	0.19	+	.	ID=MALK_01697.t1;Parent=MALK_01697
contig006	AUGUSTUS	exon	59660	60264	.	+	.	ID=MALK_01697.t1.e1;Parent=MALK_01697.t1
contig006	AUGUSTUS	exon	60295	60328	.	+	.	ID=MALK_01697.t1.e2;Parent=MALK_01697.t1
contig006	maker	gene	60480	62660	.	+	.	ID=MALK_01698;prediction_source=maker_MRET:augustus_masked-contig006-processed-gene-0.13-mRNA-1
contig006	maker	CDS	60480	62660	.	+	0	ID=MALK_01698.t1.c1;Parent=MALK_01698.t1
contig006	maker	mRNA	60480	62660	.	+	.	ID=MALK_01698.t1;Parent=MALK_01698
contig006	maker	exon	60480	62660	.	+	.	ID=MALK_01698.t1.e1;Parent=MALK_01698.t1
contig006	AUGUSTUS	gene	62894	63607	0.99	-	.	ID=MALK_01699;prediction_source=augustus:contig006.g1965.t1
contig006	AUGUSTUS	CDS	62894	63607	0.99	-	0	ID=MALK_01699.t1.c1;Parent=MALK_01699.t1
contig006	AUGUSTUS	mRNA	62894	63607	0.99	-	.	ID=MALK_01699.t1;Parent=MALK_01699
contig006	AUGUSTUS	exon	62894	63607	0.99	-	.	ID=MALK_01699.t1.e1;Parent=MALK_01699.t1
contig006	maker	gene	64069	65643	.	+	.	ID=MALK_01700;prediction_source=maker_MRET:augustus_masked-contig006-processed-gene-0.14-mRNA-1
contig006	maker	CDS	64069	65643	.	+	0	ID=MALK_01700.t1.c1;Parent=MALK_01700.t1
contig006	maker	mRNA	64069	65643	.	+	.	ID=MALK_01700.t1;Parent=MALK_01700
contig006	maker	exon	64069	65643	.	+	.	ID=MALK_01700.t1.e1;Parent=MALK_01700.t1
contig006	AUGUSTUS	gene	65773	67104	0.31	+	.	ID=MALK_01701;prediction_source=augustus:contig006.g1967.t1
contig006	AUGUSTUS	CDS	65773	67104	0.31	+	0	ID=MALK_01701.t1.c1;Parent=MALK_01701.t1
contig006	AUGUSTUS	mRNA	65773	67104	0.31	+	.	ID=MALK_01701.t1;Parent=MALK_01701
contig006	AUGUSTUS	exon	65773	67104	0.31	+	.	ID=MALK_01701.t1.e1;Parent=MALK_01701.t1
contig006	AUGUSTUS	gene	67256	67706	0.61	+	.	ID=MALK_01702;prediction_source=braker_MRET:g1712.t1
contig006	AUGUSTUS	CDS	67256	67389	0.98	+	0	ID=MALK_01702.t1.c1;Parent=MALK_01702.t1
contig006	AUGUSTUS	CDS	67440	67556	0.98	+	0	ID=MALK_01702.t1.c2;Parent=MALK_01702.t1
contig006	AUGUSTUS	CDS	67592	67706	0.98	+	0	ID=MALK_01702.t1.c3;Parent=MALK_01702.t1
contig006	AUGUSTUS	mRNA	67256	67706	0.61	+	.	ID=MALK_01702.t1;Parent=MALK_01702
contig006	AUGUSTUS	exon	67256	67389	.	+	.	ID=MALK_01702.t1.e1;Parent=MALK_01702.t1
contig006	AUGUSTUS	exon	67440	67556	.	+	.	ID=MALK_01702.t1.e2;Parent=MALK_01702.t1
contig006	AUGUSTUS	exon	67592	67706	.	+	.	ID=MALK_01702.t1.e3;Parent=MALK_01702.t1
contig006	AUGUSTUS	gene	67749	71255	0.66	-	.	ID=MALK_01703;prediction_source=braker_MRET:g1713.t1
contig006	AUGUSTUS	CDS	67749	71255	0.66	-	0	ID=MALK_01703.t1.c1;Parent=MALK_01703.t1
contig006	AUGUSTUS	mRNA	67749	71255	0.66	-	.	ID=MALK_01703.t1;Parent=MALK_01703
contig006	AUGUSTUS	exon	67749	71255	.	-	.	ID=MALK_01703.t1.e1;Parent=MALK_01703.t1
contig006	AUGUSTUS	gene	71488	72369	0.42	+	.	ID=MALK_01704;prediction_source=augustus:contig006.g1971.t1
contig006	AUGUSTUS	CDS	71488	72369	0.42	+	0	ID=MALK_01704.t1.c1;Parent=MALK_01704.t1
contig006	AUGUSTUS	mRNA	71488	72369	0.42	+	.	ID=MALK_01704.t1;Parent=MALK_01704
contig006	AUGUSTUS	exon	71488	72369	0.42	+	.	ID=MALK_01704.t1.e1;Parent=MALK_01704.t1
contig006	AUGUSTUS	gene	72394	72864	0.68	-	.	ID=MALK_01705;prediction_source=augustus:contig006.g1973.t1
contig006	AUGUSTUS	CDS	72394	72864	0.68	-	0	ID=MALK_01705.t1.c1;Parent=MALK_01705.t1

contig006	AUGUSTUS	mRNA	72394	72864	0.68	-	.	ID=MALK_01705.t1;Parent=MALK_01705
contig006	AUGUSTUS	exon	72394	72864	0.68	-	.	ID=MALK_01705.t1.e1;Parent=MALK_01705.t1
contig006	AUGUSTUS	gene	73288	73752	0.9	-	.	ID=MALK_01706;prediction_source=augustus:contig006.g1975.t1
contig006	AUGUSTUS	CDS	73288	73752	0.9	-	0	ID=MALK_01706.t1.c1;Parent=MALK_01706.t1
contig006	AUGUSTUS	mRNA	73288	73752	0.9	-	.	ID=MALK_01706.t1;Parent=MALK_01706
contig006	AUGUSTUS	exon	73288	73752	0.9	-	.	ID=MALK_01706.t1.e1;Parent=MALK_01706.t1
contig006	AUGUSTUS	gene	73945	74411	0.99	+	.	ID=MALK_01707;prediction_source=braker_MRET:g1717.t1
contig006	AUGUSTUS	CDS	73945	74172	1	+	0	ID=MALK_01707.t1.c1;Parent=MALK_01707.t1
contig006	AUGUSTUS	CDS	74259	74317	1	+	0	ID=MALK_01707.t1.c2;Parent=MALK_01707.t1
contig006	AUGUSTUS	CDS	74357	74411	1	+	0	ID=MALK_01707.t1.c3;Parent=MALK_01707.t1
contig006	AUGUSTUS	mRNA	73945	74411	0.99	+	.	ID=MALK_01707.t1;Parent=MALK_01707
contig006	AUGUSTUS	exon	73945	74172	.	+	.	ID=MALK_01707.t1.e1;Parent=MALK_01707.t1
contig006	AUGUSTUS	exon	74259	74317	.	+	.	ID=MALK_01707.t1.e2;Parent=MALK_01707.t1
contig006	AUGUSTUS	exon	74357	74411	.	+	.	ID=MALK_01707.t1.e3;Parent=MALK_01707.t1
contig006	maker	gene	74803	76200	.	+	.	ID=MALK_01708;prediction_source=maker_MRET:augustus_masked-contig006-processed-gene-0.18-mRNA-1
contig006	maker	CDS	74803	76200	.	+	0	ID=MALK_01708.t1.c1;Parent=MALK_01708.t1
contig006	maker	mRNA	74803	76200	.	+	.	ID=MALK_01708.t1;Parent=MALK_01708
contig006	maker	exon	74803	76200	.	+	.	ID=MALK_01708.t1.e1;Parent=MALK_01708.t1
contig006	AUGUSTUS	gene	76212	77636	0.49	-	.	ID=MALK_01709;prediction_source=augustus:contig006.g1977.t1
contig006	AUGUSTUS	CDS	76212	77636	0.49	-	0	ID=MALK_01709.t1.c1;Parent=MALK_01709.t1
contig006	AUGUSTUS	mRNA	76212	77636	0.49	-	.	ID=MALK_01709.t1;Parent=MALK_01709
contig006	AUGUSTUS	exon	76212	77636	0.49	-	.	ID=MALK_01709.t1.e1;Parent=MALK_01709.t1
contig006	maker	gene	77803	79134	.	+	.	ID=MALK_01710;prediction_source=maker_MRET:augustus_masked-contig006-processed-gene-0.19-mRNA-1
contig006	maker	CDS	77803	79134	.	+	0	ID=MALK_01710.t1.c1;Parent=MALK_01710.t1
contig006	maker	mRNA	77803	79134	.	+	.	ID=MALK_01710.t1;Parent=MALK_01710
contig006	maker	exon	77803	79134	.	+	.	ID=MALK_01710.t1.e1;Parent=MALK_01710.t1
contig006	AUGUSTUS	gene	79525	80328	0.76	+	.	ID=MALK_01711;prediction_source=braker_MRET:g1721.t1
contig006	AUGUSTUS	CDS	79525	80328	0.76	+	0	ID=MALK_01711.t1.c1;Parent=MALK_01711.t1
contig006	AUGUSTUS	mRNA	79525	80328	0.76	+	.	ID=MALK_01711.t1;Parent=MALK_01711
contig006	AUGUSTUS	exon	79525	80328	.	+	.	ID=MALK_01711.t1.e1;Parent=MALK_01711.t1
contig006	AUGUSTUS	gene	80862	82704	0.66	+	.	ID=MALK_01712;prediction_source=braker_MRET:g1722.t1
contig006	AUGUSTUS	CDS	80862	80916	0.83	+	0	ID=MALK_01712.t1.c1;Parent=MALK_01712.t1
contig006	AUGUSTUS	CDS	80964	81056	0.83	+	0	ID=MALK_01712.t1.c2;Parent=MALK_01712.t1
contig006	AUGUSTUS	CDS	81086	81178	0.83	+	0	ID=MALK_01712.t1.c3;Parent=MALK_01712.t1
contig006	AUGUSTUS	CDS	81207	81423	0.83	+	0	ID=MALK_01712.t1.c4;Parent=MALK_01712.t1
contig006	AUGUSTUS	CDS	81453	81510	0.83	+	0	ID=MALK_01712.t1.c5;Parent=MALK_01712.t1
contig006	AUGUSTUS	CDS	81549	81570	0.83	+	0	ID=MALK_01712.t1.c6;Parent=MALK_01712.t1
contig006	AUGUSTUS	CDS	81623	82704	0.83	+	0	ID=MALK_01712.t1.c7;Parent=MALK_01712.t1
contig006	AUGUSTUS	mRNA	80862	82704	0.66	+	.	ID=MALK_01712.t1;Parent=MALK_01712
contig006	AUGUSTUS	exon	80862	80916	.	+	.	ID=MALK_01712.t1.e1;Parent=MALK_01712.t1
contig006	AUGUSTUS	exon	80964	81056	.	+	.	ID=MALK_01712.t1.e2;Parent=MALK_01712.t1
contig006	AUGUSTUS	exon	81086	81178	.	+	.	ID=MALK_01712.t1.e3;Parent=MALK_01712.t1
contig006	AUGUSTUS	exon	81207	81423	.	+	.	ID=MALK_01712.t1.e4;Parent=MALK_01712.t1

contig006	AUGUSTUS	exon	81453	81510	.	+	.	ID=MALK_01712.t1.e5;Parent=MALK_01712.t1
contig006	AUGUSTUS	exon	81549	81570	.	+	.	ID=MALK_01712.t1.e6;Parent=MALK_01712.t1
contig006	AUGUSTUS	exon	81623	82704	.	+	.	ID=MALK_01712.t1.e7;Parent=MALK_01712.t1
contig006	AUGUSTUS	gene	82676	83611	0.76	-	.	ID=MALK_01713;prediction_source=augustus:contig006.g1981.t1
contig006	AUGUSTUS	CDS	82676	83611	0.76	-	0	ID=MALK_01713.t1.c1;Parent=MALK_01713.t1
contig006	AUGUSTUS	mRNA	82676	83611	0.76	-	.	ID=MALK_01713.t1;Parent=MALK_01713
contig006	AUGUSTUS	exon	82676	83611	0.76	-	.	ID=MALK_01713.t1.e1;Parent=MALK_01713.t1
contig006	AUGUSTUS	gene	83682	84425	0.98	-	.	ID=MALK_01714;prediction_source=augustus:contig006.g1982.t1
contig006	AUGUSTUS	CDS	83682	84425	0.98	-	0	ID=MALK_01714.t1.c1;Parent=MALK_01714.t1
contig006	AUGUSTUS	mRNA	83682	84425	0.98	-	.	ID=MALK_01714.t1;Parent=MALK_01714
contig006	AUGUSTUS	exon	83682	84425	0.98	-	.	ID=MALK_01714.t1.e1;Parent=MALK_01714.t1
contig006	AUGUSTUS	gene	84461	85276	0.89	+	.	ID=MALK_01715;prediction_source=augustus:contig006.g1983.t1
contig006	AUGUSTUS	CDS	84461	85276	0.89	+	0	ID=MALK_01715.t1.c1;Parent=MALK_01715.t1
contig006	AUGUSTUS	mRNA	84461	85276	0.89	+	.	ID=MALK_01715.t1;Parent=MALK_01715
contig006	AUGUSTUS	exon	84461	85276	0.89	+	.	ID=MALK_01715.t1.e1;Parent=MALK_01715.t1
contig006	maker	gene	85273	86295	.	-	.	ID=MALK_01716;prediction_source=maker_MRET:augustus_masked-contig006-processed-gene-0.42-mRNA-1
contig006	maker	CDS	85273	86295	.	-	0	ID=MALK_01716.t1.c1;Parent=MALK_01716.t1
contig006	maker	mRNA	85273	86295	.	-	.	ID=MALK_01716.t1;Parent=MALK_01716
contig006	maker	exon	85273	86295	.	-	.	ID=MALK_01716.t1.e1;Parent=MALK_01716.t1
contig006	AUGUSTUS	gene	86369	87445	0.24	+	.	ID=MALK_01717;prediction_source=augustus:contig006.g1985.t1
contig006	AUGUSTUS	CDS	86369	87445	0.24	+	0	ID=MALK_01717.t1.c1;Parent=MALK_01717.t1
contig006	AUGUSTUS	mRNA	86369	87445	0.24	+	.	ID=MALK_01717.t1;Parent=MALK_01717
contig006	AUGUSTUS	exon	86369	87445	0.24	+	.	ID=MALK_01717.t1.e1;Parent=MALK_01717.t1
contig006	AUGUSTUS	gene	87433	88530	0.95	-	.	ID=MALK_01718;prediction_source=augustus:contig006.g1986.t1
contig006	AUGUSTUS	CDS	88491	88530	0.96	-	0	ID=MALK_01718.t1.c2;Parent=MALK_01718.t1
contig006	AUGUSTUS	CDS	87433	88430	0.96	-	0	ID=MALK_01718.t1.c1;Parent=MALK_01718.t1
contig006	AUGUSTUS	mRNA	87433	88530	0.95	-	.	ID=MALK_01718.t1;Parent=MALK_01718
contig006	AUGUSTUS	exon	88491	88530	0.96	-	.	ID=MALK_01718.t1.e2;Parent=MALK_01718.t1
contig006	AUGUSTUS	exon	87433	88430	0.96	-	.	ID=MALK_01718.t1.e1;Parent=MALK_01718.t1
contig006	maker	gene	88692	89453	.	+	.	ID=MALK_01719;prediction_source=maker_MRET:augustus_masked-contig006-processed-gene-0.22-mRNA-1
contig006	maker	CDS	88692	89453	.	+	0	ID=MALK_01719.t1.c1;Parent=MALK_01719.t1
contig006	maker	mRNA	88692	89453	.	+	.	ID=MALK_01719.t1;Parent=MALK_01719
contig006	maker	exon	88692	89453	.	+	.	ID=MALK_01719.t1.e1;Parent=MALK_01719.t1
contig006	AUGUSTUS	gene	89491	90483	0.94	-	.	ID=MALK_01720;prediction_source=augustus:contig006.g1988.t1
contig006	AUGUSTUS	CDS	89491	90483	0.94	-	0	ID=MALK_01720.t1.c1;Parent=MALK_01720.t1
contig006	AUGUSTUS	mRNA	89491	90483	0.94	-	.	ID=MALK_01720.t1;Parent=MALK_01720
contig006	AUGUSTUS	exon	89491	90483	0.94	-	.	ID=MALK_01720.t1.e1;Parent=MALK_01720.t1
contig006	AUGUSTUS	gene	90950	91339	0.89	-	.	ID=MALK_01721;prediction_source=augustus:contig006.g1989.t1
contig006	AUGUSTUS	CDS	90950	91339	0.89	-	0	ID=MALK_01721.t1.c1;Parent=MALK_01721.t1
contig006	AUGUSTUS	mRNA	90950	91339	0.89	-	.	ID=MALK_01721.t1;Parent=MALK_01721
contig006	AUGUSTUS	exon	90950	91339	0.89	-	.	ID=MALK_01721.t1.e1;Parent=MALK_01721.t1
contig006	AUGUSTUS	gene	91643	91944	1	-	.	ID=MALK_01722;prediction_source=braker_MRET:g1731.t1
contig006	AUGUSTUS	CDS	91914	91944	1	-	0	ID=MALK_01722.t1.c2;Parent=MALK_01722.t1

contig006	AUGUSTUS	CDS	91643	91875	1	-	0	ID=MALK_01722.t1.c1;Parent=MALK_01722.t1
contig006	AUGUSTUS	mRNA	91643	91944	1	-	.	ID=MALK_01722.t1;Parent=MALK_01722
contig006	AUGUSTUS	exon	91914	91944	.	-	.	ID=MALK_01722.t1.e2;Parent=MALK_01722.t1
contig006	AUGUSTUS	exon	91643	91875	.	-	.	ID=MALK_01722.t1.e1;Parent=MALK_01722.t1
contig006	maker	gene	92721	93809	.	+	.	ID=MALK_01723;prediction_source=maker_MRET:augustus_masked-contig006-processed-gene-0.23-mRNA-1
contig006	maker	CDS	92721	93809	.	+	0	ID=MALK_01723.t1.c1;Parent=MALK_01723.t1
contig006	maker	mRNA	92721	93809	.	+	.	ID=MALK_01723.t1;Parent=MALK_01723
contig006	maker	exon	92721	93809	.	+	.	ID=MALK_01723.t1.e1;Parent=MALK_01723.t1
contig006	AUGUSTUS	gene	93862	94730	0.45	+	.	ID=MALK_01724;prediction_source=braker_MRET:g1733.t1
contig006	AUGUSTUS	CDS	93862	94058	0.8	+	0	ID=MALK_01724.t1.c1;Parent=MALK_01724.t1
contig006	AUGUSTUS	CDS	94095	94672	0.8	+	0	ID=MALK_01724.t1.c2;Parent=MALK_01724.t1
contig006	AUGUSTUS	CDS	94711	94730	0.8	+	0	ID=MALK_01724.t1.c3;Parent=MALK_01724.t1
contig006	AUGUSTUS	mRNA	93862	94730	0.45	+	.	ID=MALK_01724.t1;Parent=MALK_01724
contig006	AUGUSTUS	exon	93862	94058	.	+	.	ID=MALK_01724.t1.e1;Parent=MALK_01724.t1
contig006	AUGUSTUS	exon	94095	94672	.	+	.	ID=MALK_01724.t1.e2;Parent=MALK_01724.t1
contig006	AUGUSTUS	exon	94711	94730	.	+	.	ID=MALK_01724.t1.e3;Parent=MALK_01724.t1
contig006	maker	gene	94797	99155	.	-	.	ID=MALK_01725;prediction_source=maker_MRET:augustus_masked-contig006-processed-gene-1.1-mRNA-1
contig006	maker	CDS	94797	99155	.	-	0	ID=MALK_01725.t1.c1;Parent=MALK_01725.t1
contig006	maker	mRNA	94797	99155	.	-	.	ID=MALK_01725.t1;Parent=MALK_01725
contig006	maker	exon	94797	99155	.	-	.	ID=MALK_01725.t1.e1;Parent=MALK_01725.t1
contig006	AUGUSTUS	gene	99356	103867	0.56	+	.	ID=MALK_01726;prediction_source=augustus:contig006.g1994.t1
contig006	AUGUSTUS	CDS	99356	103867	0.56	+	0	ID=MALK_01726.t1.c1;Parent=MALK_01726.t1
contig006	AUGUSTUS	mRNA	99356	103867	0.56	+	.	ID=MALK_01726.t1;Parent=MALK_01726
contig006	AUGUSTUS	exon	99356	103867	0.56	+	.	ID=MALK_01726.t1.e1;Parent=MALK_01726.t1
contig006	maker	gene	103859	107047	.	-	.	ID=MALK_01727;prediction_source=maker_MRET:augustus_masked-contig006-processed-gene-1.19-mRNA-1
contig006	maker	CDS	103859	107047	.	-	0	ID=MALK_01727.t1.c1;Parent=MALK_01727.t1
contig006	maker	mRNA	103859	107047	.	-	.	ID=MALK_01727.t1;Parent=MALK_01727
contig006	maker	exon	103859	107047	.	-	.	ID=MALK_01727.t1.e1;Parent=MALK_01727.t1
contig006	maker	gene	107593	109047	.	+	.	ID=MALK_01728;prediction_source=maker_MRET:augustus_masked-contig006-processed-gene-1.2-mRNA-1
contig006	maker	CDS	107593	109047	.	+	0	ID=MALK_01728.t1.c1;Parent=MALK_01728.t1
contig006	maker	mRNA	107593	109047	.	+	.	ID=MALK_01728.t1;Parent=MALK_01728
contig006	maker	exon	107593	109047	.	+	.	ID=MALK_01728.t1.e1;Parent=MALK_01728.t1
contig006	maker	gene	109745	110731	.	+	.	ID=MALK_01729;prediction_source=maker_MRET:augustus_masked-contig006-processed-gene-1.3-mRNA-1
contig006	maker	CDS	109745	110731	.	+	0	ID=MALK_01729.t1.c1;Parent=MALK_01729.t1
contig006	maker	mRNA	109745	110731	.	+	.	ID=MALK_01729.t1;Parent=MALK_01729
contig006	maker	exon	109745	110731	.	+	.	ID=MALK_01729.t1.e1;Parent=MALK_01729.t1
contig006	maker	gene	110841	111224	.	-	.	ID=MALK_01730;prediction_source=maker_MRET:augustus_masked-contig006-processed-gene-1.20-mRNA-1
contig006	maker	CDS	110841	111224	.	-	0	ID=MALK_01730.t1.c1;Parent=MALK_01730.t1
contig006	maker	mRNA	110841	111224	.	-	.	ID=MALK_01730.t1;Parent=MALK_01730
contig006	maker	exon	110841	111224	.	-	.	ID=MALK_01730.t1.e1;Parent=MALK_01730.t1
contig006	maker	gene	111343	112575	.	-	.	ID=MALK_01731;prediction_source=maker_MRET:augustus_masked-contig006-processed-gene-1.21-mRNA-1
contig006	maker	CDS	111343	112575	.	-	0	ID=MALK_01731.t1.c1;Parent=MALK_01731.t1
contig006	maker	mRNA	111343	112575	.	-	.	ID=MALK_01731.t1;Parent=MALK_01731

contig006	maker	exon	111343	112575	.	-	.	ID=MALK_01731.t1.e1;Parent=MALK_01731.t1
contig006	maker	gene	112654	114084	.	-	.	ID=MALK_01732;prediction_source=maker_MRET:augustus_masked-contig006-processed-gene-1.22-mRNA-1
contig006	maker	CDS	112654	114084	.	-	0	ID=MALK_01732.t1.c1;Parent=MALK_01732.t1
contig006	maker	mRNA	112654	114084	.	-	.	ID=MALK_01732.t1;Parent=MALK_01732
contig006	maker	exon	112654	114084	.	-	.	ID=MALK_01732.t1.e1;Parent=MALK_01732.t1
contig006	AUGUSTUS	gene	114416	114845	0.21	+	.	ID=MALK_01733;prediction_source=braker_MRET:g1741.t1
contig006	AUGUSTUS	CDS	114416	114520	0.32	+	0	ID=MALK_01733.t1.c1;Parent=MALK_01733.t1
contig006	AUGUSTUS	CDS	114584	114614	0.32	+	0	ID=MALK_01733.t1.c2;Parent=MALK_01733.t1
contig006	AUGUSTUS	CDS	114688	114845	0.32	+	0	ID=MALK_01733.t1.c3;Parent=MALK_01733.t1
contig006	AUGUSTUS	mRNA	114416	114845	0.21	+	.	ID=MALK_01733.t1;Parent=MALK_01733
contig006	AUGUSTUS	exon	114416	114520	.	+	.	ID=MALK_01733.t1.e1;Parent=MALK_01733.t1
contig006	AUGUSTUS	exon	114584	114614	.	+	.	ID=MALK_01733.t1.e2;Parent=MALK_01733.t1
contig006	AUGUSTUS	exon	114688	114845	.	+	.	ID=MALK_01733.t1.e3;Parent=MALK_01733.t1
contig006	AUGUSTUS	gene	114967	115376	0.5	-	.	ID=MALK_01734;prediction_source=braker_MRET:g1742.t1
contig006	AUGUSTUS	CDS	115198	115376	0.9	-	0	ID=MALK_01734.t1.c3;Parent=MALK_01734.t1
contig006	AUGUSTUS	CDS	115097	115154	0.9	-	0	ID=MALK_01734.t1.c2;Parent=MALK_01734.t1
contig006	AUGUSTUS	CDS	114967	115062	0.9	-	0	ID=MALK_01734.t1.c1;Parent=MALK_01734.t1
contig006	AUGUSTUS	mRNA	114967	115376	0.5	-	.	ID=MALK_01734.t1;Parent=MALK_01734
contig006	AUGUSTUS	exon	115198	115376	.	-	.	ID=MALK_01734.t1.e3;Parent=MALK_01734.t1
contig006	AUGUSTUS	exon	115097	115154	.	-	.	ID=MALK_01734.t1.e2;Parent=MALK_01734.t1
contig006	AUGUSTUS	exon	114967	115062	.	-	.	ID=MALK_01734.t1.e1;Parent=MALK_01734.t1
contig006	maker	gene	115468	116511	.	+	.	ID=MALK_01735;prediction_source=maker_MRET:augustus_masked-contig006-processed-gene-1.5-mRNA-1
contig006	maker	CDS	115468	116511	.	+	0	ID=MALK_01735.t1.c1;Parent=MALK_01735.t1
contig006	maker	mRNA	115468	116511	.	+	.	ID=MALK_01735.t1;Parent=MALK_01735
contig006	maker	exon	115468	116511	.	+	.	ID=MALK_01735.t1.e1;Parent=MALK_01735.t1
contig006	maker	gene	116630	118387	.	+	.	ID=MALK_01736;prediction_source=maker_MRET:augustus_masked-contig006-processed-gene-1.6-mRNA-1
contig006	maker	CDS	116630	118387	.	+	0	ID=MALK_01736.t1.c1;Parent=MALK_01736.t1
contig006	maker	mRNA	116630	118387	.	+	.	ID=MALK_01736.t1;Parent=MALK_01736
contig006	maker	exon	116630	118387	.	+	.	ID=MALK_01736.t1.e1;Parent=MALK_01736.t1
contig006	AUGUSTUS	gene	118382	119440	0.24	-	.	ID=MALK_01737;prediction_source=braker_MRET:g1745.t1
contig006	AUGUSTUS	CDS	118382	119440	0.24	-	0	ID=MALK_01737.t1.c1;Parent=MALK_01737.t1
contig006	AUGUSTUS	mRNA	118382	119440	0.24	-	.	ID=MALK_01737.t1;Parent=MALK_01737
contig006	AUGUSTUS	exon	118382	119440	.	-	.	ID=MALK_01737.t1.e1;Parent=MALK_01737.t1
contig006	AUGUSTUS	gene	119454	120596	0.54	+	.	ID=MALK_01738;prediction_source=augustus:contig006.g2005.t1
contig006	AUGUSTUS	CDS	119454	120596	0.54	+	0	ID=MALK_01738.t1.c1;Parent=MALK_01738.t1
contig006	AUGUSTUS	mRNA	119454	120596	0.54	+	.	ID=MALK_01738.t1;Parent=MALK_01738
contig006	AUGUSTUS	exon	119454	120596	0.54	+	.	ID=MALK_01738.t1.e1;Parent=MALK_01738.t1
contig006	AUGUSTUS	gene	120593	123520	0.97	-	.	ID=MALK_01739;prediction_source=augustus:contig006.g2006.t1
contig006	AUGUSTUS	CDS	120593	123520	0.97	-	0	ID=MALK_01739.t1.c1;Parent=MALK_01739.t1
contig006	AUGUSTUS	mRNA	120593	123520	0.97	-	.	ID=MALK_01739.t1;Parent=MALK_01739
contig006	AUGUSTUS	exon	120593	123520	0.97	-	.	ID=MALK_01739.t1.e1;Parent=MALK_01739.t1
contig006	AUGUSTUS	gene	123622	124085	0.43	+	.	ID=MALK_01740;prediction_source=braker_MRET:g1748.t1
contig006	AUGUSTUS	CDS	123622	123691	0.55	+	0	ID=MALK_01740.t1.c1;Parent=MALK_01740.t1

contig006	AUGUSTUS	CDS	123720	123967	0.55	+	0	ID=MALK_01740.t1.c2;Parent=MALK_01740.t1
contig006	AUGUSTUS	CDS	123996	124085	0.55	+	0	ID=MALK_01740.t1.c3;Parent=MALK_01740.t1
contig006	AUGUSTUS	mRNA	123622	124085	0.43	+	.	ID=MALK_01740.t1;Parent=MALK_01740
contig006	AUGUSTUS	exon	123622	123691	.	+	.	ID=MALK_01740.t1.e1;Parent=MALK_01740.t1
contig006	AUGUSTUS	exon	123720	123967	.	+	.	ID=MALK_01740.t1.e2;Parent=MALK_01740.t1
contig006	AUGUSTUS	exon	123996	124085	.	+	.	ID=MALK_01740.t1.e3;Parent=MALK_01740.t1
contig006	AUGUSTUS	gene	124148	124481	0.7	-	.	ID=MALK_01741;prediction_source=braker_MRET:g1749.t1
contig006	AUGUSTUS	CDS	124400	124481	0.94	-	0	ID=MALK_01741.t1.c2;Parent=MALK_01741.t1
contig006	AUGUSTUS	CDS	124148	124368	0.94	-	0	ID=MALK_01741.t1.c1;Parent=MALK_01741.t1
contig006	AUGUSTUS	mRNA	124148	124481	0.7	-	.	ID=MALK_01741.t1;Parent=MALK_01741
contig006	AUGUSTUS	exon	124400	124481	.	-	.	ID=MALK_01741.t1.e2;Parent=MALK_01741.t1
contig006	AUGUSTUS	exon	124148	124368	.	-	.	ID=MALK_01741.t1.e1;Parent=MALK_01741.t1
contig006	maker	gene	124485	125504	.	+	.	ID=MALK_01742;prediction_source=maker_MRET:augustus_masked-contig006-processed-gene-1.7-mRNA-1
contig006	maker	CDS	124485	125504	.	+	0	ID=MALK_01742.t1.c1;Parent=MALK_01742.t1
contig006	maker	mRNA	124485	125504	.	+	.	ID=MALK_01742.t1;Parent=MALK_01742
contig006	maker	exon	124485	125504	.	+	.	ID=MALK_01742.t1.e1;Parent=MALK_01742.t1
contig006	AUGUSTUS	gene	125537	126469	0.76	-	.	ID=MALK_01743;prediction_source=braker_MRET:g1751.t1
contig006	AUGUSTUS	CDS	126365	126469	0.77	-	0	ID=MALK_01743.t1.c2;Parent=MALK_01743.t1
contig006	AUGUSTUS	CDS	125537	126328	0.77	-	0	ID=MALK_01743.t1.c1;Parent=MALK_01743.t1
contig006	AUGUSTUS	mRNA	125537	126469	0.76	-	.	ID=MALK_01743.t1;Parent=MALK_01743
contig006	AUGUSTUS	exon	126365	126469	.	-	.	ID=MALK_01743.t1.e2;Parent=MALK_01743.t1
contig006	AUGUSTUS	exon	125537	126328	.	-	.	ID=MALK_01743.t1.e1;Parent=MALK_01743.t1
contig006	AUGUSTUS	gene	126596	126910	0.48	+	.	ID=MALK_01744;prediction_source=braker_MRET:g1752.t1
contig006	AUGUSTUS	CDS	126596	126619	0.48	+	0	ID=MALK_01744.t1.c1;Parent=MALK_01744.t1
contig006	AUGUSTUS	CDS	126650	126910	0.48	+	0	ID=MALK_01744.t1.c2;Parent=MALK_01744.t1
contig006	AUGUSTUS	mRNA	126596	126910	0.48	+	.	ID=MALK_01744.t1;Parent=MALK_01744
contig006	AUGUSTUS	exon	126596	126619	.	+	.	ID=MALK_01744.t1.e1;Parent=MALK_01744.t1
contig006	AUGUSTUS	exon	126650	126910	.	+	.	ID=MALK_01744.t1.e2;Parent=MALK_01744.t1
contig006	AUGUSTUS	gene	127058	127510	0.9	-	.	ID=MALK_01745;prediction_source=braker_MRET:g1753.t1
contig006	AUGUSTUS	CDS	127058	127510	0.9	-	0	ID=MALK_01745.t1.c1;Parent=MALK_01745.t1
contig006	AUGUSTUS	mRNA	127058	127510	0.9	-	.	ID=MALK_01745.t1;Parent=MALK_01745
contig006	AUGUSTUS	exon	127058	127510	.	-	.	ID=MALK_01745.t1.e1;Parent=MALK_01745.t1
contig006	maker	gene	127604	129076	.	+	.	ID=MALK_01746;prediction_source=maker_MRET:augustus_masked-contig006-processed-gene-1.8-mRNA-1
contig006	maker	CDS	127604	129076	.	+	0	ID=MALK_01746.t1.c1;Parent=MALK_01746.t1
contig006	maker	mRNA	127604	129076	.	+	.	ID=MALK_01746.t1;Parent=MALK_01746
contig006	maker	exon	127604	129076	.	+	.	ID=MALK_01746.t1.e1;Parent=MALK_01746.t1
contig006	maker	gene	129725	130828	.	-	.	ID=MALK_01747;prediction_source=maker_MRET:augustus_masked-contig006-processed-gene-1.26-mRNA-1
contig006	maker	CDS	129725	130828	.	-	0	ID=MALK_01747.t1.c1;Parent=MALK_01747.t1
contig006	maker	mRNA	129725	130828	.	-	.	ID=MALK_01747.t1;Parent=MALK_01747
contig006	maker	exon	129725	130828	.	-	.	ID=MALK_01747.t1.e1;Parent=MALK_01747.t1
contig006	AUGUSTUS	gene	130901	131333	0.42	-	.	ID=MALK_01748;prediction_source=braker_MRET:g1756.t1
contig006	AUGUSTUS	CDS	131315	131333	0.49	-	0	ID=MALK_01748.t1.c3;Parent=MALK_01748.t1
contig006	AUGUSTUS	CDS	131276	131286	0.49	-	0	ID=MALK_01748.t1.c2;Parent=MALK_01748.t1

contig006	AUGUSTUS	CDS	130901	131233	0.49	-	0	ID=MALK_01748.t1.c1;Parent=MALK_01748.t1
contig006	AUGUSTUS	mRNA	130901	131333	0.42	-	.	ID=MALK_01748.t1;Parent=MALK_01748
contig006	AUGUSTUS	exon	131315	131333	.	-	.	ID=MALK_01748.t1.e3;Parent=MALK_01748.t1
contig006	AUGUSTUS	exon	131276	131286	.	-	.	ID=MALK_01748.t1.e2;Parent=MALK_01748.t1
contig006	AUGUSTUS	exon	130901	131233	.	-	.	ID=MALK_01748.t1.e1;Parent=MALK_01748.t1
contig006	maker	gene	132216	133715	.	+	.	ID=MALK_01749;prediction_source=maker_MRET:augustus_masked-contig006-processed-gene-1.9-mRNA-1
contig006	maker	CDS	132216	133715	.	+	0	ID=MALK_01749.t1.c1;Parent=MALK_01749.t1
contig006	maker	mRNA	132216	133715	.	+	.	ID=MALK_01749.t1;Parent=MALK_01749
contig006	maker	exon	132216	133715	.	+	.	ID=MALK_01749.t1.e1;Parent=MALK_01749.t1
contig006	AUGUSTUS	gene	133967	134622	0.7	-	.	ID=MALK_01750;prediction_source=braker_MRET:g1758.t1
contig006	AUGUSTUS	CDS	134608	134622	0.71	-	0	ID=MALK_01750.t1.c4;Parent=MALK_01750.t1
contig006	AUGUSTUS	CDS	134522	134524	0.71	-	0	ID=MALK_01750.t1.c3;Parent=MALK_01750.t1
contig006	AUGUSTUS	CDS	134123	134403	0.71	-	0	ID=MALK_01750.t1.c2;Parent=MALK_01750.t1
contig006	AUGUSTUS	CDS	133967	134024	0.71	-	0	ID=MALK_01750.t1.c1;Parent=MALK_01750.t1
contig006	AUGUSTUS	mRNA	133967	134622	0.7	-	.	ID=MALK_01750.t1;Parent=MALK_01750
contig006	AUGUSTUS	exon	134608	134622	.	-	.	ID=MALK_01750.t1.e4;Parent=MALK_01750.t1
contig006	AUGUSTUS	exon	134522	134524	.	-	.	ID=MALK_01750.t1.e3;Parent=MALK_01750.t1
contig006	AUGUSTUS	exon	134123	134403	.	-	.	ID=MALK_01750.t1.e2;Parent=MALK_01750.t1
contig006	AUGUSTUS	exon	133967	134024	.	-	.	ID=MALK_01750.t1.e1;Parent=MALK_01750.t1
contig006	AUGUSTUS	gene	134868	135252	1	+	.	ID=MALK_01751;prediction_source=braker_MRET:g1759.t1
contig006	AUGUSTUS	CDS	134868	134884	1	+	0	ID=MALK_01751.t1.c1;Parent=MALK_01751.t1
contig006	AUGUSTUS	CDS	134978	135039	1	+	0	ID=MALK_01751.t1.c2;Parent=MALK_01751.t1
contig006	AUGUSTUS	CDS	135083	135252	1	+	0	ID=MALK_01751.t1.c3;Parent=MALK_01751.t1
contig006	AUGUSTUS	mRNA	134868	135252	1	+	.	ID=MALK_01751.t1;Parent=MALK_01751
contig006	AUGUSTUS	exon	134868	134884	.	+	.	ID=MALK_01751.t1.e1;Parent=MALK_01751.t1
contig006	AUGUSTUS	exon	134978	135039	.	+	.	ID=MALK_01751.t1.e2;Parent=MALK_01751.t1
contig006	AUGUSTUS	exon	135083	135252	.	+	.	ID=MALK_01751.t1.e3;Parent=MALK_01751.t1
contig006	AUGUSTUS	gene	135738	136425	0.97	-	.	ID=MALK_01752;prediction_source=braker_MRET:g1760.t1
contig006	AUGUSTUS	CDS	136318	136425	0.97	-	0	ID=MALK_01752.t1.c4;Parent=MALK_01752.t1
contig006	AUGUSTUS	CDS	135949	136287	0.97	-	0	ID=MALK_01752.t1.c3;Parent=MALK_01752.t1
contig006	AUGUSTUS	CDS	135829	135908	0.97	-	0	ID=MALK_01752.t1.c2;Parent=MALK_01752.t1
contig006	AUGUSTUS	CDS	135738	135789	0.97	-	0	ID=MALK_01752.t1.c1;Parent=MALK_01752.t1
contig006	AUGUSTUS	mRNA	135738	136425	0.97	-	.	ID=MALK_01752.t1;Parent=MALK_01752
contig006	AUGUSTUS	exon	136318	136425	.	-	.	ID=MALK_01752.t1.e4;Parent=MALK_01752.t1
contig006	AUGUSTUS	exon	135949	136287	.	-	.	ID=MALK_01752.t1.e3;Parent=MALK_01752.t1
contig006	AUGUSTUS	exon	135829	135908	.	-	.	ID=MALK_01752.t1.e2;Parent=MALK_01752.t1
contig006	AUGUSTUS	exon	135738	135789	.	-	.	ID=MALK_01752.t1.e1;Parent=MALK_01752.t1
contig006	AUGUSTUS	gene	136919	138494	0.74	+	.	ID=MALK_01753;prediction_source=braker_MRET:g1761.t1
contig006	AUGUSTUS	CDS	136919	137134	1	+	0	ID=MALK_01753.t1.c1;Parent=MALK_01753.t1
contig006	AUGUSTUS	CDS	137175	138494	1	+	0	ID=MALK_01753.t1.c2;Parent=MALK_01753.t1
contig006	AUGUSTUS	mRNA	136919	138494	0.74	+	.	ID=MALK_01753.t1;Parent=MALK_01753
contig006	AUGUSTUS	exon	136919	137134	.	+	.	ID=MALK_01753.t1.e1;Parent=MALK_01753.t1
contig006	AUGUSTUS	exon	137175	138494	.	+	.	ID=MALK_01753.t1.e2;Parent=MALK_01753.t1

contig006	AUGUSTUS	gene	138514	140595	1	-	.	ID=MALK_01754;prediction_source=augustus:contig006.g2017.t1
contig006	AUGUSTUS	CDS	138514	140595	1	-	0	ID=MALK_01754.t1.c1;Parent=MALK_01754.t1
contig006	AUGUSTUS	mRNA	138514	140595	1	-	.	ID=MALK_01754.t1;Parent=MALK_01754
contig006	AUGUSTUS	exon	138514	140595	1	-	.	ID=MALK_01754.t1.e1;Parent=MALK_01754.t1
contig006	maker	gene	140673	141662	.	+	.	ID=MALK_01755;prediction_source=maker_MRET:augustus_masked-contig006-processed-gene-1.11-mRNA-1
contig006	maker	CDS	140673	141662	.	+	0	ID=MALK_01755.t1.c1;Parent=MALK_01755.t1
contig006	maker	mRNA	140673	141662	.	+	.	ID=MALK_01755.t1;Parent=MALK_01755
contig006	maker	exon	140673	141662	.	+	.	ID=MALK_01755.t1.e1;Parent=MALK_01755.t1
contig006	AUGUSTUS	gene	141768	143426	0.67	-	.	ID=MALK_01756;prediction_source=augustus:contig006.g2019.t1
contig006	AUGUSTUS	CDS	141768	143426	0.67	-	0	ID=MALK_01756.t1.c1;Parent=MALK_01756.t1
contig006	AUGUSTUS	mRNA	141768	143426	0.67	-	.	ID=MALK_01756.t1;Parent=MALK_01756
contig006	AUGUSTUS	exon	141768	143426	0.67	-	.	ID=MALK_01756.t1.e1;Parent=MALK_01756.t1
contig006	AUGUSTUS	gene	143680	146661	0.62	+	.	ID=MALK_01757;prediction_source=augustus:contig006.g2020.t1
contig006	AUGUSTUS	CDS	143680	146661	0.62	+	0	ID=MALK_01757.t1.c1;Parent=MALK_01757.t1
contig006	AUGUSTUS	mRNA	143680	146661	0.62	+	.	ID=MALK_01757.t1;Parent=MALK_01757
contig006	AUGUSTUS	exon	143680	146661	0.62	+	.	ID=MALK_01757.t1.e1;Parent=MALK_01757.t1
contig006	maker	gene	146747	150304	.	-	.	ID=MALK_01758;prediction_source=maker_MRET:augustus_masked-contig006-processed-gene-1.32-mRNA-1
contig006	maker	CDS	146747	150304	.	-	0	ID=MALK_01758.t1.c1;Parent=MALK_01758.t1
contig006	maker	mRNA	146747	150304	.	-	.	ID=MALK_01758.t1;Parent=MALK_01758
contig006	maker	exon	146747	150304	.	-	.	ID=MALK_01758.t1.e1;Parent=MALK_01758.t1
contig006	maker	gene	150474	152051	.	-	.	ID=MALK_01759;prediction_source=maker_MRET:augustus_masked-contig006-processed-gene-1.33-mRNA-1
contig006	maker	CDS	150474	152051	.	-	0	ID=MALK_01759.t1.c1;Parent=MALK_01759.t1
contig006	maker	mRNA	150474	152051	.	-	.	ID=MALK_01759.t1;Parent=MALK_01759
contig006	maker	exon	150474	152051	.	-	.	ID=MALK_01759.t1.e1;Parent=MALK_01759.t1
contig006	maker	gene	152175	153416	.	+	.	ID=MALK_01760;prediction_source=maker_MRET:augustus_masked-contig006-processed-gene-1.13-mRNA-1
contig006	maker	CDS	152175	153416	.	+	0	ID=MALK_01760.t1.c1;Parent=MALK_01760.t1
contig006	maker	mRNA	152175	153416	.	+	.	ID=MALK_01760.t1;Parent=MALK_01760
contig006	maker	exon	152175	153416	.	+	.	ID=MALK_01760.t1.e1;Parent=MALK_01760.t1
contig006	maker	gene	153458	156682	.	-	.	ID=MALK_01761;prediction_source=maker_MRET:augustus_masked-contig006-processed-gene-1.34-mRNA-1
contig006	maker	CDS	156647	156682	.	-	0	ID=MALK_01761.t1.c1;Parent=MALK_01761.t1
contig006	maker	CDS	154850	156234	.	-	0	ID=MALK_01761.t1.c2;Parent=MALK_01761.t1
contig006	maker	CDS	153458	154601	.	-	0	ID=MALK_01761.t1.c3;Parent=MALK_01761.t1
contig006	maker	mRNA	153458	156682	.	-	.	ID=MALK_01761.t1;Parent=MALK_01761
contig006	maker	exon	156647	156682	.	-	.	ID=MALK_01761.t1.e1;Parent=MALK_01761.t1
contig006	maker	exon	154850	156234	.	-	.	ID=MALK_01761.t1.e2;Parent=MALK_01761.t1
contig006	maker	exon	153458	154601	.	-	.	ID=MALK_01761.t1.e3;Parent=MALK_01761.t1
contig006	AUGUSTUS	gene	157072	157812	0.37	-	.	ID=MALK_01762;prediction_source=augustus:contig006.g2026.t1
contig006	AUGUSTUS	CDS	157072	157812	0.37	-	0	ID=MALK_01762.t1.c1;Parent=MALK_01762.t1
contig006	AUGUSTUS	mRNA	157072	157812	0.37	-	.	ID=MALK_01762.t1;Parent=MALK_01762
contig006	AUGUSTUS	exon	157072	157812	0.37	-	.	ID=MALK_01762.t1.e1;Parent=MALK_01762.t1
contig006	AUGUSTUS	gene	157930	162861	0.97	-	.	ID=MALK_01763;prediction_source=augustus:contig006.g2027.t1
contig006	AUGUSTUS	CDS	157930	162861	0.97	-	0	ID=MALK_01763.t1.c1;Parent=MALK_01763.t1
contig006	AUGUSTUS	mRNA	157930	162861	0.97	-	.	ID=MALK_01763.t1;Parent=MALK_01763

contig006	AUGUSTUS	exon	157930	162861	0.97	-	.	ID=MALK_01763.t1.e1;Parent=MALK_01763.t1
contig006	AUGUSTUS	gene	163050	165809	0.61	+	.	ID=MALK_01764;prediction_source=augustus:contig006.g2028.t1
contig006	AUGUSTUS	CDS	163050	165809	0.61	+	0	ID=MALK_01764.t1.c1;Parent=MALK_01764.t1
contig006	AUGUSTUS	mRNA	163050	165809	0.61	+	.	ID=MALK_01764.t1;Parent=MALK_01764
contig006	AUGUSTUS	exon	163050	165809	0.61	+	.	ID=MALK_01764.t1.e1;Parent=MALK_01764.t1
contig006	AUGUSTUS	gene	165806	166555	0.96	-	.	ID=MALK_01765;prediction_source=augustus:contig006.g2029.t1
contig006	AUGUSTUS	CDS	165806	166555	0.96	-	0	ID=MALK_01765.t1.c1;Parent=MALK_01765.t1
contig006	AUGUSTUS	mRNA	165806	166555	0.96	-	.	ID=MALK_01765.t1;Parent=MALK_01765
contig006	AUGUSTUS	exon	165806	166555	0.96	-	.	ID=MALK_01765.t1.e1;Parent=MALK_01765.t1
contig006	AUGUSTUS	gene	166604	171223	0.5	-	.	ID=MALK_01766;prediction_source=braker_MRET:g1774.t1
contig006	AUGUSTUS	CDS	166604	171223	0.5	-	0	ID=MALK_01766.t1.c1;Parent=MALK_01766.t1
contig006	AUGUSTUS	mRNA	166604	171223	0.5	-	.	ID=MALK_01766.t1;Parent=MALK_01766
contig006	AUGUSTUS	exon	166604	171223	.	-	.	ID=MALK_01766.t1.e1;Parent=MALK_01766.t1
contig006	maker	gene	171337	177658	.	-	.	ID=MALK_01767;prediction_source=maker_MRET:augustus_masked-contig006-processed-gene-1.38-mRNA-1
contig006	maker	CDS	177617	177658	.	-	0	ID=MALK_01767.t1.c1;Parent=MALK_01767.t1
contig006	maker	CDS	171337	177477	.	-	0	ID=MALK_01767.t1.c2;Parent=MALK_01767.t1
contig006	maker	mRNA	171337	177658	.	-	.	ID=MALK_01767.t1;Parent=MALK_01767
contig006	maker	exon	177617	177658	.	-	.	ID=MALK_01767.t1.e1;Parent=MALK_01767.t1
contig006	maker	exon	171337	177477	.	-	.	ID=MALK_01767.t1.e2;Parent=MALK_01767.t1
contig006	AUGUSTUS	gene	177699	180866	0.9	-	.	ID=MALK_01768;prediction_source=augustus:contig006.g2033.t1
contig006	AUGUSTUS	CDS	177699	180866	0.9	-	0	ID=MALK_01768.t1.c1;Parent=MALK_01768.t1
contig006	AUGUSTUS	mRNA	177699	180866	0.9	-	.	ID=MALK_01768.t1;Parent=MALK_01768
contig006	AUGUSTUS	exon	177699	180866	0.9	-	.	ID=MALK_01768.t1.e1;Parent=MALK_01768.t1
contig006	AUGUSTUS	gene	180978	182082	0.97	-	.	ID=MALK_01769;prediction_source=braker_MRET:g1777.t1
contig006	AUGUSTUS	CDS	181505	182082	0.97	-	0	ID=MALK_01769.t1.c2;Parent=MALK_01769.t1
contig006	AUGUSTUS	CDS	180978	181476	0.97	-	0	ID=MALK_01769.t1.c1;Parent=MALK_01769.t1
contig006	AUGUSTUS	mRNA	180978	182082	0.97	-	.	ID=MALK_01769.t1;Parent=MALK_01769
contig006	AUGUSTUS	exon	181505	182082	.	-	.	ID=MALK_01769.t1.e2;Parent=MALK_01769.t1
contig006	AUGUSTUS	exon	180978	181476	.	-	.	ID=MALK_01769.t1.e1;Parent=MALK_01769.t1
contig006	AUGUSTUS	gene	182202	183991	0.29	+	.	ID=MALK_01770;prediction_source=braker_MRET:g1778.t1
contig006	AUGUSTUS	CDS	182202	183680	0.46	+	0	ID=MALK_01770.t1.c1;Parent=MALK_01770.t1
contig006	AUGUSTUS	CDS	183761	183991	0.46	+	0	ID=MALK_01770.t1.c2;Parent=MALK_01770.t1
contig006	AUGUSTUS	mRNA	182202	183991	0.29	+	.	ID=MALK_01770.t1;Parent=MALK_01770
contig006	AUGUSTUS	exon	182202	183680	.	+	.	ID=MALK_01770.t1.e1;Parent=MALK_01770.t1
contig006	AUGUSTUS	exon	183761	183991	.	+	.	ID=MALK_01770.t1.e2;Parent=MALK_01770.t1
contig006	AUGUSTUS	gene	184525	185400	0.81	-	.	ID=MALK_01771;prediction_source=augustus:contig006.g2037.t1
contig006	AUGUSTUS	CDS	184525	185400	0.81	-	0	ID=MALK_01771.t1.c1;Parent=MALK_01771.t1
contig006	AUGUSTUS	mRNA	184525	185400	0.81	-	.	ID=MALK_01771.t1;Parent=MALK_01771
contig006	AUGUSTUS	exon	184525	185400	0.81	-	.	ID=MALK_01771.t1.e1;Parent=MALK_01771.t1
contig006	maker	gene	185529	186896	.	+	.	ID=MALK_01772;prediction_source=maker_MRET:augustus_masked-contig006-processed-gene-1.16-mRNA-1
contig006	maker	CDS	185529	186896	.	+	0	ID=MALK_01772.t1.c1;Parent=MALK_01772.t1
contig006	maker	mRNA	185529	186896	.	+	.	ID=MALK_01772.t1;Parent=MALK_01772
contig006	maker	exon	185529	186896	.	+	.	ID=MALK_01772.t1.e1;Parent=MALK_01772.t1

contig006	AUGUSTUS	gene	187069	188208	0.75	+	.	ID=MALK_01773;prediction_source=braker_MRET:g1781.t1
contig006	AUGUSTUS	CDS	187069	188208	0.75	+	0	ID=MALK_01773.t1.c1;Parent=MALK_01773.t1
contig006	AUGUSTUS	mRNA	187069	188208	0.75	+	.	ID=MALK_01773.t1;Parent=MALK_01773
contig006	AUGUSTUS	exon	187069	188208	.	+	.	ID=MALK_01773.t1.e1;Parent=MALK_01773.t1
contig006	AUGUSTUS	gene	188231	189097	0.9	-	.	ID=MALK_01774;prediction_source=augustus:contig006.g2040.t1
contig006	AUGUSTUS	CDS	188231	189097	0.9	-	0	ID=MALK_01774.t1.c1;Parent=MALK_01774.t1
contig006	AUGUSTUS	mRNA	188231	189097	0.9	-	.	ID=MALK_01774.t1;Parent=MALK_01774
contig006	AUGUSTUS	exon	188231	189097	0.9	-	.	ID=MALK_01774.t1.e1;Parent=MALK_01774.t1
contig006	AUGUSTUS	gene	189243	191399	0.42	+	.	ID=MALK_01775;prediction_source=augustus:contig006.g2041.t1
contig006	AUGUSTUS	CDS	189243	191399	0.42	+	0	ID=MALK_01775.t1.c1;Parent=MALK_01775.t1
contig006	AUGUSTUS	mRNA	189243	191399	0.42	+	.	ID=MALK_01775.t1;Parent=MALK_01775
contig006	AUGUSTUS	exon	189243	191399	0.42	+	.	ID=MALK_01775.t1.e1;Parent=MALK_01775.t1
contig006	AUGUSTUS	gene	191407	193071	1	-	.	ID=MALK_01776;prediction_source=augustus:contig006.g2042.t1
contig006	AUGUSTUS	CDS	191407	193071	1	-	0	ID=MALK_01776.t1.c1;Parent=MALK_01776.t1
contig006	AUGUSTUS	mRNA	191407	193071	1	-	.	ID=MALK_01776.t1;Parent=MALK_01776
contig006	AUGUSTUS	exon	191407	193071	1	-	.	ID=MALK_01776.t1.e1;Parent=MALK_01776.t1
contig006	AUGUSTUS	gene	193262	194854	0.87	+	.	ID=MALK_01777;prediction_source=augustus:contig006.g2043.t1
contig006	AUGUSTUS	CDS	193262	194854	0.87	+	0	ID=MALK_01777.t1.c1;Parent=MALK_01777.t1
contig006	AUGUSTUS	mRNA	193262	194854	0.87	+	.	ID=MALK_01777.t1;Parent=MALK_01777
contig006	AUGUSTUS	exon	193262	194854	0.87	+	.	ID=MALK_01777.t1.e1;Parent=MALK_01777.t1
contig006	maker	gene	194891	196607	.	-	.	ID=MALK_01778;prediction_source=maker_MRET:augustus_masked-contig006-processed-gene-2.60-mRNA-1
contig006	maker	CDS	195945	196607	.	-	0	ID=MALK_01778.t1.c1;Parent=MALK_01778.t1
contig006	maker	CDS	194891	195859	.	-	0	ID=MALK_01778.t1.c2;Parent=MALK_01778.t1
contig006	maker	mRNA	194891	196607	.	-	.	ID=MALK_01778.t1;Parent=MALK_01778
contig006	maker	exon	195945	196607	.	-	.	ID=MALK_01778.t1.e1;Parent=MALK_01778.t1
contig006	maker	exon	194891	195859	.	-	.	ID=MALK_01778.t1.e2;Parent=MALK_01778.t1
contig006	maker	gene	196765	198399	.	+	.	ID=MALK_01779;prediction_source=maker_MRET:augustus_masked-contig006-processed-gene-2.59-mRNA-1
contig006	maker	CDS	196765	198399	.	+	0	ID=MALK_01779.t1.c1;Parent=MALK_01779.t1
contig006	maker	mRNA	196765	198399	.	+	.	ID=MALK_01779.t1;Parent=MALK_01779
contig006	maker	exon	196765	198399	.	+	.	ID=MALK_01779.t1.e1;Parent=MALK_01779.t1
contig006	maker	gene	198559	199896	.	-	.	ID=MALK_01780;prediction_source=maker_MRET:augustus_masked-contig006-processed-gene-2.61-mRNA-1
contig006	maker	CDS	198559	199896	.	-	0	ID=MALK_01780.t1.c1;Parent=MALK_01780.t1
contig006	maker	mRNA	198559	199896	.	-	.	ID=MALK_01780.t1;Parent=MALK_01780
contig006	maker	exon	198559	199896	.	-	.	ID=MALK_01780.t1.e1;Parent=MALK_01780.t1
contig006	AUGUSTUS	gene	200096	204364	0.66	-	.	ID=MALK_01781;prediction_source=augustus:contig006.g2048.t1
contig006	AUGUSTUS	CDS	200096	204364	0.66	-	0	ID=MALK_01781.t1.c1;Parent=MALK_01781.t1
contig006	AUGUSTUS	mRNA	200096	204364	0.66	-	.	ID=MALK_01781.t1;Parent=MALK_01781
contig006	AUGUSTUS	exon	200096	204364	0.66	-	.	ID=MALK_01781.t1.e1;Parent=MALK_01781.t1
contig006	maker	gene	204587	205384	.	-	.	ID=MALK_01782;prediction_source=maker_MRET:augustus_masked-contig006-processed-gene-2.86-mRNA-1
contig006	maker	CDS	204587	205384	.	-	0	ID=MALK_01782.t1.c1;Parent=MALK_01782.t1
contig006	maker	mRNA	204587	205384	.	-	.	ID=MALK_01782.t1;Parent=MALK_01782
contig006	maker	exon	204587	205384	.	-	.	ID=MALK_01782.t1.e1;Parent=MALK_01782.t1
contig006	maker	gene	205582	208719	.	+	.	ID=MALK_01783;prediction_source=maker_MRET:augustus_masked-contig006-processed-gene-2.62-mRNA-1

contig006	maker	CDS	205582	208719	.	+	0	ID=MALK_01783.t1.c1;Parent=MALK_01783.t1
contig006	maker	mRNA	205582	208719	.	+	.	ID=MALK_01783.t1;Parent=MALK_01783
contig006	maker	exon	205582	208719	.	+	.	ID=MALK_01783.t1.e1;Parent=MALK_01783.t1
contig006	AUGUSTUS	gene	208732	210948	0.95	-	.	ID=MALK_01784;prediction_source=augustus:contig006.g2053.t1
contig006	AUGUSTUS	CDS	208732	210948	0.95	-	0	ID=MALK_01784.t1.c1;Parent=MALK_01784.t1
contig006	AUGUSTUS	mRNA	208732	210948	0.95	-	.	ID=MALK_01784.t1;Parent=MALK_01784
contig006	AUGUSTUS	exon	208732	210948	0.95	-	.	ID=MALK_01784.t1.e1;Parent=MALK_01784.t1
contig006	AUGUSTUS	gene	211013	211942	1	+	.	ID=MALK_01785;prediction_source=braker_MRET:g1793.t1
contig006	AUGUSTUS	CDS	211013	211942	1	+	0	ID=MALK_01785.t1.c1;Parent=MALK_01785.t1
contig006	AUGUSTUS	mRNA	211013	211942	1	+	.	ID=MALK_01785.t1;Parent=MALK_01785
contig006	AUGUSTUS	exon	211013	211942	.	+	.	ID=MALK_01785.t1.e1;Parent=MALK_01785.t1
contig006	AUGUSTUS	gene	211948	212529	0.46	-	.	ID=MALK_01786;prediction_source=augustus:contig006.g2055.t1
contig006	AUGUSTUS	CDS	211948	212529	0.46	-	0	ID=MALK_01786.t1.c1;Parent=MALK_01786.t1
contig006	AUGUSTUS	mRNA	211948	212529	0.46	-	.	ID=MALK_01786.t1;Parent=MALK_01786
contig006	AUGUSTUS	exon	211948	212529	0.46	-	.	ID=MALK_01786.t1.e1;Parent=MALK_01786.t1
contig006	AUGUSTUS	gene	212570	214593	0.18	-	.	ID=MALK_01787;prediction_source=braker_MRET:g1795.t1
contig006	AUGUSTUS	CDS	214478	214593	0.19	-	0	ID=MALK_01787.t1.c2;Parent=MALK_01787.t1
contig006	AUGUSTUS	CDS	212570	214283	0.19	-	0	ID=MALK_01787.t1.c1;Parent=MALK_01787.t1
contig006	AUGUSTUS	mRNA	212570	214593	0.18	-	.	ID=MALK_01787.t1;Parent=MALK_01787
contig006	AUGUSTUS	exon	214478	214593	.	-	.	ID=MALK_01787.t1.e2;Parent=MALK_01787.t1
contig006	AUGUSTUS	exon	212570	214283	.	-	.	ID=MALK_01787.t1.e1;Parent=MALK_01787.t1
contig006	AUGUSTUS	gene	214671	215315	0.99	+	.	ID=MALK_01788;prediction_source=augustus:contig006.g2057.t1
contig006	AUGUSTUS	CDS	214671	215315	0.99	+	0	ID=MALK_01788.t1.c1;Parent=MALK_01788.t1
contig006	AUGUSTUS	mRNA	214671	215315	0.99	+	.	ID=MALK_01788.t1;Parent=MALK_01788
contig006	AUGUSTUS	exon	214671	215315	0.99	+	.	ID=MALK_01788.t1.e1;Parent=MALK_01788.t1
contig006	maker	gene	215329	216411	.	-	.	ID=MALK_01789;prediction_source=maker_MRET:augustus_masked-contig006-processed-gene-2.90-mRNA-1
contig006	maker	CDS	215329	216411	.	-	0	ID=MALK_01789.t1.c1;Parent=MALK_01789.t1
contig006	maker	mRNA	215329	216411	.	-	.	ID=MALK_01789.t1;Parent=MALK_01789
contig006	maker	exon	215329	216411	.	-	.	ID=MALK_01789.t1.e1;Parent=MALK_01789.t1
contig006	maker	gene	216597	217109	.	+	.	ID=MALK_01790;prediction_source=maker_MRET:augustus_masked-contig006-processed-gene-2.64-mRNA-1
contig006	maker	CDS	216597	216845	.	+	0	ID=MALK_01790.t1.c1;Parent=MALK_01790.t1
contig006	maker	CDS	216906	217109	.	+	0	ID=MALK_01790.t1.c2;Parent=MALK_01790.t1
contig006	maker	mRNA	216597	217109	.	+	.	ID=MALK_01790.t1;Parent=MALK_01790
contig006	maker	exon	216597	216845	.	+	.	ID=MALK_01790.t1.e1;Parent=MALK_01790.t1
contig006	maker	exon	216906	217109	.	+	.	ID=MALK_01790.t1.e2;Parent=MALK_01790.t1
contig006	maker	gene	217240	218382	.	-	.	ID=MALK_01791;prediction_source=maker_MRET:augustus_masked-contig006-processed-gene-2.91-mRNA-1
contig006	maker	CDS	217240	218382	.	-	0	ID=MALK_01791.t1.c1;Parent=MALK_01791.t1
contig006	maker	mRNA	217240	218382	.	-	.	ID=MALK_01791.t1;Parent=MALK_01791
contig006	maker	exon	217240	218382	.	-	.	ID=MALK_01791.t1.e1;Parent=MALK_01791.t1
contig006	maker	gene	218680	221104	.	-	.	ID=MALK_01792;prediction_source=maker_MRET:augustus_masked-contig006-processed-gene-2.92-mRNA-1
contig006	maker	CDS	219525	221104	.	-	0	ID=MALK_01792.t1.c1;Parent=MALK_01792.t1
contig006	maker	CDS	218680	219205	.	-	0	ID=MALK_01792.t1.c2;Parent=MALK_01792.t1
contig006	maker	mRNA	218680	221104	.	-	.	ID=MALK_01792.t1;Parent=MALK_01792

contig006	maker	exon	219525	221104	.	-	.	ID=MALK_01792.t1.e1;Parent=MALK_01792.t1
contig006	maker	exon	218680	219205	.	-	.	ID=MALK_01792.t1.e2;Parent=MALK_01792.t1
contig006	AUGUSTUS	gene	221562	222529	0.47	-	.	ID=MALK_01793;prediction_source=braker_MRET:g1801.t1
contig006	AUGUSTUS	CDS	222083	222529	0.96	-	0	ID=MALK_01793.t1.c3;Parent=MALK_01793.t1
contig006	AUGUSTUS	CDS	221643	222050	0.96	-	0	ID=MALK_01793.t1.c2;Parent=MALK_01793.t1
contig006	AUGUSTUS	CDS	221562	221594	0.96	-	0	ID=MALK_01793.t1.c1;Parent=MALK_01793.t1
contig006	AUGUSTUS	mRNA	221562	222529	0.47	-	.	ID=MALK_01793.t1;Parent=MALK_01793
contig006	AUGUSTUS	exon	222083	222529	.	-	.	ID=MALK_01793.t1.e3;Parent=MALK_01793.t1
contig006	AUGUSTUS	exon	221643	222050	.	-	.	ID=MALK_01793.t1.e2;Parent=MALK_01793.t1
contig006	AUGUSTUS	exon	221562	221594	.	-	.	ID=MALK_01793.t1.e1;Parent=MALK_01793.t1
contig006	AUGUSTUS	gene	222679	224074	0.43	+	.	ID=MALK_01794;prediction_source=augustus:contig006.g2063.t1
contig006	AUGUSTUS	CDS	222679	222730	0.44	+	0	ID=MALK_01794.t1.c1;Parent=MALK_01794.t1
contig006	AUGUSTUS	CDS	222792	224074	0.44	+	0	ID=MALK_01794.t1.c2;Parent=MALK_01794.t1
contig006	AUGUSTUS	mRNA	222679	224074	0.43	+	.	ID=MALK_01794.t1;Parent=MALK_01794
contig006	AUGUSTUS	exon	222679	222730	0.44	+	.	ID=MALK_01794.t1.e1;Parent=MALK_01794.t1
contig006	AUGUSTUS	exon	222792	224074	0.44	+	.	ID=MALK_01794.t1.e2;Parent=MALK_01794.t1
contig006	AUGUSTUS	gene	224140	224545	0.64	-	.	ID=MALK_01795;prediction_source=braker_MRET:g1803.t1
contig006	AUGUSTUS	CDS	224543	224545	1	-	0	ID=MALK_01795.t1.c4;Parent=MALK_01795.t1
contig006	AUGUSTUS	CDS	224423	224466	1	-	0	ID=MALK_01795.t1.c3;Parent=MALK_01795.t1
contig006	AUGUSTUS	CDS	224271	224387	1	-	0	ID=MALK_01795.t1.c2;Parent=MALK_01795.t1
contig006	AUGUSTUS	CDS	224140	224236	1	-	0	ID=MALK_01795.t1.c1;Parent=MALK_01795.t1
contig006	AUGUSTUS	mRNA	224140	224545	0.64	-	.	ID=MALK_01795.t1;Parent=MALK_01795
contig006	AUGUSTUS	exon	224543	224545	.	-	.	ID=MALK_01795.t1.e4;Parent=MALK_01795.t1
contig006	AUGUSTUS	exon	224423	224466	.	-	.	ID=MALK_01795.t1.e3;Parent=MALK_01795.t1
contig006	AUGUSTUS	exon	224271	224387	.	-	.	ID=MALK_01795.t1.e2;Parent=MALK_01795.t1
contig006	AUGUSTUS	exon	224140	224236	.	-	.	ID=MALK_01795.t1.e1;Parent=MALK_01795.t1
contig006	AUGUSTUS	gene	224700	224974	1	+	.	ID=MALK_01796;prediction_source=braker_MRET:g1804.t1
contig006	AUGUSTUS	CDS	224700	224867	1	+	0	ID=MALK_01796.t1.c1;Parent=MALK_01796.t1
contig006	AUGUSTUS	CDS	224903	224974	1	+	0	ID=MALK_01796.t1.c2;Parent=MALK_01796.t1
contig006	AUGUSTUS	mRNA	224700	224974	1	+	.	ID=MALK_01796.t1;Parent=MALK_01796
contig006	AUGUSTUS	exon	224700	224867	.	+	.	ID=MALK_01796.t1.e1;Parent=MALK_01796.t1
contig006	AUGUSTUS	exon	224903	224974	.	+	.	ID=MALK_01796.t1.e2;Parent=MALK_01796.t1
contig006	AUGUSTUS	gene	225161	225781	0.66	+	.	ID=MALK_01797;prediction_source=augustus:contig006.g2064.t1
contig006	AUGUSTUS	CDS	225161	225781	0.66	+	0	ID=MALK_01797.t1.c1;Parent=MALK_01797.t1
contig006	AUGUSTUS	mRNA	225161	225781	0.66	+	.	ID=MALK_01797.t1;Parent=MALK_01797
contig006	AUGUSTUS	exon	225161	225781	0.66	+	.	ID=MALK_01797.t1.e1;Parent=MALK_01797.t1
contig006	AUGUSTUS	gene	225791	228154	0.93	-	.	ID=MALK_01798;prediction_source=braker_MRET:g1806.t1
contig006	AUGUSTUS	CDS	225791	228154	0.93	-	0	ID=MALK_01798.t1.c1;Parent=MALK_01798.t1
contig006	AUGUSTUS	mRNA	225791	228154	0.93	-	.	ID=MALK_01798.t1;Parent=MALK_01798
contig006	AUGUSTUS	exon	225791	228154	.	-	.	ID=MALK_01798.t1.e1;Parent=MALK_01798.t1
contig006	AUGUSTUS	gene	228276	230324	0.73	+	.	ID=MALK_01799;prediction_source=braker_MRET:g1807.t1
contig006	AUGUSTUS	CDS	228276	230324	0.73	+	0	ID=MALK_01799.t1.c1;Parent=MALK_01799.t1
contig006	AUGUSTUS	mRNA	228276	230324	0.73	+	.	ID=MALK_01799.t1;Parent=MALK_01799

contig006	AUGUSTUS	exon	228276	230324	.	+	.	ID=MALK_01799.t1.e1;Parent=MALK_01799.t1
contig006	AUGUSTUS	gene	230339	231430	0.38	-	.	ID=MALK_01800;prediction_source=augustus:contig006.g2067.t1
contig006	AUGUSTUS	CDS	230339	231430	0.38	-	0	ID=MALK_01800.t1.c1;Parent=MALK_01800.t1
contig006	AUGUSTUS	mRNA	230339	231430	0.38	-	.	ID=MALK_01800.t1;Parent=MALK_01800
contig006	AUGUSTUS	exon	230339	231430	0.38	-	.	ID=MALK_01800.t1.e1;Parent=MALK_01800.t1
contig006	AUGUSTUS	gene	231472	232254	0.95	-	.	ID=MALK_01801;prediction_source=augustus:contig006.g2068.t1
contig006	AUGUSTUS	CDS	231472	232254	0.95	-	0	ID=MALK_01801.t1.c1;Parent=MALK_01801.t1
contig006	AUGUSTUS	mRNA	231472	232254	0.95	-	.	ID=MALK_01801.t1;Parent=MALK_01801
contig006	AUGUSTUS	exon	231472	232254	0.95	-	.	ID=MALK_01801.t1.e1;Parent=MALK_01801.t1
contig006	maker	gene	232383	233166	.	-	.	ID=MALK_01802;prediction_source=maker_MRET:augustus_masked-contig006-processed-gene-2.97-mRNA-1
contig006	maker	CDS	233129	233166	.	-	0	ID=MALK_01802.t1.c1;Parent=MALK_01802.t1
contig006	maker	CDS	232383	233046	.	-	0	ID=MALK_01802.t1.c2;Parent=MALK_01802.t1
contig006	maker	mRNA	232383	233166	.	-	.	ID=MALK_01802.t1;Parent=MALK_01802
contig006	maker	exon	233129	233166	.	-	.	ID=MALK_01802.t1.e1;Parent=MALK_01802.t1
contig006	maker	exon	232383	233046	.	-	.	ID=MALK_01802.t1.e2;Parent=MALK_01802.t1
contig006	AUGUSTUS	gene	233343	234269	0.97	+	.	ID=MALK_01803;prediction_source=augustus:contig006.g2070.t1
contig006	AUGUSTUS	CDS	233343	234269	0.97	+	0	ID=MALK_01803.t1.c1;Parent=MALK_01803.t1
contig006	AUGUSTUS	mRNA	233343	234269	0.97	+	.	ID=MALK_01803.t1;Parent=MALK_01803
contig006	AUGUSTUS	exon	233343	234269	0.97	+	.	ID=MALK_01803.t1.e1;Parent=MALK_01803.t1
contig006	maker	gene	234437	236059	.	-	.	ID=MALK_01804;prediction_source=maker_MRET:augustus_masked-contig006-processed-gene-2.98-mRNA-1
contig006	maker	CDS	234437	236059	.	-	0	ID=MALK_01804.t1.c1;Parent=MALK_01804.t1
contig006	maker	mRNA	234437	236059	.	-	.	ID=MALK_01804.t1;Parent=MALK_01804
contig006	maker	exon	234437	236059	.	-	.	ID=MALK_01804.t1.e1;Parent=MALK_01804.t1
contig006	maker	gene	236112	236759	.	-	.	ID=MALK_01805;prediction_source=maker_MRET:augustus_masked-contig006-processed-gene-2.99-mRNA-1
contig006	maker	CDS	236112	236759	.	-	0	ID=MALK_01805.t1.c1;Parent=MALK_01805.t1
contig006	maker	mRNA	236112	236759	.	-	.	ID=MALK_01805.t1;Parent=MALK_01805
contig006	maker	exon	236112	236759	.	-	.	ID=MALK_01805.t1.e1;Parent=MALK_01805.t1
contig006	AUGUSTUS	gene	236858	238441	0.94	+	.	ID=MALK_01806;prediction_source=augustus:contig006.g2073.t1
contig006	AUGUSTUS	CDS	236858	238441	0.94	+	0	ID=MALK_01806.t1.c1;Parent=MALK_01806.t1
contig006	AUGUSTUS	mRNA	236858	238441	0.94	+	.	ID=MALK_01806.t1;Parent=MALK_01806
contig006	AUGUSTUS	exon	236858	238441	0.94	+	.	ID=MALK_01806.t1.e1;Parent=MALK_01806.t1
contig006	AUGUSTUS	gene	238502	239635	0.66	+	.	ID=MALK_01807;prediction_source=braker_MRET:g1814.t1
contig006	AUGUSTUS	CDS	238502	239635	0.66	+	0	ID=MALK_01807.t1.c1;Parent=MALK_01807.t1
contig006	AUGUSTUS	mRNA	238502	239635	0.66	+	.	ID=MALK_01807.t1;Parent=MALK_01807
contig006	AUGUSTUS	exon	238502	239635	.	+	.	ID=MALK_01807.t1.e1;Parent=MALK_01807.t1
contig006	AUGUSTUS	gene	239748	241337	0.98	+	.	ID=MALK_01808;prediction_source=augustus:contig006.g2074.t1
contig006	AUGUSTUS	CDS	239748	241337	0.98	+	0	ID=MALK_01808.t1.c1;Parent=MALK_01808.t1
contig006	AUGUSTUS	mRNA	239748	241337	0.98	+	.	ID=MALK_01808.t1;Parent=MALK_01808
contig006	AUGUSTUS	exon	239748	241337	0.98	+	.	ID=MALK_01808.t1.e1;Parent=MALK_01808.t1
contig006	maker	gene	241529	242497	.	+	.	ID=MALK_01809;prediction_source=maker_MRET:augustus_masked-contig006-processed-gene-2.70-mRNA-1
contig006	maker	CDS	241529	242497	.	+	0	ID=MALK_01809.t1.c1;Parent=MALK_01809.t1
contig006	maker	mRNA	241529	242497	.	+	.	ID=MALK_01809.t1;Parent=MALK_01809
contig006	maker	exon	241529	242497	.	+	.	ID=MALK_01809.t1.e1;Parent=MALK_01809.t1

contig006	AUGUSTUS	gene	242471	243245	0.92	-	.	ID=MALK_01810;prediction_source=braker_MRET:g1817.t1
contig006	AUGUSTUS	CDS	242656	243245	0.96	-	0	ID=MALK_01810.t1.c2;Parent=MALK_01810.t1
contig006	AUGUSTUS	CDS	242471	242627	0.96	-	0	ID=MALK_01810.t1.c1;Parent=MALK_01810.t1
contig006	AUGUSTUS	mRNA	242471	243245	0.92	-	.	ID=MALK_01810.t1;Parent=MALK_01810
contig006	AUGUSTUS	exon	242656	243245	.	-	.	ID=MALK_01810.t1.e2;Parent=MALK_01810.t1
contig006	AUGUSTUS	exon	242471	242627	.	-	.	ID=MALK_01810.t1.e1;Parent=MALK_01810.t1
contig006	AUGUSTUS	gene	243391	244350	0.62	+	.	ID=MALK_01811;prediction_source=braker_MRET:g1818.t1
contig006	AUGUSTUS	CDS	243391	244350	0.62	+	0	ID=MALK_01811.t1.c1;Parent=MALK_01811.t1
contig006	AUGUSTUS	mRNA	243391	244350	0.62	+	.	ID=MALK_01811.t1;Parent=MALK_01811
contig006	AUGUSTUS	exon	243391	244350	.	+	.	ID=MALK_01811.t1.e1;Parent=MALK_01811.t1
contig006	AUGUSTUS	gene	244430	246712	0.49	+	.	ID=MALK_01812;prediction_source=braker_MRET:g1819.t1
contig006	AUGUSTUS	CDS	244430	246712	0.49	+	0	ID=MALK_01812.t1.c1;Parent=MALK_01812.t1
contig006	AUGUSTUS	mRNA	244430	246712	0.49	+	.	ID=MALK_01812.t1;Parent=MALK_01812
contig006	AUGUSTUS	exon	244430	246712	.	+	.	ID=MALK_01812.t1.e1;Parent=MALK_01812.t1
contig006	AUGUSTUS	gene	246732	247468	0.21	-	.	ID=MALK_01813;prediction_source=augustus:contig006.g2078.t1
contig006	AUGUSTUS	CDS	247387	247468	0.27	-	0	ID=MALK_01813.t1.c2;Parent=MALK_01813.t1
contig006	AUGUSTUS	CDS	246732	247249	0.27	-	0	ID=MALK_01813.t1.c1;Parent=MALK_01813.t1
contig006	AUGUSTUS	mRNA	246732	247468	0.21	-	.	ID=MALK_01813.t1;Parent=MALK_01813
contig006	AUGUSTUS	exon	247387	247468	0.27	-	.	ID=MALK_01813.t1.e2;Parent=MALK_01813.t1
contig006	AUGUSTUS	exon	246732	247249	0.27	-	.	ID=MALK_01813.t1.e1;Parent=MALK_01813.t1
contig006	AUGUSTUS	gene	247467	249029	1	+	.	ID=MALK_01814;prediction_source=braker_MRET:g1821.t1
contig006	AUGUSTUS	CDS	247467	249029	1	+	0	ID=MALK_01814.t1.c1;Parent=MALK_01814.t1
contig006	AUGUSTUS	mRNA	247467	249029	1	+	.	ID=MALK_01814.t1;Parent=MALK_01814
contig006	AUGUSTUS	exon	247467	249029	.	+	.	ID=MALK_01814.t1.e1;Parent=MALK_01814.t1
contig006	maker	gene	248941	249951	.	-	.	ID=MALK_01815;prediction_source=maker_MRET:augustus_masked-contig006-processed-gene-2.101-mRNA-1
contig006	maker	CDS	248941	249951	.	-	0	ID=MALK_01815.t1.c1;Parent=MALK_01815.t1
contig006	maker	mRNA	248941	249951	.	-	.	ID=MALK_01815.t1;Parent=MALK_01815
contig006	maker	exon	248941	249951	.	-	.	ID=MALK_01815.t1.e1;Parent=MALK_01815.t1
contig006	AUGUSTUS	gene	250104	252752	0.89	+	.	ID=MALK_01816;prediction_source=braker_MRET:g1823.t1
contig006	AUGUSTUS	CDS	250104	252752	0.89	+	0	ID=MALK_01816.t1.c1;Parent=MALK_01816.t1
contig006	AUGUSTUS	mRNA	250104	252752	0.89	+	.	ID=MALK_01816.t1;Parent=MALK_01816
contig006	AUGUSTUS	exon	250104	252752	.	+	.	ID=MALK_01816.t1.e1;Parent=MALK_01816.t1
contig006	AUGUSTUS	gene	252823	254472	0.33	+	.	ID=MALK_01817;prediction_source=augustus:contig006.g2081.t1
contig006	AUGUSTUS	CDS	252823	254472	0.33	+	0	ID=MALK_01817.t1.c1;Parent=MALK_01817.t1
contig006	AUGUSTUS	mRNA	252823	254472	0.33	+	.	ID=MALK_01817.t1;Parent=MALK_01817
contig006	AUGUSTUS	exon	252823	254472	0.33	+	.	ID=MALK_01817.t1.e1;Parent=MALK_01817.t1
contig006	maker	gene	254432	257002	.	-	.	ID=MALK_01818;prediction_source=maker_MRET:augustus_masked-contig006-processed-gene-2.102-mRNA-1
contig006	maker	CDS	254432	257002	.	-	0	ID=MALK_01818.t1.c1;Parent=MALK_01818.t1
contig006	maker	mRNA	254432	257002	.	-	.	ID=MALK_01818.t1;Parent=MALK_01818
contig006	maker	exon	254432	257002	.	-	.	ID=MALK_01818.t1.e1;Parent=MALK_01818.t1
contig006	AUGUSTUS	gene	257043	258212	0.91	-	.	ID=MALK_01819;prediction_source=augustus:contig006.g2084.t1
contig006	AUGUSTUS	CDS	257043	258212	0.91	-	0	ID=MALK_01819.t1.c1;Parent=MALK_01819.t1
contig006	AUGUSTUS	mRNA	257043	258212	0.91	-	.	ID=MALK_01819.t1;Parent=MALK_01819

contig006	AUGUSTUS	exon	257043	258212	0.91	-	.	ID=MALK_01819.t1.e1;Parent=MALK_01819.t1
contig006	AUGUSTUS	gene	258281	259143	0.23	-	.	ID=MALK_01820;prediction_source=braker_MRET:g1827.t1
contig006	AUGUSTUS	CDS	259038	259143	0.54	-	0	ID=MALK_01820.t1.c3;Parent=MALK_01820.t1
contig006	AUGUSTUS	CDS	258903	259006	0.54	-	0	ID=MALK_01820.t1.c2;Parent=MALK_01820.t1
contig006	AUGUSTUS	CDS	258281	258859	0.54	-	0	ID=MALK_01820.t1.c1;Parent=MALK_01820.t1
contig006	AUGUSTUS	mRNA	258281	259143	0.23	-	.	ID=MALK_01820.t1;Parent=MALK_01820
contig006	AUGUSTUS	exon	259038	259143	.	-	.	ID=MALK_01820.t1.e3;Parent=MALK_01820.t1
contig006	AUGUSTUS	exon	258903	259006	.	-	.	ID=MALK_01820.t1.e2;Parent=MALK_01820.t1
contig006	AUGUSTUS	exon	258281	258859	.	-	.	ID=MALK_01820.t1.e1;Parent=MALK_01820.t1
contig006	maker	gene	259245	260990	.	+	.	ID=MALK_01821;prediction_source=maker_MRET:augustus_masked-contig006-processed-gene-2.73-mRNA-1
contig006	maker	CDS	259245	260990	.	+	0	ID=MALK_01821.t1.c1;Parent=MALK_01821.t1
contig006	maker	mRNA	259245	260990	.	+	.	ID=MALK_01821.t1;Parent=MALK_01821
contig006	maker	exon	259245	260990	.	+	.	ID=MALK_01821.t1.e1;Parent=MALK_01821.t1
contig006	AUGUSTUS	gene	261020	261928	1	-	.	ID=MALK_01822;prediction_source=braker_MRET:g1829.t1
contig006	AUGUSTUS	CDS	261020	261928	1	-	0	ID=MALK_01822.t1.c1;Parent=MALK_01822.t1
contig006	AUGUSTUS	mRNA	261020	261928	1	-	.	ID=MALK_01822.t1;Parent=MALK_01822
contig006	AUGUSTUS	exon	261020	261928	.	-	.	ID=MALK_01822.t1.e1;Parent=MALK_01822.t1
contig006	AUGUSTUS	gene	262050	263597	0.72	+	.	ID=MALK_01823;prediction_source=braker_MRET:g1830.t1
contig006	AUGUSTUS	CDS	262050	263597	0.72	+	0	ID=MALK_01823.t1.c1;Parent=MALK_01823.t1
contig006	AUGUSTUS	mRNA	262050	263597	0.72	+	.	ID=MALK_01823.t1;Parent=MALK_01823
contig006	AUGUSTUS	exon	262050	263597	.	+	.	ID=MALK_01823.t1.e1;Parent=MALK_01823.t1
contig006	AUGUSTUS	gene	263741	264379	0.92	-	.	ID=MALK_01824;prediction_source=braker_MRET:g1831.t1
contig006	AUGUSTUS	CDS	264352	264379	0.94	-	0	ID=MALK_01824.t1.c2;Parent=MALK_01824.t1
contig006	AUGUSTUS	CDS	263741	264321	0.94	-	0	ID=MALK_01824.t1.c1;Parent=MALK_01824.t1
contig006	AUGUSTUS	mRNA	263741	264379	0.92	-	.	ID=MALK_01824.t1;Parent=MALK_01824
contig006	AUGUSTUS	exon	264352	264379	.	-	.	ID=MALK_01824.t1.e2;Parent=MALK_01824.t1
contig006	AUGUSTUS	exon	263741	264321	.	-	.	ID=MALK_01824.t1.e1;Parent=MALK_01824.t1
contig006	AUGUSTUS	gene	264639	266916	0.78	+	.	ID=MALK_01825;prediction_source=braker_MRET:g1832.t1
contig006	AUGUSTUS	CDS	264639	264808	1	+	0	ID=MALK_01825.t1.c1;Parent=MALK_01825.t1
contig006	AUGUSTUS	CDS	264852	266916	1	+	0	ID=MALK_01825.t1.c2;Parent=MALK_01825.t1
contig006	AUGUSTUS	mRNA	264639	266916	0.78	+	.	ID=MALK_01825.t1;Parent=MALK_01825
contig006	AUGUSTUS	exon	264639	264808	.	+	.	ID=MALK_01825.t1.e1;Parent=MALK_01825.t1
contig006	AUGUSTUS	exon	264852	266916	.	+	.	ID=MALK_01825.t1.e2;Parent=MALK_01825.t1
contig006	AUGUSTUS	gene	267207	269576	1	+	.	ID=MALK_01826;prediction_source=augustus:contig006.g2092.t1
contig006	AUGUSTUS	CDS	267207	269576	1	+	0	ID=MALK_01826.t1.c1;Parent=MALK_01826.t1
contig006	AUGUSTUS	mRNA	267207	269576	1	+	.	ID=MALK_01826.t1;Parent=MALK_01826
contig006	AUGUSTUS	exon	267207	269576	1	+	.	ID=MALK_01826.t1.e1;Parent=MALK_01826.t1
contig006	AUGUSTUS	gene	269689	271476	0.86	+	.	ID=MALK_01827;prediction_source=augustus:contig006.g2093.t1
contig006	AUGUSTUS	CDS	269689	271476	0.86	+	0	ID=MALK_01827.t1.c1;Parent=MALK_01827.t1
contig006	AUGUSTUS	mRNA	269689	271476	0.86	+	.	ID=MALK_01827.t1;Parent=MALK_01827
contig006	AUGUSTUS	exon	269689	271476	0.86	+	.	ID=MALK_01827.t1.e1;Parent=MALK_01827.t1
contig006	maker	gene	271712	273088	.	+	.	ID=MALK_01828;prediction_source=maker_MRET:augustus_masked-contig006-processed-gene-2.78-mRNA-1
contig006	maker	CDS	271712	273088	.	+	0	ID=MALK_01828.t1.c1;Parent=MALK_01828.t1

contig006	maker	mRNA	271712	273088	.	+	.	ID=MALK_01828.t1;Parent=MALK_01828
contig006	maker	exon	271712	273088	.	+	.	ID=MALK_01828.t1.e1;Parent=MALK_01828.t1
contig006	AUGUSTUS	gene	273160	274080	0.94	+	.	ID=MALK_01829;prediction_source=augustus:contig006.g2095.t1
contig006	AUGUSTUS	CDS	273160	274080	0.94	+	0	ID=MALK_01829.t1.c1;Parent=MALK_01829.t1
contig006	AUGUSTUS	mRNA	273160	274080	0.94	+	.	ID=MALK_01829.t1;Parent=MALK_01829
contig006	AUGUSTUS	exon	273160	274080	0.94	+	.	ID=MALK_01829.t1.e1;Parent=MALK_01829.t1
contig006	maker	gene	274510	276348	.	+	.	ID=MALK_01830;prediction_source=maker_MRET:augustus_masked-contig006-processed-gene-2.80-mRNA-1
contig006	maker	CDS	274510	276348	.	+	0	ID=MALK_01830.t1.c1;Parent=MALK_01830.t1
contig006	maker	mRNA	274510	276348	.	+	.	ID=MALK_01830.t1;Parent=MALK_01830
contig006	maker	exon	274510	276348	.	+	.	ID=MALK_01830.t1.e1;Parent=MALK_01830.t1
contig006	AUGUSTUS	gene	276362	278252	0.9	-	.	ID=MALK_01831;prediction_source=braker_MRET:g1837.t1
contig006	AUGUSTUS	CDS	278156	278252	0.95	-	0	ID=MALK_01831.t1.c2;Parent=MALK_01831.t1
contig006	AUGUSTUS	CDS	276362	278121	0.95	-	0	ID=MALK_01831.t1.c1;Parent=MALK_01831.t1
contig006	AUGUSTUS	mRNA	276362	278252	0.9	-	.	ID=MALK_01831.t1;Parent=MALK_01831
contig006	AUGUSTUS	exon	278156	278252	.	-	.	ID=MALK_01831.t1.e2;Parent=MALK_01831.t1
contig006	AUGUSTUS	exon	276362	278121	.	-	.	ID=MALK_01831.t1.e1;Parent=MALK_01831.t1
contig006	maker	gene	278554	280137	.	+	.	ID=MALK_01832;prediction_source=maker_MRET:augustus_masked-contig006-processed-gene-2.81-mRNA-1
contig006	maker	CDS	278554	280137	.	+	0	ID=MALK_01832.t1.c1;Parent=MALK_01832.t1
contig006	maker	mRNA	278554	280137	.	+	.	ID=MALK_01832.t1;Parent=MALK_01832
contig006	maker	exon	278554	280137	.	+	.	ID=MALK_01832.t1.e1;Parent=MALK_01832.t1
contig006	AUGUSTUS	gene	280334	281620	0.83	+	.	ID=MALK_01833;prediction_source=augustus:contig006.g2099.t1
contig006	AUGUSTUS	CDS	280334	281620	0.83	+	0	ID=MALK_01833.t1.c1;Parent=MALK_01833.t1
contig006	AUGUSTUS	mRNA	280334	281620	0.83	+	.	ID=MALK_01833.t1;Parent=MALK_01833
contig006	AUGUSTUS	exon	280334	281620	0.83	+	.	ID=MALK_01833.t1.e1;Parent=MALK_01833.t1
contig006	AUGUSTUS	gene	281658	282827	0.47	+	.	ID=MALK_01834;prediction_source=braker_MRET:g1840.t1
contig006	AUGUSTUS	CDS	281658	282827	0.47	+	0	ID=MALK_01834.t1.c1;Parent=MALK_01834.t1
contig006	AUGUSTUS	mRNA	281658	282827	0.47	+	.	ID=MALK_01834.t1;Parent=MALK_01834
contig006	AUGUSTUS	exon	281658	282827	.	+	.	ID=MALK_01834.t1.e1;Parent=MALK_01834.t1
contig006	AUGUSTUS	gene	282828	284357	0.48	-	.	ID=MALK_01835;prediction_source=augustus:contig006.g2100.t1
contig006	AUGUSTUS	CDS	282828	284357	0.48	-	0	ID=MALK_01835.t1.c1;Parent=MALK_01835.t1
contig006	AUGUSTUS	mRNA	282828	284357	0.48	-	.	ID=MALK_01835.t1;Parent=MALK_01835
contig006	AUGUSTUS	exon	282828	284357	0.48	-	.	ID=MALK_01835.t1.e1;Parent=MALK_01835.t1
contig006	AUGUSTUS	gene	284454	285255	1	-	.	ID=MALK_01836;prediction_source=braker_MRET:g1842.t2
contig006	AUGUSTUS	CDS	284619	285255	1	-	0	ID=MALK_01836.t1.c2;Parent=MALK_01836.t1
contig006	AUGUSTUS	CDS	284454	284572	1	-	0	ID=MALK_01836.t1.c1;Parent=MALK_01836.t1
contig006	AUGUSTUS	mRNA	284454	285255	1	-	.	ID=MALK_01836.t1;Parent=MALK_01836
contig006	AUGUSTUS	exon	284619	285255	.	-	.	ID=MALK_01836.t1.e2;Parent=MALK_01836.t1
contig006	AUGUSTUS	exon	284454	284572	.	-	.	ID=MALK_01836.t1.e1;Parent=MALK_01836.t1
contig006	AUGUSTUS	gene	285301	286431	0.44	+	.	ID=MALK_01837;prediction_source=augustus:contig006.g2103.t1
contig006	AUGUSTUS	CDS	285301	286431	0.44	+	0	ID=MALK_01837.t1.c1;Parent=MALK_01837.t1
contig006	AUGUSTUS	mRNA	285301	286431	0.44	+	.	ID=MALK_01837.t1;Parent=MALK_01837
contig006	AUGUSTUS	exon	285301	286431	0.44	+	.	ID=MALK_01837.t1.e1;Parent=MALK_01837.t1
contig006	maker	gene	286564	287121	.	-	.	ID=MALK_01838;prediction_source=maker_MRET:augustus_masked-contig006-processed-gene-2.110-mRNA-1

contig006	maker	CDS	286564	287121	.	-	0	ID=MALK_01838.t1.c1;Parent=MALK_01838.t1
contig006	maker	mRNA	286564	287121	.	-	.	ID=MALK_01838.t1;Parent=MALK_01838
contig006	maker	exon	286564	287121	.	-	.	ID=MALK_01838.t1.e1;Parent=MALK_01838.t1
contig006	maker	gene	287401	289374	.	-	.	ID=MALK_01839;prediction_source=maker_MRET:augustus_masked-contig006-processed-gene-2.111-mRNA-1
contig006	maker	CDS	287401	289374	.	-	0	ID=MALK_01839.t1.c1;Parent=MALK_01839.t1
contig006	maker	mRNA	287401	289374	.	-	.	ID=MALK_01839.t1;Parent=MALK_01839
contig006	maker	exon	287401	289374	.	-	.	ID=MALK_01839.t1.e1;Parent=MALK_01839.t1
contig006	maker	gene	289426	290775	.	+	.	ID=MALK_01840;prediction_source=maker_MRET:augustus_masked-contig006-processed-gene-2.84-mRNA-1
contig006	maker	CDS	289426	290775	.	+	0	ID=MALK_01840.t1.c1;Parent=MALK_01840.t1
contig006	maker	mRNA	289426	290775	.	+	.	ID=MALK_01840.t1;Parent=MALK_01840
contig006	maker	exon	289426	290775	.	+	.	ID=MALK_01840.t1.e1;Parent=MALK_01840.t1
contig006	AUGUSTUS	gene	291305	292873	0.84	-	.	ID=MALK_01841;prediction_source=augustus:contig006.g2106.t1
contig006	AUGUSTUS	CDS	291305	292873	0.84	-	0	ID=MALK_01841.t1.c1;Parent=MALK_01841.t1
contig006	AUGUSTUS	mRNA	291305	292873	0.84	-	.	ID=MALK_01841.t1;Parent=MALK_01841
contig006	AUGUSTUS	exon	291305	292873	0.84	-	.	ID=MALK_01841.t1.e1;Parent=MALK_01841.t1
contig006	AUGUSTUS	gene	292947	293711	0.94	-	.	ID=MALK_01842;prediction_source=braker_MRET:g1847.t1
contig006	AUGUSTUS	CDS	292947	293711	0.94	-	0	ID=MALK_01842.t1.c1;Parent=MALK_01842.t1
contig006	AUGUSTUS	mRNA	292947	293711	0.94	-	.	ID=MALK_01842.t1;Parent=MALK_01842
contig006	AUGUSTUS	exon	292947	293711	.	-	.	ID=MALK_01842.t1.e1;Parent=MALK_01842.t1
contig006	AUGUSTUS	gene	293758	295866	0.94	-	.	ID=MALK_01843;prediction_source=braker_MRET:g1848.t1
contig006	AUGUSTUS	CDS	293758	295866	0.94	-	0	ID=MALK_01843.t1.c1;Parent=MALK_01843.t1
contig006	AUGUSTUS	mRNA	293758	295866	0.94	-	.	ID=MALK_01843.t1;Parent=MALK_01843
contig006	AUGUSTUS	exon	293758	295866	.	-	.	ID=MALK_01843.t1.e1;Parent=MALK_01843.t1
contig006	AUGUSTUS	gene	296003	296980	0.99	+	.	ID=MALK_01844;prediction_source=braker_MRET:g1849.t1
contig006	AUGUSTUS	CDS	296003	296980	0.99	+	0	ID=MALK_01844.t1.c1;Parent=MALK_01844.t1
contig006	AUGUSTUS	mRNA	296003	296980	0.99	+	.	ID=MALK_01844.t1;Parent=MALK_01844
contig006	AUGUSTUS	exon	296003	296980	.	+	.	ID=MALK_01844.t1.e1;Parent=MALK_01844.t1
contig006	AUGUSTUS	gene	297006	299086	0.95	-	.	ID=MALK_01845;prediction_source=braker_MRET:g1850.t1
contig006	AUGUSTUS	CDS	297058	299086	0.95	-	0	ID=MALK_01845.t1.c2;Parent=MALK_01845.t1
contig006	AUGUSTUS	CDS	297006	297022	0.95	-	0	ID=MALK_01845.t1.c1;Parent=MALK_01845.t1
contig006	AUGUSTUS	mRNA	297006	299086	0.95	-	.	ID=MALK_01845.t1;Parent=MALK_01845
contig006	AUGUSTUS	exon	297058	299086	.	-	.	ID=MALK_01845.t1.e2;Parent=MALK_01845.t1
contig006	AUGUSTUS	exon	297006	297022	.	-	.	ID=MALK_01845.t1.e1;Parent=MALK_01845.t1
contig006	AUGUSTUS	gene	299104	300204	0.95	-	.	ID=MALK_01846;prediction_source=braker_MRET:g1851.t1
contig006	AUGUSTUS	CDS	299104	300204	0.95	-	0	ID=MALK_01846.t1.c1;Parent=MALK_01846.t1
contig006	AUGUSTUS	mRNA	299104	300204	0.95	-	.	ID=MALK_01846.t1;Parent=MALK_01846
contig006	AUGUSTUS	exon	299104	300204	.	-	.	ID=MALK_01846.t1.e1;Parent=MALK_01846.t1
contig006	AUGUSTUS	gene	300265	303354	0.07	-	.	ID=MALK_01847;prediction_source=braker_MRET:g1852.t1
contig006	AUGUSTUS	CDS	303154	303354	0.51	-	0	ID=MALK_01847.t1.c4;Parent=MALK_01847.t1
contig006	AUGUSTUS	CDS	302586	303121	0.51	-	0	ID=MALK_01847.t1.c3;Parent=MALK_01847.t1
contig006	AUGUSTUS	CDS	302443	302553	0.51	-	0	ID=MALK_01847.t1.c2;Parent=MALK_01847.t1
contig006	AUGUSTUS	CDS	300265	302257	0.51	-	0	ID=MALK_01847.t1.c1;Parent=MALK_01847.t1
contig006	AUGUSTUS	mRNA	300265	303354	0.07	-	.	ID=MALK_01847.t1;Parent=MALK_01847

contig006	AUGUSTUS	exon	303154	303354	.	-	.	ID=MALK_01847.t1.e4;Parent=MALK_01847.t1
contig006	AUGUSTUS	exon	302586	303121	.	-	.	ID=MALK_01847.t1.e3;Parent=MALK_01847.t1
contig006	AUGUSTUS	exon	302443	302553	.	-	.	ID=MALK_01847.t1.e2;Parent=MALK_01847.t1
contig006	AUGUSTUS	exon	300265	302257	.	-	.	ID=MALK_01847.t1.e1;Parent=MALK_01847.t1
contig006	AUGUSTUS	gene	303353	307240	0.98	+	.	ID=MALK_01848;prediction_source=augustus:contig006.g2111.t1
contig006	AUGUSTUS	CDS	303353	307240	0.98	+	0	ID=MALK_01848.t1.c1;Parent=MALK_01848.t1
contig006	AUGUSTUS	mRNA	303353	307240	0.98	+	.	ID=MALK_01848.t1;Parent=MALK_01848
contig006	AUGUSTUS	exon	303353	307240	0.98	+	.	ID=MALK_01848.t1.e1;Parent=MALK_01848.t1
contig006	AUGUSTUS	gene	307284	308795	0.6	-	.	ID=MALK_01849;prediction_source=braker_MRET:g1854.t1
contig006	AUGUSTUS	CDS	308380	308795	0.6	-	0	ID=MALK_01849.t1.c2;Parent=MALK_01849.t1
contig006	AUGUSTUS	CDS	307284	308304	0.6	-	0	ID=MALK_01849.t1.c1;Parent=MALK_01849.t1
contig006	AUGUSTUS	mRNA	307284	308795	0.6	-	.	ID=MALK_01849.t1;Parent=MALK_01849
contig006	AUGUSTUS	exon	308380	308795	.	-	.	ID=MALK_01849.t1.e2;Parent=MALK_01849.t1
contig006	AUGUSTUS	exon	307284	308304	.	-	.	ID=MALK_01849.t1.e1;Parent=MALK_01849.t1
contig006	AUGUSTUS	gene	308858	309931	0.95	+	.	ID=MALK_01850;prediction_source=augustus:contig006.g2113.t1
contig006	AUGUSTUS	CDS	308858	309931	0.95	+	0	ID=MALK_01850.t1.c1;Parent=MALK_01850.t1
contig006	AUGUSTUS	mRNA	308858	309931	0.95	+	.	ID=MALK_01850.t1;Parent=MALK_01850
contig006	AUGUSTUS	exon	308858	309931	0.95	+	.	ID=MALK_01850.t1.e1;Parent=MALK_01850.t1
contig006	AUGUSTUS	gene	309909	310667	0.69	-	.	ID=MALK_01851;prediction_source=braker_MRET:g1856.t1
contig006	AUGUSTUS	CDS	309909	310667	0.69	-	0	ID=MALK_01851.t1.c1;Parent=MALK_01851.t1
contig006	AUGUSTUS	mRNA	309909	310667	0.69	-	.	ID=MALK_01851.t1;Parent=MALK_01851
contig006	AUGUSTUS	exon	309909	310667	.	-	.	ID=MALK_01851.t1.e1;Parent=MALK_01851.t1
contig006	AUGUSTUS	gene	310687	311351	0.78	-	.	ID=MALK_01852;prediction_source=braker_MRET:g1857.t1
contig006	AUGUSTUS	CDS	311103	311351	0.99	-	0	ID=MALK_01852.t1.c2;Parent=MALK_01852.t1
contig006	AUGUSTUS	CDS	310687	311073	0.99	-	0	ID=MALK_01852.t1.c1;Parent=MALK_01852.t1
contig006	AUGUSTUS	mRNA	310687	311351	0.78	-	.	ID=MALK_01852.t1;Parent=MALK_01852
contig006	AUGUSTUS	exon	311103	311351	.	-	.	ID=MALK_01852.t1.e2;Parent=MALK_01852.t1
contig006	AUGUSTUS	exon	310687	311073	.	-	.	ID=MALK_01852.t1.e1;Parent=MALK_01852.t1
contig006	AUGUSTUS	gene	311455	312570	0.65	-	.	ID=MALK_01853;prediction_source=braker_MRET:g1858.t1
contig006	AUGUSTUS	CDS	311455	312570	0.65	-	0	ID=MALK_01853.t1.c1;Parent=MALK_01853.t1
contig006	AUGUSTUS	mRNA	311455	312570	0.65	-	.	ID=MALK_01853.t1;Parent=MALK_01853
contig006	AUGUSTUS	exon	311455	312570	.	-	.	ID=MALK_01853.t1.e1;Parent=MALK_01853.t1
contig006	AUGUSTUS	gene	312718	313548	0.77	-	.	ID=MALK_01854;prediction_source=braker_MRET:g1859.t1
contig006	AUGUSTUS	CDS	312718	313548	0.77	-	0	ID=MALK_01854.t1.c1;Parent=MALK_01854.t1
contig006	AUGUSTUS	mRNA	312718	313548	0.77	-	.	ID=MALK_01854.t1;Parent=MALK_01854
contig006	AUGUSTUS	exon	312718	313548	.	-	.	ID=MALK_01854.t1.e1;Parent=MALK_01854.t1
contig006	AUGUSTUS	gene	313660	315441	0.99	+	.	ID=MALK_01855;prediction_source=augustus:contig006.g2115.t1
contig006	AUGUSTUS	CDS	313660	315441	0.99	+	0	ID=MALK_01855.t1.c1;Parent=MALK_01855.t1
contig006	AUGUSTUS	mRNA	313660	315441	0.99	+	.	ID=MALK_01855.t1;Parent=MALK_01855
contig006	AUGUSTUS	exon	313660	315441	0.99	+	.	ID=MALK_01855.t1.e1;Parent=MALK_01855.t1
contig006	AUGUSTUS	gene	315490	316839	0.91	-	.	ID=MALK_01856;prediction_source=augustus:contig006.g2116.t1
contig006	AUGUSTUS	CDS	315490	316839	0.91	-	0	ID=MALK_01856.t1.c1;Parent=MALK_01856.t1
contig006	AUGUSTUS	mRNA	315490	316839	0.91	-	.	ID=MALK_01856.t1;Parent=MALK_01856

contig006	AUGUSTUS	exon	315490	316839	0.91	-	.	ID=MALK_01856.t1.e1;Parent=MALK_01856.t1
contig006	AUGUSTUS	gene	317676	319082	0.98	-	.	ID=MALK_01857;prediction_source=augustus:contig006.g2117.t1
contig006	AUGUSTUS	CDS	317676	319082	0.98	-	0	ID=MALK_01857.t1.c1;Parent=MALK_01857.t1
contig006	AUGUSTUS	mRNA	317676	319082	0.98	-	.	ID=MALK_01857.t1;Parent=MALK_01857
contig006	AUGUSTUS	exon	317676	319082	0.98	-	.	ID=MALK_01857.t1.e1;Parent=MALK_01857.t1
contig006	maker	gene	319377	322406	.	+	.	ID=MALK_01858;prediction_source=maker_MRET:augustus_masked-contig006-processed-gene-3.71-mRNA-1
contig006	maker	CDS	319377	322406	.	+	0	ID=MALK_01858.t1.c1;Parent=MALK_01858.t1
contig006	maker	mRNA	319377	322406	.	+	.	ID=MALK_01858.t1;Parent=MALK_01858
contig006	maker	exon	319377	322406	.	+	.	ID=MALK_01858.t1.e1;Parent=MALK_01858.t1
contig006	AUGUSTUS	gene	322413	323752	0.29	-	.	ID=MALK_01859;prediction_source=braker_MRET:g1864.t1
contig006	AUGUSTUS	CDS	323687	323752	0.29	-	0	ID=MALK_01859.t1.c3;Parent=MALK_01859.t1
contig006	AUGUSTUS	CDS	323618	323647	0.29	-	0	ID=MALK_01859.t1.c2;Parent=MALK_01859.t1
contig006	AUGUSTUS	CDS	322413	323585	0.29	-	0	ID=MALK_01859.t1.c1;Parent=MALK_01859.t1
contig006	AUGUSTUS	mRNA	322413	323752	0.29	-	.	ID=MALK_01859.t1;Parent=MALK_01859
contig006	AUGUSTUS	exon	323687	323752	.	-	.	ID=MALK_01859.t1.e3;Parent=MALK_01859.t1
contig006	AUGUSTUS	exon	323618	323647	.	-	.	ID=MALK_01859.t1.e2;Parent=MALK_01859.t1
contig006	AUGUSTUS	exon	322413	323585	.	-	.	ID=MALK_01859.t1.e1;Parent=MALK_01859.t1
contig006	AUGUSTUS	gene	324149	324847	0.34	-	.	ID=MALK_01860;prediction_source=braker_MRET:g1865.t1
contig006	AUGUSTUS	CDS	324149	324847	0.34	-	0	ID=MALK_01860.t1.c1;Parent=MALK_01860.t1
contig006	AUGUSTUS	mRNA	324149	324847	0.34	-	.	ID=MALK_01860.t1;Parent=MALK_01860
contig006	AUGUSTUS	exon	324149	324847	.	-	.	ID=MALK_01860.t1.e1;Parent=MALK_01860.t1
contig006	maker	gene	325297	327832	.	+	.	ID=MALK_01861;prediction_source=maker_MRET:augustus_masked-contig006-processed-gene-3.72-mRNA-1
contig006	maker	CDS	325297	326317	.	+	0	ID=MALK_01861.t1.c1;Parent=MALK_01861.t1
contig006	maker	CDS	326460	327832	.	+	0	ID=MALK_01861.t1.c2;Parent=MALK_01861.t1
contig006	maker	mRNA	325297	327832	.	+	.	ID=MALK_01861.t1;Parent=MALK_01861
contig006	maker	exon	325297	326317	.	+	.	ID=MALK_01861.t1.e1;Parent=MALK_01861.t1
contig006	maker	exon	326460	327832	.	+	.	ID=MALK_01861.t1.e2;Parent=MALK_01861.t1
contig006	AUGUSTUS	gene	327988	328806	0.99	-	.	ID=MALK_01862;prediction_source=braker_MRET:g1867.t1
contig006	AUGUSTUS	CDS	327988	328806	0.99	-	0	ID=MALK_01862.t1.c1;Parent=MALK_01862.t1
contig006	AUGUSTUS	mRNA	327988	328806	0.99	-	.	ID=MALK_01862.t1;Parent=MALK_01862
contig006	AUGUSTUS	exon	327988	328806	.	-	.	ID=MALK_01862.t1.e1;Parent=MALK_01862.t1
contig006	AUGUSTUS	gene	328872	329590	0.49	-	.	ID=MALK_01863;prediction_source=braker_MRET:g1868.t1
contig006	AUGUSTUS	CDS	329378	329590	0.5	-	0	ID=MALK_01863.t1.c2;Parent=MALK_01863.t1
contig006	AUGUSTUS	CDS	328872	329285	0.5	-	0	ID=MALK_01863.t1.c1;Parent=MALK_01863.t1
contig006	AUGUSTUS	mRNA	328872	329590	0.49	-	.	ID=MALK_01863.t1;Parent=MALK_01863
contig006	AUGUSTUS	exon	329378	329590	.	-	.	ID=MALK_01863.t1.e2;Parent=MALK_01863.t1
contig006	AUGUSTUS	exon	328872	329285	.	-	.	ID=MALK_01863.t1.e1;Parent=MALK_01863.t1
contig006	AUGUSTUS	gene	329795	331643	0.11	+	.	ID=MALK_01864;prediction_source=braker_MRET:g1869.t1
contig006	AUGUSTUS	CDS	329795	330136	0.97	+	0	ID=MALK_01864.t1.c1;Parent=MALK_01864.t1
contig006	AUGUSTUS	CDS	330327	330354	0.97	+	0	ID=MALK_01864.t1.c2;Parent=MALK_01864.t1
contig006	AUGUSTUS	CDS	330395	330543	0.97	+	0	ID=MALK_01864.t1.c3;Parent=MALK_01864.t1
contig006	AUGUSTUS	CDS	330573	330655	0.97	+	0	ID=MALK_01864.t1.c4;Parent=MALK_01864.t1
contig006	AUGUSTUS	CDS	330684	330742	0.97	+	0	ID=MALK_01864.t1.c5;Parent=MALK_01864.t1

contig006	AUGUSTUS	CDS	330774	331574	0.97	+	0	ID=MALK_01864.t1.c6;Parent=MALK_01864.t1
contig006	AUGUSTUS	CDS	331609	331643	0.97	+	0	ID=MALK_01864.t1.c7;Parent=MALK_01864.t1
contig006	AUGUSTUS	mRNA	329795	331643	0.11	+	.	ID=MALK_01864.t1;Parent=MALK_01864
contig006	AUGUSTUS	exon	329795	330136	.	+	.	ID=MALK_01864.t1.e1;Parent=MALK_01864.t1
contig006	AUGUSTUS	exon	330327	330354	.	+	.	ID=MALK_01864.t1.e2;Parent=MALK_01864.t1
contig006	AUGUSTUS	exon	330395	330543	.	+	.	ID=MALK_01864.t1.e3;Parent=MALK_01864.t1
contig006	AUGUSTUS	exon	330573	330655	.	+	.	ID=MALK_01864.t1.e4;Parent=MALK_01864.t1
contig006	AUGUSTUS	exon	330684	330742	.	+	.	ID=MALK_01864.t1.e5;Parent=MALK_01864.t1
contig006	AUGUSTUS	exon	330774	331574	.	+	.	ID=MALK_01864.t1.e6;Parent=MALK_01864.t1
contig006	AUGUSTUS	exon	331609	331643	.	+	.	ID=MALK_01864.t1.e7;Parent=MALK_01864.t1
contig006	AUGUSTUS	gene	331820	332361	0.61	+	.	ID=MALK_01865;prediction_source=braker_MRET:g1870.t1
contig006	AUGUSTUS	CDS	331820	331914	0.88	+	0	ID=MALK_01865.t1.c1;Parent=MALK_01865.t1
contig006	AUGUSTUS	CDS	331959	331979	0.88	+	0	ID=MALK_01865.t1.c2;Parent=MALK_01865.t1
contig006	AUGUSTUS	CDS	332019	332127	0.88	+	0	ID=MALK_01865.t1.c3;Parent=MALK_01865.t1
contig006	AUGUSTUS	CDS	332287	332361	0.88	+	0	ID=MALK_01865.t1.c4;Parent=MALK_01865.t1
contig006	AUGUSTUS	mRNA	331820	332361	0.61	+	.	ID=MALK_01865.t1;Parent=MALK_01865
contig006	AUGUSTUS	exon	331820	331914	.	+	.	ID=MALK_01865.t1.e1;Parent=MALK_01865.t1
contig006	AUGUSTUS	exon	331959	331979	.	+	.	ID=MALK_01865.t1.e2;Parent=MALK_01865.t1
contig006	AUGUSTUS	exon	332019	332127	.	+	.	ID=MALK_01865.t1.e3;Parent=MALK_01865.t1
contig006	AUGUSTUS	exon	332287	332361	.	+	.	ID=MALK_01865.t1.e4;Parent=MALK_01865.t1
contig006	AUGUSTUS	gene	332762	333478	1	+	.	ID=MALK_01866;prediction_source=augustus:contig006.g2125.t1
contig006	AUGUSTUS	CDS	332762	333478	1	+	0	ID=MALK_01866.t1.c1;Parent=MALK_01866.t1
contig006	AUGUSTUS	mRNA	332762	333478	1	+	.	ID=MALK_01866.t1;Parent=MALK_01866
contig006	AUGUSTUS	exon	332762	333478	1	+	.	ID=MALK_01866.t1.e1;Parent=MALK_01866.t1
contig006	AUGUSTUS	gene	333484	334498	0.92	-	.	ID=MALK_01867;prediction_source=braker_MRET:g1872.t1
contig006	AUGUSTUS	CDS	333981	334498	0.94	-	0	ID=MALK_01867.t1.c2;Parent=MALK_01867.t1
contig006	AUGUSTUS	CDS	333484	333952	0.94	-	0	ID=MALK_01867.t1.c1;Parent=MALK_01867.t1
contig006	AUGUSTUS	mRNA	333484	334498	0.92	-	.	ID=MALK_01867.t1;Parent=MALK_01867
contig006	AUGUSTUS	exon	333981	334498	.	-	.	ID=MALK_01867.t1.e2;Parent=MALK_01867.t1
contig006	AUGUSTUS	exon	333484	333952	.	-	.	ID=MALK_01867.t1.e1;Parent=MALK_01867.t1
contig006	AUGUSTUS	gene	334609	335298	0.98	+	.	ID=MALK_01868;prediction_source=augustus:contig006.g2127.t1
contig006	AUGUSTUS	CDS	334609	335298	0.98	+	0	ID=MALK_01868.t1.c1;Parent=MALK_01868.t1
contig006	AUGUSTUS	mRNA	334609	335298	0.98	+	.	ID=MALK_01868.t1;Parent=MALK_01868
contig006	AUGUSTUS	exon	334609	335298	0.98	+	.	ID=MALK_01868.t1.e1;Parent=MALK_01868.t1
contig006	AUGUSTUS	gene	335281	336561	1	-	.	ID=MALK_01869;prediction_source=augustus:contig006.g2128.t1
contig006	AUGUSTUS	CDS	335281	336561	1	-	0	ID=MALK_01869.t1.c1;Parent=MALK_01869.t1
contig006	AUGUSTUS	mRNA	335281	336561	1	-	.	ID=MALK_01869.t1;Parent=MALK_01869
contig006	AUGUSTUS	exon	335281	336561	1	-	.	ID=MALK_01869.t1.e1;Parent=MALK_01869.t1
contig006	AUGUSTUS	gene	336727	337326	1	+	.	ID=MALK_01870;prediction_source=augustus:contig006.g2129.t1
contig006	AUGUSTUS	CDS	336727	337326	1	+	0	ID=MALK_01870.t1.c1;Parent=MALK_01870.t1
contig006	AUGUSTUS	mRNA	336727	337326	1	+	.	ID=MALK_01870.t1;Parent=MALK_01870
contig006	AUGUSTUS	exon	336727	337326	1	+	.	ID=MALK_01870.t1.e1;Parent=MALK_01870.t1
contig006	AUGUSTUS	gene	337337	339634	0.96	-	.	ID=MALK_01871;prediction_source=augustus:contig006.g2130.t1

contig006	AUGUSTUS	CDS	337337	339634	0.96	-	0	ID=MALK_01871.t1.c1;Parent=MALK_01871.t1
contig006	AUGUSTUS	mRNA	337337	339634	0.96	-	.	ID=MALK_01871.t1;Parent=MALK_01871
contig006	AUGUSTUS	exon	337337	339634	0.96	-	.	ID=MALK_01871.t1.e1;Parent=MALK_01871.t1
contig006	AUGUSTUS	gene	339782	341608	1	-	.	ID=MALK_01872;prediction_source=braker_MRET:g1877.t1
contig006	AUGUSTUS	CDS	339782	341608	1	-	0	ID=MALK_01872.t1.c1;Parent=MALK_01872.t1
contig006	AUGUSTUS	mRNA	339782	341608	1	-	.	ID=MALK_01872.t1;Parent=MALK_01872
contig006	AUGUSTUS	exon	339782	341608	.	-	.	ID=MALK_01872.t1.e1;Parent=MALK_01872.t1
contig006	AUGUSTUS	gene	341760	343550	0.95	+	.	ID=MALK_01873;prediction_source=augustus:contig006.g2132.t1
contig006	AUGUSTUS	CDS	341760	343550	0.95	+	0	ID=MALK_01873.t1.c1;Parent=MALK_01873.t1
contig006	AUGUSTUS	mRNA	341760	343550	0.95	+	.	ID=MALK_01873.t1;Parent=MALK_01873
contig006	AUGUSTUS	exon	341760	343550	0.95	+	.	ID=MALK_01873.t1.e1;Parent=MALK_01873.t1
contig006	AUGUSTUS	gene	343547	345271	0.83	-	.	ID=MALK_01874;prediction_source=augustus:contig006.g2133.t1
contig006	AUGUSTUS	CDS	343547	345271	0.83	-	0	ID=MALK_01874.t1.c1;Parent=MALK_01874.t1
contig006	AUGUSTUS	mRNA	343547	345271	0.83	-	.	ID=MALK_01874.t1;Parent=MALK_01874
contig006	AUGUSTUS	exon	343547	345271	0.83	-	.	ID=MALK_01874.t1.e1;Parent=MALK_01874.t1
contig006	AUGUSTUS	gene	345549	346667	0.95	+	.	ID=MALK_01875;prediction_source=augustus:contig006.g2135.t1
contig006	AUGUSTUS	CDS	345549	346667	0.95	+	0	ID=MALK_01875.t1.c1;Parent=MALK_01875.t1
contig006	AUGUSTUS	mRNA	345549	346667	0.95	+	.	ID=MALK_01875.t1;Parent=MALK_01875
contig006	AUGUSTUS	exon	345549	346667	0.95	+	.	ID=MALK_01875.t1.e1;Parent=MALK_01875.t1
contig006	AUGUSTUS	gene	346949	347484	0.67	-	.	ID=MALK_01876;prediction_source=braker_MRET:g1881.t1
contig006	AUGUSTUS	CDS	347182	347484	0.74	-	0	ID=MALK_01876.t1.c3;Parent=MALK_01876.t1
contig006	AUGUSTUS	CDS	347025	347136	0.74	-	0	ID=MALK_01876.t1.c2;Parent=MALK_01876.t1
contig006	AUGUSTUS	CDS	346949	346995	0.74	-	0	ID=MALK_01876.t1.c1;Parent=MALK_01876.t1
contig006	AUGUSTUS	mRNA	346949	347484	0.67	-	.	ID=MALK_01876.t1;Parent=MALK_01876
contig006	AUGUSTUS	exon	347182	347484	.	-	.	ID=MALK_01876.t1.e3;Parent=MALK_01876.t1
contig006	AUGUSTUS	exon	347025	347136	.	-	.	ID=MALK_01876.t1.e2;Parent=MALK_01876.t1
contig006	AUGUSTUS	exon	346949	346995	.	-	.	ID=MALK_01876.t1.e1;Parent=MALK_01876.t1
contig006	AUGUSTUS	gene	348693	349130	0.52	-	.	ID=MALK_01877;prediction_source=augustus:contig006.g2137.t1
contig006	AUGUSTUS	CDS	348693	349130	0.52	-	0	ID=MALK_01877.t1.c1;Parent=MALK_01877.t1
contig006	AUGUSTUS	mRNA	348693	349130	0.52	-	.	ID=MALK_01877.t1;Parent=MALK_01877
contig006	AUGUSTUS	exon	348693	349130	0.52	-	.	ID=MALK_01877.t1.e1;Parent=MALK_01877.t1
contig006	AUGUSTUS	gene	349264	349519	0.94	+	.	ID=MALK_01878;prediction_source=braker_MRET:g1883.t1
contig006	AUGUSTUS	CDS	349264	349360	0.97	+	0	ID=MALK_01878.t1.c1;Parent=MALK_01878.t1
contig006	AUGUSTUS	CDS	349401	349519	0.97	+	0	ID=MALK_01878.t1.c2;Parent=MALK_01878.t1
contig006	AUGUSTUS	mRNA	349264	349519	0.94	+	.	ID=MALK_01878.t1;Parent=MALK_01878
contig006	AUGUSTUS	exon	349264	349360	.	+	.	ID=MALK_01878.t1.e1;Parent=MALK_01878.t1
contig006	AUGUSTUS	exon	349401	349519	.	+	.	ID=MALK_01878.t1.e2;Parent=MALK_01878.t1
contig006	AUGUSTUS	gene	349951	350545	0.44	-	.	ID=MALK_01879;prediction_source=braker_MRET:g1884.t1
contig006	AUGUSTUS	CDS	350495	350545	0.44	-	0	ID=MALK_01879.t1.c3;Parent=MALK_01879.t1
contig006	AUGUSTUS	CDS	350223	350359	0.44	-	0	ID=MALK_01879.t1.c2;Parent=MALK_01879.t1
contig006	AUGUSTUS	CDS	349951	350182	0.44	-	0	ID=MALK_01879.t1.c1;Parent=MALK_01879.t1
contig006	AUGUSTUS	mRNA	349951	350545	0.44	-	.	ID=MALK_01879.t1;Parent=MALK_01879
contig006	AUGUSTUS	exon	350495	350545	.	-	.	ID=MALK_01879.t1.e3;Parent=MALK_01879.t1

contig006	AUGUSTUS	exon	350223	350359	.	-	.	ID=MALK_01879.t1.e2;Parent=MALK_01879.t1
contig006	AUGUSTUS	exon	349951	350182	.	-	.	ID=MALK_01879.t1.e1;Parent=MALK_01879.t1
contig006	maker	gene	350718	351818	.	-	.	ID=MALK_01880;prediction_source=maker_MRET:augustus_masked-contig006-processed-gene-3.99-mRNA-1
contig006	maker	CDS	350718	351818	.	-	0	ID=MALK_01880.t1.c1;Parent=MALK_01880.t1
contig006	maker	mRNA	350718	351818	.	-	.	ID=MALK_01880.t1;Parent=MALK_01880
contig006	maker	exon	350718	351818	.	-	.	ID=MALK_01880.t1.e1;Parent=MALK_01880.t1
contig006	AUGUSTUS	gene	352412	353060	0.98	+	.	ID=MALK_01881;prediction_source=braker_MRET:g1886.t1
contig006	AUGUSTUS	CDS	352412	352427	1	+	0	ID=MALK_01881.t1.c1;Parent=MALK_01881.t1
contig006	AUGUSTUS	CDS	352460	352603	1	+	0	ID=MALK_01881.t1.c2;Parent=MALK_01881.t1
contig006	AUGUSTUS	CDS	352639	353060	1	+	0	ID=MALK_01881.t1.c3;Parent=MALK_01881.t1
contig006	AUGUSTUS	mRNA	352412	353060	0.98	+	.	ID=MALK_01881.t1;Parent=MALK_01881
contig006	AUGUSTUS	exon	352412	352427	.	+	.	ID=MALK_01881.t1.e1;Parent=MALK_01881.t1
contig006	AUGUSTUS	exon	352460	352603	.	+	.	ID=MALK_01881.t1.e2;Parent=MALK_01881.t1
contig006	AUGUSTUS	exon	352639	353060	.	+	.	ID=MALK_01881.t1.e3;Parent=MALK_01881.t1
contig006	AUGUSTUS	gene	353188	355119	1	-	.	ID=MALK_01882;prediction_source=augustus:contig006.g2139.t1
contig006	AUGUSTUS	CDS	353188	355119	1	-	0	ID=MALK_01882.t1.c1;Parent=MALK_01882.t1
contig006	AUGUSTUS	mRNA	353188	355119	1	-	.	ID=MALK_01882.t1;Parent=MALK_01882
contig006	AUGUSTUS	exon	353188	355119	1	-	.	ID=MALK_01882.t1.e1;Parent=MALK_01882.t1
contig006	AUGUSTUS	gene	355486	357246	0.7	+	.	ID=MALK_01883;prediction_source=augustus:contig006.g2140.t1
contig006	AUGUSTUS	CDS	355486	357246	0.7	+	0	ID=MALK_01883.t1.c1;Parent=MALK_01883.t1
contig006	AUGUSTUS	mRNA	355486	357246	0.7	+	.	ID=MALK_01883.t1;Parent=MALK_01883
contig006	AUGUSTUS	exon	355486	357246	0.7	+	.	ID=MALK_01883.t1.e1;Parent=MALK_01883.t1
contig006	maker	gene	357250	359055	.	-	.	ID=MALK_01884;prediction_source=maker_MRET:augustus_masked-contig006-processed-gene-3.101-mRNA-1
contig006	maker	CDS	357250	359055	.	-	0	ID=MALK_01884.t1.c1;Parent=MALK_01884.t1
contig006	maker	mRNA	357250	359055	.	-	.	ID=MALK_01884.t1;Parent=MALK_01884
contig006	maker	exon	357250	359055	.	-	.	ID=MALK_01884.t1.e1;Parent=MALK_01884.t1
contig006	maker	gene	359147	360481	.	-	.	ID=MALK_01885;prediction_source=maker_MRET:augustus_masked-contig006-processed-gene-3.102-mRNA-1
contig006	maker	CDS	359147	360481	.	-	0	ID=MALK_01885.t1.c1;Parent=MALK_01885.t1
contig006	maker	mRNA	359147	360481	.	-	.	ID=MALK_01885.t1;Parent=MALK_01885
contig006	maker	exon	359147	360481	.	-	.	ID=MALK_01885.t1.e1;Parent=MALK_01885.t1
contig006	AUGUSTUS	gene	360543	361449	0.47	+	.	ID=MALK_01886;prediction_source=augustus:contig006.g2143.t1
contig006	AUGUSTUS	CDS	360543	360923	0.85	+	0	ID=MALK_01886.t1.c1;Parent=MALK_01886.t1
contig006	AUGUSTUS	CDS	361066	361449	0.85	+	0	ID=MALK_01886.t1.c2;Parent=MALK_01886.t1
contig006	AUGUSTUS	mRNA	360543	361449	0.47	+	.	ID=MALK_01886.t1;Parent=MALK_01886
contig006	AUGUSTUS	exon	360543	360923	0.85	+	.	ID=MALK_01886.t1.e1;Parent=MALK_01886.t1
contig006	AUGUSTUS	exon	361066	361449	0.85	+	.	ID=MALK_01886.t1.e2;Parent=MALK_01886.t1
contig006	AUGUSTUS	gene	361450	362526	0.95	-	.	ID=MALK_01887;prediction_source=augustus:contig006.g2144.t1
contig006	AUGUSTUS	CDS	361450	362526	0.95	-	0	ID=MALK_01887.t1.c1;Parent=MALK_01887.t1
contig006	AUGUSTUS	mRNA	361450	362526	0.95	-	.	ID=MALK_01887.t1;Parent=MALK_01887
contig006	AUGUSTUS	exon	361450	362526	0.95	-	.	ID=MALK_01887.t1.e1;Parent=MALK_01887.t1
contig006	AUGUSTUS	gene	362802	363479	0.33	-	.	ID=MALK_01888;prediction_source=braker_MRET:g1892.t1
contig006	AUGUSTUS	CDS	363021	363479	0.72	-	0	ID=MALK_01888.t1.c4;Parent=MALK_01888.t1
contig006	AUGUSTUS	CDS	362943	362974	0.72	-	0	ID=MALK_01888.t1.c3;Parent=MALK_01888.t1

contig006	AUGUSTUS	CDS	362892	362910	0.72	-	0	ID=MALK_01888.t1.c2;Parent=MALK_01888.t1
contig006	AUGUSTUS	CDS	362802	362852	0.72	-	0	ID=MALK_01888.t1.c1;Parent=MALK_01888.t1
contig006	AUGUSTUS	mRNA	362802	363479	0.33	-	.	ID=MALK_01888.t1;Parent=MALK_01888
contig006	AUGUSTUS	exon	363021	363479	.	-	.	ID=MALK_01888.t1.e4;Parent=MALK_01888.t1
contig006	AUGUSTUS	exon	362943	362974	.	-	.	ID=MALK_01888.t1.e3;Parent=MALK_01888.t1
contig006	AUGUSTUS	exon	362892	362910	.	-	.	ID=MALK_01888.t1.e2;Parent=MALK_01888.t1
contig006	AUGUSTUS	exon	362802	362852	.	-	.	ID=MALK_01888.t1.e1;Parent=MALK_01888.t1
contig006	maker	gene	363593	364057	.	+	.	ID=MALK_01889;prediction_source=maker_MRET:augustus_masked-contig006-processed-gene-3.80-mRNA-1
contig006	maker	CDS	363593	364057	.	+	0	ID=MALK_01889.t1.c1;Parent=MALK_01889.t1
contig006	maker	mRNA	363593	364057	.	+	.	ID=MALK_01889.t1;Parent=MALK_01889
contig006	maker	exon	363593	364057	.	+	.	ID=MALK_01889.t1.e1;Parent=MALK_01889.t1
contig006	AUGUSTUS	gene	364054	365695	0.15	-	.	ID=MALK_01890;prediction_source=braker_MRET:g1894.t1
contig006	AUGUSTUS	CDS	364872	365695	0.72	-	0	ID=MALK_01890.t1.c5;Parent=MALK_01890.t1
contig006	AUGUSTUS	CDS	364837	364841	0.72	-	0	ID=MALK_01890.t1.c4;Parent=MALK_01890.t1
contig006	AUGUSTUS	CDS	364727	364797	0.72	-	0	ID=MALK_01890.t1.c3;Parent=MALK_01890.t1
contig006	AUGUSTUS	CDS	364586	364682	0.72	-	0	ID=MALK_01890.t1.c2;Parent=MALK_01890.t1
contig006	AUGUSTUS	CDS	364054	364538	0.72	-	0	ID=MALK_01890.t1.c1;Parent=MALK_01890.t1
contig006	AUGUSTUS	mRNA	364054	365695	0.15	-	.	ID=MALK_01890.t1;Parent=MALK_01890
contig006	AUGUSTUS	exon	364872	365695	.	-	.	ID=MALK_01890.t1.e5;Parent=MALK_01890.t1
contig006	AUGUSTUS	exon	364837	364841	.	-	.	ID=MALK_01890.t1.e4;Parent=MALK_01890.t1
contig006	AUGUSTUS	exon	364727	364797	.	-	.	ID=MALK_01890.t1.e3;Parent=MALK_01890.t1
contig006	AUGUSTUS	exon	364586	364682	.	-	.	ID=MALK_01890.t1.e2;Parent=MALK_01890.t1
contig006	AUGUSTUS	exon	364054	364538	.	-	.	ID=MALK_01890.t1.e1;Parent=MALK_01890.t1
contig006	AUGUSTUS	gene	365961	366562	0.55	-	.	ID=MALK_01891;prediction_source=braker_MRET:g1895.t1
contig006	AUGUSTUS	CDS	366474	366562	0.63	-	0	ID=MALK_01891.t1.c4;Parent=MALK_01891.t1
contig006	AUGUSTUS	CDS	366310	366432	0.63	-	0	ID=MALK_01891.t1.c3;Parent=MALK_01891.t1
contig006	AUGUSTUS	CDS	366044	366268	0.63	-	0	ID=MALK_01891.t1.c2;Parent=MALK_01891.t1
contig006	AUGUSTUS	CDS	365961	366000	0.63	-	0	ID=MALK_01891.t1.c1;Parent=MALK_01891.t1
contig006	AUGUSTUS	mRNA	365961	366562	0.55	-	.	ID=MALK_01891.t1;Parent=MALK_01891
contig006	AUGUSTUS	exon	366474	366562	.	-	.	ID=MALK_01891.t1.e4;Parent=MALK_01891.t1
contig006	AUGUSTUS	exon	366310	366432	.	-	.	ID=MALK_01891.t1.e3;Parent=MALK_01891.t1
contig006	AUGUSTUS	exon	366044	366268	.	-	.	ID=MALK_01891.t1.e2;Parent=MALK_01891.t1
contig006	AUGUSTUS	exon	365961	366000	.	-	.	ID=MALK_01891.t1.e1;Parent=MALK_01891.t1
contig006	AUGUSTUS	gene	367096	367749	0.79	+	.	ID=MALK_01892;prediction_source=braker_MRET:g1896.t1
contig006	AUGUSTUS	CDS	367096	367749	0.79	+	0	ID=MALK_01892.t1.c1;Parent=MALK_01892.t1
contig006	AUGUSTUS	mRNA	367096	367749	0.79	+	.	ID=MALK_01892.t1;Parent=MALK_01892
contig006	AUGUSTUS	exon	367096	367749	.	+	.	ID=MALK_01892.t1.e1;Parent=MALK_01892.t1
contig006	AUGUSTUS	gene	367761	368209	0.54	-	.	ID=MALK_01893;prediction_source=braker_MRET:g1897.t1
contig006	AUGUSTUS	CDS	368158	368209	0.54	-	0	ID=MALK_01893.t1.c2;Parent=MALK_01893.t1
contig006	AUGUSTUS	CDS	367761	368116	0.54	-	0	ID=MALK_01893.t1.c1;Parent=MALK_01893.t1
contig006	AUGUSTUS	mRNA	367761	368209	0.54	-	.	ID=MALK_01893.t1;Parent=MALK_01893
contig006	AUGUSTUS	exon	368158	368209	.	-	.	ID=MALK_01893.t1.e2;Parent=MALK_01893.t1
contig006	AUGUSTUS	exon	367761	368116	.	-	.	ID=MALK_01893.t1.e1;Parent=MALK_01893.t1

contig006	maker	gene	368301	369674	.	-	.	ID=MALK_01894;prediction_source=maker_MRET:augustus_masked-contig006-processed-gene-3.107-mRNA-1
contig006	maker	CDS	368301	369674	.	-	0	ID=MALK_01894.t1.c1;Parent=MALK_01894.t1
contig006	maker	mRNA	368301	369674	.	-	.	ID=MALK_01894.t1;Parent=MALK_01894
contig006	maker	exon	368301	369674	.	-	.	ID=MALK_01894.t1.e1;Parent=MALK_01894.t1
contig006	AUGUSTUS	gene	369812	371296	0.52	+	.	ID=MALK_01895;prediction_source=augustus:contig006.g2152.t1
contig006	AUGUSTUS	CDS	369812	371296	0.52	+	0	ID=MALK_01895.t1.c1;Parent=MALK_01895.t1
contig006	AUGUSTUS	mRNA	369812	371296	0.52	+	.	ID=MALK_01895.t1;Parent=MALK_01895
contig006	AUGUSTUS	exon	369812	371296	0.52	+	.	ID=MALK_01895.t1.e1;Parent=MALK_01895.t1
contig006	AUGUSTUS	gene	371340	372773	0.92	+	.	ID=MALK_01896;prediction_source=augustus:contig006.g2153.t1
contig006	AUGUSTUS	CDS	371340	372773	0.92	+	0	ID=MALK_01896.t1.c1;Parent=MALK_01896.t1
contig006	AUGUSTUS	mRNA	371340	372773	0.92	+	.	ID=MALK_01896.t1;Parent=MALK_01896
contig006	AUGUSTUS	exon	371340	372773	0.92	+	.	ID=MALK_01896.t1.e1;Parent=MALK_01896.t1
contig006	maker	gene	372770	373471	.	-	.	ID=MALK_01897;prediction_source=maker_MRET:augustus_masked-contig006-processed-gene-3.108-mRNA-1
contig006	maker	CDS	372770	373471	.	-	0	ID=MALK_01897.t1.c1;Parent=MALK_01897.t1
contig006	maker	mRNA	372770	373471	.	-	.	ID=MALK_01897.t1;Parent=MALK_01897
contig006	maker	exon	372770	373471	.	-	.	ID=MALK_01897.t1.e1;Parent=MALK_01897.t1
contig006	maker	gene	373535	374500	.	+	.	ID=MALK_01898;prediction_source=maker_MRET:augustus_masked-contig006-processed-gene-3.82-mRNA-1
contig006	maker	CDS	373535	374500	.	+	0	ID=MALK_01898.t1.c1;Parent=MALK_01898.t1
contig006	maker	mRNA	373535	374500	.	+	.	ID=MALK_01898.t1;Parent=MALK_01898
contig006	maker	exon	373535	374500	.	+	.	ID=MALK_01898.t1.e1;Parent=MALK_01898.t1
contig006	AUGUSTUS	gene	374787	375113	1	-	.	ID=MALK_01899;prediction_source=augustus:contig006.g2156.t1
contig006	AUGUSTUS	CDS	374787	375113	1	-	0	ID=MALK_01899.t1.c1;Parent=MALK_01899.t1
contig006	AUGUSTUS	mRNA	374787	375113	1	-	.	ID=MALK_01899.t1;Parent=MALK_01899
contig006	AUGUSTUS	exon	374787	375113	1	-	.	ID=MALK_01899.t1.e1;Parent=MALK_01899.t1
contig006	maker	gene	375291	377150	.	-	.	ID=MALK_01900;prediction_source=maker_MRET:augustus_masked-contig006-processed-gene-3.110-mRNA-1
contig006	maker	CDS	375291	377150	.	-	0	ID=MALK_01900.t1.c1;Parent=MALK_01900.t1
contig006	maker	mRNA	375291	377150	.	-	.	ID=MALK_01900.t1;Parent=MALK_01900
contig006	maker	exon	375291	377150	.	-	.	ID=MALK_01900.t1.e1;Parent=MALK_01900.t1
contig006	maker	gene	377242	378390	.	-	.	ID=MALK_01901;prediction_source=maker_MRET:augustus_masked-contig006-processed-gene-3.111-mRNA-1
contig006	maker	CDS	377242	378390	.	-	0	ID=MALK_01901.t1.c1;Parent=MALK_01901.t1
contig006	maker	mRNA	377242	378390	.	-	.	ID=MALK_01901.t1;Parent=MALK_01901
contig006	maker	exon	377242	378390	.	-	.	ID=MALK_01901.t1.e1;Parent=MALK_01901.t1
contig006	AUGUSTUS	gene	378520	380466	0.96	+	.	ID=MALK_01902;prediction_source=augustus:contig006.g2160.t1
contig006	AUGUSTUS	CDS	378520	380466	0.96	+	0	ID=MALK_01902.t1.c1;Parent=MALK_01902.t1
contig006	AUGUSTUS	mRNA	378520	380466	0.96	+	.	ID=MALK_01902.t1;Parent=MALK_01902
contig006	AUGUSTUS	exon	378520	380466	0.96	+	.	ID=MALK_01902.t1.e1;Parent=MALK_01902.t1
contig006	AUGUSTUS	gene	380529	381357	0.82	+	.	ID=MALK_01903;prediction_source=braker_MRET:g1905.t1
contig006	AUGUSTUS	CDS	380529	380625	1	+	0	ID=MALK_01903.t1.c1;Parent=MALK_01903.t1
contig006	AUGUSTUS	CDS	380654	381357	1	+	0	ID=MALK_01903.t1.c2;Parent=MALK_01903.t1
contig006	AUGUSTUS	mRNA	380529	381357	0.82	+	.	ID=MALK_01903.t1;Parent=MALK_01903
contig006	AUGUSTUS	exon	380529	380625	.	+	.	ID=MALK_01903.t1.e1;Parent=MALK_01903.t1
contig006	AUGUSTUS	exon	380654	381357	.	+	.	ID=MALK_01903.t1.e2;Parent=MALK_01903.t1
contig006	AUGUSTUS	gene	381502	383031	0.8	+	.	ID=MALK_01904;prediction_source=augustus:contig006.g2161.t1

contig006	AUGUSTUS	CDS	381502	383031	0.8	+	0	ID=MALK_01904.t1.c1;Parent=MALK_01904.t1
contig006	AUGUSTUS	mRNA	381502	383031	0.8	+	.	ID=MALK_01904.t1;Parent=MALK_01904
contig006	AUGUSTUS	exon	381502	383031	0.8	+	.	ID=MALK_01904.t1.e1;Parent=MALK_01904.t1
contig006	AUGUSTUS	gene	383000	384154	0.55	-	.	ID=MALK_01905;prediction_source=braker_MRET:g1907.t1
contig006	AUGUSTUS	CDS	383000	384154	0.55	-	0	ID=MALK_01905.t1.c1;Parent=MALK_01905.t1
contig006	AUGUSTUS	mRNA	383000	384154	0.55	-	.	ID=MALK_01905.t1;Parent=MALK_01905
contig006	AUGUSTUS	exon	383000	384154	.	-	.	ID=MALK_01905.t1.e1;Parent=MALK_01905.t1
contig006	AUGUSTUS	gene	384168	386144	0.44	+	.	ID=MALK_01906;prediction_source=braker_MRET:g1908.t1
contig006	AUGUSTUS	CDS	384168	386144	0.44	+	0	ID=MALK_01906.t1.c1;Parent=MALK_01906.t1
contig006	AUGUSTUS	mRNA	384168	386144	0.44	+	.	ID=MALK_01906.t1;Parent=MALK_01906
contig006	AUGUSTUS	exon	384168	386144	.	+	.	ID=MALK_01906.t1.e1;Parent=MALK_01906.t1
contig006	AUGUSTUS	gene	386193	386975	1	+	.	ID=MALK_01907;prediction_source=braker_MRET:g1909.t1
contig006	AUGUSTUS	CDS	386193	386975	1	+	0	ID=MALK_01907.t1.c1;Parent=MALK_01907.t1
contig006	AUGUSTUS	mRNA	386193	386975	1	+	.	ID=MALK_01907.t1;Parent=MALK_01907
contig006	AUGUSTUS	exon	386193	386975	.	+	.	ID=MALK_01907.t1.e1;Parent=MALK_01907.t1
contig006	AUGUSTUS	gene	386976	387992	0.93	-	.	ID=MALK_01908;prediction_source=augustus:contig006.g2164.t1
contig006	AUGUSTUS	CDS	386976	387992	0.93	-	0	ID=MALK_01908.t1.c1;Parent=MALK_01908.t1
contig006	AUGUSTUS	mRNA	386976	387992	0.93	-	.	ID=MALK_01908.t1;Parent=MALK_01908
contig006	AUGUSTUS	exon	386976	387992	0.93	-	.	ID=MALK_01908.t1.e1;Parent=MALK_01908.t1
contig006	AUGUSTUS	gene	388099	389946	1	+	.	ID=MALK_01909;prediction_source=braker_MRET:g1911.t1
contig006	AUGUSTUS	CDS	388099	389946	1	+	0	ID=MALK_01909.t1.c1;Parent=MALK_01909.t1
contig006	AUGUSTUS	mRNA	388099	389946	1	+	.	ID=MALK_01909.t1;Parent=MALK_01909
contig006	AUGUSTUS	exon	388099	389946	.	+	.	ID=MALK_01909.t1.e1;Parent=MALK_01909.t1
contig006	AUGUSTUS	gene	389969	390613	0.94	+	.	ID=MALK_01910;prediction_source=braker_MRET:g1912.t1
contig006	AUGUSTUS	CDS	389969	390613	0.94	+	0	ID=MALK_01910.t1.c1;Parent=MALK_01910.t1
contig006	AUGUSTUS	mRNA	389969	390613	0.94	+	.	ID=MALK_01910.t1;Parent=MALK_01910
contig006	AUGUSTUS	exon	389969	390613	.	+	.	ID=MALK_01910.t1.e1;Parent=MALK_01910.t1
contig006	AUGUSTUS	gene	390643	392289	0.94	-	.	ID=MALK_01911;prediction_source=augustus:contig006.g2167.t1
contig006	AUGUSTUS	CDS	391824	392289	0.95	-	0	ID=MALK_01911.t1.c2;Parent=MALK_01911.t1
contig006	AUGUSTUS	CDS	390643	391670	0.95	-	0	ID=MALK_01911.t1.c1;Parent=MALK_01911.t1
contig006	AUGUSTUS	mRNA	390643	392289	0.94	-	.	ID=MALK_01911.t1;Parent=MALK_01911
contig006	AUGUSTUS	exon	391824	392289	0.95	-	.	ID=MALK_01911.t1.e2;Parent=MALK_01911.t1
contig006	AUGUSTUS	exon	390643	391670	0.95	-	.	ID=MALK_01911.t1.e1;Parent=MALK_01911.t1
contig006	maker	gene	392408	394789	.	+	.	ID=MALK_01912;prediction_source=maker_MRET:augustus_masked-contig006-processed-gene-4.0-mRNA-1
contig006	maker	CDS	392408	394789	.	+	0	ID=MALK_01912.t1.c1;Parent=MALK_01912.t1
contig006	maker	mRNA	392408	394789	.	+	.	ID=MALK_01912.t1;Parent=MALK_01912
contig006	maker	exon	392408	394789	.	+	.	ID=MALK_01912.t1.e1;Parent=MALK_01912.t1
contig006	AUGUSTUS	gene	394806	398243	0.96	+	.	ID=MALK_01913;prediction_source=braker_MRET:g1915.t1
contig006	AUGUSTUS	CDS	394806	398243	0.96	+	0	ID=MALK_01913.t1.c1;Parent=MALK_01913.t1
contig006	AUGUSTUS	mRNA	394806	398243	0.96	+	.	ID=MALK_01913.t1;Parent=MALK_01913
contig006	AUGUSTUS	exon	394806	398243	.	+	.	ID=MALK_01913.t1.e1;Parent=MALK_01913.t1
contig006	maker	gene	398250	398963	.	-	.	ID=MALK_01914;prediction_source=maker_MRET:augustus_masked-contig006-processed-gene-4.1-mRNA-1
contig006	maker	CDS	398250	398963	.	-	0	ID=MALK_01914.t1.c1;Parent=MALK_01914.t1

contig006	maker	mRNA	398250	398963	.	-	.	ID=MALK_01914.t1;Parent=MALK_01914
contig006	maker	exon	398250	398963	.	-	.	ID=MALK_01914.t1.e1;Parent=MALK_01914.t1
contig006	maker	gene	399010	400545	.	-	.	ID=MALK_01915;prediction_source=maker_MRET:augustus_masked-contig006-processed-gene-4.2-mRNA-1
contig006	maker	CDS	399010	400545	.	-	0	ID=MALK_01915.t1.c1;Parent=MALK_01915.t1
contig006	maker	mRNA	399010	400545	.	-	.	ID=MALK_01915.t1;Parent=MALK_01915
contig006	maker	exon	399010	400545	.	-	.	ID=MALK_01915.t1.e1;Parent=MALK_01915.t1
contig006	AUGUSTUS	gene	400686	401744	0.52	+	.	ID=MALK_01916;prediction_source=braker_MRET:g1918.t1
contig006	AUGUSTUS	CDS	400686	401744	0.52	+	0	ID=MALK_01916.t1.c1;Parent=MALK_01916.t1
contig006	AUGUSTUS	mRNA	400686	401744	0.52	+	.	ID=MALK_01916.t1;Parent=MALK_01916
contig006	AUGUSTUS	exon	400686	401744	.	+	.	ID=MALK_01916.t1.e1;Parent=MALK_01916.t1
contig006	AUGUSTUS	gene	401817	402800	0.9	+	.	ID=MALK_01917;prediction_source=augustus:contig006.g2172.t1
contig006	AUGUSTUS	CDS	401817	402800	0.9	+	0	ID=MALK_01917.t1.c1;Parent=MALK_01917.t1
contig006	AUGUSTUS	mRNA	401817	402800	0.9	+	.	ID=MALK_01917.t1;Parent=MALK_01917
contig006	AUGUSTUS	exon	401817	402800	0.9	+	.	ID=MALK_01917.t1.e1;Parent=MALK_01917.t1
contig006	AUGUSTUS	gene	402890	404264	0.97	-	.	ID=MALK_01918;prediction_source=braker_MRET:g1920.t1
contig006	AUGUSTUS	CDS	403086	404264	0.98	-	0	ID=MALK_01918.t1.c2;Parent=MALK_01918.t1
contig006	AUGUSTUS	CDS	402890	403006	0.98	-	0	ID=MALK_01918.t1.c1;Parent=MALK_01918.t1
contig006	AUGUSTUS	mRNA	402890	404264	0.97	-	.	ID=MALK_01918.t1;Parent=MALK_01918
contig006	AUGUSTUS	exon	403086	404264	.	-	.	ID=MALK_01918.t1.e2;Parent=MALK_01918.t1
contig006	AUGUSTUS	exon	402890	403006	.	-	.	ID=MALK_01918.t1.e1;Parent=MALK_01918.t1
contig006	AUGUSTUS	gene	404498	405104	0.46	+	.	ID=MALK_01919;prediction_source=braker_MRET:g1921.t1
contig006	AUGUSTUS	CDS	404498	404976	0.46	+	0	ID=MALK_01919.t1.c1;Parent=MALK_01919.t1
contig006	AUGUSTUS	CDS	405011	405104	0.46	+	0	ID=MALK_01919.t1.c2;Parent=MALK_01919.t1
contig006	AUGUSTUS	mRNA	404498	405104	0.46	+	.	ID=MALK_01919.t1;Parent=MALK_01919
contig006	AUGUSTUS	exon	404498	404976	.	+	.	ID=MALK_01919.t1.e1;Parent=MALK_01919.t1
contig006	AUGUSTUS	exon	405011	405104	.	+	.	ID=MALK_01919.t1.e2;Parent=MALK_01919.t1
contig006	AUGUSTUS	gene	405113	405673	1	-	.	ID=MALK_01920;prediction_source=braker_MRET:g1922.t1
contig006	AUGUSTUS	CDS	405113	405673	1	-	0	ID=MALK_01920.t1.c1;Parent=MALK_01920.t1
contig006	AUGUSTUS	mRNA	405113	405673	1	-	.	ID=MALK_01920.t1;Parent=MALK_01920
contig006	AUGUSTUS	exon	405113	405673	.	-	.	ID=MALK_01920.t1.e1;Parent=MALK_01920.t1
contig006	AUGUSTUS	gene	405801	407564	0.38	+	.	ID=MALK_01921;prediction_source=braker_MRET:g1923.t1
contig006	AUGUSTUS	CDS	405801	406951	0.47	+	0	ID=MALK_01921.t1.c1;Parent=MALK_01921.t1
contig006	AUGUSTUS	CDS	407129	407564	0.47	+	0	ID=MALK_01921.t1.c2;Parent=MALK_01921.t1
contig006	AUGUSTUS	mRNA	405801	407564	0.38	+	.	ID=MALK_01921.t1;Parent=MALK_01921
contig006	AUGUSTUS	exon	405801	406951	.	+	.	ID=MALK_01921.t1.e1;Parent=MALK_01921.t1
contig006	AUGUSTUS	exon	407129	407564	.	+	.	ID=MALK_01921.t1.e2;Parent=MALK_01921.t1
contig006	AUGUSTUS	gene	407640	408398	0.89	-	.	ID=MALK_01922;prediction_source=braker_MRET:g1924.t1
contig006	AUGUSTUS	CDS	407640	408398	0.89	-	0	ID=MALK_01922.t1.c1;Parent=MALK_01922.t1
contig006	AUGUSTUS	mRNA	407640	408398	0.89	-	.	ID=MALK_01922.t1;Parent=MALK_01922
contig006	AUGUSTUS	exon	407640	408398	.	-	.	ID=MALK_01922.t1.e1;Parent=MALK_01922.t1
contig006	AUGUSTUS	gene	408570	411167	0.97	+	.	ID=MALK_01923;prediction_source=braker_MRET:g1925.t1
contig006	AUGUSTUS	CDS	408570	411167	0.97	+	0	ID=MALK_01923.t1.c1;Parent=MALK_01923.t1
contig006	AUGUSTUS	mRNA	408570	411167	0.97	+	.	ID=MALK_01923.t1;Parent=MALK_01923

contig006	AUGUSTUS	exon	408570	411167	.	+	.	ID=MALK_01923.t1.e1;Parent=MALK_01923.t1
contig006	AUGUSTUS	gene	411194	412210	0.85	-	.	ID=MALK_01924;prediction_source=augustus:contig006.g2176.t1
contig006	AUGUSTUS	CDS	411194	412210	0.85	-	0	ID=MALK_01924.t1.c1;Parent=MALK_01924.t1
contig006	AUGUSTUS	mRNA	411194	412210	0.85	-	.	ID=MALK_01924.t1;Parent=MALK_01924
contig006	AUGUSTUS	exon	411194	412210	0.85	-	.	ID=MALK_01924.t1.e1;Parent=MALK_01924.t1
contig006	maker	gene	413253	414434	.	-	.	ID=MALK_01925;prediction_source=maker_MRET:augustus_masked-contig006-processed-gene-4.29-mRNA-1
contig006	maker	CDS	413253	414434	.	-	0	ID=MALK_01925.t1.c1;Parent=MALK_01925.t1
contig006	maker	mRNA	413253	414434	.	-	.	ID=MALK_01925.t1;Parent=MALK_01925
contig006	maker	exon	413253	414434	.	-	.	ID=MALK_01925.t1.e1;Parent=MALK_01925.t1
contig006	maker	gene	414577	415845	.	+	.	ID=MALK_01926;prediction_source=maker_MRET:augustus_masked-contig006-processed-gene-4.8-mRNA-1
contig006	maker	CDS	414577	415098	.	+	0	ID=MALK_01926.t1.c1;Parent=MALK_01926.t1
contig006	maker	CDS	415213	415845	.	+	0	ID=MALK_01926.t1.c2;Parent=MALK_01926.t1
contig006	maker	mRNA	414577	415845	.	+	.	ID=MALK_01926.t1;Parent=MALK_01926
contig006	maker	exon	414577	415098	.	+	.	ID=MALK_01926.t1.e1;Parent=MALK_01926.t1
contig006	maker	exon	415213	415845	.	+	.	ID=MALK_01926.t1.e2;Parent=MALK_01926.t1
contig006	maker	gene	415906	417135	.	+	.	ID=MALK_01927;prediction_source=maker_MRET:augustus_masked-contig006-processed-gene-4.9-mRNA-1
contig006	maker	CDS	415906	417135	.	+	0	ID=MALK_01927.t1.c1;Parent=MALK_01927.t1
contig006	maker	mRNA	415906	417135	.	+	.	ID=MALK_01927.t1;Parent=MALK_01927
contig006	maker	exon	415906	417135	.	+	.	ID=MALK_01927.t1.e1;Parent=MALK_01927.t1
contig006	AUGUSTUS	gene	417163	417614	0.85	-	.	ID=MALK_01928;prediction_source=braker_MRET:g1928.t1
contig006	AUGUSTUS	CDS	417518	417614	0.89	-	0	ID=MALK_01928.t1.c2;Parent=MALK_01928.t1
contig006	AUGUSTUS	CDS	417163	417488	0.89	-	0	ID=MALK_01928.t1.c1;Parent=MALK_01928.t1
contig006	AUGUSTUS	mRNA	417163	417614	0.85	-	.	ID=MALK_01928.t1;Parent=MALK_01928
contig006	AUGUSTUS	exon	417518	417614	.	-	.	ID=MALK_01928.t1.e2;Parent=MALK_01928.t1
contig006	AUGUSTUS	exon	417163	417488	.	-	.	ID=MALK_01928.t1.e1;Parent=MALK_01928.t1
contig006	maker	gene	417682	418644	.	-	.	ID=MALK_01929;prediction_source=maker_MRET:augustus_masked-contig006-processed-gene-4.30-mRNA-1
contig006	maker	CDS	417682	418644	.	-	0	ID=MALK_01929.t1.c1;Parent=MALK_01929.t1
contig006	maker	mRNA	417682	418644	.	-	.	ID=MALK_01929.t1;Parent=MALK_01929
contig006	maker	exon	417682	418644	.	-	.	ID=MALK_01929.t1.e1;Parent=MALK_01929.t1
contig006	maker	gene	418806	420986	.	+	.	ID=MALK_01930;prediction_source=maker_MRET:augustus_masked-contig006-processed-gene-4.10-mRNA-1
contig006	maker	CDS	418806	420986	.	+	0	ID=MALK_01930.t1.c1;Parent=MALK_01930.t1
contig006	maker	mRNA	418806	420986	.	+	.	ID=MALK_01930.t1;Parent=MALK_01930
contig006	maker	exon	418806	420986	.	+	.	ID=MALK_01930.t1.e1;Parent=MALK_01930.t1
contig006	AUGUSTUS	gene	421083	421560	0.56	-	.	ID=MALK_01931;prediction_source=braker_MRET:g1931.t1
contig006	AUGUSTUS	CDS	421479	421560	0.56	-	0	ID=MALK_01931.t1.c3;Parent=MALK_01931.t1
contig006	AUGUSTUS	CDS	421186	421451	0.56	-	0	ID=MALK_01931.t1.c2;Parent=MALK_01931.t1
contig006	AUGUSTUS	CDS	421083	421154	0.56	-	0	ID=MALK_01931.t1.c1;Parent=MALK_01931.t1
contig006	AUGUSTUS	mRNA	421083	421560	0.56	-	.	ID=MALK_01931.t1;Parent=MALK_01931
contig006	AUGUSTUS	exon	421479	421560	.	-	.	ID=MALK_01931.t1.e3;Parent=MALK_01931.t1
contig006	AUGUSTUS	exon	421186	421451	.	-	.	ID=MALK_01931.t1.e2;Parent=MALK_01931.t1
contig006	AUGUSTUS	exon	421083	421154	.	-	.	ID=MALK_01931.t1.e1;Parent=MALK_01931.t1
contig006	maker	gene	421921	423513	.	-	.	ID=MALK_01932;prediction_source=maker_MRET:augustus_masked-contig006-processed-gene-4.32-mRNA-1
contig006	maker	CDS	421921	423513	.	-	0	ID=MALK_01932.t1.c1;Parent=MALK_01932.t1

contig006	maker	mRNA	421921	423513	.	-	.	ID=MALK_01932.t1;Parent=MALK_01932
contig006	maker	exon	421921	423513	.	-	.	ID=MALK_01932.t1.e1;Parent=MALK_01932.t1
contig006	AUGUSTUS	gene	423623	424528	0.99	+	.	ID=MALK_01933;prediction_source=augustus:contig006.g2184.t1
contig006	AUGUSTUS	CDS	423623	424528	0.99	+	0	ID=MALK_01933.t1.c1;Parent=MALK_01933.t1
contig006	AUGUSTUS	mRNA	423623	424528	0.99	+	.	ID=MALK_01933.t1;Parent=MALK_01933
contig006	AUGUSTUS	exon	423623	424528	0.99	+	.	ID=MALK_01933.t1.e1;Parent=MALK_01933.t1
contig006	maker	gene	424530	425837	.	-	.	ID=MALK_01934;prediction_source=maker_MRET:augustus_masked-contig006-processed-gene-4.33-mRNA-1
contig006	maker	CDS	424530	425837	.	-	0	ID=MALK_01934.t1.c1;Parent=MALK_01934.t1
contig006	maker	mRNA	424530	425837	.	-	.	ID=MALK_01934.t1;Parent=MALK_01934
contig006	maker	exon	424530	425837	.	-	.	ID=MALK_01934.t1.e1;Parent=MALK_01934.t1
contig006	AUGUSTUS	gene	425950	426337	1	+	.	ID=MALK_01935;prediction_source=braker_MRET:g1935.t1
contig006	AUGUSTUS	CDS	425950	426187	1	+	0	ID=MALK_01935.t1.c1;Parent=MALK_01935.t1
contig006	AUGUSTUS	CDS	426216	426337	1	+	0	ID=MALK_01935.t1.c2;Parent=MALK_01935.t1
contig006	AUGUSTUS	mRNA	425950	426337	1	+	.	ID=MALK_01935.t1;Parent=MALK_01935
contig006	AUGUSTUS	exon	425950	426187	.	+	.	ID=MALK_01935.t1.e1;Parent=MALK_01935.t1
contig006	AUGUSTUS	exon	426216	426337	.	+	.	ID=MALK_01935.t1.e2;Parent=MALK_01935.t1
contig006	AUGUSTUS	gene	427058	427357	0.99	+	.	ID=MALK_01936;prediction_source=augustus:contig006.g2186.t1
contig006	AUGUSTUS	CDS	427058	427357	0.99	+	0	ID=MALK_01936.t1.c1;Parent=MALK_01936.t1
contig006	AUGUSTUS	mRNA	427058	427357	0.99	+	.	ID=MALK_01936.t1;Parent=MALK_01936
contig006	AUGUSTUS	exon	427058	427357	0.99	+	.	ID=MALK_01936.t1.e1;Parent=MALK_01936.t1
contig006	AUGUSTUS	gene	427508	429829	0.95	-	.	ID=MALK_01937;prediction_source=braker_MRET:g1936.t1
contig006	AUGUSTUS	CDS	427508	429829	0.95	-	0	ID=MALK_01937.t1.c1;Parent=MALK_01937.t1
contig006	AUGUSTUS	mRNA	427508	429829	0.95	-	.	ID=MALK_01937.t1;Parent=MALK_01937
contig006	AUGUSTUS	exon	427508	429829	.	-	.	ID=MALK_01937.t1.e1;Parent=MALK_01937.t1
contig006	AUGUSTUS	gene	430210	431289	1	-	.	ID=MALK_01938;prediction_source=braker_MRET:g1937.t1
contig006	AUGUSTUS	CDS	430210	431289	1	-	0	ID=MALK_01938.t1.c1;Parent=MALK_01938.t1
contig006	AUGUSTUS	mRNA	430210	431289	1	-	.	ID=MALK_01938.t1;Parent=MALK_01938
contig006	AUGUSTUS	exon	430210	431289	.	-	.	ID=MALK_01938.t1.e1;Parent=MALK_01938.t1
contig006	AUGUSTUS	gene	431384	432508	0.46	+	.	ID=MALK_01939;prediction_source=augustus:contig006.g2189.t1
contig006	AUGUSTUS	CDS	431384	432508	0.46	+	0	ID=MALK_01939.t1.c1;Parent=MALK_01939.t1
contig006	AUGUSTUS	mRNA	431384	432508	0.46	+	.	ID=MALK_01939.t1;Parent=MALK_01939
contig006	AUGUSTUS	exon	431384	432508	0.46	+	.	ID=MALK_01939.t1.e1;Parent=MALK_01939.t1
contig006	maker	gene	432587	433361	.	+	.	ID=MALK_01940;prediction_source=maker_MRET:augustus_masked-contig006-processed-gene-4.14-mRNA-1
contig006	maker	CDS	432587	433183	.	+	0	ID=MALK_01940.t1.c1;Parent=MALK_01940.t1
contig006	maker	CDS	433284	433361	.	+	0	ID=MALK_01940.t1.c2;Parent=MALK_01940.t1
contig006	maker	mRNA	432587	433361	.	+	.	ID=MALK_01940.t1;Parent=MALK_01940
contig006	maker	exon	432587	433183	.	+	.	ID=MALK_01940.t1.e1;Parent=MALK_01940.t1
contig006	maker	exon	433284	433361	.	+	.	ID=MALK_01940.t1.e2;Parent=MALK_01940.t1
contig006	AUGUSTUS	gene	433495	434433	0.98	-	.	ID=MALK_01941;prediction_source=augustus:contig006.g2191.t1
contig006	AUGUSTUS	CDS	433495	434433	0.98	-	0	ID=MALK_01941.t1.c1;Parent=MALK_01941.t1
contig006	AUGUSTUS	mRNA	433495	434433	0.98	-	.	ID=MALK_01941.t1;Parent=MALK_01941
contig006	AUGUSTUS	exon	433495	434433	0.98	-	.	ID=MALK_01941.t1.e1;Parent=MALK_01941.t1
contig006	maker	gene	434528	435631	.	-	.	ID=MALK_01942;prediction_source=maker_MRET:augustus_masked-contig006-processed-gene-4.36-mRNA-1

contig006	maker	CDS	434528	435631	.	-	0	ID=MALK_01942.t1.c1;Parent=MALK_01942.t1
contig006	maker	mRNA	434528	435631	.	-	.	ID=MALK_01942.t1;Parent=MALK_01942
contig006	maker	exon	434528	435631	.	-	.	ID=MALK_01942.t1.e1;Parent=MALK_01942.t1
contig006	AUGUSTUS	gene	435917	437884	0.96	-	.	ID=MALK_01943;prediction_source=augustus:contig006.g2193.t1
contig006	AUGUSTUS	CDS	435917	437884	0.96	-	0	ID=MALK_01943.t1.c1;Parent=MALK_01943.t1
contig006	AUGUSTUS	mRNA	435917	437884	0.96	-	.	ID=MALK_01943.t1;Parent=MALK_01943
contig006	AUGUSTUS	exon	435917	437884	0.96	-	.	ID=MALK_01943.t1.e1;Parent=MALK_01943.t1
contig006	maker	gene	438388	439899	.	-	.	ID=MALK_01944;prediction_source=maker_MRET:augustus_masked-contig006-processed-gene-4.38-mRNA-1
contig006	maker	CDS	438388	439899	.	-	0	ID=MALK_01944.t1.c1;Parent=MALK_01944.t1
contig006	maker	mRNA	438388	439899	.	-	.	ID=MALK_01944.t1;Parent=MALK_01944
contig006	maker	exon	438388	439899	.	-	.	ID=MALK_01944.t1.e1;Parent=MALK_01944.t1
contig006	AUGUSTUS	gene	440035	440553	0.72	+	.	ID=MALK_01945;prediction_source=augustus:contig006.g2195.t1
contig006	AUGUSTUS	CDS	440035	440553	0.72	+	0	ID=MALK_01945.t1.c1;Parent=MALK_01945.t1
contig006	AUGUSTUS	mRNA	440035	440553	0.72	+	.	ID=MALK_01945.t1;Parent=MALK_01945
contig006	AUGUSTUS	exon	440035	440553	0.72	+	.	ID=MALK_01945.t1.e1;Parent=MALK_01945.t1
contig006	maker	gene	440618	441475	.	-	.	ID=MALK_01946;prediction_source=maker_MRET:augustus_masked-contig006-processed-gene-4.39-mRNA-1
contig006	maker	CDS	440618	441475	.	-	0	ID=MALK_01946.t1.c1;Parent=MALK_01946.t1
contig006	maker	mRNA	440618	441475	.	-	.	ID=MALK_01946.t1;Parent=MALK_01946
contig006	maker	exon	440618	441475	.	-	.	ID=MALK_01946.t1.e1;Parent=MALK_01946.t1
contig006	AUGUSTUS	gene	441602	443140	0.46	+	.	ID=MALK_01947;prediction_source=augustus:contig006.g2197.t1
contig006	AUGUSTUS	CDS	441602	443140	0.46	+	0	ID=MALK_01947.t1.c1;Parent=MALK_01947.t1
contig006	AUGUSTUS	mRNA	441602	443140	0.46	+	.	ID=MALK_01947.t1;Parent=MALK_01947
contig006	AUGUSTUS	exon	441602	443140	0.46	+	.	ID=MALK_01947.t1.e1;Parent=MALK_01947.t1
contig006	maker	gene	443152	443757	.	-	.	ID=MALK_01948;prediction_source=maker_MRET:augustus_masked-contig006-processed-gene-4.40-mRNA-1
contig006	maker	CDS	443152	443757	.	-	0	ID=MALK_01948.t1.c1;Parent=MALK_01948.t1
contig006	maker	mRNA	443152	443757	.	-	.	ID=MALK_01948.t1;Parent=MALK_01948
contig006	maker	exon	443152	443757	.	-	.	ID=MALK_01948.t1.e1;Parent=MALK_01948.t1
contig006	AUGUSTUS	gene	444030	446147	0.79	+	.	ID=MALK_01949;prediction_source=augustus:contig006.g2199.t1
contig006	AUGUSTUS	CDS	444030	446147	0.79	+	0	ID=MALK_01949.t1.c1;Parent=MALK_01949.t1
contig006	AUGUSTUS	mRNA	444030	446147	0.79	+	.	ID=MALK_01949.t1;Parent=MALK_01949
contig006	AUGUSTUS	exon	444030	446147	0.79	+	.	ID=MALK_01949.t1.e1;Parent=MALK_01949.t1
contig006	maker	gene	446845	448096	.	+	.	ID=MALK_01950;prediction_source=maker_MRET:augustus_masked-contig006-processed-gene-4.18-mRNA-1
contig006	maker	CDS	446845	446854	.	+	0	ID=MALK_01950.t1.c1;Parent=MALK_01950.t1
contig006	maker	CDS	446964	448096	.	+	0	ID=MALK_01950.t1.c2;Parent=MALK_01950.t1
contig006	maker	mRNA	446845	448096	.	+	.	ID=MALK_01950.t1;Parent=MALK_01950
contig006	maker	exon	446845	446854	.	+	.	ID=MALK_01950.t1.e1;Parent=MALK_01950.t1
contig006	maker	exon	446964	448096	.	+	.	ID=MALK_01950.t1.e2;Parent=MALK_01950.t1
contig006	maker	gene	448110	449309	.	-	.	ID=MALK_01951;prediction_source=maker_MRET:augustus_masked-contig006-processed-gene-4.41-mRNA-1
contig006	maker	CDS	448110	449309	.	-	0	ID=MALK_01951.t1.c1;Parent=MALK_01951.t1
contig006	maker	mRNA	448110	449309	.	-	.	ID=MALK_01951.t1;Parent=MALK_01951
contig006	maker	exon	448110	449309	.	-	.	ID=MALK_01951.t1.e1;Parent=MALK_01951.t1
contig006	AUGUSTUS	gene	449381	450586	0.98	+	.	ID=MALK_01952;prediction_source=augustus:contig006.g2202.t1
contig006	AUGUSTUS	CDS	449381	450586	0.98	+	0	ID=MALK_01952.t1.c1;Parent=MALK_01952.t1

contig006	AUGUSTUS	mRNA	449381	450586	0.98	+	.	ID=MALK_01952.t1;Parent=MALK_01952
contig006	AUGUSTUS	exon	449381	450586	0.98	+	.	ID=MALK_01952.t1.e1;Parent=MALK_01952.t1
contig006	AUGUSTUS	gene	450603	451532	0.85	-	.	ID=MALK_01953;prediction_source=augustus:contig006.g2203.t1
contig006	AUGUSTUS	CDS	450603	451532	0.85	-	0	ID=MALK_01953.t1.c1;Parent=MALK_01953.t1
contig006	AUGUSTUS	mRNA	450603	451532	0.85	-	.	ID=MALK_01953.t1;Parent=MALK_01953
contig006	AUGUSTUS	exon	450603	451532	0.85	-	.	ID=MALK_01953.t1.e1;Parent=MALK_01953.t1
contig006	AUGUSTUS	gene	451682	452614	0.38	-	.	ID=MALK_01954;prediction_source=augustus:contig006.g2204.t1
contig006	AUGUSTUS	CDS	451682	452614	0.38	-	0	ID=MALK_01954.t1.c1;Parent=MALK_01954.t1
contig006	AUGUSTUS	mRNA	451682	452614	0.38	-	.	ID=MALK_01954.t1;Parent=MALK_01954
contig006	AUGUSTUS	exon	451682	452614	0.38	-	.	ID=MALK_01954.t1.e1;Parent=MALK_01954.t1
contig006	maker	gene	452789	454699	.	-	.	ID=MALK_01955;prediction_source=maker_MRET:augustus_masked-contig006-processed-gene-4.44-mRNA-1
contig006	maker	CDS	452789	454699	.	-	0	ID=MALK_01955.t1.c1;Parent=MALK_01955.t1
contig006	maker	mRNA	452789	454699	.	-	.	ID=MALK_01955.t1;Parent=MALK_01955
contig006	maker	exon	452789	454699	.	-	.	ID=MALK_01955.t1.e1;Parent=MALK_01955.t1
contig006	maker	gene	455427	455924	.	-	.	ID=MALK_01956;prediction_source=maker_MRET:augustus_masked-contig006-processed-gene-4.45-mRNA-1
contig006	maker	CDS	455859	455924	.	-	0	ID=MALK_01956.t1.c1;Parent=MALK_01956.t1
contig006	maker	CDS	455427	455789	.	-	0	ID=MALK_01956.t1.c2;Parent=MALK_01956.t1
contig006	maker	mRNA	455427	455924	.	-	.	ID=MALK_01956.t1;Parent=MALK_01956
contig006	maker	exon	455859	455924	.	-	.	ID=MALK_01956.t1.e1;Parent=MALK_01956.t1
contig006	maker	exon	455427	455789	.	-	.	ID=MALK_01956.t1.e2;Parent=MALK_01956.t1
contig006	AUGUSTUS	gene	456068	457285	0.79	-	.	ID=MALK_01957;prediction_source=augustus:contig006.g2207.t1
contig006	AUGUSTUS	CDS	456068	457285	0.79	-	0	ID=MALK_01957.t1.c1;Parent=MALK_01957.t1
contig006	AUGUSTUS	mRNA	456068	457285	0.79	-	.	ID=MALK_01957.t1;Parent=MALK_01957
contig006	AUGUSTUS	exon	456068	457285	0.79	-	.	ID=MALK_01957.t1.e1;Parent=MALK_01957.t1
contig006	maker	gene	457339	458853	.	+	.	ID=MALK_01958;prediction_source=maker_MRET:augustus_masked-contig006-processed-gene-4.20-mRNA-1
contig006	maker	CDS	457339	458853	.	+	0	ID=MALK_01958.t1.c1;Parent=MALK_01958.t1
contig006	maker	mRNA	457339	458853	.	+	.	ID=MALK_01958.t1;Parent=MALK_01958
contig006	maker	exon	457339	458853	.	+	.	ID=MALK_01958.t1.e1;Parent=MALK_01958.t1
contig006	AUGUSTUS	gene	458856	460940	0.68	-	.	ID=MALK_01959;prediction_source=augustus:contig006.g2209.t1
contig006	AUGUSTUS	CDS	458856	460940	0.68	-	0	ID=MALK_01959.t1.c1;Parent=MALK_01959.t1
contig006	AUGUSTUS	mRNA	458856	460940	0.68	-	.	ID=MALK_01959.t1;Parent=MALK_01959
contig006	AUGUSTUS	exon	458856	460940	0.68	-	.	ID=MALK_01959.t1.e1;Parent=MALK_01959.t1
contig006	AUGUSTUS	gene	461139	461731	0.55	+	.	ID=MALK_01960;prediction_source=braker_MRET:g1957.t1
contig006	AUGUSTUS	CDS	461139	461163	0.98	+	0	ID=MALK_01960.t1.c1;Parent=MALK_01960.t1
contig006	AUGUSTUS	CDS	461192	461344	0.98	+	0	ID=MALK_01960.t1.c2;Parent=MALK_01960.t1
contig006	AUGUSTUS	CDS	461395	461507	0.98	+	0	ID=MALK_01960.t1.c3;Parent=MALK_01960.t1
contig006	AUGUSTUS	CDS	461540	461731	0.98	+	0	ID=MALK_01960.t1.c4;Parent=MALK_01960.t1
contig006	AUGUSTUS	mRNA	461139	461731	0.55	+	.	ID=MALK_01960.t1;Parent=MALK_01960
contig006	AUGUSTUS	exon	461139	461163	.	+	.	ID=MALK_01960.t1.e1;Parent=MALK_01960.t1
contig006	AUGUSTUS	exon	461192	461344	.	+	.	ID=MALK_01960.t1.e2;Parent=MALK_01960.t1
contig006	AUGUSTUS	exon	461395	461507	.	+	.	ID=MALK_01960.t1.e3;Parent=MALK_01960.t1
contig006	AUGUSTUS	exon	461540	461731	.	+	.	ID=MALK_01960.t1.e4;Parent=MALK_01960.t1
contig006	maker	gene	461991	462467	.	+	.	ID=MALK_01961;prediction_source=maker_MRET:augustus_masked-contig006-processed-gene-4.21-mRNA-1

contig006	maker	CDS	461991	462467	.	+	0	ID=MALK_01961.t1.c1;Parent=MALK_01961.t1
contig006	maker	mRNA	461991	462467	.	+	.	ID=MALK_01961.t1;Parent=MALK_01961
contig006	maker	exon	461991	462467	.	+	.	ID=MALK_01961.t1.e1;Parent=MALK_01961.t1
contig006	AUGUSTUS	gene	462582	464360	0.98	-	.	ID=MALK_01962;prediction_source=augustus:contig006.g2211.t1
contig006	AUGUSTUS	CDS	462582	464360	0.98	-	0	ID=MALK_01962.t1.c1;Parent=MALK_01962.t1
contig006	AUGUSTUS	mRNA	462582	464360	0.98	-	.	ID=MALK_01962.t1;Parent=MALK_01962
contig006	AUGUSTUS	exon	462582	464360	0.98	-	.	ID=MALK_01962.t1.e1;Parent=MALK_01962.t1
contig006	AUGUSTUS	gene	464725	465666	0.61	+	.	ID=MALK_01963;prediction_source=braker_MRET:g1960.t1
contig006	AUGUSTUS	CDS	464725	465666	0.61	+	0	ID=MALK_01963.t1.c1;Parent=MALK_01963.t1
contig006	AUGUSTUS	mRNA	464725	465666	0.61	+	.	ID=MALK_01963.t1;Parent=MALK_01963
contig006	AUGUSTUS	exon	464725	465666	.	+	.	ID=MALK_01963.t1.e1;Parent=MALK_01963.t1
contig006	maker	gene	465702	467063	.	-	.	ID=MALK_01964;prediction_source=maker_MRET:augustus_masked-contig006-processed-gene-4.49-mRNA-1
contig006	maker	CDS	465702	467063	.	-	0	ID=MALK_01964.t1.c1;Parent=MALK_01964.t1
contig006	maker	mRNA	465702	467063	.	-	.	ID=MALK_01964.t1;Parent=MALK_01964
contig006	maker	exon	465702	467063	.	-	.	ID=MALK_01964.t1.e1;Parent=MALK_01964.t1
contig006	maker	gene	467144	467509	.	+	.	ID=MALK_01965;prediction_source=maker_MRET:augustus_masked-contig006-processed-gene-4.22-mRNA-1
contig006	maker	CDS	467144	467509	.	+	0	ID=MALK_01965.t1.c1;Parent=MALK_01965.t1
contig006	maker	mRNA	467144	467509	.	+	.	ID=MALK_01965.t1;Parent=MALK_01965
contig006	maker	exon	467144	467509	.	+	.	ID=MALK_01965.t1.e1;Parent=MALK_01965.t1
contig006	AUGUSTUS	gene	467859	473177	0.92	+	.	ID=MALK_01966;prediction_source=augustus:contig006.g2214.t1
contig006	AUGUSTUS	CDS	467859	473177	0.92	+	0	ID=MALK_01966.t1.c1;Parent=MALK_01966.t1
contig006	AUGUSTUS	mRNA	467859	473177	0.92	+	.	ID=MALK_01966.t1;Parent=MALK_01966
contig006	AUGUSTUS	exon	467859	473177	0.92	+	.	ID=MALK_01966.t1.e1;Parent=MALK_01966.t1
contig006	maker	gene	473179	474135	.	-	.	ID=MALK_01967;prediction_source=maker_MRET:augustus_masked-contig006-processed-gene-4.50-mRNA-1
contig006	maker	CDS	473179	474135	.	-	0	ID=MALK_01967.t1.c1;Parent=MALK_01967.t1
contig006	maker	mRNA	473179	474135	.	-	.	ID=MALK_01967.t1;Parent=MALK_01967
contig006	maker	exon	473179	474135	.	-	.	ID=MALK_01967.t1.e1;Parent=MALK_01967.t1
contig006	AUGUSTUS	gene	474263	475708	0.95	+	.	ID=MALK_01968;prediction_source=augustus:contig006.g2216.t1
contig006	AUGUSTUS	CDS	474263	475708	0.95	+	0	ID=MALK_01968.t1.c1;Parent=MALK_01968.t1
contig006	AUGUSTUS	mRNA	474263	475708	0.95	+	.	ID=MALK_01968.t1;Parent=MALK_01968
contig006	AUGUSTUS	exon	474263	475708	0.95	+	.	ID=MALK_01968.t1.e1;Parent=MALK_01968.t1
contig006	AUGUSTUS	gene	475720	477417	1	-	.	ID=MALK_01969;prediction_source=braker_MRET:g1966.t1
contig006	AUGUSTUS	CDS	475720	477417	1	-	0	ID=MALK_01969.t1.c1;Parent=MALK_01969.t1
contig006	AUGUSTUS	mRNA	475720	477417	1	-	.	ID=MALK_01969.t1;Parent=MALK_01969
contig006	AUGUSTUS	exon	475720	477417	.	-	.	ID=MALK_01969.t1.e1;Parent=MALK_01969.t1
contig006	maker	gene	477524	479323	.	+	.	ID=MALK_01970;prediction_source=maker_MRET:augustus_masked-contig006-processed-gene-4.25-mRNA-1
contig006	maker	CDS	477524	479323	.	+	0	ID=MALK_01970.t1.c1;Parent=MALK_01970.t1
contig006	maker	mRNA	477524	479323	.	+	.	ID=MALK_01970.t1;Parent=MALK_01970
contig006	maker	exon	477524	479323	.	+	.	ID=MALK_01970.t1.e1;Parent=MALK_01970.t1
contig006	AUGUSTUS	gene	479334	482675	0.82	-	.	ID=MALK_01971;prediction_source=augustus:contig006.g2219.t1
contig006	AUGUSTUS	CDS	479334	482675	0.82	-	0	ID=MALK_01971.t1.c1;Parent=MALK_01971.t1
contig006	AUGUSTUS	mRNA	479334	482675	0.82	-	.	ID=MALK_01971.t1;Parent=MALK_01971
contig006	AUGUSTUS	exon	479334	482675	0.82	-	.	ID=MALK_01971.t1.e1;Parent=MALK_01971.t1

contig006	AUGUSTUS	gene	482768	483385	1	-	.	ID=MALK_01972;prediction_source=braker_MRET:g1969.t1
contig006	AUGUSTUS	CDS	482768	483385	1	-	0	ID=MALK_01972.t1.c1;Parent=MALK_01972.t1
contig006	AUGUSTUS	mRNA	482768	483385	1	-	.	ID=MALK_01972.t1;Parent=MALK_01972
contig006	AUGUSTUS	exon	482768	483385	.	-	.	ID=MALK_01972.t1.e1;Parent=MALK_01972.t1
contig006	maker	gene	483480	484520	.	-	.	ID=MALK_01973;prediction_source=maker_MRET:augustus_masked-contig006-processed-gene-4.53-mRNA-1
contig006	maker	CDS	483480	484520	.	-	0	ID=MALK_01973.t1.c1;Parent=MALK_01973.t1
contig006	maker	mRNA	483480	484520	.	-	.	ID=MALK_01973.t1;Parent=MALK_01973
contig006	maker	exon	483480	484520	.	-	.	ID=MALK_01973.t1.e1;Parent=MALK_01973.t1
contig006	AUGUSTUS	gene	485050	486867	1	+	.	ID=MALK_01974;prediction_source=augustus:contig006.g2223.t1
contig006	AUGUSTUS	CDS	485050	486867	1	+	0	ID=MALK_01974.t1.c1;Parent=MALK_01974.t1
contig006	AUGUSTUS	mRNA	485050	486867	1	+	.	ID=MALK_01974.t1;Parent=MALK_01974
contig006	AUGUSTUS	exon	485050	486867	1	+	.	ID=MALK_01974.t1.e1;Parent=MALK_01974.t1
contig006	maker	gene	487169	492772	.	+	.	ID=MALK_01975;prediction_source=maker_MRET:augustus_masked-contig006-processed-gene-4.27-mRNA-1
contig006	maker	CDS	487169	492772	.	+	0	ID=MALK_01975.t1.c1;Parent=MALK_01975.t1
contig006	maker	mRNA	487169	492772	.	+	.	ID=MALK_01975.t1;Parent=MALK_01975
contig006	maker	exon	487169	492772	.	+	.	ID=MALK_01975.t1.e1;Parent=MALK_01975.t1
contig006	maker	gene	492775	494874	.	-	.	ID=MALK_01976;prediction_source=maker_MRET:augustus_masked-contig006-processed-gene-5.2-mRNA-1
contig006	maker	CDS	492775	494874	.	-	0	ID=MALK_01976.t1.c1;Parent=MALK_01976.t1
contig006	maker	mRNA	492775	494874	.	-	.	ID=MALK_01976.t1;Parent=MALK_01976
contig006	maker	exon	492775	494874	.	-	.	ID=MALK_01976.t1.e1;Parent=MALK_01976.t1
contig006	maker	gene	494959	496584	.	+	.	ID=MALK_01977;prediction_source=maker_MRET:augustus_masked-contig006-processed-gene-5.0-mRNA-1
contig006	maker	CDS	494959	496584	.	+	0	ID=MALK_01977.t1.c1;Parent=MALK_01977.t1
contig006	maker	mRNA	494959	496584	.	+	.	ID=MALK_01977.t1;Parent=MALK_01977
contig006	maker	exon	494959	496584	.	+	.	ID=MALK_01977.t1.e1;Parent=MALK_01977.t1
contig006	AUGUSTUS	gene	496591	498882	1	-	.	ID=MALK_01978;prediction_source=augustus:contig006.g2228.t1
contig006	AUGUSTUS	CDS	496591	498882	1	-	0	ID=MALK_01978.t1.c1;Parent=MALK_01978.t1
contig006	AUGUSTUS	mRNA	496591	498882	1	-	.	ID=MALK_01978.t1;Parent=MALK_01978
contig006	AUGUSTUS	exon	496591	498882	1	-	.	ID=MALK_01978.t1.e1;Parent=MALK_01978.t1
contig006	AUGUSTUS	gene	499351	500064	0.46	+	.	ID=MALK_01979;prediction_source=braker_MRET:g1976.t1
contig006	AUGUSTUS	CDS	499351	500064	0.46	+	0	ID=MALK_01979.t1.c1;Parent=MALK_01979.t1
contig006	AUGUSTUS	mRNA	499351	500064	0.46	+	.	ID=MALK_01979.t1;Parent=MALK_01979
contig006	AUGUSTUS	exon	499351	500064	.	+	.	ID=MALK_01979.t1.e1;Parent=MALK_01979.t1
contig006	maker	gene	500832	501242	.	+	.	ID=MALK_01980;prediction_source=maker_MRET:augustus_masked-contig006-processed-gene-5.4-mRNA-1
contig006	maker	CDS	500832	501242	.	+	0	ID=MALK_01980.t1.c1;Parent=MALK_01980.t1
contig006	maker	mRNA	500832	501242	.	+	.	ID=MALK_01980.t1;Parent=MALK_01980
contig006	maker	exon	500832	501242	.	+	.	ID=MALK_01980.t1.e1;Parent=MALK_01980.t1
contig006	maker	gene	501500	502771	.	+	.	ID=MALK_01981;prediction_source=maker_MRET:augustus_masked-contig006-processed-gene-5.5-mRNA-1
contig006	maker	CDS	501500	502771	.	+	0	ID=MALK_01981.t1.c1;Parent=MALK_01981.t1
contig006	maker	mRNA	501500	502771	.	+	.	ID=MALK_01981.t1;Parent=MALK_01981
contig006	maker	exon	501500	502771	.	+	.	ID=MALK_01981.t1.e1;Parent=MALK_01981.t1
contig006	AUGUSTUS	gene	502848	504878	1	-	.	ID=MALK_01982;prediction_source=braker_MRET:g1979.t1
contig006	AUGUSTUS	CDS	502848	504878	1	-	0	ID=MALK_01982.t1.c1;Parent=MALK_01982.t1
contig006	AUGUSTUS	mRNA	502848	504878	1	-	.	ID=MALK_01982.t1;Parent=MALK_01982

contig006	AUGUSTUS	exon	502848	504878	.	-	.	ID=MALK_01982.t1.e1;Parent=MALK_01982.t1
contig006	AUGUSTUS	gene	505046	506110	0.69	+	.	ID=MALK_01983;prediction_source=augustus:contig006.g2233.t1
contig006	AUGUSTUS	CDS	505046	506110	0.69	+	0	ID=MALK_01983.t1.c1;Parent=MALK_01983.t1
contig006	AUGUSTUS	mRNA	505046	506110	0.69	+	.	ID=MALK_01983.t1;Parent=MALK_01983
contig006	AUGUSTUS	exon	505046	506110	0.69	+	.	ID=MALK_01983.t1.e1;Parent=MALK_01983.t1
contig006	AUGUSTUS	gene	506136	507974	0.38	+	.	ID=MALK_01984;prediction_source=braker_MRET:g1981.t1
contig006	AUGUSTUS	CDS	506136	507974	0.38	+	0	ID=MALK_01984.t1.c1;Parent=MALK_01984.t1
contig006	AUGUSTUS	mRNA	506136	507974	0.38	+	.	ID=MALK_01984.t1;Parent=MALK_01984
contig006	AUGUSTUS	exon	506136	507974	.	+	.	ID=MALK_01984.t1.e1;Parent=MALK_01984.t1
contig006	AUGUSTUS	gene	507997	508407	0.66	+	.	ID=MALK_01985;prediction_source=braker_MRET:g1982.t1
contig006	AUGUSTUS	CDS	507997	508407	0.66	+	0	ID=MALK_01985.t1.c1;Parent=MALK_01985.t1
contig006	AUGUSTUS	mRNA	507997	508407	0.66	+	.	ID=MALK_01985.t1;Parent=MALK_01985
contig006	AUGUSTUS	exon	507997	508407	.	+	.	ID=MALK_01985.t1.e1;Parent=MALK_01985.t1
contig006	AUGUSTUS	gene	508420	510461	0.34	-	.	ID=MALK_01986;prediction_source=braker_MRET:g1983.t1
contig006	AUGUSTUS	CDS	509874	510461	0.35	-	0	ID=MALK_01986.t1.c2;Parent=MALK_01986.t1
contig006	AUGUSTUS	CDS	508420	509805	0.35	-	0	ID=MALK_01986.t1.c1;Parent=MALK_01986.t1
contig006	AUGUSTUS	mRNA	508420	510461	0.34	-	.	ID=MALK_01986.t1;Parent=MALK_01986
contig006	AUGUSTUS	exon	509874	510461	.	-	.	ID=MALK_01986.t1.e2;Parent=MALK_01986.t1
contig006	AUGUSTUS	exon	508420	509805	.	-	.	ID=MALK_01986.t1.e1;Parent=MALK_01986.t1
contig006	maker	gene	510535	512745	.	+	.	ID=MALK_01987;prediction_source=maker_MRET:augustus_masked-contig006-processed-gene-5.8-mRNA-1
contig006	maker	CDS	510535	512745	.	+	0	ID=MALK_01987.t1.c1;Parent=MALK_01987.t1
contig006	maker	mRNA	510535	512745	.	+	.	ID=MALK_01987.t1;Parent=MALK_01987
contig006	maker	exon	510535	512745	.	+	.	ID=MALK_01987.t1.e1;Parent=MALK_01987.t1
contig006	AUGUSTUS	gene	512985	513824	0.97	-	.	ID=MALK_01988;prediction_source=braker_MRET:g1985.t1
contig006	AUGUSTUS	CDS	512985	513824	0.97	-	0	ID=MALK_01988.t1.c1;Parent=MALK_01988.t1
contig006	AUGUSTUS	mRNA	512985	513824	0.97	-	.	ID=MALK_01988.t1;Parent=MALK_01988
contig006	AUGUSTUS	exon	512985	513824	.	-	.	ID=MALK_01988.t1.e1;Parent=MALK_01988.t1
contig006	AUGUSTUS	gene	513949	514632	0.68	+	.	ID=MALK_01989;prediction_source=augustus:contig006.g2241.t1
contig006	AUGUSTUS	CDS	513949	514632	0.68	+	0	ID=MALK_01989.t1.c1;Parent=MALK_01989.t1
contig006	AUGUSTUS	mRNA	513949	514632	0.68	+	.	ID=MALK_01989.t1;Parent=MALK_01989
contig006	AUGUSTUS	exon	513949	514632	0.68	+	.	ID=MALK_01989.t1.e1;Parent=MALK_01989.t1
contig006	AUGUSTUS	gene	514658	515413	0.83	-	.	ID=MALK_01990;prediction_source=augustus:contig006.g2242.t1
contig006	AUGUSTUS	CDS	514658	515413	0.83	-	0	ID=MALK_01990.t1.c1;Parent=MALK_01990.t1
contig006	AUGUSTUS	mRNA	514658	515413	0.83	-	.	ID=MALK_01990.t1;Parent=MALK_01990
contig006	AUGUSTUS	exon	514658	515413	0.83	-	.	ID=MALK_01990.t1.e1;Parent=MALK_01990.t1
contig006	AUGUSTUS	gene	515634	516948	0.99	+	.	ID=MALK_01991;prediction_source=braker_MRET:g1988.t1
contig006	AUGUSTUS	CDS	515634	515941	1	+	0	ID=MALK_01991.t1.c1;Parent=MALK_01991.t1
contig006	AUGUSTUS	CDS	515976	516948	1	+	0	ID=MALK_01991.t1.c2;Parent=MALK_01991.t1
contig006	AUGUSTUS	mRNA	515634	516948	0.99	+	.	ID=MALK_01991.t1;Parent=MALK_01991
contig006	AUGUSTUS	exon	515634	515941	.	+	.	ID=MALK_01991.t1.e1;Parent=MALK_01991.t1
contig006	AUGUSTUS	exon	515976	516948	.	+	.	ID=MALK_01991.t1.e2;Parent=MALK_01991.t1
contig006	AUGUSTUS	gene	517034	517495	0.77	-	.	ID=MALK_01992;prediction_source=braker_MRET:g1989.t1
contig006	AUGUSTUS	CDS	517034	517495	0.77	-	0	ID=MALK_01992.t1.c1;Parent=MALK_01992.t1

contig006	AUGUSTUS	mRNA	517034	517495	0.77	-	.	ID=MALK_01992.t1;Parent=MALK_01992
contig006	AUGUSTUS	exon	517034	517495	.	-	.	ID=MALK_01992.t1.e1;Parent=MALK_01992.t1
contig006	AUGUSTUS	gene	517612	518228	0.35	-	.	ID=MALK_01993;prediction_source=braker_MRET:g1990.t1
contig006	AUGUSTUS	CDS	517886	518228	0.48	-	0	ID=MALK_01993.t1.c2;Parent=MALK_01993.t1
contig006	AUGUSTUS	CDS	517612	517841	0.48	-	0	ID=MALK_01993.t1.c1;Parent=MALK_01993.t1
contig006	AUGUSTUS	mRNA	517612	518228	0.35	-	.	ID=MALK_01993.t1;Parent=MALK_01993
contig006	AUGUSTUS	exon	517886	518228	.	-	.	ID=MALK_01993.t1.e2;Parent=MALK_01993.t1
contig006	AUGUSTUS	exon	517612	517841	.	-	.	ID=MALK_01993.t1.e1;Parent=MALK_01993.t1
contig006	AUGUSTUS	gene	519009	519358	0.6	-	.	ID=MALK_01994;prediction_source=braker_MRET:g1991.t1
contig006	AUGUSTUS	CDS	519356	519358	1	-	0	ID=MALK_01994.t1.c4;Parent=MALK_01994.t1
contig006	AUGUSTUS	CDS	519166	519299	1	-	0	ID=MALK_01994.t1.c3;Parent=MALK_01994.t1
contig006	AUGUSTUS	CDS	519077	519129	1	-	0	ID=MALK_01994.t1.c2;Parent=MALK_01994.t1
contig006	AUGUSTUS	CDS	519009	519034	1	-	0	ID=MALK_01994.t1.c1;Parent=MALK_01994.t1
contig006	AUGUSTUS	mRNA	519009	519358	0.6	-	.	ID=MALK_01994.t1;Parent=MALK_01994
contig006	AUGUSTUS	exon	519356	519358	.	-	.	ID=MALK_01994.t1.e4;Parent=MALK_01994.t1
contig006	AUGUSTUS	exon	519166	519299	.	-	.	ID=MALK_01994.t1.e3;Parent=MALK_01994.t1
contig006	AUGUSTUS	exon	519077	519129	.	-	.	ID=MALK_01994.t1.e2;Parent=MALK_01994.t1
contig006	AUGUSTUS	exon	519009	519034	.	-	.	ID=MALK_01994.t1.e1;Parent=MALK_01994.t1
contig006	AUGUSTUS	gene	519725	520312	1	+	.	ID=MALK_01995;prediction_source=augustus:contig006.g2245.t1
contig006	AUGUSTUS	CDS	519725	520312	1	+	0	ID=MALK_01995.t1.c1;Parent=MALK_01995.t1
contig006	AUGUSTUS	mRNA	519725	520312	1	+	.	ID=MALK_01995.t1;Parent=MALK_01995
contig006	AUGUSTUS	exon	519725	520312	1	+	.	ID=MALK_01995.t1.e1;Parent=MALK_01995.t1
contig006	maker	gene	520709	522112	.	-	.	ID=MALK_01996;prediction_source=maker_MRET:augustus_masked-contig006-processed-gene-5.38-mRNA-1
contig006	maker	CDS	520709	522112	.	-	0	ID=MALK_01996.t1.c1;Parent=MALK_01996.t1
contig006	maker	mRNA	520709	522112	.	-	.	ID=MALK_01996.t1;Parent=MALK_01996
contig006	maker	exon	520709	522112	.	-	.	ID=MALK_01996.t1.e1;Parent=MALK_01996.t1
contig006	AUGUSTUS	gene	522299	522865	0.71	+	.	ID=MALK_01997;prediction_source=braker_MRET:g1994.t1
contig006	AUGUSTUS	CDS	522299	522865	0.71	+	0	ID=MALK_01997.t1.c1;Parent=MALK_01997.t1
contig006	AUGUSTUS	mRNA	522299	522865	0.71	+	.	ID=MALK_01997.t1;Parent=MALK_01997
contig006	AUGUSTUS	exon	522299	522865	.	+	.	ID=MALK_01997.t1.e1;Parent=MALK_01997.t1
contig006	maker	gene	522980	524911	.	+	.	ID=MALK_01998;prediction_source=maker_MRET:augustus_masked-contig006-processed-gene-5.13-mRNA-1
contig006	maker	CDS	522980	524911	.	+	0	ID=MALK_01998.t1.c1;Parent=MALK_01998.t1
contig006	maker	mRNA	522980	524911	.	+	.	ID=MALK_01998.t1;Parent=MALK_01998
contig006	maker	exon	522980	524911	.	+	.	ID=MALK_01998.t1.e1;Parent=MALK_01998.t1
contig006	maker	gene	525065	525856	.	+	.	ID=MALK_01999;prediction_source=maker_MRET:augustus_masked-contig006-processed-gene-5.14-mRNA-1
contig006	maker	CDS	525065	525856	.	+	0	ID=MALK_01999.t1.c1;Parent=MALK_01999.t1
contig006	maker	mRNA	525065	525856	.	+	.	ID=MALK_01999.t1;Parent=MALK_01999
contig006	maker	exon	525065	525856	.	+	.	ID=MALK_01999.t1.e1;Parent=MALK_01999.t1
contig006	AUGUSTUS	gene	526038	527132	0.7	+	.	ID=MALK_02000;prediction_source=augustus:contig006.g2251.t1
contig006	AUGUSTUS	CDS	526038	527132	0.7	+	0	ID=MALK_02000.t1.c1;Parent=MALK_02000.t1
contig006	AUGUSTUS	mRNA	526038	527132	0.7	+	.	ID=MALK_02000.t1;Parent=MALK_02000
contig006	AUGUSTUS	exon	526038	527132	0.7	+	.	ID=MALK_02000.t1.e1;Parent=MALK_02000.t1
contig006	AUGUSTUS	gene	527291	528064	0.98	-	.	ID=MALK_02001;prediction_source=augustus:contig006.g2252.t1

contig006	AUGUSTUS	CDS	527291	528064	0.98	-	0	ID=MALK_02001.t1.c1;Parent=MALK_02001.t1
contig006	AUGUSTUS	mRNA	527291	528064	0.98	-	.	ID=MALK_02001.t1;Parent=MALK_02001
contig006	AUGUSTUS	exon	527291	528064	0.98	-	.	ID=MALK_02001.t1.e1;Parent=MALK_02001.t1
contig006	maker	gene	528448	529240	.	-	.	ID=MALK_02002;prediction_source=maker_MRET:augustus_masked-contig006-processed-gene-5.40-mRNA-1
contig006	maker	CDS	528900	529240	.	-	0	ID=MALK_02002.t1.c1;Parent=MALK_02002.t1
contig006	maker	CDS	528448	528808	.	-	0	ID=MALK_02002.t1.c2;Parent=MALK_02002.t1
contig006	maker	mRNA	528448	529240	.	-	.	ID=MALK_02002.t1;Parent=MALK_02002
contig006	maker	exon	528900	529240	.	-	.	ID=MALK_02002.t1.e1;Parent=MALK_02002.t1
contig006	maker	exon	528448	528808	.	-	.	ID=MALK_02002.t1.e2;Parent=MALK_02002.t1
contig006	AUGUSTUS	gene	529377	531128	0.94	-	.	ID=MALK_02003;prediction_source=augustus:contig006.g2254.t1
contig006	AUGUSTUS	CDS	529377	531128	0.94	-	0	ID=MALK_02003.t1.c1;Parent=MALK_02003.t1
contig006	AUGUSTUS	mRNA	529377	531128	0.94	-	.	ID=MALK_02003.t1;Parent=MALK_02003
contig006	AUGUSTUS	exon	529377	531128	0.94	-	.	ID=MALK_02003.t1.e1;Parent=MALK_02003.t1
contig006	AUGUSTUS	gene	531808	533438	0.9	+	.	ID=MALK_02004;prediction_source=braker_MRET:g2001.t1
contig006	AUGUSTUS	CDS	531808	533370	0.94	+	0	ID=MALK_02004.t1.c1;Parent=MALK_02004.t1
contig006	AUGUSTUS	CDS	533430	533438	0.94	+	0	ID=MALK_02004.t1.c2;Parent=MALK_02004.t1
contig006	AUGUSTUS	mRNA	531808	533438	0.9	+	.	ID=MALK_02004.t1;Parent=MALK_02004
contig006	AUGUSTUS	exon	531808	533370	.	+	.	ID=MALK_02004.t1.e1;Parent=MALK_02004.t1
contig006	AUGUSTUS	exon	533430	533438	.	+	.	ID=MALK_02004.t1.e2;Parent=MALK_02004.t1
contig006	maker	gene	533521	534933	.	-	.	ID=MALK_02005;prediction_source=maker_MRET:augustus_masked-contig006-processed-gene-5.42-mRNA-1
contig006	maker	CDS	533521	534933	.	-	0	ID=MALK_02005.t1.c1;Parent=MALK_02005.t1
contig006	maker	mRNA	533521	534933	.	-	.	ID=MALK_02005.t1;Parent=MALK_02005
contig006	maker	exon	533521	534933	.	-	.	ID=MALK_02005.t1.e1;Parent=MALK_02005.t1
contig006	maker	gene	535079	536674	.	-	.	ID=MALK_02006;prediction_source=maker_MRET:augustus_masked-contig006-processed-gene-5.43-mRNA-1
contig006	maker	CDS	535079	536674	.	-	0	ID=MALK_02006.t1.c1;Parent=MALK_02006.t1
contig006	maker	mRNA	535079	536674	.	-	.	ID=MALK_02006.t1;Parent=MALK_02006
contig006	maker	exon	535079	536674	.	-	.	ID=MALK_02006.t1.e1;Parent=MALK_02006.t1
contig006	AUGUSTUS	gene	536881	539310	0.76	+	.	ID=MALK_02007;prediction_source=augustus:contig006.g2258.t1
contig006	AUGUSTUS	CDS	536881	539310	0.76	+	0	ID=MALK_02007.t1.c1;Parent=MALK_02007.t1
contig006	AUGUSTUS	mRNA	536881	539310	0.76	+	.	ID=MALK_02007.t1;Parent=MALK_02007
contig006	AUGUSTUS	exon	536881	539310	0.76	+	.	ID=MALK_02007.t1.e1;Parent=MALK_02007.t1
contig006	maker	gene	539394	541745	.	+	.	ID=MALK_02008;prediction_source=maker_MRET:augustus_masked-contig006-processed-gene-5.18-mRNA-1
contig006	maker	CDS	539394	541745	.	+	0	ID=MALK_02008.t1.c1;Parent=MALK_02008.t1
contig006	maker	mRNA	539394	541745	.	+	.	ID=MALK_02008.t1;Parent=MALK_02008
contig006	maker	exon	539394	541745	.	+	.	ID=MALK_02008.t1.e1;Parent=MALK_02008.t1
contig006	maker	gene	541751	542890	.	-	.	ID=MALK_02009;prediction_source=maker_MRET:augustus_masked-contig006-processed-gene-5.44-mRNA-1
contig006	maker	CDS	541751	542890	.	-	0	ID=MALK_02009.t1.c1;Parent=MALK_02009.t1
contig006	maker	mRNA	541751	542890	.	-	.	ID=MALK_02009.t1;Parent=MALK_02009
contig006	maker	exon	541751	542890	.	-	.	ID=MALK_02009.t1.e1;Parent=MALK_02009.t1
contig006	maker	gene	542937	544955	.	+	.	ID=MALK_02010;prediction_source=maker_MRET:augustus_masked-contig006-processed-gene-5.19-mRNA-1
contig006	maker	CDS	542937	544955	.	+	0	ID=MALK_02010.t1.c1;Parent=MALK_02010.t1
contig006	maker	mRNA	542937	544955	.	+	.	ID=MALK_02010.t1;Parent=MALK_02010
contig006	maker	exon	542937	544955	.	+	.	ID=MALK_02010.t1.e1;Parent=MALK_02010.t1

contig006	AUGUSTUS	gene	544972	545879	0.62	-	.	ID=MALK_02011;prediction_source=braker_MRET:g2008.t1
contig006	AUGUSTUS	CDS	545032	545879	0.71	-	0	ID=MALK_02011.t1.c2;Parent=MALK_02011.t1
contig006	AUGUSTUS	CDS	544972	544993	0.71	-	0	ID=MALK_02011.t1.c1;Parent=MALK_02011.t1
contig006	AUGUSTUS	mRNA	544972	545879	0.62	-	.	ID=MALK_02011.t1;Parent=MALK_02011
contig006	AUGUSTUS	exon	545032	545879	.	-	.	ID=MALK_02011.t1.e2;Parent=MALK_02011.t1
contig006	AUGUSTUS	exon	544972	544993	.	-	.	ID=MALK_02011.t1.e1;Parent=MALK_02011.t1
contig006	maker	gene	545955	547460	.	+	.	ID=MALK_02012;prediction_source=maker_MRET:augustus_masked-contig006-processed-gene-5.20-mRNA-1
contig006	maker	CDS	545955	547460	.	+	0	ID=MALK_02012.t1.c1;Parent=MALK_02012.t1
contig006	maker	mRNA	545955	547460	.	+	.	ID=MALK_02012.t1;Parent=MALK_02012
contig006	maker	exon	545955	547460	.	+	.	ID=MALK_02012.t1.e1;Parent=MALK_02012.t1
contig006	maker	gene	547473	548561	.	-	.	ID=MALK_02013;prediction_source=maker_MRET:augustus_masked-contig006-processed-gene-5.45-mRNA-1
contig006	maker	CDS	547473	548561	.	-	0	ID=MALK_02013.t1.c1;Parent=MALK_02013.t1
contig006	maker	mRNA	547473	548561	.	-	.	ID=MALK_02013.t1;Parent=MALK_02013
contig006	maker	exon	547473	548561	.	-	.	ID=MALK_02013.t1.e1;Parent=MALK_02013.t1
contig006	AUGUSTUS	gene	548747	550093	0.39	+	.	ID=MALK_02014;prediction_source=braker_MRET:g2011.t1
contig006	AUGUSTUS	CDS	548747	550093	0.39	+	0	ID=MALK_02014.t1.c1;Parent=MALK_02014.t1
contig006	AUGUSTUS	mRNA	548747	550093	0.39	+	.	ID=MALK_02014.t1;Parent=MALK_02014
contig006	AUGUSTUS	exon	548747	550093	.	+	.	ID=MALK_02014.t1.e1;Parent=MALK_02014.t1
contig006	AUGUSTUS	gene	550115	551803	0.18	-	.	ID=MALK_02015;prediction_source=augustus:contig006.g2269.t1
contig006	AUGUSTUS	CDS	550115	551803	0.18	-	0	ID=MALK_02015.t1.c1;Parent=MALK_02015.t1
contig006	AUGUSTUS	mRNA	550115	551803	0.18	-	.	ID=MALK_02015.t1;Parent=MALK_02015
contig006	AUGUSTUS	exon	550115	551803	0.18	-	.	ID=MALK_02015.t1.e1;Parent=MALK_02015.t1
contig006	AUGUSTUS	gene	552478	553800	0.96	+	.	ID=MALK_02016;prediction_source=augustus:contig006.g2270.t1
contig006	AUGUSTUS	CDS	552478	553800	0.96	+	0	ID=MALK_02016.t1.c1;Parent=MALK_02016.t1
contig006	AUGUSTUS	mRNA	552478	553800	0.96	+	.	ID=MALK_02016.t1;Parent=MALK_02016
contig006	AUGUSTUS	exon	552478	553800	0.96	+	.	ID=MALK_02016.t1.e1;Parent=MALK_02016.t1
contig006	maker	gene	553904	554518	.	-	.	ID=MALK_02017;prediction_source=maker_MRET:augustus_masked-contig006-processed-gene-5.47-mRNA-1
contig006	maker	CDS	553904	554518	.	-	0	ID=MALK_02017.t1.c1;Parent=MALK_02017.t1
contig006	maker	mRNA	553904	554518	.	-	.	ID=MALK_02017.t1;Parent=MALK_02017
contig006	maker	exon	553904	554518	.	-	.	ID=MALK_02017.t1.e1;Parent=MALK_02017.t1
contig006	AUGUSTUS	gene	554722	555649	0.4	-	.	ID=MALK_02018;prediction_source=braker_MRET:g2015.t1
contig006	AUGUSTUS	CDS	555585	555649	0.4	-	0	ID=MALK_02018.t1.c2;Parent=MALK_02018.t1
contig006	AUGUSTUS	CDS	554722	555556	0.4	-	0	ID=MALK_02018.t1.c1;Parent=MALK_02018.t1
contig006	AUGUSTUS	mRNA	554722	555649	0.4	-	.	ID=MALK_02018.t1;Parent=MALK_02018
contig006	AUGUSTUS	exon	555585	555649	.	-	.	ID=MALK_02018.t1.e2;Parent=MALK_02018.t1
contig006	AUGUSTUS	exon	554722	555556	.	-	.	ID=MALK_02018.t1.e1;Parent=MALK_02018.t1
contig006	maker	gene	555908	557215	.	+	.	ID=MALK_02019;prediction_source=maker_MRET:augustus_masked-contig006-processed-gene-5.23-mRNA-1
contig006	maker	CDS	555908	557215	.	+	0	ID=MALK_02019.t1.c1;Parent=MALK_02019.t1
contig006	maker	mRNA	555908	557215	.	+	.	ID=MALK_02019.t1;Parent=MALK_02019
contig006	maker	exon	555908	557215	.	+	.	ID=MALK_02019.t1.e1;Parent=MALK_02019.t1
contig006	AUGUSTUS	gene	557285	557923	0.99	+	.	ID=MALK_02020;prediction_source=augustus:contig006.g2274.t1
contig006	AUGUSTUS	CDS	557285	557923	0.99	+	0	ID=MALK_02020.t1.c1;Parent=MALK_02020.t1
contig006	AUGUSTUS	mRNA	557285	557923	0.99	+	.	ID=MALK_02020.t1;Parent=MALK_02020

contig006	AUGUSTUS	exon	557285	557923	0.99	+	.	ID=MALK_02020.t1.e1;Parent=MALK_02020.t1
contig006	maker	gene	557920	558657	.	-	.	ID=MALK_02021;prediction_source=maker_MRET:augustus_masked-contig006-processed-gene-5.49-mRNA-1
contig006	maker	CDS	557920	558657	.	-	0	ID=MALK_02021.t1.c1;Parent=MALK_02021.t1
contig006	maker	mRNA	557920	558657	.	-	.	ID=MALK_02021.t1;Parent=MALK_02021
contig006	maker	exon	557920	558657	.	-	.	ID=MALK_02021.t1.e1;Parent=MALK_02021.t1
contig006	maker	gene	558765	560021	.	+	.	ID=MALK_02022;prediction_source=maker_MRET:augustus_masked-contig006-processed-gene-5.24-mRNA-1
contig006	maker	CDS	558765	560021	.	+	0	ID=MALK_02022.t1.c1;Parent=MALK_02022.t1
contig006	maker	mRNA	558765	560021	.	+	.	ID=MALK_02022.t1;Parent=MALK_02022
contig006	maker	exon	558765	560021	.	+	.	ID=MALK_02022.t1.e1;Parent=MALK_02022.t1
contig006	AUGUSTUS	gene	560462	562519	0.94	+	.	ID=MALK_02023;prediction_source=augustus:contig006.g2277.t1
contig006	AUGUSTUS	CDS	560462	562519	0.94	+	0	ID=MALK_02023.t1.c1;Parent=MALK_02023.t1
contig006	AUGUSTUS	mRNA	560462	562519	0.94	+	.	ID=MALK_02023.t1;Parent=MALK_02023
contig006	AUGUSTUS	exon	560462	562519	0.94	+	.	ID=MALK_02023.t1.e1;Parent=MALK_02023.t1
contig006	maker	gene	562979	565420	.	-	.	ID=MALK_02024;prediction_source=maker_MRET:augustus_masked-contig006-processed-gene-5.50-mRNA-1
contig006	maker	CDS	562979	565420	.	-	0	ID=MALK_02024.t1.c1;Parent=MALK_02024.t1
contig006	maker	mRNA	562979	565420	.	-	.	ID=MALK_02024.t1;Parent=MALK_02024
contig006	maker	exon	562979	565420	.	-	.	ID=MALK_02024.t1.e1;Parent=MALK_02024.t1
contig006	maker	gene	565508	566521	.	-	.	ID=MALK_02025;prediction_source=maker_MRET:augustus_masked-contig006-processed-gene-5.51-mRNA-1
contig006	maker	CDS	565508	566521	.	-	0	ID=MALK_02025.t1.c1;Parent=MALK_02025.t1
contig006	maker	mRNA	565508	566521	.	-	.	ID=MALK_02025.t1;Parent=MALK_02025
contig006	maker	exon	565508	566521	.	-	.	ID=MALK_02025.t1.e1;Parent=MALK_02025.t1
contig006	AUGUSTUS	gene	567509	568648	0.87	+	.	ID=MALK_02026;prediction_source=braker_MRET:g2023.t1
contig006	AUGUSTUS	CDS	567509	568648	0.87	+	0	ID=MALK_02026.t1.c1;Parent=MALK_02026.t1
contig006	AUGUSTUS	mRNA	567509	568648	0.87	+	.	ID=MALK_02026.t1;Parent=MALK_02026
contig006	AUGUSTUS	exon	567509	568648	.	+	.	ID=MALK_02026.t1.e1;Parent=MALK_02026.t1
contig006	maker	gene	568678	570042	.	+	.	ID=MALK_02027;prediction_source=maker_MRET:augustus_masked-contig006-processed-gene-5.27-mRNA-1
contig006	maker	CDS	568678	570042	.	+	0	ID=MALK_02027.t1.c1;Parent=MALK_02027.t1
contig006	maker	mRNA	568678	570042	.	+	.	ID=MALK_02027.t1;Parent=MALK_02027
contig006	maker	exon	568678	570042	.	+	.	ID=MALK_02027.t1.e1;Parent=MALK_02027.t1
contig006	AUGUSTUS	gene	570128	571852	0.49	+	.	ID=MALK_02028;prediction_source=augustus:contig006.g2284.t1
contig006	AUGUSTUS	CDS	570128	571852	0.49	+	0	ID=MALK_02028.t1.c1;Parent=MALK_02028.t1
contig006	AUGUSTUS	mRNA	570128	571852	0.49	+	.	ID=MALK_02028.t1;Parent=MALK_02028
contig006	AUGUSTUS	exon	570128	571852	0.49	+	.	ID=MALK_02028.t1.e1;Parent=MALK_02028.t1
contig006	AUGUSTUS	gene	571885	573091	0.92	+	.	ID=MALK_02029;prediction_source=braker_MRET:g2026.t1
contig006	AUGUSTUS	CDS	571885	573047	0.92	+	0	ID=MALK_02029.t1.c1;Parent=MALK_02029.t1
contig006	AUGUSTUS	CDS	573082	573091	0.92	+	0	ID=MALK_02029.t1.c2;Parent=MALK_02029.t1
contig006	AUGUSTUS	mRNA	571885	573091	0.92	+	.	ID=MALK_02029.t1;Parent=MALK_02029
contig006	AUGUSTUS	exon	571885	573047	.	+	.	ID=MALK_02029.t1.e1;Parent=MALK_02029.t1
contig006	AUGUSTUS	exon	573082	573091	.	+	.	ID=MALK_02029.t1.e2;Parent=MALK_02029.t1
contig006	AUGUSTUS	gene	573131	575100	0.15	-	.	ID=MALK_02030;prediction_source=braker_MRET:g2027.t1
contig006	AUGUSTUS	CDS	574292	575100	0.23	-	0	ID=MALK_02030.t1.c2;Parent=MALK_02030.t1
contig006	AUGUSTUS	CDS	573131	574253	0.23	-	0	ID=MALK_02030.t1.c1;Parent=MALK_02030.t1
contig006	AUGUSTUS	mRNA	573131	575100	0.15	-	.	ID=MALK_02030.t1;Parent=MALK_02030

contig006	AUGUSTUS	exon	574292	575100	.	-	.	ID=MALK_02030.t1.e2;Parent=MALK_02030.t1
contig006	AUGUSTUS	exon	573131	574253	.	-	.	ID=MALK_02030.t1.e1;Parent=MALK_02030.t1
contig006	AUGUSTUS	gene	576064	576492	0.58	+	.	ID=MALK_02031;prediction_source=augustus:contig006.g2286.t1
contig006	AUGUSTUS	CDS	576064	576492	0.58	+	0	ID=MALK_02031.t1.c1;Parent=MALK_02031.t1
contig006	AUGUSTUS	mRNA	576064	576492	0.58	+	.	ID=MALK_02031.t1;Parent=MALK_02031
contig006	AUGUSTUS	exon	576064	576492	0.58	+	.	ID=MALK_02031.t1.e1;Parent=MALK_02031.t1
contig006	AUGUSTUS	gene	577646	578890	0.4	+	.	ID=MALK_02032;prediction_source=augustus:contig006.g2287.t1
contig006	AUGUSTUS	CDS	577646	578890	0.4	+	0	ID=MALK_02032.t1.c1;Parent=MALK_02032.t1
contig006	AUGUSTUS	mRNA	577646	578890	0.4	+	.	ID=MALK_02032.t1;Parent=MALK_02032
contig006	AUGUSTUS	exon	577646	578890	0.4	+	.	ID=MALK_02032.t1.e1;Parent=MALK_02032.t1
contig006	maker	gene	579197	580525	.	+	.	ID=MALK_02033;prediction_source=maker_MRET:augustus_masked-contig006-processed-gene-5.30-mRNA-1
contig006	maker	CDS	579197	580525	.	+	0	ID=MALK_02033.t1.c1;Parent=MALK_02033.t1
contig006	maker	mRNA	579197	580525	.	+	.	ID=MALK_02033.t1;Parent=MALK_02033
contig006	maker	exon	579197	580525	.	+	.	ID=MALK_02033.t1.e1;Parent=MALK_02033.t1
contig006	maker	gene	581634	582965	.	+	.	ID=MALK_02034;prediction_source=maker_MRET:augustus_masked-contig006-processed-gene-5.31-mRNA-1
contig006	maker	CDS	581634	582965	.	+	0	ID=MALK_02034.t1.c1;Parent=MALK_02034.t1
contig006	maker	mRNA	581634	582965	.	+	.	ID=MALK_02034.t1;Parent=MALK_02034
contig006	maker	exon	581634	582965	.	+	.	ID=MALK_02034.t1.e1;Parent=MALK_02034.t1
contig006	AUGUSTUS	gene	583258	584370	0.4	-	.	ID=MALK_02035;prediction_source=augustus:contig006.g2291.t1
contig006	AUGUSTUS	CDS	583258	584370	0.4	-	0	ID=MALK_02035.t1.c1;Parent=MALK_02035.t1
contig006	AUGUSTUS	mRNA	583258	584370	0.4	-	.	ID=MALK_02035.t1;Parent=MALK_02035
contig006	AUGUSTUS	exon	583258	584370	0.4	-	.	ID=MALK_02035.t1.e1;Parent=MALK_02035.t1
contig006	maker	gene	584877	586316	.	-	.	ID=MALK_02036;prediction_source=maker_MRET:augustus_masked-contig006-processed-gene-5.54-mRNA-1
contig006	maker	CDS	584877	586316	.	-	0	ID=MALK_02036.t1.c1;Parent=MALK_02036.t1
contig006	maker	mRNA	584877	586316	.	-	.	ID=MALK_02036.t1;Parent=MALK_02036
contig006	maker	exon	584877	586316	.	-	.	ID=MALK_02036.t1.e1;Parent=MALK_02036.t1
contig006	AUGUSTUS	gene	587079	588467	0.65	+	.	ID=MALK_02037;prediction_source=braker_MRET:g2034.t1
contig006	AUGUSTUS	CDS	587079	588467	0.65	+	0	ID=MALK_02037.t1.c1;Parent=MALK_02037.t1
contig006	AUGUSTUS	mRNA	587079	588467	0.65	+	.	ID=MALK_02037.t1;Parent=MALK_02037
contig006	AUGUSTUS	exon	587079	588467	.	+	.	ID=MALK_02037.t1.e1;Parent=MALK_02037.t1
contig006	AUGUSTUS	gene	588469	590388	1	-	.	ID=MALK_02038;prediction_source=braker_MRET:g2035.t1
contig006	AUGUSTUS	CDS	588469	590388	1	-	0	ID=MALK_02038.t1.c1;Parent=MALK_02038.t1
contig006	AUGUSTUS	mRNA	588469	590388	1	-	.	ID=MALK_02038.t1;Parent=MALK_02038
contig006	AUGUSTUS	exon	588469	590388	.	-	.	ID=MALK_02038.t1.e1;Parent=MALK_02038.t1
contig006	AUGUSTUS	gene	590405	592393	1	-	.	ID=MALK_02039;prediction_source=braker_MRET:g2036.t1
contig006	AUGUSTUS	CDS	590405	592393	1	-	0	ID=MALK_02039.t1.c1;Parent=MALK_02039.t1
contig006	AUGUSTUS	mRNA	590405	592393	1	-	.	ID=MALK_02039.t1;Parent=MALK_02039
contig006	AUGUSTUS	exon	590405	592393	.	-	.	ID=MALK_02039.t1.e1;Parent=MALK_02039.t1
contig006	AUGUSTUS	gene	592454	593734	1	+	.	ID=MALK_02040;prediction_source=braker_MRET:g2037.t1
contig006	AUGUSTUS	CDS	592454	593734	1	+	0	ID=MALK_02040.t1.c1;Parent=MALK_02040.t1
contig006	AUGUSTUS	mRNA	592454	593734	1	+	.	ID=MALK_02040.t1;Parent=MALK_02040
contig006	AUGUSTUS	exon	592454	593734	.	+	.	ID=MALK_02040.t1.e1;Parent=MALK_02040.t1
contig006	AUGUSTUS	gene	593743	594585	1	+	.	ID=MALK_02041;prediction_source=augustus:contig006.g2297.t1

contig006	AUGUSTUS	CDS	593743	594585	1	+	0	ID=MALK_02041.t1.c1;Parent=MALK_02041.t1
contig006	AUGUSTUS	mRNA	593743	594585	1	+	.	ID=MALK_02041.t1;Parent=MALK_02041
contig006	AUGUSTUS	exon	593743	594585	1	+	.	ID=MALK_02041.t1.e1;Parent=MALK_02041.t1
contig006	AUGUSTUS	gene	594610	596033	0.54	+	.	ID=MALK_02042;prediction_source=braker_MRET:g2039.t1
contig006	AUGUSTUS	CDS	594610	595437	0.56	+	0	ID=MALK_02042.t1.c1;Parent=MALK_02042.t1
contig006	AUGUSTUS	CDS	595548	596033	0.56	+	0	ID=MALK_02042.t1.c2;Parent=MALK_02042.t1
contig006	AUGUSTUS	mRNA	594610	596033	0.54	+	.	ID=MALK_02042.t1;Parent=MALK_02042
contig006	AUGUSTUS	exon	594610	595437	.	+	.	ID=MALK_02042.t1.e1;Parent=MALK_02042.t1
contig006	AUGUSTUS	exon	595548	596033	.	+	.	ID=MALK_02042.t1.e2;Parent=MALK_02042.t1
contig006	AUGUSTUS	gene	596058	598044	0.1	+	.	ID=MALK_02043;prediction_source=braker_MRET:g2040.t1
contig006	AUGUSTUS	CDS	596058	596222	0.47	+	0	ID=MALK_02043.t1.c1;Parent=MALK_02043.t1
contig006	AUGUSTUS	CDS	596252	596449	0.47	+	0	ID=MALK_02043.t1.c2;Parent=MALK_02043.t1
contig006	AUGUSTUS	CDS	596497	598044	0.47	+	0	ID=MALK_02043.t1.c3;Parent=MALK_02043.t1
contig006	AUGUSTUS	mRNA	596058	598044	0.1	+	.	ID=MALK_02043.t1;Parent=MALK_02043
contig006	AUGUSTUS	exon	596058	596222	.	+	.	ID=MALK_02043.t1.e1;Parent=MALK_02043.t1
contig006	AUGUSTUS	exon	596252	596449	.	+	.	ID=MALK_02043.t1.e2;Parent=MALK_02043.t1
contig006	AUGUSTUS	exon	596497	598044	.	+	.	ID=MALK_02043.t1.e3;Parent=MALK_02043.t1
contig006	maker	gene	598137	601883	.	+	.	ID=MALK_02044;prediction_source=maker_MRET:augustus_masked-contig006-processed-gene-6.50-mRNA-1
contig006	maker	CDS	598137	601883	.	+	0	ID=MALK_02044.t1.c1;Parent=MALK_02044.t1
contig006	maker	mRNA	598137	601883	.	+	.	ID=MALK_02044.t1;Parent=MALK_02044
contig006	maker	exon	598137	601883	.	+	.	ID=MALK_02044.t1.e1;Parent=MALK_02044.t1
contig006	AUGUSTUS	gene	601913	603367	1	+	.	ID=MALK_02045;prediction_source=braker_MRET:g2042.t1
contig006	AUGUSTUS	CDS	601913	603367	1	+	0	ID=MALK_02045.t1.c1;Parent=MALK_02045.t1
contig006	AUGUSTUS	mRNA	601913	603367	1	+	.	ID=MALK_02045.t1;Parent=MALK_02045
contig006	AUGUSTUS	exon	601913	603367	.	+	.	ID=MALK_02045.t1.e1;Parent=MALK_02045.t1
contig006	AUGUSTUS	gene	603729	605042	0.27	+	.	ID=MALK_02046;prediction_source=braker_MRET:g2043.t1
contig006	AUGUSTUS	CDS	603729	605042	0.27	+	0	ID=MALK_02046.t1.c1;Parent=MALK_02046.t1
contig006	AUGUSTUS	mRNA	603729	605042	0.27	+	.	ID=MALK_02046.t1;Parent=MALK_02046
contig006	AUGUSTUS	exon	603729	605042	.	+	.	ID=MALK_02046.t1.e1;Parent=MALK_02046.t1
contig006	maker	gene	605037	606266	.	-	.	ID=MALK_02047;prediction_source=maker_MRET:augustus_masked-contig006-processed-gene-6.68-mRNA-1
contig006	maker	CDS	605037	606266	.	-	0	ID=MALK_02047.t1.c1;Parent=MALK_02047.t1
contig006	maker	mRNA	605037	606266	.	-	.	ID=MALK_02047.t1;Parent=MALK_02047
contig006	maker	exon	605037	606266	.	-	.	ID=MALK_02047.t1.e1;Parent=MALK_02047.t1
contig006	maker	gene	606373	608349	.	-	.	ID=MALK_02048;prediction_source=maker_MRET:augustus_masked-contig006-processed-gene-6.69-mRNA-1
contig006	maker	CDS	606373	608349	.	-	0	ID=MALK_02048.t1.c1;Parent=MALK_02048.t1
contig006	maker	mRNA	606373	608349	.	-	.	ID=MALK_02048.t1;Parent=MALK_02048
contig006	maker	exon	606373	608349	.	-	.	ID=MALK_02048.t1.e1;Parent=MALK_02048.t1
contig006	AUGUSTUS	gene	608504	610444	0.77	-	.	ID=MALK_02049;prediction_source=augustus:contig006.g2304.t1
contig006	AUGUSTUS	CDS	608504	610444	0.77	-	0	ID=MALK_02049.t1.c1;Parent=MALK_02049.t1
contig006	AUGUSTUS	mRNA	608504	610444	0.77	-	.	ID=MALK_02049.t1;Parent=MALK_02049
contig006	AUGUSTUS	exon	608504	610444	0.77	-	.	ID=MALK_02049.t1.e1;Parent=MALK_02049.t1
contig006	AUGUSTUS	gene	610528	612216	0.98	+	.	ID=MALK_02050;prediction_source=augustus:contig006.g2305.t1
contig006	AUGUSTUS	CDS	610528	612216	0.98	+	0	ID=MALK_02050.t1.c1;Parent=MALK_02050.t1

contig006	AUGUSTUS	mRNA	610528	612216	0.98	+	.	ID=MALK_02050.t1;Parent=MALK_02050
contig006	AUGUSTUS	exon	610528	612216	0.98	+	.	ID=MALK_02050.t1.e1;Parent=MALK_02050.t1
contig006	AUGUSTUS	gene	612770	613393	1	+	.	ID=MALK_02051;prediction_source=braker_MRET:g2048.t1
contig006	AUGUSTUS	CDS	612770	612784	1	+	0	ID=MALK_02051.t1.c1;Parent=MALK_02051.t1
contig006	AUGUSTUS	CDS	612833	613049	1	+	0	ID=MALK_02051.t1.c2;Parent=MALK_02051.t1
contig006	AUGUSTUS	CDS	613359	613393	1	+	0	ID=MALK_02051.t1.c3;Parent=MALK_02051.t1
contig006	AUGUSTUS	mRNA	612770	613393	1	+	.	ID=MALK_02051.t1;Parent=MALK_02051
contig006	AUGUSTUS	exon	612770	612784	.	+	.	ID=MALK_02051.t1.e1;Parent=MALK_02051.t1
contig006	AUGUSTUS	exon	612833	613049	.	+	.	ID=MALK_02051.t1.e2;Parent=MALK_02051.t1
contig006	AUGUSTUS	exon	613359	613393	.	+	.	ID=MALK_02051.t1.e3;Parent=MALK_02051.t1
contig006	AUGUSTUS	gene	613718	616300	0.73	+	.	ID=MALK_02052;prediction_source=braker_MRET:g2049.t1
contig006	AUGUSTUS	CDS	613718	616300	0.73	+	0	ID=MALK_02052.t1.c1;Parent=MALK_02052.t1
contig006	AUGUSTUS	mRNA	613718	616300	0.73	+	.	ID=MALK_02052.t1;Parent=MALK_02052
contig006	AUGUSTUS	exon	613718	616300	.	+	.	ID=MALK_02052.t1.e1;Parent=MALK_02052.t1
contig006	AUGUSTUS	gene	616392	618377	0.99	+	.	ID=MALK_02053;prediction_source=braker_MRET:g2050.t1
contig006	AUGUSTUS	CDS	616392	618377	0.99	+	0	ID=MALK_02053.t1.c1;Parent=MALK_02053.t1
contig006	AUGUSTUS	mRNA	616392	618377	0.99	+	.	ID=MALK_02053.t1;Parent=MALK_02053
contig006	AUGUSTUS	exon	616392	618377	.	+	.	ID=MALK_02053.t1.e1;Parent=MALK_02053.t1
contig006	AUGUSTUS	gene	618387	619052	0.99	-	.	ID=MALK_02054;prediction_source=braker_MRET:g2051.t1
contig006	AUGUSTUS	CDS	618387	619052	0.99	-	0	ID=MALK_02054.t1.c1;Parent=MALK_02054.t1
contig006	AUGUSTUS	mRNA	618387	619052	0.99	-	.	ID=MALK_02054.t1;Parent=MALK_02054
contig006	AUGUSTUS	exon	618387	619052	.	-	.	ID=MALK_02054.t1.e1;Parent=MALK_02054.t1
contig006	AUGUSTUS	gene	619562	620971	0.67	+	.	ID=MALK_02055;prediction_source=augustus:contig006.g2310.t1
contig006	AUGUSTUS	CDS	619562	620971	0.67	+	0	ID=MALK_02055.t1.c1;Parent=MALK_02055.t1
contig006	AUGUSTUS	mRNA	619562	620971	0.67	+	.	ID=MALK_02055.t1;Parent=MALK_02055
contig006	AUGUSTUS	exon	619562	620971	0.67	+	.	ID=MALK_02055.t1.e1;Parent=MALK_02055.t1
contig006	maker	gene	620989	623112	.	-	.	ID=MALK_02056;prediction_source=maker_MRET:augustus_masked-contig006-processed-gene-6.71-mRNA-1
contig006	maker	CDS	620989	623112	.	-	0	ID=MALK_02056.t1.c1;Parent=MALK_02056.t1
contig006	maker	mRNA	620989	623112	.	-	.	ID=MALK_02056.t1;Parent=MALK_02056
contig006	maker	exon	620989	623112	.	-	.	ID=MALK_02056.t1.e1;Parent=MALK_02056.t1
contig006	maker	gene	623598	624896	.	+	.	ID=MALK_02057;prediction_source=maker_MRET:augustus_masked-contig006-processed-gene-6.57-mRNA-1
contig006	maker	CDS	623598	623609	.	+	0	ID=MALK_02057.t1.c1;Parent=MALK_02057.t1
contig006	maker	CDS	623685	624896	.	+	0	ID=MALK_02057.t1.c2;Parent=MALK_02057.t1
contig006	maker	mRNA	623598	624896	.	+	.	ID=MALK_02057.t1;Parent=MALK_02057
contig006	maker	exon	623598	623609	.	+	.	ID=MALK_02057.t1.e1;Parent=MALK_02057.t1
contig006	maker	exon	623685	624896	.	+	.	ID=MALK_02057.t1.e2;Parent=MALK_02057.t1
contig006	AUGUSTUS	gene	624974	625825	0.97	-	.	ID=MALK_02058;prediction_source=augustus:contig006.g2313.t1
contig006	AUGUSTUS	CDS	624974	625825	0.97	-	0	ID=MALK_02058.t1.c1;Parent=MALK_02058.t1
contig006	AUGUSTUS	mRNA	624974	625825	0.97	-	.	ID=MALK_02058.t1;Parent=MALK_02058
contig006	AUGUSTUS	exon	624974	625825	0.97	-	.	ID=MALK_02058.t1.e1;Parent=MALK_02058.t1
contig006	maker	gene	625968	626375	.	+	.	ID=MALK_02059;prediction_source=maker_MRET:augustus_masked-contig006-processed-gene-6.58-mRNA-1
contig006	maker	CDS	625968	626375	.	+	0	ID=MALK_02059.t1.c1;Parent=MALK_02059.t1
contig006	maker	mRNA	625968	626375	.	+	.	ID=MALK_02059.t1;Parent=MALK_02059

contig006	maker	exon	625968	626375	.	+	.	ID=MALK_02059.t1.e1;Parent=MALK_02059.t1
contig006	maker	gene	626417	628261	.	-	.	ID=MALK_02060;prediction_source=maker_MRET:augustus_masked-contig006-processed-gene-6.73-mRNA-1
contig006	maker	CDS	626417	628261	.	-	0	ID=MALK_02060.t1.c1;Parent=MALK_02060.t1
contig006	maker	mRNA	626417	628261	.	-	.	ID=MALK_02060.t1;Parent=MALK_02060
contig006	maker	exon	626417	628261	.	-	.	ID=MALK_02060.t1.e1;Parent=MALK_02060.t1
contig006	AUGUSTUS	gene	628437	630407	0.99	+	.	ID=MALK_02061;prediction_source=augustus:contig006.g2316.t1
contig006	AUGUSTUS	CDS	628437	630407	0.99	+	0	ID=MALK_02061.t1.c1;Parent=MALK_02061.t1
contig006	AUGUSTUS	mRNA	628437	630407	0.99	+	.	ID=MALK_02061.t1;Parent=MALK_02061
contig006	AUGUSTUS	exon	628437	630407	0.99	+	.	ID=MALK_02061.t1.e1;Parent=MALK_02061.t1
contig006	maker	gene	630442	633531	.	-	.	ID=MALK_02062;prediction_source=maker_MRET:augustus_masked-contig006-processed-gene-6.74-mRNA-1
contig006	maker	CDS	630442	633531	.	-	0	ID=MALK_02062.t1.c1;Parent=MALK_02062.t1
contig006	maker	mRNA	630442	633531	.	-	.	ID=MALK_02062.t1;Parent=MALK_02062
contig006	maker	exon	630442	633531	.	-	.	ID=MALK_02062.t1.e1;Parent=MALK_02062.t1
contig006	AUGUSTUS	gene	633719	635524	0.82	+	.	ID=MALK_02063;prediction_source=braker_MRET:g2060.t1
contig006	AUGUSTUS	CDS	633719	635524	0.82	+	0	ID=MALK_02063.t1.c1;Parent=MALK_02063.t1
contig006	AUGUSTUS	mRNA	633719	635524	0.82	+	.	ID=MALK_02063.t1;Parent=MALK_02063
contig006	AUGUSTUS	exon	633719	635524	.	+	.	ID=MALK_02063.t1.e1;Parent=MALK_02063.t1
contig006	AUGUSTUS	gene	635563	640218	1	-	.	ID=MALK_02064;prediction_source=augustus:contig006.g2319.t1
contig006	AUGUSTUS	CDS	635563	640218	1	-	0	ID=MALK_02064.t1.c1;Parent=MALK_02064.t1
contig006	AUGUSTUS	mRNA	635563	640218	1	-	.	ID=MALK_02064.t1;Parent=MALK_02064
contig006	AUGUSTUS	exon	635563	640218	1	-	.	ID=MALK_02064.t1.e1;Parent=MALK_02064.t1
contig006	maker	gene	640445	643363	.	+	.	ID=MALK_02065;prediction_source=maker_MRET:augustus_masked-contig006-processed-gene-6.61-mRNA-1
contig006	maker	CDS	640445	643363	.	+	0	ID=MALK_02065.t1.c1;Parent=MALK_02065.t1
contig006	maker	mRNA	640445	643363	.	+	.	ID=MALK_02065.t1;Parent=MALK_02065
contig006	maker	exon	640445	643363	.	+	.	ID=MALK_02065.t1.e1;Parent=MALK_02065.t1
contig006	AUGUSTUS	gene	643376	643951	0.99	-	.	ID=MALK_02066;prediction_source=braker_MRET:g2063.t1
contig006	AUGUSTUS	CDS	643376	643951	0.99	-	0	ID=MALK_02066.t1.c1;Parent=MALK_02066.t1
contig006	AUGUSTUS	mRNA	643376	643951	0.99	-	.	ID=MALK_02066.t1;Parent=MALK_02066
contig006	AUGUSTUS	exon	643376	643951	.	-	.	ID=MALK_02066.t1.e1;Parent=MALK_02066.t1
contig006	AUGUSTUS	gene	644004	645531	0.99	+	.	ID=MALK_02067;prediction_source=braker_MRET:g2064.t1
contig006	AUGUSTUS	CDS	644004	645470	1	+	0	ID=MALK_02067.t1.c1;Parent=MALK_02067.t1
contig006	AUGUSTUS	CDS	645517	645531	1	+	0	ID=MALK_02067.t1.c2;Parent=MALK_02067.t1
contig006	AUGUSTUS	mRNA	644004	645531	0.99	+	.	ID=MALK_02067.t1;Parent=MALK_02067
contig006	AUGUSTUS	exon	644004	645470	.	+	.	ID=MALK_02067.t1.e1;Parent=MALK_02067.t1
contig006	AUGUSTUS	exon	645517	645531	.	+	.	ID=MALK_02067.t1.e2;Parent=MALK_02067.t1
contig006	AUGUSTUS	gene	645534	646922	0.72	-	.	ID=MALK_02068;prediction_source=braker_MRET:g2065.t1
contig006	AUGUSTUS	CDS	645534	646922	0.72	-	0	ID=MALK_02068.t1.c1;Parent=MALK_02068.t1
contig006	AUGUSTUS	mRNA	645534	646922	0.72	-	.	ID=MALK_02068.t1;Parent=MALK_02068
contig006	AUGUSTUS	exon	645534	646922	.	-	.	ID=MALK_02068.t1.e1;Parent=MALK_02068.t1
contig006	AUGUSTUS	gene	647019	648974	0.44	-	.	ID=MALK_02069;prediction_source=braker_MRET:g2066.t1
contig006	AUGUSTUS	CDS	648104	648974	0.73	-	0	ID=MALK_02069.t1.c2;Parent=MALK_02069.t1
contig006	AUGUSTUS	CDS	647019	648076	0.73	-	0	ID=MALK_02069.t1.c1;Parent=MALK_02069.t1
contig006	AUGUSTUS	mRNA	647019	648974	0.44	-	.	ID=MALK_02069.t1;Parent=MALK_02069

contig006	AUGUSTUS	exon	648104	648974	.	-	.	ID=MALK_02069.t1.e2;Parent=MALK_02069.t1
contig006	AUGUSTUS	exon	647019	648076	.	-	.	ID=MALK_02069.t1.e1;Parent=MALK_02069.t1
contig006	maker	gene	649239	651584	.	+	.	ID=MALK_02070;prediction_source=maker_MRET:augustus_masked-contig006-processed-gene-6.62-mRNA-1
contig006	maker	CDS	649239	651584	.	+	0	ID=MALK_02070.t1.c1;Parent=MALK_02070.t1
contig006	maker	mRNA	649239	651584	.	+	.	ID=MALK_02070.t1;Parent=MALK_02070
contig006	maker	exon	649239	651584	.	+	.	ID=MALK_02070.t1.e1;Parent=MALK_02070.t1
contig006	AUGUSTUS	gene	651623	652471	0.48	+	.	ID=MALK_02071;prediction_source=augustus:contig006.g2324.t1
contig006	AUGUSTUS	CDS	651623	652471	0.48	+	0	ID=MALK_02071.t1.c1;Parent=MALK_02071.t1
contig006	AUGUSTUS	mRNA	651623	652471	0.48	+	.	ID=MALK_02071.t1;Parent=MALK_02071
contig006	AUGUSTUS	exon	651623	652471	0.48	+	.	ID=MALK_02071.t1.e1;Parent=MALK_02071.t1
contig006	AUGUSTUS	gene	652451	657430	0.96	-	.	ID=MALK_02072;prediction_source=augustus:contig006.g2325.t1
contig006	AUGUSTUS	CDS	652451	657430	0.96	-	0	ID=MALK_02072.t1.c1;Parent=MALK_02072.t1
contig006	AUGUSTUS	mRNA	652451	657430	0.96	-	.	ID=MALK_02072.t1;Parent=MALK_02072
contig006	AUGUSTUS	exon	652451	657430	0.96	-	.	ID=MALK_02072.t1.e1;Parent=MALK_02072.t1
contig006	AUGUSTUS	gene	657931	658487	0.36	-	.	ID=MALK_02073;prediction_source=braker_MRET:g2070.t1
contig006	AUGUSTUS	CDS	658187	658487	0.45	-	0	ID=MALK_02073.t1.c3;Parent=MALK_02073.t1
contig006	AUGUSTUS	CDS	658118	658146	0.45	-	0	ID=MALK_02073.t1.c2;Parent=MALK_02073.t1
contig006	AUGUSTUS	CDS	657931	658062	0.45	-	0	ID=MALK_02073.t1.c1;Parent=MALK_02073.t1
contig006	AUGUSTUS	mRNA	657931	658487	0.36	-	.	ID=MALK_02073.t1;Parent=MALK_02073
contig006	AUGUSTUS	exon	658187	658487	.	-	.	ID=MALK_02073.t1.e3;Parent=MALK_02073.t1
contig006	AUGUSTUS	exon	658118	658146	.	-	.	ID=MALK_02073.t1.e2;Parent=MALK_02073.t1
contig006	AUGUSTUS	exon	657931	658062	.	-	.	ID=MALK_02073.t1.e1;Parent=MALK_02073.t1
contig006	AUGUSTUS	gene	658547	660742	0.58	-	.	ID=MALK_02074;prediction_source=augustus:contig006.g2327.t1
contig006	AUGUSTUS	CDS	658547	660742	0.58	-	0	ID=MALK_02074.t1.c1;Parent=MALK_02074.t1
contig006	AUGUSTUS	mRNA	658547	660742	0.58	-	.	ID=MALK_02074.t1;Parent=MALK_02074
contig006	AUGUSTUS	exon	658547	660742	0.58	-	.	ID=MALK_02074.t1.e1;Parent=MALK_02074.t1
contig006	AUGUSTUS	gene	660846	662000	0.75	-	.	ID=MALK_02075;prediction_source=braker_MRET:g2072.t1
contig006	AUGUSTUS	CDS	660846	662000	0.75	-	0	ID=MALK_02075.t1.c1;Parent=MALK_02075.t1
contig006	AUGUSTUS	mRNA	660846	662000	0.75	-	.	ID=MALK_02075.t1;Parent=MALK_02075
contig006	AUGUSTUS	exon	660846	662000	.	-	.	ID=MALK_02075.t1.e1;Parent=MALK_02075.t1
contig006	AUGUSTUS	gene	662951	664885	0.48	-	.	ID=MALK_02076;prediction_source=braker_MRET:g2073.t1
contig006	AUGUSTUS	CDS	662951	664885	0.48	-	0	ID=MALK_02076.t1.c1;Parent=MALK_02076.t1
contig006	AUGUSTUS	mRNA	662951	664885	0.48	-	.	ID=MALK_02076.t1;Parent=MALK_02076
contig006	AUGUSTUS	exon	662951	664885	.	-	.	ID=MALK_02076.t1.e1;Parent=MALK_02076.t1
contig006	AUGUSTUS	gene	665354	667123	0.85	-	.	ID=MALK_02077;prediction_source=augustus:contig006.g2332.t1
contig006	AUGUSTUS	CDS	665354	667123	0.85	-	0	ID=MALK_02077.t1.c1;Parent=MALK_02077.t1
contig006	AUGUSTUS	mRNA	665354	667123	0.85	-	.	ID=MALK_02077.t1;Parent=MALK_02077
contig006	AUGUSTUS	exon	665354	667123	0.85	-	.	ID=MALK_02077.t1.e1;Parent=MALK_02077.t1
contig006	AUGUSTUS	gene	667274	668452	0.96	+	.	ID=MALK_02078;prediction_source=braker_MRET:g2075.t1
contig006	AUGUSTUS	CDS	667274	668452	0.96	+	0	ID=MALK_02078.t1.c1;Parent=MALK_02078.t1
contig006	AUGUSTUS	mRNA	667274	668452	0.96	+	.	ID=MALK_02078.t1;Parent=MALK_02078
contig006	AUGUSTUS	exon	667274	668452	.	+	.	ID=MALK_02078.t1.e1;Parent=MALK_02078.t1
contig006	AUGUSTUS	gene	668617	670329	0.83	+	.	ID=MALK_02079;prediction_source=braker_MRET:g2076.t1

contig006	AUGUSTUS	CDS	668617	670329	0.83	+	0	ID=MALK_02079.t1.c1;Parent=MALK_02079.t1
contig006	AUGUSTUS	mRNA	668617	670329	0.83	+	.	ID=MALK_02079.t1;Parent=MALK_02079
contig006	AUGUSTUS	exon	668617	670329	.	+	.	ID=MALK_02079.t1.e1;Parent=MALK_02079.t1
contig006	maker	gene	670334	670693	.	-	.	ID=MALK_02080;prediction_source=maker_MRET:augustus_masked-contig006-processed-gene-6.83-mRNA-1
contig006	maker	CDS	670334	670693	.	-	0	ID=MALK_02080.t1.c1;Parent=MALK_02080.t1
contig006	maker	mRNA	670334	670693	.	-	.	ID=MALK_02080.t1;Parent=MALK_02080
contig006	maker	exon	670334	670693	.	-	.	ID=MALK_02080.t1.e1;Parent=MALK_02080.t1
contig006	AUGUSTUS	gene	670801	672774	0.66	+	.	ID=MALK_02081;prediction_source=augustus:contig006.g2335.t1
contig006	AUGUSTUS	CDS	670801	672774	0.66	+	0	ID=MALK_02081.t1.c1;Parent=MALK_02081.t1
contig006	AUGUSTUS	mRNA	670801	672774	0.66	+	.	ID=MALK_02081.t1;Parent=MALK_02081
contig006	AUGUSTUS	exon	670801	672774	0.66	+	.	ID=MALK_02081.t1.e1;Parent=MALK_02081.t1
contig006	AUGUSTUS	gene	672778	673878	0.74	-	.	ID=MALK_02082;prediction_source=augustus:contig006.g2337.t1
contig006	AUGUSTUS	CDS	672778	673878	0.74	-	0	ID=MALK_02082.t1.c1;Parent=MALK_02082.t1
contig006	AUGUSTUS	mRNA	672778	673878	0.74	-	.	ID=MALK_02082.t1;Parent=MALK_02082
contig006	AUGUSTUS	exon	672778	673878	0.74	-	.	ID=MALK_02082.t1.e1;Parent=MALK_02082.t1
contig006	maker	gene	674067	678056	.	+	.	ID=MALK_02083;prediction_source=maker_MRET:augustus_masked-contig006-processed-gene-6.65-mRNA-1
contig006	maker	CDS	674067	678056	.	+	0	ID=MALK_02083.t1.c1;Parent=MALK_02083.t1
contig006	maker	mRNA	674067	678056	.	+	.	ID=MALK_02083.t1;Parent=MALK_02083
contig006	maker	exon	674067	678056	.	+	.	ID=MALK_02083.t1.e1;Parent=MALK_02083.t1
contig006	AUGUSTUS	gene	678078	679005	0.99	-	.	ID=MALK_02084;prediction_source=braker_MRET:g2081.t1
contig006	AUGUSTUS	CDS	678246	679005	0.99	-	0	ID=MALK_02084.t1.c2;Parent=MALK_02084.t1
contig006	AUGUSTUS	CDS	678078	678211	0.99	-	0	ID=MALK_02084.t1.c1;Parent=MALK_02084.t1
contig006	AUGUSTUS	mRNA	678078	679005	0.99	-	.	ID=MALK_02084.t1;Parent=MALK_02084
contig006	AUGUSTUS	exon	678246	679005	.	-	.	ID=MALK_02084.t1.e2;Parent=MALK_02084.t1
contig006	AUGUSTUS	exon	678078	678211	.	-	.	ID=MALK_02084.t1.e1;Parent=MALK_02084.t1
contig006	AUGUSTUS	gene	679095	680804	0.61	-	.	ID=MALK_02085;prediction_source=augustus:contig006.g2341.t1
contig006	AUGUSTUS	CDS	679095	680804	0.61	-	0	ID=MALK_02085.t1.c1;Parent=MALK_02085.t1
contig006	AUGUSTUS	mRNA	679095	680804	0.61	-	.	ID=MALK_02085.t1;Parent=MALK_02085
contig006	AUGUSTUS	exon	679095	680804	0.61	-	.	ID=MALK_02085.t1.e1;Parent=MALK_02085.t1
contig006	maker	gene	680843	684097	.	-	.	ID=MALK_02086;prediction_source=maker_MRET:augustus_masked-contig006-processed-gene-6.86-mRNA-1
contig006	maker	CDS	680843	684097	.	-	0	ID=MALK_02086.t1.c1;Parent=MALK_02086.t1
contig006	maker	mRNA	680843	684097	.	-	.	ID=MALK_02086.t1;Parent=MALK_02086
contig006	maker	exon	680843	684097	.	-	.	ID=MALK_02086.t1.e1;Parent=MALK_02086.t1
contig006	AUGUSTUS	gene	684193	685273	0.91	+	.	ID=MALK_02087;prediction_source=braker_MRET:g2083.t1
contig006	AUGUSTUS	CDS	684193	685210	1	+	0	ID=MALK_02087.t1.c1;Parent=MALK_02087.t1
contig006	AUGUSTUS	CDS	685254	685273	1	+	0	ID=MALK_02087.t1.c2;Parent=MALK_02087.t1
contig006	AUGUSTUS	mRNA	684193	685273	0.91	+	.	ID=MALK_02087.t1;Parent=MALK_02087
contig006	AUGUSTUS	exon	684193	685210	.	+	.	ID=MALK_02087.t1.e1;Parent=MALK_02087.t1
contig006	AUGUSTUS	exon	685254	685273	.	+	.	ID=MALK_02087.t1.e2;Parent=MALK_02087.t1
contig006	maker	gene	685452	686654	.	-	.	ID=MALK_02088;prediction_source=maker_MRET:augustus_masked-contig006-processed-gene-6.87-mRNA-1
contig006	maker	CDS	685452	686654	.	-	0	ID=MALK_02088.t1.c1;Parent=MALK_02088.t1
contig006	maker	mRNA	685452	686654	.	-	.	ID=MALK_02088.t1;Parent=MALK_02088
contig006	maker	exon	685452	686654	.	-	.	ID=MALK_02088.t1.e1;Parent=MALK_02088.t1

contig006	maker	gene	686795	688957	.	-	.	ID=MALK_02089;prediction_source=maker_MRET:augustus_masked-contig006-processed-gene-6.88-mRNA-1
contig006	maker	CDS	686795	688957	.	-	0	ID=MALK_02089.t1.c1;Parent=MALK_02089.t1
contig006	maker	mRNA	686795	688957	.	-	.	ID=MALK_02089.t1;Parent=MALK_02089
contig006	maker	exon	686795	688957	.	-	.	ID=MALK_02089.t1.e1;Parent=MALK_02089.t1
contig006	AUGUSTUS	gene	689082	691220	0.83	+	.	ID=MALK_02090;prediction_source=augustus:contig006.g2346.t1
contig006	AUGUSTUS	CDS	689082	691220	0.83	+	0	ID=MALK_02090.t1.c1;Parent=MALK_02090.t1
contig006	AUGUSTUS	mRNA	689082	691220	0.83	+	.	ID=MALK_02090.t1;Parent=MALK_02090
contig006	AUGUSTUS	exon	689082	691220	0.83	+	.	ID=MALK_02090.t1.e1;Parent=MALK_02090.t1
contig006	AUGUSTUS	gene	691276	691779	1	+	.	ID=MALK_02091;prediction_source=braker_MRET:g2087.t1
contig006	AUGUSTUS	CDS	691276	691779	1	+	0	ID=MALK_02091.t1.c1;Parent=MALK_02091.t1
contig006	AUGUSTUS	mRNA	691276	691779	1	+	.	ID=MALK_02091.t1;Parent=MALK_02091
contig006	AUGUSTUS	exon	691276	691779	.	+	.	ID=MALK_02091.t1.e1;Parent=MALK_02091.t1
contig006	maker	gene	691781	695698	.	-	.	ID=MALK_02092;prediction_source=maker_MRET:augustus_masked-contig006-processed-gene-7.72-mRNA-1
contig006	maker	CDS	691781	695698	.	-	0	ID=MALK_02092.t1.c1;Parent=MALK_02092.t1
contig006	maker	mRNA	691781	695698	.	-	.	ID=MALK_02092.t1;Parent=MALK_02092
contig006	maker	exon	691781	695698	.	-	.	ID=MALK_02092.t1.e1;Parent=MALK_02092.t1
contig006	maker	gene	695824	696891	.	-	.	ID=MALK_02093;prediction_source=maker_MRET:augustus_masked-contig006-processed-gene-7.73-mRNA-1
contig006	maker	CDS	695824	696891	.	-	0	ID=MALK_02093.t1.c1;Parent=MALK_02093.t1
contig006	maker	mRNA	695824	696891	.	-	.	ID=MALK_02093.t1;Parent=MALK_02093
contig006	maker	exon	695824	696891	.	-	.	ID=MALK_02093.t1.e1;Parent=MALK_02093.t1
contig006	maker	gene	697382	699799	.	+	.	ID=MALK_02094;prediction_source=maker_MRET:augustus_masked-contig006-processed-gene-7.71-mRNA-1
contig006	maker	CDS	697382	699799	.	+	0	ID=MALK_02094.t1.c1;Parent=MALK_02094.t1
contig006	maker	mRNA	697382	699799	.	+	.	ID=MALK_02094.t1;Parent=MALK_02094
contig006	maker	exon	697382	699799	.	+	.	ID=MALK_02094.t1.e1;Parent=MALK_02094.t1
contig006	AUGUSTUS	gene	699814	700939	0.41	-	.	ID=MALK_02095;prediction_source=braker_MRET:g2091.t1
contig006	AUGUSTUS	CDS	700410	700939	0.41	-	0	ID=MALK_02095.t1.c2;Parent=MALK_02095.t1
contig006	AUGUSTUS	CDS	699814	700366	0.41	-	0	ID=MALK_02095.t1.c1;Parent=MALK_02095.t1
contig006	AUGUSTUS	mRNA	699814	700939	0.41	-	.	ID=MALK_02095.t1;Parent=MALK_02095
contig006	AUGUSTUS	exon	700410	700939	.	-	.	ID=MALK_02095.t1.e2;Parent=MALK_02095.t1
contig006	AUGUSTUS	exon	699814	700366	.	-	.	ID=MALK_02095.t1.e1;Parent=MALK_02095.t1
contig006	AUGUSTUS	gene	700962	702311	0.44	+	.	ID=MALK_02096;prediction_source=braker_MRET:g2092.t1
contig006	AUGUSTUS	CDS	700962	702311	0.44	+	0	ID=MALK_02096.t1.c1;Parent=MALK_02096.t1
contig006	AUGUSTUS	mRNA	700962	702311	0.44	+	.	ID=MALK_02096.t1;Parent=MALK_02096
contig006	AUGUSTUS	exon	700962	702311	.	+	.	ID=MALK_02096.t1.e1;Parent=MALK_02096.t1
contig006	AUGUSTUS	gene	702349	703428	0.55	+	.	ID=MALK_02097;prediction_source=augustus:contig006.g2356.t1
contig006	AUGUSTUS	CDS	702349	703428	0.55	+	0	ID=MALK_02097.t1.c1;Parent=MALK_02097.t1
contig006	AUGUSTUS	mRNA	702349	703428	0.55	+	.	ID=MALK_02097.t1;Parent=MALK_02097
contig006	AUGUSTUS	exon	702349	703428	0.55	+	.	ID=MALK_02097.t1.e1;Parent=MALK_02097.t1
contig006	AUGUSTUS	gene	704678	706108	1	+	.	ID=MALK_02098;prediction_source=augustus:contig006.g2357.t1
contig006	AUGUSTUS	CDS	704678	706108	1	+	0	ID=MALK_02098.t1.c1;Parent=MALK_02098.t1
contig006	AUGUSTUS	mRNA	704678	706108	1	+	.	ID=MALK_02098.t1;Parent=MALK_02098
contig006	AUGUSTUS	exon	704678	706108	1	+	.	ID=MALK_02098.t1.e1;Parent=MALK_02098.t1
contig006	AUGUSTUS	gene	706313	707887	0.84	+	.	ID=MALK_02099;prediction_source=braker_MRET:g2095.t1

contig006	AUGUSTUS	CDS	706313	707887	0.84	+	0	ID=MALK_02099.t1.c1;Parent=MALK_02099.t1
contig006	AUGUSTUS	mRNA	706313	707887	0.84	+	.	ID=MALK_02099.t1;Parent=MALK_02099
contig006	AUGUSTUS	exon	706313	707887	.	+	.	ID=MALK_02099.t1.e1;Parent=MALK_02099.t1
contig006	maker	gene	707890	708558	.	-	.	ID=MALK_02100;prediction_source=maker_MRET:augustus_masked-contig006-processed-gene-7.105-mRNA-1
contig006	maker	CDS	707890	708558	.	-	0	ID=MALK_02100.t1.c1;Parent=MALK_02100.t1
contig006	maker	mRNA	707890	708558	.	-	.	ID=MALK_02100.t1;Parent=MALK_02100
contig006	maker	exon	707890	708558	.	-	.	ID=MALK_02100.t1.e1;Parent=MALK_02100.t1
contig006	maker	gene	708658	710664	.	+	.	ID=MALK_02101;prediction_source=maker_MRET:augustus_masked-contig006-processed-gene-7.79-mRNA-1
contig006	maker	CDS	708658	710664	.	+	0	ID=MALK_02101.t1.c1;Parent=MALK_02101.t1
contig006	maker	mRNA	708658	710664	.	+	.	ID=MALK_02101.t1;Parent=MALK_02101
contig006	maker	exon	708658	710664	.	+	.	ID=MALK_02101.t1.e1;Parent=MALK_02101.t1
contig006	AUGUSTUS	gene	710844	711512	0.65	-	.	ID=MALK_02102;prediction_source=augustus:contig006.g2362.t1
contig006	AUGUSTUS	CDS	710844	711512	0.65	-	0	ID=MALK_02102.t1.c1;Parent=MALK_02102.t1
contig006	AUGUSTUS	mRNA	710844	711512	0.65	-	.	ID=MALK_02102.t1;Parent=MALK_02102
contig006	AUGUSTUS	exon	710844	711512	0.65	-	.	ID=MALK_02102.t1.e1;Parent=MALK_02102.t1
contig006	AUGUSTUS	gene	711655	712071	0.99	+	.	ID=MALK_02103;prediction_source=braker_MRET:g2099.t1
contig006	AUGUSTUS	CDS	711655	712071	0.99	+	0	ID=MALK_02103.t1.c1;Parent=MALK_02103.t1
contig006	AUGUSTUS	mRNA	711655	712071	0.99	+	.	ID=MALK_02103.t1;Parent=MALK_02103
contig006	AUGUSTUS	exon	711655	712071	.	+	.	ID=MALK_02103.t1.e1;Parent=MALK_02103.t1
contig006	AUGUSTUS	gene	712129	716877	0.37	-	.	ID=MALK_02104;prediction_source=augustus:contig006.g2363.t1
contig006	AUGUSTUS	CDS	712129	716877	0.37	-	0	ID=MALK_02104.t1.c1;Parent=MALK_02104.t1
contig006	AUGUSTUS	mRNA	712129	716877	0.37	-	.	ID=MALK_02104.t1;Parent=MALK_02104
contig006	AUGUSTUS	exon	712129	716877	0.37	-	.	ID=MALK_02104.t1.e1;Parent=MALK_02104.t1
contig006	maker	gene	717300	719321	.	-	.	ID=MALK_02105;prediction_source=maker_MRET:augustus_masked-contig006-processed-gene-7.108-mRNA-1
contig006	maker	CDS	717300	719321	.	-	0	ID=MALK_02105.t1.c1;Parent=MALK_02105.t1
contig006	maker	mRNA	717300	719321	.	-	.	ID=MALK_02105.t1;Parent=MALK_02105
contig006	maker	exon	717300	719321	.	-	.	ID=MALK_02105.t1.e1;Parent=MALK_02105.t1
contig006	AUGUSTUS	gene	719631	721064	0.6	+	.	ID=MALK_02106;prediction_source=augustus:contig006.g2368.t1
contig006	AUGUSTUS	CDS	719631	721064	0.6	+	0	ID=MALK_02106.t1.c1;Parent=MALK_02106.t1
contig006	AUGUSTUS	mRNA	719631	721064	0.6	+	.	ID=MALK_02106.t1;Parent=MALK_02106
contig006	AUGUSTUS	exon	719631	721064	0.6	+	.	ID=MALK_02106.t1.e1;Parent=MALK_02106.t1
contig006	AUGUSTUS	gene	721092	722660	0.88	-	.	ID=MALK_02107;prediction_source=braker_MRET:g2102.t2
contig006	AUGUSTUS	CDS	722621	722660	0.88	-	0	ID=MALK_02107.t1.c2;Parent=MALK_02107.t1
contig006	AUGUSTUS	CDS	721092	722587	0.88	-	0	ID=MALK_02107.t1.c1;Parent=MALK_02107.t1
contig006	AUGUSTUS	mRNA	721092	722660	0.88	-	.	ID=MALK_02107.t1;Parent=MALK_02107
contig006	AUGUSTUS	exon	722621	722660	.	-	.	ID=MALK_02107.t1.e2;Parent=MALK_02107.t1
contig006	AUGUSTUS	exon	721092	722587	.	-	.	ID=MALK_02107.t1.e1;Parent=MALK_02107.t1
contig006	AUGUSTUS	gene	722838	724352	0.97	+	.	ID=MALK_02108;prediction_source=augustus:contig006.g2370.t1
contig006	AUGUSTUS	CDS	722838	724352	0.97	+	0	ID=MALK_02108.t1.c1;Parent=MALK_02108.t1
contig006	AUGUSTUS	mRNA	722838	724352	0.97	+	.	ID=MALK_02108.t1;Parent=MALK_02108
contig006	AUGUSTUS	exon	722838	724352	0.97	+	.	ID=MALK_02108.t1.e1;Parent=MALK_02108.t1
contig006	AUGUSTUS	gene	724357	724811	0.96	-	.	ID=MALK_02109;prediction_source=braker_MRET:g2104.t1
contig006	AUGUSTUS	CDS	724438	724811	1	-	0	ID=MALK_02109.t1.c2;Parent=MALK_02109.t1

contig006	AUGUSTUS	CDS	724357	724399	1	-	0	ID=MALK_02109.t1.c1;Parent=MALK_02109.t1
contig006	AUGUSTUS	mRNA	724357	724811	0.96	-	.	ID=MALK_02109.t1;Parent=MALK_02109
contig006	AUGUSTUS	exon	724438	724811	.	-	.	ID=MALK_02109.t1.e2;Parent=MALK_02109.t1
contig006	AUGUSTUS	exon	724357	724399	.	-	.	ID=MALK_02109.t1.e1;Parent=MALK_02109.t1
contig006	AUGUSTUS	gene	724923	726474	0.74	+	.	ID=MALK_02110;prediction_source=braker_MRET:g2105.t1
contig006	AUGUSTUS	CDS	724923	725236	0.99	+	0	ID=MALK_02110.t1.c1;Parent=MALK_02110.t1
contig006	AUGUSTUS	CDS	725284	725648	0.99	+	0	ID=MALK_02110.t1.c2;Parent=MALK_02110.t1
contig006	AUGUSTUS	CDS	725723	726474	0.99	+	0	ID=MALK_02110.t1.c3;Parent=MALK_02110.t1
contig006	AUGUSTUS	mRNA	724923	726474	0.74	+	.	ID=MALK_02110.t1;Parent=MALK_02110
contig006	AUGUSTUS	exon	724923	725236	.	+	.	ID=MALK_02110.t1.e1;Parent=MALK_02110.t1
contig006	AUGUSTUS	exon	725284	725648	.	+	.	ID=MALK_02110.t1.e2;Parent=MALK_02110.t1
contig006	AUGUSTUS	exon	725723	726474	.	+	.	ID=MALK_02110.t1.e3;Parent=MALK_02110.t1
contig006	maker	gene	726490	731517	.	-	.	ID=MALK_02111;prediction_source=maker_MRET:augustus_masked-contig006-processed-gene-7.110-mRNA-1
contig006	maker	CDS	726490	731517	.	-	0	ID=MALK_02111.t1.c1;Parent=MALK_02111.t1
contig006	maker	mRNA	726490	731517	.	-	.	ID=MALK_02111.t1;Parent=MALK_02111
contig006	maker	exon	726490	731517	.	-	.	ID=MALK_02111.t1.e1;Parent=MALK_02111.t1
contig006	AUGUSTUS	gene	731736	732908	1	+	.	ID=MALK_02112;prediction_source=augustus:contig006.g2374.t1
contig006	AUGUSTUS	CDS	731736	732908	1	+	0	ID=MALK_02112.t1.c1;Parent=MALK_02112.t1
contig006	AUGUSTUS	mRNA	731736	732908	1	+	.	ID=MALK_02112.t1;Parent=MALK_02112
contig006	AUGUSTUS	exon	731736	732908	1	+	.	ID=MALK_02112.t1.e1;Parent=MALK_02112.t1
contig006	maker	gene	732911	737917	.	-	.	ID=MALK_02113;prediction_source=maker_MRET:augustus_masked-contig006-processed-gene-7.111-mRNA-1
contig006	maker	CDS	732911	737917	.	-	0	ID=MALK_02113.t1.c1;Parent=MALK_02113.t1
contig006	maker	mRNA	732911	737917	.	-	.	ID=MALK_02113.t1;Parent=MALK_02113
contig006	maker	exon	732911	737917	.	-	.	ID=MALK_02113.t1.e1;Parent=MALK_02113.t1
contig006	AUGUSTUS	gene	738013	740190	0.55	-	.	ID=MALK_02114;prediction_source=braker_MRET:g2109.t1
contig006	AUGUSTUS	CDS	738013	740190	0.55	-	0	ID=MALK_02114.t1.c1;Parent=MALK_02114.t1
contig006	AUGUSTUS	mRNA	738013	740190	0.55	-	.	ID=MALK_02114.t1;Parent=MALK_02114
contig006	AUGUSTUS	exon	738013	740190	.	-	.	ID=MALK_02114.t1.e1;Parent=MALK_02114.t1
contig006	maker	gene	740244	741479	.	-	.	ID=MALK_02115;prediction_source=maker_MRET:augustus_masked-contig006-processed-gene-7.113-mRNA-1
contig006	maker	CDS	740244	741479	.	-	0	ID=MALK_02115.t1.c1;Parent=MALK_02115.t1
contig006	maker	mRNA	740244	741479	.	-	.	ID=MALK_02115.t1;Parent=MALK_02115
contig006	maker	exon	740244	741479	.	-	.	ID=MALK_02115.t1.e1;Parent=MALK_02115.t1
contig006	AUGUSTUS	gene	741658	743151	0.67	+	.	ID=MALK_02116;prediction_source=augustus:contig006.g2379.t1
contig006	AUGUSTUS	CDS	741658	743151	0.67	+	0	ID=MALK_02116.t1.c1;Parent=MALK_02116.t1
contig006	AUGUSTUS	mRNA	741658	743151	0.67	+	.	ID=MALK_02116.t1;Parent=MALK_02116
contig006	AUGUSTUS	exon	741658	743151	0.67	+	.	ID=MALK_02116.t1.e1;Parent=MALK_02116.t1
contig006	AUGUSTUS	gene	743148	744962	1	-	.	ID=MALK_02117;prediction_source=braker_MRET:g2112.t1
contig006	AUGUSTUS	CDS	743148	744962	1	-	0	ID=MALK_02117.t1.c1;Parent=MALK_02117.t1
contig006	AUGUSTUS	mRNA	743148	744962	1	-	.	ID=MALK_02117.t1;Parent=MALK_02117
contig006	AUGUSTUS	exon	743148	744962	.	-	.	ID=MALK_02117.t1.e1;Parent=MALK_02117.t1
contig006	AUGUSTUS	gene	745153	747042	0.64	+	.	ID=MALK_02118;prediction_source=augustus:contig006.g2382.t1
contig006	AUGUSTUS	CDS	745153	747042	0.64	+	0	ID=MALK_02118.t1.c1;Parent=MALK_02118.t1
contig006	AUGUSTUS	mRNA	745153	747042	0.64	+	.	ID=MALK_02118.t1;Parent=MALK_02118

contig006	AUGUSTUS	exon	745153	747042	0.64	+	.	ID=MALK_02118.t1.e1;Parent=MALK_02118.t1
contig006	AUGUSTUS	gene	747056	749328	0.98	-	.	ID=MALK_02119;prediction_source=braker_MRET:g2114.t1
contig006	AUGUSTUS	CDS	748471	749328	0.98	-	0	ID=MALK_02119.t1.c2;Parent=MALK_02119.t1
contig006	AUGUSTUS	CDS	747056	748168	0.98	-	0	ID=MALK_02119.t1.c1;Parent=MALK_02119.t1
contig006	AUGUSTUS	mRNA	747056	749328	0.98	-	.	ID=MALK_02119.t1;Parent=MALK_02119
contig006	AUGUSTUS	exon	748471	749328	.	-	.	ID=MALK_02119.t1.e2;Parent=MALK_02119.t1
contig006	AUGUSTUS	exon	747056	748168	.	-	.	ID=MALK_02119.t1.e1;Parent=MALK_02119.t1
contig006	AUGUSTUS	gene	749464	750693	0.9	+	.	ID=MALK_02120;prediction_source=augustus:contig006.g2384.t1
contig006	AUGUSTUS	CDS	749464	750693	0.9	+	0	ID=MALK_02120.t1.c1;Parent=MALK_02120.t1
contig006	AUGUSTUS	mRNA	749464	750693	0.9	+	.	ID=MALK_02120.t1;Parent=MALK_02120
contig006	AUGUSTUS	exon	749464	750693	0.9	+	.	ID=MALK_02120.t1.e1;Parent=MALK_02120.t1
contig006	maker	gene	750794	752512	.	+	.	ID=MALK_02121;prediction_source=maker_MRET:augustus_masked-contig006-processed-gene-7.87-mRNA-1
contig006	maker	CDS	750794	752512	.	+	0	ID=MALK_02121.t1.c1;Parent=MALK_02121.t1
contig006	maker	mRNA	750794	752512	.	+	.	ID=MALK_02121.t1;Parent=MALK_02121
contig006	maker	exon	750794	752512	.	+	.	ID=MALK_02121.t1.e1;Parent=MALK_02121.t1
contig006	AUGUSTUS	gene	752509	753219	0.75	-	.	ID=MALK_02122;prediction_source=braker_MRET:g2117.t1
contig006	AUGUSTUS	CDS	752509	753219	0.75	-	0	ID=MALK_02122.t1.c1;Parent=MALK_02122.t1
contig006	AUGUSTUS	mRNA	752509	753219	0.75	-	.	ID=MALK_02122.t1;Parent=MALK_02122
contig006	AUGUSTUS	exon	752509	753219	.	-	.	ID=MALK_02122.t1.e1;Parent=MALK_02122.t1
contig006	maker	gene	753379	755095	.	+	.	ID=MALK_02123;prediction_source=maker_MRET:augustus_masked-contig006-processed-gene-7.88-mRNA-1
contig006	maker	CDS	753379	753762	.	+	0	ID=MALK_02123.t1.c1;Parent=MALK_02123.t1
contig006	maker	CDS	753899	755095	.	+	0	ID=MALK_02123.t1.c2;Parent=MALK_02123.t1
contig006	maker	mRNA	753379	755095	.	+	.	ID=MALK_02123.t1;Parent=MALK_02123
contig006	maker	exon	753379	753762	.	+	.	ID=MALK_02123.t1.e1;Parent=MALK_02123.t1
contig006	maker	exon	753899	755095	.	+	.	ID=MALK_02123.t1.e2;Parent=MALK_02123.t1
contig006	AUGUSTUS	gene	756122	759187	0.96	-	.	ID=MALK_02124;prediction_source=braker_MRET:g2120.t1
contig006	AUGUSTUS	CDS	756122	759187	0.96	-	0	ID=MALK_02124.t1.c1;Parent=MALK_02124.t1
contig006	AUGUSTUS	mRNA	756122	759187	0.96	-	.	ID=MALK_02124.t1;Parent=MALK_02124
contig006	AUGUSTUS	exon	756122	759187	.	-	.	ID=MALK_02124.t1.e1;Parent=MALK_02124.t1
contig006	AUGUSTUS	gene	759268	760215	0.51	+	.	ID=MALK_02125;prediction_source=braker_MRET:g2121.t1
contig006	AUGUSTUS	CDS	759268	760215	0.51	+	0	ID=MALK_02125.t1.c1;Parent=MALK_02125.t1
contig006	AUGUSTUS	mRNA	759268	760215	0.51	+	.	ID=MALK_02125.t1;Parent=MALK_02125
contig006	AUGUSTUS	exon	759268	760215	.	+	.	ID=MALK_02125.t1.e1;Parent=MALK_02125.t1
contig006	maker	gene	760216	762534	.	-	.	ID=MALK_02126;prediction_source=maker_MRET:augustus_masked-contig006-processed-gene-7.116-mRNA-1
contig006	maker	CDS	760216	762534	.	-	0	ID=MALK_02126.t1.c1;Parent=MALK_02126.t1
contig006	maker	mRNA	760216	762534	.	-	.	ID=MALK_02126.t1;Parent=MALK_02126
contig006	maker	exon	760216	762534	.	-	.	ID=MALK_02126.t1.e1;Parent=MALK_02126.t1
contig006	AUGUSTUS	gene	762590	764308	0.61	-	.	ID=MALK_02127;prediction_source=braker_MRET:g2123.t1
contig006	AUGUSTUS	CDS	762590	764308	0.61	-	0	ID=MALK_02127.t1.c1;Parent=MALK_02127.t1
contig006	AUGUSTUS	mRNA	762590	764308	0.61	-	.	ID=MALK_02127.t1;Parent=MALK_02127
contig006	AUGUSTUS	exon	762590	764308	.	-	.	ID=MALK_02127.t1.e1;Parent=MALK_02127.t1
contig006	maker	gene	764652	766316	.	+	.	ID=MALK_02128;prediction_source=maker_MRET:augustus_masked-contig006-processed-gene-7.89-mRNA-1
contig006	maker	CDS	764652	766316	.	+	0	ID=MALK_02128.t1.c1;Parent=MALK_02128.t1

contig006	maker	mRNA	764652	766316	.	+	.	ID=MALK_02128.t1;Parent=MALK_02128
contig006	maker	exon	764652	766316	.	+	.	ID=MALK_02128.t1.e1;Parent=MALK_02128.t1
contig006	maker	gene	766395	772433	.	+	.	ID=MALK_02129;prediction_source=maker_MRET:augustus_masked-contig006-processed-gene-7.90-mRNA-1
contig006	maker	CDS	766395	772433	.	+	0	ID=MALK_02129.t1.c1;Parent=MALK_02129.t1
contig006	maker	mRNA	766395	772433	.	+	.	ID=MALK_02129.t1;Parent=MALK_02129
contig006	maker	exon	766395	772433	.	+	.	ID=MALK_02129.t1.e1;Parent=MALK_02129.t1
contig006	AUGUSTUS	gene	772469	774136	0.99	+	.	ID=MALK_02130;prediction_source=braker_MRET:g2126.t1
contig006	AUGUSTUS	CDS	772469	774136	0.99	+	0	ID=MALK_02130.t1.c1;Parent=MALK_02130.t1
contig006	AUGUSTUS	mRNA	772469	774136	0.99	+	.	ID=MALK_02130.t1;Parent=MALK_02130
contig006	AUGUSTUS	exon	772469	774136	.	+	.	ID=MALK_02130.t1.e1;Parent=MALK_02130.t1
contig006	AUGUSTUS	gene	774142	775872	0.46	-	.	ID=MALK_02131;prediction_source=braker_MRET:g2127.t1
contig006	AUGUSTUS	CDS	774142	775872	0.46	-	0	ID=MALK_02131.t1.c1;Parent=MALK_02131.t1
contig006	AUGUSTUS	mRNA	774142	775872	0.46	-	.	ID=MALK_02131.t1;Parent=MALK_02131
contig006	AUGUSTUS	exon	774142	775872	.	-	.	ID=MALK_02131.t1.e1;Parent=MALK_02131.t1
contig006	maker	gene	775919	776431	.	+	.	ID=MALK_02132;prediction_source=maker_MRET:augustus_masked-contig006-processed-gene-7.92-mRNA-1
contig006	maker	CDS	775919	776431	.	+	0	ID=MALK_02132.t1.c1;Parent=MALK_02132.t1
contig006	maker	mRNA	775919	776431	.	+	.	ID=MALK_02132.t1;Parent=MALK_02132
contig006	maker	exon	775919	776431	.	+	.	ID=MALK_02132.t1.e1;Parent=MALK_02132.t1
contig006	AUGUSTUS	gene	776441	776926	0.75	-	.	ID=MALK_02133;prediction_source=braker_MRET:g2129.t1
contig006	AUGUSTUS	CDS	776441	776926	0.75	-	0	ID=MALK_02133.t1.c1;Parent=MALK_02133.t1
contig006	AUGUSTUS	mRNA	776441	776926	0.75	-	.	ID=MALK_02133.t1;Parent=MALK_02133
contig006	AUGUSTUS	exon	776441	776926	.	-	.	ID=MALK_02133.t1.e1;Parent=MALK_02133.t1
contig006	AUGUSTUS	gene	777437	778411	1	-	.	ID=MALK_02134;prediction_source=braker_MRET:g2130.t1
contig006	AUGUSTUS	CDS	778409	778411	1	-	0	ID=MALK_02134.t1.c3;Parent=MALK_02134.t1
contig006	AUGUSTUS	CDS	778162	778279	1	-	0	ID=MALK_02134.t1.c2;Parent=MALK_02134.t1
contig006	AUGUSTUS	CDS	777437	778131	1	-	0	ID=MALK_02134.t1.c1;Parent=MALK_02134.t1
contig006	AUGUSTUS	mRNA	777437	778411	1	-	.	ID=MALK_02134.t1;Parent=MALK_02134
contig006	AUGUSTUS	exon	778409	778411	.	-	.	ID=MALK_02134.t1.e3;Parent=MALK_02134.t1
contig006	AUGUSTUS	exon	778162	778279	.	-	.	ID=MALK_02134.t1.e2;Parent=MALK_02134.t1
contig006	AUGUSTUS	exon	777437	778131	.	-	.	ID=MALK_02134.t1.e1;Parent=MALK_02134.t1
contig006	AUGUSTUS	gene	778583	779800	0.72	+	.	ID=MALK_02135;prediction_source=augustus:contig006.g2396.t1
contig006	AUGUSTUS	CDS	778583	779800	0.72	+	0	ID=MALK_02135.t1.c1;Parent=MALK_02135.t1
contig006	AUGUSTUS	mRNA	778583	779800	0.72	+	.	ID=MALK_02135.t1;Parent=MALK_02135
contig006	AUGUSTUS	exon	778583	779800	0.72	+	.	ID=MALK_02135.t1.e1;Parent=MALK_02135.t1
contig006	AUGUSTUS	gene	779801	782953	1	-	.	ID=MALK_02136;prediction_source=augustus:contig006.g2397.t1
contig006	AUGUSTUS	CDS	779801	782953	1	-	0	ID=MALK_02136.t1.c1;Parent=MALK_02136.t1
contig006	AUGUSTUS	mRNA	779801	782953	1	-	.	ID=MALK_02136.t1;Parent=MALK_02136
contig006	AUGUSTUS	exon	779801	782953	1	-	.	ID=MALK_02136.t1.e1;Parent=MALK_02136.t1
contig006	maker	gene	783891	784880	.	-	.	ID=MALK_02137;prediction_source=maker_MRET:augustus_masked-contig006-processed-gene-7.121-mRNA-1
contig006	maker	CDS	783891	784880	.	-	0	ID=MALK_02137.t1.c1;Parent=MALK_02137.t1
contig006	maker	mRNA	783891	784880	.	-	.	ID=MALK_02137.t1;Parent=MALK_02137
contig006	maker	exon	783891	784880	.	-	.	ID=MALK_02137.t1.e1;Parent=MALK_02137.t1
contig006	maker	gene	784930	785778	.	-	.	ID=MALK_02138;prediction_source=maker_MRET:augustus_masked-contig006-processed-gene-7.122-mRNA-1

contig006	maker	CDS	784930	785778	.	-	0	ID=MALK_02138.t1.c1;Parent=MALK_02138.t1
contig006	maker	mRNA	784930	785778	.	-	.	ID=MALK_02138.t1;Parent=MALK_02138
contig006	maker	exon	784930	785778	.	-	.	ID=MALK_02138.t1.e1;Parent=MALK_02138.t1
contig006	AUGUSTUS	gene	786014	787858	0.99	+	.	ID=MALK_02139;prediction_source=braker_MRET:g2134.t1
contig006	AUGUSTUS	CDS	786014	787858	0.99	+	0	ID=MALK_02139.t1.c1;Parent=MALK_02139.t1
contig006	AUGUSTUS	mRNA	786014	787858	0.99	+	.	ID=MALK_02139.t1;Parent=MALK_02139
contig006	AUGUSTUS	exon	786014	787858	.	+	.	ID=MALK_02139.t1.e1;Parent=MALK_02139.t1
contig006	AUGUSTUS	gene	787886	792980	0.49	-	.	ID=MALK_02140;prediction_source=braker_MRET:g2135.t1
contig006	AUGUSTUS	CDS	792971	792980	0.55	-	0	ID=MALK_02140.t1.c3;Parent=MALK_02140.t1
contig006	AUGUSTUS	CDS	792569	792694	0.55	-	0	ID=MALK_02140.t1.c2;Parent=MALK_02140.t1
contig006	AUGUSTUS	CDS	787886	792540	0.55	-	0	ID=MALK_02140.t1.c1;Parent=MALK_02140.t1
contig006	AUGUSTUS	mRNA	787886	792980	0.49	-	.	ID=MALK_02140.t1;Parent=MALK_02140
contig006	AUGUSTUS	exon	792971	792980	.	-	.	ID=MALK_02140.t1.e3;Parent=MALK_02140.t1
contig006	AUGUSTUS	exon	792569	792694	.	-	.	ID=MALK_02140.t1.e2;Parent=MALK_02140.t1
contig006	AUGUSTUS	exon	787886	792540	.	-	.	ID=MALK_02140.t1.e1;Parent=MALK_02140.t1
contig006	AUGUSTUS	gene	793484	795659	0.33	-	.	ID=MALK_02141;prediction_source=braker_MRET:g2136.t1
contig006	AUGUSTUS	CDS	795644	795659	1	-	0	ID=MALK_02141.t1.c10;Parent=MALK_02141.t1
contig006	AUGUSTUS	CDS	795524	795594	1	-	0	ID=MALK_02141.t1.c9;Parent=MALK_02141.t1
contig006	AUGUSTUS	CDS	795438	795482	1	-	0	ID=MALK_02141.t1.c8;Parent=MALK_02141.t1
contig006	AUGUSTUS	CDS	795304	795390	1	-	0	ID=MALK_02141.t1.c7;Parent=MALK_02141.t1
contig006	AUGUSTUS	CDS	795207	795265	1	-	0	ID=MALK_02141.t1.c6;Parent=MALK_02141.t1
contig006	AUGUSTUS	CDS	795096	795173	1	-	0	ID=MALK_02141.t1.c5;Parent=MALK_02141.t1
contig006	AUGUSTUS	CDS	794557	795066	1	-	0	ID=MALK_02141.t1.c4;Parent=MALK_02141.t1
contig006	AUGUSTUS	CDS	794308	794485	1	-	0	ID=MALK_02141.t1.c3;Parent=MALK_02141.t1
contig006	AUGUSTUS	CDS	793915	794095	1	-	0	ID=MALK_02141.t1.c2;Parent=MALK_02141.t1
contig006	AUGUSTUS	CDS	793484	793884	1	-	0	ID=MALK_02141.t1.c1;Parent=MALK_02141.t1
contig006	AUGUSTUS	mRNA	793484	795659	0.33	-	.	ID=MALK_02141.t1;Parent=MALK_02141
contig006	AUGUSTUS	exon	795644	795659	.	-	.	ID=MALK_02141.t1.e10;Parent=MALK_02141.t1
contig006	AUGUSTUS	exon	795524	795594	.	-	.	ID=MALK_02141.t1.e9;Parent=MALK_02141.t1
contig006	AUGUSTUS	exon	795438	795482	.	-	.	ID=MALK_02141.t1.e8;Parent=MALK_02141.t1
contig006	AUGUSTUS	exon	795304	795390	.	-	.	ID=MALK_02141.t1.e7;Parent=MALK_02141.t1
contig006	AUGUSTUS	exon	795207	795265	.	-	.	ID=MALK_02141.t1.e6;Parent=MALK_02141.t1
contig006	AUGUSTUS	exon	795096	795173	.	-	.	ID=MALK_02141.t1.e5;Parent=MALK_02141.t1
contig006	AUGUSTUS	exon	794557	795066	.	-	.	ID=MALK_02141.t1.e4;Parent=MALK_02141.t1
contig006	AUGUSTUS	exon	794308	794485	.	-	.	ID=MALK_02141.t1.e3;Parent=MALK_02141.t1
contig006	AUGUSTUS	exon	793915	794095	.	-	.	ID=MALK_02141.t1.e2;Parent=MALK_02141.t1
contig006	AUGUSTUS	exon	793484	793884	.	-	.	ID=MALK_02141.t1.e1;Parent=MALK_02141.t1
contig006	AUGUSTUS	gene	796187	796873	0.31	-	.	ID=MALK_02142;prediction_source=braker_MRET:g2137.t1
contig006	AUGUSTUS	CDS	796683	796873	0.33	-	0	ID=MALK_02142.t1.c3;Parent=MALK_02142.t1
contig006	AUGUSTUS	CDS	796593	796641	0.33	-	0	ID=MALK_02142.t1.c2;Parent=MALK_02142.t1
contig006	AUGUSTUS	CDS	796187	796561	0.33	-	0	ID=MALK_02142.t1.c1;Parent=MALK_02142.t1
contig006	AUGUSTUS	mRNA	796187	796873	0.31	-	.	ID=MALK_02142.t1;Parent=MALK_02142
contig006	AUGUSTUS	exon	796683	796873	.	-	.	ID=MALK_02142.t1.e3;Parent=MALK_02142.t1

contig006	AUGUSTUS	exon	796593	796641	.	-	.	ID=MALK_02142.t1.e2;Parent=MALK_02142.t1
contig006	AUGUSTUS	exon	796187	796561	.	-	.	ID=MALK_02142.t1.e1;Parent=MALK_02142.t1
contig006	AUGUSTUS	gene	796893	797538	0.31	+	.	ID=MALK_02143;prediction_source=braker_MRET:g2138.t1
contig006	AUGUSTUS	CDS	796893	796902	0.69	+	0	ID=MALK_02143.t1.c1;Parent=MALK_02143.t1
contig006	AUGUSTUS	CDS	796940	797538	0.69	+	0	ID=MALK_02143.t1.c2;Parent=MALK_02143.t1
contig006	AUGUSTUS	mRNA	796893	797538	0.31	+	.	ID=MALK_02143.t1;Parent=MALK_02143
contig006	AUGUSTUS	exon	796893	796902	.	+	.	ID=MALK_02143.t1.e1;Parent=MALK_02143.t1
contig006	AUGUSTUS	exon	796940	797538	.	+	.	ID=MALK_02143.t1.e2;Parent=MALK_02143.t1
contig006	AUGUSTUS	gene	797551	800151	0.96	-	.	ID=MALK_02144;prediction_source=braker_MRET:g2139.t1
contig006	AUGUSTUS	CDS	797551	800151	0.96	-	0	ID=MALK_02144.t1.c1;Parent=MALK_02144.t1
contig006	AUGUSTUS	mRNA	797551	800151	0.96	-	.	ID=MALK_02144.t1;Parent=MALK_02144
contig006	AUGUSTUS	exon	797551	800151	.	-	.	ID=MALK_02144.t1.e1;Parent=MALK_02144.t1
contig006	AUGUSTUS	gene	800202	803138	0.98	-	.	ID=MALK_02145;prediction_source=braker_MRET:g2140.t1
contig006	AUGUSTUS	CDS	800202	803138	0.98	-	0	ID=MALK_02145.t1.c1;Parent=MALK_02145.t1
contig006	AUGUSTUS	mRNA	800202	803138	0.98	-	.	ID=MALK_02145.t1;Parent=MALK_02145
contig006	AUGUSTUS	exon	800202	803138	.	-	.	ID=MALK_02145.t1.e1;Parent=MALK_02145.t1
contig006	AUGUSTUS	gene	803194	804507	0.85	+	.	ID=MALK_02146;prediction_source=augustus:contig006.g2408.t1
contig006	AUGUSTUS	CDS	803194	804507	0.85	+	0	ID=MALK_02146.t1.c1;Parent=MALK_02146.t1
contig006	AUGUSTUS	mRNA	803194	804507	0.85	+	.	ID=MALK_02146.t1;Parent=MALK_02146
contig006	AUGUSTUS	exon	803194	804507	0.85	+	.	ID=MALK_02146.t1.e1;Parent=MALK_02146.t1
contig006	maker	gene	804861	807332	.	-	.	ID=MALK_02147;prediction_source=maker_MRET:augustus_masked-contig006-processed-gene-7.126-mRNA-1
contig006	maker	CDS	804861	807332	.	-	0	ID=MALK_02147.t1.c1;Parent=MALK_02147.t1
contig006	maker	mRNA	804861	807332	.	-	.	ID=MALK_02147.t1;Parent=MALK_02147
contig006	maker	exon	804861	807332	.	-	.	ID=MALK_02147.t1.e1;Parent=MALK_02147.t1
contig006	AUGUSTUS	gene	807967	810222	0.46	-	.	ID=MALK_02148;prediction_source=augustus:contig006.g2410.t1
contig006	AUGUSTUS	CDS	807967	810222	0.46	-	0	ID=MALK_02148.t1.c1;Parent=MALK_02148.t1
contig006	AUGUSTUS	mRNA	807967	810222	0.46	-	.	ID=MALK_02148.t1;Parent=MALK_02148
contig006	AUGUSTUS	exon	807967	810222	0.46	-	.	ID=MALK_02148.t1.e1;Parent=MALK_02148.t1
contig006	AUGUSTUS	gene	810610	814308	0.45	+	.	ID=MALK_02149;prediction_source=braker_MRET:g2143.t1
contig006	AUGUSTUS	CDS	810610	814308	0.45	+	0	ID=MALK_02149.t1.c1;Parent=MALK_02149.t1
contig006	AUGUSTUS	mRNA	810610	814308	0.45	+	.	ID=MALK_02149.t1;Parent=MALK_02149
contig006	AUGUSTUS	exon	810610	814308	.	+	.	ID=MALK_02149.t1.e1;Parent=MALK_02149.t1
contig006	AUGUSTUS	gene	814350	814823	0.44	+	.	ID=MALK_02150;prediction_source=braker_MRET:g2144.t1
contig006	AUGUSTUS	CDS	814350	814823	0.44	+	0	ID=MALK_02150.t1.c1;Parent=MALK_02150.t1
contig006	AUGUSTUS	mRNA	814350	814823	0.44	+	.	ID=MALK_02150.t1;Parent=MALK_02150
contig006	AUGUSTUS	exon	814350	814823	.	+	.	ID=MALK_02150.t1.e1;Parent=MALK_02150.t1
contig006	maker	gene	814836	815873	.	-	.	ID=MALK_02151;prediction_source=maker_MRET:augustus_masked-contig006-processed-gene-7.128-mRNA-1
contig006	maker	CDS	814836	815873	.	-	0	ID=MALK_02151.t1.c1;Parent=MALK_02151.t1
contig006	maker	mRNA	814836	815873	.	-	.	ID=MALK_02151.t1;Parent=MALK_02151
contig006	maker	exon	814836	815873	.	-	.	ID=MALK_02151.t1.e1;Parent=MALK_02151.t1
contig006	AUGUSTUS	gene	816411	817781	1	+	.	ID=MALK_02152;prediction_source=augustus:contig006.g2413.t1
contig006	AUGUSTUS	CDS	816411	817781	1	+	0	ID=MALK_02152.t1.c1;Parent=MALK_02152.t1
contig006	AUGUSTUS	mRNA	816411	817781	1	+	.	ID=MALK_02152.t1;Parent=MALK_02152

contig006	AUGUSTUS	exon	816411	817781	1	+	.	ID=MALK_02152.t1.e1;Parent=MALK_02152.t1
contig006	AUGUSTUS	gene	817806	821408	0.99	-	.	ID=MALK_02153;prediction_source=braker_MRET:g2147.t1
contig006	AUGUSTUS	CDS	817806	821408	0.99	-	0	ID=MALK_02153.t1.c1;Parent=MALK_02153.t1
contig006	AUGUSTUS	mRNA	817806	821408	0.99	-	.	ID=MALK_02153.t1;Parent=MALK_02153
contig006	AUGUSTUS	exon	817806	821408	.	-	.	ID=MALK_02153.t1.e1;Parent=MALK_02153.t1
contig006	AUGUSTUS	gene	821508	821729	0.77	+	.	ID=MALK_02154;prediction_source=braker_MRET:g2148.t1
contig006	AUGUSTUS	CDS	821508	821729	0.77	+	0	ID=MALK_02154.t1.c1;Parent=MALK_02154.t1
contig006	AUGUSTUS	mRNA	821508	821729	0.77	+	.	ID=MALK_02154.t1;Parent=MALK_02154
contig006	AUGUSTUS	exon	821508	821729	.	+	.	ID=MALK_02154.t1.e1;Parent=MALK_02154.t1
contig006	AUGUSTUS	gene	821738	822961	1	-	.	ID=MALK_02155;prediction_source=braker_MRET:g2149.t1
contig006	AUGUSTUS	CDS	821738	822961	1	-	0	ID=MALK_02155.t1.c1;Parent=MALK_02155.t1
contig006	AUGUSTUS	mRNA	821738	822961	1	-	.	ID=MALK_02155.t1;Parent=MALK_02155
contig006	AUGUSTUS	exon	821738	822961	.	-	.	ID=MALK_02155.t1.e1;Parent=MALK_02155.t1
contig006	AUGUSTUS	gene	823081	824541	0.98	+	.	ID=MALK_02156;prediction_source=braker_MRET:g2150.t1
contig006	AUGUSTUS	CDS	823081	824541	0.98	+	0	ID=MALK_02156.t1.c1;Parent=MALK_02156.t1
contig006	AUGUSTUS	mRNA	823081	824541	0.98	+	.	ID=MALK_02156.t1;Parent=MALK_02156
contig006	AUGUSTUS	exon	823081	824541	.	+	.	ID=MALK_02156.t1.e1;Parent=MALK_02156.t1
contig006	maker	gene	824556	825308	.	-	.	ID=MALK_02157;prediction_source=maker_MRET:augustus_masked-contig006-processed-gene-7.131-mRNA-1
contig006	maker	CDS	824556	825308	.	-	0	ID=MALK_02157.t1.c1;Parent=MALK_02157.t1
contig006	maker	mRNA	824556	825308	.	-	.	ID=MALK_02157.t1;Parent=MALK_02157
contig006	maker	exon	824556	825308	.	-	.	ID=MALK_02157.t1.e1;Parent=MALK_02157.t1
contig006	maker	gene	825422	827032	.	+	.	ID=MALK_02158;prediction_source=maker_MRET:augustus_masked-contig006-processed-gene-7.99-mRNA-1
contig006	maker	CDS	825422	827032	.	+	0	ID=MALK_02158.t1.c1;Parent=MALK_02158.t1
contig006	maker	mRNA	825422	827032	.	+	.	ID=MALK_02158.t1;Parent=MALK_02158
contig006	maker	exon	825422	827032	.	+	.	ID=MALK_02158.t1.e1;Parent=MALK_02158.t1
contig006	AUGUSTUS	gene	827029	827523	0.96	-	.	ID=MALK_02159;prediction_source=braker_MRET:g2153.t1
contig006	AUGUSTUS	CDS	827029	827523	0.96	-	0	ID=MALK_02159.t1.c1;Parent=MALK_02159.t1
contig006	AUGUSTUS	mRNA	827029	827523	0.96	-	.	ID=MALK_02159.t1;Parent=MALK_02159
contig006	AUGUSTUS	exon	827029	827523	.	-	.	ID=MALK_02159.t1.e1;Parent=MALK_02159.t1
contig006	maker	gene	827550	830318	.	-	.	ID=MALK_02160;prediction_source=maker_MRET:augustus_masked-contig006-processed-gene-7.132-mRNA-1
contig006	maker	CDS	827550	830318	.	-	0	ID=MALK_02160.t1.c1;Parent=MALK_02160.t1
contig006	maker	mRNA	827550	830318	.	-	.	ID=MALK_02160.t1;Parent=MALK_02160
contig006	maker	exon	827550	830318	.	-	.	ID=MALK_02160.t1.e1;Parent=MALK_02160.t1
contig006	AUGUSTUS	gene	830592	833144	0.37	+	.	ID=MALK_02161;prediction_source=braker_MRET:g2155.t1
contig006	AUGUSTUS	CDS	830592	833144	0.37	+	0	ID=MALK_02161.t1.c1;Parent=MALK_02161.t1
contig006	AUGUSTUS	mRNA	830592	833144	0.37	+	.	ID=MALK_02161.t1;Parent=MALK_02161
contig006	AUGUSTUS	exon	830592	833144	.	+	.	ID=MALK_02161.t1.e1;Parent=MALK_02161.t1
contig006	AUGUSTUS	gene	833148	833657	0.94	-	.	ID=MALK_02162;prediction_source=braker_MRET:g2156.t1
contig006	AUGUSTUS	CDS	833148	833657	0.94	-	0	ID=MALK_02162.t1.c1;Parent=MALK_02162.t1
contig006	AUGUSTUS	mRNA	833148	833657	0.94	-	.	ID=MALK_02162.t1;Parent=MALK_02162
contig006	AUGUSTUS	exon	833148	833657	.	-	.	ID=MALK_02162.t1.e1;Parent=MALK_02162.t1
contig006	maker	gene	833824	834744	.	+	.	ID=MALK_02163;prediction_source=maker_MRET:augustus_masked-contig006-processed-gene-7.101-mRNA-1
contig006	maker	CDS	833824	834744	.	+	0	ID=MALK_02163.t1.c1;Parent=MALK_02163.t1

contig006	maker	mRNA	833824	834744	.	+	.	ID=MALK_02163.t1;Parent=MALK_02163
contig006	maker	exon	833824	834744	.	+	.	ID=MALK_02163.t1.e1;Parent=MALK_02163.t1
contig006	maker	gene	834757	837576	.	-	.	ID=MALK_02164;prediction_source=maker_MRET:augustus_masked-contig006-processed-gene-7.134-mRNA-1
contig006	maker	CDS	834757	837576	.	-	0	ID=MALK_02164.t1.c1;Parent=MALK_02164.t1
contig006	maker	mRNA	834757	837576	.	-	.	ID=MALK_02164.t1;Parent=MALK_02164
contig006	maker	exon	834757	837576	.	-	.	ID=MALK_02164.t1.e1;Parent=MALK_02164.t1
contig006	AUGUSTUS	gene	837575	838855	0.68	+	.	ID=MALK_02165;prediction_source=braker_MRET:g2159.t1
contig006	AUGUSTUS	CDS	837575	838855	0.68	+	0	ID=MALK_02165.t1.c1;Parent=MALK_02165.t1
contig006	AUGUSTUS	mRNA	837575	838855	0.68	+	.	ID=MALK_02165.t1;Parent=MALK_02165
contig006	AUGUSTUS	exon	837575	838855	.	+	.	ID=MALK_02165.t1.e1;Parent=MALK_02165.t1
contig006	AUGUSTUS	gene	838856	839521	0.51	-	.	ID=MALK_02166;prediction_source=augustus:contig006.g2427.t1
contig006	AUGUSTUS	CDS	838856	839521	0.51	-	0	ID=MALK_02166.t1.c1;Parent=MALK_02166.t1
contig006	AUGUSTUS	mRNA	838856	839521	0.51	-	.	ID=MALK_02166.t1;Parent=MALK_02166
contig006	AUGUSTUS	exon	838856	839521	0.51	-	.	ID=MALK_02166.t1.e1;Parent=MALK_02166.t1
contig006	AUGUSTUS	gene	839630	840625	0.52	+	.	ID=MALK_02167;prediction_source=augustus:contig006.g2428.t1
contig006	AUGUSTUS	CDS	839630	840625	0.52	+	0	ID=MALK_02167.t1.c1;Parent=MALK_02167.t1
contig006	AUGUSTUS	mRNA	839630	840625	0.52	+	.	ID=MALK_02167.t1;Parent=MALK_02167
contig006	AUGUSTUS	exon	839630	840625	0.52	+	.	ID=MALK_02167.t1.e1;Parent=MALK_02167.t1
contig006	maker	gene	840828	843590	.	+	.	ID=MALK_02168;prediction_source=maker_MRET:augustus_masked-contig006-processed-gene-7.104-mRNA-1
contig006	maker	CDS	840828	843590	.	+	0	ID=MALK_02168.t1.c1;Parent=MALK_02168.t1
contig006	maker	mRNA	840828	843590	.	+	.	ID=MALK_02168.t1;Parent=MALK_02168
contig006	maker	exon	840828	843590	.	+	.	ID=MALK_02168.t1.e1;Parent=MALK_02168.t1
contig007	maker	gene	1	1263	.	+	.	ID=MALK_02169;prediction_source=maker_MRET:augustus_masked-contig007-processed-gene-0.2-mRNA-1
contig007	maker	CDS	1	1263	.	+	0	ID=MALK_02169.t1.c1;Parent=MALK_02169.t1
contig007	maker	mRNA	1	1263	.	+	.	ID=MALK_02169.t1;Parent=MALK_02169
contig007	maker	exon	1	1263	.	+	.	ID=MALK_02169.t1.e1;Parent=MALK_02169.t1
contig007	annotation	remark	1	4573	.	+	.	gff-version=3
contig007	maker	gene	1682	4573	.	+	.	ID=MALK_02170;prediction_source=maker_MRET:augustus_masked-contig007-processed-gene-0.3-mRNA-1
contig007	maker	CDS	1682	4573	.	+	0	ID=MALK_02170.t1.c1;Parent=MALK_02170.t1
contig007	maker	mRNA	1682	4573	.	+	.	ID=MALK_02170.t1;Parent=MALK_02170
contig007	maker	exon	1682	4573	.	+	.	ID=MALK_02170.t1.e1;Parent=MALK_02170.t1
contig008	maker	gene	1	1589	.	+	.	ID=MALK_02171;prediction_source=maker_MRET:augustus_masked-contig008-processed-gene-0.0-mRNA-1
contig008	maker	CDS	1	1410	.	+	0	ID=MALK_02171.t1.c1;Parent=MALK_02171.t1
contig008	maker	CDS	1542	1589	.	+	0	ID=MALK_02171.t1.c2;Parent=MALK_02171.t1
contig008	maker	mRNA	1	1589	.	+	.	ID=MALK_02171.t1;Parent=MALK_02171
contig008	maker	exon	1	1410	.	+	.	ID=MALK_02171.t1.e1;Parent=MALK_02171.t1
contig008	maker	exon	1542	1589	.	+	.	ID=MALK_02171.t1.e2;Parent=MALK_02171.t1
contig008	annotation	remark	1	65905	.	+	.	gff-version=3
contig008	AUGUSTUS	gene	1739	2405	0.85	-	.	ID=MALK_02172;prediction_source=braker_MRET:g2164.t1
contig008	AUGUSTUS	CDS	1806	2405	0.85	-	0	ID=MALK_02172.t1.c2;Parent=MALK_02172.t1
contig008	AUGUSTUS	CDS	1739	1777	0.85	-	0	ID=MALK_02172.t1.c1;Parent=MALK_02172.t1
contig008	AUGUSTUS	mRNA	1739	2405	0.85	-	.	ID=MALK_02172.t1;Parent=MALK_02172
contig008	AUGUSTUS	exon	1806	2405	.	-	.	ID=MALK_02172.t1.e2;Parent=MALK_02172.t1

contig008	AUGUSTUS	exon	1739	1777	.	-	.	ID=MALK_02172.t1.e1;Parent=MALK_02172.t1
contig008	AUGUSTUS	gene	2777	7093	0.33	+	.	ID=MALK_02173;prediction_source=augustus:contig008.g1463.t1
contig008	AUGUSTUS	CDS	2777	7093	0.33	+	0	ID=MALK_02173.t1.c1;Parent=MALK_02173.t1
contig008	AUGUSTUS	mRNA	2777	7093	0.33	+	.	ID=MALK_02173.t1;Parent=MALK_02173
contig008	AUGUSTUS	exon	2777	7093	0.33	+	.	ID=MALK_02173.t1.e1;Parent=MALK_02173.t1
contig008	AUGUSTUS	gene	7149	8936	0.97	-	.	ID=MALK_02174;prediction_source=augustus:contig008.g1465.t1
contig008	AUGUSTUS	CDS	7149	8936	0.97	-	0	ID=MALK_02174.t1.c1;Parent=MALK_02174.t1
contig008	AUGUSTUS	mRNA	7149	8936	0.97	-	.	ID=MALK_02174.t1;Parent=MALK_02174
contig008	AUGUSTUS	exon	7149	8936	0.97	-	.	ID=MALK_02174.t1.e1;Parent=MALK_02174.t1
contig008	AUGUSTUS	gene	9450	10358	0.49	+	.	ID=MALK_02175;prediction_source=augustus:contig008.g1467.t1
contig008	AUGUSTUS	CDS	9450	10358	0.49	+	0	ID=MALK_02175.t1.c1;Parent=MALK_02175.t1
contig008	AUGUSTUS	mRNA	9450	10358	0.49	+	.	ID=MALK_02175.t1;Parent=MALK_02175
contig008	AUGUSTUS	exon	9450	10358	0.49	+	.	ID=MALK_02175.t1.e1;Parent=MALK_02175.t1
contig008	maker	gene	10522	12462	.	+	.	ID=MALK_02176;prediction_source=maker_MRET:augustus_masked-contig008-processed-gene-0.3-mRNA-1
contig008	maker	CDS	10522	12462	.	+	0	ID=MALK_02176.t1.c1;Parent=MALK_02176.t1
contig008	maker	mRNA	10522	12462	.	+	.	ID=MALK_02176.t1;Parent=MALK_02176
contig008	maker	exon	10522	12462	.	+	.	ID=MALK_02176.t1.e1;Parent=MALK_02176.t1
contig008	maker	gene	12479	14596	.	-	.	ID=MALK_02177;prediction_source=maker_MRET:augustus_masked-contig008-processed-gene-0.19-mRNA-1
contig008	maker	CDS	12479	14596	.	-	0	ID=MALK_02177.t1.c1;Parent=MALK_02177.t1
contig008	maker	mRNA	12479	14596	.	-	.	ID=MALK_02177.t1;Parent=MALK_02177
contig008	maker	exon	12479	14596	.	-	.	ID=MALK_02177.t1.e1;Parent=MALK_02177.t1
contig008	AUGUSTUS	gene	15045	15872	0.98	+	.	ID=MALK_02178;prediction_source=augustus:contig008.g1471.t1
contig008	AUGUSTUS	CDS	15045	15872	0.98	+	0	ID=MALK_02178.t1.c1;Parent=MALK_02178.t1
contig008	AUGUSTUS	mRNA	15045	15872	0.98	+	.	ID=MALK_02178.t1;Parent=MALK_02178
contig008	AUGUSTUS	exon	15045	15872	0.98	+	.	ID=MALK_02178.t1.e1;Parent=MALK_02178.t1
contig008	maker	gene	15899	17560	.	-	.	ID=MALK_02179;prediction_source=maker_MRET:augustus_masked-contig008-processed-gene-0.20-mRNA-1
contig008	maker	CDS	15899	17560	.	-	0	ID=MALK_02179.t1.c1;Parent=MALK_02179.t1
contig008	maker	mRNA	15899	17560	.	-	.	ID=MALK_02179.t1;Parent=MALK_02179
contig008	maker	exon	15899	17560	.	-	.	ID=MALK_02179.t1.e1;Parent=MALK_02179.t1
contig008	maker	gene	17705	18868	.	+	.	ID=MALK_02180;prediction_source=maker_MRET:augustus_masked-contig008-processed-gene-0.5-mRNA-1
contig008	maker	CDS	17705	18868	.	+	0	ID=MALK_02180.t1.c1;Parent=MALK_02180.t1
contig008	maker	mRNA	17705	18868	.	+	.	ID=MALK_02180.t1;Parent=MALK_02180
contig008	maker	exon	17705	18868	.	+	.	ID=MALK_02180.t1.e1;Parent=MALK_02180.t1
contig008	maker	gene	18884	19927	.	-	.	ID=MALK_02181;prediction_source=maker_MRET:augustus_masked-contig008-processed-gene-0.21-mRNA-1
contig008	maker	CDS	18884	19927	.	-	0	ID=MALK_02181.t1.c1;Parent=MALK_02181.t1
contig008	maker	mRNA	18884	19927	.	-	.	ID=MALK_02181.t1;Parent=MALK_02181
contig008	maker	exon	18884	19927	.	-	.	ID=MALK_02181.t1.e1;Parent=MALK_02181.t1
contig008	maker	gene	20086	20703	.	+	.	ID=MALK_02182;prediction_source=maker_MRET:augustus_masked-contig008-processed-gene-0.6-mRNA-1
contig008	maker	CDS	20086	20703	.	+	0	ID=MALK_02182.t1.c1;Parent=MALK_02182.t1
contig008	maker	mRNA	20086	20703	.	+	.	ID=MALK_02182.t1;Parent=MALK_02182
contig008	maker	exon	20086	20703	.	+	.	ID=MALK_02182.t1.e1;Parent=MALK_02182.t1
contig008	AUGUSTUS	gene	21338	27928	0.97	+	.	ID=MALK_02183;prediction_source=augustus:contig008.g1479.t1
contig008	AUGUSTUS	CDS	21338	27928	0.97	+	0	ID=MALK_02183.t1.c1;Parent=MALK_02183.t1

contig008	AUGUSTUS	mRNA	21338	27928	0.97	+	.	ID=MALK_02183.t1;Parent=MALK_02183
contig008	AUGUSTUS	exon	21338	27928	0.97	+	.	ID=MALK_02183.t1.e1;Parent=MALK_02183.t1
contig008	maker	gene	27989	29017	.	+	.	ID=MALK_02184;prediction_source=maker_MRET:augustus_masked-contig008-processed-gene-0.8-mRNA-1
contig008	maker	CDS	27989	29017	.	+	0	ID=MALK_02184.t1.c1;Parent=MALK_02184.t1
contig008	maker	mRNA	27989	29017	.	+	.	ID=MALK_02184.t1;Parent=MALK_02184
contig008	maker	exon	27989	29017	.	+	.	ID=MALK_02184.t1.e1;Parent=MALK_02184.t1
contig008	AUGUSTUS	gene	29039	35779	0.36	-	.	ID=MALK_02185;prediction_source=augustus:contig008.g1482.t1
contig008	AUGUSTUS	CDS	29039	35779	0.36	-	0	ID=MALK_02185.t1.c1;Parent=MALK_02185.t1
contig008	AUGUSTUS	mRNA	29039	35779	0.36	-	.	ID=MALK_02185.t1;Parent=MALK_02185
contig008	AUGUSTUS	exon	29039	35779	0.36	-	.	ID=MALK_02185.t1.e1;Parent=MALK_02185.t1
contig008	AUGUSTUS	gene	36064	36792	0.43	-	.	ID=MALK_02186;prediction_source=augustus:contig008.g1484.t1
contig008	AUGUSTUS	CDS	36724	36792	0.55	-	0	ID=MALK_02186.t1.c2;Parent=MALK_02186.t1
contig008	AUGUSTUS	CDS	36064	36582	0.55	-	0	ID=MALK_02186.t1.c1;Parent=MALK_02186.t1
contig008	AUGUSTUS	mRNA	36064	36792	0.43	-	.	ID=MALK_02186.t1;Parent=MALK_02186
contig008	AUGUSTUS	exon	36724	36792	0.55	-	.	ID=MALK_02186.t1.e2;Parent=MALK_02186.t1
contig008	AUGUSTUS	exon	36064	36582	0.55	-	.	ID=MALK_02186.t1.e1;Parent=MALK_02186.t1
contig008	AUGUSTUS	gene	37241	37586	1	+	.	ID=MALK_02187;prediction_source=braker_MRET:g2178.t1
contig008	AUGUSTUS	CDS	37241	37291	1	+	0	ID=MALK_02187.t1.c1;Parent=MALK_02187.t1
contig008	AUGUSTUS	CDS	37332	37586	1	+	0	ID=MALK_02187.t1.c2;Parent=MALK_02187.t1
contig008	AUGUSTUS	mRNA	37241	37586	1	+	.	ID=MALK_02187.t1;Parent=MALK_02187
contig008	AUGUSTUS	exon	37241	37291	.	+	.	ID=MALK_02187.t1.e1;Parent=MALK_02187.t1
contig008	AUGUSTUS	exon	37332	37586	.	+	.	ID=MALK_02187.t1.e2;Parent=MALK_02187.t1
contig008	maker	gene	37688	38917	.	-	.	ID=MALK_02188;prediction_source=maker_MRET:augustus_masked-contig008-processed-gene-0.24-mRNA-1
contig008	maker	CDS	37688	38917	.	-	0	ID=MALK_02188.t1.c1;Parent=MALK_02188.t1
contig008	maker	mRNA	37688	38917	.	-	.	ID=MALK_02188.t1;Parent=MALK_02188
contig008	maker	exon	37688	38917	.	-	.	ID=MALK_02188.t1.e1;Parent=MALK_02188.t1
contig008	maker	gene	39277	40374	.	+	.	ID=MALK_02189;prediction_source=maker_MRET:augustus_masked-contig008-processed-gene-0.10-mRNA-1
contig008	maker	CDS	39277	40374	.	+	0	ID=MALK_02189.t1.c1;Parent=MALK_02189.t1
contig008	maker	mRNA	39277	40374	.	+	.	ID=MALK_02189.t1;Parent=MALK_02189
contig008	maker	exon	39277	40374	.	+	.	ID=MALK_02189.t1.e1;Parent=MALK_02189.t1
contig008	AUGUSTUS	gene	40644	41096	1	-	.	ID=MALK_02190;prediction_source=braker_MRET:g2181.t1
contig008	AUGUSTUS	CDS	40991	41096	1	-	0	ID=MALK_02190.t1.c2;Parent=MALK_02190.t1
contig008	AUGUSTUS	CDS	40644	40948	1	-	0	ID=MALK_02190.t1.c1;Parent=MALK_02190.t1
contig008	AUGUSTUS	mRNA	40644	41096	1	-	.	ID=MALK_02190.t1;Parent=MALK_02190
contig008	AUGUSTUS	exon	40991	41096	.	-	.	ID=MALK_02190.t1.e2;Parent=MALK_02190.t1
contig008	AUGUSTUS	exon	40644	40948	.	-	.	ID=MALK_02190.t1.e1;Parent=MALK_02190.t1
contig008	maker	gene	42055	43779	.	+	.	ID=MALK_02191;prediction_source=maker_MRET:augustus_masked-contig008-processed-gene-0.11-mRNA-1
contig008	maker	CDS	42055	43779	.	+	0	ID=MALK_02191.t1.c1;Parent=MALK_02191.t1
contig008	maker	mRNA	42055	43779	.	+	.	ID=MALK_02191.t1;Parent=MALK_02191
contig008	maker	exon	42055	43779	.	+	.	ID=MALK_02191.t1.e1;Parent=MALK_02191.t1
contig008	maker	gene	43792	45402	.	-	.	ID=MALK_02192;prediction_source=maker_MRET:augustus_masked-contig008-processed-gene-0.25-mRNA-1
contig008	maker	CDS	43792	45402	.	-	0	ID=MALK_02192.t1.c1;Parent=MALK_02192.t1
contig008	maker	mRNA	43792	45402	.	-	.	ID=MALK_02192.t1;Parent=MALK_02192

contig008	maker	exon	43792	45402	.	-	.	ID=MALK_02192.t1.e1;Parent=MALK_02192.t1
contig008	maker	gene	45526	46599	.	+	.	ID=MALK_02193;prediction_source=maker_MRET:augustus_masked-contig008-processed-gene-0.12-mRNA-1
contig008	maker	CDS	45526	46599	.	+	0	ID=MALK_02193.t1.c1;Parent=MALK_02193.t1
contig008	maker	mRNA	45526	46599	.	+	.	ID=MALK_02193.t1;Parent=MALK_02193
contig008	maker	exon	45526	46599	.	+	.	ID=MALK_02193.t1.e1;Parent=MALK_02193.t1
contig008	maker	gene	46629	48689	.	-	.	ID=MALK_02194;prediction_source=maker_MRET:augustus_masked-contig008-processed-gene-0.26-mRNA-1
contig008	maker	CDS	46629	48689	.	-	0	ID=MALK_02194.t1.c1;Parent=MALK_02194.t1
contig008	maker	mRNA	46629	48689	.	-	.	ID=MALK_02194.t1;Parent=MALK_02194
contig008	maker	exon	46629	48689	.	-	.	ID=MALK_02194.t1.e1;Parent=MALK_02194.t1
contig008	maker	gene	48809	49861	.	-	.	ID=MALK_02195;prediction_source=maker_MRET:augustus_masked-contig008-processed-gene-0.27-mRNA-1
contig008	maker	CDS	48809	49861	.	-	0	ID=MALK_02195.t1.c1;Parent=MALK_02195.t1
contig008	maker	mRNA	48809	49861	.	-	.	ID=MALK_02195.t1;Parent=MALK_02195
contig008	maker	exon	48809	49861	.	-	.	ID=MALK_02195.t1.e1;Parent=MALK_02195.t1
contig008	AUGUSTUS	gene	50098	50661	0.59	+	.	ID=MALK_02196;prediction_source=braker_MRET:g2187.t1
contig008	AUGUSTUS	CDS	50098	50661	0.59	+	0	ID=MALK_02196.t1.c1;Parent=MALK_02196.t1
contig008	AUGUSTUS	mRNA	50098	50661	0.59	+	.	ID=MALK_02196.t1;Parent=MALK_02196
contig008	AUGUSTUS	exon	50098	50661	.	+	.	ID=MALK_02196.t1.e1;Parent=MALK_02196.t1
contig008	AUGUSTUS	gene	50691	53282	0.68	-	.	ID=MALK_02197;prediction_source=augustus:contig008.g1493.t1
contig008	AUGUSTUS	CDS	50691	53282	0.68	-	0	ID=MALK_02197.t1.c1;Parent=MALK_02197.t1
contig008	AUGUSTUS	mRNA	50691	53282	0.68	-	.	ID=MALK_02197.t1;Parent=MALK_02197
contig008	AUGUSTUS	exon	50691	53282	0.68	-	.	ID=MALK_02197.t1.e1;Parent=MALK_02197.t1
contig008	maker	gene	53524	55305	.	-	.	ID=MALK_02198;prediction_source=maker_MRET:augustus_masked-contig008-processed-gene-0.29-mRNA-1
contig008	maker	CDS	53524	55305	.	-	0	ID=MALK_02198.t1.c1;Parent=MALK_02198.t1
contig008	maker	mRNA	53524	55305	.	-	.	ID=MALK_02198.t1;Parent=MALK_02198
contig008	maker	exon	53524	55305	.	-	.	ID=MALK_02198.t1.e1;Parent=MALK_02198.t1
contig008	AUGUSTUS	gene	55529	56878	0.57	+	.	ID=MALK_02199;prediction_source=augustus:contig008.g1496.t1
contig008	AUGUSTUS	CDS	55529	56878	0.57	+	0	ID=MALK_02199.t1.c1;Parent=MALK_02199.t1
contig008	AUGUSTUS	mRNA	55529	56878	0.57	+	.	ID=MALK_02199.t1;Parent=MALK_02199
contig008	AUGUSTUS	exon	55529	56878	0.57	+	.	ID=MALK_02199.t1.e1;Parent=MALK_02199.t1
contig008	AUGUSTUS	gene	56879	60106	0.97	-	.	ID=MALK_02200;prediction_source=augustus:contig008.g1497.t1
contig008	AUGUSTUS	CDS	56879	60106	0.97	-	0	ID=MALK_02200.t1.c1;Parent=MALK_02200.t1
contig008	AUGUSTUS	mRNA	56879	60106	0.97	-	.	ID=MALK_02200.t1;Parent=MALK_02200
contig008	AUGUSTUS	exon	56879	60106	0.97	-	.	ID=MALK_02200.t1.e1;Parent=MALK_02200.t1
contig008	AUGUSTUS	gene	60269	62116	0.7	-	.	ID=MALK_02201;prediction_source=augustus:contig008.g1498.t1
contig008	AUGUSTUS	CDS	60269	62116	0.7	-	0	ID=MALK_02201.t1.c1;Parent=MALK_02201.t1
contig008	AUGUSTUS	mRNA	60269	62116	0.7	-	.	ID=MALK_02201.t1;Parent=MALK_02201
contig008	AUGUSTUS	exon	60269	62116	0.7	-	.	ID=MALK_02201.t1.e1;Parent=MALK_02201.t1
contig008	AUGUSTUS	gene	62879	63514	0.95	+	.	ID=MALK_02202;prediction_source=braker_MRET:g2193.t1
contig008	AUGUSTUS	CDS	62879	63514	0.95	+	0	ID=MALK_02202.t1.c1;Parent=MALK_02202.t1
contig008	AUGUSTUS	mRNA	62879	63514	0.95	+	.	ID=MALK_02202.t1;Parent=MALK_02202
contig008	AUGUSTUS	exon	62879	63514	.	+	.	ID=MALK_02202.t1.e1;Parent=MALK_02202.t1
contig008	AUGUSTUS	gene	64096	64764	1	+	.	ID=MALK_02203;prediction_source=augustus:contig008.g1501.t1
contig008	AUGUSTUS	CDS	64096	64764	1	+	0	ID=MALK_02203.t1.c1;Parent=MALK_02203.t1

contig008	AUGUSTUS	mRNA	64096	64764	1	+	.	ID=MALK_02203.t1;Parent=MALK_02203
contig008	AUGUSTUS	exon	64096	64764	1	+	.	ID=MALK_02203.t1.e1;Parent=MALK_02203.t1
contig009	maker	gene	1	779	.	-	.	ID=MALK_02204;prediction_source=maker_MRET:augustus_masked-contig009-processed-gene-0.80-mRNA-1
contig009	maker	CDS	732	779	.	-	0	ID=MALK_02204.t1.c1;Parent=MALK_02204.t1
contig009	maker	CDS	1	360	.	-	0	ID=MALK_02204.t1.c2;Parent=MALK_02204.t1
contig009	maker	mRNA	1	779	.	-	.	ID=MALK_02204.t1;Parent=MALK_02204
contig009	maker	exon	732	779	.	-	.	ID=MALK_02204.t1.e1;Parent=MALK_02204.t1
contig009	maker	exon	1	360	.	-	.	ID=MALK_02204.t1.e2;Parent=MALK_02204.t1
contig009	annotation	remark	1	773937	.	+	.	gff-version=3
contig009	AUGUSTUS	gene	1934	4528	0.71	+	.	ID=MALK_02205;prediction_source=augustus:contig009.g2432.t1
contig009	AUGUSTUS	CDS	1934	4528	0.71	+	0	ID=MALK_02205.t1.c1;Parent=MALK_02205.t1
contig009	AUGUSTUS	mRNA	1934	4528	0.71	+	.	ID=MALK_02205.t1;Parent=MALK_02205
contig009	AUGUSTUS	exon	1934	4528	0.71	+	.	ID=MALK_02205.t1.e1;Parent=MALK_02205.t1
contig009	AUGUSTUS	gene	4959	6878	0.97	+	.	ID=MALK_02206;prediction_source=augustus:contig009.g2434.t1
contig009	AUGUSTUS	CDS	4959	6878	0.97	+	0	ID=MALK_02206.t1.c1;Parent=MALK_02206.t1
contig009	AUGUSTUS	mRNA	4959	6878	0.97	+	.	ID=MALK_02206.t1;Parent=MALK_02206
contig009	AUGUSTUS	exon	4959	6878	0.97	+	.	ID=MALK_02206.t1.e1;Parent=MALK_02206.t1
contig009	maker	gene	6940	7959	.	+	.	ID=MALK_02207;prediction_source=maker_MRET:augustus_masked-contig009-processed-gene-0.56-mRNA-1
contig009	maker	CDS	6940	7959	.	+	0	ID=MALK_02207.t1.c1;Parent=MALK_02207.t1
contig009	maker	mRNA	6940	7959	.	+	.	ID=MALK_02207.t1;Parent=MALK_02207
contig009	maker	exon	6940	7959	.	+	.	ID=MALK_02207.t1.e1;Parent=MALK_02207.t1
contig009	maker	gene	8038	8454	.	+	.	ID=MALK_02208;prediction_source=maker_MRET:augustus_masked-contig009-processed-gene-0.57-mRNA-1
contig009	maker	CDS	8038	8454	.	+	0	ID=MALK_02208.t1.c1;Parent=MALK_02208.t1
contig009	maker	mRNA	8038	8454	.	+	.	ID=MALK_02208.t1;Parent=MALK_02208
contig009	maker	exon	8038	8454	.	+	.	ID=MALK_02208.t1.e1;Parent=MALK_02208.t1
contig009	AUGUSTUS	gene	8548	9654	0.65	+	.	ID=MALK_02209;prediction_source=augustus:contig009.g2438.t1
contig009	AUGUSTUS	CDS	8548	9654	0.65	+	0	ID=MALK_02209.t1.c1;Parent=MALK_02209.t1
contig009	AUGUSTUS	mRNA	8548	9654	0.65	+	.	ID=MALK_02209.t1;Parent=MALK_02209
contig009	AUGUSTUS	exon	8548	9654	0.65	+	.	ID=MALK_02209.t1.e1;Parent=MALK_02209.t1
contig009	AUGUSTUS	gene	9724	10587	0.65	+	.	ID=MALK_02210;prediction_source=augustus:contig009.g2439.t1
contig009	AUGUSTUS	CDS	9724	10587	0.65	+	0	ID=MALK_02210.t1.c1;Parent=MALK_02210.t1
contig009	AUGUSTUS	mRNA	9724	10587	0.65	+	.	ID=MALK_02210.t1;Parent=MALK_02210
contig009	AUGUSTUS	exon	9724	10587	0.65	+	.	ID=MALK_02210.t1.e1;Parent=MALK_02210.t1
contig009	AUGUSTUS	gene	10605	11051	0.91	-	.	ID=MALK_02211;prediction_source=braker_MRET:g2409.t1
contig009	AUGUSTUS	CDS	10605	11051	0.91	-	0	ID=MALK_02211.t1.c1;Parent=MALK_02211.t1
contig009	AUGUSTUS	mRNA	10605	11051	0.91	-	.	ID=MALK_02211.t1;Parent=MALK_02211
contig009	AUGUSTUS	exon	10605	11051	.	-	.	ID=MALK_02211.t1.e1;Parent=MALK_02211.t1
contig009	AUGUSTUS	gene	11240	12584	0.37	+	.	ID=MALK_02212;prediction_source=braker_MRET:g2410.t1
contig009	AUGUSTUS	CDS	11240	12472	0.75	+	0	ID=MALK_02212.t1.c1;Parent=MALK_02212.t1
contig009	AUGUSTUS	CDS	12507	12584	0.75	+	0	ID=MALK_02212.t1.c2;Parent=MALK_02212.t1
contig009	AUGUSTUS	mRNA	11240	12584	0.37	+	.	ID=MALK_02212.t1;Parent=MALK_02212
contig009	AUGUSTUS	exon	11240	12472	.	+	.	ID=MALK_02212.t1.e1;Parent=MALK_02212.t1
contig009	AUGUSTUS	exon	12507	12584	.	+	.	ID=MALK_02212.t1.e2;Parent=MALK_02212.t1

contig009	AUGUSTUS	gene	12593	13405	0.97	-	.	ID=MALK_02213;prediction_source=augustus:contig009.g2441.t1
contig009	AUGUSTUS	CDS	12593	13405	0.97	-	0	ID=MALK_02213.t1.c1;Parent=MALK_02213.t1
contig009	AUGUSTUS	mRNA	12593	13405	0.97	-	.	ID=MALK_02213.t1;Parent=MALK_02213
contig009	AUGUSTUS	exon	12593	13405	0.97	-	.	ID=MALK_02213.t1.e1;Parent=MALK_02213.t1
contig009	maker	gene	13465	14214	.	-	.	ID=MALK_02214;prediction_source=maker_MRET:augustus_masked-contig009-processed-gene-0.81-mRNA-1
contig009	maker	CDS	13465	14214	.	-	0	ID=MALK_02214.t1.c1;Parent=MALK_02214.t1
contig009	maker	mRNA	13465	14214	.	-	.	ID=MALK_02214.t1;Parent=MALK_02214
contig009	maker	exon	13465	14214	.	-	.	ID=MALK_02214.t1.e1;Parent=MALK_02214.t1
contig009	maker	gene	14368	15936	.	-	.	ID=MALK_02215;prediction_source=maker_MRET:augustus_masked-contig009-processed-gene-0.82-mRNA-1
contig009	maker	CDS	14368	15936	.	-	0	ID=MALK_02215.t1.c1;Parent=MALK_02215.t1
contig009	maker	mRNA	14368	15936	.	-	.	ID=MALK_02215.t1;Parent=MALK_02215
contig009	maker	exon	14368	15936	.	-	.	ID=MALK_02215.t1.e1;Parent=MALK_02215.t1
contig009	AUGUSTUS	gene	16057	16590	0.53	+	.	ID=MALK_02216;prediction_source=braker_MRET:g2414.t1
contig009	AUGUSTUS	CDS	16057	16148	1	+	0	ID=MALK_02216.t1.c1;Parent=MALK_02216.t1
contig009	AUGUSTUS	CDS	16225	16451	1	+	0	ID=MALK_02216.t1.c2;Parent=MALK_02216.t1
contig009	AUGUSTUS	CDS	16481	16590	1	+	0	ID=MALK_02216.t1.c3;Parent=MALK_02216.t1
contig009	AUGUSTUS	mRNA	16057	16590	0.53	+	.	ID=MALK_02216.t1;Parent=MALK_02216
contig009	AUGUSTUS	exon	16057	16148	.	+	.	ID=MALK_02216.t1.e1;Parent=MALK_02216.t1
contig009	AUGUSTUS	exon	16225	16451	.	+	.	ID=MALK_02216.t1.e2;Parent=MALK_02216.t1
contig009	AUGUSTUS	exon	16481	16590	.	+	.	ID=MALK_02216.t1.e3;Parent=MALK_02216.t1
contig009	AUGUSTUS	gene	16648	17095	1	-	.	ID=MALK_02217;prediction_source=braker_MRET:g2415.t1
contig009	AUGUSTUS	CDS	16983	17095	1	-	0	ID=MALK_02217.t1.c2;Parent=MALK_02217.t1
contig009	AUGUSTUS	CDS	16648	16942	1	-	0	ID=MALK_02217.t1.c1;Parent=MALK_02217.t1
contig009	AUGUSTUS	mRNA	16648	17095	1	-	.	ID=MALK_02217.t1;Parent=MALK_02217
contig009	AUGUSTUS	exon	16983	17095	.	-	.	ID=MALK_02217.t1.e2;Parent=MALK_02217.t1
contig009	AUGUSTUS	exon	16648	16942	.	-	.	ID=MALK_02217.t1.e1;Parent=MALK_02217.t1
contig009	maker	gene	17392	20100	.	+	.	ID=MALK_02218;prediction_source=maker_MRET:augustus_masked-contig009-processed-gene-0.61-mRNA-1
contig009	maker	CDS	17392	20100	.	+	0	ID=MALK_02218.t1.c1;Parent=MALK_02218.t1
contig009	maker	mRNA	17392	20100	.	+	.	ID=MALK_02218.t1;Parent=MALK_02218
contig009	maker	exon	17392	20100	.	+	.	ID=MALK_02218.t1.e1;Parent=MALK_02218.t1
contig009	AUGUSTUS	gene	20153	20516	0.52	-	.	ID=MALK_02219;prediction_source=braker_MRET:g2417.t1
contig009	AUGUSTUS	CDS	20259	20516	0.52	-	0	ID=MALK_02219.t1.c2;Parent=MALK_02219.t1
contig009	AUGUSTUS	CDS	20153	20224	0.52	-	0	ID=MALK_02219.t1.c1;Parent=MALK_02219.t1
contig009	AUGUSTUS	mRNA	20153	20516	0.52	-	.	ID=MALK_02219.t1;Parent=MALK_02219
contig009	AUGUSTUS	exon	20259	20516	.	-	.	ID=MALK_02219.t1.e2;Parent=MALK_02219.t1
contig009	AUGUSTUS	exon	20153	20224	.	-	.	ID=MALK_02219.t1.e1;Parent=MALK_02219.t1
contig009	AUGUSTUS	gene	20625	23838	0.76	+	.	ID=MALK_02220;prediction_source=braker_MRET:g2418.t1
contig009	AUGUSTUS	CDS	20625	22963	0.76	+	0	ID=MALK_02220.t1.c1;Parent=MALK_02220.t1
contig009	AUGUSTUS	CDS	23010	23838	0.76	+	0	ID=MALK_02220.t1.c2;Parent=MALK_02220.t1
contig009	AUGUSTUS	mRNA	20625	23838	0.76	+	.	ID=MALK_02220.t1;Parent=MALK_02220
contig009	AUGUSTUS	exon	20625	22963	.	+	.	ID=MALK_02220.t1.e1;Parent=MALK_02220.t1
contig009	AUGUSTUS	exon	23010	23838	.	+	.	ID=MALK_02220.t1.e2;Parent=MALK_02220.t1
contig009	AUGUSTUS	gene	23859	24671	0.99	+	.	ID=MALK_02221;prediction_source=braker_MRET:g2419.t1

contig009	AUGUSTUS	CDS	23859	24671	0.99	+	0	ID=MALK_02221.t1.c1;Parent=MALK_02221.t1
contig009	AUGUSTUS	mRNA	23859	24671	0.99	+	.	ID=MALK_02221.t1;Parent=MALK_02221
contig009	AUGUSTUS	exon	23859	24671	.	+	.	ID=MALK_02221.t1.e1;Parent=MALK_02221.t1
contig009	AUGUSTUS	gene	24683	25873	0.95	-	.	ID=MALK_02222;prediction_source=augustus:contig009.g2449.t1
contig009	AUGUSTUS	CDS	24683	25873	0.95	-	0	ID=MALK_02222.t1.c1;Parent=MALK_02222.t1
contig009	AUGUSTUS	mRNA	24683	25873	0.95	-	.	ID=MALK_02222.t1;Parent=MALK_02222
contig009	AUGUSTUS	exon	24683	25873	0.95	-	.	ID=MALK_02222.t1.e1;Parent=MALK_02222.t1
contig009	AUGUSTUS	gene	25968	26730	0.85	+	.	ID=MALK_02223;prediction_source=braker_MRET:g2421.t1
contig009	AUGUSTUS	CDS	25968	25976	0.85	+	0	ID=MALK_02223.t1.c1;Parent=MALK_02223.t1
contig009	AUGUSTUS	CDS	26017	26730	0.85	+	0	ID=MALK_02223.t1.c2;Parent=MALK_02223.t1
contig009	AUGUSTUS	mRNA	25968	26730	0.85	+	.	ID=MALK_02223.t1;Parent=MALK_02223
contig009	AUGUSTUS	exon	25968	25976	.	+	.	ID=MALK_02223.t1.e1;Parent=MALK_02223.t1
contig009	AUGUSTUS	exon	26017	26730	.	+	.	ID=MALK_02223.t1.e2;Parent=MALK_02223.t1
contig009	AUGUSTUS	gene	26794	28128	0.72	+	.	ID=MALK_02224;prediction_source=braker_MRET:g2422.t1
contig009	AUGUSTUS	CDS	26794	28128	0.72	+	0	ID=MALK_02224.t1.c1;Parent=MALK_02224.t1
contig009	AUGUSTUS	mRNA	26794	28128	0.72	+	.	ID=MALK_02224.t1;Parent=MALK_02224
contig009	AUGUSTUS	exon	26794	28128	.	+	.	ID=MALK_02224.t1.e1;Parent=MALK_02224.t1
contig009	AUGUSTUS	gene	28123	29985	1	-	.	ID=MALK_02225;prediction_source=augustus:contig009.g2452.t1
contig009	AUGUSTUS	CDS	28123	29985	1	-	0	ID=MALK_02225.t1.c1;Parent=MALK_02225.t1
contig009	AUGUSTUS	mRNA	28123	29985	1	-	.	ID=MALK_02225.t1;Parent=MALK_02225
contig009	AUGUSTUS	exon	28123	29985	1	-	.	ID=MALK_02225.t1.e1;Parent=MALK_02225.t1
contig009	maker	gene	30031	33330	.	-	.	ID=MALK_02226;prediction_source=maker_MRET:augustus_masked-contig009-processed-gene-0.86-mRNA-1
contig009	maker	CDS	30031	33330	.	-	0	ID=MALK_02226.t1.c1;Parent=MALK_02226.t1
contig009	maker	mRNA	30031	33330	.	-	.	ID=MALK_02226.t1;Parent=MALK_02226
contig009	maker	exon	30031	33330	.	-	.	ID=MALK_02226.t1.e1;Parent=MALK_02226.t1
contig009	maker	gene	33459	34862	.	+	.	ID=MALK_02227;prediction_source=maker_MRET:augustus_masked-contig009-processed-gene-0.65-mRNA-1
contig009	maker	CDS	33459	34862	.	+	0	ID=MALK_02227.t1.c1;Parent=MALK_02227.t1
contig009	maker	mRNA	33459	34862	.	+	.	ID=MALK_02227.t1;Parent=MALK_02227
contig009	maker	exon	33459	34862	.	+	.	ID=MALK_02227.t1.e1;Parent=MALK_02227.t1
contig009	AUGUSTUS	gene	35084	35537	0.98	+	.	ID=MALK_02228;prediction_source=braker_MRET:g2426.t1
contig009	AUGUSTUS	CDS	35084	35135	1	+	0	ID=MALK_02228.t1.c1;Parent=MALK_02228.t1
contig009	AUGUSTUS	CDS	35167	35180	1	+	0	ID=MALK_02228.t1.c2;Parent=MALK_02228.t1
contig009	AUGUSTUS	CDS	35220	35537	1	+	0	ID=MALK_02228.t1.c3;Parent=MALK_02228.t1
contig009	AUGUSTUS	mRNA	35084	35537	0.98	+	.	ID=MALK_02228.t1;Parent=MALK_02228
contig009	AUGUSTUS	exon	35084	35135	.	+	.	ID=MALK_02228.t1.e1;Parent=MALK_02228.t1
contig009	AUGUSTUS	exon	35167	35180	.	+	.	ID=MALK_02228.t1.e2;Parent=MALK_02228.t1
contig009	AUGUSTUS	exon	35220	35537	.	+	.	ID=MALK_02228.t1.e3;Parent=MALK_02228.t1
contig009	AUGUSTUS	gene	35625	36492	0.78	-	.	ID=MALK_02229;prediction_source=braker_MRET:g2427.t1
contig009	AUGUSTUS	CDS	36472	36492	0.97	-	0	ID=MALK_02229.t1.c4;Parent=MALK_02229.t1
contig009	AUGUSTUS	CDS	36329	36337	0.97	-	0	ID=MALK_02229.t1.c3;Parent=MALK_02229.t1
contig009	AUGUSTUS	CDS	35718	36291	0.97	-	0	ID=MALK_02229.t1.c2;Parent=MALK_02229.t1
contig009	AUGUSTUS	CDS	35625	35680	0.97	-	0	ID=MALK_02229.t1.c1;Parent=MALK_02229.t1
contig009	AUGUSTUS	mRNA	35625	36492	0.78	-	.	ID=MALK_02229.t1;Parent=MALK_02229

contig009	AUGUSTUS	exon	36472	36492	.	-	.	ID=MALK_02229.t1.e4;Parent=MALK_02229.t1
contig009	AUGUSTUS	exon	36329	36337	.	-	.	ID=MALK_02229.t1.e3;Parent=MALK_02229.t1
contig009	AUGUSTUS	exon	35718	36291	.	-	.	ID=MALK_02229.t1.e2;Parent=MALK_02229.t1
contig009	AUGUSTUS	exon	35625	35680	.	-	.	ID=MALK_02229.t1.e1;Parent=MALK_02229.t1
contig009	AUGUSTUS	gene	36445	37341	0.51	+	.	ID=MALK_02230;prediction_source=augustus:contig009.g2458.t1
contig009	AUGUSTUS	CDS	36445	37341	0.51	+	0	ID=MALK_02230.t1.c1;Parent=MALK_02230.t1
contig009	AUGUSTUS	mRNA	36445	37341	0.51	+	.	ID=MALK_02230.t1;Parent=MALK_02230
contig009	AUGUSTUS	exon	36445	37341	0.51	+	.	ID=MALK_02230.t1.e1;Parent=MALK_02230.t1
contig009	AUGUSTUS	gene	37511	39994	0.88	+	.	ID=MALK_02231;prediction_source=augustus:contig009.g2459.t1
contig009	AUGUSTUS	CDS	37511	39994	0.88	+	0	ID=MALK_02231.t1.c1;Parent=MALK_02231.t1
contig009	AUGUSTUS	mRNA	37511	39994	0.88	+	.	ID=MALK_02231.t1;Parent=MALK_02231
contig009	AUGUSTUS	exon	37511	39994	0.88	+	.	ID=MALK_02231.t1.e1;Parent=MALK_02231.t1
contig009	AUGUSTUS	gene	40414	41388	0.62	+	.	ID=MALK_02232;prediction_source=augustus:contig009.g2461.t1
contig009	AUGUSTUS	CDS	40414	41388	0.62	+	0	ID=MALK_02232.t1.c1;Parent=MALK_02232.t1
contig009	AUGUSTUS	mRNA	40414	41388	0.62	+	.	ID=MALK_02232.t1;Parent=MALK_02232
contig009	AUGUSTUS	exon	40414	41388	0.62	+	.	ID=MALK_02232.t1.e1;Parent=MALK_02232.t1
contig009	AUGUSTUS	gene	41352	42548	1	-	.	ID=MALK_02233;prediction_source=braker_MRET:g2431.t1
contig009	AUGUSTUS	CDS	41352	42548	1	-	0	ID=MALK_02233.t1.c1;Parent=MALK_02233.t1
contig009	AUGUSTUS	mRNA	41352	42548	1	-	.	ID=MALK_02233.t1;Parent=MALK_02233
contig009	AUGUSTUS	exon	41352	42548	.	-	.	ID=MALK_02233.t1.e1;Parent=MALK_02233.t1
contig009	AUGUSTUS	gene	42724	44859	0.71	+	.	ID=MALK_02234;prediction_source=augustus:contig009.g2462.t1
contig009	AUGUSTUS	CDS	42724	44859	0.71	+	0	ID=MALK_02234.t1.c1;Parent=MALK_02234.t1
contig009	AUGUSTUS	mRNA	42724	44859	0.71	+	.	ID=MALK_02234.t1;Parent=MALK_02234
contig009	AUGUSTUS	exon	42724	44859	0.71	+	.	ID=MALK_02234.t1.e1;Parent=MALK_02234.t1
contig009	maker	gene	45257	48517	.	-	.	ID=MALK_02235;prediction_source=maker_MRET:augustus_masked-contig009-processed-gene-0.88-mRNA-1
contig009	maker	CDS	46631	48517	.	-	0	ID=MALK_02235.t1.c1;Parent=MALK_02235.t1
contig009	maker	CDS	45257	46552	.	-	0	ID=MALK_02235.t1.c2;Parent=MALK_02235.t1
contig009	maker	mRNA	45257	48517	.	-	.	ID=MALK_02235.t1;Parent=MALK_02235
contig009	maker	exon	46631	48517	.	-	.	ID=MALK_02235.t1.e1;Parent=MALK_02235.t1
contig009	maker	exon	45257	46552	.	-	.	ID=MALK_02235.t1.e2;Parent=MALK_02235.t1
contig009	maker	gene	48951	50234	.	+	.	ID=MALK_02236;prediction_source=maker_MRET:augustus_masked-contig009-processed-gene-0.70-mRNA-1
contig009	maker	CDS	48951	50234	.	+	0	ID=MALK_02236.t1.c1;Parent=MALK_02236.t1
contig009	maker	mRNA	48951	50234	.	+	.	ID=MALK_02236.t1;Parent=MALK_02236
contig009	maker	exon	48951	50234	.	+	.	ID=MALK_02236.t1.e1;Parent=MALK_02236.t1
contig009	AUGUSTUS	gene	50350	50889	0.58	-	.	ID=MALK_02237;prediction_source=braker_MRET:g2435.t1
contig009	AUGUSTUS	CDS	50350	50889	0.58	-	0	ID=MALK_02237.t1.c1;Parent=MALK_02237.t1
contig009	AUGUSTUS	mRNA	50350	50889	0.58	-	.	ID=MALK_02237.t1;Parent=MALK_02237
contig009	AUGUSTUS	exon	50350	50889	.	-	.	ID=MALK_02237.t1.e1;Parent=MALK_02237.t1
contig009	AUGUSTUS	gene	51005	51374	0.87	+	.	ID=MALK_02238;prediction_source=braker_MRET:g2436.t1
contig009	AUGUSTUS	CDS	51005	51049	0.99	+	0	ID=MALK_02238.t1.c1;Parent=MALK_02238.t1
contig009	AUGUSTUS	CDS	51084	51170	0.99	+	0	ID=MALK_02238.t1.c2;Parent=MALK_02238.t1
contig009	AUGUSTUS	CDS	51198	51374	0.99	+	0	ID=MALK_02238.t1.c3;Parent=MALK_02238.t1
contig009	AUGUSTUS	mRNA	51005	51374	0.87	+	.	ID=MALK_02238.t1;Parent=MALK_02238

contig009	AUGUSTUS	exon	51005	51049	.	+	.	ID=MALK_02238.t1.e1;Parent=MALK_02238.t1
contig009	AUGUSTUS	exon	51084	51170	.	+	.	ID=MALK_02238.t1.e2;Parent=MALK_02238.t1
contig009	AUGUSTUS	exon	51198	51374	.	+	.	ID=MALK_02238.t1.e3;Parent=MALK_02238.t1
contig009	AUGUSTUS	gene	51390	52976	0.69	-	.	ID=MALK_02239;prediction_source=augustus:contig009.g2466.t1
contig009	AUGUSTUS	CDS	51390	52976	0.69	-	0	ID=MALK_02239.t1.c1;Parent=MALK_02239.t1
contig009	AUGUSTUS	mRNA	51390	52976	0.69	-	.	ID=MALK_02239.t1;Parent=MALK_02239
contig009	AUGUSTUS	exon	51390	52976	0.69	-	.	ID=MALK_02239.t1.e1;Parent=MALK_02239.t1
contig009	maker	gene	53117	55816	.	-	.	ID=MALK_02240;prediction_source=maker_MRET:augustus_masked-contig009-processed-gene-0.90-mRNA-1
contig009	maker	CDS	53117	55816	.	-	0	ID=MALK_02240.t1.c1;Parent=MALK_02240.t1
contig009	maker	mRNA	53117	55816	.	-	.	ID=MALK_02240.t1;Parent=MALK_02240
contig009	maker	exon	53117	55816	.	-	.	ID=MALK_02240.t1.e1;Parent=MALK_02240.t1
contig009	AUGUSTUS	gene	55861	56828	0.63	+	.	ID=MALK_02241;prediction_source=braker_MRET:g2439.t1
contig009	AUGUSTUS	CDS	55861	56741	0.98	+	0	ID=MALK_02241.t1.c1;Parent=MALK_02241.t1
contig009	AUGUSTUS	CDS	56783	56828	0.98	+	0	ID=MALK_02241.t1.c2;Parent=MALK_02241.t1
contig009	AUGUSTUS	mRNA	55861	56828	0.63	+	.	ID=MALK_02241.t1;Parent=MALK_02241
contig009	AUGUSTUS	exon	55861	56741	.	+	.	ID=MALK_02241.t1.e1;Parent=MALK_02241.t1
contig009	AUGUSTUS	exon	56783	56828	.	+	.	ID=MALK_02241.t1.e2;Parent=MALK_02241.t1
contig009	AUGUSTUS	gene	56881	57747	0.85	-	.	ID=MALK_02242;prediction_source=augustus:contig009.g2469.t1
contig009	AUGUSTUS	CDS	56881	57747	0.85	-	0	ID=MALK_02242.t1.c1;Parent=MALK_02242.t1
contig009	AUGUSTUS	mRNA	56881	57747	0.85	-	.	ID=MALK_02242.t1;Parent=MALK_02242
contig009	AUGUSTUS	exon	56881	57747	0.85	-	.	ID=MALK_02242.t1.e1;Parent=MALK_02242.t1
contig009	AUGUSTUS	gene	57830	59461	0.62	-	.	ID=MALK_02243;prediction_source=braker_MRET:g2441.t1
contig009	AUGUSTUS	CDS	59314	59461	0.81	-	0	ID=MALK_02243.t1.c2;Parent=MALK_02243.t1
contig009	AUGUSTUS	CDS	57830	59169	0.81	-	0	ID=MALK_02243.t1.c1;Parent=MALK_02243.t1
contig009	AUGUSTUS	mRNA	57830	59461	0.62	-	.	ID=MALK_02243.t1;Parent=MALK_02243
contig009	AUGUSTUS	exon	59314	59461	.	-	.	ID=MALK_02243.t1.e2;Parent=MALK_02243.t1
contig009	AUGUSTUS	exon	57830	59169	.	-	.	ID=MALK_02243.t1.e1;Parent=MALK_02243.t1
contig009	AUGUSTUS	gene	59560	60687	0.38	+	.	ID=MALK_02244;prediction_source=augustus:contig009.g2471.t1
contig009	AUGUSTUS	CDS	59560	60687	0.38	+	0	ID=MALK_02244.t1.c1;Parent=MALK_02244.t1
contig009	AUGUSTUS	mRNA	59560	60687	0.38	+	.	ID=MALK_02244.t1;Parent=MALK_02244
contig009	AUGUSTUS	exon	59560	60687	0.38	+	.	ID=MALK_02244.t1.e1;Parent=MALK_02244.t1
contig009	AUGUSTUS	gene	60688	62883	1	-	.	ID=MALK_02245;prediction_source=augustus:contig009.g2472.t1
contig009	AUGUSTUS	CDS	60688	62883	1	-	0	ID=MALK_02245.t1.c1;Parent=MALK_02245.t1
contig009	AUGUSTUS	mRNA	60688	62883	1	-	.	ID=MALK_02245.t1;Parent=MALK_02245
contig009	AUGUSTUS	exon	60688	62883	1	-	.	ID=MALK_02245.t1.e1;Parent=MALK_02245.t1
contig009	AUGUSTUS	gene	63008	63478	0.97	+	.	ID=MALK_02246;prediction_source=braker_MRET:g2444.t1
contig009	AUGUSTUS	CDS	63008	63219	1	+	0	ID=MALK_02246.t1.c1;Parent=MALK_02246.t1
contig009	AUGUSTUS	CDS	63286	63478	1	+	0	ID=MALK_02246.t1.c2;Parent=MALK_02246.t1
contig009	AUGUSTUS	mRNA	63008	63478	0.97	+	.	ID=MALK_02246.t1;Parent=MALK_02246
contig009	AUGUSTUS	exon	63008	63219	.	+	.	ID=MALK_02246.t1.e1;Parent=MALK_02246.t1
contig009	AUGUSTUS	exon	63286	63478	.	+	.	ID=MALK_02246.t1.e2;Parent=MALK_02246.t1
contig009	maker	gene	63695	66622	.	+	.	ID=MALK_02247;prediction_source=maker_MRET:augustus_masked-contig009-processed-gene-0.72-mRNA-1
contig009	maker	CDS	63695	66622	.	+	0	ID=MALK_02247.t1.c1;Parent=MALK_02247.t1

contig009	maker	mRNA	63695	66622	.	+	.	ID=MALK_02247.t1;Parent=MALK_02247
contig009	maker	exon	63695	66622	.	+	.	ID=MALK_02247.t1.e1;Parent=MALK_02247.t1
contig009	maker	gene	67033	67497	.	+	.	ID=MALK_02248;prediction_source=maker_MRET:augustus_masked-contig009-processed-gene-0.73-mRNA-1
contig009	maker	CDS	67033	67497	.	+	0	ID=MALK_02248.t1.c1;Parent=MALK_02248.t1
contig009	maker	mRNA	67033	67497	.	+	.	ID=MALK_02248.t1;Parent=MALK_02248
contig009	maker	exon	67033	67497	.	+	.	ID=MALK_02248.t1.e1;Parent=MALK_02248.t1
contig009	maker	gene	67588	67836	.	-	.	ID=MALK_02249;prediction_source=maker_MRET:augustus_masked-contig009-processed-gene-0.94-mRNA-1
contig009	maker	CDS	67588	67836	.	-	0	ID=MALK_02249.t1.c1;Parent=MALK_02249.t1
contig009	maker	mRNA	67588	67836	.	-	.	ID=MALK_02249.t1;Parent=MALK_02249
contig009	maker	exon	67588	67836	.	-	.	ID=MALK_02249.t1.e1;Parent=MALK_02249.t1
contig009	AUGUSTUS	gene	67932	69593	0.97	+	.	ID=MALK_02250;prediction_source=augustus:contig009.g2477.t1
contig009	AUGUSTUS	CDS	67932	69593	0.97	+	0	ID=MALK_02250.t1.c1;Parent=MALK_02250.t1
contig009	AUGUSTUS	mRNA	67932	69593	0.97	+	.	ID=MALK_02250.t1;Parent=MALK_02250
contig009	AUGUSTUS	exon	67932	69593	0.97	+	.	ID=MALK_02250.t1.e1;Parent=MALK_02250.t1
contig009	maker	gene	69604	71433	.	-	.	ID=MALK_02251;prediction_source=maker_MRET:augustus_masked-contig009-processed-gene-0.95-mRNA-1
contig009	maker	CDS	69604	71433	.	-	0	ID=MALK_02251.t1.c1;Parent=MALK_02251.t1
contig009	maker	mRNA	69604	71433	.	-	.	ID=MALK_02251.t1;Parent=MALK_02251
contig009	maker	exon	69604	71433	.	-	.	ID=MALK_02251.t1.e1;Parent=MALK_02251.t1
contig009	maker	gene	71510	79018	.	+	.	ID=MALK_02252;prediction_source=maker_MRET:augustus_masked-contig009-processed-gene-0.75-mRNA-1
contig009	maker	CDS	71510	79018	.	+	0	ID=MALK_02252.t1.c1;Parent=MALK_02252.t1
contig009	maker	mRNA	71510	79018	.	+	.	ID=MALK_02252.t1;Parent=MALK_02252
contig009	maker	exon	71510	79018	.	+	.	ID=MALK_02252.t1.e1;Parent=MALK_02252.t1
contig009	AUGUSTUS	gene	79208	79551	0.38	-	.	ID=MALK_02253;prediction_source=braker_MRET:g2450.t1
contig009	AUGUSTUS	CDS	79268	79551	0.38	-	0	ID=MALK_02253.t1.c2;Parent=MALK_02253.t1
contig009	AUGUSTUS	CDS	79208	79229	0.38	-	0	ID=MALK_02253.t1.c1;Parent=MALK_02253.t1
contig009	AUGUSTUS	mRNA	79208	79551	0.38	-	.	ID=MALK_02253.t1;Parent=MALK_02253
contig009	AUGUSTUS	exon	79268	79551	.	-	.	ID=MALK_02253.t1.e2;Parent=MALK_02253.t1
contig009	AUGUSTUS	exon	79208	79229	.	-	.	ID=MALK_02253.t1.e1;Parent=MALK_02253.t1
contig009	AUGUSTUS	gene	79715	82363	0.54	+	.	ID=MALK_02254;prediction_source=augustus:contig009.g2482.t1
contig009	AUGUSTUS	CDS	79715	82363	0.54	+	0	ID=MALK_02254.t1.c1;Parent=MALK_02254.t1
contig009	AUGUSTUS	mRNA	79715	82363	0.54	+	.	ID=MALK_02254.t1;Parent=MALK_02254
contig009	AUGUSTUS	exon	79715	82363	0.54	+	.	ID=MALK_02254.t1.e1;Parent=MALK_02254.t1
contig009	AUGUSTUS	gene	82430	85081	0.44	+	.	ID=MALK_02255;prediction_source=augustus:contig009.g2483.t1
contig009	AUGUSTUS	CDS	82430	85081	0.44	+	0	ID=MALK_02255.t1.c1;Parent=MALK_02255.t1
contig009	AUGUSTUS	mRNA	82430	85081	0.44	+	.	ID=MALK_02255.t1;Parent=MALK_02255
contig009	AUGUSTUS	exon	82430	85081	0.44	+	.	ID=MALK_02255.t1.e1;Parent=MALK_02255.t1
contig009	AUGUSTUS	gene	85186	87846	0.69	+	.	ID=MALK_02256;prediction_source=augustus:contig009.g2484.t1
contig009	AUGUSTUS	CDS	85186	87846	0.69	+	0	ID=MALK_02256.t1.c1;Parent=MALK_02256.t1
contig009	AUGUSTUS	mRNA	85186	87846	0.69	+	.	ID=MALK_02256.t1;Parent=MALK_02256
contig009	AUGUSTUS	exon	85186	87846	0.69	+	.	ID=MALK_02256.t1.e1;Parent=MALK_02256.t1
contig009	maker	gene	87897	90533	.	+	.	ID=MALK_02257;prediction_source=maker_MRET:augustus_masked-contig009-processed-gene-0.79-mRNA-1
contig009	maker	CDS	87897	90533	.	+	0	ID=MALK_02257.t1.c1;Parent=MALK_02257.t1
contig009	maker	mRNA	87897	90533	.	+	.	ID=MALK_02257.t1;Parent=MALK_02257

contig009	maker	exon	87897	90533	.	+	.	ID=MALK_02257.t1.e1;Parent=MALK_02257.t1
contig009	AUGUSTUS	gene	90534	91928	0.97	-	.	ID=MALK_02258;prediction_source=braker_MRET:g2455.t1
contig009	AUGUSTUS	CDS	90534	91928	0.97	-	0	ID=MALK_02258.t1.c1;Parent=MALK_02258.t1
contig009	AUGUSTUS	mRNA	90534	91928	0.97	-	.	ID=MALK_02258.t1;Parent=MALK_02258
contig009	AUGUSTUS	exon	90534	91928	.	-	.	ID=MALK_02258.t1.e1;Parent=MALK_02258.t1
contig009	AUGUSTUS	gene	91994	92557	0.99	+	.	ID=MALK_02259;prediction_source=braker_MRET:g2456.t1
contig009	AUGUSTUS	CDS	91994	92557	0.99	+	0	ID=MALK_02259.t1.c1;Parent=MALK_02259.t1
contig009	AUGUSTUS	mRNA	91994	92557	0.99	+	.	ID=MALK_02259.t1;Parent=MALK_02259
contig009	AUGUSTUS	exon	91994	92557	.	+	.	ID=MALK_02259.t1.e1;Parent=MALK_02259.t1
contig009	maker	gene	92711	93976	.	-	.	ID=MALK_02260;prediction_source=maker_MRET:augustus_masked-contig009-processed-gene-0.96-mRNA-1
contig009	maker	CDS	92711	93976	.	-	0	ID=MALK_02260.t1.c1;Parent=MALK_02260.t1
contig009	maker	mRNA	92711	93976	.	-	.	ID=MALK_02260.t1;Parent=MALK_02260
contig009	maker	exon	92711	93976	.	-	.	ID=MALK_02260.t1.e1;Parent=MALK_02260.t1
contig009	AUGUSTUS	gene	94049	95794	0.7	+	.	ID=MALK_02261;prediction_source=augustus:contig009.g2488.t1
contig009	AUGUSTUS	CDS	94049	95794	0.7	+	0	ID=MALK_02261.t1.c1;Parent=MALK_02261.t1
contig009	AUGUSTUS	mRNA	94049	95794	0.7	+	.	ID=MALK_02261.t1;Parent=MALK_02261
contig009	AUGUSTUS	exon	94049	95794	0.7	+	.	ID=MALK_02261.t1.e1;Parent=MALK_02261.t1
contig009	AUGUSTUS	gene	95823	98603	0.83	-	.	ID=MALK_02262;prediction_source=augustus:contig009.g2489.t1
contig009	AUGUSTUS	CDS	95823	98603	0.83	-	0	ID=MALK_02262.t1.c1;Parent=MALK_02262.t1
contig009	AUGUSTUS	mRNA	95823	98603	0.83	-	.	ID=MALK_02262.t1;Parent=MALK_02262
contig009	AUGUSTUS	exon	95823	98603	0.83	-	.	ID=MALK_02262.t1.e1;Parent=MALK_02262.t1
contig009	maker	gene	98749	100461	.	+	.	ID=MALK_02263;prediction_source=maker_MRET:augustus_masked-contig009-processed-gene-1.1-mRNA-1
contig009	maker	CDS	98749	100461	.	+	0	ID=MALK_02263.t1.c1;Parent=MALK_02263.t1
contig009	maker	mRNA	98749	100461	.	+	.	ID=MALK_02263.t1;Parent=MALK_02263
contig009	maker	exon	98749	100461	.	+	.	ID=MALK_02263.t1.e1;Parent=MALK_02263.t1
contig009	AUGUSTUS	gene	100432	101286	0.38	-	.	ID=MALK_02264;prediction_source=augustus:contig009.g2492.t1
contig009	AUGUSTUS	CDS	100432	101286	0.38	-	0	ID=MALK_02264.t1.c1;Parent=MALK_02264.t1
contig009	AUGUSTUS	mRNA	100432	101286	0.38	-	.	ID=MALK_02264.t1;Parent=MALK_02264
contig009	AUGUSTUS	exon	100432	101286	0.38	-	.	ID=MALK_02264.t1.e1;Parent=MALK_02264.t1
contig009	AUGUSTUS	gene	101453	101927	0.8	+	.	ID=MALK_02265;prediction_source=braker_MRET:g2462.t1
contig009	AUGUSTUS	CDS	101453	101528	1	+	0	ID=MALK_02265.t1.c1;Parent=MALK_02265.t1
contig009	AUGUSTUS	CDS	101560	101927	1	+	0	ID=MALK_02265.t1.c2;Parent=MALK_02265.t1
contig009	AUGUSTUS	mRNA	101453	101927	0.8	+	.	ID=MALK_02265.t1;Parent=MALK_02265
contig009	AUGUSTUS	exon	101453	101528	.	+	.	ID=MALK_02265.t1.e1;Parent=MALK_02265.t1
contig009	AUGUSTUS	exon	101560	101927	.	+	.	ID=MALK_02265.t1.e2;Parent=MALK_02265.t1
contig009	AUGUSTUS	gene	101997	103590	0.81	+	.	ID=MALK_02266;prediction_source=braker_MRET:g2463.t1
contig009	AUGUSTUS	CDS	101997	103513	0.82	+	0	ID=MALK_02266.t1.c1;Parent=MALK_02266.t1
contig009	AUGUSTUS	CDS	103563	103590	0.82	+	0	ID=MALK_02266.t1.c2;Parent=MALK_02266.t1
contig009	AUGUSTUS	mRNA	101997	103590	0.81	+	.	ID=MALK_02266.t1;Parent=MALK_02266
contig009	AUGUSTUS	exon	101997	103513	.	+	.	ID=MALK_02266.t1.e1;Parent=MALK_02266.t1
contig009	AUGUSTUS	exon	103563	103590	.	+	.	ID=MALK_02266.t1.e2;Parent=MALK_02266.t1
contig009	AUGUSTUS	gene	103595	104464	0.87	-	.	ID=MALK_02267;prediction_source=braker_MRET:g2464.t1
contig009	AUGUSTUS	CDS	103595	104464	0.87	-	0	ID=MALK_02267.t1.c1;Parent=MALK_02267.t1

contig009	AUGUSTUS	mRNA	103595	104464	0.87	-	.	ID=MALK_02267.t1;Parent=MALK_02267
contig009	AUGUSTUS	exon	103595	104464	.	-	.	ID=MALK_02267.t1.e1;Parent=MALK_02267.t1
contig009	AUGUSTUS	gene	104511	105431	0.9	+	.	ID=MALK_02268;prediction_source=braker_MRET:g2465.t1
contig009	AUGUSTUS	CDS	104511	105431	0.9	+	0	ID=MALK_02268.t1.c1;Parent=MALK_02268.t1
contig009	AUGUSTUS	mRNA	104511	105431	0.9	+	.	ID=MALK_02268.t1;Parent=MALK_02268
contig009	AUGUSTUS	exon	104511	105431	.	+	.	ID=MALK_02268.t1.e1;Parent=MALK_02268.t1
contig009	AUGUSTUS	gene	105433	109200	0.89	-	.	ID=MALK_02269;prediction_source=braker_MRET:g2466.t1
contig009	AUGUSTUS	CDS	105433	109200	0.89	-	0	ID=MALK_02269.t1.c1;Parent=MALK_02269.t1
contig009	AUGUSTUS	mRNA	105433	109200	0.89	-	.	ID=MALK_02269.t1;Parent=MALK_02269
contig009	AUGUSTUS	exon	105433	109200	.	-	.	ID=MALK_02269.t1.e1;Parent=MALK_02269.t1
contig009	AUGUSTUS	gene	109292	109912	0.93	+	.	ID=MALK_02270;prediction_source=braker_MRET:g2467.t1
contig009	AUGUSTUS	CDS	109292	109912	0.93	+	0	ID=MALK_02270.t1.c1;Parent=MALK_02270.t1
contig009	AUGUSTUS	mRNA	109292	109912	0.93	+	.	ID=MALK_02270.t1;Parent=MALK_02270
contig009	AUGUSTUS	exon	109292	109912	.	+	.	ID=MALK_02270.t1.e1;Parent=MALK_02270.t1
contig009	AUGUSTUS	gene	109965	110824	0.81	-	.	ID=MALK_02271;prediction_source=braker_MRET:g2468.t1
contig009	AUGUSTUS	CDS	110206	110824	0.81	-	0	ID=MALK_02271.t1.c2;Parent=MALK_02271.t1
contig009	AUGUSTUS	CDS	109965	110170	0.81	-	0	ID=MALK_02271.t1.c1;Parent=MALK_02271.t1
contig009	AUGUSTUS	mRNA	109965	110824	0.81	-	.	ID=MALK_02271.t1;Parent=MALK_02271
contig009	AUGUSTUS	exon	110206	110824	.	-	.	ID=MALK_02271.t1.e2;Parent=MALK_02271.t1
contig009	AUGUSTUS	exon	109965	110170	.	-	.	ID=MALK_02271.t1.e1;Parent=MALK_02271.t1
contig009	maker	gene	110896	111717	.	-	.	ID=MALK_02272;prediction_source=maker_MRET:augustus_masked-contig009-processed-gene-1.16-mRNA-1
contig009	maker	CDS	110896	111717	.	-	0	ID=MALK_02272.t1.c1;Parent=MALK_02272.t1
contig009	maker	mRNA	110896	111717	.	-	.	ID=MALK_02272.t1;Parent=MALK_02272
contig009	maker	exon	110896	111717	.	-	.	ID=MALK_02272.t1.e1;Parent=MALK_02272.t1
contig009	maker	gene	111823	115296	.	+	.	ID=MALK_02273;prediction_source=maker_MRET:augustus_masked-contig009-processed-gene-1.4-mRNA-1
contig009	maker	CDS	111823	115296	.	+	0	ID=MALK_02273.t1.c1;Parent=MALK_02273.t1
contig009	maker	mRNA	111823	115296	.	+	.	ID=MALK_02273.t1;Parent=MALK_02273
contig009	maker	exon	111823	115296	.	+	.	ID=MALK_02273.t1.e1;Parent=MALK_02273.t1
contig009	AUGUSTUS	gene	115293	115931	0.93	-	.	ID=MALK_02274;prediction_source=augustus:contig009.g2497.t1
contig009	AUGUSTUS	CDS	115293	115931	0.93	-	0	ID=MALK_02274.t1.c1;Parent=MALK_02274.t1
contig009	AUGUSTUS	mRNA	115293	115931	0.93	-	.	ID=MALK_02274.t1;Parent=MALK_02274
contig009	AUGUSTUS	exon	115293	115931	0.93	-	.	ID=MALK_02274.t1.e1;Parent=MALK_02274.t1
contig009	AUGUSTUS	gene	116004	117968	0.55	-	.	ID=MALK_02275;prediction_source=braker_MRET:g2472.t1
contig009	AUGUSTUS	CDS	116004	117968	0.55	-	0	ID=MALK_02275.t1.c1;Parent=MALK_02275.t1
contig009	AUGUSTUS	mRNA	116004	117968	0.55	-	.	ID=MALK_02275.t1;Parent=MALK_02275
contig009	AUGUSTUS	exon	116004	117968	.	-	.	ID=MALK_02275.t1.e1;Parent=MALK_02275.t1
contig009	AUGUSTUS	gene	118118	118978	0.86	-	.	ID=MALK_02276;prediction_source=augustus:contig009.g2499.t1
contig009	AUGUSTUS	CDS	118118	118978	0.86	-	0	ID=MALK_02276.t1.c1;Parent=MALK_02276.t1
contig009	AUGUSTUS	mRNA	118118	118978	0.86	-	.	ID=MALK_02276.t1;Parent=MALK_02276
contig009	AUGUSTUS	exon	118118	118978	0.86	-	.	ID=MALK_02276.t1.e1;Parent=MALK_02276.t1
contig009	AUGUSTUS	gene	119054	119608	0.27	+	.	ID=MALK_02277;prediction_source=braker_MRET:g2474.t1
contig009	AUGUSTUS	CDS	119054	119608	0.27	+	0	ID=MALK_02277.t1.c1;Parent=MALK_02277.t1
contig009	AUGUSTUS	mRNA	119054	119608	0.27	+	.	ID=MALK_02277.t1;Parent=MALK_02277

contig009	AUGUSTUS	exon	119054	119608	.	+	.	ID=MALK_02277.t1.e1;Parent=MALK_02277.t1
contig009	AUGUSTUS	gene	119898	122314	0.13	-	.	ID=MALK_02278;prediction_source=braker_MRET:g2475.t1
contig009	AUGUSTUS	CDS	121287	122314	0.48	-	0	ID=MALK_02278.t1.c3;Parent=MALK_02278.t1
contig009	AUGUSTUS	CDS	119977	121247	0.48	-	0	ID=MALK_02278.t1.c2;Parent=MALK_02278.t1
contig009	AUGUSTUS	CDS	119898	119929	0.48	-	0	ID=MALK_02278.t1.c1;Parent=MALK_02278.t1
contig009	AUGUSTUS	mRNA	119898	122314	0.13	-	.	ID=MALK_02278.t1;Parent=MALK_02278
contig009	AUGUSTUS	exon	121287	122314	.	-	.	ID=MALK_02278.t1.e3;Parent=MALK_02278.t1
contig009	AUGUSTUS	exon	119977	121247	.	-	.	ID=MALK_02278.t1.e2;Parent=MALK_02278.t1
contig009	AUGUSTUS	exon	119898	119929	.	-	.	ID=MALK_02278.t1.e1;Parent=MALK_02278.t1
contig009	AUGUSTUS	gene	122408	123424	1	-	.	ID=MALK_02279;prediction_source=braker_MRET:g2476.t1
contig009	AUGUSTUS	CDS	122408	123424	1	-	0	ID=MALK_02279.t1.c1;Parent=MALK_02279.t1
contig009	AUGUSTUS	mRNA	122408	123424	1	-	.	ID=MALK_02279.t1;Parent=MALK_02279
contig009	AUGUSTUS	exon	122408	123424	.	-	.	ID=MALK_02279.t1.e1;Parent=MALK_02279.t1
contig009	AUGUSTUS	gene	123589	124284	0.43	+	.	ID=MALK_02280;prediction_source=braker_MRET:g2477.t1
contig009	AUGUSTUS	CDS	123589	124284	0.43	+	0	ID=MALK_02280.t1.c1;Parent=MALK_02280.t1
contig009	AUGUSTUS	mRNA	123589	124284	0.43	+	.	ID=MALK_02280.t1;Parent=MALK_02280
contig009	AUGUSTUS	exon	123589	124284	.	+	.	ID=MALK_02280.t1.e1;Parent=MALK_02280.t1
contig009	AUGUSTUS	gene	124605	126440	0.98	-	.	ID=MALK_02281;prediction_source=augustus:contig009.g2503.t1
contig009	AUGUSTUS	CDS	124605	126440	0.98	-	0	ID=MALK_02281.t1.c1;Parent=MALK_02281.t1
contig009	AUGUSTUS	mRNA	124605	126440	0.98	-	.	ID=MALK_02281.t1;Parent=MALK_02281
contig009	AUGUSTUS	exon	124605	126440	0.98	-	.	ID=MALK_02281.t1.e1;Parent=MALK_02281.t1
contig009	maker	gene	126790	127749	.	-	.	ID=MALK_02282;prediction_source=maker_MRET:augustus_masked-contig009-processed-gene-1.21-mRNA-1
contig009	maker	CDS	126790	127749	.	-	0	ID=MALK_02282.t1.c1;Parent=MALK_02282.t1
contig009	maker	mRNA	126790	127749	.	-	.	ID=MALK_02282.t1;Parent=MALK_02282
contig009	maker	exon	126790	127749	.	-	.	ID=MALK_02282.t1.e1;Parent=MALK_02282.t1
contig009	AUGUSTUS	gene	128120	131509	0.88	-	.	ID=MALK_02283;prediction_source=braker_MRET:g2480.t1
contig009	AUGUSTUS	CDS	128120	131509	0.88	-	0	ID=MALK_02283.t1.c1;Parent=MALK_02283.t1
contig009	AUGUSTUS	mRNA	128120	131509	0.88	-	.	ID=MALK_02283.t1;Parent=MALK_02283
contig009	AUGUSTUS	exon	128120	131509	.	-	.	ID=MALK_02283.t1.e1;Parent=MALK_02283.t1
contig009	maker	gene	131743	132288	.	-	.	ID=MALK_02284;prediction_source=maker_MRET:augustus_masked-contig009-processed-gene-1.23-mRNA-1
contig009	maker	CDS	131743	132288	.	-	0	ID=MALK_02284.t1.c1;Parent=MALK_02284.t1
contig009	maker	mRNA	131743	132288	.	-	.	ID=MALK_02284.t1;Parent=MALK_02284
contig009	maker	exon	131743	132288	.	-	.	ID=MALK_02284.t1.e1;Parent=MALK_02284.t1
contig009	maker	gene	132473	133648	.	+	.	ID=MALK_02285;prediction_source=maker_MRET:augustus_masked-contig009-processed-gene-1.6-mRNA-1
contig009	maker	CDS	132473	133648	.	+	0	ID=MALK_02285.t1.c1;Parent=MALK_02285.t1
contig009	maker	mRNA	132473	133648	.	+	.	ID=MALK_02285.t1;Parent=MALK_02285
contig009	maker	exon	132473	133648	.	+	.	ID=MALK_02285.t1.e1;Parent=MALK_02285.t1
contig009	AUGUSTUS	gene	133645	135036	0.89	-	.	ID=MALK_02286;prediction_source=augustus:contig009.g2509.t1
contig009	AUGUSTUS	CDS	133645	135036	0.89	-	0	ID=MALK_02286.t1.c1;Parent=MALK_02286.t1
contig009	AUGUSTUS	mRNA	133645	135036	0.89	-	.	ID=MALK_02286.t1;Parent=MALK_02286
contig009	AUGUSTUS	exon	133645	135036	0.89	-	.	ID=MALK_02286.t1.e1;Parent=MALK_02286.t1
contig009	maker	gene	135086	136897	.	+	.	ID=MALK_02287;prediction_source=maker_MRET:augustus_masked-contig009-processed-gene-1.7-mRNA-1
contig009	maker	CDS	135086	136897	.	+	0	ID=MALK_02287.t1.c1;Parent=MALK_02287.t1

contig009	maker	mRNA	135086	136897	.	+	.	ID=MALK_02287.t1;Parent=MALK_02287
contig009	maker	exon	135086	136897	.	+	.	ID=MALK_02287.t1.e1;Parent=MALK_02287.t1
contig009	AUGUSTUS	gene	136902	137693	0.65	-	.	ID=MALK_02288;prediction_source=braker_MRET:g2485.t1
contig009	AUGUSTUS	CDS	136902	137693	0.65	-	0	ID=MALK_02288.t1.c1;Parent=MALK_02288.t1
contig009	AUGUSTUS	mRNA	136902	137693	0.65	-	.	ID=MALK_02288.t1;Parent=MALK_02288
contig009	AUGUSTUS	exon	136902	137693	.	-	.	ID=MALK_02288.t1.e1;Parent=MALK_02288.t1
contig009	maker	gene	137950	139788	.	+	.	ID=MALK_02289;prediction_source=maker_MRET:augustus_masked-contig009-processed-gene-1.8-mRNA-1
contig009	maker	CDS	137950	139788	.	+	0	ID=MALK_02289.t1.c1;Parent=MALK_02289.t1
contig009	maker	mRNA	137950	139788	.	+	.	ID=MALK_02289.t1;Parent=MALK_02289
contig009	maker	exon	137950	139788	.	+	.	ID=MALK_02289.t1.e1;Parent=MALK_02289.t1
contig009	maker	gene	139972	143532	.	+	.	ID=MALK_02290;prediction_source=maker_MRET:augustus_masked-contig009-processed-gene-1.9-mRNA-1
contig009	maker	CDS	139972	143532	.	+	0	ID=MALK_02290.t1.c1;Parent=MALK_02290.t1
contig009	maker	mRNA	139972	143532	.	+	.	ID=MALK_02290.t1;Parent=MALK_02290
contig009	maker	exon	139972	143532	.	+	.	ID=MALK_02290.t1.e1;Parent=MALK_02290.t1
contig009	AUGUSTUS	gene	143563	146709	0.34	-	.	ID=MALK_02291;prediction_source=braker_MRET:g2488.t1
contig009	AUGUSTUS	CDS	143563	146709	0.34	-	0	ID=MALK_02291.t1.c1;Parent=MALK_02291.t1
contig009	AUGUSTUS	mRNA	143563	146709	0.34	-	.	ID=MALK_02291.t1;Parent=MALK_02291
contig009	AUGUSTUS	exon	143563	146709	.	-	.	ID=MALK_02291.t1.e1;Parent=MALK_02291.t1
contig009	AUGUSTUS	gene	146791	147780	0.61	-	.	ID=MALK_02292;prediction_source=braker_MRET:g2489.t2
contig009	AUGUSTUS	CDS	146791	147780	0.61	-	0	ID=MALK_02292.t1.c1;Parent=MALK_02292.t1
contig009	AUGUSTUS	mRNA	146791	147780	0.61	-	.	ID=MALK_02292.t1;Parent=MALK_02292
contig009	AUGUSTUS	exon	146791	147780	.	-	.	ID=MALK_02292.t1.e1;Parent=MALK_02292.t1
contig009	AUGUSTUS	gene	148517	149821	0.74	-	.	ID=MALK_02293;prediction_source=braker_MRET:g2490.t1
contig009	AUGUSTUS	CDS	148517	149821	0.74	-	0	ID=MALK_02293.t1.c1;Parent=MALK_02293.t1
contig009	AUGUSTUS	mRNA	148517	149821	0.74	-	.	ID=MALK_02293.t1;Parent=MALK_02293
contig009	AUGUSTUS	exon	148517	149821	.	-	.	ID=MALK_02293.t1.e1;Parent=MALK_02293.t1
contig009	AUGUSTUS	gene	149874	150884	0.36	+	.	ID=MALK_02294;prediction_source=braker_MRET:g2491.t1
contig009	AUGUSTUS	CDS	149874	150884	0.36	+	0	ID=MALK_02294.t1.c1;Parent=MALK_02294.t1
contig009	AUGUSTUS	mRNA	149874	150884	0.36	+	.	ID=MALK_02294.t1;Parent=MALK_02294
contig009	AUGUSTUS	exon	149874	150884	.	+	.	ID=MALK_02294.t1.e1;Parent=MALK_02294.t1
contig009	maker	gene	150966	153635	.	+	.	ID=MALK_02295;prediction_source=maker_MRET:augustus_masked-contig009-processed-gene-1.10-mRNA-1
contig009	maker	CDS	150966	153635	.	+	0	ID=MALK_02295.t1.c1;Parent=MALK_02295.t1
contig009	maker	mRNA	150966	153635	.	+	.	ID=MALK_02295.t1;Parent=MALK_02295
contig009	maker	exon	150966	153635	.	+	.	ID=MALK_02295.t1.e1;Parent=MALK_02295.t1
contig009	AUGUSTUS	gene	153826	155376	0.64	+	.	ID=MALK_02296;prediction_source=braker_MRET:g2493.t1
contig009	AUGUSTUS	CDS	153826	155376	0.64	+	0	ID=MALK_02296.t1.c1;Parent=MALK_02296.t1
contig009	AUGUSTUS	mRNA	153826	155376	0.64	+	.	ID=MALK_02296.t1;Parent=MALK_02296
contig009	AUGUSTUS	exon	153826	155376	.	+	.	ID=MALK_02296.t1.e1;Parent=MALK_02296.t1
contig009	maker	gene	155417	156667	.	-	.	ID=MALK_02297;prediction_source=maker_MRET:augustus_masked-contig009-processed-gene-1.29-mRNA-1
contig009	maker	CDS	155417	156667	.	-	0	ID=MALK_02297.t1.c1;Parent=MALK_02297.t1
contig009	maker	mRNA	155417	156667	.	-	.	ID=MALK_02297.t1;Parent=MALK_02297
contig009	maker	exon	155417	156667	.	-	.	ID=MALK_02297.t1.e1;Parent=MALK_02297.t1
contig009	AUGUSTUS	gene	156736	157261	0.32	+	.	ID=MALK_02298;prediction_source=braker_MRET:g2495.t1

contig009	AUGUSTUS	CDS	156736	156792	0.99	+	0	ID=MALK_02298.t1.c1;Parent=MALK_02298.t1
contig009	AUGUSTUS	CDS	156822	156863	0.99	+	0	ID=MALK_02298.t1.c2;Parent=MALK_02298.t1
contig009	AUGUSTUS	CDS	156892	156985	0.99	+	0	ID=MALK_02298.t1.c3;Parent=MALK_02298.t1
contig009	AUGUSTUS	CDS	157016	157047	0.99	+	0	ID=MALK_02298.t1.c4;Parent=MALK_02298.t1
contig009	AUGUSTUS	CDS	157091	157261	0.99	+	0	ID=MALK_02298.t1.c5;Parent=MALK_02298.t1
contig009	AUGUSTUS	mRNA	156736	157261	0.32	+	.	ID=MALK_02298.t1;Parent=MALK_02298
contig009	AUGUSTUS	exon	156736	156792	.	+	.	ID=MALK_02298.t1.e1;Parent=MALK_02298.t1
contig009	AUGUSTUS	exon	156822	156863	.	+	.	ID=MALK_02298.t1.e2;Parent=MALK_02298.t1
contig009	AUGUSTUS	exon	156892	156985	.	+	.	ID=MALK_02298.t1.e3;Parent=MALK_02298.t1
contig009	AUGUSTUS	exon	157016	157047	.	+	.	ID=MALK_02298.t1.e4;Parent=MALK_02298.t1
contig009	AUGUSTUS	exon	157091	157261	.	+	.	ID=MALK_02298.t1.e5;Parent=MALK_02298.t1
contig009	AUGUSTUS	gene	157283	157723	1	-	.	ID=MALK_02299;prediction_source=augustus:contig009.g2523.t1
contig009	AUGUSTUS	CDS	157283	157723	1	-	0	ID=MALK_02299.t1.c1;Parent=MALK_02299.t1
contig009	AUGUSTUS	mRNA	157283	157723	1	-	.	ID=MALK_02299.t1;Parent=MALK_02299
contig009	AUGUSTUS	exon	157283	157723	1	-	.	ID=MALK_02299.t1.e1;Parent=MALK_02299.t1
contig009	AUGUSTUS	gene	157786	159600	0.67	-	.	ID=MALK_02300;prediction_source=braker_MRET:g2497.t1
contig009	AUGUSTUS	CDS	157786	159600	0.67	-	0	ID=MALK_02300.t1.c1;Parent=MALK_02300.t1
contig009	AUGUSTUS	mRNA	157786	159600	0.67	-	.	ID=MALK_02300.t1;Parent=MALK_02300
contig009	AUGUSTUS	exon	157786	159600	.	-	.	ID=MALK_02300.t1.e1;Parent=MALK_02300.t1
contig009	AUGUSTUS	gene	159735	160958	1	+	.	ID=MALK_02301;prediction_source=augustus:contig009.g2525.t1
contig009	AUGUSTUS	CDS	159735	160958	1	+	0	ID=MALK_02301.t1.c1;Parent=MALK_02301.t1
contig009	AUGUSTUS	mRNA	159735	160958	1	+	.	ID=MALK_02301.t1;Parent=MALK_02301
contig009	AUGUSTUS	exon	159735	160958	1	+	.	ID=MALK_02301.t1.e1;Parent=MALK_02301.t1
contig009	AUGUSTUS	gene	160969	163116	0.87	-	.	ID=MALK_02302;prediction_source=augustus:contig009.g2526.t1
contig009	AUGUSTUS	CDS	160969	163116	0.87	-	0	ID=MALK_02302.t1.c1;Parent=MALK_02302.t1
contig009	AUGUSTUS	mRNA	160969	163116	0.87	-	.	ID=MALK_02302.t1;Parent=MALK_02302
contig009	AUGUSTUS	exon	160969	163116	0.87	-	.	ID=MALK_02302.t1.e1;Parent=MALK_02302.t1
contig009	AUGUSTUS	gene	163341	163925	0.98	+	.	ID=MALK_02303;prediction_source=braker_MRET:g2500.t1
contig009	AUGUSTUS	CDS	163341	163925	0.98	+	0	ID=MALK_02303.t1.c1;Parent=MALK_02303.t1
contig009	AUGUSTUS	mRNA	163341	163925	0.98	+	.	ID=MALK_02303.t1;Parent=MALK_02303
contig009	AUGUSTUS	exon	163341	163925	.	+	.	ID=MALK_02303.t1.e1;Parent=MALK_02303.t1
contig009	AUGUSTUS	gene	163932	164616	0.47	-	.	ID=MALK_02304;prediction_source=braker_MRET:g2501.t1
contig009	AUGUSTUS	CDS	164007	164616	0.47	-	0	ID=MALK_02304.t1.c2;Parent=MALK_02304.t1
contig009	AUGUSTUS	CDS	163932	163966	0.47	-	0	ID=MALK_02304.t1.c1;Parent=MALK_02304.t1
contig009	AUGUSTUS	mRNA	163932	164616	0.47	-	.	ID=MALK_02304.t1;Parent=MALK_02304
contig009	AUGUSTUS	exon	164007	164616	.	-	.	ID=MALK_02304.t1.e2;Parent=MALK_02304.t1
contig009	AUGUSTUS	exon	163932	163966	.	-	.	ID=MALK_02304.t1.e1;Parent=MALK_02304.t1
contig009	AUGUSTUS	gene	164813	165280	0.66	+	.	ID=MALK_02305;prediction_source=braker_MRET:g2502.t1
contig009	AUGUSTUS	CDS	164813	165280	0.66	+	0	ID=MALK_02305.t1.c1;Parent=MALK_02305.t1
contig009	AUGUSTUS	mRNA	164813	165280	0.66	+	.	ID=MALK_02305.t1;Parent=MALK_02305
contig009	AUGUSTUS	exon	164813	165280	.	+	.	ID=MALK_02305.t1.e1;Parent=MALK_02305.t1
contig009	maker	gene	165347	166534	.	-	.	ID=MALK_02306;prediction_source=maker_MRET:augustus_masked-contig009-processed-gene-1.33-mRNA-1
contig009	maker	CDS	165347	166534	.	-	0	ID=MALK_02306.t1.c1;Parent=MALK_02306.t1

contig009	maker	mRNA	165347	166534	.	-	.	ID=MALK_02306.t1;Parent=MALK_02306
contig009	maker	exon	165347	166534	.	-	.	ID=MALK_02306.t1.e1;Parent=MALK_02306.t1
contig009	maker	gene	167407	170283	.	-	.	ID=MALK_02307;prediction_source=maker_MRET:augustus_masked-contig009-processed-gene-1.34-mRNA-1
contig009	maker	CDS	167407	170283	.	-	0	ID=MALK_02307.t1.c1;Parent=MALK_02307.t1
contig009	maker	mRNA	167407	170283	.	-	.	ID=MALK_02307.t1;Parent=MALK_02307
contig009	maker	exon	167407	170283	.	-	.	ID=MALK_02307.t1.e1;Parent=MALK_02307.t1
contig009	AUGUSTUS	gene	170458	171822	0.93	-	.	ID=MALK_02308;prediction_source=augustus:contig009.g2529.t1
contig009	AUGUSTUS	CDS	170458	171822	0.93	-	0	ID=MALK_02308.t1.c1;Parent=MALK_02308.t1
contig009	AUGUSTUS	mRNA	170458	171822	0.93	-	.	ID=MALK_02308.t1;Parent=MALK_02308
contig009	AUGUSTUS	exon	170458	171822	0.93	-	.	ID=MALK_02308.t1.e1;Parent=MALK_02308.t1
contig009	maker	gene	172172	174688	.	+	.	ID=MALK_02309;prediction_source=maker_MRET:augustus_masked-contig009-processed-gene-1.13-mRNA-1
contig009	maker	CDS	172172	174688	.	+	0	ID=MALK_02309.t1.c1;Parent=MALK_02309.t1
contig009	maker	mRNA	172172	174688	.	+	.	ID=MALK_02309.t1;Parent=MALK_02309
contig009	maker	exon	172172	174688	.	+	.	ID=MALK_02309.t1.e1;Parent=MALK_02309.t1
contig009	maker	gene	174702	175583	.	-	.	ID=MALK_02310;prediction_source=maker_MRET:augustus_masked-contig009-processed-gene-1.36-mRNA-1
contig009	maker	CDS	174702	175583	.	-	0	ID=MALK_02310.t1.c1;Parent=MALK_02310.t1
contig009	maker	mRNA	174702	175583	.	-	.	ID=MALK_02310.t1;Parent=MALK_02310
contig009	maker	exon	174702	175583	.	-	.	ID=MALK_02310.t1.e1;Parent=MALK_02310.t1
contig009	AUGUSTUS	gene	175678	178236	0.61	-	.	ID=MALK_02311;prediction_source=braker_MRET:g2508.t1
contig009	AUGUSTUS	CDS	175678	178236	0.61	-	0	ID=MALK_02311.t1.c1;Parent=MALK_02311.t1
contig009	AUGUSTUS	mRNA	175678	178236	0.61	-	.	ID=MALK_02311.t1;Parent=MALK_02311
contig009	AUGUSTUS	exon	175678	178236	.	-	.	ID=MALK_02311.t1.e1;Parent=MALK_02311.t1
contig009	AUGUSTUS	gene	178416	179075	0.95	-	.	ID=MALK_02312;prediction_source=braker_MRET:g2509.t1
contig009	AUGUSTUS	CDS	178416	179075	0.95	-	0	ID=MALK_02312.t1.c1;Parent=MALK_02312.t1
contig009	AUGUSTUS	mRNA	178416	179075	0.95	-	.	ID=MALK_02312.t1;Parent=MALK_02312
contig009	AUGUSTUS	exon	178416	179075	.	-	.	ID=MALK_02312.t1.e1;Parent=MALK_02312.t1
contig009	AUGUSTUS	gene	179105	182062	0.94	-	.	ID=MALK_02313;prediction_source=braker_MRET:g2510.t1
contig009	AUGUSTUS	CDS	179105	182062	0.94	-	0	ID=MALK_02313.t1.c1;Parent=MALK_02313.t1
contig009	AUGUSTUS	mRNA	179105	182062	0.94	-	.	ID=MALK_02313.t1;Parent=MALK_02313
contig009	AUGUSTUS	exon	179105	182062	.	-	.	ID=MALK_02313.t1.e1;Parent=MALK_02313.t1
contig009	maker	gene	182086	183102	.	-	.	ID=MALK_02314;prediction_source=maker_MRET:augustus_masked-contig009-processed-gene-1.39-mRNA-1
contig009	maker	CDS	182086	183102	.	-	0	ID=MALK_02314.t1.c1;Parent=MALK_02314.t1
contig009	maker	mRNA	182086	183102	.	-	.	ID=MALK_02314.t1;Parent=MALK_02314
contig009	maker	exon	182086	183102	.	-	.	ID=MALK_02314.t1.e1;Parent=MALK_02314.t1
contig009	maker	gene	183356	185356	.	+	.	ID=MALK_02315;prediction_source=maker_MRET:augustus_masked-contig009-processed-gene-1.14-mRNA-1
contig009	maker	CDS	183356	183872	.	+	0	ID=MALK_02315.t1.c1;Parent=MALK_02315.t1
contig009	maker	CDS	184014	185356	.	+	0	ID=MALK_02315.t1.c2;Parent=MALK_02315.t1
contig009	maker	mRNA	183356	185356	.	+	.	ID=MALK_02315.t1;Parent=MALK_02315
contig009	maker	exon	183356	183872	.	+	.	ID=MALK_02315.t1.e1;Parent=MALK_02315.t1
contig009	maker	exon	184014	185356	.	+	.	ID=MALK_02315.t1.e2;Parent=MALK_02315.t1
contig009	AUGUSTUS	gene	185392	189117	0.46	-	.	ID=MALK_02316;prediction_source=braker_MRET:g2513.t1
contig009	AUGUSTUS	CDS	185392	189117	0.46	-	0	ID=MALK_02316.t1.c1;Parent=MALK_02316.t1
contig009	AUGUSTUS	mRNA	185392	189117	0.46	-	.	ID=MALK_02316.t1;Parent=MALK_02316

contig009	AUGUSTUS	exon	185392	189117	.	-	.	ID=MALK_02316.t1.e1;Parent=MALK_02316.t1
contig009	AUGUSTUS	gene	189281	190531	0.99	-	.	ID=MALK_02317;prediction_source=augustus:contig009.g2537.t1
contig009	AUGUSTUS	CDS	189281	190531	0.99	-	0	ID=MALK_02317.t1.c1;Parent=MALK_02317.t1
contig009	AUGUSTUS	mRNA	189281	190531	0.99	-	.	ID=MALK_02317.t1;Parent=MALK_02317
contig009	AUGUSTUS	exon	189281	190531	0.99	-	.	ID=MALK_02317.t1.e1;Parent=MALK_02317.t1
contig009	AUGUSTUS	gene	192784	200532	0.29	+	.	ID=MALK_02318;prediction_source=braker_MRET:g2515.t1
contig009	AUGUSTUS	CDS	192784	200532	0.29	+	0	ID=MALK_02318.t1.c1;Parent=MALK_02318.t1
contig009	AUGUSTUS	mRNA	192784	200532	0.29	+	.	ID=MALK_02318.t1;Parent=MALK_02318
contig009	AUGUSTUS	exon	192784	200532	.	+	.	ID=MALK_02318.t1.e1;Parent=MALK_02318.t1
contig009	AUGUSTUS	gene	200538	201833	0.52	-	.	ID=MALK_02319;prediction_source=braker_MRET:g2516.t1
contig009	AUGUSTUS	CDS	200538	201833	0.52	-	0	ID=MALK_02319.t1.c1;Parent=MALK_02319.t1
contig009	AUGUSTUS	mRNA	200538	201833	0.52	-	.	ID=MALK_02319.t1;Parent=MALK_02319
contig009	AUGUSTUS	exon	200538	201833	.	-	.	ID=MALK_02319.t1.e1;Parent=MALK_02319.t1
contig009	AUGUSTUS	gene	201853	203334	0.99	-	.	ID=MALK_02320;prediction_source=braker_MRET:g2517.t1
contig009	AUGUSTUS	CDS	201853	203334	0.99	-	0	ID=MALK_02320.t1.c1;Parent=MALK_02320.t1
contig009	AUGUSTUS	mRNA	201853	203334	0.99	-	.	ID=MALK_02320.t1;Parent=MALK_02320
contig009	AUGUSTUS	exon	201853	203334	.	-	.	ID=MALK_02320.t1.e1;Parent=MALK_02320.t1
contig009	maker	gene	203500	206499	.	+	.	ID=MALK_02321;prediction_source=maker_MRET:augustus_masked-contig009-processed-gene-2.64-mRNA-1
contig009	maker	CDS	203500	206499	.	+	0	ID=MALK_02321.t1.c1;Parent=MALK_02321.t1
contig009	maker	mRNA	203500	206499	.	+	.	ID=MALK_02321.t1;Parent=MALK_02321
contig009	maker	exon	203500	206499	.	+	.	ID=MALK_02321.t1.e1;Parent=MALK_02321.t1
contig009	AUGUSTUS	gene	206496	207683	0.81	-	.	ID=MALK_02322;prediction_source=augustus:contig009.g2541.t1
contig009	AUGUSTUS	CDS	206496	207683	0.81	-	0	ID=MALK_02322.t1.c1;Parent=MALK_02322.t1
contig009	AUGUSTUS	mRNA	206496	207683	0.81	-	.	ID=MALK_02322.t1;Parent=MALK_02322
contig009	AUGUSTUS	exon	206496	207683	0.81	-	.	ID=MALK_02322.t1.e1;Parent=MALK_02322.t1
contig009	AUGUSTUS	gene	207819	209906	0.99	+	.	ID=MALK_02323;prediction_source=augustus:contig009.g2542.t1
contig009	AUGUSTUS	CDS	207819	209906	0.99	+	0	ID=MALK_02323.t1.c1;Parent=MALK_02323.t1
contig009	AUGUSTUS	mRNA	207819	209906	0.99	+	.	ID=MALK_02323.t1;Parent=MALK_02323
contig009	AUGUSTUS	exon	207819	209906	0.99	+	.	ID=MALK_02323.t1.e1;Parent=MALK_02323.t1
contig009	AUGUSTUS	gene	209911	211416	0.95	-	.	ID=MALK_02324;prediction_source=augustus:contig009.g2543.t1
contig009	AUGUSTUS	CDS	209911	211416	0.95	-	0	ID=MALK_02324.t1.c1;Parent=MALK_02324.t1
contig009	AUGUSTUS	mRNA	209911	211416	0.95	-	.	ID=MALK_02324.t1;Parent=MALK_02324
contig009	AUGUSTUS	exon	209911	211416	0.95	-	.	ID=MALK_02324.t1.e1;Parent=MALK_02324.t1
contig009	maker	gene	211510	213666	.	-	.	ID=MALK_02325;prediction_source=maker_MRET:augustus_masked-contig009-processed-gene-2.90-mRNA-1
contig009	maker	CDS	211510	213666	.	-	0	ID=MALK_02325.t1.c1;Parent=MALK_02325.t1
contig009	maker	mRNA	211510	213666	.	-	.	ID=MALK_02325.t1;Parent=MALK_02325
contig009	maker	exon	211510	213666	.	-	.	ID=MALK_02325.t1.e1;Parent=MALK_02325.t1
contig009	AUGUSTUS	gene	213920	214981	0.82	+	.	ID=MALK_02326;prediction_source=augustus:contig009.g2546.t1
contig009	AUGUSTUS	CDS	213920	214981	0.82	+	0	ID=MALK_02326.t1.c1;Parent=MALK_02326.t1
contig009	AUGUSTUS	mRNA	213920	214981	0.82	+	.	ID=MALK_02326.t1;Parent=MALK_02326
contig009	AUGUSTUS	exon	213920	214981	0.82	+	.	ID=MALK_02326.t1.e1;Parent=MALK_02326.t1
contig009	maker	gene	215650	217836	.	-	.	ID=MALK_02327;prediction_source=maker_MRET:augustus_masked-contig009-processed-gene-2.91-mRNA-1
contig009	maker	CDS	215650	217836	.	-	0	ID=MALK_02327.t1.c1;Parent=MALK_02327.t1

contig009	maker	mRNA	215650	217836	.	-	.	ID=MALK_02327.t1;Parent=MALK_02327
contig009	maker	exon	215650	217836	.	-	.	ID=MALK_02327.t1.e1;Parent=MALK_02327.t1
contig009	AUGUSTUS	gene	218425	219402	0.4	+	.	ID=MALK_02328;prediction_source=augustus:contig009.g2548.t1
contig009	AUGUSTUS	CDS	218425	219402	0.4	+	0	ID=MALK_02328.t1.c1;Parent=MALK_02328.t1
contig009	AUGUSTUS	mRNA	218425	219402	0.4	+	.	ID=MALK_02328.t1;Parent=MALK_02328
contig009	AUGUSTUS	exon	218425	219402	0.4	+	.	ID=MALK_02328.t1.e1;Parent=MALK_02328.t1
contig009	AUGUSTUS	gene	219443	221260	0.53	+	.	ID=MALK_02329;prediction_source=augustus:contig009.g2549.t1
contig009	AUGUSTUS	CDS	219443	221260	0.53	+	0	ID=MALK_02329.t1.c1;Parent=MALK_02329.t1
contig009	AUGUSTUS	mRNA	219443	221260	0.53	+	.	ID=MALK_02329.t1;Parent=MALK_02329
contig009	AUGUSTUS	exon	219443	221260	0.53	+	.	ID=MALK_02329.t1.e1;Parent=MALK_02329.t1
contig009	AUGUSTUS	gene	221257	224358	0.97	-	.	ID=MALK_02330;prediction_source=augustus:contig009.g2550.t1
contig009	AUGUSTUS	CDS	221257	224358	0.97	-	0	ID=MALK_02330.t1.c1;Parent=MALK_02330.t1
contig009	AUGUSTUS	mRNA	221257	224358	0.97	-	.	ID=MALK_02330.t1;Parent=MALK_02330
contig009	AUGUSTUS	exon	221257	224358	0.97	-	.	ID=MALK_02330.t1.e1;Parent=MALK_02330.t1
contig009	maker	gene	224476	226221	.	+	.	ID=MALK_02331;prediction_source=maker_MRET:augustus_masked-contig009-processed-gene-2.68-mRNA-1
contig009	maker	CDS	224476	226221	.	+	0	ID=MALK_02331.t1.c1;Parent=MALK_02331.t1
contig009	maker	mRNA	224476	226221	.	+	.	ID=MALK_02331.t1;Parent=MALK_02331
contig009	maker	exon	224476	226221	.	+	.	ID=MALK_02331.t1.e1;Parent=MALK_02331.t1
contig009	AUGUSTUS	gene	226233	227873	0.99	-	.	ID=MALK_02332;prediction_source=augustus:contig009.g2552.t1
contig009	AUGUSTUS	CDS	226233	227873	0.99	-	0	ID=MALK_02332.t1.c1;Parent=MALK_02332.t1
contig009	AUGUSTUS	mRNA	226233	227873	0.99	-	.	ID=MALK_02332.t1;Parent=MALK_02332
contig009	AUGUSTUS	exon	226233	227873	0.99	-	.	ID=MALK_02332.t1.e1;Parent=MALK_02332.t1
contig009	AUGUSTUS	gene	227953	228840	0.99	-	.	ID=MALK_02333;prediction_source=augustus:contig009.g2554.t1
contig009	AUGUSTUS	CDS	227953	228840	0.99	-	0	ID=MALK_02333.t1.c1;Parent=MALK_02333.t1
contig009	AUGUSTUS	mRNA	227953	228840	0.99	-	.	ID=MALK_02333.t1;Parent=MALK_02333
contig009	AUGUSTUS	exon	227953	228840	0.99	-	.	ID=MALK_02333.t1.e1;Parent=MALK_02333.t1
contig009	maker	gene	228891	229622	.	+	.	ID=MALK_02334;prediction_source=maker_MRET:augustus_masked-contig009-processed-gene-2.69-mRNA-1
contig009	maker	CDS	228891	229622	.	+	0	ID=MALK_02334.t1.c1;Parent=MALK_02334.t1
contig009	maker	mRNA	228891	229622	.	+	.	ID=MALK_02334.t1;Parent=MALK_02334
contig009	maker	exon	228891	229622	.	+	.	ID=MALK_02334.t1.e1;Parent=MALK_02334.t1
contig009	AUGUSTUS	gene	229693	230387	0.3	+	.	ID=MALK_02335;prediction_source=braker_MRET:g2532.t1
contig009	AUGUSTUS	CDS	229693	230346	0.67	+	0	ID=MALK_02335.t1.c1;Parent=MALK_02335.t1
contig009	AUGUSTUS	CDS	230376	230387	0.67	+	0	ID=MALK_02335.t1.c2;Parent=MALK_02335.t1
contig009	AUGUSTUS	mRNA	229693	230387	0.3	+	.	ID=MALK_02335.t1;Parent=MALK_02335
contig009	AUGUSTUS	exon	229693	230346	.	+	.	ID=MALK_02335.t1.e1;Parent=MALK_02335.t1
contig009	AUGUSTUS	exon	230376	230387	.	+	.	ID=MALK_02335.t1.e2;Parent=MALK_02335.t1
contig009	AUGUSTUS	gene	230391	233216	0.67	-	.	ID=MALK_02336;prediction_source=braker_MRET:g2533.t1
contig009	AUGUSTUS	CDS	230391	233216	0.67	-	0	ID=MALK_02336.t1.c1;Parent=MALK_02336.t1
contig009	AUGUSTUS	mRNA	230391	233216	0.67	-	.	ID=MALK_02336.t1;Parent=MALK_02336
contig009	AUGUSTUS	exon	230391	233216	.	-	.	ID=MALK_02336.t1.e1;Parent=MALK_02336.t1
contig009	AUGUSTUS	gene	233253	235205	1	-	.	ID=MALK_02337;prediction_source=braker_MRET:g2534.t1
contig009	AUGUSTUS	CDS	233253	235205	1	-	0	ID=MALK_02337.t1.c1;Parent=MALK_02337.t1
contig009	AUGUSTUS	mRNA	233253	235205	1	-	.	ID=MALK_02337.t1;Parent=MALK_02337

contig009	AUGUSTUS	exon	233253	235205	.	-	.	ID=MALK_02337.t1.e1;Parent=MALK_02337.t1
contig009	AUGUSTUS	gene	235528	238452	0.59	+	.	ID=MALK_02338;prediction_source=augustus:contig009.g2558.t1
contig009	AUGUSTUS	CDS	235528	238452	0.59	+	0	ID=MALK_02338.t1.c1;Parent=MALK_02338.t1
contig009	AUGUSTUS	mRNA	235528	238452	0.59	+	.	ID=MALK_02338.t1;Parent=MALK_02338
contig009	AUGUSTUS	exon	235528	238452	0.59	+	.	ID=MALK_02338.t1.e1;Parent=MALK_02338.t1
contig009	AUGUSTUS	gene	239560	240265	0.43	-	.	ID=MALK_02339;prediction_source=braker_MRET:g2536.t1
contig009	AUGUSTUS	CDS	239667	240265	0.43	-	0	ID=MALK_02339.t1.c2;Parent=MALK_02339.t1
contig009	AUGUSTUS	CDS	239560	239629	0.43	-	0	ID=MALK_02339.t1.c1;Parent=MALK_02339.t1
contig009	AUGUSTUS	mRNA	239560	240265	0.43	-	.	ID=MALK_02339.t1;Parent=MALK_02339
contig009	AUGUSTUS	exon	239667	240265	.	-	.	ID=MALK_02339.t1.e2;Parent=MALK_02339.t1
contig009	AUGUSTUS	exon	239560	239629	.	-	.	ID=MALK_02339.t1.e1;Parent=MALK_02339.t1
contig009	maker	gene	240543	241310	.	-	.	ID=MALK_02340;prediction_source=maker_MRET:augustus_masked-contig009-processed-gene-2.96-mRNA-1
contig009	maker	CDS	240543	241310	.	-	0	ID=MALK_02340.t1.c1;Parent=MALK_02340.t1
contig009	maker	mRNA	240543	241310	.	-	.	ID=MALK_02340.t1;Parent=MALK_02340
contig009	maker	exon	240543	241310	.	-	.	ID=MALK_02340.t1.e1;Parent=MALK_02340.t1
contig009	AUGUSTUS	gene	241380	242453	0.5	-	.	ID=MALK_02341;prediction_source=augustus:contig009.g2560.t1
contig009	AUGUSTUS	CDS	241380	242453	0.5	-	0	ID=MALK_02341.t1.c1;Parent=MALK_02341.t1
contig009	AUGUSTUS	mRNA	241380	242453	0.5	-	.	ID=MALK_02341.t1;Parent=MALK_02341
contig009	AUGUSTUS	exon	241380	242453	0.5	-	.	ID=MALK_02341.t1.e1;Parent=MALK_02341.t1
contig009	AUGUSTUS	gene	242541	243542	0.61	-	.	ID=MALK_02342;prediction_source=augustus:contig009.g2561.t1
contig009	AUGUSTUS	CDS	242541	243542	0.61	-	0	ID=MALK_02342.t1.c1;Parent=MALK_02342.t1
contig009	AUGUSTUS	mRNA	242541	243542	0.61	-	.	ID=MALK_02342.t1;Parent=MALK_02342
contig009	AUGUSTUS	exon	242541	243542	0.61	-	.	ID=MALK_02342.t1.e1;Parent=MALK_02342.t1
contig009	AUGUSTUS	gene	244480	245046	0.41	+	.	ID=MALK_02343;prediction_source=augustus:contig009.g2562.t1
contig009	AUGUSTUS	CDS	244480	245046	0.41	+	0	ID=MALK_02343.t1.c1;Parent=MALK_02343.t1
contig009	AUGUSTUS	mRNA	244480	245046	0.41	+	.	ID=MALK_02343.t1;Parent=MALK_02343
contig009	AUGUSTUS	exon	244480	245046	0.41	+	.	ID=MALK_02343.t1.e1;Parent=MALK_02343.t1
contig009	AUGUSTUS	gene	245033	246853	0.9	-	.	ID=MALK_02344;prediction_source=braker_MRET:g2541.t1
contig009	AUGUSTUS	CDS	245033	246853	0.9	-	0	ID=MALK_02344.t1.c1;Parent=MALK_02344.t1
contig009	AUGUSTUS	mRNA	245033	246853	0.9	-	.	ID=MALK_02344.t1;Parent=MALK_02344
contig009	AUGUSTUS	exon	245033	246853	.	-	.	ID=MALK_02344.t1.e1;Parent=MALK_02344.t1
contig009	AUGUSTUS	gene	246988	248748	0.51	-	.	ID=MALK_02345;prediction_source=augustus:contig009.g2564.t1
contig009	AUGUSTUS	CDS	246988	248748	0.51	-	0	ID=MALK_02345.t1.c1;Parent=MALK_02345.t1
contig009	AUGUSTUS	mRNA	246988	248748	0.51	-	.	ID=MALK_02345.t1;Parent=MALK_02345
contig009	AUGUSTUS	exon	246988	248748	0.51	-	.	ID=MALK_02345.t1.e1;Parent=MALK_02345.t1
contig009	maker	gene	248983	249936	.	+	.	ID=MALK_02346;prediction_source=maker_MRET:augustus_masked-contig009-processed-gene-2.71-mRNA-1
contig009	maker	CDS	248983	249936	.	+	0	ID=MALK_02346.t1.c1;Parent=MALK_02346.t1
contig009	maker	mRNA	248983	249936	.	+	.	ID=MALK_02346.t1;Parent=MALK_02346
contig009	maker	exon	248983	249936	.	+	.	ID=MALK_02346.t1.e1;Parent=MALK_02346.t1
contig009	maker	gene	249966	251584	.	-	.	ID=MALK_02347;prediction_source=maker_MRET:augustus_masked-contig009-processed-gene-2.100-mRNA-1
contig009	maker	CDS	251529	251584	.	-	0	ID=MALK_02347.t1.c1;Parent=MALK_02347.t1
contig009	maker	CDS	249966	251466	.	-	0	ID=MALK_02347.t1.c2;Parent=MALK_02347.t1
contig009	maker	mRNA	249966	251584	.	-	.	ID=MALK_02347.t1;Parent=MALK_02347

contig009	maker	exon	251529	251584	.	-	.	ID=MALK_02347.t1.e1;Parent=MALK_02347.t1
contig009	maker	exon	249966	251466	.	-	.	ID=MALK_02347.t1.e2;Parent=MALK_02347.t1
contig009	AUGUSTUS	gene	251803	252795	0.57	+	.	ID=MALK_02348;prediction_source=augustus:contig009.g2568.t1
contig009	AUGUSTUS	CDS	251803	252795	0.57	+	0	ID=MALK_02348.t1.c1;Parent=MALK_02348.t1
contig009	AUGUSTUS	mRNA	251803	252795	0.57	+	.	ID=MALK_02348.t1;Parent=MALK_02348
contig009	AUGUSTUS	exon	251803	252795	0.57	+	.	ID=MALK_02348.t1.e1;Parent=MALK_02348.t1
contig009	maker	gene	252820	253200	.	-	.	ID=MALK_02349;prediction_source=maker_MRET:augustus_masked-contig009-processed-gene-2.101-mRNA-1
contig009	maker	CDS	252820	253200	.	-	0	ID=MALK_02349.t1.c1;Parent=MALK_02349.t1
contig009	maker	mRNA	252820	253200	.	-	.	ID=MALK_02349.t1;Parent=MALK_02349
contig009	maker	exon	252820	253200	.	-	.	ID=MALK_02349.t1.e1;Parent=MALK_02349.t1
contig009	maker	gene	253452	254198	.	+	.	ID=MALK_02350;prediction_source=maker_MRET:augustus_masked-contig009-processed-gene-2.73-mRNA-1
contig009	maker	CDS	253452	254198	.	+	0	ID=MALK_02350.t1.c1;Parent=MALK_02350.t1
contig009	maker	mRNA	253452	254198	.	+	.	ID=MALK_02350.t1;Parent=MALK_02350
contig009	maker	exon	253452	254198	.	+	.	ID=MALK_02350.t1.e1;Parent=MALK_02350.t1
contig009	AUGUSTUS	gene	254278	254526	0.83	+	.	ID=MALK_02351;prediction_source=braker_MRET:g2548.t1
contig009	AUGUSTUS	CDS	254278	254526	0.83	+	0	ID=MALK_02351.t1.c1;Parent=MALK_02351.t1
contig009	AUGUSTUS	mRNA	254278	254526	0.83	+	.	ID=MALK_02351.t1;Parent=MALK_02351
contig009	AUGUSTUS	exon	254278	254526	.	+	.	ID=MALK_02351.t1.e1;Parent=MALK_02351.t1
contig009	maker	gene	254639	255205	.	-	.	ID=MALK_02352;prediction_source=maker_MRET:augustus_masked-contig009-processed-gene-2.102-mRNA-1
contig009	maker	CDS	254639	255205	.	-	0	ID=MALK_02352.t1.c1;Parent=MALK_02352.t1
contig009	maker	mRNA	254639	255205	.	-	.	ID=MALK_02352.t1;Parent=MALK_02352
contig009	maker	exon	254639	255205	.	-	.	ID=MALK_02352.t1.e1;Parent=MALK_02352.t1
contig009	maker	gene	256122	257912	.	+	.	ID=MALK_02353;prediction_source=maker_MRET:augustus_masked-contig009-processed-gene-2.74-mRNA-1
contig009	maker	CDS	256122	257912	.	+	0	ID=MALK_02353.t1.c1;Parent=MALK_02353.t1
contig009	maker	mRNA	256122	257912	.	+	.	ID=MALK_02353.t1;Parent=MALK_02353
contig009	maker	exon	256122	257912	.	+	.	ID=MALK_02353.t1.e1;Parent=MALK_02353.t1
contig009	AUGUSTUS	gene	257913	259295	0.89	-	.	ID=MALK_02354;prediction_source=braker_MRET:g2551.t1
contig009	AUGUSTUS	CDS	257913	259295	0.89	-	0	ID=MALK_02354.t1.c1;Parent=MALK_02354.t1
contig009	AUGUSTUS	mRNA	257913	259295	0.89	-	.	ID=MALK_02354.t1;Parent=MALK_02354
contig009	AUGUSTUS	exon	257913	259295	.	-	.	ID=MALK_02354.t1.e1;Parent=MALK_02354.t1
contig009	AUGUSTUS	gene	259321	260429	0.56	+	.	ID=MALK_02355;prediction_source=braker_MRET:g2552.t1
contig009	AUGUSTUS	CDS	259321	260339	0.61	+	0	ID=MALK_02355.t1.c1;Parent=MALK_02355.t1
contig009	AUGUSTUS	CDS	260378	260429	0.61	+	0	ID=MALK_02355.t1.c2;Parent=MALK_02355.t1
contig009	AUGUSTUS	mRNA	259321	260429	0.56	+	.	ID=MALK_02355.t1;Parent=MALK_02355
contig009	AUGUSTUS	exon	259321	260339	.	+	.	ID=MALK_02355.t1.e1;Parent=MALK_02355.t1
contig009	AUGUSTUS	exon	260378	260429	.	+	.	ID=MALK_02355.t1.e2;Parent=MALK_02355.t1
contig009	AUGUSTUS	gene	260458	261522	0.53	-	.	ID=MALK_02356;prediction_source=augustus:contig009.g2573.t1
contig009	AUGUSTUS	CDS	260458	261522	0.53	-	0	ID=MALK_02356.t1.c1;Parent=MALK_02356.t1
contig009	AUGUSTUS	mRNA	260458	261522	0.53	-	.	ID=MALK_02356.t1;Parent=MALK_02356
contig009	AUGUSTUS	exon	260458	261522	0.53	-	.	ID=MALK_02356.t1.e1;Parent=MALK_02356.t1
contig009	maker	gene	261638	262939	.	+	.	ID=MALK_02357;prediction_source=maker_MRET:augustus_masked-contig009-processed-gene-2.75-mRNA-1
contig009	maker	CDS	261638	262939	.	+	0	ID=MALK_02357.t1.c1;Parent=MALK_02357.t1
contig009	maker	mRNA	261638	262939	.	+	.	ID=MALK_02357.t1;Parent=MALK_02357

contig009	maker	exon	261638	262939	.	+	.	ID=MALK_02357.t1.e1;Parent=MALK_02357.t1
contig009	AUGUSTUS	gene	262941	263972	0.86	-	.	ID=MALK_02358;prediction_source=braker_MRET:g2555.t1
contig009	AUGUSTUS	CDS	262941	263972	0.86	-	0	ID=MALK_02358.t1.c1;Parent=MALK_02358.t1
contig009	AUGUSTUS	mRNA	262941	263972	0.86	-	.	ID=MALK_02358.t1;Parent=MALK_02358
contig009	AUGUSTUS	exon	262941	263972	.	-	.	ID=MALK_02358.t1.e1;Parent=MALK_02358.t1
contig009	AUGUSTUS	gene	264156	264708	0.55	-	.	ID=MALK_02359;prediction_source=braker_MRET:g2556.t1
contig009	AUGUSTUS	CDS	264279	264708	0.64	-	0	ID=MALK_02359.t1.c2;Parent=MALK_02359.t1
contig009	AUGUSTUS	CDS	264156	264247	0.64	-	0	ID=MALK_02359.t1.c1;Parent=MALK_02359.t1
contig009	AUGUSTUS	mRNA	264156	264708	0.55	-	.	ID=MALK_02359.t1;Parent=MALK_02359
contig009	AUGUSTUS	exon	264279	264708	.	-	.	ID=MALK_02359.t1.e2;Parent=MALK_02359.t1
contig009	AUGUSTUS	exon	264156	264247	.	-	.	ID=MALK_02359.t1.e1;Parent=MALK_02359.t1
contig009	AUGUSTUS	gene	265471	265905	1	+	.	ID=MALK_02360;prediction_source=braker_MRET:g2557.t1
contig009	AUGUSTUS	CDS	265471	265481	1	+	0	ID=MALK_02360.t1.c1;Parent=MALK_02360.t1
contig009	AUGUSTUS	CDS	265521	265578	1	+	0	ID=MALK_02360.t1.c2;Parent=MALK_02360.t1
contig009	AUGUSTUS	CDS	265642	265905	1	+	0	ID=MALK_02360.t1.c3;Parent=MALK_02360.t1
contig009	AUGUSTUS	mRNA	265471	265905	1	+	.	ID=MALK_02360.t1;Parent=MALK_02360
contig009	AUGUSTUS	exon	265471	265481	.	+	.	ID=MALK_02360.t1.e1;Parent=MALK_02360.t1
contig009	AUGUSTUS	exon	265521	265578	.	+	.	ID=MALK_02360.t1.e2;Parent=MALK_02360.t1
contig009	AUGUSTUS	exon	265642	265905	.	+	.	ID=MALK_02360.t1.e3;Parent=MALK_02360.t1
contig009	AUGUSTUS	gene	266257	268212	0.89	+	.	ID=MALK_02361;prediction_source=augustus:contig009.g2577.t1
contig009	AUGUSTUS	CDS	266257	268212	0.89	+	0	ID=MALK_02361.t1.c1;Parent=MALK_02361.t1
contig009	AUGUSTUS	mRNA	266257	268212	0.89	+	.	ID=MALK_02361.t1;Parent=MALK_02361
contig009	AUGUSTUS	exon	266257	268212	0.89	+	.	ID=MALK_02361.t1.e1;Parent=MALK_02361.t1
contig009	AUGUSTUS	gene	268236	268745	0.88	+	.	ID=MALK_02362;prediction_source=augustus:contig009.g2578.t1
contig009	AUGUSTUS	CDS	268236	268745	0.88	+	0	ID=MALK_02362.t1.c1;Parent=MALK_02362.t1
contig009	AUGUSTUS	mRNA	268236	268745	0.88	+	.	ID=MALK_02362.t1;Parent=MALK_02362
contig009	AUGUSTUS	exon	268236	268745	0.88	+	.	ID=MALK_02362.t1.e1;Parent=MALK_02362.t1
contig009	AUGUSTUS	gene	269278	269964	0.68	-	.	ID=MALK_02363;prediction_source=augustus:contig009.g2579.t1
contig009	AUGUSTUS	CDS	269278	269964	0.68	-	0	ID=MALK_02363.t1.c1;Parent=MALK_02363.t1
contig009	AUGUSTUS	mRNA	269278	269964	0.68	-	.	ID=MALK_02363.t1;Parent=MALK_02363
contig009	AUGUSTUS	exon	269278	269964	0.68	-	.	ID=MALK_02363.t1.e1;Parent=MALK_02363.t1
contig009	AUGUSTUS	gene	270305	271906	0.47	-	.	ID=MALK_02364;prediction_source=augustus:contig009.g2580.t1
contig009	AUGUSTUS	CDS	270305	271906	0.47	-	0	ID=MALK_02364.t1.c1;Parent=MALK_02364.t1
contig009	AUGUSTUS	mRNA	270305	271906	0.47	-	.	ID=MALK_02364.t1;Parent=MALK_02364
contig009	AUGUSTUS	exon	270305	271906	0.47	-	.	ID=MALK_02364.t1.e1;Parent=MALK_02364.t1
contig009	AUGUSTUS	gene	272976	273464	0.66	+	.	ID=MALK_02365;prediction_source=augustus:contig009.g2581.t1
contig009	AUGUSTUS	CDS	272976	273464	0.66	+	0	ID=MALK_02365.t1.c1;Parent=MALK_02365.t1
contig009	AUGUSTUS	mRNA	272976	273464	0.66	+	.	ID=MALK_02365.t1;Parent=MALK_02365
contig009	AUGUSTUS	exon	272976	273464	0.66	+	.	ID=MALK_02365.t1.e1;Parent=MALK_02365.t1
contig009	maker	gene	273898	275334	.	+	.	ID=MALK_02366;prediction_source=maker_MRET:augustus_masked-contig009-processed-gene-2.80-mRNA-1
contig009	maker	CDS	273898	275334	.	+	0	ID=MALK_02366.t1.c1;Parent=MALK_02366.t1
contig009	maker	mRNA	273898	275334	.	+	.	ID=MALK_02366.t1;Parent=MALK_02366
contig009	maker	exon	273898	275334	.	+	.	ID=MALK_02366.t1.e1;Parent=MALK_02366.t1

contig009	maker	gene	275424	276179	.	-	.	ID=MALK_02367;prediction_source=maker_MRET:augustus_masked-contig009-processed-gene-2.107-mRNA-1
contig009	maker	CDS	275424	276179	.	-	0	ID=MALK_02367.t1.c1;Parent=MALK_02367.t1
contig009	maker	mRNA	275424	276179	.	-	.	ID=MALK_02367.t1;Parent=MALK_02367
contig009	maker	exon	275424	276179	.	-	.	ID=MALK_02367.t1.e1;Parent=MALK_02367.t1
contig009	AUGUSTUS	gene	276759	277457	0.32	+	.	ID=MALK_02368;prediction_source=braker_MRET:g2564.t1
contig009	AUGUSTUS	CDS	276759	277388	0.66	+	0	ID=MALK_02368.t1.c1;Parent=MALK_02368.t1
contig009	AUGUSTUS	CDS	277428	277457	0.66	+	0	ID=MALK_02368.t1.c2;Parent=MALK_02368.t1
contig009	AUGUSTUS	mRNA	276759	277457	0.32	+	.	ID=MALK_02368.t1;Parent=MALK_02368
contig009	AUGUSTUS	exon	276759	277388	.	+	.	ID=MALK_02368.t1.e1;Parent=MALK_02368.t1
contig009	AUGUSTUS	exon	277428	277457	.	+	.	ID=MALK_02368.t1.e2;Parent=MALK_02368.t1
contig009	AUGUSTUS	gene	278252	278869	0.61	+	.	ID=MALK_02369;prediction_source=augustus:contig009.g2587.t1
contig009	AUGUSTUS	CDS	278252	278869	0.61	+	0	ID=MALK_02369.t1.c1;Parent=MALK_02369.t1
contig009	AUGUSTUS	mRNA	278252	278869	0.61	+	.	ID=MALK_02369.t1;Parent=MALK_02369
contig009	AUGUSTUS	exon	278252	278869	0.61	+	.	ID=MALK_02369.t1.e1;Parent=MALK_02369.t1
contig009	maker	gene	279164	280888	.	+	.	ID=MALK_02370;prediction_source=maker_MRET:augustus_masked-contig009-processed-gene-2.83-mRNA-1
contig009	maker	CDS	279164	280888	.	+	0	ID=MALK_02370.t1.c1;Parent=MALK_02370.t1
contig009	maker	mRNA	279164	280888	.	+	.	ID=MALK_02370.t1;Parent=MALK_02370
contig009	maker	exon	279164	280888	.	+	.	ID=MALK_02370.t1.e1;Parent=MALK_02370.t1
contig009	AUGUSTUS	gene	280885	282633	0.37	-	.	ID=MALK_02371;prediction_source=augustus:contig009.g2590.t1
contig009	AUGUSTUS	CDS	280885	282633	0.37	-	0	ID=MALK_02371.t1.c1;Parent=MALK_02371.t1
contig009	AUGUSTUS	mRNA	280885	282633	0.37	-	.	ID=MALK_02371.t1;Parent=MALK_02371
contig009	AUGUSTUS	exon	280885	282633	0.37	-	.	ID=MALK_02371.t1.e1;Parent=MALK_02371.t1
contig009	AUGUSTUS	gene	282623	283210	0.54	+	.	ID=MALK_02372;prediction_source=braker_MRET:g2568.t1
contig009	AUGUSTUS	CDS	282623	283210	0.54	+	0	ID=MALK_02372.t1.c1;Parent=MALK_02372.t1
contig009	AUGUSTUS	mRNA	282623	283210	0.54	+	.	ID=MALK_02372.t1;Parent=MALK_02372
contig009	AUGUSTUS	exon	282623	283210	.	+	.	ID=MALK_02372.t1.e1;Parent=MALK_02372.t1
contig009	AUGUSTUS	gene	283225	283620	0.78	-	.	ID=MALK_02373;prediction_source=augustus:contig009.g2592.t1
contig009	AUGUSTUS	CDS	283225	283620	0.78	-	0	ID=MALK_02373.t1.c1;Parent=MALK_02373.t1
contig009	AUGUSTUS	mRNA	283225	283620	0.78	-	.	ID=MALK_02373.t1;Parent=MALK_02373
contig009	AUGUSTUS	exon	283225	283620	0.78	-	.	ID=MALK_02373.t1.e1;Parent=MALK_02373.t1
contig009	maker	gene	284243	285997	.	+	.	ID=MALK_02374;prediction_source=maker_MRET:augustus_masked-contig009-processed-gene-2.85-mRNA-1
contig009	maker	CDS	284243	285997	.	+	0	ID=MALK_02374.t1.c1;Parent=MALK_02374.t1
contig009	maker	mRNA	284243	285997	.	+	.	ID=MALK_02374.t1;Parent=MALK_02374
contig009	maker	exon	284243	285997	.	+	.	ID=MALK_02374.t1.e1;Parent=MALK_02374.t1
contig009	AUGUSTUS	gene	286001	287332	1	-	.	ID=MALK_02375;prediction_source=augustus:contig009.g2594.t1
contig009	AUGUSTUS	CDS	286001	287332	1	-	0	ID=MALK_02375.t1.c1;Parent=MALK_02375.t1
contig009	AUGUSTUS	mRNA	286001	287332	1	-	.	ID=MALK_02375.t1;Parent=MALK_02375
contig009	AUGUSTUS	exon	286001	287332	1	-	.	ID=MALK_02375.t1.e1;Parent=MALK_02375.t1
contig009	AUGUSTUS	gene	287404	289212	0.94	-	.	ID=MALK_02376;prediction_source=braker_MRET:g2572.t1
contig009	AUGUSTUS	CDS	287404	289212	0.94	-	0	ID=MALK_02376.t1.c1;Parent=MALK_02376.t1
contig009	AUGUSTUS	mRNA	287404	289212	0.94	-	.	ID=MALK_02376.t1;Parent=MALK_02376
contig009	AUGUSTUS	exon	287404	289212	.	-	.	ID=MALK_02376.t1.e1;Parent=MALK_02376.t1
contig009	AUGUSTUS	gene	289282	290892	0.79	+	.	ID=MALK_02377;prediction_source=augustus:contig009.g2596.t1

contig009	AUGUSTUS	CDS	289282	290892	0.79	+	0	ID=MALK_02377.t1.c1;Parent=MALK_02377.t1
contig009	AUGUSTUS	mRNA	289282	290892	0.79	+	.	ID=MALK_02377.t1;Parent=MALK_02377
contig009	AUGUSTUS	exon	289282	290892	0.79	+	.	ID=MALK_02377.t1.e1;Parent=MALK_02377.t1
contig009	AUGUSTUS	gene	291018	292457	0.69	-	.	ID=MALK_02378;prediction_source=augustus:contig009.g2597.t1
contig009	AUGUSTUS	CDS	291018	292457	0.69	-	0	ID=MALK_02378.t1.c1;Parent=MALK_02378.t1
contig009	AUGUSTUS	mRNA	291018	292457	0.69	-	.	ID=MALK_02378.t1;Parent=MALK_02378
contig009	AUGUSTUS	exon	291018	292457	0.69	-	.	ID=MALK_02378.t1.e1;Parent=MALK_02378.t1
contig009	maker	gene	292591	293841	.	+	.	ID=MALK_02379;prediction_source=maker_MRET:augustus_masked-contig009-processed-gene-2.87-mRNA-1
contig009	maker	CDS	292591	293841	.	+	0	ID=MALK_02379.t1.c1;Parent=MALK_02379.t1
contig009	maker	mRNA	292591	293841	.	+	.	ID=MALK_02379.t1;Parent=MALK_02379
contig009	maker	exon	292591	293841	.	+	.	ID=MALK_02379.t1.e1;Parent=MALK_02379.t1
contig009	maker	gene	293847	294254	.	-	.	ID=MALK_02380;prediction_source=maker_MRET:augustus_masked-contig009-processed-gene-3.49-mRNA-1
contig009	maker	CDS	293847	294254	.	-	0	ID=MALK_02380.t1.c1;Parent=MALK_02380.t1
contig009	maker	mRNA	293847	294254	.	-	.	ID=MALK_02380.t1;Parent=MALK_02380
contig009	maker	exon	293847	294254	.	-	.	ID=MALK_02380.t1.e1;Parent=MALK_02380.t1
contig009	maker	gene	294519	295886	.	-	.	ID=MALK_02381;prediction_source=maker_MRET:augustus_masked-contig009-processed-gene-3.50-mRNA-1
contig009	maker	CDS	294519	295886	.	-	0	ID=MALK_02381.t1.c1;Parent=MALK_02381.t1
contig009	maker	mRNA	294519	295886	.	-	.	ID=MALK_02381.t1;Parent=MALK_02381
contig009	maker	exon	294519	295886	.	-	.	ID=MALK_02381.t1.e1;Parent=MALK_02381.t1
contig009	AUGUSTUS	gene	296065	297567	0.34	+	.	ID=MALK_02382;prediction_source=braker_MRET:g2578.t1
contig009	AUGUSTUS	CDS	296065	296175	0.88	+	0	ID=MALK_02382.t1.c1;Parent=MALK_02382.t1
contig009	AUGUSTUS	CDS	296210	296334	0.88	+	0	ID=MALK_02382.t1.c2;Parent=MALK_02382.t1
contig009	AUGUSTUS	CDS	296366	296414	0.88	+	0	ID=MALK_02382.t1.c3;Parent=MALK_02382.t1
contig009	AUGUSTUS	CDS	296445	296787	0.88	+	0	ID=MALK_02382.t1.c4;Parent=MALK_02382.t1
contig009	AUGUSTUS	CDS	296872	297329	0.88	+	0	ID=MALK_02382.t1.c5;Parent=MALK_02382.t1
contig009	AUGUSTUS	CDS	297367	297567	0.88	+	0	ID=MALK_02382.t1.c6;Parent=MALK_02382.t1
contig009	AUGUSTUS	mRNA	296065	297567	0.34	+	.	ID=MALK_02382.t1;Parent=MALK_02382
contig009	AUGUSTUS	exon	296065	296175	.	+	.	ID=MALK_02382.t1.e1;Parent=MALK_02382.t1
contig009	AUGUSTUS	exon	296210	296334	.	+	.	ID=MALK_02382.t1.e2;Parent=MALK_02382.t1
contig009	AUGUSTUS	exon	296366	296414	.	+	.	ID=MALK_02382.t1.e3;Parent=MALK_02382.t1
contig009	AUGUSTUS	exon	296445	296787	.	+	.	ID=MALK_02382.t1.e4;Parent=MALK_02382.t1
contig009	AUGUSTUS	exon	296872	297329	.	+	.	ID=MALK_02382.t1.e5;Parent=MALK_02382.t1
contig009	AUGUSTUS	exon	297367	297567	.	+	.	ID=MALK_02382.t1.e6;Parent=MALK_02382.t1
contig009	AUGUSTUS	gene	297582	298114	0.64	-	.	ID=MALK_02383;prediction_source=braker_MRET:g2579.t1
contig009	AUGUSTUS	CDS	297811	298114	0.65	-	0	ID=MALK_02383.t1.c2;Parent=MALK_02383.t1
contig009	AUGUSTUS	CDS	297582	297670	0.65	-	0	ID=MALK_02383.t1.c1;Parent=MALK_02383.t1
contig009	AUGUSTUS	mRNA	297582	298114	0.64	-	.	ID=MALK_02383.t1;Parent=MALK_02383
contig009	AUGUSTUS	exon	297811	298114	.	-	.	ID=MALK_02383.t1.e2;Parent=MALK_02383.t1
contig009	AUGUSTUS	exon	297582	297670	.	-	.	ID=MALK_02383.t1.e1;Parent=MALK_02383.t1
contig009	AUGUSTUS	gene	298302	298931	0.21	+	.	ID=MALK_02384;prediction_source=braker_MRET:g2580.t1
contig009	AUGUSTUS	CDS	298302	298415	0.93	+	0	ID=MALK_02384.t1.c1;Parent=MALK_02384.t1
contig009	AUGUSTUS	CDS	298517	298717	0.93	+	0	ID=MALK_02384.t1.c2;Parent=MALK_02384.t1
contig009	AUGUSTUS	CDS	298759	298867	0.93	+	0	ID=MALK_02384.t1.c3;Parent=MALK_02384.t1

contig009	AUGUSTUS	CDS	298915	298931	0.93	+	0	ID=MALK_02384.t1.c4;Parent=MALK_02384.t1
contig009	AUGUSTUS	mRNA	298302	298931	0.21	+	.	ID=MALK_02384.t1;Parent=MALK_02384
contig009	AUGUSTUS	exon	298302	298415	.	+	.	ID=MALK_02384.t1.e1;Parent=MALK_02384.t1
contig009	AUGUSTUS	exon	298517	298717	.	+	.	ID=MALK_02384.t1.e2;Parent=MALK_02384.t1
contig009	AUGUSTUS	exon	298759	298867	.	+	.	ID=MALK_02384.t1.e3;Parent=MALK_02384.t1
contig009	AUGUSTUS	exon	298915	298931	.	+	.	ID=MALK_02384.t1.e4;Parent=MALK_02384.t1
contig009	AUGUSTUS	gene	298934	301393	0.89	-	.	ID=MALK_02385;prediction_source=braker_MRET:g2581.t1
contig009	AUGUSTUS	CDS	298934	301393	0.89	-	0	ID=MALK_02385.t1.c1;Parent=MALK_02385.t1
contig009	AUGUSTUS	mRNA	298934	301393	0.89	-	.	ID=MALK_02385.t1;Parent=MALK_02385
contig009	AUGUSTUS	exon	298934	301393	.	-	.	ID=MALK_02385.t1.e1;Parent=MALK_02385.t1
contig009	AUGUSTUS	gene	302183	302713	0.77	-	.	ID=MALK_02386;prediction_source=augustus:contig009.g2606.t1
contig009	AUGUSTUS	CDS	302183	302713	0.77	-	0	ID=MALK_02386.t1.c1;Parent=MALK_02386.t1
contig009	AUGUSTUS	mRNA	302183	302713	0.77	-	.	ID=MALK_02386.t1;Parent=MALK_02386
contig009	AUGUSTUS	exon	302183	302713	0.77	-	.	ID=MALK_02386.t1.e1;Parent=MALK_02386.t1
contig009	maker	gene	302946	303608	.	+	.	ID=MALK_02387;prediction_source=maker_MRET:augustus_masked-contig009-processed-gene-3.52-mRNA-1
contig009	maker	CDS	302946	303608	.	+	0	ID=MALK_02387.t1.c1;Parent=MALK_02387.t1
contig009	maker	mRNA	302946	303608	.	+	.	ID=MALK_02387.t1;Parent=MALK_02387
contig009	maker	exon	302946	303608	.	+	.	ID=MALK_02387.t1.e1;Parent=MALK_02387.t1
contig009	maker	gene	303617	305101	.	-	.	ID=MALK_02388;prediction_source=maker_MRET:augustus_masked-contig009-processed-gene-3.71-mRNA-1
contig009	maker	CDS	303617	305101	.	-	0	ID=MALK_02388.t1.c1;Parent=MALK_02388.t1
contig009	maker	mRNA	303617	305101	.	-	.	ID=MALK_02388.t1;Parent=MALK_02388
contig009	maker	exon	303617	305101	.	-	.	ID=MALK_02388.t1.e1;Parent=MALK_02388.t1
contig009	maker	gene	305239	307899	.	-	.	ID=MALK_02389;prediction_source=maker_MRET:augustus_masked-contig009-processed-gene-3.72-mRNA-1
contig009	maker	CDS	305239	307899	.	-	0	ID=MALK_02389.t1.c1;Parent=MALK_02389.t1
contig009	maker	mRNA	305239	307899	.	-	.	ID=MALK_02389.t1;Parent=MALK_02389
contig009	maker	exon	305239	307899	.	-	.	ID=MALK_02389.t1.e1;Parent=MALK_02389.t1
contig009	AUGUSTUS	gene	308231	308603	0.93	+	.	ID=MALK_02390;prediction_source=braker_MRET:g2586.t1
contig009	AUGUSTUS	CDS	308231	308271	0.97	+	0	ID=MALK_02390.t1.c1;Parent=MALK_02390.t1
contig009	AUGUSTUS	CDS	308315	308603	0.97	+	0	ID=MALK_02390.t1.c2;Parent=MALK_02390.t1
contig009	AUGUSTUS	mRNA	308231	308603	0.93	+	.	ID=MALK_02390.t1;Parent=MALK_02390
contig009	AUGUSTUS	exon	308231	308271	.	+	.	ID=MALK_02390.t1.e1;Parent=MALK_02390.t1
contig009	AUGUSTUS	exon	308315	308603	.	+	.	ID=MALK_02390.t1.e2;Parent=MALK_02390.t1
contig009	maker	gene	308647	310464	.	-	.	ID=MALK_02391;prediction_source=maker_MRET:augustus_masked-contig009-processed-gene-3.73-mRNA-1
contig009	maker	CDS	308647	310464	.	-	0	ID=MALK_02391.t1.c1;Parent=MALK_02391.t1
contig009	maker	mRNA	308647	310464	.	-	.	ID=MALK_02391.t1;Parent=MALK_02391
contig009	maker	exon	308647	310464	.	-	.	ID=MALK_02391.t1.e1;Parent=MALK_02391.t1
contig009	maker	gene	310639	311742	.	+	.	ID=MALK_02392;prediction_source=maker_MRET:augustus_masked-contig009-processed-gene-3.53-mRNA-1
contig009	maker	CDS	310639	311742	.	+	0	ID=MALK_02392.t1.c1;Parent=MALK_02392.t1
contig009	maker	mRNA	310639	311742	.	+	.	ID=MALK_02392.t1;Parent=MALK_02392
contig009	maker	exon	310639	311742	.	+	.	ID=MALK_02392.t1.e1;Parent=MALK_02392.t1
contig009	AUGUSTUS	gene	311792	312904	0.99	-	.	ID=MALK_02393;prediction_source=braker_MRET:g2589.t1
contig009	AUGUSTUS	CDS	311792	312904	0.99	-	0	ID=MALK_02393.t1.c1;Parent=MALK_02393.t1
contig009	AUGUSTUS	mRNA	311792	312904	0.99	-	.	ID=MALK_02393.t1;Parent=MALK_02393

contig009	AUGUSTUS	exon	311792	312904	.	-	.	ID=MALK_02393.t1.e1;Parent=MALK_02393.t1
contig009	AUGUSTUS	gene	312920	313939	0.99	+	.	ID=MALK_02394;prediction_source=augustus:contig009.g2612.t1
contig009	AUGUSTUS	CDS	312920	313939	0.99	+	0	ID=MALK_02394.t1.c1;Parent=MALK_02394.t1
contig009	AUGUSTUS	mRNA	312920	313939	0.99	+	.	ID=MALK_02394.t1;Parent=MALK_02394
contig009	AUGUSTUS	exon	312920	313939	0.99	+	.	ID=MALK_02394.t1.e1;Parent=MALK_02394.t1
contig009	maker	gene	313905	315320	.	-	.	ID=MALK_02395;prediction_source=maker_MRET:augustus_masked-contig009-processed-gene-3.75-mRNA-1
contig009	maker	CDS	313905	315320	.	-	0	ID=MALK_02395.t1.c1;Parent=MALK_02395.t1
contig009	maker	mRNA	313905	315320	.	-	.	ID=MALK_02395.t1;Parent=MALK_02395
contig009	maker	exon	313905	315320	.	-	.	ID=MALK_02395.t1.e1;Parent=MALK_02395.t1
contig009	AUGUSTUS	gene	315389	317371	0.88	-	.	ID=MALK_02396;prediction_source=augustus:contig009.g2614.t1
contig009	AUGUSTUS	CDS	315389	317371	0.88	-	0	ID=MALK_02396.t1.c1;Parent=MALK_02396.t1
contig009	AUGUSTUS	mRNA	315389	317371	0.88	-	.	ID=MALK_02396.t1;Parent=MALK_02396
contig009	AUGUSTUS	exon	315389	317371	0.88	-	.	ID=MALK_02396.t1.e1;Parent=MALK_02396.t1
contig009	AUGUSTUS	gene	317478	318620	0.42	-	.	ID=MALK_02397;prediction_source=augustus:contig009.g2615.t1
contig009	AUGUSTUS	CDS	317478	318620	0.42	-	0	ID=MALK_02397.t1.c1;Parent=MALK_02397.t1
contig009	AUGUSTUS	mRNA	317478	318620	0.42	-	.	ID=MALK_02397.t1;Parent=MALK_02397
contig009	AUGUSTUS	exon	317478	318620	0.42	-	.	ID=MALK_02397.t1.e1;Parent=MALK_02397.t1
contig009	maker	gene	318738	320366	.	-	.	ID=MALK_02398;prediction_source=maker_MRET:augustus_masked-contig009-processed-gene-3.78-mRNA-1
contig009	maker	CDS	318738	320366	.	-	0	ID=MALK_02398.t1.c1;Parent=MALK_02398.t1
contig009	maker	mRNA	318738	320366	.	-	.	ID=MALK_02398.t1;Parent=MALK_02398
contig009	maker	exon	318738	320366	.	-	.	ID=MALK_02398.t1.e1;Parent=MALK_02398.t1
contig009	maker	gene	320570	321472	.	+	.	ID=MALK_02399;prediction_source=maker_MRET:augustus_masked-contig009-processed-gene-3.54-mRNA-1
contig009	maker	CDS	320570	321472	.	+	0	ID=MALK_02399.t1.c1;Parent=MALK_02399.t1
contig009	maker	mRNA	320570	321472	.	+	.	ID=MALK_02399.t1;Parent=MALK_02399
contig009	maker	exon	320570	321472	.	+	.	ID=MALK_02399.t1.e1;Parent=MALK_02399.t1
contig009	maker	gene	322240	323910	.	-	.	ID=MALK_02400;prediction_source=maker_MRET:augustus_masked-contig009-processed-gene-3.79-mRNA-1
contig009	maker	CDS	322240	323910	.	-	0	ID=MALK_02400.t1.c1;Parent=MALK_02400.t1
contig009	maker	mRNA	322240	323910	.	-	.	ID=MALK_02400.t1;Parent=MALK_02400
contig009	maker	exon	322240	323910	.	-	.	ID=MALK_02400.t1.e1;Parent=MALK_02400.t1
contig009	AUGUSTUS	gene	324004	325482	0.98	+	.	ID=MALK_02401;prediction_source=augustus:contig009.g2620.t1
contig009	AUGUSTUS	CDS	324004	325482	0.98	+	0	ID=MALK_02401.t1.c1;Parent=MALK_02401.t1
contig009	AUGUSTUS	mRNA	324004	325482	0.98	+	.	ID=MALK_02401.t1;Parent=MALK_02401
contig009	AUGUSTUS	exon	324004	325482	0.98	+	.	ID=MALK_02401.t1.e1;Parent=MALK_02401.t1
contig009	maker	gene	325479	326924	.	-	.	ID=MALK_02402;prediction_source=maker_MRET:augustus_masked-contig009-processed-gene-3.80-mRNA-1
contig009	maker	CDS	325479	326924	.	-	0	ID=MALK_02402.t1.c1;Parent=MALK_02402.t1
contig009	maker	mRNA	325479	326924	.	-	.	ID=MALK_02402.t1;Parent=MALK_02402
contig009	maker	exon	325479	326924	.	-	.	ID=MALK_02402.t1.e1;Parent=MALK_02402.t1
contig009	AUGUSTUS	gene	326969	328801	0.92	-	.	ID=MALK_02403;prediction_source=augustus:contig009.g2622.t1
contig009	AUGUSTUS	CDS	326969	328801	0.92	-	0	ID=MALK_02403.t1.c1;Parent=MALK_02403.t1
contig009	AUGUSTUS	mRNA	326969	328801	0.92	-	.	ID=MALK_02403.t1;Parent=MALK_02403
contig009	AUGUSTUS	exon	326969	328801	0.92	-	.	ID=MALK_02403.t1.e1;Parent=MALK_02403.t1
contig009	AUGUSTUS	gene	328872	330908	0.93	+	.	ID=MALK_02404;prediction_source=braker_MRET:g2600.t1
contig009	AUGUSTUS	CDS	328872	330908	0.93	+	0	ID=MALK_02404.t1.c1;Parent=MALK_02404.t1

contig009	AUGUSTUS	mRNA	328872	330908	0.93	+	.	ID=MALK_02404.t1;Parent=MALK_02404
contig009	AUGUSTUS	exon	328872	330908	.	+	.	ID=MALK_02404.t1.e1;Parent=MALK_02404.t1
contig009	AUGUSTUS	gene	330905	331558	0.97	-	.	ID=MALK_02405;prediction_source=augustus:contig009.g2624.t1
contig009	AUGUSTUS	CDS	330905	331558	0.97	-	0	ID=MALK_02405.t1.c1;Parent=MALK_02405.t1
contig009	AUGUSTUS	mRNA	330905	331558	0.97	-	.	ID=MALK_02405.t1;Parent=MALK_02405
contig009	AUGUSTUS	exon	330905	331558	0.97	-	.	ID=MALK_02405.t1.e1;Parent=MALK_02405.t1
contig009	maker	gene	331671	333629	.	+	.	ID=MALK_02406;prediction_source=maker_MRET:augustus_masked-contig009-processed-gene-3.57-mRNA-1
contig009	maker	CDS	331671	333629	.	+	0	ID=MALK_02406.t1.c1;Parent=MALK_02406.t1
contig009	maker	mRNA	331671	333629	.	+	.	ID=MALK_02406.t1;Parent=MALK_02406
contig009	maker	exon	331671	333629	.	+	.	ID=MALK_02406.t1.e1;Parent=MALK_02406.t1
contig009	AUGUSTUS	gene	333796	336009	0.41	+	.	ID=MALK_02407;prediction_source=augustus:contig009.g2627.t1
contig009	AUGUSTUS	CDS	333796	336009	0.41	+	0	ID=MALK_02407.t1.c1;Parent=MALK_02407.t1
contig009	AUGUSTUS	mRNA	333796	336009	0.41	+	.	ID=MALK_02407.t1;Parent=MALK_02407
contig009	AUGUSTUS	exon	333796	336009	0.41	+	.	ID=MALK_02407.t1.e1;Parent=MALK_02407.t1
contig009	AUGUSTUS	gene	336029	337522	0.89	-	.	ID=MALK_02408;prediction_source=braker_MRET:g2604.t1
contig009	AUGUSTUS	CDS	337313	337522	0.95	-	0	ID=MALK_02408.t1.c3;Parent=MALK_02408.t1
contig009	AUGUSTUS	CDS	337222	337284	0.95	-	0	ID=MALK_02408.t1.c2;Parent=MALK_02408.t1
contig009	AUGUSTUS	CDS	336029	337192	0.95	-	0	ID=MALK_02408.t1.c1;Parent=MALK_02408.t1
contig009	AUGUSTUS	mRNA	336029	337522	0.89	-	.	ID=MALK_02408.t1;Parent=MALK_02408
contig009	AUGUSTUS	exon	337313	337522	.	-	.	ID=MALK_02408.t1.e3;Parent=MALK_02408.t1
contig009	AUGUSTUS	exon	337222	337284	.	-	.	ID=MALK_02408.t1.e2;Parent=MALK_02408.t1
contig009	AUGUSTUS	exon	336029	337192	.	-	.	ID=MALK_02408.t1.e1;Parent=MALK_02408.t1
contig009	AUGUSTUS	gene	337671	338741	0.95	+	.	ID=MALK_02409;prediction_source=augustus:contig009.g2629.t1
contig009	AUGUSTUS	CDS	337671	338741	0.95	+	0	ID=MALK_02409.t1.c1;Parent=MALK_02409.t1
contig009	AUGUSTUS	mRNA	337671	338741	0.95	+	.	ID=MALK_02409.t1;Parent=MALK_02409
contig009	AUGUSTUS	exon	337671	338741	0.95	+	.	ID=MALK_02409.t1.e1;Parent=MALK_02409.t1
contig009	maker	gene	338873	339544	.	+	.	ID=MALK_02410;prediction_source=maker_MRET:augustus_masked-contig009-processed-gene-3.60-mRNA-1
contig009	maker	CDS	338873	339544	.	+	0	ID=MALK_02410.t1.c1;Parent=MALK_02410.t1
contig009	maker	mRNA	338873	339544	.	+	.	ID=MALK_02410.t1;Parent=MALK_02410
contig009	maker	exon	338873	339544	.	+	.	ID=MALK_02410.t1.e1;Parent=MALK_02410.t1
contig009	maker	gene	339579	340268	.	+	.	ID=MALK_02411;prediction_source=maker_MRET:augustus_masked-contig009-processed-gene-3.61-mRNA-1
contig009	maker	CDS	339579	340268	.	+	0	ID=MALK_02411.t1.c1;Parent=MALK_02411.t1
contig009	maker	mRNA	339579	340268	.	+	.	ID=MALK_02411.t1;Parent=MALK_02411
contig009	maker	exon	339579	340268	.	+	.	ID=MALK_02411.t1.e1;Parent=MALK_02411.t1
contig009	AUGUSTUS	gene	340611	344990	0.4	+	.	ID=MALK_02412;prediction_source=augustus:contig009.g2632.t1
contig009	AUGUSTUS	CDS	340611	344990	0.4	+	0	ID=MALK_02412.t1.c1;Parent=MALK_02412.t1
contig009	AUGUSTUS	mRNA	340611	344990	0.4	+	.	ID=MALK_02412.t1;Parent=MALK_02412
contig009	AUGUSTUS	exon	340611	344990	0.4	+	.	ID=MALK_02412.t1.e1;Parent=MALK_02412.t1
contig009	AUGUSTUS	gene	345042	348635	1	-	.	ID=MALK_02413;prediction_source=augustus:contig009.g2633.t1
contig009	AUGUSTUS	CDS	345042	348635	1	-	0	ID=MALK_02413.t1.c1;Parent=MALK_02413.t1
contig009	AUGUSTUS	mRNA	345042	348635	1	-	.	ID=MALK_02413.t1;Parent=MALK_02413
contig009	AUGUSTUS	exon	345042	348635	1	-	.	ID=MALK_02413.t1.e1;Parent=MALK_02413.t1
contig009	maker	gene	348901	349887	.	+	.	ID=MALK_02414;prediction_source=maker_MRET:augustus_masked-contig009-processed-gene-3.63-mRNA-1

contig009	maker	CDS	348901	349887	.	+	0	ID=MALK_02414.t1.c1;Parent=MALK_02414.t1
contig009	maker	mRNA	348901	349887	.	+	.	ID=MALK_02414.t1;Parent=MALK_02414
contig009	maker	exon	348901	349887	.	+	.	ID=MALK_02414.t1.e1;Parent=MALK_02414.t1
contig009	maker	gene	349913	351490	.	-	.	ID=MALK_02415;prediction_source=maker_MRET:augustus_masked-contig009-processed-gene-3.84-mRNA-1
contig009	maker	CDS	349913	351490	.	-	0	ID=MALK_02415.t1.c1;Parent=MALK_02415.t1
contig009	maker	mRNA	349913	351490	.	-	.	ID=MALK_02415.t1;Parent=MALK_02415
contig009	maker	exon	349913	351490	.	-	.	ID=MALK_02415.t1.e1;Parent=MALK_02415.t1
contig009	AUGUSTUS	gene	351745	352890	0.38	-	.	ID=MALK_02416;prediction_source=augustus:contig009.g2636.t1
contig009	AUGUSTUS	CDS	351745	352890	0.38	-	0	ID=MALK_02416.t1.c1;Parent=MALK_02416.t1
contig009	AUGUSTUS	mRNA	351745	352890	0.38	-	.	ID=MALK_02416.t1;Parent=MALK_02416
contig009	AUGUSTUS	exon	351745	352890	0.38	-	.	ID=MALK_02416.t1.e1;Parent=MALK_02416.t1
contig009	maker	gene	353007	355427	.	-	.	ID=MALK_02417;prediction_source=maker_MRET:augustus_masked-contig009-processed-gene-3.86-mRNA-1
contig009	maker	CDS	353007	355427	.	-	0	ID=MALK_02417.t1.c1;Parent=MALK_02417.t1
contig009	maker	mRNA	353007	355427	.	-	.	ID=MALK_02417.t1;Parent=MALK_02417
contig009	maker	exon	353007	355427	.	-	.	ID=MALK_02417.t1.e1;Parent=MALK_02417.t1
contig009	maker	gene	355709	356920	.	-	.	ID=MALK_02418;prediction_source=maker_MRET:augustus_masked-contig009-processed-gene-3.87-mRNA-1
contig009	maker	CDS	355709	356920	.	-	0	ID=MALK_02418.t1.c1;Parent=MALK_02418.t1
contig009	maker	mRNA	355709	356920	.	-	.	ID=MALK_02418.t1;Parent=MALK_02418
contig009	maker	exon	355709	356920	.	-	.	ID=MALK_02418.t1.e1;Parent=MALK_02418.t1
contig009	AUGUSTUS	gene	357086	361753	0.65	+	.	ID=MALK_02419;prediction_source=augustus:contig009.g2640.t1
contig009	AUGUSTUS	CDS	357086	361753	0.65	+	0	ID=MALK_02419.t1.c1;Parent=MALK_02419.t1
contig009	AUGUSTUS	mRNA	357086	361753	0.65	+	.	ID=MALK_02419.t1;Parent=MALK_02419
contig009	AUGUSTUS	exon	357086	361753	0.65	+	.	ID=MALK_02419.t1.e1;Parent=MALK_02419.t1
contig009	AUGUSTUS	gene	361750	363411	0.54	-	.	ID=MALK_02420;prediction_source=braker_MRET:g2614.t1
contig009	AUGUSTUS	CDS	361750	363411	0.54	-	0	ID=MALK_02420.t1.c1;Parent=MALK_02420.t1
contig009	AUGUSTUS	mRNA	361750	363411	0.54	-	.	ID=MALK_02420.t1;Parent=MALK_02420
contig009	AUGUSTUS	exon	361750	363411	.	-	.	ID=MALK_02420.t1.e1;Parent=MALK_02420.t1
contig009	AUGUSTUS	gene	363504	364565	0.7	-	.	ID=MALK_02421;prediction_source=augustus:contig009.g2643.t1
contig009	AUGUSTUS	CDS	363504	364565	0.7	-	0	ID=MALK_02421.t1.c1;Parent=MALK_02421.t1
contig009	AUGUSTUS	mRNA	363504	364565	0.7	-	.	ID=MALK_02421.t1;Parent=MALK_02421
contig009	AUGUSTUS	exon	363504	364565	0.7	-	.	ID=MALK_02421.t1.e1;Parent=MALK_02421.t1
contig009	maker	gene	364864	365307	.	+	.	ID=MALK_02422;prediction_source=maker_MRET:augustus_masked-contig009-processed-gene-3.65-mRNA-1
contig009	maker	CDS	364864	365307	.	+	0	ID=MALK_02422.t1.c1;Parent=MALK_02422.t1
contig009	maker	mRNA	364864	365307	.	+	.	ID=MALK_02422.t1;Parent=MALK_02422
contig009	maker	exon	364864	365307	.	+	.	ID=MALK_02422.t1.e1;Parent=MALK_02422.t1
contig009	AUGUSTUS	gene	365612	367243	0.92	-	.	ID=MALK_02423;prediction_source=augustus:contig009.g2645.t1
contig009	AUGUSTUS	CDS	365612	367243	0.92	-	0	ID=MALK_02423.t1.c1;Parent=MALK_02423.t1
contig009	AUGUSTUS	mRNA	365612	367243	0.92	-	.	ID=MALK_02423.t1;Parent=MALK_02423
contig009	AUGUSTUS	exon	365612	367243	0.92	-	.	ID=MALK_02423.t1.e1;Parent=MALK_02423.t1
contig009	maker	gene	367448	368206	.	+	.	ID=MALK_02424;prediction_source=maker_MRET:augustus_masked-contig009-processed-gene-3.66-mRNA-1
contig009	maker	CDS	367448	368206	.	+	0	ID=MALK_02424.t1.c1;Parent=MALK_02424.t1
contig009	maker	mRNA	367448	368206	.	+	.	ID=MALK_02424.t1;Parent=MALK_02424
contig009	maker	exon	367448	368206	.	+	.	ID=MALK_02424.t1.e1;Parent=MALK_02424.t1

contig009	maker	gene	368436	369377	.	-	.	ID=MALK_02425;prediction_source=maker_MRET:augustus_masked-contig009-processed-gene-3.91-mRNA-1
contig009	maker	CDS	368436	369377	.	-	0	ID=MALK_02425.t1.c1;Parent=MALK_02425.t1
contig009	maker	mRNA	368436	369377	.	-	.	ID=MALK_02425.t1;Parent=MALK_02425
contig009	maker	exon	368436	369377	.	-	.	ID=MALK_02425.t1.e1;Parent=MALK_02425.t1
contig009	maker	gene	369738	370997	.	-	.	ID=MALK_02426;prediction_source=maker_MRET:augustus_masked-contig009-processed-gene-3.92-mRNA-1
contig009	maker	CDS	369738	370997	.	-	0	ID=MALK_02426.t1.c1;Parent=MALK_02426.t1
contig009	maker	mRNA	369738	370997	.	-	.	ID=MALK_02426.t1;Parent=MALK_02426
contig009	maker	exon	369738	370997	.	-	.	ID=MALK_02426.t1.e1;Parent=MALK_02426.t1
contig009	AUGUSTUS	gene	371139	371684	0.83	+	.	ID=MALK_02427;prediction_source=braker_MRET:g2621.t1
contig009	AUGUSTUS	CDS	371139	371322	0.93	+	0	ID=MALK_02427.t1.c1;Parent=MALK_02427.t1
contig009	AUGUSTUS	CDS	371356	371684	0.93	+	0	ID=MALK_02427.t1.c2;Parent=MALK_02427.t1
contig009	AUGUSTUS	mRNA	371139	371684	0.83	+	.	ID=MALK_02427.t1;Parent=MALK_02427
contig009	AUGUSTUS	exon	371139	371322	.	+	.	ID=MALK_02427.t1.e1;Parent=MALK_02427.t1
contig009	AUGUSTUS	exon	371356	371684	.	+	.	ID=MALK_02427.t1.e2;Parent=MALK_02427.t1
contig009	maker	gene	371799	374513	.	-	.	ID=MALK_02428;prediction_source=maker_MRET:augustus_masked-contig009-processed-gene-3.93-mRNA-1
contig009	maker	CDS	371799	374513	.	-	0	ID=MALK_02428.t1.c1;Parent=MALK_02428.t1
contig009	maker	mRNA	371799	374513	.	-	.	ID=MALK_02428.t1;Parent=MALK_02428
contig009	maker	exon	371799	374513	.	-	.	ID=MALK_02428.t1.e1;Parent=MALK_02428.t1
contig009	AUGUSTUS	gene	374827	377580	0.68	-	.	ID=MALK_02429;prediction_source=augustus:contig009.g2653.t1
contig009	AUGUSTUS	CDS	374827	377580	0.68	-	0	ID=MALK_02429.t1.c1;Parent=MALK_02429.t1
contig009	AUGUSTUS	mRNA	374827	377580	0.68	-	.	ID=MALK_02429.t1;Parent=MALK_02429
contig009	AUGUSTUS	exon	374827	377580	0.68	-	.	ID=MALK_02429.t1.e1;Parent=MALK_02429.t1
contig009	AUGUSTUS	gene	377639	378136	0.99	-	.	ID=MALK_02430;prediction_source=braker_MRET:g2624.t1
contig009	AUGUSTUS	CDS	377639	378136	0.99	-	0	ID=MALK_02430.t1.c1;Parent=MALK_02430.t1
contig009	AUGUSTUS	mRNA	377639	378136	0.99	-	.	ID=MALK_02430.t1;Parent=MALK_02430
contig009	AUGUSTUS	exon	377639	378136	.	-	.	ID=MALK_02430.t1.e1;Parent=MALK_02430.t1
contig009	AUGUSTUS	gene	378177	378968	1	+	.	ID=MALK_02431;prediction_source=braker_MRET:g2625.t1
contig009	AUGUSTUS	CDS	378177	378968	1	+	0	ID=MALK_02431.t1.c1;Parent=MALK_02431.t1
contig009	AUGUSTUS	mRNA	378177	378968	1	+	.	ID=MALK_02431.t1;Parent=MALK_02431
contig009	AUGUSTUS	exon	378177	378968	.	+	.	ID=MALK_02431.t1.e1;Parent=MALK_02431.t1
contig009	AUGUSTUS	gene	378979	380098	0.44	-	.	ID=MALK_02432;prediction_source=braker_MRET:g2626.t1
contig009	AUGUSTUS	CDS	379016	380098	0.46	-	0	ID=MALK_02432.t1.c2;Parent=MALK_02432.t1
contig009	AUGUSTUS	CDS	378979	378987	0.46	-	0	ID=MALK_02432.t1.c1;Parent=MALK_02432.t1
contig009	AUGUSTUS	mRNA	378979	380098	0.44	-	.	ID=MALK_02432.t1;Parent=MALK_02432
contig009	AUGUSTUS	exon	379016	380098	.	-	.	ID=MALK_02432.t1.e2;Parent=MALK_02432.t1
contig009	AUGUSTUS	exon	378979	378987	.	-	.	ID=MALK_02432.t1.e1;Parent=MALK_02432.t1
contig009	AUGUSTUS	gene	380156	382795	0.76	+	.	ID=MALK_02433;prediction_source=braker_MRET:g2627.t1
contig009	AUGUSTUS	CDS	380156	382795	0.76	+	0	ID=MALK_02433.t1.c1;Parent=MALK_02433.t1
contig009	AUGUSTUS	mRNA	380156	382795	0.76	+	.	ID=MALK_02433.t1;Parent=MALK_02433
contig009	AUGUSTUS	exon	380156	382795	.	+	.	ID=MALK_02433.t1.e1;Parent=MALK_02433.t1
contig009	AUGUSTUS	gene	382801	386880	0.74	-	.	ID=MALK_02434;prediction_source=augustus:contig009.g2657.t1
contig009	AUGUSTUS	CDS	382801	386880	0.74	-	0	ID=MALK_02434.t1.c1;Parent=MALK_02434.t1
contig009	AUGUSTUS	mRNA	382801	386880	0.74	-	.	ID=MALK_02434.t1;Parent=MALK_02434

contig009	AUGUSTUS	exon	382801	386880	0.74	-	.	ID=MALK_02434.t1.e1;Parent=MALK_02434.t1
contig009	AUGUSTUS	gene	387095	388429	1	+	.	ID=MALK_02435;prediction_source=augustus:contig009.g2658.t1
contig009	AUGUSTUS	CDS	387095	388429	1	+	0	ID=MALK_02435.t1.c1;Parent=MALK_02435.t1
contig009	AUGUSTUS	mRNA	387095	388429	1	+	.	ID=MALK_02435.t1;Parent=MALK_02435
contig009	AUGUSTUS	exon	387095	388429	1	+	.	ID=MALK_02435.t1.e1;Parent=MALK_02435.t1
contig009	maker	gene	388447	388791	.	-	.	ID=MALK_02436;prediction_source=maker_MRET:augustus_masked-contig009-processed-gene-3.97-mRNA-1
contig009	maker	CDS	388447	388791	.	-	0	ID=MALK_02436.t1.c1;Parent=MALK_02436.t1
contig009	maker	mRNA	388447	388791	.	-	.	ID=MALK_02436.t1;Parent=MALK_02436
contig009	maker	exon	388447	388791	.	-	.	ID=MALK_02436.t1.e1;Parent=MALK_02436.t1
contig009	AUGUSTUS	gene	389296	393780	0.99	+	.	ID=MALK_02437;prediction_source=augustus:contig009.g2660.t1
contig009	AUGUSTUS	CDS	389296	393780	0.99	+	0	ID=MALK_02437.t1.c1;Parent=MALK_02437.t1
contig009	AUGUSTUS	mRNA	389296	393780	0.99	+	.	ID=MALK_02437.t1;Parent=MALK_02437
contig009	AUGUSTUS	exon	389296	393780	0.99	+	.	ID=MALK_02437.t1.e1;Parent=MALK_02437.t1
contig009	maker	gene	394033	396888	.	+	.	ID=MALK_02438;prediction_source=maker_MRET:augustus_masked-contig009-processed-gene-4.1-mRNA-1
contig009	maker	CDS	394033	396888	.	+	0	ID=MALK_02438.t1.c1;Parent=MALK_02438.t1
contig009	maker	mRNA	394033	396888	.	+	.	ID=MALK_02438.t1;Parent=MALK_02438
contig009	maker	exon	394033	396888	.	+	.	ID=MALK_02438.t1.e1;Parent=MALK_02438.t1
contig009	AUGUSTUS	gene	396968	397978	0.94	-	.	ID=MALK_02439;prediction_source=augustus:contig009.g2664.t1
contig009	AUGUSTUS	CDS	396968	397978	0.94	-	0	ID=MALK_02439.t1.c1;Parent=MALK_02439.t1
contig009	AUGUSTUS	mRNA	396968	397978	0.94	-	.	ID=MALK_02439.t1;Parent=MALK_02439
contig009	AUGUSTUS	exon	396968	397978	0.94	-	.	ID=MALK_02439.t1.e1;Parent=MALK_02439.t1
contig009	maker	gene	398439	402176	.	-	.	ID=MALK_02440;prediction_source=maker_MRET:augustus_masked-contig009-processed-gene-4.3-mRNA-1
contig009	maker	CDS	398439	402176	.	-	0	ID=MALK_02440.t1.c1;Parent=MALK_02440.t1
contig009	maker	mRNA	398439	402176	.	-	.	ID=MALK_02440.t1;Parent=MALK_02440
contig009	maker	exon	398439	402176	.	-	.	ID=MALK_02440.t1.e1;Parent=MALK_02440.t1
contig009	AUGUSTUS	gene	402622	404988	0.91	-	.	ID=MALK_02441;prediction_source=augustus:contig009.g2666.t1
contig009	AUGUSTUS	CDS	402622	404988	0.91	-	0	ID=MALK_02441.t1.c1;Parent=MALK_02441.t1
contig009	AUGUSTUS	mRNA	402622	404988	0.91	-	.	ID=MALK_02441.t1;Parent=MALK_02441
contig009	AUGUSTUS	exon	402622	404988	0.91	-	.	ID=MALK_02441.t1.e1;Parent=MALK_02441.t1
contig009	maker	gene	405152	408295	.	+	.	ID=MALK_02442;prediction_source=maker_MRET:augustus_masked-contig009-processed-gene-4.4-mRNA-1
contig009	maker	CDS	405152	408295	.	+	0	ID=MALK_02442.t1.c1;Parent=MALK_02442.t1
contig009	maker	mRNA	405152	408295	.	+	.	ID=MALK_02442.t1;Parent=MALK_02442
contig009	maker	exon	405152	408295	.	+	.	ID=MALK_02442.t1.e1;Parent=MALK_02442.t1
contig009	maker	gene	408390	409730	.	-	.	ID=MALK_02443;prediction_source=maker_MRET:augustus_masked-contig009-processed-gene-4.24-mRNA-1
contig009	maker	CDS	408390	409730	.	-	0	ID=MALK_02443.t1.c1;Parent=MALK_02443.t1
contig009	maker	mRNA	408390	409730	.	-	.	ID=MALK_02443.t1;Parent=MALK_02443
contig009	maker	exon	408390	409730	.	-	.	ID=MALK_02443.t1.e1;Parent=MALK_02443.t1
contig009	AUGUSTUS	gene	410844	411139	0.99	-	.	ID=MALK_02444;prediction_source=braker_MRET:g2638.t1
contig009	AUGUSTUS	CDS	411137	411139	0.99	-	0	ID=MALK_02444.t1.c2;Parent=MALK_02444.t1
contig009	AUGUSTUS	CDS	410844	411089	0.99	-	0	ID=MALK_02444.t1.c1;Parent=MALK_02444.t1
contig009	AUGUSTUS	mRNA	410844	411139	0.99	-	.	ID=MALK_02444.t1;Parent=MALK_02444
contig009	AUGUSTUS	exon	411137	411139	.	-	.	ID=MALK_02444.t1.e2;Parent=MALK_02444.t1
contig009	AUGUSTUS	exon	410844	411089	.	-	.	ID=MALK_02444.t1.e1;Parent=MALK_02444.t1

contig009	AUGUSTUS	gene	411346	412440	0.87	+	.	ID=MALK_02445;prediction_source=augustus:contig009.g2671.t1
contig009	AUGUSTUS	CDS	411346	412440	0.87	+	0	ID=MALK_02445.t1.c1;Parent=MALK_02445.t1
contig009	AUGUSTUS	mRNA	411346	412440	0.87	+	.	ID=MALK_02445.t1;Parent=MALK_02445
contig009	AUGUSTUS	exon	411346	412440	0.87	+	.	ID=MALK_02445.t1.e1;Parent=MALK_02445.t1
contig009	AUGUSTUS	gene	412429	415053	0.46	-	.	ID=MALK_02446;prediction_source=augustus:contig009.g2672.t1
contig009	AUGUSTUS	CDS	412429	415053	0.46	-	0	ID=MALK_02446.t1.c1;Parent=MALK_02446.t1
contig009	AUGUSTUS	mRNA	412429	415053	0.46	-	.	ID=MALK_02446.t1;Parent=MALK_02446
contig009	AUGUSTUS	exon	412429	415053	0.46	-	.	ID=MALK_02446.t1.e1;Parent=MALK_02446.t1
contig009	maker	gene	415314	417089	.	+	.	ID=MALK_02447;prediction_source=maker_MRET:augustus_masked-contig009-processed-gene-4.7-mRNA-1
contig009	maker	CDS	415314	417089	.	+	0	ID=MALK_02447.t1.c1;Parent=MALK_02447.t1
contig009	maker	mRNA	415314	417089	.	+	.	ID=MALK_02447.t1;Parent=MALK_02447
contig009	maker	exon	415314	417089	.	+	.	ID=MALK_02447.t1.e1;Parent=MALK_02447.t1
contig009	AUGUSTUS	gene	417496	417918	1	+	.	ID=MALK_02448;prediction_source=augustus:contig009.g2674.t1
contig009	AUGUSTUS	CDS	417496	417918	1	+	0	ID=MALK_02448.t1.c1;Parent=MALK_02448.t1
contig009	AUGUSTUS	mRNA	417496	417918	1	+	.	ID=MALK_02448.t1;Parent=MALK_02448
contig009	AUGUSTUS	exon	417496	417918	1	+	.	ID=MALK_02448.t1.e1;Parent=MALK_02448.t1
contig009	AUGUSTUS	gene	418330	419163	0.85	-	.	ID=MALK_02449;prediction_source=augustus:contig009.g2675.t1
contig009	AUGUSTUS	CDS	418330	419163	0.85	-	0	ID=MALK_02449.t1.c1;Parent=MALK_02449.t1
contig009	AUGUSTUS	mRNA	418330	419163	0.85	-	.	ID=MALK_02449.t1;Parent=MALK_02449
contig009	AUGUSTUS	exon	418330	419163	0.85	-	.	ID=MALK_02449.t1.e1;Parent=MALK_02449.t1
contig009	AUGUSTUS	gene	420225	421884	0.5	+	.	ID=MALK_02450;prediction_source=braker_MRET:g2644.t1
contig009	AUGUSTUS	CDS	420225	420293	1	+	0	ID=MALK_02450.t1.c1;Parent=MALK_02450.t1
contig009	AUGUSTUS	CDS	420334	420561	1	+	0	ID=MALK_02450.t1.c2;Parent=MALK_02450.t1
contig009	AUGUSTUS	CDS	420604	421884	1	+	0	ID=MALK_02450.t1.c3;Parent=MALK_02450.t1
contig009	AUGUSTUS	mRNA	420225	421884	0.5	+	.	ID=MALK_02450.t1;Parent=MALK_02450
contig009	AUGUSTUS	exon	420225	420293	.	+	.	ID=MALK_02450.t1.e1;Parent=MALK_02450.t1
contig009	AUGUSTUS	exon	420334	420561	.	+	.	ID=MALK_02450.t1.e2;Parent=MALK_02450.t1
contig009	AUGUSTUS	exon	420604	421884	.	+	.	ID=MALK_02450.t1.e3;Parent=MALK_02450.t1
contig009	maker	gene	423446	425113	.	-	.	ID=MALK_02451;prediction_source=maker_MRET:augustus_masked-contig009-processed-gene-4.27-mRNA-1
contig009	maker	CDS	423446	425113	.	-	0	ID=MALK_02451.t1.c1;Parent=MALK_02451.t1
contig009	maker	mRNA	423446	425113	.	-	.	ID=MALK_02451.t1;Parent=MALK_02451
contig009	maker	exon	423446	425113	.	-	.	ID=MALK_02451.t1.e1;Parent=MALK_02451.t1
contig009	AUGUSTUS	gene	425188	425742	0.53	-	.	ID=MALK_02452;prediction_source=braker_MRET:g2646.t1
contig009	AUGUSTUS	CDS	425188	425742	0.53	-	0	ID=MALK_02452.t1.c1;Parent=MALK_02452.t1
contig009	AUGUSTUS	mRNA	425188	425742	0.53	-	.	ID=MALK_02452.t1;Parent=MALK_02452
contig009	AUGUSTUS	exon	425188	425742	.	-	.	ID=MALK_02452.t1.e1;Parent=MALK_02452.t1
contig009	AUGUSTUS	gene	425838	426881	0.23	-	.	ID=MALK_02453;prediction_source=braker_MRET:g2647.t1
contig009	AUGUSTUS	CDS	426848	426881	0.45	-	0	ID=MALK_02453.t1.c2;Parent=MALK_02453.t1
contig009	AUGUSTUS	CDS	425838	426817	0.45	-	0	ID=MALK_02453.t1.c1;Parent=MALK_02453.t1
contig009	AUGUSTUS	mRNA	425838	426881	0.23	-	.	ID=MALK_02453.t1;Parent=MALK_02453
contig009	AUGUSTUS	exon	426848	426881	.	-	.	ID=MALK_02453.t1.e2;Parent=MALK_02453.t1
contig009	AUGUSTUS	exon	425838	426817	.	-	.	ID=MALK_02453.t1.e1;Parent=MALK_02453.t1
contig009	AUGUSTUS	gene	427242	428816	0.83	+	.	ID=MALK_02454;prediction_source=augustus:contig009.g2679.t1

contig009	AUGUSTUS	CDS	427242	428816	0.83	+	0	ID=MALK_02454.t1.c1;Parent=MALK_02454.t1
contig009	AUGUSTUS	mRNA	427242	428816	0.83	+	.	ID=MALK_02454.t1;Parent=MALK_02454
contig009	AUGUSTUS	exon	427242	428816	0.83	+	.	ID=MALK_02454.t1.e1;Parent=MALK_02454.t1
contig009	AUGUSTUS	gene	429061	430731	0.76	+	.	ID=MALK_02455;prediction_source=augustus:contig009.g2681.t1
contig009	AUGUSTUS	CDS	429061	430731	0.76	+	0	ID=MALK_02455.t1.c1;Parent=MALK_02455.t1
contig009	AUGUSTUS	mRNA	429061	430731	0.76	+	.	ID=MALK_02455.t1;Parent=MALK_02455
contig009	AUGUSTUS	exon	429061	430731	0.76	+	.	ID=MALK_02455.t1.e1;Parent=MALK_02455.t1
contig009	AUGUSTUS	gene	430770	435128	0.72	-	.	ID=MALK_02456;prediction_source=augustus:contig009.g2682.t1
contig009	AUGUSTUS	CDS	430770	435128	0.72	-	0	ID=MALK_02456.t1.c1;Parent=MALK_02456.t1
contig009	AUGUSTUS	mRNA	430770	435128	0.72	-	.	ID=MALK_02456.t1;Parent=MALK_02456
contig009	AUGUSTUS	exon	430770	435128	0.72	-	.	ID=MALK_02456.t1.e1;Parent=MALK_02456.t1
contig009	AUGUSTUS	gene	435357	437489	0.95	+	.	ID=MALK_02457;prediction_source=braker_MRET:g2651.t1
contig009	AUGUSTUS	CDS	435357	437489	0.95	+	0	ID=MALK_02457.t1.c1;Parent=MALK_02457.t1
contig009	AUGUSTUS	mRNA	435357	437489	0.95	+	.	ID=MALK_02457.t1;Parent=MALK_02457
contig009	AUGUSTUS	exon	435357	437489	.	+	.	ID=MALK_02457.t1.e1;Parent=MALK_02457.t1
contig009	AUGUSTUS	gene	437511	439053	0.83	+	.	ID=MALK_02458;prediction_source=braker_MRET:g2652.t1
contig009	AUGUSTUS	CDS	437511	438962	0.99	+	0	ID=MALK_02458.t1.c1;Parent=MALK_02458.t1
contig009	AUGUSTUS	CDS	439006	439053	0.99	+	0	ID=MALK_02458.t1.c2;Parent=MALK_02458.t1
contig009	AUGUSTUS	mRNA	437511	439053	0.83	+	.	ID=MALK_02458.t1;Parent=MALK_02458
contig009	AUGUSTUS	exon	437511	438962	.	+	.	ID=MALK_02458.t1.e1;Parent=MALK_02458.t1
contig009	AUGUSTUS	exon	439006	439053	.	+	.	ID=MALK_02458.t1.e2;Parent=MALK_02458.t1
contig009	AUGUSTUS	gene	439098	439966	0.6	-	.	ID=MALK_02459;prediction_source=braker_MRET:g2653.t1
contig009	AUGUSTUS	CDS	439505	439966	0.62	-	0	ID=MALK_02459.t1.c3;Parent=MALK_02459.t1
contig009	AUGUSTUS	CDS	439375	439419	0.62	-	0	ID=MALK_02459.t1.c2;Parent=MALK_02459.t1
contig009	AUGUSTUS	CDS	439098	439346	0.62	-	0	ID=MALK_02459.t1.c1;Parent=MALK_02459.t1
contig009	AUGUSTUS	mRNA	439098	439966	0.6	-	.	ID=MALK_02459.t1;Parent=MALK_02459
contig009	AUGUSTUS	exon	439505	439966	.	-	.	ID=MALK_02459.t1.e3;Parent=MALK_02459.t1
contig009	AUGUSTUS	exon	439375	439419	.	-	.	ID=MALK_02459.t1.e2;Parent=MALK_02459.t1
contig009	AUGUSTUS	exon	439098	439346	.	-	.	ID=MALK_02459.t1.e1;Parent=MALK_02459.t1
contig009	maker	gene	440131	441252	.	+	.	ID=MALK_02460;prediction_source=maker_MRET:augustus_masked-contig009-processed-gene-4.13-mRNA-1
contig009	maker	CDS	440131	441252	.	+	0	ID=MALK_02460.t1.c1;Parent=MALK_02460.t1
contig009	maker	mRNA	440131	441252	.	+	.	ID=MALK_02460.t1;Parent=MALK_02460
contig009	maker	exon	440131	441252	.	+	.	ID=MALK_02460.t1.e1;Parent=MALK_02460.t1
contig009	maker	gene	441256	442503	.	-	.	ID=MALK_02461;prediction_source=maker_MRET:augustus_masked-contig009-processed-gene-4.31-mRNA-1
contig009	maker	CDS	441256	442503	.	-	0	ID=MALK_02461.t1.c1;Parent=MALK_02461.t1
contig009	maker	mRNA	441256	442503	.	-	.	ID=MALK_02461.t1;Parent=MALK_02461
contig009	maker	exon	441256	442503	.	-	.	ID=MALK_02461.t1.e1;Parent=MALK_02461.t1
contig009	AUGUSTUS	gene	442743	444362	0.91	+	.	ID=MALK_02462;prediction_source=augustus:contig009.g2688.t1
contig009	AUGUSTUS	CDS	442743	444362	0.91	+	0	ID=MALK_02462.t1.c1;Parent=MALK_02462.t1
contig009	AUGUSTUS	mRNA	442743	444362	0.91	+	.	ID=MALK_02462.t1;Parent=MALK_02462
contig009	AUGUSTUS	exon	442743	444362	0.91	+	.	ID=MALK_02462.t1.e1;Parent=MALK_02462.t1
contig009	AUGUSTUS	gene	444371	447169	1	-	.	ID=MALK_02463;prediction_source=augustus:contig009.g2689.t1
contig009	AUGUSTUS	CDS	444371	447169	1	-	0	ID=MALK_02463.t1.c1;Parent=MALK_02463.t1

contig009	AUGUSTUS	mRNA	444371	447169	1	-	.	ID=MALK_02463.t1;Parent=MALK_02463
contig009	AUGUSTUS	exon	444371	447169	1	-	.	ID=MALK_02463.t1.e1;Parent=MALK_02463.t1
contig009	AUGUSTUS	gene	447285	448247	0.77	+	.	ID=MALK_02464;prediction_source=braker_MRET:g2658.t1
contig009	AUGUSTUS	CDS	447285	448247	0.77	+	0	ID=MALK_02464.t1.c1;Parent=MALK_02464.t1
contig009	AUGUSTUS	mRNA	447285	448247	0.77	+	.	ID=MALK_02464.t1;Parent=MALK_02464
contig009	AUGUSTUS	exon	447285	448247	.	+	.	ID=MALK_02464.t1.e1;Parent=MALK_02464.t1
contig009	AUGUSTUS	gene	448216	452103	0.4	+	.	ID=MALK_02465;prediction_source=augustus:contig009.g2690.t1
contig009	AUGUSTUS	CDS	448216	452103	0.4	+	0	ID=MALK_02465.t1.c1;Parent=MALK_02465.t1
contig009	AUGUSTUS	mRNA	448216	452103	0.4	+	.	ID=MALK_02465.t1;Parent=MALK_02465
contig009	AUGUSTUS	exon	448216	452103	0.4	+	.	ID=MALK_02465.t1.e1;Parent=MALK_02465.t1
contig009	AUGUSTUS	gene	452262	453545	0.84	+	.	ID=MALK_02466;prediction_source=augustus:contig009.g2691.t1
contig009	AUGUSTUS	CDS	452262	453545	0.84	+	0	ID=MALK_02466.t1.c1;Parent=MALK_02466.t1
contig009	AUGUSTUS	mRNA	452262	453545	0.84	+	.	ID=MALK_02466.t1;Parent=MALK_02466
contig009	AUGUSTUS	exon	452262	453545	0.84	+	.	ID=MALK_02466.t1.e1;Parent=MALK_02466.t1
contig009	AUGUSTUS	gene	453532	455274	0.98	-	.	ID=MALK_02467;prediction_source=augustus:contig009.g2692.t1
contig009	AUGUSTUS	CDS	453532	455274	0.98	-	0	ID=MALK_02467.t1.c1;Parent=MALK_02467.t1
contig009	AUGUSTUS	mRNA	453532	455274	0.98	-	.	ID=MALK_02467.t1;Parent=MALK_02467
contig009	AUGUSTUS	exon	453532	455274	0.98	-	.	ID=MALK_02467.t1.e1;Parent=MALK_02467.t1
contig009	AUGUSTUS	gene	456139	457326	0.54	-	.	ID=MALK_02468;prediction_source=braker_MRET:g2662.t1
contig009	AUGUSTUS	CDS	456139	457326	0.54	-	0	ID=MALK_02468.t1.c1;Parent=MALK_02468.t1
contig009	AUGUSTUS	mRNA	456139	457326	0.54	-	.	ID=MALK_02468.t1;Parent=MALK_02468
contig009	AUGUSTUS	exon	456139	457326	.	-	.	ID=MALK_02468.t1.e1;Parent=MALK_02468.t1
contig009	AUGUSTUS	gene	457517	458443	0.38	-	.	ID=MALK_02469;prediction_source=braker_MRET:g2663.t1
contig009	AUGUSTUS	CDS	457517	458443	0.38	-	0	ID=MALK_02469.t1.c1;Parent=MALK_02469.t1
contig009	AUGUSTUS	mRNA	457517	458443	0.38	-	.	ID=MALK_02469.t1;Parent=MALK_02469
contig009	AUGUSTUS	exon	457517	458443	.	-	.	ID=MALK_02469.t1.e1;Parent=MALK_02469.t1
contig009	maker	gene	458785	463224	.	+	.	ID=MALK_02470;prediction_source=maker_MRET:augustus_masked-contig009-processed-gene-4.18-mRNA-1
contig009	maker	CDS	458785	463224	.	+	0	ID=MALK_02470.t1.c1;Parent=MALK_02470.t1
contig009	maker	mRNA	458785	463224	.	+	.	ID=MALK_02470.t1;Parent=MALK_02470
contig009	maker	exon	458785	463224	.	+	.	ID=MALK_02470.t1.e1;Parent=MALK_02470.t1
contig009	maker	gene	463254	466166	.	-	.	ID=MALK_02471;prediction_source=maker_MRET:augustus_masked-contig009-processed-gene-4.35-mRNA-1
contig009	maker	CDS	463254	466166	.	-	0	ID=MALK_02471.t1.c1;Parent=MALK_02471.t1
contig009	maker	mRNA	463254	466166	.	-	.	ID=MALK_02471.t1;Parent=MALK_02471
contig009	maker	exon	463254	466166	.	-	.	ID=MALK_02471.t1.e1;Parent=MALK_02471.t1
contig009	AUGUSTUS	gene	466720	468027	0.45	-	.	ID=MALK_02472;prediction_source=augustus:contig009.g2698.t1
contig009	AUGUSTUS	CDS	466720	468027	0.45	-	0	ID=MALK_02472.t1.c1;Parent=MALK_02472.t1
contig009	AUGUSTUS	mRNA	466720	468027	0.45	-	.	ID=MALK_02472.t1;Parent=MALK_02472
contig009	AUGUSTUS	exon	466720	468027	0.45	-	.	ID=MALK_02472.t1.e1;Parent=MALK_02472.t1
contig009	AUGUSTUS	gene	469404	473102	1	-	.	ID=MALK_02473;prediction_source=augustus:contig009.g2699.t1
contig009	AUGUSTUS	CDS	469404	473102	1	-	0	ID=MALK_02473.t1.c1;Parent=MALK_02473.t1
contig009	AUGUSTUS	mRNA	469404	473102	1	-	.	ID=MALK_02473.t1;Parent=MALK_02473
contig009	AUGUSTUS	exon	469404	473102	1	-	.	ID=MALK_02473.t1.e1;Parent=MALK_02473.t1
contig009	maker	gene	473210	473656	.	-	.	ID=MALK_02474;prediction_source=maker_MRET:augustus_masked-contig009-processed-gene-4.38-mRNA-1

contig009	maker	CDS	473210	473656	.	-	0	ID=MALK_02474.t1.c1;Parent=MALK_02474.t1
contig009	maker	mRNA	473210	473656	.	-	.	ID=MALK_02474.t1;Parent=MALK_02474
contig009	maker	exon	473210	473656	.	-	.	ID=MALK_02474.t1.e1;Parent=MALK_02474.t1
contig009	AUGUSTUS	gene	473774	475129	0.95	+	.	ID=MALK_02475;prediction_source=augustus:contig009.g2701.t1
contig009	AUGUSTUS	CDS	473774	475129	0.95	+	0	ID=MALK_02475.t1.c1;Parent=MALK_02475.t1
contig009	AUGUSTUS	mRNA	473774	475129	0.95	+	.	ID=MALK_02475.t1;Parent=MALK_02475
contig009	AUGUSTUS	exon	473774	475129	0.95	+	.	ID=MALK_02475.t1.e1;Parent=MALK_02475.t1
contig009	maker	gene	475275	477203	.	-	.	ID=MALK_02476;prediction_source=maker_MRET:augustus_masked-contig009-processed-gene-4.39-mRNA-1
contig009	maker	CDS	475275	477203	.	-	0	ID=MALK_02476.t1.c1;Parent=MALK_02476.t1
contig009	maker	mRNA	475275	477203	.	-	.	ID=MALK_02476.t1;Parent=MALK_02476
contig009	maker	exon	475275	477203	.	-	.	ID=MALK_02476.t1.e1;Parent=MALK_02476.t1
contig009	maker	gene	478004	479068	.	+	.	ID=MALK_02477;prediction_source=maker_MRET:augustus_masked-contig009-processed-gene-4.20-mRNA-1
contig009	maker	CDS	478004	479068	.	+	0	ID=MALK_02477.t1.c1;Parent=MALK_02477.t1
contig009	maker	mRNA	478004	479068	.	+	.	ID=MALK_02477.t1;Parent=MALK_02477
contig009	maker	exon	478004	479068	.	+	.	ID=MALK_02477.t1.e1;Parent=MALK_02477.t1
contig009	maker	gene	479212	481254	.	-	.	ID=MALK_02478;prediction_source=maker_MRET:augustus_masked-contig009-processed-gene-4.40-mRNA-1
contig009	maker	CDS	479212	481254	.	-	0	ID=MALK_02478.t1.c1;Parent=MALK_02478.t1
contig009	maker	mRNA	479212	481254	.	-	.	ID=MALK_02478.t1;Parent=MALK_02478
contig009	maker	exon	479212	481254	.	-	.	ID=MALK_02478.t1.e1;Parent=MALK_02478.t1
contig009	AUGUSTUS	gene	481405	483927	0.56	+	.	ID=MALK_02479;prediction_source=augustus:contig009.g2705.t1
contig009	AUGUSTUS	CDS	481405	483927	0.56	+	0	ID=MALK_02479.t1.c1;Parent=MALK_02479.t1
contig009	AUGUSTUS	mRNA	481405	483927	0.56	+	.	ID=MALK_02479.t1;Parent=MALK_02479
contig009	AUGUSTUS	exon	481405	483927	0.56	+	.	ID=MALK_02479.t1.e1;Parent=MALK_02479.t1
contig009	AUGUSTUS	gene	483970	485210	0.3	-	.	ID=MALK_02480;prediction_source=braker_MRET:g2673.t1
contig009	AUGUSTUS	CDS	484087	485210	0.3	-	0	ID=MALK_02480.t1.c2;Parent=MALK_02480.t1
contig009	AUGUSTUS	CDS	483970	484051	0.3	-	0	ID=MALK_02480.t1.c1;Parent=MALK_02480.t1
contig009	AUGUSTUS	mRNA	483970	485210	0.3	-	.	ID=MALK_02480.t1;Parent=MALK_02480
contig009	AUGUSTUS	exon	484087	485210	.	-	.	ID=MALK_02480.t1.e2;Parent=MALK_02480.t1
contig009	AUGUSTUS	exon	483970	484051	.	-	.	ID=MALK_02480.t1.e1;Parent=MALK_02480.t1
contig009	AUGUSTUS	gene	485313	488457	0.52	-	.	ID=MALK_02481;prediction_source=braker_MRET:g2674.t1
contig009	AUGUSTUS	CDS	487253	488457	0.53	-	0	ID=MALK_02481.t1.c2;Parent=MALK_02481.t1
contig009	AUGUSTUS	CDS	485313	487197	0.53	-	0	ID=MALK_02481.t1.c1;Parent=MALK_02481.t1
contig009	AUGUSTUS	mRNA	485313	488457	0.52	-	.	ID=MALK_02481.t1;Parent=MALK_02481
contig009	AUGUSTUS	exon	487253	488457	.	-	.	ID=MALK_02481.t1.e2;Parent=MALK_02481.t1
contig009	AUGUSTUS	exon	485313	487197	.	-	.	ID=MALK_02481.t1.e1;Parent=MALK_02481.t1
contig009	AUGUSTUS	gene	488488	488911	0.17	+	.	ID=MALK_02482;prediction_source=braker_MRET:g2675.t1
contig009	AUGUSTUS	CDS	488488	488730	0.55	+	0	ID=MALK_02482.t1.c1;Parent=MALK_02482.t1
contig009	AUGUSTUS	CDS	488765	488911	0.55	+	0	ID=MALK_02482.t1.c2;Parent=MALK_02482.t1
contig009	AUGUSTUS	mRNA	488488	488911	0.17	+	.	ID=MALK_02482.t1;Parent=MALK_02482
contig009	AUGUSTUS	exon	488488	488730	.	+	.	ID=MALK_02482.t1.e1;Parent=MALK_02482.t1
contig009	AUGUSTUS	exon	488765	488911	.	+	.	ID=MALK_02482.t1.e2;Parent=MALK_02482.t1
contig009	AUGUSTUS	gene	488941	489726	0.6	-	.	ID=MALK_02483;prediction_source=augustus:contig009.g2707.t1
contig009	AUGUSTUS	CDS	489285	489726	0.67	-	0	ID=MALK_02483.t1.c2;Parent=MALK_02483.t1

contig009	AUGUSTUS	CDS	488941	489209	0.67	-	0	ID=MALK_02483.t1.c1;Parent=MALK_02483.t1
contig009	AUGUSTUS	mRNA	488941	489726	0.6	-	.	ID=MALK_02483.t1;Parent=MALK_02483
contig009	AUGUSTUS	exon	489285	489726	0.67	-	.	ID=MALK_02483.t1.e2;Parent=MALK_02483.t1
contig009	AUGUSTUS	exon	488941	489209	0.67	-	.	ID=MALK_02483.t1.e1;Parent=MALK_02483.t1
contig009	AUGUSTUS	gene	490172	491199	0.99	-	.	ID=MALK_02484;prediction_source=augustus:contig009.g2708.t1
contig009	AUGUSTUS	CDS	490641	491199	0.99	-	0	ID=MALK_02484.t1.c2;Parent=MALK_02484.t1
contig009	AUGUSTUS	CDS	490172	490551	0.99	-	0	ID=MALK_02484.t1.c1;Parent=MALK_02484.t1
contig009	AUGUSTUS	mRNA	490172	491199	0.99	-	.	ID=MALK_02484.t1;Parent=MALK_02484
contig009	AUGUSTUS	exon	490641	491199	0.99	-	.	ID=MALK_02484.t1.e2;Parent=MALK_02484.t1
contig009	AUGUSTUS	exon	490172	490551	0.99	-	.	ID=MALK_02484.t1.e1;Parent=MALK_02484.t1
contig009	maker	gene	491594	492697	.	+	.	ID=MALK_02485;prediction_source=maker_MRET:augustus_masked-contig009-processed-gene-4.22-mRNA-1
contig009	maker	CDS	491594	492697	.	+	0	ID=MALK_02485.t1.c1;Parent=MALK_02485.t1
contig009	maker	mRNA	491594	492697	.	+	.	ID=MALK_02485.t1;Parent=MALK_02485
contig009	maker	exon	491594	492697	.	+	.	ID=MALK_02485.t1.e1;Parent=MALK_02485.t1
contig009	AUGUSTUS	gene	493024	494016	0.98	-	.	ID=MALK_02486;prediction_source=augustus:contig009.g2711.t1
contig009	AUGUSTUS	CDS	493024	494016	0.98	-	0	ID=MALK_02486.t1.c1;Parent=MALK_02486.t1
contig009	AUGUSTUS	mRNA	493024	494016	0.98	-	.	ID=MALK_02486.t1;Parent=MALK_02486
contig009	AUGUSTUS	exon	493024	494016	0.98	-	.	ID=MALK_02486.t1.e1;Parent=MALK_02486.t1
contig009	AUGUSTUS	gene	495178	495939	0.48	-	.	ID=MALK_02487;prediction_source=braker_MRET:g2680.t1
contig009	AUGUSTUS	CDS	495931	495939	1	-	0	ID=MALK_02487.t1.c4;Parent=MALK_02487.t1
contig009	AUGUSTUS	CDS	495749	495869	1	-	0	ID=MALK_02487.t1.c3;Parent=MALK_02487.t1
contig009	AUGUSTUS	CDS	495643	495718	1	-	0	ID=MALK_02487.t1.c2;Parent=MALK_02487.t1
contig009	AUGUSTUS	CDS	495178	495610	1	-	0	ID=MALK_02487.t1.c1;Parent=MALK_02487.t1
contig009	AUGUSTUS	mRNA	495178	495939	0.48	-	.	ID=MALK_02487.t1;Parent=MALK_02487
contig009	AUGUSTUS	exon	495931	495939	.	-	.	ID=MALK_02487.t1.e4;Parent=MALK_02487.t1
contig009	AUGUSTUS	exon	495749	495869	.	-	.	ID=MALK_02487.t1.e3;Parent=MALK_02487.t1
contig009	AUGUSTUS	exon	495643	495718	.	-	.	ID=MALK_02487.t1.e2;Parent=MALK_02487.t1
contig009	AUGUSTUS	exon	495178	495610	.	-	.	ID=MALK_02487.t1.e1;Parent=MALK_02487.t1
contig009	AUGUSTUS	gene	496340	498322	0.96	+	.	ID=MALK_02488;prediction_source=augustus:contig009.g2713.t1
contig009	AUGUSTUS	CDS	496340	498322	0.96	+	0	ID=MALK_02488.t1.c1;Parent=MALK_02488.t1
contig009	AUGUSTUS	mRNA	496340	498322	0.96	+	.	ID=MALK_02488.t1;Parent=MALK_02488
contig009	AUGUSTUS	exon	496340	498322	0.96	+	.	ID=MALK_02488.t1.e1;Parent=MALK_02488.t1
contig009	maker	gene	498428	500857	.	+	.	ID=MALK_02489;prediction_source=maker_MRET:augustus_masked-contig009-processed-gene-5.50-mRNA-1
contig009	maker	CDS	498428	500857	.	+	0	ID=MALK_02489.t1.c1;Parent=MALK_02489.t1
contig009	maker	mRNA	498428	500857	.	+	.	ID=MALK_02489.t1;Parent=MALK_02489
contig009	maker	exon	498428	500857	.	+	.	ID=MALK_02489.t1.e1;Parent=MALK_02489.t1
contig009	AUGUSTUS	gene	500915	501952	0.92	-	.	ID=MALK_02490;prediction_source=augustus:contig009.g2715.t1
contig009	AUGUSTUS	CDS	500915	501952	0.92	-	0	ID=MALK_02490.t1.c1;Parent=MALK_02490.t1
contig009	AUGUSTUS	mRNA	500915	501952	0.92	-	.	ID=MALK_02490.t1;Parent=MALK_02490
contig009	AUGUSTUS	exon	500915	501952	0.92	-	.	ID=MALK_02490.t1.e1;Parent=MALK_02490.t1
contig009	AUGUSTUS	gene	502515	503693	0.57	-	.	ID=MALK_02491;prediction_source=augustus:contig009.g2717.t1
contig009	AUGUSTUS	CDS	503591	503693	0.57	-	0	ID=MALK_02491.t1.c2;Parent=MALK_02491.t1
contig009	AUGUSTUS	CDS	502515	502825	0.57	-	0	ID=MALK_02491.t1.c1;Parent=MALK_02491.t1

contig009	AUGUSTUS	mRNA	502515	503693	0.57	-	.	ID=MALK_02491.t1;Parent=MALK_02491
contig009	AUGUSTUS	exon	503591	503693	0.57	-	.	ID=MALK_02491.t1.e2;Parent=MALK_02491.t1
contig009	AUGUSTUS	exon	502515	502825	0.57	-	.	ID=MALK_02491.t1.e1;Parent=MALK_02491.t1
contig009	AUGUSTUS	gene	503746	504048	0.98	+	.	ID=MALK_02492;prediction_source=augustus:contig009.g2718.t1
contig009	AUGUSTUS	CDS	503746	504048	0.98	+	0	ID=MALK_02492.t1.c1;Parent=MALK_02492.t1
contig009	AUGUSTUS	mRNA	503746	504048	0.98	+	.	ID=MALK_02492.t1;Parent=MALK_02492
contig009	AUGUSTUS	exon	503746	504048	0.98	+	.	ID=MALK_02492.t1.e1;Parent=MALK_02492.t1
contig009	maker	gene	504503	505006	.	+	.	ID=MALK_02493;prediction_source=maker_MRET:augustus_masked-contig009-processed-gene-5.54-mRNA-1
contig009	maker	CDS	504503	505006	.	+	0	ID=MALK_02493.t1.c1;Parent=MALK_02493.t1
contig009	maker	mRNA	504503	505006	.	+	.	ID=MALK_02493.t1;Parent=MALK_02493
contig009	maker	exon	504503	505006	.	+	.	ID=MALK_02493.t1.e1;Parent=MALK_02493.t1
contig009	AUGUSTUS	gene	505018	506373	0.46	-	.	ID=MALK_02494;prediction_source=augustus:contig009.g2720.t1
contig009	AUGUSTUS	CDS	505018	506373	0.46	-	0	ID=MALK_02494.t1.c1;Parent=MALK_02494.t1
contig009	AUGUSTUS	mRNA	505018	506373	0.46	-	.	ID=MALK_02494.t1;Parent=MALK_02494
contig009	AUGUSTUS	exon	505018	506373	0.46	-	.	ID=MALK_02494.t1.e1;Parent=MALK_02494.t1
contig009	AUGUSTUS	gene	506517	508343	1	+	.	ID=MALK_02495;prediction_source=augustus:contig009.g2721.t1
contig009	AUGUSTUS	CDS	506517	508343	1	+	0	ID=MALK_02495.t1.c1;Parent=MALK_02495.t1
contig009	AUGUSTUS	mRNA	506517	508343	1	+	.	ID=MALK_02495.t1;Parent=MALK_02495
contig009	AUGUSTUS	exon	506517	508343	1	+	.	ID=MALK_02495.t1.e1;Parent=MALK_02495.t1
contig009	AUGUSTUS	gene	508481	510154	1	+	.	ID=MALK_02496;prediction_source=augustus:contig009.g2722.t1
contig009	AUGUSTUS	CDS	508481	510154	1	+	0	ID=MALK_02496.t1.c1;Parent=MALK_02496.t1
contig009	AUGUSTUS	mRNA	508481	510154	1	+	.	ID=MALK_02496.t1;Parent=MALK_02496
contig009	AUGUSTUS	exon	508481	510154	1	+	.	ID=MALK_02496.t1.e1;Parent=MALK_02496.t1
contig009	AUGUSTUS	gene	510208	512910	0.47	-	.	ID=MALK_02497;prediction_source=augustus:contig009.g2723.t1
contig009	AUGUSTUS	CDS	510208	512910	0.47	-	0	ID=MALK_02497.t1.c1;Parent=MALK_02497.t1
contig009	AUGUSTUS	mRNA	510208	512910	0.47	-	.	ID=MALK_02497.t1;Parent=MALK_02497
contig009	AUGUSTUS	exon	510208	512910	0.47	-	.	ID=MALK_02497.t1.e1;Parent=MALK_02497.t1
contig009	AUGUSTUS	gene	513076	517956	0.15	-	.	ID=MALK_02498;prediction_source=augustus:contig009.g2724.t1
contig009	AUGUSTUS	CDS	516587	517956	0.48	-	0	ID=MALK_02498.t1.c2;Parent=MALK_02498.t1
contig009	AUGUSTUS	CDS	513076	516487	0.48	-	0	ID=MALK_02498.t1.c1;Parent=MALK_02498.t1
contig009	AUGUSTUS	mRNA	513076	517956	0.15	-	.	ID=MALK_02498.t1;Parent=MALK_02498
contig009	AUGUSTUS	exon	516587	517956	0.48	-	.	ID=MALK_02498.t1.e2;Parent=MALK_02498.t1
contig009	AUGUSTUS	exon	513076	516487	0.48	-	.	ID=MALK_02498.t1.e1;Parent=MALK_02498.t1
contig009	AUGUSTUS	gene	518230	523863	0.79	+	.	ID=MALK_02499;prediction_source=augustus:contig009.g2726.t1
contig009	AUGUSTUS	CDS	518230	523863	0.79	+	0	ID=MALK_02499.t1.c1;Parent=MALK_02499.t1
contig009	AUGUSTUS	mRNA	518230	523863	0.79	+	.	ID=MALK_02499.t1;Parent=MALK_02499
contig009	AUGUSTUS	exon	518230	523863	0.79	+	.	ID=MALK_02499.t1.e1;Parent=MALK_02499.t1
contig009	AUGUSTUS	gene	523891	526524	0.85	-	.	ID=MALK_02500;prediction_source=augustus:contig009.g2727.t1
contig009	AUGUSTUS	CDS	523891	526524	0.85	-	0	ID=MALK_02500.t1.c1;Parent=MALK_02500.t1
contig009	AUGUSTUS	mRNA	523891	526524	0.85	-	.	ID=MALK_02500.t1;Parent=MALK_02500
contig009	AUGUSTUS	exon	523891	526524	0.85	-	.	ID=MALK_02500.t1.e1;Parent=MALK_02500.t1
contig009	AUGUSTUS	gene	526859	527185	0.61	+	.	ID=MALK_02501;prediction_source=braker_MRET:g2694.t1
contig009	AUGUSTUS	CDS	526859	527185	0.61	+	0	ID=MALK_02501.t1.c1;Parent=MALK_02501.t1

contig009	AUGUSTUS	mRNA	526859	527185	0.61	+	.	ID=MALK_02501.t1;Parent=MALK_02501
contig009	AUGUSTUS	exon	526859	527185	.	+	.	ID=MALK_02501.t1.e1;Parent=MALK_02501.t1
contig009	AUGUSTUS	gene	527193	527825	0.83	-	.	ID=MALK_02502;prediction_source=augustus:contig009.g2728.t1
contig009	AUGUSTUS	CDS	527193	527825	0.83	-	0	ID=MALK_02502.t1.c1;Parent=MALK_02502.t1
contig009	AUGUSTUS	mRNA	527193	527825	0.83	-	.	ID=MALK_02502.t1;Parent=MALK_02502
contig009	AUGUSTUS	exon	527193	527825	0.83	-	.	ID=MALK_02502.t1.e1;Parent=MALK_02502.t1
contig009	AUGUSTUS	gene	527959	529677	0.84	-	.	ID=MALK_02503;prediction_source=augustus:contig009.g2729.t1
contig009	AUGUSTUS	CDS	527959	529677	0.84	-	0	ID=MALK_02503.t1.c1;Parent=MALK_02503.t1
contig009	AUGUSTUS	mRNA	527959	529677	0.84	-	.	ID=MALK_02503.t1;Parent=MALK_02503
contig009	AUGUSTUS	exon	527959	529677	0.84	-	.	ID=MALK_02503.t1.e1;Parent=MALK_02503.t1
contig009	AUGUSTUS	gene	529832	530398	0.73	-	.	ID=MALK_02504;prediction_source=augustus:contig009.g2730.t1
contig009	AUGUSTUS	CDS	529832	530398	0.73	-	0	ID=MALK_02504.t1.c1;Parent=MALK_02504.t1
contig009	AUGUSTUS	mRNA	529832	530398	0.73	-	.	ID=MALK_02504.t1;Parent=MALK_02504
contig009	AUGUSTUS	exon	529832	530398	0.73	-	.	ID=MALK_02504.t1.e1;Parent=MALK_02504.t1
contig009	maker	gene	530538	532844	.	+	.	ID=MALK_02505;prediction_source=maker_MRET:augustus_masked-contig009-processed-gene-5.58-mRNA-1
contig009	maker	CDS	530538	532844	.	+	0	ID=MALK_02505.t1.c1;Parent=MALK_02505.t1
contig009	maker	mRNA	530538	532844	.	+	.	ID=MALK_02505.t1;Parent=MALK_02505
contig009	maker	exon	530538	532844	.	+	.	ID=MALK_02505.t1.e1;Parent=MALK_02505.t1
contig009	AUGUSTUS	gene	532954	533521	0.62	+	.	ID=MALK_02506;prediction_source=braker_MRET:g2698.t1
contig009	AUGUSTUS	CDS	532954	533204	0.78	+	0	ID=MALK_02506.t1.c1;Parent=MALK_02506.t1
contig009	AUGUSTUS	CDS	533272	533521	0.78	+	0	ID=MALK_02506.t1.c2;Parent=MALK_02506.t1
contig009	AUGUSTUS	mRNA	532954	533521	0.62	+	.	ID=MALK_02506.t1;Parent=MALK_02506
contig009	AUGUSTUS	exon	532954	533204	.	+	.	ID=MALK_02506.t1.e1;Parent=MALK_02506.t1
contig009	AUGUSTUS	exon	533272	533521	.	+	.	ID=MALK_02506.t1.e2;Parent=MALK_02506.t1
contig009	AUGUSTUS	gene	533565	534839	0.82	-	.	ID=MALK_02507;prediction_source=augustus:contig009.g2732.t1
contig009	AUGUSTUS	CDS	533565	534839	0.82	-	0	ID=MALK_02507.t1.c1;Parent=MALK_02507.t1
contig009	AUGUSTUS	mRNA	533565	534839	0.82	-	.	ID=MALK_02507.t1;Parent=MALK_02507
contig009	AUGUSTUS	exon	533565	534839	0.82	-	.	ID=MALK_02507.t1.e1;Parent=MALK_02507.t1
contig009	AUGUSTUS	gene	535062	536540	0.7	+	.	ID=MALK_02508;prediction_source=augustus:contig009.g2733.t1
contig009	AUGUSTUS	CDS	535062	536540	0.7	+	0	ID=MALK_02508.t1.c1;Parent=MALK_02508.t1
contig009	AUGUSTUS	mRNA	535062	536540	0.7	+	.	ID=MALK_02508.t1;Parent=MALK_02508
contig009	AUGUSTUS	exon	535062	536540	0.7	+	.	ID=MALK_02508.t1.e1;Parent=MALK_02508.t1
contig009	AUGUSTUS	gene	536578	538836	1	-	.	ID=MALK_02509;prediction_source=augustus:contig009.g2734.t1
contig009	AUGUSTUS	CDS	536578	538836	1	-	0	ID=MALK_02509.t1.c1;Parent=MALK_02509.t1
contig009	AUGUSTUS	mRNA	536578	538836	1	-	.	ID=MALK_02509.t1;Parent=MALK_02509
contig009	AUGUSTUS	exon	536578	538836	1	-	.	ID=MALK_02509.t1.e1;Parent=MALK_02509.t1
contig009	AUGUSTUS	gene	539047	540049	0.77	+	.	ID=MALK_02510;prediction_source=braker_MRET:g2702.t1
contig009	AUGUSTUS	CDS	539047	539059	1	+	0	ID=MALK_02510.t1.c1;Parent=MALK_02510.t1
contig009	AUGUSTUS	CDS	539099	539117	1	+	0	ID=MALK_02510.t1.c2;Parent=MALK_02510.t1
contig009	AUGUSTUS	CDS	539147	539162	1	+	0	ID=MALK_02510.t1.c3;Parent=MALK_02510.t1
contig009	AUGUSTUS	CDS	539204	540049	1	+	0	ID=MALK_02510.t1.c4;Parent=MALK_02510.t1
contig009	AUGUSTUS	mRNA	539047	540049	0.77	+	.	ID=MALK_02510.t1;Parent=MALK_02510
contig009	AUGUSTUS	exon	539047	539059	.	+	.	ID=MALK_02510.t1.e1;Parent=MALK_02510.t1

contig009	AUGUSTUS	exon	539099	539117	.	+	.	ID=MALK_02510.t1.e2;Parent=MALK_02510.t1
contig009	AUGUSTUS	exon	539147	539162	.	+	.	ID=MALK_02510.t1.e3;Parent=MALK_02510.t1
contig009	AUGUSTUS	exon	539204	540049	.	+	.	ID=MALK_02510.t1.e4;Parent=MALK_02510.t1
contig009	AUGUSTUS	gene	540099	541976	0.46	+	.	ID=MALK_02511;prediction_source=braker_MRET:g2703.t1
contig009	AUGUSTUS	CDS	540099	541976	0.46	+	0	ID=MALK_02511.t1.c1;Parent=MALK_02511.t1
contig009	AUGUSTUS	mRNA	540099	541976	0.46	+	.	ID=MALK_02511.t1;Parent=MALK_02511
contig009	AUGUSTUS	exon	540099	541976	.	+	.	ID=MALK_02511.t1.e1;Parent=MALK_02511.t1
contig009	AUGUSTUS	gene	541981	543321	0.5	-	.	ID=MALK_02512;prediction_source=braker_MRET:g2704.t1
contig009	AUGUSTUS	CDS	541981	543321	0.5	-	0	ID=MALK_02512.t1.c1;Parent=MALK_02512.t1
contig009	AUGUSTUS	mRNA	541981	543321	0.5	-	.	ID=MALK_02512.t1;Parent=MALK_02512
contig009	AUGUSTUS	exon	541981	543321	.	-	.	ID=MALK_02512.t1.e1;Parent=MALK_02512.t1
contig009	AUGUSTUS	gene	543452	543997	0.51	+	.	ID=MALK_02513;prediction_source=augustus:contig009.g2737.t1
contig009	AUGUSTUS	CDS	543452	543997	0.51	+	0	ID=MALK_02513.t1.c1;Parent=MALK_02513.t1
contig009	AUGUSTUS	mRNA	543452	543997	0.51	+	.	ID=MALK_02513.t1;Parent=MALK_02513
contig009	AUGUSTUS	exon	543452	543997	0.51	+	.	ID=MALK_02513.t1.e1;Parent=MALK_02513.t1
contig009	AUGUSTUS	gene	543994	545613	0.84	-	.	ID=MALK_02514;prediction_source=augustus:contig009.g2738.t1
contig009	AUGUSTUS	CDS	543994	545613	0.84	-	0	ID=MALK_02514.t1.c1;Parent=MALK_02514.t1
contig009	AUGUSTUS	mRNA	543994	545613	0.84	-	.	ID=MALK_02514.t1;Parent=MALK_02514
contig009	AUGUSTUS	exon	543994	545613	0.84	-	.	ID=MALK_02514.t1.e1;Parent=MALK_02514.t1
contig009	maker	gene	545862	548246	.	+	.	ID=MALK_02515;prediction_source=maker_MRET:augustus_masked-contig009-processed-gene-5.62-mRNA-1
contig009	maker	CDS	545862	548246	.	+	0	ID=MALK_02515.t1.c1;Parent=MALK_02515.t1
contig009	maker	mRNA	545862	548246	.	+	.	ID=MALK_02515.t1;Parent=MALK_02515
contig009	maker	exon	545862	548246	.	+	.	ID=MALK_02515.t1.e1;Parent=MALK_02515.t1
contig009	AUGUSTUS	gene	548280	550005	0.38	-	.	ID=MALK_02516;prediction_source=braker_MRET:g2708.t1
contig009	AUGUSTUS	CDS	549973	550005	1	-	0	ID=MALK_02516.t1.c7;Parent=MALK_02516.t1
contig009	AUGUSTUS	CDS	549634	549942	1	-	0	ID=MALK_02516.t1.c6;Parent=MALK_02516.t1
contig009	AUGUSTUS	CDS	549503	549602	1	-	0	ID=MALK_02516.t1.c5;Parent=MALK_02516.t1
contig009	AUGUSTUS	CDS	549090	549467	1	-	0	ID=MALK_02516.t1.c4;Parent=MALK_02516.t1
contig009	AUGUSTUS	CDS	548930	549054	1	-	0	ID=MALK_02516.t1.c3;Parent=MALK_02516.t1
contig009	AUGUSTUS	CDS	548745	548830	1	-	0	ID=MALK_02516.t1.c2;Parent=MALK_02516.t1
contig009	AUGUSTUS	CDS	548280	548715	1	-	0	ID=MALK_02516.t1.c1;Parent=MALK_02516.t1
contig009	AUGUSTUS	mRNA	548280	550005	0.38	-	.	ID=MALK_02516.t1;Parent=MALK_02516
contig009	AUGUSTUS	exon	549973	550005	.	-	.	ID=MALK_02516.t1.e7;Parent=MALK_02516.t1
contig009	AUGUSTUS	exon	549634	549942	.	-	.	ID=MALK_02516.t1.e6;Parent=MALK_02516.t1
contig009	AUGUSTUS	exon	549503	549602	.	-	.	ID=MALK_02516.t1.e5;Parent=MALK_02516.t1
contig009	AUGUSTUS	exon	549090	549467	.	-	.	ID=MALK_02516.t1.e4;Parent=MALK_02516.t1
contig009	AUGUSTUS	exon	548930	549054	.	-	.	ID=MALK_02516.t1.e3;Parent=MALK_02516.t1
contig009	AUGUSTUS	exon	548745	548830	.	-	.	ID=MALK_02516.t1.e2;Parent=MALK_02516.t1
contig009	AUGUSTUS	exon	548280	548715	.	-	.	ID=MALK_02516.t1.e1;Parent=MALK_02516.t1
contig009	AUGUSTUS	gene	550096	552300	0.87	+	.	ID=MALK_02517;prediction_source=augustus:contig009.g2740.t1
contig009	AUGUSTUS	CDS	550096	552300	0.87	+	0	ID=MALK_02517.t1.c1;Parent=MALK_02517.t1
contig009	AUGUSTUS	mRNA	550096	552300	0.87	+	.	ID=MALK_02517.t1;Parent=MALK_02517
contig009	AUGUSTUS	exon	550096	552300	0.87	+	.	ID=MALK_02517.t1.e1;Parent=MALK_02517.t1

contig009	maker	gene	552311	553720	.	-	.	ID=MALK_02518;prediction_source=maker_MRET:augustus_masked-contig009-processed-gene-5.85-mRNA-1
contig009	maker	CDS	552311	553720	.	-	0	ID=MALK_02518.t1.c1;Parent=MALK_02518.t1
contig009	maker	mRNA	552311	553720	.	-	.	ID=MALK_02518.t1;Parent=MALK_02518
contig009	maker	exon	552311	553720	.	-	.	ID=MALK_02518.t1.e1;Parent=MALK_02518.t1
contig009	maker	gene	553797	555143	.	+	.	ID=MALK_02519;prediction_source=maker_MRET:augustus_masked-contig009-processed-gene-5.64-mRNA-1
contig009	maker	CDS	553797	555143	.	+	0	ID=MALK_02519.t1.c1;Parent=MALK_02519.t1
contig009	maker	mRNA	553797	555143	.	+	.	ID=MALK_02519.t1;Parent=MALK_02519
contig009	maker	exon	553797	555143	.	+	.	ID=MALK_02519.t1.e1;Parent=MALK_02519.t1
contig009	AUGUSTUS	gene	555140	556126	0.9	-	.	ID=MALK_02520;prediction_source=augustus:contig009.g2743.t1
contig009	AUGUSTUS	CDS	555140	556126	0.9	-	0	ID=MALK_02520.t1.c1;Parent=MALK_02520.t1
contig009	AUGUSTUS	mRNA	555140	556126	0.9	-	.	ID=MALK_02520.t1;Parent=MALK_02520
contig009	AUGUSTUS	exon	555140	556126	0.9	-	.	ID=MALK_02520.t1.e1;Parent=MALK_02520.t1
contig009	AUGUSTUS	gene	556369	557394	0.6	-	.	ID=MALK_02521;prediction_source=augustus:contig009.g2744.t1
contig009	AUGUSTUS	CDS	556369	557394	0.6	-	0	ID=MALK_02521.t1.c1;Parent=MALK_02521.t1
contig009	AUGUSTUS	mRNA	556369	557394	0.6	-	.	ID=MALK_02521.t1;Parent=MALK_02521
contig009	AUGUSTUS	exon	556369	557394	0.6	-	.	ID=MALK_02521.t1.e1;Parent=MALK_02521.t1
contig009	maker	gene	557857	558516	.	-	.	ID=MALK_02522;prediction_source=maker_MRET:augustus_masked-contig009-processed-gene-5.87-mRNA-1
contig009	maker	CDS	558514	558516	.	-	0	ID=MALK_02522.t1.c1;Parent=MALK_02522.t1
contig009	maker	CDS	557857	558261	.	-	0	ID=MALK_02522.t1.c2;Parent=MALK_02522.t1
contig009	maker	mRNA	557857	558516	.	-	.	ID=MALK_02522.t1;Parent=MALK_02522
contig009	maker	exon	558514	558516	.	-	.	ID=MALK_02522.t1.e1;Parent=MALK_02522.t1
contig009	maker	exon	557857	558261	.	-	.	ID=MALK_02522.t1.e2;Parent=MALK_02522.t1
contig009	AUGUSTUS	gene	558756	559472	1	+	.	ID=MALK_02523;prediction_source=braker_MRET:g2715.t1
contig009	AUGUSTUS	CDS	558756	558776	1	+	0	ID=MALK_02523.t1.c1;Parent=MALK_02523.t1
contig009	AUGUSTUS	CDS	558821	558932	1	+	0	ID=MALK_02523.t1.c2;Parent=MALK_02523.t1
contig009	AUGUSTUS	CDS	559213	559472	1	+	0	ID=MALK_02523.t1.c3;Parent=MALK_02523.t1
contig009	AUGUSTUS	mRNA	558756	559472	1	+	.	ID=MALK_02523.t1;Parent=MALK_02523
contig009	AUGUSTUS	exon	558756	558776	.	+	.	ID=MALK_02523.t1.e1;Parent=MALK_02523.t1
contig009	AUGUSTUS	exon	558821	558932	.	+	.	ID=MALK_02523.t1.e2;Parent=MALK_02523.t1
contig009	AUGUSTUS	exon	559213	559472	.	+	.	ID=MALK_02523.t1.e3;Parent=MALK_02523.t1
contig009	AUGUSTUS	gene	559676	560655	0.26	-	.	ID=MALK_02524;prediction_source=braker_MRET:g2716.t1
contig009	AUGUSTUS	CDS	560598	560655	0.48	-	0	ID=MALK_02524.t1.c5;Parent=MALK_02524.t1
contig009	AUGUSTUS	CDS	560470	560569	0.48	-	0	ID=MALK_02524.t1.c4;Parent=MALK_02524.t1
contig009	AUGUSTUS	CDS	560302	560432	0.48	-	0	ID=MALK_02524.t1.c3;Parent=MALK_02524.t1
contig009	AUGUSTUS	CDS	560125	560273	0.48	-	0	ID=MALK_02524.t1.c2;Parent=MALK_02524.t1
contig009	AUGUSTUS	CDS	559676	560089	0.48	-	0	ID=MALK_02524.t1.c1;Parent=MALK_02524.t1
contig009	AUGUSTUS	mRNA	559676	560655	0.26	-	.	ID=MALK_02524.t1;Parent=MALK_02524
contig009	AUGUSTUS	exon	560598	560655	.	-	.	ID=MALK_02524.t1.e5;Parent=MALK_02524.t1
contig009	AUGUSTUS	exon	560470	560569	.	-	.	ID=MALK_02524.t1.e4;Parent=MALK_02524.t1
contig009	AUGUSTUS	exon	560302	560432	.	-	.	ID=MALK_02524.t1.e3;Parent=MALK_02524.t1
contig009	AUGUSTUS	exon	560125	560273	.	-	.	ID=MALK_02524.t1.e2;Parent=MALK_02524.t1
contig009	AUGUSTUS	exon	559676	560089	.	-	.	ID=MALK_02524.t1.e1;Parent=MALK_02524.t1
contig009	maker	gene	560987	561599	.	-	.	ID=MALK_02525;prediction_source=maker_MRET:augustus_masked-contig009-processed-gene-5.89-mRNA-1

contig009	maker	CDS	561390	561599	.	-	0	ID=MALK_02525.t1.c1;Parent=MALK_02525.t1
contig009	maker	CDS	560987	561280	.	-	0	ID=MALK_02525.t1.c2;Parent=MALK_02525.t1
contig009	maker	mRNA	560987	561599	.	-	.	ID=MALK_02525.t1;Parent=MALK_02525
contig009	maker	exon	561390	561599	.	-	.	ID=MALK_02525.t1.e1;Parent=MALK_02525.t1
contig009	maker	exon	560987	561280	.	-	.	ID=MALK_02525.t1.e2;Parent=MALK_02525.t1
contig009	maker	gene	561644	566329	.	+	.	ID=MALK_02526;prediction_source=maker_MRET:augustus_masked-contig009-processed-gene-5.65-mRNA-1
contig009	maker	CDS	561644	561668	.	+	0	ID=MALK_02526.t1.c1;Parent=MALK_02526.t1
contig009	maker	CDS	561750	564324	.	+	0	ID=MALK_02526.t1.c2;Parent=MALK_02526.t1
contig009	maker	CDS	564412	566329	.	+	0	ID=MALK_02526.t1.c3;Parent=MALK_02526.t1
contig009	maker	mRNA	561644	566329	.	+	.	ID=MALK_02526.t1;Parent=MALK_02526
contig009	maker	exon	561644	561668	.	+	.	ID=MALK_02526.t1.e1;Parent=MALK_02526.t1
contig009	maker	exon	561750	564324	.	+	.	ID=MALK_02526.t1.e2;Parent=MALK_02526.t1
contig009	maker	exon	564412	566329	.	+	.	ID=MALK_02526.t1.e3;Parent=MALK_02526.t1
contig009	AUGUSTUS	gene	566376	569252	0.99	-	.	ID=MALK_02527;prediction_source=augustus:contig009.g2749.t1
contig009	AUGUSTUS	CDS	566376	569252	0.99	-	0	ID=MALK_02527.t1.c1;Parent=MALK_02527.t1
contig009	AUGUSTUS	mRNA	566376	569252	0.99	-	.	ID=MALK_02527.t1;Parent=MALK_02527
contig009	AUGUSTUS	exon	566376	569252	0.99	-	.	ID=MALK_02527.t1.e1;Parent=MALK_02527.t1
contig009	AUGUSTUS	gene	569822	571369	0.8	+	.	ID=MALK_02528;prediction_source=augustus:contig009.g2751.t1
contig009	AUGUSTUS	CDS	569822	571369	0.8	+	0	ID=MALK_02528.t1.c1;Parent=MALK_02528.t1
contig009	AUGUSTUS	mRNA	569822	571369	0.8	+	.	ID=MALK_02528.t1;Parent=MALK_02528
contig009	AUGUSTUS	exon	569822	571369	0.8	+	.	ID=MALK_02528.t1.e1;Parent=MALK_02528.t1
contig009	AUGUSTUS	gene	571445	572869	1	+	.	ID=MALK_02529;prediction_source=braker_MRET:g2721.t1
contig009	AUGUSTUS	CDS	571445	572869	1	+	0	ID=MALK_02529.t1.c1;Parent=MALK_02529.t1
contig009	AUGUSTUS	mRNA	571445	572869	1	+	.	ID=MALK_02529.t1;Parent=MALK_02529
contig009	AUGUSTUS	exon	571445	572869	.	+	.	ID=MALK_02529.t1.e1;Parent=MALK_02529.t1
contig009	AUGUSTUS	gene	572902	574290	0.49	-	.	ID=MALK_02530;prediction_source=braker_MRET:g2722.t1
contig009	AUGUSTUS	CDS	572902	574290	0.49	-	0	ID=MALK_02530.t1.c1;Parent=MALK_02530.t1
contig009	AUGUSTUS	mRNA	572902	574290	0.49	-	.	ID=MALK_02530.t1;Parent=MALK_02530
contig009	AUGUSTUS	exon	572902	574290	.	-	.	ID=MALK_02530.t1.e1;Parent=MALK_02530.t1
contig009	AUGUSTUS	gene	574434	577064	0.98	-	.	ID=MALK_02531;prediction_source=braker_MRET:g2723.t1
contig009	AUGUSTUS	CDS	574434	577064	0.98	-	0	ID=MALK_02531.t1.c1;Parent=MALK_02531.t1
contig009	AUGUSTUS	mRNA	574434	577064	0.98	-	.	ID=MALK_02531.t1;Parent=MALK_02531
contig009	AUGUSTUS	exon	574434	577064	.	-	.	ID=MALK_02531.t1.e1;Parent=MALK_02531.t1
contig009	AUGUSTUS	gene	577359	578713	0.26	-	.	ID=MALK_02532;prediction_source=braker_MRET:g2724.t1
contig009	AUGUSTUS	CDS	578515	578713	0.31	-	0	ID=MALK_02532.t1.c7;Parent=MALK_02532.t1
contig009	AUGUSTUS	CDS	578064	578486	0.31	-	0	ID=MALK_02532.t1.c6;Parent=MALK_02532.t1
contig009	AUGUSTUS	CDS	577828	578035	0.31	-	0	ID=MALK_02532.t1.c5;Parent=MALK_02532.t1
contig009	AUGUSTUS	CDS	577667	577771	0.31	-	0	ID=MALK_02532.t1.c4;Parent=MALK_02532.t1
contig009	AUGUSTUS	CDS	577532	577619	0.31	-	0	ID=MALK_02532.t1.c3;Parent=MALK_02532.t1
contig009	AUGUSTUS	CDS	577439	577465	0.31	-	0	ID=MALK_02532.t1.c2;Parent=MALK_02532.t1
contig009	AUGUSTUS	CDS	577359	577385	0.31	-	0	ID=MALK_02532.t1.c1;Parent=MALK_02532.t1
contig009	AUGUSTUS	mRNA	577359	578713	0.26	-	.	ID=MALK_02532.t1;Parent=MALK_02532
contig009	AUGUSTUS	exon	578515	578713	.	-	.	ID=MALK_02532.t1.e7;Parent=MALK_02532.t1

contig009	AUGUSTUS	exon	578064	578486	.	-	.	ID=MALK_02532.t1.e6;Parent=MALK_02532.t1
contig009	AUGUSTUS	exon	577828	578035	.	-	.	ID=MALK_02532.t1.e5;Parent=MALK_02532.t1
contig009	AUGUSTUS	exon	577667	577771	.	-	.	ID=MALK_02532.t1.e4;Parent=MALK_02532.t1
contig009	AUGUSTUS	exon	577532	577619	.	-	.	ID=MALK_02532.t1.e3;Parent=MALK_02532.t1
contig009	AUGUSTUS	exon	577439	577465	.	-	.	ID=MALK_02532.t1.e2;Parent=MALK_02532.t1
contig009	AUGUSTUS	exon	577359	577385	.	-	.	ID=MALK_02532.t1.e1;Parent=MALK_02532.t1
contig009	AUGUSTUS	gene	578675	581527	0.14	+	.	ID=MALK_02533;prediction_source=augustus:contig009.g2754.t1
contig009	AUGUSTUS	CDS	578675	581527	0.14	+	0	ID=MALK_02533.t1.c1;Parent=MALK_02533.t1
contig009	AUGUSTUS	mRNA	578675	581527	0.14	+	.	ID=MALK_02533.t1;Parent=MALK_02533
contig009	AUGUSTUS	exon	578675	581527	0.14	+	.	ID=MALK_02533.t1.e1;Parent=MALK_02533.t1
contig009	AUGUSTUS	gene	581657	583579	0.31	+	.	ID=MALK_02534;prediction_source=augustus:contig009.g2755.t1
contig009	AUGUSTUS	CDS	581657	583579	0.31	+	0	ID=MALK_02534.t1.c1;Parent=MALK_02534.t1
contig009	AUGUSTUS	mRNA	581657	583579	0.31	+	.	ID=MALK_02534.t1;Parent=MALK_02534
contig009	AUGUSTUS	exon	581657	583579	0.31	+	.	ID=MALK_02534.t1.e1;Parent=MALK_02534.t1
contig009	maker	gene	583827	585431	.	+	.	ID=MALK_02535;prediction_source=maker_MRET:augustus_masked-contig009-processed-gene-5.70-mRNA-1
contig009	maker	CDS	583827	585431	.	+	0	ID=MALK_02535.t1.c1;Parent=MALK_02535.t1
contig009	maker	mRNA	583827	585431	.	+	.	ID=MALK_02535.t1;Parent=MALK_02535
contig009	maker	exon	583827	585431	.	+	.	ID=MALK_02535.t1.e1;Parent=MALK_02535.t1
contig009	maker	gene	585502	586578	.	-	.	ID=MALK_02536;prediction_source=maker_MRET:augustus_masked-contig009-processed-gene-5.92-mRNA-1
contig009	maker	CDS	585502	586578	.	-	0	ID=MALK_02536.t1.c1;Parent=MALK_02536.t1
contig009	maker	mRNA	585502	586578	.	-	.	ID=MALK_02536.t1;Parent=MALK_02536
contig009	maker	exon	585502	586578	.	-	.	ID=MALK_02536.t1.e1;Parent=MALK_02536.t1
contig009	maker	gene	586725	587624	.	+	.	ID=MALK_02537;prediction_source=maker_MRET:augustus_masked-contig009-processed-gene-5.71-mRNA-1
contig009	maker	CDS	586725	587624	.	+	0	ID=MALK_02537.t1.c1;Parent=MALK_02537.t1
contig009	maker	mRNA	586725	587624	.	+	.	ID=MALK_02537.t1;Parent=MALK_02537
contig009	maker	exon	586725	587624	.	+	.	ID=MALK_02537.t1.e1;Parent=MALK_02537.t1
contig009	maker	gene	587642	588274	.	-	.	ID=MALK_02538;prediction_source=maker_MRET:augustus_masked-contig009-processed-gene-5.93-mRNA-1
contig009	maker	CDS	587642	588274	.	-	0	ID=MALK_02538.t1.c1;Parent=MALK_02538.t1
contig009	maker	mRNA	587642	588274	.	-	.	ID=MALK_02538.t1;Parent=MALK_02538
contig009	maker	exon	587642	588274	.	-	.	ID=MALK_02538.t1.e1;Parent=MALK_02538.t1
contig009	maker	gene	588396	588605	.	+	.	ID=MALK_02539;prediction_source=maker_MRET:augustus_masked-contig009-processed-gene-5.72-mRNA-1
contig009	maker	CDS	588396	588605	.	+	0	ID=MALK_02539.t1.c1;Parent=MALK_02539.t1
contig009	maker	mRNA	588396	588605	.	+	.	ID=MALK_02539.t1;Parent=MALK_02539
contig009	maker	exon	588396	588605	.	+	.	ID=MALK_02539.t1.e1;Parent=MALK_02539.t1
contig009	maker	gene	588634	589629	.	-	.	ID=MALK_02540;prediction_source=maker_MRET:augustus_masked-contig009-processed-gene-5.94-mRNA-1
contig009	maker	CDS	588634	589629	.	-	0	ID=MALK_02540.t1.c1;Parent=MALK_02540.t1
contig009	maker	mRNA	588634	589629	.	-	.	ID=MALK_02540.t1;Parent=MALK_02540
contig009	maker	exon	588634	589629	.	-	.	ID=MALK_02540.t1.e1;Parent=MALK_02540.t1
contig009	AUGUSTUS	gene	589741	590703	0.98	+	.	ID=MALK_02541;prediction_source=braker_MRET:g2733.t1
contig009	AUGUSTUS	CDS	589741	590703	0.98	+	0	ID=MALK_02541.t1.c1;Parent=MALK_02541.t1
contig009	AUGUSTUS	mRNA	589741	590703	0.98	+	.	ID=MALK_02541.t1;Parent=MALK_02541
contig009	AUGUSTUS	exon	589741	590703	.	+	.	ID=MALK_02541.t1.e1;Parent=MALK_02541.t1
contig009	AUGUSTUS	gene	590768	591847	0.97	+	.	ID=MALK_02542;prediction_source=braker_MRET:g2734.t1

contig009	AUGUSTUS	CDS	590768	591847	0.97	+	0	ID=MALK_02542.t1.c1;Parent=MALK_02542.t1
contig009	AUGUSTUS	mRNA	590768	591847	0.97	+	.	ID=MALK_02542.t1;Parent=MALK_02542
contig009	AUGUSTUS	exon	590768	591847	.	+	.	ID=MALK_02542.t1.e1;Parent=MALK_02542.t1
contig009	AUGUSTUS	gene	591867	592739	0.96	+	.	ID=MALK_02543;prediction_source=braker_MRET:g2735.t1
contig009	AUGUSTUS	CDS	591867	592739	0.96	+	0	ID=MALK_02543.t1.c1;Parent=MALK_02543.t1
contig009	AUGUSTUS	mRNA	591867	592739	0.96	+	.	ID=MALK_02543.t1;Parent=MALK_02543
contig009	AUGUSTUS	exon	591867	592739	.	+	.	ID=MALK_02543.t1.e1;Parent=MALK_02543.t1
contig009	AUGUSTUS	gene	592791	593603	0.75	+	.	ID=MALK_02544;prediction_source=braker_MRET:g2736.t1
contig009	AUGUSTUS	CDS	592791	593603	0.75	+	0	ID=MALK_02544.t1.c1;Parent=MALK_02544.t1
contig009	AUGUSTUS	mRNA	592791	593603	0.75	+	.	ID=MALK_02544.t1;Parent=MALK_02544
contig009	AUGUSTUS	exon	592791	593603	.	+	.	ID=MALK_02544.t1.e1;Parent=MALK_02544.t1
contig009	maker	gene	593622	594554	.	-	.	ID=MALK_02545;prediction_source=maker_MRET:augustus_masked-contig009-processed-gene-6.91-mRNA-1
contig009	maker	CDS	593622	594554	.	-	0	ID=MALK_02545.t1.c1;Parent=MALK_02545.t1
contig009	maker	mRNA	593622	594554	.	-	.	ID=MALK_02545.t1;Parent=MALK_02545
contig009	maker	exon	593622	594554	.	-	.	ID=MALK_02545.t1.e1;Parent=MALK_02545.t1
contig009	maker	gene	596643	597581	.	+	.	ID=MALK_02546;prediction_source=maker_MRET:augustus_masked-contig009-processed-gene-6.89-mRNA-1
contig009	maker	CDS	596643	597581	.	+	0	ID=MALK_02546.t1.c1;Parent=MALK_02546.t1
contig009	maker	mRNA	596643	597581	.	+	.	ID=MALK_02546.t1;Parent=MALK_02546
contig009	maker	exon	596643	597581	.	+	.	ID=MALK_02546.t1.e1;Parent=MALK_02546.t1
contig009	AUGUSTUS	gene	597678	598169	0.59	-	.	ID=MALK_02547;prediction_source=braker_MRET:g2739.t1
contig009	AUGUSTUS	CDS	597678	598169	0.59	-	0	ID=MALK_02547.t1.c1;Parent=MALK_02547.t1
contig009	AUGUSTUS	mRNA	597678	598169	0.59	-	.	ID=MALK_02547.t1;Parent=MALK_02547
contig009	AUGUSTUS	exon	597678	598169	.	-	.	ID=MALK_02547.t1.e1;Parent=MALK_02547.t1
contig009	AUGUSTUS	gene	598444	600102	0.61	+	.	ID=MALK_02548;prediction_source=augustus:contig009.g2770.t1
contig009	AUGUSTUS	CDS	598444	600102	0.61	+	0	ID=MALK_02548.t1.c1;Parent=MALK_02548.t1
contig009	AUGUSTUS	mRNA	598444	600102	0.61	+	.	ID=MALK_02548.t1;Parent=MALK_02548
contig009	AUGUSTUS	exon	598444	600102	0.61	+	.	ID=MALK_02548.t1.e1;Parent=MALK_02548.t1
contig009	AUGUSTUS	gene	600145	601095	0.53	-	.	ID=MALK_02549;prediction_source=augustus:contig009.g2772.t1
contig009	AUGUSTUS	CDS	600145	601095	0.53	-	0	ID=MALK_02549.t1.c1;Parent=MALK_02549.t1
contig009	AUGUSTUS	mRNA	600145	601095	0.53	-	.	ID=MALK_02549.t1;Parent=MALK_02549
contig009	AUGUSTUS	exon	600145	601095	0.53	-	.	ID=MALK_02549.t1.e1;Parent=MALK_02549.t1
contig009	AUGUSTUS	gene	601227	603260	1	+	.	ID=MALK_02550;prediction_source=braker_MRET:g2742.t1
contig009	AUGUSTUS	CDS	601227	603260	1	+	0	ID=MALK_02550.t1.c1;Parent=MALK_02550.t1
contig009	AUGUSTUS	mRNA	601227	603260	1	+	.	ID=MALK_02550.t1;Parent=MALK_02550
contig009	AUGUSTUS	exon	601227	603260	.	+	.	ID=MALK_02550.t1.e1;Parent=MALK_02550.t1
contig009	AUGUSTUS	gene	603272	606184	1	-	.	ID=MALK_02551;prediction_source=augustus:contig009.g2773.t1
contig009	AUGUSTUS	CDS	603272	606184	1	-	0	ID=MALK_02551.t1.c1;Parent=MALK_02551.t1
contig009	AUGUSTUS	mRNA	603272	606184	1	-	.	ID=MALK_02551.t1;Parent=MALK_02551
contig009	AUGUSTUS	exon	603272	606184	1	-	.	ID=MALK_02551.t1.e1;Parent=MALK_02551.t1
contig009	maker	gene	606358	608199	.	+	.	ID=MALK_02552;prediction_source=maker_MRET:augustus_masked-contig009-processed-gene-6.93-mRNA-1
contig009	maker	CDS	606358	608199	.	+	0	ID=MALK_02552.t1.c1;Parent=MALK_02552.t1
contig009	maker	mRNA	606358	608199	.	+	.	ID=MALK_02552.t1;Parent=MALK_02552
contig009	maker	exon	606358	608199	.	+	.	ID=MALK_02552.t1.e1;Parent=MALK_02552.t1

contig009	AUGUSTUS	gene	608281	608898	0.52	+	.	ID=MALK_02553;prediction_source=augustus:contig009.g2776.t1
contig009	AUGUSTUS	CDS	608281	608898	0.52	+	0	ID=MALK_02553.t1.c1;Parent=MALK_02553.t1
contig009	AUGUSTUS	mRNA	608281	608898	0.52	+	.	ID=MALK_02553.t1;Parent=MALK_02553
contig009	AUGUSTUS	exon	608281	608898	0.52	+	.	ID=MALK_02553.t1.e1;Parent=MALK_02553.t1
contig009	maker	gene	608938	609666	.	-	.	ID=MALK_02554;prediction_source=maker_MRET:augustus_masked-contig009-processed-gene-6.129-mRNA-1
contig009	maker	CDS	608938	609666	.	-	0	ID=MALK_02554.t1.c1;Parent=MALK_02554.t1
contig009	maker	mRNA	608938	609666	.	-	.	ID=MALK_02554.t1;Parent=MALK_02554
contig009	maker	exon	608938	609666	.	-	.	ID=MALK_02554.t1.e1;Parent=MALK_02554.t1
contig009	AUGUSTUS	gene	609843	612215	0.8	+	.	ID=MALK_02555;prediction_source=augustus:contig009.g2778.t1
contig009	AUGUSTUS	CDS	609843	612215	0.8	+	0	ID=MALK_02555.t1.c1;Parent=MALK_02555.t1
contig009	AUGUSTUS	mRNA	609843	612215	0.8	+	.	ID=MALK_02555.t1;Parent=MALK_02555
contig009	AUGUSTUS	exon	609843	612215	0.8	+	.	ID=MALK_02555.t1.e1;Parent=MALK_02555.t1
contig009	AUGUSTUS	gene	612238	613668	0.88	-	.	ID=MALK_02556;prediction_source=augustus:contig009.g2781.t1
contig009	AUGUSTUS	CDS	612238	613668	0.88	-	0	ID=MALK_02556.t1.c1;Parent=MALK_02556.t1
contig009	AUGUSTUS	mRNA	612238	613668	0.88	-	.	ID=MALK_02556.t1;Parent=MALK_02556
contig009	AUGUSTUS	exon	612238	613668	0.88	-	.	ID=MALK_02556.t1.e1;Parent=MALK_02556.t1
contig009	AUGUSTUS	gene	613947	614924	0.99	-	.	ID=MALK_02557;prediction_source=augustus:contig009.g2783.t1
contig009	AUGUSTUS	CDS	613947	614924	0.99	-	0	ID=MALK_02557.t1.c1;Parent=MALK_02557.t1
contig009	AUGUSTUS	mRNA	613947	614924	0.99	-	.	ID=MALK_02557.t1;Parent=MALK_02557
contig009	AUGUSTUS	exon	613947	614924	0.99	-	.	ID=MALK_02557.t1.e1;Parent=MALK_02557.t1
contig009	AUGUSTUS	gene	615086	618577	0.99	+	.	ID=MALK_02558;prediction_source=braker_MRET:g2750.t1
contig009	AUGUSTUS	CDS	615086	618577	0.99	+	0	ID=MALK_02558.t1.c1;Parent=MALK_02558.t1
contig009	AUGUSTUS	mRNA	615086	618577	0.99	+	.	ID=MALK_02558.t1;Parent=MALK_02558
contig009	AUGUSTUS	exon	615086	618577	.	+	.	ID=MALK_02558.t1.e1;Parent=MALK_02558.t1
contig009	maker	gene	618600	620588	.	-	.	ID=MALK_02559;prediction_source=maker_MRET:augustus_masked-contig009-processed-gene-6.132-mRNA-1
contig009	maker	CDS	618600	620588	.	-	0	ID=MALK_02559.t1.c1;Parent=MALK_02559.t1
contig009	maker	mRNA	618600	620588	.	-	.	ID=MALK_02559.t1;Parent=MALK_02559
contig009	maker	exon	618600	620588	.	-	.	ID=MALK_02559.t1.e1;Parent=MALK_02559.t1
contig009	AUGUSTUS	gene	620751	622472	0.97	-	.	ID=MALK_02560;prediction_source=augustus:contig009.g2787.t1
contig009	AUGUSTUS	CDS	620751	622472	0.97	-	0	ID=MALK_02560.t1.c1;Parent=MALK_02560.t1
contig009	AUGUSTUS	mRNA	620751	622472	0.97	-	.	ID=MALK_02560.t1;Parent=MALK_02560
contig009	AUGUSTUS	exon	620751	622472	0.97	-	.	ID=MALK_02560.t1.e1;Parent=MALK_02560.t1
contig009	maker	gene	622760	625813	.	+	.	ID=MALK_02561;prediction_source=maker_MRET:augustus_masked-contig009-processed-gene-6.97-mRNA-1
contig009	maker	CDS	622760	625813	.	+	0	ID=MALK_02561.t1.c1;Parent=MALK_02561.t1
contig009	maker	mRNA	622760	625813	.	+	.	ID=MALK_02561.t1;Parent=MALK_02561
contig009	maker	exon	622760	625813	.	+	.	ID=MALK_02561.t1.e1;Parent=MALK_02561.t1
contig009	AUGUSTUS	gene	625854	626978	1	-	.	ID=MALK_02562;prediction_source=braker_MRET:g2754.t1
contig009	AUGUSTUS	CDS	625854	626978	1	-	0	ID=MALK_02562.t1.c1;Parent=MALK_02562.t1
contig009	AUGUSTUS	mRNA	625854	626978	1	-	.	ID=MALK_02562.t1;Parent=MALK_02562
contig009	AUGUSTUS	exon	625854	626978	.	-	.	ID=MALK_02562.t1.e1;Parent=MALK_02562.t1
contig009	AUGUSTUS	gene	627017	629815	0.56	-	.	ID=MALK_02563;prediction_source=augustus:contig009.g2789.t1
contig009	AUGUSTUS	CDS	627017	629815	0.56	-	0	ID=MALK_02563.t1.c1;Parent=MALK_02563.t1
contig009	AUGUSTUS	mRNA	627017	629815	0.56	-	.	ID=MALK_02563.t1;Parent=MALK_02563

contig009	AUGUSTUS	exon	627017	629815	0.56	-	.	ID=MALK_02563.t1.e1;Parent=MALK_02563.t1
contig009	maker	gene	630007	632916	.	+	.	ID=MALK_02564;prediction_source=maker_MRET:augustus_masked-contig009-processed-gene-6.98-mRNA-1
contig009	maker	CDS	630007	632916	.	+	0	ID=MALK_02564.t1.c1;Parent=MALK_02564.t1
contig009	maker	mRNA	630007	632916	.	+	.	ID=MALK_02564.t1;Parent=MALK_02564
contig009	maker	exon	630007	632916	.	+	.	ID=MALK_02564.t1.e1;Parent=MALK_02564.t1
contig009	AUGUSTUS	gene	633326	637927	0.28	+	.	ID=MALK_02565;prediction_source=augustus:contig009.g2791.t1
contig009	AUGUSTUS	CDS	633326	637927	0.28	+	0	ID=MALK_02565.t1.c1;Parent=MALK_02565.t1
contig009	AUGUSTUS	mRNA	633326	637927	0.28	+	.	ID=MALK_02565.t1;Parent=MALK_02565
contig009	AUGUSTUS	exon	633326	637927	0.28	+	.	ID=MALK_02565.t1.e1;Parent=MALK_02565.t1
contig009	maker	gene	638031	639002	.	+	.	ID=MALK_02566;prediction_source=maker_MRET:augustus_masked-contig009-processed-gene-6.100-mRNA-1
contig009	maker	CDS	638031	639002	.	+	0	ID=MALK_02566.t1.c1;Parent=MALK_02566.t1
contig009	maker	mRNA	638031	639002	.	+	.	ID=MALK_02566.t1;Parent=MALK_02566
contig009	maker	exon	638031	639002	.	+	.	ID=MALK_02566.t1.e1;Parent=MALK_02566.t1
contig009	AUGUSTUS	gene	639702	640901	0.36	+	.	ID=MALK_02567;prediction_source=augustus:contig009.g2793.t1
contig009	AUGUSTUS	CDS	639702	640901	0.36	+	0	ID=MALK_02567.t1.c1;Parent=MALK_02567.t1
contig009	AUGUSTUS	mRNA	639702	640901	0.36	+	.	ID=MALK_02567.t1;Parent=MALK_02567
contig009	AUGUSTUS	exon	639702	640901	0.36	+	.	ID=MALK_02567.t1.e1;Parent=MALK_02567.t1
contig009	AUGUSTUS	gene	641024	641644	0.72	+	.	ID=MALK_02568;prediction_source=augustus:contig009.g2794.t1
contig009	AUGUSTUS	CDS	641024	641644	0.72	+	0	ID=MALK_02568.t1.c1;Parent=MALK_02568.t1
contig009	AUGUSTUS	mRNA	641024	641644	0.72	+	.	ID=MALK_02568.t1;Parent=MALK_02568
contig009	AUGUSTUS	exon	641024	641644	0.72	+	.	ID=MALK_02568.t1.e1;Parent=MALK_02568.t1
contig009	maker	gene	642008	642634	.	+	.	ID=MALK_02569;prediction_source=maker_MRET:augustus_masked-contig009-processed-gene-6.103-mRNA-1
contig009	maker	CDS	642008	642634	.	+	0	ID=MALK_02569.t1.c1;Parent=MALK_02569.t1
contig009	maker	mRNA	642008	642634	.	+	.	ID=MALK_02569.t1;Parent=MALK_02569
contig009	maker	exon	642008	642634	.	+	.	ID=MALK_02569.t1.e1;Parent=MALK_02569.t1
contig009	AUGUSTUS	gene	642795	643847	0.76	-	.	ID=MALK_02570;prediction_source=braker_MRET:g2761.t1
contig009	AUGUSTUS	CDS	642795	643847	0.76	-	0	ID=MALK_02570.t1.c1;Parent=MALK_02570.t1
contig009	AUGUSTUS	mRNA	642795	643847	0.76	-	.	ID=MALK_02570.t1;Parent=MALK_02570
contig009	AUGUSTUS	exon	642795	643847	.	-	.	ID=MALK_02570.t1.e1;Parent=MALK_02570.t1
contig009	AUGUSTUS	gene	644374	645273	0.77	-	.	ID=MALK_02571;prediction_source=braker_MRET:g2762.t1
contig009	AUGUSTUS	CDS	644661	645273	0.88	-	0	ID=MALK_02571.t1.c3;Parent=MALK_02571.t1
contig009	AUGUSTUS	CDS	644541	644629	0.88	-	0	ID=MALK_02571.t1.c2;Parent=MALK_02571.t1
contig009	AUGUSTUS	CDS	644374	644508	0.88	-	0	ID=MALK_02571.t1.c1;Parent=MALK_02571.t1
contig009	AUGUSTUS	mRNA	644374	645273	0.77	-	.	ID=MALK_02571.t1;Parent=MALK_02571
contig009	AUGUSTUS	exon	644661	645273	.	-	.	ID=MALK_02571.t1.e3;Parent=MALK_02571.t1
contig009	AUGUSTUS	exon	644541	644629	.	-	.	ID=MALK_02571.t1.e2;Parent=MALK_02571.t1
contig009	AUGUSTUS	exon	644374	644508	.	-	.	ID=MALK_02571.t1.e1;Parent=MALK_02571.t1
contig009	AUGUSTUS	gene	645330	645771	0.47	+	.	ID=MALK_02572;prediction_source=augustus:contig009.g2797.t1
contig009	AUGUSTUS	CDS	645330	645353	0.48	+	0	ID=MALK_02572.t1.c1;Parent=MALK_02572.t1
contig009	AUGUSTUS	CDS	645418	645771	0.48	+	0	ID=MALK_02572.t1.c2;Parent=MALK_02572.t1
contig009	AUGUSTUS	mRNA	645330	645771	0.47	+	.	ID=MALK_02572.t1;Parent=MALK_02572
contig009	AUGUSTUS	exon	645330	645353	0.48	+	.	ID=MALK_02572.t1.e1;Parent=MALK_02572.t1
contig009	AUGUSTUS	exon	645418	645771	0.48	+	.	ID=MALK_02572.t1.e2;Parent=MALK_02572.t1

contig009	AUGUSTUS	gene	645810	646962	0.53	-	.	ID=MALK_02573;prediction_source=braker_MRET:g2763.t1
contig009	AUGUSTUS	CDS	646058	646962	0.99	-	0	ID=MALK_02573.t1.c3;Parent=MALK_02573.t1
contig009	AUGUSTUS	CDS	645963	646029	0.99	-	0	ID=MALK_02573.t1.c2;Parent=MALK_02573.t1
contig009	AUGUSTUS	CDS	645810	645923	0.99	-	0	ID=MALK_02573.t1.c1;Parent=MALK_02573.t1
contig009	AUGUSTUS	mRNA	645810	646962	0.53	-	.	ID=MALK_02573.t1;Parent=MALK_02573
contig009	AUGUSTUS	exon	646058	646962	.	-	.	ID=MALK_02573.t1.e3;Parent=MALK_02573.t1
contig009	AUGUSTUS	exon	645963	646029	.	-	.	ID=MALK_02573.t1.e2;Parent=MALK_02573.t1
contig009	AUGUSTUS	exon	645810	645923	.	-	.	ID=MALK_02573.t1.e1;Parent=MALK_02573.t1
contig009	AUGUSTUS	gene	647102	647513	0.95	+	.	ID=MALK_02574;prediction_source=braker_MRET:g2764.t1
contig009	AUGUSTUS	CDS	647102	647158	0.97	+	0	ID=MALK_02574.t1.c1;Parent=MALK_02574.t1
contig009	AUGUSTUS	CDS	647198	647214	0.97	+	0	ID=MALK_02574.t1.c2;Parent=MALK_02574.t1
contig009	AUGUSTUS	CDS	647324	647423	0.97	+	0	ID=MALK_02574.t1.c3;Parent=MALK_02574.t1
contig009	AUGUSTUS	CDS	647457	647513	0.97	+	0	ID=MALK_02574.t1.c4;Parent=MALK_02574.t1
contig009	AUGUSTUS	mRNA	647102	647513	0.95	+	.	ID=MALK_02574.t1;Parent=MALK_02574
contig009	AUGUSTUS	exon	647102	647158	.	+	.	ID=MALK_02574.t1.e1;Parent=MALK_02574.t1
contig009	AUGUSTUS	exon	647198	647214	.	+	.	ID=MALK_02574.t1.e2;Parent=MALK_02574.t1
contig009	AUGUSTUS	exon	647324	647423	.	+	.	ID=MALK_02574.t1.e3;Parent=MALK_02574.t1
contig009	AUGUSTUS	exon	647457	647513	.	+	.	ID=MALK_02574.t1.e4;Parent=MALK_02574.t1
contig009	AUGUSTUS	gene	647601	649499	0.98	-	.	ID=MALK_02575;prediction_source=braker_MRET:g2765.t1
contig009	AUGUSTUS	CDS	647601	649499	0.98	-	0	ID=MALK_02575.t1.c1;Parent=MALK_02575.t1
contig009	AUGUSTUS	mRNA	647601	649499	0.98	-	.	ID=MALK_02575.t1;Parent=MALK_02575
contig009	AUGUSTUS	exon	647601	649499	.	-	.	ID=MALK_02575.t1.e1;Parent=MALK_02575.t1
contig009	AUGUSTUS	gene	649579	650722	0.93	-	.	ID=MALK_02576;prediction_source=braker_MRET:g2766.t1
contig009	AUGUSTUS	CDS	650452	650722	0.95	-	0	ID=MALK_02576.t1.c2;Parent=MALK_02576.t1
contig009	AUGUSTUS	CDS	649579	650417	0.95	-	0	ID=MALK_02576.t1.c1;Parent=MALK_02576.t1
contig009	AUGUSTUS	mRNA	649579	650722	0.93	-	.	ID=MALK_02576.t1;Parent=MALK_02576
contig009	AUGUSTUS	exon	650452	650722	.	-	.	ID=MALK_02576.t1.e2;Parent=MALK_02576.t1
contig009	AUGUSTUS	exon	649579	650417	.	-	.	ID=MALK_02576.t1.e1;Parent=MALK_02576.t1
contig009	AUGUSTUS	gene	650830	651948	0.91	+	.	ID=MALK_02577;prediction_source=braker_MRET:g2767.t1
contig009	AUGUSTUS	CDS	650830	651948	0.91	+	0	ID=MALK_02577.t1.c1;Parent=MALK_02577.t1
contig009	AUGUSTUS	mRNA	650830	651948	0.91	+	.	ID=MALK_02577.t1;Parent=MALK_02577
contig009	AUGUSTUS	exon	650830	651948	.	+	.	ID=MALK_02577.t1.e1;Parent=MALK_02577.t1
contig009	AUGUSTUS	gene	651991	652765	0.14	-	.	ID=MALK_02578;prediction_source=braker_MRET:g2768.t1
contig009	AUGUSTUS	CDS	652717	652765	0.36	-	0	ID=MALK_02578.t1.c3;Parent=MALK_02578.t1
contig009	AUGUSTUS	CDS	652088	652687	0.36	-	0	ID=MALK_02578.t1.c2;Parent=MALK_02578.t1
contig009	AUGUSTUS	CDS	651991	652046	0.36	-	0	ID=MALK_02578.t1.c1;Parent=MALK_02578.t1
contig009	AUGUSTUS	mRNA	651991	652765	0.14	-	.	ID=MALK_02578.t1;Parent=MALK_02578
contig009	AUGUSTUS	exon	652717	652765	.	-	.	ID=MALK_02578.t1.e3;Parent=MALK_02578.t1
contig009	AUGUSTUS	exon	652088	652687	.	-	.	ID=MALK_02578.t1.e2;Parent=MALK_02578.t1
contig009	AUGUSTUS	exon	651991	652046	.	-	.	ID=MALK_02578.t1.e1;Parent=MALK_02578.t1
contig009	AUGUSTUS	gene	652907	653872	0.92	-	.	ID=MALK_02579;prediction_source=augustus:contig009.g2802.t1
contig009	AUGUSTUS	CDS	652907	653872	0.92	-	0	ID=MALK_02579.t1.c1;Parent=MALK_02579.t1
contig009	AUGUSTUS	mRNA	652907	653872	0.92	-	.	ID=MALK_02579.t1;Parent=MALK_02579

contig009	AUGUSTUS	exon	652907	653872	0.92	-	.	ID=MALK_02579.t1.e1;Parent=MALK_02579.t1
contig009	AUGUSTUS	gene	654010	655617	1	-	.	ID=MALK_02580;prediction_source=augustus:contig009.g2803.t1
contig009	AUGUSTUS	CDS	654010	655617	1	-	0	ID=MALK_02580.t1.c1;Parent=MALK_02580.t1
contig009	AUGUSTUS	mRNA	654010	655617	1	-	.	ID=MALK_02580.t1;Parent=MALK_02580
contig009	AUGUSTUS	exon	654010	655617	1	-	.	ID=MALK_02580.t1.e1;Parent=MALK_02580.t1
contig009	AUGUSTUS	gene	655706	656938	0.38	-	.	ID=MALK_02581;prediction_source=braker_MRET:g2771.t1
contig009	AUGUSTUS	CDS	656936	656938	0.47	-	0	ID=MALK_02581.t1.c4;Parent=MALK_02581.t1
contig009	AUGUSTUS	CDS	656575	656907	0.47	-	0	ID=MALK_02581.t1.c3;Parent=MALK_02581.t1
contig009	AUGUSTUS	CDS	655976	656532	0.47	-	0	ID=MALK_02581.t1.c2;Parent=MALK_02581.t1
contig009	AUGUSTUS	CDS	655706	655811	0.47	-	0	ID=MALK_02581.t1.c1;Parent=MALK_02581.t1
contig009	AUGUSTUS	mRNA	655706	656938	0.38	-	.	ID=MALK_02581.t1;Parent=MALK_02581
contig009	AUGUSTUS	exon	656936	656938	.	-	.	ID=MALK_02581.t1.e4;Parent=MALK_02581.t1
contig009	AUGUSTUS	exon	656575	656907	.	-	.	ID=MALK_02581.t1.e3;Parent=MALK_02581.t1
contig009	AUGUSTUS	exon	655976	656532	.	-	.	ID=MALK_02581.t1.e2;Parent=MALK_02581.t1
contig009	AUGUSTUS	exon	655706	655811	.	-	.	ID=MALK_02581.t1.e1;Parent=MALK_02581.t1
contig009	AUGUSTUS	gene	657080	657787	1	+	.	ID=MALK_02582;prediction_source=braker_MRET:g2772.t1
contig009	AUGUSTUS	CDS	657080	657787	1	+	0	ID=MALK_02582.t1.c1;Parent=MALK_02582.t1
contig009	AUGUSTUS	mRNA	657080	657787	1	+	.	ID=MALK_02582.t1;Parent=MALK_02582
contig009	AUGUSTUS	exon	657080	657787	.	+	.	ID=MALK_02582.t1.e1;Parent=MALK_02582.t1
contig009	AUGUSTUS	gene	657795	661619	0.38	-	.	ID=MALK_02583;prediction_source=augustus:contig009.g2807.t1
contig009	AUGUSTUS	CDS	657795	661619	0.38	-	0	ID=MALK_02583.t1.c1;Parent=MALK_02583.t1
contig009	AUGUSTUS	mRNA	657795	661619	0.38	-	.	ID=MALK_02583.t1;Parent=MALK_02583
contig009	AUGUSTUS	exon	657795	661619	0.38	-	.	ID=MALK_02583.t1.e1;Parent=MALK_02583.t1
contig009	maker	gene	661845	662933	.	-	.	ID=MALK_02584;prediction_source=maker_MRET:augustus_masked-contig009-processed-gene-6.143-mRNA-1
contig009	maker	CDS	661845	662933	.	-	0	ID=MALK_02584.t1.c1;Parent=MALK_02584.t1
contig009	maker	mRNA	661845	662933	.	-	.	ID=MALK_02584.t1;Parent=MALK_02584
contig009	maker	exon	661845	662933	.	-	.	ID=MALK_02584.t1.e1;Parent=MALK_02584.t1
contig009	AUGUSTUS	gene	663564	664296	0.4	-	.	ID=MALK_02585;prediction_source=braker_MRET:g2775.t1
contig009	AUGUSTUS	CDS	664294	664296	0.85	-	0	ID=MALK_02585.t1.c3;Parent=MALK_02585.t1
contig009	AUGUSTUS	CDS	663875	664193	0.85	-	0	ID=MALK_02585.t1.c2;Parent=MALK_02585.t1
contig009	AUGUSTUS	CDS	663564	663844	0.85	-	0	ID=MALK_02585.t1.c1;Parent=MALK_02585.t1
contig009	AUGUSTUS	mRNA	663564	664296	0.4	-	.	ID=MALK_02585.t1;Parent=MALK_02585
contig009	AUGUSTUS	exon	664294	664296	.	-	.	ID=MALK_02585.t1.e3;Parent=MALK_02585.t1
contig009	AUGUSTUS	exon	663875	664193	.	-	.	ID=MALK_02585.t1.e2;Parent=MALK_02585.t1
contig009	AUGUSTUS	exon	663564	663844	.	-	.	ID=MALK_02585.t1.e1;Parent=MALK_02585.t1
contig009	AUGUSTUS	gene	664640	665989	0.66	+	.	ID=MALK_02586;prediction_source=augustus:contig009.g2810.t1
contig009	AUGUSTUS	CDS	664640	665989	0.66	+	0	ID=MALK_02586.t1.c1;Parent=MALK_02586.t1
contig009	AUGUSTUS	mRNA	664640	665989	0.66	+	.	ID=MALK_02586.t1;Parent=MALK_02586
contig009	AUGUSTUS	exon	664640	665989	0.66	+	.	ID=MALK_02586.t1.e1;Parent=MALK_02586.t1
contig009	AUGUSTUS	gene	665982	667625	1	-	.	ID=MALK_02587;prediction_source=augustus:contig009.g2811.t1
contig009	AUGUSTUS	CDS	665982	667625	1	-	0	ID=MALK_02587.t1.c1;Parent=MALK_02587.t1
contig009	AUGUSTUS	mRNA	665982	667625	1	-	.	ID=MALK_02587.t1;Parent=MALK_02587
contig009	AUGUSTUS	exon	665982	667625	1	-	.	ID=MALK_02587.t1.e1;Parent=MALK_02587.t1

contig009	AUGUSTUS	gene	667789	668613	0.78	+	.	ID=MALK_02588;prediction_source=braker_MRET:g2778.t1
contig009	AUGUSTUS	CDS	667789	668613	0.78	+	0	ID=MALK_02588.t1.c1;Parent=MALK_02588.t1
contig009	AUGUSTUS	mRNA	667789	668613	0.78	+	.	ID=MALK_02588.t1;Parent=MALK_02588
contig009	AUGUSTUS	exon	667789	668613	.	+	.	ID=MALK_02588.t1.e1;Parent=MALK_02588.t1
contig009	maker	gene	668629	669069	.	-	.	ID=MALK_02589;prediction_source=maker_MRET:augustus_masked-contig009-processed-gene-6.145-mRNA-1
contig009	maker	CDS	668629	669069	.	-	0	ID=MALK_02589.t1.c1;Parent=MALK_02589.t1
contig009	maker	mRNA	668629	669069	.	-	.	ID=MALK_02589.t1;Parent=MALK_02589
contig009	maker	exon	668629	669069	.	-	.	ID=MALK_02589.t1.e1;Parent=MALK_02589.t1
contig009	maker	gene	669367	672978	.	+	.	ID=MALK_02590;prediction_source=maker_MRET:augustus_masked-contig009-processed-gene-6.106-mRNA-1
contig009	maker	CDS	669367	672978	.	+	0	ID=MALK_02590.t1.c1;Parent=MALK_02590.t1
contig009	maker	mRNA	669367	672978	.	+	.	ID=MALK_02590.t1;Parent=MALK_02590
contig009	maker	exon	669367	672978	.	+	.	ID=MALK_02590.t1.e1;Parent=MALK_02590.t1
contig009	AUGUSTUS	gene	673087	673452	0.87	+	.	ID=MALK_02591;prediction_source=braker_MRET:g2781.t1
contig009	AUGUSTUS	CDS	673087	673092	0.87	+	0	ID=MALK_02591.t1.c1;Parent=MALK_02591.t1
contig009	AUGUSTUS	CDS	673126	673452	0.87	+	0	ID=MALK_02591.t1.c2;Parent=MALK_02591.t1
contig009	AUGUSTUS	mRNA	673087	673452	0.87	+	.	ID=MALK_02591.t1;Parent=MALK_02591
contig009	AUGUSTUS	exon	673087	673092	.	+	.	ID=MALK_02591.t1.e1;Parent=MALK_02591.t1
contig009	AUGUSTUS	exon	673126	673452	.	+	.	ID=MALK_02591.t1.e2;Parent=MALK_02591.t1
contig009	AUGUSTUS	gene	673557	675258	0.98	+	.	ID=MALK_02592;prediction_source=braker_MRET:g2782.t1
contig009	AUGUSTUS	CDS	673557	675211	1	+	0	ID=MALK_02592.t1.c1;Parent=MALK_02592.t1
contig009	AUGUSTUS	CDS	675246	675258	1	+	0	ID=MALK_02592.t1.c2;Parent=MALK_02592.t1
contig009	AUGUSTUS	mRNA	673557	675258	0.98	+	.	ID=MALK_02592.t1;Parent=MALK_02592
contig009	AUGUSTUS	exon	673557	675211	.	+	.	ID=MALK_02592.t1.e1;Parent=MALK_02592.t1
contig009	AUGUSTUS	exon	675246	675258	.	+	.	ID=MALK_02592.t1.e2;Parent=MALK_02592.t1
contig009	AUGUSTUS	gene	675262	675879	0.18	-	.	ID=MALK_02593;prediction_source=augustus:contig009.g2816.t1
contig009	AUGUSTUS	CDS	675262	675879	0.18	-	0	ID=MALK_02593.t1.c1;Parent=MALK_02593.t1
contig009	AUGUSTUS	mRNA	675262	675879	0.18	-	.	ID=MALK_02593.t1;Parent=MALK_02593
contig009	AUGUSTUS	exon	675262	675879	0.18	-	.	ID=MALK_02593.t1.e1;Parent=MALK_02593.t1
contig009	AUGUSTUS	gene	676200	677096	0.53	+	.	ID=MALK_02594;prediction_source=augustus:contig009.g2817.t1
contig009	AUGUSTUS	CDS	676200	677096	0.53	+	0	ID=MALK_02594.t1.c1;Parent=MALK_02594.t1
contig009	AUGUSTUS	mRNA	676200	677096	0.53	+	.	ID=MALK_02594.t1;Parent=MALK_02594
contig009	AUGUSTUS	exon	676200	677096	0.53	+	.	ID=MALK_02594.t1.e1;Parent=MALK_02594.t1
contig009	AUGUSTUS	gene	677171	678298	1	+	.	ID=MALK_02595;prediction_source=augustus:contig009.g2818.t1
contig009	AUGUSTUS	CDS	677171	678298	1	+	0	ID=MALK_02595.t1.c1;Parent=MALK_02595.t1
contig009	AUGUSTUS	mRNA	677171	678298	1	+	.	ID=MALK_02595.t1;Parent=MALK_02595
contig009	AUGUSTUS	exon	677171	678298	1	+	.	ID=MALK_02595.t1.e1;Parent=MALK_02595.t1
contig009	AUGUSTUS	gene	678295	679911	0.73	-	.	ID=MALK_02596;prediction_source=augustus:contig009.g2819.t1
contig009	AUGUSTUS	CDS	678295	679911	0.73	-	0	ID=MALK_02596.t1.c1;Parent=MALK_02596.t1
contig009	AUGUSTUS	mRNA	678295	679911	0.73	-	.	ID=MALK_02596.t1;Parent=MALK_02596
contig009	AUGUSTUS	exon	678295	679911	0.73	-	.	ID=MALK_02596.t1.e1;Parent=MALK_02596.t1
contig009	AUGUSTUS	gene	679954	681177	0.92	-	.	ID=MALK_02597;prediction_source=augustus:contig009.g2820.t1
contig009	AUGUSTUS	CDS	679954	681177	0.92	-	0	ID=MALK_02597.t1.c1;Parent=MALK_02597.t1
contig009	AUGUSTUS	mRNA	679954	681177	0.92	-	.	ID=MALK_02597.t1;Parent=MALK_02597

contig009	AUGUSTUS	exon	679954	681177	0.92	-	.	ID=MALK_02597.t1.e1;Parent=MALK_02597.t1
contig009	AUGUSTUS	gene	681307	682359	0.8	+	.	ID=MALK_02598;prediction_source=augustus:contig009.g2821.t1
contig009	AUGUSTUS	CDS	681307	682359	0.8	+	0	ID=MALK_02598.t1.c1;Parent=MALK_02598.t1
contig009	AUGUSTUS	mRNA	681307	682359	0.8	+	.	ID=MALK_02598.t1;Parent=MALK_02598
contig009	AUGUSTUS	exon	681307	682359	0.8	+	.	ID=MALK_02598.t1.e1;Parent=MALK_02598.t1
contig009	maker	gene	682336	684600	.	-	.	ID=MALK_02599;prediction_source=maker_MRET:augustus_masked-contig009-processed-gene-6.149-mRNA-1
contig009	maker	CDS	682336	684600	.	-	0	ID=MALK_02599.t1.c1;Parent=MALK_02599.t1
contig009	maker	mRNA	682336	684600	.	-	.	ID=MALK_02599.t1;Parent=MALK_02599
contig009	maker	exon	682336	684600	.	-	.	ID=MALK_02599.t1.e1;Parent=MALK_02599.t1
contig009	AUGUSTUS	gene	684853	685838	0.23	+	.	ID=MALK_02600;prediction_source=braker_MRET:g2790.t1
contig009	AUGUSTUS	CDS	684853	684886	0.85	+	0	ID=MALK_02600.t1.c1;Parent=MALK_02600.t1
contig009	AUGUSTUS	CDS	684919	684950	0.85	+	0	ID=MALK_02600.t1.c2;Parent=MALK_02600.t1
contig009	AUGUSTUS	CDS	684991	685058	0.85	+	0	ID=MALK_02600.t1.c3;Parent=MALK_02600.t1
contig009	AUGUSTUS	CDS	685094	685196	0.85	+	0	ID=MALK_02600.t1.c4;Parent=MALK_02600.t1
contig009	AUGUSTUS	CDS	685412	685624	0.85	+	0	ID=MALK_02600.t1.c5;Parent=MALK_02600.t1
contig009	AUGUSTUS	CDS	685653	685838	0.85	+	0	ID=MALK_02600.t1.c6;Parent=MALK_02600.t1
contig009	AUGUSTUS	mRNA	684853	685838	0.23	+	.	ID=MALK_02600.t1;Parent=MALK_02600
contig009	AUGUSTUS	exon	684853	684886	.	+	.	ID=MALK_02600.t1.e1;Parent=MALK_02600.t1
contig009	AUGUSTUS	exon	684919	684950	.	+	.	ID=MALK_02600.t1.e2;Parent=MALK_02600.t1
contig009	AUGUSTUS	exon	684991	685058	.	+	.	ID=MALK_02600.t1.e3;Parent=MALK_02600.t1
contig009	AUGUSTUS	exon	685094	685196	.	+	.	ID=MALK_02600.t1.e4;Parent=MALK_02600.t1
contig009	AUGUSTUS	exon	685412	685624	.	+	.	ID=MALK_02600.t1.e5;Parent=MALK_02600.t1
contig009	AUGUSTUS	exon	685653	685838	.	+	.	ID=MALK_02600.t1.e6;Parent=MALK_02600.t1
contig009	AUGUSTUS	gene	686026	687096	0.34	-	.	ID=MALK_02601;prediction_source=augustus:contig009.g2823.t1
contig009	AUGUSTUS	CDS	686026	687096	0.34	-	0	ID=MALK_02601.t1.c1;Parent=MALK_02601.t1
contig009	AUGUSTUS	mRNA	686026	687096	0.34	-	.	ID=MALK_02601.t1;Parent=MALK_02601
contig009	AUGUSTUS	exon	686026	687096	0.34	-	.	ID=MALK_02601.t1.e1;Parent=MALK_02601.t1
contig009	AUGUSTUS	gene	687211	688815	0.49	-	.	ID=MALK_02602;prediction_source=braker_MRET:g2792.t1
contig009	AUGUSTUS	CDS	687211	688815	0.49	-	0	ID=MALK_02602.t1.c1;Parent=MALK_02602.t1
contig009	AUGUSTUS	mRNA	687211	688815	0.49	-	.	ID=MALK_02602.t1;Parent=MALK_02602
contig009	AUGUSTUS	exon	687211	688815	.	-	.	ID=MALK_02602.t1.e1;Parent=MALK_02602.t1
contig009	AUGUSTUS	gene	688963	690348	0.53	+	.	ID=MALK_02603;prediction_source=braker_MRET:g2793.t1
contig009	AUGUSTUS	CDS	688963	690348	0.53	+	0	ID=MALK_02603.t1.c1;Parent=MALK_02603.t1
contig009	AUGUSTUS	mRNA	688963	690348	0.53	+	.	ID=MALK_02603.t1;Parent=MALK_02603
contig009	AUGUSTUS	exon	688963	690348	.	+	.	ID=MALK_02603.t1.e1;Parent=MALK_02603.t1
contig009	AUGUSTUS	gene	690452	690903	0.69	-	.	ID=MALK_02604;prediction_source=braker_MRET:g2794.t1
contig009	AUGUSTUS	CDS	690547	690903	0.71	-	0	ID=MALK_02604.t1.c2;Parent=MALK_02604.t1
contig009	AUGUSTUS	CDS	690452	690460	0.71	-	0	ID=MALK_02604.t1.c1;Parent=MALK_02604.t1
contig009	AUGUSTUS	mRNA	690452	690903	0.69	-	.	ID=MALK_02604.t1;Parent=MALK_02604
contig009	AUGUSTUS	exon	690547	690903	.	-	.	ID=MALK_02604.t1.e2;Parent=MALK_02604.t1
contig009	AUGUSTUS	exon	690452	690460	.	-	.	ID=MALK_02604.t1.e1;Parent=MALK_02604.t1
contig009	AUGUSTUS	gene	690985	692895	1	+	.	ID=MALK_02605;prediction_source=braker_MRET:g2795.t1
contig009	AUGUSTUS	CDS	690985	692895	1	+	0	ID=MALK_02605.t1.c1;Parent=MALK_02605.t1

contig009	AUGUSTUS	mRNA	690985	692895	1	+	.	ID=MALK_02605.t1;Parent=MALK_02605
contig009	AUGUSTUS	exon	690985	692895	.	+	.	ID=MALK_02605.t1.e1;Parent=MALK_02605.t1
contig009	AUGUSTUS	gene	693399	697490	0.61	+	.	ID=MALK_02606;prediction_source=augustus:contig009.g2825.t1
contig009	AUGUSTUS	CDS	693399	697490	0.61	+	0	ID=MALK_02606.t1.c1;Parent=MALK_02606.t1
contig009	AUGUSTUS	mRNA	693399	697490	0.61	+	.	ID=MALK_02606.t1;Parent=MALK_02606
contig009	AUGUSTUS	exon	693399	697490	0.61	+	.	ID=MALK_02606.t1.e1;Parent=MALK_02606.t1
contig009	AUGUSTUS	gene	697506	701504	0.64	-	.	ID=MALK_02607;prediction_source=braker_MRET:g2797.t1
contig009	AUGUSTUS	CDS	701464	701504	0.66	-	0	ID=MALK_02607.t1.c3;Parent=MALK_02607.t1
contig009	AUGUSTUS	CDS	701344	701393	0.66	-	0	ID=MALK_02607.t1.c2;Parent=MALK_02607.t1
contig009	AUGUSTUS	CDS	697506	701311	0.66	-	0	ID=MALK_02607.t1.c1;Parent=MALK_02607.t1
contig009	AUGUSTUS	mRNA	697506	701504	0.64	-	.	ID=MALK_02607.t1;Parent=MALK_02607
contig009	AUGUSTUS	exon	701464	701504	.	-	.	ID=MALK_02607.t1.e3;Parent=MALK_02607.t1
contig009	AUGUSTUS	exon	701344	701393	.	-	.	ID=MALK_02607.t1.e2;Parent=MALK_02607.t1
contig009	AUGUSTUS	exon	697506	701311	.	-	.	ID=MALK_02607.t1.e1;Parent=MALK_02607.t1
contig009	maker	gene	701696	703132	.	+	.	ID=MALK_02608;prediction_source=maker_MRET:augustus_masked-contig009-processed-gene-6.111-mRNA-1
contig009	maker	CDS	701696	703132	.	+	0	ID=MALK_02608.t1.c1;Parent=MALK_02608.t1
contig009	maker	mRNA	701696	703132	.	+	.	ID=MALK_02608.t1;Parent=MALK_02608
contig009	maker	exon	701696	703132	.	+	.	ID=MALK_02608.t1.e1;Parent=MALK_02608.t1
contig009	AUGUSTUS	gene	703445	704829	0.91	-	.	ID=MALK_02609;prediction_source=braker_MRET:g2799.t1
contig009	AUGUSTUS	CDS	704188	704829	0.96	-	0	ID=MALK_02609.t1.c3;Parent=MALK_02609.t1
contig009	AUGUSTUS	CDS	703496	704025	0.96	-	0	ID=MALK_02609.t1.c2;Parent=MALK_02609.t1
contig009	AUGUSTUS	CDS	703445	703463	0.96	-	0	ID=MALK_02609.t1.c1;Parent=MALK_02609.t1
contig009	AUGUSTUS	mRNA	703445	704829	0.91	-	.	ID=MALK_02609.t1;Parent=MALK_02609
contig009	AUGUSTUS	exon	704188	704829	.	-	.	ID=MALK_02609.t1.e3;Parent=MALK_02609.t1
contig009	AUGUSTUS	exon	703496	704025	.	-	.	ID=MALK_02609.t1.e2;Parent=MALK_02609.t1
contig009	AUGUSTUS	exon	703445	703463	.	-	.	ID=MALK_02609.t1.e1;Parent=MALK_02609.t1
contig009	maker	gene	705085	705447	.	+	.	ID=MALK_02610;prediction_source=maker_MRET:augustus_masked-contig009-processed-gene-6.112-mRNA-1
contig009	maker	CDS	705085	705447	.	+	0	ID=MALK_02610.t1.c1;Parent=MALK_02610.t1
contig009	maker	mRNA	705085	705447	.	+	.	ID=MALK_02610.t1;Parent=MALK_02610
contig009	maker	exon	705085	705447	.	+	.	ID=MALK_02610.t1.e1;Parent=MALK_02610.t1
contig009	AUGUSTUS	gene	705566	706453	0.51	-	.	ID=MALK_02611;prediction_source=augustus:contig009.g2831.t1
contig009	AUGUSTUS	CDS	705566	706453	0.51	-	0	ID=MALK_02611.t1.c1;Parent=MALK_02611.t1
contig009	AUGUSTUS	mRNA	705566	706453	0.51	-	.	ID=MALK_02611.t1;Parent=MALK_02611
contig009	AUGUSTUS	exon	705566	706453	0.51	-	.	ID=MALK_02611.t1.e1;Parent=MALK_02611.t1
contig009	maker	gene	706818	709136	.	+	.	ID=MALK_02612;prediction_source=maker_MRET:augustus_masked-contig009-processed-gene-6.113-mRNA-1
contig009	maker	CDS	706818	709136	.	+	0	ID=MALK_02612.t1.c1;Parent=MALK_02612.t1
contig009	maker	mRNA	706818	709136	.	+	.	ID=MALK_02612.t1;Parent=MALK_02612
contig009	maker	exon	706818	709136	.	+	.	ID=MALK_02612.t1.e1;Parent=MALK_02612.t1
contig009	maker	gene	709160	711745	.	-	.	ID=MALK_02613;prediction_source=maker_MRET:augustus_masked-contig009-processed-gene-6.154-mRNA-1
contig009	maker	CDS	709160	711745	.	-	0	ID=MALK_02613.t1.c1;Parent=MALK_02613.t1
contig009	maker	mRNA	709160	711745	.	-	.	ID=MALK_02613.t1;Parent=MALK_02613
contig009	maker	exon	709160	711745	.	-	.	ID=MALK_02613.t1.e1;Parent=MALK_02613.t1
contig009	maker	gene	712275	713495	.	-	.	ID=MALK_02614;prediction_source=maker_MRET:augustus_masked-contig009-processed-gene-6.155-mRNA-1

contig009	maker	CDS	712275	713495	.	-	0	ID=MALK_02614.t1.c1;Parent=MALK_02614.t1
contig009	maker	mRNA	712275	713495	.	-	.	ID=MALK_02614.t1;Parent=MALK_02614
contig009	maker	exon	712275	713495	.	-	.	ID=MALK_02614.t1.e1;Parent=MALK_02614.t1
contig009	AUGUSTUS	gene	713766	715034	0.55	-	.	ID=MALK_02615;prediction_source=braker_MRET:g2805.t1
contig009	AUGUSTUS	CDS	715029	715034	0.63	-	0	ID=MALK_02615.t1.c4;Parent=MALK_02615.t1
contig009	AUGUSTUS	CDS	714942	714986	0.63	-	0	ID=MALK_02615.t1.c3;Parent=MALK_02615.t1
contig009	AUGUSTUS	CDS	714862	714910	0.63	-	0	ID=MALK_02615.t1.c2;Parent=MALK_02615.t1
contig009	AUGUSTUS	CDS	713766	714832	0.63	-	0	ID=MALK_02615.t1.c1;Parent=MALK_02615.t1
contig009	AUGUSTUS	mRNA	713766	715034	0.55	-	.	ID=MALK_02615.t1;Parent=MALK_02615
contig009	AUGUSTUS	exon	715029	715034	.	-	.	ID=MALK_02615.t1.e4;Parent=MALK_02615.t1
contig009	AUGUSTUS	exon	714942	714986	.	-	.	ID=MALK_02615.t1.e3;Parent=MALK_02615.t1
contig009	AUGUSTUS	exon	714862	714910	.	-	.	ID=MALK_02615.t1.e2;Parent=MALK_02615.t1
contig009	AUGUSTUS	exon	713766	714832	.	-	.	ID=MALK_02615.t1.e1;Parent=MALK_02615.t1
contig009	AUGUSTUS	gene	715136	718663	0.23	+	.	ID=MALK_02616;prediction_source=augustus:contig009.g2838.t1
contig009	AUGUSTUS	CDS	715136	716626	0.37	+	0	ID=MALK_02616.t1.c1;Parent=MALK_02616.t1
contig009	AUGUSTUS	CDS	717365	718663	0.37	+	0	ID=MALK_02616.t1.c2;Parent=MALK_02616.t1
contig009	AUGUSTUS	mRNA	715136	718663	0.23	+	.	ID=MALK_02616.t1;Parent=MALK_02616
contig009	AUGUSTUS	exon	715136	716626	0.37	+	.	ID=MALK_02616.t1.e1;Parent=MALK_02616.t1
contig009	AUGUSTUS	exon	717365	718663	0.37	+	.	ID=MALK_02616.t1.e2;Parent=MALK_02616.t1
contig009	maker	gene	719077	719784	.	-	.	ID=MALK_02617;prediction_source=maker_MRET:augustus_masked-contig009-processed-gene-6.157-mRNA-1
contig009	maker	CDS	719077	719784	.	-	0	ID=MALK_02617.t1.c1;Parent=MALK_02617.t1
contig009	maker	mRNA	719077	719784	.	-	.	ID=MALK_02617.t1;Parent=MALK_02617
contig009	maker	exon	719077	719784	.	-	.	ID=MALK_02617.t1.e1;Parent=MALK_02617.t1
contig009	AUGUSTUS	gene	720738	721733	0.57	+	.	ID=MALK_02618;prediction_source=augustus:contig009.g2840.t1
contig009	AUGUSTUS	CDS	720738	721733	0.57	+	0	ID=MALK_02618.t1.c1;Parent=MALK_02618.t1
contig009	AUGUSTUS	mRNA	720738	721733	0.57	+	.	ID=MALK_02618.t1;Parent=MALK_02618
contig009	AUGUSTUS	exon	720738	721733	0.57	+	.	ID=MALK_02618.t1.e1;Parent=MALK_02618.t1
contig009	AUGUSTUS	gene	722231	723718	1	+	.	ID=MALK_02619;prediction_source=augustus:contig009.g2842.t1
contig009	AUGUSTUS	CDS	722231	723718	1	+	0	ID=MALK_02619.t1.c1;Parent=MALK_02619.t1
contig009	AUGUSTUS	mRNA	722231	723718	1	+	.	ID=MALK_02619.t1;Parent=MALK_02619
contig009	AUGUSTUS	exon	722231	723718	1	+	.	ID=MALK_02619.t1.e1;Parent=MALK_02619.t1
contig009	AUGUSTUS	gene	723681	728897	0.57	-	.	ID=MALK_02620;prediction_source=augustus:contig009.g2843.t1
contig009	AUGUSTUS	CDS	723681	728897	0.57	-	0	ID=MALK_02620.t1.c1;Parent=MALK_02620.t1
contig009	AUGUSTUS	mRNA	723681	728897	0.57	-	.	ID=MALK_02620.t1;Parent=MALK_02620
contig009	AUGUSTUS	exon	723681	728897	0.57	-	.	ID=MALK_02620.t1.e1;Parent=MALK_02620.t1
contig009	AUGUSTUS	gene	728896	730437	1	+	.	ID=MALK_02621;prediction_source=augustus:contig009.g2845.t1
contig009	AUGUSTUS	CDS	728896	730437	1	+	0	ID=MALK_02621.t1.c1;Parent=MALK_02621.t1
contig009	AUGUSTUS	mRNA	728896	730437	1	+	.	ID=MALK_02621.t1;Parent=MALK_02621
contig009	AUGUSTUS	exon	728896	730437	1	+	.	ID=MALK_02621.t1.e1;Parent=MALK_02621.t1
contig009	AUGUSTUS	gene	730434	732329	1	-	.	ID=MALK_02622;prediction_source=augustus:contig009.g2846.t1
contig009	AUGUSTUS	CDS	730434	732329	1	-	0	ID=MALK_02622.t1.c1;Parent=MALK_02622.t1
contig009	AUGUSTUS	mRNA	730434	732329	1	-	.	ID=MALK_02622.t1;Parent=MALK_02622
contig009	AUGUSTUS	exon	730434	732329	1	-	.	ID=MALK_02622.t1.e1;Parent=MALK_02622.t1

contig009	AUGUSTUS	gene	732448	733255	0.95	+	.	ID=MALK_02623;prediction_source=braker_MRET:g2813.t1
contig009	AUGUSTUS	CDS	732448	733154	0.95	+	0	ID=MALK_02623.t1.c1;Parent=MALK_02623.t1
contig009	AUGUSTUS	CDS	733243	733255	0.95	+	0	ID=MALK_02623.t1.c2;Parent=MALK_02623.t1
contig009	AUGUSTUS	mRNA	732448	733255	0.95	+	.	ID=MALK_02623.t1;Parent=MALK_02623
contig009	AUGUSTUS	exon	732448	733154	.	+	.	ID=MALK_02623.t1.e1;Parent=MALK_02623.t1
contig009	AUGUSTUS	exon	733243	733255	.	+	.	ID=MALK_02623.t1.e2;Parent=MALK_02623.t1
contig009	maker	gene	733320	734678	.	-	.	ID=MALK_02624;prediction_source=maker_MRET:augustus_masked-contig009-processed-gene-6.160-mRNA-1
contig009	maker	CDS	733320	734678	.	-	0	ID=MALK_02624.t1.c1;Parent=MALK_02624.t1
contig009	maker	mRNA	733320	734678	.	-	.	ID=MALK_02624.t1;Parent=MALK_02624
contig009	maker	exon	733320	734678	.	-	.	ID=MALK_02624.t1.e1;Parent=MALK_02624.t1
contig009	AUGUSTUS	gene	734783	735369	0.89	+	.	ID=MALK_02625;prediction_source=braker_MRET:g2815.t1
contig009	AUGUSTUS	CDS	734783	734791	0.97	+	0	ID=MALK_02625.t1.c1;Parent=MALK_02625.t1
contig009	AUGUSTUS	CDS	734820	734889	0.97	+	0	ID=MALK_02625.t1.c2;Parent=MALK_02625.t1
contig009	AUGUSTUS	CDS	734927	735369	0.97	+	0	ID=MALK_02625.t1.c3;Parent=MALK_02625.t1
contig009	AUGUSTUS	mRNA	734783	735369	0.89	+	.	ID=MALK_02625.t1;Parent=MALK_02625
contig009	AUGUSTUS	exon	734783	734791	.	+	.	ID=MALK_02625.t1.e1;Parent=MALK_02625.t1
contig009	AUGUSTUS	exon	734820	734889	.	+	.	ID=MALK_02625.t1.e2;Parent=MALK_02625.t1
contig009	AUGUSTUS	exon	734927	735369	.	+	.	ID=MALK_02625.t1.e3;Parent=MALK_02625.t1
contig009	maker	gene	735373	736347	.	-	.	ID=MALK_02626;prediction_source=maker_MRET:augustus_masked-contig009-processed-gene-6.161-mRNA-1
contig009	maker	CDS	735373	736347	.	-	0	ID=MALK_02626.t1.c1;Parent=MALK_02626.t1
contig009	maker	mRNA	735373	736347	.	-	.	ID=MALK_02626.t1;Parent=MALK_02626
contig009	maker	exon	735373	736347	.	-	.	ID=MALK_02626.t1.e1;Parent=MALK_02626.t1
contig009	AUGUSTUS	gene	736484	738046	0.85	+	.	ID=MALK_02627;prediction_source=braker_MRET:g2817.t1
contig009	AUGUSTUS	CDS	736484	736508	0.85	+	0	ID=MALK_02627.t1.c1;Parent=MALK_02627.t1
contig009	AUGUSTUS	CDS	736584	738046	0.85	+	0	ID=MALK_02627.t1.c2;Parent=MALK_02627.t1
contig009	AUGUSTUS	mRNA	736484	738046	0.85	+	.	ID=MALK_02627.t1;Parent=MALK_02627
contig009	AUGUSTUS	exon	736484	736508	.	+	.	ID=MALK_02627.t1.e1;Parent=MALK_02627.t1
contig009	AUGUSTUS	exon	736584	738046	.	+	.	ID=MALK_02627.t1.e2;Parent=MALK_02627.t1
contig009	AUGUSTUS	gene	738198	743816	0.99	+	.	ID=MALK_02628;prediction_source=braker_MRET:g2818.t1
contig009	AUGUSTUS	CDS	738198	743816	0.99	+	0	ID=MALK_02628.t1.c1;Parent=MALK_02628.t1
contig009	AUGUSTUS	mRNA	738198	743816	0.99	+	.	ID=MALK_02628.t1;Parent=MALK_02628
contig009	AUGUSTUS	exon	738198	743816	.	+	.	ID=MALK_02628.t1.e1;Parent=MALK_02628.t1
contig009	maker	gene	744097	745872	.	+	.	ID=MALK_02629;prediction_source=maker_MRET:augustus_masked-contig009-processed-gene-6.120-mRNA-1
contig009	maker	CDS	744097	745872	.	+	0	ID=MALK_02629.t1.c1;Parent=MALK_02629.t1
contig009	maker	mRNA	744097	745872	.	+	.	ID=MALK_02629.t1;Parent=MALK_02629
contig009	maker	exon	744097	745872	.	+	.	ID=MALK_02629.t1.e1;Parent=MALK_02629.t1
contig009	AUGUSTUS	gene	745919	746454	0.67	-	.	ID=MALK_02630;prediction_source=braker_MRET:g2820.t1
contig009	AUGUSTUS	CDS	746039	746454	0.68	-	0	ID=MALK_02630.t1.c2;Parent=MALK_02630.t1
contig009	AUGUSTUS	CDS	745919	745994	0.68	-	0	ID=MALK_02630.t1.c1;Parent=MALK_02630.t1
contig009	AUGUSTUS	mRNA	745919	746454	0.67	-	.	ID=MALK_02630.t1;Parent=MALK_02630
contig009	AUGUSTUS	exon	746039	746454	.	-	.	ID=MALK_02630.t1.e2;Parent=MALK_02630.t1
contig009	AUGUSTUS	exon	745919	745994	.	-	.	ID=MALK_02630.t1.e1;Parent=MALK_02630.t1
contig009	AUGUSTUS	gene	746951	747784	0.76	-	.	ID=MALK_02631;prediction_source=braker_MRET:g2821.t1

contig009	AUGUSTUS	CDS	746951	747784	0.76	-	0	ID=MALK_02631.t1.c1;Parent=MALK_02631.t1
contig009	AUGUSTUS	mRNA	746951	747784	0.76	-	.	ID=MALK_02631.t1;Parent=MALK_02631
contig009	AUGUSTUS	exon	746951	747784	.	-	.	ID=MALK_02631.t1.e1;Parent=MALK_02631.t1
contig009	maker	gene	747843	751316	.	+	.	ID=MALK_02632;prediction_source=maker_MRET:augustus_masked-contig009-processed-gene-6.121-mRNA-1
contig009	maker	CDS	747843	751316	.	+	0	ID=MALK_02632.t1.c1;Parent=MALK_02632.t1
contig009	maker	mRNA	747843	751316	.	+	.	ID=MALK_02632.t1;Parent=MALK_02632
contig009	maker	exon	747843	751316	.	+	.	ID=MALK_02632.t1.e1;Parent=MALK_02632.t1
contig009	maker	gene	751322	752536	.	-	.	ID=MALK_02633;prediction_source=maker_MRET:augustus_masked-contig009-processed-gene-6.163-mRNA-1
contig009	maker	CDS	751322	752536	.	-	0	ID=MALK_02633.t1.c1;Parent=MALK_02633.t1
contig009	maker	mRNA	751322	752536	.	-	.	ID=MALK_02633.t1;Parent=MALK_02633
contig009	maker	exon	751322	752536	.	-	.	ID=MALK_02633.t1.e1;Parent=MALK_02633.t1
contig009	AUGUSTUS	gene	752971	756339	0.99	+	.	ID=MALK_02634;prediction_source=augustus:contig009.g2858.t1
contig009	AUGUSTUS	CDS	752971	756339	0.99	+	0	ID=MALK_02634.t1.c1;Parent=MALK_02634.t1
contig009	AUGUSTUS	mRNA	752971	756339	0.99	+	.	ID=MALK_02634.t1;Parent=MALK_02634
contig009	AUGUSTUS	exon	752971	756339	0.99	+	.	ID=MALK_02634.t1.e1;Parent=MALK_02634.t1
contig009	AUGUSTUS	gene	756364	757260	0.55	-	.	ID=MALK_02635;prediction_source=augustus:contig009.g2859.t1
contig009	AUGUSTUS	CDS	756364	757260	0.55	-	0	ID=MALK_02635.t1.c1;Parent=MALK_02635.t1
contig009	AUGUSTUS	mRNA	756364	757260	0.55	-	.	ID=MALK_02635.t1;Parent=MALK_02635
contig009	AUGUSTUS	exon	756364	757260	0.55	-	.	ID=MALK_02635.t1.e1;Parent=MALK_02635.t1
contig009	AUGUSTUS	gene	757303	757875	0.8	+	.	ID=MALK_02636;prediction_source=augustus:contig009.g2860.t1
contig009	AUGUSTUS	CDS	757303	757875	0.8	+	0	ID=MALK_02636.t1.c1;Parent=MALK_02636.t1
contig009	AUGUSTUS	mRNA	757303	757875	0.8	+	.	ID=MALK_02636.t1;Parent=MALK_02636
contig009	AUGUSTUS	exon	757303	757875	0.8	+	.	ID=MALK_02636.t1.e1;Parent=MALK_02636.t1
contig009	maker	gene	757879	758796	.	-	.	ID=MALK_02637;prediction_source=maker_MRET:augustus_masked-contig009-processed-gene-6.165-mRNA-1
contig009	maker	CDS	757879	758796	.	-	0	ID=MALK_02637.t1.c1;Parent=MALK_02637.t1
contig009	maker	mRNA	757879	758796	.	-	.	ID=MALK_02637.t1;Parent=MALK_02637
contig009	maker	exon	757879	758796	.	-	.	ID=MALK_02637.t1.e1;Parent=MALK_02637.t1
contig009	AUGUSTUS	gene	759331	761361	0.95	+	.	ID=MALK_02638;prediction_source=augustus:contig009.g2862.t1
contig009	AUGUSTUS	CDS	759331	761361	0.95	+	0	ID=MALK_02638.t1.c1;Parent=MALK_02638.t1
contig009	AUGUSTUS	mRNA	759331	761361	0.95	+	.	ID=MALK_02638.t1;Parent=MALK_02638
contig009	AUGUSTUS	exon	759331	761361	0.95	+	.	ID=MALK_02638.t1.e1;Parent=MALK_02638.t1
contig009	maker	gene	762334	763476	.	+	.	ID=MALK_02639;prediction_source=maker_MRET:augustus_masked-contig009-processed-gene-6.125-mRNA-1
contig009	maker	CDS	762334	763476	.	+	0	ID=MALK_02639.t1.c1;Parent=MALK_02639.t1
contig009	maker	mRNA	762334	763476	.	+	.	ID=MALK_02639.t1;Parent=MALK_02639
contig009	maker	exon	762334	763476	.	+	.	ID=MALK_02639.t1.e1;Parent=MALK_02639.t1
contig009	AUGUSTUS	gene	764725	765912	1	-	.	ID=MALK_02640;prediction_source=augustus:contig009.g2865.t1
contig009	AUGUSTUS	CDS	764725	765912	1	-	0	ID=MALK_02640.t1.c1;Parent=MALK_02640.t1
contig009	AUGUSTUS	mRNA	764725	765912	1	-	.	ID=MALK_02640.t1;Parent=MALK_02640
contig009	AUGUSTUS	exon	764725	765912	1	-	.	ID=MALK_02640.t1.e1;Parent=MALK_02640.t1
contig009	AUGUSTUS	gene	767586	768032	0.67	+	.	ID=MALK_02641;prediction_source=braker_MRET:g2831.t1
contig009	AUGUSTUS	CDS	767586	767616	0.76	+	0	ID=MALK_02641.t1.c1;Parent=MALK_02641.t1
contig009	AUGUSTUS	CDS	767725	768032	0.76	+	0	ID=MALK_02641.t1.c2;Parent=MALK_02641.t1
contig009	AUGUSTUS	mRNA	767586	768032	0.67	+	.	ID=MALK_02641.t1;Parent=MALK_02641

contig009	AUGUSTUS	exon	767586	767616	.	+	.	ID=MALK_02641.t1.e1;Parent=MALK_02641.t1
contig009	AUGUSTUS	exon	767725	768032	.	+	.	ID=MALK_02641.t1.e2;Parent=MALK_02641.t1
contig009	AUGUSTUS	gene	770153	771343	1	-	.	ID=MALK_02642;prediction_source=augustus:contig009.g2867.t1
contig009	AUGUSTUS	CDS	770153	771343	1	-	0	ID=MALK_02642.t1.c1;Parent=MALK_02642.t1
contig009	AUGUSTUS	mRNA	770153	771343	1	-	.	ID=MALK_02642.t1;Parent=MALK_02642
contig009	AUGUSTUS	exon	770153	771343	1	-	.	ID=MALK_02642.t1.e1;Parent=MALK_02642.t1
contig009	AUGUSTUS	gene	773620	773877	0.78	-	.	ID=MALK_02643;prediction_source=augustus:contig009.g2869.t1
contig009	AUGUSTUS	CDS	773620	773877	0.78	-	0	ID=MALK_02643.t1.c1;Parent=MALK_02643.t1
contig009	AUGUSTUS	mRNA	773620	773877	0.78	-	.	ID=MALK_02643.t1;Parent=MALK_02643
contig009	AUGUSTUS	exon	773620	773877	0.78	-	.	ID=MALK_02643.t1.e1;Parent=MALK_02643.t1
contig010	annotation	remark	1	826862	.	+	.	gff-version=3
contig010	AUGUSTUS	gene	55	333	0.97	-	.	ID=MALK_02644;prediction_source=augustus:contig010.g913.t1
contig010	AUGUSTUS	CDS	55	333	0.97	-	0	ID=MALK_02644.t1.c1;Parent=MALK_02644.t1
contig010	AUGUSTUS	mRNA	55	333	0.97	-	.	ID=MALK_02644.t1;Parent=MALK_02644
contig010	AUGUSTUS	exon	55	333	0.97	-	.	ID=MALK_02644.t1.e1;Parent=MALK_02644.t1
contig010	AUGUSTUS	gene	1803	3218	0.9	-	.	ID=MALK_02645;prediction_source=augustus:contig010.g914.t1
contig010	AUGUSTUS	CDS	1803	3218	0.9	-	0	ID=MALK_02645.t1.c1;Parent=MALK_02645.t1
contig010	AUGUSTUS	mRNA	1803	3218	0.9	-	.	ID=MALK_02645.t1;Parent=MALK_02645
contig010	AUGUSTUS	exon	1803	3218	0.9	-	.	ID=MALK_02645.t1.e1;Parent=MALK_02645.t1
contig010	maker	gene	3287	5104	.	-	.	ID=MALK_02646;prediction_source=maker_MRET:augustus_masked-contig010-processed-gene-0.25-mRNA-1
contig010	maker	CDS	3287	5104	.	-	0	ID=MALK_02646.t1.c1;Parent=MALK_02646.t1
contig010	maker	mRNA	3287	5104	.	-	.	ID=MALK_02646.t1;Parent=MALK_02646
contig010	maker	exon	3287	5104	.	-	.	ID=MALK_02646.t1.e1;Parent=MALK_02646.t1
contig010	maker	gene	5213	7120	.	-	.	ID=MALK_02647;prediction_source=maker_MRET:augustus_masked-contig010-processed-gene-0.26-mRNA-1
contig010	maker	CDS	5213	7120	.	-	0	ID=MALK_02647.t1.c1;Parent=MALK_02647.t1
contig010	maker	mRNA	5213	7120	.	-	.	ID=MALK_02647.t1;Parent=MALK_02647
contig010	maker	exon	5213	7120	.	-	.	ID=MALK_02647.t1.e1;Parent=MALK_02647.t1
contig010	maker	gene	7656	8057	.	-	.	ID=MALK_02648;prediction_source=maker_MRET:augustus_masked-contig010-processed-gene-0.27-mRNA-1
contig010	maker	CDS	7656	8057	.	-	0	ID=MALK_02648.t1.c1;Parent=MALK_02648.t1
contig010	maker	mRNA	7656	8057	.	-	.	ID=MALK_02648.t1;Parent=MALK_02648
contig010	maker	exon	7656	8057	.	-	.	ID=MALK_02648.t1.e1;Parent=MALK_02648.t1
contig010	AUGUSTUS	gene	8557	9026	0.52	+	.	ID=MALK_02649;prediction_source=braker_MRET:g1210.t1
contig010	AUGUSTUS	CDS	8557	8611	0.59	+	0	ID=MALK_02649.t1.c1;Parent=MALK_02649.t1
contig010	AUGUSTUS	CDS	8647	8708	0.59	+	0	ID=MALK_02649.t1.c2;Parent=MALK_02649.t1
contig010	AUGUSTUS	CDS	8755	8879	0.59	+	0	ID=MALK_02649.t1.c3;Parent=MALK_02649.t1
contig010	AUGUSTUS	CDS	8912	9026	0.59	+	0	ID=MALK_02649.t1.c4;Parent=MALK_02649.t1
contig010	AUGUSTUS	mRNA	8557	9026	0.52	+	.	ID=MALK_02649.t1;Parent=MALK_02649
contig010	AUGUSTUS	exon	8557	8611	.	+	.	ID=MALK_02649.t1.e1;Parent=MALK_02649.t1
contig010	AUGUSTUS	exon	8647	8708	.	+	.	ID=MALK_02649.t1.e2;Parent=MALK_02649.t1
contig010	AUGUSTUS	exon	8755	8879	.	+	.	ID=MALK_02649.t1.e3;Parent=MALK_02649.t1
contig010	AUGUSTUS	exon	8912	9026	.	+	.	ID=MALK_02649.t1.e4;Parent=MALK_02649.t1
contig010	AUGUSTUS	gene	9040	10590	0.35	-	.	ID=MALK_02650;prediction_source=braker_MRET:g1211.t1
contig010	AUGUSTUS	CDS	9720	10590	0.42	-	0	ID=MALK_02650.t1.c3;Parent=MALK_02650.t1

contig010	AUGUSTUS	CDS	9293	9495	0.42	-	0	ID=MALK_02650.t1.c2;Parent=MALK_02650.t1
contig010	AUGUSTUS	CDS	9040	9264	0.42	-	0	ID=MALK_02650.t1.c1;Parent=MALK_02650.t1
contig010	AUGUSTUS	mRNA	9040	10590	0.35	-	.	ID=MALK_02650.t1;Parent=MALK_02650
contig010	AUGUSTUS	exon	9720	10590	.	-	.	ID=MALK_02650.t1.e3;Parent=MALK_02650.t1
contig010	AUGUSTUS	exon	9293	9495	.	-	.	ID=MALK_02650.t1.e2;Parent=MALK_02650.t1
contig010	AUGUSTUS	exon	9040	9264	.	-	.	ID=MALK_02650.t1.e1;Parent=MALK_02650.t1
contig010	maker	gene	10846	12975	.	-	.	ID=MALK_02651;prediction_source=maker_MRET:augustus_masked-contig010-processed-gene-0.29-mRNA-1
contig010	maker	CDS	10846	12975	.	-	0	ID=MALK_02651.t1.c1;Parent=MALK_02651.t1
contig010	maker	mRNA	10846	12975	.	-	.	ID=MALK_02651.t1;Parent=MALK_02651
contig010	maker	exon	10846	12975	.	-	.	ID=MALK_02651.t1.e1;Parent=MALK_02651.t1
contig010	maker	gene	14687	17328	.	-	.	ID=MALK_02652;prediction_source=maker_MRET:augustus_masked-contig010-processed-gene-0.30-mRNA-1
contig010	maker	CDS	17255	17328	.	-	0	ID=MALK_02652.t1.c1;Parent=MALK_02652.t1
contig010	maker	CDS	14687	16982	.	-	0	ID=MALK_02652.t1.c2;Parent=MALK_02652.t1
contig010	maker	mRNA	14687	17328	.	-	.	ID=MALK_02652.t1;Parent=MALK_02652
contig010	maker	exon	17255	17328	.	-	.	ID=MALK_02652.t1.e1;Parent=MALK_02652.t1
contig010	maker	exon	14687	16982	.	-	.	ID=MALK_02652.t1.e2;Parent=MALK_02652.t1
contig010	maker	gene	17770	18318	.	-	.	ID=MALK_02653;prediction_source=maker_MRET:augustus_masked-contig010-processed-gene-0.31-mRNA-1
contig010	maker	CDS	17770	18318	.	-	0	ID=MALK_02653.t1.c1;Parent=MALK_02653.t1
contig010	maker	mRNA	17770	18318	.	-	.	ID=MALK_02653.t1;Parent=MALK_02653
contig010	maker	exon	17770	18318	.	-	.	ID=MALK_02653.t1.e1;Parent=MALK_02653.t1
contig010	AUGUSTUS	gene	18862	19920	0.98	+	.	ID=MALK_02654;prediction_source=augustus:contig010.g923.t1
contig010	AUGUSTUS	CDS	18862	19920	0.98	+	0	ID=MALK_02654.t1.c1;Parent=MALK_02654.t1
contig010	AUGUSTUS	mRNA	18862	19920	0.98	+	.	ID=MALK_02654.t1;Parent=MALK_02654
contig010	AUGUSTUS	exon	18862	19920	0.98	+	.	ID=MALK_02654.t1.e1;Parent=MALK_02654.t1
contig010	maker	gene	20057	20809	.	+	.	ID=MALK_02655;prediction_source=maker_MRET:augustus_masked-contig010-processed-gene-0.1-mRNA-1
contig010	maker	CDS	20057	20809	.	+	0	ID=MALK_02655.t1.c1;Parent=MALK_02655.t1
contig010	maker	mRNA	20057	20809	.	+	.	ID=MALK_02655.t1;Parent=MALK_02655
contig010	maker	exon	20057	20809	.	+	.	ID=MALK_02655.t1.e1;Parent=MALK_02655.t1
contig010	maker	gene	20811	21656	.	-	.	ID=MALK_02656;prediction_source=maker_MRET:augustus_masked-contig010-processed-gene-0.32-mRNA-1
contig010	maker	CDS	20811	21656	.	-	0	ID=MALK_02656.t1.c1;Parent=MALK_02656.t1
contig010	maker	mRNA	20811	21656	.	-	.	ID=MALK_02656.t1;Parent=MALK_02656
contig010	maker	exon	20811	21656	.	-	.	ID=MALK_02656.t1.e1;Parent=MALK_02656.t1
contig010	AUGUSTUS	gene	21693	23549	0.71	+	.	ID=MALK_02657;prediction_source=augustus:contig010.g926.t1
contig010	AUGUSTUS	CDS	21693	23549	0.71	+	0	ID=MALK_02657.t1.c1;Parent=MALK_02657.t1
contig010	AUGUSTUS	mRNA	21693	23549	0.71	+	.	ID=MALK_02657.t1;Parent=MALK_02657
contig010	AUGUSTUS	exon	21693	23549	0.71	+	.	ID=MALK_02657.t1.e1;Parent=MALK_02657.t1
contig010	AUGUSTUS	gene	23687	24421	0.57	+	.	ID=MALK_02658;prediction_source=augustus:contig010.g927.t1
contig010	AUGUSTUS	CDS	23687	24421	0.57	+	0	ID=MALK_02658.t1.c1;Parent=MALK_02658.t1
contig010	AUGUSTUS	mRNA	23687	24421	0.57	+	.	ID=MALK_02658.t1;Parent=MALK_02658
contig010	AUGUSTUS	exon	23687	24421	0.57	+	.	ID=MALK_02658.t1.e1;Parent=MALK_02658.t1
contig010	maker	gene	24502	25548	.	+	.	ID=MALK_02659;prediction_source=maker_MRET:augustus_masked-contig010-processed-gene-0.4-mRNA-1
contig010	maker	CDS	24502	25548	.	+	0	ID=MALK_02659.t1.c1;Parent=MALK_02659.t1
contig010	maker	mRNA	24502	25548	.	+	.	ID=MALK_02659.t1;Parent=MALK_02659

contig010	maker	exon	24502	25548	.	+	.	ID=MALK_02659.t1.e1;Parent=MALK_02659.t1
contig010	AUGUSTUS	gene	25720	26595	0.97	+	.	ID=MALK_02660;prediction_source=augustus:contig010.g930.t1
contig010	AUGUSTUS	CDS	25720	26595	0.97	+	0	ID=MALK_02660.t1.c1;Parent=MALK_02660.t1
contig010	AUGUSTUS	mRNA	25720	26595	0.97	+	.	ID=MALK_02660.t1;Parent=MALK_02660
contig010	AUGUSTUS	exon	25720	26595	0.97	+	.	ID=MALK_02660.t1.e1;Parent=MALK_02660.t1
contig010	maker	gene	26714	28411	.	-	.	ID=MALK_02661;prediction_source=maker_MRET:augustus_masked-contig010-processed-gene-0.33-mRNA-1
contig010	maker	CDS	26714	28411	.	-	0	ID=MALK_02661.t1.c1;Parent=MALK_02661.t1
contig010	maker	mRNA	26714	28411	.	-	.	ID=MALK_02661.t1;Parent=MALK_02661
contig010	maker	exon	26714	28411	.	-	.	ID=MALK_02661.t1.e1;Parent=MALK_02661.t1
contig010	AUGUSTUS	gene	28972	29826	0.69	+	.	ID=MALK_02662;prediction_source=augustus:contig010.g932.t1
contig010	AUGUSTUS	CDS	28972	29826	0.69	+	0	ID=MALK_02662.t1.c1;Parent=MALK_02662.t1
contig010	AUGUSTUS	mRNA	28972	29826	0.69	+	.	ID=MALK_02662.t1;Parent=MALK_02662
contig010	AUGUSTUS	exon	28972	29826	0.69	+	.	ID=MALK_02662.t1.e1;Parent=MALK_02662.t1
contig010	AUGUSTUS	gene	29830	30570	0.43	-	.	ID=MALK_02663;prediction_source=augustus:contig010.g933.t1
contig010	AUGUSTUS	CDS	29830	30570	0.43	-	0	ID=MALK_02663.t1.c1;Parent=MALK_02663.t1
contig010	AUGUSTUS	mRNA	29830	30570	0.43	-	.	ID=MALK_02663.t1;Parent=MALK_02663
contig010	AUGUSTUS	exon	29830	30570	0.43	-	.	ID=MALK_02663.t1.e1;Parent=MALK_02663.t1
contig010	maker	gene	30833	31504	.	-	.	ID=MALK_02664;prediction_source=maker_MRET:augustus_masked-contig010-processed-gene-0.35-mRNA-1
contig010	maker	CDS	30833	31504	.	-	0	ID=MALK_02664.t1.c1;Parent=MALK_02664.t1
contig010	maker	mRNA	30833	31504	.	-	.	ID=MALK_02664.t1;Parent=MALK_02664
contig010	maker	exon	30833	31504	.	-	.	ID=MALK_02664.t1.e1;Parent=MALK_02664.t1
contig010	maker	gene	31578	32870	.	+	.	ID=MALK_02665;prediction_source=maker_MRET:augustus_masked-contig010-processed-gene-0.7-mRNA-1
contig010	maker	CDS	31578	32870	.	+	0	ID=MALK_02665.t1.c1;Parent=MALK_02665.t1
contig010	maker	mRNA	31578	32870	.	+	.	ID=MALK_02665.t1;Parent=MALK_02665
contig010	maker	exon	31578	32870	.	+	.	ID=MALK_02665.t1.e1;Parent=MALK_02665.t1
contig010	AUGUSTUS	gene	32867	33835	0.4	-	.	ID=MALK_02666;prediction_source=augustus:contig010.g936.t1
contig010	AUGUSTUS	CDS	32867	33835	0.4	-	0	ID=MALK_02666.t1.c1;Parent=MALK_02666.t1
contig010	AUGUSTUS	mRNA	32867	33835	0.4	-	.	ID=MALK_02666.t1;Parent=MALK_02666
contig010	AUGUSTUS	exon	32867	33835	0.4	-	.	ID=MALK_02666.t1.e1;Parent=MALK_02666.t1
contig010	AUGUSTUS	gene	33936	35538	0.47	+	.	ID=MALK_02667;prediction_source=braker_MRET:g1227.t1
contig010	AUGUSTUS	CDS	33936	33957	0.7	+	0	ID=MALK_02667.t1.c1;Parent=MALK_02667.t1
contig010	AUGUSTUS	CDS	34058	35538	0.7	+	0	ID=MALK_02667.t1.c2;Parent=MALK_02667.t1
contig010	AUGUSTUS	mRNA	33936	35538	0.47	+	.	ID=MALK_02667.t1;Parent=MALK_02667
contig010	AUGUSTUS	exon	33936	33957	.	+	.	ID=MALK_02667.t1.e1;Parent=MALK_02667.t1
contig010	AUGUSTUS	exon	34058	35538	.	+	.	ID=MALK_02667.t1.e2;Parent=MALK_02667.t1
contig010	maker	gene	35747	38821	.	+	.	ID=MALK_02668;prediction_source=maker_MRET:augustus_masked-contig010-processed-gene-0.9-mRNA-1
contig010	maker	CDS	35747	38821	.	+	0	ID=MALK_02668.t1.c1;Parent=MALK_02668.t1
contig010	maker	mRNA	35747	38821	.	+	.	ID=MALK_02668.t1;Parent=MALK_02668
contig010	maker	exon	35747	38821	.	+	.	ID=MALK_02668.t1.e1;Parent=MALK_02668.t1
contig010	AUGUSTUS	gene	38923	40395	1	-	.	ID=MALK_02669;prediction_source=braker_MRET:g1229.t1
contig010	AUGUSTUS	CDS	38923	40395	1	-	0	ID=MALK_02669.t1.c1;Parent=MALK_02669.t1
contig010	AUGUSTUS	mRNA	38923	40395	1	-	.	ID=MALK_02669.t1;Parent=MALK_02669
contig010	AUGUSTUS	exon	38923	40395	.	-	.	ID=MALK_02669.t1.e1;Parent=MALK_02669.t1

contig010	AUGUSTUS	gene	40511	42097	1	-	.	ID=MALK_02670;prediction_source=braker_MRET:g1230.t1
contig010	AUGUSTUS	CDS	42058	42097	1	-	0	ID=MALK_02670.t1.c3;Parent=MALK_02670.t1
contig010	AUGUSTUS	CDS	41976	42016	1	-	0	ID=MALK_02670.t1.c2;Parent=MALK_02670.t1
contig010	AUGUSTUS	CDS	40511	41941	1	-	0	ID=MALK_02670.t1.c1;Parent=MALK_02670.t1
contig010	AUGUSTUS	mRNA	40511	42097	1	-	.	ID=MALK_02670.t1;Parent=MALK_02670
contig010	AUGUSTUS	exon	42058	42097	.	-	.	ID=MALK_02670.t1.e3;Parent=MALK_02670.t1
contig010	AUGUSTUS	exon	41976	42016	.	-	.	ID=MALK_02670.t1.e2;Parent=MALK_02670.t1
contig010	AUGUSTUS	exon	40511	41941	.	-	.	ID=MALK_02670.t1.e1;Parent=MALK_02670.t1
contig010	AUGUSTUS	gene	42198	42801	0.53	+	.	ID=MALK_02671;prediction_source=braker_MRET:g1231.t1
contig010	AUGUSTUS	CDS	42198	42234	1	+	0	ID=MALK_02671.t1.c1;Parent=MALK_02671.t1
contig010	AUGUSTUS	CDS	42279	42349	1	+	0	ID=MALK_02671.t1.c2;Parent=MALK_02671.t1
contig010	AUGUSTUS	CDS	42395	42456	1	+	0	ID=MALK_02671.t1.c3;Parent=MALK_02671.t1
contig010	AUGUSTUS	CDS	42502	42629	1	+	0	ID=MALK_02671.t1.c4;Parent=MALK_02671.t1
contig010	AUGUSTUS	CDS	42671	42801	1	+	0	ID=MALK_02671.t1.c5;Parent=MALK_02671.t1
contig010	AUGUSTUS	mRNA	42198	42801	0.53	+	.	ID=MALK_02671.t1;Parent=MALK_02671
contig010	AUGUSTUS	exon	42198	42234	.	+	.	ID=MALK_02671.t1.e1;Parent=MALK_02671.t1
contig010	AUGUSTUS	exon	42279	42349	.	+	.	ID=MALK_02671.t1.e2;Parent=MALK_02671.t1
contig010	AUGUSTUS	exon	42395	42456	.	+	.	ID=MALK_02671.t1.e3;Parent=MALK_02671.t1
contig010	AUGUSTUS	exon	42502	42629	.	+	.	ID=MALK_02671.t1.e4;Parent=MALK_02671.t1
contig010	AUGUSTUS	exon	42671	42801	.	+	.	ID=MALK_02671.t1.e5;Parent=MALK_02671.t1
contig010	AUGUSTUS	gene	42983	45007	0.75	-	.	ID=MALK_02672;prediction_source=augustus:contig010.g940.t1
contig010	AUGUSTUS	CDS	42983	45007	0.75	-	0	ID=MALK_02672.t1.c1;Parent=MALK_02672.t1
contig010	AUGUSTUS	mRNA	42983	45007	0.75	-	.	ID=MALK_02672.t1;Parent=MALK_02672
contig010	AUGUSTUS	exon	42983	45007	0.75	-	.	ID=MALK_02672.t1.e1;Parent=MALK_02672.t1
contig010	maker	gene	45152	46480	.	+	.	ID=MALK_02673;prediction_source=maker_MRET:augustus_masked-contig010-processed-gene-0.10-mRNA-1
contig010	maker	CDS	45152	46480	.	+	0	ID=MALK_02673.t1.c1;Parent=MALK_02673.t1
contig010	maker	mRNA	45152	46480	.	+	.	ID=MALK_02673.t1;Parent=MALK_02673
contig010	maker	exon	45152	46480	.	+	.	ID=MALK_02673.t1.e1;Parent=MALK_02673.t1
contig010	AUGUSTUS	gene	46555	47268	0.73	-	.	ID=MALK_02674;prediction_source=augustus:contig010.g943.t1
contig010	AUGUSTUS	CDS	46555	47268	0.73	-	0	ID=MALK_02674.t1.c1;Parent=MALK_02674.t1
contig010	AUGUSTUS	mRNA	46555	47268	0.73	-	.	ID=MALK_02674.t1;Parent=MALK_02674
contig010	AUGUSTUS	exon	46555	47268	0.73	-	.	ID=MALK_02674.t1.e1;Parent=MALK_02674.t1
contig010	AUGUSTUS	gene	47417	48052	0.89	-	.	ID=MALK_02675;prediction_source=braker_MRET:g1235.t1
contig010	AUGUSTUS	CDS	47896	48052	0.98	-	0	ID=MALK_02675.t1.c2;Parent=MALK_02675.t1
contig010	AUGUSTUS	CDS	47417	47868	0.98	-	0	ID=MALK_02675.t1.c1;Parent=MALK_02675.t1
contig010	AUGUSTUS	mRNA	47417	48052	0.89	-	.	ID=MALK_02675.t1;Parent=MALK_02675
contig010	AUGUSTUS	exon	47896	48052	.	-	.	ID=MALK_02675.t1.e2;Parent=MALK_02675.t1
contig010	AUGUSTUS	exon	47417	47868	.	-	.	ID=MALK_02675.t1.e1;Parent=MALK_02675.t1
contig010	maker	gene	48176	50638	.	-	.	ID=MALK_02676;prediction_source=maker_MRET:augustus_masked-contig010-processed-gene-0.39-mRNA-1
contig010	maker	CDS	48176	50638	.	-	0	ID=MALK_02676.t1.c1;Parent=MALK_02676.t1
contig010	maker	mRNA	48176	50638	.	-	.	ID=MALK_02676.t1;Parent=MALK_02676
contig010	maker	exon	48176	50638	.	-	.	ID=MALK_02676.t1.e1;Parent=MALK_02676.t1
contig010	AUGUSTUS	gene	51716	52555	0.97	+	.	ID=MALK_02677;prediction_source=augustus:contig010.g946.t1

contig010	AUGUSTUS	CDS	51716	52555	0.97	+	0	ID=MALK_02677.t1.c1;Parent=MALK_02677.t1
contig010	AUGUSTUS	mRNA	51716	52555	0.97	+	.	ID=MALK_02677.t1;Parent=MALK_02677
contig010	AUGUSTUS	exon	51716	52555	0.97	+	.	ID=MALK_02677.t1.e1;Parent=MALK_02677.t1
contig010	maker	gene	52620	54104	.	-	.	ID=MALK_02678;prediction_source=maker_MRET:augustus_masked-contig010-processed-gene-0.40-mRNA-1
contig010	maker	CDS	52620	54104	.	-	0	ID=MALK_02678.t1.c1;Parent=MALK_02678.t1
contig010	maker	mRNA	52620	54104	.	-	.	ID=MALK_02678.t1;Parent=MALK_02678
contig010	maker	exon	52620	54104	.	-	.	ID=MALK_02678.t1.e1;Parent=MALK_02678.t1
contig010	AUGUSTUS	gene	54253	54807	0.19	+	.	ID=MALK_02679;prediction_source=braker_MRET:g1239.t1
contig010	AUGUSTUS	CDS	54253	54807	0.19	+	0	ID=MALK_02679.t1.c1;Parent=MALK_02679.t1
contig010	AUGUSTUS	mRNA	54253	54807	0.19	+	.	ID=MALK_02679.t1;Parent=MALK_02679
contig010	AUGUSTUS	exon	54253	54807	.	+	.	ID=MALK_02679.t1.e1;Parent=MALK_02679.t1
contig010	AUGUSTUS	gene	55310	56299	0.6	+	.	ID=MALK_02680;prediction_source=augustus:contig010.g949.t1
contig010	AUGUSTUS	CDS	55310	56299	0.6	+	0	ID=MALK_02680.t1.c1;Parent=MALK_02680.t1
contig010	AUGUSTUS	mRNA	55310	56299	0.6	+	.	ID=MALK_02680.t1;Parent=MALK_02680
contig010	AUGUSTUS	exon	55310	56299	0.6	+	.	ID=MALK_02680.t1.e1;Parent=MALK_02680.t1
contig010	AUGUSTUS	gene	56899	59067	0.91	-	.	ID=MALK_02681;prediction_source=augustus:contig010.g950.t1
contig010	AUGUSTUS	CDS	56899	59067	0.91	-	0	ID=MALK_02681.t1.c1;Parent=MALK_02681.t1
contig010	AUGUSTUS	mRNA	56899	59067	0.91	-	.	ID=MALK_02681.t1;Parent=MALK_02681
contig010	AUGUSTUS	exon	56899	59067	0.91	-	.	ID=MALK_02681.t1.e1;Parent=MALK_02681.t1
contig010	AUGUSTUS	gene	59317	60135	0.59	+	.	ID=MALK_02682;prediction_source=augustus:contig010.g951.t1
contig010	AUGUSTUS	CDS	59317	60135	0.59	+	0	ID=MALK_02682.t1.c1;Parent=MALK_02682.t1
contig010	AUGUSTUS	mRNA	59317	60135	0.59	+	.	ID=MALK_02682.t1;Parent=MALK_02682
contig010	AUGUSTUS	exon	59317	60135	0.59	+	.	ID=MALK_02682.t1.e1;Parent=MALK_02682.t1
contig010	AUGUSTUS	gene	60779	62581	0.39	-	.	ID=MALK_02683;prediction_source=augustus:contig010.g952.t1
contig010	AUGUSTUS	CDS	60779	62581	0.39	-	0	ID=MALK_02683.t1.c1;Parent=MALK_02683.t1
contig010	AUGUSTUS	mRNA	60779	62581	0.39	-	.	ID=MALK_02683.t1;Parent=MALK_02683
contig010	AUGUSTUS	exon	60779	62581	0.39	-	.	ID=MALK_02683.t1.e1;Parent=MALK_02683.t1
contig010	AUGUSTUS	gene	62817	64583	0.94	-	.	ID=MALK_02684;prediction_source=augustus:contig010.g953.t1
contig010	AUGUSTUS	CDS	62817	64583	0.94	-	0	ID=MALK_02684.t1.c1;Parent=MALK_02684.t1
contig010	AUGUSTUS	mRNA	62817	64583	0.94	-	.	ID=MALK_02684.t1;Parent=MALK_02684
contig010	AUGUSTUS	exon	62817	64583	0.94	-	.	ID=MALK_02684.t1.e1;Parent=MALK_02684.t1
contig010	AUGUSTUS	gene	64943	65629	0.94	+	.	ID=MALK_02685;prediction_source=augustus:contig010.g954.t1
contig010	AUGUSTUS	CDS	64943	65629	0.94	+	0	ID=MALK_02685.t1.c1;Parent=MALK_02685.t1
contig010	AUGUSTUS	mRNA	64943	65629	0.94	+	.	ID=MALK_02685.t1;Parent=MALK_02685
contig010	AUGUSTUS	exon	64943	65629	0.94	+	.	ID=MALK_02685.t1.e1;Parent=MALK_02685.t1
contig010	AUGUSTUS	gene	65632	67029	0.98	-	.	ID=MALK_02686;prediction_source=augustus:contig010.g955.t1
contig010	AUGUSTUS	CDS	65632	67029	0.98	-	0	ID=MALK_02686.t1.c1;Parent=MALK_02686.t1
contig010	AUGUSTUS	mRNA	65632	67029	0.98	-	.	ID=MALK_02686.t1;Parent=MALK_02686
contig010	AUGUSTUS	exon	65632	67029	0.98	-	.	ID=MALK_02686.t1.e1;Parent=MALK_02686.t1
contig010	AUGUSTUS	gene	67135	68768	0.75	+	.	ID=MALK_02687;prediction_source=augustus:contig010.g956.t1
contig010	AUGUSTUS	CDS	67135	67566	0.8	+	0	ID=MALK_02687.t1.c1;Parent=MALK_02687.t1
contig010	AUGUSTUS	CDS	67626	68768	0.8	+	0	ID=MALK_02687.t1.c2;Parent=MALK_02687.t1
contig010	AUGUSTUS	mRNA	67135	68768	0.75	+	.	ID=MALK_02687.t1;Parent=MALK_02687

contig010	AUGUSTUS	exon	67135	67566	0.8	+	.	ID=MALK_02687.t1.e1;Parent=MALK_02687.t1
contig010	AUGUSTUS	exon	67626	68768	0.8	+	.	ID=MALK_02687.t1.e2;Parent=MALK_02687.t1
contig010	AUGUSTUS	gene	68765	69955	0.73	-	.	ID=MALK_02688;prediction_source=augustus:contig010.g957.t1
contig010	AUGUSTUS	CDS	68765	69955	0.73	-	0	ID=MALK_02688.t1.c1;Parent=MALK_02688.t1
contig010	AUGUSTUS	mRNA	68765	69955	0.73	-	.	ID=MALK_02688.t1;Parent=MALK_02688
contig010	AUGUSTUS	exon	68765	69955	0.73	-	.	ID=MALK_02688.t1.e1;Parent=MALK_02688.t1
contig010	AUGUSTUS	gene	70153	71844	0.94	+	.	ID=MALK_02689;prediction_source=augustus:contig010.g958.t1
contig010	AUGUSTUS	CDS	70153	71844	0.94	+	0	ID=MALK_02689.t1.c1;Parent=MALK_02689.t1
contig010	AUGUSTUS	mRNA	70153	71844	0.94	+	.	ID=MALK_02689.t1;Parent=MALK_02689
contig010	AUGUSTUS	exon	70153	71844	0.94	+	.	ID=MALK_02689.t1.e1;Parent=MALK_02689.t1
contig010	AUGUSTUS	gene	72113	73180	0.99	-	.	ID=MALK_02690;prediction_source=augustus:contig010.g960.t1
contig010	AUGUSTUS	CDS	72113	73180	0.99	-	0	ID=MALK_02690.t1.c1;Parent=MALK_02690.t1
contig010	AUGUSTUS	mRNA	72113	73180	0.99	-	.	ID=MALK_02690.t1;Parent=MALK_02690
contig010	AUGUSTUS	exon	72113	73180	0.99	-	.	ID=MALK_02690.t1.e1;Parent=MALK_02690.t1
contig010	AUGUSTUS	gene	73411	75372	0.49	-	.	ID=MALK_02691;prediction_source=augustus:contig010.g961.t1
contig010	AUGUSTUS	CDS	73411	75372	0.49	-	0	ID=MALK_02691.t1.c1;Parent=MALK_02691.t1
contig010	AUGUSTUS	mRNA	73411	75372	0.49	-	.	ID=MALK_02691.t1;Parent=MALK_02691
contig010	AUGUSTUS	exon	73411	75372	0.49	-	.	ID=MALK_02691.t1.e1;Parent=MALK_02691.t1
contig010	AUGUSTUS	gene	75491	79498	0.99	+	.	ID=MALK_02692;prediction_source=augustus:contig010.g962.t1
contig010	AUGUSTUS	CDS	75491	79498	0.99	+	0	ID=MALK_02692.t1.c1;Parent=MALK_02692.t1
contig010	AUGUSTUS	mRNA	75491	79498	0.99	+	.	ID=MALK_02692.t1;Parent=MALK_02692
contig010	AUGUSTUS	exon	75491	79498	0.99	+	.	ID=MALK_02692.t1.e1;Parent=MALK_02692.t1
contig010	AUGUSTUS	gene	79504	80007	0.54	-	.	ID=MALK_02693;prediction_source=braker_MRET:g1252.t1
contig010	AUGUSTUS	CDS	79504	80007	0.54	-	0	ID=MALK_02693.t1.c1;Parent=MALK_02693.t1
contig010	AUGUSTUS	mRNA	79504	80007	0.54	-	.	ID=MALK_02693.t1;Parent=MALK_02693
contig010	AUGUSTUS	exon	79504	80007	.	-	.	ID=MALK_02693.t1.e1;Parent=MALK_02693.t1
contig010	maker	gene	80127	80936	.	+	.	ID=MALK_02694;prediction_source=maker_MRET:augustus_masked-contig010-processed-gene-0.19-mRNA-1
contig010	maker	CDS	80127	80936	.	+	0	ID=MALK_02694.t1.c1;Parent=MALK_02694.t1
contig010	maker	mRNA	80127	80936	.	+	.	ID=MALK_02694.t1;Parent=MALK_02694
contig010	maker	exon	80127	80936	.	+	.	ID=MALK_02694.t1.e1;Parent=MALK_02694.t1
contig010	AUGUSTUS	gene	80998	81876	0.55	-	.	ID=MALK_02695;prediction_source=braker_MRET:g1254.t1
contig010	AUGUSTUS	CDS	81797	81876	0.55	-	0	ID=MALK_02695.t1.c2;Parent=MALK_02695.t1
contig010	AUGUSTUS	CDS	80998	81574	0.55	-	0	ID=MALK_02695.t1.c1;Parent=MALK_02695.t1
contig010	AUGUSTUS	mRNA	80998	81876	0.55	-	.	ID=MALK_02695.t1;Parent=MALK_02695
contig010	AUGUSTUS	exon	81797	81876	.	-	.	ID=MALK_02695.t1.e2;Parent=MALK_02695.t1
contig010	AUGUSTUS	exon	80998	81574	.	-	.	ID=MALK_02695.t1.e1;Parent=MALK_02695.t1
contig010	AUGUSTUS	gene	82221	83510	0.19	+	.	ID=MALK_02696;prediction_source=braker_MRET:g1255.t1
contig010	AUGUSTUS	CDS	82221	82359	0.68	+	0	ID=MALK_02696.t1.c1;Parent=MALK_02696.t1
contig010	AUGUSTUS	CDS	82391	82563	0.68	+	0	ID=MALK_02696.t1.c2;Parent=MALK_02696.t1
contig010	AUGUSTUS	CDS	82602	83510	0.68	+	0	ID=MALK_02696.t1.c3;Parent=MALK_02696.t1
contig010	AUGUSTUS	mRNA	82221	83510	0.19	+	.	ID=MALK_02696.t1;Parent=MALK_02696
contig010	AUGUSTUS	exon	82221	82359	.	+	.	ID=MALK_02696.t1.e1;Parent=MALK_02696.t1
contig010	AUGUSTUS	exon	82391	82563	.	+	.	ID=MALK_02696.t1.e2;Parent=MALK_02696.t1

contig010	AUGUSTUS	exon	82602	83510	.	+	.	ID=MALK_02696.t1.e3;Parent=MALK_02696.t1
contig010	maker	gene	83583	83924	.	-	.	ID=MALK_02697;prediction_source=maker_MRET:augustus_masked-contig010-processed-gene-0.49-mRNA-1
contig010	maker	CDS	83583	83924	.	-	0	ID=MALK_02697.t1.c1;Parent=MALK_02697.t1
contig010	maker	mRNA	83583	83924	.	-	.	ID=MALK_02697.t1;Parent=MALK_02697
contig010	maker	exon	83583	83924	.	-	.	ID=MALK_02697.t1.e1;Parent=MALK_02697.t1
contig010	AUGUSTUS	gene	84150	84724	0.55	-	.	ID=MALK_02698;prediction_source=braker_MRET:g1257.t1
contig010	AUGUSTUS	CDS	84704	84724	0.72	-	0	ID=MALK_02698.t1.c7;Parent=MALK_02698.t1
contig010	AUGUSTUS	CDS	84608	84667	0.72	-	0	ID=MALK_02698.t1.c6;Parent=MALK_02698.t1
contig010	AUGUSTUS	CDS	84495	84566	0.72	-	0	ID=MALK_02698.t1.c5;Parent=MALK_02698.t1
contig010	AUGUSTUS	CDS	84363	84464	0.72	-	0	ID=MALK_02698.t1.c4;Parent=MALK_02698.t1
contig010	AUGUSTUS	CDS	84292	84327	0.72	-	0	ID=MALK_02698.t1.c3;Parent=MALK_02698.t1
contig010	AUGUSTUS	CDS	84206	84261	0.72	-	0	ID=MALK_02698.t1.c2;Parent=MALK_02698.t1
contig010	AUGUSTUS	CDS	84150	84165	0.72	-	0	ID=MALK_02698.t1.c1;Parent=MALK_02698.t1
contig010	AUGUSTUS	mRNA	84150	84724	0.55	-	.	ID=MALK_02698.t1;Parent=MALK_02698
contig010	AUGUSTUS	exon	84704	84724	.	-	.	ID=MALK_02698.t1.e7;Parent=MALK_02698.t1
contig010	AUGUSTUS	exon	84608	84667	.	-	.	ID=MALK_02698.t1.e6;Parent=MALK_02698.t1
contig010	AUGUSTUS	exon	84495	84566	.	-	.	ID=MALK_02698.t1.e5;Parent=MALK_02698.t1
contig010	AUGUSTUS	exon	84363	84464	.	-	.	ID=MALK_02698.t1.e4;Parent=MALK_02698.t1
contig010	AUGUSTUS	exon	84292	84327	.	-	.	ID=MALK_02698.t1.e3;Parent=MALK_02698.t1
contig010	AUGUSTUS	exon	84206	84261	.	-	.	ID=MALK_02698.t1.e2;Parent=MALK_02698.t1
contig010	AUGUSTUS	exon	84150	84165	.	-	.	ID=MALK_02698.t1.e1;Parent=MALK_02698.t1
contig010	AUGUSTUS	gene	85411	86988	0.99	+	.	ID=MALK_02699;prediction_source=augustus:contig010.g968.t1
contig010	AUGUSTUS	CDS	85411	86988	0.99	+	0	ID=MALK_02699.t1.c1;Parent=MALK_02699.t1
contig010	AUGUSTUS	mRNA	85411	86988	0.99	+	.	ID=MALK_02699.t1;Parent=MALK_02699
contig010	AUGUSTUS	exon	85411	86988	0.99	+	.	ID=MALK_02699.t1.e1;Parent=MALK_02699.t1
contig010	maker	gene	87124	87858	.	-	.	ID=MALK_02700;prediction_source=maker_MRET:augustus_masked-contig010-processed-gene-0.50-mRNA-1
contig010	maker	CDS	87124	87858	.	-	0	ID=MALK_02700.t1.c1;Parent=MALK_02700.t1
contig010	maker	mRNA	87124	87858	.	-	.	ID=MALK_02700.t1;Parent=MALK_02700
contig010	maker	exon	87124	87858	.	-	.	ID=MALK_02700.t1.e1;Parent=MALK_02700.t1
contig010	maker	gene	87954	92684	.	-	.	ID=MALK_02701;prediction_source=maker_MRET:augustus_masked-contig010-processed-gene-0.51-mRNA-1
contig010	maker	CDS	87954	92684	.	-	0	ID=MALK_02701.t1.c1;Parent=MALK_02701.t1
contig010	maker	mRNA	87954	92684	.	-	.	ID=MALK_02701.t1;Parent=MALK_02701
contig010	maker	exon	87954	92684	.	-	.	ID=MALK_02701.t1.e1;Parent=MALK_02701.t1
contig010	maker	gene	92936	93610	.	+	.	ID=MALK_02702;prediction_source=maker_MRET:augustus_masked-contig010-processed-gene-0.22-mRNA-1
contig010	maker	CDS	92936	93610	.	+	0	ID=MALK_02702.t1.c1;Parent=MALK_02702.t1
contig010	maker	mRNA	92936	93610	.	+	.	ID=MALK_02702.t1;Parent=MALK_02702
contig010	maker	exon	92936	93610	.	+	.	ID=MALK_02702.t1.e1;Parent=MALK_02702.t1
contig010	maker	gene	93627	97043	.	-	.	ID=MALK_02703;prediction_source=maker_MRET:augustus_masked-contig010-processed-gene-1.44-mRNA-1
contig010	maker	CDS	93627	97043	.	-	0	ID=MALK_02703.t1.c1;Parent=MALK_02703.t1
contig010	maker	mRNA	93627	97043	.	-	.	ID=MALK_02703.t1;Parent=MALK_02703
contig010	maker	exon	93627	97043	.	-	.	ID=MALK_02703.t1.e1;Parent=MALK_02703.t1
contig010	maker	gene	97302	99047	.	-	.	ID=MALK_02704;prediction_source=maker_MRET:augustus_masked-contig010-processed-gene-1.45-mRNA-1
contig010	maker	CDS	97302	99047	.	-	0	ID=MALK_02704.t1.c1;Parent=MALK_02704.t1

contig010	maker	mRNA	97302	99047	.	-	.	ID=MALK_02704.t1;Parent=MALK_02704
contig010	maker	exon	97302	99047	.	-	.	ID=MALK_02704.t1.e1;Parent=MALK_02704.t1
contig010	AUGUSTUS	gene	99611	101275	0.98	-	.	ID=MALK_02705;prediction_source=augustus:contig010.g974.t1
contig010	AUGUSTUS	CDS	99611	101275	0.98	-	0	ID=MALK_02705.t1.c1;Parent=MALK_02705.t1
contig010	AUGUSTUS	mRNA	99611	101275	0.98	-	.	ID=MALK_02705.t1;Parent=MALK_02705
contig010	AUGUSTUS	exon	99611	101275	0.98	-	.	ID=MALK_02705.t1.e1;Parent=MALK_02705.t1
contig010	AUGUSTUS	gene	101506	103251	0.98	-	.	ID=MALK_02706;prediction_source=augustus:contig010.g976.t1
contig010	AUGUSTUS	CDS	101506	103251	0.98	-	0	ID=MALK_02706.t1.c1;Parent=MALK_02706.t1
contig010	AUGUSTUS	mRNA	101506	103251	0.98	-	.	ID=MALK_02706.t1;Parent=MALK_02706
contig010	AUGUSTUS	exon	101506	103251	0.98	-	.	ID=MALK_02706.t1.e1;Parent=MALK_02706.t1
contig010	AUGUSTUS	gene	103704	105452	0.82	-	.	ID=MALK_02707;prediction_source=augustus:contig010.g977.t1
contig010	AUGUSTUS	CDS	103704	105452	0.82	-	0	ID=MALK_02707.t1.c1;Parent=MALK_02707.t1
contig010	AUGUSTUS	mRNA	103704	105452	0.82	-	.	ID=MALK_02707.t1;Parent=MALK_02707
contig010	AUGUSTUS	exon	103704	105452	0.82	-	.	ID=MALK_02707.t1.e1;Parent=MALK_02707.t1
contig010	AUGUSTUS	gene	105769	107184	0.75	-	.	ID=MALK_02708;prediction_source=augustus:contig010.g978.t1
contig010	AUGUSTUS	CDS	105769	107184	0.75	-	0	ID=MALK_02708.t1.c1;Parent=MALK_02708.t1
contig010	AUGUSTUS	mRNA	105769	107184	0.75	-	.	ID=MALK_02708.t1;Parent=MALK_02708
contig010	AUGUSTUS	exon	105769	107184	0.75	-	.	ID=MALK_02708.t1.e1;Parent=MALK_02708.t1
contig010	AUGUSTUS	gene	107307	109232	0.39	+	.	ID=MALK_02709;prediction_source=braker_MRET:g1268.t1
contig010	AUGUSTUS	CDS	107307	109232	0.39	+	0	ID=MALK_02709.t1.c1;Parent=MALK_02709.t1
contig010	AUGUSTUS	mRNA	107307	109232	0.39	+	.	ID=MALK_02709.t1;Parent=MALK_02709
contig010	AUGUSTUS	exon	107307	109232	.	+	.	ID=MALK_02709.t1.e1;Parent=MALK_02709.t1
contig010	AUGUSTUS	gene	109302	110792	0.93	+	.	ID=MALK_02710;prediction_source=braker_MRET:g1269.t1
contig010	AUGUSTUS	CDS	109302	110792	0.93	+	0	ID=MALK_02710.t1.c1;Parent=MALK_02710.t1
contig010	AUGUSTUS	mRNA	109302	110792	0.93	+	.	ID=MALK_02710.t1;Parent=MALK_02710
contig010	AUGUSTUS	exon	109302	110792	.	+	.	ID=MALK_02710.t1.e1;Parent=MALK_02710.t1
contig010	AUGUSTUS	gene	111002	111697	0.63	-	.	ID=MALK_02711;prediction_source=braker_MRET:g1270.t1
contig010	AUGUSTUS	CDS	111617	111697	0.98	-	0	ID=MALK_02711.t1.c4;Parent=MALK_02711.t1
contig010	AUGUSTUS	CDS	111535	111585	0.98	-	0	ID=MALK_02711.t1.c3;Parent=MALK_02711.t1
contig010	AUGUSTUS	CDS	111131	111503	0.98	-	0	ID=MALK_02711.t1.c2;Parent=MALK_02711.t1
contig010	AUGUSTUS	CDS	111002	111102	0.98	-	0	ID=MALK_02711.t1.c1;Parent=MALK_02711.t1
contig010	AUGUSTUS	mRNA	111002	111697	0.63	-	.	ID=MALK_02711.t1;Parent=MALK_02711
contig010	AUGUSTUS	exon	111617	111697	.	-	.	ID=MALK_02711.t1.e4;Parent=MALK_02711.t1
contig010	AUGUSTUS	exon	111535	111585	.	-	.	ID=MALK_02711.t1.e3;Parent=MALK_02711.t1
contig010	AUGUSTUS	exon	111131	111503	.	-	.	ID=MALK_02711.t1.e2;Parent=MALK_02711.t1
contig010	AUGUSTUS	exon	111002	111102	.	-	.	ID=MALK_02711.t1.e1;Parent=MALK_02711.t1
contig010	maker	gene	111784	114621	.	-	.	ID=MALK_02712;prediction_source=maker_MRET:augustus_masked-contig010-processed-gene-1.67-mRNA-1
contig010	maker	CDS	111784	114621	.	-	0	ID=MALK_02712.t1.c1;Parent=MALK_02712.t1
contig010	maker	mRNA	111784	114621	.	-	.	ID=MALK_02712.t1;Parent=MALK_02712
contig010	maker	exon	111784	114621	.	-	.	ID=MALK_02712.t1.e1;Parent=MALK_02712.t1
contig010	AUGUSTUS	gene	114796	116292	1	-	.	ID=MALK_02713;prediction_source=augustus:contig010.g983.t1
contig010	AUGUSTUS	CDS	114796	116292	1	-	0	ID=MALK_02713.t1.c1;Parent=MALK_02713.t1
contig010	AUGUSTUS	mRNA	114796	116292	1	-	.	ID=MALK_02713.t1;Parent=MALK_02713

contig010	AUGUSTUS	exon	114796	116292	1	-	.	ID=MALK_02713.t1.e1;Parent=MALK_02713.t1
contig010	AUGUSTUS	gene	116413	117615	0.68	+	.	ID=MALK_02714;prediction_source=braker_MRET:g1272.t1
contig010	AUGUSTUS	CDS	116413	116686	0.95	+	0	ID=MALK_02714.t1.c1;Parent=MALK_02714.t1
contig010	AUGUSTUS	CDS	116719	117175	0.95	+	0	ID=MALK_02714.t1.c2;Parent=MALK_02714.t1
contig010	AUGUSTUS	CDS	117255	117615	0.95	+	0	ID=MALK_02714.t1.c3;Parent=MALK_02714.t1
contig010	AUGUSTUS	mRNA	116413	117615	0.68	+	.	ID=MALK_02714.t1;Parent=MALK_02714
contig010	AUGUSTUS	exon	116413	116686	.	+	.	ID=MALK_02714.t1.e1;Parent=MALK_02714.t1
contig010	AUGUSTUS	exon	116719	117175	.	+	.	ID=MALK_02714.t1.e2;Parent=MALK_02714.t1
contig010	AUGUSTUS	exon	117255	117615	.	+	.	ID=MALK_02714.t1.e3;Parent=MALK_02714.t1
contig010	AUGUSTUS	gene	117632	119059	0.6	-	.	ID=MALK_02715;prediction_source=augustus:contig010.g985.t1
contig010	AUGUSTUS	CDS	117632	119059	0.6	-	0	ID=MALK_02715.t1.c1;Parent=MALK_02715.t1
contig010	AUGUSTUS	mRNA	117632	119059	0.6	-	.	ID=MALK_02715.t1;Parent=MALK_02715
contig010	AUGUSTUS	exon	117632	119059	0.6	-	.	ID=MALK_02715.t1.e1;Parent=MALK_02715.t1
contig010	AUGUSTUS	gene	119170	119774	0.33	+	.	ID=MALK_02716;prediction_source=braker_MRET:g1274.t1
contig010	AUGUSTUS	CDS	119170	119722	0.65	+	0	ID=MALK_02716.t1.c1;Parent=MALK_02716.t1
contig010	AUGUSTUS	CDS	119752	119774	0.65	+	0	ID=MALK_02716.t1.c2;Parent=MALK_02716.t1
contig010	AUGUSTUS	mRNA	119170	119774	0.33	+	.	ID=MALK_02716.t1;Parent=MALK_02716
contig010	AUGUSTUS	exon	119170	119722	.	+	.	ID=MALK_02716.t1.e1;Parent=MALK_02716.t1
contig010	AUGUSTUS	exon	119752	119774	.	+	.	ID=MALK_02716.t1.e2;Parent=MALK_02716.t1
contig010	AUGUSTUS	gene	119937	122015	1	-	.	ID=MALK_02717;prediction_source=augustus:contig010.g987.t1
contig010	AUGUSTUS	CDS	119937	122015	1	-	0	ID=MALK_02717.t1.c1;Parent=MALK_02717.t1
contig010	AUGUSTUS	mRNA	119937	122015	1	-	.	ID=MALK_02717.t1;Parent=MALK_02717
contig010	AUGUSTUS	exon	119937	122015	1	-	.	ID=MALK_02717.t1.e1;Parent=MALK_02717.t1
contig010	maker	gene	122914	123606	.	+	.	ID=MALK_02718;prediction_source=maker_MRET:augustus_masked-contig010-processed-gene-1.50-mRNA-1
contig010	maker	CDS	122914	123606	.	+	0	ID=MALK_02718.t1.c1;Parent=MALK_02718.t1
contig010	maker	mRNA	122914	123606	.	+	.	ID=MALK_02718.t1;Parent=MALK_02718
contig010	maker	exon	122914	123606	.	+	.	ID=MALK_02718.t1.e1;Parent=MALK_02718.t1
contig010	AUGUSTUS	gene	123611	124834	0.96	-	.	ID=MALK_02719;prediction_source=braker_MRET:g1277.t1
contig010	AUGUSTUS	CDS	123611	124834	0.96	-	0	ID=MALK_02719.t1.c1;Parent=MALK_02719.t1
contig010	AUGUSTUS	mRNA	123611	124834	0.96	-	.	ID=MALK_02719.t1;Parent=MALK_02719
contig010	AUGUSTUS	exon	123611	124834	.	-	.	ID=MALK_02719.t1.e1;Parent=MALK_02719.t1
contig010	AUGUSTUS	gene	124934	125422	0.51	+	.	ID=MALK_02720;prediction_source=augustus:contig010.g990.t1
contig010	AUGUSTUS	CDS	124934	125422	0.51	+	0	ID=MALK_02720.t1.c1;Parent=MALK_02720.t1
contig010	AUGUSTUS	mRNA	124934	125422	0.51	+	.	ID=MALK_02720.t1;Parent=MALK_02720
contig010	AUGUSTUS	exon	124934	125422	0.51	+	.	ID=MALK_02720.t1.e1;Parent=MALK_02720.t1
contig010	AUGUSTUS	gene	125534	126699	0.42	-	.	ID=MALK_02721;prediction_source=braker_MRET:g1279.t1
contig010	AUGUSTUS	CDS	125578	126699	0.42	-	0	ID=MALK_02721.t1.c2;Parent=MALK_02721.t1
contig010	AUGUSTUS	CDS	125534	125536	0.42	-	0	ID=MALK_02721.t1.c1;Parent=MALK_02721.t1
contig010	AUGUSTUS	mRNA	125534	126699	0.42	-	.	ID=MALK_02721.t1;Parent=MALK_02721
contig010	AUGUSTUS	exon	125578	126699	.	-	.	ID=MALK_02721.t1.e2;Parent=MALK_02721.t1
contig010	AUGUSTUS	exon	125534	125536	.	-	.	ID=MALK_02721.t1.e1;Parent=MALK_02721.t1
contig010	maker	gene	126769	127759	.	+	.	ID=MALK_02722;prediction_source=maker_MRET:augustus_masked-contig010-processed-gene-1.51-mRNA-1
contig010	maker	CDS	126769	126871	.	+	0	ID=MALK_02722.t1.c1;Parent=MALK_02722.t1

contig010	maker	CDS	127014	127759	.	+	0	ID=MALK_02722.t1.c2;Parent=MALK_02722.t1
contig010	maker	mRNA	126769	127759	.	+	.	ID=MALK_02722.t1;Parent=MALK_02722
contig010	maker	exon	126769	126871	.	+	.	ID=MALK_02722.t1.e1;Parent=MALK_02722.t1
contig010	maker	exon	127014	127759	.	+	.	ID=MALK_02722.t1.e2;Parent=MALK_02722.t1
contig010	maker	gene	127761	129284	.	-	.	ID=MALK_02723;prediction_source=maker_MRET:augustus_masked-contig010-processed-gene-1.73-mRNA-1
contig010	maker	CDS	127761	129284	.	-	0	ID=MALK_02723.t1.c1;Parent=MALK_02723.t1
contig010	maker	mRNA	127761	129284	.	-	.	ID=MALK_02723.t1;Parent=MALK_02723
contig010	maker	exon	127761	129284	.	-	.	ID=MALK_02723.t1.e1;Parent=MALK_02723.t1
contig010	AUGUSTUS	gene	129355	131310	0.97	-	.	ID=MALK_02724;prediction_source=augustus:contig010.g994.t1
contig010	AUGUSTUS	CDS	129355	131310	0.97	-	0	ID=MALK_02724.t1.c1;Parent=MALK_02724.t1
contig010	AUGUSTUS	mRNA	129355	131310	0.97	-	.	ID=MALK_02724.t1;Parent=MALK_02724
contig010	AUGUSTUS	exon	129355	131310	0.97	-	.	ID=MALK_02724.t1.e1;Parent=MALK_02724.t1
contig010	AUGUSTUS	gene	131520	134078	0.96	-	.	ID=MALK_02725;prediction_source=braker_MRET:g1283.t1
contig010	AUGUSTUS	CDS	131520	134078	0.96	-	0	ID=MALK_02725.t1.c1;Parent=MALK_02725.t1
contig010	AUGUSTUS	mRNA	131520	134078	0.96	-	.	ID=MALK_02725.t1;Parent=MALK_02725
contig010	AUGUSTUS	exon	131520	134078	.	-	.	ID=MALK_02725.t1.e1;Parent=MALK_02725.t1
contig010	maker	gene	134465	135424	.	-	.	ID=MALK_02726;prediction_source=maker_MRET:augustus_masked-contig010-processed-gene-1.76-mRNA-1
contig010	maker	CDS	134465	135424	.	-	0	ID=MALK_02726.t1.c1;Parent=MALK_02726.t1
contig010	maker	mRNA	134465	135424	.	-	.	ID=MALK_02726.t1;Parent=MALK_02726
contig010	maker	exon	134465	135424	.	-	.	ID=MALK_02726.t1.e1;Parent=MALK_02726.t1
contig010	maker	gene	136333	137448	.	+	.	ID=MALK_02727;prediction_source=maker_MRET:augustus_masked-contig010-processed-gene-1.52-mRNA-1
contig010	maker	CDS	136333	137448	.	+	0	ID=MALK_02727.t1.c1;Parent=MALK_02727.t1
contig010	maker	mRNA	136333	137448	.	+	.	ID=MALK_02727.t1;Parent=MALK_02727
contig010	maker	exon	136333	137448	.	+	.	ID=MALK_02727.t1.e1;Parent=MALK_02727.t1
contig010	AUGUSTUS	gene	137877	139391	0.88	+	.	ID=MALK_02728;prediction_source=augustus:contig010.g999.t1
contig010	AUGUSTUS	CDS	137877	139391	0.88	+	0	ID=MALK_02728.t1.c1;Parent=MALK_02728.t1
contig010	AUGUSTUS	mRNA	137877	139391	0.88	+	.	ID=MALK_02728.t1;Parent=MALK_02728
contig010	AUGUSTUS	exon	137877	139391	0.88	+	.	ID=MALK_02728.t1.e1;Parent=MALK_02728.t1
contig010	AUGUSTUS	gene	139520	140431	0.09	+	.	ID=MALK_02729;prediction_source=braker_MRET:g1287.t1
contig010	AUGUSTUS	CDS	139520	139675	0.5	+	0	ID=MALK_02729.t1.c1;Parent=MALK_02729.t1
contig010	AUGUSTUS	CDS	139718	139991	0.5	+	0	ID=MALK_02729.t1.c2;Parent=MALK_02729.t1
contig010	AUGUSTUS	CDS	140098	140267	0.5	+	0	ID=MALK_02729.t1.c3;Parent=MALK_02729.t1
contig010	AUGUSTUS	CDS	140429	140431	0.5	+	0	ID=MALK_02729.t1.c4;Parent=MALK_02729.t1
contig010	AUGUSTUS	mRNA	139520	140431	0.09	+	.	ID=MALK_02729.t1;Parent=MALK_02729
contig010	AUGUSTUS	exon	139520	139675	.	+	.	ID=MALK_02729.t1.e1;Parent=MALK_02729.t1
contig010	AUGUSTUS	exon	139718	139991	.	+	.	ID=MALK_02729.t1.e2;Parent=MALK_02729.t1
contig010	AUGUSTUS	exon	140098	140267	.	+	.	ID=MALK_02729.t1.e3;Parent=MALK_02729.t1
contig010	AUGUSTUS	exon	140429	140431	.	+	.	ID=MALK_02729.t1.e4;Parent=MALK_02729.t1
contig010	AUGUSTUS	gene	140484	141992	0.92	-	.	ID=MALK_02730;prediction_source=braker_MRET:g1288.t1
contig010	AUGUSTUS	CDS	140484	141992	0.92	-	0	ID=MALK_02730.t1.c1;Parent=MALK_02730.t1
contig010	AUGUSTUS	mRNA	140484	141992	0.92	-	.	ID=MALK_02730.t1;Parent=MALK_02730
contig010	AUGUSTUS	exon	140484	141992	.	-	.	ID=MALK_02730.t1.e1;Parent=MALK_02730.t1
contig010	maker	gene	142029	143378	.	-	.	ID=MALK_02731;prediction_source=maker_MRET:augustus_masked-contig010-processed-gene-1.78-mRNA-1

contig010	maker	CDS	142029	143378	.	-	0	ID=MALK_02731.t1.c1;Parent=MALK_02731.t1
contig010	maker	mRNA	142029	143378	.	-	.	ID=MALK_02731.t1;Parent=MALK_02731
contig010	maker	exon	142029	143378	.	-	.	ID=MALK_02731.t1.e1;Parent=MALK_02731.t1
contig010	AUGUSTUS	gene	143402	144244	0.99	+	.	ID=MALK_02732;prediction_source=augustus:contig010.g1003.t1
contig010	AUGUSTUS	CDS	143402	144244	0.99	+	0	ID=MALK_02732.t1.c1;Parent=MALK_02732.t1
contig010	AUGUSTUS	mRNA	143402	144244	0.99	+	.	ID=MALK_02732.t1;Parent=MALK_02732
contig010	AUGUSTUS	exon	143402	144244	0.99	+	.	ID=MALK_02732.t1.e1;Parent=MALK_02732.t1
contig010	AUGUSTUS	gene	144237	145208	0.44	-	.	ID=MALK_02733;prediction_source=augustus:contig010.g1004.t1
contig010	AUGUSTUS	CDS	144237	145208	0.44	-	0	ID=MALK_02733.t1.c1;Parent=MALK_02733.t1
contig010	AUGUSTUS	mRNA	144237	145208	0.44	-	.	ID=MALK_02733.t1;Parent=MALK_02733
contig010	AUGUSTUS	exon	144237	145208	0.44	-	.	ID=MALK_02733.t1.e1;Parent=MALK_02733.t1
contig010	AUGUSTUS	gene	145605	148664	0.9	-	.	ID=MALK_02734;prediction_source=augustus:contig010.g1005.t1
contig010	AUGUSTUS	CDS	145605	148664	0.9	-	0	ID=MALK_02734.t1.c1;Parent=MALK_02734.t1
contig010	AUGUSTUS	mRNA	145605	148664	0.9	-	.	ID=MALK_02734.t1;Parent=MALK_02734
contig010	AUGUSTUS	exon	145605	148664	0.9	-	.	ID=MALK_02734.t1.e1;Parent=MALK_02734.t1
contig010	maker	gene	148774	149666	.	-	.	ID=MALK_02735;prediction_source=maker_MRET:augustus_masked-contig010-processed-gene-1.81-mRNA-1
contig010	maker	CDS	149529	149666	.	-	0	ID=MALK_02735.t1.c1;Parent=MALK_02735.t1
contig010	maker	CDS	148774	149466	.	-	0	ID=MALK_02735.t1.c2;Parent=MALK_02735.t1
contig010	maker	mRNA	148774	149666	.	-	.	ID=MALK_02735.t1;Parent=MALK_02735
contig010	maker	exon	149529	149666	.	-	.	ID=MALK_02735.t1.e1;Parent=MALK_02735.t1
contig010	maker	exon	148774	149466	.	-	.	ID=MALK_02735.t1.e2;Parent=MALK_02735.t1
contig010	AUGUSTUS	gene	150197	151713	0.3	+	.	ID=MALK_02736;prediction_source=augustus:contig010.g1009.t1
contig010	AUGUSTUS	CDS	150197	151278	0.54	+	0	ID=MALK_02736.t1.c1;Parent=MALK_02736.t1
contig010	AUGUSTUS	CDS	151359	151713	0.54	+	0	ID=MALK_02736.t1.c2;Parent=MALK_02736.t1
contig010	AUGUSTUS	mRNA	150197	151713	0.3	+	.	ID=MALK_02736.t1;Parent=MALK_02736
contig010	AUGUSTUS	exon	150197	151278	0.54	+	.	ID=MALK_02736.t1.e1;Parent=MALK_02736.t1
contig010	AUGUSTUS	exon	151359	151713	0.54	+	.	ID=MALK_02736.t1.e2;Parent=MALK_02736.t1
contig010	AUGUSTUS	gene	151718	153319	0.52	-	.	ID=MALK_02737;prediction_source=augustus:contig010.g1010.t1
contig010	AUGUSTUS	CDS	151718	153319	0.52	-	0	ID=MALK_02737.t1.c1;Parent=MALK_02737.t1
contig010	AUGUSTUS	mRNA	151718	153319	0.52	-	.	ID=MALK_02737.t1;Parent=MALK_02737
contig010	AUGUSTUS	exon	151718	153319	0.52	-	.	ID=MALK_02737.t1.e1;Parent=MALK_02737.t1
contig010	AUGUSTUS	gene	153434	158542	0.6	+	.	ID=MALK_02738;prediction_source=braker_MRET:g1296.t1
contig010	AUGUSTUS	CDS	153434	158542	0.6	+	0	ID=MALK_02738.t1.c1;Parent=MALK_02738.t1
contig010	AUGUSTUS	mRNA	153434	158542	0.6	+	.	ID=MALK_02738.t1;Parent=MALK_02738
contig010	AUGUSTUS	exon	153434	158542	.	+	.	ID=MALK_02738.t1.e1;Parent=MALK_02738.t1
contig010	AUGUSTUS	gene	159363	160364	0.68	+	.	ID=MALK_02739;prediction_source=augustus:contig010.g1012.t1
contig010	AUGUSTUS	CDS	159363	160364	0.68	+	0	ID=MALK_02739.t1.c1;Parent=MALK_02739.t1
contig010	AUGUSTUS	mRNA	159363	160364	0.68	+	.	ID=MALK_02739.t1;Parent=MALK_02739
contig010	AUGUSTUS	exon	159363	160364	0.68	+	.	ID=MALK_02739.t1.e1;Parent=MALK_02739.t1
contig010	AUGUSTUS	gene	160359	162591	0.45	-	.	ID=MALK_02740;prediction_source=braker_MRET:g1298.t1
contig010	AUGUSTUS	CDS	162274	162591	0.45	-	0	ID=MALK_02740.t1.c2;Parent=MALK_02740.t1
contig010	AUGUSTUS	CDS	160359	162146	0.45	-	0	ID=MALK_02740.t1.c1;Parent=MALK_02740.t1
contig010	AUGUSTUS	mRNA	160359	162591	0.45	-	.	ID=MALK_02740.t1;Parent=MALK_02740

contig010	AUGUSTUS	exon	162274	162591	.	-	.	ID=MALK_02740.t1.e2;Parent=MALK_02740.t1
contig010	AUGUSTUS	exon	160359	162146	.	-	.	ID=MALK_02740.t1.e1;Parent=MALK_02740.t1
contig010	AUGUSTUS	gene	163594	165519	0.83	+	.	ID=MALK_02741;prediction_source=augustus:contig010.g1013.t1
contig010	AUGUSTUS	CDS	163594	165519	0.83	+	0	ID=MALK_02741.t1.c1;Parent=MALK_02741.t1
contig010	AUGUSTUS	mRNA	163594	165519	0.83	+	.	ID=MALK_02741.t1;Parent=MALK_02741
contig010	AUGUSTUS	exon	163594	165519	0.83	+	.	ID=MALK_02741.t1.e1;Parent=MALK_02741.t1
contig010	AUGUSTUS	gene	165695	166201	0.65	-	.	ID=MALK_02742;prediction_source=braker_MRET:g1300.t1
contig010	AUGUSTUS	CDS	165877	166201	0.89	-	0	ID=MALK_02742.t1.c4;Parent=MALK_02742.t1
contig010	AUGUSTUS	CDS	165827	165847	0.89	-	0	ID=MALK_02742.t1.c3;Parent=MALK_02742.t1
contig010	AUGUSTUS	CDS	165774	165791	0.89	-	0	ID=MALK_02742.t1.c2;Parent=MALK_02742.t1
contig010	AUGUSTUS	CDS	165695	165744	0.89	-	0	ID=MALK_02742.t1.c1;Parent=MALK_02742.t1
contig010	AUGUSTUS	mRNA	165695	166201	0.65	-	.	ID=MALK_02742.t1;Parent=MALK_02742
contig010	AUGUSTUS	exon	165877	166201	.	-	.	ID=MALK_02742.t1.e4;Parent=MALK_02742.t1
contig010	AUGUSTUS	exon	165827	165847	.	-	.	ID=MALK_02742.t1.e3;Parent=MALK_02742.t1
contig010	AUGUSTUS	exon	165774	165791	.	-	.	ID=MALK_02742.t1.e2;Parent=MALK_02742.t1
contig010	AUGUSTUS	exon	165695	165744	.	-	.	ID=MALK_02742.t1.e1;Parent=MALK_02742.t1
contig010	AUGUSTUS	gene	166415	167813	0.85	-	.	ID=MALK_02743;prediction_source=braker_MRET:g1301.t1
contig010	AUGUSTUS	CDS	166652	167813	0.87	-	0	ID=MALK_02743.t1.c2;Parent=MALK_02743.t1
contig010	AUGUSTUS	CDS	166415	166623	0.87	-	0	ID=MALK_02743.t1.c1;Parent=MALK_02743.t1
contig010	AUGUSTUS	mRNA	166415	167813	0.85	-	.	ID=MALK_02743.t1;Parent=MALK_02743
contig010	AUGUSTUS	exon	166652	167813	.	-	.	ID=MALK_02743.t1.e2;Parent=MALK_02743.t1
contig010	AUGUSTUS	exon	166415	166623	.	-	.	ID=MALK_02743.t1.e1;Parent=MALK_02743.t1
contig010	AUGUSTUS	gene	167981	168637	0.87	-	.	ID=MALK_02744;prediction_source=braker_MRET:g1302.t1
contig010	AUGUSTUS	CDS	167981	168637	0.87	-	0	ID=MALK_02744.t1.c1;Parent=MALK_02744.t1
contig010	AUGUSTUS	mRNA	167981	168637	0.87	-	.	ID=MALK_02744.t1;Parent=MALK_02744
contig010	AUGUSTUS	exon	167981	168637	.	-	.	ID=MALK_02744.t1.e1;Parent=MALK_02744.t1
contig010	AUGUSTUS	gene	168804	170615	0.09	-	.	ID=MALK_02745;prediction_source=braker_MRET:g1303.t1
contig010	AUGUSTUS	CDS	170613	170615	0.57	-	0	ID=MALK_02745.t1.c7;Parent=MALK_02745.t1
contig010	AUGUSTUS	CDS	170433	170568	0.57	-	0	ID=MALK_02745.t1.c6;Parent=MALK_02745.t1
contig010	AUGUSTUS	CDS	170253	170390	0.57	-	0	ID=MALK_02745.t1.c5;Parent=MALK_02745.t1
contig010	AUGUSTUS	CDS	169994	170072	0.57	-	0	ID=MALK_02745.t1.c4;Parent=MALK_02745.t1
contig010	AUGUSTUS	CDS	169801	169959	0.57	-	0	ID=MALK_02745.t1.c3;Parent=MALK_02745.t1
contig010	AUGUSTUS	CDS	169090	169669	0.57	-	0	ID=MALK_02745.t1.c2;Parent=MALK_02745.t1
contig010	AUGUSTUS	CDS	168804	168986	0.57	-	0	ID=MALK_02745.t1.c1;Parent=MALK_02745.t1
contig010	AUGUSTUS	mRNA	168804	170615	0.09	-	.	ID=MALK_02745.t1;Parent=MALK_02745
contig010	AUGUSTUS	exon	170613	170615	.	-	.	ID=MALK_02745.t1.e7;Parent=MALK_02745.t1
contig010	AUGUSTUS	exon	170433	170568	.	-	.	ID=MALK_02745.t1.e6;Parent=MALK_02745.t1
contig010	AUGUSTUS	exon	170253	170390	.	-	.	ID=MALK_02745.t1.e5;Parent=MALK_02745.t1
contig010	AUGUSTUS	exon	169994	170072	.	-	.	ID=MALK_02745.t1.e4;Parent=MALK_02745.t1
contig010	AUGUSTUS	exon	169801	169959	.	-	.	ID=MALK_02745.t1.e3;Parent=MALK_02745.t1
contig010	AUGUSTUS	exon	169090	169669	.	-	.	ID=MALK_02745.t1.e2;Parent=MALK_02745.t1
contig010	AUGUSTUS	exon	168804	168986	.	-	.	ID=MALK_02745.t1.e1;Parent=MALK_02745.t1
contig010	AUGUSTUS	gene	170773	172119	0.65	+	.	ID=MALK_02746;prediction_source=augustus:contig010.g1017.t1

contig010	AUGUSTUS	CDS	170773	172119	0.65	+	0	ID=MALK_02746.t1.c1;Parent=MALK_02746.t1
contig010	AUGUSTUS	mRNA	170773	172119	0.65	+	.	ID=MALK_02746.t1;Parent=MALK_02746
contig010	AUGUSTUS	exon	170773	172119	0.65	+	.	ID=MALK_02746.t1.e1;Parent=MALK_02746.t1
contig010	AUGUSTUS	gene	172165	172525	0.98	-	.	ID=MALK_02747;prediction_source=braker_MRET:g1305.t1
contig010	AUGUSTUS	CDS	172472	172525	0.98	-	0	ID=MALK_02747.t1.c4;Parent=MALK_02747.t1
contig010	AUGUSTUS	CDS	172374	172431	0.98	-	0	ID=MALK_02747.t1.c3;Parent=MALK_02747.t1
contig010	AUGUSTUS	CDS	172252	172335	0.98	-	0	ID=MALK_02747.t1.c2;Parent=MALK_02747.t1
contig010	AUGUSTUS	CDS	172165	172211	0.98	-	0	ID=MALK_02747.t1.c1;Parent=MALK_02747.t1
contig010	AUGUSTUS	mRNA	172165	172525	0.98	-	.	ID=MALK_02747.t1;Parent=MALK_02747
contig010	AUGUSTUS	exon	172472	172525	.	-	.	ID=MALK_02747.t1.e4;Parent=MALK_02747.t1
contig010	AUGUSTUS	exon	172374	172431	.	-	.	ID=MALK_02747.t1.e3;Parent=MALK_02747.t1
contig010	AUGUSTUS	exon	172252	172335	.	-	.	ID=MALK_02747.t1.e2;Parent=MALK_02747.t1
contig010	AUGUSTUS	exon	172165	172211	.	-	.	ID=MALK_02747.t1.e1;Parent=MALK_02747.t1
contig010	maker	gene	172886	174472	.	+	.	ID=MALK_02748;prediction_source=maker_MRET:augustus_masked-contig010-processed-gene-1.58-mRNA-1
contig010	maker	CDS	172886	174472	.	+	0	ID=MALK_02748.t1.c1;Parent=MALK_02748.t1
contig010	maker	mRNA	172886	174472	.	+	.	ID=MALK_02748.t1;Parent=MALK_02748
contig010	maker	exon	172886	174472	.	+	.	ID=MALK_02748.t1.e1;Parent=MALK_02748.t1
contig010	AUGUSTUS	gene	175071	176117	0.51	-	.	ID=MALK_02749;prediction_source=braker_MRET:g1307.t1
contig010	AUGUSTUS	CDS	176115	176117	0.53	-	0	ID=MALK_02749.t1.c3;Parent=MALK_02749.t1
contig010	AUGUSTUS	CDS	176018	176080	0.53	-	0	ID=MALK_02749.t1.c2;Parent=MALK_02749.t1
contig010	AUGUSTUS	CDS	175071	175979	0.53	-	0	ID=MALK_02749.t1.c1;Parent=MALK_02749.t1
contig010	AUGUSTUS	mRNA	175071	176117	0.51	-	.	ID=MALK_02749.t1;Parent=MALK_02749
contig010	AUGUSTUS	exon	176115	176117	.	-	.	ID=MALK_02749.t1.e3;Parent=MALK_02749.t1
contig010	AUGUSTUS	exon	176018	176080	.	-	.	ID=MALK_02749.t1.e2;Parent=MALK_02749.t1
contig010	AUGUSTUS	exon	175071	175979	.	-	.	ID=MALK_02749.t1.e1;Parent=MALK_02749.t1
contig010	AUGUSTUS	gene	176262	178571	0.99	+	.	ID=MALK_02750;prediction_source=braker_MRET:g1308.t1
contig010	AUGUSTUS	CDS	176262	178571	0.99	+	0	ID=MALK_02750.t1.c1;Parent=MALK_02750.t1
contig010	AUGUSTUS	mRNA	176262	178571	0.99	+	.	ID=MALK_02750.t1;Parent=MALK_02750
contig010	AUGUSTUS	exon	176262	178571	.	+	.	ID=MALK_02750.t1.e1;Parent=MALK_02750.t1
contig010	AUGUSTUS	gene	178813	180642	0.33	+	.	ID=MALK_02751;prediction_source=braker_MRET:g1309.t1
contig010	AUGUSTUS	CDS	178813	180642	0.33	+	0	ID=MALK_02751.t1.c1;Parent=MALK_02751.t1
contig010	AUGUSTUS	mRNA	178813	180642	0.33	+	.	ID=MALK_02751.t1;Parent=MALK_02751
contig010	AUGUSTUS	exon	178813	180642	.	+	.	ID=MALK_02751.t1.e1;Parent=MALK_02751.t1
contig010	AUGUSTUS	gene	180872	181323	0.41	-	.	ID=MALK_02752;prediction_source=braker_MRET:g1310.t1
contig010	AUGUSTUS	CDS	181110	181323	0.41	-	0	ID=MALK_02752.t1.c2;Parent=MALK_02752.t1
contig010	AUGUSTUS	CDS	180872	181068	0.41	-	0	ID=MALK_02752.t1.c1;Parent=MALK_02752.t1
contig010	AUGUSTUS	mRNA	180872	181323	0.41	-	.	ID=MALK_02752.t1;Parent=MALK_02752
contig010	AUGUSTUS	exon	181110	181323	.	-	.	ID=MALK_02752.t1.e2;Parent=MALK_02752.t1
contig010	AUGUSTUS	exon	180872	181068	.	-	.	ID=MALK_02752.t1.e1;Parent=MALK_02752.t1
contig010	AUGUSTUS	gene	181590	182498	0.18	-	.	ID=MALK_02753;prediction_source=braker_MRET:g1311.t1
contig010	AUGUSTUS	CDS	182466	182498	0.58	-	0	ID=MALK_02753.t1.c3;Parent=MALK_02753.t1
contig010	AUGUSTUS	CDS	182225	182420	0.58	-	0	ID=MALK_02753.t1.c2;Parent=MALK_02753.t1
contig010	AUGUSTUS	CDS	181590	182194	0.58	-	0	ID=MALK_02753.t1.c1;Parent=MALK_02753.t1

contig010	AUGUSTUS	mRNA	181590	182498	0.18	-	.	ID=MALK_02753.t1;Parent=MALK_02753
contig010	AUGUSTUS	exon	182466	182498	.	-	.	ID=MALK_02753.t1.e3;Parent=MALK_02753.t1
contig010	AUGUSTUS	exon	182225	182420	.	-	.	ID=MALK_02753.t1.e2;Parent=MALK_02753.t1
contig010	AUGUSTUS	exon	181590	182194	.	-	.	ID=MALK_02753.t1.e1;Parent=MALK_02753.t1
contig010	AUGUSTUS	gene	182679	182968	0.64	+	.	ID=MALK_02754;prediction_source=braker_MRET:g1312.t1
contig010	AUGUSTUS	CDS	182679	182724	1	+	0	ID=MALK_02754.t1.c1;Parent=MALK_02754.t1
contig010	AUGUSTUS	CDS	182766	182968	1	+	0	ID=MALK_02754.t1.c2;Parent=MALK_02754.t1
contig010	AUGUSTUS	mRNA	182679	182968	0.64	+	.	ID=MALK_02754.t1;Parent=MALK_02754
contig010	AUGUSTUS	exon	182679	182724	.	+	.	ID=MALK_02754.t1.e1;Parent=MALK_02754.t1
contig010	AUGUSTUS	exon	182766	182968	.	+	.	ID=MALK_02754.t1.e2;Parent=MALK_02754.t1
contig010	AUGUSTUS	gene	183621	186821	0.97	+	.	ID=MALK_02755;prediction_source=augustus:contig010.g1023.t1
contig010	AUGUSTUS	CDS	183621	186821	0.97	+	0	ID=MALK_02755.t1.c1;Parent=MALK_02755.t1
contig010	AUGUSTUS	mRNA	183621	186821	0.97	+	.	ID=MALK_02755.t1;Parent=MALK_02755
contig010	AUGUSTUS	exon	183621	186821	0.97	+	.	ID=MALK_02755.t1.e1;Parent=MALK_02755.t1
contig010	maker	gene	186995	188698	.	+	.	ID=MALK_02756;prediction_source=maker_MRET:augustus_masked-contig010-processed-gene-1.61-mRNA-1
contig010	maker	CDS	186995	188698	.	+	0	ID=MALK_02756.t1.c1;Parent=MALK_02756.t1
contig010	maker	mRNA	186995	188698	.	+	.	ID=MALK_02756.t1;Parent=MALK_02756
contig010	maker	exon	186995	188698	.	+	.	ID=MALK_02756.t1.e1;Parent=MALK_02756.t1
contig010	AUGUSTUS	gene	188826	191663	1	+	.	ID=MALK_02757;prediction_source=augustus:contig010.g1026.t1
contig010	AUGUSTUS	CDS	188826	191663	1	+	0	ID=MALK_02757.t1.c1;Parent=MALK_02757.t1
contig010	AUGUSTUS	mRNA	188826	191663	1	+	.	ID=MALK_02757.t1;Parent=MALK_02757
contig010	AUGUSTUS	exon	188826	191663	1	+	.	ID=MALK_02757.t1.e1;Parent=MALK_02757.t1
contig010	AUGUSTUS	gene	191865	195193	0.78	-	.	ID=MALK_02758;prediction_source=braker_MRET:g1316.t1
contig010	AUGUSTUS	CDS	192076	195193	0.92	-	0	ID=MALK_02758.t1.c2;Parent=MALK_02758.t1
contig010	AUGUSTUS	CDS	191865	192034	0.92	-	0	ID=MALK_02758.t1.c1;Parent=MALK_02758.t1
contig010	AUGUSTUS	mRNA	191865	195193	0.78	-	.	ID=MALK_02758.t1;Parent=MALK_02758
contig010	AUGUSTUS	exon	192076	195193	.	-	.	ID=MALK_02758.t1.e2;Parent=MALK_02758.t1
contig010	AUGUSTUS	exon	191865	192034	.	-	.	ID=MALK_02758.t1.e1;Parent=MALK_02758.t1
contig010	maker	gene	195634	198273	.	-	.	ID=MALK_02759;prediction_source=maker_MRET:augustus_masked-contig010-processed-gene-2.2-mRNA-1
contig010	maker	CDS	195634	198273	.	-	0	ID=MALK_02759.t1.c1;Parent=MALK_02759.t1
contig010	maker	mRNA	195634	198273	.	-	.	ID=MALK_02759.t1;Parent=MALK_02759
contig010	maker	exon	195634	198273	.	-	.	ID=MALK_02759.t1.e1;Parent=MALK_02759.t1
contig010	maker	gene	198475	202533	.	+	.	ID=MALK_02760;prediction_source=maker_MRET:augustus_masked-contig010-processed-gene-2.0-mRNA-1
contig010	maker	CDS	198475	202533	.	+	0	ID=MALK_02760.t1.c1;Parent=MALK_02760.t1
contig010	maker	mRNA	198475	202533	.	+	.	ID=MALK_02760.t1;Parent=MALK_02760
contig010	maker	exon	198475	202533	.	+	.	ID=MALK_02760.t1.e1;Parent=MALK_02760.t1
contig010	AUGUSTUS	gene	202790	203772	0.75	-	.	ID=MALK_02761;prediction_source=braker_MRET:g1319.t1
contig010	AUGUSTUS	CDS	203009	203772	0.75	-	0	ID=MALK_02761.t1.c2;Parent=MALK_02761.t1
contig010	AUGUSTUS	CDS	202790	202967	0.75	-	0	ID=MALK_02761.t1.c1;Parent=MALK_02761.t1
contig010	AUGUSTUS	mRNA	202790	203772	0.75	-	.	ID=MALK_02761.t1;Parent=MALK_02761
contig010	AUGUSTUS	exon	203009	203772	.	-	.	ID=MALK_02761.t1.e2;Parent=MALK_02761.t1
contig010	AUGUSTUS	exon	202790	202967	.	-	.	ID=MALK_02761.t1.e1;Parent=MALK_02761.t1
contig010	AUGUSTUS	gene	204098	204811	0.66	-	.	ID=MALK_02762;prediction_source=augustus:contig010.g1032.t1

contig010	AUGUSTUS	CDS	204809	204811	0.7	-	0	ID=MALK_02762.t1.c2;Parent=MALK_02762.t1
contig010	AUGUSTUS	CDS	204098	204670	0.7	-	0	ID=MALK_02762.t1.c1;Parent=MALK_02762.t1
contig010	AUGUSTUS	mRNA	204098	204811	0.66	-	.	ID=MALK_02762.t1;Parent=MALK_02762
contig010	AUGUSTUS	exon	204809	204811	0.7	-	.	ID=MALK_02762.t1.e2;Parent=MALK_02762.t1
contig010	AUGUSTUS	exon	204098	204670	0.7	-	.	ID=MALK_02762.t1.e1;Parent=MALK_02762.t1
contig010	AUGUSTUS	gene	205078	205521	0.79	+	.	ID=MALK_02763;prediction_source=braker_MRET:g1321.t1
contig010	AUGUSTUS	CDS	205078	205521	0.79	+	0	ID=MALK_02763.t1.c1;Parent=MALK_02763.t1
contig010	AUGUSTUS	mRNA	205078	205521	0.79	+	.	ID=MALK_02763.t1;Parent=MALK_02763
contig010	AUGUSTUS	exon	205078	205521	.	+	.	ID=MALK_02763.t1.e1;Parent=MALK_02763.t1
contig010	AUGUSTUS	gene	205660	208704	0.72	-	.	ID=MALK_02764;prediction_source=augustus:contig010.g1034.t1
contig010	AUGUSTUS	CDS	205660	208704	0.72	-	0	ID=MALK_02764.t1.c1;Parent=MALK_02764.t1
contig010	AUGUSTUS	mRNA	205660	208704	0.72	-	.	ID=MALK_02764.t1;Parent=MALK_02764
contig010	AUGUSTUS	exon	205660	208704	0.72	-	.	ID=MALK_02764.t1.e1;Parent=MALK_02764.t1
contig010	AUGUSTUS	gene	209033	212170	0.41	-	.	ID=MALK_02765;prediction_source=augustus:contig010.g1036.t1
contig010	AUGUSTUS	CDS	209033	212170	0.41	-	0	ID=MALK_02765.t1.c1;Parent=MALK_02765.t1
contig010	AUGUSTUS	mRNA	209033	212170	0.41	-	.	ID=MALK_02765.t1;Parent=MALK_02765
contig010	AUGUSTUS	exon	209033	212170	0.41	-	.	ID=MALK_02765.t1.e1;Parent=MALK_02765.t1
contig010	maker	gene	212528	215146	.	-	.	ID=MALK_02766;prediction_source=maker_MRET:augustus_masked-contig010-processed-gene-2.26-mRNA-1
contig010	maker	CDS	212528	215146	.	-	0	ID=MALK_02766.t1.c1;Parent=MALK_02766.t1
contig010	maker	mRNA	212528	215146	.	-	.	ID=MALK_02766.t1;Parent=MALK_02766
contig010	maker	exon	212528	215146	.	-	.	ID=MALK_02766.t1.e1;Parent=MALK_02766.t1
contig010	maker	gene	215390	216814	.	+	.	ID=MALK_02767;prediction_source=maker_MRET:augustus_masked-contig010-processed-gene-2.4-mRNA-1
contig010	maker	CDS	215390	216814	.	+	0	ID=MALK_02767.t1.c1;Parent=MALK_02767.t1
contig010	maker	mRNA	215390	216814	.	+	.	ID=MALK_02767.t1;Parent=MALK_02767
contig010	maker	exon	215390	216814	.	+	.	ID=MALK_02767.t1.e1;Parent=MALK_02767.t1
contig010	maker	gene	216879	217322	.	-	.	ID=MALK_02768;prediction_source=maker_MRET:augustus_masked-contig010-processed-gene-2.27-mRNA-1
contig010	maker	CDS	216879	217322	.	-	0	ID=MALK_02768.t1.c1;Parent=MALK_02768.t1
contig010	maker	mRNA	216879	217322	.	-	.	ID=MALK_02768.t1;Parent=MALK_02768
contig010	maker	exon	216879	217322	.	-	.	ID=MALK_02768.t1.e1;Parent=MALK_02768.t1
contig010	maker	gene	217673	218292	.	+	.	ID=MALK_02769;prediction_source=maker_MRET:augustus_masked-contig010-processed-gene-2.5-mRNA-1
contig010	maker	CDS	217673	217801	.	+	0	ID=MALK_02769.t1.c1;Parent=MALK_02769.t1
contig010	maker	CDS	218071	218292	.	+	0	ID=MALK_02769.t1.c2;Parent=MALK_02769.t1
contig010	maker	mRNA	217673	218292	.	+	.	ID=MALK_02769.t1;Parent=MALK_02769
contig010	maker	exon	217673	217801	.	+	.	ID=MALK_02769.t1.e1;Parent=MALK_02769.t1
contig010	maker	exon	218071	218292	.	+	.	ID=MALK_02769.t1.e2;Parent=MALK_02769.t1
contig010	AUGUSTUS	gene	218401	219567	0.46	+	.	ID=MALK_02770;prediction_source=augustus:contig010.g1043.t1
contig010	AUGUSTUS	CDS	218401	219360	0.52	+	0	ID=MALK_02770.t1.c1;Parent=MALK_02770.t1
contig010	AUGUSTUS	CDS	219430	219567	0.52	+	0	ID=MALK_02770.t1.c2;Parent=MALK_02770.t1
contig010	AUGUSTUS	mRNA	218401	219567	0.46	+	.	ID=MALK_02770.t1;Parent=MALK_02770
contig010	AUGUSTUS	exon	218401	219360	0.52	+	.	ID=MALK_02770.t1.e1;Parent=MALK_02770.t1
contig010	AUGUSTUS	exon	219430	219567	0.52	+	.	ID=MALK_02770.t1.e2;Parent=MALK_02770.t1
contig010	maker	gene	220934	224959	.	+	.	ID=MALK_02771;prediction_source=maker_MRET:augustus_masked-contig010-processed-gene-2.7-mRNA-1
contig010	maker	CDS	220934	224959	.	+	0	ID=MALK_02771.t1.c1;Parent=MALK_02771.t1

contig010	maker	mRNA	220934	224959	.	+	.	ID=MALK_02771.t1;Parent=MALK_02771
contig010	maker	exon	220934	224959	.	+	.	ID=MALK_02771.t1.e1;Parent=MALK_02771.t1
contig010	AUGUSTUS	gene	225380	226231	0.98	+	.	ID=MALK_02772;prediction_source=augustus:contig010.g1049.t1
contig010	AUGUSTUS	CDS	225380	226231	0.98	+	0	ID=MALK_02772.t1.c1;Parent=MALK_02772.t1
contig010	AUGUSTUS	mRNA	225380	226231	0.98	+	.	ID=MALK_02772.t1;Parent=MALK_02772
contig010	AUGUSTUS	exon	225380	226231	0.98	+	.	ID=MALK_02772.t1.e1;Parent=MALK_02772.t1
contig010	AUGUSTUS	gene	226225	228864	0.7	-	.	ID=MALK_02773;prediction_source=augustus:contig010.g1050.t1
contig010	AUGUSTUS	CDS	226225	228864	0.7	-	0	ID=MALK_02773.t1.c1;Parent=MALK_02773.t1
contig010	AUGUSTUS	mRNA	226225	228864	0.7	-	.	ID=MALK_02773.t1;Parent=MALK_02773
contig010	AUGUSTUS	exon	226225	228864	0.7	-	.	ID=MALK_02773.t1.e1;Parent=MALK_02773.t1
contig010	maker	gene	229272	231431	.	-	.	ID=MALK_02774;prediction_source=maker_MRET:augustus_masked-contig010-processed-gene-2.29-mRNA-1
contig010	maker	CDS	229272	231431	.	-	0	ID=MALK_02774.t1.c1;Parent=MALK_02774.t1
contig010	maker	mRNA	229272	231431	.	-	.	ID=MALK_02774.t1;Parent=MALK_02774
contig010	maker	exon	229272	231431	.	-	.	ID=MALK_02774.t1.e1;Parent=MALK_02774.t1
contig010	maker	gene	231506	232207	.	+	.	ID=MALK_02775;prediction_source=maker_MRET:augustus_masked-contig010-processed-gene-2.9-mRNA-1
contig010	maker	CDS	231506	232207	.	+	0	ID=MALK_02775.t1.c1;Parent=MALK_02775.t1
contig010	maker	mRNA	231506	232207	.	+	.	ID=MALK_02775.t1;Parent=MALK_02775
contig010	maker	exon	231506	232207	.	+	.	ID=MALK_02775.t1.e1;Parent=MALK_02775.t1
contig010	AUGUSTUS	gene	232217	232777	0.41	-	.	ID=MALK_02776;prediction_source=augustus:contig010.g1053.t1
contig010	AUGUSTUS	CDS	232217	232777	0.41	-	0	ID=MALK_02776.t1.c1;Parent=MALK_02776.t1
contig010	AUGUSTUS	mRNA	232217	232777	0.41	-	.	ID=MALK_02776.t1;Parent=MALK_02776
contig010	AUGUSTUS	exon	232217	232777	0.41	-	.	ID=MALK_02776.t1.e1;Parent=MALK_02776.t1
contig010	AUGUSTUS	gene	233082	234662	0.66	-	.	ID=MALK_02777;prediction_source=augustus:contig010.g1054.t1
contig010	AUGUSTUS	CDS	233082	234662	0.66	-	0	ID=MALK_02777.t1.c1;Parent=MALK_02777.t1
contig010	AUGUSTUS	mRNA	233082	234662	0.66	-	.	ID=MALK_02777.t1;Parent=MALK_02777
contig010	AUGUSTUS	exon	233082	234662	0.66	-	.	ID=MALK_02777.t1.e1;Parent=MALK_02777.t1
contig010	AUGUSTUS	gene	234867	235995	0.55	+	.	ID=MALK_02778;prediction_source=braker_MRET:g1334.t1
contig010	AUGUSTUS	CDS	234867	234942	0.93	+	0	ID=MALK_02778.t1.c1;Parent=MALK_02778.t1
contig010	AUGUSTUS	CDS	234989	235995	0.93	+	0	ID=MALK_02778.t1.c2;Parent=MALK_02778.t1
contig010	AUGUSTUS	mRNA	234867	235995	0.55	+	.	ID=MALK_02778.t1;Parent=MALK_02778
contig010	AUGUSTUS	exon	234867	234942	.	+	.	ID=MALK_02778.t1.e1;Parent=MALK_02778.t1
contig010	AUGUSTUS	exon	234989	235995	.	+	.	ID=MALK_02778.t1.e2;Parent=MALK_02778.t1
contig010	AUGUSTUS	gene	236156	237286	0.27	+	.	ID=MALK_02779;prediction_source=augustus:contig010.g1057.t1
contig010	AUGUSTUS	CDS	236156	237286	0.27	+	0	ID=MALK_02779.t1.c1;Parent=MALK_02779.t1
contig010	AUGUSTUS	mRNA	236156	237286	0.27	+	.	ID=MALK_02779.t1;Parent=MALK_02779
contig010	AUGUSTUS	exon	236156	237286	0.27	+	.	ID=MALK_02779.t1.e1;Parent=MALK_02779.t1
contig010	AUGUSTUS	gene	237562	238761	0.92	+	.	ID=MALK_02780;prediction_source=braker_MRET:g1336.t1
contig010	AUGUSTUS	CDS	237562	238761	0.92	+	0	ID=MALK_02780.t1.c1;Parent=MALK_02780.t1
contig010	AUGUSTUS	mRNA	237562	238761	0.92	+	.	ID=MALK_02780.t1;Parent=MALK_02780
contig010	AUGUSTUS	exon	237562	238761	.	+	.	ID=MALK_02780.t1.e1;Parent=MALK_02780.t1
contig010	maker	gene	239737	241005	.	-	.	ID=MALK_02781;prediction_source=maker_MRET:augustus_masked-contig010-processed-gene-2.31-mRNA-1
contig010	maker	CDS	239737	241005	.	-	0	ID=MALK_02781.t1.c1;Parent=MALK_02781.t1
contig010	maker	mRNA	239737	241005	.	-	.	ID=MALK_02781.t1;Parent=MALK_02781

contig010	maker	exon	239737	241005	.	-	.	ID=MALK_02781.t1.e1;Parent=MALK_02781.t1
contig010	AUGUSTUS	gene	241523	243139	0.53	+	.	ID=MALK_02782;prediction_source=augustus:contig010.g1061.t1
contig010	AUGUSTUS	CDS	241523	243139	0.53	+	0	ID=MALK_02782.t1.c1;Parent=MALK_02782.t1
contig010	AUGUSTUS	mRNA	241523	243139	0.53	+	.	ID=MALK_02782.t1;Parent=MALK_02782
contig010	AUGUSTUS	exon	241523	243139	0.53	+	.	ID=MALK_02782.t1.e1;Parent=MALK_02782.t1
contig010	AUGUSTUS	gene	243410	244864	0.92	+	.	ID=MALK_02783;prediction_source=augustus:contig010.g1062.t1
contig010	AUGUSTUS	CDS	243410	244864	0.92	+	0	ID=MALK_02783.t1.c1;Parent=MALK_02783.t1
contig010	AUGUSTUS	mRNA	243410	244864	0.92	+	.	ID=MALK_02783.t1;Parent=MALK_02783
contig010	AUGUSTUS	exon	243410	244864	0.92	+	.	ID=MALK_02783.t1.e1;Parent=MALK_02783.t1
contig010	maker	gene	244899	245297	.	-	.	ID=MALK_02784;prediction_source=maker_MRET:augustus_masked-contig010-processed-gene-2.32-mRNA-1
contig010	maker	CDS	244899	245297	.	-	0	ID=MALK_02784.t1.c1;Parent=MALK_02784.t1
contig010	maker	mRNA	244899	245297	.	-	.	ID=MALK_02784.t1;Parent=MALK_02784
contig010	maker	exon	244899	245297	.	-	.	ID=MALK_02784.t1.e1;Parent=MALK_02784.t1
contig010	maker	gene	245680	247119	.	-	.	ID=MALK_02785;prediction_source=maker_MRET:augustus_masked-contig010-processed-gene-2.33-mRNA-1
contig010	maker	CDS	245680	247119	.	-	0	ID=MALK_02785.t1.c1;Parent=MALK_02785.t1
contig010	maker	mRNA	245680	247119	.	-	.	ID=MALK_02785.t1;Parent=MALK_02785
contig010	maker	exon	245680	247119	.	-	.	ID=MALK_02785.t1.e1;Parent=MALK_02785.t1
contig010	maker	gene	247824	248771	.	+	.	ID=MALK_02786;prediction_source=maker_MRET:augustus_masked-contig010-processed-gene-2.15-mRNA-1
contig010	maker	CDS	247824	248771	.	+	0	ID=MALK_02786.t1.c1;Parent=MALK_02786.t1
contig010	maker	mRNA	247824	248771	.	+	.	ID=MALK_02786.t1;Parent=MALK_02786
contig010	maker	exon	247824	248771	.	+	.	ID=MALK_02786.t1.e1;Parent=MALK_02786.t1
contig010	AUGUSTUS	gene	249038	249389	0.36	+	.	ID=MALK_02787;prediction_source=braker_MRET:g1343.t1
contig010	AUGUSTUS	CDS	249038	249317	1	+	0	ID=MALK_02787.t1.c1;Parent=MALK_02787.t1
contig010	AUGUSTUS	CDS	249349	249389	1	+	0	ID=MALK_02787.t1.c2;Parent=MALK_02787.t1
contig010	AUGUSTUS	mRNA	249038	249389	0.36	+	.	ID=MALK_02787.t1;Parent=MALK_02787
contig010	AUGUSTUS	exon	249038	249317	.	+	.	ID=MALK_02787.t1.e1;Parent=MALK_02787.t1
contig010	AUGUSTUS	exon	249349	249389	.	+	.	ID=MALK_02787.t1.e2;Parent=MALK_02787.t1
contig010	maker	gene	249444	250718	.	-	.	ID=MALK_02788;prediction_source=maker_MRET:augustus_masked-contig010-processed-gene-2.34-mRNA-1
contig010	maker	CDS	250086	250718	.	-	0	ID=MALK_02788.t1.c1;Parent=MALK_02788.t1
contig010	maker	CDS	249745	249969	.	-	0	ID=MALK_02788.t1.c2;Parent=MALK_02788.t1
contig010	maker	CDS	249444	249653	.	-	0	ID=MALK_02788.t1.c3;Parent=MALK_02788.t1
contig010	maker	mRNA	249444	250718	.	-	.	ID=MALK_02788.t1;Parent=MALK_02788
contig010	maker	exon	250086	250718	.	-	.	ID=MALK_02788.t1.e1;Parent=MALK_02788.t1
contig010	maker	exon	249745	249969	.	-	.	ID=MALK_02788.t1.e2;Parent=MALK_02788.t1
contig010	maker	exon	249444	249653	.	-	.	ID=MALK_02788.t1.e3;Parent=MALK_02788.t1
contig010	AUGUSTUS	gene	250812	252257	0.93	-	.	ID=MALK_02789;prediction_source=augustus:contig010.g1067.t1
contig010	AUGUSTUS	CDS	250812	252257	0.93	-	0	ID=MALK_02789.t1.c1;Parent=MALK_02789.t1
contig010	AUGUSTUS	mRNA	250812	252257	0.93	-	.	ID=MALK_02789.t1;Parent=MALK_02789
contig010	AUGUSTUS	exon	250812	252257	0.93	-	.	ID=MALK_02789.t1.e1;Parent=MALK_02789.t1
contig010	maker	gene	252472	253935	.	+	.	ID=MALK_02790;prediction_source=maker_MRET:augustus_masked-contig010-processed-gene-2.16-mRNA-1
contig010	maker	CDS	252472	253935	.	+	0	ID=MALK_02790.t1.c1;Parent=MALK_02790.t1
contig010	maker	mRNA	252472	253935	.	+	.	ID=MALK_02790.t1;Parent=MALK_02790
contig010	maker	exon	252472	253935	.	+	.	ID=MALK_02790.t1.e1;Parent=MALK_02790.t1

contig010	maker	gene	253991	254443	.	-	.	ID=MALK_02791;prediction_source=maker_MRET:augustus_masked-contig010-processed-gene-2.36-mRNA-1
contig010	maker	CDS	253991	254443	.	-	0	ID=MALK_02791.t1.c1;Parent=MALK_02791.t1
contig010	maker	mRNA	253991	254443	.	-	.	ID=MALK_02791.t1;Parent=MALK_02791
contig010	maker	exon	253991	254443	.	-	.	ID=MALK_02791.t1.e1;Parent=MALK_02791.t1
contig010	maker	gene	254578	256404	.	-	.	ID=MALK_02792;prediction_source=maker_MRET:augustus_masked-contig010-processed-gene-2.37-mRNA-1
contig010	maker	CDS	254578	256404	.	-	0	ID=MALK_02792.t1.c1;Parent=MALK_02792.t1
contig010	maker	mRNA	254578	256404	.	-	.	ID=MALK_02792.t1;Parent=MALK_02792
contig010	maker	exon	254578	256404	.	-	.	ID=MALK_02792.t1.e1;Parent=MALK_02792.t1
contig010	AUGUSTUS	gene	256573	257325	0.99	+	.	ID=MALK_02793;prediction_source=braker_MRET:g1349.t1
contig010	AUGUSTUS	CDS	256573	256620	1	+	0	ID=MALK_02793.t1.c1;Parent=MALK_02793.t1
contig010	AUGUSTUS	CDS	256651	256748	1	+	0	ID=MALK_02793.t1.c2;Parent=MALK_02793.t1
contig010	AUGUSTUS	CDS	256914	256940	1	+	0	ID=MALK_02793.t1.c3;Parent=MALK_02793.t1
contig010	AUGUSTUS	CDS	256968	257017	1	+	0	ID=MALK_02793.t1.c4;Parent=MALK_02793.t1
contig010	AUGUSTUS	CDS	257156	257325	1	+	0	ID=MALK_02793.t1.c5;Parent=MALK_02793.t1
contig010	AUGUSTUS	mRNA	256573	257325	0.99	+	.	ID=MALK_02793.t1;Parent=MALK_02793
contig010	AUGUSTUS	exon	256573	256620	.	+	.	ID=MALK_02793.t1.e1;Parent=MALK_02793.t1
contig010	AUGUSTUS	exon	256651	256748	.	+	.	ID=MALK_02793.t1.e2;Parent=MALK_02793.t1
contig010	AUGUSTUS	exon	256914	256940	.	+	.	ID=MALK_02793.t1.e3;Parent=MALK_02793.t1
contig010	AUGUSTUS	exon	256968	257017	.	+	.	ID=MALK_02793.t1.e4;Parent=MALK_02793.t1
contig010	AUGUSTUS	exon	257156	257325	.	+	.	ID=MALK_02793.t1.e5;Parent=MALK_02793.t1
contig010	AUGUSTUS	gene	257342	258481	0.71	-	.	ID=MALK_02794;prediction_source=augustus:contig010.g1072.t1
contig010	AUGUSTUS	CDS	257342	258481	0.71	-	0	ID=MALK_02794.t1.c1;Parent=MALK_02794.t1
contig010	AUGUSTUS	mRNA	257342	258481	0.71	-	.	ID=MALK_02794.t1;Parent=MALK_02794
contig010	AUGUSTUS	exon	257342	258481	0.71	-	.	ID=MALK_02794.t1.e1;Parent=MALK_02794.t1
contig010	AUGUSTUS	gene	258518	260179	0.46	-	.	ID=MALK_02795;prediction_source=augustus:contig010.g1073.t1
contig010	AUGUSTUS	CDS	258518	260179	0.46	-	0	ID=MALK_02795.t1.c1;Parent=MALK_02795.t1
contig010	AUGUSTUS	mRNA	258518	260179	0.46	-	.	ID=MALK_02795.t1;Parent=MALK_02795
contig010	AUGUSTUS	exon	258518	260179	0.46	-	.	ID=MALK_02795.t1.e1;Parent=MALK_02795.t1
contig010	AUGUSTUS	gene	260291	261323	0.89	-	.	ID=MALK_02796;prediction_source=braker_MRET:g1352.t1
contig010	AUGUSTUS	CDS	261242	261323	0.93	-	0	ID=MALK_02796.t1.c2;Parent=MALK_02796.t1
contig010	AUGUSTUS	CDS	260291	261195	0.93	-	0	ID=MALK_02796.t1.c1;Parent=MALK_02796.t1
contig010	AUGUSTUS	mRNA	260291	261323	0.89	-	.	ID=MALK_02796.t1;Parent=MALK_02796
contig010	AUGUSTUS	exon	261242	261323	.	-	.	ID=MALK_02796.t1.e2;Parent=MALK_02796.t1
contig010	AUGUSTUS	exon	260291	261195	.	-	.	ID=MALK_02796.t1.e1;Parent=MALK_02796.t1
contig010	AUGUSTUS	gene	261346	263811	0.45	-	.	ID=MALK_02797;prediction_source=braker_MRET:g1353.t1
contig010	AUGUSTUS	CDS	261346	263811	0.45	-	0	ID=MALK_02797.t1.c1;Parent=MALK_02797.t1
contig010	AUGUSTUS	mRNA	261346	263811	0.45	-	.	ID=MALK_02797.t1;Parent=MALK_02797
contig010	AUGUSTUS	exon	261346	263811	.	-	.	ID=MALK_02797.t1.e1;Parent=MALK_02797.t1
contig010	maker	gene	263883	265520	.	+	.	ID=MALK_02798;prediction_source=maker_MRET:augustus_masked-contig010-processed-gene-2.17-mRNA-1
contig010	maker	CDS	263883	265520	.	+	0	ID=MALK_02798.t1.c1;Parent=MALK_02798.t1
contig010	maker	mRNA	263883	265520	.	+	.	ID=MALK_02798.t1;Parent=MALK_02798
contig010	maker	exon	263883	265520	.	+	.	ID=MALK_02798.t1.e1;Parent=MALK_02798.t1
contig010	maker	gene	265597	266403	.	-	.	ID=MALK_02799;prediction_source=maker_MRET:augustus_masked-contig010-processed-gene-2.41-mRNA-1

contig010	maker	CDS	265597	266403	.	-	0	ID=MALK_02799.t1.c1;Parent=MALK_02799.t1
contig010	maker	mRNA	265597	266403	.	-	.	ID=MALK_02799.t1;Parent=MALK_02799
contig010	maker	exon	265597	266403	.	-	.	ID=MALK_02799.t1.e1;Parent=MALK_02799.t1
contig010	AUGUSTUS	gene	266828	268750	1	+	.	ID=MALK_02800;prediction_source=augustus:contig010.g1077.t1
contig010	AUGUSTUS	CDS	266828	268750	1	+	0	ID=MALK_02800.t1.c1;Parent=MALK_02800.t1
contig010	AUGUSTUS	mRNA	266828	268750	1	+	.	ID=MALK_02800.t1;Parent=MALK_02800
contig010	AUGUSTUS	exon	266828	268750	1	+	.	ID=MALK_02800.t1.e1;Parent=MALK_02800.t1
contig010	AUGUSTUS	gene	268880	269479	1	-	.	ID=MALK_02801;prediction_source=braker_MRET:g1357.t1
contig010	AUGUSTUS	CDS	269416	269479	1	-	0	ID=MALK_02801.t1.c2;Parent=MALK_02801.t1
contig010	AUGUSTUS	CDS	268880	269388	1	-	0	ID=MALK_02801.t1.c1;Parent=MALK_02801.t1
contig010	AUGUSTUS	mRNA	268880	269479	1	-	.	ID=MALK_02801.t1;Parent=MALK_02801
contig010	AUGUSTUS	exon	269416	269479	.	-	.	ID=MALK_02801.t1.e2;Parent=MALK_02801.t1
contig010	AUGUSTUS	exon	268880	269388	.	-	.	ID=MALK_02801.t1.e1;Parent=MALK_02801.t1
contig010	AUGUSTUS	gene	269655	270950	0.77	+	.	ID=MALK_02802;prediction_source=augustus:contig010.g1079.t1
contig010	AUGUSTUS	CDS	269655	270950	0.77	+	0	ID=MALK_02802.t1.c1;Parent=MALK_02802.t1
contig010	AUGUSTUS	mRNA	269655	270950	0.77	+	.	ID=MALK_02802.t1;Parent=MALK_02802
contig010	AUGUSTUS	exon	269655	270950	0.77	+	.	ID=MALK_02802.t1.e1;Parent=MALK_02802.t1
contig010	maker	gene	271017	272132	.	-	.	ID=MALK_02803;prediction_source=maker_MRET:augustus_masked-contig010-processed-gene-2.43-mRNA-1
contig010	maker	CDS	271017	272132	.	-	0	ID=MALK_02803.t1.c1;Parent=MALK_02803.t1
contig010	maker	mRNA	271017	272132	.	-	.	ID=MALK_02803.t1;Parent=MALK_02803
contig010	maker	exon	271017	272132	.	-	.	ID=MALK_02803.t1.e1;Parent=MALK_02803.t1
contig010	AUGUSTUS	gene	272343	276914	0.36	-	.	ID=MALK_02804;prediction_source=augustus:contig010.g1081.t1
contig010	AUGUSTUS	CDS	272343	276914	0.36	-	0	ID=MALK_02804.t1.c1;Parent=MALK_02804.t1
contig010	AUGUSTUS	mRNA	272343	276914	0.36	-	.	ID=MALK_02804.t1;Parent=MALK_02804
contig010	AUGUSTUS	exon	272343	276914	0.36	-	.	ID=MALK_02804.t1.e1;Parent=MALK_02804.t1
contig010	AUGUSTUS	gene	276974	279076	0.28	-	.	ID=MALK_02805;prediction_source=augustus:contig010.g1083.t1
contig010	AUGUSTUS	CDS	276974	279076	0.28	-	0	ID=MALK_02805.t1.c1;Parent=MALK_02805.t1
contig010	AUGUSTUS	mRNA	276974	279076	0.28	-	.	ID=MALK_02805.t1;Parent=MALK_02805
contig010	AUGUSTUS	exon	276974	279076	0.28	-	.	ID=MALK_02805.t1.e1;Parent=MALK_02805.t1
contig010	AUGUSTUS	gene	279205	281592	0.36	-	.	ID=MALK_02806;prediction_source=augustus:contig010.g1085.t1
contig010	AUGUSTUS	CDS	279205	281592	0.36	-	0	ID=MALK_02806.t1.c1;Parent=MALK_02806.t1
contig010	AUGUSTUS	mRNA	279205	281592	0.36	-	.	ID=MALK_02806.t1;Parent=MALK_02806
contig010	AUGUSTUS	exon	279205	281592	0.36	-	.	ID=MALK_02806.t1.e1;Parent=MALK_02806.t1
contig010	maker	gene	281655	283409	.	+	.	ID=MALK_02807;prediction_source=maker_MRET:augustus_masked-contig010-processed-gene-2.20-mRNA-1
contig010	maker	CDS	281655	283409	.	+	0	ID=MALK_02807.t1.c1;Parent=MALK_02807.t1
contig010	maker	mRNA	281655	283409	.	+	.	ID=MALK_02807.t1;Parent=MALK_02807
contig010	maker	exon	281655	283409	.	+	.	ID=MALK_02807.t1.e1;Parent=MALK_02807.t1
contig010	AUGUSTUS	gene	283622	284226	0.84	-	.	ID=MALK_02808;prediction_source=braker_MRET:g1364.t1
contig010	AUGUSTUS	CDS	284163	284226	0.84	-	0	ID=MALK_02808.t1.c2;Parent=MALK_02808.t1
contig010	AUGUSTUS	CDS	283622	284061	0.84	-	0	ID=MALK_02808.t1.c1;Parent=MALK_02808.t1
contig010	AUGUSTUS	mRNA	283622	284226	0.84	-	.	ID=MALK_02808.t1;Parent=MALK_02808
contig010	AUGUSTUS	exon	284163	284226	.	-	.	ID=MALK_02808.t1.e2;Parent=MALK_02808.t1
contig010	AUGUSTUS	exon	283622	284061	.	-	.	ID=MALK_02808.t1.e1;Parent=MALK_02808.t1

contig010	AUGUSTUS	gene	285897	287171	0.83	-	.	ID=MALK_02809;prediction_source=augustus:contig010.g1089.t1
contig010	AUGUSTUS	CDS	285897	287171	0.83	-	0	ID=MALK_02809.t1.c1;Parent=MALK_02809.t1
contig010	AUGUSTUS	mRNA	285897	287171	0.83	-	.	ID=MALK_02809.t1;Parent=MALK_02809
contig010	AUGUSTUS	exon	285897	287171	0.83	-	.	ID=MALK_02809.t1.e1;Parent=MALK_02809.t1
contig010	maker	gene	287868	290483	.	-	.	ID=MALK_02810;prediction_source=maker_MRET:augustus_masked-contig010-processed-gene-2.49-mRNA-1
contig010	maker	CDS	287868	290483	.	-	0	ID=MALK_02810.t1.c1;Parent=MALK_02810.t1
contig010	maker	mRNA	287868	290483	.	-	.	ID=MALK_02810.t1;Parent=MALK_02810
contig010	maker	exon	287868	290483	.	-	.	ID=MALK_02810.t1.e1;Parent=MALK_02810.t1
contig010	AUGUSTUS	gene	290615	291604	0.56	+	.	ID=MALK_02811;prediction_source=augustus:contig010.g1092.t1
contig010	AUGUSTUS	CDS	290615	291604	0.56	+	0	ID=MALK_02811.t1.c1;Parent=MALK_02811.t1
contig010	AUGUSTUS	mRNA	290615	291604	0.56	+	.	ID=MALK_02811.t1;Parent=MALK_02811
contig010	AUGUSTUS	exon	290615	291604	0.56	+	.	ID=MALK_02811.t1.e1;Parent=MALK_02811.t1
contig010	maker	gene	291608	293902	.	-	.	ID=MALK_02812;prediction_source=maker_MRET:augustus_masked-contig010-processed-gene-2.50-mRNA-1
contig010	maker	CDS	291608	293902	.	-	0	ID=MALK_02812.t1.c1;Parent=MALK_02812.t1
contig010	maker	mRNA	291608	293902	.	-	.	ID=MALK_02812.t1;Parent=MALK_02812
contig010	maker	exon	291608	293902	.	-	.	ID=MALK_02812.t1.e1;Parent=MALK_02812.t1
contig010	AUGUSTUS	gene	294144	294752	0.58	+	.	ID=MALK_02813;prediction_source=braker_MRET:g1369.t1
contig010	AUGUSTUS	CDS	294144	294231	1	+	0	ID=MALK_02813.t1.c1;Parent=MALK_02813.t1
contig010	AUGUSTUS	CDS	294272	294390	1	+	0	ID=MALK_02813.t1.c2;Parent=MALK_02813.t1
contig010	AUGUSTUS	CDS	294422	294706	1	+	0	ID=MALK_02813.t1.c3;Parent=MALK_02813.t1
contig010	AUGUSTUS	CDS	294735	294752	1	+	0	ID=MALK_02813.t1.c4;Parent=MALK_02813.t1
contig010	AUGUSTUS	mRNA	294144	294752	0.58	+	.	ID=MALK_02813.t1;Parent=MALK_02813
contig010	AUGUSTUS	exon	294144	294231	.	+	.	ID=MALK_02813.t1.e1;Parent=MALK_02813.t1
contig010	AUGUSTUS	exon	294272	294390	.	+	.	ID=MALK_02813.t1.e2;Parent=MALK_02813.t1
contig010	AUGUSTUS	exon	294422	294706	.	+	.	ID=MALK_02813.t1.e3;Parent=MALK_02813.t1
contig010	AUGUSTUS	exon	294735	294752	.	+	.	ID=MALK_02813.t1.e4;Parent=MALK_02813.t1
contig010	AUGUSTUS	gene	294862	296310	0.81	-	.	ID=MALK_02814;prediction_source=braker_MRET:g1370.t1
contig010	AUGUSTUS	CDS	296049	296310	1	-	0	ID=MALK_02814.t1.c2;Parent=MALK_02814.t1
contig010	AUGUSTUS	CDS	294862	296015	1	-	0	ID=MALK_02814.t1.c1;Parent=MALK_02814.t1
contig010	AUGUSTUS	mRNA	294862	296310	0.81	-	.	ID=MALK_02814.t1;Parent=MALK_02814
contig010	AUGUSTUS	exon	296049	296310	.	-	.	ID=MALK_02814.t1.e2;Parent=MALK_02814.t1
contig010	AUGUSTUS	exon	294862	296015	.	-	.	ID=MALK_02814.t1.e1;Parent=MALK_02814.t1
contig010	AUGUSTUS	gene	296645	297397	0.97	+	.	ID=MALK_02815;prediction_source=augustus:contig010.g1097.t1
contig010	AUGUSTUS	CDS	296645	297397	0.97	+	0	ID=MALK_02815.t1.c1;Parent=MALK_02815.t1
contig010	AUGUSTUS	mRNA	296645	297397	0.97	+	.	ID=MALK_02815.t1;Parent=MALK_02815
contig010	AUGUSTUS	exon	296645	297397	0.97	+	.	ID=MALK_02815.t1.e1;Parent=MALK_02815.t1
contig010	AUGUSTUS	gene	297403	297987	0.92	-	.	ID=MALK_02816;prediction_source=braker_MRET:g1372.t1
contig010	AUGUSTUS	CDS	297403	297987	0.92	-	0	ID=MALK_02816.t1.c1;Parent=MALK_02816.t1
contig010	AUGUSTUS	mRNA	297403	297987	0.92	-	.	ID=MALK_02816.t1;Parent=MALK_02816
contig010	AUGUSTUS	exon	297403	297987	.	-	.	ID=MALK_02816.t1.e1;Parent=MALK_02816.t1
contig010	AUGUSTUS	gene	298087	298860	1	+	.	ID=MALK_02817;prediction_source=braker_MRET:g1373.t1
contig010	AUGUSTUS	CDS	298087	298860	1	+	0	ID=MALK_02817.t1.c1;Parent=MALK_02817.t1
contig010	AUGUSTUS	mRNA	298087	298860	1	+	.	ID=MALK_02817.t1;Parent=MALK_02817

contig010	AUGUSTUS	exon	298087	298860	.	+	.	ID=MALK_02817.t1.e1;Parent=MALK_02817.t1
contig010	AUGUSTUS	gene	298864	299796	0.84	-	.	ID=MALK_02818;prediction_source=braker_MRET:g1374.t1
contig010	AUGUSTUS	CDS	298864	299796	0.84	-	0	ID=MALK_02818.t1.c1;Parent=MALK_02818.t1
contig010	AUGUSTUS	mRNA	298864	299796	0.84	-	.	ID=MALK_02818.t1;Parent=MALK_02818
contig010	AUGUSTUS	exon	298864	299796	.	-	.	ID=MALK_02818.t1.e1;Parent=MALK_02818.t1
contig010	AUGUSTUS	gene	299934	300392	0.55	+	.	ID=MALK_02819;prediction_source=augustus:contig010.g1100.t1
contig010	AUGUSTUS	CDS	299934	300392	0.55	+	0	ID=MALK_02819.t1.c1;Parent=MALK_02819.t1
contig010	AUGUSTUS	mRNA	299934	300392	0.55	+	.	ID=MALK_02819.t1;Parent=MALK_02819
contig010	AUGUSTUS	exon	299934	300392	0.55	+	.	ID=MALK_02819.t1.e1;Parent=MALK_02819.t1
contig010	AUGUSTUS	gene	300420	301652	0.48	-	.	ID=MALK_02820;prediction_source=braker_MRET:g1376.t1
contig010	AUGUSTUS	CDS	300420	301652	0.48	-	0	ID=MALK_02820.t1.c1;Parent=MALK_02820.t1
contig010	AUGUSTUS	mRNA	300420	301652	0.48	-	.	ID=MALK_02820.t1;Parent=MALK_02820
contig010	AUGUSTUS	exon	300420	301652	.	-	.	ID=MALK_02820.t1.e1;Parent=MALK_02820.t1
contig010	AUGUSTUS	gene	302859	304604	0.56	-	.	ID=MALK_02821;prediction_source=augustus:contig010.g1101.t1
contig010	AUGUSTUS	CDS	302859	304604	0.56	-	0	ID=MALK_02821.t1.c1;Parent=MALK_02821.t1
contig010	AUGUSTUS	mRNA	302859	304604	0.56	-	.	ID=MALK_02821.t1;Parent=MALK_02821
contig010	AUGUSTUS	exon	302859	304604	0.56	-	.	ID=MALK_02821.t1.e1;Parent=MALK_02821.t1
contig010	AUGUSTUS	gene	304694	306112	0.99	+	.	ID=MALK_02822;prediction_source=braker_MRET:g1378.t1
contig010	AUGUSTUS	CDS	304694	306112	0.99	+	0	ID=MALK_02822.t1.c1;Parent=MALK_02822.t1
contig010	AUGUSTUS	mRNA	304694	306112	0.99	+	.	ID=MALK_02822.t1;Parent=MALK_02822
contig010	AUGUSTUS	exon	304694	306112	.	+	.	ID=MALK_02822.t1.e1;Parent=MALK_02822.t1
contig010	AUGUSTUS	gene	306114	307351	0.78	-	.	ID=MALK_02823;prediction_source=braker_MRET:g1379.t1
contig010	AUGUSTUS	CDS	307209	307351	0.78	-	0	ID=MALK_02823.t1.c2;Parent=MALK_02823.t1
contig010	AUGUSTUS	CDS	306114	307179	0.78	-	0	ID=MALK_02823.t1.c1;Parent=MALK_02823.t1
contig010	AUGUSTUS	mRNA	306114	307351	0.78	-	.	ID=MALK_02823.t1;Parent=MALK_02823
contig010	AUGUSTUS	exon	307209	307351	.	-	.	ID=MALK_02823.t1.e2;Parent=MALK_02823.t1
contig010	AUGUSTUS	exon	306114	307179	.	-	.	ID=MALK_02823.t1.e1;Parent=MALK_02823.t1
contig010	AUGUSTUS	gene	307559	308311	1	+	.	ID=MALK_02824;prediction_source=augustus:contig010.g1104.t1
contig010	AUGUSTUS	CDS	307559	308311	1	+	0	ID=MALK_02824.t1.c1;Parent=MALK_02824.t1
contig010	AUGUSTUS	mRNA	307559	308311	1	+	.	ID=MALK_02824.t1;Parent=MALK_02824
contig010	AUGUSTUS	exon	307559	308311	1	+	.	ID=MALK_02824.t1.e1;Parent=MALK_02824.t1
contig010	AUGUSTUS	gene	308397	312131	0.98	+	.	ID=MALK_02825;prediction_source=augustus:contig010.g1105.t1
contig010	AUGUSTUS	CDS	308397	312131	0.98	+	0	ID=MALK_02825.t1.c1;Parent=MALK_02825.t1
contig010	AUGUSTUS	mRNA	308397	312131	0.98	+	.	ID=MALK_02825.t1;Parent=MALK_02825
contig010	AUGUSTUS	exon	308397	312131	0.98	+	.	ID=MALK_02825.t1.e1;Parent=MALK_02825.t1
contig010	maker	gene	312170	315688	.	+	.	ID=MALK_02826;prediction_source=maker_MRET:augustus_masked-contig010-processed-gene-3.7-mRNA-1
contig010	maker	CDS	312170	315688	.	+	0	ID=MALK_02826.t1.c1;Parent=MALK_02826.t1
contig010	maker	mRNA	312170	315688	.	+	.	ID=MALK_02826.t1;Parent=MALK_02826
contig010	maker	exon	312170	315688	.	+	.	ID=MALK_02826.t1.e1;Parent=MALK_02826.t1
contig010	AUGUSTUS	gene	315681	316259	0.98	-	.	ID=MALK_02827;prediction_source=augustus:contig010.g1107.t1
contig010	AUGUSTUS	CDS	315681	316259	0.98	-	0	ID=MALK_02827.t1.c1;Parent=MALK_02827.t1
contig010	AUGUSTUS	mRNA	315681	316259	0.98	-	.	ID=MALK_02827.t1;Parent=MALK_02827
contig010	AUGUSTUS	exon	315681	316259	0.98	-	.	ID=MALK_02827.t1.e1;Parent=MALK_02827.t1

contig010	maker	gene	316346	317935	.	+	.	ID=MALK_02828;prediction_source=maker_MRET:augustus_masked-contig010-processed-gene-3.8-mRNA-1
contig010	maker	CDS	316346	317935	.	+	0	ID=MALK_02828.t1.c1;Parent=MALK_02828.t1
contig010	maker	mRNA	316346	317935	.	+	.	ID=MALK_02828.t1;Parent=MALK_02828
contig010	maker	exon	316346	317935	.	+	.	ID=MALK_02828.t1.e1;Parent=MALK_02828.t1
contig010	AUGUSTUS	gene	317932	318933	1	-	.	ID=MALK_02829;prediction_source=augustus:contig010.g1110.t1
contig010	AUGUSTUS	CDS	317932	318933	1	-	0	ID=MALK_02829.t1.c1;Parent=MALK_02829.t1
contig010	AUGUSTUS	mRNA	317932	318933	1	-	.	ID=MALK_02829.t1;Parent=MALK_02829
contig010	AUGUSTUS	exon	317932	318933	1	-	.	ID=MALK_02829.t1.e1;Parent=MALK_02829.t1
contig010	AUGUSTUS	gene	319010	320332	0.3	-	.	ID=MALK_02830;prediction_source=augustus:contig010.g1111.t1
contig010	AUGUSTUS	CDS	319010	320332	0.3	-	0	ID=MALK_02830.t1.c1;Parent=MALK_02830.t1
contig010	AUGUSTUS	mRNA	319010	320332	0.3	-	.	ID=MALK_02830.t1;Parent=MALK_02830
contig010	AUGUSTUS	exon	319010	320332	0.3	-	.	ID=MALK_02830.t1.e1;Parent=MALK_02830.t1
contig010	AUGUSTUS	gene	320408	321199	0.36	+	.	ID=MALK_02831;prediction_source=braker_MRET:g1387.t1
contig010	AUGUSTUS	CDS	320408	320414	0.96	+	0	ID=MALK_02831.t1.c1;Parent=MALK_02831.t1
contig010	AUGUSTUS	CDS	320443	320840	0.96	+	0	ID=MALK_02831.t1.c2;Parent=MALK_02831.t1
contig010	AUGUSTUS	CDS	320885	321199	0.96	+	0	ID=MALK_02831.t1.c3;Parent=MALK_02831.t1
contig010	AUGUSTUS	mRNA	320408	321199	0.36	+	.	ID=MALK_02831.t1;Parent=MALK_02831
contig010	AUGUSTUS	exon	320408	320414	.	+	.	ID=MALK_02831.t1.e1;Parent=MALK_02831.t1
contig010	AUGUSTUS	exon	320443	320840	.	+	.	ID=MALK_02831.t1.e2;Parent=MALK_02831.t1
contig010	AUGUSTUS	exon	320885	321199	.	+	.	ID=MALK_02831.t1.e3;Parent=MALK_02831.t1
contig010	AUGUSTUS	gene	321205	323060	0.22	-	.	ID=MALK_02832;prediction_source=augustus:contig010.g1113.t1
contig010	AUGUSTUS	CDS	322929	323060	0.47	-	0	ID=MALK_02832.t1.c2;Parent=MALK_02832.t1
contig010	AUGUSTUS	CDS	321205	322827	0.47	-	0	ID=MALK_02832.t1.c1;Parent=MALK_02832.t1
contig010	AUGUSTUS	mRNA	321205	323060	0.22	-	.	ID=MALK_02832.t1;Parent=MALK_02832
contig010	AUGUSTUS	exon	322929	323060	0.47	-	.	ID=MALK_02832.t1.e2;Parent=MALK_02832.t1
contig010	AUGUSTUS	exon	321205	322827	0.47	-	.	ID=MALK_02832.t1.e1;Parent=MALK_02832.t1
contig010	AUGUSTUS	gene	323343	323867	0.63	+	.	ID=MALK_02833;prediction_source=augustus:contig010.g1114.t1
contig010	AUGUSTUS	CDS	323343	323867	0.63	+	0	ID=MALK_02833.t1.c1;Parent=MALK_02833.t1
contig010	AUGUSTUS	mRNA	323343	323867	0.63	+	.	ID=MALK_02833.t1;Parent=MALK_02833
contig010	AUGUSTUS	exon	323343	323867	0.63	+	.	ID=MALK_02833.t1.e1;Parent=MALK_02833.t1
contig010	AUGUSTUS	gene	323868	326012	0.96	-	.	ID=MALK_02834;prediction_source=augustus:contig010.g1115.t1
contig010	AUGUSTUS	CDS	323868	326012	0.96	-	0	ID=MALK_02834.t1.c1;Parent=MALK_02834.t1
contig010	AUGUSTUS	mRNA	323868	326012	0.96	-	.	ID=MALK_02834.t1;Parent=MALK_02834
contig010	AUGUSTUS	exon	323868	326012	0.96	-	.	ID=MALK_02834.t1.e1;Parent=MALK_02834.t1
contig010	AUGUSTUS	gene	326011	327873	0.88	+	.	ID=MALK_02835;prediction_source=braker_MRET:g1390.t1
contig010	AUGUSTUS	CDS	326011	327873	0.88	+	0	ID=MALK_02835.t1.c1;Parent=MALK_02835.t1
contig010	AUGUSTUS	mRNA	326011	327873	0.88	+	.	ID=MALK_02835.t1;Parent=MALK_02835
contig010	AUGUSTUS	exon	326011	327873	.	+	.	ID=MALK_02835.t1.e1;Parent=MALK_02835.t1
contig010	maker	gene	327875	329509	.	-	.	ID=MALK_02836;prediction_source=maker_MRET:augustus_masked-contig010-processed-gene-3.29-mRNA-1
contig010	maker	CDS	327875	329509	.	-	0	ID=MALK_02836.t1.c1;Parent=MALK_02836.t1
contig010	maker	mRNA	327875	329509	.	-	.	ID=MALK_02836.t1;Parent=MALK_02836
contig010	maker	exon	327875	329509	.	-	.	ID=MALK_02836.t1.e1;Parent=MALK_02836.t1
contig010	AUGUSTUS	gene	329620	331167	1	+	.	ID=MALK_02837;prediction_source=augustus:contig010.g1120.t1

contig010	AUGUSTUS	CDS	329620	331167	1	+	0	ID=MALK_02837.t1.c1;Parent=MALK_02837.t1
contig010	AUGUSTUS	mRNA	329620	331167	1	+	.	ID=MALK_02837.t1;Parent=MALK_02837
contig010	AUGUSTUS	exon	329620	331167	1	+	.	ID=MALK_02837.t1.e1;Parent=MALK_02837.t1
contig010	maker	gene	331193	337837	.	-	.	ID=MALK_02838;prediction_source=maker_MRET:augustus_masked-contig010-processed-gene-3.30-mRNA-1
contig010	maker	CDS	331193	337837	.	-	0	ID=MALK_02838.t1.c1;Parent=MALK_02838.t1
contig010	maker	mRNA	331193	337837	.	-	.	ID=MALK_02838.t1;Parent=MALK_02838
contig010	maker	exon	331193	337837	.	-	.	ID=MALK_02838.t1.e1;Parent=MALK_02838.t1
contig010	AUGUSTUS	gene	338139	339290	0.91	-	.	ID=MALK_02839;prediction_source=augustus:contig010.g1124.t1
contig010	AUGUSTUS	CDS	338139	339290	0.91	-	0	ID=MALK_02839.t1.c1;Parent=MALK_02839.t1
contig010	AUGUSTUS	mRNA	338139	339290	0.91	-	.	ID=MALK_02839.t1;Parent=MALK_02839
contig010	AUGUSTUS	exon	338139	339290	0.91	-	.	ID=MALK_02839.t1.e1;Parent=MALK_02839.t1
contig010	maker	gene	339452	342268	.	+	.	ID=MALK_02840;prediction_source=maker_MRET:augustus_masked-contig010-processed-gene-3.11-mRNA-1
contig010	maker	CDS	339452	342268	.	+	0	ID=MALK_02840.t1.c1;Parent=MALK_02840.t1
contig010	maker	mRNA	339452	342268	.	+	.	ID=MALK_02840.t1;Parent=MALK_02840
contig010	maker	exon	339452	342268	.	+	.	ID=MALK_02840.t1.e1;Parent=MALK_02840.t1
contig010	maker	gene	342280	343293	.	-	.	ID=MALK_02841;prediction_source=maker_MRET:augustus_masked-contig010-processed-gene-3.32-mRNA-1
contig010	maker	CDS	342280	343293	.	-	0	ID=MALK_02841.t1.c1;Parent=MALK_02841.t1
contig010	maker	mRNA	342280	343293	.	-	.	ID=MALK_02841.t1;Parent=MALK_02841
contig010	maker	exon	342280	343293	.	-	.	ID=MALK_02841.t1.e1;Parent=MALK_02841.t1
contig010	AUGUSTUS	gene	343598	345882	0.23	-	.	ID=MALK_02842;prediction_source=augustus:contig010.g1127.t1
contig010	AUGUSTUS	CDS	345125	345882	0.23	-	0	ID=MALK_02842.t1.c3;Parent=MALK_02842.t1
contig010	AUGUSTUS	CDS	344535	345059	0.23	-	0	ID=MALK_02842.t1.c2;Parent=MALK_02842.t1
contig010	AUGUSTUS	CDS	343598	344228	0.23	-	0	ID=MALK_02842.t1.c1;Parent=MALK_02842.t1
contig010	AUGUSTUS	mRNA	343598	345882	0.23	-	.	ID=MALK_02842.t1;Parent=MALK_02842
contig010	AUGUSTUS	exon	345125	345882	0.23	-	.	ID=MALK_02842.t1.e3;Parent=MALK_02842.t1
contig010	AUGUSTUS	exon	344535	345059	0.23	-	.	ID=MALK_02842.t1.e2;Parent=MALK_02842.t1
contig010	AUGUSTUS	exon	343598	344228	0.23	-	.	ID=MALK_02842.t1.e1;Parent=MALK_02842.t1
contig010	maker	gene	346093	348316	.	+	.	ID=MALK_02843;prediction_source=maker_MRET:augustus_masked-contig010-processed-gene-3.12-mRNA-1
contig010	maker	CDS	346093	346540	.	+	0	ID=MALK_02843.t1.c1;Parent=MALK_02843.t1
contig010	maker	CDS	346659	348316	.	+	0	ID=MALK_02843.t1.c2;Parent=MALK_02843.t1
contig010	maker	mRNA	346093	348316	.	+	.	ID=MALK_02843.t1;Parent=MALK_02843
contig010	maker	exon	346093	346540	.	+	.	ID=MALK_02843.t1.e1;Parent=MALK_02843.t1
contig010	maker	exon	346659	348316	.	+	.	ID=MALK_02843.t1.e2;Parent=MALK_02843.t1
contig010	AUGUSTUS	gene	348767	350665	0.65	+	.	ID=MALK_02844;prediction_source=augustus:contig010.g1130.t1
contig010	AUGUSTUS	CDS	348767	350665	0.65	+	0	ID=MALK_02844.t1.c1;Parent=MALK_02844.t1
contig010	AUGUSTUS	mRNA	348767	350665	0.65	+	.	ID=MALK_02844.t1;Parent=MALK_02844
contig010	AUGUSTUS	exon	348767	350665	0.65	+	.	ID=MALK_02844.t1.e1;Parent=MALK_02844.t1
contig010	AUGUSTUS	gene	350931	355760	1	-	.	ID=MALK_02845;prediction_source=augustus:contig010.g1131.t1
contig010	AUGUSTUS	CDS	350931	355760	1	-	0	ID=MALK_02845.t1.c1;Parent=MALK_02845.t1
contig010	AUGUSTUS	mRNA	350931	355760	1	-	.	ID=MALK_02845.t1;Parent=MALK_02845
contig010	AUGUSTUS	exon	350931	355760	1	-	.	ID=MALK_02845.t1.e1;Parent=MALK_02845.t1
contig010	AUGUSTUS	gene	356097	357923	0.9	+	.	ID=MALK_02846;prediction_source=augustus:contig010.g1132.t1
contig010	AUGUSTUS	CDS	356097	357923	0.9	+	0	ID=MALK_02846.t1.c1;Parent=MALK_02846.t1

contig010	AUGUSTUS	mRNA	356097	357923	0.9	+	.	ID=MALK_02846.t1;Parent=MALK_02846
contig010	AUGUSTUS	exon	356097	357923	0.9	+	.	ID=MALK_02846.t1.e1;Parent=MALK_02846.t1
contig010	AUGUSTUS	gene	357924	360362	1	-	.	ID=MALK_02847;prediction_source=augustus:contig010.g1133.t1
contig010	AUGUSTUS	CDS	357924	360362	1	-	0	ID=MALK_02847.t1.c1;Parent=MALK_02847.t1
contig010	AUGUSTUS	mRNA	357924	360362	1	-	.	ID=MALK_02847.t1;Parent=MALK_02847
contig010	AUGUSTUS	exon	357924	360362	1	-	.	ID=MALK_02847.t1.e1;Parent=MALK_02847.t1
contig010	AUGUSTUS	gene	360792	361790	0.5	+	.	ID=MALK_02848;prediction_source=braker_MRET:g1403.t1
contig010	AUGUSTUS	CDS	360792	361790	0.5	+	0	ID=MALK_02848.t1.c1;Parent=MALK_02848.t1
contig010	AUGUSTUS	mRNA	360792	361790	0.5	+	.	ID=MALK_02848.t1;Parent=MALK_02848
contig010	AUGUSTUS	exon	360792	361790	.	+	.	ID=MALK_02848.t1.e1;Parent=MALK_02848.t1
contig010	AUGUSTUS	gene	361793	363138	0.65	-	.	ID=MALK_02849;prediction_source=braker_MRET:g1404.t1
contig010	AUGUSTUS	CDS	362512	363138	0.68	-	0	ID=MALK_02849.t1.c2;Parent=MALK_02849.t1
contig010	AUGUSTUS	CDS	361793	362296	0.68	-	0	ID=MALK_02849.t1.c1;Parent=MALK_02849.t1
contig010	AUGUSTUS	mRNA	361793	363138	0.65	-	.	ID=MALK_02849.t1;Parent=MALK_02849
contig010	AUGUSTUS	exon	362512	363138	.	-	.	ID=MALK_02849.t1.e2;Parent=MALK_02849.t1
contig010	AUGUSTUS	exon	361793	362296	.	-	.	ID=MALK_02849.t1.e1;Parent=MALK_02849.t1
contig010	maker	gene	363235	364470	.	-	.	ID=MALK_02850;prediction_source=maker_MRET:augustus_masked-contig010-processed-gene-3.36-mRNA-1
contig010	maker	CDS	363235	364470	.	-	0	ID=MALK_02850.t1.c1;Parent=MALK_02850.t1
contig010	maker	mRNA	363235	364470	.	-	.	ID=MALK_02850.t1;Parent=MALK_02850
contig010	maker	exon	363235	364470	.	-	.	ID=MALK_02850.t1.e1;Parent=MALK_02850.t1
contig010	AUGUSTUS	gene	364500	366116	0.97	+	.	ID=MALK_02851;prediction_source=augustus:contig010.g1136.t1
contig010	AUGUSTUS	CDS	364500	366116	0.97	+	0	ID=MALK_02851.t1.c1;Parent=MALK_02851.t1
contig010	AUGUSTUS	mRNA	364500	366116	0.97	+	.	ID=MALK_02851.t1;Parent=MALK_02851
contig010	AUGUSTUS	exon	364500	366116	0.97	+	.	ID=MALK_02851.t1.e1;Parent=MALK_02851.t1
contig010	AUGUSTUS	gene	366113	367300	1	-	.	ID=MALK_02852;prediction_source=augustus:contig010.g1137.t1
contig010	AUGUSTUS	CDS	366113	367300	1	-	0	ID=MALK_02852.t1.c1;Parent=MALK_02852.t1
contig010	AUGUSTUS	mRNA	366113	367300	1	-	.	ID=MALK_02852.t1;Parent=MALK_02852
contig010	AUGUSTUS	exon	366113	367300	1	-	.	ID=MALK_02852.t1.e1;Parent=MALK_02852.t1
contig010	maker	gene	367398	371488	.	-	.	ID=MALK_02853;prediction_source=maker_MRET:augustus_masked-contig010-processed-gene-3.38-mRNA-1
contig010	maker	CDS	371421	371488	.	-	0	ID=MALK_02853.t1.c1;Parent=MALK_02853.t1
contig010	maker	CDS	367398	370545	.	-	0	ID=MALK_02853.t1.c2;Parent=MALK_02853.t1
contig010	maker	mRNA	367398	371488	.	-	.	ID=MALK_02853.t1;Parent=MALK_02853
contig010	maker	exon	371421	371488	.	-	.	ID=MALK_02853.t1.e1;Parent=MALK_02853.t1
contig010	maker	exon	367398	370545	.	-	.	ID=MALK_02853.t1.e2;Parent=MALK_02853.t1
contig010	maker	gene	371767	372873	.	+	.	ID=MALK_02854;prediction_source=maker_MRET:augustus_masked-contig010-processed-gene-3.17-mRNA-1
contig010	maker	CDS	371767	372873	.	+	0	ID=MALK_02854.t1.c1;Parent=MALK_02854.t1
contig010	maker	mRNA	371767	372873	.	+	.	ID=MALK_02854.t1;Parent=MALK_02854
contig010	maker	exon	371767	372873	.	+	.	ID=MALK_02854.t1.e1;Parent=MALK_02854.t1
contig010	AUGUSTUS	gene	372884	375109	0.86	-	.	ID=MALK_02855;prediction_source=augustus:contig010.g1142.t1
contig010	AUGUSTUS	CDS	372884	375109	0.86	-	0	ID=MALK_02855.t1.c1;Parent=MALK_02855.t1
contig010	AUGUSTUS	mRNA	372884	375109	0.86	-	.	ID=MALK_02855.t1;Parent=MALK_02855
contig010	AUGUSTUS	exon	372884	375109	0.86	-	.	ID=MALK_02855.t1.e1;Parent=MALK_02855.t1
contig010	AUGUSTUS	gene	375338	377611	1	+	.	ID=MALK_02856;prediction_source=braker_MRET:g1411.t1

contig010	AUGUSTUS	CDS	375338	377611	1	+	0	ID=MALK_02856.t1.c1;Parent=MALK_02856.t1
contig010	AUGUSTUS	mRNA	375338	377611	1	+	.	ID=MALK_02856.t1;Parent=MALK_02856
contig010	AUGUSTUS	exon	375338	377611	.	+	.	ID=MALK_02856.t1.e1;Parent=MALK_02856.t1
contig010	AUGUSTUS	gene	377682	378741	0.28	-	.	ID=MALK_02857;prediction_source=braker_MRET:g1412.t1
contig010	AUGUSTUS	CDS	377798	378741	0.28	-	0	ID=MALK_02857.t1.c2;Parent=MALK_02857.t1
contig010	AUGUSTUS	CDS	377682	377745	0.28	-	0	ID=MALK_02857.t1.c1;Parent=MALK_02857.t1
contig010	AUGUSTUS	mRNA	377682	378741	0.28	-	.	ID=MALK_02857.t1;Parent=MALK_02857
contig010	AUGUSTUS	exon	377798	378741	.	-	.	ID=MALK_02857.t1.e2;Parent=MALK_02857.t1
contig010	AUGUSTUS	exon	377682	377745	.	-	.	ID=MALK_02857.t1.e1;Parent=MALK_02857.t1
contig010	AUGUSTUS	gene	378984	379429	0.83	-	.	ID=MALK_02858;prediction_source=braker_MRET:g1413.t1
contig010	AUGUSTUS	CDS	379427	379429	1	-	0	ID=MALK_02858.t1.c6;Parent=MALK_02858.t1
contig010	AUGUSTUS	CDS	379335	379383	1	-	0	ID=MALK_02858.t1.c5;Parent=MALK_02858.t1
contig010	AUGUSTUS	CDS	379274	379289	1	-	0	ID=MALK_02858.t1.c4;Parent=MALK_02858.t1
contig010	AUGUSTUS	CDS	379144	379234	1	-	0	ID=MALK_02858.t1.c3;Parent=MALK_02858.t1
contig010	AUGUSTUS	CDS	379063	379104	1	-	0	ID=MALK_02858.t1.c2;Parent=MALK_02858.t1
contig010	AUGUSTUS	CDS	378984	379034	1	-	0	ID=MALK_02858.t1.c1;Parent=MALK_02858.t1
contig010	AUGUSTUS	mRNA	378984	379429	0.83	-	.	ID=MALK_02858.t1;Parent=MALK_02858
contig010	AUGUSTUS	exon	379427	379429	.	-	.	ID=MALK_02858.t1.e6;Parent=MALK_02858.t1
contig010	AUGUSTUS	exon	379335	379383	.	-	.	ID=MALK_02858.t1.e5;Parent=MALK_02858.t1
contig010	AUGUSTUS	exon	379274	379289	.	-	.	ID=MALK_02858.t1.e4;Parent=MALK_02858.t1
contig010	AUGUSTUS	exon	379144	379234	.	-	.	ID=MALK_02858.t1.e3;Parent=MALK_02858.t1
contig010	AUGUSTUS	exon	379063	379104	.	-	.	ID=MALK_02858.t1.e2;Parent=MALK_02858.t1
contig010	AUGUSTUS	exon	378984	379034	.	-	.	ID=MALK_02858.t1.e1;Parent=MALK_02858.t1
contig010	AUGUSTUS	gene	379585	380578	0.94	+	.	ID=MALK_02859;prediction_source=braker_MRET:g1414.t1
contig010	AUGUSTUS	CDS	379585	379646	0.94	+	0	ID=MALK_02859.t1.c1;Parent=MALK_02859.t1
contig010	AUGUSTUS	CDS	379675	379825	0.94	+	0	ID=MALK_02859.t1.c2;Parent=MALK_02859.t1
contig010	AUGUSTUS	CDS	379856	380578	0.94	+	0	ID=MALK_02859.t1.c3;Parent=MALK_02859.t1
contig010	AUGUSTUS	mRNA	379585	380578	0.94	+	.	ID=MALK_02859.t1;Parent=MALK_02859
contig010	AUGUSTUS	exon	379585	379646	.	+	.	ID=MALK_02859.t1.e1;Parent=MALK_02859.t1
contig010	AUGUSTUS	exon	379675	379825	.	+	.	ID=MALK_02859.t1.e2;Parent=MALK_02859.t1
contig010	AUGUSTUS	exon	379856	380578	.	+	.	ID=MALK_02859.t1.e3;Parent=MALK_02859.t1
contig010	AUGUSTUS	gene	381082	382128	0.87	+	.	ID=MALK_02860;prediction_source=augustus:contig010.g1146.t1
contig010	AUGUSTUS	CDS	381082	382128	0.87	+	0	ID=MALK_02860.t1.c1;Parent=MALK_02860.t1
contig010	AUGUSTUS	mRNA	381082	382128	0.87	+	.	ID=MALK_02860.t1;Parent=MALK_02860
contig010	AUGUSTUS	exon	381082	382128	0.87	+	.	ID=MALK_02860.t1.e1;Parent=MALK_02860.t1
contig010	AUGUSTUS	gene	382455	383092	0.81	+	.	ID=MALK_02861;prediction_source=augustus:contig010.g1147.t1
contig010	AUGUSTUS	CDS	382455	382558	0.81	+	0	ID=MALK_02861.t1.c1;Parent=MALK_02861.t1
contig010	AUGUSTUS	CDS	382672	383092	0.81	+	0	ID=MALK_02861.t1.c2;Parent=MALK_02861.t1
contig010	AUGUSTUS	mRNA	382455	383092	0.81	+	.	ID=MALK_02861.t1;Parent=MALK_02861
contig010	AUGUSTUS	exon	382455	382558	0.81	+	.	ID=MALK_02861.t1.e1;Parent=MALK_02861.t1
contig010	AUGUSTUS	exon	382672	383092	0.81	+	.	ID=MALK_02861.t1.e2;Parent=MALK_02861.t1
contig010	AUGUSTUS	gene	383250	384905	0.96	-	.	ID=MALK_02862;prediction_source=augustus:contig010.g1148.t1
contig010	AUGUSTUS	CDS	383250	384905	0.96	-	0	ID=MALK_02862.t1.c1;Parent=MALK_02862.t1

contig010	AUGUSTUS	mRNA	383250	384905	0.96	-	.	ID=MALK_02862.t1;Parent=MALK_02862
contig010	AUGUSTUS	exon	383250	384905	0.96	-	.	ID=MALK_02862.t1.e1;Parent=MALK_02862.t1
contig010	maker	gene	384959	386230	.	+	.	ID=MALK_02863;prediction_source=maker_MRET:augustus_masked-contig010-processed-gene-3.22-mRNA-1
contig010	maker	CDS	384959	386230	.	+	0	ID=MALK_02863.t1.c1;Parent=MALK_02863.t1
contig010	maker	mRNA	384959	386230	.	+	.	ID=MALK_02863.t1;Parent=MALK_02863
contig010	maker	exon	384959	386230	.	+	.	ID=MALK_02863.t1.e1;Parent=MALK_02863.t1
contig010	AUGUSTUS	gene	386910	388829	0.93	-	.	ID=MALK_02864;prediction_source=augustus:contig010.g1151.t1
contig010	AUGUSTUS	CDS	386910	388829	0.93	-	0	ID=MALK_02864.t1.c1;Parent=MALK_02864.t1
contig010	AUGUSTUS	mRNA	386910	388829	0.93	-	.	ID=MALK_02864.t1;Parent=MALK_02864
contig010	AUGUSTUS	exon	386910	388829	0.93	-	.	ID=MALK_02864.t1.e1;Parent=MALK_02864.t1
contig010	AUGUSTUS	gene	389296	391113	0.81	+	.	ID=MALK_02865;prediction_source=augustus:contig010.g1153.t1
contig010	AUGUSTUS	CDS	389296	391113	0.81	+	0	ID=MALK_02865.t1.c1;Parent=MALK_02865.t1
contig010	AUGUSTUS	mRNA	389296	391113	0.81	+	.	ID=MALK_02865.t1;Parent=MALK_02865
contig010	AUGUSTUS	exon	389296	391113	0.81	+	.	ID=MALK_02865.t1.e1;Parent=MALK_02865.t1
contig010	AUGUSTUS	gene	391326	392012	0.63	+	.	ID=MALK_02866;prediction_source=braker_MRET:g1421.t1
contig010	AUGUSTUS	CDS	391326	392012	0.63	+	0	ID=MALK_02866.t1.c1;Parent=MALK_02866.t1
contig010	AUGUSTUS	mRNA	391326	392012	0.63	+	.	ID=MALK_02866.t1;Parent=MALK_02866
contig010	AUGUSTUS	exon	391326	392012	.	+	.	ID=MALK_02866.t1.e1;Parent=MALK_02866.t1
contig010	AUGUSTUS	gene	392019	393155	0.38	-	.	ID=MALK_02867;prediction_source=braker_MRET:g1422.t1
contig010	AUGUSTUS	CDS	392019	393155	0.38	-	0	ID=MALK_02867.t1.c1;Parent=MALK_02867.t1
contig010	AUGUSTUS	mRNA	392019	393155	0.38	-	.	ID=MALK_02867.t1;Parent=MALK_02867
contig010	AUGUSTUS	exon	392019	393155	.	-	.	ID=MALK_02867.t1.e1;Parent=MALK_02867.t1
contig010	AUGUSTUS	gene	393738	394607	0.8	+	.	ID=MALK_02868;prediction_source=augustus:contig010.g1155.t1
contig010	AUGUSTUS	CDS	393738	394607	0.8	+	0	ID=MALK_02868.t1.c1;Parent=MALK_02868.t1
contig010	AUGUSTUS	mRNA	393738	394607	0.8	+	.	ID=MALK_02868.t1;Parent=MALK_02868
contig010	AUGUSTUS	exon	393738	394607	0.8	+	.	ID=MALK_02868.t1.e1;Parent=MALK_02868.t1
contig010	AUGUSTUS	gene	394886	395988	0.36	-	.	ID=MALK_02869;prediction_source=braker_MRET:g1424.t1
contig010	AUGUSTUS	CDS	394930	395988	0.36	-	0	ID=MALK_02869.t1.c2;Parent=MALK_02869.t1
contig010	AUGUSTUS	CDS	394886	394894	0.36	-	0	ID=MALK_02869.t1.c1;Parent=MALK_02869.t1
contig010	AUGUSTUS	mRNA	394886	395988	0.36	-	.	ID=MALK_02869.t1;Parent=MALK_02869
contig010	AUGUSTUS	exon	394930	395988	.	-	.	ID=MALK_02869.t1.e2;Parent=MALK_02869.t1
contig010	AUGUSTUS	exon	394886	394894	.	-	.	ID=MALK_02869.t1.e1;Parent=MALK_02869.t1
contig010	AUGUSTUS	gene	396023	399793	0.9	-	.	ID=MALK_02870;prediction_source=braker_MRET:g1425.t1
contig010	AUGUSTUS	CDS	396023	399793	0.9	-	0	ID=MALK_02870.t1.c1;Parent=MALK_02870.t1
contig010	AUGUSTUS	mRNA	396023	399793	0.9	-	.	ID=MALK_02870.t1;Parent=MALK_02870
contig010	AUGUSTUS	exon	396023	399793	.	-	.	ID=MALK_02870.t1.e1;Parent=MALK_02870.t1
contig010	maker	gene	399908	400624	.	+	.	ID=MALK_02871;prediction_source=maker_MRET:augustus_masked-contig010-processed-gene-4.0-mRNA-1
contig010	maker	CDS	399908	400624	.	+	0	ID=MALK_02871.t1.c1;Parent=MALK_02871.t1
contig010	maker	mRNA	399908	400624	.	+	.	ID=MALK_02871.t1;Parent=MALK_02871
contig010	maker	exon	399908	400624	.	+	.	ID=MALK_02871.t1.e1;Parent=MALK_02871.t1
contig010	AUGUSTUS	gene	400633	404013	0.95	-	.	ID=MALK_02872;prediction_source=augustus:contig010.g1161.t1
contig010	AUGUSTUS	CDS	400633	404013	0.95	-	0	ID=MALK_02872.t1.c1;Parent=MALK_02872.t1
contig010	AUGUSTUS	mRNA	400633	404013	0.95	-	.	ID=MALK_02872.t1;Parent=MALK_02872

contig010	AUGUSTUS	exon	400633	404013	0.95	-	.	ID=MALK_02872.t1.e1;Parent=MALK_02872.t1
contig010	AUGUSTUS	gene	404741	405469	0.68	-	.	ID=MALK_02873;prediction_source=braker_MRET:g1428.t1
contig010	AUGUSTUS	CDS	405437	405469	0.71	-	0	ID=MALK_02873.t1.c4;Parent=MALK_02873.t1
contig010	AUGUSTUS	CDS	405291	405341	0.71	-	0	ID=MALK_02873.t1.c3;Parent=MALK_02873.t1
contig010	AUGUSTUS	CDS	404866	405128	0.71	-	0	ID=MALK_02873.t1.c2;Parent=MALK_02873.t1
contig010	AUGUSTUS	CDS	404741	404822	0.71	-	0	ID=MALK_02873.t1.c1;Parent=MALK_02873.t1
contig010	AUGUSTUS	mRNA	404741	405469	0.68	-	.	ID=MALK_02873.t1;Parent=MALK_02873
contig010	AUGUSTUS	exon	405437	405469	.	-	.	ID=MALK_02873.t1.e4;Parent=MALK_02873.t1
contig010	AUGUSTUS	exon	405291	405341	.	-	.	ID=MALK_02873.t1.e3;Parent=MALK_02873.t1
contig010	AUGUSTUS	exon	404866	405128	.	-	.	ID=MALK_02873.t1.e2;Parent=MALK_02873.t1
contig010	AUGUSTUS	exon	404741	404822	.	-	.	ID=MALK_02873.t1.e1;Parent=MALK_02873.t1
contig010	AUGUSTUS	gene	405500	406309	0.5	+	.	ID=MALK_02874;prediction_source=braker_MRET:g1429.t1
contig010	AUGUSTUS	CDS	405500	406309	0.5	+	0	ID=MALK_02874.t1.c1;Parent=MALK_02874.t1
contig010	AUGUSTUS	mRNA	405500	406309	0.5	+	.	ID=MALK_02874.t1;Parent=MALK_02874
contig010	AUGUSTUS	exon	405500	406309	.	+	.	ID=MALK_02874.t1.e1;Parent=MALK_02874.t1
contig010	AUGUSTUS	gene	406377	407735	0.85	-	.	ID=MALK_02875;prediction_source=braker_MRET:g1430.t1
contig010	AUGUSTUS	CDS	406377	407735	0.85	-	0	ID=MALK_02875.t1.c1;Parent=MALK_02875.t1
contig010	AUGUSTUS	mRNA	406377	407735	0.85	-	.	ID=MALK_02875.t1;Parent=MALK_02875
contig010	AUGUSTUS	exon	406377	407735	.	-	.	ID=MALK_02875.t1.e1;Parent=MALK_02875.t1
contig010	maker	gene	408268	410196	.	+	.	ID=MALK_02876;prediction_source=maker_MRET:augustus_masked-contig010-processed-gene-4.3-mRNA-1
contig010	maker	CDS	408268	410196	.	+	0	ID=MALK_02876.t1.c1;Parent=MALK_02876.t1
contig010	maker	mRNA	408268	410196	.	+	.	ID=MALK_02876.t1;Parent=MALK_02876
contig010	maker	exon	408268	410196	.	+	.	ID=MALK_02876.t1.e1;Parent=MALK_02876.t1
contig010	maker	gene	410534	411715	.	+	.	ID=MALK_02877;prediction_source=maker_MRET:augustus_masked-contig010-processed-gene-4.4-mRNA-1
contig010	maker	CDS	410534	411715	.	+	0	ID=MALK_02877.t1.c1;Parent=MALK_02877.t1
contig010	maker	mRNA	410534	411715	.	+	.	ID=MALK_02877.t1;Parent=MALK_02877
contig010	maker	exon	410534	411715	.	+	.	ID=MALK_02877.t1.e1;Parent=MALK_02877.t1
contig010	maker	gene	411931	412914	.	+	.	ID=MALK_02878;prediction_source=maker_MRET:augustus_masked-contig010-processed-gene-4.5-mRNA-1
contig010	maker	CDS	411931	412914	.	+	0	ID=MALK_02878.t1.c1;Parent=MALK_02878.t1
contig010	maker	mRNA	411931	412914	.	+	.	ID=MALK_02878.t1;Parent=MALK_02878
contig010	maker	exon	411931	412914	.	+	.	ID=MALK_02878.t1.e1;Parent=MALK_02878.t1
contig010	AUGUSTUS	gene	413490	415454	0.42	+	.	ID=MALK_02879;prediction_source=augustus:contig010.g1168.t1
contig010	AUGUSTUS	CDS	413490	415454	0.42	+	0	ID=MALK_02879.t1.c1;Parent=MALK_02879.t1
contig010	AUGUSTUS	mRNA	413490	415454	0.42	+	.	ID=MALK_02879.t1;Parent=MALK_02879
contig010	AUGUSTUS	exon	413490	415454	0.42	+	.	ID=MALK_02879.t1.e1;Parent=MALK_02879.t1
contig010	maker	gene	415663	416469	.	-	.	ID=MALK_02880;prediction_source=maker_MRET:augustus_masked-contig010-processed-gene-4.26-mRNA-1
contig010	maker	CDS	415663	416469	.	-	0	ID=MALK_02880.t1.c1;Parent=MALK_02880.t1
contig010	maker	mRNA	415663	416469	.	-	.	ID=MALK_02880.t1;Parent=MALK_02880
contig010	maker	exon	415663	416469	.	-	.	ID=MALK_02880.t1.e1;Parent=MALK_02880.t1
contig010	AUGUSTUS	gene	416810	417655	0.94	+	.	ID=MALK_02881;prediction_source=braker_MRET:g1436.t1
contig010	AUGUSTUS	CDS	416810	416838	1	+	0	ID=MALK_02881.t1.c1;Parent=MALK_02881.t1
contig010	AUGUSTUS	CDS	416877	417460	1	+	0	ID=MALK_02881.t1.c2;Parent=MALK_02881.t1
contig010	AUGUSTUS	CDS	417489	417655	1	+	0	ID=MALK_02881.t1.c3;Parent=MALK_02881.t1

contig010	AUGUSTUS	mRNA	416810	417655	0.94	+	.	ID=MALK_02881.t1;Parent=MALK_02881
contig010	AUGUSTUS	exon	416810	416838	.	+	.	ID=MALK_02881.t1.e1;Parent=MALK_02881.t1
contig010	AUGUSTUS	exon	416877	417460	.	+	.	ID=MALK_02881.t1.e2;Parent=MALK_02881.t1
contig010	AUGUSTUS	exon	417489	417655	.	+	.	ID=MALK_02881.t1.e3;Parent=MALK_02881.t1
contig010	maker	gene	418671	420497	.	-	.	ID=MALK_02882;prediction_source=maker_MRET:augustus_masked-contig010-processed-gene-4.27-mRNA-1
contig010	maker	CDS	418671	420497	.	-	0	ID=MALK_02882.t1.c1;Parent=MALK_02882.t1
contig010	maker	mRNA	418671	420497	.	-	.	ID=MALK_02882.t1;Parent=MALK_02882
contig010	maker	exon	418671	420497	.	-	.	ID=MALK_02882.t1.e1;Parent=MALK_02882.t1
contig010	maker	gene	420608	422155	.	-	.	ID=MALK_02883;prediction_source=maker_MRET:augustus_masked-contig010-processed-gene-4.28-mRNA-1
contig010	maker	CDS	420608	422155	.	-	0	ID=MALK_02883.t1.c1;Parent=MALK_02883.t1
contig010	maker	mRNA	420608	422155	.	-	.	ID=MALK_02883.t1;Parent=MALK_02883
contig010	maker	exon	420608	422155	.	-	.	ID=MALK_02883.t1.e1;Parent=MALK_02883.t1
contig010	AUGUSTUS	gene	422444	424744	0.62	+	.	ID=MALK_02884;prediction_source=braker_MRET:g1439.t1
contig010	AUGUSTUS	CDS	422444	424744	0.62	+	0	ID=MALK_02884.t1.c1;Parent=MALK_02884.t1
contig010	AUGUSTUS	mRNA	422444	424744	0.62	+	.	ID=MALK_02884.t1;Parent=MALK_02884
contig010	AUGUSTUS	exon	422444	424744	.	+	.	ID=MALK_02884.t1.e1;Parent=MALK_02884.t1
contig010	maker	gene	424701	425747	.	-	.	ID=MALK_02885;prediction_source=maker_MRET:augustus_masked-contig010-processed-gene-4.29-mRNA-1
contig010	maker	CDS	424701	425747	.	-	0	ID=MALK_02885.t1.c1;Parent=MALK_02885.t1
contig010	maker	mRNA	424701	425747	.	-	.	ID=MALK_02885.t1;Parent=MALK_02885
contig010	maker	exon	424701	425747	.	-	.	ID=MALK_02885.t1.e1;Parent=MALK_02885.t1
contig010	maker	gene	425798	427441	.	-	.	ID=MALK_02886;prediction_source=maker_MRET:augustus_masked-contig010-processed-gene-4.30-mRNA-1
contig010	maker	CDS	425798	427441	.	-	0	ID=MALK_02886.t1.c1;Parent=MALK_02886.t1
contig010	maker	mRNA	425798	427441	.	-	.	ID=MALK_02886.t1;Parent=MALK_02886
contig010	maker	exon	425798	427441	.	-	.	ID=MALK_02886.t1.e1;Parent=MALK_02886.t1
contig010	AUGUSTUS	gene	427668	429092	0.97	+	.	ID=MALK_02887;prediction_source=augustus:contig010.g1179.t1
contig010	AUGUSTUS	CDS	427668	429092	0.97	+	0	ID=MALK_02887.t1.c1;Parent=MALK_02887.t1
contig010	AUGUSTUS	mRNA	427668	429092	0.97	+	.	ID=MALK_02887.t1;Parent=MALK_02887
contig010	AUGUSTUS	exon	427668	429092	0.97	+	.	ID=MALK_02887.t1.e1;Parent=MALK_02887.t1
contig010	maker	gene	429229	433722	.	+	.	ID=MALK_02888;prediction_source=maker_MRET:augustus_masked-contig010-processed-gene-4.10-mRNA-1
contig010	maker	CDS	429229	433722	.	+	0	ID=MALK_02888.t1.c1;Parent=MALK_02888.t1
contig010	maker	mRNA	429229	433722	.	+	.	ID=MALK_02888.t1;Parent=MALK_02888
contig010	maker	exon	429229	433722	.	+	.	ID=MALK_02888.t1.e1;Parent=MALK_02888.t1
contig010	AUGUSTUS	gene	434087	436012	1	+	.	ID=MALK_02889;prediction_source=augustus:contig010.g1181.t1
contig010	AUGUSTUS	CDS	434087	436012	1	+	0	ID=MALK_02889.t1.c1;Parent=MALK_02889.t1
contig010	AUGUSTUS	mRNA	434087	436012	1	+	.	ID=MALK_02889.t1;Parent=MALK_02889
contig010	AUGUSTUS	exon	434087	436012	1	+	.	ID=MALK_02889.t1.e1;Parent=MALK_02889.t1
contig010	AUGUSTUS	gene	436061	438279	0.23	-	.	ID=MALK_02890;prediction_source=braker_MRET:g1445.t1
contig010	AUGUSTUS	CDS	438080	438279	0.25	-	0	ID=MALK_02890.t1.c3;Parent=MALK_02890.t1
contig010	AUGUSTUS	CDS	437997	438048	0.25	-	0	ID=MALK_02890.t1.c2;Parent=MALK_02890.t1
contig010	AUGUSTUS	CDS	436061	437950	0.25	-	0	ID=MALK_02890.t1.c1;Parent=MALK_02890.t1
contig010	AUGUSTUS	mRNA	436061	438279	0.23	-	.	ID=MALK_02890.t1;Parent=MALK_02890
contig010	AUGUSTUS	exon	438080	438279	.	-	.	ID=MALK_02890.t1.e3;Parent=MALK_02890.t1
contig010	AUGUSTUS	exon	437997	438048	.	-	.	ID=MALK_02890.t1.e2;Parent=MALK_02890.t1

contig010	AUGUSTUS	exon	436061	437950	.	-	.	ID=MALK_02890.t1.e1;Parent=MALK_02890.t1
contig010	maker	gene	438529	439758	.	-	.	ID=MALK_02891;prediction_source=maker_MRET:augustus_masked-contig010-processed-gene-4.33-mRNA-1
contig010	maker	CDS	438529	439758	.	-	0	ID=MALK_02891.t1.c1;Parent=MALK_02891.t1
contig010	maker	mRNA	438529	439758	.	-	.	ID=MALK_02891.t1;Parent=MALK_02891
contig010	maker	exon	438529	439758	.	-	.	ID=MALK_02891.t1.e1;Parent=MALK_02891.t1
contig010	AUGUSTUS	gene	440238	441140	0.59	+	.	ID=MALK_02892;prediction_source=augustus:contig010.g1186.t1
contig010	AUGUSTUS	CDS	440238	441140	0.59	+	0	ID=MALK_02892.t1.c1;Parent=MALK_02892.t1
contig010	AUGUSTUS	mRNA	440238	441140	0.59	+	.	ID=MALK_02892.t1;Parent=MALK_02892
contig010	AUGUSTUS	exon	440238	441140	0.59	+	.	ID=MALK_02892.t1.e1;Parent=MALK_02892.t1
contig010	AUGUSTUS	gene	441167	442603	0.56	-	.	ID=MALK_02893;prediction_source=augustus:contig010.g1187.t1
contig010	AUGUSTUS	CDS	441167	442603	0.56	-	0	ID=MALK_02893.t1.c1;Parent=MALK_02893.t1
contig010	AUGUSTUS	mRNA	441167	442603	0.56	-	.	ID=MALK_02893.t1;Parent=MALK_02893
contig010	AUGUSTUS	exon	441167	442603	0.56	-	.	ID=MALK_02893.t1.e1;Parent=MALK_02893.t1
contig010	maker	gene	442706	447058	.	+	.	ID=MALK_02894;prediction_source=maker_MRET:augustus_masked-contig010-processed-gene-4.13-mRNA-1
contig010	maker	CDS	442706	447058	.	+	0	ID=MALK_02894.t1.c1;Parent=MALK_02894.t1
contig010	maker	mRNA	442706	447058	.	+	.	ID=MALK_02894.t1;Parent=MALK_02894
contig010	maker	exon	442706	447058	.	+	.	ID=MALK_02894.t1.e1;Parent=MALK_02894.t1
contig010	maker	gene	447347	448867	.	+	.	ID=MALK_02895;prediction_source=maker_MRET:augustus_masked-contig010-processed-gene-4.14-mRNA-1
contig010	maker	CDS	447347	448867	.	+	0	ID=MALK_02895.t1.c1;Parent=MALK_02895.t1
contig010	maker	mRNA	447347	448867	.	+	.	ID=MALK_02895.t1;Parent=MALK_02895
contig010	maker	exon	447347	448867	.	+	.	ID=MALK_02895.t1.e1;Parent=MALK_02895.t1
contig010	maker	gene	448890	449291	.	+	.	ID=MALK_02896;prediction_source=maker_MRET:augustus_masked-contig010-processed-gene-4.15-mRNA-1
contig010	maker	CDS	448890	449291	.	+	0	ID=MALK_02896.t1.c1;Parent=MALK_02896.t1
contig010	maker	mRNA	448890	449291	.	+	.	ID=MALK_02896.t1;Parent=MALK_02896
contig010	maker	exon	448890	449291	.	+	.	ID=MALK_02896.t1.e1;Parent=MALK_02896.t1
contig010	AUGUSTUS	gene	449328	451733	0.65	-	.	ID=MALK_02897;prediction_source=braker_MRET:g1452.t1
contig010	AUGUSTUS	CDS	451533	451733	0.66	-	0	ID=MALK_02897.t1.c2;Parent=MALK_02897.t1
contig010	AUGUSTUS	CDS	449328	451316	0.66	-	0	ID=MALK_02897.t1.c1;Parent=MALK_02897.t1
contig010	AUGUSTUS	mRNA	449328	451733	0.65	-	.	ID=MALK_02897.t1;Parent=MALK_02897
contig010	AUGUSTUS	exon	451533	451733	.	-	.	ID=MALK_02897.t1.e2;Parent=MALK_02897.t1
contig010	AUGUSTUS	exon	449328	451316	.	-	.	ID=MALK_02897.t1.e1;Parent=MALK_02897.t1
contig010	AUGUSTUS	gene	452227	453995	0.39	-	.	ID=MALK_02898;prediction_source=braker_MRET:g1453.t1
contig010	AUGUSTUS	CDS	453972	453995	0.82	-	0	ID=MALK_02898.t1.c9;Parent=MALK_02898.t1
contig010	AUGUSTUS	CDS	453881	453899	0.82	-	0	ID=MALK_02898.t1.c8;Parent=MALK_02898.t1
contig010	AUGUSTUS	CDS	453770	453828	0.82	-	0	ID=MALK_02898.t1.c7;Parent=MALK_02898.t1
contig010	AUGUSTUS	CDS	453675	453735	0.82	-	0	ID=MALK_02898.t1.c6;Parent=MALK_02898.t1
contig010	AUGUSTUS	CDS	453580	453645	0.82	-	0	ID=MALK_02898.t1.c5;Parent=MALK_02898.t1
contig010	AUGUSTUS	CDS	453210	453548	0.82	-	0	ID=MALK_02898.t1.c4;Parent=MALK_02898.t1
contig010	AUGUSTUS	CDS	452793	452911	0.82	-	0	ID=MALK_02898.t1.c3;Parent=MALK_02898.t1
contig010	AUGUSTUS	CDS	452324	452535	0.82	-	0	ID=MALK_02898.t1.c2;Parent=MALK_02898.t1
contig010	AUGUSTUS	CDS	452227	452281	0.82	-	0	ID=MALK_02898.t1.c1;Parent=MALK_02898.t1
contig010	AUGUSTUS	mRNA	452227	453995	0.39	-	.	ID=MALK_02898.t1;Parent=MALK_02898
contig010	AUGUSTUS	exon	453972	453995	.	-	.	ID=MALK_02898.t1.e9;Parent=MALK_02898.t1

contig010	AUGUSTUS	exon	453881	453899	.	-	.	ID=MALK_02898.t1.e8;Parent=MALK_02898.t1
contig010	AUGUSTUS	exon	453770	453828	.	-	.	ID=MALK_02898.t1.e7;Parent=MALK_02898.t1
contig010	AUGUSTUS	exon	453675	453735	.	-	.	ID=MALK_02898.t1.e6;Parent=MALK_02898.t1
contig010	AUGUSTUS	exon	453580	453645	.	-	.	ID=MALK_02898.t1.e5;Parent=MALK_02898.t1
contig010	AUGUSTUS	exon	453210	453548	.	-	.	ID=MALK_02898.t1.e4;Parent=MALK_02898.t1
contig010	AUGUSTUS	exon	452793	452911	.	-	.	ID=MALK_02898.t1.e3;Parent=MALK_02898.t1
contig010	AUGUSTUS	exon	452324	452535	.	-	.	ID=MALK_02898.t1.e2;Parent=MALK_02898.t1
contig010	AUGUSTUS	exon	452227	452281	.	-	.	ID=MALK_02898.t1.e1;Parent=MALK_02898.t1
contig010	AUGUSTUS	gene	454165	455472	0.19	-	.	ID=MALK_02899;prediction_source=braker_MRET:g1454.t1
contig010	AUGUSTUS	CDS	455451	455472	1	-	0	ID=MALK_02899.t1.c5;Parent=MALK_02899.t1
contig010	AUGUSTUS	CDS	455375	455403	1	-	0	ID=MALK_02899.t1.c4;Parent=MALK_02899.t1
contig010	AUGUSTUS	CDS	455168	455338	1	-	0	ID=MALK_02899.t1.c3;Parent=MALK_02899.t1
contig010	AUGUSTUS	CDS	455051	455138	1	-	0	ID=MALK_02899.t1.c2;Parent=MALK_02899.t1
contig010	AUGUSTUS	CDS	454165	455018	1	-	0	ID=MALK_02899.t1.c1;Parent=MALK_02899.t1
contig010	AUGUSTUS	mRNA	454165	455472	0.19	-	.	ID=MALK_02899.t1;Parent=MALK_02899
contig010	AUGUSTUS	exon	455451	455472	.	-	.	ID=MALK_02899.t1.e5;Parent=MALK_02899.t1
contig010	AUGUSTUS	exon	455375	455403	.	-	.	ID=MALK_02899.t1.e4;Parent=MALK_02899.t1
contig010	AUGUSTUS	exon	455168	455338	.	-	.	ID=MALK_02899.t1.e3;Parent=MALK_02899.t1
contig010	AUGUSTUS	exon	455051	455138	.	-	.	ID=MALK_02899.t1.e2;Parent=MALK_02899.t1
contig010	AUGUSTUS	exon	454165	455018	.	-	.	ID=MALK_02899.t1.e1;Parent=MALK_02899.t1
contig010	maker	gene	455724	456788	.	+	.	ID=MALK_02900;prediction_source=maker_MRET:augustus_masked-contig010-processed-gene-4.16-mRNA-1
contig010	maker	CDS	455724	456788	.	+	0	ID=MALK_02900.t1.c1;Parent=MALK_02900.t1
contig010	maker	mRNA	455724	456788	.	+	.	ID=MALK_02900.t1;Parent=MALK_02900
contig010	maker	exon	455724	456788	.	+	.	ID=MALK_02900.t1.e1;Parent=MALK_02900.t1
contig010	AUGUSTUS	gene	457768	458157	1	-	.	ID=MALK_02901;prediction_source=braker_MRET:g1456.t1
contig010	AUGUSTUS	CDS	457980	458157	1	-	0	ID=MALK_02901.t1.c2;Parent=MALK_02901.t1
contig010	AUGUSTUS	CDS	457768	457949	1	-	0	ID=MALK_02901.t1.c1;Parent=MALK_02901.t1
contig010	AUGUSTUS	mRNA	457768	458157	1	-	.	ID=MALK_02901.t1;Parent=MALK_02901
contig010	AUGUSTUS	exon	457980	458157	.	-	.	ID=MALK_02901.t1.e2;Parent=MALK_02901.t1
contig010	AUGUSTUS	exon	457768	457949	.	-	.	ID=MALK_02901.t1.e1;Parent=MALK_02901.t1
contig010	AUGUSTUS	gene	458304	458828	1	+	.	ID=MALK_02902;prediction_source=braker_MRET:g1457.t1
contig010	AUGUSTUS	CDS	458304	458828	1	+	0	ID=MALK_02902.t1.c1;Parent=MALK_02902.t1
contig010	AUGUSTUS	mRNA	458304	458828	1	+	.	ID=MALK_02902.t1;Parent=MALK_02902
contig010	AUGUSTUS	exon	458304	458828	.	+	.	ID=MALK_02902.t1.e1;Parent=MALK_02902.t1
contig010	AUGUSTUS	gene	458858	460120	0.77	-	.	ID=MALK_02903;prediction_source=augustus:contig010.g1197.t1
contig010	AUGUSTUS	CDS	458858	460120	0.77	-	0	ID=MALK_02903.t1.c1;Parent=MALK_02903.t1
contig010	AUGUSTUS	mRNA	458858	460120	0.77	-	.	ID=MALK_02903.t1;Parent=MALK_02903
contig010	AUGUSTUS	exon	458858	460120	0.77	-	.	ID=MALK_02903.t1.e1;Parent=MALK_02903.t1
contig010	AUGUSTUS	gene	460237	461817	1	+	.	ID=MALK_02904;prediction_source=braker_MRET:g1459.t1
contig010	AUGUSTUS	CDS	460237	461817	1	+	0	ID=MALK_02904.t1.c1;Parent=MALK_02904.t1
contig010	AUGUSTUS	mRNA	460237	461817	1	+	.	ID=MALK_02904.t1;Parent=MALK_02904
contig010	AUGUSTUS	exon	460237	461817	.	+	.	ID=MALK_02904.t1.e1;Parent=MALK_02904.t1
contig010	AUGUSTUS	gene	461819	463348	0.62	-	.	ID=MALK_02905;prediction_source=augustus:contig010.g1198.t1

contig010	AUGUSTUS	CDS	461819	463348	0.62	-	0	ID=MALK_02905.t1.c1;Parent=MALK_02905.t1
contig010	AUGUSTUS	mRNA	461819	463348	0.62	-	.	ID=MALK_02905.t1;Parent=MALK_02905
contig010	AUGUSTUS	exon	461819	463348	0.62	-	.	ID=MALK_02905.t1.e1;Parent=MALK_02905.t1
contig010	AUGUSTUS	gene	463792	466179	0.72	+	.	ID=MALK_02906;prediction_source=augustus:contig010.g1200.t1
contig010	AUGUSTUS	CDS	463792	466179	0.72	+	0	ID=MALK_02906.t1.c1;Parent=MALK_02906.t1
contig010	AUGUSTUS	mRNA	463792	466179	0.72	+	.	ID=MALK_02906.t1;Parent=MALK_02906
contig010	AUGUSTUS	exon	463792	466179	0.72	+	.	ID=MALK_02906.t1.e1;Parent=MALK_02906.t1
contig010	maker	gene	466753	468105	.	-	.	ID=MALK_02907;prediction_source=maker_MRET:augustus_masked-contig010-processed-gene-4.40-mRNA-1
contig010	maker	CDS	466954	468105	.	-	0	ID=MALK_02907.t1.c1;Parent=MALK_02907.t1
contig010	maker	CDS	466753	466887	.	-	0	ID=MALK_02907.t1.c2;Parent=MALK_02907.t1
contig010	maker	mRNA	466753	468105	.	-	.	ID=MALK_02907.t1;Parent=MALK_02907
contig010	maker	exon	466954	468105	.	-	.	ID=MALK_02907.t1.e1;Parent=MALK_02907.t1
contig010	maker	exon	466753	466887	.	-	.	ID=MALK_02907.t1.e2;Parent=MALK_02907.t1
contig010	AUGUSTUS	gene	468342	468989	0.26	-	.	ID=MALK_02908;prediction_source=braker_MRET:g1463.t1
contig010	AUGUSTUS	CDS	468685	468989	0.35	-	0	ID=MALK_02908.t1.c3;Parent=MALK_02908.t1
contig010	AUGUSTUS	CDS	468462	468527	0.35	-	0	ID=MALK_02908.t1.c2;Parent=MALK_02908.t1
contig010	AUGUSTUS	CDS	468342	468429	0.35	-	0	ID=MALK_02908.t1.c1;Parent=MALK_02908.t1
contig010	AUGUSTUS	mRNA	468342	468989	0.26	-	.	ID=MALK_02908.t1;Parent=MALK_02908
contig010	AUGUSTUS	exon	468685	468989	.	-	.	ID=MALK_02908.t1.e3;Parent=MALK_02908.t1
contig010	AUGUSTUS	exon	468462	468527	.	-	.	ID=MALK_02908.t1.e2;Parent=MALK_02908.t1
contig010	AUGUSTUS	exon	468342	468429	.	-	.	ID=MALK_02908.t1.e1;Parent=MALK_02908.t1
contig010	AUGUSTUS	gene	469056	471122	0.69	-	.	ID=MALK_02909;prediction_source=augustus:contig010.g1202.t1
contig010	AUGUSTUS	CDS	469056	471122	0.69	-	0	ID=MALK_02909.t1.c1;Parent=MALK_02909.t1
contig010	AUGUSTUS	mRNA	469056	471122	0.69	-	.	ID=MALK_02909.t1;Parent=MALK_02909
contig010	AUGUSTUS	exon	469056	471122	0.69	-	.	ID=MALK_02909.t1.e1;Parent=MALK_02909.t1
contig010	AUGUSTUS	gene	471293	472389	0.57	+	.	ID=MALK_02910;prediction_source=braker_MRET:g1465.t1
contig010	AUGUSTUS	CDS	471293	471508	0.92	+	0	ID=MALK_02910.t1.c1;Parent=MALK_02910.t1
contig010	AUGUSTUS	CDS	471574	472389	0.92	+	0	ID=MALK_02910.t1.c2;Parent=MALK_02910.t1
contig010	AUGUSTUS	mRNA	471293	472389	0.57	+	.	ID=MALK_02910.t1;Parent=MALK_02910
contig010	AUGUSTUS	exon	471293	471508	.	+	.	ID=MALK_02910.t1.e1;Parent=MALK_02910.t1
contig010	AUGUSTUS	exon	471574	472389	.	+	.	ID=MALK_02910.t1.e2;Parent=MALK_02910.t1
contig010	AUGUSTUS	gene	473189	473764	0.6	+	.	ID=MALK_02911;prediction_source=augustus:contig010.g1204.t1
contig010	AUGUSTUS	CDS	473189	473764	0.6	+	0	ID=MALK_02911.t1.c1;Parent=MALK_02911.t1
contig010	AUGUSTUS	mRNA	473189	473764	0.6	+	.	ID=MALK_02911.t1;Parent=MALK_02911
contig010	AUGUSTUS	exon	473189	473764	0.6	+	.	ID=MALK_02911.t1.e1;Parent=MALK_02911.t1
contig010	AUGUSTUS	gene	473828	475945	0.28	+	.	ID=MALK_02912;prediction_source=braker_MRET:g1467.t1
contig010	AUGUSTUS	CDS	473828	475945	0.28	+	0	ID=MALK_02912.t1.c1;Parent=MALK_02912.t1
contig010	AUGUSTUS	mRNA	473828	475945	0.28	+	.	ID=MALK_02912.t1;Parent=MALK_02912
contig010	AUGUSTUS	exon	473828	475945	.	+	.	ID=MALK_02912.t1.e1;Parent=MALK_02912.t1
contig010	AUGUSTUS	gene	475949	477229	0.62	-	.	ID=MALK_02913;prediction_source=augustus:contig010.g1207.t1
contig010	AUGUSTUS	CDS	475949	477229	0.62	-	0	ID=MALK_02913.t1.c1;Parent=MALK_02913.t1
contig010	AUGUSTUS	mRNA	475949	477229	0.62	-	.	ID=MALK_02913.t1;Parent=MALK_02913
contig010	AUGUSTUS	exon	475949	477229	0.62	-	.	ID=MALK_02913.t1.e1;Parent=MALK_02913.t1

contig010	AUGUSTUS	gene	477704	478121	0.47	+	.	ID=MALK_02914;prediction_source=braker_MRET:g1469.t1
contig010	AUGUSTUS	CDS	477704	477761	0.99	+	0	ID=MALK_02914.t1.c1;Parent=MALK_02914.t1
contig010	AUGUSTUS	CDS	477798	478040	0.99	+	0	ID=MALK_02914.t1.c2;Parent=MALK_02914.t1
contig010	AUGUSTUS	CDS	478069	478121	0.99	+	0	ID=MALK_02914.t1.c3;Parent=MALK_02914.t1
contig010	AUGUSTUS	mRNA	477704	478121	0.47	+	.	ID=MALK_02914.t1;Parent=MALK_02914
contig010	AUGUSTUS	exon	477704	477761	.	+	.	ID=MALK_02914.t1.e1;Parent=MALK_02914.t1
contig010	AUGUSTUS	exon	477798	478040	.	+	.	ID=MALK_02914.t1.e2;Parent=MALK_02914.t1
contig010	AUGUSTUS	exon	478069	478121	.	+	.	ID=MALK_02914.t1.e3;Parent=MALK_02914.t1
contig010	AUGUSTUS	gene	478193	479110	0.82	-	.	ID=MALK_02915;prediction_source=augustus:contig010.g1209.t1
contig010	AUGUSTUS	CDS	478193	479110	0.82	-	0	ID=MALK_02915.t1.c1;Parent=MALK_02915.t1
contig010	AUGUSTUS	mRNA	478193	479110	0.82	-	.	ID=MALK_02915.t1;Parent=MALK_02915
contig010	AUGUSTUS	exon	478193	479110	0.82	-	.	ID=MALK_02915.t1.e1;Parent=MALK_02915.t1
contig010	AUGUSTUS	gene	479325	479864	0.67	+	.	ID=MALK_02916;prediction_source=braker_MRET:g1471.t1
contig010	AUGUSTUS	CDS	479325	479734	0.92	+	0	ID=MALK_02916.t1.c1;Parent=MALK_02916.t1
contig010	AUGUSTUS	CDS	479762	479864	0.92	+	0	ID=MALK_02916.t1.c2;Parent=MALK_02916.t1
contig010	AUGUSTUS	mRNA	479325	479864	0.67	+	.	ID=MALK_02916.t1;Parent=MALK_02916
contig010	AUGUSTUS	exon	479325	479734	.	+	.	ID=MALK_02916.t1.e1;Parent=MALK_02916.t1
contig010	AUGUSTUS	exon	479762	479864	.	+	.	ID=MALK_02916.t1.e2;Parent=MALK_02916.t1
contig010	maker	gene	479899	481652	.	-	.	ID=MALK_02917;prediction_source=maker_MRET:augustus_masked-contig010-processed-gene-4.44-mRNA-1
contig010	maker	CDS	481476	481652	.	-	0	ID=MALK_02917.t1.c1;Parent=MALK_02917.t1
contig010	maker	CDS	479899	481371	.	-	0	ID=MALK_02917.t1.c2;Parent=MALK_02917.t1
contig010	maker	mRNA	479899	481652	.	-	.	ID=MALK_02917.t1;Parent=MALK_02917
contig010	maker	exon	481476	481652	.	-	.	ID=MALK_02917.t1.e1;Parent=MALK_02917.t1
contig010	maker	exon	479899	481371	.	-	.	ID=MALK_02917.t1.e2;Parent=MALK_02917.t1
contig010	maker	gene	482082	483011	.	+	.	ID=MALK_02918;prediction_source=maker_MRET:augustus_masked-contig010-processed-gene-4.23-mRNA-1
contig010	maker	CDS	482082	483011	.	+	0	ID=MALK_02918.t1.c1;Parent=MALK_02918.t1
contig010	maker	mRNA	482082	483011	.	+	.	ID=MALK_02918.t1;Parent=MALK_02918
contig010	maker	exon	482082	483011	.	+	.	ID=MALK_02918.t1.e1;Parent=MALK_02918.t1
contig010	AUGUSTUS	gene	483431	484705	0.71	+	.	ID=MALK_02919;prediction_source=augustus:contig010.g1213.t1
contig010	AUGUSTUS	CDS	483431	484705	0.71	+	0	ID=MALK_02919.t1.c1;Parent=MALK_02919.t1
contig010	AUGUSTUS	mRNA	483431	484705	0.71	+	.	ID=MALK_02919.t1;Parent=MALK_02919
contig010	AUGUSTUS	exon	483431	484705	0.71	+	.	ID=MALK_02919.t1.e1;Parent=MALK_02919.t1
contig010	maker	gene	484706	493534	.	-	.	ID=MALK_02920;prediction_source=maker_MRET:augustus_masked-contig010-processed-gene-4.45-mRNA-1
contig010	maker	CDS	484706	493534	.	-	0	ID=MALK_02920.t1.c1;Parent=MALK_02920.t1
contig010	maker	mRNA	484706	493534	.	-	.	ID=MALK_02920.t1;Parent=MALK_02920
contig010	maker	exon	484706	493534	.	-	.	ID=MALK_02920.t1.e1;Parent=MALK_02920.t1
contig010	maker	gene	493643	494626	.	-	.	ID=MALK_02921;prediction_source=maker_MRET:augustus_masked-contig010-processed-gene-4.46-mRNA-1
contig010	maker	CDS	493643	494626	.	-	0	ID=MALK_02921.t1.c1;Parent=MALK_02921.t1
contig010	maker	mRNA	493643	494626	.	-	.	ID=MALK_02921.t1;Parent=MALK_02921
contig010	maker	exon	493643	494626	.	-	.	ID=MALK_02921.t1.e1;Parent=MALK_02921.t1
contig010	maker	gene	495021	495797	.	+	.	ID=MALK_02922;prediction_source=maker_MRET:augustus_masked-contig010-processed-gene-5.0-mRNA-1
contig010	maker	CDS	495021	495797	.	+	0	ID=MALK_02922.t1.c1;Parent=MALK_02922.t1
contig010	maker	mRNA	495021	495797	.	+	.	ID=MALK_02922.t1;Parent=MALK_02922

contig010	maker	exon	495021	495797	.	+	.	ID=MALK_02922.t1.e1;Parent=MALK_02922.t1
contig010	AUGUSTUS	gene	495918	500378	0.89	+	.	ID=MALK_02923;prediction_source=augustus:contig010.g1218.t1
contig010	AUGUSTUS	CDS	495918	500378	0.89	+	0	ID=MALK_02923.t1.c1;Parent=MALK_02923.t1
contig010	AUGUSTUS	mRNA	495918	500378	0.89	+	.	ID=MALK_02923.t1;Parent=MALK_02923
contig010	AUGUSTUS	exon	495918	500378	0.89	+	.	ID=MALK_02923.t1.e1;Parent=MALK_02923.t1
contig010	maker	gene	501314	502984	.	-	.	ID=MALK_02924;prediction_source=maker_MRET:augustus_masked-contig010-processed-gene-5.21-mRNA-1
contig010	maker	CDS	502869	502984	.	-	0	ID=MALK_02924.t1.c1;Parent=MALK_02924.t1
contig010	maker	CDS	501314	502754	.	-	0	ID=MALK_02924.t1.c2;Parent=MALK_02924.t1
contig010	maker	mRNA	501314	502984	.	-	.	ID=MALK_02924.t1;Parent=MALK_02924
contig010	maker	exon	502869	502984	.	-	.	ID=MALK_02924.t1.e1;Parent=MALK_02924.t1
contig010	maker	exon	501314	502754	.	-	.	ID=MALK_02924.t1.e2;Parent=MALK_02924.t1
contig010	maker	gene	503340	513341	.	-	.	ID=MALK_02925;prediction_source=maker_MRET:augustus_masked-contig010-processed-gene-5.22-mRNA-1
contig010	maker	CDS	513071	513341	.	-	0	ID=MALK_02925.t1.c1;Parent=MALK_02925.t1
contig010	maker	CDS	503340	512644	.	-	0	ID=MALK_02925.t1.c2;Parent=MALK_02925.t1
contig010	maker	mRNA	503340	513341	.	-	.	ID=MALK_02925.t1;Parent=MALK_02925
contig010	maker	exon	513071	513341	.	-	.	ID=MALK_02925.t1.e1;Parent=MALK_02925.t1
contig010	maker	exon	503340	512644	.	-	.	ID=MALK_02925.t1.e2;Parent=MALK_02925.t1
contig010	maker	gene	513656	517916	.	+	.	ID=MALK_02926;prediction_source=maker_MRET:augustus_masked-contig010-processed-gene-5.2-mRNA-1
contig010	maker	CDS	513656	515396	.	+	0	ID=MALK_02926.t1.c1;Parent=MALK_02926.t1
contig010	maker	CDS	515776	517916	.	+	0	ID=MALK_02926.t1.c2;Parent=MALK_02926.t1
contig010	maker	mRNA	513656	517916	.	+	.	ID=MALK_02926.t1;Parent=MALK_02926
contig010	maker	exon	513656	515396	.	+	.	ID=MALK_02926.t1.e1;Parent=MALK_02926.t1
contig010	maker	exon	515776	517916	.	+	.	ID=MALK_02926.t1.e2;Parent=MALK_02926.t1
contig010	AUGUSTUS	gene	518567	526783	0.99	+	.	ID=MALK_02927;prediction_source=augustus:contig010.g1227.t1
contig010	AUGUSTUS	CDS	518567	526783	0.99	+	0	ID=MALK_02927.t1.c1;Parent=MALK_02927.t1
contig010	AUGUSTUS	mRNA	518567	526783	0.99	+	.	ID=MALK_02927.t1;Parent=MALK_02927
contig010	AUGUSTUS	exon	518567	526783	0.99	+	.	ID=MALK_02927.t1.e1;Parent=MALK_02927.t1
contig010	AUGUSTUS	gene	527207	532804	0.98	+	.	ID=MALK_02928;prediction_source=augustus:contig010.g1228.t1
contig010	AUGUSTUS	CDS	527207	532804	0.98	+	0	ID=MALK_02928.t1.c1;Parent=MALK_02928.t1
contig010	AUGUSTUS	mRNA	527207	532804	0.98	+	.	ID=MALK_02928.t1;Parent=MALK_02928
contig010	AUGUSTUS	exon	527207	532804	0.98	+	.	ID=MALK_02928.t1.e1;Parent=MALK_02928.t1
contig010	AUGUSTUS	gene	532776	533987	0.99	-	.	ID=MALK_02929;prediction_source=augustus:contig010.g1231.t1
contig010	AUGUSTUS	CDS	532776	533987	0.99	-	0	ID=MALK_02929.t1.c1;Parent=MALK_02929.t1
contig010	AUGUSTUS	mRNA	532776	533987	0.99	-	.	ID=MALK_02929.t1;Parent=MALK_02929
contig010	AUGUSTUS	exon	532776	533987	0.99	-	.	ID=MALK_02929.t1.e1;Parent=MALK_02929.t1
contig010	AUGUSTUS	gene	534079	534819	0.7	+	.	ID=MALK_02930;prediction_source=augustus:contig010.g1232.t1
contig010	AUGUSTUS	CDS	534079	534819	0.7	+	0	ID=MALK_02930.t1.c1;Parent=MALK_02930.t1
contig010	AUGUSTUS	mRNA	534079	534819	0.7	+	.	ID=MALK_02930.t1;Parent=MALK_02930
contig010	AUGUSTUS	exon	534079	534819	0.7	+	.	ID=MALK_02930.t1.e1;Parent=MALK_02930.t1
contig010	AUGUSTUS	gene	534834	536072	0.99	-	.	ID=MALK_02931;prediction_source=braker_MRET:g1485.t1
contig010	AUGUSTUS	CDS	534834	536072	0.99	-	0	ID=MALK_02931.t1.c1;Parent=MALK_02931.t1
contig010	AUGUSTUS	mRNA	534834	536072	0.99	-	.	ID=MALK_02931.t1;Parent=MALK_02931
contig010	AUGUSTUS	exon	534834	536072	.	-	.	ID=MALK_02931.t1.e1;Parent=MALK_02931.t1

contig010	maker	gene	536611	537978	.	+	.	ID=MALK_02932;prediction_source=maker_MRET:augustus_masked-contig010-processed-gene-5.6-mRNA-1
contig010	maker	CDS	536611	537978	.	+	0	ID=MALK_02932.t1.c1;Parent=MALK_02932.t1
contig010	maker	mRNA	536611	537978	.	+	.	ID=MALK_02932.t1;Parent=MALK_02932
contig010	maker	exon	536611	537978	.	+	.	ID=MALK_02932.t1.e1;Parent=MALK_02932.t1
contig010	AUGUSTUS	gene	538462	539616	0.81	+	.	ID=MALK_02933;prediction_source=augustus:contig010.g1236.t1
contig010	AUGUSTUS	CDS	538462	539616	0.81	+	0	ID=MALK_02933.t1.c1;Parent=MALK_02933.t1
contig010	AUGUSTUS	mRNA	538462	539616	0.81	+	.	ID=MALK_02933.t1;Parent=MALK_02933
contig010	AUGUSTUS	exon	538462	539616	0.81	+	.	ID=MALK_02933.t1.e1;Parent=MALK_02933.t1
contig010	maker	gene	539696	540082	.	-	.	ID=MALK_02934;prediction_source=maker_MRET:augustus_masked-contig010-processed-gene-5.25-mRNA-1
contig010	maker	CDS	539696	540082	.	-	0	ID=MALK_02934.t1.c1;Parent=MALK_02934.t1
contig010	maker	mRNA	539696	540082	.	-	.	ID=MALK_02934.t1;Parent=MALK_02934
contig010	maker	exon	539696	540082	.	-	.	ID=MALK_02934.t1.e1;Parent=MALK_02934.t1
contig010	AUGUSTUS	gene	540245	542848	0.88	+	.	ID=MALK_02935;prediction_source=braker_MRET:g1488.t1
contig010	AUGUSTUS	CDS	540245	542848	0.88	+	0	ID=MALK_02935.t1.c1;Parent=MALK_02935.t1
contig010	AUGUSTUS	mRNA	540245	542848	0.88	+	.	ID=MALK_02935.t1;Parent=MALK_02935
contig010	AUGUSTUS	exon	540245	542848	.	+	.	ID=MALK_02935.t1.e1;Parent=MALK_02935.t1
contig010	AUGUSTUS	gene	542978	544491	0.99	-	.	ID=MALK_02936;prediction_source=braker_MRET:g1489.t1
contig010	AUGUSTUS	CDS	544131	544491	1	-	0	ID=MALK_02936.t1.c2;Parent=MALK_02936.t1
contig010	AUGUSTUS	CDS	542978	544098	1	-	0	ID=MALK_02936.t1.c1;Parent=MALK_02936.t1
contig010	AUGUSTUS	mRNA	542978	544491	0.99	-	.	ID=MALK_02936.t1;Parent=MALK_02936
contig010	AUGUSTUS	exon	544131	544491	.	-	.	ID=MALK_02936.t1.e2;Parent=MALK_02936.t1
contig010	AUGUSTUS	exon	542978	544098	.	-	.	ID=MALK_02936.t1.e1;Parent=MALK_02936.t1
contig010	AUGUSTUS	gene	544900	547491	0.98	-	.	ID=MALK_02937;prediction_source=augustus:contig010.g1241.t1
contig010	AUGUSTUS	CDS	544900	547491	0.98	-	0	ID=MALK_02937.t1.c1;Parent=MALK_02937.t1
contig010	AUGUSTUS	mRNA	544900	547491	0.98	-	.	ID=MALK_02937.t1;Parent=MALK_02937
contig010	AUGUSTUS	exon	544900	547491	0.98	-	.	ID=MALK_02937.t1.e1;Parent=MALK_02937.t1
contig010	maker	gene	547780	548538	.	+	.	ID=MALK_02938;prediction_source=maker_MRET:augustus_masked-contig010-processed-gene-5.9-mRNA-1
contig010	maker	CDS	547780	548538	.	+	0	ID=MALK_02938.t1.c1;Parent=MALK_02938.t1
contig010	maker	mRNA	547780	548538	.	+	.	ID=MALK_02938.t1;Parent=MALK_02938
contig010	maker	exon	547780	548538	.	+	.	ID=MALK_02938.t1.e1;Parent=MALK_02938.t1
contig010	AUGUSTUS	gene	548630	550305	0.45	+	.	ID=MALK_02939;prediction_source=braker_MRET:g1492.t1
contig010	AUGUSTUS	CDS	548630	550160	0.49	+	0	ID=MALK_02939.t1.c1;Parent=MALK_02939.t1
contig010	AUGUSTUS	CDS	550190	550305	0.49	+	0	ID=MALK_02939.t1.c2;Parent=MALK_02939.t1
contig010	AUGUSTUS	mRNA	548630	550305	0.45	+	.	ID=MALK_02939.t1;Parent=MALK_02939
contig010	AUGUSTUS	exon	548630	550160	.	+	.	ID=MALK_02939.t1.e1;Parent=MALK_02939.t1
contig010	AUGUSTUS	exon	550190	550305	.	+	.	ID=MALK_02939.t1.e2;Parent=MALK_02939.t1
contig010	maker	gene	550393	551895	.	-	.	ID=MALK_02940;prediction_source=maker_MRET:augustus_masked-contig010-processed-gene-5.28-mRNA-1
contig010	maker	CDS	550393	551895	.	-	0	ID=MALK_02940.t1.c1;Parent=MALK_02940.t1
contig010	maker	mRNA	550393	551895	.	-	.	ID=MALK_02940.t1;Parent=MALK_02940
contig010	maker	exon	550393	551895	.	-	.	ID=MALK_02940.t1.e1;Parent=MALK_02940.t1
contig010	AUGUSTUS	gene	552008	553489	1	+	.	ID=MALK_02941;prediction_source=augustus:contig010.g1245.t1
contig010	AUGUSTUS	CDS	552008	553489	1	+	0	ID=MALK_02941.t1.c1;Parent=MALK_02941.t1
contig010	AUGUSTUS	mRNA	552008	553489	1	+	.	ID=MALK_02941.t1;Parent=MALK_02941

contig010	AUGUSTUS	exon	552008	553489	1	+	.	ID=MALK_02941.t1.e1;Parent=MALK_02941.t1
contig010	maker	gene	553486	554943	.	-	.	ID=MALK_02942;prediction_source=maker_MRET:augustus_masked-contig010-processed-gene-5.29-mRNA-1
contig010	maker	CDS	553486	554943	.	-	0	ID=MALK_02942.t1.c1;Parent=MALK_02942.t1
contig010	maker	mRNA	553486	554943	.	-	.	ID=MALK_02942.t1;Parent=MALK_02942
contig010	maker	exon	553486	554943	.	-	.	ID=MALK_02942.t1.e1;Parent=MALK_02942.t1
contig010	maker	gene	555082	556284	.	+	.	ID=MALK_02943;prediction_source=maker_MRET:augustus_masked-contig010-processed-gene-5.10-mRNA-1
contig010	maker	CDS	555082	555127	.	+	0	ID=MALK_02943.t1.c1;Parent=MALK_02943.t1
contig010	maker	CDS	555209	556284	.	+	0	ID=MALK_02943.t1.c2;Parent=MALK_02943.t1
contig010	maker	mRNA	555082	556284	.	+	.	ID=MALK_02943.t1;Parent=MALK_02943
contig010	maker	exon	555082	555127	.	+	.	ID=MALK_02943.t1.e1;Parent=MALK_02943.t1
contig010	maker	exon	555209	556284	.	+	.	ID=MALK_02943.t1.e2;Parent=MALK_02943.t1
contig010	AUGUSTUS	gene	556335	558596	0.87	+	.	ID=MALK_02944;prediction_source=braker_MRET:g1497.t1
contig010	AUGUSTUS	CDS	556335	557759	0.93	+	0	ID=MALK_02944.t1.c1;Parent=MALK_02944.t1
contig010	AUGUSTUS	CDS	557822	558122	0.93	+	0	ID=MALK_02944.t1.c2;Parent=MALK_02944.t1
contig010	AUGUSTUS	CDS	558151	558596	0.93	+	0	ID=MALK_02944.t1.c3;Parent=MALK_02944.t1
contig010	AUGUSTUS	mRNA	556335	558596	0.87	+	.	ID=MALK_02944.t1;Parent=MALK_02944
contig010	AUGUSTUS	exon	556335	557759	.	+	.	ID=MALK_02944.t1.e1;Parent=MALK_02944.t1
contig010	AUGUSTUS	exon	557822	558122	.	+	.	ID=MALK_02944.t1.e2;Parent=MALK_02944.t1
contig010	AUGUSTUS	exon	558151	558596	.	+	.	ID=MALK_02944.t1.e3;Parent=MALK_02944.t1
contig010	AUGUSTUS	gene	558626	559078	0.98	-	.	ID=MALK_02945;prediction_source=braker_MRET:g1498.t1
contig010	AUGUSTUS	CDS	558982	559078	0.98	-	0	ID=MALK_02945.t1.c2;Parent=MALK_02945.t1
contig010	AUGUSTUS	CDS	558626	558951	0.98	-	0	ID=MALK_02945.t1.c1;Parent=MALK_02945.t1
contig010	AUGUSTUS	mRNA	558626	559078	0.98	-	.	ID=MALK_02945.t1;Parent=MALK_02945
contig010	AUGUSTUS	exon	558982	559078	.	-	.	ID=MALK_02945.t1.e2;Parent=MALK_02945.t1
contig010	AUGUSTUS	exon	558626	558951	.	-	.	ID=MALK_02945.t1.e1;Parent=MALK_02945.t1
contig010	AUGUSTUS	gene	559198	563391	1	+	.	ID=MALK_02946;prediction_source=braker_MRET:g1499.t1
contig010	AUGUSTUS	CDS	559198	563391	1	+	0	ID=MALK_02946.t1.c1;Parent=MALK_02946.t1
contig010	AUGUSTUS	mRNA	559198	563391	1	+	.	ID=MALK_02946.t1;Parent=MALK_02946
contig010	AUGUSTUS	exon	559198	563391	.	+	.	ID=MALK_02946.t1.e1;Parent=MALK_02946.t1
contig010	maker	gene	563435	564796	.	-	.	ID=MALK_02947;prediction_source=maker_MRET:augustus_masked-contig010-processed-gene-5.30-mRNA-1
contig010	maker	CDS	563435	564796	.	-	0	ID=MALK_02947.t1.c1;Parent=MALK_02947.t1
contig010	maker	mRNA	563435	564796	.	-	.	ID=MALK_02947.t1;Parent=MALK_02947
contig010	maker	exon	563435	564796	.	-	.	ID=MALK_02947.t1.e1;Parent=MALK_02947.t1
contig010	maker	gene	565376	566392	.	-	.	ID=MALK_02948;prediction_source=maker_MRET:augustus_masked-contig010-processed-gene-5.31-mRNA-1
contig010	maker	CDS	565376	566392	.	-	0	ID=MALK_02948.t1.c1;Parent=MALK_02948.t1
contig010	maker	mRNA	565376	566392	.	-	.	ID=MALK_02948.t1;Parent=MALK_02948
contig010	maker	exon	565376	566392	.	-	.	ID=MALK_02948.t1.e1;Parent=MALK_02948.t1
contig010	AUGUSTUS	gene	566693	567322	1	+	.	ID=MALK_02949;prediction_source=augustus:contig010.g1254.t1
contig010	AUGUSTUS	CDS	566693	567322	1	+	0	ID=MALK_02949.t1.c1;Parent=MALK_02949.t1
contig010	AUGUSTUS	mRNA	566693	567322	1	+	.	ID=MALK_02949.t1;Parent=MALK_02949
contig010	AUGUSTUS	exon	566693	567322	1	+	.	ID=MALK_02949.t1.e1;Parent=MALK_02949.t1
contig010	AUGUSTUS	gene	567335	568267	0.58	-	.	ID=MALK_02950;prediction_source=augustus:contig010.g1255.t1
contig010	AUGUSTUS	CDS	567335	568267	0.58	-	0	ID=MALK_02950.t1.c1;Parent=MALK_02950.t1

contig010	AUGUSTUS	mRNA	567335	568267	0.58	-	.	ID=MALK_02950.t1;Parent=MALK_02950
contig010	AUGUSTUS	exon	567335	568267	0.58	-	.	ID=MALK_02950.t1.e1;Parent=MALK_02950.t1
contig010	AUGUSTUS	gene	568519	569826	0.29	+	.	ID=MALK_02951;prediction_source=braker_MRET:g1504.t1
contig010	AUGUSTUS	CDS	568519	568626	1	+	0	ID=MALK_02951.t1.c1;Parent=MALK_02951.t1
contig010	AUGUSTUS	CDS	568654	569197	1	+	0	ID=MALK_02951.t1.c2;Parent=MALK_02951.t1
contig010	AUGUSTUS	CDS	569249	569493	1	+	0	ID=MALK_02951.t1.c3;Parent=MALK_02951.t1
contig010	AUGUSTUS	CDS	569617	569749	1	+	0	ID=MALK_02951.t1.c4;Parent=MALK_02951.t1
contig010	AUGUSTUS	CDS	569786	569826	1	+	0	ID=MALK_02951.t1.c5;Parent=MALK_02951.t1
contig010	AUGUSTUS	mRNA	568519	569826	0.29	+	.	ID=MALK_02951.t1;Parent=MALK_02951
contig010	AUGUSTUS	exon	568519	568626	.	+	.	ID=MALK_02951.t1.e1;Parent=MALK_02951.t1
contig010	AUGUSTUS	exon	568654	569197	.	+	.	ID=MALK_02951.t1.e2;Parent=MALK_02951.t1
contig010	AUGUSTUS	exon	569249	569493	.	+	.	ID=MALK_02951.t1.e3;Parent=MALK_02951.t1
contig010	AUGUSTUS	exon	569617	569749	.	+	.	ID=MALK_02951.t1.e4;Parent=MALK_02951.t1
contig010	AUGUSTUS	exon	569786	569826	.	+	.	ID=MALK_02951.t1.e5;Parent=MALK_02951.t1
contig010	AUGUSTUS	gene	569867	571711	0.56	-	.	ID=MALK_02952;prediction_source=braker_MRET:g1505.t1
contig010	AUGUSTUS	CDS	569867	571711	0.56	-	0	ID=MALK_02952.t1.c1;Parent=MALK_02952.t1
contig010	AUGUSTUS	mRNA	569867	571711	0.56	-	.	ID=MALK_02952.t1;Parent=MALK_02952
contig010	AUGUSTUS	exon	569867	571711	.	-	.	ID=MALK_02952.t1.e1;Parent=MALK_02952.t1
contig010	AUGUSTUS	gene	571724	573107	1	-	.	ID=MALK_02953;prediction_source=braker_MRET:g1506.t1
contig010	AUGUSTUS	CDS	572882	573107	1	-	0	ID=MALK_02953.t1.c2;Parent=MALK_02953.t1
contig010	AUGUSTUS	CDS	571724	572718	1	-	0	ID=MALK_02953.t1.c1;Parent=MALK_02953.t1
contig010	AUGUSTUS	mRNA	571724	573107	1	-	.	ID=MALK_02953.t1;Parent=MALK_02953
contig010	AUGUSTUS	exon	572882	573107	.	-	.	ID=MALK_02953.t1.e2;Parent=MALK_02953.t1
contig010	AUGUSTUS	exon	571724	572718	.	-	.	ID=MALK_02953.t1.e1;Parent=MALK_02953.t1
contig010	maker	gene	573440	574357	.	+	.	ID=MALK_02954;prediction_source=maker_MRET:augustus_masked-contig010-processed-gene-5.15-mRNA-1
contig010	maker	CDS	573440	574357	.	+	0	ID=MALK_02954.t1.c1;Parent=MALK_02954.t1
contig010	maker	mRNA	573440	574357	.	+	.	ID=MALK_02954.t1;Parent=MALK_02954
contig010	maker	exon	573440	574357	.	+	.	ID=MALK_02954.t1.e1;Parent=MALK_02954.t1
contig010	maker	gene	574375	574758	.	-	.	ID=MALK_02955;prediction_source=maker_MRET:augustus_masked-contig010-processed-gene-5.34-mRNA-1
contig010	maker	CDS	574375	574758	.	-	0	ID=MALK_02955.t1.c1;Parent=MALK_02955.t1
contig010	maker	mRNA	574375	574758	.	-	.	ID=MALK_02955.t1;Parent=MALK_02955
contig010	maker	exon	574375	574758	.	-	.	ID=MALK_02955.t1.e1;Parent=MALK_02955.t1
contig010	AUGUSTUS	gene	575011	575304	1	+	.	ID=MALK_02956;prediction_source=augustus:contig010.g1259.t1
contig010	AUGUSTUS	CDS	575011	575304	1	+	0	ID=MALK_02956.t1.c1;Parent=MALK_02956.t1
contig010	AUGUSTUS	mRNA	575011	575304	1	+	.	ID=MALK_02956.t1;Parent=MALK_02956
contig010	AUGUSTUS	exon	575011	575304	1	+	.	ID=MALK_02956.t1.e1;Parent=MALK_02956.t1
contig010	AUGUSTUS	gene	575456	576364	0.6	-	.	ID=MALK_02957;prediction_source=braker_MRET:g1510.t1
contig010	AUGUSTUS	CDS	575456	576364	0.6	-	0	ID=MALK_02957.t1.c1;Parent=MALK_02957.t1
contig010	AUGUSTUS	mRNA	575456	576364	0.6	-	.	ID=MALK_02957.t1;Parent=MALK_02957
contig010	AUGUSTUS	exon	575456	576364	.	-	.	ID=MALK_02957.t1.e1;Parent=MALK_02957.t1
contig010	AUGUSTUS	gene	576446	578380	0.61	-	.	ID=MALK_02958;prediction_source=braker_MRET:g1511.t1
contig010	AUGUSTUS	CDS	576446	578380	0.61	-	0	ID=MALK_02958.t1.c1;Parent=MALK_02958.t1
contig010	AUGUSTUS	mRNA	576446	578380	0.61	-	.	ID=MALK_02958.t1;Parent=MALK_02958

contig010	AUGUSTUS	exon	576446	578380	.	-	.	ID=MALK_02958.t1.e1;Parent=MALK_02958.t1
contig010	AUGUSTUS	gene	578663	581122	0.7	+	.	ID=MALK_02959;prediction_source=braker_MRET:g1512.t1
contig010	AUGUSTUS	CDS	578663	579553	0.97	+	0	ID=MALK_02959.t1.c1;Parent=MALK_02959.t1
contig010	AUGUSTUS	CDS	579641	581122	0.97	+	0	ID=MALK_02959.t1.c2;Parent=MALK_02959.t1
contig010	AUGUSTUS	mRNA	578663	581122	0.7	+	.	ID=MALK_02959.t1;Parent=MALK_02959
contig010	AUGUSTUS	exon	578663	579553	.	+	.	ID=MALK_02959.t1.e1;Parent=MALK_02959.t1
contig010	AUGUSTUS	exon	579641	581122	.	+	.	ID=MALK_02959.t1.e2;Parent=MALK_02959.t1
contig010	AUGUSTUS	gene	581119	581658	0.98	-	.	ID=MALK_02960;prediction_source=augustus:contig010.g1261.t1
contig010	AUGUSTUS	CDS	581119	581658	0.98	-	0	ID=MALK_02960.t1.c1;Parent=MALK_02960.t1
contig010	AUGUSTUS	mRNA	581119	581658	0.98	-	.	ID=MALK_02960.t1;Parent=MALK_02960
contig010	AUGUSTUS	exon	581119	581658	0.98	-	.	ID=MALK_02960.t1.e1;Parent=MALK_02960.t1
contig010	AUGUSTUS	gene	581695	583065	0.94	-	.	ID=MALK_02961;prediction_source=augustus:contig010.g1262.t1
contig010	AUGUSTUS	CDS	581695	583065	0.94	-	0	ID=MALK_02961.t1.c1;Parent=MALK_02961.t1
contig010	AUGUSTUS	mRNA	581695	583065	0.94	-	.	ID=MALK_02961.t1;Parent=MALK_02961
contig010	AUGUSTUS	exon	581695	583065	0.94	-	.	ID=MALK_02961.t1.e1;Parent=MALK_02961.t1
contig010	AUGUSTUS	gene	583299	585056	0.85	-	.	ID=MALK_02962;prediction_source=braker_MRET:g1514.t1
contig010	AUGUSTUS	CDS	583299	585056	0.85	-	0	ID=MALK_02962.t1.c1;Parent=MALK_02962.t1
contig010	AUGUSTUS	mRNA	583299	585056	0.85	-	.	ID=MALK_02962.t1;Parent=MALK_02962
contig010	AUGUSTUS	exon	583299	585056	.	-	.	ID=MALK_02962.t1.e1;Parent=MALK_02962.t1
contig010	AUGUSTUS	gene	585472	586154	0.34	-	.	ID=MALK_02963;prediction_source=braker_MRET:g1515.t1
contig010	AUGUSTUS	CDS	585719	586154	0.35	-	0	ID=MALK_02963.t1.c2;Parent=MALK_02963.t1
contig010	AUGUSTUS	CDS	585472	585677	0.35	-	0	ID=MALK_02963.t1.c1;Parent=MALK_02963.t1
contig010	AUGUSTUS	mRNA	585472	586154	0.34	-	.	ID=MALK_02963.t1;Parent=MALK_02963
contig010	AUGUSTUS	exon	585719	586154	.	-	.	ID=MALK_02963.t1.e2;Parent=MALK_02963.t1
contig010	AUGUSTUS	exon	585472	585677	.	-	.	ID=MALK_02963.t1.e1;Parent=MALK_02963.t1
contig010	AUGUSTUS	gene	586322	587053	0.85	+	.	ID=MALK_02964;prediction_source=augustus:contig010.g1264.t1
contig010	AUGUSTUS	CDS	586322	587053	0.85	+	0	ID=MALK_02964.t1.c1;Parent=MALK_02964.t1
contig010	AUGUSTUS	mRNA	586322	587053	0.85	+	.	ID=MALK_02964.t1;Parent=MALK_02964
contig010	AUGUSTUS	exon	586322	587053	0.85	+	.	ID=MALK_02964.t1.e1;Parent=MALK_02964.t1
contig010	AUGUSTUS	gene	587167	587623	0.99	+	.	ID=MALK_02965;prediction_source=braker_MRET:g1517.t1
contig010	AUGUSTUS	CDS	587167	587271	1	+	0	ID=MALK_02965.t1.c1;Parent=MALK_02965.t1
contig010	AUGUSTUS	CDS	587307	587502	1	+	0	ID=MALK_02965.t1.c2;Parent=MALK_02965.t1
contig010	AUGUSTUS	CDS	587547	587623	1	+	0	ID=MALK_02965.t1.c3;Parent=MALK_02965.t1
contig010	AUGUSTUS	mRNA	587167	587623	0.99	+	.	ID=MALK_02965.t1;Parent=MALK_02965
contig010	AUGUSTUS	exon	587167	587271	.	+	.	ID=MALK_02965.t1.e1;Parent=MALK_02965.t1
contig010	AUGUSTUS	exon	587307	587502	.	+	.	ID=MALK_02965.t1.e2;Parent=MALK_02965.t1
contig010	AUGUSTUS	exon	587547	587623	.	+	.	ID=MALK_02965.t1.e3;Parent=MALK_02965.t1
contig010	AUGUSTUS	gene	587672	588634	0.99	-	.	ID=MALK_02966;prediction_source=augustus:contig010.g1266.t1
contig010	AUGUSTUS	CDS	587672	588634	0.99	-	0	ID=MALK_02966.t1.c1;Parent=MALK_02966.t1
contig010	AUGUSTUS	mRNA	587672	588634	0.99	-	.	ID=MALK_02966.t1;Parent=MALK_02966
contig010	AUGUSTUS	exon	587672	588634	0.99	-	.	ID=MALK_02966.t1.e1;Parent=MALK_02966.t1
contig010	maker	gene	589438	590946	.	+	.	ID=MALK_02967;prediction_source=maker_MRET:augustus_masked-contig010-processed-gene-5.19-mRNA-1
contig010	maker	CDS	589438	590946	.	+	0	ID=MALK_02967.t1.c1;Parent=MALK_02967.t1

contig010	maker	mRNA	589438	590946	.	+	.	ID=MALK_02967.t1;Parent=MALK_02967
contig010	maker	exon	589438	590946	.	+	.	ID=MALK_02967.t1.e1;Parent=MALK_02967.t1
contig010	AUGUSTUS	gene	591181	592467	0.99	-	.	ID=MALK_02968;prediction_source=braker_MRET:g1520.t1
contig010	AUGUSTUS	CDS	591181	592467	0.99	-	0	ID=MALK_02968.t1.c1;Parent=MALK_02968.t1
contig010	AUGUSTUS	mRNA	591181	592467	0.99	-	.	ID=MALK_02968.t1;Parent=MALK_02968
contig010	AUGUSTUS	exon	591181	592467	.	-	.	ID=MALK_02968.t1.e1;Parent=MALK_02968.t1
contig010	AUGUSTUS	gene	592496	593044	0.96	-	.	ID=MALK_02969;prediction_source=braker_MRET:g1521.t1
contig010	AUGUSTUS	CDS	592496	593044	0.96	-	0	ID=MALK_02969.t1.c1;Parent=MALK_02969.t1
contig010	AUGUSTUS	mRNA	592496	593044	0.96	-	.	ID=MALK_02969.t1;Parent=MALK_02969
contig010	AUGUSTUS	exon	592496	593044	.	-	.	ID=MALK_02969.t1.e1;Parent=MALK_02969.t1
contig010	AUGUSTUS	gene	593185	594675	1	+	.	ID=MALK_02970;prediction_source=braker_MRET:g1522.t1
contig010	AUGUSTUS	CDS	593185	594675	1	+	0	ID=MALK_02970.t1.c1;Parent=MALK_02970.t1
contig010	AUGUSTUS	mRNA	593185	594675	1	+	.	ID=MALK_02970.t1;Parent=MALK_02970
contig010	AUGUSTUS	exon	593185	594675	.	+	.	ID=MALK_02970.t1.e1;Parent=MALK_02970.t1
contig010	AUGUSTUS	gene	594764	595532	1	-	.	ID=MALK_02971;prediction_source=braker_MRET:g1523.t1
contig010	AUGUSTUS	CDS	594811	595532	1	-	0	ID=MALK_02971.t1.c2;Parent=MALK_02971.t1
contig010	AUGUSTUS	CDS	594764	594770	1	-	0	ID=MALK_02971.t1.c1;Parent=MALK_02971.t1
contig010	AUGUSTUS	mRNA	594764	595532	1	-	.	ID=MALK_02971.t1;Parent=MALK_02971
contig010	AUGUSTUS	exon	594811	595532	.	-	.	ID=MALK_02971.t1.e2;Parent=MALK_02971.t1
contig010	AUGUSTUS	exon	594764	594770	.	-	.	ID=MALK_02971.t1.e1;Parent=MALK_02971.t1
contig010	AUGUSTUS	gene	595610	599017	0.99	+	.	ID=MALK_02972;prediction_source=augustus:contig010.g1272.t1
contig010	AUGUSTUS	CDS	595610	599017	0.99	+	0	ID=MALK_02972.t1.c1;Parent=MALK_02972.t1
contig010	AUGUSTUS	mRNA	595610	599017	0.99	+	.	ID=MALK_02972.t1;Parent=MALK_02972
contig010	AUGUSTUS	exon	595610	599017	0.99	+	.	ID=MALK_02972.t1.e1;Parent=MALK_02972.t1
contig010	AUGUSTUS	gene	599067	599833	0.52	+	.	ID=MALK_02973;prediction_source=braker_MRET:g1525.t1
contig010	AUGUSTUS	CDS	599067	599411	0.6	+	0	ID=MALK_02973.t1.c1;Parent=MALK_02973.t1
contig010	AUGUSTUS	CDS	599449	599475	0.6	+	0	ID=MALK_02973.t1.c2;Parent=MALK_02973.t1
contig010	AUGUSTUS	CDS	599611	599614	0.6	+	0	ID=MALK_02973.t1.c3;Parent=MALK_02973.t1
contig010	AUGUSTUS	CDS	599802	599833	0.6	+	0	ID=MALK_02973.t1.c4;Parent=MALK_02973.t1
contig010	AUGUSTUS	mRNA	599067	599833	0.52	+	.	ID=MALK_02973.t1;Parent=MALK_02973
contig010	AUGUSTUS	exon	599067	599411	.	+	.	ID=MALK_02973.t1.e1;Parent=MALK_02973.t1
contig010	AUGUSTUS	exon	599449	599475	.	+	.	ID=MALK_02973.t1.e2;Parent=MALK_02973.t1
contig010	AUGUSTUS	exon	599611	599614	.	+	.	ID=MALK_02973.t1.e3;Parent=MALK_02973.t1
contig010	AUGUSTUS	exon	599802	599833	.	+	.	ID=MALK_02973.t1.e4;Parent=MALK_02973.t1
contig010	maker	gene	599919	600509	.	-	.	ID=MALK_02974;prediction_source=maker_MRET:augustus_masked-contig010-processed-gene-6.61-mRNA-1
contig010	maker	CDS	599919	600509	.	-	0	ID=MALK_02974.t1.c1;Parent=MALK_02974.t1
contig010	maker	mRNA	599919	600509	.	-	.	ID=MALK_02974.t1;Parent=MALK_02974
contig010	maker	exon	599919	600509	.	-	.	ID=MALK_02974.t1.e1;Parent=MALK_02974.t1
contig010	AUGUSTUS	gene	600588	601239	1	-	.	ID=MALK_02975;prediction_source=braker_MRET:g1527.t1
contig010	AUGUSTUS	CDS	600986	601239	1	-	0	ID=MALK_02975.t1.c3;Parent=MALK_02975.t1
contig010	AUGUSTUS	CDS	600873	600952	1	-	0	ID=MALK_02975.t1.c2;Parent=MALK_02975.t1
contig010	AUGUSTUS	CDS	600588	600703	1	-	0	ID=MALK_02975.t1.c1;Parent=MALK_02975.t1
contig010	AUGUSTUS	mRNA	600588	601239	1	-	.	ID=MALK_02975.t1;Parent=MALK_02975

contig010	AUGUSTUS	exon	600986	601239	.	-	.	ID=MALK_02975.t1.e3;Parent=MALK_02975.t1
contig010	AUGUSTUS	exon	600873	600952	.	-	.	ID=MALK_02975.t1.e2;Parent=MALK_02975.t1
contig010	AUGUSTUS	exon	600588	600703	.	-	.	ID=MALK_02975.t1.e1;Parent=MALK_02975.t1
contig010	AUGUSTUS	gene	601387	601975	0.55	+	.	ID=MALK_02976;prediction_source=braker_MRET:g1528.t1
contig010	AUGUSTUS	CDS	601387	601440	0.55	+	0	ID=MALK_02976.t1.c1;Parent=MALK_02976.t1
contig010	AUGUSTUS	CDS	601526	601975	0.55	+	0	ID=MALK_02976.t1.c2;Parent=MALK_02976.t1
contig010	AUGUSTUS	mRNA	601387	601975	0.55	+	.	ID=MALK_02976.t1;Parent=MALK_02976
contig010	AUGUSTUS	exon	601387	601440	.	+	.	ID=MALK_02976.t1.e1;Parent=MALK_02976.t1
contig010	AUGUSTUS	exon	601526	601975	.	+	.	ID=MALK_02976.t1.e2;Parent=MALK_02976.t1
contig010	AUGUSTUS	gene	602064	604208	0.56	+	.	ID=MALK_02977;prediction_source=braker_MRET:g1529.t1
contig010	AUGUSTUS	CDS	602064	604208	0.56	+	0	ID=MALK_02977.t1.c1;Parent=MALK_02977.t1
contig010	AUGUSTUS	mRNA	602064	604208	0.56	+	.	ID=MALK_02977.t1;Parent=MALK_02977
contig010	AUGUSTUS	exon	602064	604208	.	+	.	ID=MALK_02977.t1.e1;Parent=MALK_02977.t1
contig010	AUGUSTUS	gene	604271	604786	1	+	.	ID=MALK_02978;prediction_source=augustus:contig010.g1277.t1
contig010	AUGUSTUS	CDS	604271	604786	1	+	0	ID=MALK_02978.t1.c1;Parent=MALK_02978.t1
contig010	AUGUSTUS	mRNA	604271	604786	1	+	.	ID=MALK_02978.t1;Parent=MALK_02978
contig010	AUGUSTUS	exon	604271	604786	1	+	.	ID=MALK_02978.t1.e1;Parent=MALK_02978.t1
contig010	AUGUSTUS	gene	604789	605733	1	-	.	ID=MALK_02979;prediction_source=braker_MRET:g1531.t1
contig010	AUGUSTUS	CDS	604789	605733	1	-	0	ID=MALK_02979.t1.c1;Parent=MALK_02979.t1
contig010	AUGUSTUS	mRNA	604789	605733	1	-	.	ID=MALK_02979.t1;Parent=MALK_02979
contig010	AUGUSTUS	exon	604789	605733	.	-	.	ID=MALK_02979.t1.e1;Parent=MALK_02979.t1
contig010	maker	gene	605798	606934	.	+	.	ID=MALK_02980;prediction_source=maker_MRET:augustus_masked-contig010-processed-gene-6.65-mRNA-1
contig010	maker	CDS	605798	606934	.	+	0	ID=MALK_02980.t1.c1;Parent=MALK_02980.t1
contig010	maker	mRNA	605798	606934	.	+	.	ID=MALK_02980.t1;Parent=MALK_02980
contig010	maker	exon	605798	606934	.	+	.	ID=MALK_02980.t1.e1;Parent=MALK_02980.t1
contig010	maker	gene	607235	607927	.	-	.	ID=MALK_02981;prediction_source=maker_MRET:augustus_masked-contig010-processed-gene-6.89-mRNA-1
contig010	maker	CDS	607235	607927	.	-	0	ID=MALK_02981.t1.c1;Parent=MALK_02981.t1
contig010	maker	mRNA	607235	607927	.	-	.	ID=MALK_02981.t1;Parent=MALK_02981
contig010	maker	exon	607235	607927	.	-	.	ID=MALK_02981.t1.e1;Parent=MALK_02981.t1
contig010	AUGUSTUS	gene	609566	610336	0.69	+	.	ID=MALK_02982;prediction_source=augustus:contig010.g1280.t1
contig010	AUGUSTUS	CDS	609566	610336	0.69	+	0	ID=MALK_02982.t1.c1;Parent=MALK_02982.t1
contig010	AUGUSTUS	mRNA	609566	610336	0.69	+	.	ID=MALK_02982.t1;Parent=MALK_02982
contig010	AUGUSTUS	exon	609566	610336	0.69	+	.	ID=MALK_02982.t1.e1;Parent=MALK_02982.t1
contig010	maker	gene	610541	611551	.	+	.	ID=MALK_02983;prediction_source=maker_MRET:augustus_masked-contig010-processed-gene-6.67-mRNA-1
contig010	maker	CDS	610541	611551	.	+	0	ID=MALK_02983.t1.c1;Parent=MALK_02983.t1
contig010	maker	mRNA	610541	611551	.	+	.	ID=MALK_02983.t1;Parent=MALK_02983
contig010	maker	exon	610541	611551	.	+	.	ID=MALK_02983.t1.e1;Parent=MALK_02983.t1
contig010	maker	gene	611563	613884	.	-	.	ID=MALK_02984;prediction_source=maker_MRET:augustus_masked-contig010-processed-gene-6.90-mRNA-1
contig010	maker	CDS	611563	613884	.	-	0	ID=MALK_02984.t1.c1;Parent=MALK_02984.t1
contig010	maker	mRNA	611563	613884	.	-	.	ID=MALK_02984.t1;Parent=MALK_02984
contig010	maker	exon	611563	613884	.	-	.	ID=MALK_02984.t1.e1;Parent=MALK_02984.t1
contig010	AUGUSTUS	gene	614383	615891	0.77	+	.	ID=MALK_02985;prediction_source=augustus:contig010.g1283.t1
contig010	AUGUSTUS	CDS	614383	615891	0.77	+	0	ID=MALK_02985.t1.c1;Parent=MALK_02985.t1

contig010	AUGUSTUS	mRNA	614383	615891	0.77	+	.	ID=MALK_02985.t1;Parent=MALK_02985
contig010	AUGUSTUS	exon	614383	615891	0.77	+	.	ID=MALK_02985.t1.e1;Parent=MALK_02985.t1
contig010	maker	gene	615898	617757	.	-	.	ID=MALK_02986;prediction_source=maker_MRET:augustus_masked-contig010-processed-gene-6.91-mRNA-1
contig010	maker	CDS	615898	617757	.	-	0	ID=MALK_02986.t1.c1;Parent=MALK_02986.t1
contig010	maker	mRNA	615898	617757	.	-	.	ID=MALK_02986.t1;Parent=MALK_02986
contig010	maker	exon	615898	617757	.	-	.	ID=MALK_02986.t1.e1;Parent=MALK_02986.t1
contig010	AUGUSTUS	gene	617784	618941	1	-	.	ID=MALK_02987;prediction_source=braker_MRET:g1538.t1
contig010	AUGUSTUS	CDS	617784	618941	1	-	0	ID=MALK_02987.t1.c1;Parent=MALK_02987.t1
contig010	AUGUSTUS	mRNA	617784	618941	1	-	.	ID=MALK_02987.t1;Parent=MALK_02987
contig010	AUGUSTUS	exon	617784	618941	.	-	.	ID=MALK_02987.t1.e1;Parent=MALK_02987.t1
contig010	AUGUSTUS	gene	619056	619826	0.35	+	.	ID=MALK_02988;prediction_source=braker_MRET:g1539.t1
contig010	AUGUSTUS	CDS	619056	619150	0.7	+	0	ID=MALK_02988.t1.c1;Parent=MALK_02988.t1
contig010	AUGUSTUS	CDS	619181	619221	0.7	+	0	ID=MALK_02988.t1.c2;Parent=MALK_02988.t1
contig010	AUGUSTUS	CDS	619249	619826	0.7	+	0	ID=MALK_02988.t1.c3;Parent=MALK_02988.t1
contig010	AUGUSTUS	mRNA	619056	619826	0.35	+	.	ID=MALK_02988.t1;Parent=MALK_02988
contig010	AUGUSTUS	exon	619056	619150	.	+	.	ID=MALK_02988.t1.e1;Parent=MALK_02988.t1
contig010	AUGUSTUS	exon	619181	619221	.	+	.	ID=MALK_02988.t1.e2;Parent=MALK_02988.t1
contig010	AUGUSTUS	exon	619249	619826	.	+	.	ID=MALK_02988.t1.e3;Parent=MALK_02988.t1
contig010	AUGUSTUS	gene	619874	620603	0.82	+	.	ID=MALK_02989;prediction_source=braker_MRET:g1540.t1
contig010	AUGUSTUS	CDS	619874	620550	0.96	+	0	ID=MALK_02989.t1.c1;Parent=MALK_02989.t1
contig010	AUGUSTUS	CDS	620591	620603	0.96	+	0	ID=MALK_02989.t1.c2;Parent=MALK_02989.t1
contig010	AUGUSTUS	mRNA	619874	620603	0.82	+	.	ID=MALK_02989.t1;Parent=MALK_02989
contig010	AUGUSTUS	exon	619874	620550	.	+	.	ID=MALK_02989.t1.e1;Parent=MALK_02989.t1
contig010	AUGUSTUS	exon	620591	620603	.	+	.	ID=MALK_02989.t1.e2;Parent=MALK_02989.t1
contig010	AUGUSTUS	gene	620620	621801	0.99	-	.	ID=MALK_02990;prediction_source=braker_MRET:g1541.t1
contig010	AUGUSTUS	CDS	620620	621801	0.99	-	0	ID=MALK_02990.t1.c1;Parent=MALK_02990.t1
contig010	AUGUSTUS	mRNA	620620	621801	0.99	-	.	ID=MALK_02990.t1;Parent=MALK_02990
contig010	AUGUSTUS	exon	620620	621801	.	-	.	ID=MALK_02990.t1.e1;Parent=MALK_02990.t1
contig010	AUGUSTUS	gene	622112	623302	1	+	.	ID=MALK_02991;prediction_source=braker_MRET:g1542.t1
contig010	AUGUSTUS	CDS	622112	623302	1	+	0	ID=MALK_02991.t1.c1;Parent=MALK_02991.t1
contig010	AUGUSTUS	mRNA	622112	623302	1	+	.	ID=MALK_02991.t1;Parent=MALK_02991
contig010	AUGUSTUS	exon	622112	623302	.	+	.	ID=MALK_02991.t1.e1;Parent=MALK_02991.t1
contig010	AUGUSTUS	gene	623307	623831	0.36	-	.	ID=MALK_02992;prediction_source=augustus:contig010.g1289.t1
contig010	AUGUSTUS	CDS	623307	623831	0.36	-	0	ID=MALK_02992.t1.c1;Parent=MALK_02992.t1
contig010	AUGUSTUS	mRNA	623307	623831	0.36	-	.	ID=MALK_02992.t1;Parent=MALK_02992
contig010	AUGUSTUS	exon	623307	623831	0.36	-	.	ID=MALK_02992.t1.e1;Parent=MALK_02992.t1
contig010	AUGUSTUS	gene	623860	625791	0.99	-	.	ID=MALK_02993;prediction_source=braker_MRET:g1544.t1
contig010	AUGUSTUS	CDS	623860	625791	0.99	-	0	ID=MALK_02993.t1.c1;Parent=MALK_02993.t1
contig010	AUGUSTUS	mRNA	623860	625791	0.99	-	.	ID=MALK_02993.t1;Parent=MALK_02993
contig010	AUGUSTUS	exon	623860	625791	.	-	.	ID=MALK_02993.t1.e1;Parent=MALK_02993.t1
contig010	AUGUSTUS	gene	625854	627014	0.89	+	.	ID=MALK_02994;prediction_source=augustus:contig010.g1291.t1
contig010	AUGUSTUS	CDS	625854	627014	0.89	+	0	ID=MALK_02994.t1.c1;Parent=MALK_02994.t1
contig010	AUGUSTUS	mRNA	625854	627014	0.89	+	.	ID=MALK_02994.t1;Parent=MALK_02994

contig010	AUGUSTUS	exon	625854	627014	0.89	+	.	ID=MALK_02994.t1.e1;Parent=MALK_02994.t1
contig010	AUGUSTUS	gene	627037	629064	1	+	.	ID=MALK_02995;prediction_source=augustus:contig010.g1292.t1
contig010	AUGUSTUS	CDS	627037	629064	1	+	0	ID=MALK_02995.t1.c1;Parent=MALK_02995.t1
contig010	AUGUSTUS	mRNA	627037	629064	1	+	.	ID=MALK_02995.t1;Parent=MALK_02995
contig010	AUGUSTUS	exon	627037	629064	1	+	.	ID=MALK_02995.t1.e1;Parent=MALK_02995.t1
contig010	AUGUSTUS	gene	629722	630945	0.96	+	.	ID=MALK_02996;prediction_source=braker_MRET:g1547.t1
contig010	AUGUSTUS	CDS	629722	630945	0.96	+	0	ID=MALK_02996.t1.c1;Parent=MALK_02996.t1
contig010	AUGUSTUS	mRNA	629722	630945	0.96	+	.	ID=MALK_02996.t1;Parent=MALK_02996
contig010	AUGUSTUS	exon	629722	630945	.	+	.	ID=MALK_02996.t1.e1;Parent=MALK_02996.t1
contig010	AUGUSTUS	gene	630969	632429	1	+	.	ID=MALK_02997;prediction_source=braker_MRET:g1548.t1
contig010	AUGUSTUS	CDS	630969	632429	1	+	0	ID=MALK_02997.t1.c1;Parent=MALK_02997.t1
contig010	AUGUSTUS	mRNA	630969	632429	1	+	.	ID=MALK_02997.t1;Parent=MALK_02997
contig010	AUGUSTUS	exon	630969	632429	.	+	.	ID=MALK_02997.t1.e1;Parent=MALK_02997.t1
contig010	AUGUSTUS	gene	632436	635993	1	-	.	ID=MALK_02998;prediction_source=braker_MRET:g1549.t1
contig010	AUGUSTUS	CDS	632436	635993	1	-	0	ID=MALK_02998.t1.c1;Parent=MALK_02998.t1
contig010	AUGUSTUS	mRNA	632436	635993	1	-	.	ID=MALK_02998.t1;Parent=MALK_02998
contig010	AUGUSTUS	exon	632436	635993	.	-	.	ID=MALK_02998.t1.e1;Parent=MALK_02998.t1
contig010	AUGUSTUS	gene	636114	637515	1	+	.	ID=MALK_02999;prediction_source=braker_MRET:g1550.t1
contig010	AUGUSTUS	CDS	636114	636409	1	+	0	ID=MALK_02999.t1.c1;Parent=MALK_02999.t1
contig010	AUGUSTUS	CDS	636567	637515	1	+	0	ID=MALK_02999.t1.c2;Parent=MALK_02999.t1
contig010	AUGUSTUS	mRNA	636114	637515	1	+	.	ID=MALK_02999.t1;Parent=MALK_02999
contig010	AUGUSTUS	exon	636114	636409	.	+	.	ID=MALK_02999.t1.e1;Parent=MALK_02999.t1
contig010	AUGUSTUS	exon	636567	637515	.	+	.	ID=MALK_02999.t1.e2;Parent=MALK_02999.t1
contig010	AUGUSTUS	gene	637928	638392	0.99	-	.	ID=MALK_03000;prediction_source=braker_MRET:g1551.t1
contig010	AUGUSTUS	CDS	637928	638392	0.99	-	0	ID=MALK_03000.t1.c1;Parent=MALK_03000.t1
contig010	AUGUSTUS	mRNA	637928	638392	0.99	-	.	ID=MALK_03000.t1;Parent=MALK_03000
contig010	AUGUSTUS	exon	637928	638392	.	-	.	ID=MALK_03000.t1.e1;Parent=MALK_03000.t1
contig010	maker	gene	638670	639482	.	+	.	ID=MALK_03001;prediction_source=maker_MRET:augustus_masked-contig010-processed-gene-6.75-mRNA-1
contig010	maker	CDS	638670	639482	.	+	0	ID=MALK_03001.t1.c1;Parent=MALK_03001.t1
contig010	maker	mRNA	638670	639482	.	+	.	ID=MALK_03001.t1;Parent=MALK_03001
contig010	maker	exon	638670	639482	.	+	.	ID=MALK_03001.t1.e1;Parent=MALK_03001.t1
contig010	AUGUSTUS	gene	639492	641339	0.96	-	.	ID=MALK_03002;prediction_source=braker_MRET:g1553.t1
contig010	AUGUSTUS	CDS	639492	641339	0.96	-	0	ID=MALK_03002.t1.c1;Parent=MALK_03002.t1
contig010	AUGUSTUS	mRNA	639492	641339	0.96	-	.	ID=MALK_03002.t1;Parent=MALK_03002
contig010	AUGUSTUS	exon	639492	641339	.	-	.	ID=MALK_03002.t1.e1;Parent=MALK_03002.t1
contig010	AUGUSTUS	gene	641384	642886	0.98	-	.	ID=MALK_03003;prediction_source=braker_MRET:g1554.t1
contig010	AUGUSTUS	CDS	641384	642886	0.98	-	0	ID=MALK_03003.t1.c1;Parent=MALK_03003.t1
contig010	AUGUSTUS	mRNA	641384	642886	0.98	-	.	ID=MALK_03003.t1;Parent=MALK_03003
contig010	AUGUSTUS	exon	641384	642886	.	-	.	ID=MALK_03003.t1.e1;Parent=MALK_03003.t1
contig010	AUGUSTUS	gene	642993	643886	1	+	.	ID=MALK_03004;prediction_source=braker_MRET:g1555.t1
contig010	AUGUSTUS	CDS	642993	643886	1	+	0	ID=MALK_03004.t1.c1;Parent=MALK_03004.t1
contig010	AUGUSTUS	mRNA	642993	643886	1	+	.	ID=MALK_03004.t1;Parent=MALK_03004
contig010	AUGUSTUS	exon	642993	643886	.	+	.	ID=MALK_03004.t1.e1;Parent=MALK_03004.t1

contig010	maker	gene	643877	646456	.	-	.	ID=MALK_03005;prediction_source=maker_MRET:augustus_masked-contig010-processed-gene-6.95-mRNA-1
contig010	maker	CDS	643877	646456	.	-	0	ID=MALK_03005.t1.c1;Parent=MALK_03005.t1
contig010	maker	mRNA	643877	646456	.	-	.	ID=MALK_03005.t1;Parent=MALK_03005
contig010	maker	exon	643877	646456	.	-	.	ID=MALK_03005.t1.e1;Parent=MALK_03005.t1
contig010	AUGUSTUS	gene	646701	648155	0.85	+	.	ID=MALK_03006;prediction_source=braker_MRET:g1557.t1
contig010	AUGUSTUS	CDS	646701	648155	0.85	+	0	ID=MALK_03006.t1.c1;Parent=MALK_03006.t1
contig010	AUGUSTUS	mRNA	646701	648155	0.85	+	.	ID=MALK_03006.t1;Parent=MALK_03006
contig010	AUGUSTUS	exon	646701	648155	.	+	.	ID=MALK_03006.t1.e1;Parent=MALK_03006.t1
contig010	AUGUSTUS	gene	648214	649818	1	+	.	ID=MALK_03007;prediction_source=braker_MRET:g1558.t1
contig010	AUGUSTUS	CDS	648214	649818	1	+	0	ID=MALK_03007.t1.c1;Parent=MALK_03007.t1
contig010	AUGUSTUS	mRNA	648214	649818	1	+	.	ID=MALK_03007.t1;Parent=MALK_03007
contig010	AUGUSTUS	exon	648214	649818	.	+	.	ID=MALK_03007.t1.e1;Parent=MALK_03007.t1
contig010	AUGUSTUS	gene	649848	651860	1	-	.	ID=MALK_03008;prediction_source=braker_MRET:g1559.t1
contig010	AUGUSTUS	CDS	649848	651860	1	-	0	ID=MALK_03008.t1.c1;Parent=MALK_03008.t1
contig010	AUGUSTUS	mRNA	649848	651860	1	-	.	ID=MALK_03008.t1;Parent=MALK_03008
contig010	AUGUSTUS	exon	649848	651860	.	-	.	ID=MALK_03008.t1.e1;Parent=MALK_03008.t1
contig010	AUGUSTUS	gene	651962	656599	1	+	.	ID=MALK_03009;prediction_source=braker_MRET:g1560.t1
contig010	AUGUSTUS	CDS	651962	656599	1	+	0	ID=MALK_03009.t1.c1;Parent=MALK_03009.t1
contig010	AUGUSTUS	mRNA	651962	656599	1	+	.	ID=MALK_03009.t1;Parent=MALK_03009
contig010	AUGUSTUS	exon	651962	656599	.	+	.	ID=MALK_03009.t1.e1;Parent=MALK_03009.t1
contig010	AUGUSTUS	gene	656608	658362	0.81	-	.	ID=MALK_03010;prediction_source=braker_MRET:g1561.t1
contig010	AUGUSTUS	CDS	656608	658362	0.81	-	0	ID=MALK_03010.t1.c1;Parent=MALK_03010.t1
contig010	AUGUSTUS	mRNA	656608	658362	0.81	-	.	ID=MALK_03010.t1;Parent=MALK_03010
contig010	AUGUSTUS	exon	656608	658362	.	-	.	ID=MALK_03010.t1.e1;Parent=MALK_03010.t1
contig010	AUGUSTUS	gene	658697	660898	0.85	+	.	ID=MALK_03011;prediction_source=augustus:contig010.g1300.t1
contig010	AUGUSTUS	CDS	658697	660898	0.85	+	0	ID=MALK_03011.t1.c1;Parent=MALK_03011.t1
contig010	AUGUSTUS	mRNA	658697	660898	0.85	+	.	ID=MALK_03011.t1;Parent=MALK_03011
contig010	AUGUSTUS	exon	658697	660898	0.85	+	.	ID=MALK_03011.t1.e1;Parent=MALK_03011.t1
contig010	maker	gene	661306	663159	.	+	.	ID=MALK_03012;prediction_source=maker_MRET:augustus_masked-contig010-processed-gene-6.78-mRNA-1
contig010	maker	CDS	661306	663159	.	+	0	ID=MALK_03012.t1.c1;Parent=MALK_03012.t1
contig010	maker	mRNA	661306	663159	.	+	.	ID=MALK_03012.t1;Parent=MALK_03012
contig010	maker	exon	661306	663159	.	+	.	ID=MALK_03012.t1.e1;Parent=MALK_03012.t1
contig010	AUGUSTUS	gene	663218	664720	0.77	-	.	ID=MALK_03013;prediction_source=augustus:contig010.g1304.t1
contig010	AUGUSTUS	CDS	663218	664720	0.77	-	0	ID=MALK_03013.t1.c1;Parent=MALK_03013.t1
contig010	AUGUSTUS	mRNA	663218	664720	0.77	-	.	ID=MALK_03013.t1;Parent=MALK_03013
contig010	AUGUSTUS	exon	663218	664720	0.77	-	.	ID=MALK_03013.t1.e1;Parent=MALK_03013.t1
contig010	AUGUSTUS	gene	664891	666849	0.93	+	.	ID=MALK_03014;prediction_source=augustus:contig010.g1306.t1
contig010	AUGUSTUS	CDS	664891	666849	0.93	+	0	ID=MALK_03014.t1.c1;Parent=MALK_03014.t1
contig010	AUGUSTUS	mRNA	664891	666849	0.93	+	.	ID=MALK_03014.t1;Parent=MALK_03014
contig010	AUGUSTUS	exon	664891	666849	0.93	+	.	ID=MALK_03014.t1.e1;Parent=MALK_03014.t1
contig010	maker	gene	666854	669472	.	-	.	ID=MALK_03015;prediction_source=maker_MRET:augustus_masked-contig010-processed-gene-6.97-mRNA-1
contig010	maker	CDS	666854	669472	.	-	0	ID=MALK_03015.t1.c1;Parent=MALK_03015.t1
contig010	maker	mRNA	666854	669472	.	-	.	ID=MALK_03015.t1;Parent=MALK_03015

contig010	maker	exon	666854	669472	.	-	.	ID=MALK_03015.t1.e1;Parent=MALK_03015.t1
contig010	maker	gene	669628	671418	.	-	.	ID=MALK_03016;prediction_source=maker_MRET:augustus_masked-contig010-processed-gene-6.98-mRNA-1
contig010	maker	CDS	669628	671418	.	-	0	ID=MALK_03016.t1.c1;Parent=MALK_03016.t1
contig010	maker	mRNA	669628	671418	.	-	.	ID=MALK_03016.t1;Parent=MALK_03016
contig010	maker	exon	669628	671418	.	-	.	ID=MALK_03016.t1.e1;Parent=MALK_03016.t1
contig010	AUGUSTUS	gene	671701	674706	0.5	+	.	ID=MALK_03017;prediction_source=augustus:contig010.g1310.t1
contig010	AUGUSTUS	CDS	671701	674706	0.5	+	0	ID=MALK_03017.t1.c1;Parent=MALK_03017.t1
contig010	AUGUSTUS	mRNA	671701	674706	0.5	+	.	ID=MALK_03017.t1;Parent=MALK_03017
contig010	AUGUSTUS	exon	671701	674706	0.5	+	.	ID=MALK_03017.t1.e1;Parent=MALK_03017.t1
contig010	AUGUSTUS	gene	674707	675906	0.76	-	.	ID=MALK_03018;prediction_source=braker_MRET:g1569.t1
contig010	AUGUSTUS	CDS	674707	675906	0.76	-	0	ID=MALK_03018.t1.c1;Parent=MALK_03018.t1
contig010	AUGUSTUS	mRNA	674707	675906	0.76	-	.	ID=MALK_03018.t1;Parent=MALK_03018
contig010	AUGUSTUS	exon	674707	675906	.	-	.	ID=MALK_03018.t1.e1;Parent=MALK_03018.t1
contig010	AUGUSTUS	gene	676011	677444	0.99	-	.	ID=MALK_03019;prediction_source=braker_MRET:g1570.t1
contig010	AUGUSTUS	CDS	676011	677444	0.99	-	0	ID=MALK_03019.t1.c1;Parent=MALK_03019.t1
contig010	AUGUSTUS	mRNA	676011	677444	0.99	-	.	ID=MALK_03019.t1;Parent=MALK_03019
contig010	AUGUSTUS	exon	676011	677444	.	-	.	ID=MALK_03019.t1.e1;Parent=MALK_03019.t1
contig010	AUGUSTUS	gene	677676	678522	0.71	+	.	ID=MALK_03020;prediction_source=braker_MRET:g1571.t1
contig010	AUGUSTUS	CDS	677676	677743	0.9	+	0	ID=MALK_03020.t1.c1;Parent=MALK_03020.t1
contig010	AUGUSTUS	CDS	677778	678522	0.9	+	0	ID=MALK_03020.t1.c2;Parent=MALK_03020.t1
contig010	AUGUSTUS	mRNA	677676	678522	0.71	+	.	ID=MALK_03020.t1;Parent=MALK_03020
contig010	AUGUSTUS	exon	677676	677743	.	+	.	ID=MALK_03020.t1.e1;Parent=MALK_03020.t1
contig010	AUGUSTUS	exon	677778	678522	.	+	.	ID=MALK_03020.t1.e2;Parent=MALK_03020.t1
contig010	maker	gene	678540	679976	.	-	.	ID=MALK_03021;prediction_source=maker_MRET:augustus_masked-contig010-processed-gene-6.99-mRNA-1
contig010	maker	CDS	678540	679976	.	-	0	ID=MALK_03021.t1.c1;Parent=MALK_03021.t1
contig010	maker	mRNA	678540	679976	.	-	.	ID=MALK_03021.t1;Parent=MALK_03021
contig010	maker	exon	678540	679976	.	-	.	ID=MALK_03021.t1.e1;Parent=MALK_03021.t1
contig010	maker	gene	680320	682461	.	+	.	ID=MALK_03022;prediction_source=maker_MRET:augustus_masked-contig010-processed-gene-6.82-mRNA-1
contig010	maker	CDS	680320	682461	.	+	0	ID=MALK_03022.t1.c1;Parent=MALK_03022.t1
contig010	maker	mRNA	680320	682461	.	+	.	ID=MALK_03022.t1;Parent=MALK_03022
contig010	maker	exon	680320	682461	.	+	.	ID=MALK_03022.t1.e1;Parent=MALK_03022.t1
contig010	AUGUSTUS	gene	682626	683615	0.87	+	.	ID=MALK_03023;prediction_source=augustus:contig010.g1316.t1
contig010	AUGUSTUS	CDS	682626	683615	0.87	+	0	ID=MALK_03023.t1.c1;Parent=MALK_03023.t1
contig010	AUGUSTUS	mRNA	682626	683615	0.87	+	.	ID=MALK_03023.t1;Parent=MALK_03023
contig010	AUGUSTUS	exon	682626	683615	0.87	+	.	ID=MALK_03023.t1.e1;Parent=MALK_03023.t1
contig010	AUGUSTUS	gene	683648	684310	0.23	+	.	ID=MALK_03024;prediction_source=braker_MRET:g1575.t1
contig010	AUGUSTUS	CDS	683648	683876	0.42	+	0	ID=MALK_03024.t1.c1;Parent=MALK_03024.t1
contig010	AUGUSTUS	CDS	683916	684310	0.42	+	0	ID=MALK_03024.t1.c2;Parent=MALK_03024.t1
contig010	AUGUSTUS	mRNA	683648	684310	0.23	+	.	ID=MALK_03024.t1;Parent=MALK_03024
contig010	AUGUSTUS	exon	683648	683876	.	+	.	ID=MALK_03024.t1.e1;Parent=MALK_03024.t1
contig010	AUGUSTUS	exon	683916	684310	.	+	.	ID=MALK_03024.t1.e2;Parent=MALK_03024.t1
contig010	AUGUSTUS	gene	684494	686095	0.71	+	.	ID=MALK_03025;prediction_source=augustus:contig010.g1319.t1
contig010	AUGUSTUS	CDS	684494	686095	0.71	+	0	ID=MALK_03025.t1.c1;Parent=MALK_03025.t1

contig010	AUGUSTUS	mRNA	684494	686095	0.71	+	.	ID=MALK_03025.t1;Parent=MALK_03025
contig010	AUGUSTUS	exon	684494	686095	0.71	+	.	ID=MALK_03025.t1.e1;Parent=MALK_03025.t1
contig010	AUGUSTUS	gene	686105	687214	0.95	-	.	ID=MALK_03026;prediction_source=braker_MRET:g1577.t1
contig010	AUGUSTUS	CDS	686105	687214	0.95	-	0	ID=MALK_03026.t1.c1;Parent=MALK_03026.t1
contig010	AUGUSTUS	mRNA	686105	687214	0.95	-	.	ID=MALK_03026.t1;Parent=MALK_03026
contig010	AUGUSTUS	exon	686105	687214	.	-	.	ID=MALK_03026.t1.e1;Parent=MALK_03026.t1
contig010	AUGUSTUS	gene	687354	688184	0.94	+	.	ID=MALK_03027;prediction_source=braker_MRET:g1578.t1
contig010	AUGUSTUS	CDS	687354	688184	0.94	+	0	ID=MALK_03027.t1.c1;Parent=MALK_03027.t1
contig010	AUGUSTUS	mRNA	687354	688184	0.94	+	.	ID=MALK_03027.t1;Parent=MALK_03027
contig010	AUGUSTUS	exon	687354	688184	.	+	.	ID=MALK_03027.t1.e1;Parent=MALK_03027.t1
contig010	AUGUSTUS	gene	688209	689054	0.42	-	.	ID=MALK_03028;prediction_source=braker_MRET:g1579.t1
contig010	AUGUSTUS	CDS	688209	689054	0.42	-	0	ID=MALK_03028.t1.c1;Parent=MALK_03028.t1
contig010	AUGUSTUS	mRNA	688209	689054	0.42	-	.	ID=MALK_03028.t1;Parent=MALK_03028
contig010	AUGUSTUS	exon	688209	689054	.	-	.	ID=MALK_03028.t1.e1;Parent=MALK_03028.t1
contig010	AUGUSTUS	gene	689117	692164	0.41	-	.	ID=MALK_03029;prediction_source=braker_MRET:g1580.t1
contig010	AUGUSTUS	CDS	689117	692164	0.41	-	0	ID=MALK_03029.t1.c1;Parent=MALK_03029.t1
contig010	AUGUSTUS	mRNA	689117	692164	0.41	-	.	ID=MALK_03029.t1;Parent=MALK_03029
contig010	AUGUSTUS	exon	689117	692164	.	-	.	ID=MALK_03029.t1.e1;Parent=MALK_03029.t1
contig010	AUGUSTUS	gene	692205	693821	0.99	-	.	ID=MALK_03030;prediction_source=braker_MRET:g1581.t1
contig010	AUGUSTUS	CDS	692205	693821	0.99	-	0	ID=MALK_03030.t1.c1;Parent=MALK_03030.t1
contig010	AUGUSTUS	mRNA	692205	693821	0.99	-	.	ID=MALK_03030.t1;Parent=MALK_03030
contig010	AUGUSTUS	exon	692205	693821	.	-	.	ID=MALK_03030.t1.e1;Parent=MALK_03030.t1
contig010	maker	gene	693951	694481	.	+	.	ID=MALK_03031;prediction_source=maker_MRET:augustus_masked-contig010-processed-gene-6.87-mRNA-1
contig010	maker	CDS	693951	694481	.	+	0	ID=MALK_03031.t1.c1;Parent=MALK_03031.t1
contig010	maker	mRNA	693951	694481	.	+	.	ID=MALK_03031.t1;Parent=MALK_03031
contig010	maker	exon	693951	694481	.	+	.	ID=MALK_03031.t1.e1;Parent=MALK_03031.t1
contig010	AUGUSTUS	gene	694478	695782	0.85	-	.	ID=MALK_03032;prediction_source=augustus:contig010.g1324.t1
contig010	AUGUSTUS	CDS	694478	695782	0.85	-	0	ID=MALK_03032.t1.c1;Parent=MALK_03032.t1
contig010	AUGUSTUS	mRNA	694478	695782	0.85	-	.	ID=MALK_03032.t1;Parent=MALK_03032
contig010	AUGUSTUS	exon	694478	695782	0.85	-	.	ID=MALK_03032.t1.e1;Parent=MALK_03032.t1
contig010	AUGUSTUS	gene	695751	698576	0.75	+	.	ID=MALK_03033;prediction_source=augustus:contig010.g1325.t1
contig010	AUGUSTUS	CDS	695751	698576	0.75	+	0	ID=MALK_03033.t1.c1;Parent=MALK_03033.t1
contig010	AUGUSTUS	mRNA	695751	698576	0.75	+	.	ID=MALK_03033.t1;Parent=MALK_03033
contig010	AUGUSTUS	exon	695751	698576	0.75	+	.	ID=MALK_03033.t1.e1;Parent=MALK_03033.t1
contig010	AUGUSTUS	gene	698754	699898	0.31	-	.	ID=MALK_03034;prediction_source=braker_MRET:g1585.t1
contig010	AUGUSTUS	CDS	698844	699898	0.36	-	0	ID=MALK_03034.t1.c2;Parent=MALK_03034.t1
contig010	AUGUSTUS	CDS	698754	698805	0.36	-	0	ID=MALK_03034.t1.c1;Parent=MALK_03034.t1
contig010	AUGUSTUS	mRNA	698754	699898	0.31	-	.	ID=MALK_03034.t1;Parent=MALK_03034
contig010	AUGUSTUS	exon	698844	699898	.	-	.	ID=MALK_03034.t1.e2;Parent=MALK_03034.t1
contig010	AUGUSTUS	exon	698754	698805	.	-	.	ID=MALK_03034.t1.e1;Parent=MALK_03034.t1
contig010	AUGUSTUS	gene	700008	700751	1	+	.	ID=MALK_03035;prediction_source=braker_MRET:g1586.t1
contig010	AUGUSTUS	CDS	700008	700751	1	+	0	ID=MALK_03035.t1.c1;Parent=MALK_03035.t1
contig010	AUGUSTUS	mRNA	700008	700751	1	+	.	ID=MALK_03035.t1;Parent=MALK_03035

contig010	AUGUSTUS	exon	700008	700751	.	+	.	ID=MALK_03035.t1.e1;Parent=MALK_03035.t1
contig010	AUGUSTUS	gene	700767	701486	0.98	-	.	ID=MALK_03036;prediction_source=braker_MRET:g1587.t1
contig010	AUGUSTUS	CDS	700767	701486	0.98	-	0	ID=MALK_03036.t1.c1;Parent=MALK_03036.t1
contig010	AUGUSTUS	mRNA	700767	701486	0.98	-	.	ID=MALK_03036.t1;Parent=MALK_03036
contig010	AUGUSTUS	exon	700767	701486	.	-	.	ID=MALK_03036.t1.e1;Parent=MALK_03036.t1
contig010	maker	gene	701548	703287	.	-	.	ID=MALK_03037;prediction_source=maker_MRET:augustus_masked-contig010-processed-gene-7.28-mRNA-1
contig010	maker	CDS	701548	703287	.	-	0	ID=MALK_03037.t1.c1;Parent=MALK_03037.t1
contig010	maker	mRNA	701548	703287	.	-	.	ID=MALK_03037.t1;Parent=MALK_03037
contig010	maker	exon	701548	703287	.	-	.	ID=MALK_03037.t1.e1;Parent=MALK_03037.t1
contig010	AUGUSTUS	gene	703290	704416	0.74	+	.	ID=MALK_03038;prediction_source=braker_MRET:g1589.t1
contig010	AUGUSTUS	CDS	703290	704116	0.75	+	0	ID=MALK_03038.t1.c1;Parent=MALK_03038.t1
contig010	AUGUSTUS	CDS	704155	704416	0.75	+	0	ID=MALK_03038.t1.c2;Parent=MALK_03038.t1
contig010	AUGUSTUS	mRNA	703290	704416	0.74	+	.	ID=MALK_03038.t1;Parent=MALK_03038
contig010	AUGUSTUS	exon	703290	704116	.	+	.	ID=MALK_03038.t1.e1;Parent=MALK_03038.t1
contig010	AUGUSTUS	exon	704155	704416	.	+	.	ID=MALK_03038.t1.e2;Parent=MALK_03038.t1
contig010	AUGUSTUS	gene	704498	705313	0.5	-	.	ID=MALK_03039;prediction_source=braker_MRET:g1590.t1
contig010	AUGUSTUS	CDS	704498	705313	0.5	-	0	ID=MALK_03039.t1.c1;Parent=MALK_03039.t1
contig010	AUGUSTUS	mRNA	704498	705313	0.5	-	.	ID=MALK_03039.t1;Parent=MALK_03039
contig010	AUGUSTUS	exon	704498	705313	.	-	.	ID=MALK_03039.t1.e1;Parent=MALK_03039.t1
contig010	AUGUSTUS	gene	705265	705792	0.89	+	.	ID=MALK_03040;prediction_source=augustus:contig010.g1329.t1
contig010	AUGUSTUS	CDS	705265	705792	0.89	+	0	ID=MALK_03040.t1.c1;Parent=MALK_03040.t1
contig010	AUGUSTUS	mRNA	705265	705792	0.89	+	.	ID=MALK_03040.t1;Parent=MALK_03040
contig010	AUGUSTUS	exon	705265	705792	0.89	+	.	ID=MALK_03040.t1.e1;Parent=MALK_03040.t1
contig010	AUGUSTUS	gene	705789	706817	0.97	-	.	ID=MALK_03041;prediction_source=braker_MRET:g1592.t1
contig010	AUGUSTUS	CDS	705789	706817	0.97	-	0	ID=MALK_03041.t1.c1;Parent=MALK_03041.t1
contig010	AUGUSTUS	mRNA	705789	706817	0.97	-	.	ID=MALK_03041.t1;Parent=MALK_03041
contig010	AUGUSTUS	exon	705789	706817	.	-	.	ID=MALK_03041.t1.e1;Parent=MALK_03041.t1
contig010	AUGUSTUS	gene	706895	707429	1	+	.	ID=MALK_03042;prediction_source=braker_MRET:g1593.t1
contig010	AUGUSTUS	CDS	706895	706951	1	+	0	ID=MALK_03042.t1.c1;Parent=MALK_03042.t1
contig010	AUGUSTUS	CDS	706986	707429	1	+	0	ID=MALK_03042.t1.c2;Parent=MALK_03042.t1
contig010	AUGUSTUS	mRNA	706895	707429	1	+	.	ID=MALK_03042.t1;Parent=MALK_03042
contig010	AUGUSTUS	exon	706895	706951	.	+	.	ID=MALK_03042.t1.e1;Parent=MALK_03042.t1
contig010	AUGUSTUS	exon	706986	707429	.	+	.	ID=MALK_03042.t1.e2;Parent=MALK_03042.t1
contig010	maker	gene	707446	708108	.	-	.	ID=MALK_03043;prediction_source=maker_MRET:augustus_masked-contig010-processed-gene-7.30-mRNA-1
contig010	maker	CDS	707446	708108	.	-	0	ID=MALK_03043.t1.c1;Parent=MALK_03043.t1
contig010	maker	mRNA	707446	708108	.	-	.	ID=MALK_03043.t1;Parent=MALK_03043
contig010	maker	exon	707446	708108	.	-	.	ID=MALK_03043.t1.e1;Parent=MALK_03043.t1
contig010	AUGUSTUS	gene	708306	709094	0.43	+	.	ID=MALK_03044;prediction_source=augustus:contig010.g1333.t1
contig010	AUGUSTUS	CDS	708306	709094	0.43	+	0	ID=MALK_03044.t1.c1;Parent=MALK_03044.t1
contig010	AUGUSTUS	mRNA	708306	709094	0.43	+	.	ID=MALK_03044.t1;Parent=MALK_03044
contig010	AUGUSTUS	exon	708306	709094	0.43	+	.	ID=MALK_03044.t1.e1;Parent=MALK_03044.t1
contig010	AUGUSTUS	gene	709099	710655	0.98	-	.	ID=MALK_03045;prediction_source=braker_MRET:g1596.t1
contig010	AUGUSTUS	CDS	709099	710655	0.98	-	0	ID=MALK_03045.t1.c1;Parent=MALK_03045.t1

contig010	AUGUSTUS	mRNA	709099	710655	0.98	-	.	ID=MALK_03045.t1;Parent=MALK_03045
contig010	AUGUSTUS	exon	709099	710655	.	-	.	ID=MALK_03045.t1.e1;Parent=MALK_03045.t1
contig010	AUGUSTUS	gene	710774	712126	0.97	+	.	ID=MALK_03046;prediction_source=braker_MRET:g1597.t1
contig010	AUGUSTUS	CDS	710774	712126	0.97	+	0	ID=MALK_03046.t1.c1;Parent=MALK_03046.t1
contig010	AUGUSTUS	mRNA	710774	712126	0.97	+	.	ID=MALK_03046.t1;Parent=MALK_03046
contig010	AUGUSTUS	exon	710774	712126	.	+	.	ID=MALK_03046.t1.e1;Parent=MALK_03046.t1
contig010	AUGUSTUS	gene	712161	712577	0.97	+	.	ID=MALK_03047;prediction_source=braker_MRET:g1598.t1
contig010	AUGUSTUS	CDS	712161	712577	0.97	+	0	ID=MALK_03047.t1.c1;Parent=MALK_03047.t1
contig010	AUGUSTUS	mRNA	712161	712577	0.97	+	.	ID=MALK_03047.t1;Parent=MALK_03047
contig010	AUGUSTUS	exon	712161	712577	.	+	.	ID=MALK_03047.t1.e1;Parent=MALK_03047.t1
contig010	AUGUSTUS	gene	713376	713735	0.41	-	.	ID=MALK_03048;prediction_source=augustus:contig010.g1336.t1
contig010	AUGUSTUS	CDS	713376	713735	0.41	-	0	ID=MALK_03048.t1.c1;Parent=MALK_03048.t1
contig010	AUGUSTUS	mRNA	713376	713735	0.41	-	.	ID=MALK_03048.t1;Parent=MALK_03048
contig010	AUGUSTUS	exon	713376	713735	0.41	-	.	ID=MALK_03048.t1.e1;Parent=MALK_03048.t1
contig010	AUGUSTUS	gene	713766	714602	0.99	-	.	ID=MALK_03049;prediction_source=braker_MRET:g1600.t1
contig010	AUGUSTUS	CDS	713766	714602	0.99	-	0	ID=MALK_03049.t1.c1;Parent=MALK_03049.t1
contig010	AUGUSTUS	mRNA	713766	714602	0.99	-	.	ID=MALK_03049.t1;Parent=MALK_03049
contig010	AUGUSTUS	exon	713766	714602	.	-	.	ID=MALK_03049.t1.e1;Parent=MALK_03049.t1
contig010	AUGUSTUS	gene	714682	715683	1	+	.	ID=MALK_03050;prediction_source=braker_MRET:g1601.t1
contig010	AUGUSTUS	CDS	714682	715683	1	+	0	ID=MALK_03050.t1.c1;Parent=MALK_03050.t1
contig010	AUGUSTUS	mRNA	714682	715683	1	+	.	ID=MALK_03050.t1;Parent=MALK_03050
contig010	AUGUSTUS	exon	714682	715683	.	+	.	ID=MALK_03050.t1.e1;Parent=MALK_03050.t1
contig010	AUGUSTUS	gene	715711	716525	0.56	+	.	ID=MALK_03051;prediction_source=braker_MRET:g1602.t1
contig010	AUGUSTUS	CDS	715711	716311	0.56	+	0	ID=MALK_03051.t1.c1;Parent=MALK_03051.t1
contig010	AUGUSTUS	CDS	716356	716525	0.56	+	0	ID=MALK_03051.t1.c2;Parent=MALK_03051.t1
contig010	AUGUSTUS	mRNA	715711	716525	0.56	+	.	ID=MALK_03051.t1;Parent=MALK_03051
contig010	AUGUSTUS	exon	715711	716311	.	+	.	ID=MALK_03051.t1.e1;Parent=MALK_03051.t1
contig010	AUGUSTUS	exon	716356	716525	.	+	.	ID=MALK_03051.t1.e2;Parent=MALK_03051.t1
contig010	AUGUSTUS	gene	716542	717864	0.71	-	.	ID=MALK_03052;prediction_source=braker_MRET:g1603.t1
contig010	AUGUSTUS	CDS	716542	717864	0.71	-	0	ID=MALK_03052.t1.c1;Parent=MALK_03052.t1
contig010	AUGUSTUS	mRNA	716542	717864	0.71	-	.	ID=MALK_03052.t1;Parent=MALK_03052
contig010	AUGUSTUS	exon	716542	717864	.	-	.	ID=MALK_03052.t1.e1;Parent=MALK_03052.t1
contig010	AUGUSTUS	gene	717918	718580	1	+	.	ID=MALK_03053;prediction_source=braker_MRET:g1604.t1
contig010	AUGUSTUS	CDS	717918	718580	1	+	0	ID=MALK_03053.t1.c1;Parent=MALK_03053.t1
contig010	AUGUSTUS	mRNA	717918	718580	1	+	.	ID=MALK_03053.t1;Parent=MALK_03053
contig010	AUGUSTUS	exon	717918	718580	.	+	.	ID=MALK_03053.t1.e1;Parent=MALK_03053.t1
contig010	AUGUSTUS	gene	718632	719509	0.92	-	.	ID=MALK_03054;prediction_source=braker_MRET:g1605.t1
contig010	AUGUSTUS	CDS	718688	719509	0.92	-	0	ID=MALK_03054.t1.c2;Parent=MALK_03054.t1
contig010	AUGUSTUS	CDS	718632	718634	0.92	-	0	ID=MALK_03054.t1.c1;Parent=MALK_03054.t1
contig010	AUGUSTUS	mRNA	718632	719509	0.92	-	.	ID=MALK_03054.t1;Parent=MALK_03054
contig010	AUGUSTUS	exon	718688	719509	.	-	.	ID=MALK_03054.t1.e2;Parent=MALK_03054.t1
contig010	AUGUSTUS	exon	718632	718634	.	-	.	ID=MALK_03054.t1.e1;Parent=MALK_03054.t1
contig010	AUGUSTUS	gene	719599	720337	0.18	+	.	ID=MALK_03055;prediction_source=braker_MRET:g1606.t1

contig010	AUGUSTUS	CDS	719599	719825	0.59	+	0	ID=MALK_03055.t1.c1;Parent=MALK_03055.t1
contig010	AUGUSTUS	CDS	719890	720238	0.59	+	0	ID=MALK_03055.t1.c2;Parent=MALK_03055.t1
contig010	AUGUSTUS	CDS	720275	720337	0.59	+	0	ID=MALK_03055.t1.c3;Parent=MALK_03055.t1
contig010	AUGUSTUS	mRNA	719599	720337	0.18	+	.	ID=MALK_03055.t1;Parent=MALK_03055
contig010	AUGUSTUS	exon	719599	719825	.	+	.	ID=MALK_03055.t1.e1;Parent=MALK_03055.t1
contig010	AUGUSTUS	exon	719890	720238	.	+	.	ID=MALK_03055.t1.e2;Parent=MALK_03055.t1
contig010	AUGUSTUS	exon	720275	720337	.	+	.	ID=MALK_03055.t1.e3;Parent=MALK_03055.t1
contig010	AUGUSTUS	gene	720578	721327	0.8	+	.	ID=MALK_03056;prediction_source=braker_MRET:g1607.t1
contig010	AUGUSTUS	CDS	720578	721327	0.8	+	0	ID=MALK_03056.t1.c1;Parent=MALK_03056.t1
contig010	AUGUSTUS	mRNA	720578	721327	0.8	+	.	ID=MALK_03056.t1;Parent=MALK_03056
contig010	AUGUSTUS	exon	720578	721327	.	+	.	ID=MALK_03056.t1.e1;Parent=MALK_03056.t1
contig010	AUGUSTUS	gene	721370	722806	0.94	+	.	ID=MALK_03057;prediction_source=braker_MRET:g1608.t1
contig010	AUGUSTUS	CDS	721370	722806	0.94	+	0	ID=MALK_03057.t1.c1;Parent=MALK_03057.t1
contig010	AUGUSTUS	mRNA	721370	722806	0.94	+	.	ID=MALK_03057.t1;Parent=MALK_03057
contig010	AUGUSTUS	exon	721370	722806	.	+	.	ID=MALK_03057.t1.e1;Parent=MALK_03057.t1
contig010	AUGUSTUS	gene	722938	725777	0.08	+	.	ID=MALK_03058;prediction_source=braker_MRET:g1609.t1
contig010	AUGUSTUS	CDS	722938	723483	0.91	+	0	ID=MALK_03058.t1.c1;Parent=MALK_03058.t1
contig010	AUGUSTUS	CDS	723656	725018	0.91	+	0	ID=MALK_03058.t1.c2;Parent=MALK_03058.t1
contig010	AUGUSTUS	CDS	725057	725175	0.91	+	0	ID=MALK_03058.t1.c3;Parent=MALK_03058.t1
contig010	AUGUSTUS	CDS	725292	725777	0.91	+	0	ID=MALK_03058.t1.c4;Parent=MALK_03058.t1
contig010	AUGUSTUS	mRNA	722938	725777	0.08	+	.	ID=MALK_03058.t1;Parent=MALK_03058
contig010	AUGUSTUS	exon	722938	723483	.	+	.	ID=MALK_03058.t1.e1;Parent=MALK_03058.t1
contig010	AUGUSTUS	exon	723656	725018	.	+	.	ID=MALK_03058.t1.e2;Parent=MALK_03058.t1
contig010	AUGUSTUS	exon	725057	725175	.	+	.	ID=MALK_03058.t1.e3;Parent=MALK_03058.t1
contig010	AUGUSTUS	exon	725292	725777	.	+	.	ID=MALK_03058.t1.e4;Parent=MALK_03058.t1
contig010	AUGUSTUS	gene	726378	727397	1	+	.	ID=MALK_03059;prediction_source=braker_MRET:g1610.t1
contig010	AUGUSTUS	CDS	726378	727397	1	+	0	ID=MALK_03059.t1.c1;Parent=MALK_03059.t1
contig010	AUGUSTUS	mRNA	726378	727397	1	+	.	ID=MALK_03059.t1;Parent=MALK_03059
contig010	AUGUSTUS	exon	726378	727397	.	+	.	ID=MALK_03059.t1.e1;Parent=MALK_03059.t1
contig010	AUGUSTUS	gene	727407	729401	0.99	-	.	ID=MALK_03060;prediction_source=braker_MRET:g1611.t1
contig010	AUGUSTUS	CDS	727407	729401	0.99	-	0	ID=MALK_03060.t1.c1;Parent=MALK_03060.t1
contig010	AUGUSTUS	mRNA	727407	729401	0.99	-	.	ID=MALK_03060.t1;Parent=MALK_03060
contig010	AUGUSTUS	exon	727407	729401	.	-	.	ID=MALK_03060.t1.e1;Parent=MALK_03060.t1
contig010	AUGUSTUS	gene	729446	731611	1	+	.	ID=MALK_03061;prediction_source=braker_MRET:g1612.t1
contig010	AUGUSTUS	CDS	729446	731611	1	+	0	ID=MALK_03061.t1.c1;Parent=MALK_03061.t1
contig010	AUGUSTUS	mRNA	729446	731611	1	+	.	ID=MALK_03061.t1;Parent=MALK_03061
contig010	AUGUSTUS	exon	729446	731611	.	+	.	ID=MALK_03061.t1.e1;Parent=MALK_03061.t1
contig010	AUGUSTUS	gene	731613	731936	1	-	.	ID=MALK_03062;prediction_source=braker_MRET:g1613.t1
contig010	AUGUSTUS	CDS	731912	731936	1	-	0	ID=MALK_03062.t1.c2;Parent=MALK_03062.t1
contig010	AUGUSTUS	CDS	731613	731878	1	-	0	ID=MALK_03062.t1.c1;Parent=MALK_03062.t1
contig010	AUGUSTUS	mRNA	731613	731936	1	-	.	ID=MALK_03062.t1;Parent=MALK_03062
contig010	AUGUSTUS	exon	731912	731936	.	-	.	ID=MALK_03062.t1.e2;Parent=MALK_03062.t1
contig010	AUGUSTUS	exon	731613	731878	.	-	.	ID=MALK_03062.t1.e1;Parent=MALK_03062.t1

contig010	AUGUSTUS	gene	732032	732748	0.56	+	.	ID=MALK_03063;prediction_source=braker_MRET:g1614.t1
contig010	AUGUSTUS	CDS	732032	732748	0.56	+	0	ID=MALK_03063.t1.c1;Parent=MALK_03063.t1
contig010	AUGUSTUS	mRNA	732032	732748	0.56	+	.	ID=MALK_03063.t1;Parent=MALK_03063
contig010	AUGUSTUS	exon	732032	732748	.	+	.	ID=MALK_03063.t1.e1;Parent=MALK_03063.t1
contig010	AUGUSTUS	gene	732787	733471	0.92	-	.	ID=MALK_03064;prediction_source=braker_MRET:g1615.t1
contig010	AUGUSTUS	CDS	732827	733471	0.92	-	0	ID=MALK_03064.t1.c2;Parent=MALK_03064.t1
contig010	AUGUSTUS	CDS	732787	732798	0.92	-	0	ID=MALK_03064.t1.c1;Parent=MALK_03064.t1
contig010	AUGUSTUS	mRNA	732787	733471	0.92	-	.	ID=MALK_03064.t1;Parent=MALK_03064
contig010	AUGUSTUS	exon	732827	733471	.	-	.	ID=MALK_03064.t1.e2;Parent=MALK_03064.t1
contig010	AUGUSTUS	exon	732787	732798	.	-	.	ID=MALK_03064.t1.e1;Parent=MALK_03064.t1
contig010	AUGUSTUS	gene	733601	734584	0.89	+	.	ID=MALK_03065;prediction_source=braker_MRET:g1616.t1
contig010	AUGUSTUS	CDS	733601	734584	0.89	+	0	ID=MALK_03065.t1.c1;Parent=MALK_03065.t1
contig010	AUGUSTUS	mRNA	733601	734584	0.89	+	.	ID=MALK_03065.t1;Parent=MALK_03065
contig010	AUGUSTUS	exon	733601	734584	.	+	.	ID=MALK_03065.t1.e1;Parent=MALK_03065.t1
contig010	AUGUSTUS	gene	734605	735396	0.44	+	.	ID=MALK_03066;prediction_source=augustus:contig010.g1344.t1
contig010	AUGUSTUS	CDS	734605	735396	0.44	+	0	ID=MALK_03066.t1.c1;Parent=MALK_03066.t1
contig010	AUGUSTUS	mRNA	734605	735396	0.44	+	.	ID=MALK_03066.t1;Parent=MALK_03066
contig010	AUGUSTUS	exon	734605	735396	0.44	+	.	ID=MALK_03066.t1.e1;Parent=MALK_03066.t1
contig010	AUGUSTUS	gene	737615	738712	0.57	-	.	ID=MALK_03067;prediction_source=braker_MRET:g1618.t1
contig010	AUGUSTUS	CDS	738614	738712	0.88	-	0	ID=MALK_03067.t1.c4;Parent=MALK_03067.t1
contig010	AUGUSTUS	CDS	738378	738575	0.88	-	0	ID=MALK_03067.t1.c3;Parent=MALK_03067.t1
contig010	AUGUSTUS	CDS	737676	738273	0.88	-	0	ID=MALK_03067.t1.c2;Parent=MALK_03067.t1
contig010	AUGUSTUS	CDS	737615	737637	0.88	-	0	ID=MALK_03067.t1.c1;Parent=MALK_03067.t1
contig010	AUGUSTUS	mRNA	737615	738712	0.57	-	.	ID=MALK_03067.t1;Parent=MALK_03067
contig010	AUGUSTUS	exon	738614	738712	.	-	.	ID=MALK_03067.t1.e4;Parent=MALK_03067.t1
contig010	AUGUSTUS	exon	738378	738575	.	-	.	ID=MALK_03067.t1.e3;Parent=MALK_03067.t1
contig010	AUGUSTUS	exon	737676	738273	.	-	.	ID=MALK_03067.t1.e2;Parent=MALK_03067.t1
contig010	AUGUSTUS	exon	737615	737637	.	-	.	ID=MALK_03067.t1.e1;Parent=MALK_03067.t1
contig010	maker	gene	738747	739502	.	+	.	ID=MALK_03068;prediction_source=maker_MRET:augustus_masked-contig010-processed-gene-7.9-mRNA-1
contig010	maker	CDS	738747	739502	.	+	0	ID=MALK_03068.t1.c1;Parent=MALK_03068.t1
contig010	maker	mRNA	738747	739502	.	+	.	ID=MALK_03068.t1;Parent=MALK_03068
contig010	maker	exon	738747	739502	.	+	.	ID=MALK_03068.t1.e1;Parent=MALK_03068.t1
contig010	maker	gene	740061	741975	.	+	.	ID=MALK_03069;prediction_source=maker_MRET:augustus_masked-contig010-processed-gene-7.10-mRNA-1
contig010	maker	CDS	740061	740131	.	+	0	ID=MALK_03069.t1.c1;Parent=MALK_03069.t1
contig010	maker	CDS	740214	741975	.	+	0	ID=MALK_03069.t1.c2;Parent=MALK_03069.t1
contig010	maker	mRNA	740061	741975	.	+	.	ID=MALK_03069.t1;Parent=MALK_03069
contig010	maker	exon	740061	740131	.	+	.	ID=MALK_03069.t1.e1;Parent=MALK_03069.t1
contig010	maker	exon	740214	741975	.	+	.	ID=MALK_03069.t1.e2;Parent=MALK_03069.t1
contig010	AUGUSTUS	gene	742192	742771	0.23	+	.	ID=MALK_03070;prediction_source=braker_MRET:g1621.t1
contig010	AUGUSTUS	CDS	742192	742682	0.49	+	0	ID=MALK_03070.t1.c1;Parent=MALK_03070.t1
contig010	AUGUSTUS	CDS	742735	742771	0.49	+	0	ID=MALK_03070.t1.c2;Parent=MALK_03070.t1
contig010	AUGUSTUS	mRNA	742192	742771	0.23	+	.	ID=MALK_03070.t1;Parent=MALK_03070
contig010	AUGUSTUS	exon	742192	742682	.	+	.	ID=MALK_03070.t1.e1;Parent=MALK_03070.t1

contig010	AUGUSTUS	exon	742735	742771	.	+	.	ID=MALK_03070.t1.e2;Parent=MALK_03070.t1
contig010	maker	gene	742872	743894	.	-	.	ID=MALK_03071;prediction_source=maker_MRET:augustus_masked-contig010-processed-gene-7.33-mRNA-1
contig010	maker	CDS	742872	743894	.	-	0	ID=MALK_03071.t1.c1;Parent=MALK_03071.t1
contig010	maker	mRNA	742872	743894	.	-	.	ID=MALK_03071.t1;Parent=MALK_03071
contig010	maker	exon	742872	743894	.	-	.	ID=MALK_03071.t1.e1;Parent=MALK_03071.t1
contig010	maker	gene	743960	745714	.	+	.	ID=MALK_03072;prediction_source=maker_MRET:augustus_masked-contig010-processed-gene-7.11-mRNA-1
contig010	maker	CDS	743960	745714	.	+	0	ID=MALK_03072.t1.c1;Parent=MALK_03072.t1
contig010	maker	mRNA	743960	745714	.	+	.	ID=MALK_03072.t1;Parent=MALK_03072
contig010	maker	exon	743960	745714	.	+	.	ID=MALK_03072.t1.e1;Parent=MALK_03072.t1
contig010	AUGUSTUS	gene	745746	746645	0.6	+	.	ID=MALK_03073;prediction_source=augustus:contig010.g1350.t1
contig010	AUGUSTUS	CDS	745746	746645	0.6	+	0	ID=MALK_03073.t1.c1;Parent=MALK_03073.t1
contig010	AUGUSTUS	mRNA	745746	746645	0.6	+	.	ID=MALK_03073.t1;Parent=MALK_03073
contig010	AUGUSTUS	exon	745746	746645	0.6	+	.	ID=MALK_03073.t1.e1;Parent=MALK_03073.t1
contig010	maker	gene	746642	747658	.	-	.	ID=MALK_03074;prediction_source=maker_MRET:augustus_masked-contig010-processed-gene-7.34-mRNA-1
contig010	maker	CDS	746642	747658	.	-	0	ID=MALK_03074.t1.c1;Parent=MALK_03074.t1
contig010	maker	mRNA	746642	747658	.	-	.	ID=MALK_03074.t1;Parent=MALK_03074
contig010	maker	exon	746642	747658	.	-	.	ID=MALK_03074.t1.e1;Parent=MALK_03074.t1
contig010	AUGUSTUS	gene	747681	749213	0.81	+	.	ID=MALK_03075;prediction_source=augustus:contig010.g1352.t1
contig010	AUGUSTUS	CDS	747681	749213	0.81	+	0	ID=MALK_03075.t1.c1;Parent=MALK_03075.t1
contig010	AUGUSTUS	mRNA	747681	749213	0.81	+	.	ID=MALK_03075.t1;Parent=MALK_03075
contig010	AUGUSTUS	exon	747681	749213	0.81	+	.	ID=MALK_03075.t1.e1;Parent=MALK_03075.t1
contig010	AUGUSTUS	gene	749251	750081	1	+	.	ID=MALK_03076;prediction_source=braker_MRET:g1627.t1
contig010	AUGUSTUS	CDS	749251	750081	1	+	0	ID=MALK_03076.t1.c1;Parent=MALK_03076.t1
contig010	AUGUSTUS	mRNA	749251	750081	1	+	.	ID=MALK_03076.t1;Parent=MALK_03076
contig010	AUGUSTUS	exon	749251	750081	.	+	.	ID=MALK_03076.t1.e1;Parent=MALK_03076.t1
contig010	AUGUSTUS	gene	750215	751693	0.73	-	.	ID=MALK_03077;prediction_source=braker_MRET:g1628.t1
contig010	AUGUSTUS	CDS	750215	751693	0.73	-	0	ID=MALK_03077.t1.c1;Parent=MALK_03077.t1
contig010	AUGUSTUS	mRNA	750215	751693	0.73	-	.	ID=MALK_03077.t1;Parent=MALK_03077
contig010	AUGUSTUS	exon	750215	751693	.	-	.	ID=MALK_03077.t1.e1;Parent=MALK_03077.t1
contig010	AUGUSTUS	gene	751927	753848	0.22	+	.	ID=MALK_03078;prediction_source=braker_MRET:g1629.t1
contig010	AUGUSTUS	CDS	751927	753372	0.48	+	0	ID=MALK_03078.t1.c1;Parent=MALK_03078.t1
contig010	AUGUSTUS	CDS	753498	753848	0.48	+	0	ID=MALK_03078.t1.c2;Parent=MALK_03078.t1
contig010	AUGUSTUS	mRNA	751927	753848	0.22	+	.	ID=MALK_03078.t1;Parent=MALK_03078
contig010	AUGUSTUS	exon	751927	753372	.	+	.	ID=MALK_03078.t1.e1;Parent=MALK_03078.t1
contig010	AUGUSTUS	exon	753498	753848	.	+	.	ID=MALK_03078.t1.e2;Parent=MALK_03078.t1
contig010	AUGUSTUS	gene	753885	754865	0.06	-	.	ID=MALK_03079;prediction_source=braker_MRET:g1630.t1
contig010	AUGUSTUS	CDS	754816	754865	0.09	-	0	ID=MALK_03079.t1.c3;Parent=MALK_03079.t1
contig010	AUGUSTUS	CDS	753926	754616	0.09	-	0	ID=MALK_03079.t1.c2;Parent=MALK_03079.t1
contig010	AUGUSTUS	CDS	753885	753887	0.09	-	0	ID=MALK_03079.t1.c1;Parent=MALK_03079.t1
contig010	AUGUSTUS	mRNA	753885	754865	0.06	-	.	ID=MALK_03079.t1;Parent=MALK_03079
contig010	AUGUSTUS	exon	754816	754865	.	-	.	ID=MALK_03079.t1.e3;Parent=MALK_03079.t1
contig010	AUGUSTUS	exon	753926	754616	.	-	.	ID=MALK_03079.t1.e2;Parent=MALK_03079.t1
contig010	AUGUSTUS	exon	753885	753887	.	-	.	ID=MALK_03079.t1.e1;Parent=MALK_03079.t1

contig010	AUGUSTUS	gene	755792	756692	0.22	+	.	ID=MALK_03080;prediction_source=augustus:contig010.g1356.t1
contig010	AUGUSTUS	CDS	755792	756319	0.28	+	0	ID=MALK_03080.t1.c1;Parent=MALK_03080.t1
contig010	AUGUSTUS	CDS	756435	756692	0.28	+	0	ID=MALK_03080.t1.c2;Parent=MALK_03080.t1
contig010	AUGUSTUS	mRNA	755792	756692	0.22	+	.	ID=MALK_03080.t1;Parent=MALK_03080
contig010	AUGUSTUS	exon	755792	756319	0.28	+	.	ID=MALK_03080.t1.e1;Parent=MALK_03080.t1
contig010	AUGUSTUS	exon	756435	756692	0.28	+	.	ID=MALK_03080.t1.e2;Parent=MALK_03080.t1
contig010	AUGUSTUS	gene	756819	757814	0.93	+	.	ID=MALK_03081;prediction_source=augustus:contig010.g1357.t1
contig010	AUGUSTUS	CDS	756819	757814	0.93	+	0	ID=MALK_03081.t1.c1;Parent=MALK_03081.t1
contig010	AUGUSTUS	mRNA	756819	757814	0.93	+	.	ID=MALK_03081.t1;Parent=MALK_03081
contig010	AUGUSTUS	exon	756819	757814	0.93	+	.	ID=MALK_03081.t1.e1;Parent=MALK_03081.t1
contig010	maker	gene	757815	759467	.	-	.	ID=MALK_03082;prediction_source=maker_MRET:augustus_masked-contig010-processed-gene-7.37-mRNA-1
contig010	maker	CDS	757815	759467	.	-	0	ID=MALK_03082.t1.c1;Parent=MALK_03082.t1
contig010	maker	mRNA	757815	759467	.	-	.	ID=MALK_03082.t1;Parent=MALK_03082
contig010	maker	exon	757815	759467	.	-	.	ID=MALK_03082.t1.e1;Parent=MALK_03082.t1
contig010	AUGUSTUS	gene	759655	761248	0.97	+	.	ID=MALK_03083;prediction_source=braker_MRET:g1634.t1
contig010	AUGUSTUS	CDS	759655	760705	0.99	+	0	ID=MALK_03083.t1.c1;Parent=MALK_03083.t1
contig010	AUGUSTUS	CDS	760740	761248	0.99	+	0	ID=MALK_03083.t1.c2;Parent=MALK_03083.t1
contig010	AUGUSTUS	mRNA	759655	761248	0.97	+	.	ID=MALK_03083.t1;Parent=MALK_03083
contig010	AUGUSTUS	exon	759655	760705	.	+	.	ID=MALK_03083.t1.e1;Parent=MALK_03083.t1
contig010	AUGUSTUS	exon	760740	761248	.	+	.	ID=MALK_03083.t1.e2;Parent=MALK_03083.t1
contig010	AUGUSTUS	gene	762086	764089	0.63	-	.	ID=MALK_03084;prediction_source=braker_MRET:g1635.t1
contig010	AUGUSTUS	CDS	762086	764089	0.63	-	0	ID=MALK_03084.t1.c1;Parent=MALK_03084.t1
contig010	AUGUSTUS	mRNA	762086	764089	0.63	-	.	ID=MALK_03084.t1;Parent=MALK_03084
contig010	AUGUSTUS	exon	762086	764089	.	-	.	ID=MALK_03084.t1.e1;Parent=MALK_03084.t1
contig010	maker	gene	764243	765922	.	-	.	ID=MALK_03085;prediction_source=maker_MRET:augustus_masked-contig010-processed-gene-7.39-mRNA-1
contig010	maker	CDS	764243	765922	.	-	0	ID=MALK_03085.t1.c1;Parent=MALK_03085.t1
contig010	maker	mRNA	764243	765922	.	-	.	ID=MALK_03085.t1;Parent=MALK_03085
contig010	maker	exon	764243	765922	.	-	.	ID=MALK_03085.t1.e1;Parent=MALK_03085.t1
contig010	AUGUSTUS	gene	766062	767978	0.94	-	.	ID=MALK_03086;prediction_source=augustus:contig010.g1363.t1
contig010	AUGUSTUS	CDS	766062	767978	0.94	-	0	ID=MALK_03086.t1.c1;Parent=MALK_03086.t1
contig010	AUGUSTUS	mRNA	766062	767978	0.94	-	.	ID=MALK_03086.t1;Parent=MALK_03086
contig010	AUGUSTUS	exon	766062	767978	0.94	-	.	ID=MALK_03086.t1.e1;Parent=MALK_03086.t1
contig010	AUGUSTUS	gene	768622	769719	0.85	+	.	ID=MALK_03087;prediction_source=augustus:contig010.g1364.t1
contig010	AUGUSTUS	CDS	768622	769719	0.85	+	0	ID=MALK_03087.t1.c1;Parent=MALK_03087.t1
contig010	AUGUSTUS	mRNA	768622	769719	0.85	+	.	ID=MALK_03087.t1;Parent=MALK_03087
contig010	AUGUSTUS	exon	768622	769719	0.85	+	.	ID=MALK_03087.t1.e1;Parent=MALK_03087.t1
contig010	AUGUSTUS	gene	769716	771674	0.32	-	.	ID=MALK_03088;prediction_source=augustus:contig010.g1365.t1
contig010	AUGUSTUS	CDS	769716	771674	0.32	-	0	ID=MALK_03088.t1.c1;Parent=MALK_03088.t1
contig010	AUGUSTUS	mRNA	769716	771674	0.32	-	.	ID=MALK_03088.t1;Parent=MALK_03088
contig010	AUGUSTUS	exon	769716	771674	0.32	-	.	ID=MALK_03088.t1.e1;Parent=MALK_03088.t1
contig010	AUGUSTUS	gene	771746	773338	0.46	-	.	ID=MALK_03089;prediction_source=augustus:contig010.g1366.t1
contig010	AUGUSTUS	CDS	771746	773338	0.46	-	0	ID=MALK_03089.t1.c1;Parent=MALK_03089.t1
contig010	AUGUSTUS	mRNA	771746	773338	0.46	-	.	ID=MALK_03089.t1;Parent=MALK_03089

contig010	AUGUSTUS	exon	771746	773338	0.46	-	.	ID=MALK_03089.t1.e1;Parent=MALK_03089.t1
contig010	AUGUSTUS	gene	773381	774037	0.33	+	.	ID=MALK_03090;prediction_source=braker_MRET:g1641.t1
contig010	AUGUSTUS	CDS	773381	773669	0.51	+	0	ID=MALK_03090.t1.c1;Parent=MALK_03090.t1
contig010	AUGUSTUS	CDS	773760	774037	0.51	+	0	ID=MALK_03090.t1.c2;Parent=MALK_03090.t1
contig010	AUGUSTUS	mRNA	773381	774037	0.33	+	.	ID=MALK_03090.t1;Parent=MALK_03090
contig010	AUGUSTUS	exon	773381	773669	.	+	.	ID=MALK_03090.t1.e1;Parent=MALK_03090.t1
contig010	AUGUSTUS	exon	773760	774037	.	+	.	ID=MALK_03090.t1.e2;Parent=MALK_03090.t1
contig010	AUGUSTUS	gene	774059	776515	0.86	+	.	ID=MALK_03091;prediction_source=braker_MRET:g1642.t1
contig010	AUGUSTUS	CDS	774059	776515	0.86	+	0	ID=MALK_03091.t1.c1;Parent=MALK_03091.t1
contig010	AUGUSTUS	mRNA	774059	776515	0.86	+	.	ID=MALK_03091.t1;Parent=MALK_03091
contig010	AUGUSTUS	exon	774059	776515	.	+	.	ID=MALK_03091.t1.e1;Parent=MALK_03091.t1
contig010	AUGUSTUS	gene	776558	778157	0.68	+	.	ID=MALK_03092;prediction_source=braker_MRET:g1643.t1
contig010	AUGUSTUS	CDS	776558	778056	0.77	+	0	ID=MALK_03092.t1.c1;Parent=MALK_03092.t1
contig010	AUGUSTUS	CDS	778100	778157	0.77	+	0	ID=MALK_03092.t1.c2;Parent=MALK_03092.t1
contig010	AUGUSTUS	mRNA	776558	778157	0.68	+	.	ID=MALK_03092.t1;Parent=MALK_03092
contig010	AUGUSTUS	exon	776558	778056	.	+	.	ID=MALK_03092.t1.e1;Parent=MALK_03092.t1
contig010	AUGUSTUS	exon	778100	778157	.	+	.	ID=MALK_03092.t1.e2;Parent=MALK_03092.t1
contig010	AUGUSTUS	gene	778309	779520	0.75	-	.	ID=MALK_03093;prediction_source=braker_MRET:g1644.t1
contig010	AUGUSTUS	CDS	778309	779520	0.75	-	0	ID=MALK_03093.t1.c1;Parent=MALK_03093.t1
contig010	AUGUSTUS	mRNA	778309	779520	0.75	-	.	ID=MALK_03093.t1;Parent=MALK_03093
contig010	AUGUSTUS	exon	778309	779520	.	-	.	ID=MALK_03093.t1.e1;Parent=MALK_03093.t1
contig010	AUGUSTUS	gene	779703	780947	0.56	-	.	ID=MALK_03094;prediction_source=braker_MRET:g1645.t1
contig010	AUGUSTUS	CDS	779892	780947	0.6	-	0	ID=MALK_03094.t1.c2;Parent=MALK_03094.t1
contig010	AUGUSTUS	CDS	779703	779855	0.6	-	0	ID=MALK_03094.t1.c1;Parent=MALK_03094.t1
contig010	AUGUSTUS	mRNA	779703	780947	0.56	-	.	ID=MALK_03094.t1;Parent=MALK_03094
contig010	AUGUSTUS	exon	779892	780947	.	-	.	ID=MALK_03094.t1.e2;Parent=MALK_03094.t1
contig010	AUGUSTUS	exon	779703	779855	.	-	.	ID=MALK_03094.t1.e1;Parent=MALK_03094.t1
contig010	AUGUSTUS	gene	781040	782398	1	-	.	ID=MALK_03095;prediction_source=braker_MRET:g1646.t1
contig010	AUGUSTUS	CDS	781040	782398	1	-	0	ID=MALK_03095.t1.c1;Parent=MALK_03095.t1
contig010	AUGUSTUS	mRNA	781040	782398	1	-	.	ID=MALK_03095.t1;Parent=MALK_03095
contig010	AUGUSTUS	exon	781040	782398	.	-	.	ID=MALK_03095.t1.e1;Parent=MALK_03095.t1
contig010	AUGUSTUS	gene	782515	783319	0.47	+	.	ID=MALK_03096;prediction_source=braker_MRET:g1647.t1
contig010	AUGUSTUS	CDS	782515	783194	0.49	+	0	ID=MALK_03096.t1.c1;Parent=MALK_03096.t1
contig010	AUGUSTUS	CDS	783295	783319	0.49	+	0	ID=MALK_03096.t1.c2;Parent=MALK_03096.t1
contig010	AUGUSTUS	mRNA	782515	783319	0.47	+	.	ID=MALK_03096.t1;Parent=MALK_03096
contig010	AUGUSTUS	exon	782515	783194	.	+	.	ID=MALK_03096.t1.e1;Parent=MALK_03096.t1
contig010	AUGUSTUS	exon	783295	783319	.	+	.	ID=MALK_03096.t1.e2;Parent=MALK_03096.t1
contig010	AUGUSTUS	gene	783370	785010	0.52	-	.	ID=MALK_03097;prediction_source=braker_MRET:g1648.t1
contig010	AUGUSTUS	CDS	783370	785010	0.52	-	0	ID=MALK_03097.t1.c1;Parent=MALK_03097.t1
contig010	AUGUSTUS	mRNA	783370	785010	0.52	-	.	ID=MALK_03097.t1;Parent=MALK_03097
contig010	AUGUSTUS	exon	783370	785010	.	-	.	ID=MALK_03097.t1.e1;Parent=MALK_03097.t1
contig010	AUGUSTUS	gene	785752	788037	0.71	+	.	ID=MALK_03098;prediction_source=augustus:contig010.g1369.t1
contig010	AUGUSTUS	CDS	785752	788037	0.71	+	0	ID=MALK_03098.t1.c1;Parent=MALK_03098.t1

contig010	AUGUSTUS	mRNA	785752	788037	0.71	+	.	ID=MALK_03098.t1;Parent=MALK_03098
contig010	AUGUSTUS	exon	785752	788037	0.71	+	.	ID=MALK_03098.t1.e1;Parent=MALK_03098.t1
contig010	AUGUSTUS	gene	788034	788561	0.69	-	.	ID=MALK_03099;prediction_source=augustus:contig010.g1370.t1
contig010	AUGUSTUS	CDS	788034	788561	0.69	-	0	ID=MALK_03099.t1.c1;Parent=MALK_03099.t1
contig010	AUGUSTUS	mRNA	788034	788561	0.69	-	.	ID=MALK_03099.t1;Parent=MALK_03099
contig010	AUGUSTUS	exon	788034	788561	0.69	-	.	ID=MALK_03099.t1.e1;Parent=MALK_03099.t1
contig010	AUGUSTUS	gene	788616	789527	0.89	-	.	ID=MALK_03100;prediction_source=braker_MRET:g1650.t1
contig010	AUGUSTUS	CDS	788616	789527	0.89	-	0	ID=MALK_03100.t1.c1;Parent=MALK_03100.t1
contig010	AUGUSTUS	mRNA	788616	789527	0.89	-	.	ID=MALK_03100.t1;Parent=MALK_03100
contig010	AUGUSTUS	exon	788616	789527	.	-	.	ID=MALK_03100.t1.e1;Parent=MALK_03100.t1
contig010	maker	gene	789610	791507	.	-	.	ID=MALK_03101;prediction_source=maker_MRET:augustus_masked-contig010-processed-gene-7.43-mRNA-1
contig010	maker	CDS	790770	791507	.	-	0	ID=MALK_03101.t1.c1;Parent=MALK_03101.t1
contig010	maker	CDS	789610	790698	.	-	0	ID=MALK_03101.t1.c2;Parent=MALK_03101.t1
contig010	maker	mRNA	789610	791507	.	-	.	ID=MALK_03101.t1;Parent=MALK_03101
contig010	maker	exon	790770	791507	.	-	.	ID=MALK_03101.t1.e1;Parent=MALK_03101.t1
contig010	maker	exon	789610	790698	.	-	.	ID=MALK_03101.t1.e2;Parent=MALK_03101.t1
contig010	AUGUSTUS	gene	791865	794249	0.73	+	.	ID=MALK_03102;prediction_source=braker_MRET:g1652.t1
contig010	AUGUSTUS	CDS	791865	793412	0.78	+	0	ID=MALK_03102.t1.c1;Parent=MALK_03102.t1
contig010	AUGUSTUS	CDS	793614	794249	0.78	+	0	ID=MALK_03102.t1.c2;Parent=MALK_03102.t1
contig010	AUGUSTUS	mRNA	791865	794249	0.73	+	.	ID=MALK_03102.t1;Parent=MALK_03102
contig010	AUGUSTUS	exon	791865	793412	.	+	.	ID=MALK_03102.t1.e1;Parent=MALK_03102.t1
contig010	AUGUSTUS	exon	793614	794249	.	+	.	ID=MALK_03102.t1.e2;Parent=MALK_03102.t1
contig010	AUGUSTUS	gene	794323	794881	0.7	-	.	ID=MALK_03103;prediction_source=braker_MRET:g1653.t1
contig010	AUGUSTUS	CDS	794485	794881	0.73	-	0	ID=MALK_03103.t1.c2;Parent=MALK_03103.t1
contig010	AUGUSTUS	CDS	794323	794444	0.73	-	0	ID=MALK_03103.t1.c1;Parent=MALK_03103.t1
contig010	AUGUSTUS	mRNA	794323	794881	0.7	-	.	ID=MALK_03103.t1;Parent=MALK_03103
contig010	AUGUSTUS	exon	794485	794881	.	-	.	ID=MALK_03103.t1.e2;Parent=MALK_03103.t1
contig010	AUGUSTUS	exon	794323	794444	.	-	.	ID=MALK_03103.t1.e1;Parent=MALK_03103.t1
contig010	AUGUSTUS	gene	794950	796257	0.99	+	.	ID=MALK_03104;prediction_source=augustus:contig010.g1373.t1
contig010	AUGUSTUS	CDS	794950	796257	0.99	+	0	ID=MALK_03104.t1.c1;Parent=MALK_03104.t1
contig010	AUGUSTUS	mRNA	794950	796257	0.99	+	.	ID=MALK_03104.t1;Parent=MALK_03104
contig010	AUGUSTUS	exon	794950	796257	0.99	+	.	ID=MALK_03104.t1.e1;Parent=MALK_03104.t1
contig010	maker	gene	796259	797212	.	-	.	ID=MALK_03105;prediction_source=maker_MRET:augustus_masked-contig010-processed-gene-7.44-mRNA-1
contig010	maker	CDS	796259	797212	.	-	0	ID=MALK_03105.t1.c1;Parent=MALK_03105.t1
contig010	maker	mRNA	796259	797212	.	-	.	ID=MALK_03105.t1;Parent=MALK_03105
contig010	maker	exon	796259	797212	.	-	.	ID=MALK_03105.t1.e1;Parent=MALK_03105.t1
contig010	maker	gene	797292	797552	.	+	.	ID=MALK_03106;prediction_source=maker_MRET:augustus_masked-contig010-processed-gene-7.20-mRNA-1
contig010	maker	CDS	797292	797552	.	+	0	ID=MALK_03106.t1.c1;Parent=MALK_03106.t1
contig010	maker	mRNA	797292	797552	.	+	.	ID=MALK_03106.t1;Parent=MALK_03106
contig010	maker	exon	797292	797552	.	+	.	ID=MALK_03106.t1.e1;Parent=MALK_03106.t1
contig010	AUGUSTUS	gene	797575	798126	0.54	-	.	ID=MALK_03107;prediction_source=braker_MRET:g1657.t1
contig010	AUGUSTUS	CDS	797575	798126	0.54	-	0	ID=MALK_03107.t1.c1;Parent=MALK_03107.t1
contig010	AUGUSTUS	mRNA	797575	798126	0.54	-	.	ID=MALK_03107.t1;Parent=MALK_03107

contig010	AUGUSTUS	exon	797575	798126	.	-	.	ID=MALK_03107.t1.e1;Parent=MALK_03107.t1
contig010	AUGUSTUS	gene	798179	798650	0.3	+	.	ID=MALK_03108;prediction_source=braker_MRET:g1658.t1
contig010	AUGUSTUS	CDS	798179	798382	0.6	+	0	ID=MALK_03108.t1.c1;Parent=MALK_03108.t1
contig010	AUGUSTUS	CDS	798498	798650	0.6	+	0	ID=MALK_03108.t1.c2;Parent=MALK_03108.t1
contig010	AUGUSTUS	mRNA	798179	798650	0.3	+	.	ID=MALK_03108.t1;Parent=MALK_03108
contig010	AUGUSTUS	exon	798179	798382	.	+	.	ID=MALK_03108.t1.e1;Parent=MALK_03108.t1
contig010	AUGUSTUS	exon	798498	798650	.	+	.	ID=MALK_03108.t1.e2;Parent=MALK_03108.t1
contig010	AUGUSTUS	gene	798972	799643	0.78	-	.	ID=MALK_03109;prediction_source=augustus:contig010.g1376.t1
contig010	AUGUSTUS	CDS	798972	799643	0.78	-	0	ID=MALK_03109.t1.c1;Parent=MALK_03109.t1
contig010	AUGUSTUS	mRNA	798972	799643	0.78	-	.	ID=MALK_03109.t1;Parent=MALK_03109
contig010	AUGUSTUS	exon	798972	799643	0.78	-	.	ID=MALK_03109.t1.e1;Parent=MALK_03109.t1
contig010	AUGUSTUS	gene	799742	801754	1	+	.	ID=MALK_03110;prediction_source=augustus:contig010.g1377.t1
contig010	AUGUSTUS	CDS	799742	801754	1	+	0	ID=MALK_03110.t1.c1;Parent=MALK_03110.t1
contig010	AUGUSTUS	mRNA	799742	801754	1	+	.	ID=MALK_03110.t1;Parent=MALK_03110
contig010	AUGUSTUS	exon	799742	801754	1	+	.	ID=MALK_03110.t1.e1;Parent=MALK_03110.t1
contig010	AUGUSTUS	gene	802149	802574	0.43	-	.	ID=MALK_03111;prediction_source=augustus:contig010.g1378.t1
contig010	AUGUSTUS	CDS	802149	802574	0.43	-	0	ID=MALK_03111.t1.c1;Parent=MALK_03111.t1
contig010	AUGUSTUS	mRNA	802149	802574	0.43	-	.	ID=MALK_03111.t1;Parent=MALK_03111
contig010	AUGUSTUS	exon	802149	802574	0.43	-	.	ID=MALK_03111.t1.e1;Parent=MALK_03111.t1
contig010	AUGUSTUS	gene	802736	803404	0.31	+	.	ID=MALK_03112;prediction_source=augustus:contig010.g1379.t1
contig010	AUGUSTUS	CDS	802736	803404	0.31	+	0	ID=MALK_03112.t1.c1;Parent=MALK_03112.t1
contig010	AUGUSTUS	mRNA	802736	803404	0.31	+	.	ID=MALK_03112.t1;Parent=MALK_03112
contig010	AUGUSTUS	exon	802736	803404	0.31	+	.	ID=MALK_03112.t1.e1;Parent=MALK_03112.t1
contig010	AUGUSTUS	gene	803422	804543	0.91	-	.	ID=MALK_03113;prediction_source=braker_MRET:g1663.t1
contig010	AUGUSTUS	CDS	803422	804543	0.91	-	0	ID=MALK_03113.t1.c1;Parent=MALK_03113.t1
contig010	AUGUSTUS	mRNA	803422	804543	0.91	-	.	ID=MALK_03113.t1;Parent=MALK_03113
contig010	AUGUSTUS	exon	803422	804543	.	-	.	ID=MALK_03113.t1.e1;Parent=MALK_03113.t1
contig010	AUGUSTUS	gene	804711	805376	0.93	-	.	ID=MALK_03114;prediction_source=braker_MRET:g1664.t1
contig010	AUGUSTUS	CDS	804711	805376	0.93	-	0	ID=MALK_03114.t1.c1;Parent=MALK_03114.t1
contig010	AUGUSTUS	mRNA	804711	805376	0.93	-	.	ID=MALK_03114.t1;Parent=MALK_03114
contig010	AUGUSTUS	exon	804711	805376	.	-	.	ID=MALK_03114.t1.e1;Parent=MALK_03114.t1
contig010	AUGUSTUS	gene	805524	806888	0.75	+	.	ID=MALK_03115;prediction_source=augustus:contig010.g1382.t1
contig010	AUGUSTUS	CDS	805524	806888	0.75	+	0	ID=MALK_03115.t1.c1;Parent=MALK_03115.t1
contig010	AUGUSTUS	mRNA	805524	806888	0.75	+	.	ID=MALK_03115.t1;Parent=MALK_03115
contig010	AUGUSTUS	exon	805524	806888	0.75	+	.	ID=MALK_03115.t1.e1;Parent=MALK_03115.t1
contig010	AUGUSTUS	gene	807248	808618	0.48	-	.	ID=MALK_03116;prediction_source=braker_MRET:g1666.t1
contig010	AUGUSTUS	CDS	807248	808618	0.48	-	0	ID=MALK_03116.t1.c1;Parent=MALK_03116.t1
contig010	AUGUSTUS	mRNA	807248	808618	0.48	-	.	ID=MALK_03116.t1;Parent=MALK_03116
contig010	AUGUSTUS	exon	807248	808618	.	-	.	ID=MALK_03116.t1.e1;Parent=MALK_03116.t1
contig010	AUGUSTUS	gene	808728	809123	1	+	.	ID=MALK_03117;prediction_source=braker_MRET:g1667.t1
contig010	AUGUSTUS	CDS	808728	809123	1	+	0	ID=MALK_03117.t1.c1;Parent=MALK_03117.t1
contig010	AUGUSTUS	mRNA	808728	809123	1	+	.	ID=MALK_03117.t1;Parent=MALK_03117
contig010	AUGUSTUS	exon	808728	809123	.	+	.	ID=MALK_03117.t1.e1;Parent=MALK_03117.t1

contig010	AUGUSTUS	gene	809132	810484	0.8	-	.	ID=MALK_03118;prediction_source=augustus:contig010.g1384.t1
contig010	AUGUSTUS	CDS	809132	810484	0.8	-	0	ID=MALK_03118.t1.c1;Parent=MALK_03118.t1
contig010	AUGUSTUS	mRNA	809132	810484	0.8	-	.	ID=MALK_03118.t1;Parent=MALK_03118
contig010	AUGUSTUS	exon	809132	810484	0.8	-	.	ID=MALK_03118.t1.e1;Parent=MALK_03118.t1
contig010	AUGUSTUS	gene	811158	812450	1	+	.	ID=MALK_03119;prediction_source=augustus:contig010.g1385.t1
contig010	AUGUSTUS	CDS	811158	812450	1	+	0	ID=MALK_03119.t1.c1;Parent=MALK_03119.t1
contig010	AUGUSTUS	mRNA	811158	812450	1	+	.	ID=MALK_03119.t1;Parent=MALK_03119
contig010	AUGUSTUS	exon	811158	812450	1	+	.	ID=MALK_03119.t1.e1;Parent=MALK_03119.t1
contig010	AUGUSTUS	gene	812443	813151	0.65	-	.	ID=MALK_03120;prediction_source=braker_MRET:g1670.t1
contig010	AUGUSTUS	CDS	813144	813151	0.92	-	0	ID=MALK_03120.t1.c2;Parent=MALK_03120.t1
contig010	AUGUSTUS	CDS	812443	813031	0.92	-	0	ID=MALK_03120.t1.c1;Parent=MALK_03120.t1
contig010	AUGUSTUS	mRNA	812443	813151	0.65	-	.	ID=MALK_03120.t1;Parent=MALK_03120
contig010	AUGUSTUS	exon	813144	813151	.	-	.	ID=MALK_03120.t1.e2;Parent=MALK_03120.t1
contig010	AUGUSTUS	exon	812443	813031	.	-	.	ID=MALK_03120.t1.e1;Parent=MALK_03120.t1
contig010	AUGUSTUS	gene	813254	815005	0.72	+	.	ID=MALK_03121;prediction_source=augustus:contig010.g1386.t1
contig010	AUGUSTUS	CDS	813254	815005	0.72	+	0	ID=MALK_03121.t1.c1;Parent=MALK_03121.t1
contig010	AUGUSTUS	mRNA	813254	815005	0.72	+	.	ID=MALK_03121.t1;Parent=MALK_03121
contig010	AUGUSTUS	exon	813254	815005	0.72	+	.	ID=MALK_03121.t1.e1;Parent=MALK_03121.t1
contig010	AUGUSTUS	gene	815017	816627	0.94	-	.	ID=MALK_03122;prediction_source=augustus:contig010.g1387.t1
contig010	AUGUSTUS	CDS	815017	816627	0.94	-	0	ID=MALK_03122.t1.c1;Parent=MALK_03122.t1
contig010	AUGUSTUS	mRNA	815017	816627	0.94	-	.	ID=MALK_03122.t1;Parent=MALK_03122
contig010	AUGUSTUS	exon	815017	816627	0.94	-	.	ID=MALK_03122.t1.e1;Parent=MALK_03122.t1
contig010	AUGUSTUS	gene	816789	817877	0.48	+	.	ID=MALK_03123;prediction_source=augustus:contig010.g1389.t1
contig010	AUGUSTUS	CDS	816789	817877	0.48	+	0	ID=MALK_03123.t1.c1;Parent=MALK_03123.t1
contig010	AUGUSTUS	mRNA	816789	817877	0.48	+	.	ID=MALK_03123.t1;Parent=MALK_03123
contig010	AUGUSTUS	exon	816789	817877	0.48	+	.	ID=MALK_03123.t1.e1;Parent=MALK_03123.t1
contig010	AUGUSTUS	gene	817882	818384	0.49	-	.	ID=MALK_03124;prediction_source=braker_MRET:g1674.t1
contig010	AUGUSTUS	CDS	818247	818384	0.49	-	0	ID=MALK_03124.t1.c2;Parent=MALK_03124.t1
contig010	AUGUSTUS	CDS	817882	818208	0.49	-	0	ID=MALK_03124.t1.c1;Parent=MALK_03124.t1
contig010	AUGUSTUS	mRNA	817882	818384	0.49	-	.	ID=MALK_03124.t1;Parent=MALK_03124
contig010	AUGUSTUS	exon	818247	818384	.	-	.	ID=MALK_03124.t1.e2;Parent=MALK_03124.t1
contig010	AUGUSTUS	exon	817882	818208	.	-	.	ID=MALK_03124.t1.e1;Parent=MALK_03124.t1
contig010	AUGUSTUS	gene	818432	819439	0.95	-	.	ID=MALK_03125;prediction_source=augustus:contig010.g1391.t1
contig010	AUGUSTUS	CDS	818432	819439	0.95	-	0	ID=MALK_03125.t1.c1;Parent=MALK_03125.t1
contig010	AUGUSTUS	mRNA	818432	819439	0.95	-	.	ID=MALK_03125.t1;Parent=MALK_03125
contig010	AUGUSTUS	exon	818432	819439	0.95	-	.	ID=MALK_03125.t1.e1;Parent=MALK_03125.t1
contig010	maker	gene	819520	822057	.	-	.	ID=MALK_03126;prediction_source=maker_MRET:augustus_masked-contig010-processed-gene-7.53-mRNA-1
contig010	maker	CDS	819520	822057	.	-	0	ID=MALK_03126.t1.c1;Parent=MALK_03126.t1
contig010	maker	mRNA	819520	822057	.	-	.	ID=MALK_03126.t1;Parent=MALK_03126
contig010	maker	exon	819520	822057	.	-	.	ID=MALK_03126.t1.e1;Parent=MALK_03126.t1
contig010	maker	gene	822174	823337	.	+	.	ID=MALK_03127;prediction_source=maker_MRET:augustus_masked-contig010-processed-gene-7.26-mRNA-1
contig010	maker	CDS	822174	823337	.	+	0	ID=MALK_03127.t1.c1;Parent=MALK_03127.t1
contig010	maker	mRNA	822174	823337	.	+	.	ID=MALK_03127.t1;Parent=MALK_03127

contig010	maker	exon	822174	823337	.	+	.	ID=MALK_03127.t1.e1;Parent=MALK_03127.t1
contig010	AUGUSTUS	gene	823374	824006	0.86	-	.	ID=MALK_03128;prediction_source=augustus:contig010.g1394.t1
contig010	AUGUSTUS	CDS	823374	824006	0.86	-	0	ID=MALK_03128.t1.c1;Parent=MALK_03128.t1
contig010	AUGUSTUS	mRNA	823374	824006	0.86	-	.	ID=MALK_03128.t1;Parent=MALK_03128
contig010	AUGUSTUS	exon	823374	824006	0.86	-	.	ID=MALK_03128.t1.e1;Parent=MALK_03128.t1
contig010	maker	gene	824035	825438	.	-	.	ID=MALK_03129;prediction_source=maker_MRET:augustus_masked-contig010-processed-gene-7.55-mRNA-1
contig010	maker	CDS	824035	825438	.	-	0	ID=MALK_03129.t1.c1;Parent=MALK_03129.t1
contig010	maker	mRNA	824035	825438	.	-	.	ID=MALK_03129.t1;Parent=MALK_03129
contig010	maker	exon	824035	825438	.	-	.	ID=MALK_03129.t1.e1;Parent=MALK_03129.t1
contig010	AUGUSTUS	gene	825551	826222	0.99	+	.	ID=MALK_03130;prediction_source=augustus:contig010.g1396.t1
contig010	AUGUSTUS	CDS	825551	826222	0.99	+	0	ID=MALK_03130.t1.c1;Parent=MALK_03130.t1
contig010	AUGUSTUS	mRNA	825551	826222	0.99	+	.	ID=MALK_03130.t1;Parent=MALK_03130
contig010	AUGUSTUS	exon	825551	826222	0.99	+	.	ID=MALK_03130.t1.e1;Parent=MALK_03130.t1
contig010	AUGUSTUS	gene	826212	826814	0.59	-	.	ID=MALK_03131;prediction_source=augustus:contig010.g1397.t1
contig010	AUGUSTUS	CDS	826212	826814	0.59	-	0	ID=MALK_03131.t1.c1;Parent=MALK_03131.t1
contig010	AUGUSTUS	mRNA	826212	826814	0.59	-	.	ID=MALK_03131.t1;Parent=MALK_03131
contig010	AUGUSTUS	exon	826212	826814	0.59	-	.	ID=MALK_03131.t1.e1;Parent=MALK_03131.t1
contig011	annotation	remark	1	638652	.	+	.	gff-version=3
contig011	maker	gene	182	691	.	-	.	ID=MALK_03132;prediction_source=maker_MRET:augustus_masked-contig011-processed-gene-0.42-mRNA-1
contig011	maker	CDS	182	691	.	-	0	ID=MALK_03132.t1.c1;Parent=MALK_03132.t1
contig011	maker	mRNA	182	691	.	-	.	ID=MALK_03132.t1;Parent=MALK_03132
contig011	maker	exon	182	691	.	-	.	ID=MALK_03132.t1.e1;Parent=MALK_03132.t1
contig011	maker	gene	4352	4687	.	+	.	ID=MALK_03133;prediction_source=maker_MRET:augustus_masked-contig011-processed-gene-0.21-mRNA-1
contig011	maker	CDS	4352	4687	.	+	0	ID=MALK_03133.t1.c1;Parent=MALK_03133.t1
contig011	maker	mRNA	4352	4687	.	+	.	ID=MALK_03133.t1;Parent=MALK_03133
contig011	maker	exon	4352	4687	.	+	.	ID=MALK_03133.t1.e1;Parent=MALK_03133.t1
contig011	AUGUSTUS	gene	4706	5671	0.22	-	.	ID=MALK_03134;prediction_source=braker_MRET:g3485.t1
contig011	AUGUSTUS	CDS	4706	5671	0.22	-	0	ID=MALK_03134.t1.c1;Parent=MALK_03134.t1
contig011	AUGUSTUS	mRNA	4706	5671	0.22	-	.	ID=MALK_03134.t1;Parent=MALK_03134
contig011	AUGUSTUS	exon	4706	5671	.	-	.	ID=MALK_03134.t1.e1;Parent=MALK_03134.t1
contig011	AUGUSTUS	gene	5852	8217	0.08	-	.	ID=MALK_03135;prediction_source=braker_MRET:g3486.t1
contig011	AUGUSTUS	CDS	8195	8217	0.99	-	0	ID=MALK_03135.t1.c3;Parent=MALK_03135.t1
contig011	AUGUSTUS	CDS	7334	8155	0.99	-	0	ID=MALK_03135.t1.c2;Parent=MALK_03135.t1
contig011	AUGUSTUS	CDS	5852	7097	0.99	-	0	ID=MALK_03135.t1.c1;Parent=MALK_03135.t1
contig011	AUGUSTUS	mRNA	5852	8217	0.08	-	.	ID=MALK_03135.t1;Parent=MALK_03135
contig011	AUGUSTUS	exon	8195	8217	.	-	.	ID=MALK_03135.t1.e3;Parent=MALK_03135.t1
contig011	AUGUSTUS	exon	7334	8155	.	-	.	ID=MALK_03135.t1.e2;Parent=MALK_03135.t1
contig011	AUGUSTUS	exon	5852	7097	.	-	.	ID=MALK_03135.t1.e1;Parent=MALK_03135.t1
contig011	AUGUSTUS	gene	8615	9573	0.74	+	.	ID=MALK_03136;prediction_source=braker_MRET:g3487.t1
contig011	AUGUSTUS	CDS	8615	8657	0.75	+	0	ID=MALK_03136.t1.c1;Parent=MALK_03136.t1
contig011	AUGUSTUS	CDS	8702	9573	0.75	+	0	ID=MALK_03136.t1.c2;Parent=MALK_03136.t1
contig011	AUGUSTUS	mRNA	8615	9573	0.74	+	.	ID=MALK_03136.t1;Parent=MALK_03136
contig011	AUGUSTUS	exon	8615	8657	.	+	.	ID=MALK_03136.t1.e1;Parent=MALK_03136.t1

contig011	AUGUSTUS	exon	8702	9573	.	+	.	ID=MALK_03136.t1.e2;Parent=MALK_03136.t1
contig011	AUGUSTUS	gene	9582	10823	0.81	-	.	ID=MALK_03137;prediction_source=braker_MRET:g3488.t1
contig011	AUGUSTUS	CDS	9582	10823	0.81	-	0	ID=MALK_03137.t1.c1;Parent=MALK_03137.t1
contig011	AUGUSTUS	mRNA	9582	10823	0.81	-	.	ID=MALK_03137.t1;Parent=MALK_03137
contig011	AUGUSTUS	exon	9582	10823	.	-	.	ID=MALK_03137.t1.e1;Parent=MALK_03137.t1
contig011	AUGUSTUS	gene	11030	11930	0.68	+	.	ID=MALK_03138;prediction_source=braker_MRET:g3489.t1
contig011	AUGUSTUS	CDS	11030	11032	1	+	0	ID=MALK_03138.t1.c1;Parent=MALK_03138.t1
contig011	AUGUSTUS	CDS	11071	11186	1	+	0	ID=MALK_03138.t1.c2;Parent=MALK_03138.t1
contig011	AUGUSTUS	CDS	11220	11353	1	+	0	ID=MALK_03138.t1.c3;Parent=MALK_03138.t1
contig011	AUGUSTUS	CDS	11389	11930	1	+	0	ID=MALK_03138.t1.c4;Parent=MALK_03138.t1
contig011	AUGUSTUS	mRNA	11030	11930	0.68	+	.	ID=MALK_03138.t1;Parent=MALK_03138
contig011	AUGUSTUS	exon	11030	11032	.	+	.	ID=MALK_03138.t1.e1;Parent=MALK_03138.t1
contig011	AUGUSTUS	exon	11071	11186	.	+	.	ID=MALK_03138.t1.e2;Parent=MALK_03138.t1
contig011	AUGUSTUS	exon	11220	11353	.	+	.	ID=MALK_03138.t1.e3;Parent=MALK_03138.t1
contig011	AUGUSTUS	exon	11389	11930	.	+	.	ID=MALK_03138.t1.e4;Parent=MALK_03138.t1
contig011	maker	gene	12017	13249	.	-	.	ID=MALK_03139;prediction_source=maker_MRET:augustus_masked-contig011-processed-gene-0.45-mRNA-1
contig011	maker	CDS	12017	13249	.	-	0	ID=MALK_03139.t1.c1;Parent=MALK_03139.t1
contig011	maker	mRNA	12017	13249	.	-	.	ID=MALK_03139.t1;Parent=MALK_03139
contig011	maker	exon	12017	13249	.	-	.	ID=MALK_03139.t1.e1;Parent=MALK_03139.t1
contig011	maker	gene	13550	19120	.	-	.	ID=MALK_03140;prediction_source=maker_MRET:augustus_masked-contig011-processed-gene-0.46-mRNA-1
contig011	maker	CDS	19030	19120	.	-	0	ID=MALK_03140.t1.c1;Parent=MALK_03140.t1
contig011	maker	CDS	18769	18959	.	-	0	ID=MALK_03140.t1.c2;Parent=MALK_03140.t1
contig011	maker	CDS	13550	18604	.	-	0	ID=MALK_03140.t1.c3;Parent=MALK_03140.t1
contig011	maker	mRNA	13550	19120	.	-	.	ID=MALK_03140.t1;Parent=MALK_03140
contig011	maker	exon	19030	19120	.	-	.	ID=MALK_03140.t1.e1;Parent=MALK_03140.t1
contig011	maker	exon	18769	18959	.	-	.	ID=MALK_03140.t1.e2;Parent=MALK_03140.t1
contig011	maker	exon	13550	18604	.	-	.	ID=MALK_03140.t1.e3;Parent=MALK_03140.t1
contig011	maker	gene	19555	21157	.	+	.	ID=MALK_03141;prediction_source=maker_MRET:augustus_masked-contig011-processed-gene-0.22-mRNA-1
contig011	maker	CDS	19555	19645	.	+	0	ID=MALK_03141.t1.c1;Parent=MALK_03141.t1
contig011	maker	CDS	19806	21157	.	+	0	ID=MALK_03141.t1.c2;Parent=MALK_03141.t1
contig011	maker	mRNA	19555	21157	.	+	.	ID=MALK_03141.t1;Parent=MALK_03141
contig011	maker	exon	19555	19645	.	+	.	ID=MALK_03141.t1.e1;Parent=MALK_03141.t1
contig011	maker	exon	19806	21157	.	+	.	ID=MALK_03141.t1.e2;Parent=MALK_03141.t1
contig011	maker	gene	21229	24183	.	-	.	ID=MALK_03142;prediction_source=maker_MRET:augustus_masked-contig011-processed-gene-0.47-mRNA-1
contig011	maker	CDS	21229	24183	.	-	0	ID=MALK_03142.t1.c1;Parent=MALK_03142.t1
contig011	maker	mRNA	21229	24183	.	-	.	ID=MALK_03142.t1;Parent=MALK_03142
contig011	maker	exon	21229	24183	.	-	.	ID=MALK_03142.t1.e1;Parent=MALK_03142.t1
contig011	AUGUSTUS	gene	24974	25495	0.71	+	.	ID=MALK_03143;prediction_source=braker_MRET:g3494.t1
contig011	AUGUSTUS	CDS	24974	25028	0.88	+	0	ID=MALK_03143.t1.c1;Parent=MALK_03143.t1
contig011	AUGUSTUS	CDS	25062	25495	0.88	+	0	ID=MALK_03143.t1.c2;Parent=MALK_03143.t1
contig011	AUGUSTUS	mRNA	24974	25495	0.71	+	.	ID=MALK_03143.t1;Parent=MALK_03143
contig011	AUGUSTUS	exon	24974	25028	.	+	.	ID=MALK_03143.t1.e1;Parent=MALK_03143.t1
contig011	AUGUSTUS	exon	25062	25495	.	+	.	ID=MALK_03143.t1.e2;Parent=MALK_03143.t1

contig011	maker	gene	25616	28297	.	-	.	ID=MALK_03144;prediction_source=maker_MRET:augustus_masked-contig011-processed-gene-0.48-mRNA-1
contig011	maker	CDS	25616	28297	.	-	0	ID=MALK_03144.t1.c1;Parent=MALK_03144.t1
contig011	maker	mRNA	25616	28297	.	-	.	ID=MALK_03144.t1;Parent=MALK_03144
contig011	maker	exon	25616	28297	.	-	.	ID=MALK_03144.t1.e1;Parent=MALK_03144.t1
contig011	AUGUSTUS	gene	29578	30238	0.48	+	.	ID=MALK_03145;prediction_source=augustus:contig011.g1516.t1
contig011	AUGUSTUS	CDS	29578	29614	0.95	+	0	ID=MALK_03145.t1.c1;Parent=MALK_03145.t1
contig011	AUGUSTUS	CDS	29687	29808	0.95	+	0	ID=MALK_03145.t1.c2;Parent=MALK_03145.t1
contig011	AUGUSTUS	CDS	29876	30238	0.95	+	0	ID=MALK_03145.t1.c3;Parent=MALK_03145.t1
contig011	AUGUSTUS	mRNA	29578	30238	0.48	+	.	ID=MALK_03145.t1;Parent=MALK_03145
contig011	AUGUSTUS	exon	29578	29614	0.95	+	.	ID=MALK_03145.t1.e1;Parent=MALK_03145.t1
contig011	AUGUSTUS	exon	29687	29808	0.95	+	.	ID=MALK_03145.t1.e2;Parent=MALK_03145.t1
contig011	AUGUSTUS	exon	29876	30238	0.95	+	.	ID=MALK_03145.t1.e3;Parent=MALK_03145.t1
contig011	AUGUSTUS	gene	30497	31409	1	+	.	ID=MALK_03146;prediction_source=braker_MRET:g3497.t1
contig011	AUGUSTUS	CDS	30497	30518	1	+	0	ID=MALK_03146.t1.c1;Parent=MALK_03146.t1
contig011	AUGUSTUS	CDS	30560	30583	1	+	0	ID=MALK_03146.t1.c2;Parent=MALK_03146.t1
contig011	AUGUSTUS	CDS	30624	30630	1	+	0	ID=MALK_03146.t1.c3;Parent=MALK_03146.t1
contig011	AUGUSTUS	CDS	30671	31409	1	+	0	ID=MALK_03146.t1.c4;Parent=MALK_03146.t1
contig011	AUGUSTUS	mRNA	30497	31409	1	+	.	ID=MALK_03146.t1;Parent=MALK_03146
contig011	AUGUSTUS	exon	30497	30518	.	+	.	ID=MALK_03146.t1.e1;Parent=MALK_03146.t1
contig011	AUGUSTUS	exon	30560	30583	.	+	.	ID=MALK_03146.t1.e2;Parent=MALK_03146.t1
contig011	AUGUSTUS	exon	30624	30630	.	+	.	ID=MALK_03146.t1.e3;Parent=MALK_03146.t1
contig011	AUGUSTUS	exon	30671	31409	.	+	.	ID=MALK_03146.t1.e4;Parent=MALK_03146.t1
contig011	AUGUSTUS	gene	31668	32866	0.88	-	.	ID=MALK_03147;prediction_source=braker_MRET:g3498.t1
contig011	AUGUSTUS	CDS	32519	32866	0.93	-	0	ID=MALK_03147.t1.c3;Parent=MALK_03147.t1
contig011	AUGUSTUS	CDS	32412	32479	0.93	-	0	ID=MALK_03147.t1.c2;Parent=MALK_03147.t1
contig011	AUGUSTUS	CDS	31668	32382	0.93	-	0	ID=MALK_03147.t1.c1;Parent=MALK_03147.t1
contig011	AUGUSTUS	mRNA	31668	32866	0.88	-	.	ID=MALK_03147.t1;Parent=MALK_03147
contig011	AUGUSTUS	exon	32519	32866	.	-	.	ID=MALK_03147.t1.e3;Parent=MALK_03147.t1
contig011	AUGUSTUS	exon	32412	32479	.	-	.	ID=MALK_03147.t1.e2;Parent=MALK_03147.t1
contig011	AUGUSTUS	exon	31668	32382	.	-	.	ID=MALK_03147.t1.e1;Parent=MALK_03147.t1
contig011	maker	gene	33408	34779	.	+	.	ID=MALK_03148;prediction_source=maker_MRET:augustus_masked-contig011-processed-gene-0.26-mRNA-1
contig011	maker	CDS	33408	34586	.	+	0	ID=MALK_03148.t1.c1;Parent=MALK_03148.t1
contig011	maker	CDS	34747	34779	.	+	0	ID=MALK_03148.t1.c2;Parent=MALK_03148.t1
contig011	maker	mRNA	33408	34779	.	+	.	ID=MALK_03148.t1;Parent=MALK_03148
contig011	maker	exon	33408	34586	.	+	.	ID=MALK_03148.t1.e1;Parent=MALK_03148.t1
contig011	maker	exon	34747	34779	.	+	.	ID=MALK_03148.t1.e2;Parent=MALK_03148.t1
contig011	maker	gene	34948	35850	.	-	.	ID=MALK_03149;prediction_source=maker_MRET:augustus_masked-contig011-processed-gene-0.50-mRNA-1
contig011	maker	CDS	34948	35850	.	-	0	ID=MALK_03149.t1.c1;Parent=MALK_03149.t1
contig011	maker	mRNA	34948	35850	.	-	.	ID=MALK_03149.t1;Parent=MALK_03149
contig011	maker	exon	34948	35850	.	-	.	ID=MALK_03149.t1.e1;Parent=MALK_03149.t1
contig011	maker	gene	35902	37677	.	+	.	ID=MALK_03150;prediction_source=maker_MRET:augustus_masked-contig011-processed-gene-0.27-mRNA-1
contig011	maker	CDS	35902	37677	.	+	0	ID=MALK_03150.t1.c1;Parent=MALK_03150.t1
contig011	maker	mRNA	35902	37677	.	+	.	ID=MALK_03150.t1;Parent=MALK_03150

contig011	maker	exon	35902	37677	.	+	.	ID=MALK_03150.t1.e1;Parent=MALK_03150.t1
contig011	maker	gene	37742	39385	.	+	.	ID=MALK_03151;prediction_source=maker_MRET:augustus_masked-contig011-processed-gene-0.28-mRNA-1
contig011	maker	CDS	37742	39385	.	+	0	ID=MALK_03151.t1.c1;Parent=MALK_03151.t1
contig011	maker	mRNA	37742	39385	.	+	.	ID=MALK_03151.t1;Parent=MALK_03151
contig011	maker	exon	37742	39385	.	+	.	ID=MALK_03151.t1.e1;Parent=MALK_03151.t1
contig011	AUGUSTUS	gene	40904	41197	0.58	+	.	ID=MALK_03152;prediction_source=augustus:contig011.g1524.t1
contig011	AUGUSTUS	CDS	40904	41197	0.58	+	0	ID=MALK_03152.t1.c1;Parent=MALK_03152.t1
contig011	AUGUSTUS	mRNA	40904	41197	0.58	+	.	ID=MALK_03152.t1;Parent=MALK_03152
contig011	AUGUSTUS	exon	40904	41197	0.58	+	.	ID=MALK_03152.t1.e1;Parent=MALK_03152.t1
contig011	AUGUSTUS	gene	42986	43592	0.29	-	.	ID=MALK_03153;prediction_source=augustus:contig011.g1525.t1
contig011	AUGUSTUS	CDS	43554	43592	0.29	-	0	ID=MALK_03153.t1.c2;Parent=MALK_03153.t1
contig011	AUGUSTUS	CDS	42986	43372	0.29	-	0	ID=MALK_03153.t1.c1;Parent=MALK_03153.t1
contig011	AUGUSTUS	mRNA	42986	43592	0.29	-	.	ID=MALK_03153.t1;Parent=MALK_03153
contig011	AUGUSTUS	exon	43554	43592	0.29	-	.	ID=MALK_03153.t1.e2;Parent=MALK_03153.t1
contig011	AUGUSTUS	exon	42986	43372	0.29	-	.	ID=MALK_03153.t1.e1;Parent=MALK_03153.t1
contig011	AUGUSTUS	gene	43579	44161	0.49	-	.	ID=MALK_03154;prediction_source=braker_MRET:g3503.t1
contig011	AUGUSTUS	CDS	44019	44161	0.49	-	0	ID=MALK_03154.t1.c2;Parent=MALK_03154.t1
contig011	AUGUSTUS	CDS	43579	43984	0.49	-	0	ID=MALK_03154.t1.c1;Parent=MALK_03154.t1
contig011	AUGUSTUS	mRNA	43579	44161	0.49	-	.	ID=MALK_03154.t1;Parent=MALK_03154
contig011	AUGUSTUS	exon	44019	44161	.	-	.	ID=MALK_03154.t1.e2;Parent=MALK_03154.t1
contig011	AUGUSTUS	exon	43579	43984	.	-	.	ID=MALK_03154.t1.e1;Parent=MALK_03154.t1
contig011	AUGUSTUS	gene	44735	44986	1	+	.	ID=MALK_03155;prediction_source=augustus:contig011.g1526.t1
contig011	AUGUSTUS	CDS	44735	44986	1	+	0	ID=MALK_03155.t1.c1;Parent=MALK_03155.t1
contig011	AUGUSTUS	mRNA	44735	44986	1	+	.	ID=MALK_03155.t1;Parent=MALK_03155
contig011	AUGUSTUS	exon	44735	44986	1	+	.	ID=MALK_03155.t1.e1;Parent=MALK_03155.t1
contig011	AUGUSTUS	gene	45386	46918	0.99	-	.	ID=MALK_03156;prediction_source=augustus:contig011.g1527.t1
contig011	AUGUSTUS	CDS	45386	46918	0.99	-	0	ID=MALK_03156.t1.c1;Parent=MALK_03156.t1
contig011	AUGUSTUS	mRNA	45386	46918	0.99	-	.	ID=MALK_03156.t1;Parent=MALK_03156
contig011	AUGUSTUS	exon	45386	46918	0.99	-	.	ID=MALK_03156.t1.e1;Parent=MALK_03156.t1
contig011	AUGUSTUS	gene	47048	48532	0.91	+	.	ID=MALK_03157;prediction_source=augustus:contig011.g1529.t1
contig011	AUGUSTUS	CDS	47048	48532	0.91	+	0	ID=MALK_03157.t1.c1;Parent=MALK_03157.t1
contig011	AUGUSTUS	mRNA	47048	48532	0.91	+	.	ID=MALK_03157.t1;Parent=MALK_03157
contig011	AUGUSTUS	exon	47048	48532	0.91	+	.	ID=MALK_03157.t1.e1;Parent=MALK_03157.t1
contig011	AUGUSTUS	gene	48690	50221	0.25	+	.	ID=MALK_03158;prediction_source=braker_MRET:g3507.t1
contig011	AUGUSTUS	CDS	48690	48867	0.89	+	0	ID=MALK_03158.t1.c1;Parent=MALK_03158.t1
contig011	AUGUSTUS	CDS	48902	49050	0.89	+	0	ID=MALK_03158.t1.c2;Parent=MALK_03158.t1
contig011	AUGUSTUS	CDS	49217	50221	0.89	+	0	ID=MALK_03158.t1.c3;Parent=MALK_03158.t1
contig011	AUGUSTUS	mRNA	48690	50221	0.25	+	.	ID=MALK_03158.t1;Parent=MALK_03158
contig011	AUGUSTUS	exon	48690	48867	.	+	.	ID=MALK_03158.t1.e1;Parent=MALK_03158.t1
contig011	AUGUSTUS	exon	48902	49050	.	+	.	ID=MALK_03158.t1.e2;Parent=MALK_03158.t1
contig011	AUGUSTUS	exon	49217	50221	.	+	.	ID=MALK_03158.t1.e3;Parent=MALK_03158.t1
contig011	AUGUSTUS	gene	50398	52666	0.59	-	.	ID=MALK_03159;prediction_source=braker_MRET:g3508.t1
contig011	AUGUSTUS	CDS	52544	52666	0.68	-	0	ID=MALK_03159.t1.c3;Parent=MALK_03159.t1

contig011	AUGUSTUS	CDS	52464	52475	0.68	-	0	ID=MALK_03159.t1.c2;Parent=MALK_03159.t1
contig011	AUGUSTUS	CDS	50398	52416	0.68	-	0	ID=MALK_03159.t1.c1;Parent=MALK_03159.t1
contig011	AUGUSTUS	mRNA	50398	52666	0.59	-	.	ID=MALK_03159.t1;Parent=MALK_03159
contig011	AUGUSTUS	exon	52544	52666	.	-	.	ID=MALK_03159.t1.e3;Parent=MALK_03159.t1
contig011	AUGUSTUS	exon	52464	52475	.	-	.	ID=MALK_03159.t1.e2;Parent=MALK_03159.t1
contig011	AUGUSTUS	exon	50398	52416	.	-	.	ID=MALK_03159.t1.e1;Parent=MALK_03159.t1
contig011	AUGUSTUS	gene	52665	53219	0.8	+	.	ID=MALK_03160;prediction_source=augustus:contig011.g1532.t1
contig011	AUGUSTUS	CDS	52665	53219	0.8	+	0	ID=MALK_03160.t1.c1;Parent=MALK_03160.t1
contig011	AUGUSTUS	mRNA	52665	53219	0.8	+	.	ID=MALK_03160.t1;Parent=MALK_03160
contig011	AUGUSTUS	exon	52665	53219	0.8	+	.	ID=MALK_03160.t1.e1;Parent=MALK_03160.t1
contig011	AUGUSTUS	gene	55834	56386	0.76	-	.	ID=MALK_03161;prediction_source=braker_MRET:g3509.t1
contig011	AUGUSTUS	CDS	56132	56386	0.76	-	0	ID=MALK_03161.t1.c2;Parent=MALK_03161.t1
contig011	AUGUSTUS	CDS	55834	56103	0.76	-	0	ID=MALK_03161.t1.c1;Parent=MALK_03161.t1
contig011	AUGUSTUS	mRNA	55834	56386	0.76	-	.	ID=MALK_03161.t1;Parent=MALK_03161
contig011	AUGUSTUS	exon	56132	56386	.	-	.	ID=MALK_03161.t1.e2;Parent=MALK_03161.t1
contig011	AUGUSTUS	exon	55834	56103	.	-	.	ID=MALK_03161.t1.e1;Parent=MALK_03161.t1
contig011	maker	gene	56489	57406	.	-	.	ID=MALK_03162;prediction_source=maker_MRET:augustus_masked-contig011-processed-gene-0.53-mRNA-1
contig011	maker	CDS	56996	57406	.	-	0	ID=MALK_03162.t1.c1;Parent=MALK_03162.t1
contig011	maker	CDS	56489	56890	.	-	0	ID=MALK_03162.t1.c2;Parent=MALK_03162.t1
contig011	maker	mRNA	56489	57406	.	-	.	ID=MALK_03162.t1;Parent=MALK_03162
contig011	maker	exon	56996	57406	.	-	.	ID=MALK_03162.t1.e1;Parent=MALK_03162.t1
contig011	maker	exon	56489	56890	.	-	.	ID=MALK_03162.t1.e2;Parent=MALK_03162.t1
contig011	AUGUSTUS	gene	57597	65276	0.52	-	.	ID=MALK_03163;prediction_source=augustus:contig011.g1534.t1
contig011	AUGUSTUS	CDS	57597	65276	0.52	-	0	ID=MALK_03163.t1.c1;Parent=MALK_03163.t1
contig011	AUGUSTUS	mRNA	57597	65276	0.52	-	.	ID=MALK_03163.t1;Parent=MALK_03163
contig011	AUGUSTUS	exon	57597	65276	0.52	-	.	ID=MALK_03163.t1.e1;Parent=MALK_03163.t1
contig011	AUGUSTUS	gene	65777	67867	0.56	+	.	ID=MALK_03164;prediction_source=augustus:contig011.g1537.t1
contig011	AUGUSTUS	CDS	65777	67867	0.56	+	0	ID=MALK_03164.t1.c1;Parent=MALK_03164.t1
contig011	AUGUSTUS	mRNA	65777	67867	0.56	+	.	ID=MALK_03164.t1;Parent=MALK_03164
contig011	AUGUSTUS	exon	65777	67867	0.56	+	.	ID=MALK_03164.t1.e1;Parent=MALK_03164.t1
contig011	AUGUSTUS	gene	67955	69598	1	-	.	ID=MALK_03165;prediction_source=augustus:contig011.g1538.t1
contig011	AUGUSTUS	CDS	67955	69598	1	-	0	ID=MALK_03165.t1.c1;Parent=MALK_03165.t1
contig011	AUGUSTUS	mRNA	67955	69598	1	-	.	ID=MALK_03165.t1;Parent=MALK_03165
contig011	AUGUSTUS	exon	67955	69598	1	-	.	ID=MALK_03165.t1.e1;Parent=MALK_03165.t1
contig011	maker	gene	69896	72241	.	-	.	ID=MALK_03166;prediction_source=maker_MRET:augustus_masked-contig011-processed-gene-0.56-mRNA-1
contig011	maker	CDS	69896	72241	.	-	0	ID=MALK_03166.t1.c1;Parent=MALK_03166.t1
contig011	maker	mRNA	69896	72241	.	-	.	ID=MALK_03166.t1;Parent=MALK_03166
contig011	maker	exon	69896	72241	.	-	.	ID=MALK_03166.t1.e1;Parent=MALK_03166.t1
contig011	AUGUSTUS	gene	72348	73206	0.48	+	.	ID=MALK_03167;prediction_source=augustus:contig011.g1540.t1
contig011	AUGUSTUS	CDS	72348	72352	0.5	+	0	ID=MALK_03167.t1.c1;Parent=MALK_03167.t1
contig011	AUGUSTUS	CDS	72471	73206	0.5	+	0	ID=MALK_03167.t1.c2;Parent=MALK_03167.t1
contig011	AUGUSTUS	mRNA	72348	73206	0.48	+	.	ID=MALK_03167.t1;Parent=MALK_03167
contig011	AUGUSTUS	exon	72348	72352	0.5	+	.	ID=MALK_03167.t1.e1;Parent=MALK_03167.t1

contig011	AUGUSTUS	exon	72471	73206	0.5	+	.	ID=MALK_03167.t1.e2;Parent=MALK_03167.t1
contig011	AUGUSTUS	gene	73218	76259	0.63	-	.	ID=MALK_03168;prediction_source=augustus:contig011.g1541.t1
contig011	AUGUSTUS	CDS	73218	76259	0.63	-	0	ID=MALK_03168.t1.c1;Parent=MALK_03168.t1
contig011	AUGUSTUS	mRNA	73218	76259	0.63	-	.	ID=MALK_03168.t1;Parent=MALK_03168
contig011	AUGUSTUS	exon	73218	76259	0.63	-	.	ID=MALK_03168.t1.e1;Parent=MALK_03168.t1
contig011	maker	gene	76442	77539	.	+	.	ID=MALK_03169;prediction_source=maker_MRET:augustus_masked-contig011-processed-gene-0.35-mRNA-1
contig011	maker	CDS	76442	77539	.	+	0	ID=MALK_03169.t1.c1;Parent=MALK_03169.t1
contig011	maker	mRNA	76442	77539	.	+	.	ID=MALK_03169.t1;Parent=MALK_03169
contig011	maker	exon	76442	77539	.	+	.	ID=MALK_03169.t1.e1;Parent=MALK_03169.t1
contig011	AUGUSTUS	gene	78122	79195	0.78	+	.	ID=MALK_03170;prediction_source=augustus:contig011.g1544.t1
contig011	AUGUSTUS	CDS	78122	79195	0.78	+	0	ID=MALK_03170.t1.c1;Parent=MALK_03170.t1
contig011	AUGUSTUS	mRNA	78122	79195	0.78	+	.	ID=MALK_03170.t1;Parent=MALK_03170
contig011	AUGUSTUS	exon	78122	79195	0.78	+	.	ID=MALK_03170.t1.e1;Parent=MALK_03170.t1
contig011	maker	gene	79673	80464	.	+	.	ID=MALK_03171;prediction_source=maker_MRET:augustus_masked-contig011-processed-gene-0.37-mRNA-1
contig011	maker	CDS	79673	80464	.	+	0	ID=MALK_03171.t1.c1;Parent=MALK_03171.t1
contig011	maker	mRNA	79673	80464	.	+	.	ID=MALK_03171.t1;Parent=MALK_03171
contig011	maker	exon	79673	80464	.	+	.	ID=MALK_03171.t1.e1;Parent=MALK_03171.t1
contig011	AUGUSTUS	gene	80586	81890	0.31	-	.	ID=MALK_03172;prediction_source=augustus:contig011.g1548.t1
contig011	AUGUSTUS	CDS	80586	81890	0.31	-	0	ID=MALK_03172.t1.c1;Parent=MALK_03172.t1
contig011	AUGUSTUS	mRNA	80586	81890	0.31	-	.	ID=MALK_03172.t1;Parent=MALK_03172
contig011	AUGUSTUS	exon	80586	81890	0.31	-	.	ID=MALK_03172.t1.e1;Parent=MALK_03172.t1
contig011	AUGUSTUS	gene	82194	84761	0.78	+	.	ID=MALK_03173;prediction_source=augustus:contig011.g1550.t1
contig011	AUGUSTUS	CDS	82194	84761	0.78	+	0	ID=MALK_03173.t1.c1;Parent=MALK_03173.t1
contig011	AUGUSTUS	mRNA	82194	84761	0.78	+	.	ID=MALK_03173.t1;Parent=MALK_03173
contig011	AUGUSTUS	exon	82194	84761	0.78	+	.	ID=MALK_03173.t1.e1;Parent=MALK_03173.t1
contig011	maker	gene	84770	85927	.	-	.	ID=MALK_03174;prediction_source=maker_MRET:augustus_masked-contig011-processed-gene-0.58-mRNA-1
contig011	maker	CDS	84770	85927	.	-	0	ID=MALK_03174.t1.c1;Parent=MALK_03174.t1
contig011	maker	mRNA	84770	85927	.	-	.	ID=MALK_03174.t1;Parent=MALK_03174
contig011	maker	exon	84770	85927	.	-	.	ID=MALK_03174.t1.e1;Parent=MALK_03174.t1
contig011	AUGUSTUS	gene	86090	87226	0.35	+	.	ID=MALK_03175;prediction_source=augustus:contig011.g1552.t1
contig011	AUGUSTUS	CDS	86090	87226	0.35	+	0	ID=MALK_03175.t1.c1;Parent=MALK_03175.t1
contig011	AUGUSTUS	mRNA	86090	87226	0.35	+	.	ID=MALK_03175.t1;Parent=MALK_03175
contig011	AUGUSTUS	exon	86090	87226	0.35	+	.	ID=MALK_03175.t1.e1;Parent=MALK_03175.t1
contig011	maker	gene	87436	88944	.	-	.	ID=MALK_03176;prediction_source=maker_MRET:augustus_masked-contig011-processed-gene-0.59-mRNA-1
contig011	maker	CDS	87436	88944	.	-	0	ID=MALK_03176.t1.c1;Parent=MALK_03176.t1
contig011	maker	mRNA	87436	88944	.	-	.	ID=MALK_03176.t1;Parent=MALK_03176
contig011	maker	exon	87436	88944	.	-	.	ID=MALK_03176.t1.e1;Parent=MALK_03176.t1
contig011	AUGUSTUS	gene	89925	91820	0.91	+	.	ID=MALK_03177;prediction_source=braker_MRET:g3522.t1
contig011	AUGUSTUS	CDS	89925	91820	0.91	+	0	ID=MALK_03177.t1.c1;Parent=MALK_03177.t1
contig011	AUGUSTUS	mRNA	89925	91820	0.91	+	.	ID=MALK_03177.t1;Parent=MALK_03177
contig011	AUGUSTUS	exon	89925	91820	.	+	.	ID=MALK_03177.t1.e1;Parent=MALK_03177.t1
contig011	AUGUSTUS	gene	91846	92928	1	-	.	ID=MALK_03178;prediction_source=augustus:contig011.g1556.t1
contig011	AUGUSTUS	CDS	91846	92928	1	-	0	ID=MALK_03178.t1.c1;Parent=MALK_03178.t1

contig011	AUGUSTUS	mRNA	91846	92928	1	-	.	ID=MALK_03178.t1;Parent=MALK_03178
contig011	AUGUSTUS	exon	91846	92928	1	-	.	ID=MALK_03178.t1.e1;Parent=MALK_03178.t1
contig011	maker	gene	93033	96086	.	-	.	ID=MALK_03179;prediction_source=maker_MRET:augustus_masked-contig011-processed-gene-1.49-mRNA-1
contig011	maker	CDS	93033	96086	.	-	0	ID=MALK_03179.t1.c1;Parent=MALK_03179.t1
contig011	maker	mRNA	93033	96086	.	-	.	ID=MALK_03179.t1;Parent=MALK_03179
contig011	maker	exon	93033	96086	.	-	.	ID=MALK_03179.t1.e1;Parent=MALK_03179.t1
contig011	maker	gene	97078	102174	.	+	.	ID=MALK_03180;prediction_source=maker_MRET:augustus_masked-contig011-processed-gene-1.47-mRNA-1
contig011	maker	CDS	97078	102174	.	+	0	ID=MALK_03180.t1.c1;Parent=MALK_03180.t1
contig011	maker	mRNA	97078	102174	.	+	.	ID=MALK_03180.t1;Parent=MALK_03180
contig011	maker	exon	97078	102174	.	+	.	ID=MALK_03180.t1.e1;Parent=MALK_03180.t1
contig011	AUGUSTUS	gene	102557	104545	0.99	+	.	ID=MALK_03181;prediction_source=augustus:contig011.g1560.t1
contig011	AUGUSTUS	CDS	102557	104545	0.99	+	0	ID=MALK_03181.t1.c1;Parent=MALK_03181.t1
contig011	AUGUSTUS	mRNA	102557	104545	0.99	+	.	ID=MALK_03181.t1;Parent=MALK_03181
contig011	AUGUSTUS	exon	102557	104545	0.99	+	.	ID=MALK_03181.t1.e1;Parent=MALK_03181.t1
contig011	AUGUSTUS	gene	104868	106142	0.89	+	.	ID=MALK_03182;prediction_source=augustus:contig011.g1561.t1
contig011	AUGUSTUS	CDS	104868	106142	0.89	+	0	ID=MALK_03182.t1.c1;Parent=MALK_03182.t1
contig011	AUGUSTUS	mRNA	104868	106142	0.89	+	.	ID=MALK_03182.t1;Parent=MALK_03182
contig011	AUGUSTUS	exon	104868	106142	0.89	+	.	ID=MALK_03182.t1.e1;Parent=MALK_03182.t1
contig011	AUGUSTUS	gene	106681	108554	0.71	+	.	ID=MALK_03183;prediction_source=braker_MRET:g3528.t1
contig011	AUGUSTUS	CDS	106681	106710	0.72	+	0	ID=MALK_03183.t1.c1;Parent=MALK_03183.t1
contig011	AUGUSTUS	CDS	106749	108554	0.72	+	0	ID=MALK_03183.t1.c2;Parent=MALK_03183.t1
contig011	AUGUSTUS	mRNA	106681	108554	0.71	+	.	ID=MALK_03183.t1;Parent=MALK_03183
contig011	AUGUSTUS	exon	106681	106710	.	+	.	ID=MALK_03183.t1.e1;Parent=MALK_03183.t1
contig011	AUGUSTUS	exon	106749	108554	.	+	.	ID=MALK_03183.t1.e2;Parent=MALK_03183.t1
contig011	AUGUSTUS	gene	108555	109142	0.99	-	.	ID=MALK_03184;prediction_source=augustus:contig011.g1563.t1
contig011	AUGUSTUS	CDS	108555	109142	0.99	-	0	ID=MALK_03184.t1.c1;Parent=MALK_03184.t1
contig011	AUGUSTUS	mRNA	108555	109142	0.99	-	.	ID=MALK_03184.t1;Parent=MALK_03184
contig011	AUGUSTUS	exon	108555	109142	0.99	-	.	ID=MALK_03184.t1.e1;Parent=MALK_03184.t1
contig011	maker	gene	109191	112217	.	+	.	ID=MALK_03185;prediction_source=maker_MRET:augustus_masked-contig011-processed-gene-1.53-mRNA-1
contig011	maker	CDS	109191	112217	.	+	0	ID=MALK_03185.t1.c1;Parent=MALK_03185.t1
contig011	maker	mRNA	109191	112217	.	+	.	ID=MALK_03185.t1;Parent=MALK_03185
contig011	maker	exon	109191	112217	.	+	.	ID=MALK_03185.t1.e1;Parent=MALK_03185.t1
contig011	AUGUSTUS	gene	112263	114071	0.97	-	.	ID=MALK_03186;prediction_source=augustus:contig011.g1565.t1
contig011	AUGUSTUS	CDS	112263	114071	0.97	-	0	ID=MALK_03186.t1.c1;Parent=MALK_03186.t1
contig011	AUGUSTUS	mRNA	112263	114071	0.97	-	.	ID=MALK_03186.t1;Parent=MALK_03186
contig011	AUGUSTUS	exon	112263	114071	0.97	-	.	ID=MALK_03186.t1.e1;Parent=MALK_03186.t1
contig011	maker	gene	114291	116363	.	-	.	ID=MALK_03187;prediction_source=maker_MRET:augustus_masked-contig011-processed-gene-1.73-mRNA-1
contig011	maker	CDS	114291	116363	.	-	0	ID=MALK_03187.t1.c1;Parent=MALK_03187.t1
contig011	maker	mRNA	114291	116363	.	-	.	ID=MALK_03187.t1;Parent=MALK_03187
contig011	maker	exon	114291	116363	.	-	.	ID=MALK_03187.t1.e1;Parent=MALK_03187.t1
contig011	AUGUSTUS	gene	116976	118925	1	-	.	ID=MALK_03188;prediction_source=augustus:contig011.g1567.t1
contig011	AUGUSTUS	CDS	116976	118925	1	-	0	ID=MALK_03188.t1.c1;Parent=MALK_03188.t1
contig011	AUGUSTUS	mRNA	116976	118925	1	-	.	ID=MALK_03188.t1;Parent=MALK_03188

contig011	AUGUSTUS	exon	116976	118925	1	-	.	ID=MALK_03188.t1.e1;Parent=MALK_03188.t1
contig011	maker	gene	119140	120258	.	+	.	ID=MALK_03189;prediction_source=maker_MRET:augustus_masked-contig011-processed-gene-1.54-mRNA-1
contig011	maker	CDS	119140	119161	.	+	0	ID=MALK_03189.t1.c1;Parent=MALK_03189.t1
contig011	maker	CDS	119222	120258	.	+	0	ID=MALK_03189.t1.c2;Parent=MALK_03189.t1
contig011	maker	mRNA	119140	120258	.	+	.	ID=MALK_03189.t1;Parent=MALK_03189
contig011	maker	exon	119140	119161	.	+	.	ID=MALK_03189.t1.e1;Parent=MALK_03189.t1
contig011	maker	exon	119222	120258	.	+	.	ID=MALK_03189.t1.e2;Parent=MALK_03189.t1
contig011	AUGUSTUS	gene	120521	121129	1	-	.	ID=MALK_03190;prediction_source=augustus:contig011.g1569.t1
contig011	AUGUSTUS	CDS	120521	121129	1	-	0	ID=MALK_03190.t1.c1;Parent=MALK_03190.t1
contig011	AUGUSTUS	mRNA	120521	121129	1	-	.	ID=MALK_03190.t1;Parent=MALK_03190
contig011	AUGUSTUS	exon	120521	121129	1	-	.	ID=MALK_03190.t1.e1;Parent=MALK_03190.t1
contig011	AUGUSTUS	gene	121363	121845	0.73	+	.	ID=MALK_03191;prediction_source=braker_MRET:g3535.t1
contig011	AUGUSTUS	CDS	121363	121845	0.73	+	0	ID=MALK_03191.t1.c1;Parent=MALK_03191.t1
contig011	AUGUSTUS	mRNA	121363	121845	0.73	+	.	ID=MALK_03191.t1;Parent=MALK_03191
contig011	AUGUSTUS	exon	121363	121845	.	+	.	ID=MALK_03191.t1.e1;Parent=MALK_03191.t1
contig011	AUGUSTUS	gene	122021	124798	0.49	-	.	ID=MALK_03192;prediction_source=braker_MRET:g3536.t1
contig011	AUGUSTUS	CDS	122021	124798	0.49	-	0	ID=MALK_03192.t1.c1;Parent=MALK_03192.t1
contig011	AUGUSTUS	mRNA	122021	124798	0.49	-	.	ID=MALK_03192.t1;Parent=MALK_03192
contig011	AUGUSTUS	exon	122021	124798	.	-	.	ID=MALK_03192.t1.e1;Parent=MALK_03192.t1
contig011	maker	gene	125007	125702	.	-	.	ID=MALK_03193;prediction_source=maker_MRET:augustus_masked-contig011-processed-gene-1.77-mRNA-1
contig011	maker	CDS	125007	125702	.	-	0	ID=MALK_03193.t1.c1;Parent=MALK_03193.t1
contig011	maker	mRNA	125007	125702	.	-	.	ID=MALK_03193.t1;Parent=MALK_03193
contig011	maker	exon	125007	125702	.	-	.	ID=MALK_03193.t1.e1;Parent=MALK_03193.t1
contig011	AUGUSTUS	gene	125781	127694	0.48	+	.	ID=MALK_03194;prediction_source=augustus:contig011.g1573.t1
contig011	AUGUSTUS	CDS	125781	127694	0.48	+	0	ID=MALK_03194.t1.c1;Parent=MALK_03194.t1
contig011	AUGUSTUS	mRNA	125781	127694	0.48	+	.	ID=MALK_03194.t1;Parent=MALK_03194
contig011	AUGUSTUS	exon	125781	127694	0.48	+	.	ID=MALK_03194.t1.e1;Parent=MALK_03194.t1
contig011	maker	gene	127708	129033	.	-	.	ID=MALK_03195;prediction_source=maker_MRET:augustus_masked-contig011-processed-gene-1.78-mRNA-1
contig011	maker	CDS	127708	129033	.	-	0	ID=MALK_03195.t1.c1;Parent=MALK_03195.t1
contig011	maker	mRNA	127708	129033	.	-	.	ID=MALK_03195.t1;Parent=MALK_03195
contig011	maker	exon	127708	129033	.	-	.	ID=MALK_03195.t1.e1;Parent=MALK_03195.t1
contig011	AUGUSTUS	gene	129353	130269	0.47	+	.	ID=MALK_03196;prediction_source=braker_MRET:g3540.t1
contig011	AUGUSTUS	CDS	129353	129380	1	+	0	ID=MALK_03196.t1.c1;Parent=MALK_03196.t1
contig011	AUGUSTUS	CDS	129417	129492	1	+	0	ID=MALK_03196.t1.c2;Parent=MALK_03196.t1
contig011	AUGUSTUS	CDS	129561	129580	1	+	0	ID=MALK_03196.t1.c3;Parent=MALK_03196.t1
contig011	AUGUSTUS	CDS	129619	130203	1	+	0	ID=MALK_03196.t1.c4;Parent=MALK_03196.t1
contig011	AUGUSTUS	CDS	130244	130269	1	+	0	ID=MALK_03196.t1.c5;Parent=MALK_03196.t1
contig011	AUGUSTUS	mRNA	129353	130269	0.47	+	.	ID=MALK_03196.t1;Parent=MALK_03196
contig011	AUGUSTUS	exon	129353	129380	.	+	.	ID=MALK_03196.t1.e1;Parent=MALK_03196.t1
contig011	AUGUSTUS	exon	129417	129492	.	+	.	ID=MALK_03196.t1.e2;Parent=MALK_03196.t1
contig011	AUGUSTUS	exon	129561	129580	.	+	.	ID=MALK_03196.t1.e3;Parent=MALK_03196.t1
contig011	AUGUSTUS	exon	129619	130203	.	+	.	ID=MALK_03196.t1.e4;Parent=MALK_03196.t1
contig011	AUGUSTUS	exon	130244	130269	.	+	.	ID=MALK_03196.t1.e5;Parent=MALK_03196.t1

contig011	AUGUSTUS	gene	130317	131426	0.97	-	.	ID=MALK_03197;prediction_source=augustus:contig011.g1577.t1
contig011	AUGUSTUS	CDS	130317	131426	0.97	-	0	ID=MALK_03197.t1.c1;Parent=MALK_03197.t1
contig011	AUGUSTUS	mRNA	130317	131426	0.97	-	.	ID=MALK_03197.t1;Parent=MALK_03197
contig011	AUGUSTUS	exon	130317	131426	0.97	-	.	ID=MALK_03197.t1.e1;Parent=MALK_03197.t1
contig011	maker	gene	131532	133247	.	+	.	ID=MALK_03198;prediction_source=maker_MRET:augustus_masked-contig011-processed-gene-1.58-mRNA-1
contig011	maker	CDS	131532	133247	.	+	0	ID=MALK_03198.t1.c1;Parent=MALK_03198.t1
contig011	maker	mRNA	131532	133247	.	+	.	ID=MALK_03198.t1;Parent=MALK_03198
contig011	maker	exon	131532	133247	.	+	.	ID=MALK_03198.t1.e1;Parent=MALK_03198.t1
contig011	AUGUSTUS	gene	133258	135060	0.45	-	.	ID=MALK_03199;prediction_source=augustus:contig011.g1579.t1
contig011	AUGUSTUS	CDS	133258	135060	0.45	-	0	ID=MALK_03199.t1.c1;Parent=MALK_03199.t1
contig011	AUGUSTUS	mRNA	133258	135060	0.45	-	.	ID=MALK_03199.t1;Parent=MALK_03199
contig011	AUGUSTUS	exon	133258	135060	0.45	-	.	ID=MALK_03199.t1.e1;Parent=MALK_03199.t1
contig011	AUGUSTUS	gene	135184	136569	0.98	-	.	ID=MALK_03200;prediction_source=augustus:contig011.g1580.t1
contig011	AUGUSTUS	CDS	135184	136569	0.98	-	0	ID=MALK_03200.t1.c1;Parent=MALK_03200.t1
contig011	AUGUSTUS	mRNA	135184	136569	0.98	-	.	ID=MALK_03200.t1;Parent=MALK_03200
contig011	AUGUSTUS	exon	135184	136569	0.98	-	.	ID=MALK_03200.t1.e1;Parent=MALK_03200.t1
contig011	maker	gene	136722	137927	.	-	.	ID=MALK_03201;prediction_source=maker_MRET:augustus_masked-contig011-processed-gene-1.82-mRNA-1
contig011	maker	CDS	136722	137927	.	-	0	ID=MALK_03201.t1.c1;Parent=MALK_03201.t1
contig011	maker	mRNA	136722	137927	.	-	.	ID=MALK_03201.t1;Parent=MALK_03201
contig011	maker	exon	136722	137927	.	-	.	ID=MALK_03201.t1.e1;Parent=MALK_03201.t1
contig011	maker	gene	137949	139763	.	-	.	ID=MALK_03202;prediction_source=maker_MRET:augustus_masked-contig011-processed-gene-1.83-mRNA-1
contig011	maker	CDS	137949	139763	.	-	0	ID=MALK_03202.t1.c1;Parent=MALK_03202.t1
contig011	maker	mRNA	137949	139763	.	-	.	ID=MALK_03202.t1;Parent=MALK_03202
contig011	maker	exon	137949	139763	.	-	.	ID=MALK_03202.t1.e1;Parent=MALK_03202.t1
contig011	AUGUSTUS	gene	140185	141849	0.9	+	.	ID=MALK_03203;prediction_source=braker_MRET:g3547.t1
contig011	AUGUSTUS	CDS	140185	141849	0.9	+	0	ID=MALK_03203.t1.c1;Parent=MALK_03203.t1
contig011	AUGUSTUS	mRNA	140185	141849	0.9	+	.	ID=MALK_03203.t1;Parent=MALK_03203
contig011	AUGUSTUS	exon	140185	141849	.	+	.	ID=MALK_03203.t1.e1;Parent=MALK_03203.t1
contig011	AUGUSTUS	gene	141851	144787	0.99	-	.	ID=MALK_03204;prediction_source=braker_MRET:g3548.t1
contig011	AUGUSTUS	CDS	141851	144787	0.99	-	0	ID=MALK_03204.t1.c1;Parent=MALK_03204.t1
contig011	AUGUSTUS	mRNA	141851	144787	0.99	-	.	ID=MALK_03204.t1;Parent=MALK_03204
contig011	AUGUSTUS	exon	141851	144787	.	-	.	ID=MALK_03204.t1.e1;Parent=MALK_03204.t1
contig011	AUGUSTUS	gene	144835	145938	1	+	.	ID=MALK_03205;prediction_source=braker_MRET:g3549.t1
contig011	AUGUSTUS	CDS	144835	145938	1	+	0	ID=MALK_03205.t1.c1;Parent=MALK_03205.t1
contig011	AUGUSTUS	mRNA	144835	145938	1	+	.	ID=MALK_03205.t1;Parent=MALK_03205
contig011	AUGUSTUS	exon	144835	145938	.	+	.	ID=MALK_03205.t1.e1;Parent=MALK_03205.t1
contig011	AUGUSTUS	gene	145959	147178	0.4	-	.	ID=MALK_03206;prediction_source=braker_MRET:g3550.t1
contig011	AUGUSTUS	CDS	146942	147178	0.42	-	0	ID=MALK_03206.t1.c2;Parent=MALK_03206.t1
contig011	AUGUSTUS	CDS	145959	146612	0.42	-	0	ID=MALK_03206.t1.c1;Parent=MALK_03206.t1
contig011	AUGUSTUS	mRNA	145959	147178	0.4	-	.	ID=MALK_03206.t1;Parent=MALK_03206
contig011	AUGUSTUS	exon	146942	147178	.	-	.	ID=MALK_03206.t1.e2;Parent=MALK_03206.t1
contig011	AUGUSTUS	exon	145959	146612	.	-	.	ID=MALK_03206.t1.e1;Parent=MALK_03206.t1
contig011	AUGUSTUS	gene	147552	148505	0.55	+	.	ID=MALK_03207;prediction_source=braker_MRET:g3551.t1

contig011	AUGUSTUS	CDS	147552	148505	0.55	+	0	ID=MALK_03207.t1.c1;Parent=MALK_03207.t1
contig011	AUGUSTUS	mRNA	147552	148505	0.55	+	.	ID=MALK_03207.t1;Parent=MALK_03207
contig011	AUGUSTUS	exon	147552	148505	.	+	.	ID=MALK_03207.t1.e1;Parent=MALK_03207.t1
contig011	AUGUSTUS	gene	148532	152646	0.17	+	.	ID=MALK_03208;prediction_source=braker_MRET:g3552.t1
contig011	AUGUSTUS	CDS	148532	149258	0.48	+	0	ID=MALK_03208.t1.c1;Parent=MALK_03208.t1
contig011	AUGUSTUS	CDS	149408	152646	0.48	+	0	ID=MALK_03208.t1.c2;Parent=MALK_03208.t1
contig011	AUGUSTUS	mRNA	148532	152646	0.17	+	.	ID=MALK_03208.t1;Parent=MALK_03208
contig011	AUGUSTUS	exon	148532	149258	.	+	.	ID=MALK_03208.t1.e1;Parent=MALK_03208.t1
contig011	AUGUSTUS	exon	149408	152646	.	+	.	ID=MALK_03208.t1.e2;Parent=MALK_03208.t1
contig011	maker	gene	152655	153125	.	-	.	ID=MALK_03209;prediction_source=maker_MRET:augustus_masked-contig011-processed-gene-1.84-mRNA-1
contig011	maker	CDS	152655	153125	.	-	0	ID=MALK_03209.t1.c1;Parent=MALK_03209.t1
contig011	maker	mRNA	152655	153125	.	-	.	ID=MALK_03209.t1;Parent=MALK_03209
contig011	maker	exon	152655	153125	.	-	.	ID=MALK_03209.t1.e1;Parent=MALK_03209.t1
contig011	AUGUSTUS	gene	153324	155606	0.66	+	.	ID=MALK_03210;prediction_source=augustus:contig011.g1588.t1
contig011	AUGUSTUS	CDS	153324	155606	0.66	+	0	ID=MALK_03210.t1.c1;Parent=MALK_03210.t1
contig011	AUGUSTUS	mRNA	153324	155606	0.66	+	.	ID=MALK_03210.t1;Parent=MALK_03210
contig011	AUGUSTUS	exon	153324	155606	0.66	+	.	ID=MALK_03210.t1.e1;Parent=MALK_03210.t1
contig011	AUGUSTUS	gene	155625	156950	0.22	-	.	ID=MALK_03211;prediction_source=augustus:contig011.g1589.t1
contig011	AUGUSTUS	CDS	155625	156950	0.22	-	0	ID=MALK_03211.t1.c1;Parent=MALK_03211.t1
contig011	AUGUSTUS	mRNA	155625	156950	0.22	-	.	ID=MALK_03211.t1;Parent=MALK_03211
contig011	AUGUSTUS	exon	155625	156950	0.22	-	.	ID=MALK_03211.t1.e1;Parent=MALK_03211.t1
contig011	maker	gene	157057	158874	.	+	.	ID=MALK_03212;prediction_source=maker_MRET:augustus_masked-contig011-processed-gene-1.62-mRNA-1
contig011	maker	CDS	157057	158874	.	+	0	ID=MALK_03212.t1.c1;Parent=MALK_03212.t1
contig011	maker	mRNA	157057	158874	.	+	.	ID=MALK_03212.t1;Parent=MALK_03212
contig011	maker	exon	157057	158874	.	+	.	ID=MALK_03212.t1.e1;Parent=MALK_03212.t1
contig011	maker	gene	158968	160731	.	+	.	ID=MALK_03213;prediction_source=maker_MRET:augustus_masked-contig011-processed-gene-1.63-mRNA-1
contig011	maker	CDS	158968	160731	.	+	0	ID=MALK_03213.t1.c1;Parent=MALK_03213.t1
contig011	maker	mRNA	158968	160731	.	+	.	ID=MALK_03213.t1;Parent=MALK_03213
contig011	maker	exon	158968	160731	.	+	.	ID=MALK_03213.t1.e1;Parent=MALK_03213.t1
contig011	AUGUSTUS	gene	160756	161402	0.59	-	.	ID=MALK_03214;prediction_source=braker_MRET:g3558.t1
contig011	AUGUSTUS	CDS	160809	161402	0.59	-	0	ID=MALK_03214.t1.c2;Parent=MALK_03214.t1
contig011	AUGUSTUS	CDS	160756	160773	0.59	-	0	ID=MALK_03214.t1.c1;Parent=MALK_03214.t1
contig011	AUGUSTUS	mRNA	160756	161402	0.59	-	.	ID=MALK_03214.t1;Parent=MALK_03214
contig011	AUGUSTUS	exon	160809	161402	.	-	.	ID=MALK_03214.t1.e2;Parent=MALK_03214.t1
contig011	AUGUSTUS	exon	160756	160773	.	-	.	ID=MALK_03214.t1.e1;Parent=MALK_03214.t1
contig011	AUGUSTUS	gene	161525	161931	0.77	+	.	ID=MALK_03215;prediction_source=braker_MRET:g3559.t1
contig011	AUGUSTUS	CDS	161525	161534	0.8	+	0	ID=MALK_03215.t1.c1;Parent=MALK_03215.t1
contig011	AUGUSTUS	CDS	161563	161570	0.8	+	0	ID=MALK_03215.t1.c2;Parent=MALK_03215.t1
contig011	AUGUSTUS	CDS	161599	161931	0.8	+	0	ID=MALK_03215.t1.c3;Parent=MALK_03215.t1
contig011	AUGUSTUS	mRNA	161525	161931	0.77	+	.	ID=MALK_03215.t1;Parent=MALK_03215
contig011	AUGUSTUS	exon	161525	161534	.	+	.	ID=MALK_03215.t1.e1;Parent=MALK_03215.t1
contig011	AUGUSTUS	exon	161563	161570	.	+	.	ID=MALK_03215.t1.e2;Parent=MALK_03215.t1
contig011	AUGUSTUS	exon	161599	161931	.	+	.	ID=MALK_03215.t1.e3;Parent=MALK_03215.t1

contig011	AUGUSTUS	gene	161982	163493	0.96	-	.	ID=MALK_03216;prediction_source=augustus:contig011.g1593.t1
contig011	AUGUSTUS	CDS	161982	163493	0.96	-	0	ID=MALK_03216.t1.c1;Parent=MALK_03216.t1
contig011	AUGUSTUS	mRNA	161982	163493	0.96	-	.	ID=MALK_03216.t1;Parent=MALK_03216
contig011	AUGUSTUS	exon	161982	163493	0.96	-	.	ID=MALK_03216.t1.e1;Parent=MALK_03216.t1
contig011	maker	gene	163804	164628	.	+	.	ID=MALK_03217;prediction_source=maker_MRET:augustus_masked-contig011-processed-gene-1.64-mRNA-1
contig011	maker	CDS	163804	164628	.	+	0	ID=MALK_03217.t1.c1;Parent=MALK_03217.t1
contig011	maker	mRNA	163804	164628	.	+	.	ID=MALK_03217.t1;Parent=MALK_03217
contig011	maker	exon	163804	164628	.	+	.	ID=MALK_03217.t1.e1;Parent=MALK_03217.t1
contig011	AUGUSTUS	gene	164721	165378	0.97	-	.	ID=MALK_03218;prediction_source=braker_MRET:g3562.t1
contig011	AUGUSTUS	CDS	164811	165378	0.97	-	0	ID=MALK_03218.t1.c2;Parent=MALK_03218.t1
contig011	AUGUSTUS	CDS	164721	164782	0.97	-	0	ID=MALK_03218.t1.c1;Parent=MALK_03218.t1
contig011	AUGUSTUS	mRNA	164721	165378	0.97	-	.	ID=MALK_03218.t1;Parent=MALK_03218
contig011	AUGUSTUS	exon	164811	165378	.	-	.	ID=MALK_03218.t1.e2;Parent=MALK_03218.t1
contig011	AUGUSTUS	exon	164721	164782	.	-	.	ID=MALK_03218.t1.e1;Parent=MALK_03218.t1
contig011	AUGUSTUS	gene	165682	167079	0.98	+	.	ID=MALK_03219;prediction_source=braker_MRET:g3563.t1
contig011	AUGUSTUS	CDS	165682	167079	0.98	+	0	ID=MALK_03219.t1.c1;Parent=MALK_03219.t1
contig011	AUGUSTUS	mRNA	165682	167079	0.98	+	.	ID=MALK_03219.t1;Parent=MALK_03219
contig011	AUGUSTUS	exon	165682	167079	.	+	.	ID=MALK_03219.t1.e1;Parent=MALK_03219.t1
contig011	AUGUSTUS	gene	167146	168086	0.15	+	.	ID=MALK_03220;prediction_source=braker_MRET:g3564.t1
contig011	AUGUSTUS	CDS	167146	167342	0.88	+	0	ID=MALK_03220.t1.c1;Parent=MALK_03220.t1
contig011	AUGUSTUS	CDS	167395	167537	0.88	+	0	ID=MALK_03220.t1.c2;Parent=MALK_03220.t1
contig011	AUGUSTUS	CDS	167573	167899	0.88	+	0	ID=MALK_03220.t1.c3;Parent=MALK_03220.t1
contig011	AUGUSTUS	CDS	167936	167968	0.88	+	0	ID=MALK_03220.t1.c4;Parent=MALK_03220.t1
contig011	AUGUSTUS	CDS	168007	168086	0.88	+	0	ID=MALK_03220.t1.c5;Parent=MALK_03220.t1
contig011	AUGUSTUS	mRNA	167146	168086	0.15	+	.	ID=MALK_03220.t1;Parent=MALK_03220
contig011	AUGUSTUS	exon	167146	167342	.	+	.	ID=MALK_03220.t1.e1;Parent=MALK_03220.t1
contig011	AUGUSTUS	exon	167395	167537	.	+	.	ID=MALK_03220.t1.e2;Parent=MALK_03220.t1
contig011	AUGUSTUS	exon	167573	167899	.	+	.	ID=MALK_03220.t1.e3;Parent=MALK_03220.t1
contig011	AUGUSTUS	exon	167936	167968	.	+	.	ID=MALK_03220.t1.e4;Parent=MALK_03220.t1
contig011	AUGUSTUS	exon	168007	168086	.	+	.	ID=MALK_03220.t1.e5;Parent=MALK_03220.t1
contig011	maker	gene	168093	168743	.	-	.	ID=MALK_03221;prediction_source=maker_MRET:augustus_masked-contig011-processed-gene-1.87-mRNA-1
contig011	maker	CDS	168093	168743	.	-	0	ID=MALK_03221.t1.c1;Parent=MALK_03221.t1
contig011	maker	mRNA	168093	168743	.	-	.	ID=MALK_03221.t1;Parent=MALK_03221
contig011	maker	exon	168093	168743	.	-	.	ID=MALK_03221.t1.e1;Parent=MALK_03221.t1
contig011	maker	gene	168892	169680	.	+	.	ID=MALK_03222;prediction_source=maker_MRET:augustus_masked-contig011-processed-gene-1.67-mRNA-1
contig011	maker	CDS	168892	169680	.	+	0	ID=MALK_03222.t1.c1;Parent=MALK_03222.t1
contig011	maker	mRNA	168892	169680	.	+	.	ID=MALK_03222.t1;Parent=MALK_03222
contig011	maker	exon	168892	169680	.	+	.	ID=MALK_03222.t1.e1;Parent=MALK_03222.t1
contig011	AUGUSTUS	gene	169731	171503	0.86	+	.	ID=MALK_03223;prediction_source=augustus:contig011.g1603.t1
contig011	AUGUSTUS	CDS	169731	171503	0.86	+	0	ID=MALK_03223.t1.c1;Parent=MALK_03223.t1
contig011	AUGUSTUS	mRNA	169731	171503	0.86	+	.	ID=MALK_03223.t1;Parent=MALK_03223
contig011	AUGUSTUS	exon	169731	171503	0.86	+	.	ID=MALK_03223.t1.e1;Parent=MALK_03223.t1
contig011	maker	gene	171524	172237	.	-	.	ID=MALK_03224;prediction_source=maker_MRET:augustus_masked-contig011-processed-gene-1.88-mRNA-1

contig011	maker	CDS	171524	172237	.	-	0	ID=MALK_03224.t1.c1;Parent=MALK_03224.t1
contig011	maker	mRNA	171524	172237	.	-	.	ID=MALK_03224.t1;Parent=MALK_03224
contig011	maker	exon	171524	172237	.	-	.	ID=MALK_03224.t1.e1;Parent=MALK_03224.t1
contig011	maker	gene	172720	174405	.	+	.	ID=MALK_03225;prediction_source=maker_MRET:augustus_masked-contig011-processed-gene-1.69-mRNA-1
contig011	maker	CDS	172720	174405	.	+	0	ID=MALK_03225.t1.c1;Parent=MALK_03225.t1
contig011	maker	mRNA	172720	174405	.	+	.	ID=MALK_03225.t1;Parent=MALK_03225
contig011	maker	exon	172720	174405	.	+	.	ID=MALK_03225.t1.e1;Parent=MALK_03225.t1
contig011	AUGUSTUS	gene	175059	177158	0.91	+	.	ID=MALK_03226;prediction_source=augustus:contig011.g1607.t1
contig011	AUGUSTUS	CDS	175059	177158	0.91	+	0	ID=MALK_03226.t1.c1;Parent=MALK_03226.t1
contig011	AUGUSTUS	mRNA	175059	177158	0.91	+	.	ID=MALK_03226.t1;Parent=MALK_03226
contig011	AUGUSTUS	exon	175059	177158	0.91	+	.	ID=MALK_03226.t1.e1;Parent=MALK_03226.t1
contig011	maker	gene	177240	178379	.	-	.	ID=MALK_03227;prediction_source=maker_MRET:augustus_masked-contig011-processed-gene-1.89-mRNA-1
contig011	maker	CDS	177240	178379	.	-	0	ID=MALK_03227.t1.c1;Parent=MALK_03227.t1
contig011	maker	mRNA	177240	178379	.	-	.	ID=MALK_03227.t1;Parent=MALK_03227
contig011	maker	exon	177240	178379	.	-	.	ID=MALK_03227.t1.e1;Parent=MALK_03227.t1
contig011	AUGUSTUS	gene	178484	180310	0.68	-	.	ID=MALK_03228;prediction_source=augustus:contig011.g1610.t1
contig011	AUGUSTUS	CDS	178484	180310	0.68	-	0	ID=MALK_03228.t1.c1;Parent=MALK_03228.t1
contig011	AUGUSTUS	mRNA	178484	180310	0.68	-	.	ID=MALK_03228.t1;Parent=MALK_03228
contig011	AUGUSTUS	exon	178484	180310	0.68	-	.	ID=MALK_03228.t1.e1;Parent=MALK_03228.t1
contig011	AUGUSTUS	gene	180478	181542	0.93	+	.	ID=MALK_03229;prediction_source=augustus:contig011.g1611.t1
contig011	AUGUSTUS	CDS	180478	181542	0.93	+	0	ID=MALK_03229.t1.c1;Parent=MALK_03229.t1
contig011	AUGUSTUS	mRNA	180478	181542	0.93	+	.	ID=MALK_03229.t1;Parent=MALK_03229
contig011	AUGUSTUS	exon	180478	181542	0.93	+	.	ID=MALK_03229.t1.e1;Parent=MALK_03229.t1
contig011	AUGUSTUS	gene	181563	183200	0.94	-	.	ID=MALK_03230;prediction_source=augustus:contig011.g1612.t1
contig011	AUGUSTUS	CDS	181563	183200	0.94	-	0	ID=MALK_03230.t1.c1;Parent=MALK_03230.t1
contig011	AUGUSTUS	mRNA	181563	183200	0.94	-	.	ID=MALK_03230.t1;Parent=MALK_03230
contig011	AUGUSTUS	exon	181563	183200	0.94	-	.	ID=MALK_03230.t1.e1;Parent=MALK_03230.t1
contig011	maker	gene	183250	186885	.	-	.	ID=MALK_03231;prediction_source=maker_MRET:augustus_masked-contig011-processed-gene-1.92-mRNA-1
contig011	maker	CDS	183250	186885	.	-	0	ID=MALK_03231.t1.c1;Parent=MALK_03231.t1
contig011	maker	mRNA	183250	186885	.	-	.	ID=MALK_03231.t1;Parent=MALK_03231
contig011	maker	exon	183250	186885	.	-	.	ID=MALK_03231.t1.e1;Parent=MALK_03231.t1
contig011	maker	gene	187045	187890	.	+	.	ID=MALK_03232;prediction_source=maker_MRET:augustus_masked-contig011-processed-gene-2.40-mRNA-1
contig011	maker	CDS	187045	187890	.	+	0	ID=MALK_03232.t1.c1;Parent=MALK_03232.t1
contig011	maker	mRNA	187045	187890	.	+	.	ID=MALK_03232.t1;Parent=MALK_03232
contig011	maker	exon	187045	187890	.	+	.	ID=MALK_03232.t1.e1;Parent=MALK_03232.t1
contig011	maker	gene	188103	189401	.	+	.	ID=MALK_03233;prediction_source=maker_MRET:augustus_masked-contig011-processed-gene-2.41-mRNA-1
contig011	maker	CDS	188103	189401	.	+	0	ID=MALK_03233.t1.c1;Parent=MALK_03233.t1
contig011	maker	mRNA	188103	189401	.	+	.	ID=MALK_03233.t1;Parent=MALK_03233
contig011	maker	exon	188103	189401	.	+	.	ID=MALK_03233.t1.e1;Parent=MALK_03233.t1
contig011	maker	gene	189594	193817	.	+	.	ID=MALK_03234;prediction_source=maker_MRET:augustus_masked-contig011-processed-gene-2.42-mRNA-1
contig011	maker	CDS	189594	193817	.	+	0	ID=MALK_03234.t1.c1;Parent=MALK_03234.t1
contig011	maker	mRNA	189594	193817	.	+	.	ID=MALK_03234.t1;Parent=MALK_03234
contig011	maker	exon	189594	193817	.	+	.	ID=MALK_03234.t1.e1;Parent=MALK_03234.t1

contig011	maker	gene	194014	195150	.	+	.	ID=MALK_03235;prediction_source=maker_MRET:augustus_masked-contig011-processed-gene-2.43-mRNA-1
contig011	maker	CDS	194014	195150	.	+	0	ID=MALK_03235.t1.c1;Parent=MALK_03235.t1
contig011	maker	mRNA	194014	195150	.	+	.	ID=MALK_03235.t1;Parent=MALK_03235
contig011	maker	exon	194014	195150	.	+	.	ID=MALK_03235.t1.e1;Parent=MALK_03235.t1
contig011	maker	gene	195181	195600	.	-	.	ID=MALK_03236;prediction_source=maker_MRET:augustus_masked-contig011-processed-gene-2.47-mRNA-1
contig011	maker	CDS	195181	195600	.	-	0	ID=MALK_03236.t1.c1;Parent=MALK_03236.t1
contig011	maker	mRNA	195181	195600	.	-	.	ID=MALK_03236.t1;Parent=MALK_03236
contig011	maker	exon	195181	195600	.	-	.	ID=MALK_03236.t1.e1;Parent=MALK_03236.t1
contig011	AUGUSTUS	gene	195638	196726	0.36	+	.	ID=MALK_03237;prediction_source=augustus:contig011.g1622.t1
contig011	AUGUSTUS	CDS	195638	196726	0.36	+	0	ID=MALK_03237.t1.c1;Parent=MALK_03237.t1
contig011	AUGUSTUS	mRNA	195638	196726	0.36	+	.	ID=MALK_03237.t1;Parent=MALK_03237
contig011	AUGUSTUS	exon	195638	196726	0.36	+	.	ID=MALK_03237.t1.e1;Parent=MALK_03237.t1
contig011	AUGUSTUS	gene	196732	197354	0.97	-	.	ID=MALK_03238;prediction_source=braker_MRET:g3581.t1
contig011	AUGUSTUS	CDS	197340	197354	1	-	0	ID=MALK_03238.t1.c3;Parent=MALK_03238.t1
contig011	AUGUSTUS	CDS	196917	197299	1	-	0	ID=MALK_03238.t1.c2;Parent=MALK_03238.t1
contig011	AUGUSTUS	CDS	196732	196888	1	-	0	ID=MALK_03238.t1.c1;Parent=MALK_03238.t1
contig011	AUGUSTUS	mRNA	196732	197354	0.97	-	.	ID=MALK_03238.t1;Parent=MALK_03238
contig011	AUGUSTUS	exon	197340	197354	.	-	.	ID=MALK_03238.t1.e3;Parent=MALK_03238.t1
contig011	AUGUSTUS	exon	196917	197299	.	-	.	ID=MALK_03238.t1.e2;Parent=MALK_03238.t1
contig011	AUGUSTUS	exon	196732	196888	.	-	.	ID=MALK_03238.t1.e1;Parent=MALK_03238.t1
contig011	AUGUSTUS	gene	198040	199077	0.85	+	.	ID=MALK_03239;prediction_source=augustus:contig011.g1624.t1
contig011	AUGUSTUS	CDS	198040	199077	0.85	+	0	ID=MALK_03239.t1.c1;Parent=MALK_03239.t1
contig011	AUGUSTUS	mRNA	198040	199077	0.85	+	.	ID=MALK_03239.t1;Parent=MALK_03239
contig011	AUGUSTUS	exon	198040	199077	0.85	+	.	ID=MALK_03239.t1.e1;Parent=MALK_03239.t1
contig011	AUGUSTUS	gene	199090	199413	0.95	-	.	ID=MALK_03240;prediction_source=braker_MRET:g3583.t1
contig011	AUGUSTUS	CDS	199090	199413	0.95	-	0	ID=MALK_03240.t1.c1;Parent=MALK_03240.t1
contig011	AUGUSTUS	mRNA	199090	199413	0.95	-	.	ID=MALK_03240.t1;Parent=MALK_03240
contig011	AUGUSTUS	exon	199090	199413	.	-	.	ID=MALK_03240.t1.e1;Parent=MALK_03240.t1
contig011	AUGUSTUS	gene	199770	201470	0.55	+	.	ID=MALK_03241;prediction_source=augustus:contig011.g1625.t1
contig011	AUGUSTUS	CDS	199770	201470	0.55	+	0	ID=MALK_03241.t1.c1;Parent=MALK_03241.t1
contig011	AUGUSTUS	mRNA	199770	201470	0.55	+	.	ID=MALK_03241.t1;Parent=MALK_03241
contig011	AUGUSTUS	exon	199770	201470	0.55	+	.	ID=MALK_03241.t1.e1;Parent=MALK_03241.t1
contig011	maker	gene	201485	203197	.	-	.	ID=MALK_03242;prediction_source=maker_MRET:augustus_masked-contig011-processed-gene-2.72-mRNA-1
contig011	maker	CDS	201485	203197	.	-	0	ID=MALK_03242.t1.c1;Parent=MALK_03242.t1
contig011	maker	mRNA	201485	203197	.	-	.	ID=MALK_03242.t1;Parent=MALK_03242
contig011	maker	exon	201485	203197	.	-	.	ID=MALK_03242.t1.e1;Parent=MALK_03242.t1
contig011	AUGUSTUS	gene	203514	207413	0.85	+	.	ID=MALK_03243;prediction_source=augustus:contig011.g1627.t1
contig011	AUGUSTUS	CDS	203514	207413	0.85	+	0	ID=MALK_03243.t1.c1;Parent=MALK_03243.t1
contig011	AUGUSTUS	mRNA	203514	207413	0.85	+	.	ID=MALK_03243.t1;Parent=MALK_03243
contig011	AUGUSTUS	exon	203514	207413	0.85	+	.	ID=MALK_03243.t1.e1;Parent=MALK_03243.t1
contig011	AUGUSTUS	gene	207410	209035	0.22	-	.	ID=MALK_03244;prediction_source=braker_MRET:g3587.t1
contig011	AUGUSTUS	CDS	207410	209035	0.22	-	0	ID=MALK_03244.t1.c1;Parent=MALK_03244.t1
contig011	AUGUSTUS	mRNA	207410	209035	0.22	-	.	ID=MALK_03244.t1;Parent=MALK_03244

contig011	AUGUSTUS	exon	207410	209035	.	-	.	ID=MALK_03244.t1.e1;Parent=MALK_03244.t1
contig011	AUGUSTUS	gene	209140	209547	0.94	+	.	ID=MALK_03245;prediction_source=augustus:contig011.g1629.t1
contig011	AUGUSTUS	CDS	209140	209547	0.94	+	0	ID=MALK_03245.t1.c1;Parent=MALK_03245.t1
contig011	AUGUSTUS	mRNA	209140	209547	0.94	+	.	ID=MALK_03245.t1;Parent=MALK_03245
contig011	AUGUSTUS	exon	209140	209547	0.94	+	.	ID=MALK_03245.t1.e1;Parent=MALK_03245.t1
contig011	AUGUSTUS	gene	209732	210745	0.49	-	.	ID=MALK_03246;prediction_source=braker_MRET:g3589.t1
contig011	AUGUSTUS	CDS	209732	210745	0.49	-	0	ID=MALK_03246.t1.c1;Parent=MALK_03246.t1
contig011	AUGUSTUS	mRNA	209732	210745	0.49	-	.	ID=MALK_03246.t1;Parent=MALK_03246
contig011	AUGUSTUS	exon	209732	210745	.	-	.	ID=MALK_03246.t1.e1;Parent=MALK_03246.t1
contig011	maker	gene	210778	212391	.	-	.	ID=MALK_03247;prediction_source=maker_MRET:augustus_masked-contig011-processed-gene-2.74-mRNA-1
contig011	maker	CDS	210778	212391	.	-	0	ID=MALK_03247.t1.c1;Parent=MALK_03247.t1
contig011	maker	mRNA	210778	212391	.	-	.	ID=MALK_03247.t1;Parent=MALK_03247
contig011	maker	exon	210778	212391	.	-	.	ID=MALK_03247.t1.e1;Parent=MALK_03247.t1
contig011	AUGUSTUS	gene	212536	213068	0.8	-	.	ID=MALK_03248;prediction_source=braker_MRET:g3591.t1
contig011	AUGUSTUS	CDS	213031	213068	1	-	0	ID=MALK_03248.t1.c5;Parent=MALK_03248.t1
contig011	AUGUSTUS	CDS	212946	212990	1	-	0	ID=MALK_03248.t1.c4;Parent=MALK_03248.t1
contig011	AUGUSTUS	CDS	212749	212900	1	-	0	ID=MALK_03248.t1.c3;Parent=MALK_03248.t1
contig011	AUGUSTUS	CDS	212651	212703	1	-	0	ID=MALK_03248.t1.c2;Parent=MALK_03248.t1
contig011	AUGUSTUS	CDS	212536	212619	1	-	0	ID=MALK_03248.t1.c1;Parent=MALK_03248.t1
contig011	AUGUSTUS	mRNA	212536	213068	0.8	-	.	ID=MALK_03248.t1;Parent=MALK_03248
contig011	AUGUSTUS	exon	213031	213068	.	-	.	ID=MALK_03248.t1.e5;Parent=MALK_03248.t1
contig011	AUGUSTUS	exon	212946	212990	.	-	.	ID=MALK_03248.t1.e4;Parent=MALK_03248.t1
contig011	AUGUSTUS	exon	212749	212900	.	-	.	ID=MALK_03248.t1.e3;Parent=MALK_03248.t1
contig011	AUGUSTUS	exon	212651	212703	.	-	.	ID=MALK_03248.t1.e2;Parent=MALK_03248.t1
contig011	AUGUSTUS	exon	212536	212619	.	-	.	ID=MALK_03248.t1.e1;Parent=MALK_03248.t1
contig011	maker	gene	213234	214253	.	+	.	ID=MALK_03249;prediction_source=maker_MRET:augustus_masked-contig011-processed-gene-2.51-mRNA-1
contig011	maker	CDS	213234	214253	.	+	0	ID=MALK_03249.t1.c1;Parent=MALK_03249.t1
contig011	maker	mRNA	213234	214253	.	+	.	ID=MALK_03249.t1;Parent=MALK_03249
contig011	maker	exon	213234	214253	.	+	.	ID=MALK_03249.t1.e1;Parent=MALK_03249.t1
contig011	maker	gene	214460	215413	.	+	.	ID=MALK_03250;prediction_source=maker_MRET:augustus_masked-contig011-processed-gene-2.52-mRNA-1
contig011	maker	CDS	214460	215413	.	+	0	ID=MALK_03250.t1.c1;Parent=MALK_03250.t1
contig011	maker	mRNA	214460	215413	.	+	.	ID=MALK_03250.t1;Parent=MALK_03250
contig011	maker	exon	214460	215413	.	+	.	ID=MALK_03250.t1.e1;Parent=MALK_03250.t1
contig011	AUGUSTUS	gene	216524	218149	0.99	-	.	ID=MALK_03251;prediction_source=braker_MRET:g3594.t1
contig011	AUGUSTUS	CDS	216524	218149	0.99	-	0	ID=MALK_03251.t1.c1;Parent=MALK_03251.t1
contig011	AUGUSTUS	mRNA	216524	218149	0.99	-	.	ID=MALK_03251.t1;Parent=MALK_03251
contig011	AUGUSTUS	exon	216524	218149	.	-	.	ID=MALK_03251.t1.e1;Parent=MALK_03251.t1
contig011	AUGUSTUS	gene	218351	220354	0.54	+	.	ID=MALK_03252;prediction_source=augustus:contig011.g1637.t1
contig011	AUGUSTUS	CDS	218351	220354	0.54	+	0	ID=MALK_03252.t1.c1;Parent=MALK_03252.t1
contig011	AUGUSTUS	mRNA	218351	220354	0.54	+	.	ID=MALK_03252.t1;Parent=MALK_03252
contig011	AUGUSTUS	exon	218351	220354	0.54	+	.	ID=MALK_03252.t1.e1;Parent=MALK_03252.t1
contig011	maker	gene	220546	227034	.	+	.	ID=MALK_03253;prediction_source=maker_MRET:augustus_masked-contig011-processed-gene-2.54-mRNA-1
contig011	maker	CDS	220546	227034	.	+	0	ID=MALK_03253.t1.c1;Parent=MALK_03253.t1

contig011	maker	mRNA	220546	227034	.	+	.	ID=MALK_03253.t1;Parent=MALK_03253
contig011	maker	exon	220546	227034	.	+	.	ID=MALK_03253.t1.e1;Parent=MALK_03253.t1
contig011	maker	gene	227477	228130	.	+	.	ID=MALK_03254;prediction_source=maker_MRET:augustus_masked-contig011-processed-gene-2.55-mRNA-1
contig011	maker	CDS	227477	228130	.	+	0	ID=MALK_03254.t1.c1;Parent=MALK_03254.t1
contig011	maker	mRNA	227477	228130	.	+	.	ID=MALK_03254.t1;Parent=MALK_03254
contig011	maker	exon	227477	228130	.	+	.	ID=MALK_03254.t1.e1;Parent=MALK_03254.t1
contig011	AUGUSTUS	gene	228120	229352	0.76	-	.	ID=MALK_03255;prediction_source=braker_MRET:g3597.t1
contig011	AUGUSTUS	CDS	228120	229352	0.76	-	0	ID=MALK_03255.t1.c1;Parent=MALK_03255.t1
contig011	AUGUSTUS	mRNA	228120	229352	0.76	-	.	ID=MALK_03255.t1;Parent=MALK_03255
contig011	AUGUSTUS	exon	228120	229352	.	-	.	ID=MALK_03255.t1.e1;Parent=MALK_03255.t1
contig011	maker	gene	229492	230151	.	+	.	ID=MALK_03256;prediction_source=maker_MRET:augustus_masked-contig011-processed-gene-2.56-mRNA-1
contig011	maker	CDS	229492	230151	.	+	0	ID=MALK_03256.t1.c1;Parent=MALK_03256.t1
contig011	maker	mRNA	229492	230151	.	+	.	ID=MALK_03256.t1;Parent=MALK_03256
contig011	maker	exon	229492	230151	.	+	.	ID=MALK_03256.t1.e1;Parent=MALK_03256.t1
contig011	maker	gene	230377	231357	.	+	.	ID=MALK_03257;prediction_source=maker_MRET:augustus_masked-contig011-processed-gene-2.57-mRNA-1
contig011	maker	CDS	230377	231357	.	+	0	ID=MALK_03257.t1.c1;Parent=MALK_03257.t1
contig011	maker	mRNA	230377	231357	.	+	.	ID=MALK_03257.t1;Parent=MALK_03257
contig011	maker	exon	230377	231357	.	+	.	ID=MALK_03257.t1.e1;Parent=MALK_03257.t1
contig011	AUGUSTUS	gene	231329	233951	0.36	-	.	ID=MALK_03258;prediction_source=braker_MRET:g3600.t1
contig011	AUGUSTUS	CDS	233521	233951	0.89	-	0	ID=MALK_03258.t1.c2;Parent=MALK_03258.t1
contig011	AUGUSTUS	CDS	231329	233477	0.89	-	0	ID=MALK_03258.t1.c1;Parent=MALK_03258.t1
contig011	AUGUSTUS	mRNA	231329	233951	0.36	-	.	ID=MALK_03258.t1;Parent=MALK_03258
contig011	AUGUSTUS	exon	233521	233951	.	-	.	ID=MALK_03258.t1.e2;Parent=MALK_03258.t1
contig011	AUGUSTUS	exon	231329	233477	.	-	.	ID=MALK_03258.t1.e1;Parent=MALK_03258.t1
contig011	maker	gene	234169	238149	.	+	.	ID=MALK_03259;prediction_source=maker_MRET:augustus_masked-contig011-processed-gene-2.58-mRNA-1
contig011	maker	CDS	234169	238149	.	+	0	ID=MALK_03259.t1.c1;Parent=MALK_03259.t1
contig011	maker	mRNA	234169	238149	.	+	.	ID=MALK_03259.t1;Parent=MALK_03259
contig011	maker	exon	234169	238149	.	+	.	ID=MALK_03259.t1.e1;Parent=MALK_03259.t1
contig011	AUGUSTUS	gene	238301	240019	0.68	+	.	ID=MALK_03260;prediction_source=augustus:contig011.g1646.t1
contig011	AUGUSTUS	CDS	238301	240019	0.68	+	0	ID=MALK_03260.t1.c1;Parent=MALK_03260.t1
contig011	AUGUSTUS	mRNA	238301	240019	0.68	+	.	ID=MALK_03260.t1;Parent=MALK_03260
contig011	AUGUSTUS	exon	238301	240019	0.68	+	.	ID=MALK_03260.t1.e1;Parent=MALK_03260.t1
contig011	AUGUSTUS	gene	240041	240821	0.88	-	.	ID=MALK_03261;prediction_source=braker_MRET:g3603.t1
contig011	AUGUSTUS	CDS	240552	240821	0.88	-	0	ID=MALK_03261.t1.c3;Parent=MALK_03261.t1
contig011	AUGUSTUS	CDS	240445	240523	0.88	-	0	ID=MALK_03261.t1.c2;Parent=MALK_03261.t1
contig011	AUGUSTUS	CDS	240041	240417	0.88	-	0	ID=MALK_03261.t1.c1;Parent=MALK_03261.t1
contig011	AUGUSTUS	mRNA	240041	240821	0.88	-	.	ID=MALK_03261.t1;Parent=MALK_03261
contig011	AUGUSTUS	exon	240552	240821	.	-	.	ID=MALK_03261.t1.e3;Parent=MALK_03261.t1
contig011	AUGUSTUS	exon	240445	240523	.	-	.	ID=MALK_03261.t1.e2;Parent=MALK_03261.t1
contig011	AUGUSTUS	exon	240041	240417	.	-	.	ID=MALK_03261.t1.e1;Parent=MALK_03261.t1
contig011	AUGUSTUS	gene	240927	242009	0.95	-	.	ID=MALK_03262;prediction_source=augustus:contig011.g1649.t1
contig011	AUGUSTUS	CDS	240927	242009	0.95	-	0	ID=MALK_03262.t1.c1;Parent=MALK_03262.t1
contig011	AUGUSTUS	mRNA	240927	242009	0.95	-	.	ID=MALK_03262.t1;Parent=MALK_03262

contig011	AUGUSTUS	exon	240927	242009	0.95	-	.	ID=MALK_03262.t1.e1;Parent=MALK_03262.t1
contig011	AUGUSTUS	gene	242390	244570	1	+	.	ID=MALK_03263;prediction_source=augustus:contig011.g1650.t1
contig011	AUGUSTUS	CDS	242390	244570	1	+	0	ID=MALK_03263.t1.c1;Parent=MALK_03263.t1
contig011	AUGUSTUS	mRNA	242390	244570	1	+	.	ID=MALK_03263.t1;Parent=MALK_03263
contig011	AUGUSTUS	exon	242390	244570	1	+	.	ID=MALK_03263.t1.e1;Parent=MALK_03263.t1
contig011	maker	gene	244585	245808	.	-	.	ID=MALK_03264;prediction_source=maker_MRET:augustus_masked-contig011-processed-gene-2.80-mRNA-1
contig011	maker	CDS	244585	245808	.	-	0	ID=MALK_03264.t1.c1;Parent=MALK_03264.t1
contig011	maker	mRNA	244585	245808	.	-	.	ID=MALK_03264.t1;Parent=MALK_03264
contig011	maker	exon	244585	245808	.	-	.	ID=MALK_03264.t1.e1;Parent=MALK_03264.t1
contig011	maker	gene	246054	246494	.	+	.	ID=MALK_03265;prediction_source=maker_MRET:augustus_masked-contig011-processed-gene-2.61-mRNA-1
contig011	maker	CDS	246054	246494	.	+	0	ID=MALK_03265.t1.c1;Parent=MALK_03265.t1
contig011	maker	mRNA	246054	246494	.	+	.	ID=MALK_03265.t1;Parent=MALK_03265
contig011	maker	exon	246054	246494	.	+	.	ID=MALK_03265.t1.e1;Parent=MALK_03265.t1
contig011	maker	gene	246774	249626	.	+	.	ID=MALK_03266;prediction_source=maker_MRET:augustus_masked-contig011-processed-gene-2.62-mRNA-1
contig011	maker	CDS	246774	249626	.	+	0	ID=MALK_03266.t1.c1;Parent=MALK_03266.t1
contig011	maker	mRNA	246774	249626	.	+	.	ID=MALK_03266.t1;Parent=MALK_03266
contig011	maker	exon	246774	249626	.	+	.	ID=MALK_03266.t1.e1;Parent=MALK_03266.t1
contig011	maker	gene	249857	251728	.	+	.	ID=MALK_03267;prediction_source=maker_MRET:augustus_masked-contig011-processed-gene-2.63-mRNA-1
contig011	maker	CDS	249857	251728	.	+	0	ID=MALK_03267.t1.c1;Parent=MALK_03267.t1
contig011	maker	mRNA	249857	251728	.	+	.	ID=MALK_03267.t1;Parent=MALK_03267
contig011	maker	exon	249857	251728	.	+	.	ID=MALK_03267.t1.e1;Parent=MALK_03267.t1
contig011	AUGUSTUS	gene	251975	253369	0.59	+	.	ID=MALK_03268;prediction_source=augustus:contig011.g1655.t1
contig011	AUGUSTUS	CDS	251975	253369	0.59	+	0	ID=MALK_03268.t1.c1;Parent=MALK_03268.t1
contig011	AUGUSTUS	mRNA	251975	253369	0.59	+	.	ID=MALK_03268.t1;Parent=MALK_03268
contig011	AUGUSTUS	exon	251975	253369	0.59	+	.	ID=MALK_03268.t1.e1;Parent=MALK_03268.t1
contig011	AUGUSTUS	gene	253627	255243	0.46	-	.	ID=MALK_03269;prediction_source=augustus:contig011.g1656.t1
contig011	AUGUSTUS	CDS	255126	255243	0.49	-	0	ID=MALK_03269.t1.c2;Parent=MALK_03269.t1
contig011	AUGUSTUS	CDS	253627	255041	0.49	-	0	ID=MALK_03269.t1.c1;Parent=MALK_03269.t1
contig011	AUGUSTUS	mRNA	253627	255243	0.46	-	.	ID=MALK_03269.t1;Parent=MALK_03269
contig011	AUGUSTUS	exon	255126	255243	0.49	-	.	ID=MALK_03269.t1.e2;Parent=MALK_03269.t1
contig011	AUGUSTUS	exon	253627	255041	0.49	-	.	ID=MALK_03269.t1.e1;Parent=MALK_03269.t1
contig011	AUGUSTUS	gene	256231	257230	0.85	+	.	ID=MALK_03270;prediction_source=braker_MRET:g3612.t1
contig011	AUGUSTUS	CDS	256231	256465	0.99	+	0	ID=MALK_03270.t1.c1;Parent=MALK_03270.t1
contig011	AUGUSTUS	CDS	256506	257230	0.99	+	0	ID=MALK_03270.t1.c2;Parent=MALK_03270.t1
contig011	AUGUSTUS	mRNA	256231	257230	0.85	+	.	ID=MALK_03270.t1;Parent=MALK_03270
contig011	AUGUSTUS	exon	256231	256465	.	+	.	ID=MALK_03270.t1.e1;Parent=MALK_03270.t1
contig011	AUGUSTUS	exon	256506	257230	.	+	.	ID=MALK_03270.t1.e2;Parent=MALK_03270.t1
contig011	maker	gene	257310	258206	.	-	.	ID=MALK_03271;prediction_source=maker_MRET:augustus_masked-contig011-processed-gene-2.82-mRNA-1
contig011	maker	CDS	257310	258206	.	-	0	ID=MALK_03271.t1.c1;Parent=MALK_03271.t1
contig011	maker	mRNA	257310	258206	.	-	.	ID=MALK_03271.t1;Parent=MALK_03271
contig011	maker	exon	257310	258206	.	-	.	ID=MALK_03271.t1.e1;Parent=MALK_03271.t1
contig011	AUGUSTUS	gene	258332	260071	0.66	+	.	ID=MALK_03272;prediction_source=augustus:contig011.g1660.t1
contig011	AUGUSTUS	CDS	258332	260071	0.66	+	0	ID=MALK_03272.t1.c1;Parent=MALK_03272.t1

contig011	AUGUSTUS	mRNA	258332	260071	0.66	+	.	ID=MALK_03272.t1;Parent=MALK_03272
contig011	AUGUSTUS	exon	258332	260071	0.66	+	.	ID=MALK_03272.t1.e1;Parent=MALK_03272.t1
contig011	AUGUSTUS	gene	260073	262655	0.69	-	.	ID=MALK_03273;prediction_source=braker_MRET:g3615.t1
contig011	AUGUSTUS	CDS	260073	262655	0.69	-	0	ID=MALK_03273.t1.c1;Parent=MALK_03273.t1
contig011	AUGUSTUS	mRNA	260073	262655	0.69	-	.	ID=MALK_03273.t1;Parent=MALK_03273
contig011	AUGUSTUS	exon	260073	262655	.	-	.	ID=MALK_03273.t1.e1;Parent=MALK_03273.t1
contig011	AUGUSTUS	gene	263015	265363	0.97	+	.	ID=MALK_03274;prediction_source=braker_MRET:g3616.t1
contig011	AUGUSTUS	CDS	263015	265363	0.97	+	0	ID=MALK_03274.t1.c1;Parent=MALK_03274.t1
contig011	AUGUSTUS	mRNA	263015	265363	0.97	+	.	ID=MALK_03274.t1;Parent=MALK_03274
contig011	AUGUSTUS	exon	263015	265363	.	+	.	ID=MALK_03274.t1.e1;Parent=MALK_03274.t1
contig011	AUGUSTUS	gene	265443	269121	0.33	+	.	ID=MALK_03275;prediction_source=braker_MRET:g3617.t1
contig011	AUGUSTUS	CDS	265443	267818	0.98	+	0	ID=MALK_03275.t1.c1;Parent=MALK_03275.t1
contig011	AUGUSTUS	CDS	267952	267976	0.98	+	0	ID=MALK_03275.t1.c2;Parent=MALK_03275.t1
contig011	AUGUSTUS	CDS	268014	268280	0.98	+	0	ID=MALK_03275.t1.c3;Parent=MALK_03275.t1
contig011	AUGUSTUS	CDS	268322	268491	0.98	+	0	ID=MALK_03275.t1.c4;Parent=MALK_03275.t1
contig011	AUGUSTUS	CDS	268521	268595	0.98	+	0	ID=MALK_03275.t1.c5;Parent=MALK_03275.t1
contig011	AUGUSTUS	CDS	268675	269121	0.98	+	0	ID=MALK_03275.t1.c6;Parent=MALK_03275.t1
contig011	AUGUSTUS	mRNA	265443	269121	0.33	+	.	ID=MALK_03275.t1;Parent=MALK_03275
contig011	AUGUSTUS	exon	265443	267818	.	+	.	ID=MALK_03275.t1.e1;Parent=MALK_03275.t1
contig011	AUGUSTUS	exon	267952	267976	.	+	.	ID=MALK_03275.t1.e2;Parent=MALK_03275.t1
contig011	AUGUSTUS	exon	268014	268280	.	+	.	ID=MALK_03275.t1.e3;Parent=MALK_03275.t1
contig011	AUGUSTUS	exon	268322	268491	.	+	.	ID=MALK_03275.t1.e4;Parent=MALK_03275.t1
contig011	AUGUSTUS	exon	268521	268595	.	+	.	ID=MALK_03275.t1.e5;Parent=MALK_03275.t1
contig011	AUGUSTUS	exon	268675	269121	.	+	.	ID=MALK_03275.t1.e6;Parent=MALK_03275.t1
contig011	AUGUSTUS	gene	269419	270573	0.99	+	.	ID=MALK_03276;prediction_source=augustus:contig011.g1664.t1
contig011	AUGUSTUS	CDS	269419	270573	0.99	+	0	ID=MALK_03276.t1.c1;Parent=MALK_03276.t1
contig011	AUGUSTUS	mRNA	269419	270573	0.99	+	.	ID=MALK_03276.t1;Parent=MALK_03276
contig011	AUGUSTUS	exon	269419	270573	0.99	+	.	ID=MALK_03276.t1.e1;Parent=MALK_03276.t1
contig011	AUGUSTUS	gene	271274	272242	0.68	-	.	ID=MALK_03277;prediction_source=braker_MRET:g3619.t1
contig011	AUGUSTUS	CDS	272170	272242	0.76	-	0	ID=MALK_03277.t1.c2;Parent=MALK_03277.t1
contig011	AUGUSTUS	CDS	271274	272028	0.76	-	0	ID=MALK_03277.t1.c1;Parent=MALK_03277.t1
contig011	AUGUSTUS	mRNA	271274	272242	0.68	-	.	ID=MALK_03277.t1;Parent=MALK_03277
contig011	AUGUSTUS	exon	272170	272242	.	-	.	ID=MALK_03277.t1.e2;Parent=MALK_03277.t1
contig011	AUGUSTUS	exon	271274	272028	.	-	.	ID=MALK_03277.t1.e1;Parent=MALK_03277.t1
contig011	maker	gene	272272	273855	.	+	.	ID=MALK_03278;prediction_source=maker_MRET:augustus_masked-contig011-processed-gene-2.69-mRNA-1
contig011	maker	CDS	272272	273855	.	+	0	ID=MALK_03278.t1.c1;Parent=MALK_03278.t1
contig011	maker	mRNA	272272	273855	.	+	.	ID=MALK_03278.t1;Parent=MALK_03278
contig011	maker	exon	272272	273855	.	+	.	ID=MALK_03278.t1.e1;Parent=MALK_03278.t1
contig011	AUGUSTUS	gene	273856	274701	0.51	-	.	ID=MALK_03279;prediction_source=augustus:contig011.g1667.t1
contig011	AUGUSTUS	CDS	273856	274701	0.51	-	0	ID=MALK_03279.t1.c1;Parent=MALK_03279.t1
contig011	AUGUSTUS	mRNA	273856	274701	0.51	-	.	ID=MALK_03279.t1;Parent=MALK_03279
contig011	AUGUSTUS	exon	273856	274701	0.51	-	.	ID=MALK_03279.t1.e1;Parent=MALK_03279.t1
contig011	AUGUSTUS	gene	274872	275862	0.9	+	.	ID=MALK_03280;prediction_source=braker_MRET:g3621.t1

contig011	AUGUSTUS	CDS	274872	274994	0.96	+	0	ID=MALK_03280.t1.c1;Parent=MALK_03280.t1
contig011	AUGUSTUS	CDS	275032	275862	0.96	+	0	ID=MALK_03280.t1.c2;Parent=MALK_03280.t1
contig011	AUGUSTUS	mRNA	274872	275862	0.9	+	.	ID=MALK_03280.t1;Parent=MALK_03280
contig011	AUGUSTUS	exon	274872	274994	.	+	.	ID=MALK_03280.t1.e1;Parent=MALK_03280.t1
contig011	AUGUSTUS	exon	275032	275862	.	+	.	ID=MALK_03280.t1.e2;Parent=MALK_03280.t1
contig011	AUGUSTUS	gene	276118	276954	0.93	-	.	ID=MALK_03281;prediction_source=augustus:contig011.g1669.t1
contig011	AUGUSTUS	CDS	276118	276954	0.93	-	0	ID=MALK_03281.t1.c1;Parent=MALK_03281.t1
contig011	AUGUSTUS	mRNA	276118	276954	0.93	-	.	ID=MALK_03281.t1;Parent=MALK_03281
contig011	AUGUSTUS	exon	276118	276954	0.93	-	.	ID=MALK_03281.t1.e1;Parent=MALK_03281.t1
contig011	maker	gene	277060	279180	.	-	.	ID=MALK_03282;prediction_source=maker_MRET:augustus_masked-contig011-processed-gene-2.85-mRNA-1
contig011	maker	CDS	277060	279180	.	-	0	ID=MALK_03282.t1.c1;Parent=MALK_03282.t1
contig011	maker	mRNA	277060	279180	.	-	.	ID=MALK_03282.t1;Parent=MALK_03282
contig011	maker	exon	277060	279180	.	-	.	ID=MALK_03282.t1.e1;Parent=MALK_03282.t1
contig011	AUGUSTUS	gene	279248	282610	1	-	.	ID=MALK_03283;prediction_source=augustus:contig011.g1671.t1
contig011	AUGUSTUS	CDS	279248	282610	1	-	0	ID=MALK_03283.t1.c1;Parent=MALK_03283.t1
contig011	AUGUSTUS	mRNA	279248	282610	1	-	.	ID=MALK_03283.t1;Parent=MALK_03283
contig011	AUGUSTUS	exon	279248	282610	1	-	.	ID=MALK_03283.t1.e1;Parent=MALK_03283.t1
contig011	AUGUSTUS	gene	282702	289760	0.48	-	.	ID=MALK_03284;prediction_source=augustus:contig011.g1673.t1
contig011	AUGUSTUS	CDS	282702	289760	0.48	-	0	ID=MALK_03284.t1.c1;Parent=MALK_03284.t1
contig011	AUGUSTUS	mRNA	282702	289760	0.48	-	.	ID=MALK_03284.t1;Parent=MALK_03284
contig011	AUGUSTUS	exon	282702	289760	0.48	-	.	ID=MALK_03284.t1.e1;Parent=MALK_03284.t1
contig011	maker	gene	290271	292292	.	+	.	ID=MALK_03285;prediction_source=maker_MRET:augustus_masked-contig011-processed-gene-2.71-mRNA-1
contig011	maker	CDS	290271	292292	.	+	0	ID=MALK_03285.t1.c1;Parent=MALK_03285.t1
contig011	maker	mRNA	290271	292292	.	+	.	ID=MALK_03285.t1;Parent=MALK_03285
contig011	maker	exon	290271	292292	.	+	.	ID=MALK_03285.t1.e1;Parent=MALK_03285.t1
contig011	AUGUSTUS	gene	292519	293619	0.87	-	.	ID=MALK_03286;prediction_source=braker_MRET:g3626.t1
contig011	AUGUSTUS	CDS	292519	293619	0.87	-	0	ID=MALK_03286.t1.c1;Parent=MALK_03286.t1
contig011	AUGUSTUS	mRNA	292519	293619	0.87	-	.	ID=MALK_03286.t1;Parent=MALK_03286
contig011	AUGUSTUS	exon	292519	293619	.	-	.	ID=MALK_03286.t1.e1;Parent=MALK_03286.t1
contig011	AUGUSTUS	gene	293764	294140	0.98	+	.	ID=MALK_03287;prediction_source=braker_MRET:g3627.t1
contig011	AUGUSTUS	CDS	293764	294100	0.98	+	0	ID=MALK_03287.t1.c1;Parent=MALK_03287.t1
contig011	AUGUSTUS	CDS	294136	294140	0.98	+	0	ID=MALK_03287.t1.c2;Parent=MALK_03287.t1
contig011	AUGUSTUS	mRNA	293764	294140	0.98	+	.	ID=MALK_03287.t1;Parent=MALK_03287
contig011	AUGUSTUS	exon	293764	294100	.	+	.	ID=MALK_03287.t1.e1;Parent=MALK_03287.t1
contig011	AUGUSTUS	exon	294136	294140	.	+	.	ID=MALK_03287.t1.e2;Parent=MALK_03287.t1
contig011	maker	gene	294166	295173	.	-	.	ID=MALK_03288;prediction_source=maker_MRET:augustus_masked-contig011-processed-gene-3.3-mRNA-1
contig011	maker	CDS	294166	295173	.	-	0	ID=MALK_03288.t1.c1;Parent=MALK_03288.t1
contig011	maker	mRNA	294166	295173	.	-	.	ID=MALK_03288.t1;Parent=MALK_03288
contig011	maker	exon	294166	295173	.	-	.	ID=MALK_03288.t1.e1;Parent=MALK_03288.t1
contig011	maker	gene	295371	296759	.	+	.	ID=MALK_03289;prediction_source=maker_MRET:augustus_masked-contig011-processed-gene-3.0-mRNA-1
contig011	maker	CDS	295371	296759	.	+	0	ID=MALK_03289.t1.c1;Parent=MALK_03289.t1
contig011	maker	mRNA	295371	296759	.	+	.	ID=MALK_03289.t1;Parent=MALK_03289
contig011	maker	exon	295371	296759	.	+	.	ID=MALK_03289.t1.e1;Parent=MALK_03289.t1

contig011	AUGUSTUS	gene	296850	297608	0.87	+	.	ID=MALK_03290;prediction_source=augustus:contig011.g1679.t1
contig011	AUGUSTUS	CDS	296850	297608	0.87	+	0	ID=MALK_03290.t1.c1;Parent=MALK_03290.t1
contig011	AUGUSTUS	mRNA	296850	297608	0.87	+	.	ID=MALK_03290.t1;Parent=MALK_03290
contig011	AUGUSTUS	exon	296850	297608	0.87	+	.	ID=MALK_03290.t1.e1;Parent=MALK_03290.t1
contig011	AUGUSTUS	gene	297638	298264	0.19	-	.	ID=MALK_03291;prediction_source=braker_MRET:g3631.t1
contig011	AUGUSTUS	CDS	298067	298264	0.46	-	0	ID=MALK_03291.t1.c3;Parent=MALK_03291.t1
contig011	AUGUSTUS	CDS	297928	298019	0.46	-	0	ID=MALK_03291.t1.c2;Parent=MALK_03291.t1
contig011	AUGUSTUS	CDS	297638	297815	0.46	-	0	ID=MALK_03291.t1.c1;Parent=MALK_03291.t1
contig011	AUGUSTUS	mRNA	297638	298264	0.19	-	.	ID=MALK_03291.t1;Parent=MALK_03291
contig011	AUGUSTUS	exon	298067	298264	.	-	.	ID=MALK_03291.t1.e3;Parent=MALK_03291.t1
contig011	AUGUSTUS	exon	297928	298019	.	-	.	ID=MALK_03291.t1.e2;Parent=MALK_03291.t1
contig011	AUGUSTUS	exon	297638	297815	.	-	.	ID=MALK_03291.t1.e1;Parent=MALK_03291.t1
contig011	AUGUSTUS	gene	298334	298960	1	+	.	ID=MALK_03292;prediction_source=braker_MRET:g3632.t1
contig011	AUGUSTUS	CDS	298334	298960	1	+	0	ID=MALK_03292.t1.c1;Parent=MALK_03292.t1
contig011	AUGUSTUS	mRNA	298334	298960	1	+	.	ID=MALK_03292.t1;Parent=MALK_03292
contig011	AUGUSTUS	exon	298334	298960	.	+	.	ID=MALK_03292.t1.e1;Parent=MALK_03292.t1
contig011	maker	gene	299012	300145	.	-	.	ID=MALK_03293;prediction_source=maker_MRET:augustus_masked-contig011-processed-gene-3.4-mRNA-1
contig011	maker	CDS	299012	300145	.	-	0	ID=MALK_03293.t1.c1;Parent=MALK_03293.t1
contig011	maker	mRNA	299012	300145	.	-	.	ID=MALK_03293.t1;Parent=MALK_03293
contig011	maker	exon	299012	300145	.	-	.	ID=MALK_03293.t1.e1;Parent=MALK_03293.t1
contig011	maker	gene	300214	301284	.	-	.	ID=MALK_03294;prediction_source=maker_MRET:augustus_masked-contig011-processed-gene-3.32-mRNA-1
contig011	maker	CDS	300214	301284	.	-	0	ID=MALK_03294.t1.c1;Parent=MALK_03294.t1
contig011	maker	mRNA	300214	301284	.	-	.	ID=MALK_03294.t1;Parent=MALK_03294
contig011	maker	exon	300214	301284	.	-	.	ID=MALK_03294.t1.e1;Parent=MALK_03294.t1
contig011	AUGUSTUS	gene	301706	302429	0.57	-	.	ID=MALK_03295;prediction_source=augustus:contig011.g1684.t1
contig011	AUGUSTUS	CDS	302379	302429	0.97	-	0	ID=MALK_03295.t1.c2;Parent=MALK_03295.t1
contig011	AUGUSTUS	CDS	301706	302311	0.97	-	0	ID=MALK_03295.t1.c1;Parent=MALK_03295.t1
contig011	AUGUSTUS	mRNA	301706	302429	0.57	-	.	ID=MALK_03295.t1;Parent=MALK_03295
contig011	AUGUSTUS	exon	302379	302429	0.97	-	.	ID=MALK_03295.t1.e2;Parent=MALK_03295.t1
contig011	AUGUSTUS	exon	301706	302311	0.97	-	.	ID=MALK_03295.t1.e1;Parent=MALK_03295.t1
contig011	AUGUSTUS	gene	302727	304664	0.42	+	.	ID=MALK_03296;prediction_source=augustus:contig011.g1685.t1
contig011	AUGUSTUS	CDS	302727	304664	0.42	+	0	ID=MALK_03296.t1.c1;Parent=MALK_03296.t1
contig011	AUGUSTUS	mRNA	302727	304664	0.42	+	.	ID=MALK_03296.t1;Parent=MALK_03296
contig011	AUGUSTUS	exon	302727	304664	0.42	+	.	ID=MALK_03296.t1.e1;Parent=MALK_03296.t1
contig011	maker	gene	304661	306526	.	-	.	ID=MALK_03297;prediction_source=maker_MRET:augustus_masked-contig011-processed-gene-3.34-mRNA-1
contig011	maker	CDS	304661	306526	.	-	0	ID=MALK_03297.t1.c1;Parent=MALK_03297.t1
contig011	maker	mRNA	304661	306526	.	-	.	ID=MALK_03297.t1;Parent=MALK_03297
contig011	maker	exon	304661	306526	.	-	.	ID=MALK_03297.t1.e1;Parent=MALK_03297.t1
contig011	AUGUSTUS	gene	306880	309126	0.98	+	.	ID=MALK_03298;prediction_source=augustus:contig011.g1688.t1
contig011	AUGUSTUS	CDS	306880	309126	0.98	+	0	ID=MALK_03298.t1.c1;Parent=MALK_03298.t1
contig011	AUGUSTUS	mRNA	306880	309126	0.98	+	.	ID=MALK_03298.t1;Parent=MALK_03298
contig011	AUGUSTUS	exon	306880	309126	0.98	+	.	ID=MALK_03298.t1.e1;Parent=MALK_03298.t1
contig011	AUGUSTUS	gene	309135	309761	0.53	-	.	ID=MALK_03299;prediction_source=augustus:contig011.g1689.t1

contig011	AUGUSTUS	CDS	309135	309761	0.53	-	0	ID=MALK_03299.t1.c1;Parent=MALK_03299.t1
contig011	AUGUSTUS	mRNA	309135	309761	0.53	-	.	ID=MALK_03299.t1;Parent=MALK_03299
contig011	AUGUSTUS	exon	309135	309761	0.53	-	.	ID=MALK_03299.t1.e1;Parent=MALK_03299.t1
contig011	maker	gene	309871	311256	.	+	.	ID=MALK_03300;prediction_source=maker_MRET:augustus_masked-contig011-processed-gene-3.7-mRNA-1
contig011	maker	CDS	309871	311256	.	+	0	ID=MALK_03300.t1.c1;Parent=MALK_03300.t1
contig011	maker	mRNA	309871	311256	.	+	.	ID=MALK_03300.t1;Parent=MALK_03300
contig011	maker	exon	309871	311256	.	+	.	ID=MALK_03300.t1.e1;Parent=MALK_03300.t1
contig011	AUGUSTUS	gene	311341	312672	0.66	-	.	ID=MALK_03301;prediction_source=braker_MRET:g3641.t1
contig011	AUGUSTUS	CDS	312500	312672	0.76	-	0	ID=MALK_03301.t1.c3;Parent=MALK_03301.t1
contig011	AUGUSTUS	CDS	311383	312425	0.76	-	0	ID=MALK_03301.t1.c2;Parent=MALK_03301.t1
contig011	AUGUSTUS	CDS	311341	311351	0.76	-	0	ID=MALK_03301.t1.c1;Parent=MALK_03301.t1
contig011	AUGUSTUS	mRNA	311341	312672	0.66	-	.	ID=MALK_03301.t1;Parent=MALK_03301
contig011	AUGUSTUS	exon	312500	312672	.	-	.	ID=MALK_03301.t1.e3;Parent=MALK_03301.t1
contig011	AUGUSTUS	exon	311383	312425	.	-	.	ID=MALK_03301.t1.e2;Parent=MALK_03301.t1
contig011	AUGUSTUS	exon	311341	311351	.	-	.	ID=MALK_03301.t1.e1;Parent=MALK_03301.t1
contig011	maker	gene	312717	314192	.	+	.	ID=MALK_03302;prediction_source=maker_MRET:augustus_masked-contig011-processed-gene-3.8-mRNA-1
contig011	maker	CDS	312717	314192	.	+	0	ID=MALK_03302.t1.c1;Parent=MALK_03302.t1
contig011	maker	mRNA	312717	314192	.	+	.	ID=MALK_03302.t1;Parent=MALK_03302
contig011	maker	exon	312717	314192	.	+	.	ID=MALK_03302.t1.e1;Parent=MALK_03302.t1
contig011	AUGUSTUS	gene	314245	316677	0.63	-	.	ID=MALK_03303;prediction_source=braker_MRET:g3643.t1
contig011	AUGUSTUS	CDS	314245	316677	0.63	-	0	ID=MALK_03303.t1.c1;Parent=MALK_03303.t1
contig011	AUGUSTUS	mRNA	314245	316677	0.63	-	.	ID=MALK_03303.t1;Parent=MALK_03303
contig011	AUGUSTUS	exon	314245	316677	.	-	.	ID=MALK_03303.t1.e1;Parent=MALK_03303.t1
contig011	AUGUSTUS	gene	316908	319108	0.27	-	.	ID=MALK_03304;prediction_source=braker_MRET:g3644.t1
contig011	AUGUSTUS	CDS	317078	319108	0.41	-	0	ID=MALK_03304.t1.c2;Parent=MALK_03304.t1
contig011	AUGUSTUS	CDS	316908	317027	0.41	-	0	ID=MALK_03304.t1.c1;Parent=MALK_03304.t1
contig011	AUGUSTUS	mRNA	316908	319108	0.27	-	.	ID=MALK_03304.t1;Parent=MALK_03304
contig011	AUGUSTUS	exon	317078	319108	.	-	.	ID=MALK_03304.t1.e2;Parent=MALK_03304.t1
contig011	AUGUSTUS	exon	316908	317027	.	-	.	ID=MALK_03304.t1.e1;Parent=MALK_03304.t1
contig011	AUGUSTUS	gene	319263	319836	1	+	.	ID=MALK_03305;prediction_source=braker_MRET:g3645.t1
contig011	AUGUSTUS	CDS	319263	319279	1	+	0	ID=MALK_03305.t1.c1;Parent=MALK_03305.t1
contig011	AUGUSTUS	CDS	319314	319836	1	+	0	ID=MALK_03305.t1.c2;Parent=MALK_03305.t1
contig011	AUGUSTUS	mRNA	319263	319836	1	+	.	ID=MALK_03305.t1;Parent=MALK_03305
contig011	AUGUSTUS	exon	319263	319279	.	+	.	ID=MALK_03305.t1.e1;Parent=MALK_03305.t1
contig011	AUGUSTUS	exon	319314	319836	.	+	.	ID=MALK_03305.t1.e2;Parent=MALK_03305.t1
contig011	AUGUSTUS	gene	320436	322648	0.99	+	.	ID=MALK_03306;prediction_source=braker_MRET:g3646.t1
contig011	AUGUSTUS	CDS	320436	320438	1	+	0	ID=MALK_03306.t1.c1;Parent=MALK_03306.t1
contig011	AUGUSTUS	CDS	320508	320546	1	+	0	ID=MALK_03306.t1.c2;Parent=MALK_03306.t1
contig011	AUGUSTUS	CDS	320585	322648	1	+	0	ID=MALK_03306.t1.c3;Parent=MALK_03306.t1
contig011	AUGUSTUS	mRNA	320436	322648	0.99	+	.	ID=MALK_03306.t1;Parent=MALK_03306
contig011	AUGUSTUS	exon	320436	320438	.	+	.	ID=MALK_03306.t1.e1;Parent=MALK_03306.t1
contig011	AUGUSTUS	exon	320508	320546	.	+	.	ID=MALK_03306.t1.e2;Parent=MALK_03306.t1
contig011	AUGUSTUS	exon	320585	322648	.	+	.	ID=MALK_03306.t1.e3;Parent=MALK_03306.t1

contig011	AUGUSTUS	gene	322783	325236	0.92	+	.	ID=MALK_03307;prediction_source=braker_MRET:g3647.t1
contig011	AUGUSTUS	CDS	322783	325236	0.92	+	0	ID=MALK_03307.t1.c1;Parent=MALK_03307.t1
contig011	AUGUSTUS	mRNA	322783	325236	0.92	+	.	ID=MALK_03307.t1;Parent=MALK_03307
contig011	AUGUSTUS	exon	322783	325236	.	+	.	ID=MALK_03307.t1.e1;Parent=MALK_03307.t1
contig011	AUGUSTUS	gene	325267	325886	0.74	-	.	ID=MALK_03308;prediction_source=braker_MRET:g3648.t1
contig011	AUGUSTUS	CDS	325782	325886	0.91	-	0	ID=MALK_03308.t1.c3;Parent=MALK_03308.t1
contig011	AUGUSTUS	CDS	325358	325754	0.91	-	0	ID=MALK_03308.t1.c2;Parent=MALK_03308.t1
contig011	AUGUSTUS	CDS	325267	325307	0.91	-	0	ID=MALK_03308.t1.c1;Parent=MALK_03308.t1
contig011	AUGUSTUS	mRNA	325267	325886	0.74	-	.	ID=MALK_03308.t1;Parent=MALK_03308
contig011	AUGUSTUS	exon	325782	325886	.	-	.	ID=MALK_03308.t1.e3;Parent=MALK_03308.t1
contig011	AUGUSTUS	exon	325358	325754	.	-	.	ID=MALK_03308.t1.e2;Parent=MALK_03308.t1
contig011	AUGUSTUS	exon	325267	325307	.	-	.	ID=MALK_03308.t1.e1;Parent=MALK_03308.t1
contig011	AUGUSTUS	gene	326041	327315	0.99	-	.	ID=MALK_03309;prediction_source=augustus:contig011.g1698.t1
contig011	AUGUSTUS	CDS	326041	327315	0.99	-	0	ID=MALK_03309.t1.c1;Parent=MALK_03309.t1
contig011	AUGUSTUS	mRNA	326041	327315	0.99	-	.	ID=MALK_03309.t1;Parent=MALK_03309
contig011	AUGUSTUS	exon	326041	327315	0.99	-	.	ID=MALK_03309.t1.e1;Parent=MALK_03309.t1
contig011	AUGUSTUS	gene	327818	328180	1	+	.	ID=MALK_03310;prediction_source=augustus:contig011.g1699.t1
contig011	AUGUSTUS	CDS	327818	328180	1	+	0	ID=MALK_03310.t1.c1;Parent=MALK_03310.t1
contig011	AUGUSTUS	mRNA	327818	328180	1	+	.	ID=MALK_03310.t1;Parent=MALK_03310
contig011	AUGUSTUS	exon	327818	328180	1	+	.	ID=MALK_03310.t1.e1;Parent=MALK_03310.t1
contig011	maker	gene	328497	328940	.	-	.	ID=MALK_03311;prediction_source=maker_MRET:augustus_masked-contig011-processed-gene-3.39-mRNA-1
contig011	maker	CDS	328497	328940	.	-	0	ID=MALK_03311.t1.c1;Parent=MALK_03311.t1
contig011	maker	mRNA	328497	328940	.	-	.	ID=MALK_03311.t1;Parent=MALK_03311
contig011	maker	exon	328497	328940	.	-	.	ID=MALK_03311.t1.e1;Parent=MALK_03311.t1
contig011	AUGUSTUS	gene	329321	330951	0.75	+	.	ID=MALK_03312;prediction_source=braker_MRET:g3652.t1
contig011	AUGUSTUS	CDS	329321	329341	0.75	+	0	ID=MALK_03312.t1.c1;Parent=MALK_03312.t1
contig011	AUGUSTUS	CDS	329383	330951	0.75	+	0	ID=MALK_03312.t1.c2;Parent=MALK_03312.t1
contig011	AUGUSTUS	mRNA	329321	330951	0.75	+	.	ID=MALK_03312.t1;Parent=MALK_03312
contig011	AUGUSTUS	exon	329321	329341	.	+	.	ID=MALK_03312.t1.e1;Parent=MALK_03312.t1
contig011	AUGUSTUS	exon	329383	330951	.	+	.	ID=MALK_03312.t1.e2;Parent=MALK_03312.t1
contig011	AUGUSTUS	gene	331227	332170	0.48	+	.	ID=MALK_03313;prediction_source=braker_MRET:g3653.t1
contig011	AUGUSTUS	CDS	331227	331258	1	+	0	ID=MALK_03313.t1.c1;Parent=MALK_03313.t1
contig011	AUGUSTUS	CDS	331291	332170	1	+	0	ID=MALK_03313.t1.c2;Parent=MALK_03313.t1
contig011	AUGUSTUS	mRNA	331227	332170	0.48	+	.	ID=MALK_03313.t1;Parent=MALK_03313
contig011	AUGUSTUS	exon	331227	331258	.	+	.	ID=MALK_03313.t1.e1;Parent=MALK_03313.t1
contig011	AUGUSTUS	exon	331291	332170	.	+	.	ID=MALK_03313.t1.e2;Parent=MALK_03313.t1
contig011	AUGUSTUS	gene	332200	332949	0.8	-	.	ID=MALK_03314;prediction_source=braker_MRET:g3654.t1
contig011	AUGUSTUS	CDS	332200	332949	0.8	-	0	ID=MALK_03314.t1.c1;Parent=MALK_03314.t1
contig011	AUGUSTUS	mRNA	332200	332949	0.8	-	.	ID=MALK_03314.t1;Parent=MALK_03314
contig011	AUGUSTUS	exon	332200	332949	.	-	.	ID=MALK_03314.t1.e1;Parent=MALK_03314.t1
contig011	maker	gene	333068	334552	.	+	.	ID=MALK_03315;prediction_source=maker_MRET:augustus_masked-contig011-processed-gene-3.12-mRNA-1
contig011	maker	CDS	333068	334552	.	+	0	ID=MALK_03315.t1.c1;Parent=MALK_03315.t1
contig011	maker	mRNA	333068	334552	.	+	.	ID=MALK_03315.t1;Parent=MALK_03315

contig011	maker	exon	333068	334552	.	+	.	ID=MALK_03315.t1.e1;Parent=MALK_03315.t1
contig011	AUGUSTUS	gene	334566	335798	1	+	.	ID=MALK_03316;prediction_source=braker_MRET:g3656.t1
contig011	AUGUSTUS	CDS	334566	335798	1	+	0	ID=MALK_03316.t1.c1;Parent=MALK_03316.t1
contig011	AUGUSTUS	mRNA	334566	335798	1	+	.	ID=MALK_03316.t1;Parent=MALK_03316
contig011	AUGUSTUS	exon	334566	335798	.	+	.	ID=MALK_03316.t1.e1;Parent=MALK_03316.t1
contig011	AUGUSTUS	gene	335802	336992	0.97	-	.	ID=MALK_03317;prediction_source=augustus:contig011.g1706.t1
contig011	AUGUSTUS	CDS	335802	336992	0.97	-	0	ID=MALK_03317.t1.c1;Parent=MALK_03317.t1
contig011	AUGUSTUS	mRNA	335802	336992	0.97	-	.	ID=MALK_03317.t1;Parent=MALK_03317
contig011	AUGUSTUS	exon	335802	336992	0.97	-	.	ID=MALK_03317.t1.e1;Parent=MALK_03317.t1
contig011	AUGUSTUS	gene	337145	337550	0.85	-	.	ID=MALK_03318;prediction_source=braker_MRET:g3658.t1
contig011	AUGUSTUS	CDS	337537	337550	0.87	-	0	ID=MALK_03318.t1.c3;Parent=MALK_03318.t1
contig011	AUGUSTUS	CDS	337433	337509	0.87	-	0	ID=MALK_03318.t1.c2;Parent=MALK_03318.t1
contig011	AUGUSTUS	CDS	337145	337404	0.87	-	0	ID=MALK_03318.t1.c1;Parent=MALK_03318.t1
contig011	AUGUSTUS	mRNA	337145	337550	0.85	-	.	ID=MALK_03318.t1;Parent=MALK_03318
contig011	AUGUSTUS	exon	337537	337550	.	-	.	ID=MALK_03318.t1.e3;Parent=MALK_03318.t1
contig011	AUGUSTUS	exon	337433	337509	.	-	.	ID=MALK_03318.t1.e2;Parent=MALK_03318.t1
contig011	AUGUSTUS	exon	337145	337404	.	-	.	ID=MALK_03318.t1.e1;Parent=MALK_03318.t1
contig011	AUGUSTUS	gene	337668	339206	1	+	.	ID=MALK_03319;prediction_source=braker_MRET:g3659.t1
contig011	AUGUSTUS	CDS	337668	339206	1	+	0	ID=MALK_03319.t1.c1;Parent=MALK_03319.t1
contig011	AUGUSTUS	mRNA	337668	339206	1	+	.	ID=MALK_03319.t1;Parent=MALK_03319
contig011	AUGUSTUS	exon	337668	339206	.	+	.	ID=MALK_03319.t1.e1;Parent=MALK_03319.t1
contig011	AUGUSTUS	gene	339210	340138	0.52	-	.	ID=MALK_03320;prediction_source=braker_MRET:g3660.t1
contig011	AUGUSTUS	CDS	339965	340138	0.59	-	0	ID=MALK_03320.t1.c3;Parent=MALK_03320.t1
contig011	AUGUSTUS	CDS	339663	339926	0.59	-	0	ID=MALK_03320.t1.c2;Parent=MALK_03320.t1
contig011	AUGUSTUS	CDS	339210	339605	0.59	-	0	ID=MALK_03320.t1.c1;Parent=MALK_03320.t1
contig011	AUGUSTUS	mRNA	339210	340138	0.52	-	.	ID=MALK_03320.t1;Parent=MALK_03320
contig011	AUGUSTUS	exon	339965	340138	.	-	.	ID=MALK_03320.t1.e3;Parent=MALK_03320.t1
contig011	AUGUSTUS	exon	339663	339926	.	-	.	ID=MALK_03320.t1.e2;Parent=MALK_03320.t1
contig011	AUGUSTUS	exon	339210	339605	.	-	.	ID=MALK_03320.t1.e1;Parent=MALK_03320.t1
contig011	maker	gene	340334	341770	.	+	.	ID=MALK_03321;prediction_source=maker_MRET:augustus_masked-contig011-processed-gene-3.15-mRNA-1
contig011	maker	CDS	340334	341770	.	+	0	ID=MALK_03321.t1.c1;Parent=MALK_03321.t1
contig011	maker	mRNA	340334	341770	.	+	.	ID=MALK_03321.t1;Parent=MALK_03321
contig011	maker	exon	340334	341770	.	+	.	ID=MALK_03321.t1.e1;Parent=MALK_03321.t1
contig011	AUGUSTUS	gene	341889	343418	0.98	+	.	ID=MALK_03322;prediction_source=braker_MRET:g3662.t1
contig011	AUGUSTUS	CDS	341889	343418	0.98	+	0	ID=MALK_03322.t1.c1;Parent=MALK_03322.t1
contig011	AUGUSTUS	mRNA	341889	343418	0.98	+	.	ID=MALK_03322.t1;Parent=MALK_03322
contig011	AUGUSTUS	exon	341889	343418	.	+	.	ID=MALK_03322.t1.e1;Parent=MALK_03322.t1
contig011	AUGUSTUS	gene	343437	344435	0.59	-	.	ID=MALK_03323;prediction_source=augustus:contig011.g1712.t1
contig011	AUGUSTUS	CDS	343437	344435	0.59	-	0	ID=MALK_03323.t1.c1;Parent=MALK_03323.t1
contig011	AUGUSTUS	mRNA	343437	344435	0.59	-	.	ID=MALK_03323.t1;Parent=MALK_03323
contig011	AUGUSTUS	exon	343437	344435	0.59	-	.	ID=MALK_03323.t1.e1;Parent=MALK_03323.t1
contig011	AUGUSTUS	gene	344554	345084	0.98	+	.	ID=MALK_03324;prediction_source=augustus:contig011.g1713.t1
contig011	AUGUSTUS	CDS	344554	345084	0.98	+	0	ID=MALK_03324.t1.c1;Parent=MALK_03324.t1

contig011	AUGUSTUS	mRNA	344554	345084	0.98	+	.	ID=MALK_03324.t1;Parent=MALK_03324
contig011	AUGUSTUS	exon	344554	345084	0.98	+	.	ID=MALK_03324.t1.e1;Parent=MALK_03324.t1
contig011	AUGUSTUS	gene	345769	347441	0.85	+	.	ID=MALK_03325;prediction_source=braker_MRET:g3665.t1
contig011	AUGUSTUS	CDS	345769	345838	0.99	+	0	ID=MALK_03325.t1.c1;Parent=MALK_03325.t1
contig011	AUGUSTUS	CDS	345871	347441	0.99	+	0	ID=MALK_03325.t1.c2;Parent=MALK_03325.t1
contig011	AUGUSTUS	mRNA	345769	347441	0.85	+	.	ID=MALK_03325.t1;Parent=MALK_03325
contig011	AUGUSTUS	exon	345769	345838	.	+	.	ID=MALK_03325.t1.e1;Parent=MALK_03325.t1
contig011	AUGUSTUS	exon	345871	347441	.	+	.	ID=MALK_03325.t1.e2;Parent=MALK_03325.t1
contig011	AUGUSTUS	gene	347628	348197	1	-	.	ID=MALK_03326;prediction_source=braker_MRET:g3666.t1
contig011	AUGUSTUS	CDS	348195	348197	1	-	0	ID=MALK_03326.t1.c4;Parent=MALK_03326.t1
contig011	AUGUSTUS	CDS	347826	348107	1	-	0	ID=MALK_03326.t1.c3;Parent=MALK_03326.t1
contig011	AUGUSTUS	CDS	347726	347784	1	-	0	ID=MALK_03326.t1.c2;Parent=MALK_03326.t1
contig011	AUGUSTUS	CDS	347628	347682	1	-	0	ID=MALK_03326.t1.c1;Parent=MALK_03326.t1
contig011	AUGUSTUS	mRNA	347628	348197	1	-	.	ID=MALK_03326.t1;Parent=MALK_03326
contig011	AUGUSTUS	exon	348195	348197	.	-	.	ID=MALK_03326.t1.e4;Parent=MALK_03326.t1
contig011	AUGUSTUS	exon	347826	348107	.	-	.	ID=MALK_03326.t1.e3;Parent=MALK_03326.t1
contig011	AUGUSTUS	exon	347726	347784	.	-	.	ID=MALK_03326.t1.e2;Parent=MALK_03326.t1
contig011	AUGUSTUS	exon	347628	347682	.	-	.	ID=MALK_03326.t1.e1;Parent=MALK_03326.t1
contig011	AUGUSTUS	gene	348596	348960	0.71	-	.	ID=MALK_03327;prediction_source=braker_MRET:g3667.t1
contig011	AUGUSTUS	CDS	348751	348960	0.71	-	0	ID=MALK_03327.t1.c3;Parent=MALK_03327.t1
contig011	AUGUSTUS	CDS	348660	348713	0.71	-	0	ID=MALK_03327.t1.c2;Parent=MALK_03327.t1
contig011	AUGUSTUS	CDS	348596	348616	0.71	-	0	ID=MALK_03327.t1.c1;Parent=MALK_03327.t1
contig011	AUGUSTUS	mRNA	348596	348960	0.71	-	.	ID=MALK_03327.t1;Parent=MALK_03327
contig011	AUGUSTUS	exon	348751	348960	.	-	.	ID=MALK_03327.t1.e3;Parent=MALK_03327.t1
contig011	AUGUSTUS	exon	348660	348713	.	-	.	ID=MALK_03327.t1.e2;Parent=MALK_03327.t1
contig011	AUGUSTUS	exon	348596	348616	.	-	.	ID=MALK_03327.t1.e1;Parent=MALK_03327.t1
contig011	maker	gene	349145	350071	.	+	.	ID=MALK_03328;prediction_source=maker_MRET:augustus_masked-contig011-processed-gene-3.19-mRNA-1
contig011	maker	CDS	349145	350071	.	+	0	ID=MALK_03328.t1.c1;Parent=MALK_03328.t1
contig011	maker	mRNA	349145	350071	.	+	.	ID=MALK_03328.t1;Parent=MALK_03328
contig011	maker	exon	349145	350071	.	+	.	ID=MALK_03328.t1.e1;Parent=MALK_03328.t1
contig011	AUGUSTUS	gene	350133	351635	0.92	+	.	ID=MALK_03329;prediction_source=braker_MRET:g3669.t1
contig011	AUGUSTUS	CDS	350133	351635	0.92	+	0	ID=MALK_03329.t1.c1;Parent=MALK_03329.t1
contig011	AUGUSTUS	mRNA	350133	351635	0.92	+	.	ID=MALK_03329.t1;Parent=MALK_03329
contig011	AUGUSTUS	exon	350133	351635	.	+	.	ID=MALK_03329.t1.e1;Parent=MALK_03329.t1
contig011	maker	gene	351649	354765	.	-	.	ID=MALK_03330;prediction_source=maker_MRET:augustus_masked-contig011-processed-gene-3.45-mRNA-1
contig011	maker	CDS	351649	354765	.	-	0	ID=MALK_03330.t1.c1;Parent=MALK_03330.t1
contig011	maker	mRNA	351649	354765	.	-	.	ID=MALK_03330.t1;Parent=MALK_03330
contig011	maker	exon	351649	354765	.	-	.	ID=MALK_03330.t1.e1;Parent=MALK_03330.t1
contig011	AUGUSTUS	gene	355352	357917	0.66	+	.	ID=MALK_03331;prediction_source=augustus:contig011.g1719.t1
contig011	AUGUSTUS	CDS	355352	355739	0.77	+	0	ID=MALK_03331.t1.c1;Parent=MALK_03331.t1
contig011	AUGUSTUS	CDS	355879	357917	0.77	+	0	ID=MALK_03331.t1.c2;Parent=MALK_03331.t1
contig011	AUGUSTUS	mRNA	355352	357917	0.66	+	.	ID=MALK_03331.t1;Parent=MALK_03331
contig011	AUGUSTUS	exon	355352	355739	0.77	+	.	ID=MALK_03331.t1.e1;Parent=MALK_03331.t1

contig011	AUGUSTUS	exon	355879	357917	0.77	+	.	ID=MALK_03331.t1.e2;Parent=MALK_03331.t1
contig011	maker	gene	357957	358823	.	+	.	ID=MALK_03332;prediction_source=maker_MRET:augustus_masked-contig011-processed-gene-3.22-mRNA-1
contig011	maker	CDS	357957	358823	.	+	0	ID=MALK_03332.t1.c1;Parent=MALK_03332.t1
contig011	maker	mRNA	357957	358823	.	+	.	ID=MALK_03332.t1;Parent=MALK_03332
contig011	maker	exon	357957	358823	.	+	.	ID=MALK_03332.t1.e1;Parent=MALK_03332.t1
contig011	AUGUSTUS	gene	359356	361516	0.12	+	.	ID=MALK_03333;prediction_source=braker_MRET:g3673.t1
contig011	AUGUSTUS	CDS	359356	361016	1	+	0	ID=MALK_03333.t1.c1;Parent=MALK_03333.t1
contig011	AUGUSTUS	CDS	361096	361166	1	+	0	ID=MALK_03333.t1.c2;Parent=MALK_03333.t1
contig011	AUGUSTUS	CDS	361269	361404	1	+	0	ID=MALK_03333.t1.c3;Parent=MALK_03333.t1
contig011	AUGUSTUS	CDS	361504	361516	1	+	0	ID=MALK_03333.t1.c4;Parent=MALK_03333.t1
contig011	AUGUSTUS	mRNA	359356	361516	0.12	+	.	ID=MALK_03333.t1;Parent=MALK_03333
contig011	AUGUSTUS	exon	359356	361016	.	+	.	ID=MALK_03333.t1.e1;Parent=MALK_03333.t1
contig011	AUGUSTUS	exon	361096	361166	.	+	.	ID=MALK_03333.t1.e2;Parent=MALK_03333.t1
contig011	AUGUSTUS	exon	361269	361404	.	+	.	ID=MALK_03333.t1.e3;Parent=MALK_03333.t1
contig011	AUGUSTUS	exon	361504	361516	.	+	.	ID=MALK_03333.t1.e4;Parent=MALK_03333.t1
contig011	maker	gene	361526	362341	.	+	.	ID=MALK_03334;prediction_source=maker_MRET:augustus_masked-contig011-processed-gene-3.24-mRNA-1
contig011	maker	CDS	361526	362341	.	+	0	ID=MALK_03334.t1.c1;Parent=MALK_03334.t1
contig011	maker	mRNA	361526	362341	.	+	.	ID=MALK_03334.t1;Parent=MALK_03334
contig011	maker	exon	361526	362341	.	+	.	ID=MALK_03334.t1.e1;Parent=MALK_03334.t1
contig011	AUGUSTUS	gene	362624	365482	0.75	+	.	ID=MALK_03335;prediction_source=augustus:contig011.g1723.t1
contig011	AUGUSTUS	CDS	362624	365482	0.75	+	0	ID=MALK_03335.t1.c1;Parent=MALK_03335.t1
contig011	AUGUSTUS	mRNA	362624	365482	0.75	+	.	ID=MALK_03335.t1;Parent=MALK_03335
contig011	AUGUSTUS	exon	362624	365482	0.75	+	.	ID=MALK_03335.t1.e1;Parent=MALK_03335.t1
contig011	AUGUSTUS	gene	365485	367664	0.93	-	.	ID=MALK_03336;prediction_source=braker_MRET:g3676.t1
contig011	AUGUSTUS	CDS	367620	367664	0.93	-	0	ID=MALK_03336.t1.c2;Parent=MALK_03336.t1
contig011	AUGUSTUS	CDS	365485	367590	0.93	-	0	ID=MALK_03336.t1.c1;Parent=MALK_03336.t1
contig011	AUGUSTUS	mRNA	365485	367664	0.93	-	.	ID=MALK_03336.t1;Parent=MALK_03336
contig011	AUGUSTUS	exon	367620	367664	.	-	.	ID=MALK_03336.t1.e2;Parent=MALK_03336.t1
contig011	AUGUSTUS	exon	365485	367590	.	-	.	ID=MALK_03336.t1.e1;Parent=MALK_03336.t1
contig011	AUGUSTUS	gene	367975	368625	0.93	-	.	ID=MALK_03337;prediction_source=augustus:contig011.g1726.t1
contig011	AUGUSTUS	CDS	367975	368625	0.93	-	0	ID=MALK_03337.t1.c1;Parent=MALK_03337.t1
contig011	AUGUSTUS	mRNA	367975	368625	0.93	-	.	ID=MALK_03337.t1;Parent=MALK_03337
contig011	AUGUSTUS	exon	367975	368625	0.93	-	.	ID=MALK_03337.t1.e1;Parent=MALK_03337.t1
contig011	maker	gene	368779	369750	.	+	.	ID=MALK_03338;prediction_source=maker_MRET:augustus_masked-contig011-processed-gene-3.26-mRNA-1
contig011	maker	CDS	368779	369750	.	+	0	ID=MALK_03338.t1.c1;Parent=MALK_03338.t1
contig011	maker	mRNA	368779	369750	.	+	.	ID=MALK_03338.t1;Parent=MALK_03338
contig011	maker	exon	368779	369750	.	+	.	ID=MALK_03338.t1.e1;Parent=MALK_03338.t1
contig011	maker	gene	369845	370282	.	-	.	ID=MALK_03339;prediction_source=maker_MRET:augustus_masked-contig011-processed-gene-3.48-mRNA-1
contig011	maker	CDS	369845	370282	.	-	0	ID=MALK_03339.t1.c1;Parent=MALK_03339.t1
contig011	maker	mRNA	369845	370282	.	-	.	ID=MALK_03339.t1;Parent=MALK_03339
contig011	maker	exon	369845	370282	.	-	.	ID=MALK_03339.t1.e1;Parent=MALK_03339.t1
contig011	maker	gene	370430	377287	.	+	.	ID=MALK_03340;prediction_source=maker_MRET:augustus_masked-contig011-processed-gene-3.27-mRNA-1
contig011	maker	CDS	370430	377287	.	+	0	ID=MALK_03340.t1.c1;Parent=MALK_03340.t1

contig011	maker	mRNA	370430	377287	.	+	.	ID=MALK_03340.t1;Parent=MALK_03340
contig011	maker	exon	370430	377287	.	+	.	ID=MALK_03340.t1.e1;Parent=MALK_03340.t1
contig011	AUGUSTUS	gene	377314	378465	0.93	+	.	ID=MALK_03341;prediction_source=braker_MRET:g3681.t1
contig011	AUGUSTUS	CDS	377314	378465	0.93	+	0	ID=MALK_03341.t1.c1;Parent=MALK_03341.t1
contig011	AUGUSTUS	mRNA	377314	378465	0.93	+	.	ID=MALK_03341.t1;Parent=MALK_03341
contig011	AUGUSTUS	exon	377314	378465	.	+	.	ID=MALK_03341.t1.e1;Parent=MALK_03341.t1
contig011	AUGUSTUS	gene	378941	380006	0.34	-	.	ID=MALK_03342;prediction_source=braker_MRET:g3682.t1
contig011	AUGUSTUS	CDS	380004	380006	0.36	-	0	ID=MALK_03342.t1.c2;Parent=MALK_03342.t1
contig011	AUGUSTUS	CDS	378941	379948	0.36	-	0	ID=MALK_03342.t1.c1;Parent=MALK_03342.t1
contig011	AUGUSTUS	mRNA	378941	380006	0.34	-	.	ID=MALK_03342.t1;Parent=MALK_03342
contig011	AUGUSTUS	exon	380004	380006	.	-	.	ID=MALK_03342.t1.e2;Parent=MALK_03342.t1
contig011	AUGUSTUS	exon	378941	379948	.	-	.	ID=MALK_03342.t1.e1;Parent=MALK_03342.t1
contig011	AUGUSTUS	gene	380183	381412	0.49	+	.	ID=MALK_03343;prediction_source=braker_MRET:g3683.t1
contig011	AUGUSTUS	CDS	380183	381412	0.49	+	0	ID=MALK_03343.t1.c1;Parent=MALK_03343.t1
contig011	AUGUSTUS	mRNA	380183	381412	0.49	+	.	ID=MALK_03343.t1;Parent=MALK_03343
contig011	AUGUSTUS	exon	380183	381412	.	+	.	ID=MALK_03343.t1.e1;Parent=MALK_03343.t1
contig011	AUGUSTUS	gene	381707	383053	0.91	+	.	ID=MALK_03344;prediction_source=braker_MRET:g3684.t1
contig011	AUGUSTUS	CDS	381707	383053	0.91	+	0	ID=MALK_03344.t1.c1;Parent=MALK_03344.t1
contig011	AUGUSTUS	mRNA	381707	383053	0.91	+	.	ID=MALK_03344.t1;Parent=MALK_03344
contig011	AUGUSTUS	exon	381707	383053	.	+	.	ID=MALK_03344.t1.e1;Parent=MALK_03344.t1
contig011	maker	gene	384031	386387	.	+	.	ID=MALK_03345;prediction_source=maker_MRET:augustus_masked-contig011-processed-gene-3.30-mRNA-1
contig011	maker	CDS	384031	384678	.	+	0	ID=MALK_03345.t1.c1;Parent=MALK_03345.t1
contig011	maker	CDS	384988	385191	.	+	0	ID=MALK_03345.t1.c2;Parent=MALK_03345.t1
contig011	maker	CDS	385329	386387	.	+	0	ID=MALK_03345.t1.c3;Parent=MALK_03345.t1
contig011	maker	mRNA	384031	386387	.	+	.	ID=MALK_03345.t1;Parent=MALK_03345
contig011	maker	exon	384031	384678	.	+	.	ID=MALK_03345.t1.e1;Parent=MALK_03345.t1
contig011	maker	exon	384988	385191	.	+	.	ID=MALK_03345.t1.e2;Parent=MALK_03345.t1
contig011	maker	exon	385329	386387	.	+	.	ID=MALK_03345.t1.e3;Parent=MALK_03345.t1
contig011	AUGUSTUS	gene	386517	386922	0.4	-	.	ID=MALK_03346;prediction_source=braker_MRET:g3686.t1
contig011	AUGUSTUS	CDS	386830	386922	0.4	-	0	ID=MALK_03346.t1.c2;Parent=MALK_03346.t1
contig011	AUGUSTUS	CDS	386517	386792	0.4	-	0	ID=MALK_03346.t1.c1;Parent=MALK_03346.t1
contig011	AUGUSTUS	mRNA	386517	386922	0.4	-	.	ID=MALK_03346.t1;Parent=MALK_03346
contig011	AUGUSTUS	exon	386830	386922	.	-	.	ID=MALK_03346.t1.e2;Parent=MALK_03346.t1
contig011	AUGUSTUS	exon	386517	386792	.	-	.	ID=MALK_03346.t1.e1;Parent=MALK_03346.t1
contig011	AUGUSTUS	gene	387240	389567	1	-	.	ID=MALK_03347;prediction_source=augustus:contig011.g1738.t1
contig011	AUGUSTUS	CDS	387240	389567	1	-	0	ID=MALK_03347.t1.c1;Parent=MALK_03347.t1
contig011	AUGUSTUS	mRNA	387240	389567	1	-	.	ID=MALK_03347.t1;Parent=MALK_03347
contig011	AUGUSTUS	exon	387240	389567	1	-	.	ID=MALK_03347.t1.e1;Parent=MALK_03347.t1
contig011	maker	gene	389859	392762	.	+	.	ID=MALK_03348;prediction_source=maker_MRET:augustus_masked-contig011-processed-gene-3.31-mRNA-1
contig011	maker	CDS	389859	392762	.	+	0	ID=MALK_03348.t1.c1;Parent=MALK_03348.t1
contig011	maker	mRNA	389859	392762	.	+	.	ID=MALK_03348.t1;Parent=MALK_03348
contig011	maker	exon	389859	392762	.	+	.	ID=MALK_03348.t1.e1;Parent=MALK_03348.t1
contig011	AUGUSTUS	gene	392768	393738	0.97	-	.	ID=MALK_03349;prediction_source=braker_MRET:g3689.t1

contig011	AUGUSTUS	CDS	393328	393738	0.97	-	0	ID=MALK_03349.t1.c2;Parent=MALK_03349.t1
contig011	AUGUSTUS	CDS	392768	393196	0.97	-	0	ID=MALK_03349.t1.c1;Parent=MALK_03349.t1
contig011	AUGUSTUS	mRNA	392768	393738	0.97	-	.	ID=MALK_03349.t1;Parent=MALK_03349
contig011	AUGUSTUS	exon	393328	393738	.	-	.	ID=MALK_03349.t1.e2;Parent=MALK_03349.t1
contig011	AUGUSTUS	exon	392768	393196	.	-	.	ID=MALK_03349.t1.e1;Parent=MALK_03349.t1
contig011	maker	gene	393857	395830	.	+	.	ID=MALK_03350;prediction_source=maker_MRET:augustus_masked-contig011-processed-gene-4.0-mRNA-1
contig011	maker	CDS	393857	395830	.	+	0	ID=MALK_03350.t1.c1;Parent=MALK_03350.t1
contig011	maker	mRNA	393857	395830	.	+	.	ID=MALK_03350.t1;Parent=MALK_03350
contig011	maker	exon	393857	395830	.	+	.	ID=MALK_03350.t1.e1;Parent=MALK_03350.t1
contig011	AUGUSTUS	gene	395880	398555	0.93	+	.	ID=MALK_03351;prediction_source=augustus:contig011.g1741.t1
contig011	AUGUSTUS	CDS	395880	398555	0.93	+	0	ID=MALK_03351.t1.c1;Parent=MALK_03351.t1
contig011	AUGUSTUS	mRNA	395880	398555	0.93	+	.	ID=MALK_03351.t1;Parent=MALK_03351
contig011	AUGUSTUS	exon	395880	398555	0.93	+	.	ID=MALK_03351.t1.e1;Parent=MALK_03351.t1
contig011	AUGUSTUS	gene	398883	399803	0.45	+	.	ID=MALK_03352;prediction_source=braker_MRET:g3692.t1
contig011	AUGUSTUS	CDS	398883	399484	1	+	0	ID=MALK_03352.t1.c1;Parent=MALK_03352.t1
contig011	AUGUSTUS	CDS	399517	399554	1	+	0	ID=MALK_03352.t1.c2;Parent=MALK_03352.t1
contig011	AUGUSTUS	CDS	399583	399803	1	+	0	ID=MALK_03352.t1.c3;Parent=MALK_03352.t1
contig011	AUGUSTUS	mRNA	398883	399803	0.45	+	.	ID=MALK_03352.t1;Parent=MALK_03352
contig011	AUGUSTUS	exon	398883	399484	.	+	.	ID=MALK_03352.t1.e1;Parent=MALK_03352.t1
contig011	AUGUSTUS	exon	399517	399554	.	+	.	ID=MALK_03352.t1.e2;Parent=MALK_03352.t1
contig011	AUGUSTUS	exon	399583	399803	.	+	.	ID=MALK_03352.t1.e3;Parent=MALK_03352.t1
contig011	AUGUSTUS	gene	399833	400337	0.9	-	.	ID=MALK_03353;prediction_source=braker_MRET:g3693.t1
contig011	AUGUSTUS	CDS	400268	400337	0.99	-	0	ID=MALK_03353.t1.c2;Parent=MALK_03353.t1
contig011	AUGUSTUS	CDS	399833	400230	0.99	-	0	ID=MALK_03353.t1.c1;Parent=MALK_03353.t1
contig011	AUGUSTUS	mRNA	399833	400337	0.9	-	.	ID=MALK_03353.t1;Parent=MALK_03353
contig011	AUGUSTUS	exon	400268	400337	.	-	.	ID=MALK_03353.t1.e2;Parent=MALK_03353.t1
contig011	AUGUSTUS	exon	399833	400230	.	-	.	ID=MALK_03353.t1.e1;Parent=MALK_03353.t1
contig011	AUGUSTUS	gene	401095	402174	0.93	-	.	ID=MALK_03354;prediction_source=braker_MRET:g3694.t1
contig011	AUGUSTUS	CDS	402014	402174	0.99	-	0	ID=MALK_03354.t1.c3;Parent=MALK_03354.t1
contig011	AUGUSTUS	CDS	401628	401964	0.99	-	0	ID=MALK_03354.t1.c2;Parent=MALK_03354.t1
contig011	AUGUSTUS	CDS	401095	401589	0.99	-	0	ID=MALK_03354.t1.c1;Parent=MALK_03354.t1
contig011	AUGUSTUS	mRNA	401095	402174	0.93	-	.	ID=MALK_03354.t1;Parent=MALK_03354
contig011	AUGUSTUS	exon	402014	402174	.	-	.	ID=MALK_03354.t1.e3;Parent=MALK_03354.t1
contig011	AUGUSTUS	exon	401628	401964	.	-	.	ID=MALK_03354.t1.e2;Parent=MALK_03354.t1
contig011	AUGUSTUS	exon	401095	401589	.	-	.	ID=MALK_03354.t1.e1;Parent=MALK_03354.t1
contig011	AUGUSTUS	gene	402222	403343	0.81	+	.	ID=MALK_03355;prediction_source=augustus:contig011.g1745.t1
contig011	AUGUSTUS	CDS	402222	403343	0.81	+	0	ID=MALK_03355.t1.c1;Parent=MALK_03355.t1
contig011	AUGUSTUS	mRNA	402222	403343	0.81	+	.	ID=MALK_03355.t1;Parent=MALK_03355
contig011	AUGUSTUS	exon	402222	403343	0.81	+	.	ID=MALK_03355.t1.e1;Parent=MALK_03355.t1
contig011	maker	gene	403327	406821	.	-	.	ID=MALK_03356;prediction_source=maker_MRET:augustus_masked-contig011-processed-gene-4.26-mRNA-1
contig011	maker	CDS	403327	406821	.	-	0	ID=MALK_03356.t1.c1;Parent=MALK_03356.t1
contig011	maker	mRNA	403327	406821	.	-	.	ID=MALK_03356.t1;Parent=MALK_03356
contig011	maker	exon	403327	406821	.	-	.	ID=MALK_03356.t1.e1;Parent=MALK_03356.t1

contig011	AUGUSTUS	gene	407118	408617	0.99	+	.	ID=MALK_03357;prediction_source=augustus:contig011.g1748.t1
contig011	AUGUSTUS	CDS	407118	408617	0.99	+	0	ID=MALK_03357.t1.c1;Parent=MALK_03357.t1
contig011	AUGUSTUS	mRNA	407118	408617	0.99	+	.	ID=MALK_03357.t1;Parent=MALK_03357
contig011	AUGUSTUS	exon	407118	408617	0.99	+	.	ID=MALK_03357.t1.e1;Parent=MALK_03357.t1
contig011	AUGUSTUS	gene	408771	409997	1	+	.	ID=MALK_03358;prediction_source=augustus:contig011.g1749.t1
contig011	AUGUSTUS	CDS	408771	409997	1	+	0	ID=MALK_03358.t1.c1;Parent=MALK_03358.t1
contig011	AUGUSTUS	mRNA	408771	409997	1	+	.	ID=MALK_03358.t1;Parent=MALK_03358
contig011	AUGUSTUS	exon	408771	409997	1	+	.	ID=MALK_03358.t1.e1;Parent=MALK_03358.t1
contig011	maker	gene	410187	412094	.	-	.	ID=MALK_03359;prediction_source=maker_MRET:augustus_masked-contig011-processed-gene-4.27-mRNA-1
contig011	maker	CDS	410187	412094	.	-	0	ID=MALK_03359.t1.c1;Parent=MALK_03359.t1
contig011	maker	mRNA	410187	412094	.	-	.	ID=MALK_03359.t1;Parent=MALK_03359
contig011	maker	exon	410187	412094	.	-	.	ID=MALK_03359.t1.e1;Parent=MALK_03359.t1
contig011	AUGUSTUS	gene	412191	416381	0.76	-	.	ID=MALK_03360;prediction_source=augustus:contig011.g1753.t1
contig011	AUGUSTUS	CDS	412191	416381	0.76	-	0	ID=MALK_03360.t1.c1;Parent=MALK_03360.t1
contig011	AUGUSTUS	mRNA	412191	416381	0.76	-	.	ID=MALK_03360.t1;Parent=MALK_03360
contig011	AUGUSTUS	exon	412191	416381	0.76	-	.	ID=MALK_03360.t1.e1;Parent=MALK_03360.t1
contig011	maker	gene	416416	418524	.	+	.	ID=MALK_03361;prediction_source=maker_MRET:augustus_masked-contig011-processed-gene-4.6-mRNA-1
contig011	maker	CDS	416416	418524	.	+	0	ID=MALK_03361.t1.c1;Parent=MALK_03361.t1
contig011	maker	mRNA	416416	418524	.	+	.	ID=MALK_03361.t1;Parent=MALK_03361
contig011	maker	exon	416416	418524	.	+	.	ID=MALK_03361.t1.e1;Parent=MALK_03361.t1
contig011	AUGUSTUS	gene	418517	421573	0.87	-	.	ID=MALK_03362;prediction_source=augustus:contig011.g1756.t1
contig011	AUGUSTUS	CDS	418517	421573	0.87	-	0	ID=MALK_03362.t1.c1;Parent=MALK_03362.t1
contig011	AUGUSTUS	mRNA	418517	421573	0.87	-	.	ID=MALK_03362.t1;Parent=MALK_03362
contig011	AUGUSTUS	exon	418517	421573	0.87	-	.	ID=MALK_03362.t1.e1;Parent=MALK_03362.t1
contig011	maker	gene	421826	422986	.	+	.	ID=MALK_03363;prediction_source=maker_MRET:augustus_masked-contig011-processed-gene-4.7-mRNA-1
contig011	maker	CDS	421826	422986	.	+	0	ID=MALK_03363.t1.c1;Parent=MALK_03363.t1
contig011	maker	mRNA	421826	422986	.	+	.	ID=MALK_03363.t1;Parent=MALK_03363
contig011	maker	exon	421826	422986	.	+	.	ID=MALK_03363.t1.e1;Parent=MALK_03363.t1
contig011	AUGUSTUS	gene	423215	424405	0.82	+	.	ID=MALK_03364;prediction_source=braker_MRET:g3704.t1
contig011	AUGUSTUS	CDS	423215	424405	0.82	+	0	ID=MALK_03364.t1.c1;Parent=MALK_03364.t1
contig011	AUGUSTUS	mRNA	423215	424405	0.82	+	.	ID=MALK_03364.t1;Parent=MALK_03364
contig011	AUGUSTUS	exon	423215	424405	.	+	.	ID=MALK_03364.t1.e1;Parent=MALK_03364.t1
contig011	maker	gene	424420	427110	.	-	.	ID=MALK_03365;prediction_source=maker_MRET:augustus_masked-contig011-processed-gene-4.30-mRNA-1
contig011	maker	CDS	424420	427110	.	-	0	ID=MALK_03365.t1.c1;Parent=MALK_03365.t1
contig011	maker	mRNA	424420	427110	.	-	.	ID=MALK_03365.t1;Parent=MALK_03365
contig011	maker	exon	424420	427110	.	-	.	ID=MALK_03365.t1.e1;Parent=MALK_03365.t1
contig011	maker	gene	427172	428740	.	+	.	ID=MALK_03366;prediction_source=maker_MRET:augustus_masked-contig011-processed-gene-4.9-mRNA-1
contig011	maker	CDS	427172	428740	.	+	0	ID=MALK_03366.t1.c1;Parent=MALK_03366.t1
contig011	maker	mRNA	427172	428740	.	+	.	ID=MALK_03366.t1;Parent=MALK_03366
contig011	maker	exon	427172	428740	.	+	.	ID=MALK_03366.t1.e1;Parent=MALK_03366.t1
contig011	AUGUSTUS	gene	428813	430774	1	+	.	ID=MALK_03367;prediction_source=braker_MRET:g3707.t1
contig011	AUGUSTUS	CDS	428813	430774	1	+	0	ID=MALK_03367.t1.c1;Parent=MALK_03367.t1
contig011	AUGUSTUS	mRNA	428813	430774	1	+	.	ID=MALK_03367.t1;Parent=MALK_03367

contig011	AUGUSTUS	exon	428813	430774	.	+	.	ID=MALK_03367.t1.e1;Parent=MALK_03367.t1
contig011	AUGUSTUS	gene	430873	432641	0.58	+	.	ID=MALK_03368;prediction_source=braker_MRET:g3708.t1
contig011	AUGUSTUS	CDS	430873	432513	0.63	+	0	ID=MALK_03368.t1.c1;Parent=MALK_03368.t1
contig011	AUGUSTUS	CDS	432612	432641	0.63	+	0	ID=MALK_03368.t1.c2;Parent=MALK_03368.t1
contig011	AUGUSTUS	mRNA	430873	432641	0.58	+	.	ID=MALK_03368.t1;Parent=MALK_03368
contig011	AUGUSTUS	exon	430873	432513	.	+	.	ID=MALK_03368.t1.e1;Parent=MALK_03368.t1
contig011	AUGUSTUS	exon	432612	432641	.	+	.	ID=MALK_03368.t1.e2;Parent=MALK_03368.t1
contig011	maker	gene	432643	433722	.	-	.	ID=MALK_03369;prediction_source=maker_MRET:augustus_masked-contig011-processed-gene-4.31-mRNA-1
contig011	maker	CDS	432643	433722	.	-	0	ID=MALK_03369.t1.c1;Parent=MALK_03369.t1
contig011	maker	mRNA	432643	433722	.	-	.	ID=MALK_03369.t1;Parent=MALK_03369
contig011	maker	exon	432643	433722	.	-	.	ID=MALK_03369.t1.e1;Parent=MALK_03369.t1
contig011	maker	gene	433878	434757	.	+	.	ID=MALK_03370;prediction_source=maker_MRET:augustus_masked-contig011-processed-gene-4.10-mRNA-1
contig011	maker	CDS	433878	434163	.	+	0	ID=MALK_03370.t1.c1;Parent=MALK_03370.t1
contig011	maker	CDS	434234	434757	.	+	0	ID=MALK_03370.t1.c2;Parent=MALK_03370.t1
contig011	maker	mRNA	433878	434757	.	+	.	ID=MALK_03370.t1;Parent=MALK_03370
contig011	maker	exon	433878	434163	.	+	.	ID=MALK_03370.t1.e1;Parent=MALK_03370.t1
contig011	maker	exon	434234	434757	.	+	.	ID=MALK_03370.t1.e2;Parent=MALK_03370.t1
contig011	AUGUSTUS	gene	434789	436282	0.98	+	.	ID=MALK_03371;prediction_source=augustus:contig011.g1765.t1
contig011	AUGUSTUS	CDS	434789	436282	0.98	+	0	ID=MALK_03371.t1.c1;Parent=MALK_03371.t1
contig011	AUGUSTUS	mRNA	434789	436282	0.98	+	.	ID=MALK_03371.t1;Parent=MALK_03371
contig011	AUGUSTUS	exon	434789	436282	0.98	+	.	ID=MALK_03371.t1.e1;Parent=MALK_03371.t1
contig011	AUGUSTUS	gene	436285	437463	1	-	.	ID=MALK_03372;prediction_source=augustus:contig011.g1766.t1
contig011	AUGUSTUS	CDS	436285	437463	1	-	0	ID=MALK_03372.t1.c1;Parent=MALK_03372.t1
contig011	AUGUSTUS	mRNA	436285	437463	1	-	.	ID=MALK_03372.t1;Parent=MALK_03372
contig011	AUGUSTUS	exon	436285	437463	1	-	.	ID=MALK_03372.t1.e1;Parent=MALK_03372.t1
contig011	AUGUSTUS	gene	438504	440139	0.43	-	.	ID=MALK_03373;prediction_source=braker_MRET:g3713.t1
contig011	AUGUSTUS	CDS	439827	440139	0.56	-	0	ID=MALK_03373.t1.c2;Parent=MALK_03373.t1
contig011	AUGUSTUS	CDS	438504	439798	0.56	-	0	ID=MALK_03373.t1.c1;Parent=MALK_03373.t1
contig011	AUGUSTUS	mRNA	438504	440139	0.43	-	.	ID=MALK_03373.t1;Parent=MALK_03373
contig011	AUGUSTUS	exon	439827	440139	.	-	.	ID=MALK_03373.t1.e2;Parent=MALK_03373.t1
contig011	AUGUSTUS	exon	438504	439798	.	-	.	ID=MALK_03373.t1.e1;Parent=MALK_03373.t1
contig011	AUGUSTUS	gene	440223	441257	0.94	-	.	ID=MALK_03374;prediction_source=braker_MRET:g3714.t1
contig011	AUGUSTUS	CDS	440223	441257	0.94	-	0	ID=MALK_03374.t1.c1;Parent=MALK_03374.t1
contig011	AUGUSTUS	mRNA	440223	441257	0.94	-	.	ID=MALK_03374.t1;Parent=MALK_03374
contig011	AUGUSTUS	exon	440223	441257	.	-	.	ID=MALK_03374.t1.e1;Parent=MALK_03374.t1
contig011	maker	gene	441367	444162	.	-	.	ID=MALK_03375;prediction_source=maker_MRET:augustus_masked-contig011-processed-gene-4.35-mRNA-1
contig011	maker	CDS	441367	444162	.	-	0	ID=MALK_03375.t1.c1;Parent=MALK_03375.t1
contig011	maker	mRNA	441367	444162	.	-	.	ID=MALK_03375.t1;Parent=MALK_03375
contig011	maker	exon	441367	444162	.	-	.	ID=MALK_03375.t1.e1;Parent=MALK_03375.t1
contig011	AUGUSTUS	gene	444275	445189	0.27	-	.	ID=MALK_03376;prediction_source=augustus:contig011.g1770.t1
contig011	AUGUSTUS	CDS	444275	445189	0.27	-	0	ID=MALK_03376.t1.c1;Parent=MALK_03376.t1
contig011	AUGUSTUS	mRNA	444275	445189	0.27	-	.	ID=MALK_03376.t1;Parent=MALK_03376
contig011	AUGUSTUS	exon	444275	445189	0.27	-	.	ID=MALK_03376.t1.e1;Parent=MALK_03376.t1

contig011	AUGUSTUS	gene	445655	446138	0.96	+	.	ID=MALK_03377;prediction_source=braker_MRET:g3716.t1
contig011	AUGUSTUS	CDS	445655	445731	0.96	+	0	ID=MALK_03377.t1.c1;Parent=MALK_03377.t1
contig011	AUGUSTUS	CDS	445760	446138	0.96	+	0	ID=MALK_03377.t1.c2;Parent=MALK_03377.t1
contig011	AUGUSTUS	mRNA	445655	446138	0.96	+	.	ID=MALK_03377.t1;Parent=MALK_03377
contig011	AUGUSTUS	exon	445655	445731	.	+	.	ID=MALK_03377.t1.e1;Parent=MALK_03377.t1
contig011	AUGUSTUS	exon	445760	446138	.	+	.	ID=MALK_03377.t1.e2;Parent=MALK_03377.t1
contig011	maker	gene	446165	447541	.	-	.	ID=MALK_03378;prediction_source=maker_MRET:augustus_masked-contig011-processed-gene-4.37-mRNA-1
contig011	maker	CDS	446165	447541	.	-	0	ID=MALK_03378.t1.c1;Parent=MALK_03378.t1
contig011	maker	mRNA	446165	447541	.	-	.	ID=MALK_03378.t1;Parent=MALK_03378
contig011	maker	exon	446165	447541	.	-	.	ID=MALK_03378.t1.e1;Parent=MALK_03378.t1
contig011	AUGUSTUS	gene	447786	450512	1	+	.	ID=MALK_03379;prediction_source=augustus:contig011.g1773.t1
contig011	AUGUSTUS	CDS	447786	450512	1	+	0	ID=MALK_03379.t1.c1;Parent=MALK_03379.t1
contig011	AUGUSTUS	mRNA	447786	450512	1	+	.	ID=MALK_03379.t1;Parent=MALK_03379
contig011	AUGUSTUS	exon	447786	450512	1	+	.	ID=MALK_03379.t1.e1;Parent=MALK_03379.t1
contig011	AUGUSTUS	gene	450557	451678	0.98	-	.	ID=MALK_03380;prediction_source=augustus:contig011.g1774.t1
contig011	AUGUSTUS	CDS	450557	451678	0.98	-	0	ID=MALK_03380.t1.c1;Parent=MALK_03380.t1
contig011	AUGUSTUS	mRNA	450557	451678	0.98	-	.	ID=MALK_03380.t1;Parent=MALK_03380
contig011	AUGUSTUS	exon	450557	451678	0.98	-	.	ID=MALK_03380.t1.e1;Parent=MALK_03380.t1
contig011	AUGUSTUS	gene	452105	452995	0.48	+	.	ID=MALK_03381;prediction_source=braker_MRET:g3720.t1
contig011	AUGUSTUS	CDS	452105	452191	0.96	+	0	ID=MALK_03381.t1.c1;Parent=MALK_03381.t1
contig011	AUGUSTUS	CDS	452232	452305	0.96	+	0	ID=MALK_03381.t1.c2;Parent=MALK_03381.t1
contig011	AUGUSTUS	CDS	452335	452995	0.96	+	0	ID=MALK_03381.t1.c3;Parent=MALK_03381.t1
contig011	AUGUSTUS	mRNA	452105	452995	0.48	+	.	ID=MALK_03381.t1;Parent=MALK_03381
contig011	AUGUSTUS	exon	452105	452191	.	+	.	ID=MALK_03381.t1.e1;Parent=MALK_03381.t1
contig011	AUGUSTUS	exon	452232	452305	.	+	.	ID=MALK_03381.t1.e2;Parent=MALK_03381.t1
contig011	AUGUSTUS	exon	452335	452995	.	+	.	ID=MALK_03381.t1.e3;Parent=MALK_03381.t1
contig011	maker	gene	453186	454703	.	+	.	ID=MALK_03382;prediction_source=maker_MRET:augustus_masked-contig011-processed-gene-4.14-mRNA-1
contig011	maker	CDS	453186	454703	.	+	0	ID=MALK_03382.t1.c1;Parent=MALK_03382.t1
contig011	maker	mRNA	453186	454703	.	+	.	ID=MALK_03382.t1;Parent=MALK_03382
contig011	maker	exon	453186	454703	.	+	.	ID=MALK_03382.t1.e1;Parent=MALK_03382.t1
contig011	maker	gene	454835	456700	.	-	.	ID=MALK_03383;prediction_source=maker_MRET:augustus_masked-contig011-processed-gene-4.39-mRNA-1
contig011	maker	CDS	454835	456700	.	-	0	ID=MALK_03383.t1.c1;Parent=MALK_03383.t1
contig011	maker	mRNA	454835	456700	.	-	.	ID=MALK_03383.t1;Parent=MALK_03383
contig011	maker	exon	454835	456700	.	-	.	ID=MALK_03383.t1.e1;Parent=MALK_03383.t1
contig011	AUGUSTUS	gene	456848	457597	0.95	+	.	ID=MALK_03384;prediction_source=augustus:contig011.g1780.t1
contig011	AUGUSTUS	CDS	456848	457597	0.95	+	0	ID=MALK_03384.t1.c1;Parent=MALK_03384.t1
contig011	AUGUSTUS	mRNA	456848	457597	0.95	+	.	ID=MALK_03384.t1;Parent=MALK_03384
contig011	AUGUSTUS	exon	456848	457597	0.95	+	.	ID=MALK_03384.t1.e1;Parent=MALK_03384.t1
contig011	AUGUSTUS	gene	457692	458444	0.99	+	.	ID=MALK_03385;prediction_source=augustus:contig011.g1781.t1
contig011	AUGUSTUS	CDS	457692	458444	0.99	+	0	ID=MALK_03385.t1.c1;Parent=MALK_03385.t1
contig011	AUGUSTUS	mRNA	457692	458444	0.99	+	.	ID=MALK_03385.t1;Parent=MALK_03385
contig011	AUGUSTUS	exon	457692	458444	0.99	+	.	ID=MALK_03385.t1.e1;Parent=MALK_03385.t1
contig011	AUGUSTUS	gene	458446	460773	0.94	-	.	ID=MALK_03386;prediction_source=braker_MRET:g3724.t1

contig011	AUGUSTUS	CDS	460420	460773	0.98	-	0	ID=MALK_03386.t1.c2;Parent=MALK_03386.t1
contig011	AUGUSTUS	CDS	458446	460389	0.98	-	0	ID=MALK_03386.t1.c1;Parent=MALK_03386.t1
contig011	AUGUSTUS	mRNA	458446	460773	0.94	-	.	ID=MALK_03386.t1;Parent=MALK_03386
contig011	AUGUSTUS	exon	460420	460773	.	-	.	ID=MALK_03386.t1.e2;Parent=MALK_03386.t1
contig011	AUGUSTUS	exon	458446	460389	.	-	.	ID=MALK_03386.t1.e1;Parent=MALK_03386.t1
contig011	maker	gene	460903	461313	.	+	.	ID=MALK_03387;prediction_source=maker_MRET:augustus_masked-contig011-processed-gene-4.17-mRNA-1
contig011	maker	CDS	460903	461313	.	+	0	ID=MALK_03387.t1.c1;Parent=MALK_03387.t1
contig011	maker	mRNA	460903	461313	.	+	.	ID=MALK_03387.t1;Parent=MALK_03387
contig011	maker	exon	460903	461313	.	+	.	ID=MALK_03387.t1.e1;Parent=MALK_03387.t1
contig011	maker	gene	461466	462248	.	-	.	ID=MALK_03388;prediction_source=maker_MRET:augustus_masked-contig011-processed-gene-4.41-mRNA-1
contig011	maker	CDS	461466	462248	.	-	0	ID=MALK_03388.t1.c1;Parent=MALK_03388.t1
contig011	maker	mRNA	461466	462248	.	-	.	ID=MALK_03388.t1;Parent=MALK_03388
contig011	maker	exon	461466	462248	.	-	.	ID=MALK_03388.t1.e1;Parent=MALK_03388.t1
contig011	AUGUSTUS	gene	462556	463116	0.97	+	.	ID=MALK_03389;prediction_source=augustus:contig011.g1785.t1
contig011	AUGUSTUS	CDS	462556	463116	0.97	+	0	ID=MALK_03389.t1.c1;Parent=MALK_03389.t1
contig011	AUGUSTUS	mRNA	462556	463116	0.97	+	.	ID=MALK_03389.t1;Parent=MALK_03389
contig011	AUGUSTUS	exon	462556	463116	0.97	+	.	ID=MALK_03389.t1.e1;Parent=MALK_03389.t1
contig011	AUGUSTUS	gene	463173	465052	1	-	.	ID=MALK_03390;prediction_source=augustus:contig011.g1786.t1
contig011	AUGUSTUS	CDS	464057	465052	1	-	0	ID=MALK_03390.t1.c2;Parent=MALK_03390.t1
contig011	AUGUSTUS	CDS	463173	463589	1	-	0	ID=MALK_03390.t1.c1;Parent=MALK_03390.t1
contig011	AUGUSTUS	mRNA	463173	465052	1	-	.	ID=MALK_03390.t1;Parent=MALK_03390
contig011	AUGUSTUS	exon	464057	465052	1	-	.	ID=MALK_03390.t1.e2;Parent=MALK_03390.t1
contig011	AUGUSTUS	exon	463173	463589	1	-	.	ID=MALK_03390.t1.e1;Parent=MALK_03390.t1
contig011	maker	gene	465103	467430	.	-	.	ID=MALK_03391;prediction_source=maker_MRET:augustus_masked-contig011-processed-gene-4.43-mRNA-1
contig011	maker	CDS	465103	467430	.	-	0	ID=MALK_03391.t1.c1;Parent=MALK_03391.t1
contig011	maker	mRNA	465103	467430	.	-	.	ID=MALK_03391.t1;Parent=MALK_03391
contig011	maker	exon	465103	467430	.	-	.	ID=MALK_03391.t1.e1;Parent=MALK_03391.t1
contig011	AUGUSTUS	gene	467586	468128	0.99	+	.	ID=MALK_03392;prediction_source=braker_MRET:g3730.t1
contig011	AUGUSTUS	CDS	467586	468128	0.99	+	0	ID=MALK_03392.t1.c1;Parent=MALK_03392.t1
contig011	AUGUSTUS	mRNA	467586	468128	0.99	+	.	ID=MALK_03392.t1;Parent=MALK_03392
contig011	AUGUSTUS	exon	467586	468128	.	+	.	ID=MALK_03392.t1.e1;Parent=MALK_03392.t1
contig011	AUGUSTUS	gene	468200	470263	0.62	-	.	ID=MALK_03393;prediction_source=braker_MRET:g3731.t1
contig011	AUGUSTUS	CDS	468200	470263	0.62	-	0	ID=MALK_03393.t1.c1;Parent=MALK_03393.t1
contig011	AUGUSTUS	mRNA	468200	470263	0.62	-	.	ID=MALK_03393.t1;Parent=MALK_03393
contig011	AUGUSTUS	exon	468200	470263	.	-	.	ID=MALK_03393.t1.e1;Parent=MALK_03393.t1
contig011	AUGUSTUS	gene	470849	472279	0.54	-	.	ID=MALK_03394;prediction_source=braker_MRET:g3732.t1
contig011	AUGUSTUS	CDS	470849	472279	0.54	-	0	ID=MALK_03394.t1.c1;Parent=MALK_03394.t1
contig011	AUGUSTUS	mRNA	470849	472279	0.54	-	.	ID=MALK_03394.t1;Parent=MALK_03394
contig011	AUGUSTUS	exon	470849	472279	.	-	.	ID=MALK_03394.t1.e1;Parent=MALK_03394.t1
contig011	maker	gene	472429	477846	.	+	.	ID=MALK_03395;prediction_source=maker_MRET:augustus_masked-contig011-processed-gene-4.19-mRNA-1
contig011	maker	CDS	472429	477846	.	+	0	ID=MALK_03395.t1.c1;Parent=MALK_03395.t1
contig011	maker	mRNA	472429	477846	.	+	.	ID=MALK_03395.t1;Parent=MALK_03395
contig011	maker	exon	472429	477846	.	+	.	ID=MALK_03395.t1.e1;Parent=MALK_03395.t1

contig011	maker	gene	477857	478594	.	-	.	ID=MALK_03396;prediction_source=maker_MRET:augustus_masked-contig011-processed-gene-4.45-mRNA-1
contig011	maker	CDS	477857	478594	.	-	0	ID=MALK_03396.t1.c1;Parent=MALK_03396.t1
contig011	maker	mRNA	477857	478594	.	-	.	ID=MALK_03396.t1;Parent=MALK_03396
contig011	maker	exon	477857	478594	.	-	.	ID=MALK_03396.t1.e1;Parent=MALK_03396.t1
contig011	AUGUSTUS	gene	478754	479035	0.93	+	.	ID=MALK_03397;prediction_source=braker_MRET:g3735.t1
contig011	AUGUSTUS	CDS	478754	479035	0.93	+	0	ID=MALK_03397.t1.c1;Parent=MALK_03397.t1
contig011	AUGUSTUS	mRNA	478754	479035	0.93	+	.	ID=MALK_03397.t1;Parent=MALK_03397
contig011	AUGUSTUS	exon	478754	479035	.	+	.	ID=MALK_03397.t1.e1;Parent=MALK_03397.t1
contig011	AUGUSTUS	gene	479266	479550	0.82	+	.	ID=MALK_03398;prediction_source=augustus:contig011.g1793.t1
contig011	AUGUSTUS	CDS	479266	479550	0.82	+	0	ID=MALK_03398.t1.c1;Parent=MALK_03398.t1
contig011	AUGUSTUS	mRNA	479266	479550	0.82	+	.	ID=MALK_03398.t1;Parent=MALK_03398
contig011	AUGUSTUS	exon	479266	479550	0.82	+	.	ID=MALK_03398.t1.e1;Parent=MALK_03398.t1
contig011	maker	gene	479706	484130	.	+	.	ID=MALK_03399;prediction_source=maker_MRET:augustus_masked-contig011-processed-gene-4.21-mRNA-1
contig011	maker	CDS	479706	484130	.	+	0	ID=MALK_03399.t1.c1;Parent=MALK_03399.t1
contig011	maker	mRNA	479706	484130	.	+	.	ID=MALK_03399.t1;Parent=MALK_03399
contig011	maker	exon	479706	484130	.	+	.	ID=MALK_03399.t1.e1;Parent=MALK_03399.t1
contig011	AUGUSTUS	gene	484526	486079	0.72	+	.	ID=MALK_03400;prediction_source=braker_MRET:g3738.t1
contig011	AUGUSTUS	CDS	484526	486079	0.72	+	0	ID=MALK_03400.t1.c1;Parent=MALK_03400.t1
contig011	AUGUSTUS	mRNA	484526	486079	0.72	+	.	ID=MALK_03400.t1;Parent=MALK_03400
contig011	AUGUSTUS	exon	484526	486079	.	+	.	ID=MALK_03400.t1.e1;Parent=MALK_03400.t1
contig011	maker	gene	486113	487105	.	-	.	ID=MALK_03401;prediction_source=maker_MRET:augustus_masked-contig011-processed-gene-4.46-mRNA-1
contig011	maker	CDS	486113	487105	.	-	0	ID=MALK_03401.t1.c1;Parent=MALK_03401.t1
contig011	maker	mRNA	486113	487105	.	-	.	ID=MALK_03401.t1;Parent=MALK_03401
contig011	maker	exon	486113	487105	.	-	.	ID=MALK_03401.t1.e1;Parent=MALK_03401.t1
contig011	AUGUSTUS	gene	487367	488476	0.89	+	.	ID=MALK_03402;prediction_source=augustus:contig011.g1798.t1
contig011	AUGUSTUS	CDS	487367	488476	0.89	+	0	ID=MALK_03402.t1.c1;Parent=MALK_03402.t1
contig011	AUGUSTUS	mRNA	487367	488476	0.89	+	.	ID=MALK_03402.t1;Parent=MALK_03402
contig011	AUGUSTUS	exon	487367	488476	0.89	+	.	ID=MALK_03402.t1.e1;Parent=MALK_03402.t1
contig011	maker	gene	488965	490716	.	+	.	ID=MALK_03403;prediction_source=maker_MRET:augustus_masked-contig011-processed-gene-4.24-mRNA-1
contig011	maker	CDS	488965	490716	.	+	0	ID=MALK_03403.t1.c1;Parent=MALK_03403.t1
contig011	maker	mRNA	488965	490716	.	+	.	ID=MALK_03403.t1;Parent=MALK_03403
contig011	maker	exon	488965	490716	.	+	.	ID=MALK_03403.t1.e1;Parent=MALK_03403.t1
contig011	AUGUSTUS	gene	491034	491303	0.78	-	.	ID=MALK_03404;prediction_source=braker_MRET:g3742.t1
contig011	AUGUSTUS	CDS	491034	491303	0.78	-	0	ID=MALK_03404.t1.c1;Parent=MALK_03404.t1
contig011	AUGUSTUS	mRNA	491034	491303	0.78	-	.	ID=MALK_03404.t1;Parent=MALK_03404
contig011	AUGUSTUS	exon	491034	491303	.	-	.	ID=MALK_03404.t1.e1;Parent=MALK_03404.t1
contig011	AUGUSTUS	gene	491610	493055	0.97	+	.	ID=MALK_03405;prediction_source=braker_MRET:g3743.t1
contig011	AUGUSTUS	CDS	491610	493055	0.97	+	0	ID=MALK_03405.t1.c1;Parent=MALK_03405.t1
contig011	AUGUSTUS	mRNA	491610	493055	0.97	+	.	ID=MALK_03405.t1;Parent=MALK_03405
contig011	AUGUSTUS	exon	491610	493055	.	+	.	ID=MALK_03405.t1.e1;Parent=MALK_03405.t1
contig011	AUGUSTUS	gene	493373	495305	1	+	.	ID=MALK_03406;prediction_source=braker_MRET:g3744.t1
contig011	AUGUSTUS	CDS	493373	493379	1	+	0	ID=MALK_03406.t1.c1;Parent=MALK_03406.t1
contig011	AUGUSTUS	CDS	493420	495305	1	+	0	ID=MALK_03406.t1.c2;Parent=MALK_03406.t1

contig011	AUGUSTUS	mRNA	493373	495305	1	+	.	ID=MALK_03406.t1;Parent=MALK_03406
contig011	AUGUSTUS	exon	493373	493379	.	+	.	ID=MALK_03406.t1.e1;Parent=MALK_03406.t1
contig011	AUGUSTUS	exon	493420	495305	.	+	.	ID=MALK_03406.t1.e2;Parent=MALK_03406.t1
contig011	AUGUSTUS	gene	496298	497410	0.56	-	.	ID=MALK_03407;prediction_source=braker_MRET:g3745.t1
contig011	AUGUSTUS	CDS	496298	497410	0.56	-	0	ID=MALK_03407.t1.c1;Parent=MALK_03407.t1
contig011	AUGUSTUS	mRNA	496298	497410	0.56	-	.	ID=MALK_03407.t1;Parent=MALK_03407
contig011	AUGUSTUS	exon	496298	497410	.	-	.	ID=MALK_03407.t1.e1;Parent=MALK_03407.t1
contig011	AUGUSTUS	gene	497614	498930	1	-	.	ID=MALK_03408;prediction_source=braker_MRET:g3746.t1
contig011	AUGUSTUS	CDS	497614	498930	1	-	0	ID=MALK_03408.t1.c1;Parent=MALK_03408.t1
contig011	AUGUSTUS	mRNA	497614	498930	1	-	.	ID=MALK_03408.t1;Parent=MALK_03408
contig011	AUGUSTUS	exon	497614	498930	.	-	.	ID=MALK_03408.t1.e1;Parent=MALK_03408.t1
contig011	maker	gene	499145	500311	.	+	.	ID=MALK_03409;prediction_source=maker_MRET:augustus_masked-contig011-processed-gene-5.90-mRNA-1
contig011	maker	CDS	499145	500311	.	+	0	ID=MALK_03409.t1.c1;Parent=MALK_03409.t1
contig011	maker	mRNA	499145	500311	.	+	.	ID=MALK_03409.t1;Parent=MALK_03409
contig011	maker	exon	499145	500311	.	+	.	ID=MALK_03409.t1.e1;Parent=MALK_03409.t1
contig011	AUGUSTUS	gene	500577	505223	0.33	-	.	ID=MALK_03410;prediction_source=augustus:contig011.g1806.t1
contig011	AUGUSTUS	CDS	500577	505223	0.33	-	0	ID=MALK_03410.t1.c1;Parent=MALK_03410.t1
contig011	AUGUSTUS	mRNA	500577	505223	0.33	-	.	ID=MALK_03410.t1;Parent=MALK_03410
contig011	AUGUSTUS	exon	500577	505223	0.33	-	.	ID=MALK_03410.t1.e1;Parent=MALK_03410.t1
contig011	maker	gene	505601	508930	.	+	.	ID=MALK_03411;prediction_source=maker_MRET:augustus_masked-contig011-processed-gene-5.92-mRNA-1
contig011	maker	CDS	505601	508930	.	+	0	ID=MALK_03411.t1.c1;Parent=MALK_03411.t1
contig011	maker	mRNA	505601	508930	.	+	.	ID=MALK_03411.t1;Parent=MALK_03411
contig011	maker	exon	505601	508930	.	+	.	ID=MALK_03411.t1.e1;Parent=MALK_03411.t1
contig011	AUGUSTUS	gene	509071	509646	0.96	+	.	ID=MALK_03412;prediction_source=augustus:contig011.g1809.t1
contig011	AUGUSTUS	CDS	509071	509646	0.96	+	0	ID=MALK_03412.t1.c1;Parent=MALK_03412.t1
contig011	AUGUSTUS	mRNA	509071	509646	0.96	+	.	ID=MALK_03412.t1;Parent=MALK_03412
contig011	AUGUSTUS	exon	509071	509646	0.96	+	.	ID=MALK_03412.t1.e1;Parent=MALK_03412.t1
contig011	maker	gene	510018	510698	.	+	.	ID=MALK_03413;prediction_source=maker_MRET:augustus_masked-contig011-processed-gene-5.94-mRNA-1
contig011	maker	CDS	510018	510698	.	+	0	ID=MALK_03413.t1.c1;Parent=MALK_03413.t1
contig011	maker	mRNA	510018	510698	.	+	.	ID=MALK_03413.t1;Parent=MALK_03413
contig011	maker	exon	510018	510698	.	+	.	ID=MALK_03413.t1.e1;Parent=MALK_03413.t1
contig011	AUGUSTUS	gene	510800	511816	0.42	+	.	ID=MALK_03414;prediction_source=braker_MRET:g3752.t1
contig011	AUGUSTUS	CDS	510800	511023	0.48	+	0	ID=MALK_03414.t1.c1;Parent=MALK_03414.t1
contig011	AUGUSTUS	CDS	511123	511816	0.48	+	0	ID=MALK_03414.t1.c2;Parent=MALK_03414.t1
contig011	AUGUSTUS	mRNA	510800	511816	0.42	+	.	ID=MALK_03414.t1;Parent=MALK_03414
contig011	AUGUSTUS	exon	510800	511023	.	+	.	ID=MALK_03414.t1.e1;Parent=MALK_03414.t1
contig011	AUGUSTUS	exon	511123	511816	.	+	.	ID=MALK_03414.t1.e2;Parent=MALK_03414.t1
contig011	AUGUSTUS	gene	511834	512217	1	-	.	ID=MALK_03415;prediction_source=braker_MRET:g3753.t1
contig011	AUGUSTUS	CDS	511834	512217	1	-	0	ID=MALK_03415.t1.c1;Parent=MALK_03415.t1
contig011	AUGUSTUS	mRNA	511834	512217	1	-	.	ID=MALK_03415.t1;Parent=MALK_03415
contig011	AUGUSTUS	exon	511834	512217	.	-	.	ID=MALK_03415.t1.e1;Parent=MALK_03415.t1
contig011	AUGUSTUS	gene	512334	514172	1	+	.	ID=MALK_03416;prediction_source=augustus:contig011.g1811.t1
contig011	AUGUSTUS	CDS	512334	514172	1	+	0	ID=MALK_03416.t1.c1;Parent=MALK_03416.t1

contig011	AUGUSTUS	mRNA	512334	514172	1	+	.	ID=MALK_03416.t1;Parent=MALK_03416
contig011	AUGUSTUS	exon	512334	514172	1	+	.	ID=MALK_03416.t1.e1;Parent=MALK_03416.t1
contig011	maker	gene	514188	515450	.	-	.	ID=MALK_03417;prediction_source=maker_MRET:augustus_masked-contig011-processed-gene-5.125-mRNA-1
contig011	maker	CDS	514188	515450	.	-	0	ID=MALK_03417.t1.c1;Parent=MALK_03417.t1
contig011	maker	mRNA	514188	515450	.	-	.	ID=MALK_03417.t1;Parent=MALK_03417
contig011	maker	exon	514188	515450	.	-	.	ID=MALK_03417.t1.e1;Parent=MALK_03417.t1
contig011	AUGUSTUS	gene	515591	516259	0.46	+	.	ID=MALK_03418;prediction_source=augustus:contig011.g1813.t1
contig011	AUGUSTUS	CDS	515591	516259	0.46	+	0	ID=MALK_03418.t1.c1;Parent=MALK_03418.t1
contig011	AUGUSTUS	mRNA	515591	516259	0.46	+	.	ID=MALK_03418.t1;Parent=MALK_03418
contig011	AUGUSTUS	exon	515591	516259	0.46	+	.	ID=MALK_03418.t1.e1;Parent=MALK_03418.t1
contig011	AUGUSTUS	gene	516296	516883	0.6	-	.	ID=MALK_03419;prediction_source=braker_MRET:g3757.t1
contig011	AUGUSTUS	CDS	516814	516883	0.83	-	0	ID=MALK_03419.t1.c2;Parent=MALK_03419.t1
contig011	AUGUSTUS	CDS	516296	516783	0.83	-	0	ID=MALK_03419.t1.c1;Parent=MALK_03419.t1
contig011	AUGUSTUS	mRNA	516296	516883	0.6	-	.	ID=MALK_03419.t1;Parent=MALK_03419
contig011	AUGUSTUS	exon	516814	516883	.	-	.	ID=MALK_03419.t1.e2;Parent=MALK_03419.t1
contig011	AUGUSTUS	exon	516296	516783	.	-	.	ID=MALK_03419.t1.e1;Parent=MALK_03419.t1
contig011	AUGUSTUS	gene	517027	517498	0.96	+	.	ID=MALK_03420;prediction_source=braker_MRET:g3758.t1
contig011	AUGUSTUS	CDS	517027	517291	1	+	0	ID=MALK_03420.t1.c1;Parent=MALK_03420.t1
contig011	AUGUSTUS	CDS	517332	517359	1	+	0	ID=MALK_03420.t1.c2;Parent=MALK_03420.t1
contig011	AUGUSTUS	CDS	517395	517410	1	+	0	ID=MALK_03420.t1.c3;Parent=MALK_03420.t1
contig011	AUGUSTUS	CDS	517442	517498	1	+	0	ID=MALK_03420.t1.c4;Parent=MALK_03420.t1
contig011	AUGUSTUS	mRNA	517027	517498	0.96	+	.	ID=MALK_03420.t1;Parent=MALK_03420
contig011	AUGUSTUS	exon	517027	517291	.	+	.	ID=MALK_03420.t1.e1;Parent=MALK_03420.t1
contig011	AUGUSTUS	exon	517332	517359	.	+	.	ID=MALK_03420.t1.e2;Parent=MALK_03420.t1
contig011	AUGUSTUS	exon	517395	517410	.	+	.	ID=MALK_03420.t1.e3;Parent=MALK_03420.t1
contig011	AUGUSTUS	exon	517442	517498	.	+	.	ID=MALK_03420.t1.e4;Parent=MALK_03420.t1
contig011	maker	gene	517546	518196	.	-	.	ID=MALK_03421;prediction_source=maker_MRET:augustus_masked-contig011-processed-gene-5.127-mRNA-1
contig011	maker	CDS	517546	518196	.	-	0	ID=MALK_03421.t1.c1;Parent=MALK_03421.t1
contig011	maker	mRNA	517546	518196	.	-	.	ID=MALK_03421.t1;Parent=MALK_03421
contig011	maker	exon	517546	518196	.	-	.	ID=MALK_03421.t1.e1;Parent=MALK_03421.t1
contig011	AUGUSTUS	gene	518607	519104	0.74	-	.	ID=MALK_03422;prediction_source=augustus:contig011.g1816.t1
contig011	AUGUSTUS	CDS	518607	519104	0.74	-	0	ID=MALK_03422.t1.c1;Parent=MALK_03422.t1
contig011	AUGUSTUS	mRNA	518607	519104	0.74	-	.	ID=MALK_03422.t1;Parent=MALK_03422
contig011	AUGUSTUS	exon	518607	519104	0.74	-	.	ID=MALK_03422.t1.e1;Parent=MALK_03422.t1
contig011	AUGUSTUS	gene	519173	519584	0.93	+	.	ID=MALK_03423;prediction_source=braker_MRET:g3761.t1
contig011	AUGUSTUS	CDS	519173	519355	0.97	+	0	ID=MALK_03423.t1.c1;Parent=MALK_03423.t1
contig011	AUGUSTUS	CDS	519387	519584	0.97	+	0	ID=MALK_03423.t1.c2;Parent=MALK_03423.t1
contig011	AUGUSTUS	mRNA	519173	519584	0.93	+	.	ID=MALK_03423.t1;Parent=MALK_03423
contig011	AUGUSTUS	exon	519173	519355	.	+	.	ID=MALK_03423.t1.e1;Parent=MALK_03423.t1
contig011	AUGUSTUS	exon	519387	519584	.	+	.	ID=MALK_03423.t1.e2;Parent=MALK_03423.t1
contig011	AUGUSTUS	gene	519704	519949	0.26	+	.	ID=MALK_03424;prediction_source=braker_MRET:g3762.t1
contig011	AUGUSTUS	CDS	519704	519862	0.58	+	0	ID=MALK_03424.t1.c1;Parent=MALK_03424.t1
contig011	AUGUSTUS	CDS	519890	519949	0.58	+	0	ID=MALK_03424.t1.c2;Parent=MALK_03424.t1

contig011	AUGUSTUS	mRNA	519704	519949	0.26	+	.	ID=MALK_03424.t1;Parent=MALK_03424
contig011	AUGUSTUS	exon	519704	519862	.	+	.	ID=MALK_03424.t1.e1;Parent=MALK_03424.t1
contig011	AUGUSTUS	exon	519890	519949	.	+	.	ID=MALK_03424.t1.e2;Parent=MALK_03424.t1
contig011	AUGUSTUS	gene	519961	521994	0.99	-	.	ID=MALK_03425;prediction_source=augustus:contig011.g1817.t1
contig011	AUGUSTUS	CDS	519961	521994	0.99	-	0	ID=MALK_03425.t1.c1;Parent=MALK_03425.t1
contig011	AUGUSTUS	mRNA	519961	521994	0.99	-	.	ID=MALK_03425.t1;Parent=MALK_03425
contig011	AUGUSTUS	exon	519961	521994	0.99	-	.	ID=MALK_03425.t1.e1;Parent=MALK_03425.t1
contig011	AUGUSTUS	gene	522413	524149	0.76	+	.	ID=MALK_03426;prediction_source=augustus:contig011.g1818.t1
contig011	AUGUSTUS	CDS	522413	524149	0.76	+	0	ID=MALK_03426.t1.c1;Parent=MALK_03426.t1
contig011	AUGUSTUS	mRNA	522413	524149	0.76	+	.	ID=MALK_03426.t1;Parent=MALK_03426
contig011	AUGUSTUS	exon	522413	524149	0.76	+	.	ID=MALK_03426.t1.e1;Parent=MALK_03426.t1
contig011	AUGUSTUS	gene	524166	526046	0.65	-	.	ID=MALK_03427;prediction_source=augustus:contig011.g1819.t1
contig011	AUGUSTUS	CDS	524166	526046	0.65	-	0	ID=MALK_03427.t1.c1;Parent=MALK_03427.t1
contig011	AUGUSTUS	mRNA	524166	526046	0.65	-	.	ID=MALK_03427.t1;Parent=MALK_03427
contig011	AUGUSTUS	exon	524166	526046	0.65	-	.	ID=MALK_03427.t1.e1;Parent=MALK_03427.t1
contig011	AUGUSTUS	gene	526155	528071	0.99	+	.	ID=MALK_03428;prediction_source=braker_MRET:g3766.t1
contig011	AUGUSTUS	CDS	526155	528071	0.99	+	0	ID=MALK_03428.t1.c1;Parent=MALK_03428.t1
contig011	AUGUSTUS	mRNA	526155	528071	0.99	+	.	ID=MALK_03428.t1;Parent=MALK_03428
contig011	AUGUSTUS	exon	526155	528071	.	+	.	ID=MALK_03428.t1.e1;Parent=MALK_03428.t1
contig011	AUGUSTUS	gene	528496	529440	1	+	.	ID=MALK_03429;prediction_source=braker_MRET:g3767.t1
contig011	AUGUSTUS	CDS	528496	529440	1	+	0	ID=MALK_03429.t1.c1;Parent=MALK_03429.t1
contig011	AUGUSTUS	mRNA	528496	529440	1	+	.	ID=MALK_03429.t1;Parent=MALK_03429
contig011	AUGUSTUS	exon	528496	529440	.	+	.	ID=MALK_03429.t1.e1;Parent=MALK_03429.t1
contig011	AUGUSTUS	gene	529461	531059	0.75	-	.	ID=MALK_03430;prediction_source=augustus:contig011.g1821.t1
contig011	AUGUSTUS	CDS	529461	531059	0.75	-	0	ID=MALK_03430.t1.c1;Parent=MALK_03430.t1
contig011	AUGUSTUS	mRNA	529461	531059	0.75	-	.	ID=MALK_03430.t1;Parent=MALK_03430
contig011	AUGUSTUS	exon	529461	531059	0.75	-	.	ID=MALK_03430.t1.e1;Parent=MALK_03430.t1
contig011	AUGUSTUS	gene	531243	531905	0.69	+	.	ID=MALK_03431;prediction_source=augustus:contig011.g1823.t1
contig011	AUGUSTUS	CDS	531243	531905	0.69	+	0	ID=MALK_03431.t1.c1;Parent=MALK_03431.t1
contig011	AUGUSTUS	mRNA	531243	531905	0.69	+	.	ID=MALK_03431.t1;Parent=MALK_03431
contig011	AUGUSTUS	exon	531243	531905	0.69	+	.	ID=MALK_03431.t1.e1;Parent=MALK_03431.t1
contig011	AUGUSTUS	gene	532070	532522	0.39	-	.	ID=MALK_03432;prediction_source=braker_MRET:g3770.t1
contig011	AUGUSTUS	CDS	532160	532522	0.39	-	0	ID=MALK_03432.t1.c2;Parent=MALK_03432.t1
contig011	AUGUSTUS	CDS	532070	532132	0.39	-	0	ID=MALK_03432.t1.c1;Parent=MALK_03432.t1
contig011	AUGUSTUS	mRNA	532070	532522	0.39	-	.	ID=MALK_03432.t1;Parent=MALK_03432
contig011	AUGUSTUS	exon	532160	532522	.	-	.	ID=MALK_03432.t1.e2;Parent=MALK_03432.t1
contig011	AUGUSTUS	exon	532070	532132	.	-	.	ID=MALK_03432.t1.e1;Parent=MALK_03432.t1
contig011	AUGUSTUS	gene	532671	534650	0.57	+	.	ID=MALK_03433;prediction_source=braker_MRET:g3771.t1
contig011	AUGUSTUS	CDS	532671	534650	0.57	+	0	ID=MALK_03433.t1.c1;Parent=MALK_03433.t1
contig011	AUGUSTUS	mRNA	532671	534650	0.57	+	.	ID=MALK_03433.t1;Parent=MALK_03433
contig011	AUGUSTUS	exon	532671	534650	.	+	.	ID=MALK_03433.t1.e1;Parent=MALK_03433.t1
contig011	AUGUSTUS	gene	534968	537538	0.72	+	.	ID=MALK_03434;prediction_source=augustus:contig011.g1826.t1
contig011	AUGUSTUS	CDS	534968	537538	0.72	+	0	ID=MALK_03434.t1.c1;Parent=MALK_03434.t1

contig011	AUGUSTUS	mRNA	534968	537538	0.72	+	.	ID=MALK_03434.t1;Parent=MALK_03434
contig011	AUGUSTUS	exon	534968	537538	0.72	+	.	ID=MALK_03434.t1.e1;Parent=MALK_03434.t1
contig011	AUGUSTUS	gene	537718	539328	0.92	+	.	ID=MALK_03435;prediction_source=braker_MRET:g3773.t1
contig011	AUGUSTUS	CDS	537718	539328	0.92	+	0	ID=MALK_03435.t1.c1;Parent=MALK_03435.t1
contig011	AUGUSTUS	mRNA	537718	539328	0.92	+	.	ID=MALK_03435.t1;Parent=MALK_03435
contig011	AUGUSTUS	exon	537718	539328	.	+	.	ID=MALK_03435.t1.e1;Parent=MALK_03435.t1
contig011	AUGUSTUS	gene	539355	540485	0.63	-	.	ID=MALK_03436;prediction_source=augustus:contig011.g1828.t1
contig011	AUGUSTUS	CDS	539355	540485	0.63	-	0	ID=MALK_03436.t1.c1;Parent=MALK_03436.t1
contig011	AUGUSTUS	mRNA	539355	540485	0.63	-	.	ID=MALK_03436.t1;Parent=MALK_03436
contig011	AUGUSTUS	exon	539355	540485	0.63	-	.	ID=MALK_03436.t1.e1;Parent=MALK_03436.t1
contig011	AUGUSTUS	gene	540708	540977	0.78	+	.	ID=MALK_03437;prediction_source=braker_MRET:g3775.t1
contig011	AUGUSTUS	CDS	540708	540977	0.78	+	0	ID=MALK_03437.t1.c1;Parent=MALK_03437.t1
contig011	AUGUSTUS	mRNA	540708	540977	0.78	+	.	ID=MALK_03437.t1;Parent=MALK_03437
contig011	AUGUSTUS	exon	540708	540977	.	+	.	ID=MALK_03437.t1.e1;Parent=MALK_03437.t1
contig011	AUGUSTUS	gene	540980	541639	0.9	-	.	ID=MALK_03438;prediction_source=braker_MRET:g3776.t1
contig011	AUGUSTUS	CDS	540980	541639	0.9	-	0	ID=MALK_03438.t1.c1;Parent=MALK_03438.t1
contig011	AUGUSTUS	mRNA	540980	541639	0.9	-	.	ID=MALK_03438.t1;Parent=MALK_03438
contig011	AUGUSTUS	exon	540980	541639	.	-	.	ID=MALK_03438.t1.e1;Parent=MALK_03438.t1
contig011	AUGUSTUS	gene	541739	541996	0.65	+	.	ID=MALK_03439;prediction_source=braker_MRET:g3777.t1
contig011	AUGUSTUS	CDS	541739	541996	0.65	+	0	ID=MALK_03439.t1.c1;Parent=MALK_03439.t1
contig011	AUGUSTUS	mRNA	541739	541996	0.65	+	.	ID=MALK_03439.t1;Parent=MALK_03439
contig011	AUGUSTUS	exon	541739	541996	.	+	.	ID=MALK_03439.t1.e1;Parent=MALK_03439.t1
contig011	AUGUSTUS	gene	542014	547552	0.31	-	.	ID=MALK_03440;prediction_source=braker_MRET:g3778.t1
contig011	AUGUSTUS	CDS	546972	547552	0.41	-	0	ID=MALK_03440.t1.c2;Parent=MALK_03440.t1
contig011	AUGUSTUS	CDS	542014	546859	0.41	-	0	ID=MALK_03440.t1.c1;Parent=MALK_03440.t1
contig011	AUGUSTUS	mRNA	542014	547552	0.31	-	.	ID=MALK_03440.t1;Parent=MALK_03440
contig011	AUGUSTUS	exon	546972	547552	.	-	.	ID=MALK_03440.t1.e2;Parent=MALK_03440.t1
contig011	AUGUSTUS	exon	542014	546859	.	-	.	ID=MALK_03440.t1.e1;Parent=MALK_03440.t1
contig011	AUGUSTUS	gene	547621	548673	0.82	-	.	ID=MALK_03441;prediction_source=braker_MRET:g3779.t1
contig011	AUGUSTUS	CDS	547621	548673	0.82	-	0	ID=MALK_03441.t1.c1;Parent=MALK_03441.t1
contig011	AUGUSTUS	mRNA	547621	548673	0.82	-	.	ID=MALK_03441.t1;Parent=MALK_03441
contig011	AUGUSTUS	exon	547621	548673	.	-	.	ID=MALK_03441.t1.e1;Parent=MALK_03441.t1
contig011	AUGUSTUS	gene	548782	549360	0.99	+	.	ID=MALK_03442;prediction_source=braker_MRET:g3780.t1
contig011	AUGUSTUS	CDS	548782	549360	0.99	+	0	ID=MALK_03442.t1.c1;Parent=MALK_03442.t1
contig011	AUGUSTUS	mRNA	548782	549360	0.99	+	.	ID=MALK_03442.t1;Parent=MALK_03442
contig011	AUGUSTUS	exon	548782	549360	.	+	.	ID=MALK_03442.t1.e1;Parent=MALK_03442.t1
contig011	AUGUSTUS	gene	549457	550602	0.99	+	.	ID=MALK_03443;prediction_source=braker_MRET:g3781.t1
contig011	AUGUSTUS	CDS	549457	550602	0.99	+	0	ID=MALK_03443.t1.c1;Parent=MALK_03443.t1
contig011	AUGUSTUS	mRNA	549457	550602	0.99	+	.	ID=MALK_03443.t1;Parent=MALK_03443
contig011	AUGUSTUS	exon	549457	550602	.	+	.	ID=MALK_03443.t1.e1;Parent=MALK_03443.t1
contig011	AUGUSTUS	gene	550690	551916	0.66	+	.	ID=MALK_03444;prediction_source=braker_MRET:g3782.t1
contig011	AUGUSTUS	CDS	550690	551916	0.66	+	0	ID=MALK_03444.t1.c1;Parent=MALK_03444.t1
contig011	AUGUSTUS	mRNA	550690	551916	0.66	+	.	ID=MALK_03444.t1;Parent=MALK_03444

contig011	AUGUSTUS	exon	550690	551916	.	+	.	ID=MALK_03444.t1.e1;Parent=MALK_03444.t1
contig011	AUGUSTUS	gene	552106	553957	0.73	-	.	ID=MALK_03445;prediction_source=braker_MRET:g3783.t1
contig011	AUGUSTUS	CDS	552255	553957	0.73	-	0	ID=MALK_03445.t1.c2;Parent=MALK_03445.t1
contig011	AUGUSTUS	CDS	552106	552160	0.73	-	0	ID=MALK_03445.t1.c1;Parent=MALK_03445.t1
contig011	AUGUSTUS	mRNA	552106	553957	0.73	-	.	ID=MALK_03445.t1;Parent=MALK_03445
contig011	AUGUSTUS	exon	552255	553957	.	-	.	ID=MALK_03445.t1.e2;Parent=MALK_03445.t1
contig011	AUGUSTUS	exon	552106	552160	.	-	.	ID=MALK_03445.t1.e1;Parent=MALK_03445.t1
contig011	AUGUSTUS	gene	554087	554950	0.97	+	.	ID=MALK_03446;prediction_source=braker_MRET:g3784.t1
contig011	AUGUSTUS	CDS	554087	554950	0.97	+	0	ID=MALK_03446.t1.c1;Parent=MALK_03446.t1
contig011	AUGUSTUS	mRNA	554087	554950	0.97	+	.	ID=MALK_03446.t1;Parent=MALK_03446
contig011	AUGUSTUS	exon	554087	554950	.	+	.	ID=MALK_03446.t1.e1;Parent=MALK_03446.t1
contig011	AUGUSTUS	gene	554957	556171	1	-	.	ID=MALK_03447;prediction_source=braker_MRET:g3785.t1
contig011	AUGUSTUS	CDS	554957	556171	1	-	0	ID=MALK_03447.t1.c1;Parent=MALK_03447.t1
contig011	AUGUSTUS	mRNA	554957	556171	1	-	.	ID=MALK_03447.t1;Parent=MALK_03447
contig011	AUGUSTUS	exon	554957	556171	.	-	.	ID=MALK_03447.t1.e1;Parent=MALK_03447.t1
contig011	AUGUSTUS	gene	556309	556896	0.81	+	.	ID=MALK_03448;prediction_source=braker_MRET:g3786.t1
contig011	AUGUSTUS	CDS	556309	556896	0.81	+	0	ID=MALK_03448.t1.c1;Parent=MALK_03448.t1
contig011	AUGUSTUS	mRNA	556309	556896	0.81	+	.	ID=MALK_03448.t1;Parent=MALK_03448
contig011	AUGUSTUS	exon	556309	556896	.	+	.	ID=MALK_03448.t1.e1;Parent=MALK_03448.t1
contig011	AUGUSTUS	gene	557188	559563	0.89	+	.	ID=MALK_03449;prediction_source=augustus:contig011.g1835.t1
contig011	AUGUSTUS	CDS	557188	559563	0.89	+	0	ID=MALK_03449.t1.c1;Parent=MALK_03449.t1
contig011	AUGUSTUS	mRNA	557188	559563	0.89	+	.	ID=MALK_03449.t1;Parent=MALK_03449
contig011	AUGUSTUS	exon	557188	559563	0.89	+	.	ID=MALK_03449.t1.e1;Parent=MALK_03449.t1
contig011	AUGUSTUS	gene	559605	561249	0.69	-	.	ID=MALK_03450;prediction_source=braker_MRET:g3788.t1
contig011	AUGUSTUS	CDS	559741	561249	0.76	-	0	ID=MALK_03450.t1.c2;Parent=MALK_03450.t1
contig011	AUGUSTUS	CDS	559605	559616	0.76	-	0	ID=MALK_03450.t1.c1;Parent=MALK_03450.t1
contig011	AUGUSTUS	mRNA	559605	561249	0.69	-	.	ID=MALK_03450.t1;Parent=MALK_03450
contig011	AUGUSTUS	exon	559741	561249	.	-	.	ID=MALK_03450.t1.e2;Parent=MALK_03450.t1
contig011	AUGUSTUS	exon	559605	559616	.	-	.	ID=MALK_03450.t1.e1;Parent=MALK_03450.t1
contig011	AUGUSTUS	gene	561365	562234	0.76	+	.	ID=MALK_03451;prediction_source=braker_MRET:g3789.t1
contig011	AUGUSTUS	CDS	561365	562234	0.76	+	0	ID=MALK_03451.t1.c1;Parent=MALK_03451.t1
contig011	AUGUSTUS	mRNA	561365	562234	0.76	+	.	ID=MALK_03451.t1;Parent=MALK_03451
contig011	AUGUSTUS	exon	561365	562234	.	+	.	ID=MALK_03451.t1.e1;Parent=MALK_03451.t1
contig011	AUGUSTUS	gene	562307	563713	1	+	.	ID=MALK_03452;prediction_source=braker_MRET:g3790.t1
contig011	AUGUSTUS	CDS	562307	563713	1	+	0	ID=MALK_03452.t1.c1;Parent=MALK_03452.t1
contig011	AUGUSTUS	mRNA	562307	563713	1	+	.	ID=MALK_03452.t1;Parent=MALK_03452
contig011	AUGUSTUS	exon	562307	563713	.	+	.	ID=MALK_03452.t1.e1;Parent=MALK_03452.t1
contig011	maker	gene	563974	564693	.	+	.	ID=MALK_03453;prediction_source=maker_MRET:augustus_masked-contig011-processed-gene-5.108-mRNA-1
contig011	maker	CDS	563974	564693	.	+	0	ID=MALK_03453.t1.c1;Parent=MALK_03453.t1
contig011	maker	mRNA	563974	564693	.	+	.	ID=MALK_03453.t1;Parent=MALK_03453
contig011	maker	exon	563974	564693	.	+	.	ID=MALK_03453.t1.e1;Parent=MALK_03453.t1
contig011	AUGUSTUS	gene	564690	565301	0.91	-	.	ID=MALK_03454;prediction_source=augustus:contig011.g1840.t1
contig011	AUGUSTUS	CDS	564690	565301	0.91	-	0	ID=MALK_03454.t1.c1;Parent=MALK_03454.t1

contig011	AUGUSTUS	mRNA	564690	565301	0.91	-	.	ID=MALK_03454.t1;Parent=MALK_03454
contig011	AUGUSTUS	exon	564690	565301	0.91	-	.	ID=MALK_03454.t1.e1;Parent=MALK_03454.t1
contig011	AUGUSTUS	gene	565255	566244	0.95	+	.	ID=MALK_03455;prediction_source=augustus:contig011.g1841.t1
contig011	AUGUSTUS	CDS	565255	566244	0.95	+	0	ID=MALK_03455.t1.c1;Parent=MALK_03455.t1
contig011	AUGUSTUS	mRNA	565255	566244	0.95	+	.	ID=MALK_03455.t1;Parent=MALK_03455
contig011	AUGUSTUS	exon	565255	566244	0.95	+	.	ID=MALK_03455.t1.e1;Parent=MALK_03455.t1
contig011	AUGUSTUS	gene	566381	567463	0.84	+	.	ID=MALK_03456;prediction_source=augustus:contig011.g1842.t1
contig011	AUGUSTUS	CDS	566381	567463	0.84	+	0	ID=MALK_03456.t1.c1;Parent=MALK_03456.t1
contig011	AUGUSTUS	mRNA	566381	567463	0.84	+	.	ID=MALK_03456.t1;Parent=MALK_03456
contig011	AUGUSTUS	exon	566381	567463	0.84	+	.	ID=MALK_03456.t1.e1;Parent=MALK_03456.t1
contig011	maker	gene	567441	568970	.	-	.	ID=MALK_03457;prediction_source=maker_MRET:augustus_masked-contig011-processed-gene-5.136-mRNA-1
contig011	maker	CDS	567441	568970	.	-	0	ID=MALK_03457.t1.c1;Parent=MALK_03457.t1
contig011	maker	mRNA	567441	568970	.	-	.	ID=MALK_03457.t1;Parent=MALK_03457
contig011	maker	exon	567441	568970	.	-	.	ID=MALK_03457.t1.e1;Parent=MALK_03457.t1
contig011	AUGUSTUS	gene	569072	569593	0.91	+	.	ID=MALK_03458;prediction_source=braker_MRET:g3796.t1
contig011	AUGUSTUS	CDS	569072	569593	0.91	+	0	ID=MALK_03458.t1.c1;Parent=MALK_03458.t1
contig011	AUGUSTUS	mRNA	569072	569593	0.91	+	.	ID=MALK_03458.t1;Parent=MALK_03458
contig011	AUGUSTUS	exon	569072	569593	.	+	.	ID=MALK_03458.t1.e1;Parent=MALK_03458.t1
contig011	AUGUSTUS	gene	569610	570653	0.5	-	.	ID=MALK_03459;prediction_source=braker_MRET:g3797.t1
contig011	AUGUSTUS	CDS	569610	570653	0.5	-	0	ID=MALK_03459.t1.c1;Parent=MALK_03459.t1
contig011	AUGUSTUS	mRNA	569610	570653	0.5	-	.	ID=MALK_03459.t1;Parent=MALK_03459
contig011	AUGUSTUS	exon	569610	570653	.	-	.	ID=MALK_03459.t1.e1;Parent=MALK_03459.t1
contig011	AUGUSTUS	gene	570979	572034	0.53	+	.	ID=MALK_03460;prediction_source=braker_MRET:g3798.t1
contig011	AUGUSTUS	CDS	570979	572034	0.53	+	0	ID=MALK_03460.t1.c1;Parent=MALK_03460.t1
contig011	AUGUSTUS	mRNA	570979	572034	0.53	+	.	ID=MALK_03460.t1;Parent=MALK_03460
contig011	AUGUSTUS	exon	570979	572034	.	+	.	ID=MALK_03460.t1.e1;Parent=MALK_03460.t1
contig011	AUGUSTUS	gene	572132	573922	0.65	+	.	ID=MALK_03461;prediction_source=braker_MRET:g3799.t1
contig011	AUGUSTUS	CDS	572132	573922	0.65	+	0	ID=MALK_03461.t1.c1;Parent=MALK_03461.t1
contig011	AUGUSTUS	mRNA	572132	573922	0.65	+	.	ID=MALK_03461.t1;Parent=MALK_03461
contig011	AUGUSTUS	exon	572132	573922	.	+	.	ID=MALK_03461.t1.e1;Parent=MALK_03461.t1
contig011	AUGUSTUS	gene	574036	574852	0.99	+	.	ID=MALK_03462;prediction_source=braker_MRET:g3800.t1
contig011	AUGUSTUS	CDS	574036	574195	1	+	0	ID=MALK_03462.t1.c1;Parent=MALK_03462.t1
contig011	AUGUSTUS	CDS	574230	574852	1	+	0	ID=MALK_03462.t1.c2;Parent=MALK_03462.t1
contig011	AUGUSTUS	mRNA	574036	574852	0.99	+	.	ID=MALK_03462.t1;Parent=MALK_03462
contig011	AUGUSTUS	exon	574036	574195	.	+	.	ID=MALK_03462.t1.e1;Parent=MALK_03462.t1
contig011	AUGUSTUS	exon	574230	574852	.	+	.	ID=MALK_03462.t1.e2;Parent=MALK_03462.t1
contig011	AUGUSTUS	gene	575380	577161	0.98	-	.	ID=MALK_03463;prediction_source=augustus:contig011.g1845.t1
contig011	AUGUSTUS	CDS	575380	577161	0.98	-	0	ID=MALK_03463.t1.c1;Parent=MALK_03463.t1
contig011	AUGUSTUS	mRNA	575380	577161	0.98	-	.	ID=MALK_03463.t1;Parent=MALK_03463
contig011	AUGUSTUS	exon	575380	577161	0.98	-	.	ID=MALK_03463.t1.e1;Parent=MALK_03463.t1
contig011	AUGUSTUS	gene	577229	578391	0.24	-	.	ID=MALK_03464;prediction_source=braker_MRET:g3802.t1
contig011	AUGUSTUS	CDS	577793	578391	0.55	-	0	ID=MALK_03464.t1.c2;Parent=MALK_03464.t1
contig011	AUGUSTUS	CDS	577229	577688	0.55	-	0	ID=MALK_03464.t1.c1;Parent=MALK_03464.t1

contig011	AUGUSTUS	mRNA	577229	578391	0.24	-	.	ID=MALK_03464.t1;Parent=MALK_03464
contig011	AUGUSTUS	exon	577793	578391	.	-	.	ID=MALK_03464.t1.e2;Parent=MALK_03464.t1
contig011	AUGUSTUS	exon	577229	577688	.	-	.	ID=MALK_03464.t1.e1;Parent=MALK_03464.t1
contig011	AUGUSTUS	gene	578974	579888	1	-	.	ID=MALK_03465;prediction_source=braker_MRET:g3803.t1
contig011	AUGUSTUS	CDS	578974	579888	1	-	0	ID=MALK_03465.t1.c1;Parent=MALK_03465.t1
contig011	AUGUSTUS	mRNA	578974	579888	1	-	.	ID=MALK_03465.t1;Parent=MALK_03465
contig011	AUGUSTUS	exon	578974	579888	.	-	.	ID=MALK_03465.t1.e1;Parent=MALK_03465.t1
contig011	AUGUSTUS	gene	580002	580511	0.55	+	.	ID=MALK_03466;prediction_source=braker_MRET:g3804.t1
contig011	AUGUSTUS	CDS	580002	580004	0.55	+	0	ID=MALK_03466.t1.c1;Parent=MALK_03466.t1
contig011	AUGUSTUS	CDS	580036	580189	0.55	+	0	ID=MALK_03466.t1.c2;Parent=MALK_03466.t1
contig011	AUGUSTUS	CDS	580273	580511	0.55	+	0	ID=MALK_03466.t1.c3;Parent=MALK_03466.t1
contig011	AUGUSTUS	mRNA	580002	580511	0.55	+	.	ID=MALK_03466.t1;Parent=MALK_03466
contig011	AUGUSTUS	exon	580002	580004	.	+	.	ID=MALK_03466.t1.e1;Parent=MALK_03466.t1
contig011	AUGUSTUS	exon	580036	580189	.	+	.	ID=MALK_03466.t1.e2;Parent=MALK_03466.t1
contig011	AUGUSTUS	exon	580273	580511	.	+	.	ID=MALK_03466.t1.e3;Parent=MALK_03466.t1
contig011	maker	gene	580624	580956	.	-	.	ID=MALK_03467;prediction_source=maker_MRET:augustus_masked-contig011-processed-gene-5.139-mRNA-1
contig011	maker	CDS	580624	580956	.	-	0	ID=MALK_03467.t1.c1;Parent=MALK_03467.t1
contig011	maker	mRNA	580624	580956	.	-	.	ID=MALK_03467.t1;Parent=MALK_03467
contig011	maker	exon	580624	580956	.	-	.	ID=MALK_03467.t1.e1;Parent=MALK_03467.t1
contig011	maker	gene	581410	581784	.	-	.	ID=MALK_03468;prediction_source=maker_MRET:augustus_masked-contig011-processed-gene-5.140-mRNA-1
contig011	maker	CDS	581410	581784	.	-	0	ID=MALK_03468.t1.c1;Parent=MALK_03468.t1
contig011	maker	mRNA	581410	581784	.	-	.	ID=MALK_03468.t1;Parent=MALK_03468
contig011	maker	exon	581410	581784	.	-	.	ID=MALK_03468.t1.e1;Parent=MALK_03468.t1
contig011	maker	gene	581989	582616	.	+	.	ID=MALK_03469;prediction_source=maker_MRET:augustus_masked-contig011-processed-gene-5.111-mRNA-1
contig011	maker	CDS	581989	582168	.	+	0	ID=MALK_03469.t1.c1;Parent=MALK_03469.t1
contig011	maker	CDS	582256	582363	.	+	0	ID=MALK_03469.t1.c2;Parent=MALK_03469.t1
contig011	maker	CDS	582428	582616	.	+	0	ID=MALK_03469.t1.c3;Parent=MALK_03469.t1
contig011	maker	mRNA	581989	582616	.	+	.	ID=MALK_03469.t1;Parent=MALK_03469
contig011	maker	exon	581989	582168	.	+	.	ID=MALK_03469.t1.e1;Parent=MALK_03469.t1
contig011	maker	exon	582256	582363	.	+	.	ID=MALK_03469.t1.e2;Parent=MALK_03469.t1
contig011	maker	exon	582428	582616	.	+	.	ID=MALK_03469.t1.e3;Parent=MALK_03469.t1
contig011	AUGUSTUS	gene	582842	583927	0.87	+	.	ID=MALK_03470;prediction_source=augustus:contig011.g1850.t1
contig011	AUGUSTUS	CDS	582842	583927	0.87	+	0	ID=MALK_03470.t1.c1;Parent=MALK_03470.t1
contig011	AUGUSTUS	mRNA	582842	583927	0.87	+	.	ID=MALK_03470.t1;Parent=MALK_03470
contig011	AUGUSTUS	exon	582842	583927	0.87	+	.	ID=MALK_03470.t1.e1;Parent=MALK_03470.t1
contig011	AUGUSTUS	gene	583886	585760	0.94	-	.	ID=MALK_03471;prediction_source=augustus:contig011.g1851.t1
contig011	AUGUSTUS	CDS	583886	585760	0.94	-	0	ID=MALK_03471.t1.c1;Parent=MALK_03471.t1
contig011	AUGUSTUS	mRNA	583886	585760	0.94	-	.	ID=MALK_03471.t1;Parent=MALK_03471
contig011	AUGUSTUS	exon	583886	585760	0.94	-	.	ID=MALK_03471.t1.e1;Parent=MALK_03471.t1
contig011	AUGUSTUS	gene	586114	587172	0.89	+	.	ID=MALK_03472;prediction_source=braker_MRET:g3808.t1
contig011	AUGUSTUS	CDS	586114	587172	0.89	+	0	ID=MALK_03472.t1.c1;Parent=MALK_03472.t1
contig011	AUGUSTUS	mRNA	586114	587172	0.89	+	.	ID=MALK_03472.t1;Parent=MALK_03472
contig011	AUGUSTUS	exon	586114	587172	.	+	.	ID=MALK_03472.t1.e1;Parent=MALK_03472.t1

contig011	AUGUSTUS	gene	587418	587837	0.83	+	.	ID=MALK_03473;prediction_source=augustus:contig011.g1853.t1
contig011	AUGUSTUS	CDS	587418	587837	0.83	+	0	ID=MALK_03473.t1.c1;Parent=MALK_03473.t1
contig011	AUGUSTUS	mRNA	587418	587837	0.83	+	.	ID=MALK_03473.t1;Parent=MALK_03473
contig011	AUGUSTUS	exon	587418	587837	0.83	+	.	ID=MALK_03473.t1.e1;Parent=MALK_03473.t1
contig011	AUGUSTUS	gene	587985	588429	0.84	+	.	ID=MALK_03474;prediction_source=braker_MRET:g3810.t1
contig011	AUGUSTUS	CDS	587985	588048	1	+	0	ID=MALK_03474.t1.c1;Parent=MALK_03474.t1
contig011	AUGUSTUS	CDS	588083	588429	1	+	0	ID=MALK_03474.t1.c2;Parent=MALK_03474.t1
contig011	AUGUSTUS	mRNA	587985	588429	0.84	+	.	ID=MALK_03474.t1;Parent=MALK_03474
contig011	AUGUSTUS	exon	587985	588048	.	+	.	ID=MALK_03474.t1.e1;Parent=MALK_03474.t1
contig011	AUGUSTUS	exon	588083	588429	.	+	.	ID=MALK_03474.t1.e2;Parent=MALK_03474.t1
contig011	AUGUSTUS	gene	588460	588789	0.79	-	.	ID=MALK_03475;prediction_source=augustus:contig011.g1854.t1
contig011	AUGUSTUS	CDS	588460	588789	0.79	-	0	ID=MALK_03475.t1.c1;Parent=MALK_03475.t1
contig011	AUGUSTUS	mRNA	588460	588789	0.79	-	.	ID=MALK_03475.t1;Parent=MALK_03475
contig011	AUGUSTUS	exon	588460	588789	0.79	-	.	ID=MALK_03475.t1.e1;Parent=MALK_03475.t1
contig011	AUGUSTUS	gene	589106	590477	0.47	+	.	ID=MALK_03476;prediction_source=braker_MRET:g3812.t1
contig011	AUGUSTUS	CDS	589106	590400	0.87	+	0	ID=MALK_03476.t1.c1;Parent=MALK_03476.t1
contig011	AUGUSTUS	CDS	590459	590477	0.87	+	0	ID=MALK_03476.t1.c2;Parent=MALK_03476.t1
contig011	AUGUSTUS	mRNA	589106	590477	0.47	+	.	ID=MALK_03476.t1;Parent=MALK_03476
contig011	AUGUSTUS	exon	589106	590400	.	+	.	ID=MALK_03476.t1.e1;Parent=MALK_03476.t1
contig011	AUGUSTUS	exon	590459	590477	.	+	.	ID=MALK_03476.t1.e2;Parent=MALK_03476.t1
contig011	AUGUSTUS	gene	590489	592795	0.56	-	.	ID=MALK_03477;prediction_source=braker_MRET:g3813.t1
contig011	AUGUSTUS	CDS	590489	592795	0.56	-	0	ID=MALK_03477.t1.c1;Parent=MALK_03477.t1
contig011	AUGUSTUS	mRNA	590489	592795	0.56	-	.	ID=MALK_03477.t1;Parent=MALK_03477
contig011	AUGUSTUS	exon	590489	592795	.	-	.	ID=MALK_03477.t1.e1;Parent=MALK_03477.t1
contig011	AUGUSTUS	gene	592819	593731	0.46	-	.	ID=MALK_03478;prediction_source=braker_MRET:g3814.t1
contig011	AUGUSTUS	CDS	593695	593731	0.65	-	0	ID=MALK_03478.t1.c2;Parent=MALK_03478.t1
contig011	AUGUSTUS	CDS	592819	593663	0.65	-	0	ID=MALK_03478.t1.c1;Parent=MALK_03478.t1
contig011	AUGUSTUS	mRNA	592819	593731	0.46	-	.	ID=MALK_03478.t1;Parent=MALK_03478
contig011	AUGUSTUS	exon	593695	593731	.	-	.	ID=MALK_03478.t1.e2;Parent=MALK_03478.t1
contig011	AUGUSTUS	exon	592819	593663	.	-	.	ID=MALK_03478.t1.e1;Parent=MALK_03478.t1
contig011	AUGUSTUS	gene	593852	594922	1	+	.	ID=MALK_03479;prediction_source=braker_MRET:g3815.t1
contig011	AUGUSTUS	CDS	593852	594922	1	+	0	ID=MALK_03479.t1.c1;Parent=MALK_03479.t1
contig011	AUGUSTUS	mRNA	593852	594922	1	+	.	ID=MALK_03479.t1;Parent=MALK_03479
contig011	AUGUSTUS	exon	593852	594922	.	+	.	ID=MALK_03479.t1.e1;Parent=MALK_03479.t1
contig011	AUGUSTUS	gene	595014	597485	0.96	+	.	ID=MALK_03480;prediction_source=braker_MRET:g3816.t1
contig011	AUGUSTUS	CDS	595014	597485	0.96	+	0	ID=MALK_03480.t1.c1;Parent=MALK_03480.t1
contig011	AUGUSTUS	mRNA	595014	597485	0.96	+	.	ID=MALK_03480.t1;Parent=MALK_03480
contig011	AUGUSTUS	exon	595014	597485	.	+	.	ID=MALK_03480.t1.e1;Parent=MALK_03480.t1
contig011	maker	gene	597494	598996	.	-	.	ID=MALK_03481;prediction_source=maker_MRET:augustus_masked-contig011-processed-gene-5.143-mRNA-1
contig011	maker	CDS	597494	598996	.	-	0	ID=MALK_03481.t1.c1;Parent=MALK_03481.t1
contig011	maker	mRNA	597494	598996	.	-	.	ID=MALK_03481.t1;Parent=MALK_03481
contig011	maker	exon	597494	598996	.	-	.	ID=MALK_03481.t1.e1;Parent=MALK_03481.t1
contig011	maker	gene	599128	600861	.	+	.	ID=MALK_03482;prediction_source=maker_MRET:augustus_masked-contig011-processed-gene-5.117-mRNA-1

contig011	maker	CDS	599128	600861	.	+	0	ID=MALK_03482.t1.c1;Parent=MALK_03482.t1
contig011	maker	mRNA	599128	600861	.	+	.	ID=MALK_03482.t1;Parent=MALK_03482
contig011	maker	exon	599128	600861	.	+	.	ID=MALK_03482.t1.e1;Parent=MALK_03482.t1
contig011	maker	gene	600973	601656	.	+	.	ID=MALK_03483;prediction_source=maker_MRET:augustus_masked-contig011-processed-gene-5.118-mRNA-1
contig011	maker	CDS	600973	601656	.	+	0	ID=MALK_03483.t1.c1;Parent=MALK_03483.t1
contig011	maker	mRNA	600973	601656	.	+	.	ID=MALK_03483.t1;Parent=MALK_03483
contig011	maker	exon	600973	601656	.	+	.	ID=MALK_03483.t1.e1;Parent=MALK_03483.t1
contig011	maker	gene	602397	604433	.	+	.	ID=MALK_03484;prediction_source=maker_MRET:augustus_masked-contig011-processed-gene-5.119-mRNA-1
contig011	maker	CDS	602397	604433	.	+	0	ID=MALK_03484.t1.c1;Parent=MALK_03484.t1
contig011	maker	mRNA	602397	604433	.	+	.	ID=MALK_03484.t1;Parent=MALK_03484
contig011	maker	exon	602397	604433	.	+	.	ID=MALK_03484.t1.e1;Parent=MALK_03484.t1
contig011	AUGUSTUS	gene	604499	607174	0.65	-	.	ID=MALK_03485;prediction_source=braker_MRET:g3821.t1
contig011	AUGUSTUS	CDS	604499	607174	0.65	-	0	ID=MALK_03485.t1.c1;Parent=MALK_03485.t1
contig011	AUGUSTUS	mRNA	604499	607174	0.65	-	.	ID=MALK_03485.t1;Parent=MALK_03485
contig011	AUGUSTUS	exon	604499	607174	.	-	.	ID=MALK_03485.t1.e1;Parent=MALK_03485.t1
contig011	AUGUSTUS	gene	607361	617902	0.36	+	.	ID=MALK_03486;prediction_source=augustus:contig011.g1865.t1
contig011	AUGUSTUS	CDS	607361	617902	0.36	+	0	ID=MALK_03486.t1.c1;Parent=MALK_03486.t1
contig011	AUGUSTUS	mRNA	607361	617902	0.36	+	.	ID=MALK_03486.t1;Parent=MALK_03486
contig011	AUGUSTUS	exon	607361	617902	0.36	+	.	ID=MALK_03486.t1.e1;Parent=MALK_03486.t1
contig011	maker	gene	618903	620099	.	-	.	ID=MALK_03487;prediction_source=maker_MRET:augustus_masked-contig011-processed-gene-5.145-mRNA-1
contig011	maker	CDS	618903	620099	.	-	0	ID=MALK_03487.t1.c1;Parent=MALK_03487.t1
contig011	maker	mRNA	618903	620099	.	-	.	ID=MALK_03487.t1;Parent=MALK_03487
contig011	maker	exon	618903	620099	.	-	.	ID=MALK_03487.t1.e1;Parent=MALK_03487.t1
contig011	AUGUSTUS	gene	620512	622553	1	-	.	ID=MALK_03488;prediction_source=braker_MRET:g3824.t1
contig011	AUGUSTUS	CDS	620844	622553	1	-	0	ID=MALK_03488.t1.c3;Parent=MALK_03488.t1
contig011	AUGUSTUS	CDS	620709	620813	1	-	0	ID=MALK_03488.t1.c2;Parent=MALK_03488.t1
contig011	AUGUSTUS	CDS	620512	620667	1	-	0	ID=MALK_03488.t1.c1;Parent=MALK_03488.t1
contig011	AUGUSTUS	mRNA	620512	622553	1	-	.	ID=MALK_03488.t1;Parent=MALK_03488
contig011	AUGUSTUS	exon	620844	622553	.	-	.	ID=MALK_03488.t1.e3;Parent=MALK_03488.t1
contig011	AUGUSTUS	exon	620709	620813	.	-	.	ID=MALK_03488.t1.e2;Parent=MALK_03488.t1
contig011	AUGUSTUS	exon	620512	620667	.	-	.	ID=MALK_03488.t1.e1;Parent=MALK_03488.t1
contig011	maker	gene	622809	624668	.	-	.	ID=MALK_03489;prediction_source=maker_MRET:augustus_masked-contig011-processed-gene-5.147-mRNA-1
contig011	maker	CDS	622809	624668	.	-	0	ID=MALK_03489.t1.c1;Parent=MALK_03489.t1
contig011	maker	mRNA	622809	624668	.	-	.	ID=MALK_03489.t1;Parent=MALK_03489
contig011	maker	exon	622809	624668	.	-	.	ID=MALK_03489.t1.e1;Parent=MALK_03489.t1
contig011	maker	gene	624814	625740	.	+	.	ID=MALK_03490;prediction_source=maker_MRET:augustus_masked-contig011-processed-gene-5.121-mRNA-1
contig011	maker	CDS	624814	625740	.	+	0	ID=MALK_03490.t1.c1;Parent=MALK_03490.t1
contig011	maker	mRNA	624814	625740	.	+	.	ID=MALK_03490.t1;Parent=MALK_03490
contig011	maker	exon	624814	625740	.	+	.	ID=MALK_03490.t1.e1;Parent=MALK_03490.t1
contig011	AUGUSTUS	gene	625853	627169	0.37	+	.	ID=MALK_03491;prediction_source=braker_MRET:g3827.t1
contig011	AUGUSTUS	CDS	625853	626221	0.4	+	0	ID=MALK_03491.t1.c1;Parent=MALK_03491.t1
contig011	AUGUSTUS	CDS	626267	627169	0.4	+	0	ID=MALK_03491.t1.c2;Parent=MALK_03491.t1
contig011	AUGUSTUS	mRNA	625853	627169	0.37	+	.	ID=MALK_03491.t1;Parent=MALK_03491

contig011	AUGUSTUS	exon	625853	626221	.	+	.	ID=MALK_03491.t1.e1;Parent=MALK_03491.t1
contig011	AUGUSTUS	exon	626267	627169	.	+	.	ID=MALK_03491.t1.e2;Parent=MALK_03491.t1
contig011	AUGUSTUS	gene	627261	627713	0.25	+	.	ID=MALK_03492;prediction_source=braker_MRET:g3828.t2
contig011	AUGUSTUS	CDS	627261	627272	0.43	+	0	ID=MALK_03492.t1.c1;Parent=MALK_03492.t1
contig011	AUGUSTUS	CDS	627303	627713	0.43	+	0	ID=MALK_03492.t1.c2;Parent=MALK_03492.t1
contig011	AUGUSTUS	mRNA	627261	627713	0.25	+	.	ID=MALK_03492.t1;Parent=MALK_03492
contig011	AUGUSTUS	exon	627261	627272	.	+	.	ID=MALK_03492.t1.e1;Parent=MALK_03492.t1
contig011	AUGUSTUS	exon	627303	627713	.	+	.	ID=MALK_03492.t1.e2;Parent=MALK_03492.t1
contig011	maker	gene	627841	628707	.	+	.	ID=MALK_03493;prediction_source=maker_MRET:augustus_masked-contig011-processed-gene-5.122-mRNA-1
contig011	maker	CDS	627841	628707	.	+	0	ID=MALK_03493.t1.c1;Parent=MALK_03493.t1
contig011	maker	mRNA	627841	628707	.	+	.	ID=MALK_03493.t1;Parent=MALK_03493
contig011	maker	exon	627841	628707	.	+	.	ID=MALK_03493.t1.e1;Parent=MALK_03493.t1
contig011	maker	gene	628787	629953	.	+	.	ID=MALK_03494;prediction_source=maker_MRET:augustus_masked-contig011-processed-gene-5.123-mRNA-1
contig011	maker	CDS	628787	629953	.	+	0	ID=MALK_03494.t1.c1;Parent=MALK_03494.t1
contig011	maker	mRNA	628787	629953	.	+	.	ID=MALK_03494.t1;Parent=MALK_03494
contig011	maker	exon	628787	629953	.	+	.	ID=MALK_03494.t1.e1;Parent=MALK_03494.t1
contig011	AUGUSTUS	gene	630036	632420	0.99	-	.	ID=MALK_03495;prediction_source=augustus:contig011.g1875.t1
contig011	AUGUSTUS	CDS	630036	632420	0.99	-	0	ID=MALK_03495.t1.c1;Parent=MALK_03495.t1
contig011	AUGUSTUS	mRNA	630036	632420	0.99	-	.	ID=MALK_03495.t1;Parent=MALK_03495
contig011	AUGUSTUS	exon	630036	632420	0.99	-	.	ID=MALK_03495.t1.e1;Parent=MALK_03495.t1
contig011	maker	gene	632461	634284	.	-	.	ID=MALK_03496;prediction_source=maker_MRET:augustus_masked-contig011-processed-gene-5.149-mRNA-1
contig011	maker	CDS	632461	634284	.	-	0	ID=MALK_03496.t1.c1;Parent=MALK_03496.t1
contig011	maker	mRNA	632461	634284	.	-	.	ID=MALK_03496.t1;Parent=MALK_03496
contig011	maker	exon	632461	634284	.	-	.	ID=MALK_03496.t1.e1;Parent=MALK_03496.t1
contig011	AUGUSTUS	gene	634353	635157	0.71	+	.	ID=MALK_03497;prediction_source=braker_MRET:g3832.t1
contig011	AUGUSTUS	CDS	634353	634921	0.88	+	0	ID=MALK_03497.t1.c1;Parent=MALK_03497.t1
contig011	AUGUSTUS	CDS	635052	635157	0.88	+	0	ID=MALK_03497.t1.c2;Parent=MALK_03497.t1
contig011	AUGUSTUS	mRNA	634353	635157	0.71	+	.	ID=MALK_03497.t1;Parent=MALK_03497
contig011	AUGUSTUS	exon	634353	634921	.	+	.	ID=MALK_03497.t1.e1;Parent=MALK_03497.t1
contig011	AUGUSTUS	exon	635052	635157	.	+	.	ID=MALK_03497.t1.e2;Parent=MALK_03497.t1
contig011	AUGUSTUS	gene	635345	636484	0.64	-	.	ID=MALK_03498;prediction_source=augustus:contig011.g1880.t1
contig011	AUGUSTUS	CDS	635345	636484	0.64	-	0	ID=MALK_03498.t1.c1;Parent=MALK_03498.t1
contig011	AUGUSTUS	mRNA	635345	636484	0.64	-	.	ID=MALK_03498.t1;Parent=MALK_03498
contig011	AUGUSTUS	exon	635345	636484	0.64	-	.	ID=MALK_03498.t1.e1;Parent=MALK_03498.t1
contig011	AUGUSTUS	gene	636547	638473	0.28	-	.	ID=MALK_03499;prediction_source=braker_MRET:g3834.t1
contig011	AUGUSTUS	CDS	637279	638473	0.69	-	0	ID=MALK_03499.t1.c3;Parent=MALK_03499.t1
contig011	AUGUSTUS	CDS	637061	637194	0.69	-	0	ID=MALK_03499.t1.c2;Parent=MALK_03499.t1
contig011	AUGUSTUS	CDS	636547	637008	0.69	-	0	ID=MALK_03499.t1.c1;Parent=MALK_03499.t1
contig011	AUGUSTUS	mRNA	636547	638473	0.28	-	.	ID=MALK_03499.t1;Parent=MALK_03499
contig011	AUGUSTUS	exon	637279	638473	.	-	.	ID=MALK_03499.t1.e3;Parent=MALK_03499.t1
contig011	AUGUSTUS	exon	637061	637194	.	-	.	ID=MALK_03499.t1.e2;Parent=MALK_03499.t1
contig011	AUGUSTUS	exon	636547	637008	.	-	.	ID=MALK_03499.t1.e1;Parent=MALK_03499.t1
contig012	annotation	remark	1	27372	.	+	.	gff-version=3

contig012	AUGUSTUS	gene	192	1658	0.74	+	.	ID=MALK_03500;prediction_source=augustus:contig012.g3732.t1
contig012	AUGUSTUS	CDS	192	1658	0.74	+	0	ID=MALK_03500.t1.c1;Parent=MALK_03500.t1
contig012	AUGUSTUS	mRNA	192	1658	0.74	+	.	ID=MALK_03500.t1;Parent=MALK_03500
contig012	AUGUSTUS	exon	192	1658	0.74	+	.	ID=MALK_03500.t1.e1;Parent=MALK_03500.t1
contig012	AUGUSTUS	gene	1702	2271	0.95	-	.	ID=MALK_03501;prediction_source=braker_MRET:g338.t1
contig012	AUGUSTUS	CDS	1702	2271	0.95	-	0	ID=MALK_03501.t1.c1;Parent=MALK_03501.t1
contig012	AUGUSTUS	mRNA	1702	2271	0.95	-	.	ID=MALK_03501.t1;Parent=MALK_03501
contig012	AUGUSTUS	exon	1702	2271	.	-	.	ID=MALK_03501.t1.e1;Parent=MALK_03501.t1
contig012	AUGUSTUS	gene	2432	3656	0.23	+	.	ID=MALK_03502;prediction_source=braker_MRET:g339.t1
contig012	AUGUSTUS	CDS	2432	3564	0.47	+	0	ID=MALK_03502.t1.c1;Parent=MALK_03502.t1
contig012	AUGUSTUS	CDS	3596	3656	0.47	+	0	ID=MALK_03502.t1.c2;Parent=MALK_03502.t1
contig012	AUGUSTUS	mRNA	2432	3656	0.23	+	.	ID=MALK_03502.t1;Parent=MALK_03502
contig012	AUGUSTUS	exon	2432	3564	.	+	.	ID=MALK_03502.t1.e1;Parent=MALK_03502.t1
contig012	AUGUSTUS	exon	3596	3656	.	+	.	ID=MALK_03502.t1.e2;Parent=MALK_03502.t1
contig012	AUGUSTUS	gene	3717	4688	0.77	-	.	ID=MALK_03503;prediction_source=augustus:contig012.g3734.t1
contig012	AUGUSTUS	CDS	3717	4688	0.77	-	0	ID=MALK_03503.t1.c1;Parent=MALK_03503.t1
contig012	AUGUSTUS	mRNA	3717	4688	0.77	-	.	ID=MALK_03503.t1;Parent=MALK_03503
contig012	AUGUSTUS	exon	3717	4688	0.77	-	.	ID=MALK_03503.t1.e1;Parent=MALK_03503.t1
contig012	AUGUSTUS	gene	5323	8400	0.77	-	.	ID=MALK_03504;prediction_source=augustus:contig012.g3735.t1
contig012	AUGUSTUS	CDS	5323	8400	0.77	-	0	ID=MALK_03504.t1.c1;Parent=MALK_03504.t1
contig012	AUGUSTUS	mRNA	5323	8400	0.77	-	.	ID=MALK_03504.t1;Parent=MALK_03504
contig012	AUGUSTUS	exon	5323	8400	0.77	-	.	ID=MALK_03504.t1.e1;Parent=MALK_03504.t1
contig012	AUGUSTUS	gene	8471	9676	0.57	-	.	ID=MALK_03505;prediction_source=augustus:contig012.g3737.t1
contig012	AUGUSTUS	CDS	8471	9676	0.57	-	0	ID=MALK_03505.t1.c1;Parent=MALK_03505.t1
contig012	AUGUSTUS	mRNA	8471	9676	0.57	-	.	ID=MALK_03505.t1;Parent=MALK_03505
contig012	AUGUSTUS	exon	8471	9676	0.57	-	.	ID=MALK_03505.t1.e1;Parent=MALK_03505.t1
contig012	AUGUSTUS	gene	9828	10895	0.96	+	.	ID=MALK_03506;prediction_source=braker_MRET:g342.t1
contig012	AUGUSTUS	CDS	9828	10895	0.96	+	0	ID=MALK_03506.t1.c1;Parent=MALK_03506.t1
contig012	AUGUSTUS	mRNA	9828	10895	0.96	+	.	ID=MALK_03506.t1;Parent=MALK_03506
contig012	AUGUSTUS	exon	9828	10895	.	+	.	ID=MALK_03506.t1.e1;Parent=MALK_03506.t1
contig012	AUGUSTUS	gene	11027	12451	0.48	+	.	ID=MALK_03507;prediction_source=braker_MRET:g343.t1
contig012	AUGUSTUS	CDS	11027	11359	1	+	0	ID=MALK_03507.t1.c1;Parent=MALK_03507.t1
contig012	AUGUSTUS	CDS	11390	12451	1	+	0	ID=MALK_03507.t1.c2;Parent=MALK_03507.t1
contig012	AUGUSTUS	mRNA	11027	12451	0.48	+	.	ID=MALK_03507.t1;Parent=MALK_03507
contig012	AUGUSTUS	exon	11027	11359	.	+	.	ID=MALK_03507.t1.e1;Parent=MALK_03507.t1
contig012	AUGUSTUS	exon	11390	12451	.	+	.	ID=MALK_03507.t1.e2;Parent=MALK_03507.t1
contig012	AUGUSTUS	gene	12469	13755	0.81	-	.	ID=MALK_03508;prediction_source=augustus:contig012.g3739.t1
contig012	AUGUSTUS	CDS	12469	13755	0.81	-	0	ID=MALK_03508.t1.c1;Parent=MALK_03508.t1
contig012	AUGUSTUS	mRNA	12469	13755	0.81	-	.	ID=MALK_03508.t1;Parent=MALK_03508
contig012	AUGUSTUS	exon	12469	13755	0.81	-	.	ID=MALK_03508.t1.e1;Parent=MALK_03508.t1
contig012	AUGUSTUS	gene	13890	16871	0.46	+	.	ID=MALK_03509;prediction_source=augustus:contig012.g3740.t1
contig012	AUGUSTUS	CDS	13890	16871	0.46	+	0	ID=MALK_03509.t1.c1;Parent=MALK_03509.t1
contig012	AUGUSTUS	mRNA	13890	16871	0.46	+	.	ID=MALK_03509.t1;Parent=MALK_03509

contig012	AUGUSTUS	exon	13890	16871	0.46	+	.	ID=MALK_03509.t1.e1;Parent=MALK_03509.t1
contig012	AUGUSTUS	gene	16868	17482	0.97	-	.	ID=MALK_03510;prediction_source=braker_MRET:g346.t1
contig012	AUGUSTUS	CDS	16868	17482	0.97	-	0	ID=MALK_03510.t1.c1;Parent=MALK_03510.t1
contig012	AUGUSTUS	mRNA	16868	17482	0.97	-	.	ID=MALK_03510.t1;Parent=MALK_03510
contig012	AUGUSTUS	exon	16868	17482	.	-	.	ID=MALK_03510.t1.e1;Parent=MALK_03510.t1
contig012	AUGUSTUS	gene	17518	18240	0.99	+	.	ID=MALK_03511;prediction_source=braker_MRET:g347.t1
contig012	AUGUSTUS	CDS	17518	18240	0.99	+	0	ID=MALK_03511.t1.c1;Parent=MALK_03511.t1
contig012	AUGUSTUS	mRNA	17518	18240	0.99	+	.	ID=MALK_03511.t1;Parent=MALK_03511
contig012	AUGUSTUS	exon	17518	18240	.	+	.	ID=MALK_03511.t1.e1;Parent=MALK_03511.t1
contig012	AUGUSTUS	gene	18446	19611	0.73	-	.	ID=MALK_03512;prediction_source=braker_MRET:g348.t1
contig012	AUGUSTUS	CDS	18751	19611	0.96	-	0	ID=MALK_03512.t1.c2;Parent=MALK_03512.t1
contig012	AUGUSTUS	CDS	18446	18709	0.96	-	0	ID=MALK_03512.t1.c1;Parent=MALK_03512.t1
contig012	AUGUSTUS	mRNA	18446	19611	0.73	-	.	ID=MALK_03512.t1;Parent=MALK_03512
contig012	AUGUSTUS	exon	18751	19611	.	-	.	ID=MALK_03512.t1.e2;Parent=MALK_03512.t1
contig012	AUGUSTUS	exon	18446	18709	.	-	.	ID=MALK_03512.t1.e1;Parent=MALK_03512.t1
contig012	AUGUSTUS	gene	19733	20679	0.97	+	.	ID=MALK_03513;prediction_source=braker_MRET:g349.t1
contig012	AUGUSTUS	CDS	19733	20551	0.97	+	0	ID=MALK_03513.t1.c1;Parent=MALK_03513.t1
contig012	AUGUSTUS	CDS	20617	20679	0.97	+	0	ID=MALK_03513.t1.c2;Parent=MALK_03513.t1
contig012	AUGUSTUS	mRNA	19733	20679	0.97	+	.	ID=MALK_03513.t1;Parent=MALK_03513
contig012	AUGUSTUS	exon	19733	20551	.	+	.	ID=MALK_03513.t1.e1;Parent=MALK_03513.t1
contig012	AUGUSTUS	exon	20617	20679	.	+	.	ID=MALK_03513.t1.e2;Parent=MALK_03513.t1
contig012	AUGUSTUS	gene	20956	22803	0.67	-	.	ID=MALK_03514;prediction_source=braker_MRET:g350.t1
contig012	AUGUSTUS	CDS	20956	22803	0.67	-	0	ID=MALK_03514.t1.c1;Parent=MALK_03514.t1
contig012	AUGUSTUS	mRNA	20956	22803	0.67	-	.	ID=MALK_03514.t1;Parent=MALK_03514
contig012	AUGUSTUS	exon	20956	22803	.	-	.	ID=MALK_03514.t1.e1;Parent=MALK_03514.t1
contig012	AUGUSTUS	gene	22838	24565	0.52	+	.	ID=MALK_03515;prediction_source=braker_MRET:g351.t1
contig012	AUGUSTUS	CDS	22838	24565	0.52	+	0	ID=MALK_03515.t1.c1;Parent=MALK_03515.t1
contig012	AUGUSTUS	mRNA	22838	24565	0.52	+	.	ID=MALK_03515.t1;Parent=MALK_03515
contig012	AUGUSTUS	exon	22838	24565	.	+	.	ID=MALK_03515.t1.e1;Parent=MALK_03515.t1
contig012	AUGUSTUS	gene	24619	26697	0.97	+	.	ID=MALK_03516;prediction_source=braker_MRET:g352.t1
contig012	AUGUSTUS	CDS	24619	26697	0.97	+	0	ID=MALK_03516.t1.c1;Parent=MALK_03516.t1
contig012	AUGUSTUS	mRNA	24619	26697	0.97	+	.	ID=MALK_03516.t1;Parent=MALK_03516
contig012	AUGUSTUS	exon	24619	26697	.	+	.	ID=MALK_03516.t1.e1;Parent=MALK_03516.t1
contig012	AUGUSTUS	gene	27040	27318	0.76	+	.	ID=MALK_03517;prediction_source=augustus:contig012.g3743.t1
contig012	AUGUSTUS	CDS	27040	27318	0.76	+	0	ID=MALK_03517.t1.c1;Parent=MALK_03517.t1
contig012	AUGUSTUS	mRNA	27040	27318	0.76	+	.	ID=MALK_03517.t1;Parent=MALK_03517
contig012	AUGUSTUS	exon	27040	27318	0.76	+	.	ID=MALK_03517.t1.e1;Parent=MALK_03517.t1
contig013	annotation	remark	1	263122	.	+	.	gff-version=3
contig013	maker	gene	587	1714	.	+	.	ID=MALK_03518;prediction_source=maker_MRET:augustus_masked-contig013-processed-gene-0.52-mRNA-1
contig013	maker	CDS	587	1714	.	+	0	ID=MALK_03518.t1.c1;Parent=MALK_03518.t1
contig013	maker	mRNA	587	1714	.	+	.	ID=MALK_03518.t1;Parent=MALK_03518
contig013	maker	exon	587	1714	.	+	.	ID=MALK_03518.t1.e1;Parent=MALK_03518.t1
contig013	maker	gene	1930	3615	.	-	.	ID=MALK_03519;prediction_source=maker_MRET:augustus_masked-contig013-processed-gene-0.71-mRNA-1

contig013	maker	CDS	1930	3615	.	-	0	ID=MALK_03519.t1.c1;Parent=MALK_03519.t1
contig013	maker	mRNA	1930	3615	.	-	.	ID=MALK_03519.t1;Parent=MALK_03519
contig013	maker	exon	1930	3615	.	-	.	ID=MALK_03519.t1.e1;Parent=MALK_03519.t1
contig013	maker	gene	4868	5770	.	+	.	ID=MALK_03520;prediction_source=maker_MRET:augustus_masked-contig013-processed-gene-0.53-mRNA-1
contig013	maker	CDS	4868	5770	.	+	0	ID=MALK_03520.t1.c1;Parent=MALK_03520.t1
contig013	maker	mRNA	4868	5770	.	+	.	ID=MALK_03520.t1;Parent=MALK_03520
contig013	maker	exon	4868	5770	.	+	.	ID=MALK_03520.t1.e1;Parent=MALK_03520.t1
contig013	AUGUSTUS	gene	5786	6715	0.42	-	.	ID=MALK_03521;prediction_source=braker_MRET:g3838.t1
contig013	AUGUSTUS	CDS	5786	6715	0.42	-	0	ID=MALK_03521.t1.c1;Parent=MALK_03521.t1
contig013	AUGUSTUS	mRNA	5786	6715	0.42	-	.	ID=MALK_03521.t1;Parent=MALK_03521
contig013	AUGUSTUS	exon	5786	6715	.	-	.	ID=MALK_03521.t1.e1;Parent=MALK_03521.t1
contig013	AUGUSTUS	gene	8812	10074	1	-	.	ID=MALK_03522;prediction_source=augustus:contig013.g2956.t1
contig013	AUGUSTUS	CDS	8812	10074	1	-	0	ID=MALK_03522.t1.c1;Parent=MALK_03522.t1
contig013	AUGUSTUS	mRNA	8812	10074	1	-	.	ID=MALK_03522.t1;Parent=MALK_03522
contig013	AUGUSTUS	exon	8812	10074	1	-	.	ID=MALK_03522.t1.e1;Parent=MALK_03522.t1
contig013	maker	gene	10209	11708	.	-	.	ID=MALK_03523;prediction_source=maker_MRET:augustus_masked-contig013-processed-gene-0.74-mRNA-1
contig013	maker	CDS	10209	11708	.	-	0	ID=MALK_03523.t1.c1;Parent=MALK_03523.t1
contig013	maker	mRNA	10209	11708	.	-	.	ID=MALK_03523.t1;Parent=MALK_03523
contig013	maker	exon	10209	11708	.	-	.	ID=MALK_03523.t1.e1;Parent=MALK_03523.t1
contig013	maker	gene	11771	12472	.	-	.	ID=MALK_03524;prediction_source=maker_MRET:augustus_masked-contig013-processed-gene-0.75-mRNA-1
contig013	maker	CDS	11771	12472	.	-	0	ID=MALK_03524.t1.c1;Parent=MALK_03524.t1
contig013	maker	mRNA	11771	12472	.	-	.	ID=MALK_03524.t1;Parent=MALK_03524
contig013	maker	exon	11771	12472	.	-	.	ID=MALK_03524.t1.e1;Parent=MALK_03524.t1
contig013	maker	gene	12524	14497	.	-	.	ID=MALK_03525;prediction_source=maker_MRET:augustus_masked-contig013-processed-gene-0.76-mRNA-1
contig013	maker	CDS	12524	14497	.	-	0	ID=MALK_03525.t1.c1;Parent=MALK_03525.t1
contig013	maker	mRNA	12524	14497	.	-	.	ID=MALK_03525.t1;Parent=MALK_03525
contig013	maker	exon	12524	14497	.	-	.	ID=MALK_03525.t1.e1;Parent=MALK_03525.t1
contig013	maker	gene	14910	16517	.	-	.	ID=MALK_03526;prediction_source=maker_MRET:augustus_masked-contig013-processed-gene-0.77-mRNA-1
contig013	maker	CDS	14910	16517	.	-	0	ID=MALK_03526.t1.c1;Parent=MALK_03526.t1
contig013	maker	mRNA	14910	16517	.	-	.	ID=MALK_03526.t1;Parent=MALK_03526
contig013	maker	exon	14910	16517	.	-	.	ID=MALK_03526.t1.e1;Parent=MALK_03526.t1
contig013	AUGUSTUS	gene	16620	18059	0.96	+	.	ID=MALK_03527;prediction_source=augustus:contig013.g2964.t1
contig013	AUGUSTUS	CDS	16620	18059	0.96	+	0	ID=MALK_03527.t1.c1;Parent=MALK_03527.t1
contig013	AUGUSTUS	mRNA	16620	18059	0.96	+	.	ID=MALK_03527.t1;Parent=MALK_03527
contig013	AUGUSTUS	exon	16620	18059	0.96	+	.	ID=MALK_03527.t1.e1;Parent=MALK_03527.t1
contig013	AUGUSTUS	gene	18076	18609	0.47	-	.	ID=MALK_03528;prediction_source=braker_MRET:g3844.t1
contig013	AUGUSTUS	CDS	18076	18609	0.47	-	0	ID=MALK_03528.t1.c1;Parent=MALK_03528.t1
contig013	AUGUSTUS	mRNA	18076	18609	0.47	-	.	ID=MALK_03528.t1;Parent=MALK_03528
contig013	AUGUSTUS	exon	18076	18609	.	-	.	ID=MALK_03528.t1.e1;Parent=MALK_03528.t1
contig013	AUGUSTUS	gene	18728	19510	0.59	+	.	ID=MALK_03529;prediction_source=augustus:contig013.g2967.t1
contig013	AUGUSTUS	CDS	18728	19510	0.59	+	0	ID=MALK_03529.t1.c1;Parent=MALK_03529.t1
contig013	AUGUSTUS	mRNA	18728	19510	0.59	+	.	ID=MALK_03529.t1;Parent=MALK_03529
contig013	AUGUSTUS	exon	18728	19510	0.59	+	.	ID=MALK_03529.t1.e1;Parent=MALK_03529.t1

contig013	maker	gene	19609	19962	.	-	.	ID=MALK_03530;prediction_source=maker_MRET:augustus_masked-contig013-processed-gene-0.79-mRNA-1
contig013	maker	CDS	19609	19962	.	-	0	ID=MALK_03530.t1.c1;Parent=MALK_03530.t1
contig013	maker	mRNA	19609	19962	.	-	.	ID=MALK_03530.t1;Parent=MALK_03530
contig013	maker	exon	19609	19962	.	-	.	ID=MALK_03530.t1.e1;Parent=MALK_03530.t1
contig013	AUGUSTUS	gene	20089	21078	0.96	+	.	ID=MALK_03531;prediction_source=augustus:contig013.g2970.t1
contig013	AUGUSTUS	CDS	20089	21078	0.96	+	0	ID=MALK_03531.t1.c1;Parent=MALK_03531.t1
contig013	AUGUSTUS	mRNA	20089	21078	0.96	+	.	ID=MALK_03531.t1;Parent=MALK_03531
contig013	AUGUSTUS	exon	20089	21078	0.96	+	.	ID=MALK_03531.t1.e1;Parent=MALK_03531.t1
contig013	AUGUSTUS	gene	21307	23559	0.92	+	.	ID=MALK_03532;prediction_source=braker_MRET:g3848.t1
contig013	AUGUSTUS	CDS	21307	23559	0.92	+	0	ID=MALK_03532.t1.c1;Parent=MALK_03532.t1
contig013	AUGUSTUS	mRNA	21307	23559	0.92	+	.	ID=MALK_03532.t1;Parent=MALK_03532
contig013	AUGUSTUS	exon	21307	23559	.	+	.	ID=MALK_03532.t1.e1;Parent=MALK_03532.t1
contig013	AUGUSTUS	gene	23625	27454	0.42	+	.	ID=MALK_03533;prediction_source=braker_MRET:g3849.t1
contig013	AUGUSTUS	CDS	23625	27361	0.64	+	0	ID=MALK_03533.t1.c1;Parent=MALK_03533.t1
contig013	AUGUSTUS	CDS	27400	27454	0.64	+	0	ID=MALK_03533.t1.c2;Parent=MALK_03533.t1
contig013	AUGUSTUS	mRNA	23625	27454	0.42	+	.	ID=MALK_03533.t1;Parent=MALK_03533
contig013	AUGUSTUS	exon	23625	27361	.	+	.	ID=MALK_03533.t1.e1;Parent=MALK_03533.t1
contig013	AUGUSTUS	exon	27400	27454	.	+	.	ID=MALK_03533.t1.e2;Parent=MALK_03533.t1
contig013	AUGUSTUS	gene	27522	29751	0.71	-	.	ID=MALK_03534;prediction_source=braker_MRET:g3850.t1
contig013	AUGUSTUS	CDS	28378	29751	0.85	-	0	ID=MALK_03534.t1.c2;Parent=MALK_03534.t1
contig013	AUGUSTUS	CDS	27522	28331	0.85	-	0	ID=MALK_03534.t1.c1;Parent=MALK_03534.t1
contig013	AUGUSTUS	mRNA	27522	29751	0.71	-	.	ID=MALK_03534.t1;Parent=MALK_03534
contig013	AUGUSTUS	exon	28378	29751	.	-	.	ID=MALK_03534.t1.e2;Parent=MALK_03534.t1
contig013	AUGUSTUS	exon	27522	28331	.	-	.	ID=MALK_03534.t1.e1;Parent=MALK_03534.t1
contig013	maker	gene	29750	31063	.	+	.	ID=MALK_03535;prediction_source=maker_MRET:augustus_masked-contig013-processed-gene-0.58-mRNA-1
contig013	maker	CDS	29750	31063	.	+	0	ID=MALK_03535.t1.c1;Parent=MALK_03535.t1
contig013	maker	mRNA	29750	31063	.	+	.	ID=MALK_03535.t1;Parent=MALK_03535
contig013	maker	exon	29750	31063	.	+	.	ID=MALK_03535.t1.e1;Parent=MALK_03535.t1
contig013	AUGUSTUS	gene	31060	32898	0.46	-	.	ID=MALK_03536;prediction_source=augustus:contig013.g2975.t1
contig013	AUGUSTUS	CDS	31060	32898	0.46	-	0	ID=MALK_03536.t1.c1;Parent=MALK_03536.t1
contig013	AUGUSTUS	mRNA	31060	32898	0.46	-	.	ID=MALK_03536.t1;Parent=MALK_03536
contig013	AUGUSTUS	exon	31060	32898	0.46	-	.	ID=MALK_03536.t1.e1;Parent=MALK_03536.t1
contig013	maker	gene	33135	36251	.	-	.	ID=MALK_03537;prediction_source=maker_MRET:augustus_masked-contig013-processed-gene-0.82-mRNA-1
contig013	maker	CDS	33135	36251	.	-	0	ID=MALK_03537.t1.c1;Parent=MALK_03537.t1
contig013	maker	mRNA	33135	36251	.	-	.	ID=MALK_03537.t1;Parent=MALK_03537
contig013	maker	exon	33135	36251	.	-	.	ID=MALK_03537.t1.e1;Parent=MALK_03537.t1
contig013	maker	gene	36467	38398	.	+	.	ID=MALK_03538;prediction_source=maker_MRET:augustus_masked-contig013-processed-gene-0.59-mRNA-1
contig013	maker	CDS	36467	38398	.	+	0	ID=MALK_03538.t1.c1;Parent=MALK_03538.t1
contig013	maker	mRNA	36467	38398	.	+	.	ID=MALK_03538.t1;Parent=MALK_03538
contig013	maker	exon	36467	38398	.	+	.	ID=MALK_03538.t1.e1;Parent=MALK_03538.t1
contig013	AUGUSTUS	gene	39358	40743	0.75	+	.	ID=MALK_03539;prediction_source=augustus:contig013.g2980.t1
contig013	AUGUSTUS	CDS	39358	40743	0.75	+	0	ID=MALK_03539.t1.c1;Parent=MALK_03539.t1
contig013	AUGUSTUS	mRNA	39358	40743	0.75	+	.	ID=MALK_03539.t1;Parent=MALK_03539

contig013	AUGUSTUS	exon	39358	40743	0.75	+	.	ID=MALK_03539.t1.e1;Parent=MALK_03539.t1
contig013	maker	gene	40822	44331	.	+	.	ID=MALK_03540;prediction_source=maker_MRET:augustus_masked-contig013-processed-gene-0.61-mRNA-1
contig013	maker	CDS	40822	44331	.	+	0	ID=MALK_03540.t1.c1;Parent=MALK_03540.t1
contig013	maker	mRNA	40822	44331	.	+	.	ID=MALK_03540.t1;Parent=MALK_03540
contig013	maker	exon	40822	44331	.	+	.	ID=MALK_03540.t1.e1;Parent=MALK_03540.t1
contig013	maker	gene	44335	45575	.	-	.	ID=MALK_03541;prediction_source=maker_MRET:augustus_masked-contig013-processed-gene-0.83-mRNA-1
contig013	maker	CDS	45415	45575	.	-	0	ID=MALK_03541.t1.c1;Parent=MALK_03541.t1
contig013	maker	CDS	44335	45310	.	-	0	ID=MALK_03541.t1.c2;Parent=MALK_03541.t1
contig013	maker	mRNA	44335	45575	.	-	.	ID=MALK_03541.t1;Parent=MALK_03541
contig013	maker	exon	45415	45575	.	-	.	ID=MALK_03541.t1.e1;Parent=MALK_03541.t1
contig013	maker	exon	44335	45310	.	-	.	ID=MALK_03541.t1.e2;Parent=MALK_03541.t1
contig013	AUGUSTUS	gene	46370	46940	0.5	-	.	ID=MALK_03542;prediction_source=braker_MRET:g3858.t1
contig013	AUGUSTUS	CDS	46578	46940	0.58	-	0	ID=MALK_03542.t1.c2;Parent=MALK_03542.t1
contig013	AUGUSTUS	CDS	46370	46537	0.58	-	0	ID=MALK_03542.t1.c1;Parent=MALK_03542.t1
contig013	AUGUSTUS	mRNA	46370	46940	0.5	-	.	ID=MALK_03542.t1;Parent=MALK_03542
contig013	AUGUSTUS	exon	46578	46940	.	-	.	ID=MALK_03542.t1.e2;Parent=MALK_03542.t1
contig013	AUGUSTUS	exon	46370	46537	.	-	.	ID=MALK_03542.t1.e1;Parent=MALK_03542.t1
contig013	AUGUSTUS	gene	47437	48237	0.99	+	.	ID=MALK_03543;prediction_source=braker_MRET:g3859.t1
contig013	AUGUSTUS	CDS	47437	48237	0.99	+	0	ID=MALK_03543.t1.c1;Parent=MALK_03543.t1
contig013	AUGUSTUS	mRNA	47437	48237	0.99	+	.	ID=MALK_03543.t1;Parent=MALK_03543
contig013	AUGUSTUS	exon	47437	48237	.	+	.	ID=MALK_03543.t1.e1;Parent=MALK_03543.t1
contig013	AUGUSTUS	gene	48241	50475	0.97	-	.	ID=MALK_03544;prediction_source=augustus:contig013.g2984.t1
contig013	AUGUSTUS	CDS	48241	50475	0.97	-	0	ID=MALK_03544.t1.c1;Parent=MALK_03544.t1
contig013	AUGUSTUS	mRNA	48241	50475	0.97	-	.	ID=MALK_03544.t1;Parent=MALK_03544
contig013	AUGUSTUS	exon	48241	50475	0.97	-	.	ID=MALK_03544.t1.e1;Parent=MALK_03544.t1
contig013	maker	gene	50825	51859	.	+	.	ID=MALK_03545;prediction_source=maker_MRET:augustus_masked-contig013-processed-gene-0.62-mRNA-1
contig013	maker	CDS	50825	51859	.	+	0	ID=MALK_03545.t1.c1;Parent=MALK_03545.t1
contig013	maker	mRNA	50825	51859	.	+	.	ID=MALK_03545.t1;Parent=MALK_03545
contig013	maker	exon	50825	51859	.	+	.	ID=MALK_03545.t1.e1;Parent=MALK_03545.t1
contig013	AUGUSTUS	gene	52135	53648	0.41	+	.	ID=MALK_03546;prediction_source=braker_MRET:g3862.t1
contig013	AUGUSTUS	CDS	52135	52233	0.41	+	0	ID=MALK_03546.t1.c1;Parent=MALK_03546.t1
contig013	AUGUSTUS	CDS	52281	53648	0.41	+	0	ID=MALK_03546.t1.c2;Parent=MALK_03546.t1
contig013	AUGUSTUS	mRNA	52135	53648	0.41	+	.	ID=MALK_03546.t1;Parent=MALK_03546
contig013	AUGUSTUS	exon	52135	52233	.	+	.	ID=MALK_03546.t1.e1;Parent=MALK_03546.t1
contig013	AUGUSTUS	exon	52281	53648	.	+	.	ID=MALK_03546.t1.e2;Parent=MALK_03546.t1
contig013	AUGUSTUS	gene	53645	54838	0.65	-	.	ID=MALK_03547;prediction_source=augustus:contig013.g2986.t1
contig013	AUGUSTUS	CDS	53645	54838	0.65	-	0	ID=MALK_03547.t1.c1;Parent=MALK_03547.t1
contig013	AUGUSTUS	mRNA	53645	54838	0.65	-	.	ID=MALK_03547.t1;Parent=MALK_03547
contig013	AUGUSTUS	exon	53645	54838	0.65	-	.	ID=MALK_03547.t1.e1;Parent=MALK_03547.t1
contig013	AUGUSTUS	gene	55032	55410	0.98	-	.	ID=MALK_03548;prediction_source=braker_MRET:g3864.t1
contig013	AUGUSTUS	CDS	55089	55410	0.98	-	0	ID=MALK_03548.t1.c2;Parent=MALK_03548.t1
contig013	AUGUSTUS	CDS	55032	55057	0.98	-	0	ID=MALK_03548.t1.c1;Parent=MALK_03548.t1
contig013	AUGUSTUS	mRNA	55032	55410	0.98	-	.	ID=MALK_03548.t1;Parent=MALK_03548

contig013	AUGUSTUS	exon	55089	55410	.	-	.	ID=MALK_03548.t1.e2;Parent=MALK_03548.t1
contig013	AUGUSTUS	exon	55032	55057	.	-	.	ID=MALK_03548.t1.e1;Parent=MALK_03548.t1
contig013	AUGUSTUS	gene	55648	56448	0.79	-	.	ID=MALK_03549;prediction_source=braker_MRET:g3865.t1
contig013	AUGUSTUS	CDS	56355	56448	0.79	-	0	ID=MALK_03549.t1.c2;Parent=MALK_03549.t1
contig013	AUGUSTUS	CDS	55648	56309	0.79	-	0	ID=MALK_03549.t1.c1;Parent=MALK_03549.t1
contig013	AUGUSTUS	mRNA	55648	56448	0.79	-	.	ID=MALK_03549.t1;Parent=MALK_03549
contig013	AUGUSTUS	exon	56355	56448	.	-	.	ID=MALK_03549.t1.e2;Parent=MALK_03549.t1
contig013	AUGUSTUS	exon	55648	56309	.	-	.	ID=MALK_03549.t1.e1;Parent=MALK_03549.t1
contig013	AUGUSTUS	gene	56715	57590	1	+	.	ID=MALK_03550;prediction_source=augustus:contig013.g2989.t1
contig013	AUGUSTUS	CDS	56715	57590	1	+	0	ID=MALK_03550.t1.c1;Parent=MALK_03550.t1
contig013	AUGUSTUS	mRNA	56715	57590	1	+	.	ID=MALK_03550.t1;Parent=MALK_03550
contig013	AUGUSTUS	exon	56715	57590	1	+	.	ID=MALK_03550.t1.e1;Parent=MALK_03550.t1
contig013	AUGUSTUS	gene	57679	58452	0.77	+	.	ID=MALK_03551;prediction_source=augustus:contig013.g2991.t1
contig013	AUGUSTUS	CDS	57679	58452	0.77	+	0	ID=MALK_03551.t1.c1;Parent=MALK_03551.t1
contig013	AUGUSTUS	mRNA	57679	58452	0.77	+	.	ID=MALK_03551.t1;Parent=MALK_03551
contig013	AUGUSTUS	exon	57679	58452	0.77	+	.	ID=MALK_03551.t1.e1;Parent=MALK_03551.t1
contig013	AUGUSTUS	gene	58421	59821	0.92	-	.	ID=MALK_03552;prediction_source=augustus:contig013.g2992.t1
contig013	AUGUSTUS	CDS	58421	59821	0.92	-	0	ID=MALK_03552.t1.c1;Parent=MALK_03552.t1
contig013	AUGUSTUS	mRNA	58421	59821	0.92	-	.	ID=MALK_03552.t1;Parent=MALK_03552
contig013	AUGUSTUS	exon	58421	59821	0.92	-	.	ID=MALK_03552.t1.e1;Parent=MALK_03552.t1
contig013	AUGUSTUS	gene	59935	63288	1	+	.	ID=MALK_03553;prediction_source=augustus:contig013.g2993.t1
contig013	AUGUSTUS	CDS	59935	63288	1	+	0	ID=MALK_03553.t1.c1;Parent=MALK_03553.t1
contig013	AUGUSTUS	mRNA	59935	63288	1	+	.	ID=MALK_03553.t1;Parent=MALK_03553
contig013	AUGUSTUS	exon	59935	63288	1	+	.	ID=MALK_03553.t1.e1;Parent=MALK_03553.t1
contig013	AUGUSTUS	gene	63290	65839	0.99	-	.	ID=MALK_03554;prediction_source=augustus:contig013.g2994.t1
contig013	AUGUSTUS	CDS	63290	65839	0.99	-	0	ID=MALK_03554.t1.c1;Parent=MALK_03554.t1
contig013	AUGUSTUS	mRNA	63290	65839	0.99	-	.	ID=MALK_03554.t1;Parent=MALK_03554
contig013	AUGUSTUS	exon	63290	65839	0.99	-	.	ID=MALK_03554.t1.e1;Parent=MALK_03554.t1
contig013	maker	gene	66170	66664	.	-	.	ID=MALK_03555;prediction_source=maker_MRET:augustus_masked-contig013-processed-gene-0.90-mRNA-1
contig013	maker	CDS	66170	66664	.	-	0	ID=MALK_03555.t1.c1;Parent=MALK_03555.t1
contig013	maker	mRNA	66170	66664	.	-	.	ID=MALK_03555.t1;Parent=MALK_03555
contig013	maker	exon	66170	66664	.	-	.	ID=MALK_03555.t1.e1;Parent=MALK_03555.t1
contig013	AUGUSTUS	gene	66721	70360	0.61	-	.	ID=MALK_03556;prediction_source=braker_MRET:g3870.t1
contig013	AUGUSTUS	CDS	70327	70360	0.99	-	0	ID=MALK_03556.t1.c5;Parent=MALK_03556.t1
contig013	AUGUSTUS	CDS	70182	70297	0.99	-	0	ID=MALK_03556.t1.c4;Parent=MALK_03556.t1
contig013	AUGUSTUS	CDS	70081	70154	0.99	-	0	ID=MALK_03556.t1.c3;Parent=MALK_03556.t1
contig013	AUGUSTUS	CDS	69789	70046	0.99	-	0	ID=MALK_03556.t1.c2;Parent=MALK_03556.t1
contig013	AUGUSTUS	CDS	66721	69760	0.99	-	0	ID=MALK_03556.t1.c1;Parent=MALK_03556.t1
contig013	AUGUSTUS	mRNA	66721	70360	0.61	-	.	ID=MALK_03556.t1;Parent=MALK_03556
contig013	AUGUSTUS	exon	70327	70360	.	-	.	ID=MALK_03556.t1.e5;Parent=MALK_03556.t1
contig013	AUGUSTUS	exon	70182	70297	.	-	.	ID=MALK_03556.t1.e4;Parent=MALK_03556.t1
contig013	AUGUSTUS	exon	70081	70154	.	-	.	ID=MALK_03556.t1.e3;Parent=MALK_03556.t1
contig013	AUGUSTUS	exon	69789	70046	.	-	.	ID=MALK_03556.t1.e2;Parent=MALK_03556.t1

contig013	AUGUSTUS	exon	66721	69760	.	-	.	ID=MALK_03556.t1.e1;Parent=MALK_03556.t1
contig013	AUGUSTUS	gene	70468	71631	0.98	+	.	ID=MALK_03557;prediction_source=braker_MRET:g3871.t1
contig013	AUGUSTUS	CDS	70468	71631	0.98	+	0	ID=MALK_03557.t1.c1;Parent=MALK_03557.t1
contig013	AUGUSTUS	mRNA	70468	71631	0.98	+	.	ID=MALK_03557.t1;Parent=MALK_03557
contig013	AUGUSTUS	exon	70468	71631	.	+	.	ID=MALK_03557.t1.e1;Parent=MALK_03557.t1
contig013	AUGUSTUS	gene	71662	72090	0.32	+	.	ID=MALK_03558;prediction_source=braker_MRET:g3872.t1
contig013	AUGUSTUS	CDS	71662	72090	0.32	+	0	ID=MALK_03558.t1.c1;Parent=MALK_03558.t1
contig013	AUGUSTUS	mRNA	71662	72090	0.32	+	.	ID=MALK_03558.t1;Parent=MALK_03558
contig013	AUGUSTUS	exon	71662	72090	.	+	.	ID=MALK_03558.t1.e1;Parent=MALK_03558.t1
contig013	AUGUSTUS	gene	72113	73210	0.75	-	.	ID=MALK_03559;prediction_source=braker_MRET:g3873.t1
contig013	AUGUSTUS	CDS	72113	73210	0.75	-	0	ID=MALK_03559.t1.c1;Parent=MALK_03559.t1
contig013	AUGUSTUS	mRNA	72113	73210	0.75	-	.	ID=MALK_03559.t1;Parent=MALK_03559
contig013	AUGUSTUS	exon	72113	73210	.	-	.	ID=MALK_03559.t1.e1;Parent=MALK_03559.t1
contig013	AUGUSTUS	gene	73271	74377	0.67	+	.	ID=MALK_03560;prediction_source=braker_MRET:g3874.t1
contig013	AUGUSTUS	CDS	73271	73724	0.81	+	0	ID=MALK_03560.t1.c1;Parent=MALK_03560.t1
contig013	AUGUSTUS	CDS	73752	74377	0.81	+	0	ID=MALK_03560.t1.c2;Parent=MALK_03560.t1
contig013	AUGUSTUS	mRNA	73271	74377	0.67	+	.	ID=MALK_03560.t1;Parent=MALK_03560
contig013	AUGUSTUS	exon	73271	73724	.	+	.	ID=MALK_03560.t1.e1;Parent=MALK_03560.t1
contig013	AUGUSTUS	exon	73752	74377	.	+	.	ID=MALK_03560.t1.e2;Parent=MALK_03560.t1
contig013	maker	gene	74408	76621	.	-	.	ID=MALK_03561;prediction_source=maker_MRET:augustus_masked-contig013-processed-gene-0.91-mRNA-1
contig013	maker	CDS	74408	76621	.	-	0	ID=MALK_03561.t1.c1;Parent=MALK_03561.t1
contig013	maker	mRNA	74408	76621	.	-	.	ID=MALK_03561.t1;Parent=MALK_03561
contig013	maker	exon	74408	76621	.	-	.	ID=MALK_03561.t1.e1;Parent=MALK_03561.t1
contig013	maker	gene	76666	79374	.	+	.	ID=MALK_03562;prediction_source=maker_MRET:augustus_masked-contig013-processed-gene-0.67-mRNA-1
contig013	maker	CDS	76666	79374	.	+	0	ID=MALK_03562.t1.c1;Parent=MALK_03562.t1
contig013	maker	mRNA	76666	79374	.	+	.	ID=MALK_03562.t1;Parent=MALK_03562
contig013	maker	exon	76666	79374	.	+	.	ID=MALK_03562.t1.e1;Parent=MALK_03562.t1
contig013	AUGUSTUS	gene	80196	80771	0.34	-	.	ID=MALK_03563;prediction_source=braker_MRET:g3877.t1
contig013	AUGUSTUS	CDS	80697	80771	0.73	-	0	ID=MALK_03563.t1.c3;Parent=MALK_03563.t1
contig013	AUGUSTUS	CDS	80416	80660	0.73	-	0	ID=MALK_03563.t1.c2;Parent=MALK_03563.t1
contig013	AUGUSTUS	CDS	80196	80343	0.73	-	0	ID=MALK_03563.t1.c1;Parent=MALK_03563.t1
contig013	AUGUSTUS	mRNA	80196	80771	0.34	-	.	ID=MALK_03563.t1;Parent=MALK_03563
contig013	AUGUSTUS	exon	80697	80771	.	-	.	ID=MALK_03563.t1.e3;Parent=MALK_03563.t1
contig013	AUGUSTUS	exon	80416	80660	.	-	.	ID=MALK_03563.t1.e2;Parent=MALK_03563.t1
contig013	AUGUSTUS	exon	80196	80343	.	-	.	ID=MALK_03563.t1.e1;Parent=MALK_03563.t1
contig013	maker	gene	80869	83244	.	-	.	ID=MALK_03564;prediction_source=maker_MRET:augustus_masked-contig013-processed-gene-0.93-mRNA-1
contig013	maker	CDS	80869	83244	.	-	0	ID=MALK_03564.t1.c1;Parent=MALK_03564.t1
contig013	maker	mRNA	80869	83244	.	-	.	ID=MALK_03564.t1;Parent=MALK_03564
contig013	maker	exon	80869	83244	.	-	.	ID=MALK_03564.t1.e1;Parent=MALK_03564.t1
contig013	AUGUSTUS	gene	83284	84405	0.99	+	.	ID=MALK_03565;prediction_source=braker_MRET:g3879.t1
contig013	AUGUSTUS	CDS	83284	84405	0.99	+	0	ID=MALK_03565.t1.c1;Parent=MALK_03565.t1
contig013	AUGUSTUS	mRNA	83284	84405	0.99	+	.	ID=MALK_03565.t1;Parent=MALK_03565
contig013	AUGUSTUS	exon	83284	84405	.	+	.	ID=MALK_03565.t1.e1;Parent=MALK_03565.t1

contig013	AUGUSTUS	gene	84410	86033	0.73	-	.	ID=MALK_03566;prediction_source=braker_MRET:g3880.t1
contig013	AUGUSTUS	CDS	84709	86033	0.79	-	0	ID=MALK_03566.t1.c2;Parent=MALK_03566.t1
contig013	AUGUSTUS	CDS	84410	84608	0.79	-	0	ID=MALK_03566.t1.c1;Parent=MALK_03566.t1
contig013	AUGUSTUS	mRNA	84410	86033	0.73	-	.	ID=MALK_03566.t1;Parent=MALK_03566
contig013	AUGUSTUS	exon	84709	86033	.	-	.	ID=MALK_03566.t1.e2;Parent=MALK_03566.t1
contig013	AUGUSTUS	exon	84410	84608	.	-	.	ID=MALK_03566.t1.e1;Parent=MALK_03566.t1
contig013	maker	gene	86156	87589	.	+	.	ID=MALK_03567;prediction_source=maker_MRET:augustus_masked-contig013-processed-gene-0.69-mRNA-1
contig013	maker	CDS	86156	87589	.	+	0	ID=MALK_03567.t1.c1;Parent=MALK_03567.t1
contig013	maker	mRNA	86156	87589	.	+	.	ID=MALK_03567.t1;Parent=MALK_03567
contig013	maker	exon	86156	87589	.	+	.	ID=MALK_03567.t1.e1;Parent=MALK_03567.t1
contig013	AUGUSTUS	gene	87615	88940	0.98	-	.	ID=MALK_03568;prediction_source=braker_MRET:g3882.t1
contig013	AUGUSTUS	CDS	87615	88940	0.98	-	0	ID=MALK_03568.t1.c1;Parent=MALK_03568.t1
contig013	AUGUSTUS	mRNA	87615	88940	0.98	-	.	ID=MALK_03568.t1;Parent=MALK_03568
contig013	AUGUSTUS	exon	87615	88940	.	-	.	ID=MALK_03568.t1.e1;Parent=MALK_03568.t1
contig013	AUGUSTUS	gene	89040	90251	1	+	.	ID=MALK_03569;prediction_source=braker_MRET:g3883.t1
contig013	AUGUSTUS	CDS	89040	90251	1	+	0	ID=MALK_03569.t1.c1;Parent=MALK_03569.t1
contig013	AUGUSTUS	mRNA	89040	90251	1	+	.	ID=MALK_03569.t1;Parent=MALK_03569
contig013	AUGUSTUS	exon	89040	90251	.	+	.	ID=MALK_03569.t1.e1;Parent=MALK_03569.t1
contig013	AUGUSTUS	gene	90354	90951	0.32	-	.	ID=MALK_03570;prediction_source=braker_MRET:g3884.t1
contig013	AUGUSTUS	CDS	90458	90951	0.32	-	0	ID=MALK_03570.t1.c2;Parent=MALK_03570.t1
contig013	AUGUSTUS	CDS	90354	90429	0.32	-	0	ID=MALK_03570.t1.c1;Parent=MALK_03570.t1
contig013	AUGUSTUS	mRNA	90354	90951	0.32	-	.	ID=MALK_03570.t1;Parent=MALK_03570
contig013	AUGUSTUS	exon	90458	90951	.	-	.	ID=MALK_03570.t1.e2;Parent=MALK_03570.t1
contig013	AUGUSTUS	exon	90354	90429	.	-	.	ID=MALK_03570.t1.e1;Parent=MALK_03570.t1
contig013	maker	gene	91159	93165	.	-	.	ID=MALK_03571;prediction_source=maker_MRET:augustus_masked-contig013-processed-gene-0.94-mRNA-1
contig013	maker	CDS	91159	93165	.	-	0	ID=MALK_03571.t1.c1;Parent=MALK_03571.t1
contig013	maker	mRNA	91159	93165	.	-	.	ID=MALK_03571.t1;Parent=MALK_03571
contig013	maker	exon	91159	93165	.	-	.	ID=MALK_03571.t1.e1;Parent=MALK_03571.t1
contig013	maker	gene	93684	94427	.	+	.	ID=MALK_03572;prediction_source=maker_MRET:augustus_masked-contig013-processed-gene-0.70-mRNA-1
contig013	maker	CDS	93684	94427	.	+	0	ID=MALK_03572.t1.c1;Parent=MALK_03572.t1
contig013	maker	mRNA	93684	94427	.	+	.	ID=MALK_03572.t1;Parent=MALK_03572
contig013	maker	exon	93684	94427	.	+	.	ID=MALK_03572.t1.e1;Parent=MALK_03572.t1
contig013	AUGUSTUS	gene	94428	95135	0.96	-	.	ID=MALK_03573;prediction_source=braker_MRET:g3887.t1
contig013	AUGUSTUS	CDS	94428	95135	0.96	-	0	ID=MALK_03573.t1.c1;Parent=MALK_03573.t1
contig013	AUGUSTUS	mRNA	94428	95135	0.96	-	.	ID=MALK_03573.t1;Parent=MALK_03573
contig013	AUGUSTUS	exon	94428	95135	.	-	.	ID=MALK_03573.t1.e1;Parent=MALK_03573.t1
contig013	AUGUSTUS	gene	95220	97511	0.98	+	.	ID=MALK_03574;prediction_source=braker_MRET:g3888.t1
contig013	AUGUSTUS	CDS	95220	97511	0.98	+	0	ID=MALK_03574.t1.c1;Parent=MALK_03574.t1
contig013	AUGUSTUS	mRNA	95220	97511	0.98	+	.	ID=MALK_03574.t1;Parent=MALK_03574
contig013	AUGUSTUS	exon	95220	97511	.	+	.	ID=MALK_03574.t1.e1;Parent=MALK_03574.t1
contig013	AUGUSTUS	gene	97620	98969	0.97	+	.	ID=MALK_03575;prediction_source=augustus:contig013.g3011.t1
contig013	AUGUSTUS	CDS	97620	98969	0.97	+	0	ID=MALK_03575.t1.c1;Parent=MALK_03575.t1
contig013	AUGUSTUS	mRNA	97620	98969	0.97	+	.	ID=MALK_03575.t1;Parent=MALK_03575

contig013	AUGUSTUS	exon	97620	98969	0.97	+	.	ID=MALK_03575.t1.e1;Parent=MALK_03575.t1
contig013	AUGUSTUS	gene	99091	101829	0.85	+	.	ID=MALK_03576;prediction_source=braker_MRET:g3890.t1
contig013	AUGUSTUS	CDS	99091	101829	0.85	+	0	ID=MALK_03576.t1.c1;Parent=MALK_03576.t1
contig013	AUGUSTUS	mRNA	99091	101829	0.85	+	.	ID=MALK_03576.t1;Parent=MALK_03576
contig013	AUGUSTUS	exon	99091	101829	.	+	.	ID=MALK_03576.t1.e1;Parent=MALK_03576.t1
contig013	maker	gene	101896	104244	.	+	.	ID=MALK_03577;prediction_source=maker_MRET:augustus_masked-contig013-processed-gene-1.2-mRNA-1
contig013	maker	CDS	101896	104244	.	+	0	ID=MALK_03577.t1.c1;Parent=MALK_03577.t1
contig013	maker	mRNA	101896	104244	.	+	.	ID=MALK_03577.t1;Parent=MALK_03577
contig013	maker	exon	101896	104244	.	+	.	ID=MALK_03577.t1.e1;Parent=MALK_03577.t1
contig013	AUGUSTUS	gene	104441	105550	0.3	+	.	ID=MALK_03578;prediction_source=augustus:contig013.g3015.t1
contig013	AUGUSTUS	CDS	104441	105550	0.3	+	0	ID=MALK_03578.t1.c1;Parent=MALK_03578.t1
contig013	AUGUSTUS	mRNA	104441	105550	0.3	+	.	ID=MALK_03578.t1;Parent=MALK_03578
contig013	AUGUSTUS	exon	104441	105550	0.3	+	.	ID=MALK_03578.t1.e1;Parent=MALK_03578.t1
contig013	maker	gene	105600	106886	.	-	.	ID=MALK_03579;prediction_source=maker_MRET:augustus_masked-contig013-processed-gene-1.37-mRNA-1
contig013	maker	CDS	105600	106886	.	-	0	ID=MALK_03579.t1.c1;Parent=MALK_03579.t1
contig013	maker	mRNA	105600	106886	.	-	.	ID=MALK_03579.t1;Parent=MALK_03579
contig013	maker	exon	105600	106886	.	-	.	ID=MALK_03579.t1.e1;Parent=MALK_03579.t1
contig013	maker	gene	107083	108540	.	-	.	ID=MALK_03580;prediction_source=maker_MRET:augustus_masked-contig013-processed-gene-1.38-mRNA-1
contig013	maker	CDS	107083	108540	.	-	0	ID=MALK_03580.t1.c1;Parent=MALK_03580.t1
contig013	maker	mRNA	107083	108540	.	-	.	ID=MALK_03580.t1;Parent=MALK_03580
contig013	maker	exon	107083	108540	.	-	.	ID=MALK_03580.t1.e1;Parent=MALK_03580.t1
contig013	maker	gene	108797	110692	.	+	.	ID=MALK_03581;prediction_source=maker_MRET:augustus_masked-contig013-processed-gene-1.4-mRNA-1
contig013	maker	CDS	108797	110692	.	+	0	ID=MALK_03581.t1.c1;Parent=MALK_03581.t1
contig013	maker	mRNA	108797	110692	.	+	.	ID=MALK_03581.t1;Parent=MALK_03581
contig013	maker	exon	108797	110692	.	+	.	ID=MALK_03581.t1.e1;Parent=MALK_03581.t1
contig013	AUGUSTUS	gene	110689	111435	0.55	-	.	ID=MALK_03582;prediction_source=augustus:contig013.g3020.t1
contig013	AUGUSTUS	CDS	110689	111435	0.55	-	0	ID=MALK_03582.t1.c1;Parent=MALK_03582.t1
contig013	AUGUSTUS	mRNA	110689	111435	0.55	-	.	ID=MALK_03582.t1;Parent=MALK_03582
contig013	AUGUSTUS	exon	110689	111435	0.55	-	.	ID=MALK_03582.t1.e1;Parent=MALK_03582.t1
contig013	AUGUSTUS	gene	111428	112007	0.31	+	.	ID=MALK_03583;prediction_source=braker_MRET:g3896.t1
contig013	AUGUSTUS	CDS	111428	111776	0.43	+	0	ID=MALK_03583.t1.c1;Parent=MALK_03583.t1
contig013	AUGUSTUS	CDS	111838	112007	0.43	+	0	ID=MALK_03583.t1.c2;Parent=MALK_03583.t1
contig013	AUGUSTUS	mRNA	111428	112007	0.31	+	.	ID=MALK_03583.t1;Parent=MALK_03583
contig013	AUGUSTUS	exon	111428	111776	.	+	.	ID=MALK_03583.t1.e1;Parent=MALK_03583.t1
contig013	AUGUSTUS	exon	111838	112007	.	+	.	ID=MALK_03583.t1.e2;Parent=MALK_03583.t1
contig013	maker	gene	112083	113612	.	+	.	ID=MALK_03584;prediction_source=maker_MRET:augustus_masked-contig013-processed-gene-1.6-mRNA-1
contig013	maker	CDS	112083	113612	.	+	0	ID=MALK_03584.t1.c1;Parent=MALK_03584.t1
contig013	maker	mRNA	112083	113612	.	+	.	ID=MALK_03584.t1;Parent=MALK_03584
contig013	maker	exon	112083	113612	.	+	.	ID=MALK_03584.t1.e1;Parent=MALK_03584.t1
contig013	AUGUSTUS	gene	113607	114386	0.97	-	.	ID=MALK_03585;prediction_source=augustus:contig013.g3023.t1
contig013	AUGUSTUS	CDS	113607	114386	0.97	-	0	ID=MALK_03585.t1.c1;Parent=MALK_03585.t1
contig013	AUGUSTUS	mRNA	113607	114386	0.97	-	.	ID=MALK_03585.t1;Parent=MALK_03585
contig013	AUGUSTUS	exon	113607	114386	0.97	-	.	ID=MALK_03585.t1.e1;Parent=MALK_03585.t1

contig013	maker	gene	114678	115706	.	+	.	ID=MALK_03586;prediction_source=maker_MRET:augustus_masked-contig013-processed-gene-1.7-mRNA-1
contig013	maker	CDS	114678	115706	.	+	0	ID=MALK_03586.t1.c1;Parent=MALK_03586.t1
contig013	maker	mRNA	114678	115706	.	+	.	ID=MALK_03586.t1;Parent=MALK_03586
contig013	maker	exon	114678	115706	.	+	.	ID=MALK_03586.t1.e1;Parent=MALK_03586.t1
contig013	AUGUSTUS	gene	115709	117505	0.23	-	.	ID=MALK_03587;prediction_source=augustus:contig013.g3025.t1
contig013	AUGUSTUS	CDS	116749	117505	0.34	-	0	ID=MALK_03587.t1.c2;Parent=MALK_03587.t1
contig013	AUGUSTUS	CDS	115709	116379	0.34	-	0	ID=MALK_03587.t1.c1;Parent=MALK_03587.t1
contig013	AUGUSTUS	mRNA	115709	117505	0.23	-	.	ID=MALK_03587.t1;Parent=MALK_03587
contig013	AUGUSTUS	exon	116749	117505	0.34	-	.	ID=MALK_03587.t1.e2;Parent=MALK_03587.t1
contig013	AUGUSTUS	exon	115709	116379	0.34	-	.	ID=MALK_03587.t1.e1;Parent=MALK_03587.t1
contig013	AUGUSTUS	gene	117384	119423	0.18	+	.	ID=MALK_03588;prediction_source=braker_MRET:g3901.t1
contig013	AUGUSTUS	CDS	117384	118380	0.6	+	0	ID=MALK_03588.t1.c1;Parent=MALK_03588.t1
contig013	AUGUSTUS	CDS	118428	118543	0.6	+	0	ID=MALK_03588.t1.c2;Parent=MALK_03588.t1
contig013	AUGUSTUS	CDS	118863	119423	0.6	+	0	ID=MALK_03588.t1.c3;Parent=MALK_03588.t1
contig013	AUGUSTUS	mRNA	117384	119423	0.18	+	.	ID=MALK_03588.t1;Parent=MALK_03588
contig013	AUGUSTUS	exon	117384	118380	.	+	.	ID=MALK_03588.t1.e1;Parent=MALK_03588.t1
contig013	AUGUSTUS	exon	118428	118543	.	+	.	ID=MALK_03588.t1.e2;Parent=MALK_03588.t1
contig013	AUGUSTUS	exon	118863	119423	.	+	.	ID=MALK_03588.t1.e3;Parent=MALK_03588.t1
contig013	AUGUSTUS	gene	119445	120014	0.45	-	.	ID=MALK_03589;prediction_source=braker_MRET:g3902.t1
contig013	AUGUSTUS	CDS	119445	120014	0.45	-	0	ID=MALK_03589.t1.c1;Parent=MALK_03589.t1
contig013	AUGUSTUS	mRNA	119445	120014	0.45	-	.	ID=MALK_03589.t1;Parent=MALK_03589
contig013	AUGUSTUS	exon	119445	120014	.	-	.	ID=MALK_03589.t1.e1;Parent=MALK_03589.t1
contig013	AUGUSTUS	gene	120094	122895	0.67	-	.	ID=MALK_03590;prediction_source=braker_MRET:g3903.t1
contig013	AUGUSTUS	CDS	120094	122895	0.67	-	0	ID=MALK_03590.t1.c1;Parent=MALK_03590.t1
contig013	AUGUSTUS	mRNA	120094	122895	0.67	-	.	ID=MALK_03590.t1;Parent=MALK_03590
contig013	AUGUSTUS	exon	120094	122895	.	-	.	ID=MALK_03590.t1.e1;Parent=MALK_03590.t1
contig013	AUGUSTUS	gene	122984	124348	0.67	-	.	ID=MALK_03591;prediction_source=braker_MRET:g3904.t1
contig013	AUGUSTUS	CDS	122984	124348	0.67	-	0	ID=MALK_03591.t1.c1;Parent=MALK_03591.t1
contig013	AUGUSTUS	mRNA	122984	124348	0.67	-	.	ID=MALK_03591.t1;Parent=MALK_03591
contig013	AUGUSTUS	exon	122984	124348	.	-	.	ID=MALK_03591.t1.e1;Parent=MALK_03591.t1
contig013	maker	gene	124470	125390	.	+	.	ID=MALK_03592;prediction_source=maker_MRET:augustus_masked-contig013-processed-gene-1.9-mRNA-1
contig013	maker	CDS	124470	125390	.	+	0	ID=MALK_03592.t1.c1;Parent=MALK_03592.t1
contig013	maker	mRNA	124470	125390	.	+	.	ID=MALK_03592.t1;Parent=MALK_03592
contig013	maker	exon	124470	125390	.	+	.	ID=MALK_03592.t1.e1;Parent=MALK_03592.t1
contig013	AUGUSTUS	gene	126729	129047	0.88	+	.	ID=MALK_03593;prediction_source=augustus:contig013.g3029.t1
contig013	AUGUSTUS	CDS	126729	129047	0.88	+	0	ID=MALK_03593.t1.c1;Parent=MALK_03593.t1
contig013	AUGUSTUS	mRNA	126729	129047	0.88	+	.	ID=MALK_03593.t1;Parent=MALK_03593
contig013	AUGUSTUS	exon	126729	129047	0.88	+	.	ID=MALK_03593.t1.e1;Parent=MALK_03593.t1
contig013	AUGUSTUS	gene	129048	130322	0.92	-	.	ID=MALK_03594;prediction_source=braker_MRET:g3907.t1
contig013	AUGUSTUS	CDS	129048	130322	0.92	-	0	ID=MALK_03594.t1.c1;Parent=MALK_03594.t1
contig013	AUGUSTUS	mRNA	129048	130322	0.92	-	.	ID=MALK_03594.t1;Parent=MALK_03594
contig013	AUGUSTUS	exon	129048	130322	.	-	.	ID=MALK_03594.t1.e1;Parent=MALK_03594.t1
contig013	AUGUSTUS	gene	130339	131700	1	-	.	ID=MALK_03595;prediction_source=braker_MRET:g3908.t1

contig013	AUGUSTUS	CDS	130339	131700	1	-	0	ID=MALK_03595.t1.c1;Parent=MALK_03595.t1
contig013	AUGUSTUS	mRNA	130339	131700	1	-	.	ID=MALK_03595.t1;Parent=MALK_03595
contig013	AUGUSTUS	exon	130339	131700	.	-	.	ID=MALK_03595.t1.e1;Parent=MALK_03595.t1
contig013	maker	gene	131736	133763	.	-	.	ID=MALK_03596;prediction_source=maker_MRET:augustus_masked-contig013-processed-gene-1.42-mRNA-1
contig013	maker	CDS	131736	133763	.	-	0	ID=MALK_03596.t1.c1;Parent=MALK_03596.t1
contig013	maker	mRNA	131736	133763	.	-	.	ID=MALK_03596.t1;Parent=MALK_03596
contig013	maker	exon	131736	133763	.	-	.	ID=MALK_03596.t1.e1;Parent=MALK_03596.t1
contig013	AUGUSTUS	gene	133874	137425	0.91	+	.	ID=MALK_03597;prediction_source=braker_MRET:g3910.t1
contig013	AUGUSTUS	CDS	133874	137425	0.91	+	0	ID=MALK_03597.t1.c1;Parent=MALK_03597.t1
contig013	AUGUSTUS	mRNA	133874	137425	0.91	+	.	ID=MALK_03597.t1;Parent=MALK_03597
contig013	AUGUSTUS	exon	133874	137425	.	+	.	ID=MALK_03597.t1.e1;Parent=MALK_03597.t1
contig013	AUGUSTUS	gene	137545	138916	0.64	+	.	ID=MALK_03598;prediction_source=braker_MRET:g3911.t1
contig013	AUGUSTUS	CDS	137545	137655	0.94	+	0	ID=MALK_03598.t1.c1;Parent=MALK_03598.t1
contig013	AUGUSTUS	CDS	137690	138916	0.94	+	0	ID=MALK_03598.t1.c2;Parent=MALK_03598.t1
contig013	AUGUSTUS	mRNA	137545	138916	0.64	+	.	ID=MALK_03598.t1;Parent=MALK_03598
contig013	AUGUSTUS	exon	137545	137655	.	+	.	ID=MALK_03598.t1.e1;Parent=MALK_03598.t1
contig013	AUGUSTUS	exon	137690	138916	.	+	.	ID=MALK_03598.t1.e2;Parent=MALK_03598.t1
contig013	maker	gene	139027	140763	.	-	.	ID=MALK_03599;prediction_source=maker_MRET:augustus_masked-contig013-processed-gene-1.43-mRNA-1
contig013	maker	CDS	139027	140763	.	-	0	ID=MALK_03599.t1.c1;Parent=MALK_03599.t1
contig013	maker	mRNA	139027	140763	.	-	.	ID=MALK_03599.t1;Parent=MALK_03599
contig013	maker	exon	139027	140763	.	-	.	ID=MALK_03599.t1.e1;Parent=MALK_03599.t1
contig013	AUGUSTUS	gene	141076	141442	0.99	+	.	ID=MALK_03600;prediction_source=braker_MRET:g3913.t1
contig013	AUGUSTUS	CDS	141076	141078	1	+	0	ID=MALK_03600.t1.c1;Parent=MALK_03600.t1
contig013	AUGUSTUS	CDS	141119	141442	1	+	0	ID=MALK_03600.t1.c2;Parent=MALK_03600.t1
contig013	AUGUSTUS	mRNA	141076	141442	0.99	+	.	ID=MALK_03600.t1;Parent=MALK_03600
contig013	AUGUSTUS	exon	141076	141078	.	+	.	ID=MALK_03600.t1.e1;Parent=MALK_03600.t1
contig013	AUGUSTUS	exon	141119	141442	.	+	.	ID=MALK_03600.t1.e2;Parent=MALK_03600.t1
contig013	AUGUSTUS	gene	141571	142084	0.27	-	.	ID=MALK_03601;prediction_source=braker_MRET:g3914.t1
contig013	AUGUSTUS	CDS	142059	142084	0.45	-	0	ID=MALK_03601.t1.c3;Parent=MALK_03601.t1
contig013	AUGUSTUS	CDS	141916	142031	0.45	-	0	ID=MALK_03601.t1.c2;Parent=MALK_03601.t1
contig013	AUGUSTUS	CDS	141571	141866	0.45	-	0	ID=MALK_03601.t1.c1;Parent=MALK_03601.t1
contig013	AUGUSTUS	mRNA	141571	142084	0.27	-	.	ID=MALK_03601.t1;Parent=MALK_03601
contig013	AUGUSTUS	exon	142059	142084	.	-	.	ID=MALK_03601.t1.e3;Parent=MALK_03601.t1
contig013	AUGUSTUS	exon	141916	142031	.	-	.	ID=MALK_03601.t1.e2;Parent=MALK_03601.t1
contig013	AUGUSTUS	exon	141571	141866	.	-	.	ID=MALK_03601.t1.e1;Parent=MALK_03601.t1
contig013	AUGUSTUS	gene	142309	143997	0.83	+	.	ID=MALK_03602;prediction_source=braker_MRET:g3915.t1
contig013	AUGUSTUS	CDS	142309	143997	0.83	+	0	ID=MALK_03602.t1.c1;Parent=MALK_03602.t1
contig013	AUGUSTUS	mRNA	142309	143997	0.83	+	.	ID=MALK_03602.t1;Parent=MALK_03602
contig013	AUGUSTUS	exon	142309	143997	.	+	.	ID=MALK_03602.t1.e1;Parent=MALK_03602.t1
contig013	AUGUSTUS	gene	144029	145141	0.94	-	.	ID=MALK_03603;prediction_source=augustus:contig013.g3037.t1
contig013	AUGUSTUS	CDS	144029	145141	0.94	-	0	ID=MALK_03603.t1.c1;Parent=MALK_03603.t1
contig013	AUGUSTUS	mRNA	144029	145141	0.94	-	.	ID=MALK_03603.t1;Parent=MALK_03603
contig013	AUGUSTUS	exon	144029	145141	0.94	-	.	ID=MALK_03603.t1.e1;Parent=MALK_03603.t1

contig013	AUGUSTUS	gene	145317	146434	0.55	+	.	ID=MALK_03604;prediction_source=braker_MRET:g3917.t1
contig013	AUGUSTUS	CDS	145317	146337	0.57	+	0	ID=MALK_03604.t1.c1;Parent=MALK_03604.t1
contig013	AUGUSTUS	CDS	146385	146434	0.57	+	0	ID=MALK_03604.t1.c2;Parent=MALK_03604.t1
contig013	AUGUSTUS	mRNA	145317	146434	0.55	+	.	ID=MALK_03604.t1;Parent=MALK_03604
contig013	AUGUSTUS	exon	145317	146337	.	+	.	ID=MALK_03604.t1.e1;Parent=MALK_03604.t1
contig013	AUGUSTUS	exon	146385	146434	.	+	.	ID=MALK_03604.t1.e2;Parent=MALK_03604.t1
contig013	AUGUSTUS	gene	146482	148311	0.94	-	.	ID=MALK_03605;prediction_source=augustus:contig013.g3038.t1
contig013	AUGUSTUS	CDS	146482	148311	0.94	-	0	ID=MALK_03605.t1.c1;Parent=MALK_03605.t1
contig013	AUGUSTUS	mRNA	146482	148311	0.94	-	.	ID=MALK_03605.t1;Parent=MALK_03605
contig013	AUGUSTUS	exon	146482	148311	0.94	-	.	ID=MALK_03605.t1.e1;Parent=MALK_03605.t1
contig013	AUGUSTUS	gene	148430	148687	0.59	+	.	ID=MALK_03606;prediction_source=augustus:contig013.g3039.t1
contig013	AUGUSTUS	CDS	148430	148687	0.59	+	0	ID=MALK_03606.t1.c1;Parent=MALK_03606.t1
contig013	AUGUSTUS	mRNA	148430	148687	0.59	+	.	ID=MALK_03606.t1;Parent=MALK_03606
contig013	AUGUSTUS	exon	148430	148687	0.59	+	.	ID=MALK_03606.t1.e1;Parent=MALK_03606.t1
contig013	maker	gene	148754	149530	.	-	.	ID=MALK_03607;prediction_source=maker_MRET:augustus_masked-contig013-processed-gene-1.46-mRNA-1
contig013	maker	CDS	148754	149530	.	-	0	ID=MALK_03607.t1.c1;Parent=MALK_03607.t1
contig013	maker	mRNA	148754	149530	.	-	.	ID=MALK_03607.t1;Parent=MALK_03607
contig013	maker	exon	148754	149530	.	-	.	ID=MALK_03607.t1.e1;Parent=MALK_03607.t1
contig013	AUGUSTUS	gene	149633	149890	0.99	+	.	ID=MALK_03608;prediction_source=braker_MRET:g3921.t1
contig013	AUGUSTUS	CDS	149633	149890	0.99	+	0	ID=MALK_03608.t1.c1;Parent=MALK_03608.t1
contig013	AUGUSTUS	mRNA	149633	149890	0.99	+	.	ID=MALK_03608.t1;Parent=MALK_03608
contig013	AUGUSTUS	exon	149633	149890	.	+	.	ID=MALK_03608.t1.e1;Parent=MALK_03608.t1
contig013	AUGUSTUS	gene	149933	155507	0.22	-	.	ID=MALK_03609;prediction_source=braker_MRET:g3922.t1
contig013	AUGUSTUS	CDS	155434	155507	0.25	-	0	ID=MALK_03609.t1.c2;Parent=MALK_03609.t1
contig013	AUGUSTUS	CDS	149933	155399	0.25	-	0	ID=MALK_03609.t1.c1;Parent=MALK_03609.t1
contig013	AUGUSTUS	mRNA	149933	155507	0.22	-	.	ID=MALK_03609.t1;Parent=MALK_03609
contig013	AUGUSTUS	exon	155434	155507	.	-	.	ID=MALK_03609.t1.e2;Parent=MALK_03609.t1
contig013	AUGUSTUS	exon	149933	155399	.	-	.	ID=MALK_03609.t1.e1;Parent=MALK_03609.t1
contig013	AUGUSTUS	gene	155633	156442	0.28	-	.	ID=MALK_03610;prediction_source=braker_MRET:g3923.t1
contig013	AUGUSTUS	CDS	155633	156442	0.28	-	0	ID=MALK_03610.t1.c1;Parent=MALK_03610.t1
contig013	AUGUSTUS	mRNA	155633	156442	0.28	-	.	ID=MALK_03610.t1;Parent=MALK_03610
contig013	AUGUSTUS	exon	155633	156442	.	-	.	ID=MALK_03610.t1.e1;Parent=MALK_03610.t1
contig013	AUGUSTUS	gene	156559	157235	0.5	+	.	ID=MALK_03611;prediction_source=braker_MRET:g3924.t1
contig013	AUGUSTUS	CDS	156559	156667	0.53	+	0	ID=MALK_03611.t1.c1;Parent=MALK_03611.t1
contig013	AUGUSTUS	CDS	156700	156778	0.53	+	0	ID=MALK_03611.t1.c2;Parent=MALK_03611.t1
contig013	AUGUSTUS	CDS	156812	157235	0.53	+	0	ID=MALK_03611.t1.c3;Parent=MALK_03611.t1
contig013	AUGUSTUS	mRNA	156559	157235	0.5	+	.	ID=MALK_03611.t1;Parent=MALK_03611
contig013	AUGUSTUS	exon	156559	156667	.	+	.	ID=MALK_03611.t1.e1;Parent=MALK_03611.t1
contig013	AUGUSTUS	exon	156700	156778	.	+	.	ID=MALK_03611.t1.e2;Parent=MALK_03611.t1
contig013	AUGUSTUS	exon	156812	157235	.	+	.	ID=MALK_03611.t1.e3;Parent=MALK_03611.t1
contig013	AUGUSTUS	gene	157532	164323	0.84	+	.	ID=MALK_03612;prediction_source=augustus:contig013.g3045.t1
contig013	AUGUSTUS	CDS	157532	164323	0.84	+	0	ID=MALK_03612.t1.c1;Parent=MALK_03612.t1
contig013	AUGUSTUS	mRNA	157532	164323	0.84	+	.	ID=MALK_03612.t1;Parent=MALK_03612

contig013	AUGUSTUS	exon	157532	164323	0.84	+	.	ID=MALK_03612.t1.e1;Parent=MALK_03612.t1
contig013	AUGUSTUS	gene	164602	165270	0.34	+	.	ID=MALK_03613;prediction_source=braker_MRET:g3926.t1
contig013	AUGUSTUS	CDS	164602	165270	0.34	+	0	ID=MALK_03613.t1.c1;Parent=MALK_03613.t1
contig013	AUGUSTUS	mRNA	164602	165270	0.34	+	.	ID=MALK_03613.t1;Parent=MALK_03613
contig013	AUGUSTUS	exon	164602	165270	.	+	.	ID=MALK_03613.t1.e1;Parent=MALK_03613.t1
contig013	AUGUSTUS	gene	165287	166237	0.99	-	.	ID=MALK_03614;prediction_source=augustus:contig013.g3046.t1
contig013	AUGUSTUS	CDS	165287	166237	0.99	-	0	ID=MALK_03614.t1.c1;Parent=MALK_03614.t1
contig013	AUGUSTUS	mRNA	165287	166237	0.99	-	.	ID=MALK_03614.t1;Parent=MALK_03614
contig013	AUGUSTUS	exon	165287	166237	0.99	-	.	ID=MALK_03614.t1.e1;Parent=MALK_03614.t1
contig013	AUGUSTUS	gene	166414	167376	0.71	+	.	ID=MALK_03615;prediction_source=augustus:contig013.g3047.t1
contig013	AUGUSTUS	CDS	166414	167376	0.71	+	0	ID=MALK_03615.t1.c1;Parent=MALK_03615.t1
contig013	AUGUSTUS	mRNA	166414	167376	0.71	+	.	ID=MALK_03615.t1;Parent=MALK_03615
contig013	AUGUSTUS	exon	166414	167376	0.71	+	.	ID=MALK_03615.t1.e1;Parent=MALK_03615.t1
contig013	maker	gene	167462	169891	.	+	.	ID=MALK_03616;prediction_source=maker_MRET:augustus_masked-contig013-processed-gene-1.16-mRNA-1
contig013	maker	CDS	167462	169891	.	+	0	ID=MALK_03616.t1.c1;Parent=MALK_03616.t1
contig013	maker	mRNA	167462	169891	.	+	.	ID=MALK_03616.t1;Parent=MALK_03616
contig013	maker	exon	167462	169891	.	+	.	ID=MALK_03616.t1.e1;Parent=MALK_03616.t1
contig013	AUGUSTUS	gene	169927	170235	0.91	-	.	ID=MALK_03617;prediction_source=braker_MRET:g3930.t1
contig013	AUGUSTUS	CDS	169927	170235	0.91	-	0	ID=MALK_03617.t1.c1;Parent=MALK_03617.t1
contig013	AUGUSTUS	mRNA	169927	170235	0.91	-	.	ID=MALK_03617.t1;Parent=MALK_03617
contig013	AUGUSTUS	exon	169927	170235	.	-	.	ID=MALK_03617.t1.e1;Parent=MALK_03617.t1
contig013	AUGUSTUS	gene	170354	171156	0.93	+	.	ID=MALK_03618;prediction_source=braker_MRET:g3931.t1
contig013	AUGUSTUS	CDS	170354	171031	1	+	0	ID=MALK_03618.t1.c1;Parent=MALK_03618.t1
contig013	AUGUSTUS	CDS	171112	171156	1	+	0	ID=MALK_03618.t1.c2;Parent=MALK_03618.t1
contig013	AUGUSTUS	mRNA	170354	171156	0.93	+	.	ID=MALK_03618.t1;Parent=MALK_03618
contig013	AUGUSTUS	exon	170354	171031	.	+	.	ID=MALK_03618.t1.e1;Parent=MALK_03618.t1
contig013	AUGUSTUS	exon	171112	171156	.	+	.	ID=MALK_03618.t1.e2;Parent=MALK_03618.t1
contig013	maker	gene	171217	173277	.	-	.	ID=MALK_03619;prediction_source=maker_MRET:augustus_masked-contig013-processed-gene-1.49-mRNA-1
contig013	maker	CDS	171217	173277	.	-	0	ID=MALK_03619.t1.c1;Parent=MALK_03619.t1
contig013	maker	mRNA	171217	173277	.	-	.	ID=MALK_03619.t1;Parent=MALK_03619
contig013	maker	exon	171217	173277	.	-	.	ID=MALK_03619.t1.e1;Parent=MALK_03619.t1
contig013	AUGUSTUS	gene	173595	174971	0.41	+	.	ID=MALK_03620;prediction_source=augustus:contig013.g3051.t1
contig013	AUGUSTUS	CDS	173595	174971	0.41	+	0	ID=MALK_03620.t1.c1;Parent=MALK_03620.t1
contig013	AUGUSTUS	mRNA	173595	174971	0.41	+	.	ID=MALK_03620.t1;Parent=MALK_03620
contig013	AUGUSTUS	exon	173595	174971	0.41	+	.	ID=MALK_03620.t1.e1;Parent=MALK_03620.t1
contig013	AUGUSTUS	gene	174973	175426	0.93	-	.	ID=MALK_03621;prediction_source=braker_MRET:g3934.t1
contig013	AUGUSTUS	CDS	175015	175426	0.93	-	0	ID=MALK_03621.t1.c2;Parent=MALK_03621.t1
contig013	AUGUSTUS	CDS	174973	174980	0.93	-	0	ID=MALK_03621.t1.c1;Parent=MALK_03621.t1
contig013	AUGUSTUS	mRNA	174973	175426	0.93	-	.	ID=MALK_03621.t1;Parent=MALK_03621
contig013	AUGUSTUS	exon	175015	175426	.	-	.	ID=MALK_03621.t1.e2;Parent=MALK_03621.t1
contig013	AUGUSTUS	exon	174973	174980	.	-	.	ID=MALK_03621.t1.e1;Parent=MALK_03621.t1
contig013	maker	gene	175745	177541	.	+	.	ID=MALK_03622;prediction_source=maker_MRET:augustus_masked-contig013-processed-gene-1.18-mRNA-1
contig013	maker	CDS	175745	177541	.	+	0	ID=MALK_03622.t1.c1;Parent=MALK_03622.t1

contig013	maker	mRNA	175745	177541	.	+	.	ID=MALK_03622.t1;Parent=MALK_03622
contig013	maker	exon	175745	177541	.	+	.	ID=MALK_03622.t1.e1;Parent=MALK_03622.t1
contig013	maker	gene	177573	178178	.	-	.	ID=MALK_03623;prediction_source=maker_MRET:augustus_masked-contig013-processed-gene-1.50-mRNA-1
contig013	maker	CDS	177573	178178	.	-	0	ID=MALK_03623.t1.c1;Parent=MALK_03623.t1
contig013	maker	mRNA	177573	178178	.	-	.	ID=MALK_03623.t1;Parent=MALK_03623
contig013	maker	exon	177573	178178	.	-	.	ID=MALK_03623.t1.e1;Parent=MALK_03623.t1
contig013	maker	gene	178430	179491	.	+	.	ID=MALK_03624;prediction_source=maker_MRET:augustus_masked-contig013-processed-gene-1.19-mRNA-1
contig013	maker	CDS	178430	179491	.	+	0	ID=MALK_03624.t1.c1;Parent=MALK_03624.t1
contig013	maker	mRNA	178430	179491	.	+	.	ID=MALK_03624.t1;Parent=MALK_03624
contig013	maker	exon	178430	179491	.	+	.	ID=MALK_03624.t1.e1;Parent=MALK_03624.t1
contig013	maker	gene	179496	180434	.	-	.	ID=MALK_03625;prediction_source=maker_MRET:augustus_masked-contig013-processed-gene-1.51-mRNA-1
contig013	maker	CDS	179496	180434	.	-	0	ID=MALK_03625.t1.c1;Parent=MALK_03625.t1
contig013	maker	mRNA	179496	180434	.	-	.	ID=MALK_03625.t1;Parent=MALK_03625
contig013	maker	exon	179496	180434	.	-	.	ID=MALK_03625.t1.e1;Parent=MALK_03625.t1
contig013	AUGUSTUS	gene	180568	182298	1	+	.	ID=MALK_03626;prediction_source=augustus:contig013.g3056.t1
contig013	AUGUSTUS	CDS	180568	182298	1	+	0	ID=MALK_03626.t1.c1;Parent=MALK_03626.t1
contig013	AUGUSTUS	mRNA	180568	182298	1	+	.	ID=MALK_03626.t1;Parent=MALK_03626
contig013	AUGUSTUS	exon	180568	182298	1	+	.	ID=MALK_03626.t1.e1;Parent=MALK_03626.t1
contig013	AUGUSTUS	gene	182466	184733	0.69	+	.	ID=MALK_03627;prediction_source=augustus:contig013.g3057.t1
contig013	AUGUSTUS	CDS	182466	184733	0.69	+	0	ID=MALK_03627.t1.c1;Parent=MALK_03627.t1
contig013	AUGUSTUS	mRNA	182466	184733	0.69	+	.	ID=MALK_03627.t1;Parent=MALK_03627
contig013	AUGUSTUS	exon	182466	184733	0.69	+	.	ID=MALK_03627.t1.e1;Parent=MALK_03627.t1
contig013	AUGUSTUS	gene	184883	185234	0.36	-	.	ID=MALK_03628;prediction_source=braker_MRET:g3941.t1
contig013	AUGUSTUS	CDS	184922	185234	0.36	-	0	ID=MALK_03628.t1.c2;Parent=MALK_03628.t1
contig013	AUGUSTUS	CDS	184883	184887	0.36	-	0	ID=MALK_03628.t1.c1;Parent=MALK_03628.t1
contig013	AUGUSTUS	mRNA	184883	185234	0.36	-	.	ID=MALK_03628.t1;Parent=MALK_03628
contig013	AUGUSTUS	exon	184922	185234	.	-	.	ID=MALK_03628.t1.e2;Parent=MALK_03628.t1
contig013	AUGUSTUS	exon	184883	184887	.	-	.	ID=MALK_03628.t1.e1;Parent=MALK_03628.t1
contig013	AUGUSTUS	gene	185289	187901	0.83	+	.	ID=MALK_03629;prediction_source=braker_MRET:g3942.t1
contig013	AUGUSTUS	CDS	185289	187901	0.83	+	0	ID=MALK_03629.t1.c1;Parent=MALK_03629.t1
contig013	AUGUSTUS	mRNA	185289	187901	0.83	+	.	ID=MALK_03629.t1;Parent=MALK_03629
contig013	AUGUSTUS	exon	185289	187901	.	+	.	ID=MALK_03629.t1.e1;Parent=MALK_03629.t1
contig013	AUGUSTUS	gene	187950	188597	0.99	+	.	ID=MALK_03630;prediction_source=augustus:contig013.g3060.t1
contig013	AUGUSTUS	CDS	187950	188597	0.99	+	0	ID=MALK_03630.t1.c1;Parent=MALK_03630.t1
contig013	AUGUSTUS	mRNA	187950	188597	0.99	+	.	ID=MALK_03630.t1;Parent=MALK_03630
contig013	AUGUSTUS	exon	187950	188597	0.99	+	.	ID=MALK_03630.t1.e1;Parent=MALK_03630.t1
contig013	AUGUSTUS	gene	189974	191980	0.46	-	.	ID=MALK_03631;prediction_source=augustus:contig013.g3061.t1
contig013	AUGUSTUS	CDS	189974	191980	0.46	-	0	ID=MALK_03631.t1.c1;Parent=MALK_03631.t1
contig013	AUGUSTUS	mRNA	189974	191980	0.46	-	.	ID=MALK_03631.t1;Parent=MALK_03631
contig013	AUGUSTUS	exon	189974	191980	0.46	-	.	ID=MALK_03631.t1.e1;Parent=MALK_03631.t1
contig013	maker	gene	192139	194508	.	+	.	ID=MALK_03632;prediction_source=maker_MRET:augustus_masked-contig013-processed-gene-1.24-mRNA-1
contig013	maker	CDS	192139	194508	.	+	0	ID=MALK_03632.t1.c1;Parent=MALK_03632.t1
contig013	maker	mRNA	192139	194508	.	+	.	ID=MALK_03632.t1;Parent=MALK_03632

contig013	maker	exon	192139	194508	.	+	.	ID=MALK_03632.t1.e1;Parent=MALK_03632.t1
contig013	maker	gene	194510	197368	.	-	.	ID=MALK_03633;prediction_source=maker_MRET:augustus_masked-contig013-processed-gene-1.53-mRNA-1
contig013	maker	CDS	194510	197368	.	-	0	ID=MALK_03633.t1.c1;Parent=MALK_03633.t1
contig013	maker	mRNA	194510	197368	.	-	.	ID=MALK_03633.t1;Parent=MALK_03633
contig013	maker	exon	194510	197368	.	-	.	ID=MALK_03633.t1.e1;Parent=MALK_03633.t1
contig013	AUGUSTUS	gene	197415	198578	0.81	+	.	ID=MALK_03634;prediction_source=braker_MRET:g3947.t1
contig013	AUGUSTUS	CDS	197415	198578	0.81	+	0	ID=MALK_03634.t1.c1;Parent=MALK_03634.t1
contig013	AUGUSTUS	mRNA	197415	198578	0.81	+	.	ID=MALK_03634.t1;Parent=MALK_03634
contig013	AUGUSTUS	exon	197415	198578	.	+	.	ID=MALK_03634.t1.e1;Parent=MALK_03634.t1
contig013	maker	gene	198584	204049	.	-	.	ID=MALK_03635;prediction_source=maker_MRET:augustus_masked-contig013-processed-gene-1.54-mRNA-1
contig013	maker	CDS	198584	204049	.	-	0	ID=MALK_03635.t1.c1;Parent=MALK_03635.t1
contig013	maker	mRNA	198584	204049	.	-	.	ID=MALK_03635.t1;Parent=MALK_03635
contig013	maker	exon	198584	204049	.	-	.	ID=MALK_03635.t1.e1;Parent=MALK_03635.t1
contig013	maker	gene	204895	207150	.	-	.	ID=MALK_03636;prediction_source=maker_MRET:augustus_masked-contig013-processed-gene-1.55-mRNA-1
contig013	maker	CDS	204895	207150	.	-	0	ID=MALK_03636.t1.c1;Parent=MALK_03636.t1
contig013	maker	mRNA	204895	207150	.	-	.	ID=MALK_03636.t1;Parent=MALK_03636
contig013	maker	exon	204895	207150	.	-	.	ID=MALK_03636.t1.e1;Parent=MALK_03636.t1
contig013	AUGUSTUS	gene	207343	208060	0.43	-	.	ID=MALK_03637;prediction_source=braker_MRET:g3950.t1
contig013	AUGUSTUS	CDS	207992	208060	0.9	-	0	ID=MALK_03637.t1.c2;Parent=MALK_03637.t1
contig013	AUGUSTUS	CDS	207343	207954	0.9	-	0	ID=MALK_03637.t1.c1;Parent=MALK_03637.t1
contig013	AUGUSTUS	mRNA	207343	208060	0.43	-	.	ID=MALK_03637.t1;Parent=MALK_03637
contig013	AUGUSTUS	exon	207992	208060	.	-	.	ID=MALK_03637.t1.e2;Parent=MALK_03637.t1
contig013	AUGUSTUS	exon	207343	207954	.	-	.	ID=MALK_03637.t1.e1;Parent=MALK_03637.t1
contig013	AUGUSTUS	gene	208140	208619	0.95	+	.	ID=MALK_03638;prediction_source=braker_MRET:g3951.t1
contig013	AUGUSTUS	CDS	208140	208619	0.95	+	0	ID=MALK_03638.t1.c1;Parent=MALK_03638.t1
contig013	AUGUSTUS	mRNA	208140	208619	0.95	+	.	ID=MALK_03638.t1;Parent=MALK_03638
contig013	AUGUSTUS	exon	208140	208619	.	+	.	ID=MALK_03638.t1.e1;Parent=MALK_03638.t1
contig013	AUGUSTUS	gene	208634	210427	0.4	-	.	ID=MALK_03639;prediction_source=augustus:contig013.g3069.t1
contig013	AUGUSTUS	CDS	208634	210427	0.4	-	0	ID=MALK_03639.t1.c1;Parent=MALK_03639.t1
contig013	AUGUSTUS	mRNA	208634	210427	0.4	-	.	ID=MALK_03639.t1;Parent=MALK_03639
contig013	AUGUSTUS	exon	208634	210427	0.4	-	.	ID=MALK_03639.t1.e1;Parent=MALK_03639.t1
contig013	AUGUSTUS	gene	210837	211413	1	+	.	ID=MALK_03640;prediction_source=braker_MRET:g3953.t1
contig013	AUGUSTUS	CDS	210837	210882	1	+	0	ID=MALK_03640.t1.c1;Parent=MALK_03640.t1
contig013	AUGUSTUS	CDS	210917	211413	1	+	0	ID=MALK_03640.t1.c2;Parent=MALK_03640.t1
contig013	AUGUSTUS	mRNA	210837	211413	1	+	.	ID=MALK_03640.t1;Parent=MALK_03640
contig013	AUGUSTUS	exon	210837	210882	.	+	.	ID=MALK_03640.t1.e1;Parent=MALK_03640.t1
contig013	AUGUSTUS	exon	210917	211413	.	+	.	ID=MALK_03640.t1.e2;Parent=MALK_03640.t1
contig013	AUGUSTUS	gene	211424	212359	1	-	.	ID=MALK_03641;prediction_source=augustus:contig013.g3070.t1
contig013	AUGUSTUS	CDS	211424	212359	1	-	0	ID=MALK_03641.t1.c1;Parent=MALK_03641.t1
contig013	AUGUSTUS	mRNA	211424	212359	1	-	.	ID=MALK_03641.t1;Parent=MALK_03641
contig013	AUGUSTUS	exon	211424	212359	1	-	.	ID=MALK_03641.t1.e1;Parent=MALK_03641.t1
contig013	AUGUSTUS	gene	212539	213948	0.96	-	.	ID=MALK_03642;prediction_source=augustus:contig013.g3071.t1
contig013	AUGUSTUS	CDS	212539	213948	0.96	-	0	ID=MALK_03642.t1.c1;Parent=MALK_03642.t1

contig013	AUGUSTUS	mRNA	212539	213948	0.96	-	.	ID=MALK_03642.t1;Parent=MALK_03642
contig013	AUGUSTUS	exon	212539	213948	0.96	-	.	ID=MALK_03642.t1.e1;Parent=MALK_03642.t1
contig013	maker	gene	214380	215234	.	-	.	ID=MALK_03643;prediction_source=maker_MRET:augustus_masked-contig013-processed-gene-1.60-mRNA-1
contig013	maker	CDS	214380	215234	.	-	0	ID=MALK_03643.t1.c1;Parent=MALK_03643.t1
contig013	maker	mRNA	214380	215234	.	-	.	ID=MALK_03643.t1;Parent=MALK_03643
contig013	maker	exon	214380	215234	.	-	.	ID=MALK_03643.t1.e1;Parent=MALK_03643.t1
contig013	AUGUSTUS	gene	215303	216379	0.99	+	.	ID=MALK_03644;prediction_source=augustus:contig013.g3074.t1
contig013	AUGUSTUS	CDS	215303	216379	0.99	+	0	ID=MALK_03644.t1.c1;Parent=MALK_03644.t1
contig013	AUGUSTUS	mRNA	215303	216379	0.99	+	.	ID=MALK_03644.t1;Parent=MALK_03644
contig013	AUGUSTUS	exon	215303	216379	0.99	+	.	ID=MALK_03644.t1.e1;Parent=MALK_03644.t1
contig013	maker	gene	216394	216810	.	-	.	ID=MALK_03645;prediction_source=maker_MRET:augustus_masked-contig013-processed-gene-1.61-mRNA-1
contig013	maker	CDS	216394	216810	.	-	0	ID=MALK_03645.t1.c1;Parent=MALK_03645.t1
contig013	maker	mRNA	216394	216810	.	-	.	ID=MALK_03645.t1;Parent=MALK_03645
contig013	maker	exon	216394	216810	.	-	.	ID=MALK_03645.t1.e1;Parent=MALK_03645.t1
contig013	AUGUSTUS	gene	216979	218865	0.99	+	.	ID=MALK_03646;prediction_source=augustus:contig013.g3076.t1
contig013	AUGUSTUS	CDS	216979	218865	0.99	+	0	ID=MALK_03646.t1.c1;Parent=MALK_03646.t1
contig013	AUGUSTUS	mRNA	216979	218865	0.99	+	.	ID=MALK_03646.t1;Parent=MALK_03646
contig013	AUGUSTUS	exon	216979	218865	0.99	+	.	ID=MALK_03646.t1.e1;Parent=MALK_03646.t1
contig013	AUGUSTUS	gene	219071	219772	0.98	+	.	ID=MALK_03647;prediction_source=augustus:contig013.g3079.t1
contig013	AUGUSTUS	CDS	219071	219772	0.98	+	0	ID=MALK_03647.t1.c1;Parent=MALK_03647.t1
contig013	AUGUSTUS	mRNA	219071	219772	0.98	+	.	ID=MALK_03647.t1;Parent=MALK_03647
contig013	AUGUSTUS	exon	219071	219772	0.98	+	.	ID=MALK_03647.t1.e1;Parent=MALK_03647.t1
contig013	AUGUSTUS	gene	220130	220472	0.7	-	.	ID=MALK_03648;prediction_source=braker_MRET:g3960.t1
contig013	AUGUSTUS	CDS	220460	220472	0.83	-	0	ID=MALK_03648.t1.c2;Parent=MALK_03648.t1
contig013	AUGUSTUS	CDS	220130	220401	0.83	-	0	ID=MALK_03648.t1.c1;Parent=MALK_03648.t1
contig013	AUGUSTUS	mRNA	220130	220472	0.7	-	.	ID=MALK_03648.t1;Parent=MALK_03648
contig013	AUGUSTUS	exon	220460	220472	.	-	.	ID=MALK_03648.t1.e2;Parent=MALK_03648.t1
contig013	AUGUSTUS	exon	220130	220401	.	-	.	ID=MALK_03648.t1.e1;Parent=MALK_03648.t1
contig013	AUGUSTUS	gene	220566	223715	0.47	-	.	ID=MALK_03649;prediction_source=augustus:contig013.g3080.t1
contig013	AUGUSTUS	CDS	220566	223715	0.47	-	0	ID=MALK_03649.t1.c1;Parent=MALK_03649.t1
contig013	AUGUSTUS	mRNA	220566	223715	0.47	-	.	ID=MALK_03649.t1;Parent=MALK_03649
contig013	AUGUSTUS	exon	220566	223715	0.47	-	.	ID=MALK_03649.t1.e1;Parent=MALK_03649.t1
contig013	maker	gene	223761	224312	.	+	.	ID=MALK_03650;prediction_source=maker_MRET:augustus_masked-contig013-processed-gene-1.29-mRNA-1
contig013	maker	CDS	223761	224312	.	+	0	ID=MALK_03650.t1.c1;Parent=MALK_03650.t1
contig013	maker	mRNA	223761	224312	.	+	.	ID=MALK_03650.t1;Parent=MALK_03650
contig013	maker	exon	223761	224312	.	+	.	ID=MALK_03650.t1.e1;Parent=MALK_03650.t1
contig013	AUGUSTUS	gene	224315	224740	0.95	-	.	ID=MALK_03651;prediction_source=braker_MRET:g3963.t1
contig013	AUGUSTUS	CDS	224315	224740	0.95	-	0	ID=MALK_03651.t1.c1;Parent=MALK_03651.t1
contig013	AUGUSTUS	mRNA	224315	224740	0.95	-	.	ID=MALK_03651.t1;Parent=MALK_03651
contig013	AUGUSTUS	exon	224315	224740	.	-	.	ID=MALK_03651.t1.e1;Parent=MALK_03651.t1
contig013	maker	gene	224850	225704	.	+	.	ID=MALK_03652;prediction_source=maker_MRET:augustus_masked-contig013-processed-gene-1.30-mRNA-1
contig013	maker	CDS	224850	225704	.	+	0	ID=MALK_03652.t1.c1;Parent=MALK_03652.t1
contig013	maker	mRNA	224850	225704	.	+	.	ID=MALK_03652.t1;Parent=MALK_03652

contig013	maker	exon	224850	225704	.	+	.	ID=MALK_03652.t1.e1;Parent=MALK_03652.t1
contig013	AUGUSTUS	gene	225723	227063	0.96	-	.	ID=MALK_03653;prediction_source=braker_MRET:g3965.t1
contig013	AUGUSTUS	CDS	225723	227063	0.96	-	0	ID=MALK_03653.t1.c1;Parent=MALK_03653.t1
contig013	AUGUSTUS	mRNA	225723	227063	0.96	-	.	ID=MALK_03653.t1;Parent=MALK_03653
contig013	AUGUSTUS	exon	225723	227063	.	-	.	ID=MALK_03653.t1.e1;Parent=MALK_03653.t1
contig013	maker	gene	227141	228313	.	-	.	ID=MALK_03654;prediction_source=maker_MRET:augustus_masked-contig013-processed-gene-1.64-mRNA-1
contig013	maker	CDS	227141	228313	.	-	0	ID=MALK_03654.t1.c1;Parent=MALK_03654.t1
contig013	maker	mRNA	227141	228313	.	-	.	ID=MALK_03654.t1;Parent=MALK_03654
contig013	maker	exon	227141	228313	.	-	.	ID=MALK_03654.t1.e1;Parent=MALK_03654.t1
contig013	maker	gene	228585	230439	.	-	.	ID=MALK_03655;prediction_source=maker_MRET:augustus_masked-contig013-processed-gene-1.65-mRNA-1
contig013	maker	CDS	230385	230439	.	-	0	ID=MALK_03655.t1.c1;Parent=MALK_03655.t1
contig013	maker	CDS	229138	230260	.	-	0	ID=MALK_03655.t1.c2;Parent=MALK_03655.t1
contig013	maker	CDS	228585	229044	.	-	0	ID=MALK_03655.t1.c3;Parent=MALK_03655.t1
contig013	maker	mRNA	228585	230439	.	-	.	ID=MALK_03655.t1;Parent=MALK_03655
contig013	maker	exon	230385	230439	.	-	.	ID=MALK_03655.t1.e1;Parent=MALK_03655.t1
contig013	maker	exon	229138	230260	.	-	.	ID=MALK_03655.t1.e2;Parent=MALK_03655.t1
contig013	maker	exon	228585	229044	.	-	.	ID=MALK_03655.t1.e3;Parent=MALK_03655.t1
contig013	maker	gene	230717	232117	.	-	.	ID=MALK_03656;prediction_source=maker_MRET:augustus_masked-contig013-processed-gene-1.66-mRNA-1
contig013	maker	CDS	230717	232117	.	-	0	ID=MALK_03656.t1.c1;Parent=MALK_03656.t1
contig013	maker	mRNA	230717	232117	.	-	.	ID=MALK_03656.t1;Parent=MALK_03656
contig013	maker	exon	230717	232117	.	-	.	ID=MALK_03656.t1.e1;Parent=MALK_03656.t1
contig013	AUGUSTUS	gene	232116	232805	1	+	.	ID=MALK_03657;prediction_source=braker_MRET:g3970.t1
contig013	AUGUSTUS	CDS	232116	232805	1	+	0	ID=MALK_03657.t1.c1;Parent=MALK_03657.t1
contig013	AUGUSTUS	mRNA	232116	232805	1	+	.	ID=MALK_03657.t1;Parent=MALK_03657
contig013	AUGUSTUS	exon	232116	232805	.	+	.	ID=MALK_03657.t1.e1;Parent=MALK_03657.t1
contig013	AUGUSTUS	gene	232812	235055	1	-	.	ID=MALK_03658;prediction_source=augustus:contig013.g3086.t1
contig013	AUGUSTUS	CDS	232812	235055	1	-	0	ID=MALK_03658.t1.c1;Parent=MALK_03658.t1
contig013	AUGUSTUS	mRNA	232812	235055	1	-	.	ID=MALK_03658.t1;Parent=MALK_03658
contig013	AUGUSTUS	exon	232812	235055	1	-	.	ID=MALK_03658.t1.e1;Parent=MALK_03658.t1
contig013	AUGUSTUS	gene	235164	236228	0.46	-	.	ID=MALK_03659;prediction_source=augustus:contig013.g3088.t1
contig013	AUGUSTUS	CDS	235164	236228	0.46	-	0	ID=MALK_03659.t1.c1;Parent=MALK_03659.t1
contig013	AUGUSTUS	mRNA	235164	236228	0.46	-	.	ID=MALK_03659.t1;Parent=MALK_03659
contig013	AUGUSTUS	exon	235164	236228	0.46	-	.	ID=MALK_03659.t1.e1;Parent=MALK_03659.t1
contig013	AUGUSTUS	gene	236325	237026	0.56	+	.	ID=MALK_03660;prediction_source=augustus:contig013.g3089.t1
contig013	AUGUSTUS	CDS	236325	237026	0.56	+	0	ID=MALK_03660.t1.c1;Parent=MALK_03660.t1
contig013	AUGUSTUS	mRNA	236325	237026	0.56	+	.	ID=MALK_03660.t1;Parent=MALK_03660
contig013	AUGUSTUS	exon	236325	237026	0.56	+	.	ID=MALK_03660.t1.e1;Parent=MALK_03660.t1
contig013	AUGUSTUS	gene	237023	238444	0.35	-	.	ID=MALK_03661;prediction_source=augustus:contig013.g3090.t1
contig013	AUGUSTUS	CDS	237023	238444	0.35	-	0	ID=MALK_03661.t1.c1;Parent=MALK_03661.t1
contig013	AUGUSTUS	mRNA	237023	238444	0.35	-	.	ID=MALK_03661.t1;Parent=MALK_03661
contig013	AUGUSTUS	exon	237023	238444	0.35	-	.	ID=MALK_03661.t1.e1;Parent=MALK_03661.t1
contig013	AUGUSTUS	gene	238721	240892	0.62	+	.	ID=MALK_03662;prediction_source=augustus:contig013.g3091.t1
contig013	AUGUSTUS	CDS	238721	240892	0.62	+	0	ID=MALK_03662.t1.c1;Parent=MALK_03662.t1

contig013	AUGUSTUS	mRNA	238721	240892	0.62	+	.	ID=MALK_03662.t1;Parent=MALK_03662
contig013	AUGUSTUS	exon	238721	240892	0.62	+	.	ID=MALK_03662.t1.e1;Parent=MALK_03662.t1
contig013	AUGUSTUS	gene	240855	242381	0.44	-	.	ID=MALK_03663;prediction_source=augustus:contig013.g3092.t1
contig013	AUGUSTUS	CDS	240855	242381	0.44	-	0	ID=MALK_03663.t1.c1;Parent=MALK_03663.t1
contig013	AUGUSTUS	mRNA	240855	242381	0.44	-	.	ID=MALK_03663.t1;Parent=MALK_03663
contig013	AUGUSTUS	exon	240855	242381	0.44	-	.	ID=MALK_03663.t1.e1;Parent=MALK_03663.t1
contig013	AUGUSTUS	gene	242369	243100	1	+	.	ID=MALK_03664;prediction_source=braker_MRET:g3977.t1
contig013	AUGUSTUS	CDS	242369	243100	1	+	0	ID=MALK_03664.t1.c1;Parent=MALK_03664.t1
contig013	AUGUSTUS	mRNA	242369	243100	1	+	.	ID=MALK_03664.t1;Parent=MALK_03664
contig013	AUGUSTUS	exon	242369	243100	.	+	.	ID=MALK_03664.t1.e1;Parent=MALK_03664.t1
contig013	maker	gene	243108	243575	.	-	.	ID=MALK_03665;prediction_source=maker_MRET:augustus_masked-contig013-processed-gene-1.70-mRNA-1
contig013	maker	CDS	243108	243575	.	-	0	ID=MALK_03665.t1.c1;Parent=MALK_03665.t1
contig013	maker	mRNA	243108	243575	.	-	.	ID=MALK_03665.t1;Parent=MALK_03665
contig013	maker	exon	243108	243575	.	-	.	ID=MALK_03665.t1.e1;Parent=MALK_03665.t1
contig013	AUGUSTUS	gene	243753	244578	0.82	+	.	ID=MALK_03666;prediction_source=braker_MRET:g3979.t1
contig013	AUGUSTUS	CDS	243753	244423	0.82	+	0	ID=MALK_03666.t1.c1;Parent=MALK_03666.t1
contig013	AUGUSTUS	CDS	244476	244578	0.82	+	0	ID=MALK_03666.t1.c2;Parent=MALK_03666.t1
contig013	AUGUSTUS	mRNA	243753	244578	0.82	+	.	ID=MALK_03666.t1;Parent=MALK_03666
contig013	AUGUSTUS	exon	243753	244423	.	+	.	ID=MALK_03666.t1.e1;Parent=MALK_03666.t1
contig013	AUGUSTUS	exon	244476	244578	.	+	.	ID=MALK_03666.t1.e2;Parent=MALK_03666.t1
contig013	AUGUSTUS	gene	244598	245476	0.86	-	.	ID=MALK_03667;prediction_source=braker_MRET:g3980.t1
contig013	AUGUSTUS	CDS	244598	245476	0.86	-	0	ID=MALK_03667.t1.c1;Parent=MALK_03667.t1
contig013	AUGUSTUS	mRNA	244598	245476	0.86	-	.	ID=MALK_03667.t1;Parent=MALK_03667
contig013	AUGUSTUS	exon	244598	245476	.	-	.	ID=MALK_03667.t1.e1;Parent=MALK_03667.t1
contig013	AUGUSTUS	gene	245632	246282	1	+	.	ID=MALK_03668;prediction_source=augustus:contig013.g3095.t1
contig013	AUGUSTUS	CDS	245632	246282	1	+	0	ID=MALK_03668.t1.c1;Parent=MALK_03668.t1
contig013	AUGUSTUS	mRNA	245632	246282	1	+	.	ID=MALK_03668.t1;Parent=MALK_03668
contig013	AUGUSTUS	exon	245632	246282	1	+	.	ID=MALK_03668.t1.e1;Parent=MALK_03668.t1
contig013	AUGUSTUS	gene	246572	247135	0.99	-	.	ID=MALK_03669;prediction_source=braker_MRET:g3982.t1
contig013	AUGUSTUS	CDS	246970	247135	1	-	0	ID=MALK_03669.t1.c3;Parent=MALK_03669.t1
contig013	AUGUSTUS	CDS	246707	246919	1	-	0	ID=MALK_03669.t1.c2;Parent=MALK_03669.t1
contig013	AUGUSTUS	CDS	246572	246669	1	-	0	ID=MALK_03669.t1.c1;Parent=MALK_03669.t1
contig013	AUGUSTUS	mRNA	246572	247135	0.99	-	.	ID=MALK_03669.t1;Parent=MALK_03669
contig013	AUGUSTUS	exon	246970	247135	.	-	.	ID=MALK_03669.t1.e3;Parent=MALK_03669.t1
contig013	AUGUSTUS	exon	246707	246919	.	-	.	ID=MALK_03669.t1.e2;Parent=MALK_03669.t1
contig013	AUGUSTUS	exon	246572	246669	.	-	.	ID=MALK_03669.t1.e1;Parent=MALK_03669.t1
contig013	maker	gene	247438	247884	.	+	.	ID=MALK_03670;prediction_source=maker_MRET:augustus_masked-contig013-processed-gene-1.33-mRNA-1
contig013	maker	CDS	247438	247884	.	+	0	ID=MALK_03670.t1.c1;Parent=MALK_03670.t1
contig013	maker	mRNA	247438	247884	.	+	.	ID=MALK_03670.t1;Parent=MALK_03670
contig013	maker	exon	247438	247884	.	+	.	ID=MALK_03670.t1.e1;Parent=MALK_03670.t1
contig013	AUGUSTUS	gene	248826	250295	0.97	+	.	ID=MALK_03671;prediction_source=braker_MRET:g3984.t1
contig013	AUGUSTUS	CDS	248826	250295	0.97	+	0	ID=MALK_03671.t1.c1;Parent=MALK_03671.t1
contig013	AUGUSTUS	mRNA	248826	250295	0.97	+	.	ID=MALK_03671.t1;Parent=MALK_03671

contig013	AUGUSTUS	exon	248826	250295	.	+	.	ID=MALK_03671.t1.e1;Parent=MALK_03671.t1
contig013	AUGUSTUS	gene	250332	251267	0.79	+	.	ID=MALK_03672;prediction_source=braker_MRET:g3985.t1
contig013	AUGUSTUS	CDS	250332	251267	0.79	+	0	ID=MALK_03672.t1.c1;Parent=MALK_03672.t1
contig013	AUGUSTUS	mRNA	250332	251267	0.79	+	.	ID=MALK_03672.t1;Parent=MALK_03672
contig013	AUGUSTUS	exon	250332	251267	.	+	.	ID=MALK_03672.t1.e1;Parent=MALK_03672.t1
contig013	AUGUSTUS	gene	251328	252637	0.78	-	.	ID=MALK_03673;prediction_source=braker_MRET:g3986.t1
contig013	AUGUSTUS	CDS	251389	252637	0.78	-	0	ID=MALK_03673.t1.c2;Parent=MALK_03673.t1
contig013	AUGUSTUS	CDS	251328	251350	0.78	-	0	ID=MALK_03673.t1.c1;Parent=MALK_03673.t1
contig013	AUGUSTUS	mRNA	251328	252637	0.78	-	.	ID=MALK_03673.t1;Parent=MALK_03673
contig013	AUGUSTUS	exon	251389	252637	.	-	.	ID=MALK_03673.t1.e2;Parent=MALK_03673.t1
contig013	AUGUSTUS	exon	251328	251350	.	-	.	ID=MALK_03673.t1.e1;Parent=MALK_03673.t1
contig013	AUGUSTUS	gene	252669	256424	1	-	.	ID=MALK_03674;prediction_source=braker_MRET:g3987.t1
contig013	AUGUSTUS	CDS	252669	256424	1	-	0	ID=MALK_03674.t1.c1;Parent=MALK_03674.t1
contig013	AUGUSTUS	mRNA	252669	256424	1	-	.	ID=MALK_03674.t1;Parent=MALK_03674
contig013	AUGUSTUS	exon	252669	256424	.	-	.	ID=MALK_03674.t1.e1;Parent=MALK_03674.t1
contig013	AUGUSTUS	gene	256580	259597	0.48	-	.	ID=MALK_03675;prediction_source=augustus:contig013.g3100.t1
contig013	AUGUSTUS	CDS	256580	259597	0.48	-	0	ID=MALK_03675.t1.c1;Parent=MALK_03675.t1
contig013	AUGUSTUS	mRNA	256580	259597	0.48	-	.	ID=MALK_03675.t1;Parent=MALK_03675
contig013	AUGUSTUS	exon	256580	259597	0.48	-	.	ID=MALK_03675.t1.e1;Parent=MALK_03675.t1
contig013	maker	gene	259739	260722	.	+	.	ID=MALK_03676;prediction_source=maker_MRET:augustus_masked-contig013-processed-gene-1.34-mRNA-1
contig013	maker	CDS	259739	260722	.	+	0	ID=MALK_03676.t1.c1;Parent=MALK_03676.t1
contig013	maker	mRNA	259739	260722	.	+	.	ID=MALK_03676.t1;Parent=MALK_03676
contig013	maker	exon	259739	260722	.	+	.	ID=MALK_03676.t1.e1;Parent=MALK_03676.t1
contig013	AUGUSTUS	gene	260746	262458	1	+	.	ID=MALK_03677;prediction_source=braker_MRET:g3990.t1
contig013	AUGUSTUS	CDS	260746	262458	1	+	0	ID=MALK_03677.t1.c1;Parent=MALK_03677.t1
contig013	AUGUSTUS	mRNA	260746	262458	1	+	.	ID=MALK_03677.t1;Parent=MALK_03677
contig013	AUGUSTUS	exon	260746	262458	.	+	.	ID=MALK_03677.t1.e1;Parent=MALK_03677.t1
contig013	AUGUSTUS	gene	262497	263122	0.93	+	.	ID=MALK_03678;prediction_source=braker_MRET:g3991.t1
contig013	AUGUSTUS	CDS	262497	263122	0.93	+	0	ID=MALK_03678.t1.c1;Parent=MALK_03678.t1
contig013	AUGUSTUS	mRNA	262497	263122	0.93	+	.	ID=MALK_03678.t1;Parent=MALK_03678
contig013	AUGUSTUS	exon	262497	263122	.	+	.	ID=MALK_03678.t1.e1;Parent=MALK_03678.t1
contig014	annotation	remark	1	79918	.	+	.	gff-version=3
contig014	AUGUSTUS	gene	2	855	0.8	+	.	ID=MALK_03679;prediction_source=braker_MRET:g355.t1
contig014	AUGUSTUS	CDS	2	855	0.8	+	2	ID=MALK_03679.t1.c1;Parent=MALK_03679.t1
contig014	AUGUSTUS	mRNA	2	855	0.8	+	.	ID=MALK_03679.t1;Parent=MALK_03679
contig014	AUGUSTUS	exon	2	855	.	+	.	ID=MALK_03679.t1.e1;Parent=MALK_03679.t1
contig014	AUGUSTUS	gene	945	2866	0.21	-	.	ID=MALK_03680;prediction_source=braker_MRET:g356.t1
contig014	AUGUSTUS	CDS	2809	2866	0.61	-	0	ID=MALK_03680.t1.c4;Parent=MALK_03680.t1
contig014	AUGUSTUS	CDS	1906	2675	0.61	-	0	ID=MALK_03680.t1.c3;Parent=MALK_03680.t1
contig014	AUGUSTUS	CDS	1010	1867	0.61	-	0	ID=MALK_03680.t1.c2;Parent=MALK_03680.t1
contig014	AUGUSTUS	CDS	945	974	0.61	-	0	ID=MALK_03680.t1.c1;Parent=MALK_03680.t1
contig014	AUGUSTUS	mRNA	945	2866	0.21	-	.	ID=MALK_03680.t1;Parent=MALK_03680
contig014	AUGUSTUS	exon	2809	2866	.	-	.	ID=MALK_03680.t1.e4;Parent=MALK_03680.t1

contig014	AUGUSTUS	exon	1906	2675	.	-	.	ID=MALK_03680.t1.e3;Parent=MALK_03680.t1
contig014	AUGUSTUS	exon	1010	1867	.	-	.	ID=MALK_03680.t1.e2;Parent=MALK_03680.t1
contig014	AUGUSTUS	exon	945	974	.	-	.	ID=MALK_03680.t1.e1;Parent=MALK_03680.t1
contig014	AUGUSTUS	gene	2992	3687	0.97	-	.	ID=MALK_03681;prediction_source=braker_MRET:g357.t1
contig014	AUGUSTUS	CDS	2992	3687	0.97	-	0	ID=MALK_03681.t1.c1;Parent=MALK_03681.t1
contig014	AUGUSTUS	mRNA	2992	3687	0.97	-	.	ID=MALK_03681.t1;Parent=MALK_03681
contig014	AUGUSTUS	exon	2992	3687	.	-	.	ID=MALK_03681.t1.e1;Parent=MALK_03681.t1
contig014	maker	gene	3713	6934	.	-	.	ID=MALK_03682;prediction_source=maker_MRET:augustus_masked-contig014-processed-gene-0.50-mRNA-1
contig014	maker	CDS	3713	6934	.	-	0	ID=MALK_03682.t1.c1;Parent=MALK_03682.t1
contig014	maker	mRNA	3713	6934	.	-	.	ID=MALK_03682.t1;Parent=MALK_03682
contig014	maker	exon	3713	6934	.	-	.	ID=MALK_03682.t1.e1;Parent=MALK_03682.t1
contig014	AUGUSTUS	gene	7677	8770	0.62	-	.	ID=MALK_03683;prediction_source=braker_MRET:g359.t1
contig014	AUGUSTUS	CDS	8336	8770	0.82	-	0	ID=MALK_03683.t1.c3;Parent=MALK_03683.t1
contig014	AUGUSTUS	CDS	7980	8058	0.82	-	0	ID=MALK_03683.t1.c2;Parent=MALK_03683.t1
contig014	AUGUSTUS	CDS	7677	7948	0.82	-	0	ID=MALK_03683.t1.c1;Parent=MALK_03683.t1
contig014	AUGUSTUS	mRNA	7677	8770	0.62	-	.	ID=MALK_03683.t1;Parent=MALK_03683
contig014	AUGUSTUS	exon	8336	8770	.	-	.	ID=MALK_03683.t1.e3;Parent=MALK_03683.t1
contig014	AUGUSTUS	exon	7980	8058	.	-	.	ID=MALK_03683.t1.e2;Parent=MALK_03683.t1
contig014	AUGUSTUS	exon	7677	7948	.	-	.	ID=MALK_03683.t1.e1;Parent=MALK_03683.t1
contig014	maker	gene	9351	11312	.	-	.	ID=MALK_03684;prediction_source=maker_MRET:augustus_masked-contig014-processed-gene-0.51-mRNA-1
contig014	maker	CDS	9351	11312	.	-	0	ID=MALK_03684.t1.c1;Parent=MALK_03684.t1
contig014	maker	mRNA	9351	11312	.	-	.	ID=MALK_03684.t1;Parent=MALK_03684
contig014	maker	exon	9351	11312	.	-	.	ID=MALK_03684.t1.e1;Parent=MALK_03684.t1
contig014	AUGUSTUS	gene	11559	13994	0.79	+	.	ID=MALK_03685;prediction_source=augustus:contig014.g1886.t1
contig014	AUGUSTUS	CDS	11559	13994	0.79	+	0	ID=MALK_03685.t1.c1;Parent=MALK_03685.t1
contig014	AUGUSTUS	mRNA	11559	13994	0.79	+	.	ID=MALK_03685.t1;Parent=MALK_03685
contig014	AUGUSTUS	exon	11559	13994	0.79	+	.	ID=MALK_03685.t1.e1;Parent=MALK_03685.t1
contig014	AUGUSTUS	gene	14018	15778	0.66	-	.	ID=MALK_03686;prediction_source=braker_MRET:g362.t1
contig014	AUGUSTUS	CDS	14018	15778	0.66	-	0	ID=MALK_03686.t1.c1;Parent=MALK_03686.t1
contig014	AUGUSTUS	mRNA	14018	15778	0.66	-	.	ID=MALK_03686.t1;Parent=MALK_03686
contig014	AUGUSTUS	exon	14018	15778	.	-	.	ID=MALK_03686.t1.e1;Parent=MALK_03686.t1
contig014	AUGUSTUS	gene	15840	18776	1	-	.	ID=MALK_03687;prediction_source=augustus:contig014.g1887.t1
contig014	AUGUSTUS	CDS	15840	18776	1	-	0	ID=MALK_03687.t1.c1;Parent=MALK_03687.t1
contig014	AUGUSTUS	mRNA	15840	18776	1	-	.	ID=MALK_03687.t1;Parent=MALK_03687
contig014	AUGUSTUS	exon	15840	18776	1	-	.	ID=MALK_03687.t1.e1;Parent=MALK_03687.t1
contig014	maker	gene	18853	19539	.	-	.	ID=MALK_03688;prediction_source=maker_MRET:augustus_masked-contig014-processed-gene-0.53-mRNA-1
contig014	maker	CDS	18853	19539	.	-	0	ID=MALK_03688.t1.c1;Parent=MALK_03688.t1
contig014	maker	mRNA	18853	19539	.	-	.	ID=MALK_03688.t1;Parent=MALK_03688
contig014	maker	exon	18853	19539	.	-	.	ID=MALK_03688.t1.e1;Parent=MALK_03688.t1
contig014	AUGUSTUS	gene	20532	23187	0.24	+	.	ID=MALK_03689;prediction_source=augustus:contig014.g1890.t1
contig014	AUGUSTUS	CDS	20532	20573	0.27	+	0	ID=MALK_03689.t1.c1;Parent=MALK_03689.t1
contig014	AUGUSTUS	CDS	20656	23187	0.27	+	0	ID=MALK_03689.t1.c2;Parent=MALK_03689.t1
contig014	AUGUSTUS	mRNA	20532	23187	0.24	+	.	ID=MALK_03689.t1;Parent=MALK_03689

contig014	AUGUSTUS	exon	20532	20573	0.27	+	.	ID=MALK_03689.t1.e1;Parent=MALK_03689.t1
contig014	AUGUSTUS	exon	20656	23187	0.27	+	.	ID=MALK_03689.t1.e2;Parent=MALK_03689.t1
contig014	AUGUSTUS	gene	23208	24782	0.41	-	.	ID=MALK_03690;prediction_source=augustus:contig014.g1892.t1
contig014	AUGUSTUS	CDS	24543	24782	0.8	-	0	ID=MALK_03690.t1.c2;Parent=MALK_03690.t1
contig014	AUGUSTUS	CDS	23208	24476	0.8	-	0	ID=MALK_03690.t1.c1;Parent=MALK_03690.t1
contig014	AUGUSTUS	mRNA	23208	24782	0.41	-	.	ID=MALK_03690.t1;Parent=MALK_03690
contig014	AUGUSTUS	exon	24543	24782	0.8	-	.	ID=MALK_03690.t1.e2;Parent=MALK_03690.t1
contig014	AUGUSTUS	exon	23208	24476	0.8	-	.	ID=MALK_03690.t1.e1;Parent=MALK_03690.t1
contig014	AUGUSTUS	gene	27294	27572	0.25	-	.	ID=MALK_03691;prediction_source=augustus:contig014.g1893.t1
contig014	AUGUSTUS	CDS	27294	27572	0.25	-	0	ID=MALK_03691.t1.c1;Parent=MALK_03691.t1
contig014	AUGUSTUS	mRNA	27294	27572	0.25	-	.	ID=MALK_03691.t1;Parent=MALK_03691
contig014	AUGUSTUS	exon	27294	27572	0.25	-	.	ID=MALK_03691.t1.e1;Parent=MALK_03691.t1
contig014	AUGUSTUS	gene	27722	28742	0.23	-	.	ID=MALK_03692;prediction_source=augustus:contig014.g1894.t1
contig014	AUGUSTUS	CDS	28462	28742	0.52	-	0	ID=MALK_03692.t1.c3;Parent=MALK_03692.t1
contig014	AUGUSTUS	CDS	28020	28333	0.52	-	0	ID=MALK_03692.t1.c2;Parent=MALK_03692.t1
contig014	AUGUSTUS	CDS	27722	27957	0.52	-	0	ID=MALK_03692.t1.c1;Parent=MALK_03692.t1
contig014	AUGUSTUS	mRNA	27722	28742	0.23	-	.	ID=MALK_03692.t1;Parent=MALK_03692
contig014	AUGUSTUS	exon	28462	28742	0.52	-	.	ID=MALK_03692.t1.e3;Parent=MALK_03692.t1
contig014	AUGUSTUS	exon	28020	28333	0.52	-	.	ID=MALK_03692.t1.e2;Parent=MALK_03692.t1
contig014	AUGUSTUS	exon	27722	27957	0.52	-	.	ID=MALK_03692.t1.e1;Parent=MALK_03692.t1
contig014	maker	gene	28816	29940	.	-	.	ID=MALK_03693;prediction_source=maker_MRET:augustus_masked-contig014-processed-gene-0.56-mRNA-1
contig014	maker	CDS	28816	29940	.	-	0	ID=MALK_03693.t1.c1;Parent=MALK_03693.t1
contig014	maker	mRNA	28816	29940	.	-	.	ID=MALK_03693.t1;Parent=MALK_03693
contig014	maker	exon	28816	29940	.	-	.	ID=MALK_03693.t1.e1;Parent=MALK_03693.t1
contig014	maker	gene	30063	32537	.	+	.	ID=MALK_03694;prediction_source=maker_MRET:augustus_masked-contig014-processed-gene-0.39-mRNA-1
contig014	maker	CDS	30063	32537	.	+	0	ID=MALK_03694.t1.c1;Parent=MALK_03694.t1
contig014	maker	mRNA	30063	32537	.	+	.	ID=MALK_03694.t1;Parent=MALK_03694
contig014	maker	exon	30063	32537	.	+	.	ID=MALK_03694.t1.e1;Parent=MALK_03694.t1
contig014	AUGUSTUS	gene	32829	34074	0.68	+	.	ID=MALK_03695;prediction_source=braker_MRET:g370.t1
contig014	AUGUSTUS	CDS	32829	33879	1	+	0	ID=MALK_03695.t1.c1;Parent=MALK_03695.t1
contig014	AUGUSTUS	CDS	33908	34074	1	+	0	ID=MALK_03695.t1.c2;Parent=MALK_03695.t1
contig014	AUGUSTUS	mRNA	32829	34074	0.68	+	.	ID=MALK_03695.t1;Parent=MALK_03695
contig014	AUGUSTUS	exon	32829	33879	.	+	.	ID=MALK_03695.t1.e1;Parent=MALK_03695.t1
contig014	AUGUSTUS	exon	33908	34074	.	+	.	ID=MALK_03695.t1.e2;Parent=MALK_03695.t1
contig014	maker	gene	34263	35594	.	+	.	ID=MALK_03696;prediction_source=maker_MRET:augustus_masked-contig014-processed-gene-0.41-mRNA-1
contig014	maker	CDS	34263	35594	.	+	0	ID=MALK_03696.t1.c1;Parent=MALK_03696.t1
contig014	maker	mRNA	34263	35594	.	+	.	ID=MALK_03696.t1;Parent=MALK_03696
contig014	maker	exon	34263	35594	.	+	.	ID=MALK_03696.t1.e1;Parent=MALK_03696.t1
contig014	AUGUSTUS	gene	35764	36401	0.33	+	.	ID=MALK_03697;prediction_source=braker_MRET:g372.t1
contig014	AUGUSTUS	CDS	35764	35819	0.93	+	0	ID=MALK_03697.t1.c1;Parent=MALK_03697.t1
contig014	AUGUSTUS	CDS	35852	35960	0.93	+	0	ID=MALK_03697.t1.c2;Parent=MALK_03697.t1
contig014	AUGUSTUS	CDS	35988	36401	0.93	+	0	ID=MALK_03697.t1.c3;Parent=MALK_03697.t1
contig014	AUGUSTUS	mRNA	35764	36401	0.33	+	.	ID=MALK_03697.t1;Parent=MALK_03697

contig014	AUGUSTUS	exon	35764	35819	.	+	.	ID=MALK_03697.t1.e1;Parent=MALK_03697.t1
contig014	AUGUSTUS	exon	35852	35960	.	+	.	ID=MALK_03697.t1.e2;Parent=MALK_03697.t1
contig014	AUGUSTUS	exon	35988	36401	.	+	.	ID=MALK_03697.t1.e3;Parent=MALK_03697.t1
contig014	AUGUSTUS	gene	36657	37496	0.55	-	.	ID=MALK_03698;prediction_source=augustus:contig014.g1899.t1
contig014	AUGUSTUS	CDS	36657	37496	0.55	-	0	ID=MALK_03698.t1.c1;Parent=MALK_03698.t1
contig014	AUGUSTUS	mRNA	36657	37496	0.55	-	.	ID=MALK_03698.t1;Parent=MALK_03698
contig014	AUGUSTUS	exon	36657	37496	0.55	-	.	ID=MALK_03698.t1.e1;Parent=MALK_03698.t1
contig014	AUGUSTUS	gene	37653	42122	0.94	-	.	ID=MALK_03699;prediction_source=augustus:contig014.g1900.t1
contig014	AUGUSTUS	CDS	37653	42122	0.94	-	0	ID=MALK_03699.t1.c1;Parent=MALK_03699.t1
contig014	AUGUSTUS	mRNA	37653	42122	0.94	-	.	ID=MALK_03699.t1;Parent=MALK_03699
contig014	AUGUSTUS	exon	37653	42122	0.94	-	.	ID=MALK_03699.t1.e1;Parent=MALK_03699.t1
contig014	maker	gene	42385	43572	.	+	.	ID=MALK_03700;prediction_source=maker_MRET:augustus_masked-contig014-processed-gene-0.42-mRNA-1
contig014	maker	CDS	42385	43572	.	+	0	ID=MALK_03700.t1.c1;Parent=MALK_03700.t1
contig014	maker	mRNA	42385	43572	.	+	.	ID=MALK_03700.t1;Parent=MALK_03700
contig014	maker	exon	42385	43572	.	+	.	ID=MALK_03700.t1.e1;Parent=MALK_03700.t1
contig014	AUGUSTUS	gene	43622	44173	0.87	-	.	ID=MALK_03701;prediction_source=augustus:contig014.g1903.t1
contig014	AUGUSTUS	CDS	43622	44173	0.87	-	0	ID=MALK_03701.t1.c1;Parent=MALK_03701.t1
contig014	AUGUSTUS	mRNA	43622	44173	0.87	-	.	ID=MALK_03701.t1;Parent=MALK_03701
contig014	AUGUSTUS	exon	43622	44173	0.87	-	.	ID=MALK_03701.t1.e1;Parent=MALK_03701.t1
contig014	AUGUSTUS	gene	44367	46103	0.58	+	.	ID=MALK_03702;prediction_source=braker_MRET:g377.t1
contig014	AUGUSTUS	CDS	44367	46103	0.58	+	0	ID=MALK_03702.t1.c1;Parent=MALK_03702.t1
contig014	AUGUSTUS	mRNA	44367	46103	0.58	+	.	ID=MALK_03702.t1;Parent=MALK_03702
contig014	AUGUSTUS	exon	44367	46103	.	+	.	ID=MALK_03702.t1.e1;Parent=MALK_03702.t1
contig014	maker	gene	46114	48510	.	-	.	ID=MALK_03703;prediction_source=maker_MRET:augustus_masked-contig014-processed-gene-0.60-mRNA-1
contig014	maker	CDS	46114	48510	.	-	0	ID=MALK_03703.t1.c1;Parent=MALK_03703.t1
contig014	maker	mRNA	46114	48510	.	-	.	ID=MALK_03703.t1;Parent=MALK_03703
contig014	maker	exon	46114	48510	.	-	.	ID=MALK_03703.t1.e1;Parent=MALK_03703.t1
contig014	maker	gene	48648	49394	.	+	.	ID=MALK_03704;prediction_source=maker_MRET:augustus_masked-contig014-processed-gene-0.44-mRNA-1
contig014	maker	CDS	48648	49394	.	+	0	ID=MALK_03704.t1.c1;Parent=MALK_03704.t1
contig014	maker	mRNA	48648	49394	.	+	.	ID=MALK_03704.t1;Parent=MALK_03704
contig014	maker	exon	48648	49394	.	+	.	ID=MALK_03704.t1.e1;Parent=MALK_03704.t1
contig014	AUGUSTUS	gene	49660	51605	0.79	+	.	ID=MALK_03705;prediction_source=braker_MRET:g380.t1
contig014	AUGUSTUS	CDS	49660	51348	0.98	+	0	ID=MALK_03705.t1.c1;Parent=MALK_03705.t1
contig014	AUGUSTUS	CDS	51381	51605	0.98	+	0	ID=MALK_03705.t1.c2;Parent=MALK_03705.t1
contig014	AUGUSTUS	mRNA	49660	51605	0.79	+	.	ID=MALK_03705.t1;Parent=MALK_03705
contig014	AUGUSTUS	exon	49660	51348	.	+	.	ID=MALK_03705.t1.e1;Parent=MALK_03705.t1
contig014	AUGUSTUS	exon	51381	51605	.	+	.	ID=MALK_03705.t1.e2;Parent=MALK_03705.t1
contig014	AUGUSTUS	gene	51630	52298	1	-	.	ID=MALK_03706;prediction_source=braker_MRET:g381.t1
contig014	AUGUSTUS	CDS	51630	52298	1	-	0	ID=MALK_03706.t1.c1;Parent=MALK_03706.t1
contig014	AUGUSTUS	mRNA	51630	52298	1	-	.	ID=MALK_03706.t1;Parent=MALK_03706
contig014	AUGUSTUS	exon	51630	52298	.	-	.	ID=MALK_03706.t1.e1;Parent=MALK_03706.t1
contig014	maker	gene	52478	53662	.	+	.	ID=MALK_03707;prediction_source=maker_MRET:augustus_masked-contig014-processed-gene-0.46-mRNA-1
contig014	maker	CDS	52478	53662	.	+	0	ID=MALK_03707.t1.c1;Parent=MALK_03707.t1

contig014	maker	mRNA	52478	53662	.	+	.	ID=MALK_03707.t1;Parent=MALK_03707
contig014	maker	exon	52478	53662	.	+	.	ID=MALK_03707.t1.e1;Parent=MALK_03707.t1
contig014	AUGUSTUS	gene	53685	55625	0.86	-	.	ID=MALK_03708;prediction_source=augustus:contig014.g1910.t1
contig014	AUGUSTUS	CDS	53685	55625	0.86	-	0	ID=MALK_03708.t1.c1;Parent=MALK_03708.t1
contig014	AUGUSTUS	mRNA	53685	55625	0.86	-	.	ID=MALK_03708.t1;Parent=MALK_03708
contig014	AUGUSTUS	exon	53685	55625	0.86	-	.	ID=MALK_03708.t1.e1;Parent=MALK_03708.t1
contig014	AUGUSTUS	gene	55787	58330	0.98	+	.	ID=MALK_03709;prediction_source=augustus:contig014.g1911.t1
contig014	AUGUSTUS	CDS	55787	58330	0.98	+	0	ID=MALK_03709.t1.c1;Parent=MALK_03709.t1
contig014	AUGUSTUS	mRNA	55787	58330	0.98	+	.	ID=MALK_03709.t1;Parent=MALK_03709
contig014	AUGUSTUS	exon	55787	58330	0.98	+	.	ID=MALK_03709.t1.e1;Parent=MALK_03709.t1
contig014	AUGUSTUS	gene	59150	60304	0.99	-	.	ID=MALK_03710;prediction_source=augustus:contig014.g1912.t1
contig014	AUGUSTUS	CDS	59150	60304	0.99	-	0	ID=MALK_03710.t1.c1;Parent=MALK_03710.t1
contig014	AUGUSTUS	mRNA	59150	60304	0.99	-	.	ID=MALK_03710.t1;Parent=MALK_03710
contig014	AUGUSTUS	exon	59150	60304	0.99	-	.	ID=MALK_03710.t1.e1;Parent=MALK_03710.t1
contig014	AUGUSTUS	gene	60385	61083	0.94	+	.	ID=MALK_03711;prediction_source=braker_MRET:g386.t1
contig014	AUGUSTUS	CDS	60385	61083	0.94	+	0	ID=MALK_03711.t1.c1;Parent=MALK_03711.t1
contig014	AUGUSTUS	mRNA	60385	61083	0.94	+	.	ID=MALK_03711.t1;Parent=MALK_03711
contig014	AUGUSTUS	exon	60385	61083	.	+	.	ID=MALK_03711.t1.e1;Parent=MALK_03711.t1
contig014	AUGUSTUS	gene	61084	64680	0.65	-	.	ID=MALK_03712;prediction_source=augustus:contig014.g1913.t1
contig014	AUGUSTUS	CDS	61084	64680	0.65	-	0	ID=MALK_03712.t1.c1;Parent=MALK_03712.t1
contig014	AUGUSTUS	mRNA	61084	64680	0.65	-	.	ID=MALK_03712.t1;Parent=MALK_03712
contig014	AUGUSTUS	exon	61084	64680	0.65	-	.	ID=MALK_03712.t1.e1;Parent=MALK_03712.t1
contig014	maker	gene	64759	65322	.	+	.	ID=MALK_03713;prediction_source=maker_MRET:augustus_masked-contig014-processed-gene-0.48-mRNA-1
contig014	maker	CDS	64759	65322	.	+	0	ID=MALK_03713.t1.c1;Parent=MALK_03713.t1
contig014	maker	mRNA	64759	65322	.	+	.	ID=MALK_03713.t1;Parent=MALK_03713
contig014	maker	exon	64759	65322	.	+	.	ID=MALK_03713.t1.e1;Parent=MALK_03713.t1
contig014	maker	gene	65325	65768	.	-	.	ID=MALK_03714;prediction_source=maker_MRET:augustus_masked-contig014-processed-gene-0.64-mRNA-1
contig014	maker	CDS	65325	65768	.	-	0	ID=MALK_03714.t1.c1;Parent=MALK_03714.t1
contig014	maker	mRNA	65325	65768	.	-	.	ID=MALK_03714.t1;Parent=MALK_03714
contig014	maker	exon	65325	65768	.	-	.	ID=MALK_03714.t1.e1;Parent=MALK_03714.t1
contig014	AUGUSTUS	gene	65843	66185	0.99	+	.	ID=MALK_03715;prediction_source=braker_MRET:g390.t1
contig014	AUGUSTUS	CDS	65843	66040	0.99	+	0	ID=MALK_03715.t1.c1;Parent=MALK_03715.t1
contig014	AUGUSTUS	CDS	66075	66185	0.99	+	0	ID=MALK_03715.t1.c2;Parent=MALK_03715.t1
contig014	AUGUSTUS	mRNA	65843	66185	0.99	+	.	ID=MALK_03715.t1;Parent=MALK_03715
contig014	AUGUSTUS	exon	65843	66040	.	+	.	ID=MALK_03715.t1.e1;Parent=MALK_03715.t1
contig014	AUGUSTUS	exon	66075	66185	.	+	.	ID=MALK_03715.t1.e2;Parent=MALK_03715.t1
contig014	AUGUSTUS	gene	66601	68412	0.27	+	.	ID=MALK_03716;prediction_source=augustus:contig014.g1917.t1
contig014	AUGUSTUS	CDS	66601	68412	0.27	+	0	ID=MALK_03716.t1.c1;Parent=MALK_03716.t1
contig014	AUGUSTUS	mRNA	66601	68412	0.27	+	.	ID=MALK_03716.t1;Parent=MALK_03716
contig014	AUGUSTUS	exon	66601	68412	0.27	+	.	ID=MALK_03716.t1.e1;Parent=MALK_03716.t1
contig014	AUGUSTUS	gene	68497	69459	0.37	+	.	ID=MALK_03717;prediction_source=augustus:contig014.g1920.t1
contig014	AUGUSTUS	CDS	68497	69459	0.37	+	0	ID=MALK_03717.t1.c1;Parent=MALK_03717.t1
contig014	AUGUSTUS	mRNA	68497	69459	0.37	+	.	ID=MALK_03717.t1;Parent=MALK_03717

contig014	AUGUSTUS	exon	68497	69459	0.37	+	.	ID=MALK_03717.t1.e1;Parent=MALK_03717.t1
contig014	maker	gene	69476	71656	.	-	.	ID=MALK_03718;prediction_source=maker_MRET:augustus_masked-contig014-processed-gene-0.65-mRNA-1
contig014	maker	CDS	69476	71656	.	-	0	ID=MALK_03718.t1.c1;Parent=MALK_03718.t1
contig014	maker	mRNA	69476	71656	.	-	.	ID=MALK_03718.t1;Parent=MALK_03718
contig014	maker	exon	69476	71656	.	-	.	ID=MALK_03718.t1.e1;Parent=MALK_03718.t1
contig014	AUGUSTUS	gene	71759	74107	0.99	-	.	ID=MALK_03719;prediction_source=augustus:contig014.g1923.t1
contig014	AUGUSTUS	CDS	71759	74107	0.99	-	0	ID=MALK_03719.t1.c1;Parent=MALK_03719.t1
contig014	AUGUSTUS	mRNA	71759	74107	0.99	-	.	ID=MALK_03719.t1;Parent=MALK_03719
contig014	AUGUSTUS	exon	71759	74107	0.99	-	.	ID=MALK_03719.t1.e1;Parent=MALK_03719.t1
contig014	AUGUSTUS	gene	74572	76266	0.99	-	.	ID=MALK_03720;prediction_source=braker_MRET:g395.t1
contig014	AUGUSTUS	CDS	74572	76266	0.99	-	0	ID=MALK_03720.t1.c1;Parent=MALK_03720.t1
contig014	AUGUSTUS	mRNA	74572	76266	0.99	-	.	ID=MALK_03720.t1;Parent=MALK_03720
contig014	AUGUSTUS	exon	74572	76266	.	-	.	ID=MALK_03720.t1.e1;Parent=MALK_03720.t1
contig014	AUGUSTUS	gene	76411	79918	0.77	+	.	ID=MALK_03721;prediction_source=braker_MRET:g396.t1
contig014	AUGUSTUS	CDS	76411	79918	0.77	+	0	ID=MALK_03721.t1.c1;Parent=MALK_03721.t1
contig014	AUGUSTUS	mRNA	76411	79918	0.77	+	.	ID=MALK_03721.t1;Parent=MALK_03721
contig014	AUGUSTUS	exon	76411	79918	.	+	.	ID=MALK_03721.t1.e1;Parent=MALK_03721.t1
contig015	annotation	remark	1	110976	.	+	.	gff-version=3
contig015	maker	gene	533	1411	.	+	.	ID=MALK_03722;prediction_source=maker_MRET:augustus_masked-contig015-processed-gene-0.37-mRNA-1
contig015	maker	CDS	533	1411	.	+	0	ID=MALK_03722.t1.c1;Parent=MALK_03722.t1
contig015	maker	mRNA	533	1411	.	+	.	ID=MALK_03722.t1;Parent=MALK_03722
contig015	maker	exon	533	1411	.	+	.	ID=MALK_03722.t1.e1;Parent=MALK_03722.t1
contig015	AUGUSTUS	gene	1408	1938	1	-	.	ID=MALK_03723;prediction_source=braker_MRET:g2349.t1
contig015	AUGUSTUS	CDS	1408	1938	1	-	0	ID=MALK_03723.t1.c1;Parent=MALK_03723.t1
contig015	AUGUSTUS	mRNA	1408	1938	1	-	.	ID=MALK_03723.t1;Parent=MALK_03723
contig015	AUGUSTUS	exon	1408	1938	.	-	.	ID=MALK_03723.t1.e1;Parent=MALK_03723.t1
contig015	maker	gene	1962	3407	.	-	.	ID=MALK_03724;prediction_source=maker_MRET:augustus_masked-contig015-processed-gene-0.67-mRNA-1
contig015	maker	CDS	1962	3407	.	-	0	ID=MALK_03724.t1.c1;Parent=MALK_03724.t1
contig015	maker	mRNA	1962	3407	.	-	.	ID=MALK_03724.t1;Parent=MALK_03724
contig015	maker	exon	1962	3407	.	-	.	ID=MALK_03724.t1.e1;Parent=MALK_03724.t1
contig015	maker	gene	3778	6957	.	+	.	ID=MALK_03725;prediction_source=maker_MRET:augustus_masked-contig015-processed-gene-0.38-mRNA-1
contig015	maker	CDS	3778	6957	.	+	0	ID=MALK_03725.t1.c1;Parent=MALK_03725.t1
contig015	maker	mRNA	3778	6957	.	+	.	ID=MALK_03725.t1;Parent=MALK_03725
contig015	maker	exon	3778	6957	.	+	.	ID=MALK_03725.t1.e1;Parent=MALK_03725.t1
contig015	maker	gene	6995	7933	.	-	.	ID=MALK_03726;prediction_source=maker_MRET:augustus_masked-contig015-processed-gene-0.68-mRNA-1
contig015	maker	CDS	6995	7933	.	-	0	ID=MALK_03726.t1.c1;Parent=MALK_03726.t1
contig015	maker	mRNA	6995	7933	.	-	.	ID=MALK_03726.t1;Parent=MALK_03726
contig015	maker	exon	6995	7933	.	-	.	ID=MALK_03726.t1.e1;Parent=MALK_03726.t1
contig015	maker	gene	9265	10038	.	-	.	ID=MALK_03727;prediction_source=maker_MRET:augustus_masked-contig015-processed-gene-0.69-mRNA-1
contig015	maker	CDS	9265	10038	.	-	0	ID=MALK_03727.t1.c1;Parent=MALK_03727.t1
contig015	maker	mRNA	9265	10038	.	-	.	ID=MALK_03727.t1;Parent=MALK_03727
contig015	maker	exon	9265	10038	.	-	.	ID=MALK_03727.t1.e1;Parent=MALK_03727.t1
contig015	maker	gene	10106	11212	.	+	.	ID=MALK_03728;prediction_source=maker_MRET:augustus_masked-contig015-processed-gene-0.39-mRNA-1

contig015	maker	CDS	10106	11212	.	+	0	ID=MALK_03728.t1.c1;Parent=MALK_03728.t1
contig015	maker	mRNA	10106	11212	.	+	.	ID=MALK_03728.t1;Parent=MALK_03728
contig015	maker	exon	10106	11212	.	+	.	ID=MALK_03728.t1.e1;Parent=MALK_03728.t1
contig015	AUGUSTUS	gene	11253	13655	1	-	.	ID=MALK_03729;prediction_source=augustus:contig015.g1405.t1
contig015	AUGUSTUS	CDS	11253	13655	1	-	0	ID=MALK_03729.t1.c1;Parent=MALK_03729.t1
contig015	AUGUSTUS	mRNA	11253	13655	1	-	.	ID=MALK_03729.t1;Parent=MALK_03729
contig015	AUGUSTUS	exon	11253	13655	1	-	.	ID=MALK_03729.t1.e1;Parent=MALK_03729.t1
contig015	maker	gene	13879	15144	.	+	.	ID=MALK_03730;prediction_source=maker_MRET:augustus_masked-contig015-processed-gene-0.40-mRNA-1
contig015	maker	CDS	13879	15144	.	+	0	ID=MALK_03730.t1.c1;Parent=MALK_03730.t1
contig015	maker	mRNA	13879	15144	.	+	.	ID=MALK_03730.t1;Parent=MALK_03730
contig015	maker	exon	13879	15144	.	+	.	ID=MALK_03730.t1.e1;Parent=MALK_03730.t1
contig015	AUGUSTUS	gene	15388	16528	0.37	-	.	ID=MALK_03731;prediction_source=braker_MRET:g2357.t1
contig015	AUGUSTUS	CDS	16465	16528	0.63	-	0	ID=MALK_03731.t1.c2;Parent=MALK_03731.t1
contig015	AUGUSTUS	CDS	15388	16388	0.63	-	0	ID=MALK_03731.t1.c1;Parent=MALK_03731.t1
contig015	AUGUSTUS	mRNA	15388	16528	0.37	-	.	ID=MALK_03731.t1;Parent=MALK_03731
contig015	AUGUSTUS	exon	16465	16528	.	-	.	ID=MALK_03731.t1.e2;Parent=MALK_03731.t1
contig015	AUGUSTUS	exon	15388	16388	.	-	.	ID=MALK_03731.t1.e1;Parent=MALK_03731.t1
contig015	maker	gene	16655	19981	.	+	.	ID=MALK_03732;prediction_source=maker_MRET:augustus_masked-contig015-processed-gene-0.41-mRNA-1
contig015	maker	CDS	16655	19981	.	+	0	ID=MALK_03732.t1.c1;Parent=MALK_03732.t1
contig015	maker	mRNA	16655	19981	.	+	.	ID=MALK_03732.t1;Parent=MALK_03732
contig015	maker	exon	16655	19981	.	+	.	ID=MALK_03732.t1.e1;Parent=MALK_03732.t1
contig015	AUGUSTUS	gene	19987	20661	0.69	-	.	ID=MALK_03733;prediction_source=augustus:contig015.g1409.t1
contig015	AUGUSTUS	CDS	19987	20661	0.69	-	0	ID=MALK_03733.t1.c1;Parent=MALK_03733.t1
contig015	AUGUSTUS	mRNA	19987	20661	0.69	-	.	ID=MALK_03733.t1;Parent=MALK_03733
contig015	AUGUSTUS	exon	19987	20661	0.69	-	.	ID=MALK_03733.t1.e1;Parent=MALK_03733.t1
contig015	maker	gene	21268	23094	.	-	.	ID=MALK_03734;prediction_source=maker_MRET:augustus_masked-contig015-processed-gene-0.73-mRNA-1
contig015	maker	CDS	21268	23094	.	-	0	ID=MALK_03734.t1.c1;Parent=MALK_03734.t1
contig015	maker	mRNA	21268	23094	.	-	.	ID=MALK_03734.t1;Parent=MALK_03734
contig015	maker	exon	21268	23094	.	-	.	ID=MALK_03734.t1.e1;Parent=MALK_03734.t1
contig015	AUGUSTUS	gene	23541	24107	0.97	+	.	ID=MALK_03735;prediction_source=augustus:contig015.g1411.t1
contig015	AUGUSTUS	CDS	23541	24107	0.97	+	0	ID=MALK_03735.t1.c1;Parent=MALK_03735.t1
contig015	AUGUSTUS	mRNA	23541	24107	0.97	+	.	ID=MALK_03735.t1;Parent=MALK_03735
contig015	AUGUSTUS	exon	23541	24107	0.97	+	.	ID=MALK_03735.t1.e1;Parent=MALK_03735.t1
contig015	AUGUSTUS	gene	24457	26685	1	-	.	ID=MALK_03736;prediction_source=augustus:contig015.g1412.t1
contig015	AUGUSTUS	CDS	24457	26685	1	-	0	ID=MALK_03736.t1.c1;Parent=MALK_03736.t1
contig015	AUGUSTUS	mRNA	24457	26685	1	-	.	ID=MALK_03736.t1;Parent=MALK_03736
contig015	AUGUSTUS	exon	24457	26685	1	-	.	ID=MALK_03736.t1.e1;Parent=MALK_03736.t1
contig015	AUGUSTUS	gene	26912	29785	1	-	.	ID=MALK_03737;prediction_source=augustus:contig015.g1414.t1
contig015	AUGUSTUS	CDS	26912	29785	1	-	0	ID=MALK_03737.t1.c1;Parent=MALK_03737.t1
contig015	AUGUSTUS	mRNA	26912	29785	1	-	.	ID=MALK_03737.t1;Parent=MALK_03737
contig015	AUGUSTUS	exon	26912	29785	1	-	.	ID=MALK_03737.t1.e1;Parent=MALK_03737.t1
contig015	maker	gene	29916	31211	.	+	.	ID=MALK_03738;prediction_source=maker_MRET:augustus_masked-contig015-processed-gene-0.43-mRNA-1
contig015	maker	CDS	29916	31211	.	+	0	ID=MALK_03738.t1.c1;Parent=MALK_03738.t1

contig015	maker	mRNA	29916	31211	.	+	.	ID=MALK_03738.t1;Parent=MALK_03738
contig015	maker	exon	29916	31211	.	+	.	ID=MALK_03738.t1.e1;Parent=MALK_03738.t1
contig015	AUGUSTUS	gene	31437	33130	0.31	+	.	ID=MALK_03739;prediction_source=braker_MRET:g2364.t1
contig015	AUGUSTUS	CDS	31437	31597	0.7	+	0	ID=MALK_03739.t1.c1;Parent=MALK_03739.t1
contig015	AUGUSTUS	CDS	31664	32017	0.7	+	0	ID=MALK_03739.t1.c2;Parent=MALK_03739.t1
contig015	AUGUSTUS	CDS	32061	32211	0.7	+	0	ID=MALK_03739.t1.c3;Parent=MALK_03739.t1
contig015	AUGUSTUS	CDS	32252	32358	0.7	+	0	ID=MALK_03739.t1.c4;Parent=MALK_03739.t1
contig015	AUGUSTUS	CDS	32422	33130	0.7	+	0	ID=MALK_03739.t1.c5;Parent=MALK_03739.t1
contig015	AUGUSTUS	mRNA	31437	33130	0.31	+	.	ID=MALK_03739.t1;Parent=MALK_03739
contig015	AUGUSTUS	exon	31437	31597	.	+	.	ID=MALK_03739.t1.e1;Parent=MALK_03739.t1
contig015	AUGUSTUS	exon	31664	32017	.	+	.	ID=MALK_03739.t1.e2;Parent=MALK_03739.t1
contig015	AUGUSTUS	exon	32061	32211	.	+	.	ID=MALK_03739.t1.e3;Parent=MALK_03739.t1
contig015	AUGUSTUS	exon	32252	32358	.	+	.	ID=MALK_03739.t1.e4;Parent=MALK_03739.t1
contig015	AUGUSTUS	exon	32422	33130	.	+	.	ID=MALK_03739.t1.e5;Parent=MALK_03739.t1
contig015	AUGUSTUS	gene	33400	35286	0.95	+	.	ID=MALK_03740;prediction_source=braker_MRET:g2365.t1
contig015	AUGUSTUS	CDS	33400	35286	0.95	+	0	ID=MALK_03740.t1.c1;Parent=MALK_03740.t1
contig015	AUGUSTUS	mRNA	33400	35286	0.95	+	.	ID=MALK_03740.t1;Parent=MALK_03740
contig015	AUGUSTUS	exon	33400	35286	.	+	.	ID=MALK_03740.t1.e1;Parent=MALK_03740.t1
contig015	AUGUSTUS	gene	35283	35528	0.98	-	.	ID=MALK_03741;prediction_source=augustus:contig015.g1418.t1
contig015	AUGUSTUS	CDS	35283	35528	0.98	-	0	ID=MALK_03741.t1.c1;Parent=MALK_03741.t1
contig015	AUGUSTUS	mRNA	35283	35528	0.98	-	.	ID=MALK_03741.t1;Parent=MALK_03741
contig015	AUGUSTUS	exon	35283	35528	0.98	-	.	ID=MALK_03741.t1.e1;Parent=MALK_03741.t1
contig015	maker	gene	35598	36926	.	+	.	ID=MALK_03742;prediction_source=maker_MRET:augustus_masked-contig015-processed-gene-0.46-mRNA-1
contig015	maker	CDS	35598	36926	.	+	0	ID=MALK_03742.t1.c1;Parent=MALK_03742.t1
contig015	maker	mRNA	35598	36926	.	+	.	ID=MALK_03742.t1;Parent=MALK_03742
contig015	maker	exon	35598	36926	.	+	.	ID=MALK_03742.t1.e1;Parent=MALK_03742.t1
contig015	maker	gene	37078	38403	.	+	.	ID=MALK_03743;prediction_source=maker_MRET:augustus_masked-contig015-processed-gene-0.47-mRNA-1
contig015	maker	CDS	37078	38403	.	+	0	ID=MALK_03743.t1.c1;Parent=MALK_03743.t1
contig015	maker	mRNA	37078	38403	.	+	.	ID=MALK_03743.t1;Parent=MALK_03743
contig015	maker	exon	37078	38403	.	+	.	ID=MALK_03743.t1.e1;Parent=MALK_03743.t1
contig015	AUGUSTUS	gene	38430	42020	0.81	-	.	ID=MALK_03744;prediction_source=augustus:contig015.g1421.t1
contig015	AUGUSTUS	CDS	38430	42020	0.81	-	0	ID=MALK_03744.t1.c1;Parent=MALK_03744.t1
contig015	AUGUSTUS	mRNA	38430	42020	0.81	-	.	ID=MALK_03744.t1;Parent=MALK_03744
contig015	AUGUSTUS	exon	38430	42020	0.81	-	.	ID=MALK_03744.t1.e1;Parent=MALK_03744.t1
contig015	AUGUSTUS	gene	41988	44044	0.36	+	.	ID=MALK_03745;prediction_source=augustus:contig015.g1422.t1
contig015	AUGUSTUS	CDS	41988	42038	0.8	+	0	ID=MALK_03745.t1.c1;Parent=MALK_03745.t1
contig015	AUGUSTUS	CDS	42107	43457	0.8	+	0	ID=MALK_03745.t1.c2;Parent=MALK_03745.t1
contig015	AUGUSTUS	CDS	43794	44044	0.8	+	0	ID=MALK_03745.t1.c3;Parent=MALK_03745.t1
contig015	AUGUSTUS	mRNA	41988	44044	0.36	+	.	ID=MALK_03745.t1;Parent=MALK_03745
contig015	AUGUSTUS	exon	41988	42038	0.8	+	.	ID=MALK_03745.t1.e1;Parent=MALK_03745.t1
contig015	AUGUSTUS	exon	42107	43457	0.8	+	.	ID=MALK_03745.t1.e2;Parent=MALK_03745.t1
contig015	AUGUSTUS	exon	43794	44044	0.8	+	.	ID=MALK_03745.t1.e3;Parent=MALK_03745.t1
contig015	maker	gene	44521	45861	.	+	.	ID=MALK_03746;prediction_source=maker_MRET:augustus_masked-contig015-processed-gene-0.49-mRNA-1

contig015	maker	CDS	44521	45861	.	+	0	ID=MALK_03746.t1.c1;Parent=MALK_03746.t1
contig015	maker	mRNA	44521	45861	.	+	.	ID=MALK_03746.t1;Parent=MALK_03746
contig015	maker	exon	44521	45861	.	+	.	ID=MALK_03746.t1.e1;Parent=MALK_03746.t1
contig015	AUGUSTUS	gene	46014	46410	0.11	+	.	ID=MALK_03747;prediction_source=braker_MRET:g2371.t1
contig015	AUGUSTUS	CDS	46014	46179	0.38	+	0	ID=MALK_03747.t1.c1;Parent=MALK_03747.t1
contig015	AUGUSTUS	CDS	46224	46348	0.38	+	0	ID=MALK_03747.t1.c2;Parent=MALK_03747.t1
contig015	AUGUSTUS	CDS	46387	46410	0.38	+	0	ID=MALK_03747.t1.c3;Parent=MALK_03747.t1
contig015	AUGUSTUS	mRNA	46014	46410	0.11	+	.	ID=MALK_03747.t1;Parent=MALK_03747
contig015	AUGUSTUS	exon	46014	46179	.	+	.	ID=MALK_03747.t1.e1;Parent=MALK_03747.t1
contig015	AUGUSTUS	exon	46224	46348	.	+	.	ID=MALK_03747.t1.e2;Parent=MALK_03747.t1
contig015	AUGUSTUS	exon	46387	46410	.	+	.	ID=MALK_03747.t1.e3;Parent=MALK_03747.t1
contig015	maker	gene	47004	48251	.	+	.	ID=MALK_03748;prediction_source=maker_MRET:augustus_masked-contig015-processed-gene-0.50-mRNA-1
contig015	maker	CDS	47004	48251	.	+	0	ID=MALK_03748.t1.c1;Parent=MALK_03748.t1
contig015	maker	mRNA	47004	48251	.	+	.	ID=MALK_03748.t1;Parent=MALK_03748
contig015	maker	exon	47004	48251	.	+	.	ID=MALK_03748.t1.e1;Parent=MALK_03748.t1
contig015	AUGUSTUS	gene	48302	49903	0.89	-	.	ID=MALK_03749;prediction_source=augustus:contig015.g1426.t1
contig015	AUGUSTUS	CDS	48302	49903	0.89	-	0	ID=MALK_03749.t1.c1;Parent=MALK_03749.t1
contig015	AUGUSTUS	mRNA	48302	49903	0.89	-	.	ID=MALK_03749.t1;Parent=MALK_03749
contig015	AUGUSTUS	exon	48302	49903	0.89	-	.	ID=MALK_03749.t1.e1;Parent=MALK_03749.t1
contig015	AUGUSTUS	gene	50679	54029	0.98	+	.	ID=MALK_03750;prediction_source=augustus:contig015.g1427.t1
contig015	AUGUSTUS	CDS	50679	50923	0.98	+	0	ID=MALK_03750.t1.c1;Parent=MALK_03750.t1
contig015	AUGUSTUS	CDS	50987	54029	0.98	+	0	ID=MALK_03750.t1.c2;Parent=MALK_03750.t1
contig015	AUGUSTUS	mRNA	50679	54029	0.98	+	.	ID=MALK_03750.t1;Parent=MALK_03750
contig015	AUGUSTUS	exon	50679	50923	0.98	+	.	ID=MALK_03750.t1.e1;Parent=MALK_03750.t1
contig015	AUGUSTUS	exon	50987	54029	0.98	+	.	ID=MALK_03750.t1.e2;Parent=MALK_03750.t1
contig015	maker	gene	54269	55453	.	+	.	ID=MALK_03751;prediction_source=maker_MRET:augustus_masked-contig015-processed-gene-0.52-mRNA-1
contig015	maker	CDS	54269	55453	.	+	0	ID=MALK_03751.t1.c1;Parent=MALK_03751.t1
contig015	maker	mRNA	54269	55453	.	+	.	ID=MALK_03751.t1;Parent=MALK_03751
contig015	maker	exon	54269	55453	.	+	.	ID=MALK_03751.t1.e1;Parent=MALK_03751.t1
contig015	AUGUSTUS	gene	55842	58492	0.75	-	.	ID=MALK_03752;prediction_source=braker_MRET:g2376.t1
contig015	AUGUSTUS	CDS	56448	58492	0.76	-	0	ID=MALK_03752.t1.c2;Parent=MALK_03752.t1
contig015	AUGUSTUS	CDS	55842	56415	0.76	-	0	ID=MALK_03752.t1.c1;Parent=MALK_03752.t1
contig015	AUGUSTUS	mRNA	55842	58492	0.75	-	.	ID=MALK_03752.t1;Parent=MALK_03752
contig015	AUGUSTUS	exon	56448	58492	.	-	.	ID=MALK_03752.t1.e2;Parent=MALK_03752.t1
contig015	AUGUSTUS	exon	55842	56415	.	-	.	ID=MALK_03752.t1.e1;Parent=MALK_03752.t1
contig015	AUGUSTUS	gene	58735	59199	0.41	-	.	ID=MALK_03753;prediction_source=braker_MRET:g2377.t1
contig015	AUGUSTUS	CDS	59164	59199	0.65	-	0	ID=MALK_03753.t1.c3;Parent=MALK_03753.t1
contig015	AUGUSTUS	CDS	59115	59130	0.65	-	0	ID=MALK_03753.t1.c2;Parent=MALK_03753.t1
contig015	AUGUSTUS	CDS	58735	58943	0.65	-	0	ID=MALK_03753.t1.c1;Parent=MALK_03753.t1
contig015	AUGUSTUS	mRNA	58735	59199	0.41	-	.	ID=MALK_03753.t1;Parent=MALK_03753
contig015	AUGUSTUS	exon	59164	59199	.	-	.	ID=MALK_03753.t1.e3;Parent=MALK_03753.t1
contig015	AUGUSTUS	exon	59115	59130	.	-	.	ID=MALK_03753.t1.e2;Parent=MALK_03753.t1
contig015	AUGUSTUS	exon	58735	58943	.	-	.	ID=MALK_03753.t1.e1;Parent=MALK_03753.t1

contig015	AUGUSTUS	gene	59426	60204	0.99	+	.	ID=MALK_03754;prediction_source=braker_MRET:g2378.t1
contig015	AUGUSTUS	CDS	59426	59477	1	+	0	ID=MALK_03754.t1.c1;Parent=MALK_03754.t1
contig015	AUGUSTUS	CDS	59507	60204	1	+	0	ID=MALK_03754.t1.c2;Parent=MALK_03754.t1
contig015	AUGUSTUS	mRNA	59426	60204	0.99	+	.	ID=MALK_03754.t1;Parent=MALK_03754
contig015	AUGUSTUS	exon	59426	59477	.	+	.	ID=MALK_03754.t1.e1;Parent=MALK_03754.t1
contig015	AUGUSTUS	exon	59507	60204	.	+	.	ID=MALK_03754.t1.e2;Parent=MALK_03754.t1
contig015	maker	gene	60206	62023	.	-	.	ID=MALK_03755;prediction_source=maker_MRET:augustus_masked-contig015-processed-gene-0.80-mRNA-1
contig015	maker	CDS	60206	62023	.	-	0	ID=MALK_03755.t1.c1;Parent=MALK_03755.t1
contig015	maker	mRNA	60206	62023	.	-	.	ID=MALK_03755.t1;Parent=MALK_03755
contig015	maker	exon	60206	62023	.	-	.	ID=MALK_03755.t1.e1;Parent=MALK_03755.t1
contig015	AUGUSTUS	gene	62111	64462	0.99	+	.	ID=MALK_03756;prediction_source=augustus:contig015.g1434.t1
contig015	AUGUSTUS	CDS	62111	64462	0.99	+	0	ID=MALK_03756.t1.c1;Parent=MALK_03756.t1
contig015	AUGUSTUS	mRNA	62111	64462	0.99	+	.	ID=MALK_03756.t1;Parent=MALK_03756
contig015	AUGUSTUS	exon	62111	64462	0.99	+	.	ID=MALK_03756.t1.e1;Parent=MALK_03756.t1
contig015	AUGUSTUS	gene	64608	66128	0.99	+	.	ID=MALK_03757;prediction_source=augustus:contig015.g1435.t1
contig015	AUGUSTUS	CDS	64608	66128	0.99	+	0	ID=MALK_03757.t1.c1;Parent=MALK_03757.t1
contig015	AUGUSTUS	mRNA	64608	66128	0.99	+	.	ID=MALK_03757.t1;Parent=MALK_03757
contig015	AUGUSTUS	exon	64608	66128	0.99	+	.	ID=MALK_03757.t1.e1;Parent=MALK_03757.t1
contig015	maker	gene	66275	68371	.	-	.	ID=MALK_03758;prediction_source=maker_MRET:augustus_masked-contig015-processed-gene-0.81-mRNA-1
contig015	maker	CDS	66275	68371	.	-	0	ID=MALK_03758.t1.c1;Parent=MALK_03758.t1
contig015	maker	mRNA	66275	68371	.	-	.	ID=MALK_03758.t1;Parent=MALK_03758
contig015	maker	exon	66275	68371	.	-	.	ID=MALK_03758.t1.e1;Parent=MALK_03758.t1
contig015	AUGUSTUS	gene	69035	71254	0.77	+	.	ID=MALK_03759;prediction_source=augustus:contig015.g1437.t1
contig015	AUGUSTUS	CDS	69035	71254	0.77	+	0	ID=MALK_03759.t1.c1;Parent=MALK_03759.t1
contig015	AUGUSTUS	mRNA	69035	71254	0.77	+	.	ID=MALK_03759.t1;Parent=MALK_03759
contig015	AUGUSTUS	exon	69035	71254	0.77	+	.	ID=MALK_03759.t1.e1;Parent=MALK_03759.t1
contig015	maker	gene	71352	72254	.	-	.	ID=MALK_03760;prediction_source=maker_MRET:augustus_masked-contig015-processed-gene-0.82-mRNA-1
contig015	maker	CDS	71352	72254	.	-	0	ID=MALK_03760.t1.c1;Parent=MALK_03760.t1
contig015	maker	mRNA	71352	72254	.	-	.	ID=MALK_03760.t1;Parent=MALK_03760
contig015	maker	exon	71352	72254	.	-	.	ID=MALK_03760.t1.e1;Parent=MALK_03760.t1
contig015	AUGUSTUS	gene	72545	77173	0.98	+	.	ID=MALK_03761;prediction_source=augustus:contig015.g1439.t1
contig015	AUGUSTUS	CDS	72545	77173	0.98	+	0	ID=MALK_03761.t1.c1;Parent=MALK_03761.t1
contig015	AUGUSTUS	mRNA	72545	77173	0.98	+	.	ID=MALK_03761.t1;Parent=MALK_03761
contig015	AUGUSTUS	exon	72545	77173	0.98	+	.	ID=MALK_03761.t1.e1;Parent=MALK_03761.t1
contig015	AUGUSTUS	gene	77635	78582	1	+	.	ID=MALK_03762;prediction_source=augustus:contig015.g1443.t1
contig015	AUGUSTUS	CDS	77635	78582	1	+	0	ID=MALK_03762.t1.c1;Parent=MALK_03762.t1
contig015	AUGUSTUS	mRNA	77635	78582	1	+	.	ID=MALK_03762.t1;Parent=MALK_03762
contig015	AUGUSTUS	exon	77635	78582	1	+	.	ID=MALK_03762.t1.e1;Parent=MALK_03762.t1
contig015	AUGUSTUS	gene	78888	80369	0.94	+	.	ID=MALK_03763;prediction_source=augustus:contig015.g1444.t1
contig015	AUGUSTUS	CDS	78888	80369	0.94	+	0	ID=MALK_03763.t1.c1;Parent=MALK_03763.t1
contig015	AUGUSTUS	mRNA	78888	80369	0.94	+	.	ID=MALK_03763.t1;Parent=MALK_03763
contig015	AUGUSTUS	exon	78888	80369	0.94	+	.	ID=MALK_03763.t1.e1;Parent=MALK_03763.t1
contig015	AUGUSTUS	gene	80722	84270	0.99	+	.	ID=MALK_03764;prediction_source=augustus:contig015.g1445.t1

contig015	AUGUSTUS	CDS	80722	84270	0.99	+	0	ID=MALK_03764.t1.c1;Parent=MALK_03764.t1
contig015	AUGUSTUS	mRNA	80722	84270	0.99	+	.	ID=MALK_03764.t1;Parent=MALK_03764
contig015	AUGUSTUS	exon	80722	84270	0.99	+	.	ID=MALK_03764.t1.e1;Parent=MALK_03764.t1
contig015	AUGUSTUS	gene	84242	85828	1	-	.	ID=MALK_03765;prediction_source=augustus:contig015.g1446.t1
contig015	AUGUSTUS	CDS	84242	85828	1	-	0	ID=MALK_03765.t1.c1;Parent=MALK_03765.t1
contig015	AUGUSTUS	mRNA	84242	85828	1	-	.	ID=MALK_03765.t1;Parent=MALK_03765
contig015	AUGUSTUS	exon	84242	85828	1	-	.	ID=MALK_03765.t1.e1;Parent=MALK_03765.t1
contig015	AUGUSTUS	gene	86267	86584	0.42	+	.	ID=MALK_03766;prediction_source=braker_MRET:g2390.t1
contig015	AUGUSTUS	CDS	86267	86584	0.42	+	0	ID=MALK_03766.t1.c1;Parent=MALK_03766.t1
contig015	AUGUSTUS	mRNA	86267	86584	0.42	+	.	ID=MALK_03766.t1;Parent=MALK_03766
contig015	AUGUSTUS	exon	86267	86584	.	+	.	ID=MALK_03766.t1.e1;Parent=MALK_03766.t1
contig015	maker	gene	86824	88617	.	+	.	ID=MALK_03767;prediction_source=maker_MRET:augustus_masked-contig015-processed-gene-0.61-mRNA-1
contig015	maker	CDS	86824	88617	.	+	0	ID=MALK_03767.t1.c1;Parent=MALK_03767.t1
contig015	maker	mRNA	86824	88617	.	+	.	ID=MALK_03767.t1;Parent=MALK_03767
contig015	maker	exon	86824	88617	.	+	.	ID=MALK_03767.t1.e1;Parent=MALK_03767.t1
contig015	AUGUSTUS	gene	88670	91809	0.82	-	.	ID=MALK_03768;prediction_source=braker_MRET:g2392.t1
contig015	AUGUSTUS	CDS	90849	91809	0.92	-	0	ID=MALK_03768.t1.c2;Parent=MALK_03768.t1
contig015	AUGUSTUS	CDS	88670	90750	0.92	-	0	ID=MALK_03768.t1.c1;Parent=MALK_03768.t1
contig015	AUGUSTUS	mRNA	88670	91809	0.82	-	.	ID=MALK_03768.t1;Parent=MALK_03768
contig015	AUGUSTUS	exon	90849	91809	.	-	.	ID=MALK_03768.t1.e2;Parent=MALK_03768.t1
contig015	AUGUSTUS	exon	88670	90750	.	-	.	ID=MALK_03768.t1.e1;Parent=MALK_03768.t1
contig015	AUGUSTUS	gene	91884	93883	0.44	-	.	ID=MALK_03769;prediction_source=braker_MRET:g2393.t1
contig015	AUGUSTUS	CDS	93808	93883	0.48	-	0	ID=MALK_03769.t1.c2;Parent=MALK_03769.t1
contig015	AUGUSTUS	CDS	91884	93772	0.48	-	0	ID=MALK_03769.t1.c1;Parent=MALK_03769.t1
contig015	AUGUSTUS	mRNA	91884	93883	0.44	-	.	ID=MALK_03769.t1;Parent=MALK_03769
contig015	AUGUSTUS	exon	93808	93883	.	-	.	ID=MALK_03769.t1.e2;Parent=MALK_03769.t1
contig015	AUGUSTUS	exon	91884	93772	.	-	.	ID=MALK_03769.t1.e1;Parent=MALK_03769.t1
contig015	AUGUSTUS	gene	93828	95093	0.57	+	.	ID=MALK_03770;prediction_source=augustus:contig015.g1450.t1
contig015	AUGUSTUS	CDS	93828	95093	0.57	+	0	ID=MALK_03770.t1.c1;Parent=MALK_03770.t1
contig015	AUGUSTUS	mRNA	93828	95093	0.57	+	.	ID=MALK_03770.t1;Parent=MALK_03770
contig015	AUGUSTUS	exon	93828	95093	0.57	+	.	ID=MALK_03770.t1.e1;Parent=MALK_03770.t1
contig015	maker	gene	95090	96511	.	-	.	ID=MALK_03771;prediction_source=maker_MRET:augustus_masked-contig015-processed-gene-0.85-mRNA-1
contig015	maker	CDS	95090	96511	.	-	0	ID=MALK_03771.t1.c1;Parent=MALK_03771.t1
contig015	maker	mRNA	95090	96511	.	-	.	ID=MALK_03771.t1;Parent=MALK_03771
contig015	maker	exon	95090	96511	.	-	.	ID=MALK_03771.t1.e1;Parent=MALK_03771.t1
contig015	AUGUSTUS	gene	96672	99092	0.92	+	.	ID=MALK_03772;prediction_source=augustus:contig015.g1452.t1
contig015	AUGUSTUS	CDS	96672	99092	0.92	+	0	ID=MALK_03772.t1.c1;Parent=MALK_03772.t1
contig015	AUGUSTUS	mRNA	96672	99092	0.92	+	.	ID=MALK_03772.t1;Parent=MALK_03772
contig015	AUGUSTUS	exon	96672	99092	0.92	+	.	ID=MALK_03772.t1.e1;Parent=MALK_03772.t1
contig015	AUGUSTUS	gene	99118	99828	1	-	.	ID=MALK_03773;prediction_source=augustus:contig015.g1453.t1
contig015	AUGUSTUS	CDS	99118	99828	1	-	0	ID=MALK_03773.t1.c1;Parent=MALK_03773.t1
contig015	AUGUSTUS	mRNA	99118	99828	1	-	.	ID=MALK_03773.t1;Parent=MALK_03773
contig015	AUGUSTUS	exon	99118	99828	1	-	.	ID=MALK_03773.t1.e1;Parent=MALK_03773.t1

contig015	AUGUSTUS	gene	99933	100265	1	-	.	ID=MALK_03774;prediction_source=augustus:contig015.g1454.t1
contig015	AUGUSTUS	CDS	99933	100265	1	-	0	ID=MALK_03774.t1.c1;Parent=MALK_03774.t1
contig015	AUGUSTUS	mRNA	99933	100265	1	-	.	ID=MALK_03774.t1;Parent=MALK_03774
contig015	AUGUSTUS	exon	99933	100265	1	-	.	ID=MALK_03774.t1.e1;Parent=MALK_03774.t1
contig015	AUGUSTUS	gene	100852	102000	0.6	+	.	ID=MALK_03775;prediction_source=augustus:contig015.g1455.t1
contig015	AUGUSTUS	CDS	100852	102000	0.6	+	0	ID=MALK_03775.t1.c1;Parent=MALK_03775.t1
contig015	AUGUSTUS	mRNA	100852	102000	0.6	+	.	ID=MALK_03775.t1;Parent=MALK_03775
contig015	AUGUSTUS	exon	100852	102000	0.6	+	.	ID=MALK_03775.t1.e1;Parent=MALK_03775.t1
contig015	maker	gene	102016	103632	.	-	.	ID=MALK_03776;prediction_source=maker_MRET:augustus_masked-contig015-processed-gene-0.88-mRNA-1
contig015	maker	CDS	102016	103632	.	-	0	ID=MALK_03776.t1.c1;Parent=MALK_03776.t1
contig015	maker	mRNA	102016	103632	.	-	.	ID=MALK_03776.t1;Parent=MALK_03776
contig015	maker	exon	102016	103632	.	-	.	ID=MALK_03776.t1.e1;Parent=MALK_03776.t1
contig015	AUGUSTUS	gene	103706	106426	1	-	.	ID=MALK_03777;prediction_source=augustus:contig015.g1457.t1
contig015	AUGUSTUS	CDS	103706	106426	1	-	0	ID=MALK_03777.t1.c1;Parent=MALK_03777.t1
contig015	AUGUSTUS	mRNA	103706	106426	1	-	.	ID=MALK_03777.t1;Parent=MALK_03777
contig015	AUGUSTUS	exon	103706	106426	1	-	.	ID=MALK_03777.t1.e1;Parent=MALK_03777.t1
contig015	AUGUSTUS	gene	106662	107255	0.7	+	.	ID=MALK_03778;prediction_source=braker_MRET:g2401.t1
contig015	AUGUSTUS	CDS	106662	107255	0.7	+	0	ID=MALK_03778.t1.c1;Parent=MALK_03778.t1
contig015	AUGUSTUS	mRNA	106662	107255	0.7	+	.	ID=MALK_03778.t1;Parent=MALK_03778
contig015	AUGUSTUS	exon	106662	107255	.	+	.	ID=MALK_03778.t1.e1;Parent=MALK_03778.t1
contig015	maker	gene	107446	108357	.	-	.	ID=MALK_03779;prediction_source=maker_MRET:augustus_masked-contig015-processed-gene-0.90-mRNA-1
contig015	maker	CDS	107446	108357	.	-	0	ID=MALK_03779.t1.c1;Parent=MALK_03779.t1
contig015	maker	mRNA	107446	108357	.	-	.	ID=MALK_03779.t1;Parent=MALK_03779
contig015	maker	exon	107446	108357	.	-	.	ID=MALK_03779.t1.e1;Parent=MALK_03779.t1
contig015	maker	gene	108973	110622	.	+	.	ID=MALK_03780;prediction_source=maker_MRET:augustus_masked-contig015-processed-gene-0.66-mRNA-1
contig015	maker	CDS	108973	110622	.	+	0	ID=MALK_03780.t1.c1;Parent=MALK_03780.t1
contig015	maker	mRNA	108973	110622	.	+	.	ID=MALK_03780.t1;Parent=MALK_03780
contig015	maker	exon	108973	110622	.	+	.	ID=MALK_03780.t1.e1;Parent=MALK_03780.t1
contig016	annotation	remark	1	404011	.	+	.	gff-version=3
contig016	AUGUSTUS	gene	897	1886	0.31	+	.	ID=MALK_03781;prediction_source=augustus:contig016.g3744.t1
contig016	AUGUSTUS	CDS	897	1886	0.31	+	0	ID=MALK_03781.t1.c1;Parent=MALK_03781.t1
contig016	AUGUSTUS	mRNA	897	1886	0.31	+	.	ID=MALK_03781.t1;Parent=MALK_03781
contig016	AUGUSTUS	exon	897	1886	0.31	+	.	ID=MALK_03781.t1.e1;Parent=MALK_03781.t1
contig016	AUGUSTUS	gene	2153	2551	0.21	+	.	ID=MALK_03782;prediction_source=augustus:contig016.g3745.t1
contig016	AUGUSTUS	CDS	2153	2551	0.21	+	0	ID=MALK_03782.t1.c1;Parent=MALK_03782.t1
contig016	AUGUSTUS	mRNA	2153	2551	0.21	+	.	ID=MALK_03782.t1;Parent=MALK_03782
contig016	AUGUSTUS	exon	2153	2551	0.21	+	.	ID=MALK_03782.t1.e1;Parent=MALK_03782.t1
contig016	maker	gene	3942	4856	.	+	.	ID=MALK_03783;prediction_source=maker_MRET:augustus_masked-contig016-processed-gene-0.40-mRNA-1
contig016	maker	CDS	3942	4856	.	+	0	ID=MALK_03783.t1.c1;Parent=MALK_03783.t1
contig016	maker	mRNA	3942	4856	.	+	.	ID=MALK_03783.t1;Parent=MALK_03783
contig016	maker	exon	3942	4856	.	+	.	ID=MALK_03783.t1.e1;Parent=MALK_03783.t1
contig016	maker	gene	4899	7511	.	+	.	ID=MALK_03784;prediction_source=maker_MRET:augustus_masked-contig016-processed-gene-0.41-mRNA-1
contig016	maker	CDS	4899	7511	.	+	0	ID=MALK_03784.t1.c1;Parent=MALK_03784.t1

contig016	maker	mRNA	4899	7511	.	+	.	ID=MALK_03784.t1;Parent=MALK_03784
contig016	maker	exon	4899	7511	.	+	.	ID=MALK_03784.t1.e1;Parent=MALK_03784.t1
contig016	maker	gene	8052	8885	.	-	.	ID=MALK_03785;prediction_source=maker_MRET:augustus_masked-contig016-processed-gene-0.60-mRNA-1
contig016	maker	CDS	8052	8885	.	-	0	ID=MALK_03785.t1.c1;Parent=MALK_03785.t1
contig016	maker	mRNA	8052	8885	.	-	.	ID=MALK_03785.t1;Parent=MALK_03785
contig016	maker	exon	8052	8885	.	-	.	ID=MALK_03785.t1.e1;Parent=MALK_03785.t1
contig016	AUGUSTUS	gene	9156	9818	0.89	+	.	ID=MALK_03786;prediction_source=augustus:contig016.g3749.t1
contig016	AUGUSTUS	CDS	9156	9818	0.89	+	0	ID=MALK_03786.t1.c1;Parent=MALK_03786.t1
contig016	AUGUSTUS	mRNA	9156	9818	0.89	+	.	ID=MALK_03786.t1;Parent=MALK_03786
contig016	AUGUSTUS	exon	9156	9818	0.89	+	.	ID=MALK_03786.t1.e1;Parent=MALK_03786.t1
contig016	AUGUSTUS	gene	9799	10728	0.28	-	.	ID=MALK_03787;prediction_source=braker_MRET:g3274.t1
contig016	AUGUSTUS	CDS	9799	10728	0.28	-	0	ID=MALK_03787.t1.c1;Parent=MALK_03787.t1
contig016	AUGUSTUS	mRNA	9799	10728	0.28	-	.	ID=MALK_03787.t1;Parent=MALK_03787
contig016	AUGUSTUS	exon	9799	10728	.	-	.	ID=MALK_03787.t1.e1;Parent=MALK_03787.t1
contig016	maker	gene	10818	12200	.	+	.	ID=MALK_03788;prediction_source=maker_MRET:augustus_masked-contig016-processed-gene-0.43-mRNA-1
contig016	maker	CDS	10818	12200	.	+	0	ID=MALK_03788.t1.c1;Parent=MALK_03788.t1
contig016	maker	mRNA	10818	12200	.	+	.	ID=MALK_03788.t1;Parent=MALK_03788
contig016	maker	exon	10818	12200	.	+	.	ID=MALK_03788.t1.e1;Parent=MALK_03788.t1
contig016	AUGUSTUS	gene	12240	12608	0.47	+	.	ID=MALK_03789;prediction_source=braker_MRET:g3276.t1
contig016	AUGUSTUS	CDS	12240	12608	0.47	+	0	ID=MALK_03789.t1.c1;Parent=MALK_03789.t1
contig016	AUGUSTUS	mRNA	12240	12608	0.47	+	.	ID=MALK_03789.t1;Parent=MALK_03789
contig016	AUGUSTUS	exon	12240	12608	.	+	.	ID=MALK_03789.t1.e1;Parent=MALK_03789.t1
contig016	AUGUSTUS	gene	12610	13641	0.56	-	.	ID=MALK_03790;prediction_source=augustus:contig016.g3751.t1
contig016	AUGUSTUS	CDS	12610	13641	0.56	-	0	ID=MALK_03790.t1.c1;Parent=MALK_03790.t1
contig016	AUGUSTUS	mRNA	12610	13641	0.56	-	.	ID=MALK_03790.t1;Parent=MALK_03790
contig016	AUGUSTUS	exon	12610	13641	0.56	-	.	ID=MALK_03790.t1.e1;Parent=MALK_03790.t1
contig016	AUGUSTUS	gene	13771	15036	0.47	+	.	ID=MALK_03791;prediction_source=braker_MRET:g3278.t1
contig016	AUGUSTUS	CDS	13771	14352	0.88	+	0	ID=MALK_03791.t1.c1;Parent=MALK_03791.t1
contig016	AUGUSTUS	CDS	14678	14731	0.88	+	0	ID=MALK_03791.t1.c2;Parent=MALK_03791.t1
contig016	AUGUSTUS	CDS	14770	15036	0.88	+	0	ID=MALK_03791.t1.c3;Parent=MALK_03791.t1
contig016	AUGUSTUS	mRNA	13771	15036	0.47	+	.	ID=MALK_03791.t1;Parent=MALK_03791
contig016	AUGUSTUS	exon	13771	14352	.	+	.	ID=MALK_03791.t1.e1;Parent=MALK_03791.t1
contig016	AUGUSTUS	exon	14678	14731	.	+	.	ID=MALK_03791.t1.e2;Parent=MALK_03791.t1
contig016	AUGUSTUS	exon	14770	15036	.	+	.	ID=MALK_03791.t1.e3;Parent=MALK_03791.t1
contig016	AUGUSTUS	gene	15054	15470	1	-	.	ID=MALK_03792;prediction_source=braker_MRET:g3279.t1
contig016	AUGUSTUS	CDS	15054	15470	1	-	0	ID=MALK_03792.t1.c1;Parent=MALK_03792.t1
contig016	AUGUSTUS	mRNA	15054	15470	1	-	.	ID=MALK_03792.t1;Parent=MALK_03792
contig016	AUGUSTUS	exon	15054	15470	.	-	.	ID=MALK_03792.t1.e1;Parent=MALK_03792.t1
contig016	AUGUSTUS	gene	15498	16907	0.72	+	.	ID=MALK_03793;prediction_source=braker_MRET:g3280.t1
contig016	AUGUSTUS	CDS	15498	16907	0.72	+	0	ID=MALK_03793.t1.c1;Parent=MALK_03793.t1
contig016	AUGUSTUS	mRNA	15498	16907	0.72	+	.	ID=MALK_03793.t1;Parent=MALK_03793
contig016	AUGUSTUS	exon	15498	16907	.	+	.	ID=MALK_03793.t1.e1;Parent=MALK_03793.t1
contig016	AUGUSTUS	gene	16975	17409	0.73	-	.	ID=MALK_03794;prediction_source=braker_MRET:g3281.t1

contig016	AUGUSTUS	CDS	16975	17409	0.73	-	0	ID=MALK_03794.t1.c1;Parent=MALK_03794.t1
contig016	AUGUSTUS	mRNA	16975	17409	0.73	-	.	ID=MALK_03794.t1;Parent=MALK_03794
contig016	AUGUSTUS	exon	16975	17409	.	-	.	ID=MALK_03794.t1.e1;Parent=MALK_03794.t1
contig016	maker	gene	17638	19758	.	-	.	ID=MALK_03795;prediction_source=maker_MRET:augustus_masked-contig016-processed-gene-0.62-mRNA-1
contig016	maker	CDS	17638	19758	.	-	0	ID=MALK_03795.t1.c1;Parent=MALK_03795.t1
contig016	maker	mRNA	17638	19758	.	-	.	ID=MALK_03795.t1;Parent=MALK_03795
contig016	maker	exon	17638	19758	.	-	.	ID=MALK_03795.t1.e1;Parent=MALK_03795.t1
contig016	AUGUSTUS	gene	19839	20403	0.53	+	.	ID=MALK_03796;prediction_source=braker_MRET:g3283.t1
contig016	AUGUSTUS	CDS	19839	20261	0.54	+	0	ID=MALK_03796.t1.c1;Parent=MALK_03796.t1
contig016	AUGUSTUS	CDS	20386	20403	0.54	+	0	ID=MALK_03796.t1.c2;Parent=MALK_03796.t1
contig016	AUGUSTUS	mRNA	19839	20403	0.53	+	.	ID=MALK_03796.t1;Parent=MALK_03796
contig016	AUGUSTUS	exon	19839	20261	.	+	.	ID=MALK_03796.t1.e1;Parent=MALK_03796.t1
contig016	AUGUSTUS	exon	20386	20403	.	+	.	ID=MALK_03796.t1.e2;Parent=MALK_03796.t1
contig016	AUGUSTUS	gene	20540	21556	0.26	-	.	ID=MALK_03797;prediction_source=augustus:contig016.g3755.t1
contig016	AUGUSTUS	CDS	20540	21556	0.26	-	0	ID=MALK_03797.t1.c1;Parent=MALK_03797.t1
contig016	AUGUSTUS	mRNA	20540	21556	0.26	-	.	ID=MALK_03797.t1;Parent=MALK_03797
contig016	AUGUSTUS	exon	20540	21556	0.26	-	.	ID=MALK_03797.t1.e1;Parent=MALK_03797.t1
contig016	maker	gene	21670	23095	.	+	.	ID=MALK_03798;prediction_source=maker_MRET:augustus_masked-contig016-processed-gene-0.45-mRNA-1
contig016	maker	CDS	21670	22087	.	+	0	ID=MALK_03798.t1.c1;Parent=MALK_03798.t1
contig016	maker	CDS	22419	23095	.	+	0	ID=MALK_03798.t1.c2;Parent=MALK_03798.t1
contig016	maker	mRNA	21670	23095	.	+	.	ID=MALK_03798.t1;Parent=MALK_03798
contig016	maker	exon	21670	22087	.	+	.	ID=MALK_03798.t1.e1;Parent=MALK_03798.t1
contig016	maker	exon	22419	23095	.	+	.	ID=MALK_03798.t1.e2;Parent=MALK_03798.t1
contig016	maker	gene	23103	25118	.	-	.	ID=MALK_03799;prediction_source=maker_MRET:augustus_masked-contig016-processed-gene-0.64-mRNA-1
contig016	maker	CDS	23103	25118	.	-	0	ID=MALK_03799.t1.c1;Parent=MALK_03799.t1
contig016	maker	mRNA	23103	25118	.	-	.	ID=MALK_03799.t1;Parent=MALK_03799
contig016	maker	exon	23103	25118	.	-	.	ID=MALK_03799.t1.e1;Parent=MALK_03799.t1
contig016	AUGUSTUS	gene	25528	27294	0.99	+	.	ID=MALK_03800;prediction_source=braker_MRET:g3287.t1
contig016	AUGUSTUS	CDS	25528	26023	1	+	0	ID=MALK_03800.t1.c1;Parent=MALK_03800.t1
contig016	AUGUSTUS	CDS	26063	27294	1	+	0	ID=MALK_03800.t1.c2;Parent=MALK_03800.t1
contig016	AUGUSTUS	mRNA	25528	27294	0.99	+	.	ID=MALK_03800.t1;Parent=MALK_03800
contig016	AUGUSTUS	exon	25528	26023	.	+	.	ID=MALK_03800.t1.e1;Parent=MALK_03800.t1
contig016	AUGUSTUS	exon	26063	27294	.	+	.	ID=MALK_03800.t1.e2;Parent=MALK_03800.t1
contig016	maker	gene	27334	28335	.	-	.	ID=MALK_03801;prediction_source=maker_MRET:augustus_masked-contig016-processed-gene-0.65-mRNA-1
contig016	maker	CDS	27334	28335	.	-	0	ID=MALK_03801.t1.c1;Parent=MALK_03801.t1
contig016	maker	mRNA	27334	28335	.	-	.	ID=MALK_03801.t1;Parent=MALK_03801
contig016	maker	exon	27334	28335	.	-	.	ID=MALK_03801.t1.e1;Parent=MALK_03801.t1
contig016	maker	gene	28497	30515	.	-	.	ID=MALK_03802;prediction_source=maker_MRET:augustus_masked-contig016-processed-gene-0.66-mRNA-1
contig016	maker	CDS	28497	30515	.	-	0	ID=MALK_03802.t1.c1;Parent=MALK_03802.t1
contig016	maker	mRNA	28497	30515	.	-	.	ID=MALK_03802.t1;Parent=MALK_03802
contig016	maker	exon	28497	30515	.	-	.	ID=MALK_03802.t1.e1;Parent=MALK_03802.t1
contig016	maker	gene	30825	31604	.	+	.	ID=MALK_03803;prediction_source=maker_MRET:augustus_masked-contig016-processed-gene-0.48-mRNA-1
contig016	maker	CDS	30825	31604	.	+	0	ID=MALK_03803.t1.c1;Parent=MALK_03803.t1

contig016	maker	mRNA	30825	31604	.	+	.	ID=MALK_03803.t1;Parent=MALK_03803
contig016	maker	exon	30825	31604	.	+	.	ID=MALK_03803.t1.e1;Parent=MALK_03803.t1
contig016	AUGUSTUS	gene	31599	32751	0.36	-	.	ID=MALK_03804;prediction_source=augustus:contig016.g3763.t1
contig016	AUGUSTUS	CDS	32068	32751	0.36	-	0	ID=MALK_03804.t1.c2;Parent=MALK_03804.t1
contig016	AUGUSTUS	CDS	31599	32006	0.36	-	0	ID=MALK_03804.t1.c1;Parent=MALK_03804.t1
contig016	AUGUSTUS	mRNA	31599	32751	0.36	-	.	ID=MALK_03804.t1;Parent=MALK_03804
contig016	AUGUSTUS	exon	32068	32751	0.36	-	.	ID=MALK_03804.t1.e2;Parent=MALK_03804.t1
contig016	AUGUSTUS	exon	31599	32006	0.36	-	.	ID=MALK_03804.t1.e1;Parent=MALK_03804.t1
contig016	AUGUSTUS	gene	32830	35046	1	+	.	ID=MALK_03805;prediction_source=augustus:contig016.g3764.t1
contig016	AUGUSTUS	CDS	32830	35046	1	+	0	ID=MALK_03805.t1.c1;Parent=MALK_03805.t1
contig016	AUGUSTUS	mRNA	32830	35046	1	+	.	ID=MALK_03805.t1;Parent=MALK_03805
contig016	AUGUSTUS	exon	32830	35046	1	+	.	ID=MALK_03805.t1.e1;Parent=MALK_03805.t1
contig016	AUGUSTUS	gene	35063	36424	0.93	-	.	ID=MALK_03806;prediction_source=augustus:contig016.g3765.t1
contig016	AUGUSTUS	CDS	35063	36424	0.93	-	0	ID=MALK_03806.t1.c1;Parent=MALK_03806.t1
contig016	AUGUSTUS	mRNA	35063	36424	0.93	-	.	ID=MALK_03806.t1;Parent=MALK_03806
contig016	AUGUSTUS	exon	35063	36424	0.93	-	.	ID=MALK_03806.t1.e1;Parent=MALK_03806.t1
contig016	AUGUSTUS	gene	36549	37084	0.85	+	.	ID=MALK_03807;prediction_source=braker_MRET:g3293.t1
contig016	AUGUSTUS	CDS	36549	36866	0.99	+	0	ID=MALK_03807.t1.c1;Parent=MALK_03807.t1
contig016	AUGUSTUS	CDS	36904	36963	0.99	+	0	ID=MALK_03807.t1.c2;Parent=MALK_03807.t1
contig016	AUGUSTUS	CDS	36995	37084	0.99	+	0	ID=MALK_03807.t1.c3;Parent=MALK_03807.t1
contig016	AUGUSTUS	mRNA	36549	37084	0.85	+	.	ID=MALK_03807.t1;Parent=MALK_03807
contig016	AUGUSTUS	exon	36549	36866	.	+	.	ID=MALK_03807.t1.e1;Parent=MALK_03807.t1
contig016	AUGUSTUS	exon	36904	36963	.	+	.	ID=MALK_03807.t1.e2;Parent=MALK_03807.t1
contig016	AUGUSTUS	exon	36995	37084	.	+	.	ID=MALK_03807.t1.e3;Parent=MALK_03807.t1
contig016	maker	gene	37134	40349	.	-	.	ID=MALK_03808;prediction_source=maker_MRET:augustus_masked-contig016-processed-gene-0.69-mRNA-1
contig016	maker	CDS	37766	40349	.	-	0	ID=MALK_03808.t1.c1;Parent=MALK_03808.t1
contig016	maker	CDS	37134	37684	.	-	0	ID=MALK_03808.t1.c2;Parent=MALK_03808.t1
contig016	maker	mRNA	37134	40349	.	-	.	ID=MALK_03808.t1;Parent=MALK_03808
contig016	maker	exon	37766	40349	.	-	.	ID=MALK_03808.t1.e1;Parent=MALK_03808.t1
contig016	maker	exon	37134	37684	.	-	.	ID=MALK_03808.t1.e2;Parent=MALK_03808.t1
contig016	AUGUSTUS	gene	40465	41967	0.96	-	.	ID=MALK_03809;prediction_source=augustus:contig016.g3768.t1
contig016	AUGUSTUS	CDS	40465	41967	0.96	-	0	ID=MALK_03809.t1.c1;Parent=MALK_03809.t1
contig016	AUGUSTUS	mRNA	40465	41967	0.96	-	.	ID=MALK_03809.t1;Parent=MALK_03809
contig016	AUGUSTUS	exon	40465	41967	0.96	-	.	ID=MALK_03809.t1.e1;Parent=MALK_03809.t1
contig016	AUGUSTUS	gene	42177	43586	1	-	.	ID=MALK_03810;prediction_source=augustus:contig016.g3769.t1
contig016	AUGUSTUS	CDS	42177	43586	1	-	0	ID=MALK_03810.t1.c1;Parent=MALK_03810.t1
contig016	AUGUSTUS	mRNA	42177	43586	1	-	.	ID=MALK_03810.t1;Parent=MALK_03810
contig016	AUGUSTUS	exon	42177	43586	1	-	.	ID=MALK_03810.t1.e1;Parent=MALK_03810.t1
contig016	AUGUSTUS	gene	43678	45203	0.6	-	.	ID=MALK_03811;prediction_source=braker_MRET:g3297.t1
contig016	AUGUSTUS	CDS	45123	45203	1	-	0	ID=MALK_03811.t1.c4;Parent=MALK_03811.t1
contig016	AUGUSTUS	CDS	44980	45081	1	-	0	ID=MALK_03811.t1.c3;Parent=MALK_03811.t1
contig016	AUGUSTUS	CDS	44891	44930	1	-	0	ID=MALK_03811.t1.c2;Parent=MALK_03811.t1
contig016	AUGUSTUS	CDS	43678	44861	1	-	0	ID=MALK_03811.t1.c1;Parent=MALK_03811.t1

contig016	AUGUSTUS	mRNA	43678	45203	0.6	-	.	ID=MALK_03811.t1;Parent=MALK_03811
contig016	AUGUSTUS	exon	45123	45203	.	-	.	ID=MALK_03811.t1.e4;Parent=MALK_03811.t1
contig016	AUGUSTUS	exon	44980	45081	.	-	.	ID=MALK_03811.t1.e3;Parent=MALK_03811.t1
contig016	AUGUSTUS	exon	44891	44930	.	-	.	ID=MALK_03811.t1.e2;Parent=MALK_03811.t1
contig016	AUGUSTUS	exon	43678	44861	.	-	.	ID=MALK_03811.t1.e1;Parent=MALK_03811.t1
contig016	AUGUSTUS	gene	46065	47774	0.49	-	.	ID=MALK_03812;prediction_source=augustus:contig016.g3771.t1
contig016	AUGUSTUS	CDS	46065	47774	0.49	-	0	ID=MALK_03812.t1.c1;Parent=MALK_03812.t1
contig016	AUGUSTUS	mRNA	46065	47774	0.49	-	.	ID=MALK_03812.t1;Parent=MALK_03812
contig016	AUGUSTUS	exon	46065	47774	0.49	-	.	ID=MALK_03812.t1.e1;Parent=MALK_03812.t1
contig016	AUGUSTUS	gene	48693	50864	0.87	-	.	ID=MALK_03813;prediction_source=braker_MRET:g3299.t1
contig016	AUGUSTUS	CDS	50646	50864	0.94	-	0	ID=MALK_03813.t1.c3;Parent=MALK_03813.t1
contig016	AUGUSTUS	CDS	50582	50613	0.94	-	0	ID=MALK_03813.t1.c2;Parent=MALK_03813.t1
contig016	AUGUSTUS	CDS	48693	50553	0.94	-	0	ID=MALK_03813.t1.c1;Parent=MALK_03813.t1
contig016	AUGUSTUS	mRNA	48693	50864	0.87	-	.	ID=MALK_03813.t1;Parent=MALK_03813
contig016	AUGUSTUS	exon	50646	50864	.	-	.	ID=MALK_03813.t1.e3;Parent=MALK_03813.t1
contig016	AUGUSTUS	exon	50582	50613	.	-	.	ID=MALK_03813.t1.e2;Parent=MALK_03813.t1
contig016	AUGUSTUS	exon	48693	50553	.	-	.	ID=MALK_03813.t1.e1;Parent=MALK_03813.t1
contig016	maker	gene	51572	54055	.	+	.	ID=MALK_03814;prediction_source=maker_MRET:augustus_masked-contig016-processed-gene-0.51-mRNA-1
contig016	maker	CDS	51572	54055	.	+	0	ID=MALK_03814.t1.c1;Parent=MALK_03814.t1
contig016	maker	mRNA	51572	54055	.	+	.	ID=MALK_03814.t1;Parent=MALK_03814
contig016	maker	exon	51572	54055	.	+	.	ID=MALK_03814.t1.e1;Parent=MALK_03814.t1
contig016	AUGUSTUS	gene	54815	56188	0.98	+	.	ID=MALK_03815;prediction_source=augustus:contig016.g3776.t1
contig016	AUGUSTUS	CDS	54815	56188	0.98	+	0	ID=MALK_03815.t1.c1;Parent=MALK_03815.t1
contig016	AUGUSTUS	mRNA	54815	56188	0.98	+	.	ID=MALK_03815.t1;Parent=MALK_03815
contig016	AUGUSTUS	exon	54815	56188	0.98	+	.	ID=MALK_03815.t1.e1;Parent=MALK_03815.t1
contig016	maker	gene	56545	58188	.	+	.	ID=MALK_03816;prediction_source=maker_MRET:augustus_masked-contig016-processed-gene-0.53-mRNA-1
contig016	maker	CDS	56545	58188	.	+	0	ID=MALK_03816.t1.c1;Parent=MALK_03816.t1
contig016	maker	mRNA	56545	58188	.	+	.	ID=MALK_03816.t1;Parent=MALK_03816
contig016	maker	exon	56545	58188	.	+	.	ID=MALK_03816.t1.e1;Parent=MALK_03816.t1
contig016	AUGUSTUS	gene	58239	58952	0.97	-	.	ID=MALK_03817;prediction_source=braker_MRET:g3302.t1
contig016	AUGUSTUS	CDS	58890	58952	0.98	-	0	ID=MALK_03817.t1.c3;Parent=MALK_03817.t1
contig016	AUGUSTUS	CDS	58776	58861	0.98	-	0	ID=MALK_03817.t1.c2;Parent=MALK_03817.t1
contig016	AUGUSTUS	CDS	58239	58737	0.98	-	0	ID=MALK_03817.t1.c1;Parent=MALK_03817.t1
contig016	AUGUSTUS	mRNA	58239	58952	0.97	-	.	ID=MALK_03817.t1;Parent=MALK_03817
contig016	AUGUSTUS	exon	58890	58952	.	-	.	ID=MALK_03817.t1.e3;Parent=MALK_03817.t1
contig016	AUGUSTUS	exon	58776	58861	.	-	.	ID=MALK_03817.t1.e2;Parent=MALK_03817.t1
contig016	AUGUSTUS	exon	58239	58737	.	-	.	ID=MALK_03817.t1.e1;Parent=MALK_03817.t1
contig016	AUGUSTUS	gene	59703	63380	1	+	.	ID=MALK_03818;prediction_source=augustus:contig016.g3779.t1
contig016	AUGUSTUS	CDS	59703	63380	1	+	0	ID=MALK_03818.t1.c1;Parent=MALK_03818.t1
contig016	AUGUSTUS	mRNA	59703	63380	1	+	.	ID=MALK_03818.t1;Parent=MALK_03818
contig016	AUGUSTUS	exon	59703	63380	1	+	.	ID=MALK_03818.t1.e1;Parent=MALK_03818.t1
contig016	maker	gene	63433	65172	.	-	.	ID=MALK_03819;prediction_source=maker_MRET:augustus_masked-contig016-processed-gene-0.76-mRNA-1
contig016	maker	CDS	63433	65172	.	-	0	ID=MALK_03819.t1.c1;Parent=MALK_03819.t1

contig016	maker	mRNA	63433	65172	.	-	.	ID=MALK_03819.t1;Parent=MALK_03819
contig016	maker	exon	63433	65172	.	-	.	ID=MALK_03819.t1.e1;Parent=MALK_03819.t1
contig016	AUGUSTUS	gene	65197	66354	0.6	+	.	ID=MALK_03820;prediction_source=augustus:contig016.g3781.t1
contig016	AUGUSTUS	CDS	65197	66354	0.6	+	0	ID=MALK_03820.t1.c1;Parent=MALK_03820.t1
contig016	AUGUSTUS	mRNA	65197	66354	0.6	+	.	ID=MALK_03820.t1;Parent=MALK_03820
contig016	AUGUSTUS	exon	65197	66354	0.6	+	.	ID=MALK_03820.t1.e1;Parent=MALK_03820.t1
contig016	AUGUSTUS	gene	66481	68763	0.78	+	.	ID=MALK_03821;prediction_source=braker_MRET:g3306.t1
contig016	AUGUSTUS	CDS	66481	68763	0.78	+	0	ID=MALK_03821.t1.c1;Parent=MALK_03821.t1
contig016	AUGUSTUS	mRNA	66481	68763	0.78	+	.	ID=MALK_03821.t1;Parent=MALK_03821
contig016	AUGUSTUS	exon	66481	68763	.	+	.	ID=MALK_03821.t1.e1;Parent=MALK_03821.t1
contig016	AUGUSTUS	gene	70289	70567	0.89	-	.	ID=MALK_03822;prediction_source=braker_MRET:g3307.t1
contig016	AUGUSTUS	CDS	70289	70567	0.89	-	0	ID=MALK_03822.t1.c1;Parent=MALK_03822.t1
contig016	AUGUSTUS	mRNA	70289	70567	0.89	-	.	ID=MALK_03822.t1;Parent=MALK_03822
contig016	AUGUSTUS	exon	70289	70567	.	-	.	ID=MALK_03822.t1.e1;Parent=MALK_03822.t1
contig016	maker	gene	71463	72632	.	+	.	ID=MALK_03823;prediction_source=maker_MRET:augustus_masked-contig016-processed-gene-0.56-mRNA-1
contig016	maker	CDS	71463	72632	.	+	0	ID=MALK_03823.t1.c1;Parent=MALK_03823.t1
contig016	maker	mRNA	71463	72632	.	+	.	ID=MALK_03823.t1;Parent=MALK_03823
contig016	maker	exon	71463	72632	.	+	.	ID=MALK_03823.t1.e1;Parent=MALK_03823.t1
contig016	AUGUSTUS	gene	72877	73170	0.79	-	.	ID=MALK_03824;prediction_source=braker_MRET:g3309.t1
contig016	AUGUSTUS	CDS	72877	73170	0.79	-	0	ID=MALK_03824.t1.c1;Parent=MALK_03824.t1
contig016	AUGUSTUS	mRNA	72877	73170	0.79	-	.	ID=MALK_03824.t1;Parent=MALK_03824
contig016	AUGUSTUS	exon	72877	73170	.	-	.	ID=MALK_03824.t1.e1;Parent=MALK_03824.t1
contig016	AUGUSTUS	gene	73904	75313	0.96	+	.	ID=MALK_03825;prediction_source=augustus:contig016.g3786.t1
contig016	AUGUSTUS	CDS	73904	75313	0.96	+	0	ID=MALK_03825.t1.c1;Parent=MALK_03825.t1
contig016	AUGUSTUS	mRNA	73904	75313	0.96	+	.	ID=MALK_03825.t1;Parent=MALK_03825
contig016	AUGUSTUS	exon	73904	75313	0.96	+	.	ID=MALK_03825.t1.e1;Parent=MALK_03825.t1
contig016	maker	gene	75575	76984	.	+	.	ID=MALK_03826;prediction_source=maker_MRET:augustus_masked-contig016-processed-gene-0.58-mRNA-1
contig016	maker	CDS	75575	76984	.	+	0	ID=MALK_03826.t1.c1;Parent=MALK_03826.t1
contig016	maker	mRNA	75575	76984	.	+	.	ID=MALK_03826.t1;Parent=MALK_03826
contig016	maker	exon	75575	76984	.	+	.	ID=MALK_03826.t1.e1;Parent=MALK_03826.t1
contig016	AUGUSTUS	gene	76991	78310	1	-	.	ID=MALK_03827;prediction_source=braker_MRET:g3312.t1
contig016	AUGUSTUS	CDS	76991	78310	1	-	0	ID=MALK_03827.t1.c1;Parent=MALK_03827.t1
contig016	AUGUSTUS	mRNA	76991	78310	1	-	.	ID=MALK_03827.t1;Parent=MALK_03827
contig016	AUGUSTUS	exon	76991	78310	.	-	.	ID=MALK_03827.t1.e1;Parent=MALK_03827.t1
contig016	AUGUSTUS	gene	78420	79032	0.35	+	.	ID=MALK_03828;prediction_source=braker_MRET:g3313.t1
contig016	AUGUSTUS	CDS	78420	78979	0.72	+	0	ID=MALK_03828.t1.c1;Parent=MALK_03828.t1
contig016	AUGUSTUS	CDS	79020	79032	0.72	+	0	ID=MALK_03828.t1.c2;Parent=MALK_03828.t1
contig016	AUGUSTUS	mRNA	78420	79032	0.35	+	.	ID=MALK_03828.t1;Parent=MALK_03828
contig016	AUGUSTUS	exon	78420	78979	.	+	.	ID=MALK_03828.t1.e1;Parent=MALK_03828.t1
contig016	AUGUSTUS	exon	79020	79032	.	+	.	ID=MALK_03828.t1.e2;Parent=MALK_03828.t1
contig016	maker	gene	79088	81781	.	-	.	ID=MALK_03829;prediction_source=maker_MRET:augustus_masked-contig016-processed-gene-0.79-mRNA-1
contig016	maker	CDS	79088	81781	.	-	0	ID=MALK_03829.t1.c1;Parent=MALK_03829.t1
contig016	maker	mRNA	79088	81781	.	-	.	ID=MALK_03829.t1;Parent=MALK_03829

contig016	maker	exon	79088	81781	.	-	.	ID=MALK_03829.t1.e1;Parent=MALK_03829.t1
contig016	AUGUSTUS	gene	81828	87542	0.97	+	.	ID=MALK_03830;prediction_source=braker_MRET:g3315.t1
contig016	AUGUSTUS	CDS	81828	87542	0.97	+	0	ID=MALK_03830.t1.c1;Parent=MALK_03830.t1
contig016	AUGUSTUS	mRNA	81828	87542	0.97	+	.	ID=MALK_03830.t1;Parent=MALK_03830
contig016	AUGUSTUS	exon	81828	87542	.	+	.	ID=MALK_03830.t1.e1;Parent=MALK_03830.t1
contig016	AUGUSTUS	gene	87586	89742	0.99	+	.	ID=MALK_03831;prediction_source=braker_MRET:g3316.t1
contig016	AUGUSTUS	CDS	87586	89742	0.99	+	0	ID=MALK_03831.t1.c1;Parent=MALK_03831.t1
contig016	AUGUSTUS	mRNA	87586	89742	0.99	+	.	ID=MALK_03831.t1;Parent=MALK_03831
contig016	AUGUSTUS	exon	87586	89742	.	+	.	ID=MALK_03831.t1.e1;Parent=MALK_03831.t1
contig016	AUGUSTUS	gene	89744	92116	0.31	-	.	ID=MALK_03832;prediction_source=augustus:contig016.g3793.t1
contig016	AUGUSTUS	CDS	89744	92116	0.31	-	0	ID=MALK_03832.t1.c1;Parent=MALK_03832.t1
contig016	AUGUSTUS	mRNA	89744	92116	0.31	-	.	ID=MALK_03832.t1;Parent=MALK_03832
contig016	AUGUSTUS	exon	89744	92116	0.31	-	.	ID=MALK_03832.t1.e1;Parent=MALK_03832.t1
contig016	AUGUSTUS	gene	92236	93020	0.58	-	.	ID=MALK_03833;prediction_source=braker_MRET:g3318.t1
contig016	AUGUSTUS	CDS	92887	93020	0.59	-	0	ID=MALK_03833.t1.c2;Parent=MALK_03833.t1
contig016	AUGUSTUS	CDS	92236	92854	0.59	-	0	ID=MALK_03833.t1.c1;Parent=MALK_03833.t1
contig016	AUGUSTUS	mRNA	92236	93020	0.58	-	.	ID=MALK_03833.t1;Parent=MALK_03833
contig016	AUGUSTUS	exon	92887	93020	.	-	.	ID=MALK_03833.t1.e2;Parent=MALK_03833.t1
contig016	AUGUSTUS	exon	92236	92854	.	-	.	ID=MALK_03833.t1.e1;Parent=MALK_03833.t1
contig016	AUGUSTUS	gene	93081	98915	0.96	+	.	ID=MALK_03834;prediction_source=augustus:contig016.g3795.t1
contig016	AUGUSTUS	CDS	93081	98915	0.96	+	0	ID=MALK_03834.t1.c1;Parent=MALK_03834.t1
contig016	AUGUSTUS	mRNA	93081	98915	0.96	+	.	ID=MALK_03834.t1;Parent=MALK_03834
contig016	AUGUSTUS	exon	93081	98915	0.96	+	.	ID=MALK_03834.t1.e1;Parent=MALK_03834.t1
contig016	AUGUSTUS	gene	99595	100155	1	-	.	ID=MALK_03835;prediction_source=braker_MRET:g3320.t1
contig016	AUGUSTUS	CDS	100153	100155	1	-	0	ID=MALK_03835.t1.c2;Parent=MALK_03835.t1
contig016	AUGUSTUS	CDS	99595	100110	1	-	0	ID=MALK_03835.t1.c1;Parent=MALK_03835.t1
contig016	AUGUSTUS	mRNA	99595	100155	1	-	.	ID=MALK_03835.t1;Parent=MALK_03835
contig016	AUGUSTUS	exon	100153	100155	.	-	.	ID=MALK_03835.t1.e2;Parent=MALK_03835.t1
contig016	AUGUSTUS	exon	99595	100110	.	-	.	ID=MALK_03835.t1.e1;Parent=MALK_03835.t1
contig016	AUGUSTUS	gene	100417	101154	0.39	+	.	ID=MALK_03836;prediction_source=braker_MRET:g3321.t1
contig016	AUGUSTUS	CDS	100417	100503	1	+	0	ID=MALK_03836.t1.c1;Parent=MALK_03836.t1
contig016	AUGUSTUS	CDS	100553	100660	1	+	0	ID=MALK_03836.t1.c2;Parent=MALK_03836.t1
contig016	AUGUSTUS	CDS	100699	100710	1	+	0	ID=MALK_03836.t1.c3;Parent=MALK_03836.t1
contig016	AUGUSTUS	CDS	100771	100792	1	+	0	ID=MALK_03836.t1.c4;Parent=MALK_03836.t1
contig016	AUGUSTUS	CDS	100836	100944	1	+	0	ID=MALK_03836.t1.c5;Parent=MALK_03836.t1
contig016	AUGUSTUS	CDS	100984	100985	1	+	0	ID=MALK_03836.t1.c6;Parent=MALK_03836.t1
contig016	AUGUSTUS	CDS	101052	101088	1	+	0	ID=MALK_03836.t1.c7;Parent=MALK_03836.t1
contig016	AUGUSTUS	CDS	101130	101154	1	+	0	ID=MALK_03836.t1.c8;Parent=MALK_03836.t1
contig016	AUGUSTUS	mRNA	100417	101154	0.39	+	.	ID=MALK_03836.t1;Parent=MALK_03836
contig016	AUGUSTUS	exon	100417	100503	.	+	.	ID=MALK_03836.t1.e1;Parent=MALK_03836.t1
contig016	AUGUSTUS	exon	100553	100660	.	+	.	ID=MALK_03836.t1.e2;Parent=MALK_03836.t1
contig016	AUGUSTUS	exon	100699	100710	.	+	.	ID=MALK_03836.t1.e3;Parent=MALK_03836.t1
contig016	AUGUSTUS	exon	100771	100792	.	+	.	ID=MALK_03836.t1.e4;Parent=MALK_03836.t1

contig016	AUGUSTUS	exon	100836	100944	.	+	.	ID=MALK_03836.t1.e5;Parent=MALK_03836.t1
contig016	AUGUSTUS	exon	100984	100985	.	+	.	ID=MALK_03836.t1.e6;Parent=MALK_03836.t1
contig016	AUGUSTUS	exon	101052	101088	.	+	.	ID=MALK_03836.t1.e7;Parent=MALK_03836.t1
contig016	AUGUSTUS	exon	101130	101154	.	+	.	ID=MALK_03836.t1.e8;Parent=MALK_03836.t1
contig016	AUGUSTUS	gene	101373	101738	0.81	-	.	ID=MALK_03837;prediction_source=braker_MRET:g3322.t1
contig016	AUGUSTUS	CDS	101642	101738	0.97	-	0	ID=MALK_03837.t1.c3;Parent=MALK_03837.t1
contig016	AUGUSTUS	CDS	101550	101603	0.97	-	0	ID=MALK_03837.t1.c2;Parent=MALK_03837.t1
contig016	AUGUSTUS	CDS	101373	101509	0.97	-	0	ID=MALK_03837.t1.c1;Parent=MALK_03837.t1
contig016	AUGUSTUS	mRNA	101373	101738	0.81	-	.	ID=MALK_03837.t1;Parent=MALK_03837
contig016	AUGUSTUS	exon	101642	101738	.	-	.	ID=MALK_03837.t1.e3;Parent=MALK_03837.t1
contig016	AUGUSTUS	exon	101550	101603	.	-	.	ID=MALK_03837.t1.e2;Parent=MALK_03837.t1
contig016	AUGUSTUS	exon	101373	101509	.	-	.	ID=MALK_03837.t1.e1;Parent=MALK_03837.t1
contig016	AUGUSTUS	gene	102031	104175	0.48	+	.	ID=MALK_03838;prediction_source=augustus:contig016.g3799.t1
contig016	AUGUSTUS	CDS	102031	104175	0.48	+	0	ID=MALK_03838.t1.c1;Parent=MALK_03838.t1
contig016	AUGUSTUS	mRNA	102031	104175	0.48	+	.	ID=MALK_03838.t1;Parent=MALK_03838
contig016	AUGUSTUS	exon	102031	104175	0.48	+	.	ID=MALK_03838.t1.e1;Parent=MALK_03838.t1
contig016	AUGUSTUS	gene	104209	105093	0.99	-	.	ID=MALK_03839;prediction_source=augustus:contig016.g3800.t1
contig016	AUGUSTUS	CDS	104209	105093	0.99	-	0	ID=MALK_03839.t1.c1;Parent=MALK_03839.t1
contig016	AUGUSTUS	mRNA	104209	105093	0.99	-	.	ID=MALK_03839.t1;Parent=MALK_03839
contig016	AUGUSTUS	exon	104209	105093	0.99	-	.	ID=MALK_03839.t1.e1;Parent=MALK_03839.t1
contig016	AUGUSTUS	gene	105461	106686	0.7	+	.	ID=MALK_03840;prediction_source=augustus:contig016.g3801.t1
contig016	AUGUSTUS	CDS	105461	105578	0.7	+	0	ID=MALK_03840.t1.c1;Parent=MALK_03840.t1
contig016	AUGUSTUS	CDS	105683	106686	0.7	+	0	ID=MALK_03840.t1.c2;Parent=MALK_03840.t1
contig016	AUGUSTUS	mRNA	105461	106686	0.7	+	.	ID=MALK_03840.t1;Parent=MALK_03840
contig016	AUGUSTUS	exon	105461	105578	0.7	+	.	ID=MALK_03840.t1.e1;Parent=MALK_03840.t1
contig016	AUGUSTUS	exon	105683	106686	0.7	+	.	ID=MALK_03840.t1.e2;Parent=MALK_03840.t1
contig016	AUGUSTUS	gene	107048	108898	0.75	-	.	ID=MALK_03841;prediction_source=augustus:contig016.g3802.t1
contig016	AUGUSTUS	CDS	107048	108898	0.75	-	0	ID=MALK_03841.t1.c1;Parent=MALK_03841.t1
contig016	AUGUSTUS	mRNA	107048	108898	0.75	-	.	ID=MALK_03841.t1;Parent=MALK_03841
contig016	AUGUSTUS	exon	107048	108898	0.75	-	.	ID=MALK_03841.t1.e1;Parent=MALK_03841.t1
contig016	maker	gene	109405	110412	.	+	.	ID=MALK_03842;prediction_source=maker_MRET:augustus_masked-contig016-processed-gene-1.5-mRNA-1
contig016	maker	CDS	109405	110412	.	+	0	ID=MALK_03842.t1.c1;Parent=MALK_03842.t1
contig016	maker	mRNA	109405	110412	.	+	.	ID=MALK_03842.t1;Parent=MALK_03842
contig016	maker	exon	109405	110412	.	+	.	ID=MALK_03842.t1.e1;Parent=MALK_03842.t1
contig016	AUGUSTUS	gene	110973	113384	0.97	+	.	ID=MALK_03843;prediction_source=augustus:contig016.g3805.t1
contig016	AUGUSTUS	CDS	110973	113384	0.97	+	0	ID=MALK_03843.t1.c1;Parent=MALK_03843.t1
contig016	AUGUSTUS	mRNA	110973	113384	0.97	+	.	ID=MALK_03843.t1;Parent=MALK_03843
contig016	AUGUSTUS	exon	110973	113384	0.97	+	.	ID=MALK_03843.t1.e1;Parent=MALK_03843.t1
contig016	maker	gene	113700	115694	.	+	.	ID=MALK_03844;prediction_source=maker_MRET:augustus_masked-contig016-processed-gene-1.7-mRNA-1
contig016	maker	CDS	113700	115694	.	+	0	ID=MALK_03844.t1.c1;Parent=MALK_03844.t1
contig016	maker	mRNA	113700	115694	.	+	.	ID=MALK_03844.t1;Parent=MALK_03844
contig016	maker	exon	113700	115694	.	+	.	ID=MALK_03844.t1.e1;Parent=MALK_03844.t1
contig016	maker	gene	115783	116379	.	-	.	ID=MALK_03845;prediction_source=maker_MRET:augustus_masked-contig016-processed-gene-1.28-mRNA-1

contig016	maker	CDS	115783	116379	.	-	0	ID=MALK_03845.t1.c1;Parent=MALK_03845.t1
contig016	maker	mRNA	115783	116379	.	-	.	ID=MALK_03845.t1;Parent=MALK_03845
contig016	maker	exon	115783	116379	.	-	.	ID=MALK_03845.t1.e1;Parent=MALK_03845.t1
contig016	AUGUSTUS	gene	116585	117559	1	+	.	ID=MALK_03846;prediction_source=braker_MRET:g3331.t1
contig016	AUGUSTUS	CDS	116585	117559	1	+	0	ID=MALK_03846.t1.c1;Parent=MALK_03846.t1
contig016	AUGUSTUS	mRNA	116585	117559	1	+	.	ID=MALK_03846.t1;Parent=MALK_03846
contig016	AUGUSTUS	exon	116585	117559	.	+	.	ID=MALK_03846.t1.e1;Parent=MALK_03846.t1
contig016	AUGUSTUS	gene	117605	120256	0.99	-	.	ID=MALK_03847;prediction_source=augustus:contig016.g3810.t1
contig016	AUGUSTUS	CDS	117605	120256	0.99	-	0	ID=MALK_03847.t1.c1;Parent=MALK_03847.t1
contig016	AUGUSTUS	mRNA	117605	120256	0.99	-	.	ID=MALK_03847.t1;Parent=MALK_03847
contig016	AUGUSTUS	exon	117605	120256	0.99	-	.	ID=MALK_03847.t1.e1;Parent=MALK_03847.t1
contig016	AUGUSTUS	gene	120385	121304	0.38	+	.	ID=MALK_03848;prediction_source=braker_MRET:g3333.t1
contig016	AUGUSTUS	CDS	120385	120400	0.98	+	0	ID=MALK_03848.t1.c1;Parent=MALK_03848.t1
contig016	AUGUSTUS	CDS	120450	120773	0.98	+	0	ID=MALK_03848.t1.c2;Parent=MALK_03848.t1
contig016	AUGUSTUS	CDS	120810	121116	0.98	+	0	ID=MALK_03848.t1.c3;Parent=MALK_03848.t1
contig016	AUGUSTUS	CDS	121166	121304	0.98	+	0	ID=MALK_03848.t1.c4;Parent=MALK_03848.t1
contig016	AUGUSTUS	mRNA	120385	121304	0.38	+	.	ID=MALK_03848.t1;Parent=MALK_03848
contig016	AUGUSTUS	exon	120385	120400	.	+	.	ID=MALK_03848.t1.e1;Parent=MALK_03848.t1
contig016	AUGUSTUS	exon	120450	120773	.	+	.	ID=MALK_03848.t1.e2;Parent=MALK_03848.t1
contig016	AUGUSTUS	exon	120810	121116	.	+	.	ID=MALK_03848.t1.e3;Parent=MALK_03848.t1
contig016	AUGUSTUS	exon	121166	121304	.	+	.	ID=MALK_03848.t1.e4;Parent=MALK_03848.t1
contig016	AUGUSTUS	gene	121314	121886	0.97	-	.	ID=MALK_03849;prediction_source=augustus:contig016.g3814.t1
contig016	AUGUSTUS	CDS	121314	121886	0.97	-	0	ID=MALK_03849.t1.c1;Parent=MALK_03849.t1
contig016	AUGUSTUS	mRNA	121314	121886	0.97	-	.	ID=MALK_03849.t1;Parent=MALK_03849
contig016	AUGUSTUS	exon	121314	121886	0.97	-	.	ID=MALK_03849.t1.e1;Parent=MALK_03849.t1
contig016	maker	gene	122252	123385	.	+	.	ID=MALK_03850;prediction_source=maker_MRET:augustus_masked-contig016-processed-gene-1.10-mRNA-1
contig016	maker	CDS	122252	123385	.	+	0	ID=MALK_03850.t1.c1;Parent=MALK_03850.t1
contig016	maker	mRNA	122252	123385	.	+	.	ID=MALK_03850.t1;Parent=MALK_03850
contig016	maker	exon	122252	123385	.	+	.	ID=MALK_03850.t1.e1;Parent=MALK_03850.t1
contig016	maker	gene	123388	127984	.	-	.	ID=MALK_03851;prediction_source=maker_MRET:augustus_masked-contig016-processed-gene-1.31-mRNA-1
contig016	maker	CDS	127798	127984	.	-	0	ID=MALK_03851.t1.c1;Parent=MALK_03851.t1
contig016	maker	CDS	123388	127697	.	-	0	ID=MALK_03851.t1.c2;Parent=MALK_03851.t1
contig016	maker	mRNA	123388	127984	.	-	.	ID=MALK_03851.t1;Parent=MALK_03851
contig016	maker	exon	127798	127984	.	-	.	ID=MALK_03851.t1.e1;Parent=MALK_03851.t1
contig016	maker	exon	123388	127697	.	-	.	ID=MALK_03851.t1.e2;Parent=MALK_03851.t1
contig016	AUGUSTUS	gene	128389	130191	0.51	-	.	ID=MALK_03852;prediction_source=augustus:contig016.g3818.t1
contig016	AUGUSTUS	CDS	128389	130191	0.51	-	0	ID=MALK_03852.t1.c1;Parent=MALK_03852.t1
contig016	AUGUSTUS	mRNA	128389	130191	0.51	-	.	ID=MALK_03852.t1;Parent=MALK_03852
contig016	AUGUSTUS	exon	128389	130191	0.51	-	.	ID=MALK_03852.t1.e1;Parent=MALK_03852.t1
contig016	maker	gene	130298	131116	.	+	.	ID=MALK_03853;prediction_source=maker_MRET:augustus_masked-contig016-processed-gene-1.11-mRNA-1
contig016	maker	CDS	130298	131116	.	+	0	ID=MALK_03853.t1.c1;Parent=MALK_03853.t1
contig016	maker	mRNA	130298	131116	.	+	.	ID=MALK_03853.t1;Parent=MALK_03853
contig016	maker	exon	130298	131116	.	+	.	ID=MALK_03853.t1.e1;Parent=MALK_03853.t1

contig016	AUGUSTUS	gene	131151	135332	0.82	-	.	ID=MALK_03854;prediction_source=augustus:contig016.g3820.t1
contig016	AUGUSTUS	CDS	131151	135332	0.82	-	0	ID=MALK_03854.t1.c1;Parent=MALK_03854.t1
contig016	AUGUSTUS	mRNA	131151	135332	0.82	-	.	ID=MALK_03854.t1;Parent=MALK_03854
contig016	AUGUSTUS	exon	131151	135332	0.82	-	.	ID=MALK_03854.t1.e1;Parent=MALK_03854.t1
contig016	maker	gene	135786	137171	.	+	.	ID=MALK_03855;prediction_source=maker_MRET:augustus_masked-contig016-processed-gene-1.12-mRNA-1
contig016	maker	CDS	135786	137171	.	+	0	ID=MALK_03855.t1.c1;Parent=MALK_03855.t1
contig016	maker	mRNA	135786	137171	.	+	.	ID=MALK_03855.t1;Parent=MALK_03855
contig016	maker	exon	135786	137171	.	+	.	ID=MALK_03855.t1.e1;Parent=MALK_03855.t1
contig016	AUGUSTUS	gene	137201	140695	0.94	+	.	ID=MALK_03856;prediction_source=augustus:contig016.g3823.t1
contig016	AUGUSTUS	CDS	137201	140695	0.94	+	0	ID=MALK_03856.t1.c1;Parent=MALK_03856.t1
contig016	AUGUSTUS	mRNA	137201	140695	0.94	+	.	ID=MALK_03856.t1;Parent=MALK_03856
contig016	AUGUSTUS	exon	137201	140695	0.94	+	.	ID=MALK_03856.t1.e1;Parent=MALK_03856.t1
contig016	maker	gene	140767	142143	.	+	.	ID=MALK_03857;prediction_source=maker_MRET:augustus_masked-contig016-processed-gene-1.14-mRNA-1
contig016	maker	CDS	140767	142143	.	+	0	ID=MALK_03857.t1.c1;Parent=MALK_03857.t1
contig016	maker	mRNA	140767	142143	.	+	.	ID=MALK_03857.t1;Parent=MALK_03857
contig016	maker	exon	140767	142143	.	+	.	ID=MALK_03857.t1.e1;Parent=MALK_03857.t1
contig016	maker	gene	142718	144331	.	-	.	ID=MALK_03858;prediction_source=maker_MRET:augustus_masked-contig016-processed-gene-1.34-mRNA-1
contig016	maker	CDS	142718	144331	.	-	0	ID=MALK_03858.t1.c1;Parent=MALK_03858.t1
contig016	maker	mRNA	142718	144331	.	-	.	ID=MALK_03858.t1;Parent=MALK_03858
contig016	maker	exon	142718	144331	.	-	.	ID=MALK_03858.t1.e1;Parent=MALK_03858.t1
contig016	AUGUSTUS	gene	144383	145090	0.81	+	.	ID=MALK_03859;prediction_source=augustus:contig016.g3826.t1
contig016	AUGUSTUS	CDS	144383	145090	0.81	+	0	ID=MALK_03859.t1.c1;Parent=MALK_03859.t1
contig016	AUGUSTUS	mRNA	144383	145090	0.81	+	.	ID=MALK_03859.t1;Parent=MALK_03859
[truncated: 142,222 more chars]
